# Genome-wide high-throughput SNP discovery and genotyping for understanding natural (functional) allelic diversity and domestication patterns in wild chickpea

**DOI:** 10.1038/srep12468

**Published:** 2015-07-24

**Authors:** Deepak Bajaj, Shouvik Das, Saurabh Badoni, Vinod Kumar, Mohar Singh, Kailash C. Bansal, Akhilesh K. Tyagi, Swarup K. Parida

**Affiliations:** 1 National Institute of Plant Genome Research (NIPGR), Aruna Asaf Ali Marg, New Delhi 110067, India; 2 National Research Centre on Plant Biotechnology (NRCPB), New Delhi-110012, India; 3 National Bureau of Plant Genetic Resources (NBPGR), New Delhi-110012, India

## Abstract

We identified 82489 high-quality genome-wide SNPs from 93 wild and cultivated *Cicer* accessions through integrated reference genome- and *de novo*-based GBS assays. High intra- and inter-specific polymorphic potential (66–85%) and broader natural allelic diversity (6–64%) detected by genome-wide SNPs among accessions signify their efficacy for monitoring introgression and transferring target trait-regulating genomic (gene) regions/allelic variants from wild to cultivated *Cicer* gene pools for genetic improvement. The population-specific assignment of wild *Cicer* accessions pertaining to the primary gene pool are more influenced by geographical origin/phenotypic characteristics than species/gene-pools of origination. The functional significance of allelic variants (non-synonymous and regulatory SNPs) scanned from transcription factors and stress-responsive genes in differentiating wild accessions (with potential known sources of yield-contributing and stress tolerance traits) from cultivated *desi* and *kabuli* accessions, fine-mapping/map-based cloning of QTLs and determination of LD patterns across wild and cultivated gene-pools are suitably elucidated. The correlation between phenotypic (agromorphological traits) and molecular diversity-based admixed domestication patterns within six structured populations of wild and cultivated accessions via genome-wide SNPs was apparent. This suggests utility of whole genome SNPs as a potential resource for identifying naturally selected trait-regulating genomic targets/functional allelic variants adaptive to diverse agroclimatic regions for genetic enhancement of cultivated gene-pools.

In chickpea, a plethora of array-based SNP (single nucleotide polymorphism) genotyping approaches [including Illumina GoldenGate/Infinium (Bead Xpress array) and KBioscience Competitive Allele-Specific Polymerase chain reaction (KASPar) assays] are known to greatly expedite the large-scale validation and high-throughput genotyping of previously discovered SNPs in diverse accessions, specifically for genetic diversity studies, phylogenetics and genetic linkage map construction^1^,^2^,^3^,^4^,^5^. The implications of RAD-seq (restriction-site-associated DNA sequencing) that relies on barcoded multiplexing and the RE (restriction enzyme)-based NGS (next-generation sequencing) approach for simultaneous mining and genotyping of genome-wide SNPs are well illustrated in chickpea for comprehending the molecular diversity pattern among its diverse *desi*, *kabuli* and wild accessions^6^. Recently, with the emergence of an improved (high barcoded genotype multiplexing capacity), user friendly and resource-saving NGS-based genotyping-by-sequencing (GBS) assay, the potential of simultaneous high-throughput genome-wide SNPs discovery and genotyping have improved manifold, involving diverse crop accessions^7^,^8^,^9^. The fast SNP mining and genotyping capacity of the GBS assay gives it an edge over other available high-throughput genotyping assays that have been utilized in myriad genomics-assisted breeding applications, such as genome-wide high-resolution trait association and genetic mapping in diverse crop plants^9^,^10^,^11^,^12^,^13^,^14^,^15^,^16^,^17^,^18^,^19^,^20^,^21^. With readily available chickpea draft genome sequences^22^,^6^, the large-scale validation and high-throughput genotyping of SNPs at a genome-wide scale employing the GBS approach seems rational and feasible for diverse chickpea accessions. Unfortunately, hitherto, no such efforts have been made in chickpea, specifically in cases of diverse, naturally occurring wild germplasm accessions.

Chickpea [*Cicer arietinum* (L.)] supposedly originated from the central region of the Fertile Crescent (presently South Eastern Turkey and Syria) along with its close progenitors, the wild annual *Cicer* species^23^,^24^,^25^,^26^,^27^. The genus *Cicer* consists of 44 annual and perennial species grouped into three gene pools (primary, secondary and tertiary) according to their crossability with cultivated chickpea^28^,^29^. The annual wild species (*C. reticulatum*) representing the primary gene pool with contrasting characteristics of high crossability with cultivated *C. arietinum* (*desi* and *kabuli*) serves as a potential source to broaden the genetic base and enhance the yield-component and stress tolerance traits in the cultivated gene pool^30^,^31^,^32^,^33^,^34^,^35^. To accelerate the process of genetic improvement through inter-specific hybridization, specifically the understanding of molecular diversity and domestication patterns among wild and cultivated *Cicer* accessions representing each of the three gene pools at a genome-wide scale is essential. Furthermore, this process can be complemented with marker-based introgression of trait-associated novel genes, QTLs (quantitative trait loci) and natural allelic variants scanned from diverse wild gene pools into cultivated accessions.

Significant efforts have been made towards understanding the genetic diversity pattern, population structure and phylogenetic relationship among diverse annual and perennial *Cicer* species using various random and sequence-based robust SSR (simple sequence repeat) and SNP markers^6^,^29^,^36^,^37^,^38^,^39^,^40^,^41^,^42^,^43^,^44^,^45^,^46^,^47^,^48^. However, the use of a limited number of selected markers in all these studies might provide unrealistic estimates of genetic variability with ambiguous molecular diversity and phylogenetic information among wild and cultivated *Cicer* accessions. In this context, discovery, large-scale validation and high-throughput genotyping of numerous genome-wide SNP markers using a highly efficient GBS assay could be an attractive approach to more precisely understanding the extent of natural allelic diversity, phylogenetic relationship and domestication pattern among wild and cultivated *Cicer* accessions. This approach will facilitate mining of novel allelic variants from wild accessions with beneficial traits for introgression breeding and broadening the genetic base of chickpea cultivars through inter-specific hybridization for their genetic enhancement.

The present study was therefore undertaken to discover, validate and genotype SNPs in 93 wild and cultivated *Cicer* accessions at a genome-wide scale using a GBS assay. The functional significance of identified genome-wide SNPs in trait association and their utility in studying the natural allelic diversity, population genetic structure, phylogeny and LD (linkage disequilibrium) pattern in wild and cultivated *Cicer* gene pools were determined.

## Results and discussion

### Genome-wide discovery and high-throughput genotyping of SNPs using a GBS assay

The sequencing of 96-plex *Ape*KI libraries through a GBS assay generated approximately 279.8 million raw sequence reads from 93 wild and cultivated *Cicer* accessions (Table S1). This altogether produced 248.3 million (88.7%) high-quality sequence reads (approximately 20-fold sequencing depth of coverage) that varied from 2.02 to 3.97 with a mean of 2.87 million reads per accession (Fig. S1A). On average, approximately 83.5% (ranging from 80.2 to 89.9%) and approximately 86.8% (80.1 to 91.5%) high-quality reads were evenly distributed across 93 *Cicer* accessions and mapped to unique physical locations on *desi* and *kabuli* reference draft genomes, respectively (Fig. S1B). These uniquely mapped non-redundant sequence reads effectively covered approximately 20.7% (153.6 Mb) and 21.5% (159.4 Mb) [ranging from 17.4% (129.3 Mb) to 34.3% (254.1 Mb)] of *desi* and *kabuli* chickpea genomes, respectively, with an estimated size of approximately 740 Mb. Notably, 107.6 (14.5%) and 126.7 (17.1%) Mb genomic regions of *desi* and *kabuli* chickpea genomes, respectively, represented by >90% and/or all 93 wild and cultivated *Cicer* accessions, were further utilized for genome-wide discovery and genotyping of SNPs among these accessions. These sequencing data have been submitted to the NCBI SRA (short read achieve) database (http://www.ncbi.nlm.nih.gov/sra) with accession number SRX971856, which will be made freely accessible on the 31^st^ of August 2015.

A total of 82489 high-quality SNPs were identified, including 38511 and 43978 (with read-depth: ≥10, SNP base quality: ≥20, missing data: <10% and heterozygosity: approximately 2% in each accession) from *desi* and *kabuli* genomes, respectively. These SNPs were discovered using both reference and *de novo*-based GBS assays (Table 1, S2, S3). The minor allele frequency (MAF) of GBS-based SNPs detected among 93 *Cicer* accessions varied from 2 to 21% with an average of 14%. The significant difference in the number of SNPs identified from *desi* and *kabuli* genomes could be due to uneven sequence assemblies and lengths of chromosomal pseudomolecules (*desi*: 124.37 Mb and *kabuli*: 347.24 Mb) between these two genomes^22^,^6^. This difference in sequenced fractions between *desi* and *kabuli* genomes encouraged us to employ both these genomes as references in the GBS assay to enrich the large-scale mining and genotyping of SNPs at a genome-wide scale in wild *Cicer* accessions. A little bit low-quality and uneven whole genome sequence assembly, including a smaller size chromosomal pseudomolecule of *desi* compared with *kabuli* has been documented recently by Ruperao *et al.*^49^. We therefore, provide details regarding the frequency and characteristics of reference and *de novo*-based GBS-SNPs mined from the *desi* genome in the Text S1 and Table S3.

**Figure 1:**
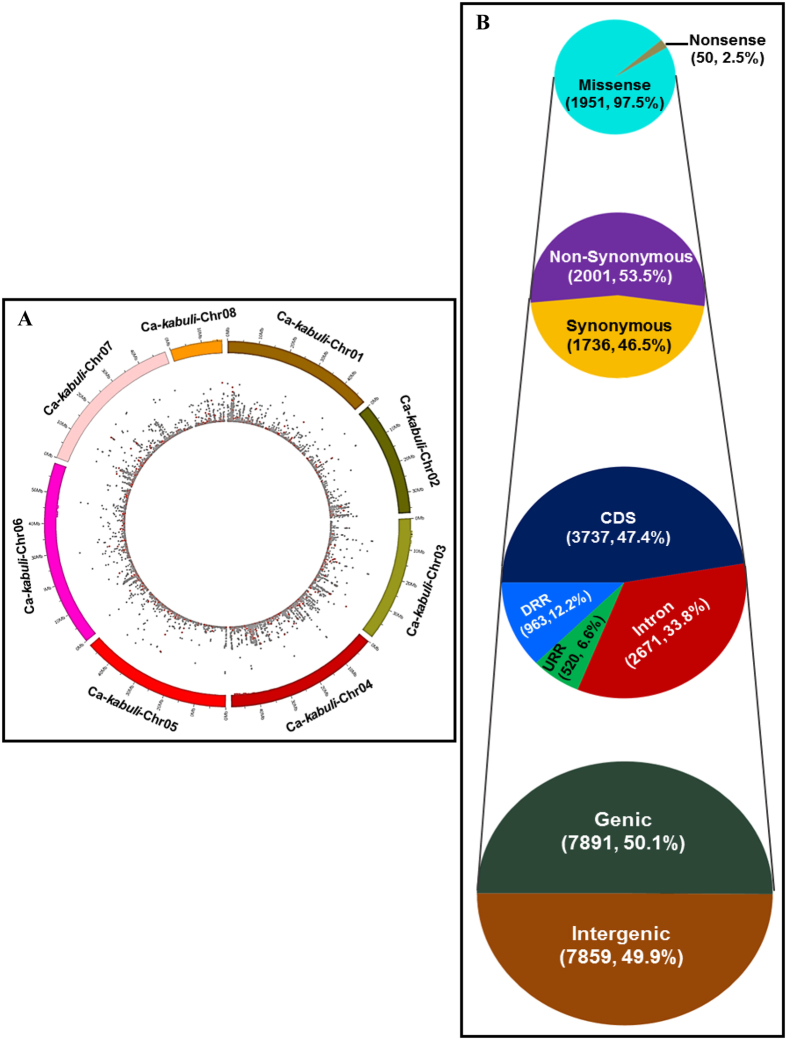
Genomic distribution and relative frequency of 11989 GBS-based SNPs structurally annotated on chickpea genome/genes. (**A**). The distribution of 11989 GBS-based genome-wide SNPs physically mapped on eight *kabuli* chickpea chromosomes is illustrated in the Circos circular ideogram. The outermost and innermost circles represent the different colours-coded chromosomes and distribution of SNPs, including non-synonymous SNPs (marked with red dots), respectively. (**B**) Frequency distribution of 15750 GBS-based SNPs mined in the intergenic regions and diverse coding and non-coding sequence components of 3371 genes annotated from *kabuli* genome. Number and proportion of SNPs, including synonymous and non-synonymous SNPs annotated in the coding as well as non-coding intronic and regulatory sequences of *kabuli* chickpea genes and intergenic regions are depicted. The URR (upstream regulatory region) and DRR (downstream regulatory region) of genes were defined as per the gene annotation information of *kabuli* genome (Varshney *et al.*^6^).

The GBS-based SNPs identified from *kabuli* chickpea consisted of 15,750 and 28,228 reference genome- and *de novo*-based SNPs, respectively. The reference genome-based SNPs included 11989 SNPs that were physically mapped on eight *kabuli* chromosomes with an average map density of 28.9 kb (Table 1, Fig. 1A). The remaining 3761 SNPs were physically mapped on *kabuli* genome scaffolds. The average SNP map density was highest on *kabuli* chromosome 4 (20.3 kb) and lowest on chromosome 5 (39.1 kb). A higher proportion of SNPs were physically mapped on *kabuli* chromosome 4 (20.2%, 2,418) (Table 1). The number of SNPs physically mapped on eight *kabuli* and *desi* (Text S1, Table S3, Fig. S2A) chromosomes revealed a direct correlation with their pseudomolecule size (bp). The constructed SNP-based physical maps of *desi* and *kabuli* chromosomes could serve as references for faster selection of genome-wide SNPs for manifold high-throughput marker-aided genetic analysis, including targeted mapping of genomes and trait-regulatory genes/QTLs in wild *Cicer* as well as comparative genome mapping involving chickpea and other legumes. The transitions were more frequent than transversions, which made up slightly more than half (54.7%; 45099 SNPs) of the 82489 identified SNPs (Table S4). A higher frequency of A/G transitions (51.2%, 23108 SNPs) compared to C/G (30.8%, 11,510) and G/T (30.2%, 11290) transversions in both *desi* and *kabuli* genomes was apparent. In total, 15750 reference *kabuli* genome-based SNPs have been submitted to NCBI dbSNP (http://www.ncbi.nlm.nih.gov/SNP/snp_viewTable.cgi?handle=NIPGR) with SNP submission (SS) accession numbers 1399931543 to 1399947292. Collectively, the integrated reference (*desi* and *kabuli*)- and *de novo*-based GBS strategies employed in our study for large-scale mining and high-throughput genotyping of SNPs in wild and cultivated *Cicer* accessions at a genome-wide scale could have multidimensional applicability in genomics-assisted breeding of chickpea.

### Structural and functional annotation of GBS-based SNPs

The structural annotation of 15750 *kabuli* reference genome-based SNPs detected 7,859 (49.9%) SNPs in the intergenic regions and 7,891 (50.1%) SNPs in 3,371 genes (Fig. 1B). The number of SNPs identified in the *kabuli* genes ranged from 1 to 7, with a mean of 2.3 SNPs/gene. The highest proportion (47.4%, 3,737 SNPs) of SNPs was annotated in the exons (CDS), followed by introns (33.8%, 2671) and DRRs (12.2%, 963 SNPs) and the lowest was found (6.6%, 520) in URRs (Fig. 1B). A total of 1,736 (46.5%) and 2,001 (53.5%) coding SNPs in 1,090 and 1,171 genes revealed synonymous and non-synonymous substitutions, respectively. The non-synonymous SNPs included 1,951 (97.5%) missense and 50 (2.5%) nonsense SNPs in 1,152 and 49 genes, respectively. The details regarding structural and functional annotation of 12,112 *desi* reference genome-based GBS-SNPs are noted in the Text S2 and illustrated in the Fig. S2B, S3A. The KOG-based functional annotation (excluding unknown and general functions) of SNP-carrying *kabuli* genes exhibited their primary roles in the signal transduction mechanism (13.7%), followed by transcription (11.2%) (Fig. S3B). The structurally and functionally annotated SNPs can be deployed to select functionally relevant gene-derived non-synonymous and regulatory SNPs for rapidly establishing efficient marker-trait linkages and identifying genes/QTLs controlling traits of agricultural importance in wild chickpea.

### Large-scale validation and polymorphic potential of SNPs

The comparison of 12112 *desi* (ICC 4958) reference genome-based SNPs mined from 93 wild and cultivated *Cicer* accessions with the available SNP database (31,019 SNPs) of four chickpea accessions (ICC 4958, ICC 4951, ICC 12968 and ICC 17160) revealed a correlation of 1716 SNPs (7.3%) between past genome resequencing and our present GBS data. These results are based on congruent physical positions and the type/nature of SNPs (Table S5). Among these, a maximum number of SNPs (831 SNPs, 10.7%) were found to be common and polymorphic between ICC 4958 and ICC 17160. The PCR amplicon sequencing of 96 selected SNPs identified by the reference genome- and *de novo*-based GBS assays efficiently validated 87 (90.1%) SNPs in a representative set of wild and cultivated *Cicer* accessions (Fig. S4). Therefore, a high degree of reproducibility (100%) as well as *in silico* and experimental validation success rate (90%) of SNPs discovered from 93 wild and cultivated *Cicer* accessions by use of both reference (*desi* and *kabuli*)- and *de novo*-based GBS approaches was evident. This suggests the robustness of the GBS assay in rapid discovery and high-throughput genotyping of high-quality and non-erroneous SNPs covering the whole genome in wild chickpea.

A total of 27862 SNPs (MAF ≥0.05), including 12112 and 15750 SNPs identified from *desi* and *kabuli* genomes, respectively, exhibited polymorphism among 93 diverse wild and cultivated *Cicer* accessions with a higher PIC (0.01 to 0.45, mean 0.35) and nucleotide diversity (θπ: 0.26 and θω: 0.25) (Table S6). Inter-specific polymorphism among species (23,794, 85.4% polymorphism and mean PIC: 0.41) was higher than that of intra-specific polymorphism within wild (15,790, 66.4% and 0.35) and cultivated (10,602, 38.1% and 0.27) species. The intra-specific polymorphic potential detected by SNPs was highest in *C. judaicum* (47.7% polymorphism, mean PIC: 0.39, θπ: 0.30 and θω: 0.29), followed by *C. reticulatum* (45.2%, 0.37, θπ: 0.27 and θω: 0.28) and lowest in *C. arietinum* (38.1%, 0.27, 0.16 and 0.18) (Table S6). The SNPs mapped on *desi* chromosomes (mean θπ: 0.28 and θω: 0.33) showed an almost comparable nucleotide diversity level with those mapped on the *kabuli* chromosomes (0.24 and 0.34) (Table 1, S3). The nucleotide diversity was highest on chromosomes 8 (mean θπ: 0.34 and θω: 0.33) and 4 (0.30 and 0.35) of *desi* and *kabuli*, respectively. The polymorphic potential detected by SNPs among 56 accessions representing the diverse species of the secondary gene pool (73.8% polymorphism, mean PIC: 0.39, θπ: 0.36 and θω: 0.33) was higher in contrast to that among the 36 accessions of the primary gene pool (65.7%, 0.31, 0.27 and 0.30) (Table S6). According to the geographical origin of 93 wild and cultivated *Cicer* accessions, 76 accessions belonging to different wild species were categorized under the Fertile Crescent (Turkey, Syrian Arab Republic, Jordan, Lebanon and Israel) type, while 17 accessions were grouped under Central Asia (India). Species/accessions originating from the Fertile Crescent had a higher SNP polymorphic potential (90.4% polymorphism, mean PIC: 0.37, θπ: 0.37 and θω: 0.35) than those from Central Asia (44.1%, 0.26, 0.24 and 0.28) (Table S6). The inter- and intra-specific polymorphic potential (66–85%) and PIC (0.35–0.41) detected by genome-wide GBS-based SNPs among 93 wild and cultivated *Cicer* accessions are higher/comparable to those estimated earlier using large-scale SNP, intron-spanning and microsatellite markers^29^,^39^,^41^,^43^,^47^. Therefore, an appreciable sum of polymorphic potential assessed by GBS-based SNPs covering the whole genome by their simultaneous high-throughput discovery and genotyping in wild and cultivated *Cicer* accessions can be used to assess the natural allelic diversity and domestication pattern in wild and cultivated chickpea.

### Genome-wide SNP-based molecular diversity and population genetic structure in wild chickpea

The use of 27862 genome-wide SNPs (MAF ≥0.05) for studying the molecular diversity among 93 wild and cultivated *Cicer* accessions belonging to six populations (POP I–POP VI) exhibited a wider range of genetic distances from 0.06 (POP I: ICC 4951 between POP I: ICCV 92311) to 0.64 (POP I: IC 296132 between POP IV: ILWC 32) with a mean of 0.43 (Table S7). This is higher/comparable to the molecular diversity level documented in earlier studies^41^,^44^,^45^,^50^ but lower than the data obtained using large-scale microsatellite and SNP markers^47^,^29^. The genetic distance among the accessions within wild species based on 15790 genome-wide SNPs varied from 0.06 (POP IV: ILWC 285 and POP IV: IG 136796) to 0.64 (POP II: ILWC 288 and POP VI: *C. microphyllum*) with an average of 0.42 (Table S7). The accessions belonging to POP III (mean genetic distance: 0.31) had the highest molecular diversity, followed by POP II (0.28), POP IV (0.23) and POP I (0.19) (Table 2). The species/accessions included under the secondary gene pool (mean genetic distance: 0.28) exhibited greater genetic diversity than those in the primary gene pool (0.21). The accessions/species originating from the Fertile Crescent (mean genetic distance: 0.27) possessed greater diversity than those from Central Asia (0.20). The SNPs revealing a wider genetic base and broader molecular diversity among wild and cultivated *Cicer* accessions could have significance in the selection of useful diverse parental wild and cultivated accessions as well as in the precise identification of true inter-specific hybrids in introgression breeding programs. Consequently, it will be helpful to scan the transfer of target genomic (gene) regions governing diverse agronomic traits (yield contributing and abiotic/biotic stress tolerance traits) from wild *Cicer* gene pools^51^ into the backgrounds of cultivated (*desi* and *kabuli*) species for their genetic improvement. The GBS-based SNPs developed at a genome-wide scale have the potential to distinguish among wild and cultivated *Cicer* accessions and would thus be of importance for chickpea variety improvement.

**Figure 2:**
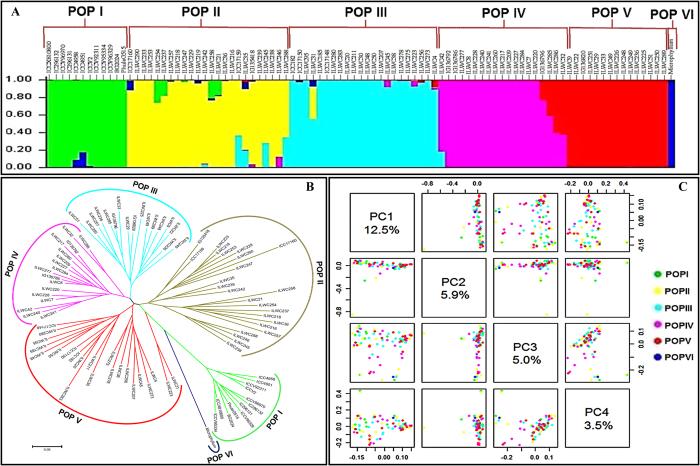
Genome-wide SNP-based molecular diversity, phylogeny and population genetic structure among 93 wild and cultivated *Cicer* accessions. (**A**) Population genetic structure among 93 wild and cultivated accessions using 27862 genome-wide SNPs. These SNPs assigned 93 accessions into six populations (POP I, POP II, POP III, POP IV, POP V and POP VI) that majorly grouped as per their species and gene pools of origination. The accessions represented by vertical bars along the horizontal axis were classified into K colour segments based on their estimated membership fraction in each K cluster. Six diverse colours represent different population groups based on optimal population number K = 6. (**B**) Unrooted phylogram illustrating the genetic relationships (Nei’s genetic distance) among 93 wild and cultivated accessions belonging to seven *Cicer* species using 27862 genome-wide SNPs. The phylogenetic tree clearly differentiated 93 accessions into six diverse groups, which correspond to their species and gene pools of origination. (**C**) Principal component analysis (PCA) differentiating the 93 wild and cultivated accessions belonging to seven *Cicer* species into six populations (POP I, POP II, POP III, POP IV, POP V and POP VI) as determined by population genetic structure. The PC1, PC2, PC3, PC4, PC5 and PC6 explained 10.2%, 7.8%, 3.7%, 3.4%, 2.7%, 2.4% and 2.3% of the total variance, respectively.

The determination of the population genetic structure among 93 wild and cultivated *Cicer* accessions using 27862 genome-wide SNPs at varying levels of possible population numbers (K = 1 to 10) with 20 replications revealed the most apparent inflection of average LnP(D) (log-likelihood) at one of the best replicates of K = 6. The population numbers (K) were further validated using the second order statistics of STRUCTURE by ΔK estimation. Overall, these analyses support the classification of 93 accessions into six distinct populations (POP I–VI) with a high-resolution population structure (Fig. 2A). POP I consisted of 12 cultivated *desi* and *kabuli* accessions of *C. arietinum*. POP II consisted of 24 wild accessions from *C. reticulatum* (16) and *C. echinospermum* (8). POP III, IV, V and VI included accessions from *C. judaicum* (22 accessions), *C. bijugum* (18), *C. pinnatifidum* (16) and *C. microphyllum* (1), respectively. The high-resolution assignment of 93 accessions into six populations was comparable with their clustering patterns and phylogeny obtained by the neighbor-joining unrooted phylogram (Fig. 2B) and PCA (Fig. 2C). The classification of species/accessions representing members of primary (*C. arietinum*, *C. reticulatum* and *C. echinospermum*) and secondary (*C. bijugum*, *C. pinnatifidum* and *C. judaicum*) gene pools into six diverse populations is comparable with previous molecular diversity, population genetic structure and evolutionary studies^6^,^29^,^36^,^38^,^39^,^40^,^41^,^42^,^44^,^45^,^46^,^47^,^52^,^53^,^54^,^55^. The inclusion of accessions from wild annual *Cicer* species (*C. reticulatum* and *C. echinospermum*) into one population (POP II) infers that geographical origin (Turkey), including phenotypic characteristics (growth habits), rather than species/gene pools of origination had a greater impact on the clustering patterns in a wild population^51^.

The ability of SNPs to detect polymorphism among the six populations revealed that the highest polymorphic potential occurred in POP III (mean PIC: 0.39, MAF: 0.21, θπ: 0.30 and θω: 0.29), followed by POP II and lowest in POP I (Table 2). A narrow genetic base and lower molecular diversity among cultivated *desi* and *kabuli* accessions of *C. arietinum* (POP I) was observed despite being closely related to the more diverse *C. reticulatum* and *C. echinospermum* (POP II). This is possibly due to the combined impacts of sequential evolutionary bottlenecks (such as founder effects, adaption-based selection pressure and modern breeding efforts) during the evolutionary divergence and domestication of cultivated gene pools with wild gene pools in South Eastern Turkey^24^,^25^,^26^,^29^,^56^. These observations are well supported by our estimation of higher molecular diversity in the wild gene pools originating from the Fertile Crescent than the gene pools from Central Asia. The molecular genetic variation among and within six populations of 93 *Cicer* accessions using 27862 genome-wide SNPs revealed a wider level of significant genetic and population differentiation based on pair-wise F_ST_ (P < 0.001) that varied from 0.17 (POP I and POP V) to 0.48 (POP III and POP V) with an average of 0.39. Higher divergence between populations (F_ST_: 0.43) compared to that estimated among accessions within populations (0.35) was evident. The SNPs physically mapped on the *desi* chromosomes (F_ST_ varied from 0.02 to 0.42, mean: 0.28) showed a lower potential for population differentiation compared to those on *kabuli* chromosomes (0.02 to 0.47, mean: 0.37) (Fig. S5). The maximum average F_ST_ of 0.26 and 0.33 were detected by SNPs mapped on *desi* and *kabuli* chromosomes 8 and 4, respectively.

All 93 wild and cultivated *Cicer* accessions clearly belonged to a structured population, with approximately 93% inferred ancestry derived from one of the model-based populations and the remaining approximately 7% containing admixed ancestry. The highest admixed ancestry (approximately 9%) was observed in POP IV, followed by POP II (approximately 5%) and lowest in POP I (approximately 1%). The occurrence of admix ancestry among six populations gave clues regarding origination and domestication of wild and cultivated *Cicer* from a common ancestor along with founder crops at the Fertile Crescent (Eastern Mediterranean region) approximately 10000–12000 years ago^24^,^57^. Diverse admixture patterns observed among the six populations could have evolved through complex domestication patterns involving inter-crossing/introgression coupled with various strong adaptive selection pressures among wild and cultivated *Cicer* accessions after their subsequent evolutionary divergence from progenitors. POP IV (*C. bijugum*) had the highest admixed ancestry (approximately 20%) with POP V (*C. pinnatifidum*), which is expected because they both belong to secondary gene pools. POP I showed the greatest admixtures (approximately 10%) with POP II and POP VI. The admixture was not detected in 11.8% (11 accessions) of chickpea accessions, while 15% (14) of the accessions exhibited >10% admixed ancestry. The highest admixtures among the members of the primary gene pool POP I (*C. arietinum*) and POP II (*C. reticulatum* and *C. echinospermum*) reflect their close phylogenetic relationship during their domestication at the archaeological sites of South Eastern Turkey^24^,^29^. A higher admixed ancestry of POP VI (Indian originated perennial wild accession *C. microphyllum*) with POP I (*C. arietinum*) and POP II (*C. reticulatum* and *C. echinospermum*) primary gene pools is in line with an earlier study^29^.

To infer the precise domestication patterns among 93 wild and cultivated *Cicer* accessions, the molecular diversity level, phylogeny and population genetic structure-related information revealed by genome-wide GBS-based SNPs were correlated with their qualitative and quantitative agromorphological traits, evaluated at two diverse agro-climatic zones in North Western India. A wider level of phenotypic variation, for instance, in three quantitative traits, BN (5 to 64), PN (1 to 309) and SW (1.0 to 70 g), was observed among 93 wild and cultivated *Cicer* accessions belonging to six populations (POP I to POP VI) based on two years of multi-location replicated field data (Table S8). All 81 wild accessions belonging to five populations also had higher phenotypic diversity levels for three agromorphological traits (BN: 5–64, PN: 1–309 and SW: 1–18 g) (Table S8). A wider phenotypic diversity among *Cicer* accessions was further supported by their broader Euclidean genetic distance (1.56 to 7.89, mean: 4.63) and Shannon-Weaver diversity coefficient (0.26 to 0.79, mean: 0.48). The dendrogram and PCA-based clustering of *Cicer* accessions into six populations (POP I to POP VI) and trends of phenotypic diversity level observed among the six populations using the phenotypic diversity coefficient remained similar to those determined by the molecular diversity distance matrix and population structure using genome-wide GBS-based SNPs. A wider phenotypic diversity among wild *Cicer* gene pools might be due to the diverse geographical origination and domestication of accessions included under wild species across various agro-climatic regions of the world. The correlation between phenotypic (agromorphological) and molecular diversity-based domestication pattern across wild and cultivated accessions could assist us in deciphering the effect of natural adaptive selection at a genome-wide scale on *Cicer* gene pools that are adapted to diverse agro-climatic regions. This will eventually lead to the delineation of trait-associated target genomic regions and natural allelic variants functionally relevant for chickpea genetic enhancement.

Recently, the added advantages of selecting genome-wide markers from LD/haplotype blocks of chromosomes and their effective use in detecting a wider spectrum of natural allelic diversity have been well demonstrated in HapMap projects of many crop plants^58^,^59^,^60^. In this context, the genome-wide GBS-based SNP genotyping data utilized in our study for realistic estimation of molecular diversity, genetic structure, phylogeny and domestication pattern among 93 wild and cultivated *Cicer* accessions seem rational. This is relevant considering the suitability of genome-wide GBS-based SNPs in distinct differentiation of these accessions belonging to wild and cultivated gene pools into six populations and correspondence of phylogenetic relationships with their species/gene pools and geographical origination. This precise diversity-related information generated by us could eventually facilitate diverse genomics-assisted breeding applications, including association and genetic (QTL) mapping to identify functionally relevant genes/QTLs regulating important agronomic traits in wild chickpea.

### Genome-wide and population-specific LD patterns in wild chickpea

The LD estimates (average r^2^) and extent of LD decay using all possible pair-combinations of the 4672 and 11989 genome-wide *desi* and *kabuli* SNPs (physically mapped on eight chromosomes) were determined among 93 wild and cultivated *Cicer* accessions belonging to six populations (POP I-POP VI). In the entire population and across eight *desi* and *kabuli* chickpea chromosomes, a proportion of 4.4–21% SNP-pairs exhibited significant LD (P < 0.0001) (Table 3, S9), indicating a moderate LD level in a diversity panel of wild and cultivated chickpea accessions. The significant LD (%) was highest in *desi* (13.3%) and *kabuli* (10.2%) chromosome 4. The LD estimates ranged from 0.53 in POP I to 0.85 in POP III with an average of 0.74 (Table 4). The LD estimates varied from 0.36 in *desi* chromosomes 4 and 7 to 0.67 in chromosome 2 with an average of 0.52, while in *kabuli*, it ranged from 0.38 in chromosome 7 to 0.67 in chromosome 1 with a mean density of 0.48 (Table 3, S9). The primary gene pool (0.80) had a higher LD estimate than the secondary gene pool (0.76). The LD estimate in the species/accessions originating from the Fertile Crescent (0.73) was higher than that in the species/accessions originating from Central Asia (0.59) (Table 5).

**Figure 3:**
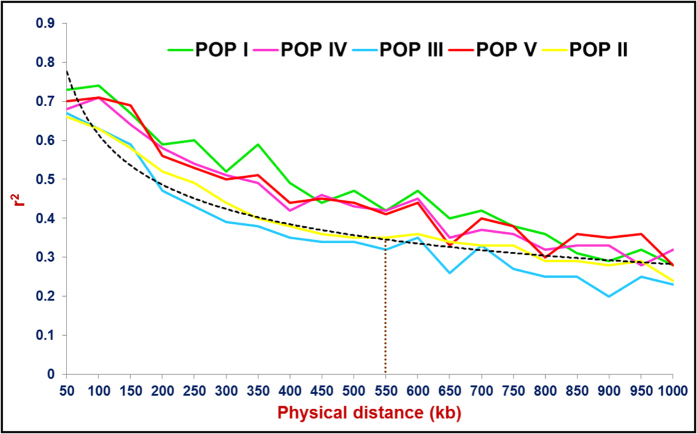
LD decay (mean r^2^) estimated in five structured populations (POP I–POP V) using 11989 *kabuli* genome (chromosome)-derived GBS-SNPs. The average r^2^ estimated among SNPs spaced with uniform 50 kb physical intervals from 0 to 1000 kb are illustrated by the plotted curved lines. The LD decay for POP VI could not be calculated due to inclusion of only one wild accession within this population group. The dotted lines were plotted between pooled r^2^ (across entire five populations) and physical distance (kb) based on nonlinear regression model considering the r^2^ = 1 at marker physical distance of 0 kb and the trend of LD decay were estimated in wild and cultivated *Cicer* accessions.

The LD decay of 4672 *desi* and 11989 *kabuli* genome-based SNPs was determined individually across six populations by pooling the r^2^ estimates across eight chromosomes and plotting their average r^2^ against the 50 kb uniform physical distance of 0–1000 kb (Fig. 3, S6). A non-linear regression curve depicted a decreasing trend of LD decay with an increase in the physical distance (kb) in the six populations. No significant decay of LD (r^2^ ≤ 0.1) was observed in any of the six populations (POP I–POP VI) up to a 1000 kb physical distance for both *desi* and *kabuli* chromosomes (Fig. 3, S6). However, the r^2^ decreased to half of its maximum value at an approximately 450–500 kb physical distance in *desi* chromosomes and 500–550 kb in *kabuli* chromosomes (Fig. 3, S6). The LD estimates and chromosomal LD decay estimated in the six populations using the genome-wide GBS-based SNPs are much higher compared to those reported earlier in cross-pollinated^58^,^61^,^62^,^63^,^64^,^65^ and self-pollinated^66^,^67^ plant species.

These findings reflect the substantial reduction of genetic diversity in wild and cultivated *Cicer* gene pools compared to that of other self- and cross-pollinated plant species, which is possibly due to four sequential evolutionary bottlenecks during the domestication of chickpea^24^,^25^,^26^,^27^. The diverse populations POP III (*C. judaicum*) and POP II (*C. reticulatum* and *C. echinospermum*) exhibited almost comparable LD decay (400–450 kb in *desi* and *kabuli* chromosomes) with other less diverse populations (450–550 kb) (Fig. 3, S6). These observations overall suggest that the SNP genome coverage/density as well as the molecular diversity, genetic structure and domestication pattern had significant contributions towards shaping the LD patterns in wild and cultivated *Cicer* gene pools^68^,^69^. The number as well as characteristics of wild and cultivated *Cicer* accessions included under a population could also be an important contributing factor for such comparable LD decay between cultivated and wild *Cicer* gene pools. However, the effective correlation of potential bias introduced by sequence reads mapping procedures and/or the non-randomness of missing GBS-based SNP data scanned from diverse genotyped accessions with their degree of LD decay at a genome-wide scale cannot be overruled. The population and chromosome-specific LD patterns determined in 93 wild and cultivated *Cicer* accessions by use of genome-wide GBS-based SNPs could assist us in anticipating the marker density required to more precisely estimate natural allelic diversity over various genomic regions and for efficient high-resolution genome-wide association study (GWAS) and genetic/QTL mapping, which will result in the identification of potential trait-regulatory genes/QTLs in chickpea.

### Functional significance of genome-wide SNPs

**Figure 4:**
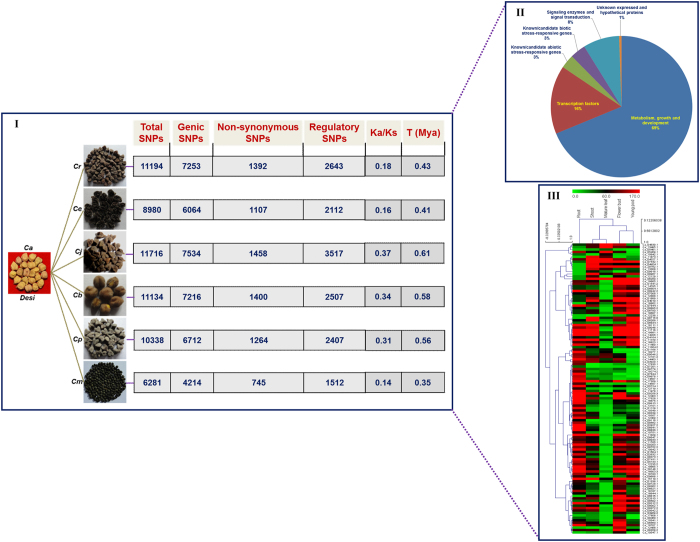
The functional relevance of non-synonymous and regulatory SNPs-carrying genes differentiating the accessions belonging to six wild *Cicer* species from accessions of cultivated *C. arietinum desi* (I) demonstrated by their non-synonymous (Ka) to synonymous (Ks) substitution rates and time of divergence (million years ago/Mya). Functional annotation of non-synonymous and regulatory SNPs-carrying genes discriminated the wild species/accessions from *desi* (II) accessions. The differential expression profiles (preferential and tissue-specific expression) of non-synonymous and regulatory SNPs-carrying genes discriminated the wild accessions from *desi* (III) accessions assayed in diverse vegetative and reproductive tissues of ICC 4958 are represented through Hierarchical cluster display. The average log signal expression values of genes in various tissues are marked with a colour scale at the top. The low, medium and high level of gene expression are indicated with green, black and red colors, respectively. At the top and right side of expression map, the tissues and genes, respectively utilized for expression profiling are mentioned. *Ca*: *C. arietinum*, *Cr*: *C. reticulatum*, *Ce*: *C. echinospermum*, *Cj*: *C. judaicum*, *Cb*: *C. bijugum*, *Cp*: *C. pinnatifidum* and *Cm*: *C. microphyllum*.

**Figure 5:**
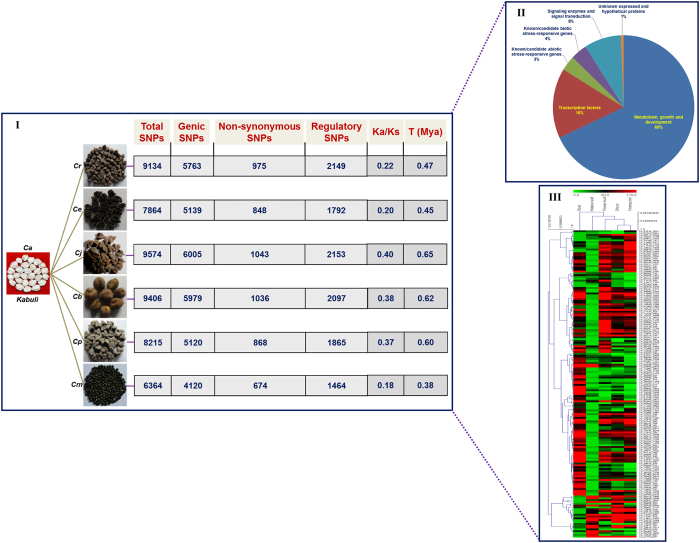
The functional relevance of non-synonymous and regulatory SNPs-carrying genes differentiating the accessions belonging to six wild *Cicer* species from accessions of cultivated *C. arietinum kabuli* (I) demonstrated by their non-synonymous (Ka) to synonymous (Ks) substitution rates and time of divergence (million years ago/Mya). Functional annotation of non-synonymous and regulatory SNPs-carrying genes discriminated the wild species/accessions from *kabuli* (II) accessions. The differential expression profiles (preferential and tissue-specific expression) of non-synonymous and regulatory SNPs-carrying genes discriminated the wild accessions from *kabuli* (III) accessions assayed in diverse vegetative and reproductive tissues of ICC 4958 are represented through Hierarchical cluster display. The average log signal expression values of genes in various tissues are marked with a colour scale at the top. The low, medium and high level of gene expression are indicated with green, black and red colors, respectively. At the top and right side of expression map, the tissues and genes, respectively utilized for expression profiling are mentioned. *Ca*: *C. arietinum*, *Cr*: *C. reticulatum*, *Ce*: *C. echinospermum*, *Cj*: *C. judaicum*, *Cb*: *C. bijugum*, *Cp*: *C. pinnatifidum* and *Cm*: *C. microphyllum*.

To understand the functional significance of the SNPs identified from 93 wild and cultivated *Cicer* accessions at a genome-wide scale, the SNPs, including non-synonymous and regulatory SNPs differentiating accessions belonging to each of six wild species (with potential known sources of yield contributing and stress tolerance traits), from accessions of cultivated *desi* and *kabuli* were documented systematically (Figs 4I and 5I). For instance, a total of 11194 SNPs (7253 genic SNPs), including 1392 non-synonymous and 2643 regulatory SNPs discriminating the accessions belonging to wild *C. reticulatum* species from *desi* cultivated *C. arietinum* species were identified (Fig. 4I). Likewise, 9574 SNPs (6005 genic SNPs), including 1043 non-synonymous and 2153 regulatory SNPs showing differentiation between wild *C. judaicum* and sensitive *kabuli* cultivated *C. arietinum* species were detected (Fig. 5I). The identified informative SNP-carrying genes (1800 genes between *desi* vs. wild species and 1925 genes between *kabuli* vs. wild species) were functionally annotated. Furthermore, differential expression pattern of these genes were determined through *in silico* digital expression profiling using the global transcript profiling data from the same genes available in diverse vegetative and reproductive *desi* accession (ICC 4958) tissues.

A significant proportion of these regulatory and non-synonymous SNP-carrying genes corresponded to transcription factors (16%), abiotic (3%)/biotic (3–4%) stress-responsive proteins and signal transduction enzymes (8%) (Figs 4II and 5II). Notably, 294 and 205 SNPs revealing non-synonymous (missense and nonsense) amino acid substitutions within the functional domains encoding transcription factors and biotic/abiotic stress-responsive genes discriminated the wild *Cicer* species/accessions from sensitive accessions of cultivated *desi* and *kabuli*, respectively (Fig. S4A, S4B). Selected 586 *desi* and 800 *kabuli* non-synonymous and regulatory SNP-containing genes, respectively exhibited significant differential expression (≥two-fold) in diverse vegetative and reproductive tissues of ICC 4958 (Figs 4III and 5III). A pronounced differential expression (≥10-fold) of these 119 *desi* and 168 *kabuli* SNP-carrying genes was apparent. Approximately 28–35% of these genes with SNPs had preferential and tissue-specific expression (≥4-fold), particularly in the roots, flower buds and young pods of ICC 4958 (Figs 4III and 5III). The significance of non-synonymous and regulatory SNPs in governing differential expression (particularly tissue-specific expression) and the possible transcriptional mechanism of genes associated with multiple agronomic traits have been demonstrated in rice, soybean and chickpea^70^,^71^,^72^,^73^,^74^.

To assess the potential of identified genome-wide SNPs for fine-mapping/map-based cloning of genes underlying QTLs, the markers linked to/flanking the 20 known QTLs for yield component and stress tolerance traits mapped previously on diverse inter- and intra-specific genetic linkage maps^75^,^76^,^77^,^78^,^79^,^80^,^81^,^82^,^83^,^84^,^85^,^86^,^87^ were selected. The genetic positions of markers flanking these known QTL intervals were compared/correlated with those of a high-density inter-specific genetic linkage map (ICC 4958 × PI 489777)^88^, which was used earlier as a reference for constructing physical maps and chromosome pseudomolecules for the *kabuli* genome^6^. The integration of genetic position-related information of the QTLs mapped on the reference inter-specific genetic linkage map (ICC 4958 × PI 489777) with that of physical map of *kabuli* chromosome pseudomolecules resulted in the ability to define the physical positions of markers flanking the 10 known stress tolerance trait-regulating QTL intervals mapped on five *kabuli* chromosomes.

The structural and functional annotation as well as *in silico* digital expression profiling (using the available global transcriptome sequencing data in diverse vegetative and reproductive tissues of ICC 4958) of SNP-carrying genes harboring these 10 known QTLs revealed significant differential expression, including preferential and tissue-specific expression of some of the selected genes with non-synonymous and regulatory SNPs. For instance, one “*QTL-hotspot*” region [ICCM0249 (54.9 cM)-GA24 (79.2 cM)] governing drought tolerance traits, particularly the root traits (58.2% PVE) mapped on an intra-specific genetic map (ICC 4958 × ICC 1882) of linkage group (LG) 4, was selected to evaluate the potential of our identified genome-wide GBS-based SNPs for fine mapping. A major genomic region (9102618–16180940 bp spanning 7.08 Mb on chromosome 4) harboring this robust QTL was defined by integrating that region with inter-specific genetic linkage (ICC 4958 × PI 489777) and physical map of the *kabuli* genome. The comprehensive GBS-based SNP analysis at this 7.08 Mb target QTL interval among 93 wild and cultivated *Cicer* accessions detected 375 SNPs. The structural annotation of these SNPs on the *kabuli* genome revealed the presence of 161 SNPs in the intergenic regions with 214, including 36 (29 genes) non-synonymous and 58 (31) regulatory, SNPs in the 60 genes. The functional annotation of 60 non-synonymous and regulatory SNP-carrying genes exhibited maximum correspondence to transcription factors (25%) and stress-responsive genes (33%). The differential expression analysis of 60 non-synonymous and regulatory SNP-containing genes based on *in silico* digital expression profiling showed pronounced differential up-/down-regulation (2 to 18.5-fold) of 51 genes in at least one of the tissues of ICC 4958 compared to others. The preferential and tissue-specific expression (≥4-fold) in the roots compared to the leaves of ICC 4958 was evident. The LD estimates and LD decay using 439 SNPs (with significant LD at MAF ≥0.05) identified and annotated on a 7.08 Mb drought responsive QTL region of *kabuli* chromosome 4 were determined. The LD estimates in the QTL region varied from 0.09 to 0.52 with a mean of 0.21, which is much lower than the LD estimates measured on all of *kabuli* chromosome 4 (mean LD: 0.39) using 2418 SNPs. The LD decay (r^2^ decreased to half of its maximum value) determined at a 200 kb physical distance from the target QTL is much faster compared to all eight *kabuli* chromosomes, including *kabuli* chromosome 4 (500–550 kb).

The estimation of the proportionate distribution of Ka to Ks substitution rates across SNP-carrying genes conserved between each of the wild and cultivated *Cicer* species depicted that a larger fraction (>90%) of such genes had Ka/Ks < 1.0, indicating negative/purifying selection pressure (Figs 4I and 5I). This is consistent with the average fraction of non-synonymous to synonymous SNPs (Ka/Ks < 1.0) estimated previously in chickpea genes/transcripts^6^,^22^,^89^,^90^. The Ka/Ks was highest between *C. arietinum* [*desi* (0.37)/*kabuli* (0.40)] and *C. judaicum*, and lowest between *C. arietinum* [*desi* (0.14)/*kabuli* (0.18)] and *C. microphyllum*. The time of divergence estimation based on synonymous substitution rates (Ks) among conserved SNP-carrying genes between each of the wild and cultivated *Cicer* species revealed the highest Ks between *C. arietinum* (*desi*/*kabuli*) and *C. judaicum*, giving their approximate divergence time at 0.61 and 0.65 Mya, respectively (Figs 4I and 5I). This was followed by that between *C. arietinum* [*desi* (0.58 Mya)/*kabuli* (0.62 Mya)] and *C. bijugum* and by the minimum between *C. arietinum* [*desi* (0.35 Mya)/*kabuli* (0.38 Mya)] and *C. microphyllum* (Figs 4I and 5I). The Ka/Ks-based estimates of divergence time measured among wild and cultivated *Cicer* species (specifically between *C. arietinum* and *C. reticulatum*) is consistent with a number of previous studies^6^,^22^,^89^,^90^.

The wild accessions constituting six annual and perennial *Cicer* species from diverse geographical origins selected for genome-wide discovery and high-throughput SNP genotyping using GBS assay had considerable phenotypic diversity with regard to multiple agromorphological and disease resistance traits based on their multi-location replicated phenotyping at two diverse agro-climatic zones of North Western India by Singh *et al.*^51^. For instance, the early flowering *C. reticulatum* accession ILWC 36 originating from Turkey (52 days), *C. judaicum* accessions (ILWC 4 and ILWC 273) from Lebanon (41 days) and *C. pinnatifidum* accessions (ILWC 226 and ILWC 251) also from Turkey (41 days) displayed multiple resistance against *Ascochyta* blight and root knot nematodes. One accession of *C. judaicum* (ILWC 256) originally from Jordan containing a higher number of pods/plant (284) vis-a-vis cultivated *desi* and *kabuli Cicer* species showed resistance to both *Ascochyta* blight and *Botrytis* gray mold. Therefore, large-scale non-synonymous and regulatory SNPs in the stress-responsive known/candidate genes and transcription factors differentiating the wild *Cicer* accessions (with potential known sources of various seed and pod yield contributing and stress tolerance traits) from sensitive cultivated *desi* and *kabuli* accessions scanned by us could potentially be utilized as functional markers for various large-scale genetic analyses in chickpea. The practical significance of these functional alleles (SNPs) mined at a genome-wide scale was apparent for fine-mapping/map-based cloning of genes underlying QTLs as well as for understanding natural allelic diversity and domestication pattern among diverse wild and cultivated *Cicer* species/accessions across gene pools based on their population genetic structure and LD patterning. These findings could help us to identify potential natural targets for adaptive trait genetic enhancement in cultivated gene pools that are domesticated in diverse agro-climatic regions. This would eventually accelerate genomics-assisted breeding, including hybridization breeding programs by introgressing diverse traits of agronomic importance from the wild *Cicer* gene pools into cultivated *Cicer* species for genetic improvement.

## Methods

### Mining and high-throughput genotyping of SNPs

For genome-wide discovery and genotyping of SNPs, 93 diverse *Cicer* accessions representing one annual cultivated *C. arietinum* (12 accessions), five annual wild species, namely *C. reticulatum* (16), *C. echinospermum* (8), *C. judaicum* (22), *C. bijugum* (19) and *C. pinnatifidum* (15) and one perennial species *C. microphyllum* were selected (Table S1). The genomic DNA from the young leaf samples of these accessions were isolated using QIAGEN DNeasy 96 Plant Kit (QIAGEN, USA). Two accessions (one each representing the *C. reticulatum* and *C. judaicum*) were used as biological replicates to evaluate the reproducibility of GBS-based SNPs. To constitute 96-plex GBS libraries, the genomic DNA of 95 accessions was digested with *Ape*KI and ligated to adapters carrying unique barcodes, while one sample (without genomic DNA) was considered as control. These libraries were pooled together and sequenced (100-bp single end) using Illumina HiSeq2000 adopting the methods of Elshire *et al.*^8^ and Spindel *et al.*^13^. The high-quality FASTQ sequence reads generated from accessions were de-multiplexed according to their unique barcodes and mapped to reference *desi* (ICC 4958^22^) and *kabuli* (CDC Frontier^6^) draft chickpea genome sequences using Bowtie v2.1.0. To identify accurate and high-quality SNPs (with SNP base-quality ≥20 supported by minimum sequence read-depth of 10) from 95 accessions, the sequence alignment map files generated from *desi* and *kabuli* genomes were analyzed individually using reference-based GBS pipeline of STACKS. The sequence reads remained unaligned on *desi* and *kabuli* reference genomes were further analyzed individually using the *de novo* genotyping approach of STACKS v1.0 (http://creskolab.uoregon.edu/stacks) to identify the high-quality and non-erroneous SNPs from 93 accessions.

### Annotation and validation of SNPs

To deduce the genomic distribution of GBS-based SNPs across chickpea chromosomes, the total SNPs, including non-synonymous SNPs were plotted individually based on their unique physical positions (bp) on eight chromosomes (pseudomolecules) of *desi* and *kabuli* genomes and visualised using Circos visualisation tool. For structural and functional annotation of SNPs in diverse coding and non-coding sequence components of genes and genomes (chromosomes/pseudomolecules and scaffolds), the physical positions (bp) of reference-based SNPs were correlated with the GFF of *desi*^22^ and *kabuli*^6^ chickpea genome annotation. The outputs were further analysed using the customised perl scripts, single-nucleotide polymorphism effect predictor (SnpEff v3.1h; http://snpeff.sourceforge.net), PFAM database v27.0 (http://pfam.sanger.ac.uk) and KOGnitor NCBI database (ftp://ftp.ncbi.nih.gov/pub/COG/KOG).

The SNP genotyping data obtained from 93 wild and cultivated *Cicer* accessions was correlated with an available *in silico* SNP database of four chickpea accessions (ICC 4958, ICC 4951, ICC 12968 and ICC 17160) based on their nature/types and congruent physical positions on the *desi* (ICC 4958) genome^22^ to validate the GBS-derived SNPs. For experimental validation of SNPs, the primers designed from 200-bp flanking sequences of 96 selected SNPs were PCR amplified using the genomic DNA of a representative set of wild and cultivated *Cicer* accessions. The PCR amplicons were sequenced and aligned to detect SNPs among accessions as per Kujur *et al.*^80^,^81^.

### Evaluation of polymorphic potential, molecular diversity and population genetic structure

The SNP genotyping information (minor allele frequency/MAF ≥5% with <10% missing data) generated from 93 wild and cultivated *Cicer* accessions was analysed with PowerMarker v3.51, MEGA v5.0 and TASSEL v3.0 (http://www.maizegenetics.net). Based on these outcomes, the PIC (polymorphism information content), average pair-wise nucleotide diversity (θπ) and Watterson’s estimator of segregating sites (θω) were measured and an unrooted neighbour-joining (NJ)-based phylogenetic tree (with 1000 bootstrap replicates) among accessions was constructed. To determine optimal value of population number (K) and population structure among 93 accessions, the SNP genotyping information was analyzed in STRUCTURE v2.3.4 following Kujur *et al.*^80^ and Saxena *et al.*^29^. The principal component analysis (PCA) among accessions was performed using TASSEL v3.0 and GAPIT.

### Assessment of phenotypic diversity

The replicated multilocation (New Delhi and Himachal Pradesh) field phenotyping information of 93 wild and cultivated *Cicer* accessions for diverse yield contributing qualitative and quantitative traits [plant pigmentation, plant hairiness, seed colour, days to flowering and maturity time, plant height, 100-seed weight (SW), branch number/plant (BN) and pod number/plant (PN)] were obtained from Singh *et al.*^51^. The detailed analysis of these agronomic traits based on diverse statistical measures (mean, standard deviation and coefficient of variation) were performed following Singh *et al.*^51^. The standardization of phenotypic data, estimation of genetic distance based on Euclidean coefficient (EUCLID), PCA, Shannon-Weaver diversity index and cluster analysis/tree construction among *Cicer* accessions were executed using NTSYS-pc V2.1 and POPGENE V1.32, and the methods of Zhang *et al.*^91^.

### Determination of LD patterns

To access the genome-wide and population-specific LD patterns (r^2^; average correlation coefficient among pairs of alleles across a pair of SNP loci) individually in *desi* and *kabuli* chickpea, the genotyping information of SNPs physically mapped on eight chromosomes were analysed using PLINK and the full-matrix approach of TASSEL, following Zhao *et al.*^59^ and Saxena *et al.*^29^. The LD decay was estimated by plotting average r^2^ against 50 kb uniform physical intervals across eight *desi* and *kabuli* chromosomes using aforementioned methods.

### Evaluation of functional significance of SNPs

To determine the functional significance of SNPs, the gene-derived SNPs (including non-synonymous coding and regulatory SNPs) differentiating accessions belonging to each of the wild *Cicer* species from accessions of cultivated (*desi* and *kabuli*) species were functionally annotated. To determine the differential expression pattern of these SNPs-carrying genes, *in silico* digital expression profiling was employed. For this, the global transcript profiling data (whole genome transcriptome sequencing) available for selected informative non-synonymous and regulatory SNPs-carrying genes in different vegetative (shoot, root and mature leaf) and reproductive (flower bud and young pod) tissues of chickpea (ICC 4958; Chickpea Transcriptome Database; http://www.nipgr.res.in/ctdb.html) were obtained. The differential expression profiling data assayed by SNPs-carrying genes in diverse vegetative and reproductive tissues of ICC 4958 was compared and a heat map was constructed using the TIGR MultiExperiment Viewer (MeV, http://www.tm4.org/mev.html).

The potential of GBS-based SNPs identified in our study for fine mapping/map-based cloning of QTLs was further assessed. For this, the genetic positions of markers linked/flanking the known QTLs regulating various yield component and stress tolerant traits mapped on eight LGs/chromosomes of diverse chickpea intra- and inter-specific genetic linkage maps were correlated and integrated with that of available high-resolution reference genetic linkage map (ICC 4958 × PI 489777)^88^ and physical map of *kabuli* genome^6^. The SNPs localized in the known trait-regulating QTL intervals based on their physical positions were structurally and functionally annotated. To determine the differential expression pattern of informative SNPs-carrying genes harbouring these known QTLs, the *in silico* digital expression profiling and heat map construction were performed following aforesaid methods.

### Estimation of divergence time among wild and cultivated chickpea

For estimation of divergence time, the synonymous (Ks) and non-synonymous (Ka) substitution rates of the aligned amino acid sequences and their corresponding cDNA sequences of SNPs-carrying genes conserved across each of seven wild and cultivated *Cicer* species were analyzed using the CODEML program in PAML interface tool of PAL2NAL (http://www.bork.embl.de/pal2nal). The time (million years ago, Mya) of divergence among *Cicer* species was measured using a synonymous mutation rate of λ substitutions per synonymous site per year as T = Ks/2λ (λ = 6.5 × 10^−9^)^92^.

## Additional Information

**How to cite this article**: Bajaj, D. *et al.* Genome-wide high-throughput SNP discovery and genotyping for understanding natural (functional) allelic diversity and domestication patterns in wild chickpea. *Sci. Rep.* **5**, 12468; doi: 10.1038/srep12468 (2015).

## Supplementary Material

Supplementary Information

## Figures and Tables

**Table 1:** Genomic distribution and nucleotide diversity potential of *kabuli* reference genome- and *de novo*-based GBS-SNPs among 93 wild and cultivated *Cicer* accessions.

				**Nucleotide diversity**	
**Chromosomes**	**Size (Mb) of chromosomes (pseudomolecules)**	**Number (%) of SNPs mapped**	**Average map density (kb)**	**θπ**	**θω**
Ca- *kabuli*-Chr01	48.36	1934 (16.1)	25.0	0.23	0.34
Ca- *kabuli*-Chr02	36.63	1118 (9.3)	32.8	0.28	0.34
Ca- *kabuli*-Chr03	39.99	1295 (10.8)	30.9	0.22	0.33
Ca- *kabuli*-Chr04	49.19	2418 (20.2)	20.3	0.30	0.35
Ca- *kabuli*-Chr05	48.17	1232 (10.3)	39.1	0.18	0.34
Ca- *kabuli*-Chr06	59.46	1900 (15.8)	31.3	0.21	0.33
Ca- *kabuli*-Chr07	48.96	1551 (12.9)	31.6	0.26	0.32
Ca- *kabuli*-Chr08	16.48	541 (4.5)	30.5	0.25	0.34
**Total**	**347.24**	**11989**	**28.9**	**0.24**	**0.34**
Ca-*kabuli*-Scaffold	NA	3761	NA	0.21	0.37
Ca-*kabuli*-*de novo*	NA	28228	NA	0.28	0.35
**Total**	**NA**	**43978**	**NA**	**0.24**	**0.33**

Ca-*kabuli*-Chr: *Cicer arietinum kabuli* chromosome.

θπ: Average pair-wise nucleotide diversity.

θω: Watterson’s estimator of segregating sites.

**Table 2:** Polymorphic and molecular diversity potential estimated within six structured populations using GBS-based genome-wide SNPs.

**Populations**	**Number of accessions**	**PIC (polymorphism information content)**	**Minor allele frequency (MAF)**	**Nucleotide diversity**		**Genetic distance**	
				**θπ**	**θω**	**Range**	**Mean**
POP I	12	0.08–0.38 (0.27)	0.10	0.16	0.18	0.06 (ICC4951–ICCV92311) to 0.32 (ICC4958–ICCV95334)	0.19
POP II	24	0.06–0.42 (0.36)	0.17	0.28	0.29	0.01 (ILWC253–ILWC242) to 0.44 (ILWC233–ILWC258)	0.28
POP III	15	0.04–0.45 (0.39)	0.21	0.30	0.29	0.11 (ILWC9–ILWC22) to 0.53 (ILWC51–ILWC9)	0.31
POP IV	19	0.05–0.38 (0.35)	0.18	0.26	0.27	0.07 (IG136796–ILWC285) to 0.39 (ILWC42–ILWC32)	0.23
POP V	22	0.06–0.37 (0.30)	0.16	0.25	0.26	0.12 (ICC182–ILWC20) to 0.40 (ILWC283–ILWC273)	0.21
POP VI	1	NA	NA	NA	NA	NA	NA
All POP	**93**	**0.01**–**0.45** (**0.35)**	**0.15**	**0.26**	**0.25**	**0.06 (ICC4951**–**ICCV92311) to 0.53 (ILWC51**–**ILWC9)**	**0.30**

**Table 3:** Chromosome-wise LD estimates in 93 wild and cultivated *Cicer* accessions using *kabuli* reference genome-based GBS-SNP.

**Chromosomes**	**Number of marker-pairs used**	a**Number of significant marker-pairs in LD**	b**Significant LD (%)**	**Extent of LD (r^2^)**
Ca-*kabuli*-Chr01	1165945	52455	4.5	0.67
Ca-*kabuli*-Chr02	624404	37643	6.0	0.40
Ca-*kabuli*-Chr03	836571	23832	2.8	0.42
Ca-*kabuli*-Chr04	1516603	155216	10.2	0.39
Ca-*kabuli*-Chr05	758296	7816	1.0	0.51
Ca-*kabuli*-Chr06	1148703	39687	3.5	0.58
Ca-*kabuli*-Chr07	1053625	33542	3.2	0.38
Ca-*kabuli*-Chr08	1053625	5822	4.0	0.46
**Total chromosomes**	**1019720**	**44500**	**4.4**	**0.48**

^a^Percentage of SNP marker locus-pairs in significant (P < 0.001) LD.

^b^Significant threshold (P < 0.001) at which pair-wise LD estimates is significant statistically.

**Table 4:** LD estimates in a structured population encompassing 93 wild and cultivated *Cicer* accessions using GBS-based SNPs.

**Populations**	**Number of marker-pairs used**	a**Number of significant marker-pairs in LD**	b**Significant LD (%)**	**Extent of LD (r^2^)**
POP I	831775	124766	15	0.53
POP II	831775	216260	26	0.82
POP III	831775	191308	23	0.85
POP IV	831775	158037	19	0.69
POP V	831775	141402	17	0.66
**Total populations**	**831775**	**174672**	**21**	**0.74**

^a^Percentage of SNP marker locus-pairs in significant (P < 0.001) LD.

^b^Significant threshold (P < 0.001) at which pair-wise LD estimates is significant statistically.

**Table 5:** Gene pools- and geographical area-wise LD estimates 93 wild and cultivated *Cicer* accessions using GBS-based SNPs.

**Characteristics**	**Number of marker-pairs used**	a**Number of significant marker-pairs in LD**	b**Significant LD (%)**	**Extent of LD (r^2^)**
Primary gene pool(36 accessions)	831775	24254	2.9	0.80
Secondary gene pool(76 accessions)	831775	32708	3.9	0.76
Geographical area(Central Asia)	831775	19131	2.3	0.59
Geographical area(Fertile Crescent)	831775	41650	5.0	0.73

^a^Percentage of SNP marker locus-pairs in significant (P < 0.001) LD.

^b^Significant threshold (P < 0.001) at which pair-wise LD estimates is significant statistically.
