# Supplementary material for: Genome-wide high-throughput SNP discovery and genotyping for understanding natural (functional) allelic diversity and domestication patterns in wild chickpea
Source: Sci Rep. 2015 Jul 24;5:12468. doi: 10.1038/srep12468 (PMC4513697; doi:10.1038/srep12468)
Supplement: Supplementary Information [file srep12468-s1.pdf]

Genome-wide high-throughput SNP discovery and genotyping for understanding natural (functional) allelic diversity and domestication patterns in wild chickpea

Deepak Bajaj[1a], Shouvik Das[1], Saurabh Badoni[1], Vinod Kumar[2], Mohar Singh[3], Kailash C. Bansal[3], Akhilesh K. Tyagi[1], Swarup K. Parida[1]*

[1]National Institute of Plant Genome Research (NIPGR), Aruna Asaf Ali Marg, New Delhi 110067, India

[2]National Research Centre on Plant Biotechnology (NRCPB), New Delhi-110012, India

[3]National Bureau of Plant Genetic Resources (NBPGR), New Delhi-110012, India

*Corresponding author

Text S1

The GBS-based SNPs identified from *desi* chickpea consisted of 12112 and 26399 reference genome- and *de novo*-based SNPs, respectively. The reference genome-based SNPs included 4672 SNPs that were physically mapped on eight *desi* chromosomes with an average map density of 26.6 kb (**Table S3, Fig. S2A**). The remaining 7440 SNPs were physically mapped on *desi* genome scaffolds. The average SNP map density was highest on *desi* chromosome 6 (22.2 kb) and lowest on chromosome 5 (35.9 kb). A higher proportion of SNPs were physically mapped on *desi* chromosome 4 (19.9%, 930 SNPs) (**Table S3**).

Text S2

The structural annotation of 12112 *desi* reference genome-based SNPs identified 5071 (41.9%) SNPs in the intergenic regions and 7041 (58.1%) SNPs in 2827 genes (**Fig. S2B**). The number of SNPs detected in the *desi* genes ranged from 1 to 6, with a mean of 2.5 SNPs/gene. The detailed annotation of SNPs in diverse sequence components of genes showed the occurrence of a maximum proportion (44.4%, 3123 SNPs) of SNPs in the exons (CDS), followed by introns (35.9%, 2531) and 1000-bp downstream regulatory regions (DRRs) (12.8%, 900) and a minimum (6.9%, 487) in the 2000-bp upstream regulatory regions (URRs) (**Fig. S2B**). The coding SNPs included 1838 (58.9%) SNPs within 894 genes revealed synonymous substitutions, while 1285 (41.1%) SNPs in 878 genes showed non-synonymous substitutions. The non-synonymous SNPs comprised of 1258 (97.9%) missense SNPs in 864 genes and 27 (2.1%) nonsense SNPs in 26 genes (**Fig. S2B**). The KOG-based functional annotation (excluding unknown and general functions) of *desi* genes with SNPs showed their primary roles in signal transduction mechanisms (15.9% 13.7%), followed by transcription (11.8%) (**Fig. S3A**).

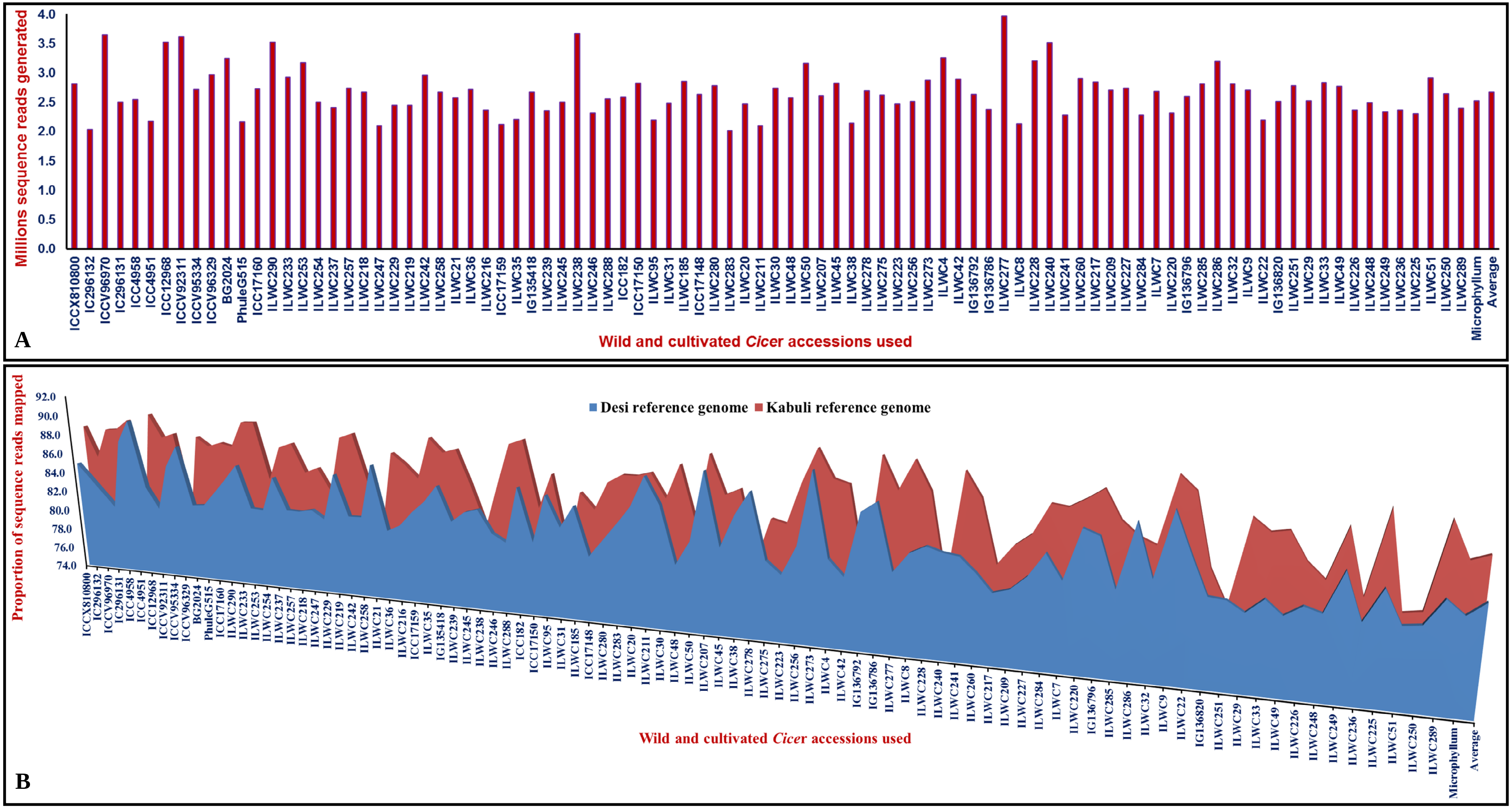

Fig. S1: (A) Frequency distribution of millions sequence reads generated from each of the 93 wild and cultivated *Cicer* accessions using a 96-plex GBS assay and proportion of these sequence reads mapped on *desi* and *kabuli* reference genomes (B).

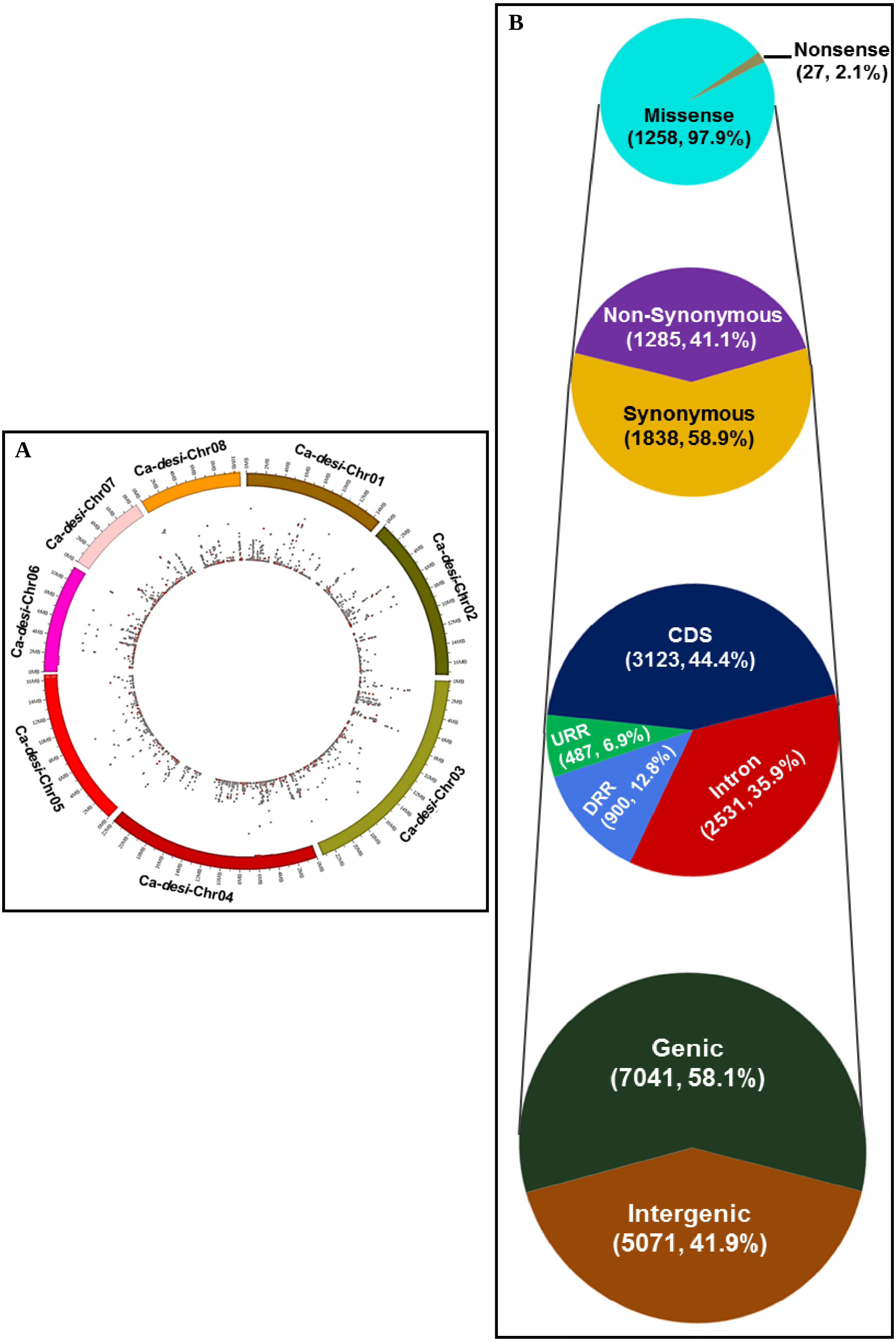

Fig. S2: The distribution of 4672 GBS-based genome-wide SNPs physically mapped on eight *desi* chickpea chromosomes are illustrated in the Circos circular ideogram (A). The outermost and innermost circles represent the different colours-coded chromosomes and distribution of SNPs, including non-synonymous SNPs (marked with red dots), respectively. (B) Frequency distribution of 12112 GBS-based SNPs mined in the intergenic regions and diverse coding and non-coding sequence components of 2827 genes annotated from *desi* genome. Number and proportion of SNPs, including synonymous and non-synonymous SNPs annotated in the coding as well as non-coding intronic and regulatory sequences of *desi* chickpea genes and intergenic regions are depicted. The URR (upstream regulatory region) and DRR (downstream regulatory region) of genes were defined as per the gene annotation information of *desi* genome (Jain et al.[22]).

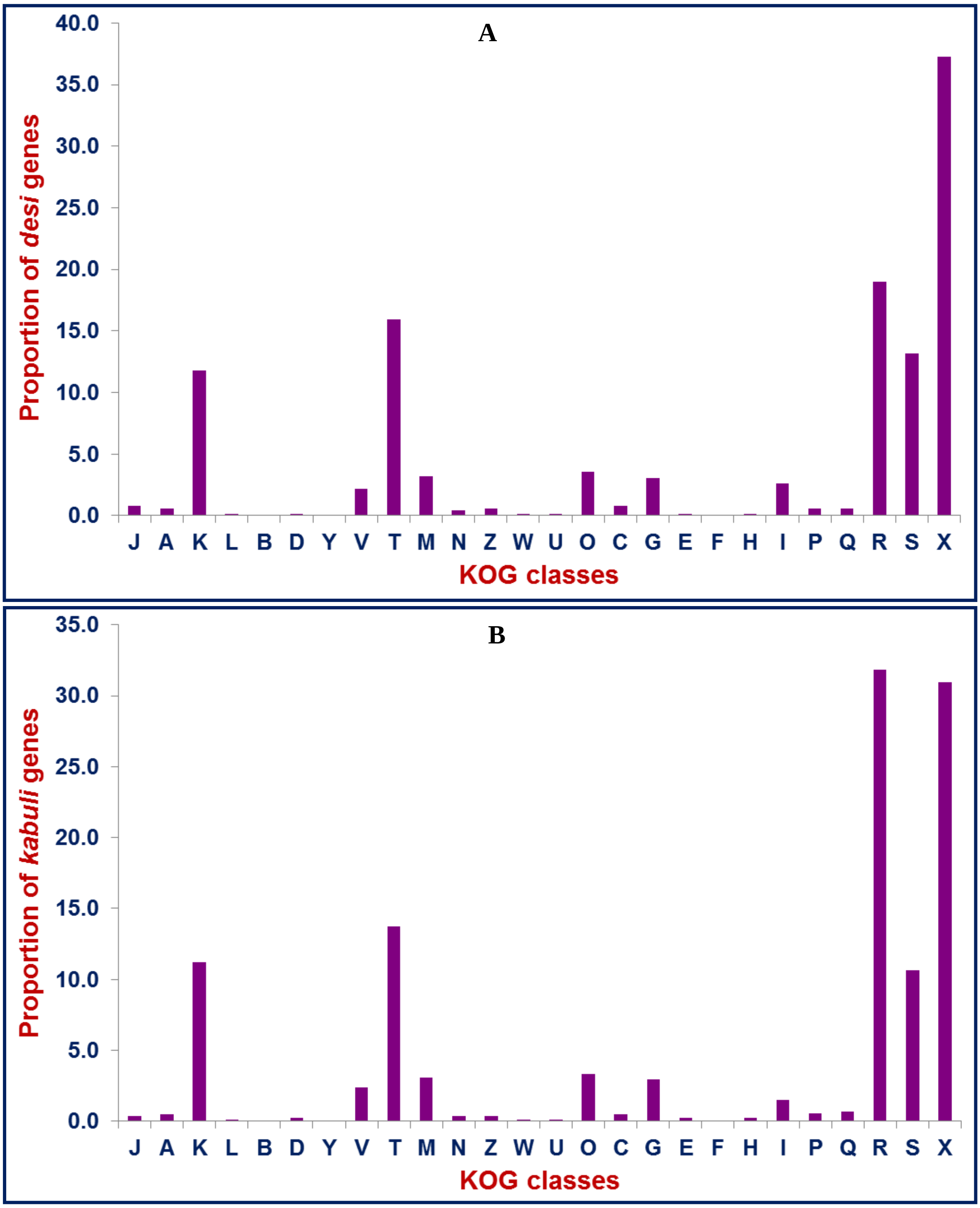

Fig. S3: KOG-based putative function determination of SNPs-carrying *desi* (A) and *kabuli* (B) genes. The diverse KOG classes are defined as J: Translation, ribosomal structure and biogenesis, A: RNA processing and modification, K: Transcription, L: Replication, recombination and repair, D: Cell cycle control, cell division and chromosome partitioning, Y: Nuclear structure, V: Defence mechanisms, T: Signal transduction mechanisms, M: Cell wall/membrane/envelope biogenesis, N: Cell motility, Z: Cytoskeleton, W: Extracellular structures, O: Posttranslational modification, protein turnover and chaperones, C: Energy production and conversion, G: Carbohydrate transport and metabolism, E: Amino acid transport and metabolism, H: Coenzyme transport and metabolism, I: Lipid transport and metabolism, P: Inorganic ion transport and metabolism, Q: Secondary metabolites biosynthesis, transport and catabolism, R: General function prediction only, S: Function unknown and X: Unnamed proteins.

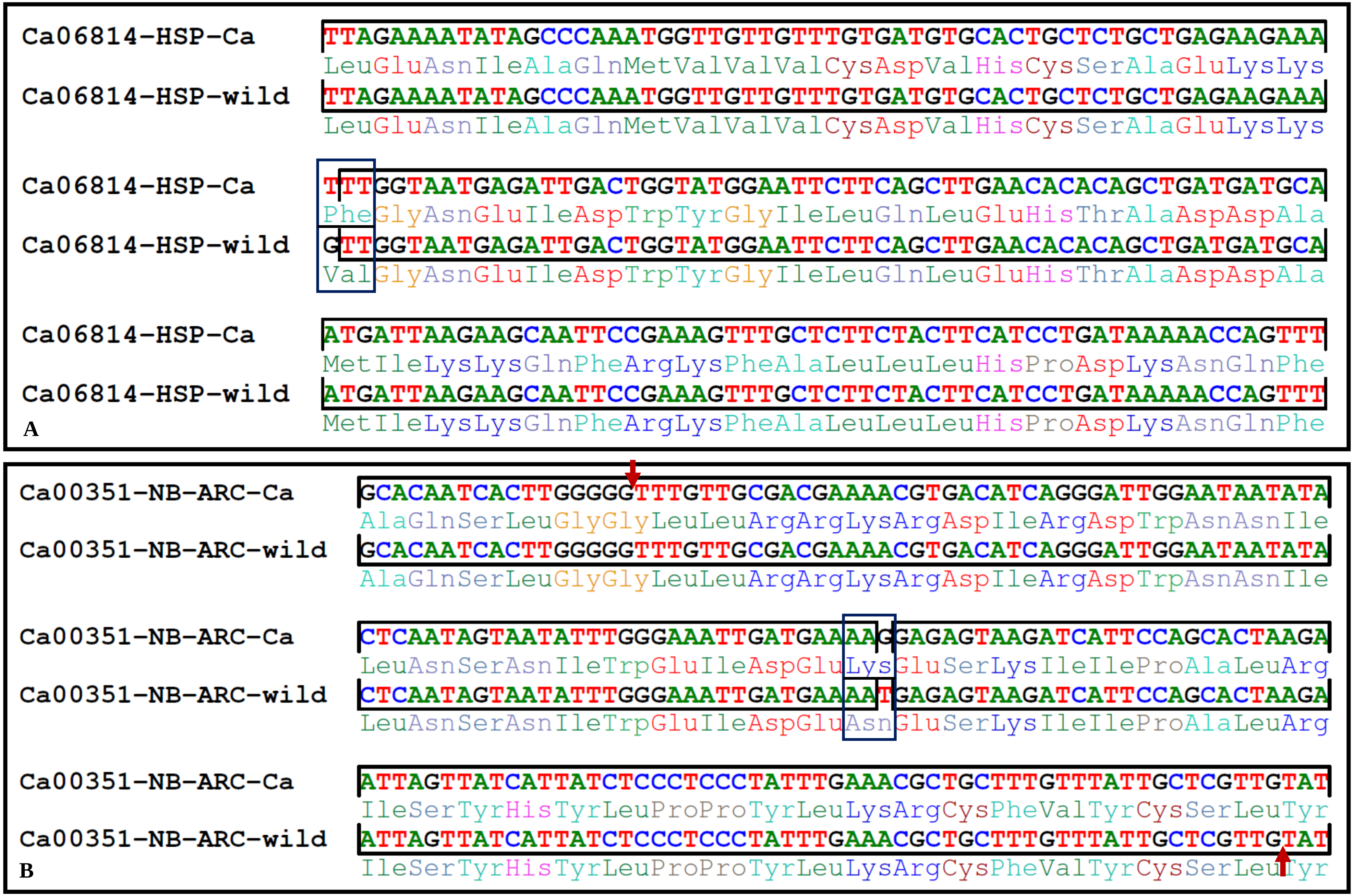

Fig. S4: A representative figure depicting the validation of SNP loci (T to G) and (G to T) revealing missense non-synonymous amino acid substitutions [phenylalanine (TTT) to valine (GTT)] and [lysine (AAG) to asparagine (AAT)] in the abiotic and biotic stress responsive HSP (A) and NB-ARC functional domain (marked with arrows) encoding (B) *kabuli* genes, respectively differentiated the known stress tolerant wild *Cicer* accessions from sensitive cultivated *C. arietinum* (Ca) accessions. The non-synonymous SNPs are highlighted with rectangular boxes.

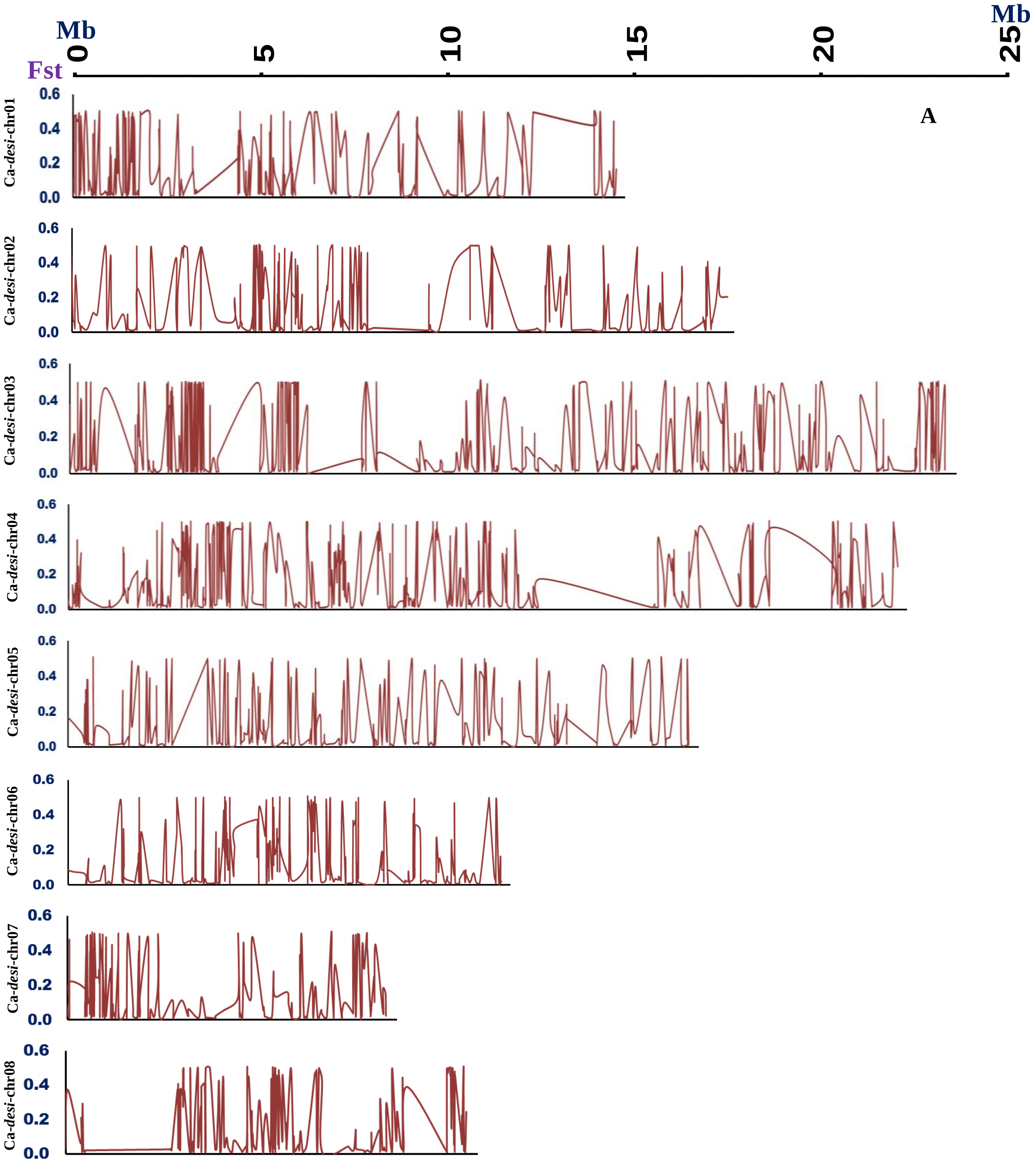
Mb
0
5
10
15
20
25
Mb
Fst
A
Ca-desi-chr01
Ca-desi-chr02
Ca-desi-chr03
Ca-desi-chr04
Ca-desi-chr05
Ca-desi-chr06
Ca-desi-chr07
Ca-desi-chr08
0.6
0.4
0.2
0.0

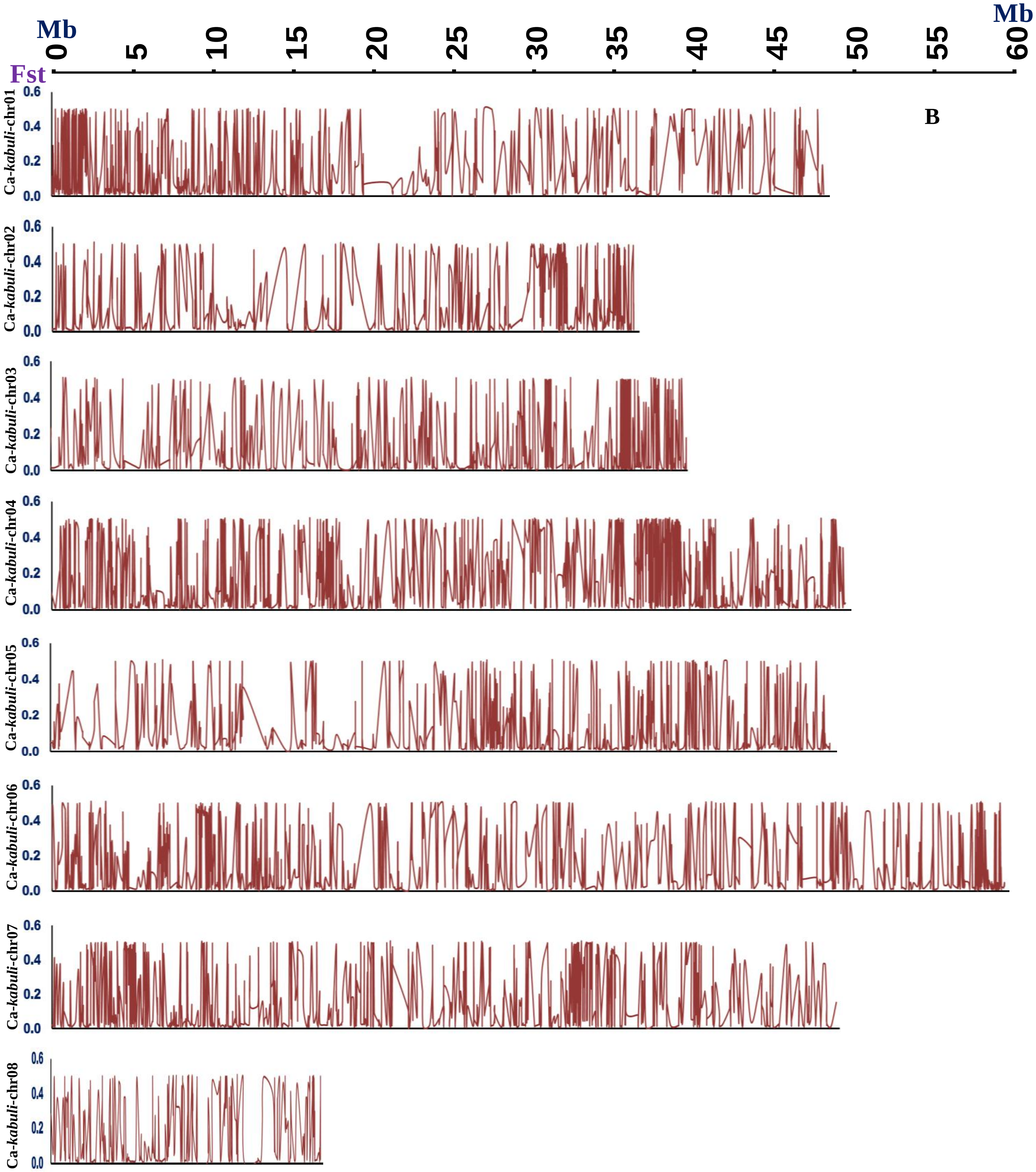

Fig. S5: Genomic distribution plots of F_{ST} in 93 wild and cultivated accessions determined across eight *desi* (A) and *kabuli* (B) chickpea chromosomes using 4672 and 11989 physically mapped GBS-based SNPs, respectively.

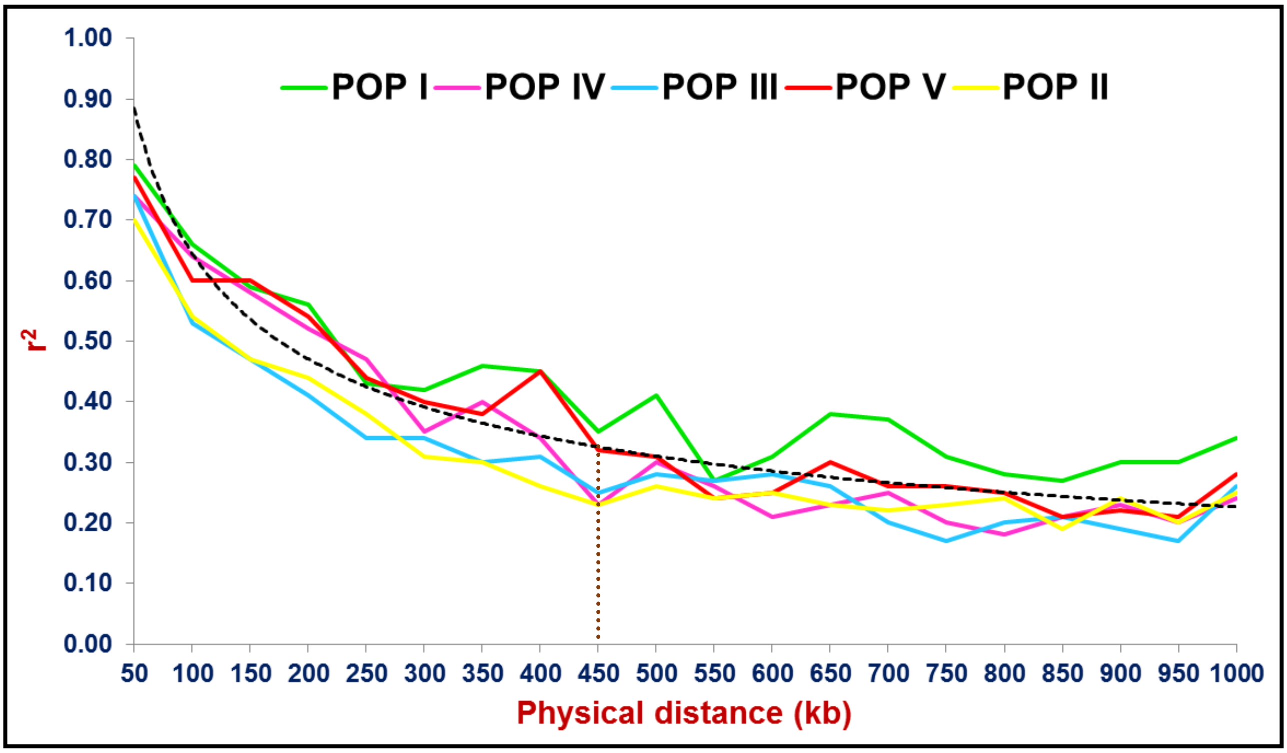

Fig. S6: LD decay (mean r^2) estimated in five structured populations (POP I - POP V) using 4672 *desi* genome (chromosome)-derived GBS-SNPs. The average r^2 estimated among SNPs spaced with uniform 50 kb physical intervals from 0 to 1000 kb are illustrated by the plotted curved lines. The LD decay for POP VI could not be calculated due to inclusion of only one wild accession within this population group. The dotted lines were plotted between pooled r^2 (across entire five populations) and physical distance (kb) based on nonlinear regression model considering the $r^2 = 1$ at marker physical distance of 0 kb and the trend of LD decay were estimated in wild and cultivated *Cicer* accessions.

Table S1: Wild and cultivated *Cicer* accessions selected for genome-wide discovery and high-throughput genotyping of SNPs using GBS-assay

S.N.	*Cicer* species	Accession No.	Cultivar types	Geographical origin
1	**C. arietinum (annual cultivated)**	ICCX810800	*desi*	India
2		IC296132	*desi*	India
3		ICCV96970	*desi*	India
4		IC296131	*desi*	India
5		ICC4958	*desi*	India
6		ICC4951	*desi*	India
7		ICC12968	*kabuli*	India
8		ICCV92311	*kabuli*	India
9		ICCV95334	*kabuli*	India
10		ICCV96329	*kabuli*	India
11		BG2024	*kabuli*	India
12		PhuleG515	*kabuli*	India
13	***C. reticulatum* (annual wild)**	ICC17160	Wild	Turkey
14		ILWC290	Wild	Turkey
15		ILWC233	Wild	Turkey
16		ILWC253	Wild	Turkey
17		ILWC254	Wild	Turkey
18		ILWC237	Wild	Turkey
19		ILWC257	Wild	Turkey
20		ILWC218	Wild	Turkey
21		ILWC247	Wild	Turkey
22		ILWC229	Wild	Turkey
23		ILWC219	Wild	Turkey
24		ILWC242	Wild	Turkey
25		ILWC258	Wild	Turkey
26		ILWC21	Wild	Turkey
27		ILWC36	Wild	Turkey
28		ILWC216	Wild	Turkey
29	***C. echinospermum* (annual wild)**	ICC17159	Wild	Turkey
30		ILWC35	Wild	Turkey
31		IG135418	Wild	Syrian Arab Republic
32		ILWC239	Wild	Turkey
33		ILWC245	Wild	Turkey
34		ILWC238	Wild	Turkey
35		ILWC246	Wild	Turkey
36		ILWC288	Wild	Turkey
37	***C. judaicum* (annual wild)**	ICC182	Wild	ICRISAT, India
38		ICC17150	Wild	Lebanon
39		ILWC95	Wild	ICRISAT, India
40		ILWC31	Wild	Jordan
41		ILWC185	Wild	ICRISAT, India
42		ICC17148	Wild	Lebanon
43		ILWC280	Wild	Syrian Arab Republic
44		ILWC283	Wild	Syrian Arab Republic
45		ILWC20	Wild	Israel
46		ILWC211	Wild	Syrian Arab Republic
47		ILWC30	Wild	Israel

48		ILWC48	Wild	Syrian Arab Republic
49		ILWC50	Wild	Syrian Arab Republic
50		ILWC207	Wild	Syrian Arab Republic
51		ILWC45	Wild	Syrian Arab Republic
52		ILWC38	Wild	Lebanon
53		ILWC278	Wild	Syrian Arab Republic
54		ILWC275	Wild	Lebanon
55		ILWC223	Wild	Lebanon
56		ILWC256	Wild	Jordan
57		ILWC273	Wild	Lebanon
58		ILWC4	Wild	Lebanon
59	**_C. bijugum_ (annual wild)**	ILWC42	Wild	Syrian Arab Republic
60		IG136792	Wild	Syrian Arab Republic
61		IG136786	Wild	Syrian Arab Republic
62		ILWC277	Wild	Syrian Arab Republic
63		ILWC8	Wild	Turkey
64		ILWC228	Wild	Turkey
65		ILWC240	Wild	Turkey
66		ILWC241	Wild	Turkey
67		ILWC260	Wild	Turkey
68		ILWC217	Wild	Turkey
69		ILWC209	Wild	Syrian Arab Republic
70		ILWC227	Wild	Turkey
71		ILWC284	Wild	Turkey
72		ILWC7	Wild	Turkey
73		ILWC220	Wild	Turkey
74		IG136796	Wild	Syrian Arab Republic
75		ILWC285	Wild	Turkey
76		ILWC286	Wild	Turkey
77		ILWC32	Wild	Turkey
78	**_C. pinnatifidum_ (annual wild)**	ILWC9	Wild	Turkey
79		ILWC22	Wild	Turkey
80		IG136820	Wild	Syrian Arab Republic
81		ILWC251	Wild	Turkey
82		ILWC29	Wild	Turkey
83		ILWC33	Wild	Turkey
84		ILWC49	Wild	Syrian Arab Republic
85		ILWC226	Wild	Turkey
86		ILWC248	Wild	Turkey
87		ILWC249	Wild	Turkey
88		ILWC236	Wild	Turkey
89		ILWC225	Wild	Turkey
90		ILWC51	Wild	Turkey
91		ILWC250	Wild	Turkey
92		ILWC289	Wild	Turkey
93	**_C. microphyllum_ (perennial wild)**	_microphyllum_	Wild	India

Table S2: Structural and functional annotation of 82489 SNPs discovered from 93 wild and cultivated *Cicer* accessions using integrated reference *desi* and *kabuli* genomes- and *de novo*-based GBS assay

SNP IDs	NCBI dbSNP accession IDs (NCBI_SS#)	Chromosomes	Physical positions (bp)	SNPs	Gene accession IDs	Sequence components of genome	Putative functions
Kabuli_wild_462	1399931543	*Ca_kabuli_Chr01*	462	G/C	NA	INTERGENIC	NA
Kabuli_wild_523	1399931544	*Ca_kabuli_Chr01*	523	G/C	NA	INTERGENIC	NA
Kabuli_wild_548	1399931545	*Ca_kabuli_Chr01*	548	G/C	NA	INTERGENIC	NA
Kabuli_wild_578	1399931546	*Ca_kabuli_Chr01*	578	G/A	NA	INTERGENIC	NA
Kabuli_wild_579	1399931547	*Ca_kabuli_Chr01*	579	T/C	NA	INTERGENIC	NA
Kabuli_wild_589	1399931548	*Ca_kabuli_Chr01*	589	A/C	NA	INTERGENIC	NA
Kabuli_wild_627	1399931549	*Ca_kabuli_Chr01*	627	C/A	NA	INTERGENIC	NA
Kabuli_wild_839	1399931550	*Ca_kabuli_Chr01*	839	A/T	NA	INTERGENIC	NA
Kabuli_wild_903	1399931551	*Ca_kabuli_Chr01*	903	G/T	NA	INTERGENIC	NA
Kabuli_wild_927	1399931552	*Ca_kabuli_Chr01*	927	C/A	NA	INTERGENIC	NA
Kabuli_wild_46421	1399931553	*Ca_kabuli_Chr01*	46421	C/T	Gene_Ca_00004	UPSTREAM	NA
Kabuli_wild_98992	1399931554	*Ca_kabuli_Chr01*	98992	G/T	Gene_Ca_00007	INTRON	RNA recognition motif domain
Kabuli_wild_108979	1399931555	*Ca_kabuli_Chr01*	108979	T/G	Gene_Ca_00008	INTRON	NA
Kabuli_wild_108984	1399931556	*Ca_kabuli_Chr01*	108984	G/A	Gene_Ca_00008	INTRON	NA
Kabuli_wild_129401	1399931557	*Ca_kabuli_Chr01*	129401	A/G	Gene_Ca_00009	INTRON	Oligo peptide transporter
Kabuli_wild_215000	1399931558	*Ca_kabuli_Chr01*	215000	A/T	Gene_Ca_00017	INTRON	Protein kinase,catalytic domain
Kabuli_wild_215006	1399931559	*Ca_kabuli_Chr01*	215006	C/T	Gene_Ca_00017	INTRON	Protein kinase,catalytic domain
Kabuli_wild_215027	1399931560	*Ca_kabuli_Chr01*	215027	T/C	Gene_Ca_00017	INTRON	Protein kinase,catalytic domain
Kabuli_wild_215029	1399931561	*Ca_kabuli_Chr01*	215029	C/A	Gene_Ca_00017	INTRON	Protein kinase,catalytic domain
Kabuli_wild_215048	1399931562	*Ca_kabuli_Chr01*	215048	C/T	Gene_Ca_00017	INTRON	Protein kinase,catalytic domain
Kabuli_wild_215053	1399931563	*Ca_kabuli_Chr01*	215053	T/G	Gene_Ca_00017	INTRON	Protein kinase,catalytic domain
Kabuli_wild_215058	1399931564	*Ca_kabuli_Chr01*	215058	C/T	Gene_Ca_00017	INTRON	Protein kinase,catalytic domain
Kabuli_wild_215066	1399931565	*Ca_kabuli_Chr01*	215066	G/T	Gene_Ca_00017	INTRON	Protein kinase,catalytic domain
Kabuli_wild_236588	1399931566	*Ca_kabuli_Chr01*	236588	T/C	NA	INTERGENIC	NA
Kabuli_wild_292962	1399931567	*Ca_kabuli_Chr01*	292962	T/C	Gene_Ca_00028	SYNONYMOUS_CODING	Proteasome component (PCI) domain
Kabuli_wild_292972	1399931568	*Ca_kabuli_Chr01*	292972	G/A	Gene_Ca_00028	NON_SYNONYMOUS_CODING	Proteasome component (PCI) domain
Kabuli_wild_292998	1399931569	*Ca_kabuli_Chr01*	292998	T/C	Gene_Ca_00028	SYNONYMOUS_CODING	Proteasome component (PCI) domain
Kabuli_wild_347620	1399931570	*Ca_kabuli_Chr01*	347620	A/C	Gene_Ca_00036	DOWNSTREAM	WD40 repeat
Kabuli_wild_372530	1399931571	*Ca_kabuli_Chr01*	372530	A/G	Gene_Ca_00038	INTRON	Protein of unknown function DUF803
Kabuli_wild_372865	1399931572	*Ca_kabuli_Chr01*	372865	G/A	Gene_Ca_00038	INTRON	Protein of unknown function DUF803
Kabuli_wild_389327	1399931573	*Ca_kabuli_Chr01*	389327	C/G	Gene_Ca_00040	NON_SYNONYMOUS_CODING	Transcription factor ,TCP
Kabuli_wild_406570	1399931574	*Ca_kabuli_Chr01*	406570	A/G	Gene_Ca_00043	SYNONYMOUS_CODING	Glycosyl-phosphatidylinositol-anchored,plant
Kabuli_wild_420890	1399931575	*Ca_kabuli_Chr01*	420890	G/T	NA	INTERGENIC	NA
Kabuli_wild_421346	1399931576	*Ca_kabuli_Chr01*	421346	T/C	NA	INTERGENIC	NA
Kabuli_wild_425076	1399931577	*Ca_kabuli_Chr01*	425076	A/G	Gene_Ca_00048	DOWNSTREAM	Nonaspanin(TM9SF)
Kabuli_wild_425149	1399931578	*Ca_kabuli_Chr01*	425149	T/C	Gene_Ca_00048	DOWNSTREAM	Nonaspanin(TM9SF)
Kabuli_wild_435259	1399931579	*Ca_kabuli_Chr01*	435259	A/G	NA	INTERGENIC	NA
Kabuli_wild_435263	1399931580	*Ca_kabuli_Chr01*	435263	A/T	NA	INTERGENIC	NA
Kabuli_wild_435264	1399931581	*Ca_kabuli_Chr01*	435264	G/T	NA	INTERGENIC	NA
Kabuli_wild_435274	1399931582	*Ca_kabuli_Chr01*	435274	G/T	NA	INTERGENIC	NA
Kabuli_wild_435280	1399931583	*Ca_kabuli_Chr01*	435280	T/C	NA	INTERGENIC	NA

SNP IDs	NCBI dbSNP accession IDs (NCBI_SS#)	Chromosomes	Physical positions (bp)	SNPs	Gene accession IDs	Sequence components of genome	Putative functions
Kabuli_wild_435297	1399931584	*Ca_kabuli_Chr01*	435297	G/A	NA	INTERGENIC	NA
Kabuli_wild_435304	1399931585	*Ca_kabuli_Chr01*	435304	G/T	NA	INTERGENIC	NA
Kabuli_wild_435310	1399931586	*Ca_kabuli_Chr01*	435310	C/T	NA	INTERGENIC	NA
Kabuli_wild_435338	1399931587	*Ca_kabuli_Chr01*	435338	T/C	NA	INTERGENIC	NA
Kabuli_wild_435343	1399931588	*Ca_kabuli_Chr01*	435343	C/A	NA	INTERGENIC	NA
Kabuli_wild_441286	1399931589	*Ca_kabuli_Chr01*	441286	C/T	Gene_Ca_00050	INTRON	Thiamin emonophosphate synthase
Kabuli_wild_441288	1399931590	*Ca_kabuli_Chr01*	441288	T/A	Gene_Ca_00050	INTRON	Thiamin emonophosphate synthase
Kabuli_wild_441343	1399931591	*Ca_kabuli_Chr01*	441343	T/A	Gene_Ca_00050	SYNONYMOUS_CODING	Thiamin emonophosphate synthase
Kabuli_wild_445947	1399931592	*Ca_kabuli_Chr01*	445947	T/G	Gene_Ca_00051	INTRON	Helicase,C-terminal
Kabuli_wild_446409	1399931593	*Ca_kabuli_Chr01*	446409	T/C	Gene_Ca_00051	SYNONYMOUS_CODING	Helicase,C-terminal
Kabuli_wild_446443	1399931594	*Ca_kabuli_Chr01*	446443	A/G	Gene_Ca_00051	NON_SYNONYMOUS_CODING	Helicase,C-terminal
Kabuli_wild_446484	1399931595	*Ca_kabuli_Chr01*	446484	C/T	Gene_Ca_00051	SYNONYMOUS_CODING	Helicase,C-terminal
Kabuli_wild_446558	1399931596	*Ca_kabuli_Chr01*	446558	G/T	Gene_Ca_00051	INTRON	Helicase,C-terminal
Kabuli_wild_450199	1399931597	*Ca_kabuli_Chr01*	450199	C/T	Gene_Ca_00051	INTRON	Helicase,C-terminal
Kabuli_wild_459968	1399931598	*Ca_kabuli_Chr01*	459968	T/G	Gene_Ca_00053	INTRON	CMP/dCMPdeaminase,zinc-binding
Kabuli_wild_463636	1399931599	*Ca_kabuli_Chr01*	463636	T/G	Gene_Ca_00053	INTRON	CMP/dCMPdeaminase,zinc-binding
Kabuli_wild_463687	1399931600	*Ca_kabuli_Chr01*	463687	G/A	Gene_Ca_00053	INTRON	CMP/dCMPdeaminase,zinc-binding
Kabuli_wild_464039	1399931601	*Ca_kabuli_Chr01*	464039	A/C	Gene_Ca_00053	SYNONYMOUS_CODING	CMP/dCMPdeaminase,zinc-binding
Kabuli_wild_464040	1399931602	*Ca_kabuli_Chr01*	464040	G/C	Gene_Ca_00053	SYNONYMOUS_CODING	CMP/dCMPdeaminase,zinc-binding
Kabuli_wild_464043	1399931603	*Ca_kabuli_Chr01*	464043	C/G	Gene_Ca_00053	SYNONYMOUS_CODING	CMP/dCMPdeaminase,zinc-binding
Kabuli_wild_554677	1399931604	*Ca_kabuli_Chr01*	554677	G/A	Gene_Ca_00064	INTRON	Glycosyltransferase,family8
Kabuli_wild_571002	1399931605	*Ca_kabuli_Chr01*	571002	A/G	Gene_Ca_00064	INTRON	Glycosyltransferase,family9
Kabuli_wild_571020	1399931606	*Ca_kabuli_Chr01*	571020	T/G	Gene_Ca_00064	INTRON	Glycosyltransferase,family10
Kabuli_wild_571044	1399931607	*Ca_kabuli_Chr01*	571044	T/G	Gene_Ca_00064	INTRON	Glycosyltransferase,family11
Kabuli_wild_591609	1399931608	*Ca_kabuli_Chr01*	591609	T/C	Gene_Ca_00066	INTRON	PeptidaseS16,Lonprotease,C-terminal
Kabuli_wild_609751	1399931609	*Ca_kabuli_Chr01*	609751	T/A	NA	INTERGENIC	NA
Kabuli_wild_661732	1399931610	*Ca_kabuli_Chr01*	661732	T/G	Gene_Ca_00079	DOWNSTREAM	NA
Kabuli_wild_696923	1399931611	*Ca_kabuli_Chr01*	696923	C/A	NA	INTERGENIC	NA
Kabuli_wild_712110	1399931612	*Ca_kabuli_Chr01*	712110	T/C	Gene_Ca_00086	NON_SYNONYMOUS_CODING	PeptidaseC50,separase
Kabuli_wild_741514	1399931613	*Ca_kabuli_Chr01*	741514	G/T	NA	INTERGENIC	NA
Kabuli_wild_741535	1399931614	*Ca_kabuli_Chr01*	741535	C/T	NA	INTERGENIC	NA
Kabuli_wild_742026	1399931615	*Ca_kabuli_Chr01*	742026	T/G	NA	INTERGENIC	NA
Kabuli_wild_742043	1399931616	*Ca_kabuli_Chr01*	742043	G/A	NA	INTERGENIC	NA
Kabuli_wild_750693	1399931617	*Ca_kabuli_Chr01*	750693	C/T	NA	INTERGENIC	NA
Kabuli_wild_777240	1399931618	*Ca_kabuli_Chr01*	777240	G/A	NA	INTERGENIC	NA
Kabuli_wild_777273	1399931619	*Ca_kabuli_Chr01*	777273	C/T	NA	INTERGENIC	NA
Kabuli_wild_790000	1399931620	*Ca_kabuli_Chr01*	790000	C/T	NA	INTERGENIC	NA
Kabuli_wild_790102	1399931621	*Ca_kabuli_Chr01*	790102	G/T	NA	INTERGENIC	NA
Kabuli_wild_864351	1399931622	*Ca_kabuli_Chr01*	864351	T/C	NA	INTERGENIC	NA
Kabuli_wild_864381	1399931623	*Ca_kabuli_Chr01*	864381	G/A	NA	INTERGENIC	NA
Kabuli_wild_864382	1399931624	*Ca_kabuli_Chr01*	864382	T/C	NA	INTERGENIC	NA
Kabuli_wild_866872	1399931625	*Ca_kabuli_Chr01*	866872	T/C	NA	INTERGENIC	NA
Kabuli_wild_876633	1399931626	*Ca_kabuli_Chr01*	876633	T/A	NA	INTERGENIC	NA
Kabuli_wild_881543	1399931627	*Ca_kabuli_Chr01*	881543	C/A	NA	INTERGENIC	NA

SNP IDs	NCBI dbSNP accession IDs (NCBI_SS#)	Chromosomes	Physical positions (bp)	SNPs	Gene accession IDs	Sequence components of genome	Putative functions
Kabuli_wild_881703	1399931628	*Ca_kabuli_Chr01*	881703	G/T	NA	INTERGENIC	NA
Kabuli_wild_890948	1399931629	*Ca_kabuli_Chr01*	890948	C/T	Gene_Ca_00110	INTRON	Calcium-bindingEF-hand
Kabuli_wild_892050	1399931630	*Ca_kabuli_Chr01*	892050	G/T	Gene_Ca_00110	INTRON	Calcium-bindingEF-hand
Kabuli_wild_892257	1399931631	*Ca_kabuli_Chr01*	892257	T/C	Gene_Ca_00110	SYNONYMOUS_CODING	Calcium-bindingEF-hand
Kabuli_wild_910486	1399931632	*Ca_kabuli_Chr01*	910486	T/A	NA	INTERGENIC	NA
Kabuli_wild_968054	1399931633	*Ca_kabuli_Chr01*	968054	G/C	Gene_Ca_00120	INTRON	Sulphate transporter/antisigma-factor antagonist STAS
Kabuli_wild_968075	1399931634	*Ca_kabuli_Chr01*	968075	T/A	Gene_Ca_00120	INTRON	Sulphate transporter/antisigma-factor antagonist STAS
Kabuli_wild_968079	1399931635	*Ca_kabuli_Chr01*	968079	C/A	Gene_Ca_00120	INTRON	Sulphate transporter/antisigma-factor antagonist STAS
Kabuli_wild_968116	1399931636	*Ca_kabuli_Chr01*	968116	C/T	Gene_Ca_00120	INTRON	Sulphate transporter/antisigma-factor antagonist STAS
Kabuli_wild_996439	1399931637	*Ca_kabuli_Chr01*	996439	T/C	Gene_Ca_00124	DOWNSTREAM	Beta-hydroxyacyl-(acyl-carrier-protein)dehydratase,FabA/FabZ
Kabuli_wild_1008895	1399931638	*Ca_kabuli_Chr01*	1008895	A/G	Gene_Ca_00126	DOWNSTREAM	Protein kinase,catalytic domain
Kabuli_wild_1009058	1399931639	*Ca_kabuli_Chr01*	1009058	A/C	NA	INTERGENIC	NA
Kabuli_wild_1018966	1399931640	*Ca_kabuli_Chr01*	1018966	C/G	NA	INTERGENIC	NA
Kabuli_wild_1023550	1399931641	*Ca_kabuli_Chr01*	1023550	C/T	NA	INTERGENIC	NA
Kabuli_wild_1032491	1399931642	*Ca_kabuli_Chr01*	1032491	A/C	Gene_Ca_00128	SYNONYMOUS_CODING	K+ potassium transporter
Kabuli_wild_1032518	1399931643	*Ca_kabuli_Chr01*	1032518	G/A	Gene_Ca_00128	INTRON	K+ potassium transporter
Kabuli_wild_1032598	1399931644	*Ca_kabuli_Chr01*	1032598	C/A	Gene_Ca_00128	INTRON	K+ potassium transporter
Kabuli_wild_1032734	1399931645	*Ca_kabuli_Chr01*	1032734	A/T	Gene_Ca_00128	NON_SYNONYMOUS_CODING	K+ potassium transporter
Kabuli_wild_1032898	1399931646	*Ca_kabuli_Chr01*	1032898	T/A	Gene_Ca_00128	INTRON	K+ potassium transporter
Kabuli_wild_1045095	1399931647	*Ca_kabuli_Chr01*	1045095	G/C	NA	INTERGENIC	NA
Kabuli_wild_1045119	1399931648	*Ca_kabuli_Chr01*	1045119	C/G	NA	INTERGENIC	NA
Kabuli_wild_1059209	1399931649	*Ca_kabuli_Chr01*	1059209	A/G	Gene_Ca_00129	NON_SYNONYMOUS_CODING	IQ motif,EF-handbinding site
Kabuli_wild_1059311	1399931650	*Ca_kabuli_Chr01*	1059311	A/G	Gene_Ca_00129	NON_SYNONYMOUS_CODING	IQ motif,EF-handbinding site
Kabuli_wild_1083388	1399931651	*Ca_kabuli_Chr01*	1083388	G/A	NA	INTERGENIC	NA
Kabuli_wild_1083403	1399931652	*Ca_kabuli_Chr01*	1083403	C/T	NA	INTERGENIC	NA
Kabuli_wild_1085228	1399931653	*Ca_kabuli_Chr01*	1085228	A/C	Gene_Ca_00134	INTRON	Peptidase C14,caspase catalytic
Kabuli_wild_1085230	1399931654	*Ca_kabuli_Chr01*	1085230	T/G	Gene_Ca_00134	INTRON	Peptidase C14,caspase catalytic
Kabuli_wild_1085238	1399931655	*Ca_kabuli_Chr01*	1085238	A/G	Gene_Ca_00134	INTRON	Peptidase C14,caspase catalytic
Kabuli_wild_1085254	1399931656	*Ca_kabuli_Chr01*	1085254	A/C	Gene_Ca_00134	INTRON	Peptidase C14,caspase catalytic
Kabuli_wild_1085263	1399931657	*Ca_kabuli_Chr01*	1085263	A/C	Gene_Ca_00134	INTRON	Peptidase C14,caspase catalytic
Kabuli_wild_1085293	1399931658	*Ca_kabuli_Chr01*	1085293	G/A	Gene_Ca_00134	INTRON	Peptidase C14,caspase catalytic
Kabuli_wild_1091442	1399931659	*Ca_kabuli_Chr01*	1091442	A/C	NA	INTERGENIC	NA
Kabuli_wild_1091504	1399931660	*Ca_kabuli_Chr01*	1091504	C/T	NA	INTERGENIC	NA
Kabuli_wild_1091534	1399931661	*Ca_kabuli_Chr01*	1091534	A/C	NA	INTERGENIC	NA
Kabuli_wild_1091548	1399931662	*Ca_kabuli_Chr01*	1091548	T/A	NA	INTERGENIC	NA
Kabuli_wild_1098703	1399931663	*Ca_kabuli_Chr01*	1098703	C/G	NA	INTERGENIC	NA
Kabuli_wild_1098789	1399931664	*Ca_kabuli_Chr01*	1098789	A/G	NA	INTERGENIC	NA
Kabuli_wild_1115668	1399931665	*Ca_kabuli_Chr01*	1115668	C/T	NA	INTERGENIC	NA
Kabuli_wild_1116043	1399931666	*Ca_kabuli_Chr01*	1116043	C/T	NA	INTERGENIC	NA
Kabuli_wild_1140313	1399931667	*Ca_kabuli_Chr01*	1140313	A/C	NA	INTERGENIC	NA
Kabuli_wild_1140376	1399931668	*Ca_kabuli_Chr01*	1140376	A/G	NA	INTERGENIC	NA
Kabuli_wild_1140405	1399931669	*Ca_kabuli_Chr01*	1140405	A/T	NA	INTERGENIC	NA
Kabuli_wild_1150973	1399931670	*Ca_kabuli_Chr01*	1150973	C/T	NA	INTERGENIC	NA
Kabuli_wild_1159383	1399931671	*Ca_kabuli_Chr01*	1159383	C/A	NA	INTERGENIC	NA

SNP IDs	NCBI dbSNP accession IDs (NCBI_SS#)	Chromosomes	Physical positions (bp)	SNPs	Gene accession IDs	Sequence components of genome	Putative functions
Kabuli_wild_1204160	1399931672	*Ca_kabuli_Chr01*	1204160	A/C	Gene_Ca_00148	INTRON	Terpenesynthase-like
Kabuli_wild_1204350	1399931673	*Ca_kabuli_Chr01*	1204350	A/G	Gene_Ca_00148	INTRON	Terpenesynthase-like
Kabuli_wild_1204357	1399931674	*Ca_kabuli_Chr01*	1204357	C/A	Gene_Ca_00148	INTRON	Terpenesynthase-like
Kabuli_wild_1204368	1399931675	*Ca_kabuli_Chr01*	1204368	C/G	Gene_Ca_00148	INTRON	Terpenesynthase-like
Kabuli_wild_1215024	1399931676	*Ca_kabuli_Chr01*	1215024	T/C	NA	INTERGENIC	NA
Kabuli_wild_1225244	1399931677	*Ca_kabuli_Chr01*	1225244	G/T	NA	INTERGENIC	NA
Kabuli_wild_1225345	1399931678	*Ca_kabuli_Chr01*	1225345	G/A	Gene_Ca_00151	INTRON	Peptidyl-prolylcis-transisomerase,FKBP-type,domain
Kabuli_wild_1225376	1399931679	*Ca_kabuli_Chr01*	1225376	C/A	Gene_Ca_00151	INTRON	Peptidyl-prolylcis-transisomerase,FKBP-type,domain
Kabuli_wild_1251800	1399931680	*Ca_kabuli_Chr01*	1251800	G/A	Gene_Ca_00154	INTRON	Carbonicanhydrase
Kabuli_wild_1269180	1399931681	*Ca_kabuli_Chr01*	1269180	C/A	Gene_Ca_00155	NON_SYNONYMOUS_CODING	Peptidase C48, SUMO/Sentrin/Ubl1
Kabuli_wild_1278854	1399931682	*Ca_kabuli_Chr01*	1278854	G/T	Gene_Ca_00158	DOWNSTREAM	Golgi apparatus membrane protein TVP15
Kabuli_wild_1278884	1399931683	*Ca_kabuli_Chr01*	1278884	G/T	Gene_Ca_00158	DOWNSTREAM	Golgi apparatus membrane protein TVP15
Kabuli_wild_1278997	1399931684	*Ca_kabuli_Chr01*	1278997	T/A	Gene_Ca_00158	DOWNSTREAM	Golgi apparatus membrane protein TVP15
Kabuli_wild_1295527	1399931685	*Ca_kabuli_Chr01*	1295527	T/G	NA	INTERGENIC	NA
Kabuli_wild_1335518	1399931686	*Ca_kabuli_Chr01*	1335518	A/C	Gene_Ca_00167	UPSTREAM	Pentatrico peptide repeat
Kabuli_wild_1385553	1399931687	*Ca_kabuli_Chr01*	1385553	C/A	NA	INTERGENIC	NA
Kabuli_wild_1385554	1399931688	*Ca_kabuli_Chr01*	1385554	G/T	NA	INTERGENIC	NA
Kabuli_wild_1390073	1399931689	*Ca_kabuli_Chr01*	1390073	A/G	NA	INTERGENIC	NA
Kabuli_wild_1411339	1399931690	*Ca_kabuli_Chr01*	1411339	C/T	NA	INTERGENIC	NA
Kabuli_wild_1411495	1399931691	*Ca_kabuli_Chr01*	1411495	A/G	NA	INTERGENIC	NA
Kabuli_wild_1411498	1399931692	*Ca_kabuli_Chr01*	1411498	T/A	NA	INTERGENIC	NA
Kabuli_wild_1411501	1399931693	*Ca_kabuli_Chr01*	1411501	C/T	NA	INTERGENIC	NA
Kabuli_wild_1428222	1399931694	*Ca_kabuli_Chr01*	1428222	C/G	NA	INTERGENIC	NA
Kabuli_wild_1428251	1399931695	*Ca_kabuli_Chr01*	1428251	T/G	NA	INTERGENIC	NA
Kabuli_wild_1428264	1399931696	*Ca_kabuli_Chr01*	1428264	T/G	NA	INTERGENIC	NA
Kabuli_wild_1434121	1399931697	*Ca_kabuli_Chr01*	1434121	A/G	Gene_Ca_00174	NON_SYNONYMOUS_CODING	DNA polymerase V
Kabuli_wild_1434875	1399931698	*Ca_kabuli_Chr01*	1434875	A/G	Gene_Ca_00175	DOWNSTREAM	Bromodomain
Kabuli_wild_1434883	1399931699	*Ca_kabuli_Chr01*	1434883	A/G	Gene_Ca_00175	DOWNSTREAM	Bromodomain
Kabuli_wild_1449848	1399931700	*Ca_kabuli_Chr01*	1449848	G/A	Gene_Ca_00177	SYNONYMOUS_CODING	Phox/Bem1p
Kabuli_wild_1449913	1399931701	*Ca_kabuli_Chr01*	1449913	T/G	Gene_Ca_00177	NON_SYNONYMOUS_CODING	Phox/Bem1p
Kabuli_wild_1449949	1399931702	*Ca_kabuli_Chr01*	1449949	G/A	Gene_Ca_00177	NON_SYNONYMOUS_CODING	Phox/Bem1p
Kabuli_wild_1449980	1399931703	*Ca_kabuli_Chr01*	1449980	A/G	Gene_Ca_00177	SYNONYMOUS_CODING	Phox/Bem1p
Kabuli_wild_1461574	1399931704	*Ca_kabuli_Chr01*	1461574	T/A	NA	INTERGENIC	NA
Kabuli_wild_1461600	1399931705	*Ca_kabuli_Chr01*	1461600	T/C	NA	INTERGENIC	NA
Kabuli_wild_1467056	1399931706	*Ca_kabuli_Chr01*	1467056	A/G	NA	INTERGENIC	NA
Kabuli_wild_1467123	1399931707	*Ca_kabuli_Chr01*	1467123	A/T	NA	INTERGENIC	NA
Kabuli_wild_1528325	1399931708	*Ca_kabuli_Chr01*	1528325	G/A	Gene_Ca_00185	INTRON	Inorganic pyrophosphatase
Kabuli_wild_1546679	1399931709	*Ca_kabuli_Chr01*	1546679	G/C	Gene_Ca_00187	SYNONYMOUS_CODING	NA
Kabuli_wild_1554144	1399931710	*Ca_kabuli_Chr01*	1554144	G/A	NA	INTERGENIC	NA
Kabuli_wild_1615004	1399931711	*Ca_kabuli_Chr01*	1615004	G/A	NA	INTERGENIC	NA
Kabuli_wild_1655012	1399931712	*Ca_kabuli_Chr01*	1655012	G/T	Gene_Ca_00203	NON_SYNONYMOUS_CODING	Kinesin,motordomain
Kabuli_wild_1655018	1399931713	*Ca_kabuli_Chr01*	1655018	G/A	Gene_Ca_00203	NON_SYNONYMOUS_CODING	Kinesin,motordomain
Kabuli_wild_1738179	1399931714	*Ca_kabuli_Chr01*	1738179	C/G	NA	INTERGENIC	NA
Kabuli_wild_1738185	1399931715	*Ca_kabuli_Chr01*	1738185	T/C	NA	INTERGENIC	NA

SNP IDs	NCBI dbSNP accession IDs (NCBI_SS#)	Chromosomes	Physical positions (bp)	SNPs	Gene accession IDs	Sequence components of genome	Putative functions
Kabuli_wild_1738341	1399931716	*Ca_kabuli_Chr01*	1738341	A/C	NA	INTERGENIC	NA
Kabuli_wild_1772813	1399931717	*Ca_kabuli_Chr01*	1772813	T/C	NA	INTERGENIC	NA
Kabuli_wild_1782760	1399931718	*Ca_kabuli_Chr01*	1782760	G/A	Gene_Ca_00224	INTRON	Zinc finger, RING-type
Kabuli_wild_1782793	1399931719	*Ca_kabuli_Chr01*	1782793	G/T	Gene_Ca_00224	INTRON	Zinc finger, RING-type
Kabuli_wild_1782808	1399931720	*Ca_kabuli_Chr01*	1782808	G/C	Gene_Ca_00224	INTRON	Zinc finger, RING-type
Kabuli_wild_1782929	1399931721	*Ca_kabuli_Chr01*	1782929	C/T	Gene_Ca_00224	INTRON	Zinc finger, RING-type
Kabuli_wild_1782930	1399931722	*Ca_kabuli_Chr01*	1782930	A/G	Gene_Ca_00224	INTRON	Zinc finger, RING-type
Kabuli_wild_1782948	1399931723	*Ca_kabuli_Chr01*	1782948	C/T	Gene_Ca_00224	INTRON	Zinc finger, RING-type
Kabuli_wild_1782953	1399931724	*Ca_kabuli_Chr01*	1782953	G/A	Gene_Ca_00224	INTRON	Zinc finger, RING-type
Kabuli_wild_1783207	1399931725	*Ca_kabuli_Chr01*	1783207	G/A	Gene_Ca_00224	INTRON	Zinc finger, RING-type
Kabuli_wild_1783277	1399931726	*Ca_kabuli_Chr01*	1783277	G/A	Gene_Ca_00224	INTRON	Zinc finger, RING-type
Kabuli_wild_1783280	1399931727	*Ca_kabuli_Chr01*	1783280	A/C	Gene_Ca_00224	INTRON	Zinc finger, RING-type
Kabuli_wild_1783343	1399931728	*Ca_kabuli_Chr01*	1783343	T/C	Gene_Ca_00224	INTRON	Zinc finger, RING-type
Kabuli_wild_1783388	1399931729	*Ca_kabuli_Chr01*	1783388	C/A	Gene_Ca_00224	INTRON	Zinc finger, RING-type
Kabuli_wild_1783389	1399931730	*Ca_kabuli_Chr01*	1783389	A/G	Gene_Ca_00224	INTRON	Zinc finger, RING-type
Kabuli_wild_1783923	1399931731	*Ca_kabuli_Chr01*	1783923	C/A	Gene_Ca_00224	NON_SYNONYMOUS_CODING	Zinc finger, RING-type
Kabuli_wild_1784006	1399931732	*Ca_kabuli_Chr01*	1784006	T/C	Gene_Ca_00224	SYNONYMOUS_CODING	Zinc finger, RING-type
Kabuli_wild_1788958	1399931733	*Ca_kabuli_Chr01*	1788958	T/A	NA	INTERGENIC	NA
Kabuli_wild_1789041	1399931734	*Ca_kabuli_Chr01*	1789041	C/T	NA	INTERGENIC	NA
Kabuli_wild_1789087	1399931735	*Ca_kabuli_Chr01*	1789087	T/C	NA	INTERGENIC	NA
Kabuli_wild_1802992	1399931736	*Ca_kabuli_Chr01*	1802992	C/G	NA	INTERGENIC	NA
Kabuli_wild_1825840	1399931737	*Ca_kabuli_Chr01*	1825840	G/A	Gene_Ca_00230	SYNONYMOUS_CODING	Zinc finger, RING-type
Kabuli_wild_1858073	1399931738	*Ca_kabuli_Chr01*	1858073	C/T	Gene_Ca_00233	INTRON	Inositol monophosphatase
Kabuli_wild_1858078	1399931739	*Ca_kabuli_Chr01*	1858078	T/C	Gene_Ca_00233	INTRON	Inositol monophosphatase
Kabuli_wild_1858084	1399931740	*Ca_kabuli_Chr01*	1858084	C/T	Gene_Ca_00233	INTRON	Inositol monophosphatase
Kabuli_wild_1858132	1399931741	*Ca_kabuli_Chr01*	1858132	T/A	Gene_Ca_00233	INTRON	Inositol monophosphatase
Kabuli_wild_1858156	1399931742	*Ca_kabuli_Chr01*	1858156	C/T	Gene_Ca_00233	INTRON	Inositol monophosphatase
Kabuli_wild_1858159	1399931743	*Ca_kabuli_Chr01*	1858159	G/A	Gene_Ca_00233	INTRON	Inositol monophosphatase
Kabuli_wild_1858177	1399931744	*Ca_kabuli_Chr01*	1858177	T/C	Gene_Ca_00233	SYNONYMOUS_CODING	Inositol monophosphatase
Kabuli_wild_1858202	1399931745	*Ca_kabuli_Chr01*	1858202	C/T	Gene_Ca_00233	NON_SYNONYMOUS_CODING	Inositol monophosphatase
Kabuli_wild_1858226	1399931746	*Ca_kabuli_Chr01*	1858226	A/G	Gene_Ca_00233	NON_SYNONYMOUS_CODING	Inositol monophosphatase
Kabuli_wild_1858509	1399931747	*Ca_kabuli_Chr01*	1858509	C/T	Gene_Ca_00233	SYNONYMOUS_CODING	Inositol monophosphatase
Kabuli_wild_1858524	1399931748	*Ca_kabuli_Chr01*	1858524	C/G	Gene_Ca_00233	NON_SYNONYMOUS_CODING	Inositol monophosphatase
Kabuli_wild_1858600	1399931749	*Ca_kabuli_Chr01*	1858600	G/A	Gene_Ca_00233	INTRON	Inositol monophosphatase
Kabuli_wild_1858608	1399931750	*Ca_kabuli_Chr01*	1858608	T/A	Gene_Ca_00233	INTRON	Inositol monophosphatase
Kabuli_wild_1858664	1399931751	*Ca_kabuli_Chr01*	1858664	G/A	Gene_Ca_00233	INTRON	Inositol monophosphatase
Kabuli_wild_1859789	1399931752	*Ca_kabuli_Chr01*	1859789	C/T	Gene_Ca_00233	INTRON	Inositol monophosphatase
Kabuli_wild_1859857	1399931753	*Ca_kabuli_Chr01*	1859857	A/G	Gene_Ca_00233	INTRON	Inositol monophosphatase
Kabuli_wild_1859902	1399931754	*Ca_kabuli_Chr01*	1859902	G/A	Gene_Ca_00233	INTRON	Inositol monophosphatase
Kabuli_wild_1859914	1399931755	*Ca_kabuli_Chr01*	1859914	G/A	Gene_Ca_00233	INTRON	Inositol monophosphatase
Kabuli_wild_1861696	1399931756	*Ca_kabuli_Chr01*	1861696	C/T	Gene_Ca_00233	SYNONYMOUS_CODING	Inositol monophosphatase
Kabuli_wild_1861707	1399931757	*Ca_kabuli_Chr01*	1861707	T/G	Gene_Ca_00233	INTRON	Inositol monophosphatase
Kabuli_wild_1861722	1399931758	*Ca_kabuli_Chr01*	1861722	T/A	Gene_Ca_00233	INTRON	Inositol monophosphatase
Kabuli_wild_1861729	1399931759	*Ca_kabuli_Chr01*	1861729	T/C	Gene_Ca_00233	INTRON	Inositol monophosphatase

SNP IDs	NCBI dbSNP accession IDs (NCBI_SS#)	Chromosomes	Physical positions (bp)	SNPs	Gene accession IDs	Sequence components of genome	Putative functions
Kabuli_wild_1861738	1399931760	*Ca_kabuli_Chr01*	1861738	T/G	Gene_Ca_00233	INTRON	Inositol monophosphatase
Kabuli_wild_1861754	1399931761	*Ca_kabuli_Chr01*	1861754	G/A	Gene_Ca_00233	INTRON	Inositol monophosphatase
Kabuli_wild_1861837	1399931762	*Ca_kabuli_Chr01*	1861837	T/G	Gene_Ca_00233	SYNONYMOUS_CODING	Inositol monophosphatase
Kabuli_wild_1870469	1399931763	*Ca_kabuli_Chr01*	1870469	C/A	Gene_Ca_00235	NON_SYNONYMOUS_CODING	Pyridine nucleotide-disulphideoxido reductase,NAD-binding region
Kabuli_wild_1904230	1399931764	*Ca_kabuli_Chr01*	1904230	G/T	Gene_Ca_00238	INTRON	Reversetranscriptase
Kabuli_wild_1904233	1399931765	*Ca_kabuli_Chr01*	1904233	T/C	Gene_Ca_00238	INTRON	Reversetranscriptase
Kabuli_wild_1904365	1399931766	*Ca_kabuli_Chr01*	1904365	G/A	Gene_Ca_00238	INTRON	Reversetranscriptase
Kabuli_wild_1904391	1399931767	*Ca_kabuli_Chr01*	1904391	A/G	Gene_Ca_00238	INTRON	Reversetranscriptase
Kabuli_wild_1904474	1399931768	*Ca_kabuli_Chr01*	1904474	G/T	Gene_Ca_00238	NON_SYNONYMOUS_CODING	Reversetranscriptase
Kabuli_wild_1904477	1399931769	*Ca_kabuli_Chr01*	1904477	A/C	Gene_Ca_00238	SYNONYMOUS_CODING	Reversetranscriptase
Kabuli_wild_1904494	1399931770	*Ca_kabuli_Chr01*	1904494	A/G	Gene_Ca_00238	NON_SYNONYMOUS_CODING	Reversetranscriptase
Kabuli_wild_1935729	1399931771	*Ca_kabuli_Chr01*	1935729	A/G	Gene_Ca_00241	DOWNSTREAM	Diacylglycerolkinase,catalytic domain
Kabuli_wild_1962876	1399931772	*Ca_kabuli_Chr01*	1962876	A/G	Gene_Ca_00244	NON_SYNONYMOUS_CODING	Transcriptional factor B3
Kabuli_wild_1968828	1399931773	*Ca_kabuli_Chr01*	1968828	G/A	NA	INTERGENIC	NA
Kabuli_wild_1971924	1399931774	*Ca_kabuli_Chr01*	1971924	T/G	NA	INTERGENIC	NA
Kabuli_wild_1973618	1399931775	*Ca_kabuli_Chr01*	1973618	A/T	Gene_Ca_00246	INTRON	RNA-processing protein, HAThelix
Kabuli_wild_1986964	1399931776	*Ca_kabuli_Chr01*	1986964	T/C	NA	INTERGENIC	NA
Kabuli_wild_1987101	1399931777	*Ca_kabuli_Chr01*	1987101	C/T	NA	INTERGENIC	NA
Kabuli_wild_1987366	1399931778	*Ca_kabuli_Chr01*	1987366	T/C	NA	INTERGENIC	NA
Kabuli_wild_2002656	1399931779	*Ca_kabuli_Chr01*	2002656	T/C	NA	INTERGENIC	NA
Kabuli_wild_2002844	1399931780	*Ca_kabuli_Chr01*	2002844	G/A	NA	INTERGENIC	NA
Kabuli_wild_2002922	1399931781	*Ca_kabuli_Chr01*	2002922	G/T	NA	INTERGENIC	NA
Kabuli_wild_2040887	1399931782	*Ca_kabuli_Chr01*	2040887	A/G	Gene_Ca_00258	UPSTREAM	Disease resistance protein
Kabuli_wild_2040943	1399931783	*Ca_kabuli_Chr01*	2040943	C/T	Gene_Ca_00257	DOWNSTREAM	NA
Kabuli_wild_2040946	1399931784	*Ca_kabuli_Chr01*	2040946	G/T	Gene_Ca_00257	DOWNSTREAM	NA
Kabuli_wild_2041027	1399931785	*Ca_kabuli_Chr01*	2041027	C/G	Gene_Ca_00257	DOWNSTREAM	NA
Kabuli_wild_2041126	1399931786	*Ca_kabuli_Chr01*	2041126	C/A	Gene_Ca_00257	DOWNSTREAM	NA
Kabuli_wild_2041132	1399931787	*Ca_kabuli_Chr01*	2041132	C/T	Gene_Ca_00257	DOWNSTREAM	NA
Kabuli_wild_2041154	1399931788	*Ca_kabuli_Chr01*	2041154	G/C	NA	INTERGENIC	NA
Kabuli_wild_2041174	1399931789	*Ca_kabuli_Chr01*	2041174	C/T	NA	INTERGENIC	NA
Kabuli_wild_2041176	1399931790	*Ca_kabuli_Chr01*	2041176	A/G	NA	INTERGENIC	NA
Kabuli_wild_2041178	1399931791	*Ca_kabuli_Chr01*	2041178	A/C	NA	INTERGENIC	NA
Kabuli_wild_2041180	1399931792	*Ca_kabuli_Chr01*	2041180	T/C	NA	INTERGENIC	NA
Kabuli_wild_2041190	1399931793	*Ca_kabuli_Chr01*	2041190	A/C	NA	INTERGENIC	NA
Kabuli_wild_2042071	1399931794	*Ca_kabuli_Chr01*	2042071	C/G	NA	INTERGENIC	NA
Kabuli_wild_2042149	1399931795	*Ca_kabuli_Chr01*	2042149	G/T	NA	INTERGENIC	NA
Kabuli_wild_2042152	1399931796	*Ca_kabuli_Chr01*	2042152	G/C	NA	INTERGENIC	NA
Kabuli_wild_2042185	1399931797	*Ca_kabuli_Chr01*	2042185	T/C	NA	INTERGENIC	NA
Kabuli_wild_2043957	1399931798	*Ca_kabuli_Chr01*	2043957	C/G	NA	INTERGENIC	NA
Kabuli_wild_2044263	1399931799	*Ca_kabuli_Chr01*	2044263	A/C	Gene_Ca_00259	DOWNSTREAM	SCAMP
Kabuli_wild_2044289	1399931800	*Ca_kabuli_Chr01*	2044289	A/G	Gene_Ca_00259	DOWNSTREAM	SCAMP
Kabuli_wild_2044359	1399931801	*Ca_kabuli_Chr01*	2044359	C/T	Gene_Ca_00259	DOWNSTREAM	SCAMP
Kabuli_wild_2044380	1399931802	*Ca_kabuli_Chr01*	2044380	G/C	Gene_Ca_00259	DOWNSTREAM	SCAMP
Kabuli_wild_2044404	1399931803	*Ca_kabuli_Chr01*	2044404	T/C	Gene_Ca_00259	DOWNSTREAM	SCAMP

SNP IDs	NCBI dbSNP accession IDs (NCBI_SS#)	Chromosomes	Physical positions (bp)	SNPs	Gene accession IDs	Sequence components of genome	Putative functions
Kabuli_wild_2044408	1399931804	*Ca_kabuli_Chr01*	2044408	A/C	Gene_Ca_00259	DOWNSTREAM	SCAMP
Kabuli_wild_2044414	1399931805	*Ca_kabuli_Chr01*	2044414	G/A	Gene_Ca_00259	DOWNSTREAM	SCAMP
Kabuli_wild_2044474	1399931806	*Ca_kabuli_Chr01*	2044474	A/G	Gene_Ca_00259	DOWNSTREAM	SCAMP
Kabuli_wild_2050187	1399931807	*Ca_kabuli_Chr01*	2050187	C/G	Gene_Ca_00260	UPSTREAM	Disease resistance protein
Kabuli_wild_2050189	1399931808	*Ca_kabuli_Chr01*	2050189	T/C	Gene_Ca_00260	UPSTREAM	Disease resistance protein
Kabuli_wild_2050232	1399931809	*Ca_kabuli_Chr01*	2050232	A/T	Gene_Ca_00259	UPSTREAM	SCAMP
Kabuli_wild_2050278	1399931810	*Ca_kabuli_Chr01*	2050278	C/G	Gene_Ca_00259	UPSTREAM	SCAMP
Kabuli_wild_2050300	1399931811	*Ca_kabuli_Chr01*	2050300	C/G	NA	INTERGENIC	NA
Kabuli_wild_2050309	1399931812	*Ca_kabuli_Chr01*	2050309	T/C	NA	INTERGENIC	NA
Kabuli_wild_2050313	1399931813	*Ca_kabuli_Chr01*	2050313	C/T	NA	INTERGENIC	NA
Kabuli_wild_2050320	1399931814	*Ca_kabuli_Chr01*	2050320	T/A	NA	INTERGENIC	NA
Kabuli_wild_2050321	1399931815	*Ca_kabuli_Chr01*	2050321	A/C	NA	INTERGENIC	NA
Kabuli_wild_2050328	1399931816	*Ca_kabuli_Chr01*	2050328	C/T	NA	INTERGENIC	NA
Kabuli_wild_2050415	1399931817	*Ca_kabuli_Chr01*	2050415	C/A	NA	INTERGENIC	NA
Kabuli_wild_2050421	1399931818	*Ca_kabuli_Chr01*	2050421	C/T	NA	INTERGENIC	NA
Kabuli_wild_2050463	1399931819	*Ca_kabuli_Chr01*	2050463	C/T	NA	INTERGENIC	NA
Kabuli_wild_2050465	1399931820	*Ca_kabuli_Chr01*	2050465	A/G	NA	INTERGENIC	NA
Kabuli_wild_2050467	1399931821	*Ca_kabuli_Chr01*	2050467	A/C	NA	INTERGENIC	NA
Kabuli_wild_2050469	1399931822	*Ca_kabuli_Chr01*	2050469	T/C	NA	INTERGENIC	NA
Kabuli_wild_2050475	1399931823	*Ca_kabuli_Chr01*	2050475	T/C	NA	INTERGENIC	NA
Kabuli_wild_2050479	1399931824	*Ca_kabuli_Chr01*	2050479	A/C	NA	INTERGENIC	NA
Kabuli_wild_2050484	1399931825	*Ca_kabuli_Chr01*	2050484	C/G	NA	INTERGENIC	NA
Kabuli_wild_2051354	1399931826	*Ca_kabuli_Chr01*	2051354	A/C	NA	INTERGENIC	NA
Kabuli_wild_2051360	1399931827	*Ca_kabuli_Chr01*	2051360	A/G	NA	INTERGENIC	NA
Kabuli_wild_2051379	1399931828	*Ca_kabuli_Chr01*	2051379	C/T	NA	INTERGENIC	NA
Kabuli_wild_2053662	1399931829	*Ca_kabuli_Chr01*	2053662	G/T	Gene_Ca_00260	NON_SYNONYMOUS_CODING	Disease resistance protein
Kabuli_wild_2053677	1399931830	*Ca_kabuli_Chr01*	2053677	A/G	Gene_Ca_00260	NON_SYNONYMOUS_CODING	Disease resistance protein
Kabuli_wild_2053732	1399931831	*Ca_kabuli_Chr01*	2053732	C/T	Gene_Ca_00260	NON_SYNONYMOUS_CODING	Disease resistance protein
Kabuli_wild_2053744	1399931832	*Ca_kabuli_Chr01*	2053744	C/A	Gene_Ca_00260	NON_SYNONYMOUS_CODING	Disease resistance protein
Kabuli_wild_2053749	1399931833	*Ca_kabuli_Chr01*	2053749	T/A	Gene_Ca_00260	NON_SYNONYMOUS_CODING	Disease resistance protein
Kabuli_wild_2053755	1399931834	*Ca_kabuli_Chr01*	2053755	A/T	Gene_Ca_00260	NON_SYNONYMOUS_CODING	Disease resistance protein
Kabuli_wild_2053756	1399931835	*Ca_kabuli_Chr01*	2053756	G/T	Gene_Ca_00260	NON_SYNONYMOUS_CODING	Disease resistance protein
Kabuli_wild_2053758	1399931836	*Ca_kabuli_Chr01*	2053758	A/G	Gene_Ca_00260	NON_SYNONYMOUS_CODING	Disease resistance protein
Kabuli_wild_2053784	1399931837	*Ca_kabuli_Chr01*	2053784	C/A	Gene_Ca_00260	NON_SYNONYMOUS_CODING	Disease resistance protein
Kabuli_wild_2053788	1399931838	*Ca_kabuli_Chr01*	2053788	A/G	Gene_Ca_00260	NON_SYNONYMOUS_CODING	Disease resistance protein
Kabuli_wild_2053790	1399931839	*Ca_kabuli_Chr01*	2053790	G/A	Gene_Ca_00260	SYNONYMOUS_CODING	Disease resistance protein
Kabuli_wild_2053808	1399931840	*Ca_kabuli_Chr01*	2053808	G/T	Gene_Ca_00260	SYNONYMOUS_CODING	Disease resistance protein
Kabuli_wild_2053818	1399931841	*Ca_kabuli_Chr01*	2053818	G/C	Gene_Ca_00260	NON_SYNONYMOUS_CODING	Disease resistance protein
Kabuli_wild_2053819	1399931842	*Ca_kabuli_Chr01*	2053819	C/A	Gene_Ca_00260	SYNONYMOUS_CODING	Disease resistance protein
Kabuli_wild_2053830	1399931843	*Ca_kabuli_Chr01*	2053830	A/G	Gene_Ca_00260	SYNONYMOUS_CODING	Disease resistance protein
Kabuli_wild_2053833	1399931844	*Ca_kabuli_Chr01*	2053833	T/G	Gene_Ca_00260	SYNONYMOUS_CODING	Disease resistance protein
Kabuli_wild_2053856	1399931845	*Ca_kabuli_Chr01*	2053856	T/G	Gene_Ca_00260	SYNONYMOUS_CODING	Disease resistance protein
Kabuli_wild_2071424	1399931846	*Ca_kabuli_Chr01*	2071424	A/T	Gene_Ca_00263	UPSTREAM	Disease resistance protein
Kabuli_wild_2071448	1399931847	*Ca_kabuli_Chr01*	2071448	A/T	Gene_Ca_00263	UPSTREAM	Disease resistance protein

SNP IDs	NCBI dbSNP accession IDs (NCBI_SS#)	Chromosomes	Physical positions (bp)	SNPs	Gene accession IDs	Sequence components of genome	Putative functions
Kabuli_wild_2071456	1399931848	*Ca_kabuli_Chr01*	2071456	T/A	Gene_Ca_00263	UPSTREAM	Disease resistance protein
Kabuli_wild_2071502	1399931849	*Ca_kabuli_Chr01*	2071502	C/G	Gene_Ca_00263	UPSTREAM	Disease resistance protein
Kabuli_wild_2071524	1399931850	*Ca_kabuli_Chr01*	2071524	C/G	Gene_Ca_00263	UPSTREAM	Disease resistance protein
Kabuli_wild_2071533	1399931851	*Ca_kabuli_Chr01*	2071533	T/C	Gene_Ca_00263	UPSTREAM	Disease resistance protein
Kabuli_wild_2071537	1399931852	*Ca_kabuli_Chr01*	2071537	C/T	Gene_Ca_00263	UPSTREAM	Disease resistance protein
Kabuli_wild_2071544	1399931853	*Ca_kabuli_Chr01*	2071544	T/A	Gene_Ca_00263	UPSTREAM	Disease resistance protein
Kabuli_wild_2071545	1399931854	*Ca_kabuli_Chr01*	2071545	A/C	Gene_Ca_00263	UPSTREAM	Disease resistance protein
Kabuli_wild_2071552	1399931855	*Ca_kabuli_Chr01*	2071552	C/T	Gene_Ca_00263	UPSTREAM	Disease resistance protein
Kabuli_wild_2071639	1399931856	*Ca_kabuli_Chr01*	2071639	C/A	Gene_Ca_00263	UPSTREAM	Disease resistance protein
Kabuli_wild_2071645	1399931857	*Ca_kabuli_Chr01*	2071645	C/T	Gene_Ca_00263	UPSTREAM	Disease resistance protein
Kabuli_wild_2071687	1399931858	*Ca_kabuli_Chr01*	2071687	C/T	Gene_Ca_00263	UPSTREAM	Disease resistance protein
Kabuli_wild_2071689	1399931859	*Ca_kabuli_Chr01*	2071689	A/G	Gene_Ca_00263	UPSTREAM	Disease resistance protein
Kabuli_wild_2071691	1399931860	*Ca_kabuli_Chr01*	2071691	A/C	Gene_Ca_00263	UPSTREAM	Disease resistance protein
Kabuli_wild_2071693	1399931861	*Ca_kabuli_Chr01*	2071693	T/C	Gene_Ca_00263	UPSTREAM	Disease resistance protein
Kabuli_wild_2071699	1399931862	*Ca_kabuli_Chr01*	2071699	T/C	Gene_Ca_00263	UPSTREAM	Disease resistance protein
Kabuli_wild_2071703	1399931863	*Ca_kabuli_Chr01*	2071703	A/C	Gene_Ca_00263	UPSTREAM	Disease resistance protein
Kabuli_wild_2071708	1399931864	*Ca_kabuli_Chr01*	2071708	C/G	Gene_Ca_00263	UPSTREAM	Disease resistance protein
Kabuli_wild_2072613	1399931865	*Ca_kabuli_Chr01*	2072613	G/A	Gene_Ca_00264	UPSTREAM	NA
Kabuli_wild_2072627	1399931866	*Ca_kabuli_Chr01*	2072627	A/T	Gene_Ca_00264	UPSTREAM	NA
Kabuli_wild_2075009	1399931867	*Ca_kabuli_Chr01*	2075009	C/A	NA	INTERGENIC	NA
Kabuli_wild_2075013	1399931868	*Ca_kabuli_Chr01*	2075013	A/G	NA	INTERGENIC	NA
Kabuli_wild_2075015	1399931869	*Ca_kabuli_Chr01*	2075015	G/A	NA	INTERGENIC	NA
Kabuli_wild_2075043	1399931870	*Ca_kabuli_Chr01*	2075043	C/A	NA	INTERGENIC	NA
Kabuli_wild_2075044	1399931871	*Ca_kabuli_Chr01*	2075044	A/G	NA	INTERGENIC	NA
Kabuli_wild_2075045	1399931872	*Ca_kabuli_Chr01*	2075045	C/A	NA	INTERGENIC	NA
Kabuli_wild_2075057	1399931873	*Ca_kabuli_Chr01*	2075057	G/T	NA	INTERGENIC	NA
Kabuli_wild_2075087	1399931874	*Ca_kabuli_Chr01*	2075087	A/T	NA	INTERGENIC	NA
Kabuli_wild_2082760	1399931875	*Ca_kabuli_Chr01*	2082760	C/G	Gene_Ca_00265	INTRON	Protein of unknown function DUF707
Kabuli_wild_2082801	1399931876	*Ca_kabuli_Chr01*	2082801	G/A	Gene_Ca_00265	INTRON	Protein of unknown function DUF707
Kabuli_wild_2083003	1399931877	*Ca_kabuli_Chr01*	2083003	G/C	Gene_Ca_00265	INTRON	Protein of unknown function DUF707
Kabuli_wild_2083059	1399931878	*Ca_kabuli_Chr01*	2083059	A/T	Gene_Ca_00265	INTRON	Protein of unknown function DUF707
Kabuli_wild_2083073	1399931879	*Ca_kabuli_Chr01*	2083073	C/T	Gene_Ca_00265	INTRON	Protein of unknown function DUF707
Kabuli_wild_2126267	1399931880	*Ca_kabuli_Chr01*	2126267	G/A	Gene_Ca_00268	INTRON	Protein kinase,catalytic domain
Kabuli_wild_2148583	1399931881	*Ca_kabuli_Chr01*	2148583	T/G	Gene_Ca_00270	DOWNSTREAM	NA
Kabuli_wild_2170090	1399931882	*Ca_kabuli_Chr01*	2170090	G/C	Gene_Ca_00273	DOWNSTREAM	LisHdimerisationmotif
Kabuli_wild_2170935	1399931883	*Ca_kabuli_Chr01*	2170935	A/C	Gene_Ca_00272	DOWNSTREAM	NA
Kabuli_wild_2172076	1399931884	*Ca_kabuli_Chr01*	2172076	T/C	Gene_Ca_00272	DOWNSTREAM	NA
Kabuli_wild_2172113	1399931885	*Ca_kabuli_Chr01*	2172113	G/A	Gene_Ca_00272	DOWNSTREAM	NA
Kabuli_wild_2172143	1399931886	*Ca_kabuli_Chr01*	2172143	T/G	Gene_Ca_00272	DOWNSTREAM	NA
Kabuli_wild_2183544	1399931887	*Ca_kabuli_Chr01*	2183544	A/G	Gene_Ca_00274	INTRON	SNF2-related
Kabuli_wild_2212073	1399931888	*Ca_kabuli_Chr01*	2212073	A/G	Gene_Ca_00276	NON_SYNONYMOUS_CODING	NA
Kabuli_wild_2232779	1399931889	*Ca_kabuli_Chr01*	2232779	A/G	Gene_Ca_00279	INTRON	Amidohydrolase1
Kabuli_wild_2252270	1399931890	*Ca_kabuli_Chr01*	2252270	C/T	Gene_Ca_00281	INTRON	Zinc finger, RING-type
Kabuli_wild_2252343	1399931891	*Ca_kabuli_Chr01*	2252343	G/T	Gene_Ca_00281	INTRON	NA

SNP IDs	NCBI dbSNP accession IDs (NCBI_SS#)	Chromosomes	Physical positions (bp)	SNPs	Gene accession IDs	Sequence components of genome	Putative functions
Kabuli_wild_2252473	1399931892	*Ca_kabuli_Chr01*	2252473	G/A	Gene_Ca_00281	INTRON	NA
Kabuli_wild_2252513	1399931893	*Ca_kabuli_Chr01*	2252513	C/T	Gene_Ca_00281	INTRON	NA
Kabuli_wild_2269303	1399931894	*Ca_kabuli_Chr01*	2269303	T/C	NA	INTERGENIC	NA
Kabuli_wild_2269319	1399931895	*Ca_kabuli_Chr01*	2269319	T/G	NA	INTERGENIC	NA
Kabuli_wild_2269489	1399931896	*Ca_kabuli_Chr01*	2269489	A/C	NA	INTERGENIC	NA
Kabuli_wild_2277459	1399931897	*Ca_kabuli_Chr01*	2277459	G/A	Gene_Ca_00285	INTRON	RNA recognition motif domain
Kabuli_wild_2285879	1399931898	*Ca_kabuli_Chr01*	2285879	G/A	Gene_Ca_00287	DOWNSTREAM	NA
Kabuli_wild_2287408	1399931899	*Ca_kabuli_Chr01*	2287408	A/T	Gene_Ca_00287	NON_SYNONYMOUS_CODING	NA
Kabuli_wild_2290092	1399931900	*Ca_kabuli_Chr01*	2290092	A/G	Gene_Ca_00287	INTRON	NA
Kabuli_wild_2290150	1399931901	*Ca_kabuli_Chr01*	2290150	C/A	Gene_Ca_00287	INTRON	NA
Kabuli_wild_2305216	1399931902	*Ca_kabuli_Chr01*	2305216	T/G	NA	INTERGENIC	NA
Kabuli_wild_2305246	1399931903	*Ca_kabuli_Chr01*	2305246	T/A	NA	INTERGENIC	NA
Kabuli_wild_2305351	1399931904	*Ca_kabuli_Chr01*	2305351	T/A	NA	INTERGENIC	NA
Kabuli_wild_2305786	1399931905	*Ca_kabuli_Chr01*	2305786	A/T	NA	INTERGENIC	NA
Kabuli_wild_2305849	1399931906	*Ca_kabuli_Chr01*	2305849	G/A	NA	INTERGENIC	NA
Kabuli_wild_2305903	1399931907	*Ca_kabuli_Chr01*	2305903	G/A	NA	INTERGENIC	NA
Kabuli_wild_2306287	1399931908	*Ca_kabuli_Chr01*	2306287	G/A	NA	INTERGENIC	NA
Kabuli_wild_2306323	1399931909	*Ca_kabuli_Chr01*	2306323	C/T	NA	INTERGENIC	NA
Kabuli_wild_2306340	1399931910	*Ca_kabuli_Chr01*	2306340	T/C	NA	INTERGENIC	NA
Kabuli_wild_2366409	1399931911	*Ca_kabuli_Chr01*	2366409	T/A	NA	INTERGENIC	NA
Kabuli_wild_2366413	1399931912	*Ca_kabuli_Chr01*	2366413	T/C	NA	INTERGENIC	NA
Kabuli_wild_2373280	1399931913	*Ca_kabuli_Chr01*	2373280	T/C	Gene_Ca_00301	INTRON	Ribosoma lRNA methyltransferase RrmJ/FtsJ
Kabuli_wild_2373480	1399931914	*Ca_kabuli_Chr01*	2373480	G/T	Gene_Ca_00301	INTRON	Ribosoma lRNA methyltransferase RrmJ/FtsJ
Kabuli_wild_2373862	1399931915	*Ca_kabuli_Chr01*	2373862	G/A	Gene_Ca_00301	INTRON	Ribosoma lRNA methyltransferase RrmJ/FtsJ
Kabuli_wild_2373910	1399931916	*Ca_kabuli_Chr01*	2373910	C/T	Gene_Ca_00301	INTRON	Ribosoma lRNA methyltransferase RrmJ/FtsJ
Kabuli_wild_2373954	1399931917	*Ca_kabuli_Chr01*	2373954	G/T	Gene_Ca_00301	INTRON	Ribosoma lRNA methyltransferase RrmJ/FtsJ
Kabuli_wild_2377689	1399931918	*Ca_kabuli_Chr01*	2377689	T/A	NA	INTERGENIC	NA
Kabuli_wild_2400799	1399931919	*Ca_kabuli_Chr01*	2400799	T/G	Gene_Ca_00305	UPSTREAM	Protein of unknown function DUF2921
Kabuli_wild_2428531	1399931920	*Ca_kabuli_Chr01*	2428531	A/G	Gene_Ca_00308	UPSTREAM	Protein of unknown function DUF616
Kabuli_wild_2439915	1399931921	*Ca_kabuli_Chr01*	2439915	G/A	NA	INTERGENIC	NA
Kabuli_wild_2439917	1399931922	*Ca_kabuli_Chr01*	2439917	A/G	NA	INTERGENIC	NA
Kabuli_wild_2457925	1399931923	*Ca_kabuli_Chr01*	2457925	C/T	Gene_Ca_00311	INTRON	Protein kinase,catalytic domain
Kabuli_wild_2473309	1399931924	*Ca_kabuli_Chr01*	2473309	T/C	NA	INTERGENIC	NA
Kabuli_wild_2513383	1399931925	*Ca_kabuli_Chr01*	2513383	T/C	Gene_Ca_00314	SYNONYMOUS_CODING	Telomerase activating protein Est1
Kabuli_wild_2513516	1399931926	*Ca_kabuli_Chr01*	2513516	T/A	Gene_Ca_00314	NON_SYNONYMOUS_CODING	Telomerase activating protein Est1
Kabuli_wild_2513535	1399931927	*Ca_kabuli_Chr01*	2513535	T/C	Gene_Ca_00314	NON_SYNONYMOUS_CODING	Telomerase activating protein Est1
Kabuli_wild_2513737	1399931928	*Ca_kabuli_Chr01*	2513737	T/C	Gene_Ca_00314	SYNONYMOUS_CODING	Telomerase activating protein Est1
Kabuli_wild_2513792	1399931929	*Ca_kabuli_Chr01*	2513792	A/G	Gene_Ca_00314	NON_SYNONYMOUS_CODING	Telomerase activating protein Est1
Kabuli_wild_2513854	1399931930	*Ca_kabuli_Chr01*	2513854	G/A	Gene_Ca_00314	SYNONYMOUS_CODING	Telomerase activating protein Est1
Kabuli_wild_2513860	1399931931	*Ca_kabuli_Chr01*	2513860	G/A	Gene_Ca_00314	SYNONYMOUS_CODING	Telomerase activating protein Est1
Kabuli_wild_2542652	1399931932	*Ca_kabuli_Chr01*	2542652	G/A	NA	INTERGENIC	NA
Kabuli_wild_2607973	1399931933	*Ca_kabuli_Chr01*	2607973	C/G	NA	INTERGENIC	NA
Kabuli_wild_2607974	1399931934	*Ca_kabuli_Chr01*	2607974	G/T	NA	INTERGENIC	NA
Kabuli_wild_2607994	1399931935	*Ca_kabuli_Chr01*	2607994	A/G	NA	INTERGENIC	NA

SNP IDs	NCBI dbSNP accession IDs (NCBI_SS#)	Chromosomes	Physical positions (bp)	SNPs	Gene accession IDs	Sequence components of genome	Putative functions
Kabuli_wild_2649864	1399931936	*Ca_kabuli_Chr01*	2649864	C/A	Gene_Ca_00326	SYNONYMOUS_CODING	Pathogenesis-related transcriptional factor/ERF, DNA-binding
Kabuli_wild_2649974	1399931937	*Ca_kabuli_Chr01*	2649974	A/C	Gene_Ca_00326	SYNONYMOUS_CODING	Pathogenesis-related transcriptional factor/ERF, DNA-binding
Kabuli_wild_2649976	1399931938	*Ca_kabuli_Chr01*	2649976	A/T	Gene_Ca_00326	NON_SYNONYMOUS_CODING	Pathogenesis-related transcriptional factor/ERF, DNA-binding
Kabuli_wild_2671403	1399931939	*Ca_kabuli_Chr01*	2671403	G/T	Gene_Ca_00327	SYNONYMOUS_CODING	Pathogenesis-related transcriptional factor/ERF, DNA-binding
Kabuli_wild_2702124	1399931940	*Ca_kabuli_Chr01*	2702124	T/C	NA	INTERGENIC	NA
Kabuli_wild_2702277	1399931941	*Ca_kabuli_Chr01*	2702277	T/C	NA	INTERGENIC	NA
Kabuli_wild_2745486	1399931942	*Ca_kabuli_Chr01*	2745486	T/C	Gene_Ca_00338	UPSTREAM	Pentatrico peptide repeat
Kabuli_wild_2745499	1399931943	*Ca_kabuli_Chr01*	2745499	C/A	Gene_Ca_00338	UPSTREAM	Pentatrico peptide repeat
Kabuli_wild_2745514	1399931944	*Ca_kabuli_Chr01*	2745514	A/C	Gene_Ca_00338	UPSTREAM	Pentatrico peptide repeat
Kabuli_wild_2745519	1399931945	*Ca_kabuli_Chr01*	2745519	G/C	Gene_Ca_00338	UPSTREAM	Pentatrico peptide repeat
Kabuli_wild_2754998	1399931946	*Ca_kabuli_Chr01*	2754998	C/T	Gene_Ca_00340	SYNONYMOUS_CODING	Disease resistance protein
Kabuli_wild_2755002	1399931947	*Ca_kabuli_Chr01*	2755002	A/C	Gene_Ca_00340	NON_SYNONYMOUS_CODING	Disease resistance protein
Kabuli_wild_2755012	1399931948	*Ca_kabuli_Chr01*	2755012	C/T	Gene_Ca_00340	SYNONYMOUS_CODING	Disease resistance protein
Kabuli_wild_2755015	1399931949	*Ca_kabuli_Chr01*	2755015	T/C	Gene_Ca_00340	SYNONYMOUS_CODING	Disease resistance protein
Kabuli_wild_2755021	1399931950	*Ca_kabuli_Chr01*	2755021	T/G	Gene_Ca_00340	NON_SYNONYMOUS_CODING	Disease resistance protein
Kabuli_wild_2755028	1399931951	*Ca_kabuli_Chr01*	2755028	G/T	Gene_Ca_00340	NON_SYNONYMOUS_CODING	Disease resistance protein
Kabuli_wild_2755056	1399931952	*Ca_kabuli_Chr01*	2755056	C/T	Gene_Ca_00340	SYNONYMOUS_CODING	Disease resistance protein
Kabuli_wild_2755443	1399931953	*Ca_kabuli_Chr01*	2755443	T/A	Gene_Ca_00340	NON_SYNONYMOUS_CODING	Disease resistance protein
Kabuli_wild_2755455	1399931954	*Ca_kabuli_Chr01*	2755455	G/A	Gene_Ca_00340	SYNONYMOUS_CODING	Disease resistance protein
Kabuli_wild_2755457	1399931955	*Ca_kabuli_Chr01*	2755457	T/C	Gene_Ca_00340	SYNONYMOUS_CODING	Disease resistance protein
Kabuli_wild_2755459	1399931956	*Ca_kabuli_Chr01*	2755459	T/G	Gene_Ca_00340	SYNONYMOUS_CODING	Disease resistance protein
Kabuli_wild_2755465	1399931957	*Ca_kabuli_Chr01*	2755465	T/A	Gene_Ca_00340	SYNONYMOUS_CODING	Disease resistance protein
Kabuli_wild_2755466	1399931958	*Ca_kabuli_Chr01*	2755466	C/G	Gene_Ca_00340	SYNONYMOUS_CODING	Disease resistance protein
Kabuli_wild_2755470	1399931959	*Ca_kabuli_Chr01*	2755470	A/T	Gene_Ca_00340	SYNONYMOUS_CODING	Disease resistance protein
Kabuli_wild_2755471	1399931960	*Ca_kabuli_Chr01*	2755471	C/A	Gene_Ca_00340	SYNONYMOUS_CODING	Disease resistance protein
Kabuli_wild_2755489	1399931961	*Ca_kabuli_Chr01*	2755489	G/A	Gene_Ca_00340	NON_SYNONYMOUS_CODING	Disease resistance protein
Kabuli_wild_2755494	1399931962	*Ca_kabuli_Chr01*	2755494	T/C	Gene_Ca_00340	SYNONYMOUS_CODING	Disease resistance protein
Kabuli_wild_2755496	1399931963	*Ca_kabuli_Chr01*	2755496	G/A	Gene_Ca_00340	SYNONYMOUS_CODING	Disease resistance protein
Kabuli_wild_2755596	1399931964	*Ca_kabuli_Chr01*	2755596	A/C	Gene_Ca_00340	SYNONYMOUS_CODING	Disease resistance protein
Kabuli_wild_2755604	1399931965	*Ca_kabuli_Chr01*	2755604	C/A	Gene_Ca_00340	SYNONYMOUS_CODING	Disease resistance protein
Kabuli_wild_2755613	1399931966	*Ca_kabuli_Chr01*	2755613	T/G	Gene_Ca_00340	SYNONYMOUS_CODING	Disease resistance protein
Kabuli_wild_2757678	1399931967	*Ca_kabuli_Chr01*	2757678	A/G	Gene_Ca_00340	SYNONYMOUS_CODING	Disease resistance protein
Kabuli_wild_2757693	1399931968	*Ca_kabuli_Chr01*	2757693	C/T	Gene_Ca_00340	SYNONYMOUS_CODING	Disease resistance protein
Kabuli_wild_2757696	1399931969	*Ca_kabuli_Chr01*	2757696	A/G	Gene_Ca_00340	SYNONYMOUS_CODING	Disease resistance protein
Kabuli_wild_2757711	1399931970	*Ca_kabuli_Chr01*	2757711	T/G	Gene_Ca_00340	SYNONYMOUS_CODING	Disease resistance protein
Kabuli_wild_2758731	1399931971	*Ca_kabuli_Chr01*	2758731	G/A	Gene_Ca_00340	SYNONYMOUS_CODING	Disease resistance protein
Kabuli_wild_2758738	1399931972	*Ca_kabuli_Chr01*	2758738	T/G	Gene_Ca_00340	NON_SYNONYMOUS_CODING	Disease resistance protein
Kabuli_wild_2758739	1399931973	*Ca_kabuli_Chr01*	2758739	A/T	Gene_Ca_00340	NON_SYNONYMOUS_CODING	Disease resistance protein
Kabuli_wild_2758746	1399931974	*Ca_kabuli_Chr01*	2758746	G/A	Gene_Ca_00340	SYNONYMOUS_CODING	Disease resistance protein
Kabuli_wild_2758750	1399931975	*Ca_kabuli_Chr01*	2758750	A/G	Gene_Ca_00340	NON_SYNONYMOUS_CODING	Disease resistance protein
Kabuli_wild_2758759	1399931976	*Ca_kabuli_Chr01*	2758759	G/C	Gene_Ca_00340	NON_SYNONYMOUS_CODING	Disease resistance protein
Kabuli_wild_2758781	1399931977	*Ca_kabuli_Chr01*	2758781	G/C	Gene_Ca_00340	NON_SYNONYMOUS_CODING	Disease resistance protein
Kabuli_wild_2758827	1399931978	*Ca_kabuli_Chr01*	2758827	A/T	Gene_Ca_00340	NON_SYNONYMOUS_CODING	Disease resistance protein
Kabuli_wild_2758835	1399931979	*Ca_kabuli_Chr01*	2758835	T/A	Gene_Ca_00340	UPSTREAM	Disease resistance protein

SNP IDs	NCBI dbSNP accession IDs (NCBI_SS#)	Chromosomes	Physical positions (bp)	SNPs	Gene accession IDs	Sequence components of genome	Putative functions
Kabuli_wild_2758859	1399931980	*Ca_kabuli_Chr01*	2758859	A/T	Gene_Ca_00340	UPSTREAM	Disease resistance protein
Kabuli_wild_2786970	1399931981	*Ca_kabuli_Chr01*	2786970	T/A	NA	INTERGENIC	NA
Kabuli_wild_2786974	1399931982	*Ca_kabuli_Chr01*	2786974	T/C	NA	INTERGENIC	NA
Kabuli_wild_2786976	1399931983	*Ca_kabuli_Chr01*	2786976	A/C	NA	INTERGENIC	NA
Kabuli_wild_2786989	1399931984	*Ca_kabuli_Chr01*	2786989	T/C	NA	INTERGENIC	NA
Kabuli_wild_2786994	1399931985	*Ca_kabuli_Chr01*	2786994	A/C	NA	INTERGENIC	NA
Kabuli_wild_2807604	1399931986	*Ca_kabuli_Chr01*	2807604	T/C	Gene_Ca_00345	SYNONYMOUS_CODING	NA
Kabuli_wild_2807605	1399931987	*Ca_kabuli_Chr01*	2807605	G/C	Gene_Ca_00345	NON_SYNONYMOUS_CODING	NA
Kabuli_wild_2807616	1399931988	*Ca_kabuli_Chr01*	2807616	G/A	Gene_Ca_00345	NON_SYNONYMOUS_CODING	NA
Kabuli_wild_2807670	1399931989	*Ca_kabuli_Chr01*	2807670	A/C	Gene_Ca_00345	SYNONYMOUS_CODING	NA
Kabuli_wild_2808019	1399931990	*Ca_kabuli_Chr01*	2808019	T/C	Gene_Ca_00345	SYNONYMOUS_CODING	NA
Kabuli_wild_2808035	1399931991	*Ca_kabuli_Chr01*	2808035	C/A	Gene_Ca_00345	SYNONYMOUS_CODING	NA
Kabuli_wild_2808039	1399931992	*Ca_kabuli_Chr01*	2808039	A/G	Gene_Ca_00345	SYNONYMOUS_CODING	NA
Kabuli_wild_2808079	1399931993	*Ca_kabuli_Chr01*	2808079	T/G	Gene_Ca_00345	SYNONYMOUS_CODING	NA
Kabuli_wild_2832792	1399931994	*Ca_kabuli_Chr01*	2832792	G/A	NA	INTERGENIC	NA
Kabuli_wild_2832939	1399931995	*Ca_kabuli_Chr01*	2832939	G/T	Gene_Ca_00347	NON_SYNONYMOUS_CODING	Transcriptional factor B3
Kabuli_wild_2832969	1399931996	*Ca_kabuli_Chr01*	2832969	G/A	Gene_Ca_00347	SYNONYMOUS_CODING	Transcriptional factor B3
Kabuli_wild_2833448	1399931997	*Ca_kabuli_Chr01*	2833448	C/A	Gene_Ca_00347	NON_SYNONYMOUS_CODING	Transcriptional factor B3
Kabuli_wild_2833454	1399931998	*Ca_kabuli_Chr01*	2833454	T/G	Gene_Ca_00347	SYNONYMOUS_CODING	Transcriptional factor B3
Kabuli_wild_2833560	1399931999	*Ca_kabuli_Chr01*	2833560	T/C	Gene_Ca_00347	SYNONYMOUS_CODING	Transcriptional factor B3
Kabuli_wild_2834380	1399932000	*Ca_kabuli_Chr01*	2834380	G/A	Gene_Ca_00347	INTRON	Transcriptional factor B3
Kabuli_wild_2834456	1399932001	*Ca_kabuli_Chr01*	2834456	T/A	Gene_Ca_00347	INTRON	Transcriptional factor B3
Kabuli_wild_2855857	1399932002	*Ca_kabuli_Chr01*	2855857	T/G	NA	INTERGENIC	NA
Kabuli_wild_2855866	1399932003	*Ca_kabuli_Chr01*	2855866	T/G	NA	INTERGENIC	NA
Kabuli_wild_2855927	1399932004	*Ca_kabuli_Chr01*	2855927	T/A	NA	INTERGENIC	NA
Kabuli_wild_2856003	1399932005	*Ca_kabuli_Chr01*	2856003	T/C	NA	INTERGENIC	NA
Kabuli_wild_2856045	1399932006	*Ca_kabuli_Chr01*	2856045	C/T	NA	INTERGENIC	NA
Kabuli_wild_2856079	1399932007	*Ca_kabuli_Chr01*	2856079	T/G	NA	INTERGENIC	NA
Kabuli_wild_2856094	1399932008	*Ca_kabuli_Chr01*	2856094	T/G	NA	INTERGENIC	NA
Kabuli_wild_2856120	1399932009	*Ca_kabuli_Chr01*	2856120	T/G	NA	INTERGENIC	NA
Kabuli_wild_2856126	1399932010	*Ca_kabuli_Chr01*	2856126	C/T	NA	INTERGENIC	NA
Kabuli_wild_2856534	1399932011	*Ca_kabuli_Chr01*	2856534	T/A	NA	INTERGENIC	NA
Kabuli_wild_2858250	1399932012	*Ca_kabuli_Chr01*	2858250	G/A	NA	INTERGENIC	NA
Kabuli_wild_2858258	1399932013	*Ca_kabuli_Chr01*	2858258	T/C	NA	INTERGENIC	NA
Kabuli_wild_2858263	1399932014	*Ca_kabuli_Chr01*	2858263	A/G	NA	INTERGENIC	NA
Kabuli_wild_2858299	1399932015	*Ca_kabuli_Chr01*	2858299	C/A	NA	INTERGENIC	NA
Kabuli_wild_2868942	1399932016	*Ca_kabuli_Chr01*	2868942	G/A	NA	INTERGENIC	NA
Kabuli_wild_2881386	1399932017	*Ca_kabuli_Chr01*	2881386	G/T	Gene_Ca_00351	NON_SYNONYMOUS_CODING	Disease resistance protein
Kabuli_wild_2881389	1399932018	*Ca_kabuli_Chr01*	2881389	G/C	Gene_Ca_00351	NON_SYNONYMOUS_CODING	Disease resistance protein
Kabuli_wild_2881422	1399932019	*Ca_kabuli_Chr01*	2881422	T/C	Gene_Ca_00351	SYNONYMOUS_CODING	Disease resistance protein
Kabuli_wild_2883625	1399932020	*Ca_kabuli_Chr01*	2883625	T/A	Gene_Ca_00351	STOP_GAINED	Disease resistance protein
Kabuli_wild_2883634	1399932021	*Ca_kabuli_Chr01*	2883634	T/A	Gene_Ca_00351	DOWNSTREAM	Disease resistance protein
Kabuli_wild_2883635	1399932022	*Ca_kabuli_Chr01*	2883635	A/G	Gene_Ca_00351	DOWNSTREAM	Disease resistance protein
Kabuli_wild_2883660	1399932023	*Ca_kabuli_Chr01*	2883660	C/A	Gene_Ca_00351	DOWNSTREAM	Disease resistance protein

SNP IDs	NCBI dbSNP accession IDs (NCBI_SS#)	Chromosomes	Physical positions (bp)	SNPs	Gene accession IDs	Sequence components of genome	Putative functions
Kabuli_wild_2883666	1399932024	*Ca_kabuli_Chr01*	2883666	G/A	Gene_Ca_00351	DOWNSTREAM	Disease resistance protein
Kabuli_wild_2883684	1399932025	*Ca_kabuli_Chr01*	2883684	T/G	Gene_Ca_00351	DOWNSTREAM	Disease resistance protein
Kabuli_wild_2883716	1399932026	*Ca_kabuli_Chr01*	2883716	A/C	Gene_Ca_00351	DOWNSTREAM	Disease resistance protein
Kabuli_wild_2883717	1399932027	*Ca_kabuli_Chr01*	2883717	C/A	Gene_Ca_00351	DOWNSTREAM	Disease resistance protein
Kabuli_wild_2885508	1399932028	*Ca_kabuli_Chr01*	2885508	T/C	Gene_Ca_00351	DOWNSTREAM	Disease resistance protein
Kabuli_wild_2885525	1399932029	*Ca_kabuli_Chr01*	2885525	T/C	Gene_Ca_00351	DOWNSTREAM	Disease resistance protein
Kabuli_wild_2885531	1399932030	*Ca_kabuli_Chr01*	2885531	C/T	Gene_Ca_00351	DOWNSTREAM	Disease resistance protein
Kabuli_wild_2885580	1399932031	*Ca_kabuli_Chr01*	2885580	A/G	Gene_Ca_00351	DOWNSTREAM	Disease resistance protein
Kabuli_wild_2885626	1399932032	*Ca_kabuli_Chr01*	2885626	A/G	Gene_Ca_00351	DOWNSTREAM	Disease resistance protein
Kabuli_wild_2885673	1399932033	*Ca_kabuli_Chr01*	2885673	G/C	NA	INTERGENIC	NA
Kabuli_wild_2885892	1399932034	*Ca_kabuli_Chr01*	2885892	T/A	NA	INTERGENIC	NA
Kabuli_wild_2885923	1399932035	*Ca_kabuli_Chr01*	2885923	C/G	NA	INTERGENIC	NA
Kabuli_wild_2885955	1399932036	*Ca_kabuli_Chr01*	2885955	C/T	NA	INTERGENIC	NA
Kabuli_wild_2885987	1399932037	*Ca_kabuli_Chr01*	2885987	T/A	NA	INTERGENIC	NA
Kabuli_wild_2885988	1399932038	*Ca_kabuli_Chr01*	2885988	C/G	NA	INTERGENIC	NA
Kabuli_wild_2885997	1399932039	*Ca_kabuli_Chr01*	2885997	G/A	NA	INTERGENIC	NA
Kabuli_wild_2886005	1399932040	*Ca_kabuli_Chr01*	2886005	G/A	NA	INTERGENIC	NA
Kabuli_wild_2886025	1399932041	*Ca_kabuli_Chr01*	2886025	A/G	NA	INTERGENIC	NA
Kabuli_wild_2948211	1399932042	*Ca_kabuli_Chr01*	2948211	C/T	NA	INTERGENIC	NA
Kabuli_wild_2948249	1399932043	*Ca_kabuli_Chr01*	2948249	C/A	NA	INTERGENIC	NA
Kabuli_wild_2989941	1399932044	*Ca_kabuli_Chr01*	2989941	G/A	NA	INTERGENIC	NA
Kabuli_wild_3001002	1399932045	*Ca_kabuli_Chr01*	3001002	G/T	Gene_Ca_00364	INTRON	Helicase,C-terminal
Kabuli_wild_3001003	1399932046	*Ca_kabuli_Chr01*	3001003	A/T	Gene_Ca_00364	INTRON	Helicase,C-terminal
Kabuli_wild_3001141	1399932047	*Ca_kabuli_Chr01*	3001141	G/A	Gene_Ca_00364	SYNONYMOUS_CODING	Helicase,C-terminal
Kabuli_wild_3032061	1399932048	*Ca_kabuli_Chr01*	3032061	C/A	NA	INTERGENIC	NA
Kabuli_wild_3047634	1399932049	*Ca_kabuli_Chr01*	3047634	G/A	Gene_Ca_00371	INTRON	Lipase,GDSL
Kabuli_wild_3052100	1399932050	*Ca_kabuli_Chr01*	3052100	T/G	Gene_Ca_00372	UPSTREAM	Thocomplexsubunit7/Mft1p
Kabuli_wild_3052101	1399932051	*Ca_kabuli_Chr01*	3052101	T/A	Gene_Ca_00372	UPSTREAM	Thocomplexsubunit7/Mft1p
Kabuli_wild_3081850	1399932052	*Ca_kabuli_Chr01*	3081850	C/A	Gene_Ca_00376	INTRON	Lipase,GDSL
Kabuli_wild_3081851	1399932053	*Ca_kabuli_Chr01*	3081851	T/G	Gene_Ca_00376	INTRON	Lipase,GDSL
Kabuli_wild_3081902	1399932054	*Ca_kabuli_Chr01*	3081902	A/C	Gene_Ca_00376	INTRON	Lipase,GDSL
Kabuli_wild_3081938	1399932055	*Ca_kabuli_Chr01*	3081938	A/G	Gene_Ca_00376	INTRON	Lipase,GDSL
Kabuli_wild_3081945	1399932056	*Ca_kabuli_Chr01*	3081945	A/G	Gene_Ca_00376	INTRON	Lipase,GDSL
Kabuli_wild_3081954	1399932057	*Ca_kabuli_Chr01*	3081954	G/A	Gene_Ca_00376	INTRON	Lipase,GDSL
Kabuli_wild_3082280	1399932058	*Ca_kabuli_Chr01*	3082280	G/A	Gene_Ca_00376	SYNONYMOUS_CODING	Lipase,GDSL
Kabuli_wild_3114779	1399932059	*Ca_kabuli_Chr01*	3114779	C/T	NA	INTERGENIC	NA
Kabuli_wild_3114789	1399932060	*Ca_kabuli_Chr01*	3114789	T/G	NA	INTERGENIC	NA
Kabuli_wild_3114810	1399932061	*Ca_kabuli_Chr01*	3114810	G/C	NA	INTERGENIC	NA
Kabuli_wild_3114814	1399932062	*Ca_kabuli_Chr01*	3114814	T/C	NA	INTERGENIC	NA
Kabuli_wild_3140881	1399932063	*Ca_kabuli_Chr01*	3140881	T/G	NA	INTERGENIC	NA
Kabuli_wild_3141059	1399932064	*Ca_kabuli_Chr01*	3141059	T/C	NA	INTERGENIC	NA
Kabuli_wild_3145280	1399932065	*Ca_kabuli_Chr01*	3145280	G/A	NA	INTERGENIC	NA
Kabuli_wild_3150964	1399932066	*Ca_kabuli_Chr01*	3150964	C/G	Gene_Ca_00385	UPSTREAM	LPPG:FO2-phospho-L-lactatetransferaseCofD/UPF0052
Kabuli_wild_3150986	1399932067	*Ca_kabuli_Chr01*	3150986	T/C	Gene_Ca_00385	UPSTREAM	LPPG:FO2-phospho-L-lactatetransferaseCofD/UPF0052

SNP IDs	NCBI dbSNP accession IDs (NCBI_SS#)	Chromosomes	Physical positions (bp)	SNPs	Gene accession IDs	Sequence components of genome	Putative functions
Kabuli_wild_3151007	1399932068	*Ca_kabuli_Chr01*	3151007	G/A	Gene_Ca_00385	UPSTREAM	LPPG:FO2-phospho-L-lactatetransferaseCofD/UPF0052
Kabuli_wild_3258027	1399932069	*Ca_kabuli_Chr01*	3258027	G/A	NA	INTERGENIC	NA
Kabuli_wild_3298327	1399932070	*Ca_kabuli_Chr01*	3298327	C/G	NA	INTERGENIC	NA
Kabuli_wild_3338775	1399932071	*Ca_kabuli_Chr01*	3338775	T/G	Gene_Ca_00403	NON_SYNONYMOUS_CODING	Protein kinase,catalytic domain
Kabuli_wild_3347041	1399932072	*Ca_kabuli_Chr01*	3347041	C/G	NA	INTERGENIC	NA
Kabuli_wild_3349259	1399932073	*Ca_kabuli_Chr01*	3349259	C/G	Gene_Ca_00404	DOWNSTREAM	Xanthine/uracil/vitamin C permease
Kabuli_wild_3364952	1399932074	*Ca_kabuli_Chr01*	3364952	A/C	NA	INTERGENIC	NA
Kabuli_wild_3503059	1399932075	*Ca_kabuli_Chr01*	3503059	C/A	Gene_Ca_00425	NON_SYNONYMOUS_CODING	Armadillo
Kabuli_wild_3542263	1399932076	*Ca_kabuli_Chr01*	3542263	A/C	NA	INTERGENIC	NA
Kabuli_wild_3611231	1399932077	*Ca_kabuli_Chr01*	3611231	C/A	NA	INTERGENIC	NA
Kabuli_wild_3711498	1399932078	*Ca_kabuli_Chr01*	3711498	T/G	NA	INTERGENIC	NA
Kabuli_wild_3711525	1399932079	*Ca_kabuli_Chr01*	3711525	T/G	NA	INTERGENIC	NA
Kabuli_wild_3751829	1399932080	*Ca_kabuli_Chr01*	3751829	T/C	Gene_Ca_00456	INTRON	Proteasome-interactingthioredoxin-likedomain,C-terminal
Kabuli_wild_3777137	1399932081	*Ca_kabuli_Chr01*	3777137	T/C	Gene_Ca_00457	DOWNSTREAM	ABC transporter,trans membrane domain
Kabuli_wild_3777292	1399932082	*Ca_kabuli_Chr01*	3777292	C/G	Gene_Ca_00457	DOWNSTREAM	ABC transporter,trans membrane domain
Kabuli_wild_3806415	1399932083	*Ca_kabuli_Chr01*	3806415	G/T	NA	INTERGENIC	NA
Kabuli_wild_3806532	1399932084	*Ca_kabuli_Chr01*	3806532	G/A	NA	INTERGENIC	NA
Kabuli_wild_3852035	1399932085	*Ca_kabuli_Chr01*	3852035	A/C	Gene_Ca_00467	INTRON	AUX/IAAprotein
Kabuli_wild_3852476	1399932086	*Ca_kabuli_Chr01*	3852476	A/C	Gene_Ca_00467	INTRON	AUX/IAAprotein
Kabuli_wild_4073941	1399932087	*Ca_kabuli_Chr01*	4073941	A/C	NA	INTERGENIC	NA
Kabuli_wild_4189333	1399932088	*Ca_kabuli_Chr01*	4189333	C/T	NA	INTERGENIC	NA
Kabuli_wild_4244206	1399932089	*Ca_kabuli_Chr01*	4244206	G/A	NA	INTERGENIC	NA
Kabuli_wild_4244650	1399932090	*Ca_kabuli_Chr01*	4244650	A/C	Gene_Ca_00506	UPSTREAM	RNA recognition motif domain
Kabuli_wild_4245058	1399932091	*Ca_kabuli_Chr01*	4245058	C/G	Gene_Ca_00506	UPSTREAM	RNA recognition motif domain
Kabuli_wild_4245073	1399932092	*Ca_kabuli_Chr01*	4245073	C/G	Gene_Ca_00506	UPSTREAM	RNA recognition motif domain
Kabuli_wild_4245176	1399932093	*Ca_kabuli_Chr01*	4245176	G/T	Gene_Ca_00506	UPSTREAM	RNA recognition motif domain
Kabuli_wild_4292981	1399932094	*Ca_kabuli_Chr01*	4292981	G/C	Gene_Ca_00510	DOWNSTREAM	RibosomalproteinL18a
Kabuli_wild_4302669	1399932095	*Ca_kabuli_Chr01*	4302669	G/C	NA	INTERGENIC	NA
Kabuli_wild_4302751	1399932096	*Ca_kabuli_Chr01*	4302751	T/C	NA	INTERGENIC	NA
Kabuli_wild_4302770	1399932097	*Ca_kabuli_Chr01*	4302770	A/C	NA	INTERGENIC	NA
Kabuli_wild_4334299	1399932098	*Ca_kabuli_Chr01*	4334299	A/T	Gene_Ca_00517	INTRON	Tetra tricopeptide repeat-containing
Kabuli_wild_4334336	1399932099	*Ca_kabuli_Chr01*	4334336	A/G	Gene_Ca_00517	INTRON	Tetra tricopeptide repeat-containing
Kabuli_wild_4375322	1399932100	*Ca_kabuli_Chr01*	4375322	G/C	Gene_Ca_00522	INTRON	Protein kinase,catalytic domain
Kabuli_wild_4375710	1399932101	*Ca_kabuli_Chr01*	4375710	T/C	Gene_Ca_00522	SYNONYMOUS_CODING	Protein kinase,catalytic domain
Kabuli_wild_4386111	1399932102	*Ca_kabuli_Chr01*	4386111	T/C	Gene_Ca_00523	INTRON	SNF2-related
Kabuli_wild_4386114	1399932103	*Ca_kabuli_Chr01*	4386114	T/G	Gene_Ca_00523	INTRON	SNF2-related
Kabuli_wild_4392680	1399932104	*Ca_kabuli_Chr01*	4392680	A/G	Gene_Ca_00524	INTRON	Lipase,class3
Kabuli_wild_4414410	1399932105	*Ca_kabuli_Chr01*	4414410	A/T	NA	INTERGENIC	NA
Kabuli_wild_4418679	1399932106	*Ca_kabuli_Chr01*	4418679	A/G	NA	INTERGENIC	NA
Kabuli_wild_4429044	1399932107	*Ca_kabuli_Chr01*	4429044	C/T	Gene_Ca_00530	NON_SYNONYMOUS_CODING	DNA-directed RNA polymerase,subunit 2, domain 6
Kabuli_wild_4456648	1399932108	*Ca_kabuli_Chr01*	4456648	T/C	Gene_Ca_00531	NON_SYNONYMOUS_CODING	DNA-directed RNA polymerase,subunit 2, domain 6
Kabuli_wild_4456654	1399932109	*Ca_kabuli_Chr01*	4456654	A/C	Gene_Ca_00531	SYNONYMOUS_CODING	DNA-directed RNA polymerase,subunit 2, domain 6
Kabuli_wild_4456662	1399932110	*Ca_kabuli_Chr01*	4456662	G/A	Gene_Ca_00531	NON_SYNONYMOUS_CODING	DNA-directed RNA polymerase,subunit 2, domain 6
Kabuli_wild_4457588	1399932111	*Ca_kabuli_Chr01*	4457588	A/G	Gene_Ca_00531	INTRON	DNA-directed RNA polymerase,subunit 2, domain 6

SNP IDs	NCBI dbSNP accession IDs (NCBI_SS#)	Chromosomes	Physical positions (bp)	SNPs	Gene accession IDs	Sequence components of genome	Putative functions
Kabuli_wild_4460112	1399932112	*Ca_kabuli_Chr01*	4460112	A/T	Gene_Ca_00531	INTRON	DNA-directed RNA polymerase,subunit 2, domain 6
Kabuli_wild_4494270	1399932113	*Ca_kabuli_Chr01*	4494270	C/T	Gene_Ca_00534	SYNONYMOUS_CODING	Protein kinase,catalytic domain
Kabuli_wild_4494467	1399932114	*Ca_kabuli_Chr01*	4494467	A/G	Gene_Ca_00534	INTRON	Protein kinase,catalytic domain
Kabuli_wild_4512566	1399932115	*Ca_kabuli_Chr01*	4512566	T/C	NA	INTERGENIC	NA
Kabuli_wild_4512835	1399932116	*Ca_kabuli_Chr01*	4512835	T/C	NA	INTERGENIC	NA
Kabuli_wild_4528117	1399932117	*Ca_kabuli_Chr01*	4528117	A/G	Gene_Ca_00537	INTRON	NA
Kabuli_wild_4528184	1399932118	*Ca_kabuli_Chr01*	4528184	C/T	Gene_Ca_00537	INTRON	NA
Kabuli_wild_4530304	1399932119	*Ca_kabuli_Chr01*	4530304	T/C	Gene_Ca_00537	SYNONYMOUS_CODING	NA
Kabuli_wild_4560792	1399932120	*Ca_kabuli_Chr01*	4560792	C/T	Gene_Ca_00541	SYNONYMOUS_CODING	Protein kinase,catalytic domain
Kabuli_wild_4560920	1399932121	*Ca_kabuli_Chr01*	4560920	G/A	Gene_Ca_00541	NON_SYNONYMOUS_CODING	Protein kinase,catalytic domain
Kabuli_wild_4571539	1399932122	*Ca_kabuli_Chr01*	4571539	G/A	Gene_Ca_00542	DOWNSTREAM	NA
Kabuli_wild_4571540	1399932123	*Ca_kabuli_Chr01*	4571540	C/G	Gene_Ca_00542	DOWNSTREAM	NA
Kabuli_wild_4576888	1399932124	*Ca_kabuli_Chr01*	4576888	A/G	Gene_Ca_00543	NON_SYNONYMOUS_CODING	BRCT
Kabuli_wild_4578967	1399932125	*Ca_kabuli_Chr01*	4578967	T/G	Gene_Ca_00543	INTRON	BRCT
Kabuli_wild_4578999	1399932126	*Ca_kabuli_Chr01*	4578999	T/A	Gene_Ca_00543	INTRON	BRCT
Kabuli_wild_4582189	1399932127	*Ca_kabuli_Chr01*	4582189	C/T	NA	INTERGENIC	NA
Kabuli_wild_4626099	1399932128	*Ca_kabuli_Chr01*	4626099	T/G	NA	INTERGENIC	NA
Kabuli_wild_4626148	1399932129	*Ca_kabuli_Chr01*	4626148	A/C	NA	INTERGENIC	NA
Kabuli_wild_4626149	1399932130	*Ca_kabuli_Chr01*	4626149	G/C	NA	INTERGENIC	NA
Kabuli_wild_4631467	1399932131	*Ca_kabuli_Chr01*	4631467	G/A	NA	INTERGENIC	NA
Kabuli_wild_4632188	1399932132	*Ca_kabuli_Chr01*	4632188	G/A	NA	INTERGENIC	NA
Kabuli_wild_4632965	1399932133	*Ca_kabuli_Chr01*	4632965	G/A	NA	INTERGENIC	NA
Kabuli_wild_4634413	1399932134	*Ca_kabuli_Chr01*	4634413	C/T	Gene_Ca_00548	SYNONYMOUS_CODING	Dehydrogenase,E1component
Kabuli_wild_4662564	1399932135	*Ca_kabuli_Chr01*	4662564	A/G	Gene_Ca_00550	INTRON	Transcription factor ,SBP-box
Kabuli_wild_4672439	1399932136	*Ca_kabuli_Chr01*	4672439	G/A	Gene_Ca_00551	NON_SYNONYMOUS_CODING	Sugar/inositol transporter
Kabuli_wild_4715563	1399932137	*Ca_kabuli_Chr01*	4715563	T/C	Gene_Ca_00555	NON_SYNONYMOUS_CODING	Protein kinase,catalytic domain
Kabuli_wild_4723311	1399932138	*Ca_kabuli_Chr01*	4723311	C/T	Gene_Ca_00555	INTRON	Protein kinase,catalytic domain
Kabuli_wild_4723345	1399932139	*Ca_kabuli_Chr01*	4723345	C/T	Gene_Ca_00555	INTRON	Protein kinase,catalytic domain
Kabuli_wild_4723367	1399932140	*Ca_kabuli_Chr01*	4723367	A/G	Gene_Ca_00555	INTRON	Protein kinase,catalytic domain
Kabuli_wild_4723434	1399932141	*Ca_kabuli_Chr01*	4723434	A/T	Gene_Ca_00555	INTRON	Protein kinase,catalytic domain
Kabuli_wild_4780172	1399932142	*Ca_kabuli_Chr01*	4780172	T/C	Gene_Ca_00562	SYNONYMOUS_CODING	Zinc finger, RING-type
Kabuli_wild_4780345	1399932143	*Ca_kabuli_Chr01*	4780345	G/T	Gene_Ca_00562	NON_SYNONYMOUS_CODING	Zinc finger, RING-type
Kabuli_wild_4780496	1399932144	*Ca_kabuli_Chr01*	4780496	G/T	Gene_Ca_00562	INTRON	Zinc finger, RING-type
Kabuli_wild_4780544	1399932145	*Ca_kabuli_Chr01*	4780544	C/T	Gene_Ca_00562	INTRON	Zinc finger, RING-type
Kabuli_wild_4781362	1399932146	*Ca_kabuli_Chr01*	4781362	C/T	Gene_Ca_00562	SYNONYMOUS_CODING	Zinc finger, RING-type
Kabuli_wild_4781416	1399932147	*Ca_kabuli_Chr01*	4781416	G/A	Gene_Ca_00562	SYNONYMOUS_CODING	Zinc finger, RING-type
Kabuli_wild_4781704	1399932148	*Ca_kabuli_Chr01*	4781704	G/A	Gene_Ca_00562	SYNONYMOUS_CODING	Zinc finger, RING-type
Kabuli_wild_4808910	1399932149	*Ca_kabuli_Chr01*	4808910	A/T	Gene_Ca_00566	INTRON	SAC3/GANP/Nin1/mts3/eIF-3p25
Kabuli_wild_4878683	1399932150	*Ca_kabuli_Chr01*	4878683	T/C	NA	INTERGENIC	NA
Kabuli_wild_4878691	1399932151	*Ca_kabuli_Chr01*	4878691	A/C	NA	INTERGENIC	NA
Kabuli_wild_4878695	1399932152	*Ca_kabuli_Chr01*	4878695	T/C	NA	INTERGENIC	NA
Kabuli_wild_4878823	1399932153	*Ca_kabuli_Chr01*	4878823	A/G	NA	INTERGENIC	NA
Kabuli_wild_4878845	1399932154	*Ca_kabuli_Chr01*	4878845	G/T	NA	INTERGENIC	NA
Kabuli_wild_4903185	1399932155	*Ca_kabuli_Chr01*	4903185	T/A	NA	INTERGENIC	NA

SNP IDs	NCBI dbSNP accession IDs (NCBI_SS#)	Chromosomes	Physical positions (bp)	SNPs	Gene accession IDs	Sequence components of genome	Putative functions
Kabuli_wild_4906816	1399932156	*Ca_kabuli_Chr01*	4906816	A/G	Gene_Ca_00577	DOWNSTREAM	Disease resistance protein
Kabuli_wild_4906854	1399932157	*Ca_kabuli_Chr01*	4906854	A/G	Gene_Ca_00577	DOWNSTREAM	Disease resistance protein
Kabuli_wild_4906859	1399932158	*Ca_kabuli_Chr01*	4906859	T/C	Gene_Ca_00577	DOWNSTREAM	Disease resistance protein
Kabuli_wild_4906862	1399932159	*Ca_kabuli_Chr01*	4906862	C/G	Gene_Ca_00577	DOWNSTREAM	Disease resistance protein
Kabuli_wild_4909253	1399932160	*Ca_kabuli_Chr01*	4909253	A/G	Gene_Ca_00577	SYNONYMOUS_CODING	Disease resistance protein
Kabuli_wild_4909268	1399932161	*Ca_kabuli_Chr01*	4909268	C/T	Gene_Ca_00577	SYNONYMOUS_CODING	Disease resistance protein
Kabuli_wild_4909271	1399932162	*Ca_kabuli_Chr01*	4909271	A/G	Gene_Ca_00577	SYNONYMOUS_CODING	Disease resistance protein
Kabuli_wild_4909286	1399932163	*Ca_kabuli_Chr01*	4909286	T/G	Gene_Ca_00577	SYNONYMOUS_CODING	Disease resistance protein
Kabuli_wild_4910494	1399932164	*Ca_kabuli_Chr01*	4910494	C/A	Gene_Ca_00577	SYNONYMOUS_CODING	Disease resistance protein
Kabuli_wild_4910497	1399932165	*Ca_kabuli_Chr01*	4910497	A/G	Gene_Ca_00577	SYNONYMOUS_CODING	Disease resistance protein
Kabuli_wild_4910553	1399932166	*Ca_kabuli_Chr01*	4910553	T/C	Gene_Ca_00577	UPSTREAM	Disease resistance protein
Kabuli_wild_4948608	1399932167	*Ca_kabuli_Chr01*	4948608	G/A	Gene_Ca_00583	DOWNSTREAM	Protein synthesis factor, GTP-binding
Kabuli_wild_4948669	1399932168	*Ca_kabuli_Chr01*	4948669	T/C	Gene_Ca_00583	DOWNSTREAM	Protein synthesis factor, GTP-binding
Kabuli_wild_4948740	1399932169	*Ca_kabuli_Chr01*	4948740	A/T	Gene_Ca_00583	DOWNSTREAM	Protein synthesis factor, GTP-binding
Kabuli_wild_4948764	1399932170	*Ca_kabuli_Chr01*	4948764	G/T	Gene_Ca_00583	DOWNSTREAM	Protein synthesis factor, GTP-binding
Kabuli_wild_4949509	1399932171	*Ca_kabuli_Chr01*	4949509	A/G	Gene_Ca_00583	DOWNSTREAM	Protein synthesis factor, GTP-binding
Kabuli_wild_4949578	1399932172	*Ca_kabuli_Chr01*	4949578	A/T	NA	INTERGENIC	NA
Kabuli_wild_4950726	1399932173	*Ca_kabuli_Chr01*	4950726	G/T	NA	INTERGENIC	NA
Kabuli_wild_4959562	1399932174	*Ca_kabuli_Chr01*	4959562	A/C	NA	INTERGENIC	NA
Kabuli_wild_4961673	1399932175	*Ca_kabuli_Chr01*	4961673	A/G	Gene_Ca_00586	INTRON	Protein kinase,catalytic domain
Kabuli_wild_4996404	1399932176	*Ca_kabuli_Chr01*	4996404	A/C	Gene_Ca_00587	SYNONYMOUS_CODING	NA
Kabuli_wild_4996459	1399932177	*Ca_kabuli_Chr01*	4996459	T/C	Gene_Ca_00587	SYNONYMOUS_CODING	NA
Kabuli_wild_4996476	1399932178	*Ca_kabuli_Chr01*	4996476	G/A	Gene_Ca_00587	SYNONYMOUS_CODING	NA
Kabuli_wild_4996536	1399932179	*Ca_kabuli_Chr01*	4996536	C/T	Gene_Ca_00587	SYNONYMOUS_CODING	NA
Kabuli_wild_5071158	1399932180	*Ca_kabuli_Chr01*	5071158	G/A	Gene_Ca_00593	INTRON	RNA recognition motif domain
Kabuli_wild_5072022	1399932181	*Ca_kabuli_Chr01*	5072022	C/A	Gene_Ca_00593	SYNONYMOUS_CODING	RNA recognition motif domain
Kabuli_wild_5080345	1399932182	*Ca_kabuli_Chr01*	5080345	A/G	NA	INTERGENIC	NA
Kabuli_wild_5080358	1399932183	*Ca_kabuli_Chr01*	5080358	G/A	NA	INTERGENIC	NA
Kabuli_wild_5080445	1399932184	*Ca_kabuli_Chr01*	5080445	T/A	NA	INTERGENIC	NA
Kabuli_wild_5087428	1399932185	*Ca_kabuli_Chr01*	5087428	T/C	NA	INTERGENIC	NA
Kabuli_wild_5093607	1399932186	*Ca_kabuli_Chr01*	5093607	T/C	Gene_Ca_00597	DOWNSTREAM	NA
Kabuli_wild_5101381	1399932187	*Ca_kabuli_Chr01*	5101381	G/A	Gene_Ca_00599	DOWNSTREAM	Adenosine/AMPdeaminase
Kabuli_wild_5101449	1399932188	*Ca_kabuli_Chr01*	5101449	T/A	Gene_Ca_00599	DOWNSTREAM	Adenosine/AMPdeaminase
Kabuli_wild_5101453	1399932189	*Ca_kabuli_Chr01*	5101453	G/C	Gene_Ca_00599	DOWNSTREAM	Adenosine/AMPdeaminase
Kabuli_wild_5122663	1399932190	*Ca_kabuli_Chr01*	5122663	A/G	Gene_Ca_00602	DOWNSTREAM	Glycosidehydrolase,family1
Kabuli_wild_5140801	1399932191	*Ca_kabuli_Chr01*	5140801	C/A	NA	INTERGENIC	NA
Kabuli_wild_5141009	1399932192	*Ca_kabuli_Chr01*	5141009	G/C	NA	INTERGENIC	NA
Kabuli_wild_5146657	1399932193	*Ca_kabuli_Chr01*	5146657	G/A	NA	INTERGENIC	NA
Kabuli_wild_5146658	1399932194	*Ca_kabuli_Chr01*	5146658	C/A	NA	INTERGENIC	NA
Kabuli_wild_5146696	1399932195	*Ca_kabuli_Chr01*	5146696	T/A	NA	INTERGENIC	NA
Kabuli_wild_5164127	1399932196	*Ca_kabuli_Chr01*	5164127	A/G	Gene_Ca_00607	DOWNSTREAM	Domain of unknown function DUF231,plant
Kabuli_wild_5164136	1399932197	*Ca_kabuli_Chr01*	5164136	C/T	Gene_Ca_00607	DOWNSTREAM	Domain of unknown function DUF231,plant
Kabuli_wild_5164198	1399932198	*Ca_kabuli_Chr01*	5164198	C/A	Gene_Ca_00607	DOWNSTREAM	Domain of unknown function DUF231,plant
Kabuli_wild_5197461	1399932199	*Ca_kabuli_Chr01*	5197461	C/T	Gene_Ca_00608	INTRON	Uncharacterised protein family,carbohydratekinase-related

SNP IDs	NCBI dbSNP accession IDs (NCBI_SS#)	Chromosomes	Physical positions (bp)	SNPs	Gene accession IDs	Sequence components of genome	Putative functions
Kabuli_wild_5197475	1399932200	*Ca_kabuli_Chr01*	5197475	T/A	Gene_Ca_00608	INTRON	Uncharacterised protein family,carbohydratekinase-related
Kabuli_wild_5197531	1399932201	*Ca_kabuli_Chr01*	5197531	T/G	Gene_Ca_00608	INTRON	Uncharacterised protein family,carbohydratekinase-related
Kabuli_wild_5197540	1399932202	*Ca_kabuli_Chr01*	5197540	G/T	Gene_Ca_00608	INTRON	Uncharacterised protein family,carbohydratekinase-related
Kabuli_wild_5200250	1399932203	*Ca_kabuli_Chr01*	5200250	T/G	Gene_Ca_00608	INTRON	Uncharacterised protein family,carbohydratekinase-related
Kabuli_wild_5200289	1399932204	*Ca_kabuli_Chr01*	5200289	G/T	Gene_Ca_00608	INTRON	Uncharacterised protein family,carbohydratekinase-related
Kabuli_wild_5249839	1399932205	*Ca_kabuli_Chr01*	5249839	G/A	Gene_Ca_00613	NON_SYNONYMOUS_CODING	Protein of unknown function DUF584
Kabuli_wild_5249846	1399932206	*Ca_kabuli_Chr01*	5249846	A/C	Gene_Ca_00613	SYNONYMOUS_CODING	Protein of unknown function DUF584
Kabuli_wild_5249868	1399932207	*Ca_kabuli_Chr01*	5249868	G/A	Gene_Ca_00613	SYNONYMOUS_CODING	Protein of unknown function DUF584
Kabuli_wild_5288262	1399932208	*Ca_kabuli_Chr01*	5288262	T/G	Gene_Ca_00616	SYNONYMOUS_CODING	Heavymetal transport/detoxification protein
Kabuli_wild_5288421	1399932209	*Ca_kabuli_Chr01*	5288421	A/C	Gene_Ca_00616	SYNONYMOUS_CODING	Heavymetal transport/detoxification protein
Kabuli_wild_5321398	1399932210	*Ca_kabuli_Chr01*	5321398	A/G	Gene_Ca_00618	DOWNSTREAM	RassmallGTPase,Rabtype
Kabuli_wild_5430700	1399932211	*Ca_kabuli_Chr01*	5430700	A/C	Gene_Ca_00628	SYNONYMOUS_CODING	Protein of unknown function DUF584
Kabuli_wild_5430703	1399932212	*Ca_kabuli_Chr01*	5430703	T/G	Gene_Ca_00628	SYNONYMOUS_CODING	Protein of unknown function DUF584
Kabuli_wild_5430809	1399932213	*Ca_kabuli_Chr01*	5430809	G/A	Gene_Ca_00628	NON_SYNONYMOUS_CODING	Protein of unknown function DUF584
Kabuli_wild_5444130	1399932214	*Ca_kabuli_Chr01*	5444130	A/C	Gene_Ca_00630	UPSTREAM	NA
Kabuli_wild_5444142	1399932215	*Ca_kabuli_Chr01*	5444142	A/T	Gene_Ca_00630	UPSTREAM	NA
Kabuli_wild_5487535	1399932216	*Ca_kabuli_Chr01*	5487535	A/G	Gene_Ca_00634	NON_SYNONYMOUS_CODING	NA
Kabuli_wild_5489806	1399932217	*Ca_kabuli_Chr01*	5489806	C/G	Gene_Ca_00634	INTRON	NA
Kabuli_wild_5498150	1399932218	*Ca_kabuli_Chr01*	5498150	G/A	NA	INTERGENIC	NA
Kabuli_wild_5498164	1399932219	*Ca_kabuli_Chr01*	5498164	C/T	NA	INTERGENIC	NA
Kabuli_wild_5498186	1399932220	*Ca_kabuli_Chr01*	5498186	G/T	NA	INTERGENIC	NA
Kabuli_wild_5607717	1399932221	*Ca_kabuli_Chr01*	5607717	T/A	NA	INTERGENIC	NA
Kabuli_wild_5607745	1399932222	*Ca_kabuli_Chr01*	5607745	A/T	NA	INTERGENIC	NA
Kabuli_wild_5635435	1399932223	*Ca_kabuli_Chr01*	5635435	G/A	Gene_Ca_00651	UPSTREAM	NA
Kabuli_wild_5637388	1399932224	*Ca_kabuli_Chr01*	5637388	T/A	NA	INTERGENIC	NA
Kabuli_wild_5637442	1399932225	*Ca_kabuli_Chr01*	5637442	C/T	NA	INTERGENIC	NA
Kabuli_wild_5666178	1399932226	*Ca_kabuli_Chr01*	5666178	A/C	Gene_Ca_00656	SYNONYMOUS_CODING	DNA-binding WRKY
Kabuli_wild_5666217	1399932227	*Ca_kabuli_Chr01*	5666217	C/A	Gene_Ca_00656	NON_SYNONYMOUS_CODING	DNA-binding WRKY
Kabuli_wild_5666260	1399932228	*Ca_kabuli_Chr01*	5666260	T/G	Gene_Ca_00656	SYNONYMOUS_CODING	DNA-binding WRKY
Kabuli_wild_5666263	1399932229	*Ca_kabuli_Chr01*	5666263	T/G	Gene_Ca_00656	SYNONYMOUS_CODING	DNA-binding WRKY
Kabuli_wild_5666266	1399932230	*Ca_kabuli_Chr01*	5666266	T/G	Gene_Ca_00656	NON_SYNONYMOUS_CODING	DNA-binding WRKY
Kabuli_wild_5666355	1399932231	*Ca_kabuli_Chr01*	5666355	T/G	Gene_Ca_00656	NON_SYNONYMOUS_CODING	DNA-binding WRKY
Kabuli_wild_5666401	1399932232	*Ca_kabuli_Chr01*	5666401	T/G	Gene_Ca_00656	SYNONYMOUS_CODING	DNA-binding WRKY
Kabuli_wild_5667605	1399932233	*Ca_kabuli_Chr01*	5667605	G/A	Gene_Ca_00656	SYNONYMOUS_CODING	DNA-binding WRKY
Kabuli_wild_5706344	1399932234	*Ca_kabuli_Chr01*	5706344	A/C	Gene_Ca_00662	SYNONYMOUS_CODING	Glycosyltransferase,family14
Kabuli_wild_5706345	1399932235	*Ca_kabuli_Chr01*	5706345	G/T	Gene_Ca_00662	NON_SYNONYMOUS_CODING	Glycosyltransferase,family14
Kabuli_wild_5706858	1399932236	*Ca_kabuli_Chr01*	5706858	A/T	Gene_Ca_00663	DOWNSTREAM	Transcription factor jumonji/aspartylbeta-hydroxylase
Kabuli_wild_5706920	1399932237	*Ca_kabuli_Chr01*	5706920	G/A	Gene_Ca_00663	DOWNSTREAM	Transcription factor jumonji/aspartylbeta-hydroxylase
Kabuli_wild_5706944	1399932238	*Ca_kabuli_Chr01*	5706944	T/G	Gene_Ca_00663	DOWNSTREAM	Transcription factor jumonji/aspartylbeta-hydroxylase
Kabuli_wild_5706948	1399932239	*Ca_kabuli_Chr01*	5706948	A/T	Gene_Ca_00663	DOWNSTREAM	Transcription factor jumonji/aspartylbeta-hydroxylase
Kabuli_wild_5706961	1399932240	*Ca_kabuli_Chr01*	5706961	G/T	Gene_Ca_00663	DOWNSTREAM	Transcription factor jumonji/aspartylbeta-hydroxylase
Kabuli_wild_5721616	1399932241	*Ca_kabuli_Chr01*	5721616	A/T	Gene_Ca_00664	INTRON	Protein kinase,catalytic domain
Kabuli_wild_5749456	1399932242	*Ca_kabuli_Chr01*	5749456	C/T	NA	INTERGENIC	NA
Kabuli_wild_5791118	1399932243	*Ca_kabuli_Chr01*	5791118	T/G	NA	INTERGENIC	NA

SNP IDs	NCBI dbSNP accession IDs (NCBI_SS#)	Chromosomes	Physical positions (bp)	SNPs	Gene accession IDs	Sequence components of genome	Putative functions
Kabuli_wild_5793815	1399932244	*Ca_kabuli_Chr01*	5793815	G/A	NA	INTERGENIC	NA
Kabuli_wild_5796146	1399932245	*Ca_kabuli_Chr01*	5796146	C/T	NA	INTERGENIC	NA
Kabuli_wild_5796886	1399932246	*Ca_kabuli_Chr01*	5796886	A/G	Gene_Ca_00670	INTRON	UBX
Kabuli_wild_5807492	1399932247	*Ca_kabuli_Chr01*	5807492	T/C	Gene_Ca_00671	UPSTREAM	ATPase,AAA+type,core
Kabuli_wild_5812283	1399932248	*Ca_kabuli_Chr01*	5812283	G/T	NA	INTERGENIC	NA
Kabuli_wild_5862565	1399932249	*Ca_kabuli_Chr01*	5862565	C/T	NA	INTERGENIC	NA
Kabuli_wild_6011689	1399932250	*Ca_kabuli_Chr01*	6011689	G/A	NA	INTERGENIC	NA
Kabuli_wild_6011697	1399932251	*Ca_kabuli_Chr01*	6011697	T/G	NA	INTERGENIC	NA
Kabuli_wild_6011761	1399932252	*Ca_kabuli_Chr01*	6011761	C/T	NA	INTERGENIC	NA
Kabuli_wild_6066933	1399932253	*Ca_kabuli_Chr01*	6066933	C/T	Gene_Ca_00693	INTRON	RHO protein GDP dissociation inhibitor
Kabuli_wild_6066936	1399932254	*Ca_kabuli_Chr01*	6066936	A/G	Gene_Ca_00693	INTRON	RHO protein GDP dissociation inhibitor
Kabuli_wild_6066972	1399932255	*Ca_kabuli_Chr01*	6066972	C/T	Gene_Ca_00693	INTRON	RHO protein GDP dissociation inhibitor
Kabuli_wild_6067846	1399932256	*Ca_kabuli_Chr01*	6067846	T/C	Gene_Ca_00693	INTRON	RHO protein GDP dissociation inhibitor
Kabuli_wild_6088283	1399932257	*Ca_kabuli_Chr01*	6088283	C/T	NA	INTERGENIC	NA
Kabuli_wild_6126772	1399932258	*Ca_kabuli_Chr01*	6126772	G/A	Gene_Ca_00697	INTRON	RibosomalproteinL14b/L23e
Kabuli_wild_6257653	1399932259	*Ca_kabuli_Chr01*	6257653	C/G	Gene_Ca_07885	INTRON	Thymidinekinase
Kabuli_wild_6257712	1399932260	*Ca_kabuli_Chr01*	6257712	A/T	Gene_Ca_07885	INTRON	Thymidinekinase
Kabuli_wild_6262461	1399932261	*Ca_kabuli_Chr01*	6262461	A/G	NA	INTERGENIC	NA
Kabuli_wild_6262529	1399932262	*Ca_kabuli_Chr01*	6262529	T/G	NA	INTERGENIC	NA
Kabuli_wild_6262577	1399932263	*Ca_kabuli_Chr01*	6262577	A/G	NA	INTERGENIC	NA
Kabuli_wild_6268630	1399932264	*Ca_kabuli_Chr01*	6268630	G/A	Gene_Ca_07887	UPSTREAM	Transcription facto rGRAS
Kabuli_wild_6268776	1399932265	*Ca_kabuli_Chr01*	6268776	C/T	Gene_Ca_07887	UPSTREAM	Transcription facto rGRAS
Kabuli_wild_6278572	1399932266	*Ca_kabuli_Chr01*	6278572	T/C	NA	INTERGENIC	NA
Kabuli_wild_6278625	1399932267	*Ca_kabuli_Chr01*	6278625	T/C	NA	INTERGENIC	NA
Kabuli_wild_6279644	1399932268	*Ca_kabuli_Chr01*	6279644	G/T	NA	INTERGENIC	NA
Kabuli_wild_6296858	1399932269	*Ca_kabuli_Chr01*	6296858	G/A	Gene_Ca_07890	INTRON	Uncharacterised protein family UPF0497,trans-membrane plant
Kabuli_wild_6296986	1399932270	*Ca_kabuli_Chr01*	6296986	C/A	Gene_Ca_07890	INTRON	Uncharacterised protein family UPF0497,trans-membrane plant
Kabuli_wild_6315337	1399932271	*Ca_kabuli_Chr01*	6315337	G/T	Gene_Ca_07892	INTRON	Zinc finger,ZZ-type
Kabuli_wild_6315397	1399932272	*Ca_kabuli_Chr01*	6315397	C/G	Gene_Ca_07892	INTRON	Zinc finger,ZZ-type
Kabuli_wild_6315399	1399932273	*Ca_kabuli_Chr01*	6315399	T/G	Gene_Ca_07892	INTRON	Zinc finger,ZZ-type
Kabuli_wild_6315402	1399932274	*Ca_kabuli_Chr01*	6315402	C/T	Gene_Ca_07892	INTRON	Zinc finger,ZZ-type
Kabuli_wild_6328801	1399932275	*Ca_kabuli_Chr01*	6328801	T/A	NA	INTERGENIC	NA
Kabuli_wild_6328957	1399932276	*Ca_kabuli_Chr01*	6328957	C/T	NA	INTERGENIC	NA
Kabuli_wild_6328963	1399932277	*Ca_kabuli_Chr01*	6328963	A/G	NA	INTERGENIC	NA
Kabuli_wild_6329466	1399932278	*Ca_kabuli_Chr01*	6329466	G/A	Gene_Ca_07893	SYNONYMOUS_CODING	Bromoadjacenthomology(BAH)domain
Kabuli_wild_6329814	1399932279	*Ca_kabuli_Chr01*	6329814	T/A	Gene_Ca_07893	SYNONYMOUS_CODING	Bromoadjacenthomology(BAH)domain
Kabuli_wild_6329847	1399932280	*Ca_kabuli_Chr01*	6329847	A/G	Gene_Ca_07893	SYNONYMOUS_CODING	Bromoadjacenthomology(BAH)domain
Kabuli_wild_6331686	1399932281	*Ca_kabuli_Chr01*	6331686	G/A	Gene_Ca_07893	INTRON	Bromoadjacenthomology(BAH)domain
Kabuli_wild_6332104	1399932282	*Ca_kabuli_Chr01*	6332104	G/A	Gene_Ca_07893	INTRON	Bromoadjacenthomology(BAH)domain
Kabuli_wild_6381191	1399932283	*Ca_kabuli_Chr01*	6381191	T/C	Gene_Ca_07900	DOWNSTREAM	NA
Kabuli_wild_6390541	1399932284	*Ca_kabuli_Chr01*	6390541	T/A	Gene_Ca_07902	DOWNSTREAM	Smrprotein/MutS2C-terminal
Kabuli_wild_6390775	1399932285	*Ca_kabuli_Chr01*	6390775	A/C	Gene_Ca_07901	UPSTREAM	Smrprotein/MutS2C-terminal
Kabuli_wild_6390849	1399932286	*Ca_kabuli_Chr01*	6390849	G/A	NA	INTERGENIC	NA
Kabuli_wild_6390882	1399932287	*Ca_kabuli_Chr01*	6390882	T/C	NA	INTERGENIC	NA

SNP IDs	NCBI dbSNP accession IDs (NCBI_SS#)	Chromosomes	Physical positions (bp)	SNPs	Gene accession IDs	Sequence components of genome	Putative functions
Kabuli_wild_6391017	1399932288	*Ca_kabuli_Chr01*	6391017	T/C	NA	INTERGENIC	NA
Kabuli_wild_6414867	1399932289	*Ca_kabuli_Chr01*	6414867	T/C	Gene_Ca_07905	INTRON	Heavymetal transport/detoxification protein
Kabuli_wild_6433592	1399932290	*Ca_kabuli_Chr01*	6433592	T/G	NA	INTERGENIC	NA
Kabuli_wild_6433826	1399932291	*Ca_kabuli_Chr01*	6433826	G/A	Gene_Ca_07907	SYNONYMOUS_CODING	NUDIX hydrolase domain
Kabuli_wild_6433838	1399932292	*Ca_kabuli_Chr01*	6433838	C/T	Gene_Ca_07907	SYNONYMOUS_CODING	NUDIX hydrolase domain
Kabuli_wild_6433841	1399932293	*Ca_kabuli_Chr01*	6433841	C/T	Gene_Ca_07907	SYNONYMOUS_CODING	NUDIX hydrolase domain
Kabuli_wild_6433883	1399932294	*Ca_kabuli_Chr01*	6433883	A/G	Gene_Ca_07907	SYNONYMOUS_CODING	NUDIX hydrolase domain
Kabuli_wild_6433925	1399932295	*Ca_kabuli_Chr01*	6433925	C/T	Gene_Ca_07907	SYNONYMOUS_CODING	NUDIX hydrolase domain
Kabuli_wild_6433934	1399932296	*Ca_kabuli_Chr01*	6433934	T/A	Gene_Ca_07907	SYNONYMOUS_CODING	NUDIX hydrolase domain
Kabuli_wild_6435499	1399932297	*Ca_kabuli_Chr01*	6435499	T/C	Gene_Ca_07907	INTRON	NUDIX hydrolase domain
Kabuli_wild_6437643	1399932298	*Ca_kabuli_Chr01*	6437643	G/A	NA	INTERGENIC	NA
Kabuli_wild_6470708	1399932299	*Ca_kabuli_Chr01*	6470708	T/C	Gene_Ca_07910	SYNONYMOUS_CODING	tRNA(guanine-N1-)-methyltransferase,eukaryotic
Kabuli_wild_6478736	1399932300	*Ca_kabuli_Chr01*	6478736	A/G	NA	INTERGENIC	NA
Kabuli_wild_6478758	1399932301	*Ca_kabuli_Chr01*	6478758	A/G	NA	INTERGENIC	NA
Kabuli_wild_6486446	1399932302	*Ca_kabuli_Chr01*	6486446	G/C	Gene_Ca_07911	INTRON	NA
Kabuli_wild_6490978	1399932303	*Ca_kabuli_Chr01*	6490978	A/T	Gene_Ca_07911	INTRON	NA
Kabuli_wild_6491008	1399932304	*Ca_kabuli_Chr01*	6491008	A/T	Gene_Ca_07911	INTRON	NA
Kabuli_wild_6491017	1399932305	*Ca_kabuli_Chr01*	6491017	T/C	Gene_Ca_07911	INTRON	NA
Kabuli_wild_6491030	1399932306	*Ca_kabuli_Chr01*	6491030	T/G	Gene_Ca_07911	INTRON	NA
Kabuli_wild_6521670	1399932307	*Ca_kabuli_Chr01*	6521670	C/T	NA	INTERGENIC	NA
Kabuli_wild_6521673	1399932308	*Ca_kabuli_Chr01*	6521673	G/A	NA	INTERGENIC	NA
Kabuli_wild_6521856	1399932309	*Ca_kabuli_Chr01*	6521856	A/G	NA	INTERGENIC	NA
Kabuli_wild_6521859	1399932310	*Ca_kabuli_Chr01*	6521859	T/A	NA	INTERGENIC	NA
Kabuli_wild_6521970	1399932311	*Ca_kabuli_Chr01*	6521970	C/T	Gene_Ca_07917	DOWNSTREAM	Cellular retinaldehyde-binding/triplefunction, C-terminal
Kabuli_wild_6540056	1399932312	*Ca_kabuli_Chr01*	6540056	C/T	Gene_Ca_07918	DOWNSTREAM	NA
Kabuli_wild_6540170	1399932313	*Ca_kabuli_Chr01*	6540170	G/A	Gene_Ca_07918	DOWNSTREAM	NA
Kabuli_wild_6551979	1399932314	*Ca_kabuli_Chr01*	6551979	A/C	NA	INTERGENIC	NA
Kabuli_wild_6551981	1399932315	*Ca_kabuli_Chr01*	6551981	T/C	NA	INTERGENIC	NA
Kabuli_wild_6552001	1399932316	*Ca_kabuli_Chr01*	6552001	T/C	NA	INTERGENIC	NA
Kabuli_wild_6552013	1399932317	*Ca_kabuli_Chr01*	6552013	G/A	NA	INTERGENIC	NA
Kabuli_wild_6553107	1399932318	*Ca_kabuli_Chr01*	6553107	A/C	Gene_Ca_07920	INTRON	NA
Kabuli_wild_6554029	1399932319	*Ca_kabuli_Chr01*	6554029	A/C	Gene_Ca_07920	INTRON	NA
Kabuli_wild_6564716	1399932320	*Ca_kabuli_Chr01*	6564716	C/T	Gene_Ca_07921	SYNONYMOUS_CODING	Polyprenylsynthetase
Kabuli_wild_6591506	1399932321	*Ca_kabuli_Chr01*	6591506	G/C	NA	INTERGENIC	NA
Kabuli_wild_6599917	1399932322	*Ca_kabuli_Chr01*	6599917	C/A	NA	INTERGENIC	NA
Kabuli_wild_6634482	1399932323	*Ca_kabuli_Chr01*	6634482	G/C	NA	INTERGENIC	NA
Kabuli_wild_6635366	1399932324	*Ca_kabuli_Chr01*	6635366	T/C	NA	INTERGENIC	NA
Kabuli_wild_6658519	1399932325	*Ca_kabuli_Chr01*	6658519	A/T	NA	INTERGENIC	NA
Kabuli_wild_6658558	1399932326	*Ca_kabuli_Chr01*	6658558	T/C	NA	INTERGENIC	NA
Kabuli_wild_6679253	1399932327	*Ca_kabuli_Chr01*	6679253	A/G	Gene_Ca_07932	SYNONYMOUS_CODING	WD40 repeat
Kabuli_wild_6679329	1399932328	*Ca_kabuli_Chr01*	6679329	A/G	Gene_Ca_07932	NON_SYNONYMOUS_CODING	WD40 repeat
Kabuli_wild_6765539	1399932329	*Ca_kabuli_Chr01*	6765539	C/T	Gene_Ca_07941	INTRON	Histidine phosphatase superfamily,clade-1
Kabuli_wild_6803364	1399932330	*Ca_kabuli_Chr01*	6803364	G/A	NA	INTERGENIC	NA
Kabuli_wild_6804110	1399932331	*Ca_kabuli_Chr01*	6804110	A/T	NA	INTERGENIC	NA

SNP IDs	NCBI dbSNP accession IDs (NCBI_SS#)	Chromosomes	Physical positions (bp)	SNPs	Gene accession IDs	Sequence components of genome	Putative functions
Kabuli_wild_6833811	1399932332	*Ca_kabuli_Chr01*	6833811	G/A	NA	INTERGENIC	NA
Kabuli_wild_6833846	1399932333	*Ca_kabuli_Chr01*	6833846	C/A	NA	INTERGENIC	NA
Kabuli_wild_6848560	1399932334	*Ca_kabuli_Chr01*	6848560	A/C	Gene_Ca_07951	INTRON	Vps53-like,N-terminal
Kabuli_wild_6849437	1399932335	*Ca_kabuli_Chr01*	6849437	C/T	Gene_Ca_07951	INTRON	Vps53-like,N-terminal
Kabuli_wild_6849512	1399932336	*Ca_kabuli_Chr01*	6849512	G/C	Gene_Ca_07951	INTRON	Vps53-like,N-terminal
Kabuli_wild_6880837	1399932337	*Ca_kabuli_Chr01*	6880837	A/G	NA	INTERGENIC	NA
Kabuli_wild_6880867	1399932338	*Ca_kabuli_Chr01*	6880867	T/C	NA	INTERGENIC	NA
Kabuli_wild_6904278	1399932339	*Ca_kabuli_Chr01*	6904278	A/C	Gene_Ca_07956	NON_SYNONYMOUS_CODING	Protein kinase,catalytic domain
Kabuli_wild_6904338	1399932340	*Ca_kabuli_Chr01*	6904338	C/T	Gene_Ca_07956	INTRON	Protein kinase,catalytic domain
Kabuli_wild_6988656	1399932341	*Ca_kabuli_Chr01*	6988656	A/C	NA	INTERGENIC	NA
Kabuli_wild_7050825	1399932342	*Ca_kabuli_Chr01*	7050825	G/A	NA	INTERGENIC	NA
Kabuli_wild_7050953	1399932343	*Ca_kabuli_Chr01*	7050953	C/T	NA	INTERGENIC	NA
Kabuli_wild_7050958	1399932344	*Ca_kabuli_Chr01*	7050958	C/T	NA	INTERGENIC	NA
Kabuli_wild_7104985	1399932345	*Ca_kabuli_Chr01*	7104985	G/T	NA	INTERGENIC	NA
Kabuli_wild_7127843	1399932346	*Ca_kabuli_Chr01*	7127843	A/G	Gene_Ca_07973	SYNONYMOUS_CODING	Sec8 exocyst complex component specific domain
Kabuli_wild_7136677	1399932347	*Ca_kabuli_Chr01*	7136677	T/C	Gene_Ca_07973	DOWNSTREAM	Sec8 exocyst complex component specific domain
Kabuli_wild_7147651	1399932348	*Ca_kabuli_Chr01*	7147651	T/A	Gene_Ca_07974	INTRON	NA
Kabuli_wild_7154433	1399932349	*Ca_kabuli_Chr01*	7154433	C/T	Gene_Ca_07974	INTRON	NA
Kabuli_wild_7155344	1399932350	*Ca_kabuli_Chr01*	7155344	A/G	Gene_Ca_07974	INTRON	NA
Kabuli_wild_7157057	1399932351	*Ca_kabuli_Chr01*	7157057	A/C	Gene_Ca_07974	INTRON	NA
Kabuli_wild_7157112	1399932352	*Ca_kabuli_Chr01*	7157112	T/C	Gene_Ca_07974	INTRON	NA
Kabuli_wild_7160649	1399932353	*Ca_kabuli_Chr01*	7160649	T/C	Gene_Ca_07974	INTRON	NA
Kabuli_wild_7160695	1399932354	*Ca_kabuli_Chr01*	7160695	G/A	Gene_Ca_07974	INTRON	NA
Kabuli_wild_7160754	1399932355	*Ca_kabuli_Chr01*	7160754	A/T	Gene_Ca_07974	INTRON	NA
Kabuli_wild_7160773	1399932356	*Ca_kabuli_Chr01*	7160773	G/A	Gene_Ca_07974	INTRON	NA
Kabuli_wild_7160781	1399932357	*Ca_kabuli_Chr01*	7160781	A/C	Gene_Ca_07974	INTRON	NA
Kabuli_wild_7160868	1399932358	*Ca_kabuli_Chr01*	7160868	C/T	Gene_Ca_07974	INTRON	NA
Kabuli_wild_7184813	1399932359	*Ca_kabuli_Chr01*	7184813	A/C	Gene_Ca_07975	SYNONYMOUS_CODING	Root hai rdefective 3GTP-binding
Kabuli_wild_7187934	1399932360	*Ca_kabuli_Chr01*	7187934	A/G	Gene_Ca_07976	DOWNSTREAM	NA
Kabuli_wild_7196570	1399932361	*Ca_kabuli_Chr01*	7196570	T/C	NA	INTERGENIC	NA
Kabuli_wild_7196601	1399932362	*Ca_kabuli_Chr01*	7196601	T/C	NA	INTERGENIC	NA
Kabuli_wild_7231750	1399932363	*Ca_kabuli_Chr01*	7231750	A/C	NA	INTERGENIC	NA
Kabuli_wild_7267812	1399932364	*Ca_kabuli_Chr01*	7267812	G/C	Gene_Ca_07984	INTRON	Plantperoxidase
Kabuli_wild_7325164	1399932365	*Ca_kabuli_Chr01*	7325164	T/A	NA	INTERGENIC	NA
Kabuli_wild_7417788	1399932366	*Ca_kabuli_Chr01*	7417788	G/A	Gene_Ca_07994	INTRON	Homeobox
Kabuli_wild_7417997	1399932367	*Ca_kabuli_Chr01*	7417997	A/G	Gene_Ca_07994	SYNONYMOUS_CODING	Homeobox
Kabuli_wild_7439427	1399932368	*Ca_kabuli_Chr01*	7439427	C/T	Gene_Ca_07995	NON_SYNONYMOUS_CODING	CCAAT-bindingTranscription factor ,subunitB
Kabuli_wild_7467280	1399932369	*Ca_kabuli_Chr01*	7467280	A/G	Gene_Ca_07999	INTRON	AMP-dependentsynthetase/ligase
Kabuli_wild_7478022	1399932370	*Ca_kabuli_Chr01*	7478022	G/A	Gene_Ca_08000	SYNONYMOUS_CODING	NA
Kabuli_wild_7478046	1399932371	*Ca_kabuli_Chr01*	7478046	T/G	Gene_Ca_08000	NON_SYNONYMOUS_CODING	NA
Kabuli_wild_7478121	1399932372	*Ca_kabuli_Chr01*	7478121	C/T	Gene_Ca_08000	SYNONYMOUS_CODING	NA
Kabuli_wild_7486102	1399932373	*Ca_kabuli_Chr01*	7486102	C/T	NA	INTERGENIC	NA
Kabuli_wild_7486148	1399932374	*Ca_kabuli_Chr01*	7486148	T/G	NA	INTERGENIC	NA
Kabuli_wild_7486263	1399932375	*Ca_kabuli_Chr01*	7486263	C/A	NA	INTERGENIC	NA

SNP IDs	NCBI dbSNP accession IDs (NCBI_SS#)	Chromosomes	Physical positions (bp)	SNPs	Gene accession IDs	Sequence components of genome	Putative functions
Kabuli_wild_7486273	1399932376	*Ca_kabuli_Chr01*	7486273	T/C	NA	INTERGENIC	NA
Kabuli_wild_7486282	1399932377	*Ca_kabuli_Chr01*	7486282	G/A	NA	INTERGENIC	NA
Kabuli_wild_7497334	1399932378	*Ca_kabuli_Chr01*	7497334	C/A	Gene_Ca_08003	UPSTREAM	Protein kinase,catalytic domain
Kabuli_wild_7497362	1399932379	*Ca_kabuli_Chr01*	7497362	T/C	Gene_Ca_08003	UPSTREAM	Protein kinase,catalytic domain
Kabuli_wild_7500494	1399932380	*Ca_kabuli_Chr01*	7500494	T/G	Gene_Ca_08003	SYNONYMOUS_CODING	Protein kinase,catalytic domain
Kabuli_wild_7500524	1399932381	*Ca_kabuli_Chr01*	7500524	T/C	Gene_Ca_08003	SYNONYMOUS_CODING	Protein kinase,catalytic domain
Kabuli_wild_7500728	1399932382	*Ca_kabuli_Chr01*	7500728	G/A	Gene_Ca_08003	SYNONYMOUS_CODING	Protein kinase,catalytic domain
Kabuli_wild_7505779	1399932383	*Ca_kabuli_Chr01*	7505779	A/T	NA	INTERGENIC	NA
Kabuli_wild_7515002	1399932384	*Ca_kabuli_Chr01*	7515002	T/C	NA	INTERGENIC	NA
Kabuli_wild_7515029	1399932385	*Ca_kabuli_Chr01*	7515029	A/C	NA	INTERGENIC	NA
Kabuli_wild_7515578	1399932386	*Ca_kabuli_Chr01*	7515578	A/C	NA	INTERGENIC	NA
Kabuli_wild_7515605	1399932387	*Ca_kabuli_Chr01*	7515605	T/C	NA	INTERGENIC	NA
Kabuli_wild_7549977	1399932388	*Ca_kabuli_Chr01*	7549977	C/G	Gene_Ca_08008	SYNONYMOUS_CODING	NA
Kabuli_wild_7550130	1399932389	*Ca_kabuli_Chr01*	7550130	A/G	Gene_Ca_08008	INTRON	NA
Kabuli_wild_7550169	1399932390	*Ca_kabuli_Chr01*	7550169	G/A	Gene_Ca_08008	INTRON	NA
Kabuli_wild_7550170	1399932391	*Ca_kabuli_Chr01*	7550170	A/G	Gene_Ca_08008	INTRON	NA
Kabuli_wild_7552484	1399932392	*Ca_kabuli_Chr01*	7552484	T/C	Gene_Ca_08008	SYNONYMOUS_CODING	NA
Kabuli_wild_7552499	1399932393	*Ca_kabuli_Chr01*	7552499	G/A	Gene_Ca_08008	SYNONYMOUS_CODING	NA
Kabuli_wild_7552507	1399932394	*Ca_kabuli_Chr01*	7552507	G/T	Gene_Ca_08008	NON_SYNONYMOUS_CODING	NA
Kabuli_wild_7557751	1399932395	*Ca_kabuli_Chr01*	7557751	T/G	Gene_Ca_08009	INTRON	Histidinol dehydrogenase, conservedsite
Kabuli_wild_7557811	1399932396	*Ca_kabuli_Chr01*	7557811	A/T	Gene_Ca_08009	INTRON	Histidinol dehydrogenase, conservedsite
Kabuli_wild_7690613	1399932397	*Ca_kabuli_Chr01*	7690613	T/G	Gene_Ca_08021	NON_SYNONYMOUS_CODING	NA
Kabuli_wild_7690620	1399932398	*Ca_kabuli_Chr01*	7690620	T/G	Gene_Ca_08021	NON_SYNONYMOUS_CODING	NA
Kabuli_wild_7690625	1399932399	*Ca_kabuli_Chr01*	7690625	A/C	Gene_Ca_08021	SYNONYMOUS_CODING	NA
Kabuli_wild_7695776	1399932400	*Ca_kabuli_Chr01*	7695776	T/C	Gene_Ca_08021	INTRON	NA
Kabuli_wild_7695844	1399932401	*Ca_kabuli_Chr01*	7695844	T/C	Gene_Ca_08021	INTRON	NA
Kabuli_wild_7722337	1399932402	*Ca_kabuli_Chr01*	7722337	T/C	Gene_Ca_08024	SYNONYMOUS_CODING	FADlinkedoxidase,N-terminal
Kabuli_wild_7772059	1399932403	*Ca_kabuli_Chr01*	7772059	G/A	NA	INTERGENIC	NA
Kabuli_wild_7811388	1399932404	*Ca_kabuli_Chr01*	7811388	C/T	NA	INTERGENIC	NA
Kabuli_wild_7925666	1399932405	*Ca_kabuli_Chr01*	7925666	A/C	Gene_Ca_08042	SYNONYMOUS_CODING	Biopterin transport-related protein BT1
Kabuli_wild_7925774	1399932406	*Ca_kabuli_Chr01*	7925774	C/T	Gene_Ca_08042	SYNONYMOUS_CODING	Biopterin transport-related protein BT1
Kabuli_wild_7969202	1399932407	*Ca_kabuli_Chr01*	7969202	A/C	NA	INTERGENIC	NA
Kabuli_wild_7969218	1399932408	*Ca_kabuli_Chr01*	7969218	T/C	NA	INTERGENIC	NA
Kabuli_wild_8036666	1399932409	*Ca_kabuli_Chr01*	8036666	C/T	Gene_Ca_08051	INTRON	NAD-dependentepimerase/dehydratase
Kabuli_wild_8036706	1399932410	*Ca_kabuli_Chr01*	8036706	A/G	Gene_Ca_08051	INTRON	NAD-dependentepimerase/dehydratase
Kabuli_wild_8036903	1399932411	*Ca_kabuli_Chr01*	8036903	A/T	Gene_Ca_08051	INTRON	NAD-dependentepimerase/dehydratase
Kabuli_wild_8036917	1399932412	*Ca_kabuli_Chr01*	8036917	T/G	Gene_Ca_08051	INTRON	NAD-dependentepimerase/dehydratase
Kabuli_wild_8075742	1399932413	*Ca_kabuli_Chr01*	8075742	G/T	NA	INTERGENIC	NA
Kabuli_wild_8075776	1399932414	*Ca_kabuli_Chr01*	8075776	A/C	NA	INTERGENIC	NA
Kabuli_wild_8101072	1399932415	*Ca_kabuli_Chr01*	8101072	C/T	NA	INTERGENIC	NA
Kabuli_wild_8107967	1399932416	*Ca_kabuli_Chr01*	8107967	A/C	Gene_Ca_08057	NON_SYNONYMOUS_CODING	RNAhelicase,ATP-dependent,DEAD-box,conservedsite
Kabuli_wild_8108014	1399932417	*Ca_kabuli_Chr01*	8108014	C/T	Gene_Ca_08057	NON_SYNONYMOUS_CODING	RNAhelicase,ATP-dependent,DEAD-box,conservedsite
Kabuli_wild_8111588	1399932418	*Ca_kabuli_Chr01*	8111588	G/A	Gene_Ca_08057	INTRON	RNAhelicase,ATP-dependent,DEAD-box,conservedsite
Kabuli_wild_8112247	1399932419	*Ca_kabuli_Chr01*	8112247	A/G	Gene_Ca_08057	SYNONYMOUS_CODING	RNAhelicase,ATP-dependent,DEAD-box,conservedsite

SNP IDs	NCBI dbSNP accession IDs (NCBI_SS#)	Chromosomes	Physical positions (bp)	SNPs	Gene accession IDs	Sequence components of genome	Putative functions
Kabuli_wild_8112343	1399932420	*Ca_kabuli_Chr01*	8112343	C/T	Gene_Ca_08057	SYNONYMOUS_CODING	RNAhelicase,ATP-dependent,DEAD-box,conservedsite
Kabuli_wild_8112996	1399932421	*Ca_kabuli_Chr01*	8112996	T/G	Gene_Ca_08057	SYNONYMOUS_CODING	RNAhelicase,ATP-dependent,DEAD-box,conservedsite
Kabuli_wild_8113235	1399932422	*Ca_kabuli_Chr01*	8113235	G/T	Gene_Ca_08057	NON_SYNONYMOUS_CODING	RNAhelicase,ATP-dependent,DEAD-box,conservedsite
Kabuli_wild_8113398	1399932423	*Ca_kabuli_Chr01*	8113398	C/G	Gene_Ca_08057	NON_SYNONYMOUS_CODING	RNAhelicase,ATP-dependent,DEAD-box,conservedsite
Kabuli_wild_8113403	1399932424	*Ca_kabuli_Chr01*	8113403	A/T	Gene_Ca_08057	NON_SYNONYMOUS_CODING	RNAhelicase,ATP-dependent,DEAD-box,conservedsite
Kabuli_wild_8113449	1399932425	*Ca_kabuli_Chr01*	8113449	T/A	Gene_Ca_08057	SYNONYMOUS_CODING	RNAhelicase,ATP-dependent,DEAD-box,conservedsite
Kabuli_wild_8123033	1399932426	*Ca_kabuli_Chr01*	8123033	A/G	NA	INTERGENIC	NA
Kabuli_wild_8123257	1399932427	*Ca_kabuli_Chr01*	8123257	G/A	NA	INTERGENIC	NA
Kabuli_wild_8123481	1399932428	*Ca_kabuli_Chr01*	8123481	G/A	NA	INTERGENIC	NA
Kabuli_wild_8131096	1399932429	*Ca_kabuli_Chr01*	8131096	A/C	Gene_Ca_08059	SYNONYMOUS_CODING	Bicarbonatetransporter,eukaryotic
Kabuli_wild_8131157	1399932430	*Ca_kabuli_Chr01*	8131157	T/G	Gene_Ca_08059	NON_SYNONYMOUS_CODING	Bicarbonatetransporter,eukaryotic
Kabuli_wild_8134368	1399932431	*Ca_kabuli_Chr01*	8134368	T/C	NA	INTERGENIC	NA
Kabuli_wild_8192580	1399932432	*Ca_kabuli_Chr01*	8192580	T/C	Gene_Ca_08066	DOWNSTREAM	Ribosomal protein S1 ,RNA-binding domain
Kabuli_wild_8238639	1399932433	*Ca_kabuli_Chr01*	8238639	A/C	Gene_Ca_08068	SYNONYMOUS_CODING	HECT
Kabuli_wild_8278870	1399932434	*Ca_kabuli_Chr01*	8278870	T/G	NA	INTERGENIC	NA
Kabuli_wild_8422972	1399932435	*Ca_kabuli_Chr01*	8422972	G/A	NA	INTERGENIC	NA
Kabuli_wild_8464621	1399932436	*Ca_kabuli_Chr01*	8464621	C/G	Gene_Ca_08082	INTRON	NA
Kabuli_wild_8665705	1399932437	*Ca_kabuli_Chr01*	8665705	T/G	Gene_Ca_02944	INTRON	ATPase,P-type,K/Mg/Cd/Cu/Zn/Na/Ca/Na/H-transporter
Kabuli_wild_8677819	1399932438	*Ca_kabuli_Chr01*	8677819	C/A	Gene_Ca_02944	INTRON	ATPase,P-type,K/Mg/Cd/Cu/Zn/Na/Ca/Na/H-transporter
Kabuli_wild_8677877	1399932439	*Ca_kabuli_Chr01*	8677877	A/C	Gene_Ca_02944	INTRON	ATPase,P-type,K/Mg/Cd/Cu/Zn/Na/Ca/Na/H-transporter
Kabuli_wild_8677900	1399932440	*Ca_kabuli_Chr01*	8677900	T/C	Gene_Ca_02944	INTRON	ATPase,P-type,K/Mg/Cd/Cu/Zn/Na/Ca/Na/H-transporter
Kabuli_wild_8694455	1399932441	*Ca_kabuli_Chr01*	8694455	G/C	Gene_Ca_02942	INTRON	Sodium/calciumexchangermembraneregion
Kabuli_wild_8697177	1399932442	*Ca_kabuli_Chr01*	8697177	G/T	Gene_Ca_02942	INTRON	Sodium/calciumexchangermembraneregion
Kabuli_wild_8735727	1399932443	*Ca_kabuli_Chr01*	8735727	G/C	Gene_Ca_02937	INTRON	Ubiquitin-associated/translation elongation factor EF1B, N-terminal
Kabuli_wild_8735740	1399932444	*Ca_kabuli_Chr01*	8735740	G/T	Gene_Ca_02937	INTRON	Ubiquitin-associated/translation elongation factor EF1B, N-terminal
Kabuli_wild_8736512	1399932445	*Ca_kabuli_Chr01*	8736512	G/A	Gene_Ca_02937	INTRON	Ubiquitin-associated/translation elongation factor EF1B, N-terminal
Kabuli_wild_8736711	1399932446	*Ca_kabuli_Chr01*	8736711	G/T	Gene_Ca_02937	INTRON	Ubiquitin-associated/translation elongation factor EF1B, N-terminal
Kabuli_wild_8736733	1399932447	*Ca_kabuli_Chr01*	8736733	G/A	Gene_Ca_02937	INTRON	Ubiquitin-associated/translation elongation factor EF1B, N-terminal
Kabuli_wild_8737380	1399932448	*Ca_kabuli_Chr01*	8737380	A/G	Gene_Ca_02937	SYNONYMOUS_CODING	Ubiquitin-associated/translation elongation factor EF1B, N-terminal
Kabuli_wild_8767121	1399932449	*Ca_kabuli_Chr01*	8767121	A/T	Gene_Ca_02933	INTRON	Pentatrico peptide repeat
Kabuli_wild_8767122	1399932450	*Ca_kabuli_Chr01*	8767122	T/A	Gene_Ca_02933	INTRON	Pentatrico peptide repeat
Kabuli_wild_8798877	1399932451	*Ca_kabuli_Chr01*	8798877	G/T	NA	INTERGENIC	NA
Kabuli_wild_8799685	1399932452	*Ca_kabuli_Chr01*	8799685	T/G	Gene_Ca_02929	DOWNSTREAM	KinasebindingproteinCGI-121
Kabuli_wild_8799701	1399932453	*Ca_kabuli_Chr01*	8799701	T/C	Gene_Ca_02929	DOWNSTREAM	KinasebindingproteinCGI-121
Kabuli_wild_8799708	1399932454	*Ca_kabuli_Chr01*	8799708	T/C	Gene_Ca_02929	DOWNSTREAM	KinasebindingproteinCGI-121
Kabuli_wild_8809121	1399932455	*Ca_kabuli_Chr01*	8809121	T/C	Gene_Ca_02928	INTRON	ABC transporter,trans membrane domain
Kabuli_wild_8809188	1399932456	*Ca_kabuli_Chr01*	8809188	T/A	Gene_Ca_02928	INTRON	ABC transporter,trans membrane domain
Kabuli_wild_8809191	1399932457	*Ca_kabuli_Chr01*	8809191	C/T	Gene_Ca_02928	INTRON	ABC transporter,trans membrane domain
Kabuli_wild_8809295	1399932458	*Ca_kabuli_Chr01*	8809295	A/T	Gene_Ca_02928	INTRON	ABC transporter,trans membrane domain
Kabuli_wild_8809517	1399932459	*Ca_kabuli_Chr01*	8809517	C/G	Gene_Ca_02928	INTRON	ABC transporter,trans membrane domain
Kabuli_wild_8822366	1399932460	*Ca_kabuli_Chr01*	8822366	T/A	NA	INTERGENIC	NA
Kabuli_wild_8822375	1399932461	*Ca_kabuli_Chr01*	8822375	G/A	NA	INTERGENIC	NA
Kabuli_wild_8852863	1399932462	*Ca_kabuli_Chr01*	8852863	G/T	NA	INTERGENIC	NA
Kabuli_wild_8852928	1399932463	*Ca_kabuli_Chr01*	8852928	T/A	NA	INTERGENIC	NA

SNP IDs	NCBI dbSNP accession IDs (NCBI_SS#)	Chromosomes	Physical positions (bp)	SNPs	Gene accession IDs	Sequence components of genome	Putative functions
Kabuli_wild_8852943	1399932464	*Ca_kabuli_Chr01*	8852943	C/A	NA	INTERGENIC	NA
Kabuli_wild_8852981	1399932465	*Ca_kabuli_Chr01*	8852981	C/A	NA	INTERGENIC	NA
Kabuli_wild_8853010	1399932466	*Ca_kabuli_Chr01*	8853010	A/G	NA	INTERGENIC	NA
Kabuli_wild_8854211	1399932467	*Ca_kabuli_Chr01*	8854211	G/T	Gene_Ca_02923	INTRON	Natural resistance-associated macrophage protein
Kabuli_wild_8891438	1399932468	*Ca_kabuli_Chr01*	8891438	T/G	Gene_Ca_02918	SYNONYMOUS_CODING	ATPase, P-type, H+ transporting proton pump
Kabuli_wild_8891443	1399932469	*Ca_kabuli_Chr01*	8891443	C/G	Gene_Ca_02918	NON_SYNONYMOUS_CODING	ATPase, P-type, H+ transporting proton pump
Kabuli_wild_8891458	1399932470	*Ca_kabuli_Chr01*	8891458	T/G	Gene_Ca_02918	INTRON	ATPase, P-type, H+ transporting proton pump
Kabuli_wild_8891469	1399932471	*Ca_kabuli_Chr01*	8891469	T/A	Gene_Ca_02918	INTRON	ATPase, P-type, H+ transporting proton pump
Kabuli_wild_8891485	1399932472	*Ca_kabuli_Chr01*	8891485	T/G	Gene_Ca_02918	INTRON	ATPase, P-type, H+ transporting proton pump
Kabuli_wild_8891508	1399932473	*Ca_kabuli_Chr01*	8891508	G/T	Gene_Ca_02918	INTRON	ATPase, P-type, H+ transporting proton pump
Kabuli_wild_8891512	1399932474	*Ca_kabuli_Chr01*	8891512	C/T	Gene_Ca_02918	INTRON	ATPase, P-type, H+ transporting proton pump
Kabuli_wild_8893985	1399932475	*Ca_kabuli_Chr01*	8893985	G/A	NA	INTERGENIC	NA
Kabuli_wild_8901769	1399932476	*Ca_kabuli_Chr01*	8901769	C/A	NA	INTERGENIC	NA
Kabuli_wild_8901842	1399932477	*Ca_kabuli_Chr01*	8901842	T/A	NA	INTERGENIC	NA
Kabuli_wild_8941004	1399932478	*Ca_kabuli_Chr01*	8941004	A/C	NA	INTERGENIC	NA
Kabuli_wild_8941029	1399932479	*Ca_kabuli_Chr01*	8941029	G/A	NA	INTERGENIC	NA
Kabuli_wild_8941031	1399932480	*Ca_kabuli_Chr01*	8941031	G/C	NA	INTERGENIC	NA
Kabuli_wild_8941471	1399932481	*Ca_kabuli_Chr01*	8941471	T/C	NA	INTERGENIC	NA
Kabuli_wild_8942131	1399932482	*Ca_kabuli_Chr01*	8942131	T/C	Gene_Ca_02909	NON_SYNONYMOUS_CODING	Zinc finger,UBP-type
Kabuli_wild_8942137	1399932483	*Ca_kabuli_Chr01*	8942137	T/C	Gene_Ca_02909	NON_SYNONYMOUS_CODING	Zinc finger,UBP-type
Kabuli_wild_8942179	1399932484	*Ca_kabuli_Chr01*	8942179	C/G	Gene_Ca_02909	NON_SYNONYMOUS_CODING	Zinc finger,UBP-type
Kabuli_wild_8942198	1399932485	*Ca_kabuli_Chr01*	8942198	A/G	Gene_Ca_02909	SYNONYMOUS_CODING	Zinc finger,UBP-type
Kabuli_wild_8949723	1399932486	*Ca_kabuli_Chr01*	8949723	G/T	NA	INTERGENIC	NA
Kabuli_wild_8960630	1399932487	*Ca_kabuli_Chr01*	8960630	T/C	NA	INTERGENIC	NA
Kabuli_wild_8969321	1399932488	*Ca_kabuli_Chr01*	8969321	C/T	Gene_Ca_02904	INTRON	NA
Kabuli_wild_8971718	1399932489	*Ca_kabuli_Chr01*	8971718	C/G	Gene_Ca_02904	NON_SYNONYMOUS_CODING	NA
Kabuli_wild_8976169	1399932490	*Ca_kabuli_Chr01*	8976169	A/T	Gene_Ca_02904	NON_SYNONYMOUS_CODING	NA
Kabuli_wild_9008179	1399932491	*Ca_kabuli_Chr01*	9008179	G/T	Gene_Ca_02900	SYNONYMOUS_CODING	Aminoacid transporter,transmembrane
Kabuli_wild_9008180	1399932492	*Ca_kabuli_Chr01*	9008180	C/G	Gene_Ca_02900	SYNONYMOUS_CODING	Aminoacid transporter,transmembrane
Kabuli_wild_9031825	1399932493	*Ca_kabuli_Chr01*	9031825	G/T	Gene_Ca_02896	UPSTREAM	DNAglycosylase/APlyase,catalytic domain
Kabuli_wild_9035744	1399932494	*Ca_kabuli_Chr01*	9035744	G/A	NA	INTERGENIC	NA
Kabuli_wild_9040419	1399932495	*Ca_kabuli_Chr01*	9040419	T/A	NA	INTERGENIC	NA
Kabuli_wild_9048675	1399932496	*Ca_kabuli_Chr01*	9048675	A/T	NA	INTERGENIC	NA
Kabuli_wild_9063898	1399932497	*Ca_kabuli_Chr01*	9063898	G/T	Gene_Ca_02891	NON_SYNONYMOUS_CODING	Heat shock protein DnaJ, cysteine-richdomain
Kabuli_wild_9065933	1399932498	*Ca_kabuli_Chr01*	9065933	T/C	Gene_Ca_02891	UPSTREAM	Heat shock protein DnaJ, cysteine-richdomain
Kabuli_wild_9074283	1399932499	*Ca_kabuli_Chr01*	9074283	G/T	Gene_Ca_02889	SYNONYMOUS_CODING	Mov34/MPN/PAD-1
Kabuli_wild_9093812	1399932500	*Ca_kabuli_Chr01*	9093812	C/G	NA	INTERGENIC	NA
Kabuli_wild_9093964	1399932501	*Ca_kabuli_Chr01*	9093964	T/A	NA	INTERGENIC	NA
Kabuli_wild_9097869	1399932502	*Ca_kabuli_Chr01*	9097869	G/T	Gene_Ca_02886	UPSTREAM	Signal transduction histidine kinase,phosphotransfer (Hpt) domain
Kabuli_wild_9097877	1399932503	*Ca_kabuli_Chr01*	9097877	C/A	Gene_Ca_02886	UPSTREAM	Signal transduction histidine kinase,phosphotransfer (Hpt) domain
Kabuli_wild_9097878	1399932504	*Ca_kabuli_Chr01*	9097878	A/G	Gene_Ca_02886	UPSTREAM	Signal transduction histidine kinase,phosphotransfer (Hpt) domain
Kabuli_wild_9097879	1399932505	*Ca_kabuli_Chr01*	9097879	C/A	Gene_Ca_02886	UPSTREAM	Signal transduction histidine kinase,phosphotransfer (Hpt) domain
Kabuli_wild_9097881	1399932506	*Ca_kabuli_Chr01*	9097881	C/G	Gene_Ca_02886	UPSTREAM	Signal transduction histidine kinase,phosphotransfer (Hpt) domain
Kabuli_wild_9097885	1399932507	*Ca_kabuli_Chr01*	9097885	A/G	Gene_Ca_02886	UPSTREAM	Signal transduction histidine kinase,phosphotransfer (Hpt) domain

SNP IDs	NCBI dbSNP accession IDs (NCBI_SS#)	Chromosomes	Physical positions (bp)	SNPs	Gene accession IDs	Sequence components of genome	Putative functions
Kabuli_wild_9097949	1399932508	*Ca_kabuli_Chr01*	9097949	A/C	Gene_Ca_02886	UPSTREAM	Signal transduction histidine kinase,phosphotransfer (Hpt) domain
Kabuli_wild_9098003	1399932509	*Ca_kabuli_Chr01*	9098003	C/T	Gene_Ca_02886	UPSTREAM	Signal transduction histidine kinase,phosphotransfer (Hpt) domain
Kabuli_wild_9105432	1399932510	*Ca_kabuli_Chr01*	9105432	C/T	Gene_Ca_02885	SYNONYMOUS_CODING	Initiation factor2B-related
Kabuli_wild_9169979	1399932511	*Ca_kabuli_Chr01*	9169979	A/G	NA	INTERGENIC	NA
Kabuli_wild_9170005	1399932512	*Ca_kabuli_Chr01*	9170005	T/A	NA	INTERGENIC	NA
Kabuli_wild_9170035	1399932513	*Ca_kabuli_Chr01*	9170035	G/A	NA	INTERGENIC	NA
Kabuli_wild_9195312	1399932514	*Ca_kabuli_Chr01*	9195312	G/A	NA	INTERGENIC	NA
Kabuli_wild_9199591	1399932515	*Ca_kabuli_Chr01*	9199591	T/C	NA	INTERGENIC	NA
Kabuli_wild_9199610	1399932516	*Ca_kabuli_Chr01*	9199610	A/G	NA	INTERGENIC	NA
Kabuli_wild_9199616	1399932517	*Ca_kabuli_Chr01*	9199616	C/T	NA	INTERGENIC	NA
Kabuli_wild_9199619	1399932518	*Ca_kabuli_Chr01*	9199619	A/G	NA	INTERGENIC	NA
Kabuli_wild_9206737	1399932519	*Ca_kabuli_Chr01*	9206737	G/A	NA	INTERGENIC	NA
Kabuli_wild_9206740	1399932520	*Ca_kabuli_Chr01*	9206740	T/C	NA	INTERGENIC	NA
Kabuli_wild_9206742	1399932521	*Ca_kabuli_Chr01*	9206742	C/G	NA	INTERGENIC	NA
Kabuli_wild_9237766	1399932522	*Ca_kabuli_Chr01*	9237766	T/G	NA	INTERGENIC	NA
Kabuli_wild_9237805	1399932523	*Ca_kabuli_Chr01*	9237805	T/C	NA	INTERGENIC	NA
Kabuli_wild_9237813	1399932524	*Ca_kabuli_Chr01*	9237813	G/T	NA	INTERGENIC	NA
Kabuli_wild_9264758	1399932525	*Ca_kabuli_Chr01*	9264758	G/T	Gene_Ca_02867	INTRON	Nucleoporininter acting component Nup93/Nic96
Kabuli_wild_9264794	1399932526	*Ca_kabuli_Chr01*	9264794	T/G	Gene_Ca_02867	INTRON	Nucleoporininter acting component Nup93/Nic96
Kabuli_wild_9264912	1399932527	*Ca_kabuli_Chr01*	9264912	C/T	Gene_Ca_02867	SYNONYMOUS_CODING	Nucleoporininter acting component Nup93/Nic96
Kabuli_wild_9269888	1399932528	*Ca_kabuli_Chr01*	9269888	T/A	Gene_Ca_02867	NON_SYNONYMOUS_CODING	Nucleoporininter acting component Nup93/Nic96
Kabuli_wild_9379381	1399932529	*Ca_kabuli_Chr01*	9379381	G/A	NA	INTERGENIC	NA
Kabuli_wild_9392590	1399932530	*Ca_kabuli_Chr01*	9392590	G/C	NA	INTERGENIC	NA
Kabuli_wild_9433717	1399932531	*Ca_kabuli_Chr01*	9433717	A/G	Gene_Ca_02853	INTRON	Ferredoxin
Kabuli_wild_9434678	1399932532	*Ca_kabuli_Chr01*	9434678	A/C	Gene_Ca_02853	UPSTREAM	Ferredoxin
Kabuli_wild_9544561	1399932533	*Ca_kabuli_Chr01*	9544561	A/G	NA	INTERGENIC	NA
Kabuli_wild_9603896	1399932534	*Ca_kabuli_Chr01*	9603896	T/A	Gene_Ca_02837	SYNONYMOUS_CODING	WD40 repeat
Kabuli_wild_9634823	1399932535	*Ca_kabuli_Chr01*	9634823	C/T	Gene_Ca_02835	SYNONYMOUS_CODING	PeptidaseC14,caspasenon-catalyticsubunitp10
Kabuli_wild_9659316	1399932536	*Ca_kabuli_Chr01*	9659316	C/T	NA	INTERGENIC	NA
Kabuli_wild_9705162	1399932537	*Ca_kabuli_Chr01*	9705162	C/A	NA	INTERGENIC	NA
Kabuli_wild_9736498	1399932538	*Ca_kabuli_Chr01*	9736498	A/C	NA	INTERGENIC	NA
Kabuli_wild_9886208	1399932539	*Ca_kabuli_Chr01*	9886208	A/G	Gene_Ca_02799	SYNONYMOUS_CODING	NA
Kabuli_wild_9918080	1399932540	*Ca_kabuli_Chr01*	9918080	A/G	Gene_Ca_02796	SYNONYMOUS_CODING	RibonucleaseZc3h12a-like
Kabuli_wild_10022317	1399932541	*Ca_kabuli_Chr01*	10022317	A/T	Gene_Ca_02786	NON_SYNONYMOUS_CODING	SANT domain,DNA binding
Kabuli_wild_10032933	1399932542	*Ca_kabuli_Chr01*	10032933	A/T	NA	INTERGENIC	NA
Kabuli_wild_10038569	1399932543	*Ca_kabuli_Chr01*	10038569	A/G	NA	INTERGENIC	NA
Kabuli_wild_10191578	1399932544	*Ca_kabuli_Chr01*	10191578	A/C	NA	INTERGENIC	NA
Kabuli_wild_10213111	1399932545	*Ca_kabuli_Chr01*	10213111	T/C	NA	INTERGENIC	NA
Kabuli_wild_10364986	1399932546	*Ca_kabuli_Chr01*	10364986	C/G	Gene_Ca_02742	DOWNSTREAM	Protein kinase,catalytic domain
Kabuli_wild_10364999	1399932547	*Ca_kabuli_Chr01*	10364999	G/A	Gene_Ca_02742	DOWNSTREAM	Protein kinase,catalytic domain
Kabuli_wild_10365024	1399932548	*Ca_kabuli_Chr01*	10365024	G/A	Gene_Ca_02742	DOWNSTREAM	Protein kinase,catalytic domain
Kabuli_wild_10365029	1399932549	*Ca_kabuli_Chr01*	10365029	T/A	Gene_Ca_02742	DOWNSTREAM	Protein kinase,catalytic domain
Kabuli_wild_10366420	1399932550	*Ca_kabuli_Chr01*	10366420	A/C	Gene_Ca_02742	DOWNSTREAM	Protein kinase,catalytic domain
Kabuli_wild_10377691	1399932551	*Ca_kabuli_Chr01*	10377691	T/C	Gene_Ca_02740	INTRON	Glycosidehydrolase,family35

SNP IDs	NCBI dbSNP accession IDs (NCBI_SS#)	Chromosomes	Physical positions (bp)	SNPs	Gene accession IDs	Sequence components of genome	Putative functions
Kabuli_wild_10377698	1399932552	*Ca_kabuli_Chr01*	10377698	T/C	Gene_Ca_02740	INTRON	Glycosidehydrolase,family35
Kabuli_wild_10377700	1399932553	*Ca_kabuli_Chr01*	10377700	G/C	Gene_Ca_02740	INTRON	Glycosidehydrolase,family35
Kabuli_wild_10377860	1399932554	*Ca_kabuli_Chr01*	10377860	T/A	Gene_Ca_02740	INTRON	Glycosidehydrolase,family35
Kabuli_wild_10527733	1399932555	*Ca_kabuli_Chr01*	10527733	A/G	NA	INTERGENIC	NA
Kabuli_wild_10601318	1399932556	*Ca_kabuli_Chr01*	10601318	T/G	Gene_Ca_02710	NON_SYNONYMOUS_CODING	Pentatrico peptide repeat
Kabuli_wild_10623359	1399932557	*Ca_kabuli_Chr01*	10623359	T/G	Gene_Ca_02705	UPSTREAM	Rab-GAP/TBCdomain
Kabuli_wild_10655633	1399932558	*Ca_kabuli_Chr01*	10655633	G/T	Gene_Ca_02700	INTRON	Protein of unknown function DUF3755
Kabuli_wild_10672248	1399932559	*Ca_kabuli_Chr01*	10672248	A/C	NA	INTERGENIC	NA
Kabuli_wild_10697955	1399932560	*Ca_kabuli_Chr01*	10697955	T/C	Gene_Ca_02694	INTRON	Protein of unknown function DUF936,plant
Kabuli_wild_10710672	1399932561	*Ca_kabuli_Chr01*	10710672	T/G	NA	INTERGENIC	NA
Kabuli_wild_10713001	1399932562	*Ca_kabuli_Chr01*	10713001	T/A	Gene_Ca_02691	DOWNSTREAM	Electron transfer flavoprotein-ubiquinoneoxido reductase
Kabuli_wild_10715185	1399932563	*Ca_kabuli_Chr01*	10715185	G/A	Gene_Ca_02691	INTRON	Electron transfer flavoprotein-ubiquinoneoxido reductase
Kabuli_wild_10761609	1399932564	*Ca_kabuli_Chr01*	10761609	G/T	NA	INTERGENIC	NA
Kabuli_wild_10837203	1399932565	*Ca_kabuli_Chr01*	10837203	C/T	NA	INTERGENIC	NA
Kabuli_wild_10861326	1399932566	*Ca_kabuli_Chr01*	10861326	T/G	Gene_Ca_02673	INTRON	Glycosidehydrolase,family1
Kabuli_wild_10861332	1399932567	*Ca_kabuli_Chr01*	10861332	C/G	Gene_Ca_02673	INTRON	Glycosidehydrolase,family1
Kabuli_wild_10869336	1399932568	*Ca_kabuli_Chr01*	10869336	G/A	Gene_Ca_02671	INTRON	Oxidoreductase,N-terminal
Kabuli_wild_10869361	1399932569	*Ca_kabuli_Chr01*	10869361	C/A	Gene_Ca_02671	INTRON	Oxidoreductase,N-terminal
Kabuli_wild_10896308	1399932570	*Ca_kabuli_Chr01*	10896308	A/G	Gene_Ca_02669	NON_SYNONYMOUS_CODING	Ethylenein sensitive3
Kabuli_wild_11027385	1399932571	*Ca_kabuli_Chr01*	11027385	G/A	NA	INTERGENIC	NA
Kabuli_wild_11103176	1399932572	*Ca_kabuli_Chr01*	11103176	T/C	NA	INTERGENIC	NA
Kabuli_wild_11185782	1399932573	*Ca_kabuli_Chr01*	11185782	T/A	NA	INTERGENIC	NA
Kabuli_wild_11193242	1399932574	*Ca_kabuli_Chr01*	11193242	G/A	NA	INTERGENIC	NA
Kabuli_wild_11193246	1399932575	*Ca_kabuli_Chr01*	11193246	A/C	NA	INTERGENIC	NA
Kabuli_wild_11193277	1399932576	*Ca_kabuli_Chr01*	11193277	G/C	NA	INTERGENIC	NA
Kabuli_wild_11193282	1399932577	*Ca_kabuli_Chr01*	11193282	C/T	NA	INTERGENIC	NA
Kabuli_wild_11193334	1399932578	*Ca_kabuli_Chr01*	11193334	G/C	NA	INTERGENIC	NA
Kabuli_wild_11225828	1399932579	*Ca_kabuli_Chr01*	11225828	T/C	NA	INTERGENIC	NA
Kabuli_wild_11227613	1399932580	*Ca_kabuli_Chr01*	11227613	G/A	NA	INTERGENIC	NA
Kabuli_wild_11227614	1399932581	*Ca_kabuli_Chr01*	11227614	T/C	NA	INTERGENIC	NA
Kabuli_wild_11230109	1399932582	*Ca_kabuli_Chr01*	11230109	T/C	Gene_Ca_02632	NON_SYNONYMOUS_CODING	Pumilio RNA-binding repeat
Kabuli_wild_11230115	1399932583	*Ca_kabuli_Chr01*	11230115	G/C	Gene_Ca_02632	NON_SYNONYMOUS_CODING	Pumilio RNA-binding repeat
Kabuli_wild_11230116	1399932584	*Ca_kabuli_Chr01*	11230116	T/C	Gene_Ca_02632	SYNONYMOUS_CODING	Pumilio RNA-binding repeat
Kabuli_wild_11230118	1399932585	*Ca_kabuli_Chr01*	11230118	A/T	Gene_Ca_02632	NON_SYNONYMOUS_CODING	Pumilio RNA-binding repeat
Kabuli_wild_11230125	1399932586	*Ca_kabuli_Chr01*	11230125	A/C	Gene_Ca_02632	SYNONYMOUS_CODING	Pumilio RNA-binding repeat
Kabuli_wild_11230126	1399932587	*Ca_kabuli_Chr01*	11230126	G/T	Gene_Ca_02632	NON_SYNONYMOUS_CODING	Pumilio RNA-binding repeat
Kabuli_wild_11230127	1399932588	*Ca_kabuli_Chr01*	11230127	G/T	Gene_Ca_02632	NON_SYNONYMOUS_CODING	Pumilio RNA-binding repeat
Kabuli_wild_11230128	1399932589	*Ca_kabuli_Chr01*	11230128	T/G	Gene_Ca_02632	SYNONYMOUS_CODING	Pumilio RNA-binding repeat
Kabuli_wild_11230133	1399932590	*Ca_kabuli_Chr01*	11230133	G/A	Gene_Ca_02632	NON_SYNONYMOUS_CODING	Pumilio RNA-binding repeat
Kabuli_wild_11230998	1399932591	*Ca_kabuli_Chr01*	11230998	A/T	Gene_Ca_02632	SYNONYMOUS_CODING	Pumilio RNA-binding repeat
Kabuli_wild_11231064	1399932592	*Ca_kabuli_Chr01*	11231064	A/G	Gene_Ca_02632	SYNONYMOUS_CODING	Pumilio RNA-binding repeat
Kabuli_wild_11231077	1399932593	*Ca_kabuli_Chr01*	11231077	T/C	Gene_Ca_02632	NON_SYNONYMOUS_CODING	Pumilio RNA-binding repeat
Kabuli_wild_11232152	1399932594	*Ca_kabuli_Chr01*	11232152	T/C	Gene_Ca_02632	SYNONYMOUS_CODING	Pumilio RNA-binding repeat
Kabuli_wild_11235211	1399932595	*Ca_kabuli_Chr01*	11235211	C/T	NA	INTERGENIC	NA

SNP IDs	NCBI dbSNP accession IDs (NCBI_SS#)	Chromosomes	Physical positions (bp)	SNPs	Gene accession IDs	Sequence components of genome	Putative functions
Kabuli_wild_11246551	1399932596	*Ca_kabuli_Chr01*	11246551	T/C	NA	INTERGENIC	NA
Kabuli_wild_11265367	1399932597	*Ca_kabuli_Chr01*	11265367	T/C	NA	INTERGENIC	NA
Kabuli_wild_11277703	1399932598	*Ca_kabuli_Chr01*	11277703	T/A	NA	INTERGENIC	NA
Kabuli_wild_11277964	1399932599	*Ca_kabuli_Chr01*	11277964	C/T	NA	INTERGENIC	NA
Kabuli_wild_11284183	1399932600	*Ca_kabuli_Chr01*	11284183	T/C	NA	INTERGENIC	NA
Kabuli_wild_11285192	1399932601	*Ca_kabuli_Chr01*	11285192	G/A	Gene_Ca_02625	SYNONYMOUS_CODING	Transcription facto rGRAS
Kabuli_wild_11285247	1399932602	*Ca_kabuli_Chr01*	11285247	T/C	Gene_Ca_02625	SYNONYMOUS_CODING	Transcription facto rGRAS
Kabuli_wild_11291516	1399932603	*Ca_kabuli_Chr01*	11291516	G/A	NA	INTERGENIC	NA
Kabuli_wild_11291737	1399932604	*Ca_kabuli_Chr01*	11291737	C/A	Gene_Ca_02624	INTRON	SANT domain,DNA binding
Kabuli_wild_11291740	1399932605	*Ca_kabuli_Chr01*	11291740	T/A	Gene_Ca_02624	INTRON	SANT domain,DNA binding
Kabuli_wild_11291741	1399932606	*Ca_kabuli_Chr01*	11291741	A/C	Gene_Ca_02624	INTRON	SANT domain,DNA binding
Kabuli_wild_11293696	1399932607	*Ca_kabuli_Chr01*	11293696	A/G	NA	INTERGENIC	NA
Kabuli_wild_11319112	1399932608	*Ca_kabuli_Chr01*	11319112	A/T	NA	INTERGENIC	NA
Kabuli_wild_11319264	1399932609	*Ca_kabuli_Chr01*	11319264	A/C	NA	INTERGENIC	NA
Kabuli_wild_11321670	1399932610	*Ca_kabuli_Chr01*	11321670	G/C	Gene_Ca_02623	DOWNSTREAM	Rab5-interacting
Kabuli_wild_11324018	1399932611	*Ca_kabuli_Chr01*	11324018	C/A	NA	INTERGENIC	NA
Kabuli_wild_11324023	1399932612	*Ca_kabuli_Chr01*	11324023	C/T	NA	INTERGENIC	NA
Kabuli_wild_11324035	1399932613	*Ca_kabuli_Chr01*	11324035	C/T	NA	INTERGENIC	NA
Kabuli_wild_11324050	1399932614	*Ca_kabuli_Chr01*	11324050	G/T	NA	INTERGENIC	NA
Kabuli_wild_11328770	1399932615	*Ca_kabuli_Chr01*	11328770	T/C	NA	INTERGENIC	NA
Kabuli_wild_11328785	1399932616	*Ca_kabuli_Chr01*	11328785	A/C	NA	INTERGENIC	NA
Kabuli_wild_11328920	1399932617	*Ca_kabuli_Chr01*	11328920	A/T	NA	INTERGENIC	NA
Kabuli_wild_11342388	1399932618	*Ca_kabuli_Chr01*	11342388	T/C	NA	INTERGENIC	NA
Kabuli_wild_11359718	1399932619	*Ca_kabuli_Chr01*	11359718	G/A	NA	INTERGENIC	NA
Kabuli_wild_11361583	1399932620	*Ca_kabuli_Chr01*	11361583	T/C	Gene_Ca_02617	SYNONYMOUS_CODING	Protein kinase,catalytic domain
Kabuli_wild_11361588	1399932621	*Ca_kabuli_Chr01*	11361588	T/G	Gene_Ca_02617	NON_SYNONYMOUS_CODING	Protein kinase,catalytic domain
Kabuli_wild_11405015	1399932622	*Ca_kabuli_Chr01*	11405015	A/G	Gene_Ca_02611	NON_SYNONYMOUS_CODING	Protein kinase,catalytic domain
Kabuli_wild_11411702	1399932623	*Ca_kabuli_Chr01*	11411702	C/G	NA	INTERGENIC	NA
Kabuli_wild_11423778	1399932624	*Ca_kabuli_Chr01*	11423778	A/C	Gene_Ca_02609	NON_SYNONYMOUS_CODING	Zinc finger, RING-type
Kabuli_wild_11502148	1399932625	*Ca_kabuli_Chr01*	11502148	C/A	NA	INTERGENIC	NA
Kabuli_wild_11514888	1399932626	*Ca_kabuli_Chr01*	11514888	T/A	Gene_Ca_02599	INTRON	HistoneH2A
Kabuli_wild_11522875	1399932627	*Ca_kabuli_Chr01*	11522875	C/T	Gene_Ca_02598	SYNONYMOUS_CODING	HistoneH2A
Kabuli_wild_11592120	1399932628	*Ca_kabuli_Chr01*	11592120	A/T	NA	INTERGENIC	NA
Kabuli_wild_11592128	1399932629	*Ca_kabuli_Chr01*	11592128	A/T	NA	INTERGENIC	NA
Kabuli_wild_11601347	1399932630	*Ca_kabuli_Chr01*	11601347	T/C	Gene_Ca_02586	INTRON	RNA recognition motif domain
Kabuli_wild_11617567	1399932631	*Ca_kabuli_Chr01*	11617567	C/T	NA	INTERGENIC	NA
Kabuli_wild_11624419	1399932632	*Ca_kabuli_Chr01*	11624419	C/G	NA	INTERGENIC	NA
Kabuli_wild_11624464	1399932633	*Ca_kabuli_Chr01*	11624464	C/T	NA	INTERGENIC	NA
Kabuli_wild_11669794	1399932634	*Ca_kabuli_Chr01*	11669794	T/G	Gene_Ca_02583	SYNONYMOUS_CODING	Ankyrinrepeat
Kabuli_wild_11673478	1399932635	*Ca_kabuli_Chr01*	11673478	A/C	NA	INTERGENIC	NA
Kabuli_wild_11673667	1399932636	*Ca_kabuli_Chr01*	11673667	T/G	NA	INTERGENIC	NA
Kabuli_wild_11673673	1399932637	*Ca_kabuli_Chr01*	11673673	T/A	NA	INTERGENIC	NA
Kabuli_wild_11677097	1399932638	*Ca_kabuli_Chr01*	11677097	C/A	NA	INTERGENIC	NA
Kabuli_wild_11685769	1399932639	*Ca_kabuli_Chr01*	11685769	A/G	Gene_Ca_02582	SYNONYMOUS_CODING	Protein kinase,catalytic domain

SNP IDs	NCBI dbSNP accession IDs (NCBI_SS#)	Chromosomes	Physical positions (bp)	SNPs	Gene accession IDs	Sequence components of genome	Putative functions
Kabuli_wild_11685790	1399932640	*Ca_kabuli_Chr01*	11685790	T/C	Gene_Ca_02582	SYNONYMOUS_CODING	Protein kinase,catalytic domain
Kabuli_wild_11685865	1399932641	*Ca_kabuli_Chr01*	11685865	T/C	Gene_Ca_02582	SYNONYMOUS_CODING	Protein kinase,catalytic domain
Kabuli_wild_11793443	1399932642	*Ca_kabuli_Chr01*	11793443	C/A	Gene_Ca_02573	NON_SYNONYMOUS_CODING	Alcohol dehydrogenase superfamily, zinc-containing
Kabuli_wild_11822305	1399932643	*Ca_kabuli_Chr01*	11822305	G/T	Gene_Ca_02567	DOWNSTREAM	Glycosidehydrolase,family29
Kabuli_wild_11822309	1399932644	*Ca_kabuli_Chr01*	11822309	C/A	Gene_Ca_02567	DOWNSTREAM	Glycosidehydrolase,family29
Kabuli_wild_11898340	1399932645	*Ca_kabuli_Chr01*	11898340	T/C	Gene_Ca_02562	INTRON	NA
Kabuli_wild_12017225	1399932646	*Ca_kabuli_Chr01*	12017225	T/C	Gene_Ca_02553	INTRON	SNF2-related
Kabuli_wild_12169598	1399932647	*Ca_kabuli_Chr01*	12169598	G/A	Gene_Ca_02539	INTRON	Homeobox
Kabuli_wild_12189822	1399932648	*Ca_kabuli_Chr01*	12189822	T/G	Gene_Ca_02538	SYNONYMOUS_CODING	Double-strandedRNA-binding
Kabuli_wild_12198858	1399932649	*Ca_kabuli_Chr01*	12198858	C/A	Gene_Ca_02537	NON_SYNONYMOUS_CODING	Vacuola rfusion protein MON1
Kabuli_wild_12326230	1399932650	*Ca_kabuli_Chr01*	12326230	A/G	NA	INTERGENIC	NA
Kabuli_wild_12337445	1399932651	*Ca_kabuli_Chr01*	12337445	C/T	Gene_Ca_02516	SYNONYMOUS_CODING	AUX/IAAprotein
Kabuli_wild_12391141	1399932652	*Ca_kabuli_Chr01*	12391141	C/A	Gene_Ca_02511	UPSTREAM	NA
Kabuli_wild_12413012	1399932653	*Ca_kabuli_Chr01*	12413012	G/T	Gene_Ca_02509	NON_SYNONYMOUS_CODING	NA
Kabuli_wild_12413016	1399932654	*Ca_kabuli_Chr01*	12413016	T/A	Gene_Ca_02509	NON_SYNONYMOUS_CODING	NA
Kabuli_wild_12413025	1399932655	*Ca_kabuli_Chr01*	12413025	G/C	Gene_Ca_02509	NON_SYNONYMOUS_CODING	NA
Kabuli_wild_12465927	1399932656	*Ca_kabuli_Chr01*	12465927	T/A	NA	INTERGENIC	NA
Kabuli_wild_12465978	1399932657	*Ca_kabuli_Chr01*	12465978	C/T	NA	INTERGENIC	NA
Kabuli_wild_12588525	1399932658	*Ca_kabuli_Chr01*	12588525	A/G	Gene_Ca_02496	INTRON	NA
Kabuli_wild_12674088	1399932659	*Ca_kabuli_Chr01*	12674088	G/T	Gene_Ca_02486	INTRON	Haloaciddehydrogenase/epoxidehydrolase
Kabuli_wild_12688435	1399932660	*Ca_kabuli_Chr01*	12688435	A/C	NA	INTERGENIC	NA
Kabuli_wild_12736353	1399932661	*Ca_kabuli_Chr01*	12736353	A/C	NA	INTERGENIC	NA
Kabuli_wild_12736354	1399932662	*Ca_kabuli_Chr01*	12736354	G/C	NA	INTERGENIC	NA
Kabuli_wild_12800597	1399932663	*Ca_kabuli_Chr01*	12800597	G/T	NA	INTERGENIC	NA
Kabuli_wild_12800601	1399932664	*Ca_kabuli_Chr01*	12800601	T/A	NA	INTERGENIC	NA
Kabuli_wild_12800607	1399932665	*Ca_kabuli_Chr01*	12800607	T/A	NA	INTERGENIC	NA
Kabuli_wild_12800610	1399932666	*Ca_kabuli_Chr01*	12800610	A/C	NA	INTERGENIC	NA
Kabuli_wild_12845445	1399932667	*Ca_kabuli_Chr01*	12845445	A/C	NA	INTERGENIC	NA
Kabuli_wild_12880408	1399932668	*Ca_kabuli_Chr01*	12880408	G/A	Gene_Ca_02457	DOWNSTREAM	No apical meristem (NAM) protein
Kabuli_wild_12912656	1399932669	*Ca_kabuli_Chr01*	12912656	C/A	NA	INTERGENIC	NA
Kabuli_wild_12947367	1399932670	*Ca_kabuli_Chr01*	12947367	T/C	NA	INTERGENIC	NA
Kabuli_wild_12947378	1399932671	*Ca_kabuli_Chr01*	12947378	G/A	NA	INTERGENIC	NA
Kabuli_wild_12947395	1399932672	*Ca_kabuli_Chr01*	12947395	C/T	NA	INTERGENIC	NA
Kabuli_wild_12948174	1399932673	*Ca_kabuli_Chr01*	12948174	A/G	NA	INTERGENIC	NA
Kabuli_wild_12948219	1399932674	*Ca_kabuli_Chr01*	12948219	G/A	NA	INTERGENIC	NA
Kabuli_wild_12948257	1399932675	*Ca_kabuli_Chr01*	12948257	A/T	NA	INTERGENIC	NA
Kabuli_wild_12992423	1399932676	*Ca_kabuli_Chr01*	12992423	A/C	NA	INTERGENIC	NA
Kabuli_wild_12992445	1399932677	*Ca_kabuli_Chr01*	12992445	A/C	NA	INTERGENIC	NA
Kabuli_wild_12992569	1399932678	*Ca_kabuli_Chr01*	12992569	A/G	NA	INTERGENIC	NA
Kabuli_wild_13034707	1399932679	*Ca_kabuli_Chr01*	13034707	C/A	NA	INTERGENIC	NA
Kabuli_wild_13034708	1399932680	*Ca_kabuli_Chr01*	13034708	T/G	NA	INTERGENIC	NA
Kabuli_wild_13040645	1399932681	*Ca_kabuli_Chr01*	13040645	T/G	Gene_Ca_14068	INTRON	ATPase,P-type,K/Mg/Cd/Cu/Zn/Na/Ca/Na/H-transporter
Kabuli_wild_13076695	1399932682	*Ca_kabuli_Chr01*	13076695	G/A	Gene_Ca_14071	NON_SYNONYMOUS_CODING	Zinc finger, RING-type
Kabuli_wild_13156427	1399932683	*Ca_kabuli_Chr01*	13156427	A/C	NA	INTERGENIC	NA

SNP IDs	NCBI dbSNP accession IDs (NCBI_SS#)	Chromosomes	Physical positions (bp)	SNPs	Gene accession IDs	Sequence components of genome	Putative functions
Kabuli_wild_13156467	1399932684	*Ca_kabuli_Chr01*	13156467	A/C	NA	INTERGENIC	NA
Kabuli_wild_13156473	1399932685	*Ca_kabuli_Chr01*	13156473	A/C	NA	INTERGENIC	NA
Kabuli_wild_13158100	1399932686	*Ca_kabuli_Chr01*	13158100	A/G	NA	INTERGENIC	NA
Kabuli_wild_13158110	1399932687	*Ca_kabuli_Chr01*	13158110	G/C	NA	INTERGENIC	NA
Kabuli_wild_13158139	1399932688	*Ca_kabuli_Chr01*	13158139	G/A	NA	INTERGENIC	NA
Kabuli_wild_13158142	1399932689	*Ca_kabuli_Chr01*	13158142	C/T	NA	INTERGENIC	NA
Kabuli_wild_13158171	1399932690	*Ca_kabuli_Chr01*	13158171	T/A	NA	INTERGENIC	NA
Kabuli_wild_13158192	1399932691	*Ca_kabuli_Chr01*	13158192	T/C	NA	INTERGENIC	NA
Kabuli_wild_13158201	1399932692	*Ca_kabuli_Chr01*	13158201	C/T	NA	INTERGENIC	NA
Kabuli_wild_13158244	1399932693	*Ca_kabuli_Chr01*	13158244	C/T	NA	INTERGENIC	NA
Kabuli_wild_13369666	1399932694	*Ca_kabuli_Chr01*	13369666	T/C	Gene_Ca_14101	INTRON	Protein kinase,catalytic domain
Kabuli_wild_13370020	1399932695	*Ca_kabuli_Chr01*	13370020	C/A	Gene_Ca_14101	NON_SYNONYMOUS_CODING	Protein kinase,catalytic domain
Kabuli_wild_13370021	1399932696	*Ca_kabuli_Chr01*	13370021	A/G	Gene_Ca_14101	SYNONYMOUS_CODING	Protein kinase,catalytic domain
Kabuli_wild_13406001	1399932697	*Ca_kabuli_Chr01*	13406001	A/C	Gene_Ca_14104	UPSTREAM	Formiminotransferase,N-terminalsubdomain
Kabuli_wild_13497488	1399932698	*Ca_kabuli_Chr01*	13497488	G/A	Gene_Ca_14116	INTRON	PhotosystemIIPsp29,biogenesis
Kabuli_wild_13497572	1399932699	*Ca_kabuli_Chr01*	13497572	G/T	Gene_Ca_14116	INTRON	PhotosystemIIPsp29,biogenesis
Kabuli_wild_13549635	1399932700	*Ca_kabuli_Chr01*	13549635	T/C	Gene_Ca_14121	SYNONYMOUS_CODING	Kinesin,motordomain
Kabuli_wild_13550600	1399932701	*Ca_kabuli_Chr01*	13550600	G/A	Gene_Ca_14121	INTRON	Kinesin,motordomain
Kabuli_wild_13560364	1399932702	*Ca_kabuli_Chr01*	13560364	T/G	Gene_Ca_14122	UPSTREAM	Delayed-early response protein/equilibrative nucleoside transporter
Kabuli_wild_13590903	1399932703	*Ca_kabuli_Chr01*	13590903	G/T	Gene_Ca_14125	SYNONYMOUS_CODING	Protein kinase,catalytic domain
Kabuli_wild_13591177	1399932704	*Ca_kabuli_Chr01*	13591177	G/A	Gene_Ca_14125	NON_SYNONYMOUS_CODING	Protein kinase,catalytic domain
Kabuli_wild_13591203	1399932705	*Ca_kabuli_Chr01*	13591203	C/G	Gene_Ca_14125	NON_SYNONYMOUS_CODING	Protein kinase,catalytic domain
Kabuli_wild_13596411	1399932706	*Ca_kabuli_Chr01*	13596411	G/T	NA	INTERGENIC	NA
Kabuli_wild_13596528	1399932707	*Ca_kabuli_Chr01*	13596528	T/A	NA	INTERGENIC	NA
Kabuli_wild_13596539	1399932708	*Ca_kabuli_Chr01*	13596539	A/G	NA	INTERGENIC	NA
Kabuli_wild_13596590	1399932709	*Ca_kabuli_Chr01*	13596590	T/C	NA	INTERGENIC	NA
Kabuli_wild_13596593	1399932710	*Ca_kabuli_Chr01*	13596593	A/G	NA	INTERGENIC	NA
Kabuli_wild_13596621	1399932711	*Ca_kabuli_Chr01*	13596621	C/T	NA	INTERGENIC	NA
Kabuli_wild_13596631	1399932712	*Ca_kabuli_Chr01*	13596631	C/T	NA	INTERGENIC	NA
Kabuli_wild_13596659	1399932713	*Ca_kabuli_Chr01*	13596659	T/A	NA	INTERGENIC	NA
Kabuli_wild_13607141	1399932714	*Ca_kabuli_Chr01*	13607141	G/A	Gene_Ca_14127	SYNONYMOUS_CODING	Protein kinase,catalytic domain
Kabuli_wild_13607486	1399932715	*Ca_kabuli_Chr01*	13607486	A/C	Gene_Ca_14127	SYNONYMOUS_CODING	Protein kinase,catalytic domain
Kabuli_wild_13641293	1399932716	*Ca_kabuli_Chr01*	13641293	T/A	Gene_Ca_14129	INTRON	CCAAT-binding factor
Kabuli_wild_13668759	1399932717	*Ca_kabuli_Chr01*	13668759	T/A	Gene_Ca_14130	DOWNSTREAM	Calcium-bindingEF-hand
Kabuli_wild_13680008	1399932718	*Ca_kabuli_Chr01*	13680008	G/C	NA	INTERGENIC	NA
Kabuli_wild_13757012	1399932719	*Ca_kabuli_Chr01*	13757012	C/T	Gene_Ca_14138	SYNONYMOUS_CODING	SET domain
Kabuli_wild_13757052	1399932720	*Ca_kabuli_Chr01*	13757052	A/C	Gene_Ca_14138	SYNONYMOUS_CODING	SET domain
Kabuli_wild_13762410	1399932721	*Ca_kabuli_Chr01*	13762410	C/A	Gene_Ca_14138	NON_SYNONYMOUS_CODING	SET domain
Kabuli_wild_13769771	1399932722	*Ca_kabuli_Chr01*	13769771	T/C	NA	INTERGENIC	NA
Kabuli_wild_13769805	1399932723	*Ca_kabuli_Chr01*	13769805	C/T	NA	INTERGENIC	NA
Kabuli_wild_13782049	1399932724	*Ca_kabuli_Chr01*	13782049	C/T	Gene_Ca_14141	SYNONYMOUS_CODING	Glycosidehydrolase,family14B,plant
Kabuli_wild_13828744	1399932725	*Ca_kabuli_Chr01*	13828744	A/T	Gene_Ca_14148	INTRON	CCTdomain
Kabuli_wild_14015757	1399932726	*Ca_kabuli_Chr01*	14015757	T/C	Gene_Ca_07128	NON_SYNONYMOUS_CODING	Ionotropicglutamate receptor
Kabuli_wild_14015865	1399932727	*Ca_kabuli_Chr01*	14015865	A/C	Gene_Ca_07128	NON_SYNONYMOUS_CODING	Ionotropicglutamate receptor

SNP IDs	NCBI dbSNP accession IDs (NCBI_SS#)	Chromosomes	Physical positions (bp)	SNPs	Gene accession IDs	Sequence components of genome	Putative functions
Kabuli_wild_14047190	1399932728	*Ca_kabuli_Chr01*	14047190	A/G	Gene_Ca_07124	NON_SYNONYMOUS_CODING	AconitaseA/isopropylmalatedehydratasesmallsubunit,swivel
Kabuli_wild_14101640	1399932729	*Ca_kabuli_Chr01*	14101640	T/C	Gene_Ca_07122	INTRON	Glycosyltransferase,family31
Kabuli_wild_14163718	1399932730	*Ca_kabuli_Chr01*	14163718	T/G	Gene_Ca_07118	NON_SYNONYMOUS_CODING	Prephenate dehydratase
Kabuli_wild_14237273	1399932731	*Ca_kabuli_Chr01*	14237273	A/T	Gene_Ca_07112	NON_SYNONYMOUS_CODING	Cellulose synthase
Kabuli_wild_14402048	1399932732	*Ca_kabuli_Chr01*	14402048	A/G	Gene_Ca_07094	SYNONYMOUS_CODING	Metallo-dependentphosphatase
Kabuli_wild_14511051	1399932733	*Ca_kabuli_Chr01*	14511051	T/A	Gene_Ca_07083	INTRON	Protein kinase,catalytic domain
Kabuli_wild_14511053	1399932734	*Ca_kabuli_Chr01*	14511053	T/A	Gene_Ca_07083	INTRON	Protein kinase,catalytic domain
Kabuli_wild_14511057	1399932735	*Ca_kabuli_Chr01*	14511057	A/C	Gene_Ca_07083	INTRON	Protein kinase,catalytic domain
Kabuli_wild_14623271	1399932736	*Ca_kabuli_Chr01*	14623271	T/C	Gene_Ca_07072	UPSTREAM	Dcp1-likedecapping
Kabuli_wild_14623294	1399932737	*Ca_kabuli_Chr01*	14623294	T/A	Gene_Ca_07072	UPSTREAM	Dcp1-likedecapping
Kabuli_wild_14698492	1399932738	*Ca_kabuli_Chr01*	14698492	G/C	Gene_Ca_07061	INTRON	Glycosidehydrolase,family17
Kabuli_wild_14698499	1399932739	*Ca_kabuli_Chr01*	14698499	T/A	Gene_Ca_07061	INTRON	Glycosidehydrolase,family17
Kabuli_wild_14769866	1399932740	*Ca_kabuli_Chr01*	14769866	T/C	Gene_Ca_07053	INTRON	NA
Kabuli_wild_14769887	1399932741	*Ca_kabuli_Chr01*	14769887	G/A	Gene_Ca_07053	INTRON	NA
Kabuli_wild_14916633	1399932742	*Ca_kabuli_Chr01*	14916633	G/C	Gene_Ca_07041	UPSTREAM	Ribosomal protein 60S
Kabuli_wild_14959965	1399932743	*Ca_kabuli_Chr01*	14959965	G/C	Gene_Ca_07037	INTRON	Golgin,RAB6-interacting
Kabuli_wild_15140980	1399932744	*Ca_kabuli_Chr01*	15140980	T/G	Gene_Ca_07020	INTRON	Zinc finger,PHD-type
Kabuli_wild_15149240	1399932745	*Ca_kabuli_Chr01*	15149240	A/C	Gene_Ca_07019	INTRON	Clathrin/coatomeradaptor,adaptin-like,N-terminal
Kabuli_wild_15241302	1399932746	*Ca_kabuli_Chr01*	15241302	A/G	Gene_Ca_07010	INTRON	ATP aseassembly factor ATP10, mitochondria
Kabuli_wild_15246375	1399932747	*Ca_kabuli_Chr01*	15246375	T/G	NA	INTERGENIC	NA
Kabuli_wild_15253285	1399932748	*Ca_kabuli_Chr01*	15253285	A/G	Gene_Ca_07009	UPSTREAM	PIG-P
Kabuli_wild_15291773	1399932749	*Ca_kabuli_Chr01*	15291773	C/G	NA	INTERGENIC	NA
Kabuli_wild_15319181	1399932750	*Ca_kabuli_Chr01*	15319181	T/C	Gene_Ca_07002	SYNONYMOUS_CODING	Zinc finger,PHD-type
Kabuli_wild_15613071	1399932751	*Ca_kabuli_Chr01*	15613071	A/C	Gene_Ca_06983	INTRON	RasGTPase
Kabuli_wild_15636900	1399932752	*Ca_kabuli_Chr01*	15636900	A/C	NA	INTERGENIC	NA
Kabuli_wild_15683101	1399932753	*Ca_kabuli_Chr01*	15683101	A/C	NA	INTERGENIC	NA
Kabuli_wild_15683117	1399932754	*Ca_kabuli_Chr01*	15683117	G/T	NA	INTERGENIC	NA
Kabuli_wild_15760638	1399932755	*Ca_kabuli_Chr01*	15760638	C/A	Gene_Ca_06971	INTRON	Domain of unknown function DUF1692
Kabuli_wild_15783972	1399932756	*Ca_kabuli_Chr01*	15783972	T/G	Gene_Ca_06969	SYNONYMOUS_CODING	Rab-GAP/TBCdomain
Kabuli_wild_15862538	1399932757	*Ca_kabuli_Chr01*	15862538	G/T	Gene_Ca_06964	STOP_GAINED	Oxoglutarate/iron-dependent oxygenase
Kabuli_wild_15960425	1399932758	*Ca_kabuli_Chr01*	15960425	A/C	Gene_Ca_06958	INTRON	Glycosyltransferase,family8
Kabuli_wild_15965278	1399932759	*Ca_kabuli_Chr01*	15965278	G/A	NA	INTERGENIC	NA
Kabuli_wild_15989032	1399932760	*Ca_kabuli_Chr01*	15989032	C/T	Gene_Ca_06953	NON_SYNONYMOUS_CODING	Kinesin,motordomain
Kabuli_wild_16061385	1399932761	*Ca_kabuli_Chr01*	16061385	A/G	Gene_Ca_06944	DOWNSTREAM	Translation initiation factor1A(eIF-1A)
Kabuli_wild_16081360	1399932762	*Ca_kabuli_Chr01*	16081360	A/C	NA	INTERGENIC	NA
Kabuli_wild_16099437	1399932763	*Ca_kabuli_Chr01*	16099437	T/G	NA	INTERGENIC	NA
Kabuli_wild_16099438	1399932764	*Ca_kabuli_Chr01*	16099438	G/A	NA	INTERGENIC	NA
Kabuli_wild_16099444	1399932765	*Ca_kabuli_Chr01*	16099444	T/C	NA	INTERGENIC	NA
Kabuli_wild_16099457	1399932766	*Ca_kabuli_Chr01*	16099457	T/C	NA	INTERGENIC	NA
Kabuli_wild_16099459	1399932767	*Ca_kabuli_Chr01*	16099459	T/C	NA	INTERGENIC	NA
Kabuli_wild_16099460	1399932768	*Ca_kabuli_Chr01*	16099460	G/A	NA	INTERGENIC	NA
Kabuli_wild_16099468	1399932769	*Ca_kabuli_Chr01*	16099468	G/A	NA	INTERGENIC	NA
Kabuli_wild_16099476	1399932770	*Ca_kabuli_Chr01*	16099476	T/G	NA	INTERGENIC	NA
Kabuli_wild_16104255	1399932771	*Ca_kabuli_Chr01*	16104255	G/T	Gene_Ca_06941	DOWNSTREAM	Integrase,catalyticcore

SNP IDs	NCBI dbSNP accession IDs (NCBI_SS#)	Chromosomes	Physical positions (bp)	SNPs	Gene accession IDs	Sequence components of genome	Putative functions
Kabuli_wild_16104275	1399932772	*Ca_kabuli_Chr01*	16104275	C/T	Gene_Ca_06941	DOWNSTREAM	Integrase,catalyticcore
Kabuli_wild_16104298	1399932773	*Ca_kabuli_Chr01*	16104298	C/T	Gene_Ca_06941	DOWNSTREAM	Integrase,catalyticcore
Kabuli_wild_16104334	1399932774	*Ca_kabuli_Chr01*	16104334	T/G	Gene_Ca_06941	DOWNSTREAM	Integrase,catalyticcore
Kabuli_wild_16104351	1399932775	*Ca_kabuli_Chr01*	16104351	G/A	Gene_Ca_06941	DOWNSTREAM	Integrase,catalyticcore
Kabuli_wild_16114607	1399932776	*Ca_kabuli_Chr01*	16114607	C/A	Gene_Ca_06940	NON_SYNONYMOUS_CODING	General substrate transporter
Kabuli_wild_16196930	1399932777	*Ca_kabuli_Chr01*	16196930	A/G	NA	INTERGENIC	NA
Kabuli_wild_16228039	1399932778	*Ca_kabuli_Chr01*	16228039	C/A	Gene_Ca_06935	INTRON	NA
Kabuli_wild_16228049	1399932779	*Ca_kabuli_Chr01*	16228049	C/A	Gene_Ca_06935	INTRON	NA
Kabuli_wild_16329161	1399932780	*Ca_kabuli_Chr01*	16329161	G/A	NA	INTERGENIC	NA
Kabuli_wild_16376254	1399932781	*Ca_kabuli_Chr01*	16376254	G/T	Gene_Ca_06927	NON_SYNONYMOUS_CODING	Ubiquitin-associated/translation elongation factor EF1B, N-terminal
Kabuli_wild_16379239	1399932782	*Ca_kabuli_Chr01*	16379239	A/G	Gene_Ca_06927	INTRON	NADPH-dependent FMN reductase
Kabuli_wild_16379258	1399932783	*Ca_kabuli_Chr01*	16379258	C/T	Gene_Ca_06927	INTRON	NADPH-dependent FMN reductase
Kabuli_wild_16385920	1399932784	*Ca_kabuli_Chr01*	16385920	A/G	NA	INTERGENIC	NA
Kabuli_wild_16454432	1399932785	*Ca_kabuli_Chr01*	16454432	C/T	NA	INTERGENIC	NA
Kabuli_wild_16454462	1399932786	*Ca_kabuli_Chr01*	16454462	T/C	NA	INTERGENIC	NA
Kabuli_wild_16474738	1399932787	*Ca_kabuli_Chr01*	16474738	A/G	Gene_Ca_06916	INTRON	IQ motif,EF-handbinding site
Kabuli_wild_16493894	1399932788	*Ca_kabuli_Chr01*	16493894	A/G	Gene_Ca_06914	SYNONYMOUS_CODING	PeptidaseS54,rhomboid
Kabuli_wild_16575167	1399932789	*Ca_kabuli_Chr01*	16575167	A/G	Gene_Ca_06906	INTRON	Proteinphosphatase2C-like
Kabuli_wild_16578172	1399932790	*Ca_kabuli_Chr01*	16578172	C/T	NA	INTERGENIC	NA
Kabuli_wild_16813611	1399932791	*Ca_kabuli_Chr01*	16813611	T/G	Gene_Ca_06890	INTRON	NA
Kabuli_wild_16933430	1399932792	*Ca_kabuli_Chr01*	16933430	C/A	NA	INTERGENIC	NA
Kabuli_wild_16933431	1399932793	*Ca_kabuli_Chr01*	16933431	T/G	NA	INTERGENIC	NA
Kabuli_wild_17023111	1399932794	*Ca_kabuli_Chr01*	17023111	G/C	Gene_Ca_06866	NON_SYNONYMOUS_CODING	Protein kinase,catalytic domain
Kabuli_wild_17093640	1399932795	*Ca_kabuli_Chr01*	17093640	T/C	Gene_Ca_06863	NON_SYNONYMOUS_CODING	NA
Kabuli_wild_17167019	1399932796	*Ca_kabuli_Chr01*	17167019	G/A	NA	INTERGENIC	NA
Kabuli_wild_17167030	1399932797	*Ca_kabuli_Chr01*	17167030	A/G	NA	INTERGENIC	NA
Kabuli_wild_17170727	1399932798	*Ca_kabuli_Chr01*	17170727	T/C	NA	INTERGENIC	NA
Kabuli_wild_17194835	1399932799	*Ca_kabuli_Chr01*	17194835	C/T	NA	INTERGENIC	NA
Kabuli_wild_17326647	1399932800	*Ca_kabuli_Chr01*	17326647	A/C	NA	INTERGENIC	NA
Kabuli_wild_17328721	1399932801	*Ca_kabuli_Chr01*	17328721	G/A	Gene_Ca_22781	SYNONYMOUS_CODING	Bromodomain
Kabuli_wild_17359686	1399932802	*Ca_kabuli_Chr01*	17359686	C/T	Gene_Ca_22785	INTRON	Domain of unknown function DUF625
Kabuli_wild_17359688	1399932803	*Ca_kabuli_Chr01*	17359688	A/C	Gene_Ca_22785	INTRON	Domain of unknown function DUF625
Kabuli_wild_17363322	1399932804	*Ca_kabuli_Chr01*	17363322	G/A	Gene_Ca_22785	INTRON	Domain of unknown function DUF625
Kabuli_wild_17386808	1399932805	*Ca_kabuli_Chr01*	17386808	T/C	Gene_Ca_22785	INTRON	Domain of unknown function DUF625
Kabuli_wild_17395543	1399932806	*Ca_kabuli_Chr01*	17395543	C/A	Gene_Ca_22785	NON_SYNONYMOUS_CODING	Domain of unknown function DUF625
Kabuli_wild_17404511	1399932807	*Ca_kabuli_Chr01*	17404511	C/A	NA	INTERGENIC	NA
Kabuli_wild_17437336	1399932808	*Ca_kabuli_Chr01*	17437336	G/C	Gene_Ca_14789	SYNONYMOUS_CODING	Glycosidehydrolase,family19,catalytic
Kabuli_wild_17462563	1399932809	*Ca_kabuli_Chr01*	17462563	C/A	NA	INTERGENIC	NA
Kabuli_wild_17462564	1399932810	*Ca_kabuli_Chr01*	17462564	G/A	NA	INTERGENIC	NA
Kabuli_wild_17462577	1399932811	*Ca_kabuli_Chr01*	17462577	C/T	NA	INTERGENIC	NA
Kabuli_wild_17462593	1399932812	*Ca_kabuli_Chr01*	17462593	G/T	NA	INTERGENIC	NA
Kabuli_wild_17560161	1399932813	*Ca_kabuli_Chr01*	17560161	C/T	Gene_Ca_14780	NON_SYNONYMOUS_CODING	Signal transduction response regulator, receiverdomain
Kabuli_wild_17763947	1399932814	*Ca_kabuli_Chr01*	17763947	G/A	Gene_Ca_14761	SYNONYMOUS_CODING	Lipase,class3
Kabuli_wild_17799655	1399932815	*Ca_kabuli_Chr01*	17799655	C/A	Gene_Ca_14758	NON_SYNONYMOUS_CODING	Pathogenesis-related transcriptional factor/ERF, DNA-binding

SNP IDs	NCBI dbSNP accession IDs (NCBI_SS#)	Chromosomes	Physical positions (bp)	SNPs	Gene accession IDs	Sequence components of genome	Putative functions
Kabuli_wild_17963953	1399932816	*Ca_kabuli_Chr01*	17963953	A/C	Gene_Ca_14746	SYNONYMOUS_CODING	Crotonase,core
Kabuli_wild_17983301	1399932817	*Ca_kabuli_Chr01*	17983301	T/G	Gene_Ca_14744	SYNONYMOUS_CODING	RAG1-activatingprotein-1-related
Kabuli_wild_17983310	1399932818	*Ca_kabuli_Chr01*	17983310	T/G	Gene_Ca_14744	SYNONYMOUS_CODING	RAG1-activatingprotein-1-related
Kabuli_wild_17983319	1399932819	*Ca_kabuli_Chr01*	17983319	T/G	Gene_Ca_14744	SYNONYMOUS_CODING	RAG1-activatingprotein-1-related
Kabuli_wild_18015888	1399932820	*Ca_kabuli_Chr01*	18015888	A/G	Gene_Ca_14741	NON_SYNONYMOUS_CODING	Pentatrico peptide repeat
Kabuli_wild_18016481	1399932821	*Ca_kabuli_Chr01*	18016481	G/A	Gene_Ca_14741	NON_SYNONYMOUS_CODING	Pentatrico peptide repeat
Kabuli_wild_18017316	1399932822	*Ca_kabuli_Chr01*	18017316	T/C	Gene_Ca_14741	DOWNSTREAM	Pentatrico peptide repeat
Kabuli_wild_18064930	1399932823	*Ca_kabuli_Chr01*	18064930	C/T	Gene_Ca_14736	NON_SYNONYMOUS_CODING	NA
Kabuli_wild_18065022	1399932824	*Ca_kabuli_Chr01*	18065022	G/A	Gene_Ca_14736	SYNONYMOUS_CODING	NA
Kabuli_wild_18065239	1399932825	*Ca_kabuli_Chr01*	18065239	T/C	Gene_Ca_14736	NON_SYNONYMOUS_CODING	NA
Kabuli_wild_18068126	1399932826	*Ca_kabuli_Chr01*	18068126	T/C	Gene_Ca_14736	NON_SYNONYMOUS_CODING	NA
Kabuli_wild_18092704	1399932827	*Ca_kabuli_Chr01*	18092704	T/G	NA	INTERGENIC	NA
Kabuli_wild_18131516	1399932828	*Ca_kabuli_Chr01*	18131516	T/G	NA	INTERGENIC	NA
Kabuli_wild_18150178	1399932829	*Ca_kabuli_Chr01*	18150178	G/A	NA	INTERGENIC	NA
Kabuli_wild_18156634	1399932830	*Ca_kabuli_Chr01*	18156634	A/G	Gene_Ca_14728	UPSTREAM	Skb1 methyltransferase
Kabuli_wild_18172762	1399932831	*Ca_kabuli_Chr01*	18172762	G/T	NA	INTERGENIC	NA
Kabuli_wild_18198444	1399932832	*Ca_kabuli_Chr01*	18198444	C/T	Gene_Ca_14723	INTRON	NA
Kabuli_wild_18488172	1399932833	*Ca_kabuli_Chr01*	18488172	G/A	NA	INTERGENIC	NA
Kabuli_wild_18763911	1399932834	*Ca_kabuli_Chr01*	18763911	C/A	Gene_Ca_17380	INTRON	SET domain
Kabuli_wild_18763916	1399932835	*Ca_kabuli_Chr01*	18763916	G/A	Gene_Ca_17380	INTRON	SET domain
Kabuli_wild_18764225	1399932836	*Ca_kabuli_Chr01*	18764225	C/T	Gene_Ca_17380	INTRON	SET domain
Kabuli_wild_18797321	1399932837	*Ca_kabuli_Chr01*	18797321	C/G	NA	INTERGENIC	NA
Kabuli_wild_18890616	1399932838	*Ca_kabuli_Chr01*	18890616	G/A	Gene_Ca_17371	SYNONYMOUS_CODING	Parallelbeta-helixrepeat
Kabuli_wild_18910202	1399932839	*Ca_kabuli_Chr01*	18910202	G/A	NA	INTERGENIC	NA
Kabuli_wild_19000445	1399932840	*Ca_kabuli_Chr01*	19000445	T/C	NA	INTERGENIC	NA
Kabuli_wild_19130872	1399932841	*Ca_kabuli_Chr01*	19130872	G/A	NA	INTERGENIC	NA
Kabuli_wild_19130883	1399932842	*Ca_kabuli_Chr01*	19130883	C/A	NA	INTERGENIC	NA
Kabuli_wild_19130884	1399932843	*Ca_kabuli_Chr01*	19130884	C/A	NA	INTERGENIC	NA
Kabuli_wild_19130894	1399932844	*Ca_kabuli_Chr01*	19130894	T/C	NA	INTERGENIC	NA
Kabuli_wild_19165730	1399932845	*Ca_kabuli_Chr01*	19165730	A/T	NA	INTERGENIC	NA
Kabuli_wild_19165742	1399932846	*Ca_kabuli_Chr01*	19165742	G/T	NA	INTERGENIC	NA
Kabuli_wild_19218067	1399932847	*Ca_kabuli_Chr01*	19218067	G/A	NA	INTERGENIC	NA
Kabuli_wild_19218069	1399932848	*Ca_kabuli_Chr01*	19218069	T/A	NA	INTERGENIC	NA
Kabuli_wild_19218093	1399932849	*Ca_kabuli_Chr01*	19218093	T/G	NA	INTERGENIC	NA
Kabuli_wild_19218136	1399932850	*Ca_kabuli_Chr01*	19218136	G/A	NA	INTERGENIC	NA
Kabuli_wild_19218140	1399932851	*Ca_kabuli_Chr01*	19218140	C/A	NA	INTERGENIC	NA
Kabuli_wild_19254161	1399932852	*Ca_kabuli_Chr01*	19254161	A/G	Gene_Ca_17349	INTRON	NA
Kabuli_wild_19359812	1399932853	*Ca_kabuli_Chr01*	19359812	A/C	Gene_Ca_17340	SYNONYMOUS_CODING	Pentatrico peptide repeat
Kabuli_wild_19359844	1399932854	*Ca_kabuli_Chr01*	19359844	C/A	Gene_Ca_17340	NON_SYNONYMOUS_CODING	Pentatrico peptide repeat
Kabuli_wild_19526320	1399932855	*Ca_kabuli_Chr01*	19526320	T/C	Gene_Ca_08868	NON_SYNONYMOUS_CODING	Aldose1-epimerase
Kabuli_wild_19526652	1399932856	*Ca_kabuli_Chr01*	19526652	G/T	Gene_Ca_08868	INTRON	Aldose1-epimerase
Kabuli_wild_19572408	1399932857	*Ca_kabuli_Chr01*	19572408	C/T	Gene_Ca_08866	INTRON	Pumilio RNA-binding repeat
Kabuli_wild_19572494	1399932858	*Ca_kabuli_Chr01*	19572494	G/C	Gene_Ca_08866	INTRON	Pumilio RNA-binding repeat
Kabuli_wild_19591671	1399932859	*Ca_kabuli_Chr01*	19591671	A/G	Gene_Ca_08863	DOWNSTREAM	Domain of unknown function DUF630

SNP IDs	NCBI dbSNP accession IDs (NCBI_SS#)	Chromosomes	Physical positions (bp)	SNPs	Gene accession IDs	Sequence components of genome	Putative functions
Kabuli_wild_19787122	1399932860	*Ca_kabuli_Chr01*	19787122	G/A	NA	INTERGENIC	NA
Kabuli_wild_19903665	1399932861	*Ca_kabuli_Chr01*	19903665	A/G	Gene_Ca_08847	INTRON	RNA polymerase I associated factor, A49-like
Kabuli_wild_19903683	1399932862	*Ca_kabuli_Chr01*	19903683	G/A	Gene_Ca_08847	INTRON	RNA polymerase I associated factor, A49-like
Kabuli_wild_19903686	1399932863	*Ca_kabuli_Chr01*	19903686	T/A	Gene_Ca_08847	INTRON	RNA polymerase I associated factor, A49-like
Kabuli_wild_19903690	1399932864	*Ca_kabuli_Chr01*	19903690	A/G	Gene_Ca_08847	INTRON	RNA polymerase I associated factor, A49-like
Kabuli_wild_19903693	1399932865	*Ca_kabuli_Chr01*	19903693	G/T	Gene_Ca_08847	INTRON	RNA polymerase I associated factor, A49-like
Kabuli_wild_19903701	1399932866	*Ca_kabuli_Chr01*	19903701	G/A	Gene_Ca_08847	INTRON	RNA polymerase I associated factor, A49-like
Kabuli_wild_19903704	1399932867	*Ca_kabuli_Chr01*	19903704	G/A	Gene_Ca_08847	INTRON	RNA polymerase I associated factor, A49-like
Kabuli_wild_20016525	1399932868	*Ca_kabuli_Chr01*	20016525	C/T	Gene_Ca_08842	DOWNSTREAM	NA
Kabuli_wild_20021166	1399932869	*Ca_kabuli_Chr01*	20021166	T/C	Gene_Ca_08841	INTRON	ANTH
Kabuli_wild_20069962	1399932870	*Ca_kabuli_Chr01*	20069962	A/G	Gene_Ca_08835	UPSTREAM	NA
Kabuli_wild_20069963	1399932871	*Ca_kabuli_Chr01*	20069963	A/G	Gene_Ca_08835	UPSTREAM	NA
Kabuli_wild_20070090	1399932872	*Ca_kabuli_Chr01*	20070090	G/A	Gene_Ca_08835	UPSTREAM	NA
Kabuli_wild_20070102	1399932873	*Ca_kabuli_Chr01*	20070102	C/T	Gene_Ca_08835	UPSTREAM	NA
Kabuli_wild_20070103	1399932874	*Ca_kabuli_Chr01*	20070103	C/T	Gene_Ca_08835	UPSTREAM	NA
Kabuli_wild_20070104	1399932875	*Ca_kabuli_Chr01*	20070104	A/G	Gene_Ca_08835	UPSTREAM	NA
Kabuli_wild_20091291	1399932876	*Ca_kabuli_Chr01*	20091291	T/G	Gene_Ca_08834	NON_SYNONYMOUS_CODING	BRCT
Kabuli_wild_20113571	1399932877	*Ca_kabuli_Chr01*	20113571	G/C	Gene_Ca_08833	INTRON	Metallo-dependentphosphatase
Kabuli_wild_21577377	1399932878	*Ca_kabuli_Chr01*	21577377	C/T	Gene_Ca_22120	NON_SYNONYMOUS_CODING	NA
Kabuli_wild_21881142	1399932879	*Ca_kabuli_Chr01*	21881142	G/A	Gene_Ca_22109	NON_SYNONYMOUS_CODING	NA
Kabuli_wild_21881174	1399932880	*Ca_kabuli_Chr01*	21881174	G/T	Gene_Ca_22109	SYNONYMOUS_CODING	NA
Kabuli_wild_21881178	1399932881	*Ca_kabuli_Chr01*	21881178	G/T	Gene_Ca_22109	NON_SYNONYMOUS_CODING	NA
Kabuli_wild_22422226	1399932882	*Ca_kabuli_Chr01*	22422226	C/T	Gene_Ca_20627	SYNONYMOUS_CODING	NA
Kabuli_wild_22465555	1399932883	*Ca_kabuli_Chr01*	22465555	C/T	NA	INTERGENIC	NA
Kabuli_wild_22719581	1399932884	*Ca_kabuli_Chr01*	22719581	T/G	NA	INTERGENIC	NA
Kabuli_wild_22743361	1399932885	*Ca_kabuli_Chr01*	22743361	T/G	Gene_Ca_20613	NON_SYNONYMOUS_CODING	Nucleosome assembl yprotein (NAP)
Kabuli_wild_23129094	1399932886	*Ca_kabuli_Chr01*	23129094	C/T	Gene_Ca_22358	INTRON	Helicase,C-terminal
Kabuli_wild_23131178	1399932887	*Ca_kabuli_Chr01*	23131178	A/C	Gene_Ca_22358	INTRON	Helicase,C-terminal
Kabuli_wild_23148736	1399932888	*Ca_kabuli_Chr01*	23148736	A/C	Gene_Ca_22359	SYNONYMOUS_CODING	Initiation factor2B-related
Kabuli_wild_23149053	1399932889	*Ca_kabuli_Chr01*	23149053	A/G	Gene_Ca_22359	SYNONYMOUS_CODING	Initiation factor2B-related
Kabuli_wild_23149096	1399932890	*Ca_kabuli_Chr01*	23149096	A/C	Gene_Ca_22359	NON_SYNONYMOUS_CODING	Initiation factor2B-related
Kabuli_wild_23382483	1399932891	*Ca_kabuli_Chr01*	23382483	T/A	NA	INTERGENIC	NA
Kabuli_wild_23382682	1399932892	*Ca_kabuli_Chr01*	23382682	T/A	NA	INTERGENIC	NA
Kabuli_wild_23522587	1399932893	*Ca_kabuli_Chr01*	23522587	G/A	NA	INTERGENIC	NA
Kabuli_wild_23555158	1399932894	*Ca_kabuli_Chr01*	23555158	T/G	Gene_Ca_19312	SYNONYMOUS_CODING	vonWille brand factor,typeA
Kabuli_wild_23724947	1399932895	*Ca_kabuli_Chr01*	23724947	C/A	Gene_Ca_19301	NON_SYNONYMOUS_CODING	Peptidase C48, SUMO/Sentrin/Ubl1
Kabuli_wild_23885494	1399932896	*Ca_kabuli_Chr01*	23885494	G/C	NA	INTERGENIC	NA
Kabuli_wild_23885509	1399932897	*Ca_kabuli_Chr01*	23885509	T/C	NA	INTERGENIC	NA
Kabuli_wild_23926963	1399932898	*Ca_kabuli_Chr01*	23926963	T/C	NA	INTERGENIC	NA
Kabuli_wild_23926980	1399932899	*Ca_kabuli_Chr01*	23926980	C/T	NA	INTERGENIC	NA
Kabuli_wild_23926983	1399932900	*Ca_kabuli_Chr01*	23926983	G/T	NA	INTERGENIC	NA
Kabuli_wild_23926985	1399932901	*Ca_kabuli_Chr01*	23926985	C/T	NA	INTERGENIC	NA
Kabuli_wild_23926986	1399932902	*Ca_kabuli_Chr01*	23926986	T/C	NA	INTERGENIC	NA
Kabuli_wild_23927016	1399932903	*Ca_kabuli_Chr01*	23927016	G/A	NA	INTERGENIC	NA

SNP IDs	NCBI dbSNP accession IDs (NCBI_SS#)	Chromosomes	Physical positions (bp)	SNPs	Gene accession IDs	Sequence components of genome	Putative functions
Kabuli_wild_23927018	1399932904	*Ca_kabuli_Chr01*	23927018	T/A	NA	INTERGENIC	NA
Kabuli_wild_23927022	1399932905	*Ca_kabuli_Chr01*	23927022	T/A	NA	INTERGENIC	NA
Kabuli_wild_23941438	1399932906	*Ca_kabuli_Chr01*	23941438	G/A	NA	INTERGENIC	NA
Kabuli_wild_24021013	1399932907	*Ca_kabuli_Chr01*	24021013	C/T	NA	INTERGENIC	NA
Kabuli_wild_24071014	1399932908	*Ca_kabuli_Chr01*	24071014	T/G	Gene_Ca_19289	INTRON	AUX/IAAprotein
Kabuli_wild_24173273	1399932909	*Ca_kabuli_Chr01*	24173273	A/G	NA	INTERGENIC	NA
Kabuli_wild_24226808	1399932910	*Ca_kabuli_Chr01*	24226808	A/G	Gene_Ca_19284	NON_SYNONYMOUS_CODING	Beta-ketoacylsynthase
Kabuli_wild_24450415	1399932911	*Ca_kabuli_Chr01*	24450415	T/C	Gene_Ca_18343	INTRON	Ribulose-phosphate3-epimerase
Kabuli_wild_24450483	1399932912	*Ca_kabuli_Chr01*	24450483	T/G	Gene_Ca_18343	INTRON	Ribulose-phosphate3-epimerase
Kabuli_wild_24450511	1399932913	*Ca_kabuli_Chr01*	24450511	A/G	Gene_Ca_18343	INTRON	Ribulose-phosphate3-epimerase
Kabuli_wild_24560005	1399932914	*Ca_kabuli_Chr01*	24560005	A/C	NA	INTERGENIC	NA
Kabuli_wild_24560013	1399932915	*Ca_kabuli_Chr01*	24560013	A/C	NA	INTERGENIC	NA
Kabuli_wild_24625378	1399932916	*Ca_kabuli_Chr01*	24625378	G/A	NA	INTERGENIC	NA
Kabuli_wild_24625381	1399932917	*Ca_kabuli_Chr01*	24625381	G/A	NA	INTERGENIC	NA
Kabuli_wild_24625390	1399932918	*Ca_kabuli_Chr01*	24625390	T/A	NA	INTERGENIC	NA
Kabuli_wild_24625417	1399932919	*Ca_kabuli_Chr01*	24625417	G/A	NA	INTERGENIC	NA
Kabuli_wild_24625442	1399932920	*Ca_kabuli_Chr01*	24625442	G/A	NA	INTERGENIC	NA
Kabuli_wild_24625496	1399932921	*Ca_kabuli_Chr01*	24625496	A/G	NA	INTERGENIC	NA
Kabuli_wild_24636949	1399932922	*Ca_kabuli_Chr01*	24636949	C/T	NA	INTERGENIC	NA
Kabuli_wild_24658560	1399932923	*Ca_kabuli_Chr01*	24658560	A/T	Gene_Ca_18337	STOP_GAINED	AuxinresponsiveSAURprotein
Kabuli_wild_24693443	1399932924	*Ca_kabuli_Chr01*	24693443	G/A	NA	INTERGENIC	NA
Kabuli_wild_24693477	1399932925	*Ca_kabuli_Chr01*	24693477	C/T	NA	INTERGENIC	NA
Kabuli_wild_24693518	1399932926	*Ca_kabuli_Chr01*	24693518	G/A	NA	INTERGENIC	NA
Kabuli_wild_24693521	1399932927	*Ca_kabuli_Chr01*	24693521	G/A	NA	INTERGENIC	NA
Kabuli_wild_24693527	1399932928	*Ca_kabuli_Chr01*	24693527	G/A	NA	INTERGENIC	NA
Kabuli_wild_24693533	1399932929	*Ca_kabuli_Chr01*	24693533	G/A	NA	INTERGENIC	NA
Kabuli_wild_24693550	1399932930	*Ca_kabuli_Chr01*	24693550	G/A	NA	INTERGENIC	NA
Kabuli_wild_24693555	1399932931	*Ca_kabuli_Chr01*	24693555	G/A	NA	INTERGENIC	NA
Kabuli_wild_24693571	1399932932	*Ca_kabuli_Chr01*	24693571	G/A	NA	INTERGENIC	NA
Kabuli_wild_24693585	1399932933	*Ca_kabuli_Chr01*	24693585	T/A	NA	INTERGENIC	NA
Kabuli_wild_24693603	1399932934	*Ca_kabuli_Chr01*	24693603	T/C	NA	INTERGENIC	NA
Kabuli_wild_24828245	1399932935	*Ca_kabuli_Chr01*	24828245	A/C	NA	INTERGENIC	NA
Kabuli_wild_24845148	1399932936	*Ca_kabuli_Chr01*	24845148	A/C	Gene_Ca_18325	SYNONYMOUS_CODING	Zinc finger, RING-type
Kabuli_wild_25083482	1399932937	*Ca_kabuli_Chr01*	25083482	A/C	Gene_Ca_18313	INTRON	Pectinesterase,catalytic
Kabuli_wild_25121972	1399932938	*Ca_kabuli_Chr01*	25121972	G/A	Gene_Ca_18312	UPSTREAM	Pectinesterase,catalytic
Kabuli_wild_25524375	1399932939	*Ca_kabuli_Chr01*	25524375	C/T	Gene_Ca_25081	NON_SYNONYMOUS_CODING	Peptidase C48, SUMO/Sentrin/Ubl1
Kabuli_wild_25524377	1399932940	*Ca_kabuli_Chr01*	25524377	A/G	Gene_Ca_25081	START_LOST	Peptidase C48, SUMO/Sentrin/Ubl1
Kabuli_wild_25524382	1399932941	*Ca_kabuli_Chr01*	25524382	C/T	NA	INTERGENIC	NA
Kabuli_wild_25524410	1399932942	*Ca_kabuli_Chr01*	25524410	C/T	NA	INTERGENIC	NA
Kabuli_wild_25524423	1399932943	*Ca_kabuli_Chr01*	25524423	G/T	NA	INTERGENIC	NA
Kabuli_wild_25524425	1399932944	*Ca_kabuli_Chr01*	25524425	C/T	NA	INTERGENIC	NA
Kabuli_wild_25524441	1399932945	*Ca_kabuli_Chr01*	25524441	T/C	NA	INTERGENIC	NA
Kabuli_wild_25524450	1399932946	*Ca_kabuli_Chr01*	25524450	C/T	NA	INTERGENIC	NA
Kabuli_wild_25524451	1399932947	*Ca_kabuli_Chr01*	25524451	A/T	NA	INTERGENIC	NA

SNP IDs	NCBI dbSNP accession IDs (NCBI_SS#)	Chromosomes	Physical positions (bp)	SNPs	Gene accession IDs	Sequence components of genome	Putative functions
Kabuli_wild_25524452	1399932948	*Ca_kabuli_Chr01*	25524452	G/A	NA	INTERGENIC	NA
Kabuli_wild_25524453	1399932949	*Ca_kabuli_Chr01*	25524453	C/T	NA	INTERGENIC	NA
Kabuli_wild_25524455	1399932950	*Ca_kabuli_Chr01*	25524455	A/G	NA	INTERGENIC	NA
Kabuli_wild_25524456	1399932951	*Ca_kabuli_Chr01*	25524456	C/T	NA	INTERGENIC	NA
Kabuli_wild_25524458	1399932952	*Ca_kabuli_Chr01*	25524458	G/A	NA	INTERGENIC	NA
Kabuli_wild_25524460	1399932953	*Ca_kabuli_Chr01*	25524460	G/A	NA	INTERGENIC	NA
Kabuli_wild_25524465	1399932954	*Ca_kabuli_Chr01*	25524465	A/G	NA	INTERGENIC	NA
Kabuli_wild_25524470	1399932955	*Ca_kabuli_Chr01*	25524470	C/T	NA	INTERGENIC	NA
Kabuli_wild_25524473	1399932956	*Ca_kabuli_Chr01*	25524473	C/T	NA	INTERGENIC	NA
Kabuli_wild_25524478	1399932957	*Ca_kabuli_Chr01*	25524478	C/A	NA	INTERGENIC	NA
Kabuli_wild_25524480	1399932958	*Ca_kabuli_Chr01*	25524480	G/A	NA	INTERGENIC	NA
Kabuli_wild_25524481	1399932959	*Ca_kabuli_Chr01*	25524481	T/A	NA	INTERGENIC	NA
Kabuli_wild_25524488	1399932960	*Ca_kabuli_Chr01*	25524488	G/T	NA	INTERGENIC	NA
Kabuli_wild_25524494	1399932961	*Ca_kabuli_Chr01*	25524494	A/G	NA	INTERGENIC	NA
Kabuli_wild_25524504	1399932962	*Ca_kabuli_Chr01*	25524504	C/T	NA	INTERGENIC	NA
Kabuli_wild_25524505	1399932963	*Ca_kabuli_Chr01*	25524505	A/G	NA	INTERGENIC	NA
Kabuli_wild_25524519	1399932964	*Ca_kabuli_Chr01*	25524519	C/T	NA	INTERGENIC	NA
Kabuli_wild_25524520	1399932965	*Ca_kabuli_Chr01*	25524520	C/T	NA	INTERGENIC	NA
Kabuli_wild_25524524	1399932966	*Ca_kabuli_Chr01*	25524524	C/T	NA	INTERGENIC	NA
Kabuli_wild_25524537	1399932967	*Ca_kabuli_Chr01*	25524537	C/T	NA	INTERGENIC	NA
Kabuli_wild_25664814	1399932968	*Ca_kabuli_Chr01*	25664814	T/C	NA	INTERGENIC	NA
Kabuli_wild_25887539	1399932969	*Ca_kabuli_Chr01*	25887539	G/A	NA	INTERGENIC	NA
Kabuli_wild_26129816	1399932970	*Ca_kabuli_Chr01*	26129816	A/G	Gene_Ca_18595	SYNONYMOUS_CODING	S-adenosylmethioninesynthetase
Kabuli_wild_26347869	1399932971	*Ca_kabuli_Chr01*	26347869	T/C	NA	INTERGENIC	NA
Kabuli_wild_26347894	1399932972	*Ca_kabuli_Chr01*	26347894	T/C	NA	INTERGENIC	NA
Kabuli_wild_26348859	1399932973	*Ca_kabuli_Chr01*	26348859	G/A	NA	INTERGENIC	NA
Kabuli_wild_26574061	1399932974	*Ca_kabuli_Chr01*	26574061	G/A	Gene_Ca_18614	NON_SYNONYMOUS_CODING	Peptidase C48, SUMO/Sentrin/Ubl1
Kabuli_wild_26574062	1399932975	*Ca_kabuli_Chr01*	26574062	C/A	Gene_Ca_18614	NON_SYNONYMOUS_CODING	Peptidase C48, SUMO/Sentrin/Ubl1
Kabuli_wild_26574133	1399932976	*Ca_kabuli_Chr01*	26574133	A/G	Gene_Ca_18614	NON_SYNONYMOUS_CODING	Peptidase C48, SUMO/Sentrin/Ubl1
Kabuli_wild_26574138	1399932977	*Ca_kabuli_Chr01*	26574138	G/A	Gene_Ca_18614	SYNONYMOUS_CODING	Peptidase C48, SUMO/Sentrin/Ubl1
Kabuli_wild_26574146	1399932978	*Ca_kabuli_Chr01*	26574146	C/T	Gene_Ca_18614	NON_SYNONYMOUS_CODING	Peptidase C48, SUMO/Sentrin/Ubl1
Kabuli_wild_26574172	1399932979	*Ca_kabuli_Chr01*	26574172	C/T	Gene_Ca_18614	NON_SYNONYMOUS_CODING	Peptidase C48, SUMO/Sentrin/Ubl1
Kabuli_wild_26579621	1399932980	*Ca_kabuli_Chr01*	26579621	A/C	NA	INTERGENIC	NA
Kabuli_wild_26633828	1399932981	*Ca_kabuli_Chr01*	26633828	G/A	Gene_Ca_18617	NON_SYNONYMOUS_CODING	UDP-glucuronosyl/UDP-glucosyltransferase
Kabuli_wild_26807395	1399932982	*Ca_kabuli_Chr01*	26807395	T/G	NA	INTERGENIC	NA
Kabuli_wild_26807401	1399932983	*Ca_kabuli_Chr01*	26807401	A/G	NA	INTERGENIC	NA
Kabuli_wild_26856541	1399932984	*Ca_kabuli_Chr01*	26856541	A/T	Gene_Ca_20289	NON_SYNONYMOUS_CODING	NA
Kabuli_wild_26856691	1399932985	*Ca_kabuli_Chr01*	26856691	G/A	Gene_Ca_20289	SYNONYMOUS_CODING	NA
Kabuli_wild_26921471	1399932986	*Ca_kabuli_Chr01*	26921471	C/T	Gene_Ca_20293	DOWNSTREAM	Pentatrico peptide repeat
Kabuli_wild_26922011	1399932987	*Ca_kabuli_Chr01*	26922011	T/G	Gene_Ca_20293	DOWNSTREAM	Pentatrico peptide repeat
Kabuli_wild_26922526	1399932988	*Ca_kabuli_Chr01*	26922526	A/G	NA	INTERGENIC	NA
Kabuli_wild_26953535	1399932989	*Ca_kabuli_Chr01*	26953535	C/T	NA	INTERGENIC	NA
Kabuli_wild_27012495	1399932990	*Ca_kabuli_Chr01*	27012495	C/T	NA	INTERGENIC	NA
Kabuli_wild_27012660	1399932991	*Ca_kabuli_Chr01*	27012660	G/C	NA	INTERGENIC	NA

SNP IDs	NCBI dbSNP accession IDs (NCBI_SS#)	Chromosomes	Physical positions (bp)	SNPs	Gene accession IDs	Sequence components of genome	Putative functions
Kabuli_wild_27014895	1399932992	*Ca_kabuli_Chr01*	27014895	T/G	NA	INTERGENIC	NA
Kabuli_wild_27014951	1399932993	*Ca_kabuli_Chr01*	27014951	A/C	NA	INTERGENIC	NA
Kabuli_wild_27014981	1399932994	*Ca_kabuli_Chr01*	27014981	A/G	NA	INTERGENIC	NA
Kabuli_wild_27016101	1399932995	*Ca_kabuli_Chr01*	27016101	G/A	NA	INTERGENIC	NA
Kabuli_wild_27016206	1399932996	*Ca_kabuli_Chr01*	27016206	T/A	NA	INTERGENIC	NA
Kabuli_wild_27017559	1399932997	*Ca_kabuli_Chr01*	27017559	G/A	Gene_Ca_20299	INTRON	Cystathioninebeta-synthase,core
Kabuli_wild_27017560	1399932998	*Ca_kabuli_Chr01*	27017560	A/G	Gene_Ca_20299	INTRON	Cystathioninebeta-synthase,core
Kabuli_wild_27017570	1399932999	*Ca_kabuli_Chr01*	27017570	A/G	Gene_Ca_20299	INTRON	Cystathioninebeta-synthase,core
Kabuli_wild_27017604	1399933000	*Ca_kabuli_Chr01*	27017604	T/G	Gene_Ca_20299	INTRON	Cystathioninebeta-synthase,core
Kabuli_wild_27017606	1399933001	*Ca_kabuli_Chr01*	27017606	C/T	Gene_Ca_20299	INTRON	Cystathioninebeta-synthase,core
Kabuli_wild_27017617	1399933002	*Ca_kabuli_Chr01*	27017617	G/A	Gene_Ca_20299	INTRON	Cystathioninebeta-synthase,core
Kabuli_wild_27017626	1399933003	*Ca_kabuli_Chr01*	27017626	A/C	Gene_Ca_20299	INTRON	Cystathioninebeta-synthase,core
Kabuli_wild_27017628	1399933004	*Ca_kabuli_Chr01*	27017628	C/A	Gene_Ca_20299	INTRON	Cystathioninebeta-synthase,core
Kabuli_wild_27017644	1399933005	*Ca_kabuli_Chr01*	27017644	C/T	Gene_Ca_20299	INTRON	Cystathioninebeta-synthase,core
Kabuli_wild_27017734	1399933006	*Ca_kabuli_Chr01*	27017734	T/C	Gene_Ca_20299	INTRON	Cystathioninebeta-synthase,core
Kabuli_wild_27017765	1399933007	*Ca_kabuli_Chr01*	27017765	A/T	Gene_Ca_20299	INTRON	Cystathioninebeta-synthase,core
Kabuli_wild_27017771	1399933008	*Ca_kabuli_Chr01*	27017771	G/A	Gene_Ca_20299	INTRON	Cystathioninebeta-synthase,core
Kabuli_wild_27020193	1399933009	*Ca_kabuli_Chr01*	27020193	T/C	Gene_Ca_20299	INTRON	Cystathioninebeta-synthase,core
Kabuli_wild_27020354	1399933010	*Ca_kabuli_Chr01*	27020354	C/A	Gene_Ca_20299	INTRON	Cystathioninebeta-synthase,core
Kabuli_wild_27385788	1399933011	*Ca_kabuli_Chr01*	27385788	G/A	NA	INTERGENIC	NA
Kabuli_wild_27385816	1399933012	*Ca_kabuli_Chr01*	27385816	A/G	NA	INTERGENIC	NA
Kabuli_wild_27461930	1399933013	*Ca_kabuli_Chr01*	27461930	T/C	NA	INTERGENIC	NA
Kabuli_wild_27958553	1399933014	*Ca_kabuli_Chr01*	27958553	T/C	Gene_Ca_18528	SYNONYMOUS_CODING	Reverse transcriptase, RNA-dependent DNA polymerase
Kabuli_wild_27958570	1399933015	*Ca_kabuli_Chr01*	27958570	T/A	Gene_Ca_18528	NON_SYNONYMOUS_CODING	Reverse transcriptase, RNA-dependent DNA polymerase
Kabuli_wild_28088501	1399933016	*Ca_kabuli_Chr01*	28088501	T/C	Gene_Ca_18523	SYNONYMOUS_CODING	Vps54-like
Kabuli_wild_28106756	1399933017	*Ca_kabuli_Chr01*	28106756	G/A	NA	INTERGENIC	NA
Kabuli_wild_28126010	1399933018	*Ca_kabuli_Chr01*	28126010	A/G	NA	INTERGENIC	NA
Kabuli_wild_28133814	1399933019	*Ca_kabuli_Chr01*	28133814	C/T	Gene_Ca_18521	INTRON	Leucine-rich repeat
Kabuli_wild_28133830	1399933020	*Ca_kabuli_Chr01*	28133830	A/T	Gene_Ca_18521	INTRON	Leucine-rich repeat
Kabuli_wild_28133844	1399933021	*Ca_kabuli_Chr01*	28133844	T/C	Gene_Ca_18521	INTRON	Leucine-rich repeat
Kabuli_wild_28133847	1399933022	*Ca_kabuli_Chr01*	28133847	A/G	Gene_Ca_18521	INTRON	Leucine-rich repeat
Kabuli_wild_28143955	1399933023	*Ca_kabuli_Chr01*	28143955	G/A	Gene_Ca_18521	INTRON	Leucine-rich repeat
Kabuli_wild_28152260	1399933024	*Ca_kabuli_Chr01*	28152260	A/G	Gene_Ca_18521	INTRON	Leucine-rich repeat
Kabuli_wild_28656197	1399933025	*Ca_kabuli_Chr01*	28656197	C/A	NA	INTERGENIC	NA
Kabuli_wild_28907617	1399933026	*Ca_kabuli_Chr01*	28907617	G/A	Gene_Ca_18486	NON_SYNONYMOUS_CODING	Sugar/inositol transporter
Kabuli_wild_28909019	1399933027	*Ca_kabuli_Chr01*	28909019	T/G	Gene_Ca_18486	SYNONYMOUS_CODING	Sugar/inositol transporter
Kabuli_wild_28909059	1399933028	*Ca_kabuli_Chr01*	28909059	T/A	Gene_Ca_18486	NON_SYNONYMOUS_CODING	Sugar/inositol transporter
Kabuli_wild_29020220	1399933029	*Ca_kabuli_Chr01*	29020220	T/G	NA	INTERGENIC	NA
Kabuli_wild_29149338	1399933030	*Ca_kabuli_Chr01*	29149338	G/A	Gene_Ca_22571	NON_SYNONYMOUS_CODING	CCTdomain
Kabuli_wild_29171283	1399933031	*Ca_kabuli_Chr01*	29171283	T/G	Gene_Ca_22570	NON_SYNONYMOUS_CODING	Pentatrico peptide repeat
Kabuli_wild_29331630	1399933032	*Ca_kabuli_Chr01*	29331630	A/G	Gene_Ca_22563	INTRON	Peptidase C48, SUMO/Sentrin/Ubl1
Kabuli_wild_29331681	1399933033	*Ca_kabuli_Chr01*	29331681	A/G	Gene_Ca_22563	SYNONYMOUS_CODING	Peptidase C48, SUMO/Sentrin/Ubl1
Kabuli_wild_29331706	1399933034	*Ca_kabuli_Chr01*	29331706	C/A	Gene_Ca_22563	SYNONYMOUS_CODING	Peptidase C48, SUMO/Sentrin/Ubl1
Kabuli_wild_29460440	1399933035	*Ca_kabuli_Chr01*	29460440	C/A	Gene_Ca_22559	NON_SYNONYMOUS_CODING	NA

SNP IDs	NCBI dbSNP accession IDs (NCBI_SS#)	Chromosomes	Physical positions (bp)	SNPs	Gene accession IDs	Sequence components of genome	Putative functions
Kabuli_wild_29637342	1399933036	*Ca_kabuli_Chr01*	29637342	A/G	Gene_Ca_24382	INTRON	K Homology,type1
Kabuli_wild_29637509	1399933037	*Ca_kabuli_Chr01*	29637509	A/C	Gene_Ca_24382	INTRON	K Homology,type1
Kabuli_wild_29637526	1399933038	*Ca_kabuli_Chr01*	29637526	C/T	Gene_Ca_24382	INTRON	K Homology,type1
Kabuli_wild_29637571	1399933039	*Ca_kabuli_Chr01*	29637571	T/G	Gene_Ca_24382	INTRON	K Homology,type1
Kabuli_wild_29637572	1399933040	*Ca_kabuli_Chr01*	29637572	T/G	Gene_Ca_24382	INTRON	K Homology,type1
Kabuli_wild_29661949	1399933041	*Ca_kabuli_Chr01*	29661949	T/C	Gene_Ca_24383	SYNONYMOUS_CODING	NA
Kabuli_wild_29661962	1399933042	*Ca_kabuli_Chr01*	29661962	A/C	Gene_Ca_24383	NON_SYNONYMOUS_CODING	NA
Kabuli_wild_30105943	1399933043	*Ca_kabuli_Chr01*	30105943	G/T	NA	INTERGENIC	NA
Kabuli_wild_30210662	1399933044	*Ca_kabuli_Chr01*	30210662	G/C	NA	INTERGENIC	NA
Kabuli_wild_30232233	1399933045	*Ca_kabuli_Chr01*	30232233	T/C	NA	INTERGENIC	NA
Kabuli_wild_30261132	1399933046	*Ca_kabuli_Chr01*	30261132	T/C	Gene_Ca_22465	SYNONYMOUS_CODING	Kinesin,motordomain
Kabuli_wild_30261572	1399933047	*Ca_kabuli_Chr01*	30261572	G/A	Gene_Ca_22465	NON_SYNONYMOUS_CODING	Kinesin,motordomain
Kabuli_wild_30262124	1399933048	*Ca_kabuli_Chr01*	30262124	A/G	Gene_Ca_22465	NON_SYNONYMOUS_CODING	Kinesin,motordomain
Kabuli_wild_30447050	1399933049	*Ca_kabuli_Chr01*	30447050	A/G	NA	INTERGENIC	NA
Kabuli_wild_30630357	1399933050	*Ca_kabuli_Chr01*	30630357	T/C	NA	INTERGENIC	NA
Kabuli_wild_30948055	1399933051	*Ca_kabuli_Chr01*	30948055	T/C	NA	INTERGENIC	NA
Kabuli_wild_30948066	1399933052	*Ca_kabuli_Chr01*	30948066	A/G	NA	INTERGENIC	NA
Kabuli_wild_30990317	1399933053	*Ca_kabuli_Chr01*	30990317	G/A	NA	INTERGENIC	NA
Kabuli_wild_31080673	1399933054	*Ca_kabuli_Chr01*	31080673	T/G	Gene_Ca_25827	INTRON	Enolase
Kabuli_wild_31080792	1399933055	*Ca_kabuli_Chr01*	31080792	T/C	Gene_Ca_25827	INTRON	NA
Kabuli_wild_31240370	1399933056	*Ca_kabuli_Chr01*	31240370	A/G	NA	INTERGENIC	NA
Kabuli_wild_31240491	1399933057	*Ca_kabuli_Chr01*	31240491	G/A	NA	INTERGENIC	NA
Kabuli_wild_31359837	1399933058	*Ca_kabuli_Chr01*	31359837	G/A	NA	INTERGENIC	NA
Kabuli_wild_31359855	1399933059	*Ca_kabuli_Chr01*	31359855	G/A	NA	INTERGENIC	NA
Kabuli_wild_31359868	1399933060	*Ca_kabuli_Chr01*	31359868	G/A	NA	INTERGENIC	NA
Kabuli_wild_31359912	1399933061	*Ca_kabuli_Chr01*	31359912	T/C	NA	INTERGENIC	NA
Kabuli_wild_31360001	1399933062	*Ca_kabuli_Chr01*	31360001	T/C	NA	INTERGENIC	NA
Kabuli_wild_31360028	1399933063	*Ca_kabuli_Chr01*	31360028	A/G	NA	INTERGENIC	NA
Kabuli_wild_31360032	1399933064	*Ca_kabuli_Chr01*	31360032	A/T	NA	INTERGENIC	NA
Kabuli_wild_31369599	1399933065	*Ca_kabuli_Chr01*	31369599	T/C	Gene_Ca_21855	INTRON	PeptidaseC13,legumain
Kabuli_wild_31393408	1399933066	*Ca_kabuli_Chr01*	31393408	A/T	NA	INTERGENIC	NA
Kabuli_wild_31535190	1399933067	*Ca_kabuli_Chr01*	31535190	A/C	Gene_Ca_21865	INTRON	PeptidaseS49
Kabuli_wild_31590263	1399933068	*Ca_kabuli_Chr01*	31590263	C/G	Gene_Ca_21868	INTRON	Orotidine5'-phosphatedecarboxylasedomain
Kabuli_wild_31590510	1399933069	*Ca_kabuli_Chr01*	31590510	G/A	Gene_Ca_21868	SYNONYMOUS_CODING	Orotidine5'-phosphatedecarboxylasedomain
Kabuli_wild_31590548	1399933070	*Ca_kabuli_Chr01*	31590548	C/T	Gene_Ca_21868	NON_SYNONYMOUS_CODING	Orotidine5'-phosphatedecarboxylasedomain
Kabuli_wild_31638260	1399933071	*Ca_kabuli_Chr01*	31638260	A/G	NA	INTERGENIC	NA
Kabuli_wild_31638267	1399933072	*Ca_kabuli_Chr01*	31638267	G/T	NA	INTERGENIC	NA
Kabuli_wild_31638511	1399933073	*Ca_kabuli_Chr01*	31638511	T/C	NA	INTERGENIC	NA
Kabuli_wild_31838192	1399933074	*Ca_kabuli_Chr01*	31838192	C/T	NA	INTERGENIC	NA
Kabuli_wild_31838198	1399933075	*Ca_kabuli_Chr01*	31838198	C/T	NA	INTERGENIC	NA
Kabuli_wild_31838228	1399933076	*Ca_kabuli_Chr01*	31838228	C/T	NA	INTERGENIC	NA
Kabuli_wild_31838250	1399933077	*Ca_kabuli_Chr01*	31838250	A/C	NA	INTERGENIC	NA
Kabuli_wild_32286466	1399933078	*Ca_kabuli_Chr01*	32286466	A/G	NA	INTERGENIC	NA
Kabuli_wild_32286469	1399933079	*Ca_kabuli_Chr01*	32286469	G/A	NA	INTERGENIC	NA

SNP IDs	NCBI dbSNP accession IDs (NCBI_SS#)	Chromosomes	Physical positions (bp)	SNPs	Gene accession IDs	Sequence components of genome	Putative functions
Kabuli_wild_32286517	1399933080	*Ca_kabuli_Chr01*	32286517	G/A	NA	INTERGENIC	NA
Kabuli_wild_32286518	1399933081	*Ca_kabuli_Chr01*	32286518	C/A	NA	INTERGENIC	NA
Kabuli_wild_32286534	1399933082	*Ca_kabuli_Chr01*	32286534	G/C	NA	INTERGENIC	NA
Kabuli_wild_32286536	1399933083	*Ca_kabuli_Chr01*	32286536	G/A	NA	INTERGENIC	NA
Kabuli_wild_32286538	1399933084	*Ca_kabuli_Chr01*	32286538	C/T	NA	INTERGENIC	NA
Kabuli_wild_32286550	1399933085	*Ca_kabuli_Chr01*	32286550	G/A	NA	INTERGENIC	NA
Kabuli_wild_32286551	1399933086	*Ca_kabuli_Chr01*	32286551	C/T	NA	INTERGENIC	NA
Kabuli_wild_32286558	1399933087	*Ca_kabuli_Chr01*	32286558	A/G	NA	INTERGENIC	NA
Kabuli_wild_32286564	1399933088	*Ca_kabuli_Chr01*	32286564	C/T	NA	INTERGENIC	NA
Kabuli_wild_32286566	1399933089	*Ca_kabuli_Chr01*	32286566	C/A	NA	INTERGENIC	NA
Kabuli_wild_32286567	1399933090	*Ca_kabuli_Chr01*	32286567	C/T	NA	INTERGENIC	NA
Kabuli_wild_32286568	1399933091	*Ca_kabuli_Chr01*	32286568	A/C	NA	INTERGENIC	NA
Kabuli_wild_32286578	1399933092	*Ca_kabuli_Chr01*	32286578	G/A	NA	INTERGENIC	NA
Kabuli_wild_32468053	1399933093	*Ca_kabuli_Chr01*	32468053	A/C	NA	INTERGENIC	NA
Kabuli_wild_32468080	1399933094	*Ca_kabuli_Chr01*	32468080	A/T	NA	INTERGENIC	NA
Kabuli_wild_32468105	1399933095	*Ca_kabuli_Chr01*	32468105	C/G	NA	INTERGENIC	NA
Kabuli_wild_32468116	1399933096	*Ca_kabuli_Chr01*	32468116	A/G	NA	INTERGENIC	NA
Kabuli_wild_32468119	1399933097	*Ca_kabuli_Chr01*	32468119	T/C	NA	INTERGENIC	NA
Kabuli_wild_32871698	1399933098	*Ca_kabuli_Chr01*	32871698	C/T	NA	INTERGENIC	NA
Kabuli_wild_32871716	1399933099	*Ca_kabuli_Chr01*	32871716	T/C	NA	INTERGENIC	NA
Kabuli_wild_32956292	1399933100	*Ca_kabuli_Chr01*	32956292	T/G	NA	INTERGENIC	NA
Kabuli_wild_32956357	1399933101	*Ca_kabuli_Chr01*	32956357	C/G	NA	INTERGENIC	NA
Kabuli_wild_33058743	1399933102	*Ca_kabuli_Chr01*	33058743	T/C	Gene_Ca_19437	INTRON	Argonaute/Dicerprotein,PAZ
Kabuli_wild_33063207	1399933103	*Ca_kabuli_Chr01*	33063207	C/T	Gene_Ca_19437	INTRON	Argonaute/Dicerprotein,PAZ
Kabuli_wild_33095557	1399933104	*Ca_kabuli_Chr01*	33095557	T/C	NA	INTERGENIC	NA
Kabuli_wild_33095577	1399933105	*Ca_kabuli_Chr01*	33095577	C/T	NA	INTERGENIC	NA
Kabuli_wild_33095591	1399933106	*Ca_kabuli_Chr01*	33095591	G/A	NA	INTERGENIC	NA
Kabuli_wild_33095594	1399933107	*Ca_kabuli_Chr01*	33095594	C/A	NA	INTERGENIC	NA
Kabuli_wild_33125824	1399933108	*Ca_kabuli_Chr01*	33125824	C/T	Gene_Ca_19439	INTRON	NA
Kabuli_wild_33125909	1399933109	*Ca_kabuli_Chr01*	33125909	C/T	Gene_Ca_19439	INTRON	NA
Kabuli_wild_33194600	1399933110	*Ca_kabuli_Chr01*	33194600	T/C	Gene_Ca_19440	INTRON	Helicase,C-terminal
Kabuli_wild_33495709	1399933111	*Ca_kabuli_Chr01*	33495709	C/T	NA	INTERGENIC	NA
Kabuli_wild_33804148	1399933112	*Ca_kabuli_Chr01*	33804148	C/T	Gene_Ca_19463	SYNONYMOUS_CODING	NA
Kabuli_wild_33804152	1399933113	*Ca_kabuli_Chr01*	33804152	C/T	Gene_Ca_19463	NON_SYNONYMOUS_CODING	NA
Kabuli_wild_33804153	1399933114	*Ca_kabuli_Chr01*	33804153	C/T	Gene_Ca_19463	SYNONYMOUS_CODING	NA
Kabuli_wild_33804155	1399933115	*Ca_kabuli_Chr01*	33804155	T/C	Gene_Ca_19463	NON_SYNONYMOUS_CODING	NA
Kabuli_wild_33804186	1399933116	*Ca_kabuli_Chr01*	33804186	G/T	Gene_Ca_19463	NON_SYNONYMOUS_CODING	NA
Kabuli_wild_33804189	1399933117	*Ca_kabuli_Chr01*	33804189	C/T	Gene_Ca_19463	NON_SYNONYMOUS_CODING	NA
Kabuli_wild_33804201	1399933118	*Ca_kabuli_Chr01*	33804201	C/T	Gene_Ca_19463	NON_SYNONYMOUS_CODING	NA
Kabuli_wild_33804232	1399933119	*Ca_kabuli_Chr01*	33804232	T/G	Gene_Ca_19463	SYNONYMOUS_CODING	NA
Kabuli_wild_33804242	1399933120	*Ca_kabuli_Chr01*	33804242	T/G	Gene_Ca_19463	NON_SYNONYMOUS_CODING	NA
Kabuli_wild_33804246	1399933121	*Ca_kabuli_Chr01*	33804246	C/G	Gene_Ca_19463	NON_SYNONYMOUS_CODING	NA
Kabuli_wild_33804247	1399933122	*Ca_kabuli_Chr01*	33804247	A/G	Gene_Ca_19463	SYNONYMOUS_CODING	NA
Kabuli_wild_33804248	1399933123	*Ca_kabuli_Chr01*	33804248	G/A	Gene_Ca_19463	NON_SYNONYMOUS_CODING	NA

SNP IDs	NCBI dbSNP accession IDs (NCBI_SS#)	Chromosomes	Physical positions (bp)	SNPs	Gene accession IDs	Sequence components of genome	Putative functions
Kabuli_wild_33804261	1399933124	*Ca_kabuli_Chr01*	33804261	C/T	Gene_Ca_19463	NON_SYNONYMOUS_CODING	NA
Kabuli_wild_33804262	1399933125	*Ca_kabuli_Chr01*	33804262	G/C	Gene_Ca_19463	SYNONYMOUS_CODING	NA
Kabuli_wild_33804263	1399933126	*Ca_kabuli_Chr01*	33804263	G/A	Gene_Ca_19463	NON_SYNONYMOUS_CODING	NA
Kabuli_wild_33804274	1399933127	*Ca_kabuli_Chr01*	33804274	T/C	Gene_Ca_19463	SYNONYMOUS_CODING	NA
Kabuli_wild_34161921	1399933128	*Ca_kabuli_Chr01*	34161921	G/A	NA	INTERGENIC	NA
Kabuli_wild_34193834	1399933129	*Ca_kabuli_Chr01*	34193834	A/G	NA	INTERGENIC	NA
Kabuli_wild_34261155	1399933130	*Ca_kabuli_Chr01*	34261155	C/A	Gene_Ca_25475	INTRON	Glycosyltransferase,family48
Kabuli_wild_34261158	1399933131	*Ca_kabuli_Chr01*	34261158	C/A	Gene_Ca_25475	INTRON	Glycosyltransferase,family48
Kabuli_wild_34261166	1399933132	*Ca_kabuli_Chr01*	34261166	C/A	Gene_Ca_25475	INTRON	Glycosyltransferase,family48
Kabuli_wild_34272001	1399933133	*Ca_kabuli_Chr01*	34272001	C/G	NA	INTERGENIC	NA
Kabuli_wild_34272024	1399933134	*Ca_kabuli_Chr01*	34272024	A/G	NA	INTERGENIC	NA
Kabuli_wild_34272025	1399933135	*Ca_kabuli_Chr01*	34272025	A/G	NA	INTERGENIC	NA
Kabuli_wild_34311145	1399933136	*Ca_kabuli_Chr01*	34311145	T/G	Gene_Ca_25478	SYNONYMOUS_CODING	GTP-bindingprotein,HSR1-related
Kabuli_wild_34437096	1399933137	*Ca_kabuli_Chr01*	34437096	G/A	NA	INTERGENIC	NA
Kabuli_wild_34437121	1399933138	*Ca_kabuli_Chr01*	34437121	G/T	NA	INTERGENIC	NA
Kabuli_wild_34437144	1399933139	*Ca_kabuli_Chr01*	34437144	G/A	NA	INTERGENIC	NA
Kabuli_wild_34475825	1399933140	*Ca_kabuli_Chr01*	34475825	A/C	Gene_Ca_27319	NON_SYNONYMOUS_CODING	NA
Kabuli_wild_34524314	1399933141	*Ca_kabuli_Chr01*	34524314	T/G	Gene_Ca_27321	INTRON	Methyltransferasetype11
Kabuli_wild_34524315	1399933142	*Ca_kabuli_Chr01*	34524315	G/T	Gene_Ca_27321	INTRON	Methyltransferasetype11
Kabuli_wild_34727251	1399933143	*Ca_kabuli_Chr01*	34727251	T/G	Gene_Ca_21419	SYNONYMOUS_CODING	Zinc finger,CCCH-type
Kabuli_wild_34728328	1399933144	*Ca_kabuli_Chr01*	34728328	T/C	Gene_Ca_21419	SYNONYMOUS_CODING	Zinc finger,CCCH-type
Kabuli_wild_34772813	1399933145	*Ca_kabuli_Chr01*	34772813	T/A	Gene_Ca_21416	NON_SYNONYMOUS_CODING	NA
Kabuli_wild_34866709	1399933146	*Ca_kabuli_Chr01*	34866709	C/A	NA	INTERGENIC	NA
Kabuli_wild_34948934	1399933147	*Ca_kabuli_Chr01*	34948934	A/G	Gene_Ca_21408	INTRON	Proteasome,alpha-subunit,conservedsite
Kabuli_wild_34969122	1399933148	*Ca_kabuli_Chr01*	34969122	T/C	NA	INTERGENIC	NA
Kabuli_wild_35041241	1399933149	*Ca_kabuli_Chr01*	35041241	T/G	NA	INTERGENIC	NA
Kabuli_wild_35041324	1399933150	*Ca_kabuli_Chr01*	35041324	G/T	NA	INTERGENIC	NA
Kabuli_wild_35260363	1399933151	*Ca_kabuli_Chr01*	35260363	G/A	NA	INTERGENIC	NA
Kabuli_wild_35260397	1399933152	*Ca_kabuli_Chr01*	35260397	T/A	NA	INTERGENIC	NA
Kabuli_wild_35313277	1399933153	*Ca_kabuli_Chr01*	35313277	A/G	NA	INTERGENIC	NA
Kabuli_wild_35313309	1399933154	*Ca_kabuli_Chr01*	35313309	G/A	NA	INTERGENIC	NA
Kabuli_wild_35315327	1399933155	*Ca_kabuli_Chr01*	35315327	T/A	NA	INTERGENIC	NA
Kabuli_wild_35315347	1399933156	*Ca_kabuli_Chr01*	35315347	C/T	NA	INTERGENIC	NA
Kabuli_wild_35315379	1399933157	*Ca_kabuli_Chr01*	35315379	A/T	NA	INTERGENIC	NA
Kabuli_wild_35315381	1399933158	*Ca_kabuli_Chr01*	35315381	T/A	NA	INTERGENIC	NA
Kabuli_wild_35315419	1399933159	*Ca_kabuli_Chr01*	35315419	C/T	NA	INTERGENIC	NA
Kabuli_wild_35315436	1399933160	*Ca_kabuli_Chr01*	35315436	G/A	NA	INTERGENIC	NA
Kabuli_wild_35315454	1399933161	*Ca_kabuli_Chr01*	35315454	G/A	NA	INTERGENIC	NA
Kabuli_wild_35315467	1399933162	*Ca_kabuli_Chr01*	35315467	C/A	NA	INTERGENIC	NA
Kabuli_wild_35315476	1399933163	*Ca_kabuli_Chr01*	35315476	G/A	NA	INTERGENIC	NA
Kabuli_wild_35315483	1399933164	*Ca_kabuli_Chr01*	35315483	G/A	NA	INTERGENIC	NA
Kabuli_wild_35315488	1399933165	*Ca_kabuli_Chr01*	35315488	C/T	NA	INTERGENIC	NA
Kabuli_wild_35315496	1399933166	*Ca_kabuli_Chr01*	35315496	T/A	NA	INTERGENIC	NA
Kabuli_wild_35315504	1399933167	*Ca_kabuli_Chr01*	35315504	T/G	NA	INTERGENIC	NA

SNP IDs	NCBI dbSNP accession IDs (NCBI_SS#)	Chromosomes	Physical positions (bp)	SNPs	Gene accession IDs	Sequence components of genome	Putative functions
Kabuli_wild_35315505	1399933168	*Ca_kabuli_Chr01*	35315505	G/T	NA	INTERGENIC	NA
Kabuli_wild_35315507	1399933169	*Ca_kabuli_Chr01*	35315507	G/T	NA	INTERGENIC	NA
Kabuli_wild_35315509	1399933170	*Ca_kabuli_Chr01*	35315509	C/T	NA	INTERGENIC	NA
Kabuli_wild_35379871	1399933171	*Ca_kabuli_Chr01*	35379871	G/C	NA	INTERGENIC	NA
Kabuli_wild_35708841	1399933172	*Ca_kabuli_Chr01*	35708841	C/T	Gene_Ca_24110	SYNONYMOUS_CODING	Mediatorcomplex,subunitMed10
Kabuli_wild_35790429	1399933173	*Ca_kabuli_Chr01*	35790429	T/C	NA	INTERGENIC	NA
Kabuli_wild_35838114	1399933174	*Ca_kabuli_Chr01*	35838114	C/T	NA	INTERGENIC	NA
Kabuli_wild_35838121	1399933175	*Ca_kabuli_Chr01*	35838121	T/C	NA	INTERGENIC	NA
Kabuli_wild_36105280	1399933176	*Ca_kabuli_Chr01*	36105280	G/A	NA	INTERGENIC	NA
Kabuli_wild_36126090	1399933177	*Ca_kabuli_Chr01*	36126090	C/T	NA	INTERGENIC	NA
Kabuli_wild_36296608	1399933178	*Ca_kabuli_Chr01*	36296608	G/A	NA	INTERGENIC	NA
Kabuli_wild_36296637	1399933179	*Ca_kabuli_Chr01*	36296637	T/A	NA	INTERGENIC	NA
Kabuli_wild_36296641	1399933180	*Ca_kabuli_Chr01*	36296641	G/A	NA	INTERGENIC	NA
Kabuli_wild_36296692	1399933181	*Ca_kabuli_Chr01*	36296692	G/A	NA	INTERGENIC	NA
Kabuli_wild_36328960	1399933182	*Ca_kabuli_Chr01*	36328960	C/T	Gene_Ca_21682	DOWNSTREAM	NA
Kabuli_wild_36528365	1399933183	*Ca_kabuli_Chr01*	36528365	T/C	NA	INTERGENIC	NA
Kabuli_wild_36794727	1399933184	*Ca_kabuli_Chr01*	36794727	G/A	Gene_Ca_21700	SYNONYMOUS_CODING	Peptidase C48, SUMO/Sentrin/Ubl1
Kabuli_wild_36794729	1399933185	*Ca_kabuli_Chr01*	36794729	C/T	Gene_Ca_21700	NON_SYNONYMOUS_CODING	Peptidase C48, SUMO/Sentrin/Ubl1
Kabuli_wild_36794731	1399933186	*Ca_kabuli_Chr01*	36794731	A/G	Gene_Ca_21700	NON_SYNONYMOUS_CODING	Peptidase C48, SUMO/Sentrin/Ubl1
Kabuli_wild_36794779	1399933187	*Ca_kabuli_Chr01*	36794779	C/T	Gene_Ca_21700	SYNONYMOUS_CODING	Peptidase C48, SUMO/Sentrin/Ubl1
Kabuli_wild_36794797	1399933188	*Ca_kabuli_Chr01*	36794797	G/A	Gene_Ca_21700	NON_SYNONYMOUS_CODING	Peptidase C48, SUMO/Sentrin/Ubl1
Kabuli_wild_36794800	1399933189	*Ca_kabuli_Chr01*	36794800	G/T	Gene_Ca_21700	NON_SYNONYMOUS_CODING	Peptidase C48, SUMO/Sentrin/Ubl1
Kabuli_wild_36794806	1399933190	*Ca_kabuli_Chr01*	36794806	G/T	Gene_Ca_21700	NON_SYNONYMOUS_CODING	Peptidase C48, SUMO/Sentrin/Ubl1
Kabuli_wild_36794810	1399933191	*Ca_kabuli_Chr01*	36794810	C/A	Gene_Ca_21700	NON_SYNONYMOUS_CODING	Peptidase C48, SUMO/Sentrin/Ubl1
Kabuli_wild_36794813	1399933192	*Ca_kabuli_Chr01*	36794813	C/T	Gene_Ca_21700	SYNONYMOUS_CODING	Peptidase C48, SUMO/Sentrin/Ubl1
Kabuli_wild_36794815	1399933193	*Ca_kabuli_Chr01*	36794815	G/A	Gene_Ca_21700	NON_SYNONYMOUS_CODING	Peptidase C48, SUMO/Sentrin/Ubl1
Kabuli_wild_36794826	1399933194	*Ca_kabuli_Chr01*	36794826	A/G	Gene_Ca_21700	SYNONYMOUS_CODING	Peptidase C48, SUMO/Sentrin/Ubl1
Kabuli_wild_36794832	1399933195	*Ca_kabuli_Chr01*	36794832	T/A	Gene_Ca_21700	NON_SYNONYMOUS_CODING	Peptidase C48, SUMO/Sentrin/Ubl1
Kabuli_wild_36794839	1399933196	*Ca_kabuli_Chr01*	36794839	G/C	Gene_Ca_21700	NON_SYNONYMOUS_CODING	Peptidase C48, SUMO/Sentrin/Ubl1
Kabuli_wild_36794849	1399933197	*Ca_kabuli_Chr01*	36794849	G/C	Gene_Ca_21700	NON_SYNONYMOUS_CODING	Peptidase C48, SUMO/Sentrin/Ubl1
Kabuli_wild_36794850	1399933198	*Ca_kabuli_Chr01*	36794850	T/A	Gene_Ca_21700	NON_SYNONYMOUS_CODING	Peptidase C48, SUMO/Sentrin/Ubl1
Kabuli_wild_36794855	1399933199	*Ca_kabuli_Chr01*	36794855	C/G	Gene_Ca_21700	NON_SYNONYMOUS_CODING	Peptidase C48, SUMO/Sentrin/Ubl1
Kabuli_wild_36794856	1399933200	*Ca_kabuli_Chr01*	36794856	A/G	Gene_Ca_21700	SYNONYMOUS_CODING	Peptidase C48, SUMO/Sentrin/Ubl1
Kabuli_wild_36794857	1399933201	*Ca_kabuli_Chr01*	36794857	G/A	Gene_Ca_21700	NON_SYNONYMOUS_CODING	Peptidase C48, SUMO/Sentrin/Ubl1
Kabuli_wild_36794865	1399933202	*Ca_kabuli_Chr01*	36794865	A/T	Gene_Ca_21700	SYNONYMOUS_CODING	Peptidase C48, SUMO/Sentrin/Ubl1
Kabuli_wild_36794866	1399933203	*Ca_kabuli_Chr01*	36794866	C/T	Gene_Ca_21700	NON_SYNONYMOUS_CODING	Peptidase C48, SUMO/Sentrin/Ubl1
Kabuli_wild_36794870	1399933204	*Ca_kabuli_Chr01*	36794870	C/T	Gene_Ca_21700	NON_SYNONYMOUS_CODING	Peptidase C48, SUMO/Sentrin/Ubl1
Kabuli_wild_36794873	1399933205	*Ca_kabuli_Chr01*	36794873	G/A	Gene_Ca_21700	NON_SYNONYMOUS_CODING	Peptidase C48, SUMO/Sentrin/Ubl1
Kabuli_wild_36794883	1399933206	*Ca_kabuli_Chr01*	36794883	T/C	Gene_Ca_21700	SYNONYMOUS_CODING	Peptidase C48, SUMO/Sentrin/Ubl1
Kabuli_wild_36794887	1399933207	*Ca_kabuli_Chr01*	36794887	G/A	Gene_Ca_21700	NON_SYNONYMOUS_CODING	Peptidase C48, SUMO/Sentrin/Ubl1
Kabuli_wild_36794891	1399933208	*Ca_kabuli_Chr01*	36794891	C/T	Gene_Ca_21700	NON_SYNONYMOUS_CODING	Peptidase C48, SUMO/Sentrin/Ubl1
Kabuli_wild_36794906	1399933209	*Ca_kabuli_Chr01*	36794906	A/T	Gene_Ca_21700	NON_SYNONYMOUS_CODING	Peptidase C48, SUMO/Sentrin/Ubl1
Kabuli_wild_36794908	1399933210	*Ca_kabuli_Chr01*	36794908	T/C	Gene_Ca_21700	NON_SYNONYMOUS_CODING	Peptidase C48, SUMO/Sentrin/Ubl1
Kabuli_wild_36794912	1399933211	*Ca_kabuli_Chr01*	36794912	G/T	Gene_Ca_21700	NON_SYNONYMOUS_CODING	Peptidase C48, SUMO/Sentrin/Ubl1

SNP IDs	NCBI dbSNP accession IDs (NCBI_SS#)	Chromosomes	Physical positions (bp)	SNPs	Gene accession IDs	Sequence components of genome	Putative functions
Kabuli_wild_36794917	1399933212	*Ca_kabuli_Chr01*	36794917	T/C	Gene_Ca_21700	NON_SYNONYMOUS_CODING	Peptidase C48, SUMO/Sentrin/Ubl1
Kabuli_wild_36892535	1399933213	*Ca_kabuli_Chr01*	36892535	G/A	Gene_Ca_27231	NON_SYNONYMOUS_CODING	Peptidase C48, SUMO/Sentrin/Ubl1
Kabuli_wild_36892550	1399933214	*Ca_kabuli_Chr01*	36892550	C/T	Gene_Ca_27231	SYNONYMOUS_CODING	Peptidase C48, SUMO/Sentrin/Ubl1
Kabuli_wild_36892558	1399933215	*Ca_kabuli_Chr01*	36892558	T/C	Gene_Ca_27231	SYNONYMOUS_CODING	Peptidase C48, SUMO/Sentrin/Ubl1
Kabuli_wild_36892592	1399933216	*Ca_kabuli_Chr01*	36892592	C/T	Gene_Ca_27231	SYNONYMOUS_CODING	Peptidase C48, SUMO/Sentrin/Ubl1
Kabuli_wild_36892599	1399933217	*Ca_kabuli_Chr01*	36892599	G/A	Gene_Ca_27231	NON_SYNONYMOUS_CODING	Peptidase C48, SUMO/Sentrin/Ubl1
Kabuli_wild_36892602	1399933218	*Ca_kabuli_Chr01*	36892602	G/A	Gene_Ca_27231	NON_SYNONYMOUS_CODING	Peptidase C48, SUMO/Sentrin/Ubl1
Kabuli_wild_36892608	1399933219	*Ca_kabuli_Chr01*	36892608	C/T	Gene_Ca_27231	NON_SYNONYMOUS_CODING	Peptidase C48, SUMO/Sentrin/Ubl1
Kabuli_wild_36892617	1399933220	*Ca_kabuli_Chr01*	36892617	C/T	Gene_Ca_27231	NON_SYNONYMOUS_CODING	Peptidase C48, SUMO/Sentrin/Ubl1
Kabuli_wild_37026302	1399933221	*Ca_kabuli_Chr01*	37026302	C/T	NA	INTERGENIC	NA
Kabuli_wild_37607868	1399933222	*Ca_kabuli_Chr01*	37607868	A/T	NA	INTERGENIC	NA
Kabuli_wild_37624771	1399933223	*Ca_kabuli_Chr01*	37624771	C/T	NA	INTERGENIC	NA
Kabuli_wild_37624794	1399933224	*Ca_kabuli_Chr01*	37624794	T/C	NA	INTERGENIC	NA
Kabuli_wild_37624807	1399933225	*Ca_kabuli_Chr01*	37624807	C/T	NA	INTERGENIC	NA
Kabuli_wild_37624823	1399933226	*Ca_kabuli_Chr01*	37624823	G/A	NA	INTERGENIC	NA
Kabuli_wild_37642546	1399933227	*Ca_kabuli_Chr01*	37642546	G/A	NA	INTERGENIC	NA
Kabuli_wild_37690416	1399933228	*Ca_kabuli_Chr01*	37690416	G/A	Gene_Ca_23533	INTRON	Myb,DNA-binding
Kabuli_wild_37690465	1399933229	*Ca_kabuli_Chr01*	37690465	T/C	Gene_Ca_23533	INTRON	Myb,DNA-binding
Kabuli_wild_37690497	1399933230	*Ca_kabuli_Chr01*	37690497	A/T	Gene_Ca_23533	INTRON	Myb,DNA-binding
Kabuli_wild_37690498	1399933231	*Ca_kabuli_Chr01*	37690498	C/A	Gene_Ca_23533	INTRON	Myb,DNA-binding
Kabuli_wild_37699036	1399933232	*Ca_kabuli_Chr01*	37699036	T/C	NA	INTERGENIC	NA
Kabuli_wild_37699039	1399933233	*Ca_kabuli_Chr01*	37699039	T/G	NA	INTERGENIC	NA
Kabuli_wild_37722095	1399933234	*Ca_kabuli_Chr01*	37722095	G/A	Gene_Ca_23534	SYNONYMOUS_CODING	Protein of unknown function DUF620
Kabuli_wild_37722143	1399933235	*Ca_kabuli_Chr01*	37722143	G/C	Gene_Ca_23534	SYNONYMOUS_CODING	Protein of unknown function DUF620
Kabuli_wild_37768179	1399933236	*Ca_kabuli_Chr01*	37768179	T/A	NA	INTERGENIC	NA
Kabuli_wild_37768299	1399933237	*Ca_kabuli_Chr01*	37768299	A/G	NA	INTERGENIC	NA
Kabuli_wild_37768333	1399933238	*Ca_kabuli_Chr01*	37768333	C/T	NA	INTERGENIC	NA
Kabuli_wild_37787046	1399933239	*Ca_kabuli_Chr01*	37787046	C/A	NA	INTERGENIC	NA
Kabuli_wild_37787052	1399933240	*Ca_kabuli_Chr01*	37787052	A/G	NA	INTERGENIC	NA
Kabuli_wild_37869136	1399933241	*Ca_kabuli_Chr01*	37869136	G/T	Gene_Ca_23539	INTRON	Zinc finger,PHD-type
Kabuli_wild_37880995	1399933242	*Ca_kabuli_Chr01*	37880995	T/C	Gene_Ca_23539	INTRON	Zinc finger,PHD-type
Kabuli_wild_37892088	1399933243	*Ca_kabuli_Chr01*	37892088	A/C	Gene_Ca_23539	DOWNSTREAM	Zinc finger,PHD-type
Kabuli_wild_37892089	1399933244	*Ca_kabuli_Chr01*	37892089	C/T	Gene_Ca_23539	DOWNSTREAM	Zinc finger,PHD-type
Kabuli_wild_37892139	1399933245	*Ca_kabuli_Chr01*	37892139	A/T	Gene_Ca_23539	DOWNSTREAM	Zinc finger,PHD-type
Kabuli_wild_38023935	1399933246	*Ca_kabuli_Chr01*	38023935	G/A	Gene_Ca_27443	INTRON	Zinc finger,GRF-type
Kabuli_wild_38023940	1399933247	*Ca_kabuli_Chr01*	38023940	C/T	Gene_Ca_27443	INTRON	Zinc finger,GRF-type
Kabuli_wild_38023969	1399933248	*Ca_kabuli_Chr01*	38023969	A/G	Gene_Ca_27443	INTRON	Zinc finger,GRF-type
Kabuli_wild_38023972	1399933249	*Ca_kabuli_Chr01*	38023972	C/T	Gene_Ca_27443	INTRON	Zinc finger,GRF-type
Kabuli_wild_38023974	1399933250	*Ca_kabuli_Chr01*	38023974	G/A	Gene_Ca_27443	INTRON	Zinc finger,GRF-type
Kabuli_wild_38024016	1399933251	*Ca_kabuli_Chr01*	38024016	A/C	Gene_Ca_27443	INTRON	Zinc finger,GRF-type
Kabuli_wild_38025854	1399933252	*Ca_kabuli_Chr01*	38025854	G/A	Gene_Ca_27443	SYNONYMOUS_CODING	Zinc finger,GRF-type
Kabuli_wild_38049721	1399933253	*Ca_kabuli_Chr01*	38049721	C/T	Gene_Ca_27444	NON_SYNONYMOUS_CODING	Cation efflux protein
Kabuli_wild_38226909	1399933254	*Ca_kabuli_Chr01*	38226909	G/A	NA	INTERGENIC	NA
Kabuli_wild_38782963	1399933255	*Ca_kabuli_Chr01*	38782963	C/A	NA	INTERGENIC	NA

SNP IDs	NCBI dbSNP accession IDs (NCBI_SS#)	Chromosomes	Physical positions (bp)	SNPs	Gene accession IDs	Sequence components of genome	Putative functions
Kabuli_wild_38877670	1399933256	*Ca_kabuli_Chr01*	38877670	T/C	NA	INTERGENIC	NA
Kabuli_wild_39107076	1399933257	*Ca_kabuli_Chr01*	39107076	A/C	NA	INTERGENIC	NA
Kabuli_wild_39107216	1399933258	*Ca_kabuli_Chr01*	39107216	A/T	NA	INTERGENIC	NA
Kabuli_wild_39436182	1399933259	*Ca_kabuli_Chr01*	39436182	T/C	Gene_Ca_13931	SYNONYMOUS_CODING	IQ motif,EF-handbinding site
Kabuli_wild_39436291	1399933260	*Ca_kabuli_Chr01*	39436291	T/C	Gene_Ca_13931	INTRON	IQ motif,EF-handbinding site
Kabuli_wild_39438070	1399933261	*Ca_kabuli_Chr01*	39438070	A/C	Gene_Ca_13931	NON_SYNONYMOUS_CODING	IQ motif,EF-handbinding site
Kabuli_wild_39587402	1399933262	*Ca_kabuli_Chr01*	39587402	A/G	NA	INTERGENIC	NA
Kabuli_wild_39599655	1399933263	*Ca_kabuli_Chr01*	39599655	C/T	NA	INTERGENIC	NA
Kabuli_wild_39601982	1399933264	*Ca_kabuli_Chr01*	39601982	T/G	NA	INTERGENIC	NA
Kabuli_wild_39602021	1399933265	*Ca_kabuli_Chr01*	39602021	T/C	NA	INTERGENIC	NA
Kabuli_wild_39602029	1399933266	*Ca_kabuli_Chr01*	39602029	G/T	NA	INTERGENIC	NA
Kabuli_wild_39616139	1399933267	*Ca_kabuli_Chr01*	39616139	A/C	Gene_Ca_13924	SYNONYMOUS_CODING	CTPsynthase,N-terminal
Kabuli_wild_39663432	1399933268	*Ca_kabuli_Chr01*	39663432	C/G	Gene_Ca_13922	INTRON	ABC transporter-like
Kabuli_wild_39712581	1399933269	*Ca_kabuli_Chr01*	39712581	G/A	Gene_Ca_13918	DOWNSTREAM	NA
Kabuli_wild_39746444	1399933270	*Ca_kabuli_Chr01*	39746444	C/G	NA	INTERGENIC	NA
Kabuli_wild_39904837	1399933271	*Ca_kabuli_Chr01*	39904837	G/C	NA	INTERGENIC	NA
Kabuli_wild_40199841	1399933272	*Ca_kabuli_Chr01*	40199841	T/A	NA	INTERGENIC	NA
Kabuli_wild_40199917	1399933273	*Ca_kabuli_Chr01*	40199917	T/C	NA	INTERGENIC	NA
Kabuli_wild_40199920	1399933274	*Ca_kabuli_Chr01*	40199920	G/C	NA	INTERGENIC	NA
Kabuli_wild_40199922	1399933275	*Ca_kabuli_Chr01*	40199922	C/A	NA	INTERGENIC	NA
Kabuli_wild_40199972	1399933276	*Ca_kabuli_Chr01*	40199972	T/C	NA	INTERGENIC	NA
Kabuli_wild_40224804	1399933277	*Ca_kabuli_Chr01*	40224804	A/G	NA	INTERGENIC	NA
Kabuli_wild_40224834	1399933278	*Ca_kabuli_Chr01*	40224834	C/G	NA	INTERGENIC	NA
Kabuli_wild_40224835	1399933279	*Ca_kabuli_Chr01*	40224835	T/A	NA	INTERGENIC	NA
Kabuli_wild_40343216	1399933280	*Ca_kabuli_Chr01*	40343216	G/T	Gene_Ca_13893	INTRON	Tetratricopeptide,MLP1/MLP2-like
Kabuli_wild_40346280	1399933281	*Ca_kabuli_Chr01*	40346280	A/C	Gene_Ca_13893	INTRON	Tetratricopeptide,MLP1/MLP2-like
Kabuli_wild_40358693	1399933282	*Ca_kabuli_Chr01*	40358693	C/T	Gene_Ca_13893	INTRON	Tetratricopeptide,MLP1/MLP2-like
Kabuli_wild_40358747	1399933283	*Ca_kabuli_Chr01*	40358747	T/G	Gene_Ca_13893	INTRON	Tetratricopeptide,MLP1/MLP2-like
Kabuli_wild_40366858	1399933284	*Ca_kabuli_Chr01*	40366858	T/C	Gene_Ca_13893	INTRON	Tetratricopeptide,MLP1/MLP2-like
Kabuli_wild_40366862	1399933285	*Ca_kabuli_Chr01*	40366862	G/A	Gene_Ca_13893	INTRON	Tetratricopeptide,MLP1/MLP2-like
Kabuli_wild_40366866	1399933286	*Ca_kabuli_Chr01*	40366866	G/C	Gene_Ca_13893	INTRON	Tetratricopeptide,MLP1/MLP2-like
Kabuli_wild_40367940	1399933287	*Ca_kabuli_Chr01*	40367940	G/A	Gene_Ca_13893	INTRON	Tetratricopeptide,MLP1/MLP2-like
Kabuli_wild_40499629	1399933288	*Ca_kabuli_Chr01*	40499629	C/T	Gene_Ca_18443	NON_SYNONYMOUS_CODING	CWC16 protein
Kabuli_wild_41008946	1399933289	*Ca_kabuli_Chr01*	41008946	C/A	Gene_Ca_18469	INTRON	X8
Kabuli_wild_41029732	1399933290	*Ca_kabuli_Chr01*	41029732	C/T	NA	INTERGENIC	NA
Kabuli_wild_41169218	1399933291	*Ca_kabuli_Chr01*	41169218	G/T	NA	INTERGENIC	NA
Kabuli_wild_41185512	1399933292	*Ca_kabuli_Chr01*	41185512	T/G	NA	INTERGENIC	NA
Kabuli_wild_41380061	1399933293	*Ca_kabuli_Chr01*	41380061	G/T	Gene_Ca_23111	SYNONYMOUS_CODING	GlutathioneS-transferase,N-terminal
Kabuli_wild_41451912	1399933294	*Ca_kabuli_Chr01*	41451912	G/T	Gene_Ca_23119	SYNONYMOUS_CODING	Peptidase C48, SUMO/Sentrin/Ubl1
Kabuli_wild_41451929	1399933295	*Ca_kabuli_Chr01*	41451929	C/T	Gene_Ca_23119	NON_SYNONYMOUS_CODING	Peptidase C48, SUMO/Sentrin/Ubl1
Kabuli_wild_41451974	1399933296	*Ca_kabuli_Chr01*	41451974	C/T	Gene_Ca_23119	NON_SYNONYMOUS_CODING	Peptidase C48, SUMO/Sentrin/Ubl1
Kabuli_wild_41564809	1399933297	*Ca_kabuli_Chr01*	41564809	T/C	NA	INTERGENIC	NA
Kabuli_wild_41566032	1399933298	*Ca_kabuli_Chr01*	41566032	T/A	Gene_Ca_25795	UPSTREAM	BolAprotein
Kabuli_wild_41571912	1399933299	*Ca_kabuli_Chr01*	41571912	T/C	NA	INTERGENIC	NA

SNP IDs	NCBI dbSNP accession IDs (NCBI_SS#)	Chromosomes	Physical positions (bp)	SNPs	Gene accession IDs	Sequence components of genome	Putative functions
Kabuli_wild_41730109	1399933300	*Ca_kabuli_Chr01*	41730109	T/A	Gene_Ca_22515	NON_SYNONYMOUS_CODING	RNA-bindingS4
Kabuli_wild_41730114	1399933301	*Ca_kabuli_Chr01*	41730114	A/C	Gene_Ca_22515	NON_SYNONYMOUS_CODING	RNA-bindingS4
Kabuli_wild_41782890	1399933302	*Ca_kabuli_Chr01*	41782890	A/G	Gene_Ca_22517	NON_SYNONYMOUS_CODING	ClassIIaldolase/adducin,N-terminal
Kabuli_wild_41827388	1399933303	*Ca_kabuli_Chr01*	41827388	T/C	NA	INTERGENIC	NA
Kabuli_wild_41867035	1399933304	*Ca_kabuli_Chr01*	41867035	C/T	NA	INTERGENIC	NA
Kabuli_wild_41897211	1399933305	*Ca_kabuli_Chr01*	41897211	C/A	Gene_Ca_22528	INTRON	NA
Kabuli_wild_41897212	1399933306	*Ca_kabuli_Chr01*	41897212	G/T	Gene_Ca_22528	INTRON	NA
Kabuli_wild_41897216	1399933307	*Ca_kabuli_Chr01*	41897216	C/A	Gene_Ca_22528	INTRON	NA
Kabuli_wild_41897304	1399933308	*Ca_kabuli_Chr01*	41897304	A/C	Gene_Ca_22528	INTRON	NA
Kabuli_wild_41946005	1399933309	*Ca_kabuli_Chr01*	41946005	A/G	NA	INTERGENIC	NA
Kabuli_wild_42015396	1399933310	*Ca_kabuli_Chr01*	42015396	G/T	NA	INTERGENIC	NA
Kabuli_wild_42160998	1399933311	*Ca_kabuli_Chr01*	42160998	C/T	NA	INTERGENIC	NA
Kabuli_wild_42161200	1399933312	*Ca_kabuli_Chr01*	42161200	A/C	NA	INTERGENIC	NA
Kabuli_wild_42161207	1399933313	*Ca_kabuli_Chr01*	42161207	C/T	NA	INTERGENIC	NA
Kabuli_wild_42161225	1399933314	*Ca_kabuli_Chr01*	42161225	C/T	NA	INTERGENIC	NA
Kabuli_wild_42161231	1399933315	*Ca_kabuli_Chr01*	42161231	C/T	NA	INTERGENIC	NA
Kabuli_wild_42439646	1399933316	*Ca_kabuli_Chr01*	42439646	C/T	Gene_Ca_22662	DOWNSTREAM	NA
Kabuli_wild_42439650	1399933317	*Ca_kabuli_Chr01*	42439650	C/A	Gene_Ca_22662	DOWNSTREAM	NA
Kabuli_wild_42439722	1399933318	*Ca_kabuli_Chr01*	42439722	T/G	Gene_Ca_22662	DOWNSTREAM	NA
Kabuli_wild_42439795	1399933319	*Ca_kabuli_Chr01*	42439795	A/C	Gene_Ca_22662	DOWNSTREAM	NA
Kabuli_wild_42452939	1399933320	*Ca_kabuli_Chr01*	42452939	G/T	Gene_Ca_13620	NON_SYNONYMOUS_CODING	Transcription factor CBF/NF-Y/archaeal histone
Kabuli_wild_42551585	1399933321	*Ca_kabuli_Chr01*	42551585	C/A	NA	INTERGENIC	NA
Kabuli_wild_42552658	1399933322	*Ca_kabuli_Chr01*	42552658	A/C	Gene_Ca_13613	DOWNSTREAM	PeptidaseA1
Kabuli_wild_42552710	1399933323	*Ca_kabuli_Chr01*	42552710	A/C	Gene_Ca_13613	DOWNSTREAM	PeptidaseA1
Kabuli_wild_42605239	1399933324	*Ca_kabuli_Chr01*	42605239	T/G	NA	INTERGENIC	NA
Kabuli_wild_42613060	1399933325	*Ca_kabuli_Chr01*	42613060	T/G	NA	INTERGENIC	NA
Kabuli_wild_43048137	1399933326	*Ca_kabuli_Chr01*	43048137	G/T	NA	INTERGENIC	NA
Kabuli_wild_43053424	1399933327	*Ca_kabuli_Chr01*	43053424	G/C	NA	INTERGENIC	NA
Kabuli_wild_43053583	1399933328	*Ca_kabuli_Chr01*	43053583	A/C	NA	INTERGENIC	NA
Kabuli_wild_43273910	1399933329	*Ca_kabuli_Chr01*	43273910	G/T	NA	INTERGENIC	NA
Kabuli_wild_43273922	1399933330	*Ca_kabuli_Chr01*	43273922	T/C	NA	INTERGENIC	NA
Kabuli_wild_43273925	1399933331	*Ca_kabuli_Chr01*	43273925	A/G	NA	INTERGENIC	NA
Kabuli_wild_43273931	1399933332	*Ca_kabuli_Chr01*	43273931	G/A	NA	INTERGENIC	NA
Kabuli_wild_43273941	1399933333	*Ca_kabuli_Chr01*	43273941	C/G	NA	INTERGENIC	NA
Kabuli_wild_43273943	1399933334	*Ca_kabuli_Chr01*	43273943	A/G	NA	INTERGENIC	NA
Kabuli_wild_43273961	1399933335	*Ca_kabuli_Chr01*	43273961	C/T	NA	INTERGENIC	NA
Kabuli_wild_43273962	1399933336	*Ca_kabuli_Chr01*	43273962	A/G	NA	INTERGENIC	NA
Kabuli_wild_43273968	1399933337	*Ca_kabuli_Chr01*	43273968	T/G	NA	INTERGENIC	NA
Kabuli_wild_43273972	1399933338	*Ca_kabuli_Chr01*	43273972	C/T	NA	INTERGENIC	NA
Kabuli_wild_43273981	1399933339	*Ca_kabuli_Chr01*	43273981	G/T	NA	INTERGENIC	NA
Kabuli_wild_43273985	1399933340	*Ca_kabuli_Chr01*	43273985	G/A	NA	INTERGENIC	NA
Kabuli_wild_43386968	1399933341	*Ca_kabuli_Chr01*	43386968	G/A	Gene_Ca_13577	NON_SYNONYMOUS_CODING	Cyclin
Kabuli_wild_43725269	1399933342	*Ca_kabuli_Chr01*	43725269	T/G	Gene_Ca_13554	SYNONYMOUS_CODING	NA
Kabuli_wild_43727884	1399933343	*Ca_kabuli_Chr01*	43727884	G/A	Gene_Ca_13554	NON_SYNONYMOUS_CODING	NA

SNP IDs	NCBI dbSNP accession IDs (NCBI_SS#)	Chromosomes	Physical positions (bp)	SNPs	Gene accession IDs	Sequence components of genome	Putative functions
Kabuli_wild_43752723	1399933344	*Ca_kabuli_Chr01*	43752723	A/C	Gene_Ca_13553	SYNONYMOUS_CODING	NA
Kabuli_wild_43753900	1399933345	*Ca_kabuli_Chr01*	43753900	C/T	Gene_Ca_13553	NON_SYNONYMOUS_CODING	NA
Kabuli_wild_43782277	1399933346	*Ca_kabuli_Chr01*	43782277	G/T	Gene_Ca_13549	INTRON	NA
Kabuli_wild_43792565	1399933347	*Ca_kabuli_Chr01*	43792565	G/A	NA	INTERGENIC	NA
Kabuli_wild_43924593	1399933348	*Ca_kabuli_Chr01*	43924593	T/G	Gene_Ca_13540	INTRON	NA
Kabuli_wild_43930138	1399933349	*Ca_kabuli_Chr01*	43930138	T/C	NA	INTERGENIC	NA
Kabuli_wild_43930143	1399933350	*Ca_kabuli_Chr01*	43930143	A/T	NA	INTERGENIC	NA
Kabuli_wild_43933629	1399933351	*Ca_kabuli_Chr01*	43933629	T/C	Gene_Ca_13539	SYNONYMOUS_CODING	NA
Kabuli_wild_43933984	1399933352	*Ca_kabuli_Chr01*	43933984	T/C	NA	INTERGENIC	NA
Kabuli_wild_44085069	1399933353	*Ca_kabuli_Chr01*	44085069	C/T	Gene_Ca_13530	INTRON	Endosulphine
Kabuli_wild_44349004	1399933354	*Ca_kabuli_Chr01*	44349004	T/C	Gene_Ca_23498	NON_SYNONYMOUS_CODING	RibonucleaseII/R
Kabuli_wild_44350953	1399933355	*Ca_kabuli_Chr01*	44350953	G/T	Gene_Ca_23498	INTRON	RibonucleaseII/R
Kabuli_wild_44635858	1399933356	*Ca_kabuli_Chr01*	44635858	G/T	NA	INTERGENIC	NA
Kabuli_wild_44635893	1399933357	*Ca_kabuli_Chr01*	44635893	G/A	NA	INTERGENIC	NA
Kabuli_wild_44635926	1399933358	*Ca_kabuli_Chr01*	44635926	G/A	NA	INTERGENIC	NA
Kabuli_wild_44635939	1399933359	*Ca_kabuli_Chr01*	44635939	T/C	NA	INTERGENIC	NA
Kabuli_wild_44643830	1399933360	*Ca_kabuli_Chr01*	44643830	T/C	NA	INTERGENIC	NA
Kabuli_wild_44643859	1399933361	*Ca_kabuli_Chr01*	44643859	T/A	NA	INTERGENIC	NA
Kabuli_wild_44976735	1399933362	*Ca_kabuli_Chr01*	44976735	C/T	Gene_Ca_25713	INTRON	Protein kinase,catalytic domain
Kabuli_wild_44976738	1399933363	*Ca_kabuli_Chr01*	44976738	T/A	Gene_Ca_25713	INTRON	Protein kinase,catalytic domain
Kabuli_wild_44988344	1399933364	*Ca_kabuli_Chr01*	44988344	A/G	NA	INTERGENIC	NA
Kabuli_wild_45003190	1399933365	*Ca_kabuli_Chr01*	45003190	G/A	Gene_Ca_25714	NON_SYNONYMOUS_CODING	NA
Kabuli_wild_45003229	1399933366	*Ca_kabuli_Chr01*	45003229	A/G	Gene_Ca_25714	NON_SYNONYMOUS_CODING	NA
Kabuli_wild_45003230	1399933367	*Ca_kabuli_Chr01*	45003230	A/G	Gene_Ca_25714	SYNONYMOUS_CODING	NA
Kabuli_wild_45003270	1399933368	*Ca_kabuli_Chr01*	45003270	T/A	Gene_Ca_25714	SYNONYMOUS_CODING	NA
Kabuli_wild_45003329	1399933369	*Ca_kabuli_Chr01*	45003329	T/G	Gene_Ca_25714	NON_SYNONYMOUS_CODING	NA
Kabuli_wild_45003434	1399933370	*Ca_kabuli_Chr01*	45003434	A/G	Gene_Ca_25714	INTRON	NA
Kabuli_wild_45003450	1399933371	*Ca_kabuli_Chr01*	45003450	G/T	Gene_Ca_25714	INTRON	NA
Kabuli_wild_45003456	1399933372	*Ca_kabuli_Chr01*	45003456	C/A	Gene_Ca_25714	INTRON	NA
Kabuli_wild_45003505	1399933373	*Ca_kabuli_Chr01*	45003505	A/C	Gene_Ca_25714	INTRON	NA
Kabuli_wild_45003509	1399933374	*Ca_kabuli_Chr01*	45003509	C/A	Gene_Ca_25714	INTRON	NA
Kabuli_wild_45003514	1399933375	*Ca_kabuli_Chr01*	45003514	G/A	Gene_Ca_25714	NON_SYNONYMOUS_CODING	NA
Kabuli_wild_45003938	1399933376	*Ca_kabuli_Chr01*	45003938	A/G	Gene_Ca_25714	INTRON	NA
Kabuli_wild_45003939	1399933377	*Ca_kabuli_Chr01*	45003939	T/A	Gene_Ca_25714	INTRON	NA
Kabuli_wild_45003940	1399933378	*Ca_kabuli_Chr01*	45003940	T/G	Gene_Ca_25714	INTRON	NA
Kabuli_wild_45003944	1399933379	*Ca_kabuli_Chr01*	45003944	T/C	Gene_Ca_25714	INTRON	NA
Kabuli_wild_45003945	1399933380	*Ca_kabuli_Chr01*	45003945	T/A	Gene_Ca_25714	INTRON	NA
Kabuli_wild_45003946	1399933381	*Ca_kabuli_Chr01*	45003946	T/G	Gene_Ca_25714	INTRON	NA
Kabuli_wild_45003947	1399933382	*Ca_kabuli_Chr01*	45003947	C/A	Gene_Ca_25714	INTRON	NA
Kabuli_wild_45003965	1399933383	*Ca_kabuli_Chr01*	45003965	T/C	Gene_Ca_25714	INTRON	NA
Kabuli_wild_45003972	1399933384	*Ca_kabuli_Chr01*	45003972	A/G	Gene_Ca_25714	INTRON	NA
Kabuli_wild_45003973	1399933385	*Ca_kabuli_Chr01*	45003973	T/C	Gene_Ca_25714	INTRON	NA
Kabuli_wild_45003985	1399933386	*Ca_kabuli_Chr01*	45003985	G/T	Gene_Ca_25714	INTRON	NA
Kabuli_wild_45003998	1399933387	*Ca_kabuli_Chr01*	45003998	G/A	Gene_Ca_25714	INTRON	NA

SNP IDs	NCBI dbSNP accession IDs (NCBI_SS#)	Chromosomes	Physical positions (bp)	SNPs	Gene accession IDs	Sequence components of genome	Putative functions
Kabuli_wild_45004001	1399933388	*Ca_kabuli_Chr01*	45004001	C/T	Gene_Ca_25714	INTRON	NA
Kabuli_wild_45004016	1399933389	*Ca_kabuli_Chr01*	45004016	G/T	Gene_Ca_25714	INTRON	NA
Kabuli_wild_45004017	1399933390	*Ca_kabuli_Chr01*	45004017	C/G	Gene_Ca_25714	INTRON	NA
Kabuli_wild_45004200	1399933391	*Ca_kabuli_Chr01*	45004200	G/T	Gene_Ca_25714	INTRON	NA
Kabuli_wild_45004205	1399933392	*Ca_kabuli_Chr01*	45004205	C/T	Gene_Ca_25714	INTRON	NA
Kabuli_wild_45004284	1399933393	*Ca_kabuli_Chr01*	45004284	G/A	Gene_Ca_25714	SYNONYMOUS_CODING	NA
Kabuli_wild_45007032	1399933394	*Ca_kabuli_Chr01*	45007032	A/C	Gene_Ca_25714	INTRON	NA
Kabuli_wild_45007090	1399933395	*Ca_kabuli_Chr01*	45007090	G/A	Gene_Ca_25714	INTRON	NA
Kabuli_wild_45007194	1399933396	*Ca_kabuli_Chr01*	45007194	G/T	Gene_Ca_25714	INTRON	NA
Kabuli_wild_45007197	1399933397	*Ca_kabuli_Chr01*	45007197	T/A	Gene_Ca_25714	INTRON	NA
Kabuli_wild_45007200	1399933398	*Ca_kabuli_Chr01*	45007200	T/A	Gene_Ca_25714	INTRON	NA
Kabuli_wild_45007223	1399933399	*Ca_kabuli_Chr01*	45007223	A/C	Gene_Ca_25714	INTRON	NA
Kabuli_wild_45007227	1399933400	*Ca_kabuli_Chr01*	45007227	T/C	Gene_Ca_25714	INTRON	NA
Kabuli_wild_45007238	1399933401	*Ca_kabuli_Chr01*	45007238	A/G	Gene_Ca_25714	INTRON	NA
Kabuli_wild_45007256	1399933402	*Ca_kabuli_Chr01*	45007256	A/T	Gene_Ca_25714	INTRON	NA
Kabuli_wild_45007259	1399933403	*Ca_kabuli_Chr01*	45007259	A/G	Gene_Ca_25714	INTRON	NA
Kabuli_wild_45007331	1399933404	*Ca_kabuli_Chr01*	45007331	C/T	Gene_Ca_25714	INTRON	NA
Kabuli_wild_45008004	1399933405	*Ca_kabuli_Chr01*	45008004	C/T	Gene_Ca_25714	INTRON	NA
Kabuli_wild_45008006	1399933406	*Ca_kabuli_Chr01*	45008006	C/A	Gene_Ca_25714	INTRON	NA
Kabuli_wild_45008009	1399933407	*Ca_kabuli_Chr01*	45008009	C/T	Gene_Ca_25714	INTRON	NA
Kabuli_wild_45008015	1399933408	*Ca_kabuli_Chr01*	45008015	G/A	Gene_Ca_25714	INTRON	NA
Kabuli_wild_45008016	1399933409	*Ca_kabuli_Chr01*	45008016	T/A	Gene_Ca_25714	INTRON	NA
Kabuli_wild_45008044	1399933410	*Ca_kabuli_Chr01*	45008044	T/C	Gene_Ca_25714	INTRON	NA
Kabuli_wild_45008050	1399933411	*Ca_kabuli_Chr01*	45008050	C/G	Gene_Ca_25714	INTRON	NA
Kabuli_wild_45008071	1399933412	*Ca_kabuli_Chr01*	45008071	G/C	Gene_Ca_25714	INTRON	NA
Kabuli_wild_45008073	1399933413	*Ca_kabuli_Chr01*	45008073	T/G	Gene_Ca_25714	INTRON	NA
Kabuli_wild_45008075	1399933414	*Ca_kabuli_Chr01*	45008075	T/C	Gene_Ca_25714	INTRON	NA
Kabuli_wild_45008076	1399933415	*Ca_kabuli_Chr01*	45008076	G/A	Gene_Ca_25714	INTRON	NA
Kabuli_wild_45250802	1399933416	*Ca_kabuli_Chr01*	45250802	C/T	NA	INTERGENIC	NA
Kabuli_wild_45250803	1399933417	*Ca_kabuli_Chr01*	45250803	A/G	NA	INTERGENIC	NA
Kabuli_wild_45250804	1399933418	*Ca_kabuli_Chr01*	45250804	G/A	NA	INTERGENIC	NA
Kabuli_wild_45250805	1399933419	*Ca_kabuli_Chr01*	45250805	A/C	NA	INTERGENIC	NA
Kabuli_wild_45250807	1399933420	*Ca_kabuli_Chr01*	45250807	C/G	NA	INTERGENIC	NA
Kabuli_wild_45250828	1399933421	*Ca_kabuli_Chr01*	45250828	G/C	NA	INTERGENIC	NA
Kabuli_wild_45250834	1399933422	*Ca_kabuli_Chr01*	45250834	A/G	NA	INTERGENIC	NA
Kabuli_wild_45250862	1399933423	*Ca_kabuli_Chr01*	45250862	A/T	NA	INTERGENIC	NA
Kabuli_wild_45250863	1399933424	*Ca_kabuli_Chr01*	45250863	C/T	NA	INTERGENIC	NA
Kabuli_wild_45250869	1399933425	*Ca_kabuli_Chr01*	45250869	G/A	NA	INTERGENIC	NA
Kabuli_wild_45250872	1399933426	*Ca_kabuli_Chr01*	45250872	G/T	NA	INTERGENIC	NA
Kabuli_wild_45250874	1399933427	*Ca_kabuli_Chr01*	45250874	G/A	NA	INTERGENIC	NA
Kabuli_wild_46429309	1399933428	*Ca_kabuli_Chr01*	46429309	T/C	Gene_Ca_21547	INTRON	Adenylatekinase
Kabuli_wild_46437023	1399933429	*Ca_kabuli_Chr01*	46437023	T/G	Gene_Ca_21547	SYNONYMOUS_CODING	Adenylatekinase
Kabuli_wild_46523221	1399933430	*Ca_kabuli_Chr01*	46523221	C/T	Gene_Ca_21550	SYNONYMOUS_CODING	NA
Kabuli_wild_46597131	1399933431	*Ca_kabuli_Chr01*	46597131	T/G	NA	INTERGENIC	NA

SNP IDs	NCBI dbSNP accession IDs (NCBI_SS#)	Chromosomes	Physical positions (bp)	SNPs	Gene accession IDs	Sequence components of genome	Putative functions
Kabuli_wild_46597160	1399933432	*Ca_kabuli_Chr01*	46597160	A/C	NA	INTERGENIC	NA
Kabuli_wild_46597180	1399933433	*Ca_kabuli_Chr01*	46597180	G/A	NA	INTERGENIC	NA
Kabuli_wild_46597197	1399933434	*Ca_kabuli_Chr01*	46597197	C/T	NA	INTERGENIC	NA
Kabuli_wild_46597199	1399933435	*Ca_kabuli_Chr01*	46597199	A/G	NA	INTERGENIC	NA
Kabuli_wild_46597220	1399933436	*Ca_kabuli_Chr01*	46597220	G/T	NA	INTERGENIC	NA
Kabuli_wild_46597221	1399933437	*Ca_kabuli_Chr01*	46597221	G/A	NA	INTERGENIC	NA
Kabuli_wild_46597223	1399933438	*Ca_kabuli_Chr01*	46597223	C/T	NA	INTERGENIC	NA
Kabuli_wild_46597235	1399933439	*Ca_kabuli_Chr01*	46597235	C/T	NA	INTERGENIC	NA
Kabuli_wild_46597238	1399933440	*Ca_kabuli_Chr01*	46597238	C/T	NA	INTERGENIC	NA
Kabuli_wild_46717271	1399933441	*Ca_kabuli_Chr01*	46717271	C/T	Gene_Ca_25108	INTRON	NA
Kabuli_wild_46737928	1399933442	*Ca_kabuli_Chr01*	46737928	A/C	Gene_Ca_25111	INTRON	Protein of unknown function DUF3754
Kabuli_wild_46745628	1399933443	*Ca_kabuli_Chr01*	46745628	C/A	Gene_Ca_25111	INTRON	Protein of unknown function DUF3754
Kabuli_wild_46778814	1399933444	*Ca_kabuli_Chr01*	46778814	A/C	Gene_Ca_25114	NON_SYNONYMOUS_CODING	Protein kinase,catalytic domain
Kabuli_wild_46778958	1399933445	*Ca_kabuli_Chr01*	46778958	A/G	Gene_Ca_25114	SYNONYMOUS_CODING	Protein kinase,catalytic domain
Kabuli_wild_46791939	1399933446	*Ca_kabuli_Chr01*	46791939	T/G	NA	INTERGENIC	NA
Kabuli_wild_46793357	1399933447	*Ca_kabuli_Chr01*	46793357	T/C	NA	INTERGENIC	NA
Kabuli_wild_46816041	1399933448	*Ca_kabuli_Chr01*	46816041	A/T	NA	INTERGENIC	NA
Kabuli_wild_46825084	1399933449	*Ca_kabuli_Chr01*	46825084	C/A	Gene_Ca_25116	INTRON	NA
Kabuli_wild_46825211	1399933450	*Ca_kabuli_Chr01*	46825211	G/A	Gene_Ca_25116	INTRON	NA
Kabuli_wild_46825213	1399933451	*Ca_kabuli_Chr01*	46825213	G/C	Gene_Ca_25116	INTRON	NA
Kabuli_wild_46829662	1399933452	*Ca_kabuli_Chr01*	46829662	C/T	Gene_Ca_25116	INTRON	NA
Kabuli_wild_46832306	1399933453	*Ca_kabuli_Chr01*	46832306	T/G	Gene_Ca_25116	INTRON	NA
Kabuli_wild_46832383	1399933454	*Ca_kabuli_Chr01*	46832383	T/C	Gene_Ca_25116	INTRON	NA
Kabuli_wild_46832395	1399933455	*Ca_kabuli_Chr01*	46832395	A/G	Gene_Ca_25116	INTRON	NA
Kabuli_wild_46920524	1399933456	*Ca_kabuli_Chr01*	46920524	C/T	Gene_Ca_12949	INTRON	NA
Kabuli_wild_46936201	1399933457	*Ca_kabuli_Chr01*	46936201	C/T	NA	INTERGENIC	NA
Kabuli_wild_46969221	1399933458	*Ca_kabuli_Chr01*	46969221	T/A	Gene_Ca_12942	INTRON	Glycosyltransferase,family20
Kabuli_wild_46969650	1399933459	*Ca_kabuli_Chr01*	46969650	C/T	Gene_Ca_12942	SYNONYMOUS_CODING	Glycosyltransferase,family20
Kabuli_wild_47042798	1399933460	*Ca_kabuli_Chr01*	47042798	T/A	Gene_Ca_12938	NON_SYNONYMOUS_CODING	Protein of unknown function DUF92,TMEM19
Kabuli_wild_47181534	1399933461	*Ca_kabuli_Chr01*	47181534	C/A	Gene_Ca_12931	INTRON	Protein kinase,catalytic domain
Kabuli_wild_47331080	1399933462	*Ca_kabuli_Chr01*	47331080	T/C	NA	INTERGENIC	NA
Kabuli_wild_47331082	1399933463	*Ca_kabuli_Chr01*	47331082	T/C	NA	INTERGENIC	NA
Kabuli_wild_47891286	1399933464	*Ca_kabuli_Chr01*	47891286	T/A	Gene_Ca_12888	NON_SYNONYMOUS_CODING	Phosphoenolpyruvatecarboxylase,activesite
Kabuli_wild_47891289	1399933465	*Ca_kabuli_Chr01*	47891289	C/A	Gene_Ca_12888	SYNONYMOUS_CODING	Phosphoenolpyruvatecarboxylase,activesite
Kabuli_wild_47897914	1399933466	*Ca_kabuli_Chr01*	47897914	C/T	Gene_Ca_12887	INTRON	GPI mannosyl transferase
Kabuli_wild_47938884	1399933467	*Ca_kabuli_Chr01*	47938884	T/G	Gene_Ca_12883	INTRON	Ubiquitin-conjugating enzyme, E2
Kabuli_wild_47947163	1399933468	*Ca_kabuli_Chr01*	47947163	G/A	Gene_Ca_12882	STOP_GAINED	TetratricopeptideTPR-1
Kabuli_wild_48026664	1399933469	*Ca_kabuli_Chr01*	48026664	G/T	NA	INTERGENIC	NA
Kabuli_wild_48026727	1399933470	*Ca_kabuli_Chr01*	48026727	A/C	NA	INTERGENIC	NA
Kabuli_wild_48028094	1399933471	*Ca_kabuli_Chr01*	48028094	G/A	NA	INTERGENIC	NA
Kabuli_wild_48028282	1399933472	*Ca_kabuli_Chr01*	48028282	T/G	NA	INTERGENIC	NA
Kabuli_wild_48132521	1399933473	*Ca_kabuli_Chr01*	48132521	A/C	NA	INTERGENIC	NA
Kabuli_wild_48154076	1399933474	*Ca_kabuli_Chr01*	48154076	C/A	NA	INTERGENIC	NA
Kabuli_wild_48191532	1399933475	*Ca_kabuli_Chr01*	48191532	C/A	Gene_Ca_12861	UPSTREAM	Plantnuclearmatrix1

SNP IDs	NCBI dbSNP accession IDs (NCBI_SS#)	Chromosomes	Physical positions (bp)	SNPs	Gene accession IDs	Sequence components of genome	Putative functions
Kabuli_wild_48265590	1399933476	*Ca_kabuli_Chr01*	48265590	T/G	Gene_Ca_12856	SYNONYMOUS_CODING	NA
Kabuli_wild_137850	1399933477	*Ca_kabuli_Chr02*	137850	C/A	NA	INTERGENIC	NA
Kabuli_wild_233589	1399933478	*Ca_kabuli_Chr02*	233589	C/T	Gene_Ca_16975	DOWNSTREAM	NA
Kabuli_wild_354695	1399933479	*Ca_kabuli_Chr02*	354695	G/T	Gene_Ca_16982	NON_SYNONYMOUS_CODING	Lateral organ boundaries, LOB
Kabuli_wild_355813	1399933480	*Ca_kabuli_Chr02*	355813	T/G	Gene_Ca_16982	NON_SYNONYMOUS_CODING	Lateral organ boundaries, LOB
Kabuli_wild_355818	1399933481	*Ca_kabuli_Chr02*	355818	T/G	Gene_Ca_16982	SYNONYMOUS_CODING	Lateral organ boundaries, LOB
Kabuli_wild_355930	1399933482	*Ca_kabuli_Chr02*	355930	T/G	Gene_Ca_16982	NON_SYNONYMOUS_CODING	Lateral organ boundaries, LOB
Kabuli_wild_522943	1399933483	*Ca_kabuli_Chr02*	522943	A/G	NA	INTERGENIC	NA
Kabuli_wild_669303	1399933484	*Ca_kabuli_Chr02*	669303	G/A	Gene_Ca_16997	INTRON	14-3-3protein
Kabuli_wild_737183	1399933485	*Ca_kabuli_Chr02*	737183	T/G	Gene_Ca_17000	SYNONYMOUS_CODING	NA
Kabuli_wild_746353	1399933486	*Ca_kabuli_Chr02*	746353	C/A	Gene_Ca_17000	INTRON	NA
Kabuli_wild_770150	1399933487	*Ca_kabuli_Chr02*	770150	C/T	Gene_Ca_17001	NON_SYNONYMOUS_CODING	Peptidase C48, SUMO/Sentrin/Ubl1
Kabuli_wild_770152	1399933488	*Ca_kabuli_Chr02*	770152	G/A	Gene_Ca_17001	NON_SYNONYMOUS_CODING	Peptidase C48, SUMO/Sentrin/Ubl1
Kabuli_wild_770164	1399933489	*Ca_kabuli_Chr02*	770164	A/C	Gene_Ca_17001	SYNONYMOUS_CODING	Peptidase C48, SUMO/Sentrin/Ubl1
Kabuli_wild_770183	1399933490	*Ca_kabuli_Chr02*	770183	C/T	Gene_Ca_17001	NON_SYNONYMOUS_CODING	Peptidase C48, SUMO/Sentrin/Ubl1
Kabuli_wild_770194	1399933491	*Ca_kabuli_Chr02*	770194	G/C	Gene_Ca_17001	NON_SYNONYMOUS_CODING	Peptidase C48, SUMO/Sentrin/Ubl1
Kabuli_wild_770225	1399933492	*Ca_kabuli_Chr02*	770225	C/T	Gene_Ca_17001	NON_SYNONYMOUS_CODING	Peptidase C48, SUMO/Sentrin/Ubl1
Kabuli_wild_770231	1399933493	*Ca_kabuli_Chr02*	770231	C/T	Gene_Ca_17001	NON_SYNONYMOUS_CODING	Peptidase C48, SUMO/Sentrin/Ubl1
Kabuli_wild_770234	1399933494	*Ca_kabuli_Chr02*	770234	C/T	Gene_Ca_17001	NON_SYNONYMOUS_CODING	Peptidase C48, SUMO/Sentrin/Ubl1
Kabuli_wild_770237	1399933495	*Ca_kabuli_Chr02*	770237	C/A	Gene_Ca_17001	NON_SYNONYMOUS_CODING	Peptidase C48, SUMO/Sentrin/Ubl1
Kabuli_wild_770248	1399933496	*Ca_kabuli_Chr02*	770248	A/G	Gene_Ca_17001	NON_SYNONYMOUS_CODING	Peptidase C48, SUMO/Sentrin/Ubl1
Kabuli_wild_770259	1399933497	*Ca_kabuli_Chr02*	770259	G/A	Gene_Ca_17001	SYNONYMOUS_CODING	Peptidase C48, SUMO/Sentrin/Ubl1
Kabuli_wild_770260	1399933498	*Ca_kabuli_Chr02*	770260	C/T	Gene_Ca_17001	NON_SYNONYMOUS_CODING	Peptidase C48, SUMO/Sentrin/Ubl1
Kabuli_wild_770266	1399933499	*Ca_kabuli_Chr02*	770266	C/A	Gene_Ca_17001	NON_SYNONYMOUS_CODING	Peptidase C48, SUMO/Sentrin/Ubl1
Kabuli_wild_770283	1399933500	*Ca_kabuli_Chr02*	770283	G/A	Gene_Ca_17001	SYNONYMOUS_CODING	Peptidase C48, SUMO/Sentrin/Ubl1
Kabuli_wild_770290	1399933501	*Ca_kabuli_Chr02*	770290	G/A	Gene_Ca_17001	NON_SYNONYMOUS_CODING	Peptidase C48, SUMO/Sentrin/Ubl1
Kabuli_wild_770299	1399933502	*Ca_kabuli_Chr02*	770299	A/C	Gene_Ca_17001	NON_SYNONYMOUS_CODING	Peptidase C48, SUMO/Sentrin/Ubl1
Kabuli_wild_1198469	1399933503	*Ca_kabuli_Chr02*	1198469	A/G	NA	INTERGENIC	NA
Kabuli_wild_1198497	1399933504	*Ca_kabuli_Chr02*	1198497	G/T	NA	INTERGENIC	NA
Kabuli_wild_1198509	1399933505	*Ca_kabuli_Chr02*	1198509	G/A	NA	INTERGENIC	NA
Kabuli_wild_1198529	1399933506	*Ca_kabuli_Chr02*	1198529	A/T	NA	INTERGENIC	NA
Kabuli_wild_1231500	1399933507	*Ca_kabuli_Chr02*	1231500	A/G	NA	INTERGENIC	NA
Kabuli_wild_1231618	1399933508	*Ca_kabuli_Chr02*	1231618	G/A	NA	INTERGENIC	NA
Kabuli_wild_1234932	1399933509	*Ca_kabuli_Chr02*	1234932	C/T	NA	INTERGENIC	NA
Kabuli_wild_1234983	1399933510	*Ca_kabuli_Chr02*	1234983	T/C	NA	INTERGENIC	NA
Kabuli_wild_1368910	1399933511	*Ca_kabuli_Chr02*	1368910	C/A	NA	INTERGENIC	NA
Kabuli_wild_1368911	1399933512	*Ca_kabuli_Chr02*	1368911	A/G	NA	INTERGENIC	NA
Kabuli_wild_1389661	1399933513	*Ca_kabuli_Chr02*	1389661	A/G	NA	INTERGENIC	NA
Kabuli_wild_1493971	1399933514	*Ca_kabuli_Chr02*	1493971	A/G	NA	INTERGENIC	NA
Kabuli_wild_1537000	1399933515	*Ca_kabuli_Chr02*	1537000	T/C	NA	INTERGENIC	NA
Kabuli_wild_1610477	1399933516	*Ca_kabuli_Chr02*	1610477	G/T	Gene_Ca_12629	NON_SYNONYMOUS_CODING	Diacylglycerolkinase,catalytic domain
Kabuli_wild_1668116	1399933517	*Ca_kabuli_Chr02*	1668116	A/G	Gene_Ca_12622	UPSTREAM	NA
Kabuli_wild_1671279	1399933518	*Ca_kabuli_Chr02*	1671279	T/C	Gene_Ca_12621	INTRON	HEAT
Kabuli_wild_1704328	1399933519	*Ca_kabuli_Chr02*	1704328	G/T	NA	INTERGENIC	NA

SNP IDs	NCBI dbSNP accession IDs (NCBI_SS#)	Chromosomes	Physical positions (bp)	SNPs	Gene accession IDs	Sequence components of genome	Putative functions
Kabuli_wild_1713343	1399933520	*Ca_kabuli_Chr02*	1713343	T/C	Gene_Ca_12614	UPSTREAM	RNA-binding,CRMdomain
Kabuli_wild_1802536	1399933521	*Ca_kabuli_Chr02*	1802536	T/C	Gene_Ca_12605	INTRON	Domain of unknown function DUF3730
Kabuli_wild_1828406	1399933522	*Ca_kabuli_Chr02*	1828406	G/A	Gene_Ca_12602	NON_SYNONYMOUS_CODING	Protein of unknown function DUF1421
Kabuli_wild_1867041	1399933523	*Ca_kabuli_Chr02*	1867041	C/A	Gene_Ca_12598	INTRON	BacterialIg-like,group2
Kabuli_wild_1960343	1399933524	*Ca_kabuli_Chr02*	1960343	T/G	Gene_Ca_12584	NON_SYNONYMOUS_CODING	Exoribonuclease,phosphorolytic domain1
Kabuli_wild_2230056	1399933525	*Ca_kabuli_Chr02*	2230056	T/C	NA	INTERGENIC	NA
Kabuli_wild_2278984	1399933526	*Ca_kabuli_Chr02*	2278984	A/G	Gene_Ca_12550	UPSTREAM	NA
Kabuli_wild_2279017	1399933527	*Ca_kabuli_Chr02*	2279017	A/T	Gene_Ca_12550	UPSTREAM	NA
Kabuli_wild_2311915	1399933528	*Ca_kabuli_Chr02*	2311915	C/A	NA	INTERGENIC	NA
Kabuli_wild_2311917	1399933529	*Ca_kabuli_Chr02*	2311917	A/C	NA	INTERGENIC	NA
Kabuli_wild_2311948	1399933530	*Ca_kabuli_Chr02*	2311948	T/C	NA	INTERGENIC	NA
Kabuli_wild_2312029	1399933531	*Ca_kabuli_Chr02*	2312029	C/T	NA	INTERGENIC	NA
Kabuli_wild_2312032	1399933532	*Ca_kabuli_Chr02*	2312032	T/C	NA	INTERGENIC	NA
Kabuli_wild_2312034	1399933533	*Ca_kabuli_Chr02*	2312034	C/A	NA	INTERGENIC	NA
Kabuli_wild_2312037	1399933534	*Ca_kabuli_Chr02*	2312037	T/G	NA	INTERGENIC	NA
Kabuli_wild_2312104	1399933535	*Ca_kabuli_Chr02*	2312104	C/T	NA	INTERGENIC	NA
Kabuli_wild_2344777	1399933536	*Ca_kabuli_Chr02*	2344777	A/G	Gene_Ca_12544	SYNONYMOUS_CODING	Oligo peptide transporter
Kabuli_wild_2344778	1399933537	*Ca_kabuli_Chr02*	2344778	A/G	Gene_Ca_12544	NON_SYNONYMOUS_CODING	Oligo peptide transporter
Kabuli_wild_2344786	1399933538	*Ca_kabuli_Chr02*	2344786	A/G	Gene_Ca_12544	SYNONYMOUS_CODING	Oligo peptide transporter
Kabuli_wild_2344807	1399933539	*Ca_kabuli_Chr02*	2344807	T/G	Gene_Ca_12544	SYNONYMOUS_CODING	Oligo peptide transporter
Kabuli_wild_2344835	1399933540	*Ca_kabuli_Chr02*	2344835	G/T	Gene_Ca_12544	STOP_GAINED	Oligo peptide transporter
Kabuli_wild_2416422	1399933541	*Ca_kabuli_Chr02*	2416422	C/T	NA	INTERGENIC	NA
Kabuli_wild_2650373	1399933542	*Ca_kabuli_Chr02*	2650373	A/T	NA	INTERGENIC	NA
Kabuli_wild_2650375	1399933543	*Ca_kabuli_Chr02*	2650375	T/C	NA	INTERGENIC	NA
Kabuli_wild_2697688	1399933544	*Ca_kabuli_Chr02*	2697688	G/C	Gene_Ca_10574	NON_SYNONYMOUS_CODING	RNA-binding,CRMdomain
Kabuli_wild_2708805	1399933545	*Ca_kabuli_Chr02*	2708805	G/C	NA	INTERGENIC	NA
Kabuli_wild_2780302	1399933546	*Ca_kabuli_Chr02*	2780302	C/T	NA	INTERGENIC	NA
Kabuli_wild_2780325	1399933547	*Ca_kabuli_Chr02*	2780325	C/A	NA	INTERGENIC	NA
Kabuli_wild_2783727	1399933548	*Ca_kabuli_Chr02*	2783727	G/A	NA	INTERGENIC	NA
Kabuli_wild_2846667	1399933549	*Ca_kabuli_Chr02*	2846667	T/C	Gene_Ca_10559	INTRON	Phosphoglucoseisomerase(PGI)
Kabuli_wild_2846774	1399933550	*Ca_kabuli_Chr02*	2846774	C/A	Gene_Ca_10559	NON_SYNONYMOUS_CODING	Phosphoglucoseisomerase(PGI)
Kabuli_wild_2849370	1399933551	*Ca_kabuli_Chr02*	2849370	A/G	Gene_Ca_10559	INTRON	Phosphoglucoseisomerase(PGI)
Kabuli_wild_2965867	1399933552	*Ca_kabuli_Chr02*	2965867	G/A	NA	INTERGENIC	NA
Kabuli_wild_3093987	1399933553	*Ca_kabuli_Chr02*	3093987	T/G	Gene_Ca_10541	SYNONYMOUS_CODING	Protein kinase,catalytic domain
Kabuli_wild_3100116	1399933554	*Ca_kabuli_Chr02*	3100116	A/G	NA	INTERGENIC	NA
Kabuli_wild_3113514	1399933555	*Ca_kabuli_Chr02*	3113514	G/A	Gene_Ca_10538	INTRON	Auxin efflux carrier
Kabuli_wild_3113560	1399933556	*Ca_kabuli_Chr02*	3113560	G/A	Gene_Ca_10538	INTRON	Auxin efflux carrier
Kabuli_wild_3141128	1399933557	*Ca_kabuli_Chr02*	3141128	A/C	Gene_Ca_10534	DOWNSTREAM	NA
Kabuli_wild_3151192	1399933558	*Ca_kabuli_Chr02*	3151192	C/A	Gene_Ca_10532	NON_SYNONYMOUS_CODING	WD40 repeat
Kabuli_wild_3164285	1399933559	*Ca_kabuli_Chr02*	3164285	T/C	Gene_Ca_10531	DOWNSTREAM	Cysteaminedioxygenase
Kabuli_wild_3164287	1399933560	*Ca_kabuli_Chr02*	3164287	T/A	Gene_Ca_10531	DOWNSTREAM	Cysteaminedioxygenase
Kabuli_wild_3164292	1399933561	*Ca_kabuli_Chr02*	3164292	T/A	Gene_Ca_10531	DOWNSTREAM	Cysteaminedioxygenase
Kabuli_wild_3164304	1399933562	*Ca_kabuli_Chr02*	3164304	C/T	Gene_Ca_10531	DOWNSTREAM	Cysteaminedioxygenase
Kabuli_wild_3164308	1399933563	*Ca_kabuli_Chr02*	3164308	T/A	Gene_Ca_10531	DOWNSTREAM	Cysteaminedioxygenase

SNP IDs	NCBI dbSNP accession IDs (NCBI_SS#)	Chromosomes	Physical positions (bp)	SNPs	Gene accession IDs	Sequence components of genome	Putative functions
Kabuli_wild_3164323	1399933564	*Ca_kabuli_Chr02*	3164323	G/T	Gene_Ca_10531	DOWNSTREAM	Cysteaminedioxygenase
Kabuli_wild_3164326	1399933565	*Ca_kabuli_Chr02*	3164326	A/C	Gene_Ca_10531	DOWNSTREAM	Cysteaminedioxygenase
Kabuli_wild_3164355	1399933566	*Ca_kabuli_Chr02*	3164355	G/A	Gene_Ca_10531	DOWNSTREAM	Cysteaminedioxygenase
Kabuli_wild_3164404	1399933567	*Ca_kabuli_Chr02*	3164404	A/G	Gene_Ca_10531	DOWNSTREAM	Cysteaminedioxygenase
Kabuli_wild_3174868	1399933568	*Ca_kabuli_Chr02*	3174868	G/C	NA	INTERGENIC	NA
Kabuli_wild_3450904	1399933569	*Ca_kabuli_Chr02*	3450904	C/A	Gene_Ca_10514	SYNONYMOUS_CODING	ZF-HD homeobox protein,Cys/His-rich dimerisation domain
Kabuli_wild_3482714	1399933570	*Ca_kabuli_Chr02*	3482714	A/C	Gene_Ca_10512	NON_SYNONYMOUS_CODING	Glycosyltransferase,group1
Kabuli_wild_3503750	1399933571	*Ca_kabuli_Chr02*	3503750	C/G	NA	INTERGENIC	NA
Kabuli_wild_3503753	1399933572	*Ca_kabuli_Chr02*	3503753	C/G	NA	INTERGENIC	NA
Kabuli_wild_3503759	1399933573	*Ca_kabuli_Chr02*	3503759	T/A	NA	INTERGENIC	NA
Kabuli_wild_3503763	1399933574	*Ca_kabuli_Chr02*	3503763	T/G	NA	INTERGENIC	NA
Kabuli_wild_3552688	1399933575	*Ca_kabuli_Chr02*	3552688	A/G	NA	INTERGENIC	NA
Kabuli_wild_3553182	1399933576	*Ca_kabuli_Chr02*	3553182	G/T	NA	INTERGENIC	NA
Kabuli_wild_3561979	1399933577	*Ca_kabuli_Chr02*	3561979	A/T	NA	INTERGENIC	NA
Kabuli_wild_3789808	1399933578	*Ca_kabuli_Chr02*	3789808	G/T	NA	INTERGENIC	NA
Kabuli_wild_3870877	1399933579	*Ca_kabuli_Chr02*	3870877	G/A	NA	INTERGENIC	NA
Kabuli_wild_3904814	1399933580	*Ca_kabuli_Chr02*	3904814	C/A	NA	INTERGENIC	NA
Kabuli_wild_3916353	1399933581	*Ca_kabuli_Chr02*	3916353	A/C	Gene_Ca_10464	SYNONYMOUS_CODING	Biotin/lipoylattachment
Kabuli_wild_3941397	1399933582	*Ca_kabuli_Chr02*	3941397	A/T	NA	INTERGENIC	NA
Kabuli_wild_3994150	1399933583	*Ca_kabuli_Chr02*	3994150	A/C	NA	INTERGENIC	NA
Kabuli_wild_4039854	1399933584	*Ca_kabuli_Chr02*	4039854	G/A	NA	INTERGENIC	NA
Kabuli_wild_4404733	1399933585	*Ca_kabuli_Chr02*	4404733	G/A	Gene_Ca_21106	INTRON	Glycosylhydrolase,family13,catalytic domain
Kabuli_wild_4404779	1399933586	*Ca_kabuli_Chr02*	4404779	G/A	Gene_Ca_21106	INTRON	Glycosylhydrolase,family13,catalytic domain
Kabuli_wild_4404813	1399933587	*Ca_kabuli_Chr02*	4404813	C/T	Gene_Ca_21106	INTRON	Glycosylhydrolase,family13,catalytic domain
Kabuli_wild_4452054	1399933588	*Ca_kabuli_Chr02*	4452054	T/C	NA	INTERGENIC	NA
Kabuli_wild_4678076	1399933589	*Ca_kabuli_Chr02*	4678076	G/A	NA	INTERGENIC	NA
Kabuli_wild_4678087	1399933590	*Ca_kabuli_Chr02*	4678087	A/G	NA	INTERGENIC	NA
Kabuli_wild_4678099	1399933591	*Ca_kabuli_Chr02*	4678099	A/G	NA	INTERGENIC	NA
Kabuli_wild_4678140	1399933592	*Ca_kabuli_Chr02*	4678140	C/T	NA	INTERGENIC	NA
Kabuli_wild_4678155	1399933593	*Ca_kabuli_Chr02*	4678155	C/T	NA	INTERGENIC	NA
Kabuli_wild_4678564	1399933594	*Ca_kabuli_Chr02*	4678564	G/T	NA	INTERGENIC	NA
Kabuli_wild_4966216	1399933595	*Ca_kabuli_Chr02*	4966216	C/T	Gene_Ca_14714	NON_SYNONYMOUS_CODING	DNAligase,ATP-dependent,N-terminal
Kabuli_wild_4968352	1399933596	*Ca_kabuli_Chr02*	4968352	A/G	Gene_Ca_14714	INTRON	DNAligase,ATP-dependent,N-terminal
Kabuli_wild_4981626	1399933597	*Ca_kabuli_Chr02*	4981626	A/G	NA	INTERGENIC	NA
Kabuli_wild_4981666	1399933598	*Ca_kabuli_Chr02*	4981666	G/C	NA	INTERGENIC	NA
Kabuli_wild_4994657	1399933599	*Ca_kabuli_Chr02*	4994657	C/T	Gene_Ca_14711	SYNONYMOUS_CODING	Sugar/inositol transporter
Kabuli_wild_5079639	1399933600	*Ca_kabuli_Chr02*	5079639	C/T	Gene_Ca_14702	NON_SYNONYMOUS_CODING	Zinc finger, RING-type
Kabuli_wild_5165847	1399933601	*Ca_kabuli_Chr02*	5165847	A/G	NA	INTERGENIC	NA
Kabuli_wild_5247557	1399933602	*Ca_kabuli_Chr02*	5247557	A/C	Gene_Ca_14685	DOWNSTREAM	Cystinosin/ERS1prepeat
Kabuli_wild_5247558	1399933603	*Ca_kabuli_Chr02*	5247558	G/T	Gene_Ca_14685	DOWNSTREAM	Cystinosin/ERS1prepeat
Kabuli_wild_5259254	1399933604	*Ca_kabuli_Chr02*	5259254	T/C	Gene_Ca_14682	NON_SYNONYMOUS_CODING	NA
Kabuli_wild_5342864	1399933605	*Ca_kabuli_Chr02*	5342864	C/A	NA	INTERGENIC	NA
Kabuli_wild_5342923	1399933606	*Ca_kabuli_Chr02*	5342923	A/G	NA	INTERGENIC	NA
Kabuli_wild_5343118	1399933607	*Ca_kabuli_Chr02*	5343118	A/C	NA	INTERGENIC	NA

SNP IDs	NCBI dbSNP accession IDs (NCBI_SS#)	Chromosomes	Physical positions (bp)	SNPs	Gene accession IDs	Sequence components of genome	Putative functions
Kabuli_wild_5419121	1399933608	*Ca_kabuli_Chr02*	5419121	C/G	NA	INTERGENIC	NA
Kabuli_wild_5449978	1399933609	*Ca_kabuli_Chr02*	5449978	T/C	NA	INTERGENIC	NA
Kabuli_wild_5475344	1399933610	*Ca_kabuli_Chr02*	5475344	A/G	Gene_Ca_14660	INTRON	Proteasome component (PCI) domain
Kabuli_wild_5787862	1399933611	*Ca_kabuli_Chr02*	5787862	G/A	Gene_Ca_19683	SYNONYMOUS_CODING	Plan tspecific mitochondrial import receptor subunit TOM20
Kabuli_wild_5787887	1399933612	*Ca_kabuli_Chr02*	5787887	A/C	Gene_Ca_19683	INTRON	Plan tspecific mitochondrial import receptor subunit TOM20
Kabuli_wild_6052668	1399933613	*Ca_kabuli_Chr02*	6052668	A/C	Gene_Ca_19707	NON_SYNONYMOUS_CODING	Transcription factor TFIIB-related
Kabuli_wild_6060151	1399933614	*Ca_kabuli_Chr02*	6060151	C/T	Gene_Ca_19707	INTRON	Transcription factor TFIIB-related
Kabuli_wild_6162378	1399933615	*Ca_kabuli_Chr02*	6162378	T/G	Gene_Ca_19714	INTRON	Aminoacid/polyaminetransporterI
Kabuli_wild_6213520	1399933616	*Ca_kabuli_Chr02*	6213520	G/T	Gene_Ca_20925	NON_SYNONYMOUS_CODING	NA
Kabuli_wild_6213530	1399933617	*Ca_kabuli_Chr02*	6213530	C/A	Gene_Ca_20925	NON_SYNONYMOUS_CODING	NA
Kabuli_wild_6213566	1399933618	*Ca_kabuli_Chr02*	6213566	C/T	Gene_Ca_20925	NON_SYNONYMOUS_CODING	NA
Kabuli_wild_6213582	1399933619	*Ca_kabuli_Chr02*	6213582	A/T	Gene_Ca_20925	SYNONYMOUS_CODING	NA
Kabuli_wild_6349208	1399933620	*Ca_kabuli_Chr02*	6349208	T/G	Gene_Ca_20931	INTRON	Helicase/RNaseDC-terminal,HRDCdomain
Kabuli_wild_6367704	1399933621	*Ca_kabuli_Chr02*	6367704	A/G	NA	INTERGENIC	NA
Kabuli_wild_6408406	1399933622	*Ca_kabuli_Chr02*	6408406	C/A	Gene_Ca_20937	INTRON	Double-strandedRNA-binding
Kabuli_wild_6478646	1399933623	*Ca_kabuli_Chr02*	6478646	T/C	NA	INTERGENIC	NA
Kabuli_wild_6478747	1399933624	*Ca_kabuli_Chr02*	6478747	T/C	NA	INTERGENIC	NA
Kabuli_wild_6478754	1399933625	*Ca_kabuli_Chr02*	6478754	G/T	NA	INTERGENIC	NA
Kabuli_wild_6534776	1399933626	*Ca_kabuli_Chr02*	6534776	C/A	Gene_Ca_20948	INTRON	Target SNARE coiled-coil domain
Kabuli_wild_6728716	1399933627	*Ca_kabuli_Chr02*	6728716	A/T	Gene_Ca_19158	INTRON	Glutamate/phenylalanine/leucine/valinedehydrogenase
Kabuli_wild_6728719	1399933628	*Ca_kabuli_Chr02*	6728719	C/T	Gene_Ca_19158	INTRON	Glutamate/phenylalanine/leucine/valinedehydrogenase
Kabuli_wild_6936872	1399933629	*Ca_kabuli_Chr02*	6936872	A/C	Gene_Ca_19141	SYNONYMOUS_CODING	Phosphatidylethanolamine-binding,conservedsite
Kabuli_wild_7027093	1399933630	*Ca_kabuli_Chr02*	7027093	G/A	Gene_Ca_19136	INTRON	RNA recognition motif domain
Kabuli_wild_7125992	1399933631	*Ca_kabuli_Chr02*	7125992	T/G	Gene_Ca_19128	NON_SYNONYMOUS_CODING	Oxidoreductase,N-terminal
Kabuli_wild_7507259	1399933632	*Ca_kabuli_Chr02*	7507259	C/A	Gene_Ca_18111	NON_SYNONYMOUS_CODING	PeptidaseC19,ubiquitincarboxyl-terminalhydrolase2
Kabuli_wild_7591207	1399933633	*Ca_kabuli_Chr02*	7591207	T/C	NA	INTERGENIC	NA
Kabuli_wild_7633515	1399933634	*Ca_kabuli_Chr02*	7633515	G/T	NA	INTERGENIC	NA
Kabuli_wild_7633520	1399933635	*Ca_kabuli_Chr02*	7633520	G/A	NA	INTERGENIC	NA
Kabuli_wild_7633547	1399933636	*Ca_kabuli_Chr02*	7633547	C/T	NA	INTERGENIC	NA
Kabuli_wild_7633564	1399933637	*Ca_kabuli_Chr02*	7633564	G/T	NA	INTERGENIC	NA
Kabuli_wild_7633572	1399933638	*Ca_kabuli_Chr02*	7633572	T/C	NA	INTERGENIC	NA
Kabuli_wild_7633585	1399933639	*Ca_kabuli_Chr02*	7633585	G/A	NA	INTERGENIC	NA
Kabuli_wild_7633592	1399933640	*Ca_kabuli_Chr02*	7633592	G/A	NA	INTERGENIC	NA
Kabuli_wild_7633631	1399933641	*Ca_kabuli_Chr02*	7633631	C/A	NA	INTERGENIC	NA
Kabuli_wild_7633711	1399933642	*Ca_kabuli_Chr02*	7633711	C/T	NA	INTERGENIC	NA
Kabuli_wild_7633793	1399933643	*Ca_kabuli_Chr02*	7633793	C/T	NA	INTERGENIC	NA
Kabuli_wild_7633813	1399933644	*Ca_kabuli_Chr02*	7633813	G/T	NA	INTERGENIC	NA
Kabuli_wild_7633816	1399933645	*Ca_kabuli_Chr02*	7633816	G/A	NA	INTERGENIC	NA
Kabuli_wild_7633818	1399933646	*Ca_kabuli_Chr02*	7633818	G/A	NA	INTERGENIC	NA
Kabuli_wild_7633821	1399933647	*Ca_kabuli_Chr02*	7633821	C/T	NA	INTERGENIC	NA
Kabuli_wild_7633826	1399933648	*Ca_kabuli_Chr02*	7633826	G/A	NA	INTERGENIC	NA
Kabuli_wild_7633869	1399933649	*Ca_kabuli_Chr02*	7633869	G/A	NA	INTERGENIC	NA
Kabuli_wild_7633955	1399933650	*Ca_kabuli_Chr02*	7633955	G/A	NA	INTERGENIC	NA
Kabuli_wild_7633994	1399933651	*Ca_kabuli_Chr02*	7633994	C/T	NA	INTERGENIC	NA

SNP IDs	NCBI dbSNP accession IDs (NCBI_SS#)	Chromosomes	Physical positions (bp)	SNPs	Gene accession IDs	Sequence components of genome	Putative functions
Kabuli_wild_7634002	1399933652	*Ca_kabuli_Chr02*	7634002	T/C	NA	INTERGENIC	NA
Kabuli_wild_7668490	1399933653	*Ca_kabuli_Chr02*	7668490	C/T	NA	INTERGENIC	NA
Kabuli_wild_7668502	1399933654	*Ca_kabuli_Chr02*	7668502	A/C	NA	INTERGENIC	NA
Kabuli_wild_7668537	1399933655	*Ca_kabuli_Chr02*	7668537	A/C	NA	INTERGENIC	NA
Kabuli_wild_7679627	1399933656	*Ca_kabuli_Chr02*	7679627	A/T	Gene_Ca_18121	NON_SYNONYMOUS_CODING	Multiantimicrobial extrusion protein
Kabuli_wild_7679693	1399933657	*Ca_kabuli_Chr02*	7679693	C/G	Gene_Ca_18121	NON_SYNONYMOUS_CODING	Multiantimicrobial extrusion protein
Kabuli_wild_7686563	1399933658	*Ca_kabuli_Chr02*	7686563	C/G	NA	INTERGENIC	NA
Kabuli_wild_7686598	1399933659	*Ca_kabuli_Chr02*	7686598	T/G	NA	INTERGENIC	NA
Kabuli_wild_7686606	1399933660	*Ca_kabuli_Chr02*	7686606	T/A	NA	INTERGENIC	NA
Kabuli_wild_7701189	1399933661	*Ca_kabuli_Chr02*	7701189	T/G	Gene_Ca_18123	NON_SYNONYMOUS_CODING	Multiantimicrobial extrusion protein
Kabuli_wild_7701199	1399933662	*Ca_kabuli_Chr02*	7701199	T/G	Gene_Ca_18123	NON_SYNONYMOUS_CODING	Multiantimicrobial extrusion protein
Kabuli_wild_7701230	1399933663	*Ca_kabuli_Chr02*	7701230	T/G	Gene_Ca_18123	SYNONYMOUS_CODING	Multiantimicrobial extrusion protein
Kabuli_wild_7701249	1399933664	*Ca_kabuli_Chr02*	7701249	A/G	Gene_Ca_18123	NON_SYNONYMOUS_CODING	Multiantimicrobial extrusion protein
Kabuli_wild_7702468	1399933665	*Ca_kabuli_Chr02*	7702468	A/T	Gene_Ca_18123	INTRON	Multiantimicrobial extrusion protein
Kabuli_wild_7702505	1399933666	*Ca_kabuli_Chr02*	7702505	G/T	Gene_Ca_18123	INTRON	Multiantimicrobial extrusion protein
Kabuli_wild_7702520	1399933667	*Ca_kabuli_Chr02*	7702520	T/C	Gene_Ca_18123	NON_SYNONYMOUS_CODING	Multiantimicrobial extrusion protein
Kabuli_wild_7708596	1399933668	*Ca_kabuli_Chr02*	7708596	T/C	NA	INTERGENIC	NA
Kabuli_wild_7708630	1399933669	*Ca_kabuli_Chr02*	7708630	C/A	NA	INTERGENIC	NA
Kabuli_wild_7708660	1399933670	*Ca_kabuli_Chr02*	7708660	G/A	NA	INTERGENIC	NA
Kabuli_wild_7708663	1399933671	*Ca_kabuli_Chr02*	7708663	T/C	NA	INTERGENIC	NA
Kabuli_wild_7708669	1399933672	*Ca_kabuli_Chr02*	7708669	G/T	NA	INTERGENIC	NA
Kabuli_wild_7708678	1399933673	*Ca_kabuli_Chr02*	7708678	C/T	NA	INTERGENIC	NA
Kabuli_wild_7712181	1399933674	*Ca_kabuli_Chr02*	7712181	C/T	NA	INTERGENIC	NA
Kabuli_wild_7714220	1399933675	*Ca_kabuli_Chr02*	7714220	G/A	NA	INTERGENIC	NA
Kabuli_wild_7714258	1399933676	*Ca_kabuli_Chr02*	7714258	T/C	Gene_Ca_18124	SYNONYMOUS_CODING	NA
Kabuli_wild_7714392	1399933677	*Ca_kabuli_Chr02*	7714392	G/A	Gene_Ca_18124	NON_SYNONYMOUS_CODING	NA
Kabuli_wild_7714454	1399933678	*Ca_kabuli_Chr02*	7714454	A/G	Gene_Ca_18124	SYNONYMOUS_CODING	NA
Kabuli_wild_7714463	1399933679	*Ca_kabuli_Chr02*	7714463	T/C	Gene_Ca_18124	SYNONYMOUS_CODING	NA
Kabuli_wild_7714488	1399933680	*Ca_kabuli_Chr02*	7714488	A/G	Gene_Ca_18124	SYNONYMOUS_CODING	NA
Kabuli_wild_7714491	1399933681	*Ca_kabuli_Chr02*	7714491	C/T	Gene_Ca_18124	SYNONYMOUS_CODING	NA
Kabuli_wild_7714495	1399933682	*Ca_kabuli_Chr02*	7714495	G/A	Gene_Ca_18124	SYNONYMOUS_CODING	NA
Kabuli_wild_7714499	1399933683	*Ca_kabuli_Chr02*	7714499	C/T	Gene_Ca_18124	SYNONYMOUS_CODING	NA
Kabuli_wild_7714506	1399933684	*Ca_kabuli_Chr02*	7714506	A/C	Gene_Ca_18124	SYNONYMOUS_CODING	NA
Kabuli_wild_7753419	1399933685	*Ca_kabuli_Chr02*	7753419	G/C	NA	INTERGENIC	NA
Kabuli_wild_7753425	1399933686	*Ca_kabuli_Chr02*	7753425	T/C	NA	INTERGENIC	NA
Kabuli_wild_7753440	1399933687	*Ca_kabuli_Chr02*	7753440	C/T	NA	INTERGENIC	NA
Kabuli_wild_7819281	1399933688	*Ca_kabuli_Chr02*	7819281	T/C	NA	INTERGENIC	NA
Kabuli_wild_7870919	1399933689	*Ca_kabuli_Chr02*	7870919	G/T	NA	INTERGENIC	NA
Kabuli_wild_7870943	1399933690	*Ca_kabuli_Chr02*	7870943	T/G	NA	INTERGENIC	NA
Kabuli_wild_7938890	1399933691	*Ca_kabuli_Chr02*	7938890	C/T	Gene_Ca_18144	SYNONYMOUS_CODING	Allinase,C-terminal
Kabuli_wild_7956166	1399933692	*Ca_kabuli_Chr02*	7956166	C/A	Gene_Ca_18145	INTRON	NA
Kabuli_wild_8201025	1399933693	*Ca_kabuli_Chr02*	8201025	A/C	Gene_Ca_21477	SYNONYMOUS_CODING	Protein kinase,catalytic domain
Kabuli_wild_8337517	1399933694	*Ca_kabuli_Chr02*	8337517	C/T	Gene_Ca_21468	INTRON	Phosphatidylinositol3-/4-kinase,catalytic
Kabuli_wild_8429212	1399933695	*Ca_kabuli_Chr02*	8429212	T/A	NA	INTERGENIC	NA

SNP IDs	NCBI dbSNP accession IDs (NCBI_SS#)	Chromosomes	Physical positions (bp)	SNPs	Gene accession IDs	Sequence components of genome	Putative functions
Kabuli_wild_8484840	1399933696	*Ca_kabuli_Chr02*	8484840	A/T	NA	INTERGENIC	NA
Kabuli_wild_8487331	1399933697	*Ca_kabuli_Chr02*	8487331	T/A	Gene_Ca_21454	UPSTREAM	NA
Kabuli_wild_8487343	1399933698	*Ca_kabuli_Chr02*	8487343	C/T	Gene_Ca_21454	UPSTREAM	NA
Kabuli_wild_8487346	1399933699	*Ca_kabuli_Chr02*	8487346	C/T	Gene_Ca_21454	UPSTREAM	NA
Kabuli_wild_8489172	1399933700	*Ca_kabuli_Chr02*	8489172	C/A	NA	INTERGENIC	NA
Kabuli_wild_8489237	1399933701	*Ca_kabuli_Chr02*	8489237	G/A	NA	INTERGENIC	NA
Kabuli_wild_8489241	1399933702	*Ca_kabuli_Chr02*	8489241	C/A	NA	INTERGENIC	NA
Kabuli_wild_8730067	1399933703	*Ca_kabuli_Chr02*	8730067	C/T	Gene_Ca_22308	INTRON	Clathrin/coatomeradaptor,adaptin-like,N-terminal
Kabuli_wild_8782756	1399933704	*Ca_kabuli_Chr02*	8782756	T/C	NA	INTERGENIC	NA
Kabuli_wild_8782820	1399933705	*Ca_kabuli_Chr02*	8782820	G/T	Gene_Ca_22304	NON_SYNONYMOUS_CODING	Aminoacyl-tRNAsynthetase,classII(G/H/P/S),conserveddomain
Kabuli_wild_8802121	1399933706	*Ca_kabuli_Chr02*	8802121	T/C	NA	INTERGENIC	NA
Kabuli_wild_9231268	1399933707	*Ca_kabuli_Chr02*	9231268	T/C	NA	INTERGENIC	NA
Kabuli_wild_9231314	1399933708	*Ca_kabuli_Chr02*	9231314	A/G	NA	INTERGENIC	NA
Kabuli_wild_9599251	1399933709	*Ca_kabuli_Chr02*	9599251	C/A	Gene_Ca_25087	NON_SYNONYMOUS_CODING	Transposase,Ptta/En/Spm,plant
Kabuli_wild_9599253	1399933710	*Ca_kabuli_Chr02*	9599253	C/A	Gene_Ca_25087	NON_SYNONYMOUS_CODING	Transposase,Ptta/En/Spm,plant
Kabuli_wild_9621953	1399933711	*Ca_kabuli_Chr02*	9621953	A/C	Gene_Ca_25089	SYNONYMOUS_CODING	Actin-bindingFH2/DRFautoregulatory
Kabuli_wild_9739955	1399933712	*Ca_kabuli_Chr02*	9739955	C/T	NA	INTERGENIC	NA
Kabuli_wild_9739959	1399933713	*Ca_kabuli_Chr02*	9739959	T/C	NA	INTERGENIC	NA
Kabuli_wild_9739965	1399933714	*Ca_kabuli_Chr02*	9739965	A/G	NA	INTERGENIC	NA
Kabuli_wild_9739969	1399933715	*Ca_kabuli_Chr02*	9739969	A/G	NA	INTERGENIC	NA
Kabuli_wild_9739974	1399933716	*Ca_kabuli_Chr02*	9739974	T/C	NA	INTERGENIC	NA
Kabuli_wild_9739980	1399933717	*Ca_kabuli_Chr02*	9739980	G/C	NA	INTERGENIC	NA
Kabuli_wild_9739998	1399933718	*Ca_kabuli_Chr02*	9739998	A/G	NA	INTERGENIC	NA
Kabuli_wild_9740007	1399933719	*Ca_kabuli_Chr02*	9740007	A/G	NA	INTERGENIC	NA
Kabuli_wild_9740010	1399933720	*Ca_kabuli_Chr02*	9740010	A/G	NA	INTERGENIC	NA
Kabuli_wild_9756910	1399933721	*Ca_kabuli_Chr02*	9756910	G/A	NA	INTERGENIC	NA
Kabuli_wild_9756955	1399933722	*Ca_kabuli_Chr02*	9756955	G/A	NA	INTERGENIC	NA
Kabuli_wild_9835940	1399933723	*Ca_kabuli_Chr02*	9835940	T/G	NA	INTERGENIC	NA
Kabuli_wild_9841774	1399933724	*Ca_kabuli_Chr02*	9841774	A/T	NA	INTERGENIC	NA
Kabuli_wild_9862794	1399933725	*Ca_kabuli_Chr02*	9862794	A/G	Gene_Ca_20401	INTRON	Protein kinase,catalytic domain
Kabuli_wild_10048153	1399933726	*Ca_kabuli_Chr02*	10048153	T/A	Gene_Ca_20381	INTRON	NA
Kabuli_wild_10060196	1399933727	*Ca_kabuli_Chr02*	10060196	G/T	NA	INTERGENIC	NA
Kabuli_wild_10060200	1399933728	*Ca_kabuli_Chr02*	10060200	G/T	NA	INTERGENIC	NA
Kabuli_wild_10060207	1399933729	*Ca_kabuli_Chr02*	10060207	C/T	NA	INTERGENIC	NA
Kabuli_wild_10067195	1399933730	*Ca_kabuli_Chr02*	10067195	T/A	Gene_Ca_20380	INTRON	SANT domain,DNA binding
Kabuli_wild_10067451	1399933731	*Ca_kabuli_Chr02*	10067451	A/T	Gene_Ca_20380	INTRON	SANT domain,DNA binding
Kabuli_wild_10070602	1399933732	*Ca_kabuli_Chr02*	10070602	A/C	NA	INTERGENIC	NA
Kabuli_wild_10115838	1399933733	*Ca_kabuli_Chr02*	10115838	A/G	NA	INTERGENIC	NA
Kabuli_wild_10131438	1399933734	*Ca_kabuli_Chr02*	10131438	G/A	NA	INTERGENIC	NA
Kabuli_wild_10131443	1399933735	*Ca_kabuli_Chr02*	10131443	T/C	NA	INTERGENIC	NA
Kabuli_wild_10220396	1399933736	*Ca_kabuli_Chr02*	10220396	T/C	NA	INTERGENIC	NA
Kabuli_wild_10294635	1399933737	*Ca_kabuli_Chr02*	10294635	T/C	Gene_Ca_17594	INTRON	ATPase,P-type,K/Mg/Cd/Cu/Zn/Na/Ca/Na/H-transporter
Kabuli_wild_10294712	1399933738	*Ca_kabuli_Chr02*	10294712	C/A	Gene_Ca_17594	NON_SYNONYMOUS_CODING	ATPase,P-type,K/Mg/Cd/Cu/Zn/Na/Ca/Na/H-transporter
Kabuli_wild_10540735	1399933739	*Ca_kabuli_Chr02*	10540735	C/T	Gene_Ca_17577	NON_SYNONYMOUS_CODING	NA

SNP IDs	NCBI dbSNP accession IDs (NCBI_SS#)	Chromosomes	Physical positions (bp)	SNPs	Gene accession IDs	Sequence components of genome	Putative functions
Kabuli_wild_10540812	1399933740	*Ca_kabuli_Chr02*	10540812	G/A	Gene_Ca_17577	NON_SYNONYMOUS_CODING	NA
Kabuli_wild_10705898	1399933741	*Ca_kabuli_Chr02*	10705898	C/A	Gene_Ca_17568	DOWNSTREAM	NA
Kabuli_wild_10705915	1399933742	*Ca_kabuli_Chr02*	10705915	C/T	Gene_Ca_17568	DOWNSTREAM	NA
Kabuli_wild_10705957	1399933743	*Ca_kabuli_Chr02*	10705957	C/A	Gene_Ca_17568	DOWNSTREAM	NA
Kabuli_wild_10705963	1399933744	*Ca_kabuli_Chr02*	10705963	C/T	Gene_Ca_17568	DOWNSTREAM	NA
Kabuli_wild_10705976	1399933745	*Ca_kabuli_Chr02*	10705976	T/C	Gene_Ca_17568	DOWNSTREAM	NA
Kabuli_wild_10705982	1399933746	*Ca_kabuli_Chr02*	10705982	C/T	Gene_Ca_17568	DOWNSTREAM	NA
Kabuli_wild_10760939	1399933747	*Ca_kabuli_Chr02*	10760939	G/A	NA	INTERGENIC	NA
Kabuli_wild_10761036	1399933748	*Ca_kabuli_Chr02*	10761036	C/G	NA	INTERGENIC	NA
Kabuli_wild_10852911	1399933749	*Ca_kabuli_Chr02*	10852911	T/C	NA	INTERGENIC	NA
Kabuli_wild_10852945	1399933750	*Ca_kabuli_Chr02*	10852945	C/T	NA	INTERGENIC	NA
Kabuli_wild_10852949	1399933751	*Ca_kabuli_Chr02*	10852949	C/T	NA	INTERGENIC	NA
Kabuli_wild_10942528	1399933752	*Ca_kabuli_Chr02*	10942528	A/G	Gene_Ca_17555	INTRON	5-formyltetrahydrofolatecyclo-ligase
Kabuli_wild_11064243	1399933753	*Ca_kabuli_Chr02*	11064243	G/A	NA	INTERGENIC	NA
Kabuli_wild_11121418	1399933754	*Ca_kabuli_Chr02*	11121418	C/T	Gene_Ca_17550	SYNONYMOUS_CODING	Protein kinase,catalytic domain
Kabuli_wild_11457037	1399933755	*Ca_kabuli_Chr02*	11457037	A/T	NA	INTERGENIC	NA
Kabuli_wild_11702730	1399933756	*Ca_kabuli_Chr02*	11702730	G/T	Gene_Ca_18774	INTRON	Helix-loop-helixDNA-binding domain
Kabuli_wild_11702738	1399933757	*Ca_kabuli_Chr02*	11702738	T/A	Gene_Ca_18774	INTRON	Helix-loop-helixDNA-binding domain
Kabuli_wild_11702740	1399933758	*Ca_kabuli_Chr02*	11702740	T/A	Gene_Ca_18774	INTRON	Helix-loop-helixDNA-binding domain
Kabuli_wild_11799459	1399933759	*Ca_kabuli_Chr02*	11799459	G/T	NA	INTERGENIC	NA
Kabuli_wild_11799472	1399933760	*Ca_kabuli_Chr02*	11799472	G/A	NA	INTERGENIC	NA
Kabuli_wild_11799510	1399933761	*Ca_kabuli_Chr02*	11799510	A/G	NA	INTERGENIC	NA
Kabuli_wild_11799555	1399933762	*Ca_kabuli_Chr02*	11799555	G/A	NA	INTERGENIC	NA
Kabuli_wild_11819827	1399933763	*Ca_kabuli_Chr02*	11819827	A/G	NA	INTERGENIC	NA
Kabuli_wild_11955578	1399933764	*Ca_kabuli_Chr02*	11955578	C/A	Gene_Ca_18763	INTRON	Transposase,Ptta/En/Spm,plant
Kabuli_wild_11955608	1399933765	*Ca_kabuli_Chr02*	11955608	C/A	Gene_Ca_18763	INTRON	Transposase,Ptta/En/Spm,plant
Kabuli_wild_12135192	1399933766	*Ca_kabuli_Chr02*	12135192	C/T	NA	INTERGENIC	NA
Kabuli_wild_12135216	1399933767	*Ca_kabuli_Chr02*	12135216	T/G	NA	INTERGENIC	NA
Kabuli_wild_12135242	1399933768	*Ca_kabuli_Chr02*	12135242	C/G	NA	INTERGENIC	NA
Kabuli_wild_12211028	1399933769	*Ca_kabuli_Chr02*	12211028	A/G	Gene_Ca_18057	NON_SYNONYMOUS_CODING	Peptidase C48, SUMO/Sentrin/Ubl1
Kabuli_wild_12211033	1399933770	*Ca_kabuli_Chr02*	12211033	C/T	Gene_Ca_18057	SYNONYMOUS_CODING	Peptidase C48, SUMO/Sentrin/Ubl1
Kabuli_wild_12211052	1399933771	*Ca_kabuli_Chr02*	12211052	C/T	Gene_Ca_18057	NON_SYNONYMOUS_CODING	Peptidase C48, SUMO/Sentrin/Ubl1
Kabuli_wild_12211128	1399933772	*Ca_kabuli_Chr02*	12211128	A/G	Gene_Ca_18057	SYNONYMOUS_CODING	Peptidase C48, SUMO/Sentrin/Ubl1
Kabuli_wild_12252370	1399933773	*Ca_kabuli_Chr02*	12252370	A/G	NA	INTERGENIC	NA
Kabuli_wild_12327894	1399933774	*Ca_kabuli_Chr02*	12327894	G/A	NA	INTERGENIC	NA
Kabuli_wild_12389083	1399933775	*Ca_kabuli_Chr02*	12389083	C/T	NA	INTERGENIC	NA
Kabuli_wild_12389100	1399933776	*Ca_kabuli_Chr02*	12389100	C/T	NA	INTERGENIC	NA
Kabuli_wild_12389109	1399933777	*Ca_kabuli_Chr02*	12389109	A/G	NA	INTERGENIC	NA
Kabuli_wild_12389111	1399933778	*Ca_kabuli_Chr02*	12389111	A/G	NA	INTERGENIC	NA
Kabuli_wild_12389112	1399933779	*Ca_kabuli_Chr02*	12389112	G/A	NA	INTERGENIC	NA
Kabuli_wild_12389161	1399933780	*Ca_kabuli_Chr02*	12389161	G/A	NA	INTERGENIC	NA
Kabuli_wild_12389166	1399933781	*Ca_kabuli_Chr02*	12389166	G/T	NA	INTERGENIC	NA
Kabuli_wild_12389168	1399933782	*Ca_kabuli_Chr02*	12389168	C/T	NA	INTERGENIC	NA
Kabuli_wild_12518246	1399933783	*Ca_kabuli_Chr02*	12518246	A/T	Gene_Ca_18078	INTRON	NA

SNP IDs	NCBI dbSNP accession IDs (NCBI_SS#)	Chromosomes	Physical positions (bp)	SNPs	Gene accession IDs	Sequence components of genome	Putative functions
Kabuli_wild_12528825	1399933784	*Ca_kabuli_Chr02*	12528825	C/G	Gene_Ca_18079	NON_SYNONYMOUS_CODING	Zinc finger,Dof-type
Kabuli_wild_12583471	1399933785	*Ca_kabuli_Chr02*	12583471	G/A	NA	INTERGENIC	NA
Kabuli_wild_12583487	1399933786	*Ca_kabuli_Chr02*	12583487	T/C	NA	INTERGENIC	NA
Kabuli_wild_12583488	1399933787	*Ca_kabuli_Chr02*	12583488	A/C	NA	INTERGENIC	NA
Kabuli_wild_12583504	1399933788	*Ca_kabuli_Chr02*	12583504	G/T	NA	INTERGENIC	NA
Kabuli_wild_12583517	1399933789	*Ca_kabuli_Chr02*	12583517	G/A	NA	INTERGENIC	NA
Kabuli_wild_12792903	1399933790	*Ca_kabuli_Chr02*	12792903	T/C	Gene_Ca_18093	INTRON	EF-HAND2
Kabuli_wild_12794438	1399933791	*Ca_kabuli_Chr02*	12794438	G/A	Gene_Ca_18093	NON_SYNONYMOUS_CODING	EF-HAND2
Kabuli_wild_13079445	1399933792	*Ca_kabuli_Chr02*	13079445	T/G	Gene_Ca_11686	SYNONYMOUS_CODING	Protein of unknown function DUF3110
Kabuli_wild_13312498	1399933793	*Ca_kabuli_Chr02*	13312498	T/C	NA	INTERGENIC	NA
Kabuli_wild_13355018	1399933794	*Ca_kabuli_Chr02*	13355018	T/G	NA	INTERGENIC	NA
Kabuli_wild_13355022	1399933795	*Ca_kabuli_Chr02*	13355022	G/T	NA	INTERGENIC	NA
Kabuli_wild_13355033	1399933796	*Ca_kabuli_Chr02*	13355033	A/T	NA	INTERGENIC	NA
Kabuli_wild_13355615	1399933797	*Ca_kabuli_Chr02*	13355615	T/G	NA	INTERGENIC	NA
Kabuli_wild_13434995	1399933798	*Ca_kabuli_Chr02*	13434995	A/C	Gene_Ca_11665	INTRON	Glutamyl-tRNA(Gln)amidotransferase,subunitB/E,N-terminal
Kabuli_wild_13437317	1399933799	*Ca_kabuli_Chr02*	13437317	A/G	Gene_Ca_11665	INTRON	Glutamyl-tRNA(Gln)amidotransferase,subunitB/E,N-terminal
Kabuli_wild_13529057	1399933800	*Ca_kabuli_Chr02*	13529057	G/A	NA	INTERGENIC	NA
Kabuli_wild_13782905	1399933801	*Ca_kabuli_Chr02*	13782905	G/C	Gene_Ca_11639	INTRON	Remorin,C-terminal
Kabuli_wild_13782906	1399933802	*Ca_kabuli_Chr02*	13782906	C/A	Gene_Ca_11639	INTRON	Remorin,C-terminal
Kabuli_wild_14136013	1399933803	*Ca_kabuli_Chr02*	14136013	T/G	Gene_Ca_11622	DOWNSTREAM	NA
Kabuli_wild_14136195	1399933804	*Ca_kabuli_Chr02*	14136195	A/C	Gene_Ca_11622	DOWNSTREAM	NA
Kabuli_wild_14136223	1399933805	*Ca_kabuli_Chr02*	14136223	A/C	Gene_Ca_11622	DOWNSTREAM	NA
Kabuli_wild_15234951	1399933806	*Ca_kabuli_Chr02*	15234951	C/A	Gene_Ca_18564	SYNONYMOUS_CODING	Protein of unknown function DUF1675
Kabuli_wild_15390662	1399933807	*Ca_kabuli_Chr02*	15390662	T/C	Gene_Ca_18558	INTRON	Protein kinase,catalytic domain
Kabuli_wild_15390673	1399933808	*Ca_kabuli_Chr02*	15390673	A/G	Gene_Ca_18558	INTRON	Protein kinase,catalytic domain
Kabuli_wild_15412962	1399933809	*Ca_kabuli_Chr02*	15412962	G/A	NA	INTERGENIC	NA
Kabuli_wild_15470128	1399933810	*Ca_kabuli_Chr02*	15470128	T/C	NA	INTERGENIC	NA
Kabuli_wild_15690131	1399933811	*Ca_kabuli_Chr02*	15690131	T/C	Gene_Ca_18540	NON_SYNONYMOUS_CODING	Alpha-1,4-glucan-proteinsynthase,UDP-forming
Kabuli_wild_16460097	1399933812	*Ca_kabuli_Chr02*	16460097	A/C	NA	INTERGENIC	NA
Kabuli_wild_16460129	1399933813	*Ca_kabuli_Chr02*	16460129	C/A	NA	INTERGENIC	NA
Kabuli_wild_16460142	1399933814	*Ca_kabuli_Chr02*	16460142	A/G	NA	INTERGENIC	NA
Kabuli_wild_16460174	1399933815	*Ca_kabuli_Chr02*	16460174	G/A	NA	INTERGENIC	NA
Kabuli_wild_16460175	1399933816	*Ca_kabuli_Chr02*	16460175	C/A	NA	INTERGENIC	NA
Kabuli_wild_16462089	1399933817	*Ca_kabuli_Chr02*	16462089	C/T	NA	INTERGENIC	NA
Kabuli_wild_16462095	1399933818	*Ca_kabuli_Chr02*	16462095	A/G	NA	INTERGENIC	NA
Kabuli_wild_16555733	1399933819	*Ca_kabuli_Chr02*	16555733	C/T	Gene_Ca_22041	INTRON	BSD
Kabuli_wild_16555790	1399933820	*Ca_kabuli_Chr02*	16555790	C/G	Gene_Ca_22041	INTRON	BSD
Kabuli_wild_16555835	1399933821	*Ca_kabuli_Chr02*	16555835	C/T	Gene_Ca_22041	INTRON	BSD
Kabuli_wild_16563117	1399933822	*Ca_kabuli_Chr02*	16563117	A/T	Gene_Ca_22041	INTRON	BSD
Kabuli_wild_16563121	1399933823	*Ca_kabuli_Chr02*	16563121	A/T	Gene_Ca_22041	INTRON	BSD
Kabuli_wild_16563123	1399933824	*Ca_kabuli_Chr02*	16563123	C/T	Gene_Ca_22041	INTRON	BSD
Kabuli_wild_16563125	1399933825	*Ca_kabuli_Chr02*	16563125	A/T	Gene_Ca_22041	INTRON	BSD
Kabuli_wild_16563133	1399933826	*Ca_kabuli_Chr02*	16563133	A/T	Gene_Ca_22041	INTRON	BSD
Kabuli_wild_16705119	1399933827	*Ca_kabuli_Chr02*	16705119	C/T	NA	INTERGENIC	NA

SNP IDs	NCBI dbSNP accession IDs (NCBI_SS#)	Chromosomes	Physical positions (bp)	SNPs	Gene accession IDs	Sequence components of genome	Putative functions
Kabuli_wild_17296609	1399933828	*Ca_kabuli_Chr02*	17296609	T/C	NA	INTERGENIC	NA
Kabuli_wild_17578623	1399933829	*Ca_kabuli_Chr02*	17578623	G/A	NA	INTERGENIC	NA
Kabuli_wild_17578628	1399933830	*Ca_kabuli_Chr02*	17578628	C/T	NA	INTERGENIC	NA
Kabuli_wild_17578633	1399933831	*Ca_kabuli_Chr02*	17578633	C/G	NA	INTERGENIC	NA
Kabuli_wild_17578666	1399933832	*Ca_kabuli_Chr02*	17578666	T/A	NA	INTERGENIC	NA
Kabuli_wild_17578676	1399933833	*Ca_kabuli_Chr02*	17578676	T/C	NA	INTERGENIC	NA
Kabuli_wild_17578677	1399933834	*Ca_kabuli_Chr02*	17578677	G/C	NA	INTERGENIC	NA
Kabuli_wild_17578689	1399933835	*Ca_kabuli_Chr02*	17578689	G/A	NA	INTERGENIC	NA
Kabuli_wild_17578695	1399933836	*Ca_kabuli_Chr02*	17578695	G/A	NA	INTERGENIC	NA
Kabuli_wild_17578701	1399933837	*Ca_kabuli_Chr02*	17578701	A/C	NA	INTERGENIC	NA
Kabuli_wild_17578706	1399933838	*Ca_kabuli_Chr02*	17578706	A/T	NA	INTERGENIC	NA
Kabuli_wild_17578707	1399933839	*Ca_kabuli_Chr02*	17578707	T/A	NA	INTERGENIC	NA
Kabuli_wild_17578740	1399933840	*Ca_kabuli_Chr02*	17578740	A/G	NA	INTERGENIC	NA
Kabuli_wild_17681915	1399933841	*Ca_kabuli_Chr02*	17681915	C/T	Gene_Ca_15998	DOWNSTREAM	Transposase,Ptta/En/Spm,plant
Kabuli_wild_17789421	1399933842	*Ca_kabuli_Chr02*	17789421	A/G	Gene_Ca_15993	INTRON	Protein kinase,catalytic domain
Kabuli_wild_17916096	1399933843	*Ca_kabuli_Chr02*	17916096	C/T	NA	INTERGENIC	NA
Kabuli_wild_17916100	1399933844	*Ca_kabuli_Chr02*	17916100	G/T	NA	INTERGENIC	NA
Kabuli_wild_17916119	1399933845	*Ca_kabuli_Chr02*	17916119	T/C	NA	INTERGENIC	NA
Kabuli_wild_17916125	1399933846	*Ca_kabuli_Chr02*	17916125	C/T	NA	INTERGENIC	NA
Kabuli_wild_17916132	1399933847	*Ca_kabuli_Chr02*	17916132	C/T	NA	INTERGENIC	NA
Kabuli_wild_17916148	1399933848	*Ca_kabuli_Chr02*	17916148	G/A	NA	INTERGENIC	NA
Kabuli_wild_17916158	1399933849	*Ca_kabuli_Chr02*	17916158	G/A	NA	INTERGENIC	NA
Kabuli_wild_18312200	1399933850	*Ca_kabuli_Chr02*	18312200	T/C	NA	INTERGENIC	NA
Kabuli_wild_18355889	1399933851	*Ca_kabuli_Chr02*	18355889	G/A	NA	INTERGENIC	NA
Kabuli_wild_18395410	1399933852	*Ca_kabuli_Chr02*	18395410	T/C	Gene_Ca_15975	DOWNSTREAM	NA
Kabuli_wild_18581752	1399933853	*Ca_kabuli_Chr02*	18581752	A/G	NA	INTERGENIC	NA
Kabuli_wild_18631522	1399933854	*Ca_kabuli_Chr02*	18631522	A/C	Gene_Ca_15968	UPSTREAM	Zinc/ironpermease
Kabuli_wild_18671488	1399933855	*Ca_kabuli_Chr02*	18671488	A/G	NA	INTERGENIC	NA
Kabuli_wild_18671574	1399933856	*Ca_kabuli_Chr02*	18671574	G/A	NA	INTERGENIC	NA
Kabuli_wild_18671618	1399933857	*Ca_kabuli_Chr02*	18671618	T/G	NA	INTERGENIC	NA
Kabuli_wild_18671637	1399933858	*Ca_kabuli_Chr02*	18671637	G/A	NA	INTERGENIC	NA
Kabuli_wild_18671645	1399933859	*Ca_kabuli_Chr02*	18671645	C/T	NA	INTERGENIC	NA
Kabuli_wild_18671666	1399933860	*Ca_kabuli_Chr02*	18671666	C/T	NA	INTERGENIC	NA
Kabuli_wild_18671672	1399933861	*Ca_kabuli_Chr02*	18671672	T/A	NA	INTERGENIC	NA
Kabuli_wild_18671680	1399933862	*Ca_kabuli_Chr02*	18671680	C/T	NA	INTERGENIC	NA
Kabuli_wild_18671692	1399933863	*Ca_kabuli_Chr02*	18671692	C/A	NA	INTERGENIC	NA
Kabuli_wild_18671696	1399933864	*Ca_kabuli_Chr02*	18671696	G/A	NA	INTERGENIC	NA
Kabuli_wild_18671723	1399933865	*Ca_kabuli_Chr02*	18671723	C/G	NA	INTERGENIC	NA
Kabuli_wild_18671746	1399933866	*Ca_kabuli_Chr02*	18671746	C/T	NA	INTERGENIC	NA
Kabuli_wild_18671756	1399933867	*Ca_kabuli_Chr02*	18671756	T/A	NA	INTERGENIC	NA
Kabuli_wild_18671765	1399933868	*Ca_kabuli_Chr02*	18671765	C/A	NA	INTERGENIC	NA
Kabuli_wild_18671791	1399933869	*Ca_kabuli_Chr02*	18671791	A/C	NA	INTERGENIC	NA
Kabuli_wild_18671830	1399933870	*Ca_kabuli_Chr02*	18671830	A/G	NA	INTERGENIC	NA
Kabuli_wild_18712473	1399933871	*Ca_kabuli_Chr02*	18712473	C/T	NA	INTERGENIC	NA

SNP IDs	NCBI dbSNP accession IDs (NCBI_SS#)	Chromosomes	Physical positions (bp)	SNPs	Gene accession IDs	Sequence components of genome	Putative functions
Kabuli_wild_18712505	1399933872	*Ca_kabuli_Chr02*	18712505	T/A	NA	INTERGENIC	NA
Kabuli_wild_18823154	1399933873	*Ca_kabuli_Chr02*	18823154	T/C	NA	INTERGENIC	NA
Kabuli_wild_19395571	1399933874	*Ca_kabuli_Chr02*	19395571	G/T	NA	INTERGENIC	NA
Kabuli_wild_19395619	1399933875	*Ca_kabuli_Chr02*	19395619	C/T	NA	INTERGENIC	NA
Kabuli_wild_19653215	1399933876	*Ca_kabuli_Chr02*	19653215	A/G	Gene_Ca_24537	SYNONYMOUS_CODING	Aminotransferase-like,plant mobile domain
Kabuli_wild_19765839	1399933877	*Ca_kabuli_Chr02*	19765839	T/C	NA	INTERGENIC	NA
Kabuli_wild_19765846	1399933878	*Ca_kabuli_Chr02*	19765846	G/A	NA	INTERGENIC	NA
Kabuli_wild_19765873	1399933879	*Ca_kabuli_Chr02*	19765873	A/G	NA	INTERGENIC	NA
Kabuli_wild_19765908	1399933880	*Ca_kabuli_Chr02*	19765908	T/C	NA	INTERGENIC	NA
Kabuli_wild_19765917	1399933881	*Ca_kabuli_Chr02*	19765917	A/T	NA	INTERGENIC	NA
Kabuli_wild_20392224	1399933882	*Ca_kabuli_Chr02*	20392224	A/G	Gene_Ca_25924	SYNONYMOUS_CODING	NA
Kabuli_wild_20762180	1399933883	*Ca_kabuli_Chr02*	20762180	T/A	Gene_Ca_23762	NON_SYNONYMOUS_CODING	NA
Kabuli_wild_20762199	1399933884	*Ca_kabuli_Chr02*	20762199	T/C	Gene_Ca_23762	SYNONYMOUS_CODING	NA
Kabuli_wild_20762205	1399933885	*Ca_kabuli_Chr02*	20762205	A/T	Gene_Ca_23762	SYNONYMOUS_CODING	NA
Kabuli_wild_20762209	1399933886	*Ca_kabuli_Chr02*	20762209	T/C	Gene_Ca_23762	NON_SYNONYMOUS_CODING	NA
Kabuli_wild_20762222	1399933887	*Ca_kabuli_Chr02*	20762222	C/T	Gene_Ca_23762	NON_SYNONYMOUS_CODING	NA
Kabuli_wild_20762223	1399933888	*Ca_kabuli_Chr02*	20762223	G/A	Gene_Ca_23762	SYNONYMOUS_CODING	NA
Kabuli_wild_21000433	1399933889	*Ca_kabuli_Chr02*	21000433	C/T	NA	INTERGENIC	NA
Kabuli_wild_21000463	1399933890	*Ca_kabuli_Chr02*	21000463	C/T	NA	INTERGENIC	NA
Kabuli_wild_21000466	1399933891	*Ca_kabuli_Chr02*	21000466	C/T	NA	INTERGENIC	NA
Kabuli_wild_21000498	1399933892	*Ca_kabuli_Chr02*	21000498	A/C	NA	INTERGENIC	NA
Kabuli_wild_21000509	1399933893	*Ca_kabuli_Chr02*	21000509	C/T	NA	INTERGENIC	NA
Kabuli_wild_21159203	1399933894	*Ca_kabuli_Chr02*	21159203	C/A	NA	INTERGENIC	NA
Kabuli_wild_21159222	1399933895	*Ca_kabuli_Chr02*	21159222	T/C	NA	INTERGENIC	NA
Kabuli_wild_21159228	1399933896	*Ca_kabuli_Chr02*	21159228	C/T	NA	INTERGENIC	NA
Kabuli_wild_21159254	1399933897	*Ca_kabuli_Chr02*	21159254	C/T	NA	INTERGENIC	NA
Kabuli_wild_21159275	1399933898	*Ca_kabuli_Chr02*	21159275	G/A	NA	INTERGENIC	NA
Kabuli_wild_21159306	1399933899	*Ca_kabuli_Chr02*	21159306	C/T	NA	INTERGENIC	NA
Kabuli_wild_21372522	1399933900	*Ca_kabuli_Chr02*	21372522	C/T	Gene_Ca_24629	INTRON	Heat shock protein DnaJ, cysteine-richdomain
Kabuli_wild_21849839	1399933901	*Ca_kabuli_Chr02*	21849839	C/G	Gene_Ca_23059	INTRON	NA
Kabuli_wild_21849874	1399933902	*Ca_kabuli_Chr02*	21849874	A/T	Gene_Ca_23059	INTRON	NA
Kabuli_wild_21965347	1399933903	*Ca_kabuli_Chr02*	21965347	C/A	NA	INTERGENIC	NA
Kabuli_wild_21965365	1399933904	*Ca_kabuli_Chr02*	21965365	T/C	NA	INTERGENIC	NA
Kabuli_wild_21965371	1399933905	*Ca_kabuli_Chr02*	21965371	G/A	NA	INTERGENIC	NA
Kabuli_wild_21965404	1399933906	*Ca_kabuli_Chr02*	21965404	T/G	NA	INTERGENIC	NA
Kabuli_wild_21965423	1399933907	*Ca_kabuli_Chr02*	21965423	A/G	NA	INTERGENIC	NA
Kabuli_wild_22112434	1399933908	*Ca_kabuli_Chr02*	22112434	A/G	NA	INTERGENIC	NA
Kabuli_wild_22158496	1399933909	*Ca_kabuli_Chr02*	22158496	C/A	Gene_Ca_15007	NON_SYNONYMOUS_CODING	Peptidase C48, SUMO/Sentrin/Ubl1
Kabuli_wild_22158517	1399933910	*Ca_kabuli_Chr02*	22158517	G/A	Gene_Ca_15007	NON_SYNONYMOUS_CODING	Peptidase C48, SUMO/Sentrin/Ubl1
Kabuli_wild_22158518	1399933911	*Ca_kabuli_Chr02*	22158518	C/T	Gene_Ca_15007	NON_SYNONYMOUS_CODING	Peptidase C48, SUMO/Sentrin/Ubl1
Kabuli_wild_22158520	1399933912	*Ca_kabuli_Chr02*	22158520	G/A	Gene_Ca_15007	NON_SYNONYMOUS_CODING	Peptidase C48, SUMO/Sentrin/Ubl1
Kabuli_wild_22158543	1399933913	*Ca_kabuli_Chr02*	22158543	T/A	Gene_Ca_15007	SYNONYMOUS_CODING	Peptidase C48, SUMO/Sentrin/Ubl1
Kabuli_wild_22158554	1399933914	*Ca_kabuli_Chr02*	22158554	C/T	Gene_Ca_15007	NON_SYNONYMOUS_CODING	Peptidase C48, SUMO/Sentrin/Ubl1
Kabuli_wild_22158578	1399933915	*Ca_kabuli_Chr02*	22158578	T/A	Gene_Ca_15007	NON_SYNONYMOUS_CODING	Peptidase C48, SUMO/Sentrin/Ubl1

SNP IDs	NCBI dbSNP accession IDs (NCBI_SS#)	Chromosomes	Physical positions (bp)	SNPs	Gene accession IDs	Sequence components of genome	Putative functions
Kabuli_wild_22158602	1399933916	*Ca_kabuli_Chr02*	22158602	C/T	Gene_Ca_15007	NON_SYNONYMOUS_CODING	Peptidase C48, SUMO/Sentrin/Ubl1
Kabuli_wild_22158604	1399933917	*Ca_kabuli_Chr02*	22158604	G/A	Gene_Ca_15007	NON_SYNONYMOUS_CODING	Peptidase C48, SUMO/Sentrin/Ubl1
Kabuli_wild_22190621	1399933918	*Ca_kabuli_Chr02*	22190621	C/G	NA	INTERGENIC	NA
Kabuli_wild_22190743	1399933919	*Ca_kabuli_Chr02*	22190743	C/T	NA	INTERGENIC	NA
Kabuli_wild_22190748	1399933920	*Ca_kabuli_Chr02*	22190748	G/T	NA	INTERGENIC	NA
Kabuli_wild_22190751	1399933921	*Ca_kabuli_Chr02*	22190751	C/T	NA	INTERGENIC	NA
Kabuli_wild_22190753	1399933922	*Ca_kabuli_Chr02*	22190753	C/T	NA	INTERGENIC	NA
Kabuli_wild_22190776	1399933923	*Ca_kabuli_Chr02*	22190776	G/A	NA	INTERGENIC	NA
Kabuli_wild_22190815	1399933924	*Ca_kabuli_Chr02*	22190815	C/T	NA	INTERGENIC	NA
Kabuli_wild_22347405	1399933925	*Ca_kabuli_Chr02*	22347405	C/T	Gene_Ca_15004	INTRON	NA
Kabuli_wild_22457183	1399933926	*Ca_kabuli_Chr02*	22457183	C/T	NA	INTERGENIC	NA
Kabuli_wild_22457215	1399933927	*Ca_kabuli_Chr02*	22457215	G/A	NA	INTERGENIC	NA
Kabuli_wild_22457216	1399933928	*Ca_kabuli_Chr02*	22457216	C/T	NA	INTERGENIC	NA
Kabuli_wild_22457233	1399933929	*Ca_kabuli_Chr02*	22457233	C/T	NA	INTERGENIC	NA
Kabuli_wild_22457234	1399933930	*Ca_kabuli_Chr02*	22457234	G/A	NA	INTERGENIC	NA
Kabuli_wild_22457237	1399933931	*Ca_kabuli_Chr02*	22457237	G/A	NA	INTERGENIC	NA
Kabuli_wild_22457257	1399933932	*Ca_kabuli_Chr02*	22457257	G/A	NA	INTERGENIC	NA
Kabuli_wild_22457263	1399933933	*Ca_kabuli_Chr02*	22457263	G/A	NA	INTERGENIC	NA
Kabuli_wild_22457345	1399933934	*Ca_kabuli_Chr02*	22457345	C/T	NA	INTERGENIC	NA
Kabuli_wild_22457347	1399933935	*Ca_kabuli_Chr02*	22457347	G/A	NA	INTERGENIC	NA
Kabuli_wild_22457360	1399933936	*Ca_kabuli_Chr02*	22457360	C/T	NA	INTERGENIC	NA
Kabuli_wild_22483000	1399933937	*Ca_kabuli_Chr02*	22483000	C/T	NA	INTERGENIC	NA
Kabuli_wild_22483003	1399933938	*Ca_kabuli_Chr02*	22483003	A/C	NA	INTERGENIC	NA
Kabuli_wild_22483019	1399933939	*Ca_kabuli_Chr02*	22483019	A/T	NA	INTERGENIC	NA
Kabuli_wild_22483022	1399933940	*Ca_kabuli_Chr02*	22483022	C/G	NA	INTERGENIC	NA
Kabuli_wild_22483052	1399933941	*Ca_kabuli_Chr02*	22483052	C/T	NA	INTERGENIC	NA
Kabuli_wild_22483067	1399933942	*Ca_kabuli_Chr02*	22483067	G/A	NA	INTERGENIC	NA
Kabuli_wild_22483072	1399933943	*Ca_kabuli_Chr02*	22483072	C/T	NA	INTERGENIC	NA
Kabuli_wild_22536226	1399933944	*Ca_kabuli_Chr02*	22536226	T/C	Gene_Ca_14996	SYNONYMOUS_CODING	NA
Kabuli_wild_22702616	1399933945	*Ca_kabuli_Chr02*	22702616	T/C	Gene_Ca_14990	INTRON	Cyclin,C-terminal
Kabuli_wild_22703091	1399933946	*Ca_kabuli_Chr02*	22703091	A/C	Gene_Ca_14990	INTRON	Cyclin,C-terminal
Kabuli_wild_22720115	1399933947	*Ca_kabuli_Chr02*	22720115	A/G	Gene_Ca_14990	NON_SYNONYMOUS_CODING	Cyclin,C-terminal
Kabuli_wild_22820588	1399933948	*Ca_kabuli_Chr02*	22820588	A/C	Gene_Ca_14985	INTRON	PeptidaseC19,ubiquitincarboxyl-terminalhydrolase2
Kabuli_wild_22820616	1399933949	*Ca_kabuli_Chr02*	22820616	C/G	Gene_Ca_14985	INTRON	Rho GTPase-activating protein domain
Kabuli_wild_22849000	1399933950	*Ca_kabuli_Chr02*	22849000	A/G	Gene_Ca_14984	SYNONYMOUS_CODING	Domain of unknown function DUF296
Kabuli_wild_22897647	1399933951	*Ca_kabuli_Chr02*	22897647	T/C	NA	INTERGENIC	NA
Kabuli_wild_22897694	1399933952	*Ca_kabuli_Chr02*	22897694	A/G	NA	INTERGENIC	NA
Kabuli_wild_22943923	1399933953	*Ca_kabuli_Chr02*	22943923	G/A	NA	INTERGENIC	NA
Kabuli_wild_23111588	1399933954	*Ca_kabuli_Chr02*	23111588	T/C	NA	INTERGENIC	NA
Kabuli_wild_23193577	1399933955	*Ca_kabuli_Chr02*	23193577	A/G	NA	INTERGENIC	NA
Kabuli_wild_23193620	1399933956	*Ca_kabuli_Chr02*	23193620	C/A	NA	INTERGENIC	NA
Kabuli_wild_23193632	1399933957	*Ca_kabuli_Chr02*	23193632	C/T	NA	INTERGENIC	NA
Kabuli_wild_23193680	1399933958	*Ca_kabuli_Chr02*	23193680	G/T	NA	INTERGENIC	NA
Kabuli_wild_23219939	1399933959	*Ca_kabuli_Chr02*	23219939	C/A	Gene_Ca_14970	NON_SYNONYMOUS_CODING	Protein kinase,catalytic domain

SNP IDs	NCBI dbSNP accession IDs (NCBI_SS#)	Chromosomes	Physical positions (bp)	SNPs	Gene accession IDs	Sequence components of genome	Putative functions
Kabuli_wild_23247801	1399933960	*Ca_kabuli_Chr02*	23247801	C/T	NA	INTERGENIC	NA
Kabuli_wild_23282374	1399933961	*Ca_kabuli_Chr02*	23282374	T/C	Gene_Ca_14968	DOWNSTREAM	Zinc finger,GATA-type
Kabuli_wild_23329424	1399933962	*Ca_kabuli_Chr02*	23329424	C/T	NA	INTERGENIC	NA
Kabuli_wild_23329440	1399933963	*Ca_kabuli_Chr02*	23329440	G/A	NA	INTERGENIC	NA
Kabuli_wild_23346297	1399933964	*Ca_kabuli_Chr02*	23346297	A/C	Gene_Ca_14963	INTRON	Domain of unknown function FMP27,domain-4
Kabuli_wild_23429747	1399933965	*Ca_kabuli_Chr02*	23429747	A/C	Gene_Ca_14961	SYNONYMOUS_CODING	HECT
Kabuli_wild_23439966	1399933966	*Ca_kabuli_Chr02*	23439966	T/C	Gene_Ca_14960	NON_SYNONYMOUS_CODING	NA
Kabuli_wild_23442057	1399933967	*Ca_kabuli_Chr02*	23442057	T/C	Gene_Ca_14960	NON_SYNONYMOUS_CODING	Ubiquitin-associated/translation elongation factor EF1B, N-terminal
Kabuli_wild_23797957	1399933968	*Ca_kabuli_Chr02*	23797957	C/T	NA	INTERGENIC	NA
Kabuli_wild_23797987	1399933969	*Ca_kabuli_Chr02*	23797987	T/C	NA	INTERGENIC	NA
Kabuli_wild_23798081	1399933970	*Ca_kabuli_Chr02*	23798081	T/C	NA	INTERGENIC	NA
Kabuli_wild_23798111	1399933971	*Ca_kabuli_Chr02*	23798111	G/T	NA	INTERGENIC	NA
Kabuli_wild_23819055	1399933972	*Ca_kabuli_Chr02*	23819055	C/A	Gene_Ca_14945	NON_SYNONYMOUS_CODING	NA
Kabuli_wild_23910254	1399933973	*Ca_kabuli_Chr02*	23910254	T/C	NA	INTERGENIC	NA
Kabuli_wild_23923393	1399933974	*Ca_kabuli_Chr02*	23923393	G/C	NA	INTERGENIC	NA
Kabuli_wild_24048087	1399933975	*Ca_kabuli_Chr02*	24048087	A/C	Gene_Ca_21673	INTRON	PUCC protein
Kabuli_wild_24226527	1399933976	*Ca_kabuli_Chr02*	24226527	T/C	Gene_Ca_21666	INTRON	SNF2-related
Kabuli_wild_24226618	1399933977	*Ca_kabuli_Chr02*	24226618	G/T	Gene_Ca_21666	INTRON	SNF2-related
Kabuli_wild_24226892	1399933978	*Ca_kabuli_Chr02*	24226892	C/A	Gene_Ca_21666	INTRON	SNF2-related
Kabuli_wild_24353119	1399933979	*Ca_kabuli_Chr02*	24353119	A/C	NA	INTERGENIC	NA
Kabuli_wild_24353120	1399933980	*Ca_kabuli_Chr02*	24353120	G/C	NA	INTERGENIC	NA
Kabuli_wild_24353123	1399933981	*Ca_kabuli_Chr02*	24353123	C/G	NA	INTERGENIC	NA
Kabuli_wild_24407365	1399933982	*Ca_kabuli_Chr02*	24407365	C/T	NA	INTERGENIC	NA
Kabuli_wild_24511053	1399933983	*Ca_kabuli_Chr02*	24511053	G/A	NA	INTERGENIC	NA
Kabuli_wild_24511114	1399933984	*Ca_kabuli_Chr02*	24511114	G/A	NA	INTERGENIC	NA
Kabuli_wild_24511116	1399933985	*Ca_kabuli_Chr02*	24511116	C/A	NA	INTERGENIC	NA
Kabuli_wild_24511119	1399933986	*Ca_kabuli_Chr02*	24511119	T/G	NA	INTERGENIC	NA
Kabuli_wild_24511121	1399933987	*Ca_kabuli_Chr02*	24511121	G/A	NA	INTERGENIC	NA
Kabuli_wild_24709295	1399933988	*Ca_kabuli_Chr02*	24709295	G/A	Gene_Ca_22892	INTRON	DNA mismatch repair protein MutS,C-terminal domain
Kabuli_wild_24741195	1399933989	*Ca_kabuli_Chr02*	24741195	C/T	NA	INTERGENIC	NA
Kabuli_wild_24817895	1399933990	*Ca_kabuli_Chr02*	24817895	C/T	NA	INTERGENIC	NA
Kabuli_wild_24817921	1399933991	*Ca_kabuli_Chr02*	24817921	G/A	NA	INTERGENIC	NA
Kabuli_wild_24817928	1399933992	*Ca_kabuli_Chr02*	24817928	G/A	NA	INTERGENIC	NA
Kabuli_wild_24817934	1399933993	*Ca_kabuli_Chr02*	24817934	A/G	NA	INTERGENIC	NA
Kabuli_wild_24817940	1399933994	*Ca_kabuli_Chr02*	24817940	G/A	NA	INTERGENIC	NA
Kabuli_wild_24817945	1399933995	*Ca_kabuli_Chr02*	24817945	C/T	NA	INTERGENIC	NA
Kabuli_wild_24817953	1399933996	*Ca_kabuli_Chr02*	24817953	C/A	NA	INTERGENIC	NA
Kabuli_wild_24817957	1399933997	*Ca_kabuli_Chr02*	24817957	G/A	NA	INTERGENIC	NA
Kabuli_wild_25022971	1399933998	*Ca_kabuli_Chr02*	25022971	A/G	Gene_Ca_14236	NON_SYNONYMOUS_CODING	Zinc finger,B-box
Kabuli_wild_25163062	1399933999	*Ca_kabuli_Chr02*	25163062	C/G	Gene_Ca_14248	INTRON	Glycosi dehydrolase,family 3, N-terminal
Kabuli_wild_25317391	1399934000	*Ca_kabuli_Chr02*	25317391	A/G	Gene_Ca_14265	SYNONYMOUS_CODING	ABC transporter-like
Kabuli_wild_25318862	1399934001	*Ca_kabuli_Chr02*	25318862	A/T	Gene_Ca_14265	INTRON	ABC transporter-like
Kabuli_wild_25493973	1399934002	*Ca_kabuli_Chr02*	25493973	T/G	Gene_Ca_14279	SYNONYMOUS_CODING	ChaperoninClpA/B
Kabuli_wild_25508605	1399934003	*Ca_kabuli_Chr02*	25508605	T/C	Gene_Ca_14280	NON_SYNONYMOUS_CODING	Zinc finger,CCCH-type

SNP IDs	NCBI dbSNP accession IDs (NCBI_SS#)	Chromosomes	Physical positions (bp)	SNPs	Gene accession IDs	Sequence components of genome	Putative functions
Kabuli_wild_25508679	1399934004	*Ca_kabuli_Chr02*	25508679	A/G	Gene_Ca_14280	NON_SYNONYMOUS_CODING	Zinc finger,CCCH-type
Kabuli_wild_25725688	1399934005	*Ca_kabuli_Chr02*	25725688	G/A	NA	INTERGENIC	NA
Kabuli_wild_25725726	1399934006	*Ca_kabuli_Chr02*	25725726	C/A	NA	INTERGENIC	NA
Kabuli_wild_25725748	1399934007	*Ca_kabuli_Chr02*	25725748	T/C	NA	INTERGENIC	NA
Kabuli_wild_25725816	1399934008	*Ca_kabuli_Chr02*	25725816	G/T	NA	INTERGENIC	NA
Kabuli_wild_25751994	1399934009	*Ca_kabuli_Chr02*	25751994	T/C	NA	INTERGENIC	NA
Kabuli_wild_25752001	1399934010	*Ca_kabuli_Chr02*	25752001	C/G	NA	INTERGENIC	NA
Kabuli_wild_25788209	1399934011	*Ca_kabuli_Chr02*	25788209	A/G	Gene_Ca_14295	INTRON	RNA recognition motif domain
Kabuli_wild_25930490	1399934012	*Ca_kabuli_Chr02*	25930490	G/A	Gene_Ca_14304	NON_SYNONYMOUS_CODING	Phosphoglycerate/bisphosphoglyceratemutase,activesite
Kabuli_wild_25941107	1399934013	*Ca_kabuli_Chr02*	25941107	T/A	Gene_Ca_14304	INTRON	Phosphoglycerate/bisphosphoglyceratemutase,activesite
Kabuli_wild_25941128	1399934014	*Ca_kabuli_Chr02*	25941128	A/T	Gene_Ca_14304	INTRON	Phosphoglycerate/bisphosphoglyceratemutase,activesite
Kabuli_wild_25941178	1399934015	*Ca_kabuli_Chr02*	25941178	C/T	Gene_Ca_14304	INTRON	Phosphoglycerate/bisphosphoglyceratemutase,activesite
Kabuli_wild_26035894	1399934016	*Ca_kabuli_Chr02*	26035894	C/T	Gene_Ca_14310	SYNONYMOUS_CODING	NA
Kabuli_wild_26051667	1399934017	*Ca_kabuli_Chr02*	26051667	T/G	Gene_Ca_14311	UPSTREAM	Protein kinase,catalytic domain
Kabuli_wild_26092881	1399934018	*Ca_kabuli_Chr02*	26092881	C/G	NA	INTERGENIC	NA
Kabuli_wild_26092913	1399934019	*Ca_kabuli_Chr02*	26092913	C/A	NA	INTERGENIC	NA
Kabuli_wild_26092928	1399934020	*Ca_kabuli_Chr02*	26092928	T/G	NA	INTERGENIC	NA
Kabuli_wild_26121491	1399934021	*Ca_kabuli_Chr02*	26121491	T/G	Gene_Ca_14316	SYNONYMOUS_CODING	NA
Kabuli_wild_26121495	1399934022	*Ca_kabuli_Chr02*	26121495	A/C	Gene_Ca_14316	SYNONYMOUS_CODING	NA
Kabuli_wild_26457480	1399934023	*Ca_kabuli_Chr02*	26457480	A/G	Gene_Ca_17247	INTRON	Metallo-dependentphosphatase
Kabuli_wild_26461867	1399934024	*Ca_kabuli_Chr02*	26461867	C/G	Gene_Ca_17247	NON_SYNONYMOUS_CODING	Metallo-dependentphosphatase
Kabuli_wild_26486039	1399934025	*Ca_kabuli_Chr02*	26486039	G/T	NA	INTERGENIC	NA
Kabuli_wild_26486125	1399934026	*Ca_kabuli_Chr02*	26486125	G/A	NA	INTERGENIC	NA
Kabuli_wild_26502153	1399934027	*Ca_kabuli_Chr02*	26502153	T/C	NA	INTERGENIC	NA
Kabuli_wild_26503263	1399934028	*Ca_kabuli_Chr02*	26503263	A/G	NA	INTERGENIC	NA
Kabuli_wild_26503308	1399934029	*Ca_kabuli_Chr02*	26503308	T/G	NA	INTERGENIC	NA
Kabuli_wild_26567054	1399934030	*Ca_kabuli_Chr02*	26567054	T/G	NA	INTERGENIC	NA
Kabuli_wild_26704451	1399934031	*Ca_kabuli_Chr02*	26704451	A/G	Gene_Ca_17261	DOWNSTREAM	NA
Kabuli_wild_26704515	1399934032	*Ca_kabuli_Chr02*	26704515	A/C	Gene_Ca_17261	DOWNSTREAM	NA
Kabuli_wild_26706782	1399934033	*Ca_kabuli_Chr02*	26706782	G/A	NA	INTERGENIC	NA
Kabuli_wild_26706849	1399934034	*Ca_kabuli_Chr02*	26706849	A/G	NA	INTERGENIC	NA
Kabuli_wild_26771724	1399934035	*Ca_kabuli_Chr02*	26771724	T/C	NA	INTERGENIC	NA
Kabuli_wild_26994834	1399934036	*Ca_kabuli_Chr02*	26994834	A/T	NA	INTERGENIC	NA
Kabuli_wild_26994861	1399934037	*Ca_kabuli_Chr02*	26994861	T/A	NA	INTERGENIC	NA
Kabuli_wild_26994912	1399934038	*Ca_kabuli_Chr02*	26994912	C/T	NA	INTERGENIC	NA
Kabuli_wild_26994929	1399934039	*Ca_kabuli_Chr02*	26994929	A/T	NA	INTERGENIC	NA
Kabuli_wild_27044272	1399934040	*Ca_kabuli_Chr02*	27044272	T/C	Gene_Ca_15635	INTRON	NA
Kabuli_wild_27057582	1399934041	*Ca_kabuli_Chr02*	27057582	C/T	Gene_Ca_15636	INTRON	NA
Kabuli_wild_27063749	1399934042	*Ca_kabuli_Chr02*	27063749	C/A	Gene_Ca_15636	NON_SYNONYMOUS_CODING	NA
Kabuli_wild_27081030	1399934043	*Ca_kabuli_Chr02*	27081030	G/A	NA	INTERGENIC	NA
Kabuli_wild_27134560	1399934044	*Ca_kabuli_Chr02*	27134560	G/T	NA	INTERGENIC	NA
Kabuli_wild_27134574	1399934045	*Ca_kabuli_Chr02*	27134574	A/G	NA	INTERGENIC	NA
Kabuli_wild_27172582	1399934046	*Ca_kabuli_Chr02*	27172582	G/A	NA	INTERGENIC	NA
Kabuli_wild_27182855	1399934047	*Ca_kabuli_Chr02*	27182855	T/C	Gene_Ca_15643	DOWNSTREAM	Protein kinase,catalytic domain

SNP IDs	NCBI dbSNP accession IDs (NCBI_SS#)	Chromosomes	Physical positions (bp)	SNPs	Gene accession IDs	Sequence components of genome	Putative functions
Kabuli_wild_27703400	1399934048	*Ca_kabuli_Chr02*	27703400	A/C	Gene_Ca_15672	SYNONYMOUS_CODING	Ubiquitin-conjugating enzyme, E2
Kabuli_wild_27724425	1399934049	*Ca_kabuli_Chr02*	27724425	G/T	Gene_Ca_15674	SYNONYMOUS_CODING	BTB/POZ-like
Kabuli_wild_27766561	1399934050	*Ca_kabuli_Chr02*	27766561	T/A	Gene_Ca_15677	NON_SYNONYMOUS_CODING	Cation efflux protein
Kabuli_wild_27766594	1399934051	*Ca_kabuli_Chr02*	27766594	C/A	Gene_Ca_15677	NON_SYNONYMOUS_CODING	Cation efflux protein
Kabuli_wild_27766605	1399934052	*Ca_kabuli_Chr02*	27766605	A/C	Gene_Ca_15677	SYNONYMOUS_CODING	Cation efflux protein
Kabuli_wild_27831355	1399934053	*Ca_kabuli_Chr02*	27831355	A/G	Gene_Ca_15681	INTRON	ABC transporter,trans membrane domain
Kabuli_wild_27835825	1399934054	*Ca_kabuli_Chr02*	27835825	T/A	NA	INTERGENIC	NA
Kabuli_wild_27835832	1399934055	*Ca_kabuli_Chr02*	27835832	T/C	NA	INTERGENIC	NA
Kabuli_wild_27835837	1399934056	*Ca_kabuli_Chr02*	27835837	C/T	NA	INTERGENIC	NA
Kabuli_wild_27835843	1399934057	*Ca_kabuli_Chr02*	27835843	C/A	NA	INTERGENIC	NA
Kabuli_wild_27835846	1399934058	*Ca_kabuli_Chr02*	27835846	A/G	NA	INTERGENIC	NA
Kabuli_wild_27835850	1399934059	*Ca_kabuli_Chr02*	27835850	G/A	NA	INTERGENIC	NA
Kabuli_wild_27835869	1399934060	*Ca_kabuli_Chr02*	27835869	G/A	NA	INTERGENIC	NA
Kabuli_wild_27835871	1399934061	*Ca_kabuli_Chr02*	27835871	G/A	NA	INTERGENIC	NA
Kabuli_wild_28017165	1399934062	*Ca_kabuli_Chr02*	28017165	C/T	Gene_Ca_15693	DOWNSTREAM	BURP
Kabuli_wild_28018158	1399934063	*Ca_kabuli_Chr02*	28018158	T/C	NA	INTERGENIC	NA
Kabuli_wild_28061049	1399934064	*Ca_kabuli_Chr02*	28061049	G/T	Gene_Ca_14397	STOP_GAINED	Uncharacterised protein family UPF0497,trans-membrane plant
Kabuli_wild_28084953	1399934065	*Ca_kabuli_Chr02*	28084953	C/T	NA	INTERGENIC	NA
Kabuli_wild_28097357	1399934066	*Ca_kabuli_Chr02*	28097357	C/T	Gene_Ca_14393	INTRON	SET domain
Kabuli_wild_28097394	1399934067	*Ca_kabuli_Chr02*	28097394	C/T	Gene_Ca_14393	INTRON	SET domain
Kabuli_wild_28261718	1399934068	*Ca_kabuli_Chr02*	28261718	G/T	NA	INTERGENIC	NA
Kabuli_wild_28261722	1399934069	*Ca_kabuli_Chr02*	28261722	C/A	NA	INTERGENIC	NA
Kabuli_wild_28261723	1399934070	*Ca_kabuli_Chr02*	28261723	A/T	NA	INTERGENIC	NA
Kabuli_wild_28407316	1399934071	*Ca_kabuli_Chr02*	28407316	T/C	Gene_Ca_14370	DOWNSTREAM	Transposase,Ptta/En/Spm,plant
Kabuli_wild_28407324	1399934072	*Ca_kabuli_Chr02*	28407324	C/T	Gene_Ca_14370	DOWNSTREAM	Transposase,Ptta/En/Spm,plant
Kabuli_wild_28407356	1399934073	*Ca_kabuli_Chr02*	28407356	C/T	Gene_Ca_14370	DOWNSTREAM	Transposase,Ptta/En/Spm,plant
Kabuli_wild_28407362	1399934074	*Ca_kabuli_Chr02*	28407362	A/C	Gene_Ca_14370	DOWNSTREAM	Transposase,Ptta/En/Spm,plant
Kabuli_wild_28534235	1399934075	*Ca_kabuli_Chr02*	28534235	T/C	NA	INTERGENIC	NA
Kabuli_wild_28707104	1399934076	*Ca_kabuli_Chr02*	28707104	T/A	Gene_Ca_14360	NON_SYNONYMOUS_CODING	CCAAT-binding factor
Kabuli_wild_28727817	1399934077	*Ca_kabuli_Chr02*	28727817	C/T	Gene_Ca_14358	SYNONYMOUS_CODING	NA
Kabuli_wild_28741764	1399934078	*Ca_kabuli_Chr02*	28741764	T/C	NA	INTERGENIC	NA
Kabuli_wild_28813403	1399934079	*Ca_kabuli_Chr02*	28813403	G/A	NA	INTERGENIC	NA
Kabuli_wild_28813493	1399934080	*Ca_kabuli_Chr02*	28813493	A/G	NA	INTERGENIC	NA
Kabuli_wild_28813495	1399934081	*Ca_kabuli_Chr02*	28813495	G/A	NA	INTERGENIC	NA
Kabuli_wild_28887773	1399934082	*Ca_kabuli_Chr02*	28887773	T/C	NA	INTERGENIC	NA
Kabuli_wild_28944707	1399934083	*Ca_kabuli_Chr02*	28944707	A/C	NA	INTERGENIC	NA
Kabuli_wild_28944712	1399934084	*Ca_kabuli_Chr02*	28944712	T/C	NA	INTERGENIC	NA
Kabuli_wild_28949911	1399934085	*Ca_kabuli_Chr02*	28949911	A/C	Gene_Ca_14342	NON_SYNONYMOUS_CODING	Glycosidehydrolase,family17
Kabuli_wild_28949919	1399934086	*Ca_kabuli_Chr02*	28949919	A/T	Gene_Ca_14342	NON_SYNONYMOUS_CODING	Glycosidehydrolase,family17
Kabuli_wild_29044466	1399934087	*Ca_kabuli_Chr02*	29044466	C/T	Gene_Ca_14334	INTRON	RNA polymerase I associated factor, A49-like
Kabuli_wild_29122589	1399934088	*Ca_kabuli_Chr02*	29122589	T/G	Gene_Ca_14327	NON_SYNONYMOUS_CODING	PeptidaseA1
Kabuli_wild_29208805	1399934089	*Ca_kabuli_Chr02*	29208805	T/C	NA	INTERGENIC	NA
Kabuli_wild_29778330	1399934090	*Ca_kabuli_Chr02*	29778330	C/T	Gene_Ca_16110	NON_SYNONYMOUS_CODING	Peptidyl-prolylcis-transisomerase,FKBP-type,domain
Kabuli_wild_29778340	1399934091	*Ca_kabuli_Chr02*	29778340	C/T	Gene_Ca_16110	SYNONYMOUS_CODING	Peptidyl-prolylcis-transisomerase,FKBP-type,domain

SNP IDs	NCBI dbSNP accession IDs (NCBI_SS#)	Chromosomes	Physical positions (bp)	SNPs	Gene accession IDs	Sequence components of genome	Putative functions
Kabuli_wild_29778357	1399934092	*Ca_kabuli_Chr02*	29778357	T/C	Gene_Ca_16110	NON_SYNONYMOUS_CODING	Peptidyl-prolylcis-transisomerase,FKBP-type,domain
Kabuli_wild_30133060	1399934093	*Ca_kabuli_Chr02*	30133060	G/C	Gene_Ca_16079	NON_SYNONYMOUS_CODING	PeptidaseS8/S53,subtilisin/kexin/sedolisin
Kabuli_wild_30133068	1399934094	*Ca_kabuli_Chr02*	30133068	A/G	Gene_Ca_16079	SYNONYMOUS_CODING	PeptidaseS8/S53,subtilisin/kexin/sedolisin
Kabuli_wild_30133074	1399934095	*Ca_kabuli_Chr02*	30133074	T/C	Gene_Ca_16079	SYNONYMOUS_CODING	PeptidaseS8/S53,subtilisin/kexin/sedolisin
Kabuli_wild_30133076	1399934096	*Ca_kabuli_Chr02*	30133076	C/T	Gene_Ca_16079	NON_SYNONYMOUS_CODING	PeptidaseS8/S53,subtilisin/kexin/sedolisin
Kabuli_wild_30133084	1399934097	*Ca_kabuli_Chr02*	30133084	T/A	Gene_Ca_16079	NON_SYNONYMOUS_CODING	PeptidaseS8/S53,subtilisin/kexin/sedolisin
Kabuli_wild_30133085	1399934098	*Ca_kabuli_Chr02*	30133085	C/T	Gene_Ca_16079	NON_SYNONYMOUS_CODING	PeptidaseS8/S53,subtilisin/kexin/sedolisin
Kabuli_wild_30133107	1399934099	*Ca_kabuli_Chr02*	30133107	A/T	Gene_Ca_16079	SYNONYMOUS_CODING	PeptidaseS8/S53,subtilisin/kexin/sedolisin
Kabuli_wild_30133110	1399934100	*Ca_kabuli_Chr02*	30133110	C/T	Gene_Ca_16079	SYNONYMOUS_CODING	PeptidaseS8/S53,subtilisin/kexin/sedolisin
Kabuli_wild_30133113	1399934101	*Ca_kabuli_Chr02*	30133113	T/G	Gene_Ca_16079	SYNONYMOUS_CODING	PeptidaseS8/S53,subtilisin/kexin/sedolisin
Kabuli_wild_30133119	1399934102	*Ca_kabuli_Chr02*	30133119	G/A	Gene_Ca_16079	SYNONYMOUS_CODING	PeptidaseS8/S53,subtilisin/kexin/sedolisin
Kabuli_wild_30133128	1399934103	*Ca_kabuli_Chr02*	30133128	A/T	Gene_Ca_16079	SYNONYMOUS_CODING	PeptidaseS8/S53,subtilisin/kexin/sedolisin
Kabuli_wild_30133134	1399934104	*Ca_kabuli_Chr02*	30133134	A/T	Gene_Ca_16079	SYNONYMOUS_CODING	PeptidaseS8/S53,subtilisin/kexin/sedolisin
Kabuli_wild_30133140	1399934105	*Ca_kabuli_Chr02*	30133140	G/A	Gene_Ca_16079	SYNONYMOUS_CODING	PeptidaseS8/S53,subtilisin/kexin/sedolisin
Kabuli_wild_30230544	1399934106	*Ca_kabuli_Chr02*	30230544	T/A	NA	INTERGENIC	NA
Kabuli_wild_30335160	1399934107	*Ca_kabuli_Chr02*	30335160	T/C	Gene_Ca_12520	UPSTREAM	GDP-fucoseproteinO-fucosyltransferase
Kabuli_wild_30335194	1399934108	*Ca_kabuli_Chr02*	30335194	A/G	Gene_Ca_12520	UPSTREAM	GDP-fucoseproteinO-fucosyltransferase
Kabuli_wild_30335212	1399934109	*Ca_kabuli_Chr02*	30335212	T/A	Gene_Ca_12520	UPSTREAM	GDP-fucoseproteinO-fucosyltransferase
Kabuli_wild_30363957	1399934110	*Ca_kabuli_Chr02*	30363957	C/A	Gene_Ca_12516	DOWNSTREAM	NA
Kabuli_wild_30364025	1399934111	*Ca_kabuli_Chr02*	30364025	T/C	Gene_Ca_12516	DOWNSTREAM	NA
Kabuli_wild_30364042	1399934112	*Ca_kabuli_Chr02*	30364042	A/G	Gene_Ca_12516	DOWNSTREAM	NA
Kabuli_wild_30364073	1399934113	*Ca_kabuli_Chr02*	30364073	T/A	Gene_Ca_12516	DOWNSTREAM	NA
Kabuli_wild_30370148	1399934114	*Ca_kabuli_Chr02*	30370148	G/T	NA	INTERGENIC	NA
Kabuli_wild_30370411	1399934115	*Ca_kabuli_Chr02*	30370411	T/C	NA	INTERGENIC	NA
Kabuli_wild_30387702	1399934116	*Ca_kabuli_Chr02*	30387702	A/G	NA	INTERGENIC	NA
Kabuli_wild_30389175	1399934117	*Ca_kabuli_Chr02*	30389175	A/C	Gene_Ca_12512	SYNONYMOUS_CODING	Leucine-rich repeat,cysteine-containingsubtype
Kabuli_wild_30389433	1399934118	*Ca_kabuli_Chr02*	30389433	C/T	Gene_Ca_12512	SYNONYMOUS_CODING	Leucine-rich repeat,cysteine-containingsubtype
Kabuli_wild_30467551	1399934119	*Ca_kabuli_Chr02*	30467551	A/T	Gene_Ca_12503	INTRON	Mov34/MPN/PAD-1
Kabuli_wild_30508454	1399934120	*Ca_kabuli_Chr02*	30508454	T/C	Gene_Ca_12501	INTRON	EXTL2,alpha-1,4-N-acetylhexosaminyltransferase
Kabuli_wild_30521544	1399934121	*Ca_kabuli_Chr02*	30521544	G/A	NA	INTERGENIC	NA
Kabuli_wild_30530607	1399934122	*Ca_kabuli_Chr02*	30530607	T/G	Gene_Ca_12498	NON_SYNONYMOUS_CODING	RibosomalproteinL10/acidicP0
Kabuli_wild_30530642	1399934123	*Ca_kabuli_Chr02*	30530642	A/T	Gene_Ca_12498	SYNONYMOUS_CODING	RibosomalproteinL10/acidicP0
Kabuli_wild_30537667	1399934124	*Ca_kabuli_Chr02*	30537667	A/T	NA	INTERGENIC	NA
Kabuli_wild_30537673	1399934125	*Ca_kabuli_Chr02*	30537673	A/G	NA	INTERGENIC	NA
Kabuli_wild_30537687	1399934126	*Ca_kabuli_Chr02*	30537687	T/C	NA	INTERGENIC	NA
Kabuli_wild_30537701	1399934127	*Ca_kabuli_Chr02*	30537701	T/A	NA	INTERGENIC	NA
Kabuli_wild_30537953	1399934128	*Ca_kabuli_Chr02*	30537953	T/C	NA	INTERGENIC	NA
Kabuli_wild_30686920	1399934129	*Ca_kabuli_Chr02*	30686920	C/T	Gene_Ca_12488	INTRON	HAD-superfamilyhydrolase,subfamilyIG,5'-nucleotidase
Kabuli_wild_30867547	1399934130	*Ca_kabuli_Chr02*	30867547	G/A	NA	INTERGENIC	NA
Kabuli_wild_30867931	1399934131	*Ca_kabuli_Chr02*	30867931	A/G	NA	INTERGENIC	NA
Kabuli_wild_30880148	1399934132	*Ca_kabuli_Chr02*	30880148	A/G	Gene_Ca_12473	INTRON	Synaptobrevin
Kabuli_wild_30880243	1399934133	*Ca_kabuli_Chr02*	30880243	G/C	Gene_Ca_12473	INTRON	Synaptobrevin
Kabuli_wild_30880246	1399934134	*Ca_kabuli_Chr02*	30880246	G/A	Gene_Ca_12473	INTRON	Synaptobrevin
Kabuli_wild_30880282	1399934135	*Ca_kabuli_Chr02*	30880282	A/G	Gene_Ca_12473	INTRON	Synaptobrevin

SNP IDs	NCBI dbSNP accession IDs (NCBI_SS#)	Chromosomes	Physical positions (bp)	SNPs	Gene accession IDs	Sequence components of genome	Putative functions
Kabuli_wild_30880302	1399934136	*Ca_kabuli_Chr02*	30880302	T/C	Gene_Ca_12473	INTRON	Synaptobrevin
Kabuli_wild_30880303	1399934137	*Ca_kabuli_Chr02*	30880303	T/A	Gene_Ca_12473	INTRON	Synaptobrevin
Kabuli_wild_30920461	1399934138	*Ca_kabuli_Chr02*	30920461	C/T	Gene_Ca_12470	INTRON	NA
Kabuli_wild_30921092	1399934139	*Ca_kabuli_Chr02*	30921092	C/A	Gene_Ca_12470	SYNONYMOUS_CODING	NA
Kabuli_wild_30921562	1399934140	*Ca_kabuli_Chr02*	30921562	C/T	Gene_Ca_12470	INTRON	NA
Kabuli_wild_30921579	1399934141	*Ca_kabuli_Chr02*	30921579	T/C	Gene_Ca_12470	INTRON	NA
Kabuli_wild_30921583	1399934142	*Ca_kabuli_Chr02*	30921583	T/C	Gene_Ca_12470	INTRON	NA
Kabuli_wild_30921602	1399934143	*Ca_kabuli_Chr02*	30921602	C/T	Gene_Ca_12470	INTRON	NA
Kabuli_wild_30921629	1399934144	*Ca_kabuli_Chr02*	30921629	A/C	Gene_Ca_12470	INTRON	NA
Kabuli_wild_30921645	1399934145	*Ca_kabuli_Chr02*	30921645	A/C	Gene_Ca_12470	INTRON	NA
Kabuli_wild_30921758	1399934146	*Ca_kabuli_Chr02*	30921758	C/T	Gene_Ca_12470	INTRON	NA
Kabuli_wild_30921917	1399934147	*Ca_kabuli_Chr02*	30921917	C/A	Gene_Ca_12470	INTRON	NA
Kabuli_wild_30921923	1399934148	*Ca_kabuli_Chr02*	30921923	A/G	Gene_Ca_12470	INTRON	NA
Kabuli_wild_30925755	1399934149	*Ca_kabuli_Chr02*	30925755	A/T	Gene_Ca_12470	INTRON	NA
Kabuli_wild_30925784	1399934150	*Ca_kabuli_Chr02*	30925784	C/T	Gene_Ca_12470	INTRON	NA
Kabuli_wild_30951724	1399934151	*Ca_kabuli_Chr02*	30951724	T/A	Gene_Ca_12469	SYNONYMOUS_CODING	Clathrin,heavychain/VPS,7-foldrepeat
Kabuli_wild_30954305	1399934152	*Ca_kabuli_Chr02*	30954305	T/G	Gene_Ca_12469	INTRON	Clathrin,heavychain/VPS,7-foldrepeat
Kabuli_wild_30954317	1399934153	*Ca_kabuli_Chr02*	30954317	G/A	Gene_Ca_12469	INTRON	Clathrin,heavychain/VPS,7-foldrepeat
Kabuli_wild_30977508	1399934154	*Ca_kabuli_Chr02*	30977508	A/T	NA	INTERGENIC	NA
Kabuli_wild_30977522	1399934155	*Ca_kabuli_Chr02*	30977522	A/T	NA	INTERGENIC	NA
Kabuli_wild_30977528	1399934156	*Ca_kabuli_Chr02*	30977528	C/T	NA	INTERGENIC	NA
Kabuli_wild_30977572	1399934157	*Ca_kabuli_Chr02*	30977572	A/G	NA	INTERGENIC	NA
Kabuli_wild_30997769	1399934158	*Ca_kabuli_Chr02*	30997769	T/A	Gene_Ca_12465	SYNONYMOUS_CODING	Pathogenesis-related transcriptional factor/ERF, DNA-binding
Kabuli_wild_30997780	1399934159	*Ca_kabuli_Chr02*	30997780	A/C	Gene_Ca_12465	SYNONYMOUS_CODING	Pathogenesis-related transcriptional factor/ERF, DNA-binding
Kabuli_wild_30997784	1399934160	*Ca_kabuli_Chr02*	30997784	T/A	Gene_Ca_12465	SYNONYMOUS_CODING	Pathogenesis-related transcriptional factor/ERF, DNA-binding
Kabuli_wild_31005670	1399934161	*Ca_kabuli_Chr02*	31005670	G/T	Gene_Ca_12464	SYNONYMOUS_CODING	Lateral organ boundaries, LOB
Kabuli_wild_31005845	1399934162	*Ca_kabuli_Chr02*	31005845	C/T	Gene_Ca_12464	SYNONYMOUS_CODING	Lateral organ boundaries, LOB
Kabuli_wild_31005847	1399934163	*Ca_kabuli_Chr02*	31005847	T/G	Gene_Ca_12464	SYNONYMOUS_CODING	Lateral organ boundaries, LOB
Kabuli_wild_31014263	1399934164	*Ca_kabuli_Chr02*	31014263	C/T	NA	INTERGENIC	NA
Kabuli_wild_31015879	1399934165	*Ca_kabuli_Chr02*	31015879	G/A	NA	INTERGENIC	NA
Kabuli_wild_31015888	1399934166	*Ca_kabuli_Chr02*	31015888	A/G	NA	INTERGENIC	NA
Kabuli_wild_31055007	1399934167	*Ca_kabuli_Chr02*	31055007	G/A	Gene_Ca_12459	INTRON	Rad21/Rec8-likeprotein,C-terminal,eukaryotic
Kabuli_wild_31055148	1399934168	*Ca_kabuli_Chr02*	31055148	G/A	Gene_Ca_12459	INTRON	Rad21/Rec8-likeprotein,C-terminal,eukaryotic
Kabuli_wild_31055163	1399934169	*Ca_kabuli_Chr02*	31055163	A/T	Gene_Ca_12459	INTRON	Rad21/Rec8-likeprotein,C-terminal,eukaryotic
Kabuli_wild_31055164	1399934170	*Ca_kabuli_Chr02*	31055164	C/T	Gene_Ca_12459	INTRON	Rad21/Rec8-likeprotein,C-terminal,eukaryotic
Kabuli_wild_31055173	1399934171	*Ca_kabuli_Chr02*	31055173	T/C	Gene_Ca_12459	INTRON	Rad21/Rec8-likeprotein,C-terminal,eukaryotic
Kabuli_wild_31088645	1399934172	*Ca_kabuli_Chr02*	31088645	A/C	NA	INTERGENIC	NA
Kabuli_wild_31088648	1399934173	*Ca_kabuli_Chr02*	31088648	A/G	NA	INTERGENIC	NA
Kabuli_wild_31088670	1399934174	*Ca_kabuli_Chr02*	31088670	C/T	NA	INTERGENIC	NA
Kabuli_wild_31088676	1399934175	*Ca_kabuli_Chr02*	31088676	G/C	NA	INTERGENIC	NA
Kabuli_wild_31088681	1399934176	*Ca_kabuli_Chr02*	31088681	G/T	NA	INTERGENIC	NA
Kabuli_wild_31088778	1399934177	*Ca_kabuli_Chr02*	31088778	A/T	NA	INTERGENIC	NA
Kabuli_wild_31088800	1399934178	*Ca_kabuli_Chr02*	31088800	T/C	NA	INTERGENIC	NA
Kabuli_wild_31088882	1399934179	*Ca_kabuli_Chr02*	31088882	A/G	NA	INTERGENIC	NA

SNP IDs	NCBI dbSNP accession IDs (NCBI_SS#)	Chromosomes	Physical positions (bp)	SNPs	Gene accession IDs	Sequence components of genome	Putative functions
Kabuli_wild_31103457	1399934180	*Ca_kabuli_Chr02*	31103457	C/A	Gene_Ca_12452	INTRON	Glutamineamidotransferasetype1
Kabuli_wild_31109311	1399934181	*Ca_kabuli_Chr02*	31109311	G/T	NA	INTERGENIC	NA
Kabuli_wild_31109358	1399934182	*Ca_kabuli_Chr02*	31109358	C/T	NA	INTERGENIC	NA
Kabuli_wild_31109427	1399934183	*Ca_kabuli_Chr02*	31109427	C/T	NA	INTERGENIC	NA
Kabuli_wild_31109435	1399934184	*Ca_kabuli_Chr02*	31109435	G/A	NA	INTERGENIC	NA
Kabuli_wild_31109441	1399934185	*Ca_kabuli_Chr02*	31109441	C/G	NA	INTERGENIC	NA
Kabuli_wild_31109481	1399934186	*Ca_kabuli_Chr02*	31109481	G/A	NA	INTERGENIC	NA
Kabuli_wild_31113429	1399934187	*Ca_kabuli_Chr02*	31113429	C/A	Gene_Ca_12451	INTRON	Glutamineamidotransferasetype1
Kabuli_wild_31113485	1399934188	*Ca_kabuli_Chr02*	31113485	G/T	Gene_Ca_12451	INTRON	Glutamineamidotransferasetype1
Kabuli_wild_31113601	1399934189	*Ca_kabuli_Chr02*	31113601	A/C	Gene_Ca_12451	INTRON	Glutamineamidotransferasetype1
Kabuli_wild_31113621	1399934190	*Ca_kabuli_Chr02*	31113621	G/A	Gene_Ca_12451	INTRON	Glutamineamidotransferasetype1
Kabuli_wild_31113636	1399934191	*Ca_kabuli_Chr02*	31113636	G/A	Gene_Ca_12451	INTRON	Glutamineamidotransferasetype1
Kabuli_wild_31141019	1399934192	*Ca_kabuli_Chr02*	31141019	G/A	NA	INTERGENIC	NA
Kabuli_wild_31188355	1399934193	*Ca_kabuli_Chr02*	31188355	A/G	Gene_Ca_12445	SYNONYMOUS_CODING	Multicopperoxidase,type1
Kabuli_wild_31188546	1399934194	*Ca_kabuli_Chr02*	31188546	T/C	Gene_Ca_12445	INTRON	Multicopperoxidase,type1
Kabuli_wild_31188553	1399934195	*Ca_kabuli_Chr02*	31188553	A/T	Gene_Ca_12445	INTRON	Multicopperoxidase,type1
Kabuli_wild_31201859	1399934196	*Ca_kabuli_Chr02*	31201859	T/A	Gene_Ca_12444	INTRON	Phoxhomologousdomain
Kabuli_wild_31201872	1399934197	*Ca_kabuli_Chr02*	31201872	T/C	Gene_Ca_12444	INTRON	Phoxhomologousdomain
Kabuli_wild_31203154	1399934198	*Ca_kabuli_Chr02*	31203154	G/A	Gene_Ca_12444	SYNONYMOUS_CODING	Phoxhomologousdomain
Kabuli_wild_31306639	1399934199	*Ca_kabuli_Chr02*	31306639	T/C	NA	INTERGENIC	NA
Kabuli_wild_31427861	1399934200	*Ca_kabuli_Chr02*	31427861	A/G	Gene_Ca_12419	NON_SYNONYMOUS_CODING	Protein of unknown function DUF827,plant
Kabuli_wild_31427902	1399934201	*Ca_kabuli_Chr02*	31427902	G/C	Gene_Ca_12419	NON_SYNONYMOUS_CODING	Protein of unknown function DUF827,plant
Kabuli_wild_31428024	1399934202	*Ca_kabuli_Chr02*	31428024	T/G	Gene_Ca_12419	NON_SYNONYMOUS_CODING	Protein of unknown function DUF827,plant
Kabuli_wild_31428132	1399934203	*Ca_kabuli_Chr02*	31428132	G/A	Gene_Ca_12419	SYNONYMOUS_CODING	Protein of unknown function DUF827,plant
Kabuli_wild_31428279	1399934204	*Ca_kabuli_Chr02*	31428279	T/C	Gene_Ca_12419	SYNONYMOUS_CODING	Protein of unknown function DUF827,plant
Kabuli_wild_31428405	1399934205	*Ca_kabuli_Chr02*	31428405	T/C	Gene_Ca_12419	SYNONYMOUS_CODING	Protein of unknown function DUF827,plant
Kabuli_wild_31428429	1399934206	*Ca_kabuli_Chr02*	31428429	T/G	Gene_Ca_12419	NON_SYNONYMOUS_CODING	Protein of unknown function DUF827,plant
Kabuli_wild_31534265	1399934207	*Ca_kabuli_Chr02*	31534265	A/C	NA	INTERGENIC	NA
Kabuli_wild_31534283	1399934208	*Ca_kabuli_Chr02*	31534283	G/A	NA	INTERGENIC	NA
Kabuli_wild_31534455	1399934209	*Ca_kabuli_Chr02*	31534455	T/C	NA	INTERGENIC	NA
Kabuli_wild_31534463	1399934210	*Ca_kabuli_Chr02*	31534463	T/C	NA	INTERGENIC	NA
Kabuli_wild_31584313	1399934211	*Ca_kabuli_Chr02*	31584313	A/C	Gene_Ca_17856	INTRON	Indole-3-glycerolphosphatesynthase,conservedsite
Kabuli_wild_31686714	1399934212	*Ca_kabuli_Chr02*	31686714	C/T	NA	INTERGENIC	NA
Kabuli_wild_31715974	1399934213	*Ca_kabuli_Chr02*	31715974	T/A	NA	INTERGENIC	NA
Kabuli_wild_31756777	1399934214	*Ca_kabuli_Chr02*	31756777	G/T	NA	INTERGENIC	NA
Kabuli_wild_31847033	1399934215	*Ca_kabuli_Chr02*	31847033	A/C	NA	INTERGENIC	NA
Kabuli_wild_31881918	1399934216	*Ca_kabuli_Chr02*	31881918	G/T	Gene_Ca_17830	NON_SYNONYMOUS_CODING	RZZcomplex,subunitZw10
Kabuli_wild_31885934	1399934217	*Ca_kabuli_Chr02*	31885934	A/G	NA	INTERGENIC	NA
Kabuli_wild_31908694	1399934218	*Ca_kabuli_Chr02*	31908694	G/A	Gene_Ca_17826	SYNONYMOUS_CODING	Zinc finger, RING-type
Kabuli_wild_31908742	1399934219	*Ca_kabuli_Chr02*	31908742	G/A	Gene_Ca_17826	SYNONYMOUS_CODING	Zinc finger, RING-type
Kabuli_wild_31908787	1399934220	*Ca_kabuli_Chr02*	31908787	G/A	Gene_Ca_17826	INTRON	Zinc finger, RING-type
Kabuli_wild_31908803	1399934221	*Ca_kabuli_Chr02*	31908803	C/T	Gene_Ca_17826	INTRON	Zinc finger, RING-type
Kabuli_wild_31908845	1399934222	*Ca_kabuli_Chr02*	31908845	T/A	Gene_Ca_17826	INTRON	Zinc finger, RING-type
Kabuli_wild_31908857	1399934223	*Ca_kabuli_Chr02*	31908857	C/T	Gene_Ca_17826	INTRON	Zinc finger, RING-type

SNP IDs	NCBI dbSNP accession IDs (NCBI_SS#)	Chromosomes	Physical positions (bp)	SNPs	Gene accession IDs	Sequence components of genome	Putative functions
Kabuli_wild_31941463	1399934224	*Ca_kabuli_Chr02*	31941463	T/C	Gene_Ca_17823	SYNONYMOUS_CODING	Zinc finger,CCCH-type
Kabuli_wild_31941554	1399934225	*Ca_kabuli_Chr02*	31941554	A/T	Gene_Ca_17823	INTRON	Zinc finger,CCCH-type
Kabuli_wild_32003536	1399934226	*Ca_kabuli_Chr02*	32003536	G/T	NA	INTERGENIC	NA
Kabuli_wild_32017361	1399934227	*Ca_kabuli_Chr02*	32017361	G/A	NA	INTERGENIC	NA
Kabuli_wild_32049523	1399934228	*Ca_kabuli_Chr02*	32049523	C/A	Gene_Ca_17817	NON_SYNONYMOUS_CODING	NA
Kabuli_wild_32049525	1399934229	*Ca_kabuli_Chr02*	32049525	A/C	Gene_Ca_17817	SYNONYMOUS_CODING	NA
Kabuli_wild_32058591	1399934230	*Ca_kabuli_Chr02*	32058591	T/C	Gene_Ca_17816	SYNONYMOUS_CODING	Ankyrinrepeat
Kabuli_wild_32061053	1399934231	*Ca_kabuli_Chr02*	32061053	C/T	Gene_Ca_17816	INTRON	Ankyrinrepeat
Kabuli_wild_32061054	1399934232	*Ca_kabuli_Chr02*	32061054	T/C	Gene_Ca_17816	INTRON	Ankyrinrepeat
Kabuli_wild_32061083	1399934233	*Ca_kabuli_Chr02*	32061083	A/G	Gene_Ca_17816	INTRON	Ankyrinrepeat
Kabuli_wild_32062010	1399934234	*Ca_kabuli_Chr02*	32062010	A/C	Gene_Ca_17816	SYNONYMOUS_CODING	Ankyrinrepeat
Kabuli_wild_32071084	1399934235	*Ca_kabuli_Chr02*	32071084	G/T	NA	INTERGENIC	NA
Kabuli_wild_32071107	1399934236	*Ca_kabuli_Chr02*	32071107	C/G	NA	INTERGENIC	NA
Kabuli_wild_32080002	1399934237	*Ca_kabuli_Chr02*	32080002	T/C	NA	INTERGENIC	NA
Kabuli_wild_32080007	1399934238	*Ca_kabuli_Chr02*	32080007	C/T	NA	INTERGENIC	NA
Kabuli_wild_32080010	1399934239	*Ca_kabuli_Chr02*	32080010	C/G	NA	INTERGENIC	NA
Kabuli_wild_32080016	1399934240	*Ca_kabuli_Chr02*	32080016	A/T	NA	INTERGENIC	NA
Kabuli_wild_32080022	1399934241	*Ca_kabuli_Chr02*	32080022	C/A	NA	INTERGENIC	NA
Kabuli_wild_32080037	1399934242	*Ca_kabuli_Chr02*	32080037	A/G	NA	INTERGENIC	NA
Kabuli_wild_32096172	1399934243	*Ca_kabuli_Chr02*	32096172	C/T	NA	INTERGENIC	NA
Kabuli_wild_32096177	1399934244	*Ca_kabuli_Chr02*	32096177	C/T	NA	INTERGENIC	NA
Kabuli_wild_32096180	1399934245	*Ca_kabuli_Chr02*	32096180	C/G	NA	INTERGENIC	NA
Kabuli_wild_32096186	1399934246	*Ca_kabuli_Chr02*	32096186	T/A	NA	INTERGENIC	NA
Kabuli_wild_32096192	1399934247	*Ca_kabuli_Chr02*	32096192	A/C	NA	INTERGENIC	NA
Kabuli_wild_32099907	1399934248	*Ca_kabuli_Chr02*	32099907	T/C	NA	INTERGENIC	NA
Kabuli_wild_32109805	1399934249	*Ca_kabuli_Chr02*	32109805	G/A	NA	INTERGENIC	NA
Kabuli_wild_32109811	1399934250	*Ca_kabuli_Chr02*	32109811	G/A	NA	INTERGENIC	NA
Kabuli_wild_32109813	1399934251	*Ca_kabuli_Chr02*	32109813	C/T	NA	INTERGENIC	NA
Kabuli_wild_32109821	1399934252	*Ca_kabuli_Chr02*	32109821	G/A	NA	INTERGENIC	NA
Kabuli_wild_32109827	1399934253	*Ca_kabuli_Chr02*	32109827	T/C	NA	INTERGENIC	NA
Kabuli_wild_32109859	1399934254	*Ca_kabuli_Chr02*	32109859	A/C	NA	INTERGENIC	NA
Kabuli_wild_32109892	1399934255	*Ca_kabuli_Chr02*	32109892	T/A	NA	INTERGENIC	NA
Kabuli_wild_32109895	1399934256	*Ca_kabuli_Chr02*	32109895	A/G	NA	INTERGENIC	NA
Kabuli_wild_32109904	1399934257	*Ca_kabuli_Chr02*	32109904	T/A	NA	INTERGENIC	NA
Kabuli_wild_32119094	1399934258	*Ca_kabuli_Chr02*	32119094	A/T	NA	INTERGENIC	NA
Kabuli_wild_32119268	1399934259	*Ca_kabuli_Chr02*	32119268	T/A	NA	INTERGENIC	NA
Kabuli_wild_32119274	1399934260	*Ca_kabuli_Chr02*	32119274	T/C	NA	INTERGENIC	NA
Kabuli_wild_32119328	1399934261	*Ca_kabuli_Chr02*	32119328	T/G	NA	INTERGENIC	NA
Kabuli_wild_32119331	1399934262	*Ca_kabuli_Chr02*	32119331	A/T	NA	INTERGENIC	NA
Kabuli_wild_32119358	1399934263	*Ca_kabuli_Chr02*	32119358	A/T	NA	INTERGENIC	NA
Kabuli_wild_32129272	1399934264	*Ca_kabuli_Chr02*	32129272	T/C	Gene_Ca_17812	DOWNSTREAM	Ubiquitin
Kabuli_wild_32129395	1399934265	*Ca_kabuli_Chr02*	32129395	C/G	Gene_Ca_17812	DOWNSTREAM	Ubiquitin
Kabuli_wild_32140939	1399934266	*Ca_kabuli_Chr02*	32140939	A/T	NA	INTERGENIC	NA
Kabuli_wild_32140948	1399934267	*Ca_kabuli_Chr02*	32140948	A/C	NA	INTERGENIC	NA

SNP IDs	NCBI dbSNP accession IDs (NCBI_SS#)	Chromosomes	Physical positions (bp)	SNPs	Gene accession IDs	Sequence components of genome	Putative functions
Kabuli_wild_32140987	1399934268	*Ca_kabuli_Chr02*	32140987	A/G	NA	INTERGENIC	NA
Kabuli_wild_32140990	1399934269	*Ca_kabuli_Chr02*	32140990	G/A	NA	INTERGENIC	NA
Kabuli_wild_32140998	1399934270	*Ca_kabuli_Chr02*	32140998	G/T	NA	INTERGENIC	NA
Kabuli_wild_32141002	1399934271	*Ca_kabuli_Chr02*	32141002	A/T	NA	INTERGENIC	NA
Kabuli_wild_32141013	1399934272	*Ca_kabuli_Chr02*	32141013	G/C	NA	INTERGENIC	NA
Kabuli_wild_32141019	1399934273	*Ca_kabuli_Chr02*	32141019	G/A	NA	INTERGENIC	NA
Kabuli_wild_32141038	1399934274	*Ca_kabuli_Chr02*	32141038	T/C	NA	INTERGENIC	NA
Kabuli_wild_32141047	1399934275	*Ca_kabuli_Chr02*	32141047	A/G	NA	INTERGENIC	NA
Kabuli_wild_32183297	1399934276	*Ca_kabuli_Chr02*	32183297	A/T	NA	INTERGENIC	NA
Kabuli_wild_32183325	1399934277	*Ca_kabuli_Chr02*	32183325	T/A	NA	INTERGENIC	NA
Kabuli_wild_32183328	1399934278	*Ca_kabuli_Chr02*	32183328	T/C	NA	INTERGENIC	NA
Kabuli_wild_32183364	1399934279	*Ca_kabuli_Chr02*	32183364	T/C	NA	INTERGENIC	NA
Kabuli_wild_32183408	1399934280	*Ca_kabuli_Chr02*	32183408	A/C	NA	INTERGENIC	NA
Kabuli_wild_32202980	1399934281	*Ca_kabuli_Chr02*	32202980	G/T	Gene_Ca_10295	SYNONYMOUS_CODING	PeptidaseS8/S53,subtilisin/kexin/sedolisin
Kabuli_wild_32203030	1399934282	*Ca_kabuli_Chr02*	32203030	C/T	Gene_Ca_10295	NON_SYNONYMOUS_CODING	PeptidaseS8/S53,subtilisin/kexin/sedolisin
Kabuli_wild_32203032	1399934283	*Ca_kabuli_Chr02*	32203032	A/G	Gene_Ca_10295	SYNONYMOUS_CODING	PeptidaseS8/S53,subtilisin/kexin/sedolisin
Kabuli_wild_32203036	1399934284	*Ca_kabuli_Chr02*	32203036	T/A	Gene_Ca_10295	SYNONYMOUS_CODING	PeptidaseS8/S53,subtilisin/kexin/sedolisin
Kabuli_wild_32203053	1399934285	*Ca_kabuli_Chr02*	32203053	T/A	Gene_Ca_10295	SYNONYMOUS_CODING	PeptidaseS8/S53,subtilisin/kexin/sedolisin
Kabuli_wild_32203056	1399934286	*Ca_kabuli_Chr02*	32203056	A/G	Gene_Ca_10295	SYNONYMOUS_CODING	PeptidaseS8/S53,subtilisin/kexin/sedolisin
Kabuli_wild_32242759	1399934287	*Ca_kabuli_Chr02*	32242759	T/G	NA	INTERGENIC	NA
Kabuli_wild_32242818	1399934288	*Ca_kabuli_Chr02*	32242818	T/G	NA	INTERGENIC	NA
Kabuli_wild_32276589	1399934289	*Ca_kabuli_Chr02*	32276589	C/T	Gene_Ca_10288	INTRON	UBX
Kabuli_wild_32348054	1399934290	*Ca_kabuli_Chr02*	32348054	T/A	Gene_Ca_10283	INTRON	NA
Kabuli_wild_32348087	1399934291	*Ca_kabuli_Chr02*	32348087	A/C	Gene_Ca_10283	INTRON	NA
Kabuli_wild_32348119	1399934292	*Ca_kabuli_Chr02*	32348119	G/T	Gene_Ca_10283	INTRON	NA
Kabuli_wild_32348689	1399934293	*Ca_kabuli_Chr02*	32348689	T/C	Gene_Ca_10283	SYNONYMOUS_CODING	NA
Kabuli_wild_32348793	1399934294	*Ca_kabuli_Chr02*	32348793	G/A	Gene_Ca_10283	INTRON	NA
Kabuli_wild_32348809	1399934295	*Ca_kabuli_Chr02*	32348809	A/C	Gene_Ca_10283	INTRON	NA
Kabuli_wild_32348870	1399934296	*Ca_kabuli_Chr02*	32348870	T/C	Gene_Ca_10283	INTRON	NA
Kabuli_wild_32351821	1399934297	*Ca_kabuli_Chr02*	32351821	T/C	Gene_Ca_10283	SYNONYMOUS_CODING	NA
Kabuli_wild_32351839	1399934298	*Ca_kabuli_Chr02*	32351839	G/A	Gene_Ca_10283	SYNONYMOUS_CODING	NA
Kabuli_wild_32351977	1399934299	*Ca_kabuli_Chr02*	32351977	T/C	Gene_Ca_10283	INTRON	NA
Kabuli_wild_32351986	1399934300	*Ca_kabuli_Chr02*	32351986	C/A	Gene_Ca_10283	INTRON	NA
Kabuli_wild_32351989	1399934301	*Ca_kabuli_Chr02*	32351989	T/A	Gene_Ca_10283	INTRON	NA
Kabuli_wild_32352022	1399934302	*Ca_kabuli_Chr02*	32352022	T/C	Gene_Ca_10283	INTRON	NA
Kabuli_wild_32352044	1399934303	*Ca_kabuli_Chr02*	32352044	C/T	Gene_Ca_10283	INTRON	NA
Kabuli_wild_32352045	1399934304	*Ca_kabuli_Chr02*	32352045	A/G	Gene_Ca_10283	INTRON	NA
Kabuli_wild_32352046	1399934305	*Ca_kabuli_Chr02*	32352046	T/C	Gene_Ca_10283	INTRON	NA
Kabuli_wild_32411986	1399934306	*Ca_kabuli_Chr02*	32411986	G/T	NA	INTERGENIC	NA
Kabuli_wild_32411994	1399934307	*Ca_kabuli_Chr02*	32411994	G/T	NA	INTERGENIC	NA
Kabuli_wild_32412000	1399934308	*Ca_kabuli_Chr02*	32412000	G/T	NA	INTERGENIC	NA
Kabuli_wild_32415809	1399934309	*Ca_kabuli_Chr02*	32415809	C/T	Gene_Ca_10278	SYNONYMOUS_CODING	Protein kinase,catalytic domain
Kabuli_wild_32415818	1399934310	*Ca_kabuli_Chr02*	32415818	C/T	Gene_Ca_10278	SYNONYMOUS_CODING	Protein kinase,catalytic domain
Kabuli_wild_32416098	1399934311	*Ca_kabuli_Chr02*	32416098	G/A	Gene_Ca_10278	INTRON	Protein kinase,catalytic domain

SNP IDs	NCBI dbSNP accession IDs (NCBI_SS#)	Chromosomes	Physical positions (bp)	SNPs	Gene accession IDs	Sequence components of genome	Putative functions
Kabuli_wild_32416166	1399934312	*Ca_kabuli_Chr02*	32416166	T/C	Gene_Ca_10278	SYNONYMOUS_CODING	Protein kinase,catalytic domain
Kabuli_wild_32425910	1399934313	*Ca_kabuli_Chr02*	32425910	A/C	NA	INTERGENIC	NA
Kabuli_wild_32481969	1399934314	*Ca_kabuli_Chr02*	32481969	C/T	NA	INTERGENIC	NA
Kabuli_wild_32481983	1399934315	*Ca_kabuli_Chr02*	32481983	G/T	NA	INTERGENIC	NA
Kabuli_wild_32482739	1399934316	*Ca_kabuli_Chr02*	32482739	T/C	Gene_Ca_10272	INTRON	Domain of unknown function DUF221
Kabuli_wild_32488307	1399934317	*Ca_kabuli_Chr02*	32488307	A/C	NA	INTERGENIC	NA
Kabuli_wild_32509087	1399934318	*Ca_kabuli_Chr02*	32509087	A/G	Gene_Ca_10271	INTRON	PeptidaseC19,ubiquitincarboxyl-terminalhydrolase2
Kabuli_wild_32545320	1399934319	*Ca_kabuli_Chr02*	32545320	A/G	Gene_Ca_10268	SYNONYMOUS_CODING	Zinc finger,CCHC-type
Kabuli_wild_32564427	1399934320	*Ca_kabuli_Chr02*	32564427	T/C	Gene_Ca_10266	INTRON	RecF/RecN/SMC
Kabuli_wild_32564480	1399934321	*Ca_kabuli_Chr02*	32564480	G/A	Gene_Ca_10266	INTRON	RecF/RecN/SMC
Kabuli_wild_32564579	1399934322	*Ca_kabuli_Chr02*	32564579	G/A	Gene_Ca_10266	INTRON	RecF/RecN/SMC
Kabuli_wild_32564701	1399934323	*Ca_kabuli_Chr02*	32564701	C/T	Gene_Ca_10266	INTRON	RecF/RecN/SMC
Kabuli_wild_32564770	1399934324	*Ca_kabuli_Chr02*	32564770	G/T	Gene_Ca_10266	INTRON	RecF/RecN/SMC
Kabuli_wild_32564796	1399934325	*Ca_kabuli_Chr02*	32564796	A/T	Gene_Ca_10266	INTRON	RecF/RecN/SMC
Kabuli_wild_32570394	1399934326	*Ca_kabuli_Chr02*	32570394	G/A	Gene_Ca_10266	INTRON	RecF/RecN/SMC
Kabuli_wild_32570429	1399934327	*Ca_kabuli_Chr02*	32570429	G/A	Gene_Ca_10266	INTRON	RecF/RecN/SMC
Kabuli_wild_32570504	1399934328	*Ca_kabuli_Chr02*	32570504	G/C	Gene_Ca_10266	INTRON	RecF/RecN/SMC
Kabuli_wild_32577285	1399934329	*Ca_kabuli_Chr02*	32577285	G/T	Gene_Ca_10266	INTRON	RecF/RecN/SMC
Kabuli_wild_32579849	1399934330	*Ca_kabuli_Chr02*	32579849	T/C	Gene_Ca_10266	SYNONYMOUS_CODING	RecF/RecN/SMC
Kabuli_wild_32579900	1399934331	*Ca_kabuli_Chr02*	32579900	C/G	Gene_Ca_10266	SYNONYMOUS_CODING	RecF/RecN/SMC
Kabuli_wild_32611601	1399934332	*Ca_kabuli_Chr02*	32611601	G/T	Gene_Ca_10261	NON_SYNONYMOUS_CODING	Aldehydeoxidase/xanthinedehydrogenase,a/bhammerhead
Kabuli_wild_32611624	1399934333	*Ca_kabuli_Chr02*	32611624	C/T	Gene_Ca_10261	SYNONYMOUS_CODING	Aldehydeoxidase/xanthinedehydrogenase,a/bhammerhead
Kabuli_wild_32670181	1399934334	*Ca_kabuli_Chr02*	32670181	T/G	Gene_Ca_10254	NON_SYNONYMOUS_CODING	BTB/POZ-like
Kabuli_wild_32790977	1399934335	*Ca_kabuli_Chr02*	32790977	A/T	Gene_Ca_10242	INTRON	Transferrinreceptor-like,dimerisationdomain
Kabuli_wild_32844751	1399934336	*Ca_kabuli_Chr02*	32844751	C/T	Gene_Ca_10238	NON_SYNONYMOUS_CODING	Late embryo genesis abundant protein, group2
Kabuli_wild_32844754	1399934337	*Ca_kabuli_Chr02*	32844754	T/G	Gene_Ca_10238	NON_SYNONYMOUS_CODING	Late embryo genesis abundant protein, group2
Kabuli_wild_32936908	1399934338	*Ca_kabuli_Chr02*	32936908	G/A	Gene_Ca_10230	NON_SYNONYMOUS_CODING	Proteinofunknown functionDM15
Kabuli_wild_32958034	1399934339	*Ca_kabuli_Chr02*	32958034	C/G	NA	INTERGENIC	NA
Kabuli_wild_32958148	1399934340	*Ca_kabuli_Chr02*	32958148	T/G	NA	INTERGENIC	NA
Kabuli_wild_32989205	1399934341	*Ca_kabuli_Chr02*	32989205	G/T	NA	INTERGENIC	NA
Kabuli_wild_33103766	1399934342	*Ca_kabuli_Chr02*	33103766	G/A	NA	INTERGENIC	NA
Kabuli_wild_33184262	1399934343	*Ca_kabuli_Chr02*	33184262	T/C	Gene_Ca_10203	NON_SYNONYMOUS_CODING	NA
Kabuli_wild_33198837	1399934344	*Ca_kabuli_Chr02*	33198837	A/C	Gene_Ca_10203	INTRON	NA
Kabuli_wild_33198838	1399934345	*Ca_kabuli_Chr02*	33198838	G/C	Gene_Ca_10203	INTRON	NA
Kabuli_wild_33326509	1399934346	*Ca_kabuli_Chr02*	33326509	A/C	Gene_Ca_10194	DOWNSTREAM	Synaptobrevin
Kabuli_wild_33326510	1399934347	*Ca_kabuli_Chr02*	33326510	C/G	Gene_Ca_10194	DOWNSTREAM	Synaptobrevin
Kabuli_wild_33398527	1399934348	*Ca_kabuli_Chr02*	33398527	T/G	NA	INTERGENIC	NA
Kabuli_wild_33398549	1399934349	*Ca_kabuli_Chr02*	33398549	T/C	NA	INTERGENIC	NA
Kabuli_wild_33398550	1399934350	*Ca_kabuli_Chr02*	33398550	G/A	NA	INTERGENIC	NA
Kabuli_wild_33398561	1399934351	*Ca_kabuli_Chr02*	33398561	C/T	NA	INTERGENIC	NA
Kabuli_wild_33399045	1399934352	*Ca_kabuli_Chr02*	33399045	A/C	Gene_Ca_10186	SYNONYMOUS_CODING	Disease resistance protein
Kabuli_wild_33399063	1399934353	*Ca_kabuli_Chr02*	33399063	C/T	Gene_Ca_10186	SYNONYMOUS_CODING	Disease resistance protein
Kabuli_wild_33399098	1399934354	*Ca_kabuli_Chr02*	33399098	G/A	Gene_Ca_10186	NON_SYNONYMOUS_CODING	Disease resistance protein
Kabuli_wild_33399342	1399934355	*Ca_kabuli_Chr02*	33399342	A/C	Gene_Ca_10186	NON_SYNONYMOUS_CODING	Disease resistance protein

SNP IDs	NCBI dbSNP accession IDs (NCBI_SS#)	Chromosomes	Physical positions (bp)	SNPs	Gene accession IDs	Sequence components of genome	Putative functions
Kabuli_wild_33399381	1399934356	*Ca_kabuli_Chr02*	33399381	C/T	Gene_Ca_10186	SYNONYMOUS_CODING	Disease resistance protein
Kabuli_wild_33399393	1399934357	*Ca_kabuli_Chr02*	33399393	C/A	Gene_Ca_10186	NON_SYNONYMOUS_CODING	Disease resistance protein
Kabuli_wild_33399397	1399934358	*Ca_kabuli_Chr02*	33399397	C/T	Gene_Ca_10186	NON_SYNONYMOUS_CODING	Disease resistance protein
Kabuli_wild_33399404	1399934359	*Ca_kabuli_Chr02*	33399404	C/A	Gene_Ca_10186	NON_SYNONYMOUS_CODING	Disease resistance protein
Kabuli_wild_33399405	1399934360	*Ca_kabuli_Chr02*	33399405	C/T	Gene_Ca_10186	SYNONYMOUS_CODING	Disease resistance protein
Kabuli_wild_33399619	1399934361	*Ca_kabuli_Chr02*	33399619	C/T	Gene_Ca_10186	STOP_GAINED	Disease resistance protein
Kabuli_wild_33401608	1399934362	*Ca_kabuli_Chr02*	33401608	A/G	Gene_Ca_10186	SYNONYMOUS_CODING	Disease resistance protein
Kabuli_wild_33490058	1399934363	*Ca_kabuli_Chr02*	33490058	C/A	Gene_Ca_10183	NON_SYNONYMOUS_CODING	Telomerase activating protein Est1
Kabuli_wild_33575286	1399934364	*Ca_kabuli_Chr02*	33575286	A/C	Gene_Ca_10178	UPSTREAM	Fcf2pre-rRNAprocessing
Kabuli_wild_33575432	1399934365	*Ca_kabuli_Chr02*	33575432	G/A	Gene_Ca_10178	UPSTREAM	Fcf2pre-rRNAprocessing
Kabuli_wild_33582897	1399934366	*Ca_kabuli_Chr02*	33582897	G/A	Gene_Ca_10177	NON_SYNONYMOUS_CODING	Peptidase C48, SUMO/Sentrin/Ubl1
Kabuli_wild_33582900	1399934367	*Ca_kabuli_Chr02*	33582900	G/A	Gene_Ca_10177	NON_SYNONYMOUS_CODING	Peptidase C48, SUMO/Sentrin/Ubl1
Kabuli_wild_33582907	1399934368	*Ca_kabuli_Chr02*	33582907	C/T	Gene_Ca_10177	NON_SYNONYMOUS_CODING	Peptidase C48, SUMO/Sentrin/Ubl1
Kabuli_wild_33582909	1399934369	*Ca_kabuli_Chr02*	33582909	G/A	Gene_Ca_10177	NON_SYNONYMOUS_CODING	Peptidase C48, SUMO/Sentrin/Ubl1
Kabuli_wild_33582925	1399934370	*Ca_kabuli_Chr02*	33582925	G/T	Gene_Ca_10177	NON_SYNONYMOUS_CODING	Peptidase C48, SUMO/Sentrin/Ubl1
Kabuli_wild_33582948	1399934371	*Ca_kabuli_Chr02*	33582948	G/A	Gene_Ca_10177	NON_SYNONYMOUS_CODING	Peptidase C48, SUMO/Sentrin/Ubl1
Kabuli_wild_33582959	1399934372	*Ca_kabuli_Chr02*	33582959	A/C	Gene_Ca_10177	SYNONYMOUS_CODING	Peptidase C48, SUMO/Sentrin/Ubl1
Kabuli_wild_33582967	1399934373	*Ca_kabuli_Chr02*	33582967	G/A	Gene_Ca_10177	SYNONYMOUS_CODING	Peptidase C48, SUMO/Sentrin/Ubl1
Kabuli_wild_33582984	1399934374	*Ca_kabuli_Chr02*	33582984	G/T	Gene_Ca_10177	NON_SYNONYMOUS_CODING	Peptidase C48, SUMO/Sentrin/Ubl1
Kabuli_wild_33650447	1399934375	*Ca_kabuli_Chr02*	33650447	A/T	NA	INTERGENIC	NA
Kabuli_wild_33651408	1399934376	*Ca_kabuli_Chr02*	33651408	A/G	NA	INTERGENIC	NA
Kabuli_wild_33651426	1399934377	*Ca_kabuli_Chr02*	33651426	G/A	Gene_Ca_10173	SYNONYMOUS_CODING	Pentatrico peptide repeat
Kabuli_wild_33661177	1399934378	*Ca_kabuli_Chr02*	33661177	C/T	Gene_Ca_10172	SYNONYMOUS_CODING	NA
Kabuli_wild_33713018	1399934379	*Ca_kabuli_Chr02*	33713018	G/A	NA	INTERGENIC	NA
Kabuli_wild_33782771	1399934380	*Ca_kabuli_Chr02*	33782771	T/C	NA	INTERGENIC	NA
Kabuli_wild_33786243	1399934381	*Ca_kabuli_Chr02*	33786243	G/A	Gene_Ca_10158	DOWNSTREAM	Multiantimicrobial extrusion protein
Kabuli_wild_33788913	1399934382	*Ca_kabuli_Chr02*	33788913	A/C	NA	INTERGENIC	NA
Kabuli_wild_33816213	1399934383	*Ca_kabuli_Chr02*	33816213	C/T	NA	INTERGENIC	NA
Kabuli_wild_33920720	1399934384	*Ca_kabuli_Chr02*	33920720	A/G	NA	INTERGENIC	NA
Kabuli_wild_34152352	1399934385	*Ca_kabuli_Chr02*	34152352	T/C	Gene_Ca_15238	INTRON	NA
Kabuli_wild_34155090	1399934386	*Ca_kabuli_Chr02*	34155090	A/G	Gene_Ca_15238	INTRON	NA
Kabuli_wild_34191825	1399934387	*Ca_kabuli_Chr02*	34191825	T/G	NA	INTERGENIC	NA
Kabuli_wild_34223049	1399934388	*Ca_kabuli_Chr02*	34223049	T/C	Gene_Ca_15247	NON_SYNONYMOUS_CODING	ConservedoligomericGolgicomplex,subunit5
Kabuli_wild_34250067	1399934389	*Ca_kabuli_Chr02*	34250067	C/T	NA	INTERGENIC	NA
Kabuli_wild_34266320	1399934390	*Ca_kabuli_Chr02*	34266320	T/G	Gene_Ca_15250	SYNONYMOUS_CODING	Protein kinase,catalytic domain
Kabuli_wild_34288116	1399934391	*Ca_kabuli_Chr02*	34288116	G/C	Gene_Ca_15252	DOWNSTREAM	NA
Kabuli_wild_34299074	1399934392	*Ca_kabuli_Chr02*	34299074	A/C	Gene_Ca_15256	DOWNSTREAM	Pentatrico peptide repeat
Kabuli_wild_34299079	1399934393	*Ca_kabuli_Chr02*	34299079	A/C	Gene_Ca_15256	DOWNSTREAM	Pentatrico peptide repeat
Kabuli_wild_34343478	1399934394	*Ca_kabuli_Chr02*	34343478	C/G	NA	INTERGENIC	NA
Kabuli_wild_34343705	1399934395	*Ca_kabuli_Chr02*	34343705	G/A	NA	INTERGENIC	NA
Kabuli_wild_34346842	1399934396	*Ca_kabuli_Chr02*	34346842	T/C	NA	INTERGENIC	NA
Kabuli_wild_34346849	1399934397	*Ca_kabuli_Chr02*	34346849	C/T	NA	INTERGENIC	NA
Kabuli_wild_34372503	1399934398	*Ca_kabuli_Chr02*	34372503	T/G	NA	INTERGENIC	NA
Kabuli_wild_34428978	1399934399	*Ca_kabuli_Chr02*	34428978	A/C	Gene_Ca_15270	DOWNSTREAM	NA

SNP IDs	NCBI dbSNP accession IDs (NCBI_SS#)	Chromosomes	Physical positions (bp)	SNPs	Gene accession IDs	Sequence components of genome	Putative functions
Kabuli_wild_34428979	1399934400	*Ca_kabuli_Chr02*	34428979	G/C	Gene_Ca_15270	DOWNSTREAM	NA
Kabuli_wild_34551849	1399934401	*Ca_kabuli_Chr02*	34551849	A/C	Gene_Ca_15282	UPSTREAM	Drug/metabolitetransporter
Kabuli_wild_34554164	1399934402	*Ca_kabuli_Chr02*	34554164	A/C	Gene_Ca_15281	UPSTREAM	Vacuolar protein sorting-associated protein Like-Smribonucleo protein (LSM) domain
Kabuli_wild_34601444	1399934403	*Ca_kabuli_Chr02*	34601444	C/A	Gene_Ca_15284	SYNONYMOUS_CODING	Lipocalin
Kabuli_wild_34630993	1399934404	*Ca_kabuli_Chr02*	34630993	G/A	NA	INTERGENIC	NA
Kabuli_wild_34638955	1399934405	*Ca_kabuli_Chr02*	34638955	T/A	NA	INTERGENIC	NA
Kabuli_wild_34734379	1399934406	*Ca_kabuli_Chr02*	34734379	G/A	Gene_Ca_16897	SYNONYMOUS_CODING	Heat shock protein Hsp70
Kabuli_wild_34734475	1399934407	*Ca_kabuli_Chr02*	34734475	A/G	Gene_Ca_16897	SYNONYMOUS_CODING	Heat shock protein Hsp70
Kabuli_wild_34820075	1399934408	*Ca_kabuli_Chr02*	34820075	C/T	NA	INTERGENIC	NA
Kabuli_wild_34830995	1399934409	*Ca_kabuli_Chr02*	34830995	A/G	NA	INTERGENIC	NA
Kabuli_wild_34847681	1399934410	*Ca_kabuli_Chr02*	34847681	C/A	Gene_Ca_16883	SYNONYMOUS_CODING	Bromodomain
Kabuli_wild_34848053	1399934411	*Ca_kabuli_Chr02*	34848053	G/A	Gene_Ca_16883	SYNONYMOUS_CODING	Bromodomain
Kabuli_wild_34848480	1399934412	*Ca_kabuli_Chr02*	34848480	T/C	Gene_Ca_16883	NON_SYNONYMOUS_CODING	Bromodomain
Kabuli_wild_34862276	1399934413	*Ca_kabuli_Chr02*	34862276	C/G	Gene_Ca_16882	NON_SYNONYMOUS_CODING	Lipase,GDSL
Kabuli_wild_34884068	1399934414	*Ca_kabuli_Chr02*	34884068	T/G	Gene_Ca_16880	SYNONYMOUS_CODING	NA
Kabuli_wild_34884122	1399934415	*Ca_kabuli_Chr02*	34884122	T/C	Gene_Ca_16880	SYNONYMOUS_CODING	NA
Kabuli_wild_34884228	1399934416	*Ca_kabuli_Chr02*	34884228	A/G	Gene_Ca_16880	NON_SYNONYMOUS_CODING	NA
Kabuli_wild_34888414	1399934417	*Ca_kabuli_Chr02*	34888414	T/C	Gene_Ca_16880	DOWNSTREAM	NA
Kabuli_wild_35013084	1399934418	*Ca_kabuli_Chr02*	35013084	T/C	Gene_Ca_16871	DOWNSTREAM	Celldifferentiation,Rcd1-like
Kabuli_wild_35013088	1399934419	*Ca_kabuli_Chr02*	35013088	G/C	Gene_Ca_16871	DOWNSTREAM	Celldifferentiation,Rcd1-like
Kabuli_wild_35046954	1399934420	*Ca_kabuli_Chr02*	35046954	C/A	Gene_Ca_16869	NON_SYNONYMOUS_CODING	SWAP/Surp
Kabuli_wild_35052930	1399934421	*Ca_kabuli_Chr02*	35052930	C/A	Gene_Ca_16868	INTRON	Aspartate carbamoyltransferase, eukaryotic
Kabuli_wild_35054244	1399934422	*Ca_kabuli_Chr02*	35054244	C/A	Gene_Ca_16868	INTRON	Aspartate carbamoyltransferase, eukaryotic
Kabuli_wild_35059434	1399934423	*Ca_kabuli_Chr02*	35059434	T/C	NA	INTERGENIC	NA
Kabuli_wild_35059446	1399934424	*Ca_kabuli_Chr02*	35059446	A/T	NA	INTERGENIC	NA
Kabuli_wild_35060171	1399934425	*Ca_kabuli_Chr02*	35060171	A/G	NA	INTERGENIC	NA
Kabuli_wild_35060178	1399934426	*Ca_kabuli_Chr02*	35060178	A/T	NA	INTERGENIC	NA
Kabuli_wild_35060228	1399934427	*Ca_kabuli_Chr02*	35060228	T/C	NA	INTERGENIC	NA
Kabuli_wild_35060264	1399934428	*Ca_kabuli_Chr02*	35060264	A/T	NA	INTERGENIC	NA
Kabuli_wild_35060273	1399934429	*Ca_kabuli_Chr02*	35060273	G/A	NA	INTERGENIC	NA
Kabuli_wild_35064514	1399934430	*Ca_kabuli_Chr02*	35064514	A/T	Gene_Ca_16867	UPSTREAM	UBA/THIF-typeNAD/FADbindingfold
Kabuli_wild_35064519	1399934431	*Ca_kabuli_Chr02*	35064519	T/C	Gene_Ca_16867	UPSTREAM	UBA/THIF-typeNAD/FADbindingfold
Kabuli_wild_35066138	1399934432	*Ca_kabuli_Chr02*	35066138	G/A	NA	INTERGENIC	NA
Kabuli_wild_35096629	1399934433	*Ca_kabuli_Chr02*	35096629	C/A	Gene_Ca_16862	INTRON	Microtubule-associatedprotein,MAP65/ASE1-type
Kabuli_wild_35096630	1399934434	*Ca_kabuli_Chr02*	35096630	C/G	Gene_Ca_16862	INTRON	Microtubule-associatedprotein,MAP65/ASE1-type
Kabuli_wild_35096631	1399934435	*Ca_kabuli_Chr02*	35096631	A/C	Gene_Ca_16862	INTRON	Microtubule-associatedprotein,MAP65/ASE1-type
Kabuli_wild_35096633	1399934436	*Ca_kabuli_Chr02*	35096633	C/T	Gene_Ca_16862	INTRON	Microtubule-associatedprotein,MAP65/ASE1-type
Kabuli_wild_35102042	1399934437	*Ca_kabuli_Chr02*	35102042	A/C	NA	INTERGENIC	NA
Kabuli_wild_35120761	1399934438	*Ca_kabuli_Chr02*	35120761	G/A	Gene_Ca_16857	SYNONYMOUS_CODING	Metallo-dependentphosphatase
Kabuli_wild_35122109	1399934439	*Ca_kabuli_Chr02*	35122109	C/G	Gene_Ca_16857	INTRON	Metallo-dependentphosphatase
Kabuli_wild_35154603	1399934440	*Ca_kabuli_Chr02*	35154603	G/A	Gene_Ca_16851	DOWNSTREAM	Pentatrico peptide repeat
Kabuli_wild_35154629	1399934441	*Ca_kabuli_Chr02*	35154629	C/A	Gene_Ca_16851	DOWNSTREAM	Pentatrico peptide repeat
Kabuli_wild_35171882	1399934442	*Ca_kabuli_Chr02*	35171882	T/G	Gene_Ca_16849	SYNONYMOUS_CODING	Glycerophosphoryldiester phosphodiesterase
Kabuli_wild_35189471	1399934443	*Ca_kabuli_Chr02*	35189471	C/T	NA	INTERGENIC	NA

SNP IDs	NCBI dbSNP accession IDs (NCBI_SS#)	Chromosomes	Physical positions (bp)	SNPs	Gene accession IDs	Sequence components of genome	Putative functions
Kabuli_wild_35189750	1399934444	*Ca_kabuli_Chr02*	35189750	T/A	NA	INTERGENIC	NA
Kabuli_wild_35209940	1399934445	*Ca_kabuli_Chr02*	35209940	C/T	Gene_Ca_16844	NON_SYNONYMOUS_CODING	C2 calcium-dependent membrane targeting
Kabuli_wild_35362587	1399934446	*Ca_kabuli_Chr02*	35362587	A/G	NA	INTERGENIC	NA
Kabuli_wild_35362642	1399934447	*Ca_kabuli_Chr02*	35362642	A/G	NA	INTERGENIC	NA
Kabuli_wild_35362729	1399934448	*Ca_kabuli_Chr02*	35362729	A/G	Gene_Ca_09693	SYNONYMOUS_CODING	Frigida-like
Kabuli_wild_35363501	1399934449	*Ca_kabuli_Chr02*	35363501	T/C	Gene_Ca_09693	NON_SYNONYMOUS_CODING	Frigida-like
Kabuli_wild_35365029	1399934450	*Ca_kabuli_Chr02*	35365029	C/T	Gene_Ca_09693	NON_SYNONYMOUS_CODING	Frigida-like
Kabuli_wild_35377057	1399934451	*Ca_kabuli_Chr02*	35377057	A/T	Gene_Ca_09694	SYNONYMOUS_CODING	DNA-binding WRKY
Kabuli_wild_35413400	1399934452	*Ca_kabuli_Chr02*	35413400	T/A	NA	INTERGENIC	NA
Kabuli_wild_35441174	1399934453	*Ca_kabuli_Chr02*	35441174	T/G	Gene_Ca_09704	UPSTREAM	Pentatrico peptide repeat
Kabuli_wild_35441180	1399934454	*Ca_kabuli_Chr02*	35441180	A/G	Gene_Ca_09704	UPSTREAM	Pentatrico peptide repeat
Kabuli_wild_35447976	1399934455	*Ca_kabuli_Chr02*	35447976	A/G	NA	INTERGENIC	NA
Kabuli_wild_35449653	1399934456	*Ca_kabuli_Chr02*	35449653	G/A	Gene_Ca_09705	INTRON	ABC transporter,trans membrane domain
Kabuli_wild_35473250	1399934457	*Ca_kabuli_Chr02*	35473250	C/T	Gene_Ca_09708	UPSTREAM	Domain of unknown function DUF2040
Kabuli_wild_35505791	1399934458	*Ca_kabuli_Chr02*	35505791	C/G	NA	INTERGENIC	NA
Kabuli_wild_35505874	1399934459	*Ca_kabuli_Chr02*	35505874	T/G	NA	INTERGENIC	NA
Kabuli_wild_35593103	1399934460	*Ca_kabuli_Chr02*	35593103	T/A	NA	INTERGENIC	NA
Kabuli_wild_35593355	1399934461	*Ca_kabuli_Chr02*	35593355	C/G	NA	INTERGENIC	NA
Kabuli_wild_35593363	1399934462	*Ca_kabuli_Chr02*	35593363	C/T	NA	INTERGENIC	NA
Kabuli_wild_35593518	1399934463	*Ca_kabuli_Chr02*	35593518	G/A	Gene_Ca_09728	NON_SYNONYMOUS_CODING	BacterialFmu(Sun)/eukaryoticnucleolarNOL1/Nop2p
Kabuli_wild_35593692	1399934464	*Ca_kabuli_Chr02*	35593692	C/G	Gene_Ca_09728	INTRON	BacterialFmu(Sun)/eukaryoticnucleolarNOL1/Nop2p
Kabuli_wild_35593713	1399934465	*Ca_kabuli_Chr02*	35593713	T/C	Gene_Ca_09728	INTRON	BacterialFmu(Sun)/eukaryoticnucleolarNOL1/Nop2p
Kabuli_wild_35593731	1399934466	*Ca_kabuli_Chr02*	35593731	A/G	Gene_Ca_09728	INTRON	BacterialFmu(Sun)/eukaryoticnucleolarNOL1/Nop2p
Kabuli_wild_35593749	1399934467	*Ca_kabuli_Chr02*	35593749	T/C	Gene_Ca_09728	SYNONYMOUS_CODING	BacterialFmu(Sun)/eukaryoticnucleolarNOL1/Nop2p
Kabuli_wild_35593797	1399934468	*Ca_kabuli_Chr02*	35593797	G/T	Gene_Ca_09728	SYNONYMOUS_CODING	BacterialFmu(Sun)/eukaryoticnucleolarNOL1/Nop2p
Kabuli_wild_35593824	1399934469	*Ca_kabuli_Chr02*	35593824	A/C	Gene_Ca_09728	SYNONYMOUS_CODING	BacterialFmu(Sun)/eukaryoticnucleolarNOL1/Nop2p
Kabuli_wild_35593827	1399934470	*Ca_kabuli_Chr02*	35593827	A/G	Gene_Ca_09728	SYNONYMOUS_CODING	BacterialFmu(Sun)/eukaryoticnucleolarNOL1/Nop2p
Kabuli_wild_35593940	1399934471	*Ca_kabuli_Chr02*	35593940	G/A	Gene_Ca_09728	INTRON	BacterialFmu(Sun)/eukaryoticnucleolarNOL1/Nop2p
Kabuli_wild_35593956	1399934472	*Ca_kabuli_Chr02*	35593956	A/G	Gene_Ca_09728	INTRON	BacterialFmu(Sun)/eukaryoticnucleolarNOL1/Nop2p
Kabuli_wild_35593965	1399934473	*Ca_kabuli_Chr02*	35593965	C/T	Gene_Ca_09728	INTRON	BacterialFmu(Sun)/eukaryoticnucleolarNOL1/Nop2p
Kabuli_wild_35593985	1399934474	*Ca_kabuli_Chr02*	35593985	A/G	Gene_Ca_09728	INTRON	BacterialFmu(Sun)/eukaryoticnucleolarNOL1/Nop2p
Kabuli_wild_35594013	1399934475	*Ca_kabuli_Chr02*	35594013	C/T	Gene_Ca_09728	INTRON	BacterialFmu(Sun)/eukaryoticnucleolarNOL1/Nop2p
Kabuli_wild_35594018	1399934476	*Ca_kabuli_Chr02*	35594018	A/T	Gene_Ca_09728	INTRON	BacterialFmu(Sun)/eukaryoticnucleolarNOL1/Nop2p
Kabuli_wild_35601566	1399934477	*Ca_kabuli_Chr02*	35601566	T/C	Gene_Ca_09729	DOWNSTREAM	Pathogenic type III effect or avirulence factor Avr cleavage site
Kabuli_wild_35601571	1399934478	*Ca_kabuli_Chr02*	35601571	A/C	Gene_Ca_09729	DOWNSTREAM	Pathogenic type III effect or avirulence factor Avr cleavage site
Kabuli_wild_35601578	1399934479	*Ca_kabuli_Chr02*	35601578	C/T	Gene_Ca_09729	DOWNSTREAM	Pathogenic type III effect or avirulence factor Avr cleavage site
Kabuli_wild_35601632	1399934480	*Ca_kabuli_Chr02*	35601632	G/T	Gene_Ca_09729	DOWNSTREAM	Pathogenic type III effect or avirulence factor Avr cleavage site
Kabuli_wild_35601641	1399934481	*Ca_kabuli_Chr02*	35601641	T/A	Gene_Ca_09729	DOWNSTREAM	Pathogenic type III effect or avirulence factor Avr cleavage site
Kabuli_wild_35601657	1399934482	*Ca_kabuli_Chr02*	35601657	T/C	Gene_Ca_09729	DOWNSTREAM	Pathogenic type III effect or avirulence factor Avr cleavage site
Kabuli_wild_35601691	1399934483	*Ca_kabuli_Chr02*	35601691	A/G	Gene_Ca_09729	DOWNSTREAM	Pathogenic type III effect or avirulence factor Avr cleavage site
Kabuli_wild_35612587	1399934484	*Ca_kabuli_Chr02*	35612587	C/A	Gene_Ca_09731	SYNONYMOUS_CODING	Protein of unknown function DUF827,plant
Kabuli_wild_35612644	1399934485	*Ca_kabuli_Chr02*	35612644	G/A	Gene_Ca_09731	SYNONYMOUS_CODING	Protein of unknown function DUF827,plant
Kabuli_wild_35612895	1399934486	*Ca_kabuli_Chr02*	35612895	T/C	Gene_Ca_09731	NON_SYNONYMOUS_CODING	Protein of unknown function DUF827,plant
Kabuli_wild_35612900	1399934487	*Ca_kabuli_Chr02*	35612900	C/T	Gene_Ca_09731	NON_SYNONYMOUS_CODING	Protein of unknown function DUF827,plant

SNP IDs	NCBI dbSNP accession IDs (NCBI_SS#)	Chromosomes	Physical positions (bp)	SNPs	Gene accession IDs	Sequence components of genome	Putative functions
Kabuli_wild_35613135	1399934488	*Ca_kabuli_Chr02*	35613135	T/C	Gene_Ca_09731	DOWNSTREAM	Protein of unknown function DUF827,plant
Kabuli_wild_35618351	1399934489	*Ca_kabuli_Chr02*	35618351	T/C	Gene_Ca_09732	SYNONYMOUS_CODING	Sucraseferredoxin-like
Kabuli_wild_35658537	1399934490	*Ca_kabuli_Chr02*	35658537	T/C	Gene_Ca_09735	SYNONYMOUS_CODING	Oligo peptide transporterOPTsuperfamily
Kabuli_wild_35661053	1399934491	*Ca_kabuli_Chr02*	35661053	C/T	Gene_Ca_09735	NON_SYNONYMOUS_CODING	Oligo peptide transporterOPTsuperfamily
Kabuli_wild_35670478	1399934492	*Ca_kabuli_Chr02*	35670478	A/G	Gene_Ca_09737	INTRON	Protein of unknown function DUF1296
Kabuli_wild_35672503	1399934493	*Ca_kabuli_Chr02*	35672503	T/G	Gene_Ca_09737	NON_SYNONYMOUS_CODING	Protein of unknown function DUF1296
Kabuli_wild_35673262	1399934494	*Ca_kabuli_Chr02*	35673262	T/C	Gene_Ca_09737	INTRON	Protein of unknown function DUF1296
Kabuli_wild_35676127	1399934495	*Ca_kabuli_Chr02*	35676127	G/A	Gene_Ca_09737	SYNONYMOUS_CODING	Protein of unknown function DUF1296
Kabuli_wild_35676160	1399934496	*Ca_kabuli_Chr02*	35676160	C/T	Gene_Ca_09737	SYNONYMOUS_CODING	Protein of unknown function DUF1296
Kabuli_wild_35704018	1399934497	*Ca_kabuli_Chr02*	35704018	A/G	NA	INTERGENIC	NA
Kabuli_wild_35704069	1399934498	*Ca_kabuli_Chr02*	35704069	G/A	NA	INTERGENIC	NA
Kabuli_wild_35704104	1399934499	*Ca_kabuli_Chr02*	35704104	G/T	NA	INTERGENIC	NA
Kabuli_wild_35704178	1399934500	*Ca_kabuli_Chr02*	35704178	G/A	NA	INTERGENIC	NA
Kabuli_wild_35704180	1399934501	*Ca_kabuli_Chr02*	35704180	A/G	NA	INTERGENIC	NA
Kabuli_wild_35704200	1399934502	*Ca_kabuli_Chr02*	35704200	T/C	NA	INTERGENIC	NA
Kabuli_wild_35704204	1399934503	*Ca_kabuli_Chr02*	35704204	T/C	NA	INTERGENIC	NA
Kabuli_wild_35704213	1399934504	*Ca_kabuli_Chr02*	35704213	A/G	NA	INTERGENIC	NA
Kabuli_wild_35704228	1399934505	*Ca_kabuli_Chr02*	35704228	C/A	NA	INTERGENIC	NA
Kabuli_wild_35721109	1399934506	*Ca_kabuli_Chr02*	35721109	T/C	NA	INTERGENIC	NA
Kabuli_wild_35721111	1399934507	*Ca_kabuli_Chr02*	35721111	C/G	NA	INTERGENIC	NA
Kabuli_wild_35721124	1399934508	*Ca_kabuli_Chr02*	35721124	G/A	NA	INTERGENIC	NA
Kabuli_wild_35721134	1399934509	*Ca_kabuli_Chr02*	35721134	C/A	NA	INTERGENIC	NA
Kabuli_wild_35721150	1399934510	*Ca_kabuli_Chr02*	35721150	T/C	NA	INTERGENIC	NA
Kabuli_wild_35727800	1399934511	*Ca_kabuli_Chr02*	35727800	A/G	Gene_Ca_09744	DOWNSTREAM	NA
Kabuli_wild_35728285	1399934512	*Ca_kabuli_Chr02*	35728285	T/A	Gene_Ca_09744	DOWNSTREAM	NA
Kabuli_wild_35754287	1399934513	*Ca_kabuli_Chr02*	35754287	T/C	NA	INTERGENIC	NA
Kabuli_wild_35754296	1399934514	*Ca_kabuli_Chr02*	35754296	A/G	NA	INTERGENIC	NA
Kabuli_wild_35754350	1399934515	*Ca_kabuli_Chr02*	35754350	A/G	NA	INTERGENIC	NA
Kabuli_wild_35754352	1399934516	*Ca_kabuli_Chr02*	35754352	A/G	NA	INTERGENIC	NA
Kabuli_wild_35754557	1399934517	*Ca_kabuli_Chr02*	35754557	C/T	Gene_Ca_09749	DOWNSTREAM	NA
Kabuli_wild_35754617	1399934518	*Ca_kabuli_Chr02*	35754617	G/T	Gene_Ca_09749	DOWNSTREAM	Regulatory protein RecX
Kabuli_wild_35754656	1399934519	*Ca_kabuli_Chr02*	35754656	C/T	Gene_Ca_09749	DOWNSTREAM	Regulatory protein RecX
Kabuli_wild_35754680	1399934520	*Ca_kabuli_Chr02*	35754680	C/T	Gene_Ca_09749	DOWNSTREAM	Regulatory protein RecX
Kabuli_wild_35754681	1399934521	*Ca_kabuli_Chr02*	35754681	T/G	Gene_Ca_09749	DOWNSTREAM	Regulatory protein RecX
Kabuli_wild_35754682	1399934522	*Ca_kabuli_Chr02*	35754682	A/G	Gene_Ca_09749	DOWNSTREAM	Regulatory protein RecX
Kabuli_wild_35754757	1399934523	*Ca_kabuli_Chr02*	35754757	G/C	Gene_Ca_09749	DOWNSTREAM	Regulatory protein RecX
Kabuli_wild_35754806	1399934524	*Ca_kabuli_Chr02*	35754806	G/C	Gene_Ca_09749	DOWNSTREAM	Regulatory protein RecX
Kabuli_wild_35759932	1399934525	*Ca_kabuli_Chr02*	35759932	A/T	Gene_Ca_09749	INTRON	Regulatory protein RecX
Kabuli_wild_35759940	1399934526	*Ca_kabuli_Chr02*	35759940	T/C	Gene_Ca_09749	INTRON	Regulatory protein RecX
Kabuli_wild_35759981	1399934527	*Ca_kabuli_Chr02*	35759981	G/A	Gene_Ca_09749	INTRON	Regulatory protein RecX
Kabuli_wild_35760062	1399934528	*Ca_kabuli_Chr02*	35760062	G/C	Gene_Ca_09749	INTRON	Regulatory protein RecX
Kabuli_wild_35760073	1399934529	*Ca_kabuli_Chr02*	35760073	A/T	Gene_Ca_09749	INTRON	Regulatory protein RecX
Kabuli_wild_35760155	1399934530	*Ca_kabuli_Chr02*	35760155	G/A	Gene_Ca_09749	INTRON	Regulatory protein RecX
Kabuli_wild_35760170	1399934531	*Ca_kabuli_Chr02*	35760170	A/G	Gene_Ca_09749	INTRON	Regulatory protein RecX

SNP IDs	NCBI dbSNP accession IDs (NCBI_SS#)	Chromosomes	Physical positions (bp)	SNPs	Gene accession IDs	Sequence components of genome	Putative functions
Kabuli_wild_35795383	1399934532	*Ca_kabuli_Chr02*	35795383	T/G	Gene_Ca_09754	SYNONYMOUS_CODING	PeptidaseS8/S53,subtilisin/kexin/sedolisin
Kabuli_wild_35795395	1399934533	*Ca_kabuli_Chr02*	35795395	A/G	Gene_Ca_09754	SYNONYMOUS_CODING	PeptidaseS8/S53,subtilisin/kexin/sedolisin
Kabuli_wild_35795494	1399934534	*Ca_kabuli_Chr02*	35795494	G/A	Gene_Ca_09754	SYNONYMOUS_CODING	PeptidaseS8/S53,subtilisin/kexin/sedolisin
Kabuli_wild_35795496	1399934535	*Ca_kabuli_Chr02*	35795496	G/T	Gene_Ca_09754	NON_SYNONYMOUS_CODING	PeptidaseS8/S53,subtilisin/kexin/sedolisin
Kabuli_wild_35795521	1399934536	*Ca_kabuli_Chr02*	35795521	T/C	Gene_Ca_09754	SYNONYMOUS_CODING	PeptidaseS8/S53,subtilisin/kexin/sedolisin
Kabuli_wild_35835232	1399934537	*Ca_kabuli_Chr02*	35835232	A/C	NA	INTERGENIC	NA
Kabuli_wild_35835234	1399934538	*Ca_kabuli_Chr02*	35835234	C/T	NA	INTERGENIC	NA
Kabuli_wild_35842676	1399934539	*Ca_kabuli_Chr02*	35842676	C/T	NA	INTERGENIC	NA
Kabuli_wild_35862878	1399934540	*Ca_kabuli_Chr02*	35862878	T/G	Gene_Ca_09762	INTRON	Armadillo
Kabuli_wild_35862891	1399934541	*Ca_kabuli_Chr02*	35862891	C/A	Gene_Ca_09762	INTRON	Armadillo
Kabuli_wild_35917717	1399934542	*Ca_kabuli_Chr02*	35917717	A/G	Gene_Ca_09767	INTRON	Nucleotidyltransferase
Kabuli_wild_35930699	1399934543	*Ca_kabuli_Chr02*	35930699	T/G	NA	INTERGENIC	NA
Kabuli_wild_35987869	1399934544	*Ca_kabuli_Chr02*	35987869	T/C	Gene_Ca_09773	DOWNSTREAM	Disease resistance protein
Kabuli_wild_35987871	1399934545	*Ca_kabuli_Chr02*	35987871	T/C	Gene_Ca_09773	DOWNSTREAM	Disease resistance protein
Kabuli_wild_35987920	1399934546	*Ca_kabuli_Chr02*	35987920	A/C	Gene_Ca_09773	DOWNSTREAM	Disease resistance protein
Kabuli_wild_35993178	1399934547	*Ca_kabuli_Chr02*	35993178	G/A	Gene_Ca_09773	SYNONYMOUS_CODING	Disease resistance protein
Kabuli_wild_36013809	1399934548	*Ca_kabuli_Chr02*	36013809	A/G	Gene_Ca_09776	INTRON	Nucleoporin,Nup133/Nup155-like,C-terminal
Kabuli_wild_36016810	1399934549	*Ca_kabuli_Chr02*	36016810	C/T	Gene_Ca_09776	INTRON	Nucleoporin,Nup133/Nup155-like,C-terminal
Kabuli_wild_36039364	1399934550	*Ca_kabuli_Chr02*	36039364	G/T	Gene_Ca_09779	DOWNSTREAM	ChlorophyllA-Bbindingprotein,plant
Kabuli_wild_36039428	1399934551	*Ca_kabuli_Chr02*	36039428	C/T	Gene_Ca_09779	DOWNSTREAM	ChlorophyllA-Bbindingprotein,plant
Kabuli_wild_36046593	1399934552	*Ca_kabuli_Chr02*	36046593	G/T	NA	INTERGENIC	NA
Kabuli_wild_36046595	1399934553	*Ca_kabuli_Chr02*	36046595	T/G	NA	INTERGENIC	NA
Kabuli_wild_36046720	1399934554	*Ca_kabuli_Chr02*	36046720	T/A	NA	INTERGENIC	NA
Kabuli_wild_36046741	1399934555	*Ca_kabuli_Chr02*	36046741	G/A	NA	INTERGENIC	NA
Kabuli_wild_36046767	1399934556	*Ca_kabuli_Chr02*	36046767	T/A	NA	INTERGENIC	NA
Kabuli_wild_36046820	1399934557	*Ca_kabuli_Chr02*	36046820	T/A	NA	INTERGENIC	NA
Kabuli_wild_36051175	1399934558	*Ca_kabuli_Chr02*	36051175	T/G	NA	INTERGENIC	NA
Kabuli_wild_36054956	1399934559	*Ca_kabuli_Chr02*	36054956	G/T	NA	INTERGENIC	NA
Kabuli_wild_36057418	1399934560	*Ca_kabuli_Chr02*	36057418	A/G	Gene_Ca_09782	DOWNSTREAM	Zinc finger,PHD-type
Kabuli_wild_36068124	1399934561	*Ca_kabuli_Chr02*	36068124	C/T	Gene_Ca_09784	SYNONYMOUS_CODING	Signal transduction response regulator, receiverdomain
Kabuli_wild_36069340	1399934562	*Ca_kabuli_Chr02*	36069340	T/C	Gene_Ca_09784	SYNONYMOUS_CODING	Signal transduction response regulator, receiverdomain
Kabuli_wild_36072247	1399934563	*Ca_kabuli_Chr02*	36072247	C/A	Gene_Ca_09785	UPSTREAM	X8
Kabuli_wild_36074147	1399934564	*Ca_kabuli_Chr02*	36074147	T/C	NA	INTERGENIC	NA
Kabuli_wild_36088577	1399934565	*Ca_kabuli_Chr02*	36088577	A/T	Gene_Ca_09787	DOWNSTREAM	DNA-binding WRKY
Kabuli_wild_36088676	1399934566	*Ca_kabuli_Chr02*	36088676	T/C	Gene_Ca_09787	DOWNSTREAM	DNA-binding WRKY
Kabuli_wild_36088853	1399934567	*Ca_kabuli_Chr02*	36088853	G/A	Gene_Ca_09787	DOWNSTREAM	DNA-binding WRKY
Kabuli_wild_36101358	1399934568	*Ca_kabuli_Chr02*	36101358	A/G	NA	INTERGENIC	NA
Kabuli_wild_36101379	1399934569	*Ca_kabuli_Chr02*	36101379	A/G	NA	INTERGENIC	NA
Kabuli_wild_36117467	1399934570	*Ca_kabuli_Chr02*	36117467	T/C	Gene_Ca_09790	SYNONYMOUS_CODING	RNA recognition motif domain
Kabuli_wild_36117616	1399934571	*Ca_kabuli_Chr02*	36117616	A/C	Gene_Ca_09790	DOWNSTREAM	RNA recognition motif domain
Kabuli_wild_36117647	1399934572	*Ca_kabuli_Chr02*	36117647	G/A	Gene_Ca_09790	DOWNSTREAM	RNA recognition motif domain
Kabuli_wild_36117695	1399934573	*Ca_kabuli_Chr02*	36117695	T/A	Gene_Ca_09790	DOWNSTREAM	RNA recognition motif domain
Kabuli_wild_36276791	1399934574	*Ca_kabuli_Chr02*	36276791	G/A	NA	INTERGENIC	NA
Kabuli_wild_36326361	1399934575	*Ca_kabuli_Chr02*	36326361	A/C	NA	INTERGENIC	NA

SNP IDs	NCBI dbSNP accession IDs (NCBI_SS#)	Chromosomes	Physical positions (bp)	SNPs	Gene accession IDs	Sequence components of genome	Putative functions
Kabuli_wild_36335252	1399934576	*Ca_kabuli_Chr02*	36335252	A/C	NA	INTERGENIC	NA
Kabuli_wild_36346216	1399934577	*Ca_kabuli_Chr02*	36346216	A/T	Gene_Ca_09821	DOWNSTREAM	CoproporphyrinogenIIIoxidase
Kabuli_wild_36346336	1399934578	*Ca_kabuli_Chr02*	36346336	A/G	Gene_Ca_09821	DOWNSTREAM	CoproporphyrinogenIIIoxidase
Kabuli_wild_36387245	1399934579	*Ca_kabuli_Chr02*	36387245	T/C	Gene_Ca_09828	NON_SYNONYMOUS_CODING	GDP-fucoseproteinO-fucosyltransferase
Kabuli_wild_36403621	1399934580	*Ca_kabuli_Chr02*	36403621	T/C	NA	INTERGENIC	NA
Kabuli_wild_36429512	1399934581	*Ca_kabuli_Chr02*	36429512	T/C	Gene_Ca_09832	NON_SYNONYMOUS_CODING	Phox/Bem1p
Kabuli_wild_36444409	1399934582	*Ca_kabuli_Chr02*	36444409	T/G	Gene_Ca_09833	INTRON	Sterol-sensingdomain
Kabuli_wild_36461953	1399934583	*Ca_kabuli_Chr02*	36461953	C/A	NA	INTERGENIC	NA
Kabuli_wild_36467100	1399934584	*Ca_kabuli_Chr02*	36467100	A/C	NA	INTERGENIC	NA
Kabuli_wild_36469921	1399934585	*Ca_kabuli_Chr02*	36469921	A/G	Gene_Ca_09835	INTRON	EPS15homology(EH)
Kabuli_wild_36484643	1399934586	*Ca_kabuli_Chr02*	36484643	G/A	Gene_Ca_09836	SYNONYMOUS_CODING	Protein kinase,catalytic domain
Kabuli_wild_36505316	1399934587	*Ca_kabuli_Chr02*	36505316	C/A	NA	INTERGENIC	NA
Kabuli_wild_36609730	1399934588	*Ca_kabuli_Chr02*	36609730	T/A	NA	INTERGENIC	NA
Kabuli_wild_36609743	1399934589	*Ca_kabuli_Chr02*	36609743	C/T	NA	INTERGENIC	NA
Kabuli_wild_36609744	1399934590	*Ca_kabuli_Chr02*	36609744	G/C	NA	INTERGENIC	NA
Kabuli_wild_36609950	1399934591	*Ca_kabuli_Chr02*	36609950	C/T	NA	INTERGENIC	NA
Kabuli_wild_36609968	1399934592	*Ca_kabuli_Chr02*	36609968	C/T	NA	INTERGENIC	NA
Kabuli_wild_36634234	1399934593	*Ca_kabuli_Chr02*	36634234	T/C	NA	INTERGENIC	NA
Kabuli_wild_36634297	1399934594	*Ca_kabuli_Chr02*	36634297	C/G	NA	INTERGENIC	NA
Kabuli_wild_171675	1399934595	*Ca_kabuli_Chr03*	171675	T/A	Gene_Ca_22938	SYNONYMOUS_CODING	NAD-dependentepimerase/dehydratase
Kabuli_wild_220568	1399934596	*Ca_kabuli_Chr03*	220568	A/T	Gene_Ca_22935	INTRON	Glycosyltransferase,family48
Kabuli_wild_220572	1399934597	*Ca_kabuli_Chr03*	220572	T/C	Gene_Ca_22935	INTRON	Glycosyltransferase,family48
Kabuli_wild_220573	1399934598	*Ca_kabuli_Chr03*	220573	G/T	Gene_Ca_22935	INTRON	Glycosyltransferase,family48
Kabuli_wild_419715	1399934599	*Ca_kabuli_Chr03*	419715	T/G	NA	INTERGENIC	NA
Kabuli_wild_972745	1399934600	*Ca_kabuli_Chr03*	972745	A/G	NA	INTERGENIC	NA
Kabuli_wild_976189	1399934601	*Ca_kabuli_Chr03*	976189	T/G	NA	INTERGENIC	NA
Kabuli_wild_981846	1399934602	*Ca_kabuli_Chr03*	981846	A/G	NA	INTERGENIC	NA
Kabuli_wild_981852	1399934603	*Ca_kabuli_Chr03*	981852	T/C	NA	INTERGENIC	NA
Kabuli_wild_988477	1399934604	*Ca_kabuli_Chr03*	988477	G/A	NA	INTERGENIC	NA
Kabuli_wild_1499006	1399934605	*Ca_kabuli_Chr03*	1499006	A/G	NA	INTERGENIC	NA
Kabuli_wild_1499021	1399934606	*Ca_kabuli_Chr03*	1499021	G/A	NA	INTERGENIC	NA
Kabuli_wild_1499025	1399934607	*Ca_kabuli_Chr03*	1499025	G/T	NA	INTERGENIC	NA
Kabuli_wild_1499038	1399934608	*Ca_kabuli_Chr03*	1499038	G/A	NA	INTERGENIC	NA
Kabuli_wild_1507113	1399934609	*Ca_kabuli_Chr03*	1507113	T/G	NA	INTERGENIC	NA
Kabuli_wild_1507162	1399934610	*Ca_kabuli_Chr03*	1507162	G/A	NA	INTERGENIC	NA
Kabuli_wild_1507166	1399934611	*Ca_kabuli_Chr03*	1507166	A/T	NA	INTERGENIC	NA
Kabuli_wild_1507167	1399934612	*Ca_kabuli_Chr03*	1507167	C/T	NA	INTERGENIC	NA
Kabuli_wild_1507177	1399934613	*Ca_kabuli_Chr03*	1507177	C/T	NA	INTERGENIC	NA
Kabuli_wild_1507187	1399934614	*Ca_kabuli_Chr03*	1507187	T/C	NA	INTERGENIC	NA
Kabuli_wild_1507225	1399934615	*Ca_kabuli_Chr03*	1507225	C/T	NA	INTERGENIC	NA
Kabuli_wild_1624802	1399934616	*Ca_kabuli_Chr03*	1624802	T/G	Gene_Ca_19409	NON_SYNONYMOUS_CODING	Pyridine nucleotide-disulphideoxido reductase,NAD-binding region
Kabuli_wild_1746388	1399934617	*Ca_kabuli_Chr03*	1746388	C/A	Gene_Ca_19405	INTRON	Helicase,C-terminal
Kabuli_wild_1746389	1399934618	*Ca_kabuli_Chr03*	1746389	A/G	Gene_Ca_19405	INTRON	NA
Kabuli_wild_1829414	1399934619	*Ca_kabuli_Chr03*	1829414	T/C	NA	INTERGENIC	NA

SNP IDs	NCBI dbSNP accession IDs (NCBI_SS#)	Chromosomes	Physical positions (bp)	SNPs	Gene accession IDs	Sequence components of genome	Putative functions
Kabuli_wild_1829423	1399934620	*Ca_kabuli_Chr03*	1829423	A/C	NA	INTERGENIC	NA
Kabuli_wild_1923783	1399934621	*Ca_kabuli_Chr03*	1923783	A/T	NA	INTERGENIC	NA
Kabuli_wild_2097376	1399934622	*Ca_kabuli_Chr03*	2097376	T/G	Gene_Ca_19398	DOWNSTREAM	Alpha/betahydrolasefold-3
Kabuli_wild_2097413	1399934623	*Ca_kabuli_Chr03*	2097413	A/G	Gene_Ca_19398	DOWNSTREAM	Alpha/betahydrolasefold-3
Kabuli_wild_2240403	1399934624	*Ca_kabuli_Chr03*	2240403	G/A	NA	INTERGENIC	NA
Kabuli_wild_2391515	1399934625	*Ca_kabuli_Chr03*	2391515	T/G	Gene_Ca_26372	SYNONYMOUS_CODING	NHLrepeat
Kabuli_wild_2461914	1399934626	*Ca_kabuli_Chr03*	2461914	G/A	NA	INTERGENIC	NA
Kabuli_wild_2708703	1399934627	*Ca_kabuli_Chr03*	2708703	C/T	Gene_Ca_22148	NON_SYNONYMOUS_CODING	Zinc finger,B-box
Kabuli_wild_2825464	1399934628	*Ca_kabuli_Chr03*	2825464	C/A	NA	INTERGENIC	NA
Kabuli_wild_2825546	1399934629	*Ca_kabuli_Chr03*	2825546	G/T	NA	INTERGENIC	NA
Kabuli_wild_2828273	1399934630	*Ca_kabuli_Chr03*	2828273	A/G	Gene_Ca_22150	SYNONYMOUS_CODING	Protein kinase,catalytic domain
Kabuli_wild_2850400	1399934631	*Ca_kabuli_Chr03*	2850400	T/A	NA	INTERGENIC	NA
Kabuli_wild_2927148	1399934632	*Ca_kabuli_Chr03*	2927148	T/A	NA	INTERGENIC	NA
Kabuli_wild_2927150	1399934633	*Ca_kabuli_Chr03*	2927150	A/T	NA	INTERGENIC	NA
Kabuli_wild_2930974	1399934634	*Ca_kabuli_Chr03*	2930974	A/C	Gene_Ca_22158	INTRON	Tetra tricopeptide repeat-containing
Kabuli_wild_2931012	1399934635	*Ca_kabuli_Chr03*	2931012	A/C	Gene_Ca_22158	INTRON	Tetra tricopeptide repeat-containing
Kabuli_wild_2969615	1399934636	*Ca_kabuli_Chr03*	2969615	A/G	NA	INTERGENIC	NA
Kabuli_wild_2975179	1399934637	*Ca_kabuli_Chr03*	2975179	T/C	Gene_Ca_22161	SYNONYMOUS_CODING	NA
Kabuli_wild_2998671	1399934638	*Ca_kabuli_Chr03*	2998671	A/G	NA	INTERGENIC	NA
Kabuli_wild_3127079	1399934639	*Ca_kabuli_Chr03*	3127079	G/T	NA	INTERGENIC	NA
Kabuli_wild_3182276	1399934640	*Ca_kabuli_Chr03*	3182276	T/G	NA	INTERGENIC	NA
Kabuli_wild_3280478	1399934641	*Ca_kabuli_Chr03*	3280478	C/T	Gene_Ca_24555	NON_SYNONYMOUS_CODING	RNA recognition motif domain
Kabuli_wild_3299145	1399934642	*Ca_kabuli_Chr03*	3299145	G/A	NA	INTERGENIC	NA
Kabuli_wild_3442270	1399934643	*Ca_kabuli_Chr03*	3442270	G/A	NA	INTERGENIC	NA
Kabuli_wild_3617555	1399934644	*Ca_kabuli_Chr03*	3617555	G/A	Gene_Ca_21346	NON_SYNONYMOUS_CODING	ZF-HD homeobox protein,Cys/His-rich dimerisation domain
Kabuli_wild_3701370	1399934645	*Ca_kabuli_Chr03*	3701370	G/A	Gene_Ca_21344	INTRON	Gibberellin regulated protein
Kabuli_wild_3701394	1399934646	*Ca_kabuli_Chr03*	3701394	A/T	Gene_Ca_21344	INTRON	Gibberellin regulated protein
Kabuli_wild_3701396	1399934647	*Ca_kabuli_Chr03*	3701396	C/T	Gene_Ca_21344	INTRON	Gibberellin regulated protein
Kabuli_wild_3701398	1399934648	*Ca_kabuli_Chr03*	3701398	T/A	Gene_Ca_21344	INTRON	Gibberellin regulated protein
Kabuli_wild_3701401	1399934649	*Ca_kabuli_Chr03*	3701401	A/T	Gene_Ca_21344	INTRON	Gibberellin regulated protein
Kabuli_wild_3701405	1399934650	*Ca_kabuli_Chr03*	3701405	G/T	Gene_Ca_21344	INTRON	Gibberellin regulated protein
Kabuli_wild_3701415	1399934651	*Ca_kabuli_Chr03*	3701415	C/T	Gene_Ca_21344	INTRON	Gibberellin regulated protein
Kabuli_wild_3701422	1399934652	*Ca_kabuli_Chr03*	3701422	C/T	Gene_Ca_21344	INTRON	Gibberellin regulated protein
Kabuli_wild_3701426	1399934653	*Ca_kabuli_Chr03*	3701426	T/C	Gene_Ca_21344	INTRON	Gibberellin regulated protein
Kabuli_wild_3701435	1399934654	*Ca_kabuli_Chr03*	3701435	A/T	Gene_Ca_21344	INTRON	Gibberellin regulated protein
Kabuli_wild_3701437	1399934655	*Ca_kabuli_Chr03*	3701437	A/T	Gene_Ca_21344	INTRON	Gibberellin regulated protein
Kabuli_wild_3701440	1399934656	*Ca_kabuli_Chr03*	3701440	G/T	Gene_Ca_21344	INTRON	Gibberellin regulated protein
Kabuli_wild_3701441	1399934657	*Ca_kabuli_Chr03*	3701441	T/C	Gene_Ca_21344	INTRON	Gibberellin regulated protein
Kabuli_wild_3701442	1399934658	*Ca_kabuli_Chr03*	3701442	C/T	Gene_Ca_21344	INTRON	Gibberellin regulated protein
Kabuli_wild_3723580	1399934659	*Ca_kabuli_Chr03*	3723580	C/A	NA	INTERGENIC	NA
Kabuli_wild_3825806	1399934660	*Ca_kabuli_Chr03*	3825806	G/A	Gene_Ca_21335	DOWNSTREAM	Transposase,Ptta/En/Spm,plant
Kabuli_wild_3825807	1399934661	*Ca_kabuli_Chr03*	3825807	C/T	Gene_Ca_21335	DOWNSTREAM	Transposase,Ptta/En/Spm,plant
Kabuli_wild_3825824	1399934662	*Ca_kabuli_Chr03*	3825824	T/C	Gene_Ca_21335	DOWNSTREAM	Transposase,Ptta/En/Spm,plant
Kabuli_wild_3825852	1399934663	*Ca_kabuli_Chr03*	3825852	G/T	Gene_Ca_21335	DOWNSTREAM	Transposase,Ptta/En/Spm,plant

SNP IDs	NCBI dbSNP accession IDs (NCBI_SS#)	Chromosomes	Physical positions (bp)	SNPs	Gene accession IDs	Sequence components of genome	Putative functions
Kabuli_wild_3825858	1399934664	*Ca_kabuli_Chr03*	3825858	G/A	Gene_Ca_21335	DOWNSTREAM	Transposase,Ptta/En/Spm,plant
Kabuli_wild_3825903	1399934665	*Ca_kabuli_Chr03*	3825903	T/A	Gene_Ca_21335	DOWNSTREAM	Transposase,Ptta/En/Spm,plant
Kabuli_wild_3831950	1399934666	*Ca_kabuli_Chr03*	3831950	C/T	NA	INTERGENIC	NA
Kabuli_wild_3858878	1399934667	*Ca_kabuli_Chr03*	3858878	T/G	Gene_Ca_21333	INTRON	Major intrinsic protein
Kabuli_wild_3915645	1399934668	*Ca_kabuli_Chr03*	3915645	T/C	Gene_Ca_21331	NON_SYNONYMOUS_CODING	Peptidase C48, SUMO/Sentrin/Ubl1
Kabuli_wild_3915794	1399934669	*Ca_kabuli_Chr03*	3915794	C/T	Gene_Ca_21331	NON_SYNONYMOUS_CODING	Peptidase C48, SUMO/Sentrin/Ubl1
Kabuli_wild_4035581	1399934670	*Ca_kabuli_Chr03*	4035581	A/C	NA	INTERGENIC	NA
Kabuli_wild_4035583	1399934671	*Ca_kabuli_Chr03*	4035583	G/A	NA	INTERGENIC	NA
Kabuli_wild_4145743	1399934672	*Ca_kabuli_Chr03*	4145743	G/A	Gene_Ca_22385	INTRON	Domain of unknown function DUF298
Kabuli_wild_4178154	1399934673	*Ca_kabuli_Chr03*	4178154	C/T	NA	INTERGENIC	NA
Kabuli_wild_4266680	1399934674	*Ca_kabuli_Chr03*	4266680	T/G	NA	INTERGENIC	NA
Kabuli_wild_4266696	1399934675	*Ca_kabuli_Chr03*	4266696	G/A	NA	INTERGENIC	NA
Kabuli_wild_4266705	1399934676	*Ca_kabuli_Chr03*	4266705	G/A	NA	INTERGENIC	NA
Kabuli_wild_4266708	1399934677	*Ca_kabuli_Chr03*	4266708	C/T	NA	INTERGENIC	NA
Kabuli_wild_4266716	1399934678	*Ca_kabuli_Chr03*	4266716	A/G	NA	INTERGENIC	NA
Kabuli_wild_4266721	1399934679	*Ca_kabuli_Chr03*	4266721	G/A	NA	INTERGENIC	NA
Kabuli_wild_4266733	1399934680	*Ca_kabuli_Chr03*	4266733	C/G	NA	INTERGENIC	NA
Kabuli_wild_4629747	1399934681	*Ca_kabuli_Chr03*	4629747	A/C	Gene_Ca_22084	INTRON	NA
Kabuli_wild_4639410	1399934682	*Ca_kabuli_Chr03*	4639410	T/C	NA	INTERGENIC	NA
Kabuli_wild_4878361	1399934683	*Ca_kabuli_Chr03*	4878361	A/G	Gene_Ca_22093	INTRON	Biotin/lipoylattachment
Kabuli_wild_5224781	1399934684	*Ca_kabuli_Chr03*	5224781	A/G	Gene_Ca_23868	NON_SYNONYMOUS_CODING	NA
Kabuli_wild_5224976	1399934685	*Ca_kabuli_Chr03*	5224976	A/G	Gene_Ca_23868	NON_SYNONYMOUS_CODING	NA
Kabuli_wild_5225108	1399934686	*Ca_kabuli_Chr03*	5225108	T/G	Gene_Ca_23868	NON_SYNONYMOUS_CODING	NA
Kabuli_wild_5410879	1399934687	*Ca_kabuli_Chr03*	5410879	C/A	NA	INTERGENIC	NA
Kabuli_wild_5410880	1399934688	*Ca_kabuli_Chr03*	5410880	A/G	NA	INTERGENIC	NA
Kabuli_wild_5415765	1399934689	*Ca_kabuli_Chr03*	5415765	T/G	Gene_Ca_20660	DOWNSTREAM	Alpha-1,4-glucan-proteinsynthase,UDP-forming
Kabuli_wild_5436267	1399934690	*Ca_kabuli_Chr03*	5436267	A/G	NA	INTERGENIC	NA
Kabuli_wild_5436272	1399934691	*Ca_kabuli_Chr03*	5436272	T/C	NA	INTERGENIC	NA
Kabuli_wild_5436277	1399934692	*Ca_kabuli_Chr03*	5436277	C/T	NA	INTERGENIC	NA
Kabuli_wild_5436288	1399934693	*Ca_kabuli_Chr03*	5436288	G/T	NA	INTERGENIC	NA
Kabuli_wild_5436295	1399934694	*Ca_kabuli_Chr03*	5436295	G/A	NA	INTERGENIC	NA
Kabuli_wild_5436296	1399934695	*Ca_kabuli_Chr03*	5436296	C/T	NA	INTERGENIC	NA
Kabuli_wild_5436299	1399934696	*Ca_kabuli_Chr03*	5436299	A/C	NA	INTERGENIC	NA
Kabuli_wild_5436317	1399934697	*Ca_kabuli_Chr03*	5436317	C/T	NA	INTERGENIC	NA
Kabuli_wild_5436331	1399934698	*Ca_kabuli_Chr03*	5436331	C/A	NA	INTERGENIC	NA
Kabuli_wild_5514176	1399934699	*Ca_kabuli_Chr03*	5514176	A/G	NA	INTERGENIC	NA
Kabuli_wild_5514206	1399934700	*Ca_kabuli_Chr03*	5514206	G/T	NA	INTERGENIC	NA
Kabuli_wild_5514287	1399934701	*Ca_kabuli_Chr03*	5514287	C/A	NA	INTERGENIC	NA
Kabuli_wild_6169510	1399934702	*Ca_kabuli_Chr03*	6169510	A/C	Gene_Ca_24827	DOWNSTREAM	Leucine-rich repeat,cysteine-containingsubtype
Kabuli_wild_6467014	1399934703	*Ca_kabuli_Chr03*	6467014	G/A	NA	INTERGENIC	NA
Kabuli_wild_6683587	1399934704	*Ca_kabuli_Chr03*	6683587	T/A	Gene_Ca_25772	INTRON	NA
Kabuli_wild_6683646	1399934705	*Ca_kabuli_Chr03*	6683646	A/G	Gene_Ca_25772	INTRON	NA
Kabuli_wild_6807698	1399934706	*Ca_kabuli_Chr03*	6807698	G/A	NA	INTERGENIC	NA
Kabuli_wild_6828181	1399934707	*Ca_kabuli_Chr03*	6828181	A/T	NA	INTERGENIC	NA

SNP IDs	NCBI dbSNP accession IDs (NCBI_SS#)	Chromosomes	Physical positions (bp)	SNPs	Gene accession IDs	Sequence components of genome	Putative functions
Kabuli_wild_6840644	1399934708	*Ca_kabuli_Chr03*	6840644	C/T	Gene_Ca_16550	SYNONYMOUS_CODING	WD40 repeat
Kabuli_wild_6948882	1399934709	*Ca_kabuli_Chr03*	6948882	G/C	NA	INTERGENIC	NA
Kabuli_wild_7013496	1399934710	*Ca_kabuli_Chr03*	7013496	A/C	Gene_Ca_16540	INTRON	Zinc finger,ZZ-type
Kabuli_wild_7015213	1399934711	*Ca_kabuli_Chr03*	7015213	T/G	Gene_Ca_16540	NON_SYNONYMOUS_CODING	Zinc finger,ZZ-type
Kabuli_wild_7026579	1399934712	*Ca_kabuli_Chr03*	7026579	T/G	Gene_Ca_16540	SYNONYMOUS_CODING	Zinc finger,ZZ-type
Kabuli_wild_7203321	1399934713	*Ca_kabuli_Chr03*	7203321	C/A	NA	INTERGENIC	NA
Kabuli_wild_7203327	1399934714	*Ca_kabuli_Chr03*	7203327	A/G	NA	INTERGENIC	NA
Kabuli_wild_7204053	1399934715	*Ca_kabuli_Chr03*	7204053	G/A	NA	INTERGENIC	NA
Kabuli_wild_7204071	1399934716	*Ca_kabuli_Chr03*	7204071	T/C	NA	INTERGENIC	NA
Kabuli_wild_7204087	1399934717	*Ca_kabuli_Chr03*	7204087	C/T	NA	INTERGENIC	NA
Kabuli_wild_7204088	1399934718	*Ca_kabuli_Chr03*	7204088	G/C	NA	INTERGENIC	NA
Kabuli_wild_7204098	1399934719	*Ca_kabuli_Chr03*	7204098	G/A	NA	INTERGENIC	NA
Kabuli_wild_7204099	1399934720	*Ca_kabuli_Chr03*	7204099	C/T	NA	INTERGENIC	NA
Kabuli_wild_7204111	1399934721	*Ca_kabuli_Chr03*	7204111	G/A	NA	INTERGENIC	NA
Kabuli_wild_7204112	1399934722	*Ca_kabuli_Chr03*	7204112	G/A	NA	INTERGENIC	NA
Kabuli_wild_7204133	1399934723	*Ca_kabuli_Chr03*	7204133	G/T	NA	INTERGENIC	NA
Kabuli_wild_7226632	1399934724	*Ca_kabuli_Chr03*	7226632	G/T	Gene_Ca_16534	NON_SYNONYMOUS_CODING	Peptidase C48, SUMO/Sentrin/Ubl1
Kabuli_wild_7226661	1399934725	*Ca_kabuli_Chr03*	7226661	G/T	Gene_Ca_16534	NON_SYNONYMOUS_CODING	Peptidase C48, SUMO/Sentrin/Ubl1
Kabuli_wild_7226689	1399934726	*Ca_kabuli_Chr03*	7226689	T/C	Gene_Ca_16534	NON_SYNONYMOUS_CODING	Peptidase C48, SUMO/Sentrin/Ubl1
Kabuli_wild_7420705	1399934727	*Ca_kabuli_Chr03*	7420705	G/A	NA	INTERGENIC	NA
Kabuli_wild_7461812	1399934728	*Ca_kabuli_Chr03*	7461812	T/A	Gene_Ca_16524	SYNONYMOUS_CODING	Smrprotein/MutS2C-terminal
Kabuli_wild_7482871	1399934729	*Ca_kabuli_Chr03*	7482871	C/T	NA	INTERGENIC	NA
Kabuli_wild_7606901	1399934730	*Ca_kabuli_Chr03*	7606901	T/C	NA	INTERGENIC	NA
Kabuli_wild_7608075	1399934731	*Ca_kabuli_Chr03*	7608075	T/C	NA	INTERGENIC	NA
Kabuli_wild_7660311	1399934732	*Ca_kabuli_Chr03*	7660311	A/G	NA	INTERGENIC	NA
Kabuli_wild_7660504	1399934733	*Ca_kabuli_Chr03*	7660504	A/C	NA	INTERGENIC	NA
Kabuli_wild_7665072	1399934734	*Ca_kabuli_Chr03*	7665072	G/T	NA	INTERGENIC	NA
Kabuli_wild_7665073	1399934735	*Ca_kabuli_Chr03*	7665073	C/T	NA	INTERGENIC	NA
Kabuli_wild_7665075	1399934736	*Ca_kabuli_Chr03*	7665075	G/A	NA	INTERGENIC	NA
Kabuli_wild_7665090	1399934737	*Ca_kabuli_Chr03*	7665090	C/T	NA	INTERGENIC	NA
Kabuli_wild_7665098	1399934738	*Ca_kabuli_Chr03*	7665098	C/T	NA	INTERGENIC	NA
Kabuli_wild_7665112	1399934739	*Ca_kabuli_Chr03*	7665112	C/T	NA	INTERGENIC	NA
Kabuli_wild_7665114	1399934740	*Ca_kabuli_Chr03*	7665114	G/A	NA	INTERGENIC	NA
Kabuli_wild_7665118	1399934741	*Ca_kabuli_Chr03*	7665118	G/C	NA	INTERGENIC	NA
Kabuli_wild_7665123	1399934742	*Ca_kabuli_Chr03*	7665123	G/A	NA	INTERGENIC	NA
Kabuli_wild_7665142	1399934743	*Ca_kabuli_Chr03*	7665142	G/A	NA	INTERGENIC	NA
Kabuli_wild_7665149	1399934744	*Ca_kabuli_Chr03*	7665149	T/G	NA	INTERGENIC	NA
Kabuli_wild_7665156	1399934745	*Ca_kabuli_Chr03*	7665156	G/A	NA	INTERGENIC	NA
Kabuli_wild_7665157	1399934746	*Ca_kabuli_Chr03*	7665157	C/T	NA	INTERGENIC	NA
Kabuli_wild_7665172	1399934747	*Ca_kabuli_Chr03*	7665172	C/A	NA	INTERGENIC	NA
Kabuli_wild_7665173	1399934748	*Ca_kabuli_Chr03*	7665173	C/T	NA	INTERGENIC	NA
Kabuli_wild_7665184	1399934749	*Ca_kabuli_Chr03*	7665184	G/A	NA	INTERGENIC	NA
Kabuli_wild_8279444	1399934750	*Ca_kabuli_Chr03*	8279444	C/A	NA	INTERGENIC	NA
Kabuli_wild_8279461	1399934751	*Ca_kabuli_Chr03*	8279461	C/T	NA	INTERGENIC	NA

SNP IDs	NCBI dbSNP accession IDs (NCBI_SS#)	Chromosomes	Physical positions (bp)	SNPs	Gene accession IDs	Sequence components of genome	Putative functions
Kabuli_wild_8279519	1399934752	*Ca_kabuli_Chr03*	8279519	C/T	NA	INTERGENIC	NA
Kabuli_wild_8280956	1399934753	*Ca_kabuli_Chr03*	8280956	G/A	Gene_Ca_24378	UPSTREAM	Domain of unknown function DUF231,plant
Kabuli_wild_8503308	1399934754	*Ca_kabuli_Chr03*	8503308	T/C	NA	INTERGENIC	NA
Kabuli_wild_8536213	1399934755	*Ca_kabuli_Chr03*	8536213	T/C	NA	INTERGENIC	NA
Kabuli_wild_8536275	1399934756	*Ca_kabuli_Chr03*	8536275	C/T	NA	INTERGENIC	NA
Kabuli_wild_8536295	1399934757	*Ca_kabuli_Chr03*	8536295	A/T	NA	INTERGENIC	NA
Kabuli_wild_8536301	1399934758	*Ca_kabuli_Chr03*	8536301	G/T	NA	INTERGENIC	NA
Kabuli_wild_8536425	1399934759	*Ca_kabuli_Chr03*	8536425	C/A	NA	INTERGENIC	NA
Kabuli_wild_8536498	1399934760	*Ca_kabuli_Chr03*	8536498	T/A	NA	INTERGENIC	NA
Kabuli_wild_8638915	1399934761	*Ca_kabuli_Chr03*	8638915	A/C	NA	INTERGENIC	NA
Kabuli_wild_8638935	1399934762	*Ca_kabuli_Chr03*	8638935	T/C	NA	INTERGENIC	NA
Kabuli_wild_8668628	1399934763	*Ca_kabuli_Chr03*	8668628	A/C	NA	INTERGENIC	NA
Kabuli_wild_8668858	1399934764	*Ca_kabuli_Chr03*	8668858	A/T	Gene_Ca_23713	NON_SYNONYMOUS_CODING	Protein kinase,catalytic domain
Kabuli_wild_8669004	1399934765	*Ca_kabuli_Chr03*	8669004	A/T	Gene_Ca_23713	NON_SYNONYMOUS_CODING	Protein kinase,catalytic domain
Kabuli_wild_8669132	1399934766	*Ca_kabuli_Chr03*	8669132	T/A	Gene_Ca_23713	SYNONYMOUS_CODING	Protein kinase,catalytic domain
Kabuli_wild_8674483	1399934767	*Ca_kabuli_Chr03*	8674483	A/C	NA	INTERGENIC	NA
Kabuli_wild_8697186	1399934768	*Ca_kabuli_Chr03*	8697186	A/G	NA	INTERGENIC	NA
Kabuli_wild_8706645	1399934769	*Ca_kabuli_Chr03*	8706645	G/C	Gene_Ca_23716	INTRON	Aspartate/glutamate/uridylatekinase
Kabuli_wild_8706658	1399934770	*Ca_kabuli_Chr03*	8706658	A/G	Gene_Ca_23716	INTRON	Aspartate/glutamate/uridylatekinase
Kabuli_wild_8933037	1399934771	*Ca_kabuli_Chr03*	8933037	T/C	Gene_Ca_25280	INTRON	ABC transporter-like
Kabuli_wild_8933084	1399934772	*Ca_kabuli_Chr03*	8933084	G/A	Gene_Ca_25280	INTRON	ABC transporter-like
Kabuli_wild_8933086	1399934773	*Ca_kabuli_Chr03*	8933086	C/A	Gene_Ca_25280	INTRON	ABC transporter-like
Kabuli_wild_8933183	1399934774	*Ca_kabuli_Chr03*	8933183	A/G	Gene_Ca_25280	INTRON	ABC transporter-like
Kabuli_wild_8933221	1399934775	*Ca_kabuli_Chr03*	8933221	T/A	Gene_Ca_25280	INTRON	ABC transporter-like
Kabuli_wild_8933255	1399934776	*Ca_kabuli_Chr03*	8933255	A/T	Gene_Ca_25280	INTRON	ABC transporter-like
Kabuli_wild_8933266	1399934777	*Ca_kabuli_Chr03*	8933266	G/T	Gene_Ca_25280	INTRON	ABC transporter-like
Kabuli_wild_8933355	1399934778	*Ca_kabuli_Chr03*	8933355	A/T	Gene_Ca_25280	INTRON	ABC transporter-like
Kabuli_wild_8933362	1399934779	*Ca_kabuli_Chr03*	8933362	C/T	Gene_Ca_25280	INTRON	ABC transporter-like
Kabuli_wild_8933370	1399934780	*Ca_kabuli_Chr03*	8933370	A/G	Gene_Ca_25280	INTRON	ABC transporter-like
Kabuli_wild_8933409	1399934781	*Ca_kabuli_Chr03*	8933409	A/C	Gene_Ca_25280	INTRON	ABC transporter-like
Kabuli_wild_9146761	1399934782	*Ca_kabuli_Chr03*	9146761	T/C	NA	INTERGENIC	NA
Kabuli_wild_9146812	1399934783	*Ca_kabuli_Chr03*	9146812	G/A	NA	INTERGENIC	NA
Kabuli_wild_9223875	1399934784	*Ca_kabuli_Chr03*	9223875	G/A	NA	INTERGENIC	NA
Kabuli_wild_9296469	1399934785	*Ca_kabuli_Chr03*	9296469	T/G	Gene_Ca_20960	SYNONYMOUS_CODING	Arf GTPase activating protein
Kabuli_wild_9384227	1399934786	*Ca_kabuli_Chr03*	9384227	A/T	Gene_Ca_20966	DOWNSTREAM	NA
Kabuli_wild_9411779	1399934787	*Ca_kabuli_Chr03*	9411779	C/T	Gene_Ca_20969	UPSTREAM	Pectinesterase,catalytic
Kabuli_wild_9411780	1399934788	*Ca_kabuli_Chr03*	9411780	G/A	Gene_Ca_20969	UPSTREAM	Pectinesterase,catalytic
Kabuli_wild_9411810	1399934789	*Ca_kabuli_Chr03*	9411810	A/G	Gene_Ca_20969	UPSTREAM	Pectinesterase,catalytic
Kabuli_wild_9411842	1399934790	*Ca_kabuli_Chr03*	9411842	T/C	Gene_Ca_20969	UPSTREAM	Pectinesterase,catalytic
Kabuli_wild_9411849	1399934791	*Ca_kabuli_Chr03*	9411849	T/G	Gene_Ca_20969	UPSTREAM	Pectinesterase,catalytic
Kabuli_wild_9577170	1399934792	*Ca_kabuli_Chr03*	9577170	G/A	Gene_Ca_20977	NON_SYNONYMOUS_CODING	NA
Kabuli_wild_9577402	1399934793	*Ca_kabuli_Chr03*	9577402	G/A	Gene_Ca_20977	NON_SYNONYMOUS_CODING	NA
Kabuli_wild_9579805	1399934794	*Ca_kabuli_Chr03*	9579805	A/T	Gene_Ca_20977	DOWNSTREAM	NA
Kabuli_wild_9579808	1399934795	*Ca_kabuli_Chr03*	9579808	C/T	Gene_Ca_20977	DOWNSTREAM	NA

SNP IDs	NCBI dbSNP accession IDs (NCBI_SS#)	Chromosomes	Physical positions (bp)	SNPs	Gene accession IDs	Sequence components of genome	Putative functions
Kabuli_wild_9579811	1399934796	*Ca_kabuli_Chr03*	9579811	G/C	Gene_Ca_20977	DOWNSTREAM	NA
Kabuli_wild_9579817	1399934797	*Ca_kabuli_Chr03*	9579817	G/A	Gene_Ca_20977	DOWNSTREAM	NA
Kabuli_wild_9579818	1399934798	*Ca_kabuli_Chr03*	9579818	G/T	Gene_Ca_20977	DOWNSTREAM	NA
Kabuli_wild_9579824	1399934799	*Ca_kabuli_Chr03*	9579824	A/G	Gene_Ca_20977	DOWNSTREAM	NA
Kabuli_wild_9579830	1399934800	*Ca_kabuli_Chr03*	9579830	T/A	Gene_Ca_20977	DOWNSTREAM	NA
Kabuli_wild_9579831	1399934801	*Ca_kabuli_Chr03*	9579831	C/G	Gene_Ca_20977	DOWNSTREAM	NA
Kabuli_wild_9894316	1399934802	*Ca_kabuli_Chr03*	9894316	C/T	Gene_Ca_18799	DOWNSTREAM	Reversetranscriptase
Kabuli_wild_10142152	1399934803	*Ca_kabuli_Chr03*	10142152	C/A	Gene_Ca_18811	INTRON	WD40 repeat
Kabuli_wild_10159836	1399934804	*Ca_kabuli_Chr03*	10159836	A/C	NA	INTERGENIC	NA
Kabuli_wild_10159859	1399934805	*Ca_kabuli_Chr03*	10159859	G/A	NA	INTERGENIC	NA
Kabuli_wild_10159867	1399934806	*Ca_kabuli_Chr03*	10159867	C/T	NA	INTERGENIC	NA
Kabuli_wild_10159871	1399934807	*Ca_kabuli_Chr03*	10159871	C/T	NA	INTERGENIC	NA
Kabuli_wild_10159875	1399934808	*Ca_kabuli_Chr03*	10159875	C/T	NA	INTERGENIC	NA
Kabuli_wild_10159941	1399934809	*Ca_kabuli_Chr03*	10159941	C/A	NA	INTERGENIC	NA
Kabuli_wild_10159943	1399934810	*Ca_kabuli_Chr03*	10159943	G/A	NA	INTERGENIC	NA
Kabuli_wild_10159944	1399934811	*Ca_kabuli_Chr03*	10159944	T/C	NA	INTERGENIC	NA
Kabuli_wild_10186857	1399934812	*Ca_kabuli_Chr03*	10186857	A/G	Gene_Ca_18812	INTRON	NA
Kabuli_wild_10186861	1399934813	*Ca_kabuli_Chr03*	10186861	G/T	Gene_Ca_18812	INTRON	NA
Kabuli_wild_10186880	1399934814	*Ca_kabuli_Chr03*	10186880	A/G	Gene_Ca_18812	INTRON	NA
Kabuli_wild_10186889	1399934815	*Ca_kabuli_Chr03*	10186889	G/A	Gene_Ca_18812	INTRON	NA
Kabuli_wild_10186891	1399934816	*Ca_kabuli_Chr03*	10186891	C/G	Gene_Ca_18812	INTRON	NA
Kabuli_wild_10186893	1399934817	*Ca_kabuli_Chr03*	10186893	G/C	Gene_Ca_18812	INTRON	NA
Kabuli_wild_10201361	1399934818	*Ca_kabuli_Chr03*	10201361	G/T	Gene_Ca_18813	SYNONYMOUS_CODING	VRR-NUC
Kabuli_wild_10201393	1399934819	*Ca_kabuli_Chr03*	10201393	T/C	Gene_Ca_18813	NON_SYNONYMOUS_CODING	VRR-NUC
Kabuli_wild_10722771	1399934820	*Ca_kabuli_Chr03*	10722771	G/C	Gene_Ca_18831	INTRON	NA
Kabuli_wild_10722779	1399934821	*Ca_kabuli_Chr03*	10722779	G/A	Gene_Ca_18831	INTRON	NA
Kabuli_wild_10722780	1399934822	*Ca_kabuli_Chr03*	10722780	G/T	Gene_Ca_18831	INTRON	NA
Kabuli_wild_10722818	1399934823	*Ca_kabuli_Chr03*	10722818	T/C	Gene_Ca_18831	INTRON	NA
Kabuli_wild_10722829	1399934824	*Ca_kabuli_Chr03*	10722829	C/G	Gene_Ca_18831	INTRON	NA
Kabuli_wild_10722859	1399934825	*Ca_kabuli_Chr03*	10722859	G/A	Gene_Ca_18831	INTRON	NA
Kabuli_wild_10722901	1399934826	*Ca_kabuli_Chr03*	10722901	T/C	Gene_Ca_18831	INTRON	NA
Kabuli_wild_10722904	1399934827	*Ca_kabuli_Chr03*	10722904	G/T	Gene_Ca_18831	INTRON	NA
Kabuli_wild_11143751	1399934828	*Ca_kabuli_Chr03*	11143751	G/A	Gene_Ca_19368	NON_SYNONYMOUS_CODING	NA
Kabuli_wild_11143754	1399934829	*Ca_kabuli_Chr03*	11143754	G/A	Gene_Ca_19368	NON_SYNONYMOUS_CODING	NA
Kabuli_wild_11143818	1399934830	*Ca_kabuli_Chr03*	11143818	G/A	Gene_Ca_19368	SYNONYMOUS_CODING	NA
Kabuli_wild_11143833	1399934831	*Ca_kabuli_Chr03*	11143833	C/T	Gene_Ca_19368	NON_SYNONYMOUS_CODING	NA
Kabuli_wild_11273777	1399934832	*Ca_kabuli_Chr03*	11273777	A/C	NA	INTERGENIC	NA
Kabuli_wild_11378425	1399934833	*Ca_kabuli_Chr03*	11378425	G/T	Gene_Ca_19376	NON_SYNONYMOUS_CODING	ABC transporter,trans membrane domain
Kabuli_wild_11470115	1399934834	*Ca_kabuli_Chr03*	11470115	C/T	NA	INTERGENIC	NA
Kabuli_wild_11470185	1399934835	*Ca_kabuli_Chr03*	11470185	A/G	NA	INTERGENIC	NA
Kabuli_wild_11470186	1399934836	*Ca_kabuli_Chr03*	11470186	C/T	NA	INTERGENIC	NA
Kabuli_wild_11504763	1399934837	*Ca_kabuli_Chr03*	11504763	C/T	NA	INTERGENIC	NA
Kabuli_wild_11504764	1399934838	*Ca_kabuli_Chr03*	11504764	T/C	NA	INTERGENIC	NA
Kabuli_wild_11590793	1399934839	*Ca_kabuli_Chr03*	11590793	C/T	NA	INTERGENIC	NA

SNP IDs	NCBI dbSNP accession IDs (NCBI_SS#)	Chromosomes	Physical positions (bp)	SNPs	Gene accession IDs	Sequence components of genome	Putative functions
Kabuli_wild_11590814	1399934840	*Ca_kabuli_Chr03*	11590814	T/C	NA	INTERGENIC	NA
Kabuli_wild_11590832	1399934841	*Ca_kabuli_Chr03*	11590832	G/A	NA	INTERGENIC	NA
Kabuli_wild_11590860	1399934842	*Ca_kabuli_Chr03*	11590860	G/A	NA	INTERGENIC	NA
Kabuli_wild_11590874	1399934843	*Ca_kabuli_Chr03*	11590874	A/G	NA	INTERGENIC	NA
Kabuli_wild_11610455	1399934844	*Ca_kabuli_Chr03*	11610455	G/C	NA	INTERGENIC	NA
Kabuli_wild_11610459	1399934845	*Ca_kabuli_Chr03*	11610459	C/T	NA	INTERGENIC	NA
Kabuli_wild_11610460	1399934846	*Ca_kabuli_Chr03*	11610460	A/G	NA	INTERGENIC	NA
Kabuli_wild_11610461	1399934847	*Ca_kabuli_Chr03*	11610461	C/T	NA	INTERGENIC	NA
Kabuli_wild_11610481	1399934848	*Ca_kabuli_Chr03*	11610481	C/T	NA	INTERGENIC	NA
Kabuli_wild_11610482	1399934849	*Ca_kabuli_Chr03*	11610482	A/G	NA	INTERGENIC	NA
Kabuli_wild_11610500	1399934850	*Ca_kabuli_Chr03*	11610500	A/G	NA	INTERGENIC	NA
Kabuli_wild_11653927	1399934851	*Ca_kabuli_Chr03*	11653927	G/C	NA	INTERGENIC	NA
Kabuli_wild_11684683	1399934852	*Ca_kabuli_Chr03*	11684683	A/C	NA	INTERGENIC	NA
Kabuli_wild_11811685	1399934853	*Ca_kabuli_Chr03*	11811685	A/G	Gene_Ca_19392	INTRON	Putativezinc-fingerdomain
Kabuli_wild_12051826	1399934854	*Ca_kabuli_Chr03*	12051826	A/C	NA	INTERGENIC	NA
Kabuli_wild_12056637	1399934855	*Ca_kabuli_Chr03*	12056637	C/T	Gene_Ca_22246	SYNONYMOUS_CODING	NA
Kabuli_wild_12058576	1399934856	*Ca_kabuli_Chr03*	12058576	T/C	Gene_Ca_22246	INTRON	NA
Kabuli_wild_12067458	1399934857	*Ca_kabuli_Chr03*	12067458	A/C	Gene_Ca_22246	INTRON	NA
Kabuli_wild_12069026	1399934858	*Ca_kabuli_Chr03*	12069026	A/C	Gene_Ca_22246	SYNONYMOUS_CODING	NA
Kabuli_wild_12069092	1399934859	*Ca_kabuli_Chr03*	12069092	T/G	Gene_Ca_22246	SYNONYMOUS_CODING	NA
Kabuli_wild_12095571	1399934860	*Ca_kabuli_Chr03*	12095571	T/G	Gene_Ca_22245	SYNONYMOUS_CODING	Crotonase,core
Kabuli_wild_12095632	1399934861	*Ca_kabuli_Chr03*	12095632	C/T	Gene_Ca_22245	NON_SYNONYMOUS_CODING	Crotonase,core
Kabuli_wild_12124099	1399934862	*Ca_kabuli_Chr03*	12124099	G/A	NA	INTERGENIC	NA
Kabuli_wild_12129021	1399934863	*Ca_kabuli_Chr03*	12129021	A/G	NA	INTERGENIC	NA
Kabuli_wild_12294240	1399934864	*Ca_kabuli_Chr03*	12294240	C/T	NA	INTERGENIC	NA
Kabuli_wild_12394742	1399934865	*Ca_kabuli_Chr03*	12394742	G/A	NA	INTERGENIC	NA
Kabuli_wild_12599496	1399934866	*Ca_kabuli_Chr03*	12599496	A/C	Gene_Ca_21021	SYNONYMOUS_CODING	Zinc finger,Dof-type
Kabuli_wild_12622503	1399934867	*Ca_kabuli_Chr03*	12622503	G/A	NA	INTERGENIC	NA
Kabuli_wild_12622534	1399934868	*Ca_kabuli_Chr03*	12622534	T/C	NA	INTERGENIC	NA
Kabuli_wild_12622543	1399934869	*Ca_kabuli_Chr03*	12622543	G/A	NA	INTERGENIC	NA
Kabuli_wild_12622567	1399934870	*Ca_kabuli_Chr03*	12622567	A/C	NA	INTERGENIC	NA
Kabuli_wild_12622578	1399934871	*Ca_kabuli_Chr03*	12622578	G/A	NA	INTERGENIC	NA
Kabuli_wild_12627143	1399934872	*Ca_kabuli_Chr03*	12627143	T/G	Gene_Ca_21019	DOWNSTREAM	Integrase,catalyticcore
Kabuli_wild_12627144	1399934873	*Ca_kabuli_Chr03*	12627144	G/A	Gene_Ca_21019	DOWNSTREAM	Integrase,catalyticcore
Kabuli_wild_12627154	1399934874	*Ca_kabuli_Chr03*	12627154	C/T	Gene_Ca_21019	DOWNSTREAM	Integrase,catalyticcore
Kabuli_wild_12627166	1399934875	*Ca_kabuli_Chr03*	12627166	G/A	Gene_Ca_21019	DOWNSTREAM	Integrase,catalyticcore
Kabuli_wild_12627174	1399934876	*Ca_kabuli_Chr03*	12627174	G/A	Gene_Ca_21019	DOWNSTREAM	Integrase,catalyticcore
Kabuli_wild_12627233	1399934877	*Ca_kabuli_Chr03*	12627233	T/A	Gene_Ca_21019	DOWNSTREAM	Integrase,catalyticcore
Kabuli_wild_12709003	1399934878	*Ca_kabuli_Chr03*	12709003	G/A	Gene_Ca_21017	NON_SYNONYMOUS_CODING	NA
Kabuli_wild_12709005	1399934879	*Ca_kabuli_Chr03*	12709005	T/A	Gene_Ca_21017	NON_SYNONYMOUS_CODING	NA
Kabuli_wild_12709027	1399934880	*Ca_kabuli_Chr03*	12709027	G/A	Gene_Ca_21017	NON_SYNONYMOUS_CODING	NA
Kabuli_wild_12709036	1399934881	*Ca_kabuli_Chr03*	12709036	C/T	Gene_Ca_21017	NON_SYNONYMOUS_CODING	NA
Kabuli_wild_12709044	1399934882	*Ca_kabuli_Chr03*	12709044	G/A	Gene_Ca_21017	SYNONYMOUS_CODING	NA
Kabuli_wild_12709048	1399934883	*Ca_kabuli_Chr03*	12709048	G/A	Gene_Ca_21017	NON_SYNONYMOUS_CODING	NA

SNP IDs	NCBI dbSNP accession IDs (NCBI_SS#)	Chromosomes	Physical positions (bp)	SNPs	Gene accession IDs	Sequence components of genome	Putative functions
Kabuli_wild_12709050	1399934884	*Ca_kabuli_Chr03*	12709050	G/A	Gene_Ca_21017	SYNONYMOUS_CODING	NA
Kabuli_wild_12709080	1399934885	*Ca_kabuli_Chr03*	12709080	T/G	Gene_Ca_21017	SYNONYMOUS_CODING	NA
Kabuli_wild_12809286	1399934886	*Ca_kabuli_Chr03*	12809286	A/G	Gene_Ca_21015	INTRON	Kinesin,motordomain
Kabuli_wild_12809794	1399934887	*Ca_kabuli_Chr03*	12809794	T/C	Gene_Ca_21015	NON_SYNONYMOUS_CODING	Kinesin,motordomain
Kabuli_wild_12814558	1399934888	*Ca_kabuli_Chr03*	12814558	A/G	NA	INTERGENIC	NA
Kabuli_wild_12814571	1399934889	*Ca_kabuli_Chr03*	12814571	C/A	NA	INTERGENIC	NA
Kabuli_wild_12814597	1399934890	*Ca_kabuli_Chr03*	12814597	T/A	NA	INTERGENIC	NA
Kabuli_wild_12814599	1399934891	*Ca_kabuli_Chr03*	12814599	A/T	NA	INTERGENIC	NA
Kabuli_wild_12814600	1399934892	*Ca_kabuli_Chr03*	12814600	A/G	NA	INTERGENIC	NA
Kabuli_wild_12814603	1399934893	*Ca_kabuli_Chr03*	12814603	T/C	NA	INTERGENIC	NA
Kabuli_wild_12814631	1399934894	*Ca_kabuli_Chr03*	12814631	C/T	NA	INTERGENIC	NA
Kabuli_wild_12897831	1399934895	*Ca_kabuli_Chr03*	12897831	C/A	NA	INTERGENIC	NA
Kabuli_wild_12897839	1399934896	*Ca_kabuli_Chr03*	12897839	G/A	NA	INTERGENIC	NA
Kabuli_wild_12897841	1399934897	*Ca_kabuli_Chr03*	12897841	C/A	NA	INTERGENIC	NA
Kabuli_wild_12897869	1399934898	*Ca_kabuli_Chr03*	12897869	C/T	NA	INTERGENIC	NA
Kabuli_wild_12897881	1399934899	*Ca_kabuli_Chr03*	12897881	C/T	NA	INTERGENIC	NA
Kabuli_wild_12897882	1399934900	*Ca_kabuli_Chr03*	12897882	T/G	NA	INTERGENIC	NA
Kabuli_wild_12897883	1399934901	*Ca_kabuli_Chr03*	12897883	G/T	NA	INTERGENIC	NA
Kabuli_wild_12897884	1399934902	*Ca_kabuli_Chr03*	12897884	C/T	NA	INTERGENIC	NA
Kabuli_wild_12897886	1399934903	*Ca_kabuli_Chr03*	12897886	G/A	NA	INTERGENIC	NA
Kabuli_wild_12897887	1399934904	*Ca_kabuli_Chr03*	12897887	T/C	NA	INTERGENIC	NA
Kabuli_wild_12897898	1399934905	*Ca_kabuli_Chr03*	12897898	G/T	NA	INTERGENIC	NA
Kabuli_wild_12897906	1399934906	*Ca_kabuli_Chr03*	12897906	C/A	NA	INTERGENIC	NA
Kabuli_wild_12897926	1399934907	*Ca_kabuli_Chr03*	12897926	A/C	NA	INTERGENIC	NA
Kabuli_wild_13041991	1399934908	*Ca_kabuli_Chr03*	13041991	T/G	NA	INTERGENIC	NA
Kabuli_wild_13041993	1399934909	*Ca_kabuli_Chr03*	13041993	T/A	NA	INTERGENIC	NA
Kabuli_wild_13042006	1399934910	*Ca_kabuli_Chr03*	13042006	C/T	NA	INTERGENIC	NA
Kabuli_wild_13042042	1399934911	*Ca_kabuli_Chr03*	13042042	G/C	NA	INTERGENIC	NA
Kabuli_wild_13042056	1399934912	*Ca_kabuli_Chr03*	13042056	C/G	NA	INTERGENIC	NA
Kabuli_wild_13185490	1399934913	*Ca_kabuli_Chr03*	13185490	G/T	Gene_Ca_22687	NON_SYNONYMOUS_CODING	NA
Kabuli_wild_13185493	1399934914	*Ca_kabuli_Chr03*	13185493	G/A	Gene_Ca_22687	NON_SYNONYMOUS_CODING	NA
Kabuli_wild_13185499	1399934915	*Ca_kabuli_Chr03*	13185499	A/G	Gene_Ca_22687	NON_SYNONYMOUS_CODING	NA
Kabuli_wild_13185502	1399934916	*Ca_kabuli_Chr03*	13185502	G/A	Gene_Ca_22687	NON_SYNONYMOUS_CODING	NA
Kabuli_wild_13185508	1399934917	*Ca_kabuli_Chr03*	13185508	C/T	Gene_Ca_22687	NON_SYNONYMOUS_CODING	NA
Kabuli_wild_13185516	1399934918	*Ca_kabuli_Chr03*	13185516	C/T	Gene_Ca_22687	SYNONYMOUS_CODING	NA
Kabuli_wild_13185518	1399934919	*Ca_kabuli_Chr03*	13185518	G/T	Gene_Ca_22687	NON_SYNONYMOUS_CODING	NA
Kabuli_wild_13185530	1399934920	*Ca_kabuli_Chr03*	13185530	C/T	Gene_Ca_22687	NON_SYNONYMOUS_CODING	NA
Kabuli_wild_13185532	1399934921	*Ca_kabuli_Chr03*	13185532	G/T	Gene_Ca_22687	NON_SYNONYMOUS_CODING	NA
Kabuli_wild_13185541	1399934922	*Ca_kabuli_Chr03*	13185541	G/A	Gene_Ca_22687	NON_SYNONYMOUS_CODING	NA
Kabuli_wild_13185560	1399934923	*Ca_kabuli_Chr03*	13185560	G/A	Gene_Ca_22687	NON_SYNONYMOUS_CODING	NA
Kabuli_wild_13185567	1399934924	*Ca_kabuli_Chr03*	13185567	T/G	Gene_Ca_22687	SYNONYMOUS_CODING	NA
Kabuli_wild_13185574	1399934925	*Ca_kabuli_Chr03*	13185574	G/A	Gene_Ca_22687	NON_SYNONYMOUS_CODING	NA
Kabuli_wild_13185575	1399934926	*Ca_kabuli_Chr03*	13185575	C/T	Gene_Ca_22687	NON_SYNONYMOUS_CODING	NA
Kabuli_wild_13185591	1399934927	*Ca_kabuli_Chr03*	13185591	C/T	Gene_Ca_22687	SYNONYMOUS_CODING	NA

SNP IDs	NCBI dbSNP accession IDs (NCBI_SS#)	Chromosomes	Physical positions (bp)	SNPs	Gene accession IDs	Sequence components of genome	Putative functions
Kabuli_wild_13185602	1399934928	*Ca_kabuli_Chr03*	13185602	G/A	Gene_Ca_22687	NON_SYNONYMOUS_CODING	NA
Kabuli_wild_13202952	1399934929	*Ca_kabuli_Chr03*	13202952	G/A	Gene_Ca_22688	NON_SYNONYMOUS_CODING	NA
Kabuli_wild_13202959	1399934930	*Ca_kabuli_Chr03*	13202959	C/T	Gene_Ca_22688	NON_SYNONYMOUS_CODING	NA
Kabuli_wild_13203014	1399934931	*Ca_kabuli_Chr03*	13203014	C/T	Gene_Ca_22688	SYNONYMOUS_CODING	NA
Kabuli_wild_13203019	1399934932	*Ca_kabuli_Chr03*	13203019	G/A	Gene_Ca_22688	SYNONYMOUS_CODING	NA
Kabuli_wild_13203037	1399934933	*Ca_kabuli_Chr03*	13203037	C/T	Gene_Ca_22688	NON_SYNONYMOUS_CODING	NA
Kabuli_wild_13303783	1399934934	*Ca_kabuli_Chr03*	13303783	T/A	NA	INTERGENIC	NA
Kabuli_wild_13396906	1399934935	*Ca_kabuli_Chr03*	13396906	A/C	Gene_Ca_22695	INTRON	Glycosyltransferase,family2
Kabuli_wild_13396925	1399934936	*Ca_kabuli_Chr03*	13396925	A/T	Gene_Ca_22695	INTRON	Glycosyltransferase,family2
Kabuli_wild_13397207	1399934937	*Ca_kabuli_Chr03*	13397207	A/C	Gene_Ca_22695	INTRON	Glycosyltransferase,family2
Kabuli_wild_13420872	1399934938	*Ca_kabuli_Chr03*	13420872	T/C	Gene_Ca_22697	UPSTREAM	Kinesin,motordomain
Kabuli_wild_13617948	1399934939	*Ca_kabuli_Chr03*	13617948	A/C	Gene_Ca_25319	SYNONYMOUS_CODING	NA
Kabuli_wild_13654047	1399934940	*Ca_kabuli_Chr03*	13654047	T/C	Gene_Ca_25322	NON_SYNONYMOUS_CODING	Exo70 exocystcomplex subunit
Kabuli_wild_13746900	1399934941	*Ca_kabuli_Chr03*	13746900	C/T	Gene_Ca_25324	SYNONYMOUS_CODING	NA
Kabuli_wild_13923713	1399934942	*Ca_kabuli_Chr03*	13923713	G/T	NA	INTERGENIC	NA
Kabuli_wild_13923718	1399934943	*Ca_kabuli_Chr03*	13923718	G/A	NA	INTERGENIC	NA
Kabuli_wild_13923728	1399934944	*Ca_kabuli_Chr03*	13923728	T/C	NA	INTERGENIC	NA
Kabuli_wild_13923761	1399934945	*Ca_kabuli_Chr03*	13923761	T/C	NA	INTERGENIC	NA
Kabuli_wild_14200381	1399934946	*Ca_kabuli_Chr03*	14200381	A/C	NA	INTERGENIC	NA
Kabuli_wild_14470345	1399934947	*Ca_kabuli_Chr03*	14470345	T/A	NA	INTERGENIC	NA
Kabuli_wild_14497290	1399934948	*Ca_kabuli_Chr03*	14497290	C/G	NA	INTERGENIC	NA
Kabuli_wild_14497374	1399934949	*Ca_kabuli_Chr03*	14497374	C/A	NA	INTERGENIC	NA
Kabuli_wild_14653193	1399934950	*Ca_kabuli_Chr03*	14653193	T/G	Gene_Ca_22505	INTRON	Zinc finger, RING-type
Kabuli_wild_14653194	1399934951	*Ca_kabuli_Chr03*	14653194	C/T	Gene_Ca_22505	INTRON	Zinc finger, RING-type
Kabuli_wild_14653195	1399934952	*Ca_kabuli_Chr03*	14653195	G/T	Gene_Ca_22505	INTRON	Zinc finger, RING-type
Kabuli_wild_14653270	1399934953	*Ca_kabuli_Chr03*	14653270	C/A	Gene_Ca_22505	INTRON	Zinc finger, RING-type
Kabuli_wild_14653271	1399934954	*Ca_kabuli_Chr03*	14653271	G/A	Gene_Ca_22505	INTRON	Zinc finger, RING-type
Kabuli_wild_14658175	1399934955	*Ca_kabuli_Chr03*	14658175	A/C	Gene_Ca_22505	INTRON	Zinc finger, RING-type
Kabuli_wild_14812960	1399934956	*Ca_kabuli_Chr03*	14812960	A/C	Gene_Ca_22500	NON_SYNONYMOUS_CODING	UDP-glucuronosyl/UDP-glucosyltransferase
Kabuli_wild_15042402	1399934957	*Ca_kabuli_Chr03*	15042402	C/T	NA	INTERGENIC	NA
Kabuli_wild_15145910	1399934958	*Ca_kabuli_Chr03*	15145910	A/C	Gene_Ca_17017	SYNONYMOUS_CODING	NA
Kabuli_wild_15217116	1399934959	*Ca_kabuli_Chr03*	15217116	A/G	NA	INTERGENIC	NA
Kabuli_wild_15386481	1399934960	*Ca_kabuli_Chr03*	15386481	A/G	Gene_Ca_17028	SYNONYMOUS_CODING	NA
Kabuli_wild_15386673	1399934961	*Ca_kabuli_Chr03*	15386673	A/G	Gene_Ca_17028	SYNONYMOUS_CODING	NA
Kabuli_wild_15596603	1399934962	*Ca_kabuli_Chr03*	15596603	G/T	Gene_Ca_17041	STOP_GAINED	NA
Kabuli_wild_15596639	1399934963	*Ca_kabuli_Chr03*	15596639	G/T	Gene_Ca_17041	NON_SYNONYMOUS_CODING	NA
Kabuli_wild_15745152	1399934964	*Ca_kabuli_Chr03*	15745152	A/C	Gene_Ca_17048	INTRON	Fork head-associated (FHA) domain
Kabuli_wild_15758068	1399934965	*Ca_kabuli_Chr03*	15758068	A/C	Gene_Ca_17049	NON_SYNONYMOUS_CODING	Pectinesterase,catalytic
Kabuli_wild_16202937	1399934966	*Ca_kabuli_Chr03*	16202937	A/T	NA	INTERGENIC	NA
Kabuli_wild_16202975	1399934967	*Ca_kabuli_Chr03*	16202975	T/C	NA	INTERGENIC	NA
Kabuli_wild_16243228	1399934968	*Ca_kabuli_Chr03*	16243228	C/T	Gene_Ca_18253	UPSTREAM	NA
Kabuli_wild_16396582	1399934969	*Ca_kabuli_Chr03*	16396582	G/T	Gene_Ca_18261	NON_SYNONYMOUS_CODING	Glyoxalase/bleomycin resistance protein/dioxygenase
Kabuli_wild_16396673	1399934970	*Ca_kabuli_Chr03*	16396673	C/A	Gene_Ca_18261	NON_SYNONYMOUS_CODING	Glyoxalase/bleomycin resistance protein/dioxygenase
Kabuli_wild_16436250	1399934971	*Ca_kabuli_Chr03*	16436250	C/T	NA	INTERGENIC	NA

SNP IDs	NCBI dbSNP accession IDs (NCBI_SS#)	Chromosomes	Physical positions (bp)	SNPs	Gene accession IDs	Sequence components of genome	Putative functions
Kabuli_wild_16436259	1399934972	*Ca_kabuli_Chr03*	16436259	C/T	NA	INTERGENIC	NA
Kabuli_wild_16436300	1399934973	*Ca_kabuli_Chr03*	16436300	G/A	NA	INTERGENIC	NA
Kabuli_wild_16436319	1399934974	*Ca_kabuli_Chr03*	16436319	G/A	NA	INTERGENIC	NA
Kabuli_wild_16436334	1399934975	*Ca_kabuli_Chr03*	16436334	C/T	NA	INTERGENIC	NA
Kabuli_wild_16436337	1399934976	*Ca_kabuli_Chr03*	16436337	C/T	NA	INTERGENIC	NA
Kabuli_wild_16436342	1399934977	*Ca_kabuli_Chr03*	16436342	T/C	NA	INTERGENIC	NA
Kabuli_wild_16436349	1399934978	*Ca_kabuli_Chr03*	16436349	C/T	NA	INTERGENIC	NA
Kabuli_wild_16587623	1399934979	*Ca_kabuli_Chr03*	16587623	A/G	Gene_Ca_18274	INTRON	Ribosomal protein L32e
Kabuli_wild_16624487	1399934980	*Ca_kabuli_Chr03*	16624487	T/C	NA	INTERGENIC	NA
Kabuli_wild_16648598	1399934981	*Ca_kabuli_Chr03*	16648598	G/A	Gene_Ca_18277	SYNONYMOUS_CODING	U box domain
Kabuli_wild_16682375	1399934982	*Ca_kabuli_Chr03*	16682375	C/G	NA	INTERGENIC	NA
Kabuli_wild_17126652	1399934983	*Ca_kabuli_Chr03*	17126652	A/G	NA	INTERGENIC	NA
Kabuli_wild_17136027	1399934984	*Ca_kabuli_Chr03*	17136027	G/A	NA	INTERGENIC	NA
Kabuli_wild_17136071	1399934985	*Ca_kabuli_Chr03*	17136071	C/G	NA	INTERGENIC	NA
Kabuli_wild_17136097	1399934986	*Ca_kabuli_Chr03*	17136097	C/T	NA	INTERGENIC	NA
Kabuli_wild_17334924	1399934987	*Ca_kabuli_Chr03*	17334924	G/T	NA	INTERGENIC	NA
Kabuli_wild_17362983	1399934988	*Ca_kabuli_Chr03*	17362983	C/G	NA	INTERGENIC	NA
Kabuli_wild_17460012	1399934989	*Ca_kabuli_Chr03*	17460012	C/A	NA	INTERGENIC	NA
Kabuli_wild_17679404	1399934990	*Ca_kabuli_Chr03*	17679404	C/T	Gene_Ca_21115	SYNONYMOUS_CODING	NA
Kabuli_wild_17679409	1399934991	*Ca_kabuli_Chr03*	17679409	C/T	Gene_Ca_21115	SYNONYMOUS_CODING	NA
Kabuli_wild_17679436	1399934992	*Ca_kabuli_Chr03*	17679436	G/A	Gene_Ca_21115	NON_SYNONYMOUS_CODING	NA
Kabuli_wild_17679479	1399934993	*Ca_kabuli_Chr03*	17679479	G/C	Gene_Ca_21115	NON_SYNONYMOUS_CODING	NA
Kabuli_wild_17679510	1399934994	*Ca_kabuli_Chr03*	17679510	G/A	Gene_Ca_21115	SYNONYMOUS_CODING	NA
Kabuli_wild_17914677	1399934995	*Ca_kabuli_Chr03*	17914677	T/G	Gene_Ca_21129	DOWNSTREAM	Zinc finger,N-recognin
Kabuli_wild_17936677	1399934996	*Ca_kabuli_Chr03*	17936677	A/G	NA	INTERGENIC	NA
Kabuli_wild_17936842	1399934997	*Ca_kabuli_Chr03*	17936842	A/G	NA	INTERGENIC	NA
Kabuli_wild_17984978	1399934998	*Ca_kabuli_Chr03*	17984978	T/G	Gene_Ca_21132	SYNONYMOUS_CODING	Protein kinase,catalytic domain
Kabuli_wild_17986521	1399934999	*Ca_kabuli_Chr03*	17986521	G/C	Gene_Ca_21132	INTRON	Protein kinase,catalytic domain
Kabuli_wild_17986525	1399935000	*Ca_kabuli_Chr03*	17986525	G/T	Gene_Ca_21132	INTRON	Protein kinase,catalytic domain
Kabuli_wild_17986529	1399935001	*Ca_kabuli_Chr03*	17986529	A/G	Gene_Ca_21132	NON_SYNONYMOUS_CODING	Protein kinase,catalytic domain
Kabuli_wild_18024415	1399935002	*Ca_kabuli_Chr03*	18024415	A/C	NA	INTERGENIC	NA
Kabuli_wild_18110655	1399935003	*Ca_kabuli_Chr03*	18110655	T/C	Gene_Ca_21138	NON_SYNONYMOUS_CODING	Transcription facto rGRAS
Kabuli_wild_18215321	1399935004	*Ca_kabuli_Chr03*	18215321	G/C	Gene_Ca_19612	SYNONYMOUS_CODING	Oligo peptide transporter
Kabuli_wild_18215332	1399935005	*Ca_kabuli_Chr03*	18215332	T/C	Gene_Ca_19612	NON_SYNONYMOUS_CODING	Oligo peptide transporter
Kabuli_wild_18264564	1399935006	*Ca_kabuli_Chr03*	18264564	A/G	Gene_Ca_19613	INTRON	PI31proteasomeregulator
Kabuli_wild_18264593	1399935007	*Ca_kabuli_Chr03*	18264593	T/C	Gene_Ca_19613	INTRON	PI31proteasomeregulator
Kabuli_wild_18264613	1399935008	*Ca_kabuli_Chr03*	18264613	C/T	Gene_Ca_19613	INTRON	PI31proteasomeregulator
Kabuli_wild_18264614	1399935009	*Ca_kabuli_Chr03*	18264614	A/T	Gene_Ca_19613	INTRON	PI31proteasomeregulator
Kabuli_wild_18265036	1399935010	*Ca_kabuli_Chr03*	18265036	A/G	Gene_Ca_19613	NON_SYNONYMOUS_CODING	PI31proteasomeregulator
Kabuli_wild_18330208	1399935011	*Ca_kabuli_Chr03*	18330208	G/A	NA	INTERGENIC	NA
Kabuli_wild_18355427	1399935012	*Ca_kabuli_Chr03*	18355427	T/C	Gene_Ca_19620	SYNONYMOUS_CODING	Protein of unknown function DUF3411
Kabuli_wild_18355445	1399935013	*Ca_kabuli_Chr03*	18355445	T/C	Gene_Ca_19620	SYNONYMOUS_CODING	Protein of unknown function DUF3411
Kabuli_wild_18375547	1399935014	*Ca_kabuli_Chr03*	18375547	T/C	Gene_Ca_19623	DOWNSTREAM	GlutathioneS-transferase,N-terminal
Kabuli_wild_18382404	1399935015	*Ca_kabuli_Chr03*	18382404	T/C	Gene_Ca_19625	DOWNSTREAM	GlutathioneS-transferase,N-terminal

SNP IDs	NCBI dbSNP accession IDs (NCBI_SS#)	Chromosomes	Physical positions (bp)	SNPs	Gene accession IDs	Sequence components of genome	Putative functions
Kabuli_wild_18383884	1399935016	*Ca_kabuli_Chr03*	18383884	T/C	NA	INTERGENIC	NA
Kabuli_wild_18383900	1399935017	*Ca_kabuli_Chr03*	18383900	C/G	NA	INTERGENIC	NA
Kabuli_wild_18467707	1399935018	*Ca_kabuli_Chr03*	18467707	C/G	Gene_Ca_19629	INTRON	NA
Kabuli_wild_18476006	1399935019	*Ca_kabuli_Chr03*	18476006	A/G	NA	INTERGENIC	NA
Kabuli_wild_18476065	1399935020	*Ca_kabuli_Chr03*	18476065	C/A	NA	INTERGENIC	NA
Kabuli_wild_18658494	1399935021	*Ca_kabuli_Chr03*	18658494	T/G	NA	INTERGENIC	NA
Kabuli_wild_19372722	1399935022	*Ca_kabuli_Chr03*	19372722	G/T	NA	INTERGENIC	NA
Kabuli_wild_19442839	1399935023	*Ca_kabuli_Chr03*	19442839	G/A	NA	INTERGENIC	NA
Kabuli_wild_19442847	1399935024	*Ca_kabuli_Chr03*	19442847	C/T	NA	INTERGENIC	NA
Kabuli_wild_19442884	1399935025	*Ca_kabuli_Chr03*	19442884	G/C	NA	INTERGENIC	NA
Kabuli_wild_19445920	1399935026	*Ca_kabuli_Chr03*	19445920	T/A	NA	INTERGENIC	NA
Kabuli_wild_19445925	1399935027	*Ca_kabuli_Chr03*	19445925	A/G	NA	INTERGENIC	NA
Kabuli_wild_19475095	1399935028	*Ca_kabuli_Chr03*	19475095	G/T	Gene_Ca_19344	DOWNSTREAM	Alcohol dehydrogenase superfamily, zinc-containing
Kabuli_wild_19490587	1399935029	*Ca_kabuli_Chr03*	19490587	G/A	Gene_Ca_19346	INTRON	Sodium/calciumexchangermembraneregion
Kabuli_wild_19742530	1399935030	*Ca_kabuli_Chr03*	19742530	C/T	NA	INTERGENIC	NA
Kabuli_wild_19742549	1399935031	*Ca_kabuli_Chr03*	19742549	C/T	NA	INTERGENIC	NA
Kabuli_wild_19742600	1399935032	*Ca_kabuli_Chr03*	19742600	G/A	NA	INTERGENIC	NA
Kabuli_wild_19788512	1399935033	*Ca_kabuli_Chr03*	19788512	G/A	NA	INTERGENIC	NA
Kabuli_wild_19802763	1399935034	*Ca_kabuli_Chr03*	19802763	C/T	Gene_Ca_09383	INTRON	Adenosine/AMPdeaminase
Kabuli_wild_19804985	1399935035	*Ca_kabuli_Chr03*	19804985	T/C	Gene_Ca_09383	SYNONYMOUS_CODING	Adenosine/AMPdeaminase
Kabuli_wild_19805063	1399935036	*Ca_kabuli_Chr03*	19805063	A/C	Gene_Ca_09383	SYNONYMOUS_CODING	Adenosine/AMPdeaminase
Kabuli_wild_19829510	1399935037	*Ca_kabuli_Chr03*	19829510	G/T	Gene_Ca_09385	UPSTREAM	Disease resistance protein
Kabuli_wild_19850532	1399935038	*Ca_kabuli_Chr03*	19850532	T/C	Gene_Ca_09386	DOWNSTREAM	Ubi Aprenyltransferase family
Kabuli_wild_19850589	1399935039	*Ca_kabuli_Chr03*	19850589	C/T	Gene_Ca_09386	DOWNSTREAM	Ubi Aprenyltransferase family
Kabuli_wild_19851504	1399935040	*Ca_kabuli_Chr03*	19851504	T/C	Gene_Ca_09386	DOWNSTREAM	Ubi Aprenyltransferase family
Kabuli_wild_19851520	1399935041	*Ca_kabuli_Chr03*	19851520	T/C	Gene_Ca_09386	DOWNSTREAM	Ubi Aprenyltransferase family
Kabuli_wild_19857613	1399935042	*Ca_kabuli_Chr03*	19857613	T/A	Gene_Ca_09388	INTRON	PeptidaseM24,structural domain
Kabuli_wild_19878913	1399935043	*Ca_kabuli_Chr03*	19878913	T/C	NA	INTERGENIC	NA
Kabuli_wild_19883927	1399935044	*Ca_kabuli_Chr03*	19883927	A/G	Gene_Ca_09392	NON_SYNONYMOUS_CODING	Importin-beta,N-terminal
Kabuli_wild_19896800	1399935045	*Ca_kabuli_Chr03*	19896800	C/A	Gene_Ca_09393	NON_SYNONYMOUS_CODING	Peptidase C48, SUMO/Sentrin/Ubl1
Kabuli_wild_19896809	1399935046	*Ca_kabuli_Chr03*	19896809	A/G	Gene_Ca_09393	NON_SYNONYMOUS_CODING	Peptidase C48, SUMO/Sentrin/Ubl1
Kabuli_wild_19896819	1399935047	*Ca_kabuli_Chr03*	19896819	C/T	Gene_Ca_09393	NON_SYNONYMOUS_CODING	Peptidase C48, SUMO/Sentrin/Ubl1
Kabuli_wild_19896848	1399935048	*Ca_kabuli_Chr03*	19896848	C/T	Gene_Ca_09393	NON_SYNONYMOUS_CODING	Peptidase C48, SUMO/Sentrin/Ubl1
Kabuli_wild_19896858	1399935049	*Ca_kabuli_Chr03*	19896858	C/T	Gene_Ca_09393	NON_SYNONYMOUS_CODING	Peptidase C48, SUMO/Sentrin/Ubl1
Kabuli_wild_19896867	1399935050	*Ca_kabuli_Chr03*	19896867	C/T	Gene_Ca_09393	NON_SYNONYMOUS_CODING	Peptidase C48, SUMO/Sentrin/Ubl1
Kabuli_wild_19907774	1399935051	*Ca_kabuli_Chr03*	19907774	T/C	NA	INTERGENIC	NA
Kabuli_wild_19956343	1399935052	*Ca_kabuli_Chr03*	19956343	C/T	NA	INTERGENIC	NA
Kabuli_wild_19956391	1399935053	*Ca_kabuli_Chr03*	19956391	C/T	Gene_Ca_09398	INTRON	Ubiquitin
Kabuli_wild_19956452	1399935054	*Ca_kabuli_Chr03*	19956452	G/A	Gene_Ca_09398	INTRON	Ubiquitin
Kabuli_wild_19978334	1399935055	*Ca_kabuli_Chr03*	19978334	A/C	Gene_Ca_09399	NON_SYNONYMOUS_CODING	Ammoniumtransporter
Kabuli_wild_19978343	1399935056	*Ca_kabuli_Chr03*	19978343	A/C	Gene_Ca_09399	NON_SYNONYMOUS_CODING	Ammoniumtransporter
Kabuli_wild_19978407	1399935057	*Ca_kabuli_Chr03*	19978407	C/T	Gene_Ca_09399	SYNONYMOUS_CODING	Ammoniumtransporter
Kabuli_wild_19979288	1399935058	*Ca_kabuli_Chr03*	19979288	A/T	Gene_Ca_09399	SYNONYMOUS_CODING	Ammoniumtransporter
Kabuli_wild_19979318	1399935059	*Ca_kabuli_Chr03*	19979318	C/A	Gene_Ca_09399	SYNONYMOUS_CODING	Ammoniumtransporter

SNP IDs	NCBI dbSNP accession IDs (NCBI_SS#)	Chromosomes	Physical positions (bp)	SNPs	Gene accession IDs	Sequence components of genome	Putative functions
Kabuli_wild_20000967	1399935060	*Ca_kabuli_Chr03*	20000967	C/G	Gene_Ca_09401	UPSTREAM	Aminotransferase-like,plant mobile domain
Kabuli_wild_20021912	1399935061	*Ca_kabuli_Chr03*	20021912	C/A	Gene_Ca_09405	NON_SYNONYMOUS_CODING	Tetra tricopeptide repeat
Kabuli_wild_20022247	1399935062	*Ca_kabuli_Chr03*	20022247	G/A	Gene_Ca_09405	SYNONYMOUS_CODING	Tetra tricopeptide repeat
Kabuli_wild_20022253	1399935063	*Ca_kabuli_Chr03*	20022253	G/A	Gene_Ca_09405	SYNONYMOUS_CODING	Tetra tricopeptide repeat
Kabuli_wild_20025220	1399935064	*Ca_kabuli_Chr03*	20025220	A/G	Gene_Ca_09405	INTRON	Tetra tricopeptide repeat
Kabuli_wild_20025221	1399935065	*Ca_kabuli_Chr03*	20025221	T/G	Gene_Ca_09405	INTRON	Tetra tricopeptide repeat
Kabuli_wild_20025423	1399935066	*Ca_kabuli_Chr03*	20025423	A/T	Gene_Ca_09405	INTRON	Tetra tricopeptide repeat
Kabuli_wild_20030298	1399935067	*Ca_kabuli_Chr03*	20030298	G/A	Gene_Ca_09405	DOWNSTREAM	Tetra tricopeptide repeat
Kabuli_wild_20240118	1399935068	*Ca_kabuli_Chr03*	20240118	T/G	Gene_Ca_09424	INTRON	CCAAT-binding factor
Kabuli_wild_20242663	1399935069	*Ca_kabuli_Chr03*	20242663	C/T	Gene_Ca_09424	INTRON	CCAAT-binding factor
Kabuli_wild_20269329	1399935070	*Ca_kabuli_Chr03*	20269329	G/A	Gene_Ca_09427	NON_SYNONYMOUS_CODING	NA
Kabuli_wild_20365442	1399935071	*Ca_kabuli_Chr03*	20365442	A/C	NA	INTERGENIC	NA
Kabuli_wild_20415615	1399935072	*Ca_kabuli_Chr03*	20415615	A/G	NA	INTERGENIC	NA
Kabuli_wild_20487325	1399935073	*Ca_kabuli_Chr03*	20487325	A/T	NA	INTERGENIC	NA
Kabuli_wild_20552836	1399935074	*Ca_kabuli_Chr03*	20552836	T/G	Gene_Ca_09446	NON_SYNONYMOUS_CODING	NA
Kabuli_wild_20794976	1399935075	*Ca_kabuli_Chr03*	20794976	A/C	Gene_Ca_09455	INTRON	Integrase,catalyticcore
Kabuli_wild_20794993	1399935076	*Ca_kabuli_Chr03*	20794993	A/G	Gene_Ca_09455	INTRON	Integrase,catalyticcore
Kabuli_wild_20795014	1399935077	*Ca_kabuli_Chr03*	20795014	C/T	Gene_Ca_09455	INTRON	Integrase,catalyticcore
Kabuli_wild_20795015	1399935078	*Ca_kabuli_Chr03*	20795015	A/C	Gene_Ca_09455	INTRON	Integrase,catalyticcore
Kabuli_wild_20799763	1399935079	*Ca_kabuli_Chr03*	20799763	T/G	Gene_Ca_09455	UPSTREAM	Integrase,catalyticcore
Kabuli_wild_20799811	1399935080	*Ca_kabuli_Chr03*	20799811	C/T	Gene_Ca_09455	UPSTREAM	Integrase,catalyticcore
Kabuli_wild_20799817	1399935081	*Ca_kabuli_Chr03*	20799817	G/A	Gene_Ca_09455	UPSTREAM	Integrase,catalyticcore
Kabuli_wild_20799820	1399935082	*Ca_kabuli_Chr03*	20799820	A/G	Gene_Ca_09455	UPSTREAM	Integrase,catalyticcore
Kabuli_wild_20799835	1399935083	*Ca_kabuli_Chr03*	20799835	A/G	Gene_Ca_09455	UPSTREAM	Integrase,catalyticcore
Kabuli_wild_20799841	1399935084	*Ca_kabuli_Chr03*	20799841	C/T	Gene_Ca_09455	UPSTREAM	Integrase,catalyticcore
Kabuli_wild_20829399	1399935085	*Ca_kabuli_Chr03*	20829399	A/G	Gene_Ca_09459	INTRON	HEAT
Kabuli_wild_21069672	1399935086	*Ca_kabuli_Chr03*	21069672	A/G	Gene_Ca_09474	SYNONYMOUS_CODING	Aminoacyl-tRNAsynthetase,classII(D/K/N)
Kabuli_wild_21069714	1399935087	*Ca_kabuli_Chr03*	21069714	G/A	Gene_Ca_09474	INTRON	Aminoacyl-tRNAsynthetase,classII(D/K/N)
Kabuli_wild_21069910	1399935088	*Ca_kabuli_Chr03*	21069910	C/T	Gene_Ca_09474	SYNONYMOUS_CODING	Aminoacyl-tRNAsynthetase,classII(D/K/N)
Kabuli_wild_21069916	1399935089	*Ca_kabuli_Chr03*	21069916	C/T	Gene_Ca_09474	SYNONYMOUS_CODING	Aminoacyl-tRNAsynthetase,classII(D/K/N)
Kabuli_wild_21069917	1399935090	*Ca_kabuli_Chr03*	21069917	G/A	Gene_Ca_09474	NON_SYNONYMOUS_CODING	Aminoacyl-tRNAsynthetase,classII(D/K/N)
Kabuli_wild_21069959	1399935091	*Ca_kabuli_Chr03*	21069959	G/A	Gene_Ca_09474	INTRON	Aminoacyl-tRNAsynthetase,classII(D/K/N)
Kabuli_wild_21069986	1399935092	*Ca_kabuli_Chr03*	21069986	A/C	Gene_Ca_09474	INTRON	Aminoacyl-tRNAsynthetase,classII(D/K/N)
Kabuli_wild_21246629	1399935093	*Ca_kabuli_Chr03*	21246629	T/C	Gene_Ca_09487	INTRON	TranslationelongationfactorP/YeiP,central
Kabuli_wild_21246877	1399935094	*Ca_kabuli_Chr03*	21246877	T/C	Gene_Ca_09487	INTRON	TranslationelongationfactorP/YeiP,central
Kabuli_wild_21280166	1399935095	*Ca_kabuli_Chr03*	21280166	A/G	Gene_Ca_09490	SYNONYMOUS_CODING	Protein kinase,catalytic domain
Kabuli_wild_21385460	1399935096	*Ca_kabuli_Chr03*	21385460	A/C	Gene_Ca_09496	NON_SYNONYMOUS_CODING	ATPase,P-type,K/Mg/Cd/Cu/Zn/Na/Ca/Na/H-transporter
Kabuli_wild_21421082	1399935097	*Ca_kabuli_Chr03*	21421082	A/G	NA	INTERGENIC	NA
Kabuli_wild_21421146	1399935098	*Ca_kabuli_Chr03*	21421146	C/A	NA	INTERGENIC	NA
Kabuli_wild_21421162	1399935099	*Ca_kabuli_Chr03*	21421162	G/A	NA	INTERGENIC	NA
Kabuli_wild_21421358	1399935100	*Ca_kabuli_Chr03*	21421358	G/A	NA	INTERGENIC	NA
Kabuli_wild_21421362	1399935101	*Ca_kabuli_Chr03*	21421362	T/A	NA	INTERGENIC	NA
Kabuli_wild_21421369	1399935102	*Ca_kabuli_Chr03*	21421369	G/A	NA	INTERGENIC	NA
Kabuli_wild_21421424	1399935103	*Ca_kabuli_Chr03*	21421424	G/A	NA	INTERGENIC	NA

SNP IDs	NCBI dbSNP accession IDs (NCBI_SS#)	Chromosomes	Physical positions (bp)	SNPs	Gene accession IDs	Sequence components of genome	Putative functions
Kabuli_wild_21421440	1399935104	*Ca_kabuli_Chr03*	21421440	C/T	NA	INTERGENIC	NA
Kabuli_wild_21489004	1399935105	*Ca_kabuli_Chr03*	21489004	T/G	Gene_Ca_09505	NON_SYNONYMOUS_CODING	Ubiquitin-conjugating enzyme, E2
Kabuli_wild_21500178	1399935106	*Ca_kabuli_Chr03*	21500178	G/A	Gene_Ca_09506	DOWNSTREAM	RasGTPase
Kabuli_wild_21500179	1399935107	*Ca_kabuli_Chr03*	21500179	A/T	Gene_Ca_09506	DOWNSTREAM	RasGTPase
Kabuli_wild_21524296	1399935108	*Ca_kabuli_Chr03*	21524296	G/T	NA	INTERGENIC	NA
Kabuli_wild_21524379	1399935109	*Ca_kabuli_Chr03*	21524379	A/G	NA	INTERGENIC	NA
Kabuli_wild_21558354	1399935110	*Ca_kabuli_Chr03*	21558354	G/A	NA	INTERGENIC	NA
Kabuli_wild_21558357	1399935111	*Ca_kabuli_Chr03*	21558357	T/G	NA	INTERGENIC	NA
Kabuli_wild_21687953	1399935112	*Ca_kabuli_Chr03*	21687953	C/T	Gene_Ca_09523	NON_SYNONYMOUS_CODING	NA
Kabuli_wild_21687956	1399935113	*Ca_kabuli_Chr03*	21687956	C/T	Gene_Ca_09523	SYNONYMOUS_CODING	NA
Kabuli_wild_21687959	1399935114	*Ca_kabuli_Chr03*	21687959	T/C	Gene_Ca_09523	NON_SYNONYMOUS_CODING	NA
Kabuli_wild_21687960	1399935115	*Ca_kabuli_Chr03*	21687960	G/A	Gene_Ca_09523	NON_SYNONYMOUS_CODING	NA
Kabuli_wild_21687961	1399935116	*Ca_kabuli_Chr03*	21687961	C/A	Gene_Ca_09523	STOP_GAINED	NA
Kabuli_wild_21687966	1399935117	*Ca_kabuli_Chr03*	21687966	C/A	Gene_Ca_09523	NON_SYNONYMOUS_CODING	NA
Kabuli_wild_21741769	1399935118	*Ca_kabuli_Chr03*	21741769	C/T	NA	INTERGENIC	NA
Kabuli_wild_21809498	1399935119	*Ca_kabuli_Chr03*	21809498	T/G	NA	INTERGENIC	NA
Kabuli_wild_21809503	1399935120	*Ca_kabuli_Chr03*	21809503	T/C	NA	INTERGENIC	NA
Kabuli_wild_21809546	1399935121	*Ca_kabuli_Chr03*	21809546	T/A	NA	INTERGENIC	NA
Kabuli_wild_21950053	1399935122	*Ca_kabuli_Chr03*	21950053	C/G	Gene_Ca_20431	INTRON	Domain of unknown function DUF296
Kabuli_wild_22008087	1399935123	*Ca_kabuli_Chr03*	22008087	G/A	Gene_Ca_20426	NON_SYNONYMOUS_CODING	SEC7-like
Kabuli_wild_22026111	1399935124	*Ca_kabuli_Chr03*	22026111	A/G	Gene_Ca_20423	INTRON	Armadillo
Kabuli_wild_22062091	1399935125	*Ca_kabuli_Chr03*	22062091	T/G	Gene_Ca_20420	INTRON	Fork head-associated (FHA) domain
Kabuli_wild_22062137	1399935126	*Ca_kabuli_Chr03*	22062137	A/T	Gene_Ca_20420	INTRON	Fork head-associated (FHA) domain
Kabuli_wild_22062965	1399935127	*Ca_kabuli_Chr03*	22062965	C/T	Gene_Ca_20420	INTRON	Fork head-associated (FHA) domain
Kabuli_wild_22188933	1399935128	*Ca_kabuli_Chr03*	22188933	C/T	Gene_Ca_20411	SYNONYMOUS_CODING	Domain of unknown function DUF1088
Kabuli_wild_22200927	1399935129	*Ca_kabuli_Chr03*	22200927	T/C	Gene_Ca_20410	SYNONYMOUS_CODING	NA
Kabuli_wild_22202485	1399935130	*Ca_kabuli_Chr03*	22202485	T/G	Gene_Ca_20410	NON_SYNONYMOUS_CODING	NA
Kabuli_wild_22253842	1399935131	*Ca_kabuli_Chr03*	22253842	T/C	Gene_Ca_20405	INTRON	Glycosyltransferase,family8
Kabuli_wild_22346294	1399935132	*Ca_kabuli_Chr03*	22346294	T/C	Gene_Ca_06276	NON_SYNONYMOUS_CODING	PeptidaseS8/S53,subtilisin/kexin/sedolisin
Kabuli_wild_22404660	1399935133	*Ca_kabuli_Chr03*	22404660	G/T	NA	INTERGENIC	NA
Kabuli_wild_22539182	1399935134	*Ca_kabuli_Chr03*	22539182	C/T	Gene_Ca_06260	NON_SYNONYMOUS_CODING	Armadillo
Kabuli_wild_22659967	1399935135	*Ca_kabuli_Chr03*	22659967	A/C	Gene_Ca_06245	NON_SYNONYMOUS_CODING	SPX,N-terminal
Kabuli_wild_22727393	1399935136	*Ca_kabuli_Chr03*	22727393	G/A	Gene_Ca_06238	NON_SYNONYMOUS_CODING	Malicoxidoreductase
Kabuli_wild_22727439	1399935137	*Ca_kabuli_Chr03*	22727439	A/C	Gene_Ca_06238	SYNONYMOUS_CODING	Malicoxidoreductase
Kabuli_wild_22730700	1399935138	*Ca_kabuli_Chr03*	22730700	T/G	Gene_Ca_06238	SYNONYMOUS_CODING	Malicoxidoreductase
Kabuli_wild_22730976	1399935139	*Ca_kabuli_Chr03*	22730976	A/G	Gene_Ca_06238	NON_SYNONYMOUS_CODING	Malicoxidoreductase
Kabuli_wild_22765862	1399935140	*Ca_kabuli_Chr03*	22765862	T/C	NA	INTERGENIC	NA
Kabuli_wild_22765987	1399935141	*Ca_kabuli_Chr03*	22765987	G/T	NA	INTERGENIC	NA
Kabuli_wild_22772579	1399935142	*Ca_kabuli_Chr03*	22772579	A/G	NA	INTERGENIC	NA
Kabuli_wild_22846051	1399935143	*Ca_kabuli_Chr03*	22846051	G/A	Gene_Ca_06225	INTRON	RNAhelicase,ATP-dependent,DEAD-box,conservedsite
Kabuli_wild_22990523	1399935144	*Ca_kabuli_Chr03*	22990523	C/T	Gene_Ca_06210	NON_SYNONYMOUS_CODING	Glycosidehydrolase,family14
Kabuli_wild_23042249	1399935145	*Ca_kabuli_Chr03*	23042249	A/C	Gene_Ca_06206	NON_SYNONYMOUS_CODING	Mediatorcomplex,subunitMed12
Kabuli_wild_23053066	1399935146	*Ca_kabuli_Chr03*	23053066	A/C	NA	INTERGENIC	NA
Kabuli_wild_23088854	1399935147	*Ca_kabuli_Chr03*	23088854	C/G	Gene_Ca_06203	NON_SYNONYMOUS_CODING	Down-regulated-in-metastasisprotein

SNP IDs	NCBI dbSNP accession IDs (NCBI_SS#)	Chromosomes	Physical positions (bp)	SNPs	Gene accession IDs	Sequence components of genome	Putative functions
Kabuli_wild_23279589	1399935148	*Ca_kabuli_Chr03*	23279589	T/C	Gene_Ca_06189	NON_SYNONYMOUS_CODING	SNF2-related
Kabuli_wild_23299089	1399935149	*Ca_kabuli_Chr03*	23299089	G/A	Gene_Ca_06188	NON_SYNONYMOUS_CODING	ARID/BRIGHTDNA-binding domain
Kabuli_wild_23341087	1399935150	*Ca_kabuli_Chr03*	23341087	A/C	Gene_Ca_06186	INTRON	Protein of unknown function DUF3522
Kabuli_wild_23341416	1399935151	*Ca_kabuli_Chr03*	23341416	C/A	Gene_Ca_06186	INTRON	Protein of unknown function DUF3522
Kabuli_wild_23400864	1399935152	*Ca_kabuli_Chr03*	23400864	A/C	NA	INTERGENIC	NA
Kabuli_wild_23455008	1399935153	*Ca_kabuli_Chr03*	23455008	A/G	Gene_Ca_06180	DOWNSTREAM	Glycosidehydrolase,family28
Kabuli_wild_23455128	1399935154	*Ca_kabuli_Chr03*	23455128	A/G	Gene_Ca_06180	DOWNSTREAM	Glycosidehydrolase,family28
Kabuli_wild_23461370	1399935155	*Ca_kabuli_Chr03*	23461370	C/A	NA	INTERGENIC	NA
Kabuli_wild_23570083	1399935156	*Ca_kabuli_Chr03*	23570083	C/A	Gene_Ca_06172	UPSTREAM	D-isomerspecific2-hydroxyaciddehydrogenase,NAD-binding
Kabuli_wild_23593275	1399935157	*Ca_kabuli_Chr03*	23593275	G/A	NA	INTERGENIC	NA
Kabuli_wild_23593287	1399935158	*Ca_kabuli_Chr03*	23593287	T/G	NA	INTERGENIC	NA
Kabuli_wild_23593288	1399935159	*Ca_kabuli_Chr03*	23593288	G/A	NA	INTERGENIC	NA
Kabuli_wild_23593294	1399935160	*Ca_kabuli_Chr03*	23593294	T/C	NA	INTERGENIC	NA
Kabuli_wild_23593308	1399935161	*Ca_kabuli_Chr03*	23593308	G/A	NA	INTERGENIC	NA
Kabuli_wild_23593309	1399935162	*Ca_kabuli_Chr03*	23593309	T/C	NA	INTERGENIC	NA
Kabuli_wild_23593314	1399935163	*Ca_kabuli_Chr03*	23593314	C/G	NA	INTERGENIC	NA
Kabuli_wild_23593318	1399935164	*Ca_kabuli_Chr03*	23593318	G/A	NA	INTERGENIC	NA
Kabuli_wild_23593326	1399935165	*Ca_kabuli_Chr03*	23593326	T/G	NA	INTERGENIC	NA
Kabuli_wild_23593328	1399935166	*Ca_kabuli_Chr03*	23593328	C/A	NA	INTERGENIC	NA
Kabuli_wild_23695061	1399935167	*Ca_kabuli_Chr03*	23695061	G/T	Gene_Ca_06159	NON_SYNONYMOUS_CODING	PeptidaseS8/S53,subtilisin/kexin/sedolisin
Kabuli_wild_23744259	1399935168	*Ca_kabuli_Chr03*	23744259	T/C	Gene_Ca_06154	INTRON	UDP-glucuronosyl/UDP-glucosyltransferase
Kabuli_wild_23786875	1399935169	*Ca_kabuli_Chr03*	23786875	T/G	NA	INTERGENIC	NA
Kabuli_wild_23918105	1399935170	*Ca_kabuli_Chr03*	23918105	T/C	NA	INTERGENIC	NA
Kabuli_wild_23966110	1399935171	*Ca_kabuli_Chr03*	23966110	G/A	Gene_Ca_06124	SYNONYMOUS_CODING	DNA-binding WRKY
Kabuli_wild_24024507	1399935172	*Ca_kabuli_Chr03*	24024507	A/C	NA	INTERGENIC	NA
Kabuli_wild_24024509	1399935173	*Ca_kabuli_Chr03*	24024509	T/C	NA	INTERGENIC	NA
Kabuli_wild_24024513	1399935174	*Ca_kabuli_Chr03*	24024513	A/C	NA	INTERGENIC	NA
Kabuli_wild_24099599	1399935175	*Ca_kabuli_Chr03*	24099599	A/T	NA	INTERGENIC	NA
Kabuli_wild_24136623	1399935176	*Ca_kabuli_Chr03*	24136623	A/G	Gene_Ca_06105	NON_SYNONYMOUS_CODING	Short-chaindehydrogenase/reductaseSDR
Kabuli_wild_24161791	1399935177	*Ca_kabuli_Chr03*	24161791	T/C	Gene_Ca_06103	NON_SYNONYMOUS_CODING	Lecithin:cholesterolacyltransferase
Kabuli_wild_24194574	1399935178	*Ca_kabuli_Chr03*	24194574	C/T	NA	INTERGENIC	NA
Kabuli_wild_24209881	1399935179	*Ca_kabuli_Chr03*	24209881	T/A	NA	INTERGENIC	NA
Kabuli_wild_24297894	1399935180	*Ca_kabuli_Chr03*	24297894	C/A	Gene_Ca_06091	NON_SYNONYMOUS_CODING	NA
Kabuli_wild_24355197	1399935181	*Ca_kabuli_Chr03*	24355197	T/G	NA	INTERGENIC	NA
Kabuli_wild_24417498	1399935182	*Ca_kabuli_Chr03*	24417498	A/C	Gene_Ca_06076	SYNONYMOUS_CODING	Actin-bindingFH2/DRFautoregulatory
Kabuli_wild_24417507	1399935183	*Ca_kabuli_Chr03*	24417507	A/C	Gene_Ca_06076	SYNONYMOUS_CODING	Actin-bindingFH2/DRFautoregulatory
Kabuli_wild_24464677	1399935184	*Ca_kabuli_Chr03*	24464677	G/A	Gene_Ca_06070	NON_SYNONYMOUS_CODING	Protein phosphatase 2C, manganese/magnesium aspartate binding site
Kabuli_wild_24475663	1399935185	*Ca_kabuli_Chr03*	24475663	A/T	Gene_Ca_06069	INTRON	Sedlin
Kabuli_wild_24475712	1399935186	*Ca_kabuli_Chr03*	24475712	A/G	Gene_Ca_06069	INTRON	Sedlin
Kabuli_wild_24531720	1399935187	*Ca_kabuli_Chr03*	24531720	C/T	NA	INTERGENIC	NA
Kabuli_wild_24538726	1399935188	*Ca_kabuli_Chr03*	24538726	A/C	NA	INTERGENIC	NA
Kabuli_wild_24637834	1399935189	*Ca_kabuli_Chr03*	24637834	G/T	NA	INTERGENIC	NA
Kabuli_wild_24637837	1399935190	*Ca_kabuli_Chr03*	24637837	A/C	NA	INTERGENIC	NA
Kabuli_wild_24664550	1399935191	*Ca_kabuli_Chr03*	24664550	T/G	NA	INTERGENIC	NA

SNP IDs	NCBI dbSNP accession IDs (NCBI_SS#)	Chromosomes	Physical positions (bp)	SNPs	Gene accession IDs	Sequence components of genome	Putative functions
Kabuli_wild_24664582	1399935192	*Ca_kabuli_Chr03*	24664582	G/T	NA	INTERGENIC	NA
Kabuli_wild_25062362	1399935193	*Ca_kabuli_Chr03*	25062362	G/A	Gene_Ca_06022	SYNONYMOUS_CODING	Protein kinase,catalytic domain
Kabuli_wild_25145247	1399935194	*Ca_kabuli_Chr03*	25145247	T/C	Gene_Ca_06015	SYNONYMOUS_CODING	LisHdimerisationmotif
Kabuli_wild_25241115	1399935195	*Ca_kabuli_Chr03*	25241115	G/A	Gene_Ca_06005	NON_SYNONYMOUS_CODING	DNArepairmetallo-beta-lactamase
Kabuli_wild_25244194	1399935196	*Ca_kabuli_Chr03*	25244194	A/C	Gene_Ca_06004	SYNONYMOUS_CODING	Methyltransferasesmall
Kabuli_wild_25462086	1399935197	*Ca_kabuli_Chr03*	25462086	A/G	Gene_Ca_05988	NON_SYNONYMOUS_CODING	Glycerophosphoryldiester phosphodiesterase
Kabuli_wild_25462227	1399935198	*Ca_kabuli_Chr03*	25462227	A/C	Gene_Ca_05988	NON_SYNONYMOUS_CODING	Glycerophosphoryldiester phosphodiesterase
Kabuli_wild_25504708	1399935199	*Ca_kabuli_Chr03*	25504708	A/G	Gene_Ca_05985	DOWNSTREAM	Zinc finger,B-box
Kabuli_wild_25504743	1399935200	*Ca_kabuli_Chr03*	25504743	G/A	Gene_Ca_05985	DOWNSTREAM	Zinc finger,B-box
Kabuli_wild_25580072	1399935201	*Ca_kabuli_Chr03*	25580072	A/T	Gene_Ca_05977	NON_SYNONYMOUS_CODING	Nodulin-like
Kabuli_wild_25614515	1399935202	*Ca_kabuli_Chr03*	25614515	T/G	NA	INTERGENIC	NA
Kabuli_wild_25688341	1399935203	*Ca_kabuli_Chr03*	25688341	G/A	NA	INTERGENIC	NA
Kabuli_wild_25718827	1399935204	*Ca_kabuli_Chr03*	25718827	T/A	NA	INTERGENIC	NA
Kabuli_wild_25718829	1399935205	*Ca_kabuli_Chr03*	25718829	C/A	NA	INTERGENIC	NA
Kabuli_wild_25757693	1399935206	*Ca_kabuli_Chr03*	25757693	C/T	Gene_Ca_23882	SYNONYMOUS_CODING	SET domain
Kabuli_wild_25822511	1399935207	*Ca_kabuli_Chr03*	25822511	C/A	NA	INTERGENIC	NA
Kabuli_wild_25822530	1399935208	*Ca_kabuli_Chr03*	25822530	T/C	NA	INTERGENIC	NA
Kabuli_wild_25822560	1399935209	*Ca_kabuli_Chr03*	25822560	C/T	NA	INTERGENIC	NA
Kabuli_wild_25822584	1399935210	*Ca_kabuli_Chr03*	25822584	A/T	NA	INTERGENIC	NA
Kabuli_wild_25987849	1399935211	*Ca_kabuli_Chr03*	25987849	A/T	Gene_Ca_08296	NON_SYNONYMOUS_CODING	Protein of unknown function DUF677
Kabuli_wild_26163449	1399935212	*Ca_kabuli_Chr03*	26163449	G/A	Gene_Ca_08289	NON_SYNONYMOUS_CODING	Calcium-bindingEF-hand
Kabuli_wild_26335889	1399935213	*Ca_kabuli_Chr03*	26335889	T/A	Gene_Ca_08268	SYNONYMOUS_CODING	Exportin-1/Importin-beta-like
Kabuli_wild_26339286	1399935214	*Ca_kabuli_Chr03*	26339286	T/C	Gene_Ca_08268	NON_SYNONYMOUS_CODING	Exportin-1/Importin-beta-like
Kabuli_wild_26562046	1399935215	*Ca_kabuli_Chr03*	26562046	T/C	Gene_Ca_08253	INTRON	Thioredoxin
Kabuli_wild_26573902	1399935216	*Ca_kabuli_Chr03*	26573902	A/G	Gene_Ca_08252	NON_SYNONYMOUS_CODING	NotCCR4-Notcomplexcomponent,N-terminal
Kabuli_wild_26574831	1399935217	*Ca_kabuli_Chr03*	26574831	A/G	Gene_Ca_08252	SYNONYMOUS_CODING	NotCCR4-Notcomplexcomponent,N-terminal
Kabuli_wild_26577216	1399935218	*Ca_kabuli_Chr03*	26577216	C/T	Gene_Ca_08252	STOP_GAINED	NotCCR4-Notcomplexcomponent,N-terminal
Kabuli_wild_26627150	1399935219	*Ca_kabuli_Chr03*	26627150	T/G	Gene_Ca_08246	INTRON	YTHdomain
Kabuli_wild_26678262	1399935220	*Ca_kabuli_Chr03*	26678262	G/A	NA	INTERGENIC	NA
Kabuli_wild_26678433	1399935221	*Ca_kabuli_Chr03*	26678433	T/G	NA	INTERGENIC	NA
Kabuli_wild_26726897	1399935222	*Ca_kabuli_Chr03*	26726897	A/C	NA	INTERGENIC	NA
Kabuli_wild_26752814	1399935223	*Ca_kabuli_Chr03*	26752814	A/G	Gene_Ca_08231	INTRON	HEAT
Kabuli_wild_26754514	1399935224	*Ca_kabuli_Chr03*	26754514	A/G	Gene_Ca_08231	INTRON	HEAT
Kabuli_wild_26758452	1399935225	*Ca_kabuli_Chr03*	26758452	C/A	Gene_Ca_08231	NON_SYNONYMOUS_CODING	HEAT
Kabuli_wild_26788423	1399935226	*Ca_kabuli_Chr03*	26788423	C/T	NA	INTERGENIC	NA
Kabuli_wild_26848870	1399935227	*Ca_kabuli_Chr03*	26848870	G/C	Gene_Ca_08222	NON_SYNONYMOUS_CODING	Regulatorofchromosomecondensation,RCC1
Kabuli_wild_26849288	1399935228	*Ca_kabuli_Chr03*	26849288	T/A	Gene_Ca_08222	NON_SYNONYMOUS_CODING	Regulatorofchromosomecondensation,RCC1
Kabuli_wild_26878028	1399935229	*Ca_kabuli_Chr03*	26878028	A/T	Gene_Ca_08220	DOWNSTREAM	Protein of unknown function DUF284,transmembraneeukaryotic
Kabuli_wild_26900271	1399935230	*Ca_kabuli_Chr03*	26900271	T/A	NA	INTERGENIC	NA
Kabuli_wild_26901920	1399935231	*Ca_kabuli_Chr03*	26901920	C/T	Gene_Ca_08215	INTRON	MIF4G-like,type3
Kabuli_wild_26902037	1399935232	*Ca_kabuli_Chr03*	26902037	C/T	Gene_Ca_08215	INTRON	MIF4G-like,type4
Kabuli_wild_26911443	1399935233	*Ca_kabuli_Chr03*	26911443	A/T	Gene_Ca_08214	INTRON	Methionine synthase, vitamin-B12 independent
Kabuli_wild_26911514	1399935234	*Ca_kabuli_Chr03*	26911514	A/G	Gene_Ca_08214	INTRON	Methionine synthase, vitamin-B12 independent
Kabuli_wild_26911723	1399935235	*Ca_kabuli_Chr03*	26911723	T/A	Gene_Ca_08214	INTRON	Methionine synthase, vitamin-B12 independent

SNP IDs	NCBI dbSNP accession IDs (NCBI_SS#)	Chromosomes	Physical positions (bp)	SNPs	Gene accession IDs	Sequence components of genome	Putative functions
Kabuli_wild_26911792	1399935236	*Ca_kabuli_Chr03*	26911792	A/G	Gene_Ca_08214	SYNONYMOUS_CODING	Methionine synthase, vitamin-B12 independent
Kabuli_wild_26932964	1399935237	*Ca_kabuli_Chr03*	26932964	C/A	NA	INTERGENIC	NA
Kabuli_wild_27010875	1399935238	*Ca_kabuli_Chr03*	27010875	G/T	NA	INTERGENIC	NA
Kabuli_wild_27043121	1399935239	*Ca_kabuli_Chr03*	27043121	A/T	Gene_Ca_08199	INTRON	BTB/POZ-like
Kabuli_wild_27047847	1399935240	*Ca_kabuli_Chr03*	27047847	A/C	Gene_Ca_08199	SYNONYMOUS_CODING	BTB/POZ-like
Kabuli_wild_27074877	1399935241	*Ca_kabuli_Chr03*	27074877	T/A	NA	INTERGENIC	NA
Kabuli_wild_27074884	1399935242	*Ca_kabuli_Chr03*	27074884	G/A	NA	INTERGENIC	NA
Kabuli_wild_27074913	1399935243	*Ca_kabuli_Chr03*	27074913	T/C	NA	INTERGENIC	NA
Kabuli_wild_27076716	1399935244	*Ca_kabuli_Chr03*	27076716	T/C	NA	INTERGENIC	NA
Kabuli_wild_27076735	1399935245	*Ca_kabuli_Chr03*	27076735	T/G	NA	INTERGENIC	NA
Kabuli_wild_27076744	1399935246	*Ca_kabuli_Chr03*	27076744	C/A	NA	INTERGENIC	NA
Kabuli_wild_27105602	1399935247	*Ca_kabuli_Chr03*	27105602	A/G	NA	INTERGENIC	NA
Kabuli_wild_27157852	1399935248	*Ca_kabuli_Chr03*	27157852	C/A	Gene_Ca_08188	INTRON	Transcription factor TFIIB-related
Kabuli_wild_27338521	1399935249	*Ca_kabuli_Chr03*	27338521	T/C	NA	INTERGENIC	NA
Kabuli_wild_27478173	1399935250	*Ca_kabuli_Chr03*	27478173	C/T	Gene_Ca_08159	UPSTREAM	Cellularretinaldehydebinding/alpha-tocopheroltransport
Kabuli_wild_27529608	1399935251	*Ca_kabuli_Chr03*	27529608	T/C	NA	INTERGENIC	NA
Kabuli_wild_27611527	1399935252	*Ca_kabuli_Chr03*	27611527	A/G	Gene_Ca_08147	UPSTREAM	NA
Kabuli_wild_27632100	1399935253	*Ca_kabuli_Chr03*	27632100	G/A	Gene_Ca_08144	NON_SYNONYMOUS_CODING	Myb,DNA-binding
Kabuli_wild_27654798	1399935254	*Ca_kabuli_Chr03*	27654798	A/C	Gene_Ca_08142	SYNONYMOUS_CODING	RNA recognition motif domain
Kabuli_wild_27673727	1399935255	*Ca_kabuli_Chr03*	27673727	C/A	Gene_Ca_08139	NON_SYNONYMOUS_CODING	NA
Kabuli_wild_27716358	1399935256	*Ca_kabuli_Chr03*	27716358	T/C	NA	INTERGENIC	NA
Kabuli_wild_27716396	1399935257	*Ca_kabuli_Chr03*	27716396	C/T	NA	INTERGENIC	NA
Kabuli_wild_27824683	1399935258	*Ca_kabuli_Chr03*	27824683	C/T	Gene_Ca_08124	NON_SYNONYMOUS_CODING	VQ
Kabuli_wild_27877479	1399935259	*Ca_kabuli_Chr03*	27877479	C/T	Gene_Ca_08121	SYNONYMOUS_CODING	Ubiquitin-associated/translation elongation factor EF1B, N-terminal
Kabuli_wild_27887405	1399935260	*Ca_kabuli_Chr03*	27887405	C/T	Gene_Ca_08120	INTRON	Ribosomal protein S1 ,RNA-binding domain
Kabuli_wild_27889107	1399935261	*Ca_kabuli_Chr03*	27889107	A/G	Gene_Ca_08120	NON_SYNONYMOUS_CODING	Ribosomal protein S1 ,RNA-binding domain
Kabuli_wild_27890801	1399935262	*Ca_kabuli_Chr03*	27890801	A/G	Gene_Ca_08120	SYNONYMOUS_CODING	Ribosomal protein S1 ,RNA-binding domain
Kabuli_wild_27890849	1399935263	*Ca_kabuli_Chr03*	27890849	C/T	Gene_Ca_08120	SYNONYMOUS_CODING	Ribosomal protein S1 ,RNA-binding domain
Kabuli_wild_27906044	1399935264	*Ca_kabuli_Chr03*	27906044	A/C	Gene_Ca_08119	NON_SYNONYMOUS_CODING	NA
Kabuli_wild_27969207	1399935265	*Ca_kabuli_Chr03*	27969207	C/T	Gene_Ca_08113	NON_SYNONYMOUS_CODING	Zinc finger,C2H2-type
Kabuli_wild_28174497	1399935266	*Ca_kabuli_Chr03*	28174497	G/T	Gene_Ca_26949	INTRON	WD40 repeat
Kabuli_wild_28176333	1399935267	*Ca_kabuli_Chr03*	28176333	A/C	Gene_Ca_26949	DOWNSTREAM	WD40 repeat
Kabuli_wild_28176380	1399935268	*Ca_kabuli_Chr03*	28176380	A/C	Gene_Ca_26949	DOWNSTREAM	WD40 repeat
Kabuli_wild_28277883	1399935269	*Ca_kabuli_Chr03*	28277883	T/C	Gene_Ca_07143	INTRON	Single-strandednucleicacidbindingR3H
Kabuli_wild_28375082	1399935270	*Ca_kabuli_Chr03*	28375082	G/A	Gene_Ca_07151	INTRON	Transcriptionelongationfactor,TFIIS/CRSP70,N-terminal,sub-type
Kabuli_wild_28420948	1399935271	*Ca_kabuli_Chr03*	28420948	C/T	NA	INTERGENIC	NA
Kabuli_wild_28680710	1399935272	*Ca_kabuli_Chr03*	28680710	A/G	Gene_Ca_07175	INTRON	Glutamineamidotransferase,class-II
Kabuli_wild_28680858	1399935273	*Ca_kabuli_Chr03*	28680858	C/T	Gene_Ca_07175	INTRON	Glutamineamidotransferase,class-II
Kabuli_wild_28716340	1399935274	*Ca_kabuli_Chr03*	28716340	A/G	Gene_Ca_07177	NON_SYNONYMOUS_CODING	Cellular retinaldehyde-binding/triplefunction, C-terminal
Kabuli_wild_28747799	1399935275	*Ca_kabuli_Chr03*	28747799	T/C	Gene_Ca_07179	INTRON	Fattyacidhydroxylase
Kabuli_wild_28845958	1399935276	*Ca_kabuli_Chr03*	28845958	A/C	NA	INTERGENIC	NA
Kabuli_wild_28943989	1399935277	*Ca_kabuli_Chr03*	28943989	G/T	NA	INTERGENIC	NA
Kabuli_wild_28964133	1399935278	*Ca_kabuli_Chr03*	28964133	A/C	Gene_Ca_07204	SYNONYMOUS_CODING	N-acetylglucosaminyltransferasecomponent
Kabuli_wild_29056768	1399935279	*Ca_kabuli_Chr03*	29056768	T/C	Gene_Ca_07211	SYNONYMOUS_CODING	ATPase,AAA+type,core

SNP IDs	NCBI dbSNP accession IDs (NCBI_SS#)	Chromosomes	Physical positions (bp)	SNPs	Gene accession IDs	Sequence components of genome	Putative functions
Kabuli_wild_29107776	1399935280	*Ca_kabuli_Chr03*	29107776	A/G	Gene_Ca_07215	INTRON	Heat shock protein DnaJ,N-terminal
Kabuli_wild_29155741	1399935281	*Ca_kabuli_Chr03*	29155741	C/G	Gene_Ca_07220	INTRON	Rhoterminationfactor,N-terminal
Kabuli_wild_29177885	1399935282	*Ca_kabuli_Chr03*	29177885	A/C	Gene_Ca_07223	UPSTREAM	NA
Kabuli_wild_29207338	1399935283	*Ca_kabuli_Chr03*	29207338	G/A	NA	INTERGENIC	NA
Kabuli_wild_29293798	1399935284	*Ca_kabuli_Chr03*	29293798	C/T	Gene_Ca_07236	INTRON	NA
Kabuli_wild_29302251	1399935285	*Ca_kabuli_Chr03*	29302251	C/T	Gene_Ca_07237	INTRON	Pathogenesis-related transcriptional factor/ERF, DNA-binding
Kabuli_wild_29303144	1399935286	*Ca_kabuli_Chr03*	29303144	A/C	Gene_Ca_07237	INTRON	Pathogenesis-related transcriptional factor/ERF, DNA-binding
Kabuli_wild_29304116	1399935287	*Ca_kabuli_Chr03*	29304116	A/G	Gene_Ca_07237	UPSTREAM	Pathogenesis-related transcriptional factor/ERF, DNA-binding
Kabuli_wild_29508831	1399935288	*Ca_kabuli_Chr03*	29508831	C/T	Gene_Ca_07256	NON_SYNONYMOUS_CODING	Protein kinase,catalytic domain
Kabuli_wild_29509310	1399935289	*Ca_kabuli_Chr03*	29509310	G/T	Gene_Ca_07256	SYNONYMOUS_CODING	Protein kinase,catalytic domain
Kabuli_wild_29568218	1399935290	*Ca_kabuli_Chr03*	29568218	A/T	Gene_Ca_07261	SYNONYMOUS_CODING	Zinc finger,CCCH-type
Kabuli_wild_29568611	1399935291	*Ca_kabuli_Chr03*	29568611	A/G	Gene_Ca_07261	SYNONYMOUS_CODING	Zinc finger,CCCH-type
Kabuli_wild_29603720	1399935292	*Ca_kabuli_Chr03*	29603720	T/C	NA	INTERGENIC	NA
Kabuli_wild_29666633	1399935293	*Ca_kabuli_Chr03*	29666633	C/T	NA	INTERGENIC	NA
Kabuli_wild_29698166	1399935294	*Ca_kabuli_Chr03*	29698166	G/T	NA	INTERGENIC	NA
Kabuli_wild_29698273	1399935295	*Ca_kabuli_Chr03*	29698273	A/C	NA	INTERGENIC	NA
Kabuli_wild_29698289	1399935296	*Ca_kabuli_Chr03*	29698289	G/A	NA	INTERGENIC	NA
Kabuli_wild_29730019	1399935297	*Ca_kabuli_Chr03*	29730019	G/T	Gene_Ca_07280	DOWNSTREAM	tRNA intronendo nuclease, catalytic domain-like
Kabuli_wild_29744878	1399935298	*Ca_kabuli_Chr03*	29744878	T/G	Gene_Ca_07282	SYNONYMOUS_CODING	NA
Kabuli_wild_29803866	1399935299	*Ca_kabuli_Chr03*	29803866	C/T	NA	INTERGENIC	NA
Kabuli_wild_29803962	1399935300	*Ca_kabuli_Chr03*	29803962	A/G	NA	INTERGENIC	NA
Kabuli_wild_29807091	1399935301	*Ca_kabuli_Chr03*	29807091	C/G	NA	INTERGENIC	NA
Kabuli_wild_29807248	1399935302	*Ca_kabuli_Chr03*	29807248	A/G	NA	INTERGENIC	NA
Kabuli_wild_29836337	1399935303	*Ca_kabuli_Chr03*	29836337	C/A	Gene_Ca_07291	UPSTREAM	Leucine-rich repeat
Kabuli_wild_29840012	1399935304	*Ca_kabuli_Chr03*	29840012	A/T	NA	INTERGENIC	NA
Kabuli_wild_29840022	1399935305	*Ca_kabuli_Chr03*	29840022	C/A	NA	INTERGENIC	NA
Kabuli_wild_29840028	1399935306	*Ca_kabuli_Chr03*	29840028	T/C	NA	INTERGENIC	NA
Kabuli_wild_29840029	1399935307	*Ca_kabuli_Chr03*	29840029	C/G	NA	INTERGENIC	NA
Kabuli_wild_29844509	1399935308	*Ca_kabuli_Chr03*	29844509	T/A	Gene_Ca_07292	INTRON	Basic-leucine zipper (bZIP) Transcription factor
Kabuli_wild_29869190	1399935309	*Ca_kabuli_Chr03*	29869190	G/T	Gene_Ca_07295	INTRON	CARPmotif
Kabuli_wild_29869380	1399935310	*Ca_kabuli_Chr03*	29869380	G/A	Gene_Ca_07295	NON_SYNONYMOUS_CODING	CARPmotif
Kabuli_wild_29869381	1399935311	*Ca_kabuli_Chr03*	29869381	G/A	Gene_Ca_07295	NON_SYNONYMOUS_CODING	CARPmotif
Kabuli_wild_29872643	1399935312	*Ca_kabuli_Chr03*	29872643	A/G	Gene_Ca_07295	INTRON	CARPmotif
Kabuli_wild_29872682	1399935313	*Ca_kabuli_Chr03*	29872682	G/A	Gene_Ca_07295	INTRON	CARPmotif
Kabuli_wild_29912488	1399935314	*Ca_kabuli_Chr03*	29912488	A/G	NA	INTERGENIC	NA
Kabuli_wild_29915510	1399935315	*Ca_kabuli_Chr03*	29915510	G/A	Gene_Ca_07303	DOWNSTREAM	Myb,DNA-binding
Kabuli_wild_29932378	1399935316	*Ca_kabuli_Chr03*	29932378	C/G	Gene_Ca_07304	INTRON	Myb,DNA-binding
Kabuli_wild_29932502	1399935317	*Ca_kabuli_Chr03*	29932502	G/C	Gene_Ca_07304	INTRON	Myb,DNA-binding
Kabuli_wild_29932504	1399935318	*Ca_kabuli_Chr03*	29932504	T/A	Gene_Ca_07304	INTRON	Myb,DNA-binding
Kabuli_wild_29954498	1399935319	*Ca_kabuli_Chr03*	29954498	G/A	NA	INTERGENIC	NA
Kabuli_wild_29954533	1399935320	*Ca_kabuli_Chr03*	29954533	A/G	NA	INTERGENIC	NA
Kabuli_wild_29954612	1399935321	*Ca_kabuli_Chr03*	29954612	T/G	NA	INTERGENIC	NA
Kabuli_wild_29954648	1399935322	*Ca_kabuli_Chr03*	29954648	A/T	NA	INTERGENIC	NA
Kabuli_wild_29958891	1399935323	*Ca_kabuli_Chr03*	29958891	G/C	Gene_Ca_07306	NON_SYNONYMOUS_CODING	Nse4

SNP IDs	NCBI dbSNP accession IDs (NCBI_SS#)	Chromosomes	Physical positions (bp)	SNPs	Gene accession IDs	Sequence components of genome	Putative functions
Kabuli_wild_29974532	1399935324	*Ca_kabuli_Chr03*	29974532	A/G	Gene_Ca_07307	SYNONYMOUS_CODING	Zinc finger,CCCH-type
Kabuli_wild_29974595	1399935325	*Ca_kabuli_Chr03*	29974595	C/T	Gene_Ca_07307	SYNONYMOUS_CODING	Zinc finger,CCCH-type
Kabuli_wild_29982638	1399935326	*Ca_kabuli_Chr03*	29982638	A/G	NA	INTERGENIC	NA
Kabuli_wild_29982644	1399935327	*Ca_kabuli_Chr03*	29982644	T/C	NA	INTERGENIC	NA
Kabuli_wild_29982671	1399935328	*Ca_kabuli_Chr03*	29982671	A/G	NA	INTERGENIC	NA
Kabuli_wild_30004204	1399935329	*Ca_kabuli_Chr03*	30004204	G/T	NA	INTERGENIC	NA
Kabuli_wild_30004207	1399935330	*Ca_kabuli_Chr03*	30004207	C/T	NA	INTERGENIC	NA
Kabuli_wild_30004233	1399935331	*Ca_kabuli_Chr03*	30004233	A/T	NA	INTERGENIC	NA
Kabuli_wild_30004295	1399935332	*Ca_kabuli_Chr03*	30004295	A/G	NA	INTERGENIC	NA
Kabuli_wild_30004311	1399935333	*Ca_kabuli_Chr03*	30004311	T/G	NA	INTERGENIC	NA
Kabuli_wild_30009751	1399935334	*Ca_kabuli_Chr03*	30009751	A/C	NA	INTERGENIC	NA
Kabuli_wild_30011584	1399935335	*Ca_kabuli_Chr03*	30011584	T/C	NA	INTERGENIC	NA
Kabuli_wild_30011620	1399935336	*Ca_kabuli_Chr03*	30011620	C/T	NA	INTERGENIC	NA
Kabuli_wild_30011621	1399935337	*Ca_kabuli_Chr03*	30011621	C/T	NA	INTERGENIC	NA
Kabuli_wild_30011642	1399935338	*Ca_kabuli_Chr03*	30011642	T/A	NA	INTERGENIC	NA
Kabuli_wild_30011669	1399935339	*Ca_kabuli_Chr03*	30011669	A/G	NA	INTERGENIC	NA
Kabuli_wild_30011759	1399935340	*Ca_kabuli_Chr03*	30011759	T/C	NA	INTERGENIC	NA
Kabuli_wild_30029817	1399935341	*Ca_kabuli_Chr03*	30029817	T/G	Gene_Ca_07318	INTRON	NUDIX hydrolase domain
Kabuli_wild_30029842	1399935342	*Ca_kabuli_Chr03*	30029842	G/C	Gene_Ca_07318	INTRON	NUDIX hydrolase domain
Kabuli_wild_30029858	1399935343	*Ca_kabuli_Chr03*	30029858	T/A	Gene_Ca_07318	NON_SYNONYMOUS_CODING	NUDIX hydrolase domain
Kabuli_wild_30084154	1399935344	*Ca_kabuli_Chr03*	30084154	T/C	Gene_Ca_07322	DOWNSTREAM	Homeobox
Kabuli_wild_30101476	1399935345	*Ca_kabuli_Chr03*	30101476	A/C	Gene_Ca_07324	INTRON	Methyltransferase-related
Kabuli_wild_30144223	1399935346	*Ca_kabuli_Chr03*	30144223	T/G	Gene_Ca_07331	UPSTREAM	NA
Kabuli_wild_30148952	1399935347	*Ca_kabuli_Chr03*	30148952	G/T	NA	INTERGENIC	NA
Kabuli_wild_30148953	1399935348	*Ca_kabuli_Chr03*	30148953	A/G	NA	INTERGENIC	NA
Kabuli_wild_30206461	1399935349	*Ca_kabuli_Chr03*	30206461	T/C	Gene_Ca_07336	INTRON	Kinesin,motordomain
Kabuli_wild_30311541	1399935350	*Ca_kabuli_Chr03*	30311541	A/C	Gene_Ca_07347	UPSTREAM	NA
Kabuli_wild_30311582	1399935351	*Ca_kabuli_Chr03*	30311582	C/T	Gene_Ca_07347	UPSTREAM	NA
Kabuli_wild_30311715	1399935352	*Ca_kabuli_Chr03*	30311715	A/G	Gene_Ca_07347	UPSTREAM	NA
Kabuli_wild_30321177	1399935353	*Ca_kabuli_Chr03*	30321177	C/G	Gene_Ca_07349	DOWNSTREAM	BSD
Kabuli_wild_30376319	1399935354	*Ca_kabuli_Chr03*	30376319	G/A	Gene_Ca_07358	SYNONYMOUS_CODING	Short-chaindehydrogenase/reductaseSDR
Kabuli_wild_30383534	1399935355	*Ca_kabuli_Chr03*	30383534	T/C	Gene_Ca_07359	SYNONYMOUS_CODING	NA
Kabuli_wild_30385153	1399935356	*Ca_kabuli_Chr03*	30385153	T/A	Gene_Ca_07359	INTRON	NA
Kabuli_wild_30397261	1399935357	*Ca_kabuli_Chr03*	30397261	C/A	Gene_Ca_07359	INTRON	NA
Kabuli_wild_30397262	1399935358	*Ca_kabuli_Chr03*	30397262	A/T	Gene_Ca_07359	INTRON	NA
Kabuli_wild_30397271	1399935359	*Ca_kabuli_Chr03*	30397271	C/T	Gene_Ca_07359	INTRON	NA
Kabuli_wild_30397293	1399935360	*Ca_kabuli_Chr03*	30397293	C/T	Gene_Ca_07359	INTRON	NA
Kabuli_wild_30510333	1399935361	*Ca_kabuli_Chr03*	30510333	C/T	Gene_Ca_07371	UPSTREAM	NA
Kabuli_wild_30525213	1399935362	*Ca_kabuli_Chr03*	30525213	A/C	Gene_Ca_07374	SYNONYMOUS_CODING	RasGTPase
Kabuli_wild_30614369	1399935363	*Ca_kabuli_Chr03*	30614369	T/C	NA	INTERGENIC	NA
Kabuli_wild_30675468	1399935364	*Ca_kabuli_Chr03*	30675468	T/G	Gene_Ca_07388	DOWNSTREAM	Helicase,C-terminal
Kabuli_wild_30837265	1399935365	*Ca_kabuli_Chr03*	30837265	T/C	NA	INTERGENIC	NA
Kabuli_wild_30837284	1399935366	*Ca_kabuli_Chr03*	30837284	C/T	NA	INTERGENIC	NA
Kabuli_wild_31091987	1399935367	*Ca_kabuli_Chr03*	31091987	A/C	Gene_Ca_12206	SYNONYMOUS_CODING	HistoneH1/H5

SNP IDs	NCBI dbSNP accession IDs (NCBI_SS#)	Chromosomes	Physical positions (bp)	SNPs	Gene accession IDs	Sequence components of genome	Putative functions
Kabuli_wild_31267639	1399935368	*Ca_kabuli_Chr03*	31267639	T/A	NA	INTERGENIC	NA
Kabuli_wild_31267675	1399935369	*Ca_kabuli_Chr03*	31267675	A/G	NA	INTERGENIC	NA
Kabuli_wild_31267681	1399935370	*Ca_kabuli_Chr03*	31267681	G/A	NA	INTERGENIC	NA
Kabuli_wild_31267828	1399935371	*Ca_kabuli_Chr03*	31267828	A/G	NA	INTERGENIC	NA
Kabuli_wild_31267927	1399935372	*Ca_kabuli_Chr03*	31267927	T/G	NA	INTERGENIC	NA
Kabuli_wild_31268143	1399935373	*Ca_kabuli_Chr03*	31268143	A/G	NA	INTERGENIC	NA
Kabuli_wild_31271040	1399935374	*Ca_kabuli_Chr03*	31271040	T/C	NA	INTERGENIC	NA
Kabuli_wild_31279605	1399935375	*Ca_kabuli_Chr03*	31279605	G/A	Gene_Ca_12224	INTRON	Protein of unknown function DUF647
Kabuli_wild_31279606	1399935376	*Ca_kabuli_Chr03*	31279606	G/A	Gene_Ca_12224	INTRON	Protein of unknown function DUF647
Kabuli_wild_31279616	1399935377	*Ca_kabuli_Chr03*	31279616	T/A	Gene_Ca_12224	INTRON	Protein of unknown function DUF647
Kabuli_wild_31287909	1399935378	*Ca_kabuli_Chr03*	31287909	A/G	Gene_Ca_12225	SYNONYMOUS_CODING	General substrate transporter
Kabuli_wild_31288008	1399935379	*Ca_kabuli_Chr03*	31288008	T/G	Gene_Ca_12225	SYNONYMOUS_CODING	General substrate transporter
Kabuli_wild_31288143	1399935380	*Ca_kabuli_Chr03*	31288143	T/C	Gene_Ca_12225	SYNONYMOUS_CODING	General substrate transporter
Kabuli_wild_31301783	1399935381	*Ca_kabuli_Chr03*	31301783	A/G	NA	INTERGENIC	NA
Kabuli_wild_31302060	1399935382	*Ca_kabuli_Chr03*	31302060	T/C	NA	INTERGENIC	NA
Kabuli_wild_31313021	1399935383	*Ca_kabuli_Chr03*	31313021	G/T	NA	INTERGENIC	NA
Kabuli_wild_31317521	1399935384	*Ca_kabuli_Chr03*	31317521	T/G	NA	INTERGENIC	NA
Kabuli_wild_31321725	1399935385	*Ca_kabuli_Chr03*	31321725	A/G	NA	INTERGENIC	NA
Kabuli_wild_31321778	1399935386	*Ca_kabuli_Chr03*	31321778	G/A	NA	INTERGENIC	NA
Kabuli_wild_31321850	1399935387	*Ca_kabuli_Chr03*	31321850	A/G	NA	INTERGENIC	NA
Kabuli_wild_31341335	1399935388	*Ca_kabuli_Chr03*	31341335	C/T	Gene_Ca_12234	SYNONYMOUS_CODING	Domain of unknown function DUF640
Kabuli_wild_31341386	1399935389	*Ca_kabuli_Chr03*	31341386	A/C	Gene_Ca_12234	SYNONYMOUS_CODING	Domain of unknown function DUF640
Kabuli_wild_31342119	1399935390	*Ca_kabuli_Chr03*	31342119	A/T	NA	INTERGENIC	NA
Kabuli_wild_31354988	1399935391	*Ca_kabuli_Chr03*	31354988	C/G	NA	INTERGENIC	NA
Kabuli_wild_31355001	1399935392	*Ca_kabuli_Chr03*	31355001	T/C	NA	INTERGENIC	NA
Kabuli_wild_31355026	1399935393	*Ca_kabuli_Chr03*	31355026	C/T	NA	INTERGENIC	NA
Kabuli_wild_31357799	1399935394	*Ca_kabuli_Chr03*	31357799	T/C	Gene_Ca_12236	SYNONYMOUS_CODING	Transcription factor jumonji/aspartylbeta-hydroxylase
Kabuli_wild_31358024	1399935395	*Ca_kabuli_Chr03*	31358024	C/T	Gene_Ca_12236	SYNONYMOUS_CODING	Transcription factor jumonji/aspartylbeta-hydroxylase
Kabuli_wild_31358137	1399935396	*Ca_kabuli_Chr03*	31358137	G/T	Gene_Ca_12236	NON_SYNONYMOUS_CODING	Transcription factor jumonji/aspartylbeta-hydroxylase
Kabuli_wild_31431216	1399935397	*Ca_kabuli_Chr03*	31431216	C/T	Gene_Ca_12240	INTRON	Protein kinase,catalytic domain
Kabuli_wild_31431297	1399935398	*Ca_kabuli_Chr03*	31431297	C/T	Gene_Ca_12240	INTRON	Protein kinase,catalytic domain
Kabuli_wild_31457191	1399935399	*Ca_kabuli_Chr03*	31457191	C/T	Gene_Ca_12244	SYNONYMOUS_CODING	Phytochelatinsynthase
Kabuli_wild_31478804	1399935400	*Ca_kabuli_Chr03*	31478804	T/C	Gene_Ca_12246	SYNONYMOUS_CODING	Glucose-methanol-cholineoxidoreductase,N-terminal
Kabuli_wild_31478882	1399935401	*Ca_kabuli_Chr03*	31478882	C/T	Gene_Ca_12246	SYNONYMOUS_CODING	Glucose-methanol-cholineoxidoreductase,N-terminal
Kabuli_wild_31480603	1399935402	*Ca_kabuli_Chr03*	31480603	T/C	NA	INTERGENIC	NA
Kabuli_wild_31516662	1399935403	*Ca_kabuli_Chr03*	31516662	G/T	NA	INTERGENIC	NA
Kabuli_wild_31516766	1399935404	*Ca_kabuli_Chr03*	31516766	T/C	NA	INTERGENIC	NA
Kabuli_wild_31517136	1399935405	*Ca_kabuli_Chr03*	31517136	A/G	NA	INTERGENIC	NA
Kabuli_wild_31517201	1399935406	*Ca_kabuli_Chr03*	31517201	G/A	NA	INTERGENIC	NA
Kabuli_wild_31519880	1399935407	*Ca_kabuli_Chr03*	31519880	C/T	Gene_Ca_12250	UPSTREAM	Oligo peptide transporter
Kabuli_wild_31520119	1399935408	*Ca_kabuli_Chr03*	31520119	C/T	Gene_Ca_12250	UPSTREAM	Oligo peptide transporter
Kabuli_wild_31520190	1399935409	*Ca_kabuli_Chr03*	31520190	T/C	Gene_Ca_12250	UPSTREAM	Oligo peptide transporter
Kabuli_wild_31520198	1399935410	*Ca_kabuli_Chr03*	31520198	G/A	NA	INTERGENIC	NA
Kabuli_wild_31520225	1399935411	*Ca_kabuli_Chr03*	31520225	A/T	NA	INTERGENIC	NA

SNP IDs	NCBI dbSNP accession IDs (NCBI_SS#)	Chromosomes	Physical positions (bp)	SNPs	Gene accession IDs	Sequence components of genome	Putative functions
Kabuli_wild_31521142	1399935412	*Ca_kabuli_Chr03*	31521142	T/C	NA	INTERGENIC	NA
Kabuli_wild_31521155	1399935413	*Ca_kabuli_Chr03*	31521155	T/A	NA	INTERGENIC	NA
Kabuli_wild_31589329	1399935414	*Ca_kabuli_Chr03*	31589329	C/T	Gene_Ca_12258	INTRON	SET domain
Kabuli_wild_31589355	1399935415	*Ca_kabuli_Chr03*	31589355	A/G	Gene_Ca_12258	INTRON	SET domain
Kabuli_wild_31626736	1399935416	*Ca_kabuli_Chr03*	31626736	C/T	Gene_Ca_12261	INTRON	RNA-processing protein, HAThelix
Kabuli_wild_31630195	1399935417	*Ca_kabuli_Chr03*	31630195	T/C	Gene_Ca_12262	INTRON	RNA recognition motif domain
Kabuli_wild_31630512	1399935418	*Ca_kabuli_Chr03*	31630512	T/A	Gene_Ca_12262	INTRON	RNA recognition motif domain
Kabuli_wild_31631451	1399935419	*Ca_kabuli_Chr03*	31631451	A/C	Gene_Ca_12262	NON_SYNONYMOUS_CODING	RNA recognition motif domain
Kabuli_wild_31631534	1399935420	*Ca_kabuli_Chr03*	31631534	A/G	Gene_Ca_12262	SYNONYMOUS_CODING	RNA recognition motif domain
Kabuli_wild_31631561	1399935421	*Ca_kabuli_Chr03*	31631561	G/A	Gene_Ca_12262	SYNONYMOUS_CODING	RNA recognition motif domain
Kabuli_wild_31631622	1399935422	*Ca_kabuli_Chr03*	31631622	G/A	Gene_Ca_12262	INTRON	RNA recognition motif domain
Kabuli_wild_31631644	1399935423	*Ca_kabuli_Chr03*	31631644	A/C	Gene_Ca_12262	INTRON	RNA recognition motif domain
Kabuli_wild_31646802	1399935424	*Ca_kabuli_Chr03*	31646802	G/A	NA	INTERGENIC	NA
Kabuli_wild_31683906	1399935425	*Ca_kabuli_Chr03*	31683906	A/G	Gene_Ca_12270	NON_SYNONYMOUS_CODING	PeptidaseS8/S53,subtilisin/kexin/sedolisin
Kabuli_wild_31689128	1399935426	*Ca_kabuli_Chr03*	31689128	C/T	NA	INTERGENIC	NA
Kabuli_wild_31698493	1399935427	*Ca_kabuli_Chr03*	31698493	A/C	Gene_Ca_12272	INTRON	ABC transporter,trans membrane domain
Kabuli_wild_31771314	1399935428	*Ca_kabuli_Chr03*	31771314	C/A	NA	INTERGENIC	NA
Kabuli_wild_31814796	1399935429	*Ca_kabuli_Chr03*	31814796	T/A	Gene_Ca_12284	SYNONYMOUS_CODING	WD40 repeat
Kabuli_wild_31931014	1399935430	*Ca_kabuli_Chr03*	31931014	A/C	Gene_Ca_12294	SYNONYMOUS_CODING	NA
Kabuli_wild_31951994	1399935431	*Ca_kabuli_Chr03*	31951994	G/A	Gene_Ca_12295	SYNONYMOUS_CODING	HEAT
Kabuli_wild_31952316	1399935432	*Ca_kabuli_Chr03*	31952316	T/G	Gene_Ca_12295	SYNONYMOUS_CODING	HEAT
Kabuli_wild_31952326	1399935433	*Ca_kabuli_Chr03*	31952326	C/A	Gene_Ca_12295	NON_SYNONYMOUS_CODING	HEAT
Kabuli_wild_31952330	1399935434	*Ca_kabuli_Chr03*	31952330	T/C	Gene_Ca_12295	SYNONYMOUS_CODING	HEAT
Kabuli_wild_31952333	1399935435	*Ca_kabuli_Chr03*	31952333	T/G	Gene_Ca_12295	SYNONYMOUS_CODING	HEAT
Kabuli_wild_31952348	1399935436	*Ca_kabuli_Chr03*	31952348	T/A	Gene_Ca_12295	NON_SYNONYMOUS_CODING	HEAT
Kabuli_wild_31952354	1399935437	*Ca_kabuli_Chr03*	31952354	G/A	Gene_Ca_12295	SYNONYMOUS_CODING	HEAT
Kabuli_wild_31952361	1399935438	*Ca_kabuli_Chr03*	31952361	G/A	Gene_Ca_12295	NON_SYNONYMOUS_CODING	HEAT
Kabuli_wild_31952364	1399935439	*Ca_kabuli_Chr03*	31952364	G/A	Gene_Ca_12295	NON_SYNONYMOUS_CODING	HEAT
Kabuli_wild_31953466	1399935440	*Ca_kabuli_Chr03*	31953466	G/A	Gene_Ca_12295	INTRON	HEAT
Kabuli_wild_31953517	1399935441	*Ca_kabuli_Chr03*	31953517	A/G	Gene_Ca_12295	INTRON	HEAT
Kabuli_wild_32351031	1399935442	*Ca_kabuli_Chr03*	32351031	G/C	Gene_Ca_11958	SYNONYMOUS_CODING	Heat shock protein Hsp70
Kabuli_wild_32351052	1399935443	*Ca_kabuli_Chr03*	32351052	C/T	Gene_Ca_11958	SYNONYMOUS_CODING	Heat shock protein Hsp70
Kabuli_wild_32357578	1399935444	*Ca_kabuli_Chr03*	32357578	G/C	Gene_Ca_11959	SYNONYMOUS_CODING	Heat shock protein Hsp70
Kabuli_wild_32357599	1399935445	*Ca_kabuli_Chr03*	32357599	C/T	Gene_Ca_11959	SYNONYMOUS_CODING	Heat shock protein Hsp70
Kabuli_wild_32357604	1399935446	*Ca_kabuli_Chr03*	32357604	T/A	Gene_Ca_11959	NON_SYNONYMOUS_CODING	Heat shock protein Hsp70
Kabuli_wild_32466597	1399935447	*Ca_kabuli_Chr03*	32466597	A/G	Gene_Ca_11969	INTRON	Proteasome component (PCI) domain
Kabuli_wild_32473017	1399935448	*Ca_kabuli_Chr03*	32473017	A/T	Gene_Ca_11970	DOWNSTREAM	RNA recognition motif domain
Kabuli_wild_32476774	1399935449	*Ca_kabuli_Chr03*	32476774	A/G	NA	INTERGENIC	NA
Kabuli_wild_32510033	1399935450	*Ca_kabuli_Chr03*	32510033	T/G	NA	INTERGENIC	NA
Kabuli_wild_32510071	1399935451	*Ca_kabuli_Chr03*	32510071	G/T	NA	INTERGENIC	NA
Kabuli_wild_32510081	1399935452	*Ca_kabuli_Chr03*	32510081	C/T	NA	INTERGENIC	NA
Kabuli_wild_32510090	1399935453	*Ca_kabuli_Chr03*	32510090	A/G	NA	INTERGENIC	NA
Kabuli_wild_32510101	1399935454	*Ca_kabuli_Chr03*	32510101	G/A	NA	INTERGENIC	NA
Kabuli_wild_32510111	1399935455	*Ca_kabuli_Chr03*	32510111	C/T	NA	INTERGENIC	NA

SNP IDs	NCBI dbSNP accession IDs (NCBI_SS#)	Chromosomes	Physical positions (bp)	SNPs	Gene accession IDs	Sequence components of genome	Putative functions
Kabuli_wild_32510124	1399935456	*Ca_kabuli_Chr03*	32510124	C/T	NA	INTERGENIC	NA
Kabuli_wild_32514859	1399935457	*Ca_kabuli_Chr03*	32514859	C/T	NA	INTERGENIC	NA
Kabuli_wild_32514867	1399935458	*Ca_kabuli_Chr03*	32514867	C/T	NA	INTERGENIC	NA
Kabuli_wild_32514868	1399935459	*Ca_kabuli_Chr03*	32514868	A/G	NA	INTERGENIC	NA
Kabuli_wild_32514883	1399935460	*Ca_kabuli_Chr03*	32514883	A/G	NA	INTERGENIC	NA
Kabuli_wild_32514889	1399935461	*Ca_kabuli_Chr03*	32514889	C/T	NA	INTERGENIC	NA
Kabuli_wild_32514908	1399935462	*Ca_kabuli_Chr03*	32514908	A/G	NA	INTERGENIC	NA
Kabuli_wild_32525128	1399935463	*Ca_kabuli_Chr03*	32525128	G/T	Gene_Ca_11978	NON_SYNONYMOUS_CODING	RasGTPase
Kabuli_wild_32535281	1399935464	*Ca_kabuli_Chr03*	32535281	A/G	NA	INTERGENIC	NA
Kabuli_wild_32663059	1399935465	*Ca_kabuli_Chr03*	32663059	A/C	Gene_Ca_11994	NON_SYNONYMOUS_CODING	RNA recognition motif domain
Kabuli_wild_32743093	1399935466	*Ca_kabuli_Chr03*	32743093	C/T	NA	INTERGENIC	NA
Kabuli_wild_32757632	1399935467	*Ca_kabuli_Chr03*	32757632	A/T	NA	INTERGENIC	NA
Kabuli_wild_32785350	1399935468	*Ca_kabuli_Chr03*	32785350	T/G	Gene_Ca_12012	NON_SYNONYMOUS_CODING	Protein kinase,catalytic domain
Kabuli_wild_32810787	1399935469	*Ca_kabuli_Chr03*	32810787	T/C	NA	INTERGENIC	NA
Kabuli_wild_32827062	1399935470	*Ca_kabuli_Chr03*	32827062	A/G	NA	INTERGENIC	NA
Kabuli_wild_32827065	1399935471	*Ca_kabuli_Chr03*	32827065	T/G	NA	INTERGENIC	NA
Kabuli_wild_32827067	1399935472	*Ca_kabuli_Chr03*	32827067	G/C	NA	INTERGENIC	NA
Kabuli_wild_32827069	1399935473	*Ca_kabuli_Chr03*	32827069	G/C	NA	INTERGENIC	NA
Kabuli_wild_32845395	1399935474	*Ca_kabuli_Chr03*	32845395	C/A	NA	INTERGENIC	NA
Kabuli_wild_32865530	1399935475	*Ca_kabuli_Chr03*	32865530	C/G	Gene_Ca_12023	INTRON	Helicase,C-terminal
Kabuli_wild_32949471	1399935476	*Ca_kabuli_Chr03*	32949471	A/G	NA	INTERGENIC	NA
Kabuli_wild_32965908	1399935477	*Ca_kabuli_Chr03*	32965908	C/T	Gene_Ca_12035	INTRON	WD40 repeat
Kabuli_wild_33056117	1399935478	*Ca_kabuli_Chr03*	33056117	T/G	Gene_Ca_12043	NON_SYNONYMOUS_CODING	Natural resistance-associated macrophage protein
Kabuli_wild_33080350	1399935479	*Ca_kabuli_Chr03*	33080350	C/T	NA	INTERGENIC	NA
Kabuli_wild_33091737	1399935480	*Ca_kabuli_Chr03*	33091737	A/C	Gene_Ca_12046	INTRON	SNF2-related
Kabuli_wild_33120825	1399935481	*Ca_kabuli_Chr03*	33120825	G/A	NA	INTERGENIC	NA
Kabuli_wild_33492669	1399935482	*Ca_kabuli_Chr03*	33492669	A/G	Gene_Ca_23669	INTRON	NA
Kabuli_wild_33520560	1399935483	*Ca_kabuli_Chr03*	33520560	A/G	Gene_Ca_23669	NON_SYNONYMOUS_CODING	NA
Kabuli_wild_33520615	1399935484	*Ca_kabuli_Chr03*	33520615	A/C	Gene_Ca_23669	NON_SYNONYMOUS_CODING	NA
Kabuli_wild_33522974	1399935485	*Ca_kabuli_Chr03*	33522974	G/A	Gene_Ca_23669	INTRON	NA
Kabuli_wild_33621317	1399935486	*Ca_kabuli_Chr03*	33621317	G/A	Gene_Ca_23664	INTRON	NA
Kabuli_wild_33627826	1399935487	*Ca_kabuli_Chr03*	33627826	A/C	NA	INTERGENIC	NA
Kabuli_wild_33715215	1399935488	*Ca_kabuli_Chr03*	33715215	G/T	Gene_Ca_20869	INTRON	Alpha/betahydrolasefold-1
Kabuli_wild_33900522	1399935489	*Ca_kabuli_Chr03*	33900522	A/G	Gene_Ca_20884	INTRON	ABC transporter,trans membrane domain
Kabuli_wild_33904394	1399935490	*Ca_kabuli_Chr03*	33904394	T/A	Gene_Ca_20884	DOWNSTREAM	ABC transporter,trans membrane domain
Kabuli_wild_33904409	1399935491	*Ca_kabuli_Chr03*	33904409	C/T	Gene_Ca_20884	DOWNSTREAM	ABC transporter,trans membrane domain
Kabuli_wild_34167672	1399935492	*Ca_kabuli_Chr03*	34167672	G/T	Gene_Ca_20896	NON_SYNONYMOUS_CODING	NA
Kabuli_wild_34167695	1399935493	*Ca_kabuli_Chr03*	34167695	A/C	Gene_Ca_20896	SYNONYMOUS_CODING	NA
Kabuli_wild_34247736	1399935494	*Ca_kabuli_Chr03*	34247736	T/G	Gene_Ca_00702	NON_SYNONYMOUS_CODING	ABC transporter,trans membrane domain
Kabuli_wild_34352385	1399935495	*Ca_kabuli_Chr03*	34352385	A/C	NA	INTERGENIC	NA
Kabuli_wild_34496982	1399935496	*Ca_kabuli_Chr03*	34496982	G/C	Gene_Ca_00725	SYNONYMOUS_CODING	WD40 repeat
Kabuli_wild_34524656	1399935497	*Ca_kabuli_Chr03*	34524656	C/A	Gene_Ca_00726	NON_SYNONYMOUS_CODING	NA
Kabuli_wild_34543039	1399935498	*Ca_kabuli_Chr03*	34543039	C/T	Gene_Ca_00726	NON_SYNONYMOUS_CODING	NA
Kabuli_wild_34543111	1399935499	*Ca_kabuli_Chr03*	34543111	A/G	Gene_Ca_00726	NON_SYNONYMOUS_CODING	NA

SNP IDs	NCBI dbSNP accession IDs (NCBI_SS#)	Chromosomes	Physical positions (bp)	SNPs	Gene accession IDs	Sequence components of genome	Putative functions
Kabuli_wild_34547052	1399935500	*Ca_kabuli_Chr03*	34547052	A/T	Gene_Ca_00726	NON_SYNONYMOUS_CODING	NA
Kabuli_wild_34547069	1399935501	*Ca_kabuli_Chr03*	34547069	G/T	Gene_Ca_00726	NON_SYNONYMOUS_CODING	NA
Kabuli_wild_34603669	1399935502	*Ca_kabuli_Chr03*	34603669	T/A	Gene_Ca_00732	NON_SYNONYMOUS_CODING	Zinc finger,PHD-type
Kabuli_wild_34662736	1399935503	*Ca_kabuli_Chr03*	34662736	T/G	Gene_Ca_00740	INTRON	RNA recognition motif domain
Kabuli_wild_34699073	1399935504	*Ca_kabuli_Chr03*	34699073	C/A	Gene_Ca_00745	NON_SYNONYMOUS_CODING	HECT
Kabuli_wild_34699076	1399935505	*Ca_kabuli_Chr03*	34699076	T/C	Gene_Ca_00745	NON_SYNONYMOUS_CODING	HECT
Kabuli_wild_34699114	1399935506	*Ca_kabuli_Chr03*	34699114	C/A	Gene_Ca_00745	NON_SYNONYMOUS_CODING	HECT
Kabuli_wild_34722508	1399935507	*Ca_kabuli_Chr03*	34722508	A/T	Gene_Ca_00746	DOWNSTREAM	Exostosin-like
Kabuli_wild_34722931	1399935508	*Ca_kabuli_Chr03*	34722931	A/G	NA	INTERGENIC	NA
Kabuli_wild_34722979	1399935509	*Ca_kabuli_Chr03*	34722979	T/C	NA	INTERGENIC	NA
Kabuli_wild_34730463	1399935510	*Ca_kabuli_Chr03*	34730463	T/C	Gene_Ca_00748	INTRON	Protein of unknown function DUF81
Kabuli_wild_34730954	1399935511	*Ca_kabuli_Chr03*	34730954	G/A	Gene_Ca_00748	DOWNSTREAM	Protein of unknown function DUF81
Kabuli_wild_34736963	1399935512	*Ca_kabuli_Chr03*	34736963	T/C	Gene_Ca_00749	NON_SYNONYMOUS_CODING	RibosomalproteinL24e
Kabuli_wild_34757571	1399935513	*Ca_kabuli_Chr03*	34757571	T/C	Gene_Ca_00751	INTRON	F-boxdomain,cyclin-like
Kabuli_wild_34757578	1399935514	*Ca_kabuli_Chr03*	34757578	T/C	Gene_Ca_00751	INTRON	F-boxdomain,cyclin-like
Kabuli_wild_34852030	1399935515	*Ca_kabuli_Chr03*	34852030	G/A	NA	INTERGENIC	NA
Kabuli_wild_34852070	1399935516	*Ca_kabuli_Chr03*	34852070	T/G	NA	INTERGENIC	NA
Kabuli_wild_34889316	1399935517	*Ca_kabuli_Chr03*	34889316	C/G	Gene_Ca_00767	SYNONYMOUS_CODING	Fructose-bisphosphatealdolase,class-I
Kabuli_wild_34911621	1399935518	*Ca_kabuli_Chr03*	34911621	G/A	Gene_Ca_00768	SYNONYMOUS_CODING	Pathogenesis-related transcriptional factor/ERF, DNA-binding
Kabuli_wild_34982544	1399935519	*Ca_kabuli_Chr03*	34982544	T/C	Gene_Ca_00773	INTRON	Glycosylhydrolase,family13,catalytic domain
Kabuli_wild_35071821	1399935520	*Ca_kabuli_Chr03*	35071821	A/G	Gene_Ca_00779	INTRON	Protein kinase,catalytic domain
Kabuli_wild_35071852	1399935521	*Ca_kabuli_Chr03*	35071852	T/G	Gene_Ca_00779	INTRON	Protein kinase,catalytic domain
Kabuli_wild_35085559	1399935522	*Ca_kabuli_Chr03*	35085559	T/A	Gene_Ca_00781	SYNONYMOUS_CODING	PeptidaseS8/S53,subtilisin/kexin/sedolisin
Kabuli_wild_35085658	1399935523	*Ca_kabuli_Chr03*	35085658	C/A	Gene_Ca_00781	SYNONYMOUS_CODING	PeptidaseS8/S53,subtilisin/kexin/sedolisin
Kabuli_wild_35126649	1399935524	*Ca_kabuli_Chr03*	35126649	C/G	Gene_Ca_00786	NON_SYNONYMOUS_CODING	NA
Kabuli_wild_35126760	1399935525	*Ca_kabuli_Chr03*	35126760	G/A	Gene_Ca_00786	NON_SYNONYMOUS_CODING	NA
Kabuli_wild_35241163	1399935526	*Ca_kabuli_Chr03*	35241163	G/A	NA	INTERGENIC	NA
Kabuli_wild_35332088	1399935527	*Ca_kabuli_Chr03*	35332088	T/A	Gene_Ca_00812	NON_SYNONYMOUS_CODING	Kinesin,motordomain
Kabuli_wild_35345385	1399935528	*Ca_kabuli_Chr03*	35345385	A/T	Gene_Ca_00814	NON_SYNONYMOUS_CODING	Oxoglutarate/iron-dependent oxygenase
Kabuli_wild_35383318	1399935529	*Ca_kabuli_Chr03*	35383318	C/T	Gene_Ca_00819	INTRON	Peptidyl-prolylcis-transisomerase,cyclophilin-type
Kabuli_wild_35403074	1399935530	*Ca_kabuli_Chr03*	35403074	A/G	NA	INTERGENIC	NA
Kabuli_wild_35420099	1399935531	*Ca_kabuli_Chr03*	35420099	A/G	Gene_Ca_00824	INTRON	LETM1-like
Kabuli_wild_35420108	1399935532	*Ca_kabuli_Chr03*	35420108	T/A	Gene_Ca_00824	INTRON	LETM1-like
Kabuli_wild_35424877	1399935533	*Ca_kabuli_Chr03*	35424877	C/T	NA	INTERGENIC	NA
Kabuli_wild_35424914	1399935534	*Ca_kabuli_Chr03*	35424914	G/C	NA	INTERGENIC	NA
Kabuli_wild_35424994	1399935535	*Ca_kabuli_Chr03*	35424994	T/C	NA	INTERGENIC	NA
Kabuli_wild_35483932	1399935536	*Ca_kabuli_Chr03*	35483932	T/C	NA	INTERGENIC	NA
Kabuli_wild_35483960	1399935537	*Ca_kabuli_Chr03*	35483960	T/C	NA	INTERGENIC	NA
Kabuli_wild_35484034	1399935538	*Ca_kabuli_Chr03*	35484034	G/C	NA	INTERGENIC	NA
Kabuli_wild_35487736	1399935539	*Ca_kabuli_Chr03*	35487736	T/C	Gene_Ca_00831	UPSTREAM	Zinc finger,MYND-type
Kabuli_wild_35488597	1399935540	*Ca_kabuli_Chr03*	35488597	T/C	NA	INTERGENIC	NA
Kabuli_wild_35490951	1399935541	*Ca_kabuli_Chr03*	35490951	C/T	NA	INTERGENIC	NA
Kabuli_wild_35490991	1399935542	*Ca_kabuli_Chr03*	35490991	A/G	NA	INTERGENIC	NA
Kabuli_wild_35543614	1399935543	*Ca_kabuli_Chr03*	35543614	C/T	NA	INTERGENIC	NA

SNP IDs	NCBI dbSNP accession IDs (NCBI_SS#)	Chromosomes	Physical positions (bp)	SNPs	Gene accession IDs	Sequence components of genome	Putative functions
Kabuli_wild_35631679	1399935544	*Ca_kabuli_Chr03*	35631679	C/G	NA	INTERGENIC	NA
Kabuli_wild_35631689	1399935545	*Ca_kabuli_Chr03*	35631689	C/A	NA	INTERGENIC	NA
Kabuli_wild_35631723	1399935546	*Ca_kabuli_Chr03*	35631723	A/G	NA	INTERGENIC	NA
Kabuli_wild_35653816	1399935547	*Ca_kabuli_Chr03*	35653816	T/G	Gene_Ca_00853	SYNONYMOUS_CODING	NA
Kabuli_wild_35673890	1399935548	*Ca_kabuli_Chr03*	35673890	T/C	Gene_Ca_00855	INTRON	Glycosyltransferase,group1
Kabuli_wild_35747394	1399935549	*Ca_kabuli_Chr03*	35747394	C/G	NA	INTERGENIC	NA
Kabuli_wild_35763648	1399935550	*Ca_kabuli_Chr03*	35763648	T/C	Gene_Ca_00862	INTRON	NA
Kabuli_wild_35807265	1399935551	*Ca_kabuli_Chr03*	35807265	T/C	NA	INTERGENIC	NA
Kabuli_wild_35807407	1399935552	*Ca_kabuli_Chr03*	35807407	A/T	NA	INTERGENIC	NA
Kabuli_wild_35829834	1399935553	*Ca_kabuli_Chr03*	35829834	A/G	NA	INTERGENIC	NA
Kabuli_wild_35876164	1399935554	*Ca_kabuli_Chr03*	35876164	T/A	NA	INTERGENIC	NA
Kabuli_wild_35876175	1399935555	*Ca_kabuli_Chr03*	35876175	A/G	NA	INTERGENIC	NA
Kabuli_wild_35898221	1399935556	*Ca_kabuli_Chr03*	35898221	C/A	NA	INTERGENIC	NA
Kabuli_wild_35920684	1399935557	*Ca_kabuli_Chr03*	35920684	A/C	NA	INTERGENIC	NA
Kabuli_wild_35948487	1399935558	*Ca_kabuli_Chr03*	35948487	G/A	Gene_Ca_00889	DOWNSTREAM	RibosomalproteinL35A
Kabuli_wild_35979293	1399935559	*Ca_kabuli_Chr03*	35979293	A/G	Gene_Ca_00892	INTRON	FAD-linkedoxidase,C-terminal
Kabuli_wild_35979412	1399935560	*Ca_kabuli_Chr03*	35979412	G/T	Gene_Ca_00892	SYNONYMOUS_CODING	FAD-linkedoxidase,C-terminal
Kabuli_wild_35991024	1399935561	*Ca_kabuli_Chr03*	35991024	A/G	NA	INTERGENIC	NA
Kabuli_wild_36003682	1399935562	*Ca_kabuli_Chr03*	36003682	G/T	Gene_Ca_00896	INTRON	Protease-associateddomain,PA
Kabuli_wild_36003699	1399935563	*Ca_kabuli_Chr03*	36003699	G/A	Gene_Ca_00896	INTRON	Protease-associateddomain,PA
Kabuli_wild_36004668	1399935564	*Ca_kabuli_Chr03*	36004668	A/G	Gene_Ca_00896	INTRON	Protease-associateddomain,PA
Kabuli_wild_36010614	1399935565	*Ca_kabuli_Chr03*	36010614	T/C	NA	INTERGENIC	NA
Kabuli_wild_36034934	1399935566	*Ca_kabuli_Chr03*	36034934	A/C	Gene_Ca_00902	INTRON	Ubiquitin-associated/translation elongation factor EF1B, N-terminal
Kabuli_wild_36046938	1399935567	*Ca_kabuli_Chr03*	36046938	C/T	Gene_Ca_00903	SYNONYMOUS_CODING	SWAP/Surp
Kabuli_wild_36046956	1399935568	*Ca_kabuli_Chr03*	36046956	A/C	Gene_Ca_00903	SYNONYMOUS_CODING	SWAP/Surp
Kabuli_wild_36050259	1399935569	*Ca_kabuli_Chr03*	36050259	T/G	Gene_Ca_00903	SYNONYMOUS_CODING	SWAP/Surp
Kabuli_wild_36050319	1399935570	*Ca_kabuli_Chr03*	36050319	T/C	Gene_Ca_00903	SYNONYMOUS_CODING	SWAP/Surp
Kabuli_wild_36071033	1399935571	*Ca_kabuli_Chr03*	36071033	G/A	Gene_Ca_00904	DOWNSTREAM	Cleavage/polyadenylation specificity factor, A subunit, C-terminalr
Kabuli_wild_36085966	1399935572	*Ca_kabuli_Chr03*	36085966	G/A	NA	INTERGENIC	NA
Kabuli_wild_36117521	1399935573	*Ca_kabuli_Chr03*	36117521	A/G	Gene_Ca_00911	SYNONYMOUS_CODING	NA
Kabuli_wild_36117734	1399935574	*Ca_kabuli_Chr03*	36117734	T/C	Gene_Ca_00911	INTRON	NA
Kabuli_wild_36118120	1399935575	*Ca_kabuli_Chr03*	36118120	T/C	Gene_Ca_00911	INTRON	NA
Kabuli_wild_36118156	1399935576	*Ca_kabuli_Chr03*	36118156	C/T	Gene_Ca_00911	INTRON	NA
Kabuli_wild_36119592	1399935577	*Ca_kabuli_Chr03*	36119592	T/C	Gene_Ca_00911	NON_SYNONYMOUS_CODING	NA
Kabuli_wild_36119602	1399935578	*Ca_kabuli_Chr03*	36119602	C/A	Gene_Ca_00911	NON_SYNONYMOUS_CODING	NA
Kabuli_wild_36130561	1399935579	*Ca_kabuli_Chr03*	36130561	T/A	NA	INTERGENIC	NA
Kabuli_wild_36130610	1399935580	*Ca_kabuli_Chr03*	36130610	C/A	NA	INTERGENIC	NA
Kabuli_wild_36132401	1399935581	*Ca_kabuli_Chr03*	36132401	C/T	Gene_Ca_00912	INTRON	NPL4
Kabuli_wild_36132437	1399935582	*Ca_kabuli_Chr03*	36132437	C/T	Gene_Ca_00912	INTRON	NPL4
Kabuli_wild_36132440	1399935583	*Ca_kabuli_Chr03*	36132440	T/C	Gene_Ca_00912	INTRON	NPL4
Kabuli_wild_36132480	1399935584	*Ca_kabuli_Chr03*	36132480	C/T	Gene_Ca_00912	INTRON	NPL4
Kabuli_wild_36132539	1399935585	*Ca_kabuli_Chr03*	36132539	C/T	Gene_Ca_00912	INTRON	NPL4
Kabuli_wild_36132554	1399935586	*Ca_kabuli_Chr03*	36132554	A/T	Gene_Ca_00912	INTRON	NPL4
Kabuli_wild_36132570	1399935587	*Ca_kabuli_Chr03*	36132570	G/A	Gene_Ca_00912	INTRON	NPL4

SNP IDs	NCBI dbSNP accession IDs (NCBI_SS#)	Chromosomes	Physical positions (bp)	SNPs	Gene accession IDs	Sequence components of genome	Putative functions
Kabuli_wild_36132586	1399935588	*Ca_kabuli_Chr03*	36132586	T/G	Gene_Ca_00912	INTRON	NPL4
Kabuli_wild_36150717	1399935589	*Ca_kabuli_Chr03*	36150717	T/C	NA	INTERGENIC	NA
Kabuli_wild_36150722	1399935590	*Ca_kabuli_Chr03*	36150722	G/A	NA	INTERGENIC	NA
Kabuli_wild_36164925	1399935591	*Ca_kabuli_Chr03*	36164925	A/G	NA	INTERGENIC	NA
Kabuli_wild_36167136	1399935592	*Ca_kabuli_Chr03*	36167136	G/T	Gene_Ca_00918	DOWNSTREAM	BRCT
Kabuli_wild_36177201	1399935593	*Ca_kabuli_Chr03*	36177201	T/C	Gene_Ca_00919	DOWNSTREAM	Short-chain dehydrogenase/reductase SDR
Kabuli_wild_36205806	1399935594	*Ca_kabuli_Chr03*	36205806	T/G	NA	INTERGENIC	NA
Kabuli_wild_36205819	1399935595	*Ca_kabuli_Chr03*	36205819	A/G	NA	INTERGENIC	NA
Kabuli_wild_36205845	1399935596	*Ca_kabuli_Chr03*	36205845	A/G	NA	INTERGENIC	NA
Kabuli_wild_36206046	1399935597	*Ca_kabuli_Chr03*	36206046	T/C	NA	INTERGENIC	NA
Kabuli_wild_36206615	1399935598	*Ca_kabuli_Chr03*	36206615	A/C	Gene_Ca_00925	INTRON	rRNAprocessing
Kabuli_wild_36212809	1399935599	*Ca_kabuli_Chr03*	36212809	G/A	NA	INTERGENIC	NA
Kabuli_wild_36212843	1399935600	*Ca_kabuli_Chr03*	36212843	T/A	NA	INTERGENIC	NA
Kabuli_wild_36217686	1399935601	*Ca_kabuli_Chr03*	36217686	G/A	Gene_Ca_00927	INTRON	Zinc finger,B-box
Kabuli_wild_36227548	1399935602	*Ca_kabuli_Chr03*	36227548	G/T	Gene_Ca_00929	UPSTREAM	Ribosomal protein S19/S15
Kabuli_wild_36227565	1399935603	*Ca_kabuli_Chr03*	36227565	T/A	Gene_Ca_00929	UPSTREAM	Ribosomal protein S19/S15
Kabuli_wild_36227566	1399935604	*Ca_kabuli_Chr03*	36227566	T/A	Gene_Ca_00929	UPSTREAM	Ribosomal protein S19/S15
Kabuli_wild_36227567	1399935605	*Ca_kabuli_Chr03*	36227567	T/C	Gene_Ca_00929	UPSTREAM	Ribosomal protein S19/S15
Kabuli_wild_36227948	1399935606	*Ca_kabuli_Chr03*	36227948	A/G	Gene_Ca_00929	UPSTREAM	Ribosomal protein S19/S15
Kabuli_wild_36228032	1399935607	*Ca_kabuli_Chr03*	36228032	G/T	Gene_Ca_00929	UPSTREAM	Ribosomal protein S19/S15
Kabuli_wild_36228121	1399935608	*Ca_kabuli_Chr03*	36228121	T/A	Gene_Ca_00929	INTRON	Ribosomal protein S19/S15
Kabuli_wild_36228142	1399935609	*Ca_kabuli_Chr03*	36228142	A/G	Gene_Ca_00929	INTRON	Ribosomal protein S19/S15
Kabuli_wild_36242480	1399935610	*Ca_kabuli_Chr03*	36242480	G/C	NA	INTERGENIC	NA
Kabuli_wild_36242490	1399935611	*Ca_kabuli_Chr03*	36242490	G/T	NA	INTERGENIC	NA
Kabuli_wild_36242504	1399935612	*Ca_kabuli_Chr03*	36242504	C/T	NA	INTERGENIC	NA
Kabuli_wild_36247792	1399935613	*Ca_kabuli_Chr03*	36247792	T/C	Gene_Ca_00932	DOWNSTREAM	Ovariantumour,otubain
Kabuli_wild_36260865	1399935614	*Ca_kabuli_Chr03*	36260865	C/A	NA	INTERGENIC	NA
Kabuli_wild_36282091	1399935615	*Ca_kabuli_Chr03*	36282091	T/G	Gene_Ca_00938	NON_SYNONYMOUS_CODING	Domain of unknown function DUF255
Kabuli_wild_36293589	1399935616	*Ca_kabuli_Chr03*	36293589	A/G	Gene_Ca_00939	SYNONYMOUS_CODING	Zinc finger,CW-type
Kabuli_wild_36293625	1399935617	*Ca_kabuli_Chr03*	36293625	C/T	Gene_Ca_00939	SYNONYMOUS_CODING	Zinc finger,CW-type
Kabuli_wild_36293637	1399935618	*Ca_kabuli_Chr03*	36293637	C/T	Gene_Ca_00939	SYNONYMOUS_CODING	Zinc finger,CW-type
Kabuli_wild_36298180	1399935619	*Ca_kabuli_Chr03*	36298180	G/A	Gene_Ca_00939	NON_SYNONYMOUS_CODING	Zinc finger,CW-type
Kabuli_wild_36298200	1399935620	*Ca_kabuli_Chr03*	36298200	T/G	Gene_Ca_00939	INTRON	Zinc finger,CW-type
Kabuli_wild_36298273	1399935621	*Ca_kabuli_Chr03*	36298273	A/G	Gene_Ca_00939	INTRON	Zinc finger,CW-type
Kabuli_wild_36330750	1399935622	*Ca_kabuli_Chr03*	36330750	A/G	Gene_Ca_00942	NON_SYNONYMOUS_CODING	Homeobox
Kabuli_wild_36330815	1399935623	*Ca_kabuli_Chr03*	36330815	T/C	Gene_Ca_00942	SYNONYMOUS_CODING	Homeobox
Kabuli_wild_36334577	1399935624	*Ca_kabuli_Chr03*	36334577	G/C	Gene_Ca_00942	INTRON	Homeobox
Kabuli_wild_36334594	1399935625	*Ca_kabuli_Chr03*	36334594	G/A	Gene_Ca_00942	INTRON	Homeobox
Kabuli_wild_36334595	1399935626	*Ca_kabuli_Chr03*	36334595	C/T	Gene_Ca_00942	INTRON	Homeobox
Kabuli_wild_36334653	1399935627	*Ca_kabuli_Chr03*	36334653	G/A	Gene_Ca_00942	INTRON	Homeobox
Kabuli_wild_36345158	1399935628	*Ca_kabuli_Chr03*	36345158	T/C	Gene_Ca_00943	NON_SYNONYMOUS_CODING	Zinc finger,C2H2-type
Kabuli_wild_36357622	1399935629	*Ca_kabuli_Chr03*	36357622	A/G	Gene_Ca_00944	NON_SYNONYMOUS_CODING	NA
Kabuli_wild_36361339	1399935630	*Ca_kabuli_Chr03*	36361339	C/T	Gene_Ca_00944	UPSTREAM	NA
Kabuli_wild_36378294	1399935631	*Ca_kabuli_Chr03*	36378294	G/T	Gene_Ca_00946	SYNONYMOUS_CODING	Protein kinase,catalytic domain

SNP IDs	NCBI dbSNP accession IDs (NCBI_SS#)	Chromosomes	Physical positions (bp)	SNPs	Gene accession IDs	Sequence components of genome	Putative functions
Kabuli_wild_36378299	1399935632	*Ca_kabuli_Chr03*	36378299	C/G	Gene_Ca_00946	INTRON	Protein kinase,catalytic domain
Kabuli_wild_36378358	1399935633	*Ca_kabuli_Chr03*	36378358	G/A	Gene_Ca_00946	INTRON	Protein kinase,catalytic domain
Kabuli_wild_36387615	1399935634	*Ca_kabuli_Chr03*	36387615	A/T	NA	INTERGENIC	NA
Kabuli_wild_36387718	1399935635	*Ca_kabuli_Chr03*	36387718	C/T	NA	INTERGENIC	NA
Kabuli_wild_36387732	1399935636	*Ca_kabuli_Chr03*	36387732	G/A	NA	INTERGENIC	NA
Kabuli_wild_36396559	1399935637	*Ca_kabuli_Chr03*	36396559	C/A	NA	INTERGENIC	NA
Kabuli_wild_36396604	1399935638	*Ca_kabuli_Chr03*	36396604	T/A	NA	INTERGENIC	NA
Kabuli_wild_36417896	1399935639	*Ca_kabuli_Chr03*	36417896	A/G	Gene_Ca_00954	SYNONYMOUS_CODING	Glycosyltransferase,ALG3
Kabuli_wild_36417921	1399935640	*Ca_kabuli_Chr03*	36417921	G/A	Gene_Ca_00954	SYNONYMOUS_CODING	Glycosyltransferase,ALG3
Kabuli_wild_36432389	1399935641	*Ca_kabuli_Chr03*	36432389	G/A	Gene_Ca_00957	SYNONYMOUS_CODING	Thiamin pyrophospho kinase,catalytic domain
Kabuli_wild_36441575	1399935642	*Ca_kabuli_Chr03*	36441575	G/A	NA	INTERGENIC	NA
Kabuli_wild_36441593	1399935643	*Ca_kabuli_Chr03*	36441593	T/A	NA	INTERGENIC	NA
Kabuli_wild_36441594	1399935644	*Ca_kabuli_Chr03*	36441594	G/A	NA	INTERGENIC	NA
Kabuli_wild_36441595	1399935645	*Ca_kabuli_Chr03*	36441595	T/C	NA	INTERGENIC	NA
Kabuli_wild_36441977	1399935646	*Ca_kabuli_Chr03*	36441977	T/C	NA	INTERGENIC	NA
Kabuli_wild_36447274	1399935647	*Ca_kabuli_Chr03*	36447274	G/A	NA	INTERGENIC	NA
Kabuli_wild_36447364	1399935648	*Ca_kabuli_Chr03*	36447364	T/A	NA	INTERGENIC	NA
Kabuli_wild_36447520	1399935649	*Ca_kabuli_Chr03*	36447520	T/A	NA	INTERGENIC	NA
Kabuli_wild_36447525	1399935650	*Ca_kabuli_Chr03*	36447525	C/T	NA	INTERGENIC	NA
Kabuli_wild_36447605	1399935651	*Ca_kabuli_Chr03*	36447605	C/T	NA	INTERGENIC	NA
Kabuli_wild_36447622	1399935652	*Ca_kabuli_Chr03*	36447622	C/A	NA	INTERGENIC	NA
Kabuli_wild_36447666	1399935653	*Ca_kabuli_Chr03*	36447666	A/T	NA	INTERGENIC	NA
Kabuli_wild_36462914	1399935654	*Ca_kabuli_Chr03*	36462914	C/T	Gene_Ca_00963	DOWNSTREAM	NA
Kabuli_wild_36462916	1399935655	*Ca_kabuli_Chr03*	36462916	T/C	Gene_Ca_00963	DOWNSTREAM	NA
Kabuli_wild_36488722	1399935656	*Ca_kabuli_Chr03*	36488722	T/A	Gene_Ca_00966	DOWNSTREAM	Alpha/betahydrolasefold-1
Kabuli_wild_36488736	1399935657	*Ca_kabuli_Chr03*	36488736	T/C	Gene_Ca_00966	DOWNSTREAM	Alpha/betahydrolasefold-1
Kabuli_wild_36488853	1399935658	*Ca_kabuli_Chr03*	36488853	A/G	Gene_Ca_00966	DOWNSTREAM	Alpha/betahydrolasefold-1
Kabuli_wild_36503906	1399935659	*Ca_kabuli_Chr03*	36503906	C/T	Gene_Ca_00967	NON_SYNONYMOUS_CODING	Small-subunitprocessome,Utp14
Kabuli_wild_36503912	1399935660	*Ca_kabuli_Chr03*	36503912	T/C	Gene_Ca_00967	NON_SYNONYMOUS_CODING	Small-subunitprocessome,Utp14
Kabuli_wild_36524598	1399935661	*Ca_kabuli_Chr03*	36524598	G/C	NA	INTERGENIC	NA
Kabuli_wild_36524635	1399935662	*Ca_kabuli_Chr03*	36524635	A/G	NA	INTERGENIC	NA
Kabuli_wild_36524659	1399935663	*Ca_kabuli_Chr03*	36524659	T/C	NA	INTERGENIC	NA
Kabuli_wild_36531151	1399935664	*Ca_kabuli_Chr03*	36531151	C/A	NA	INTERGENIC	NA
Kabuli_wild_36588856	1399935665	*Ca_kabuli_Chr03*	36588856	C/T	Gene_Ca_00978	SYNONYMOUS_CODING	Pectinesterase,catalytic
Kabuli_wild_36588896	1399935666	*Ca_kabuli_Chr03*	36588896	C/G	Gene_Ca_00978	NON_SYNONYMOUS_CODING	Pectinesterase,catalytic
Kabuli_wild_36609043	1399935667	*Ca_kabuli_Chr03*	36609043	G/A	Gene_Ca_00980	NON_SYNONYMOUS_CODING	Metal-dependent phosphohydrolase, HD domain
Kabuli_wild_36616855	1399935668	*Ca_kabuli_Chr03*	36616855	T/C	NA	INTERGENIC	NA
Kabuli_wild_36644117	1399935669	*Ca_kabuli_Chr03*	36644117	A/T	Gene_Ca_00985	SYNONYMOUS_CODING	Target SNARE coiled-coil domain
Kabuli_wild_36721275	1399935670	*Ca_kabuli_Chr03*	36721275	C/T	Gene_Ca_00996	INTRON	Lipase,class3
Kabuli_wild_36733103	1399935671	*Ca_kabuli_Chr03*	36733103	T/G	Gene_Ca_00997	SYNONYMOUS_CODING	Heat shock protein DnaJ,N-terminal
Kabuli_wild_36733211	1399935672	*Ca_kabuli_Chr03*	36733211	A/G	Gene_Ca_00997	SYNONYMOUS_CODING	Heat shock protein DnaJ,N-terminal
Kabuli_wild_36749775	1399935673	*Ca_kabuli_Chr03*	36749775	T/A	Gene_Ca_00998	SYNONYMOUS_CODING	Glycosi dehydrolase,family 3, N-terminal
Kabuli_wild_36749826	1399935674	*Ca_kabuli_Chr03*	36749826	G/A	Gene_Ca_00998	SYNONYMOUS_CODING	Glycosi dehydrolase,family 3, N-terminal
Kabuli_wild_36749829	1399935675	*Ca_kabuli_Chr03*	36749829	T/C	Gene_Ca_00998	NON_SYNONYMOUS_CODING	Glycosi dehydrolase,family 3, N-terminal

SNP IDs	NCBI dbSNP accession IDs (NCBI_SS#)	Chromosomes	Physical positions (bp)	SNPs	Gene accession IDs	Sequence components of genome	Putative functions
Kabuli_wild_36749831	1399935676	*Ca_kabuli_Chr03*	36749831	T/C	Gene_Ca_00998	NON_SYNONYMOUS_CODING	Glycosi dehydrolase,family 3, N-terminal
Kabuli_wild_36749871	1399935677	*Ca_kabuli_Chr03*	36749871	T/C	Gene_Ca_00998	SYNONYMOUS_CODING	Glycosi dehydrolase,family 3, N-terminal
Kabuli_wild_36749916	1399935678	*Ca_kabuli_Chr03*	36749916	C/A	Gene_Ca_00998	NON_SYNONYMOUS_CODING	Glycosi dehydrolase,family 3, N-terminal
Kabuli_wild_36770026	1399935679	*Ca_kabuli_Chr03*	36770026	G/A	NA	INTERGENIC	NA
Kabuli_wild_36783494	1399935680	*Ca_kabuli_Chr03*	36783494	T/A	NA	INTERGENIC	NA
Kabuli_wild_36860134	1399935681	*Ca_kabuli_Chr03*	36860134	A/G	Gene_Ca_01010	DOWNSTREAM	DNA-binding WRKY
Kabuli_wild_36866054	1399935682	*Ca_kabuli_Chr03*	36866054	G/A	NA	INTERGENIC	NA
Kabuli_wild_36873683	1399935683	*Ca_kabuli_Chr03*	36873683	C/T	Gene_Ca_01013	NON_SYNONYMOUS_CODING	Protein kinase,catalytic domain
Kabuli_wild_36883268	1399935684	*Ca_kabuli_Chr03*	36883268	T/C	NA	INTERGENIC	NA
Kabuli_wild_36883333	1399935685	*Ca_kabuli_Chr03*	36883333	A/T	NA	INTERGENIC	NA
Kabuli_wild_36895362	1399935686	*Ca_kabuli_Chr03*	36895362	T/C	Gene_Ca_01015	INTRON	NA
Kabuli_wild_36938148	1399935687	*Ca_kabuli_Chr03*	36938148	A/G	Gene_Ca_01019	INTRON	RNA recognition motif domain
Kabuli_wild_36938271	1399935688	*Ca_kabuli_Chr03*	36938271	G/C	Gene_Ca_01019	INTRON	RNA recognition motif domain
Kabuli_wild_36943514	1399935689	*Ca_kabuli_Chr03*	36943514	A/T	Gene_Ca_01021	UPSTREAM	Zinc finger,CCHC-type
Kabuli_wild_36943522	1399935690	*Ca_kabuli_Chr03*	36943522	A/G	Gene_Ca_01021	UPSTREAM	Zinc finger,CCHC-type
Kabuli_wild_36967269	1399935691	*Ca_kabuli_Chr03*	36967269	C/G	NA	INTERGENIC	NA
Kabuli_wild_36991718	1399935692	*Ca_kabuli_Chr03*	36991718	C/A	Gene_Ca_01027	INTRON	RassmallGTPase,Rabtype
Kabuli_wild_37003976	1399935693	*Ca_kabuli_Chr03*	37003976	C/T	NA	INTERGENIC	NA
Kabuli_wild_37004026	1399935694	*Ca_kabuli_Chr03*	37004026	A/G	NA	INTERGENIC	NA
Kabuli_wild_37067095	1399935695	*Ca_kabuli_Chr03*	37067095	T/G	Gene_Ca_01033	SYNONYMOUS_CODING	Zinc finger, RING-type
Kabuli_wild_37072312	1399935696	*Ca_kabuli_Chr03*	37072312	T/C	Gene_Ca_01034	INTRON	WD40 repeat
Kabuli_wild_37077212	1399935697	*Ca_kabuli_Chr03*	37077212	T/A	Gene_Ca_01034	INTRON	Transcription factor ,SBP-box
Kabuli_wild_37090968	1399935698	*Ca_kabuli_Chr03*	37090968	T/C	NA	INTERGENIC	NA
Kabuli_wild_37093962	1399935699	*Ca_kabuli_Chr03*	37093962	G/T	Gene_Ca_01036	DOWNSTREAM	Transcription factor ,SBP-box
Kabuli_wild_37097271	1399935700	*Ca_kabuli_Chr03*	37097271	A/G	Gene_Ca_01036	INTRON	Transcription factor ,SBP-box
Kabuli_wild_37100897	1399935701	*Ca_kabuli_Chr03*	37100897	G/T	Gene_Ca_01036	INTRON	Transcription factor ,SBP-box
Kabuli_wild_37101339	1399935702	*Ca_kabuli_Chr03*	37101339	A/T	Gene_Ca_01036	NON_SYNONYMOUS_CODING	Transcription factor ,SBP-box
Kabuli_wild_37101409	1399935703	*Ca_kabuli_Chr03*	37101409	A/G	Gene_Ca_01036	SYNONYMOUS_CODING	Transcription factor ,SBP-box
Kabuli_wild_37101433	1399935704	*Ca_kabuli_Chr03*	37101433	T/G	Gene_Ca_01036	NON_SYNONYMOUS_CODING	Transcription factor ,SBP-box
Kabuli_wild_37108904	1399935705	*Ca_kabuli_Chr03*	37108904	A/T	Gene_Ca_01037	INTRON	Protein kinase,catalytic domain
Kabuli_wild_37108908	1399935706	*Ca_kabuli_Chr03*	37108908	A/G	Gene_Ca_01037	INTRON	Protein kinase,catalytic domain
Kabuli_wild_37110070	1399935707	*Ca_kabuli_Chr03*	37110070	A/G	Gene_Ca_01037	NON_SYNONYMOUS_CODING	Protein kinase,catalytic domain
Kabuli_wild_37110124	1399935708	*Ca_kabuli_Chr03*	37110124	A/T	Gene_Ca_01037	INTRON	Protein kinase,catalytic domain
Kabuli_wild_37146766	1399935709	*Ca_kabuli_Chr03*	37146766	C/G	Gene_Ca_01044	NON_SYNONYMOUS_CODING	Potassiumchannel,twopore-domain
Kabuli_wild_37146840	1399935710	*Ca_kabuli_Chr03*	37146840	A/G	Gene_Ca_01044	SYNONYMOUS_CODING	Potassiumchannel,twopore-domain
Kabuli_wild_37154761	1399935711	*Ca_kabuli_Chr03*	37154761	T/G	Gene_Ca_01045	DOWNSTREAM	Transferase
Kabuli_wild_37154857	1399935712	*Ca_kabuli_Chr03*	37154857	T/C	Gene_Ca_01045	DOWNSTREAM	Transferase
Kabuli_wild_37154938	1399935713	*Ca_kabuli_Chr03*	37154938	A/C	Gene_Ca_01045	DOWNSTREAM	Transferase
Kabuli_wild_37155925	1399935714	*Ca_kabuli_Chr03*	37155925	G/A	Gene_Ca_01045	DOWNSTREAM	Transferase
Kabuli_wild_37156951	1399935715	*Ca_kabuli_Chr03*	37156951	A/G	NA	INTERGENIC	NA
Kabuli_wild_37160822	1399935716	*Ca_kabuli_Chr03*	37160822	G/T	Gene_Ca_01047	SYNONYMOUS_CODING	Zinc finger,Sec23/Sec24-type
Kabuli_wild_37160939	1399935717	*Ca_kabuli_Chr03*	37160939	A/G	Gene_Ca_01047	SYNONYMOUS_CODING	Zinc finger,Sec23/Sec24-type
Kabuli_wild_37183961	1399935718	*Ca_kabuli_Chr03*	37183961	G/A	NA	INTERGENIC	NA
Kabuli_wild_37184049	1399935719	*Ca_kabuli_Chr03*	37184049	A/T	NA	INTERGENIC	NA

SNP IDs	NCBI dbSNP accession IDs (NCBI_SS#)	Chromosomes	Physical positions (bp)	SNPs	Gene accession IDs	Sequence components of genome	Putative functions
Kabuli_wild_37303478	1399935720	*Ca_kabuli_Chr03*	37303478	G/A	Gene_Ca_01064	SYNONYMOUS_CODING	WD40 repeat
Kabuli_wild_37303655	1399935721	*Ca_kabuli_Chr03*	37303655	T/A	Gene_Ca_01064	SYNONYMOUS_CODING	WD40 repeat
Kabuli_wild_37309420	1399935722	*Ca_kabuli_Chr03*	37309420	A/C	Gene_Ca_01064	INTRON	WD40 repeat
Kabuli_wild_37355013	1399935723	*Ca_kabuli_Chr03*	37355013	A/G	Gene_Ca_01069	NON_SYNONYMOUS_CODING	PAS
Kabuli_wild_37444451	1399935724	*Ca_kabuli_Chr03*	37444451	C/A	NA	INTERGENIC	NA
Kabuli_wild_37467145	1399935725	*Ca_kabuli_Chr03*	37467145	T/C	Gene_Ca_01080	SYNONYMOUS_CODING	Domain of unknown function DUF88
Kabuli_wild_37535860	1399935726	*Ca_kabuli_Chr03*	37535860	C/G	NA	INTERGENIC	NA
Kabuli_wild_37535992	1399935727	*Ca_kabuli_Chr03*	37535992	A/G	NA	INTERGENIC	NA
Kabuli_wild_37566766	1399935728	*Ca_kabuli_Chr03*	37566766	T/C	Gene_Ca_01093	UPSTREAM	Clathrinadaptor,musubunit
Kabuli_wild_37599142	1399935729	*Ca_kabuli_Chr03*	37599142	T/C	NA	INTERGENIC	NA
Kabuli_wild_37630502	1399935730	*Ca_kabuli_Chr03*	37630502	T/C	NA	INTERGENIC	NA
Kabuli_wild_37635909	1399935731	*Ca_kabuli_Chr03*	37635909	G/A	NA	INTERGENIC	NA
Kabuli_wild_37685596	1399935732	*Ca_kabuli_Chr03*	37685596	A/G	Gene_Ca_01107	NON_SYNONYMOUS_CODING	NHLrepeat
Kabuli_wild_37688838	1399935733	*Ca_kabuli_Chr03*	37688838	A/G	NA	INTERGENIC	NA
Kabuli_wild_37734399	1399935734	*Ca_kabuli_Chr03*	37734399	T/C	Gene_Ca_01110	INTRON	NHLrepeat
Kabuli_wild_37759329	1399935735	*Ca_kabuli_Chr03*	37759329	G/A	Gene_Ca_01114	INTRON	Armadillo
Kabuli_wild_37765702	1399935736	*Ca_kabuli_Chr03*	37765702	G/A	Gene_Ca_01115	UPSTREAM	Zinc finger,GATA-type
Kabuli_wild_37765767	1399935737	*Ca_kabuli_Chr03*	37765767	A/C	Gene_Ca_01115	UPSTREAM	Zinc finger,GATA-type
Kabuli_wild_37887009	1399935738	*Ca_kabuli_Chr03*	37887009	A/G	NA	INTERGENIC	NA
Kabuli_wild_37896181	1399935739	*Ca_kabuli_Chr03*	37896181	A/G	NA	INTERGENIC	NA
Kabuli_wild_37908034	1399935740	*Ca_kabuli_Chr03*	37908034	C/A	NA	INTERGENIC	NA
Kabuli_wild_37908035	1399935741	*Ca_kabuli_Chr03*	37908035	C/G	NA	INTERGENIC	NA
Kabuli_wild_37908317	1399935742	*Ca_kabuli_Chr03*	37908317	T/C	NA	INTERGENIC	NA
Kabuli_wild_37908492	1399935743	*Ca_kabuli_Chr03*	37908492	A/C	NA	INTERGENIC	NA
Kabuli_wild_37910881	1399935744	*Ca_kabuli_Chr03*	37910881	A/G	NA	INTERGENIC	NA
Kabuli_wild_37942807	1399935745	*Ca_kabuli_Chr03*	37942807	A/G	NA	INTERGENIC	NA
Kabuli_wild_37942962	1399935746	*Ca_kabuli_Chr03*	37942962	T/C	NA	INTERGENIC	NA
Kabuli_wild_37949374	1399935747	*Ca_kabuli_Chr03*	37949374	A/G	Gene_Ca_01133	INTRON	NA
Kabuli_wild_37985776	1399935748	*Ca_kabuli_Chr03*	37985776	T/A	NA	INTERGENIC	NA
Kabuli_wild_37985947	1399935749	*Ca_kabuli_Chr03*	37985947	A/T	NA	INTERGENIC	NA
Kabuli_wild_37985977	1399935750	*Ca_kabuli_Chr03*	37985977	A/G	NA	INTERGENIC	NA
Kabuli_wild_37992875	1399935751	*Ca_kabuli_Chr03*	37992875	G/C	NA	INTERGENIC	NA
Kabuli_wild_38015973	1399935752	*Ca_kabuli_Chr03*	38015973	C/G	NA	INTERGENIC	NA
Kabuli_wild_38026450	1399935753	*Ca_kabuli_Chr03*	38026450	G/A	NA	INTERGENIC	NA
Kabuli_wild_38026539	1399935754	*Ca_kabuli_Chr03*	38026539	A/G	NA	INTERGENIC	NA
Kabuli_wild_38026725	1399935755	*Ca_kabuli_Chr03*	38026725	G/A	NA	INTERGENIC	NA
Kabuli_wild_38029095	1399935756	*Ca_kabuli_Chr03*	38029095	G/C	Gene_Ca_01143	NON_SYNONYMOUS_CODING	NA
Kabuli_wild_38033969	1399935757	*Ca_kabuli_Chr03*	38033969	A/G	NA	INTERGENIC	NA
Kabuli_wild_38046510	1399935758	*Ca_kabuli_Chr03*	38046510	T/C	Gene_Ca_01146	UPSTREAM	WASH complex, F-actincapping protein, alpha subunit
Kabuli_wild_38046522	1399935759	*Ca_kabuli_Chr03*	38046522	A/G	Gene_Ca_01146	UPSTREAM	WASH complex, F-actincapping protein, alpha subunit
Kabuli_wild_38046954	1399935760	*Ca_kabuli_Chr03*	38046954	G/A	Gene_Ca_01146	UPSTREAM	WASH complex, F-actincapping protein, alpha subunit
Kabuli_wild_38049940	1399935761	*Ca_kabuli_Chr03*	38049940	A/G	NA	INTERGENIC	NA
Kabuli_wild_38049996	1399935762	*Ca_kabuli_Chr03*	38049996	A/G	NA	INTERGENIC	NA
Kabuli_wild_38051020	1399935763	*Ca_kabuli_Chr03*	38051020	A/G	Gene_Ca_01147	UPSTREAM	Tafazzin

SNP IDs	NCBI dbSNP accession IDs (NCBI_SS#)	Chromosomes	Physical positions (bp)	SNPs	Gene accession IDs	Sequence components of genome	Putative functions
Kabuli_wild_38059303	1399935764	*Ca_kabuli_Chr03*	38059303	T/C	NA	INTERGENIC	NA
Kabuli_wild_38059311	1399935765	*Ca_kabuli_Chr03*	38059311	T/A	NA	INTERGENIC	NA
Kabuli_wild_38060742	1399935766	*Ca_kabuli_Chr03*	38060742	C/T	NA	INTERGENIC	NA
Kabuli_wild_38060755	1399935767	*Ca_kabuli_Chr03*	38060755	T/C	NA	INTERGENIC	NA
Kabuli_wild_38060814	1399935768	*Ca_kabuli_Chr03*	38060814	A/G	NA	INTERGENIC	NA
Kabuli_wild_38074259	1399935769	*Ca_kabuli_Chr03*	38074259	C/A	NA	INTERGENIC	NA
Kabuli_wild_38103375	1399935770	*Ca_kabuli_Chr03*	38103375	T/C	Gene_Ca_01154	INTRON	PeptidaseS1/S6,chymotrypsin/Hap
Kabuli_wild_38103378	1399935771	*Ca_kabuli_Chr03*	38103378	C/T	Gene_Ca_01154	INTRON	PeptidaseS1/S6,chymotrypsin/Hap
Kabuli_wild_38103385	1399935772	*Ca_kabuli_Chr03*	38103385	C/T	Gene_Ca_01154	INTRON	PeptidaseS1/S6,chymotrypsin/Hap
Kabuli_wild_38103524	1399935773	*Ca_kabuli_Chr03*	38103524	C/T	Gene_Ca_01154	SYNONYMOUS_CODING	PeptidaseS1/S6,chymotrypsin/Hap
Kabuli_wild_38112801	1399935774	*Ca_kabuli_Chr03*	38112801	C/T	NA	INTERGENIC	NA
Kabuli_wild_38112802	1399935775	*Ca_kabuli_Chr03*	38112802	T/C	NA	INTERGENIC	NA
Kabuli_wild_38112971	1399935776	*Ca_kabuli_Chr03*	38112971	A/G	NA	INTERGENIC	NA
Kabuli_wild_38117169	1399935777	*Ca_kabuli_Chr03*	38117169	C/T	Gene_Ca_01156	INTRON	Acyl-CoA-bindingprotein,ACBP
Kabuli_wild_38130410	1399935778	*Ca_kabuli_Chr03*	38130410	A/G	Gene_Ca_01157	DOWNSTREAM	SANT,eukarya
Kabuli_wild_38145010	1399935779	*Ca_kabuli_Chr03*	38145010	T/G	Gene_Ca_01158	NON_SYNONYMOUS_CODING	Cystathioninebeta-synthase,core
Kabuli_wild_38145115	1399935780	*Ca_kabuli_Chr03*	38145115	C/T	Gene_Ca_01158	SYNONYMOUS_CODING	Cystathioninebeta-synthase,core
Kabuli_wild_38169101	1399935781	*Ca_kabuli_Chr03*	38169101	G/A	Gene_Ca_01160	INTRON	DNA-directedDNApolymerase,familyB,exonucleasedomain
Kabuli_wild_38169102	1399935782	*Ca_kabuli_Chr03*	38169102	G/T	Gene_Ca_01160	INTRON	DNA-directedDNApolymerase,familyB,exonucleasedomain
Kabuli_wild_38169157	1399935783	*Ca_kabuli_Chr03*	38169157	A/G	Gene_Ca_01160	INTRON	DNA-directedDNApolymerase,familyB,exonucleasedomain
Kabuli_wild_38171434	1399935784	*Ca_kabuli_Chr03*	38171434	G/A	Gene_Ca_01160	SYNONYMOUS_CODING	DNA-directedDNApolymerase,familyB,exonucleasedomain
Kabuli_wild_38199284	1399935785	*Ca_kabuli_Chr03*	38199284	A/T	Gene_Ca_01163	SYNONYMOUS_CODING	Creatinase
Kabuli_wild_38200668	1399935786	*Ca_kabuli_Chr03*	38200668	T/C	Gene_Ca_01163	NON_SYNONYMOUS_CODING	Creatinase
Kabuli_wild_38201469	1399935787	*Ca_kabuli_Chr03*	38201469	G/A	Gene_Ca_01163	INTRON	Creatinase
Kabuli_wild_38201557	1399935788	*Ca_kabuli_Chr03*	38201557	G/A	Gene_Ca_01163	NON_SYNONYMOUS_CODING	Creatinase
Kabuli_wild_38202223	1399935789	*Ca_kabuli_Chr03*	38202223	T/C	Gene_Ca_01163	INTRON	Creatinase
Kabuli_wild_38205470	1399935790	*Ca_kabuli_Chr03*	38205470	A/G	Gene_Ca_01163	INTRON	Creatinase
Kabuli_wild_38205481	1399935791	*Ca_kabuli_Chr03*	38205481	A/T	Gene_Ca_01163	INTRON	Creatinase
Kabuli_wild_38205491	1399935792	*Ca_kabuli_Chr03*	38205491	C/A	Gene_Ca_01163	INTRON	Creatinase
Kabuli_wild_38205503	1399935793	*Ca_kabuli_Chr03*	38205503	G/C	Gene_Ca_01163	INTRON	Creatinase
Kabuli_wild_38205525	1399935794	*Ca_kabuli_Chr03*	38205525	A/G	Gene_Ca_01163	INTRON	Creatinase
Kabuli_wild_38220910	1399935795	*Ca_kabuli_Chr03*	38220910	T/C	NA	INTERGENIC	NA
Kabuli_wild_38220958	1399935796	*Ca_kabuli_Chr03*	38220958	A/G	NA	INTERGENIC	NA
Kabuli_wild_38222733	1399935797	*Ca_kabuli_Chr03*	38222733	C/G	NA	INTERGENIC	NA
Kabuli_wild_38222747	1399935798	*Ca_kabuli_Chr03*	38222747	G/A	NA	INTERGENIC	NA
Kabuli_wild_38222750	1399935799	*Ca_kabuli_Chr03*	38222750	A/C	NA	INTERGENIC	NA
Kabuli_wild_38228118	1399935800	*Ca_kabuli_Chr03*	38228118	G/T	NA	INTERGENIC	NA
Kabuli_wild_38230625	1399935801	*Ca_kabuli_Chr03*	38230625	C/T	NA	INTERGENIC	NA
Kabuli_wild_38237347	1399935802	*Ca_kabuli_Chr03*	38237347	G/A	Gene_Ca_01169	INTRON	Glycosidehydrolase,family2,immunoglobulin-likebeta-sandwich
Kabuli_wild_38237606	1399935803	*Ca_kabuli_Chr03*	38237606	A/G	Gene_Ca_01169	INTRON	Glycosidehydrolase,family2,immunoglobulin-likebeta-sandwich
Kabuli_wild_38237628	1399935804	*Ca_kabuli_Chr03*	38237628	A/G	Gene_Ca_01169	INTRON	Glycosidehydrolase,family2,immunoglobulin-likebeta-sandwich
Kabuli_wild_38294315	1399935805	*Ca_kabuli_Chr03*	38294315	C/A	Gene_Ca_01177	DOWNSTREAM	Signalpeptidase22kDasubunit
Kabuli_wild_38386392	1399935806	*Ca_kabuli_Chr03*	38386392	T/C	NA	INTERGENIC	NA
Kabuli_wild_38452060	1399935807	*Ca_kabuli_Chr03*	38452060	A/C	Gene_Ca_01198	NON_SYNONYMOUS_CODING	Heat shock protein DnaJ,N-terminal

SNP IDs	NCBI dbSNP accession IDs (NCBI_SS#)	Chromosomes	Physical positions (bp)	SNPs	Gene accession IDs	Sequence components of genome	Putative functions
Kabuli_wild_38539721	1399935808	*Ca_kabuli_Chr03*	38539721	T/G	Gene_Ca_01207	NON_SYNONYMOUS_CODING	Alpha/betahydrolasefold-1
Kabuli_wild_38551142	1399935809	*Ca_kabuli_Chr03*	38551142	C/T	Gene_Ca_01208	INTRON	WD40 repeat
Kabuli_wild_38629643	1399935810	*Ca_kabuli_Chr03*	38629643	A/G	Gene_Ca_01218	SYNONYMOUS_CODING	NA
Kabuli_wild_38672176	1399935811	*Ca_kabuli_Chr03*	38672176	G/A	NA	INTERGENIC	NA
Kabuli_wild_38672863	1399935812	*Ca_kabuli_Chr03*	38672863	C/A	Gene_Ca_01223	UPSTREAM	Pseudouridinesynthase,TruD
Kabuli_wild_38731466	1399935813	*Ca_kabuli_Chr03*	38731466	T/G	Gene_Ca_01231	NON_SYNONYMOUS_CODING	Ammoniumtransporter
Kabuli_wild_38764390	1399935814	*Ca_kabuli_Chr03*	38764390	A/C	Gene_Ca_01234	DOWNSTREAM	Basic-leucine zipper (bZIP) Transcription factor
Kabuli_wild_38766174	1399935815	*Ca_kabuli_Chr03*	38766174	A/G	NA	INTERGENIC	NA
Kabuli_wild_38766311	1399935816	*Ca_kabuli_Chr03*	38766311	A/C	NA	INTERGENIC	NA
Kabuli_wild_38776419	1399935817	*Ca_kabuli_Chr03*	38776419	C/T	Gene_Ca_01236	SYNONYMOUS_CODING	Mediatorcomplex,subunitMed14
Kabuli_wild_38805831	1399935818	*Ca_kabuli_Chr03*	38805831	A/C	Gene_Ca_01240	INTRON	Ankyrinrepeat
Kabuli_wild_38818313	1399935819	*Ca_kabuli_Chr03*	38818313	T/G	Gene_Ca_01243	DOWNSTREAM	NA
Kabuli_wild_38821990	1399935820	*Ca_kabuli_Chr03*	38821990	A/G	NA	INTERGENIC	NA
Kabuli_wild_38831533	1399935821	*Ca_kabuli_Chr03*	38831533	T/C	Gene_Ca_01244	UPSTREAM	Xklp2targetingprotein
Kabuli_wild_38848407	1399935822	*Ca_kabuli_Chr03*	38848407	T/C	Gene_Ca_01245	NON_SYNONYMOUS_CODING	K Homology
Kabuli_wild_38848409	1399935823	*Ca_kabuli_Chr03*	38848409	C/T	Gene_Ca_01245	SYNONYMOUS_CODING	K Homology
Kabuli_wild_38848412	1399935824	*Ca_kabuli_Chr03*	38848412	C/G	Gene_Ca_01245	SYNONYMOUS_CODING	K Homology
Kabuli_wild_38878839	1399935825	*Ca_kabuli_Chr03*	38878839	A/G	NA	INTERGENIC	NA
Kabuli_wild_38890736	1399935826	*Ca_kabuli_Chr03*	38890736	G/T	Gene_Ca_01248	NON_SYNONYMOUS_CODING	IQ motif,EF-handbinding site
Kabuli_wild_38913449	1399935827	*Ca_kabuli_Chr03*	38913449	T/C	NA	INTERGENIC	NA
Kabuli_wild_38923321	1399935828	*Ca_kabuli_Chr03*	38923321	C/T	Gene_Ca_01252	SYNONYMOUS_CODING	Glycosyltransferase,family8
Kabuli_wild_38965610	1399935829	*Ca_kabuli_Chr03*	38965610	T/A	Gene_Ca_01258	NON_SYNONYMOUS_CODING	SANT domain,DNA binding
Kabuli_wild_38969199	1399935830	*Ca_kabuli_Chr03*	38969199	C/T	Gene_Ca_01258	SYNONYMOUS_CODING	SANT domain,DNA binding
Kabuli_wild_38982481	1399935831	*Ca_kabuli_Chr03*	38982481	A/G	Gene_Ca_01260	INTRON	NA
Kabuli_wild_38987916	1399935832	*Ca_kabuli_Chr03*	38987916	A/C	NA	INTERGENIC	NA
Kabuli_wild_39034246	1399935833	*Ca_kabuli_Chr03*	39034246	C/T	Gene_Ca_01265	NON_SYNONYMOUS_CODING	NA
Kabuli_wild_39036130	1399935834	*Ca_kabuli_Chr03*	39036130	T/C	Gene_Ca_01265	INTRON	NA
Kabuli_wild_39053719	1399935835	*Ca_kabuli_Chr03*	39053719	T/G	Gene_Ca_01268	INTRON	NA
Kabuli_wild_39055529	1399935836	*Ca_kabuli_Chr03*	39055529	C/T	Gene_Ca_01268	INTRON	EF-Hand1,calcium-bindingsite
Kabuli_wild_39081040	1399935837	*Ca_kabuli_Chr03*	39081040	A/G	NA	INTERGENIC	NA
Kabuli_wild_39084916	1399935838	*Ca_kabuli_Chr03*	39084916	T/A	Gene_Ca_01271	STOP_GAINED	Domain of unknown function DUF296
Kabuli_wild_39084979	1399935839	*Ca_kabuli_Chr03*	39084979	A/C	Gene_Ca_01271	SYNONYMOUS_CODING	Domain of unknown function DUF296
Kabuli_wild_39093215	1399935840	*Ca_kabuli_Chr03*	39093215	G/A	NA	INTERGENIC	NA
Kabuli_wild_39093242	1399935841	*Ca_kabuli_Chr03*	39093242	G/A	NA	INTERGENIC	NA
Kabuli_wild_39093243	1399935842	*Ca_kabuli_Chr03*	39093243	C/A	NA	INTERGENIC	NA
Kabuli_wild_39093245	1399935843	*Ca_kabuli_Chr03*	39093245	T/A	NA	INTERGENIC	NA
Kabuli_wild_39115274	1399935844	*Ca_kabuli_Chr03*	39115274	A/C	Gene_Ca_01274	INTRON	HEAT
Kabuli_wild_39115970	1399935845	*Ca_kabuli_Chr03*	39115970	T/G	Gene_Ca_01274	INTRON	HEAT
Kabuli_wild_39115972	1399935846	*Ca_kabuli_Chr03*	39115972	T/A	Gene_Ca_01274	INTRON	HEAT
Kabuli_wild_39115979	1399935847	*Ca_kabuli_Chr03*	39115979	A/G	Gene_Ca_01274	INTRON	HEAT
Kabuli_wild_39115986	1399935848	*Ca_kabuli_Chr03*	39115986	C/G	Gene_Ca_01274	INTRON	HEAT
Kabuli_wild_39118510	1399935849	*Ca_kabuli_Chr03*	39118510	C/T	Gene_Ca_01274	INTRON	HEAT
Kabuli_wild_39124896	1399935850	*Ca_kabuli_Chr03*	39124896	A/T	Gene_Ca_01274	INTRON	HEAT
Kabuli_wild_39126018	1399935851	*Ca_kabuli_Chr03*	39126018	A/G	Gene_Ca_01274	INTRON	HEAT

SNP IDs	NCBI dbSNP accession IDs (NCBI_SS#)	Chromosomes	Physical positions (bp)	SNPs	Gene accession IDs	Sequence components of genome	Putative functions
Kabuli_wild_39200690	1399935852	*Ca_kabuli_Chr03*	39200690	C/T	Gene_Ca_01277	INTRON	CytoplasmicFMR1-interacting
Kabuli_wild_39207112	1399935853	*Ca_kabuli_Chr03*	39207112	A/C	Gene_Ca_01277	SYNONYMOUS_CODING	CytoplasmicFMR1-interacting
Kabuli_wild_39215327	1399935854	*Ca_kabuli_Chr03*	39215327	C/T	Gene_Ca_01278	NON_SYNONYMOUS_CODING	Armadillo
Kabuli_wild_39215476	1399935855	*Ca_kabuli_Chr03*	39215476	G/A	Gene_Ca_01278	SYNONYMOUS_CODING	Armadillo
Kabuli_wild_39254527	1399935856	*Ca_kabuli_Chr03*	39254527	T/G	NA	INTERGENIC	NA
Kabuli_wild_39255709	1399935857	*Ca_kabuli_Chr03*	39255709	A/T	NA	INTERGENIC	NA
Kabuli_wild_39255713	1399935858	*Ca_kabuli_Chr03*	39255713	C/A	NA	INTERGENIC	NA
Kabuli_wild_39255714	1399935859	*Ca_kabuli_Chr03*	39255714	C/T	NA	INTERGENIC	NA
Kabuli_wild_39255715	1399935860	*Ca_kabuli_Chr03*	39255715	G/A	NA	INTERGENIC	NA
Kabuli_wild_39255721	1399935861	*Ca_kabuli_Chr03*	39255721	A/C	NA	INTERGENIC	NA
Kabuli_wild_39255754	1399935862	*Ca_kabuli_Chr03*	39255754	A/C	NA	INTERGENIC	NA
Kabuli_wild_39257226	1399935863	*Ca_kabuli_Chr03*	39257226	A/G	Gene_Ca_01285	INTRON	NA
Kabuli_wild_39321667	1399935864	*Ca_kabuli_Chr03*	39321667	T/A	NA	INTERGENIC	NA
Kabuli_wild_39405252	1399935865	*Ca_kabuli_Chr03*	39405252	C/A	Gene_Ca_01306	NON_SYNONYMOUS_CODING	Lipase,class3
Kabuli_wild_39426267	1399935866	*Ca_kabuli_Chr03*	39426267	A/G	NA	INTERGENIC	NA
Kabuli_wild_39426273	1399935867	*Ca_kabuli_Chr03*	39426273	A/G	NA	INTERGENIC	NA
Kabuli_wild_39485205	1399935868	*Ca_kabuli_Chr03*	39485205	C/T	NA	INTERGENIC	NA
Kabuli_wild_39504242	1399935869	*Ca_kabuli_Chr03*	39504242	A/G	Gene_Ca_01321	DOWNSTREAM	Zinc finger, RING-type
Kabuli_wild_39546789	1399935870	*Ca_kabuli_Chr03*	39546789	T/G	Gene_Ca_01325	NON_SYNONYMOUS_CODING	Pathogenesis-related transcriptional factor/ERF, DNA-binding
Kabuli_wild_39592340	1399935871	*Ca_kabuli_Chr03*	39592340	C/T	NA	INTERGENIC	NA
Kabuli_wild_39612447	1399935872	*Ca_kabuli_Chr03*	39612447	C/A	Gene_Ca_01338	INTRON	Profilin/allergen
Kabuli_wild_39612458	1399935873	*Ca_kabuli_Chr03*	39612458	C/A	Gene_Ca_01338	INTRON	Profilin/allergen
Kabuli_wild_39612466	1399935874	*Ca_kabuli_Chr03*	39612466	T/C	Gene_Ca_01338	INTRON	Profilin/allergen
Kabuli_wild_39612568	1399935875	*Ca_kabuli_Chr03*	39612568	A/T	Gene_Ca_01338	NON_SYNONYMOUS_CODING	Profilin/allergen
Kabuli_wild_39628916	1399935876	*Ca_kabuli_Chr03*	39628916	G/A	NA	INTERGENIC	NA
Kabuli_wild_39715789	1399935877	*Ca_kabuli_Chr03*	39715789	C/T	NA	INTERGENIC	NA
Kabuli_wild_39722314	1399935878	*Ca_kabuli_Chr03*	39722314	C/T	Gene_Ca_01355	NON_SYNONYMOUS_CODING	Dehydrogenase,E1component
Kabuli_wild_39787513	1399935879	*Ca_kabuli_Chr03*	39787513	A/C	Gene_Ca_01365	SYNONYMOUS_CODING	NA
Kabuli_wild_39801213	1399935880	*Ca_kabuli_Chr03*	39801213	A/G	Gene_Ca_01367	INTRON	RibonucleaseIII
Kabuli_wild_39817122	1399935881	*Ca_kabuli_Chr03*	39817122	G/A	Gene_Ca_01368	SYNONYMOUS_CODING	Transcriptional factor B3
Kabuli_wild_39839742	1399935882	*Ca_kabuli_Chr03*	39839742	T/C	Gene_Ca_01370	INTRON	ATPase, P-type, H+ transporting proton pump
Kabuli_wild_39839763	1399935883	*Ca_kabuli_Chr03*	39839763	T/G	Gene_Ca_01370	INTRON	ATPase, P-type, H+ transporting proton pump
Kabuli_wild_39839767	1399935884	*Ca_kabuli_Chr03*	39839767	T/G	Gene_Ca_01370	INTRON	ATPase, P-type, H+ transporting proton pump
Kabuli_wild_39890991	1399935885	*Ca_kabuli_Chr03*	39890991	A/T	Gene_Ca_01375	INTRON	Exportin-1/Importin-beta-like
Kabuli_wild_39908983	1399935886	*Ca_kabuli_Chr03*	39908983	C/G	NA	INTERGENIC	NA
Kabuli_wild_39909183	1399935887	*Ca_kabuli_Chr03*	39909183	A/C	NA	INTERGENIC	NA
Kabuli_wild_39909824	1399935888	*Ca_kabuli_Chr03*	39909824	C/G	NA	INTERGENIC	NA
Kabuli_wild_39914344	1399935889	*Ca_kabuli_Chr03*	39914344	T/G	Gene_Ca_01376	INTRON	RNA recognition motif domain
Kabuli_wild_20066	1399935890	*Ca_kabuli_Chr04*	20066	T/G	Gene_Ca_27693	SYNONYMOUS_CODING	Basic-leucine zipper (bZIP) Transcription factor
Kabuli_wild_20297	1399935891	*Ca_kabuli_Chr04*	20297	A/G	Gene_Ca_27693	SYNONYMOUS_CODING	Basic-leucine zipper (bZIP) Transcription factor
Kabuli_wild_20466	1399935892	*Ca_kabuli_Chr04*	20466	G/T	Gene_Ca_27693	INTRON	Basic-leucine zipper (bZIP) Transcription factor
Kabuli_wild_20495	1399935893	*Ca_kabuli_Chr04*	20495	G/C	Gene_Ca_27693	INTRON	Basic-leucine zipper (bZIP) Transcription factor
Kabuli_wild_115171	1399935894	*Ca_kabuli_Chr04*	115171	C/G	NA	INTERGENIC	NA
Kabuli_wild_115173	1399935895	*Ca_kabuli_Chr04*	115173	C/T	NA	INTERGENIC	NA

SNP IDs	NCBI dbSNP accession IDs (NCBI_SS#)	Chromosomes	Physical positions (bp)	SNPs	Gene accession IDs	Sequence components of genome	Putative functions
Kabuli_wild_122309	1399935896	*Ca_kabuli_Chr04*	122309	C/G	NA	INTERGENIC	NA
Kabuli_wild_122311	1399935897	*Ca_kabuli_Chr04*	122311	C/T	NA	INTERGENIC	NA
Kabuli_wild_167229	1399935898	*Ca_kabuli_Chr04*	167229	T/G	Gene_Ca_07652	SYNONYMOUS_CODING	LNS2,Lipin/Ned1/Smp2
Kabuli_wild_338721	1399935899	*Ca_kabuli_Chr04*	338721	C/T	NA	INTERGENIC	NA
Kabuli_wild_349309	1399935900	*Ca_kabuli_Chr04*	349309	C/T	Gene_Ca_07667	NON_SYNONYMOUS_CODING	Multiantimicrobial extrusion protein
Kabuli_wild_369258	1399935901	*Ca_kabuli_Chr04*	369258	C/T	Gene_Ca_07669	NON_SYNONYMOUS_CODING	Multiantimicrobial extrusion protein
Kabuli_wild_394661	1399935902	*Ca_kabuli_Chr04*	394661	C/G	Gene_Ca_07671	NON_SYNONYMOUS_CODING	Inorganic pyrophosphatase
Kabuli_wild_412017	1399935903	*Ca_kabuli_Chr04*	412017	C/A	Gene_Ca_07673	NON_SYNONYMOUS_CODING	Arf GTPase activating protein
Kabuli_wild_455361	1399935904	*Ca_kabuli_Chr04*	455361	A/G	Gene_Ca_07679	INTRON	ArgininebiosynthesisproteinArgJ
Kabuli_wild_479818	1399935905	*Ca_kabuli_Chr04*	479818	A/C	Gene_Ca_07682	SYNONYMOUS_CODING	NA
Kabuli_wild_480138	1399935906	*Ca_kabuli_Chr04*	480138	C/A	Gene_Ca_07682	NON_SYNONYMOUS_CODING	NA
Kabuli_wild_586558	1399935907	*Ca_kabuli_Chr04*	586558	C/A	NA	INTERGENIC	NA
Kabuli_wild_586703	1399935908	*Ca_kabuli_Chr04*	586703	T/C	NA	INTERGENIC	NA
Kabuli_wild_589191	1399935909	*Ca_kabuli_Chr04*	589191	A/G	Gene_Ca_07691	DOWNSTREAM	Helix-loop-helixDNA-binding domain
Kabuli_wild_589204	1399935910	*Ca_kabuli_Chr04*	589204	G/A	Gene_Ca_07691	DOWNSTREAM	Helix-loop-helixDNA-binding domain
Kabuli_wild_589220	1399935911	*Ca_kabuli_Chr04*	589220	A/C	Gene_Ca_07691	DOWNSTREAM	Helix-loop-helixDNA-binding domain
Kabuli_wild_595982	1399935912	*Ca_kabuli_Chr04*	595982	C/G	Gene_Ca_07691	SYNONYMOUS_CODING	Helix-loop-helixDNA-binding domain
Kabuli_wild_646567	1399935913	*Ca_kabuli_Chr04*	646567	C/T	Gene_Ca_07695	DOWNSTREAM	Protein of unknown function DUF3741
Kabuli_wild_660029	1399935914	*Ca_kabuli_Chr04*	660029	T/G	NA	INTERGENIC	NA
Kabuli_wild_660034	1399935915	*Ca_kabuli_Chr04*	660034	A/C	NA	INTERGENIC	NA
Kabuli_wild_663666	1399935916	*Ca_kabuli_Chr04*	663666	T/G	NA	INTERGENIC	NA
Kabuli_wild_705393	1399935917	*Ca_kabuli_Chr04*	705393	C/G	Gene_Ca_07701	INTRON	IQ motif,EF-handbinding site
Kabuli_wild_783262	1399935918	*Ca_kabuli_Chr04*	783262	G/A	NA	INTERGENIC	NA
Kabuli_wild_789090	1399935919	*Ca_kabuli_Chr04*	789090	C/T	Gene_Ca_07707	UPSTREAM	Beta-ketoacylsynthase
Kabuli_wild_827593	1399935920	*Ca_kabuli_Chr04*	827593	G/T	Gene_Ca_07712	DOWNSTREAM	ABC transporter,trans membrane domain
Kabuli_wild_1190184	1399935921	*Ca_kabuli_Chr04*	1190184	A/C	Gene_Ca_07749	INTRON	Ribosomal protein L21e
Kabuli_wild_1228320	1399935922	*Ca_kabuli_Chr04*	1228320	A/G	Gene_Ca_07755	SYNONYMOUS_CODING	Pathogenesis-related transcriptional factor/ERF, DNA-binding
Kabuli_wild_1492741	1399935923	*Ca_kabuli_Chr04*	1492741	A/C	Gene_Ca_07779	NON_SYNONYMOUS_CODING	DNA-binding WRKY
Kabuli_wild_1493946	1399935924	*Ca_kabuli_Chr04*	1493946	T/G	Gene_Ca_07779	NON_SYNONYMOUS_CODING	DNA-binding WRKY
Kabuli_wild_1526270	1399935925	*Ca_kabuli_Chr04*	1526270	T/C	NA	INTERGENIC	NA
Kabuli_wild_1687346	1399935926	*Ca_kabuli_Chr04*	1687346	A/G	NA	INTERGENIC	NA
Kabuli_wild_1691555	1399935927	*Ca_kabuli_Chr04*	1691555	T/G	NA	INTERGENIC	NA
Kabuli_wild_1708370	1399935928	*Ca_kabuli_Chr04*	1708370	C/T	Gene_Ca_07797	SYNONYMOUS_CODING	BEACHdomain
Kabuli_wild_1711864	1399935929	*Ca_kabuli_Chr04*	1711864	A/C	Gene_Ca_07797	SYNONYMOUS_CODING	BEACHdomain
Kabuli_wild_1711865	1399935930	*Ca_kabuli_Chr04*	1711865	G/A	Gene_Ca_07797	SYNONYMOUS_CODING	BEACHdomain
Kabuli_wild_1720642	1399935931	*Ca_kabuli_Chr04*	1720642	A/G	Gene_Ca_07797	INTRON	BEACHdomain
Kabuli_wild_1755066	1399935932	*Ca_kabuli_Chr04*	1755066	G/C	NA	INTERGENIC	NA
Kabuli_wild_1773886	1399935933	*Ca_kabuli_Chr04*	1773886	C/G	NA	INTERGENIC	NA
Kabuli_wild_1773939	1399935934	*Ca_kabuli_Chr04*	1773939	C/T	NA	INTERGENIC	NA
Kabuli_wild_1773951	1399935935	*Ca_kabuli_Chr04*	1773951	C/G	NA	INTERGENIC	NA
Kabuli_wild_1783500	1399935936	*Ca_kabuli_Chr04*	1783500	T/C	Gene_Ca_07801	SYNONYMOUS_CODING	Protein kinase,catalytic domain
Kabuli_wild_1785705	1399935937	*Ca_kabuli_Chr04*	1785705	G/C	Gene_Ca_07801	INTRON	Protein kinase,catalytic domain
Kabuli_wild_1791439	1399935938	*Ca_kabuli_Chr04*	1791439	C/T	Gene_Ca_07801	INTRON	Protein kinase,catalytic domain
Kabuli_wild_1791448	1399935939	*Ca_kabuli_Chr04*	1791448	C/T	Gene_Ca_07801	INTRON	Protein kinase,catalytic domain

SNP IDs	NCBI dbSNP accession IDs (NCBI_SS#)	Chromosomes	Physical positions (bp)	SNPs	Gene accession IDs	Sequence components of genome	Putative functions
Kabuli_wild_1806745	1399935940	*Ca_kabuli_Chr04*	1806745	G/A	Gene_Ca_07801	INTRON	Protein kinase,catalytic domain
Kabuli_wild_1833725	1399935941	*Ca_kabuli_Chr04*	1833725	C/T	NA	INTERGENIC	NA
Kabuli_wild_1833872	1399935942	*Ca_kabuli_Chr04*	1833872	A/C	NA	INTERGENIC	NA
Kabuli_wild_1842147	1399935943	*Ca_kabuli_Chr04*	1842147	C/T	Gene_Ca_07806	DOWNSTREAM	Protein kinase,catalytic domain
Kabuli_wild_1844095	1399935944	*Ca_kabuli_Chr04*	1844095	G/C	Gene_Ca_07805	UPSTREAM	Oxoglutarate/iron-dependent oxygenase
Kabuli_wild_1867921	1399935945	*Ca_kabuli_Chr04*	1867921	G/A	Gene_Ca_07807	INTRON	Sulphate transporter/antisigma-factor antagonist STAS
Kabuli_wild_1869577	1399935946	*Ca_kabuli_Chr04*	1869577	C/T	Gene_Ca_07807	DOWNSTREAM	Sulphate transporter/antisigma-factor antagonist STAS
Kabuli_wild_1870317	1399935947	*Ca_kabuli_Chr04*	1870317	T/C	NA	INTERGENIC	NA
Kabuli_wild_1870333	1399935948	*Ca_kabuli_Chr04*	1870333	A/G	NA	INTERGENIC	NA
Kabuli_wild_1870666	1399935949	*Ca_kabuli_Chr04*	1870666	G/C	NA	INTERGENIC	NA
Kabuli_wild_1870802	1399935950	*Ca_kabuli_Chr04*	1870802	C/T	NA	INTERGENIC	NA
Kabuli_wild_1870806	1399935951	*Ca_kabuli_Chr04*	1870806	C/A	NA	INTERGENIC	NA
Kabuli_wild_1873733	1399935952	*Ca_kabuli_Chr04*	1873733	T/C	Gene_Ca_07808	NON_SYNONYMOUS_CODING	KIP1-like
Kabuli_wild_1873758	1399935953	*Ca_kabuli_Chr04*	1873758	A/G	Gene_Ca_07808	SYNONYMOUS_CODING	KIP1-like
Kabuli_wild_1941089	1399935954	*Ca_kabuli_Chr04*	1941089	C/T	NA	INTERGENIC	NA
Kabuli_wild_2008053	1399935955	*Ca_kabuli_Chr04*	2008053	A/C	Gene_Ca_07819	INTRON	NA
Kabuli_wild_2032449	1399935956	*Ca_kabuli_Chr04*	2032449	G/A	Gene_Ca_07822	SYNONYMOUS_CODING	RNA-processing protein, HAThelix
Kabuli_wild_2032475	1399935957	*Ca_kabuli_Chr04*	2032475	G/A	Gene_Ca_07822	INTRON	RNA-processing protein, HAThelix
Kabuli_wild_2032480	1399935958	*Ca_kabuli_Chr04*	2032480	C/T	Gene_Ca_07822	INTRON	RNA-processing protein, HAThelix
Kabuli_wild_2032481	1399935959	*Ca_kabuli_Chr04*	2032481	T/G	Gene_Ca_07822	INTRON	RNA-processing protein, HAThelix
Kabuli_wild_2032510	1399935960	*Ca_kabuli_Chr04*	2032510	T/A	Gene_Ca_07822	INTRON	RNA-processing protein, HAThelix
Kabuli_wild_2032529	1399935961	*Ca_kabuli_Chr04*	2032529	A/G	Gene_Ca_07822	INTRON	RNA-processing protein, HAThelix
Kabuli_wild_2032773	1399935962	*Ca_kabuli_Chr04*	2032773	T/A	Gene_Ca_07822	INTRON	RNA-processing protein, HAThelix
Kabuli_wild_2034266	1399935963	*Ca_kabuli_Chr04*	2034266	T/C	Gene_Ca_07822	SYNONYMOUS_CODING	RNA-processing protein, HAThelix
Kabuli_wild_2034304	1399935964	*Ca_kabuli_Chr04*	2034304	A/G	Gene_Ca_07822	SYNONYMOUS_CODING	RNA-processing protein, HAThelix
Kabuli_wild_2034340	1399935965	*Ca_kabuli_Chr04*	2034340	C/A	Gene_Ca_07822	SYNONYMOUS_CODING	RNA-processing protein, HAThelix
Kabuli_wild_2034385	1399935966	*Ca_kabuli_Chr04*	2034385	C/A	Gene_Ca_07822	INTRON	RNA-processing protein, HAThelix
Kabuli_wild_2034396	1399935967	*Ca_kabuli_Chr04*	2034396	A/G	Gene_Ca_07822	INTRON	RNA-processing protein, HAThelix
Kabuli_wild_2034398	1399935968	*Ca_kabuli_Chr04*	2034398	C/T	Gene_Ca_07822	INTRON	RNA-processing protein, HAThelix
Kabuli_wild_2034429	1399935969	*Ca_kabuli_Chr04*	2034429	C/A	Gene_Ca_07822	INTRON	RNA-processing protein, HAThelix
Kabuli_wild_2034739	1399935970	*Ca_kabuli_Chr04*	2034739	A/C	Gene_Ca_07822	INTRON	RNA-processing protein, HAThelix
Kabuli_wild_2035797	1399935971	*Ca_kabuli_Chr04*	2035797	G/A	Gene_Ca_07822	SYNONYMOUS_CODING	RNA-processing protein, HAThelix
Kabuli_wild_2035801	1399935972	*Ca_kabuli_Chr04*	2035801	G/A	Gene_Ca_07822	SYNONYMOUS_CODING	RNA-processing protein, HAThelix
Kabuli_wild_2035841	1399935973	*Ca_kabuli_Chr04*	2035841	C/T	Gene_Ca_07823	DOWNSTREAM	Reverse transcriptase, RNA-dependent DNA polymerase
Kabuli_wild_2035894	1399935974	*Ca_kabuli_Chr04*	2035894	C/A	Gene_Ca_07823	DOWNSTREAM	Reverse transcriptase, RNA-dependent DNA polymerase
Kabuli_wild_2113227	1399935975	*Ca_kabuli_Chr04*	2113227	C/T	NA	INTERGENIC	NA
Kabuli_wild_2125774	1399935976	*Ca_kabuli_Chr04*	2125774	G/A	Gene_Ca_07833	NON_SYNONYMOUS_CODING	RasGTPase
Kabuli_wild_2126436	1399935977	*Ca_kabuli_Chr04*	2126436	T/C	Gene_Ca_07833	INTRON	RasGTPase
Kabuli_wild_2213710	1399935978	*Ca_kabuli_Chr04*	2213710	G/A	NA	INTERGENIC	NA
Kabuli_wild_2311779	1399935979	*Ca_kabuli_Chr04*	2311779	T/G	Gene_Ca_07853	INTRON	Glucosylceramidase
Kabuli_wild_2311789	1399935980	*Ca_kabuli_Chr04*	2311789	T/G	Gene_Ca_07853	INTRON	Glucosylceramidase
Kabuli_wild_2311813	1399935981	*Ca_kabuli_Chr04*	2311813	G/A	Gene_Ca_07853	INTRON	Glucosylceramidase
Kabuli_wild_2361038	1399935982	*Ca_kabuli_Chr04*	2361038	C/T	Gene_Ca_07858	INTRON	ABC transporter-like
Kabuli_wild_2361088	1399935983	*Ca_kabuli_Chr04*	2361088	T/A	Gene_Ca_07858	INTRON	ABC transporter-like

SNP IDs	NCBI dbSNP accession IDs (NCBI_SS#)	Chromosomes	Physical positions (bp)	SNPs	Gene accession IDs	Sequence components of genome	Putative functions
Kabuli_wild_2362761	1399935984	*Ca_kabuli_Chr04*	2362761	T/A	Gene_Ca_07858	INTRON	ABC transporter-like
Kabuli_wild_2380206	1399935985	*Ca_kabuli_Chr04*	2380206	A/G	Gene_Ca_07859	DOWNSTREAM	PWWP
Kabuli_wild_2393033	1399935986	*Ca_kabuli_Chr04*	2393033	T/G	Gene_Ca_07861	INTRON	DNAhelicase,UvrD/REPtype
Kabuli_wild_2411925	1399935987	*Ca_kabuli_Chr04*	2411925	G/T	Gene_Ca_07861	INTRON	DNAhelicase,UvrD/REPtype
Kabuli_wild_2411967	1399935988	*Ca_kabuli_Chr04*	2411967	G/A	Gene_Ca_07861	INTRON	DNAhelicase,UvrD/REPtype
Kabuli_wild_2449947	1399935989	*Ca_kabuli_Chr04*	2449947	C/T	Gene_Ca_07864	SYNONYMOUS_CODING	NA
Kabuli_wild_2609945	1399935990	*Ca_kabuli_Chr04*	2609945	C/T	Gene_Ca_12061	DOWNSTREAM	Acidphosphatase(ClassB)
Kabuli_wild_2632382	1399935991	*Ca_kabuli_Chr04*	2632382	T/G	Gene_Ca_12066	SYNONYMOUS_CODING	RNA recognition motif domain
Kabuli_wild_2632395	1399935992	*Ca_kabuli_Chr04*	2632395	A/G	Gene_Ca_12066	NON_SYNONYMOUS_CODING	Signal transduction response regulator, receiverdomain
Kabuli_wild_2676174	1399935993	*Ca_kabuli_Chr04*	2676174	C/T	Gene_Ca_12071	SYNONYMOUS_CODING	Actin-like
Kabuli_wild_2726353	1399935994	*Ca_kabuli_Chr04*	2726353	G/A	Gene_Ca_12075	INTRON	General substrate transporter
Kabuli_wild_2886775	1399935995	*Ca_kabuli_Chr04*	2886775	T/G	Gene_Ca_12085	SYNONYMOUS_CODING	Thioredoxin domain
Kabuli_wild_2907919	1399935996	*Ca_kabuli_Chr04*	2907919	G/A	Gene_Ca_12088	UPSTREAM	Tetratrico peptide TPR-1
Kabuli_wild_3037899	1399935997	*Ca_kabuli_Chr04*	3037899	A/C	NA	INTERGENIC	NA
Kabuli_wild_3037929	1399935998	*Ca_kabuli_Chr04*	3037929	T/G	NA	INTERGENIC	NA
Kabuli_wild_3037986	1399935999	*Ca_kabuli_Chr04*	3037986	T/A	NA	INTERGENIC	NA
Kabuli_wild_3038022	1399936000	*Ca_kabuli_Chr04*	3038022	C/T	NA	INTERGENIC	NA
Kabuli_wild_3046920	1399936001	*Ca_kabuli_Chr04*	3046920	A/G	Gene_Ca_12103	INTRON	NA
Kabuli_wild_3158321	1399936002	*Ca_kabuli_Chr04*	3158321	A/G	NA	INTERGENIC	NA
Kabuli_wild_3158323	1399936003	*Ca_kabuli_Chr04*	3158323	T/G	NA	INTERGENIC	NA
Kabuli_wild_3158328	1399936004	*Ca_kabuli_Chr04*	3158328	G/T	NA	INTERGENIC	NA
Kabuli_wild_3158331	1399936005	*Ca_kabuli_Chr04*	3158331	A/T	NA	INTERGENIC	NA
Kabuli_wild_3158426	1399936006	*Ca_kabuli_Chr04*	3158426	G/A	NA	INTERGENIC	NA
Kabuli_wild_3158504	1399936007	*Ca_kabuli_Chr04*	3158504	A/T	NA	INTERGENIC	NA
Kabuli_wild_3158525	1399936008	*Ca_kabuli_Chr04*	3158525	A/G	NA	INTERGENIC	NA
Kabuli_wild_3158549	1399936009	*Ca_kabuli_Chr04*	3158549	A/G	NA	INTERGENIC	NA
Kabuli_wild_3164083	1399936010	*Ca_kabuli_Chr04*	3164083	G/A	Gene_Ca_12117	DOWNSTREAM	NA
Kabuli_wild_3164168	1399936011	*Ca_kabuli_Chr04*	3164168	G/A	Gene_Ca_12117	DOWNSTREAM	NA
Kabuli_wild_3164195	1399936012	*Ca_kabuli_Chr04*	3164195	T/C	Gene_Ca_12117	DOWNSTREAM	NA
Kabuli_wild_3164223	1399936013	*Ca_kabuli_Chr04*	3164223	C/T	Gene_Ca_12117	DOWNSTREAM	NA
Kabuli_wild_3166657	1399936014	*Ca_kabuli_Chr04*	3166657	C/T	Gene_Ca_12117	INTRON	NA
Kabuli_wild_3166663	1399936015	*Ca_kabuli_Chr04*	3166663	C/T	Gene_Ca_12117	INTRON	NA
Kabuli_wild_3166674	1399936016	*Ca_kabuli_Chr04*	3166674	G/T	Gene_Ca_12117	INTRON	NA
Kabuli_wild_3171162	1399936017	*Ca_kabuli_Chr04*	3171162	G/A	Gene_Ca_12117	SYNONYMOUS_CODING	NA
Kabuli_wild_3171358	1399936018	*Ca_kabuli_Chr04*	3171358	A/T	Gene_Ca_12117	INTRON	NA
Kabuli_wild_3171385	1399936019	*Ca_kabuli_Chr04*	3171385	A/G	Gene_Ca_12117	INTRON	NA
Kabuli_wild_3171428	1399936020	*Ca_kabuli_Chr04*	3171428	T/C	Gene_Ca_12117	INTRON	NA
Kabuli_wild_3171551	1399936021	*Ca_kabuli_Chr04*	3171551	C/T	Gene_Ca_12117	INTRON	NA
Kabuli_wild_3245405	1399936022	*Ca_kabuli_Chr04*	3245405	C/T	Gene_Ca_12124	INTRON	Protein kinase,catalytic domain
Kabuli_wild_3264351	1399936023	*Ca_kabuli_Chr04*	3264351	T/C	Gene_Ca_12126	SYNONYMOUS_CODING	Domain of unknown function DUF640
Kabuli_wild_3280217	1399936024	*Ca_kabuli_Chr04*	3280217	A/G	NA	INTERGENIC	NA
Kabuli_wild_3281050	1399936025	*Ca_kabuli_Chr04*	3281050	T/G	NA	INTERGENIC	NA
Kabuli_wild_3290298	1399936026	*Ca_kabuli_Chr04*	3290298	T/G	NA	INTERGENIC	NA
Kabuli_wild_3290400	1399936027	*Ca_kabuli_Chr04*	3290400	A/C	NA	INTERGENIC	NA

SNP IDs	NCBI dbSNP accession IDs (NCBI_SS#)	Chromosomes	Physical positions (bp)	SNPs	Gene accession IDs	Sequence components of genome	Putative functions
Kabuli_wild_3307908	1399936028	*Ca_kabuli_Chr04*	3307908	A/T	Gene_Ca_12131	INTRON	Actin-binding,cofilin/tropomyosintype
Kabuli_wild_3353586	1399936029	*Ca_kabuli_Chr04*	3353586	G/A	Gene_Ca_12138	INTRON	Mg2+transporterprotein,CorA-like
Kabuli_wild_3356242	1399936030	*Ca_kabuli_Chr04*	3356242	C/G	NA	INTERGENIC	NA
Kabuli_wild_3356252	1399936031	*Ca_kabuli_Chr04*	3356252	C/G	NA	INTERGENIC	NA
Kabuli_wild_3400828	1399936032	*Ca_kabuli_Chr04*	3400828	G/C	Gene_Ca_12141	INTRON	Glycosidehydrolase,family47
Kabuli_wild_3427396	1399936033	*Ca_kabuli_Chr04*	3427396	A/G	Gene_Ca_12144	INTRON	Castor/Polluxionchannel
Kabuli_wild_3428929	1399936034	*Ca_kabuli_Chr04*	3428929	T/C	Gene_Ca_12144	SYNONYMOUS_CODING	Castor/Polluxionchannel
Kabuli_wild_3429035	1399936035	*Ca_kabuli_Chr04*	3429035	A/G	Gene_Ca_12144	INTRON	Castor/Polluxionchannel
Kabuli_wild_3429059	1399936036	*Ca_kabuli_Chr04*	3429059	A/C	Gene_Ca_12144	INTRON	Castor/Polluxionchannel
Kabuli_wild_3453072	1399936037	*Ca_kabuli_Chr04*	3453072	A/G	NA	INTERGENIC	NA
Kabuli_wild_3453084	1399936038	*Ca_kabuli_Chr04*	3453084	C/G	NA	INTERGENIC	NA
Kabuli_wild_3453306	1399936039	*Ca_kabuli_Chr04*	3453306	G/A	NA	INTERGENIC	NA
Kabuli_wild_3453320	1399936040	*Ca_kabuli_Chr04*	3453320	G/A	Gene_Ca_12146	SYNONYMOUS_CODING	Armadillo
Kabuli_wild_3453350	1399936041	*Ca_kabuli_Chr04*	3453350	T/A	Gene_Ca_12146	SYNONYMOUS_CODING	Armadillo
Kabuli_wild_3455047	1399936042	*Ca_kabuli_Chr04*	3455047	G/A	Gene_Ca_12146	NON_SYNONYMOUS_CODING	Armadillo
Kabuli_wild_3456434	1399936043	*Ca_kabuli_Chr04*	3456434	C/T	Gene_Ca_12146	NON_SYNONYMOUS_CODING	Armadillo
Kabuli_wild_3689840	1399936044	*Ca_kabuli_Chr04*	3689840	T/C	Gene_Ca_12165	INTRON	ATPase,AAA+type,core
Kabuli_wild_3735377	1399936045	*Ca_kabuli_Chr04*	3735377	T/C	NA	INTERGENIC	NA
Kabuli_wild_3741522	1399936046	*Ca_kabuli_Chr04*	3741522	T/G	NA	INTERGENIC	NA
Kabuli_wild_3741539	1399936047	*Ca_kabuli_Chr04*	3741539	T/G	NA	INTERGENIC	NA
Kabuli_wild_3762559	1399936048	*Ca_kabuli_Chr04*	3762559	T/C	NA	INTERGENIC	NA
Kabuli_wild_3762561	1399936049	*Ca_kabuli_Chr04*	3762561	C/A	NA	INTERGENIC	NA
Kabuli_wild_3762585	1399936050	*Ca_kabuli_Chr04*	3762585	T/G	NA	INTERGENIC	NA
Kabuli_wild_3764547	1399936051	*Ca_kabuli_Chr04*	3764547	T/G	Gene_Ca_12178	DOWNSTREAM	NA
Kabuli_wild_3794764	1399936052	*Ca_kabuli_Chr04*	3794764	G/A	Gene_Ca_12180	DOWNSTREAM	NA
Kabuli_wild_3794885	1399936053	*Ca_kabuli_Chr04*	3794885	C/A	Gene_Ca_12180	DOWNSTREAM	NA
Kabuli_wild_3888185	1399936054	*Ca_kabuli_Chr04*	3888185	A/G	Gene_Ca_03864	UPSTREAM	NA
Kabuli_wild_3888328	1399936055	*Ca_kabuli_Chr04*	3888328	C/T	Gene_Ca_03864	UPSTREAM	NA
Kabuli_wild_3888529	1399936056	*Ca_kabuli_Chr04*	3888529	T/G	Gene_Ca_03864	UPSTREAM	NA
Kabuli_wild_3888535	1399936057	*Ca_kabuli_Chr04*	3888535	T/G	Gene_Ca_03864	UPSTREAM	NA
Kabuli_wild_4027829	1399936058	*Ca_kabuli_Chr04*	4027829	A/T	NA	INTERGENIC	NA
Kabuli_wild_4027872	1399936059	*Ca_kabuli_Chr04*	4027872	A/C	NA	INTERGENIC	NA
Kabuli_wild_4097935	1399936060	*Ca_kabuli_Chr04*	4097935	T/C	Gene_Ca_03846	INTRON	UBX
Kabuli_wild_4097989	1399936061	*Ca_kabuli_Chr04*	4097989	G/A	Gene_Ca_03846	SYNONYMOUS_CODING	UBX
Kabuli_wild_4144618	1399936062	*Ca_kabuli_Chr04*	4144618	A/G	NA	INTERGENIC	NA
Kabuli_wild_4148061	1399936063	*Ca_kabuli_Chr04*	4148061	T/C	Gene_Ca_03841	SYNONYMOUS_CODING	Plan tspecific mitochondrial import receptor subunit TOM20
Kabuli_wild_4148250	1399936064	*Ca_kabuli_Chr04*	4148250	A/G	Gene_Ca_03841	INTRON	Plant specific mitochondrial import receptor subunit TOM21
Kabuli_wild_4154507	1399936065	*Ca_kabuli_Chr04*	4154507	G/T	Gene_Ca_03840	INTRON	NA
Kabuli_wild_4305891	1399936066	*Ca_kabuli_Chr04*	4305891	A/C	Gene_Ca_03829	SYNONYMOUS_CODING	Inositolpolyphosphate-relatedphosphatase
Kabuli_wild_4327229	1399936067	*Ca_kabuli_Chr04*	4327229	A/G	Gene_Ca_03827	INTRON	RNA recognition motif domain
Kabuli_wild_4410351	1399936068	*Ca_kabuli_Chr04*	4410351	T/C	Gene_Ca_03820	SYNONYMOUS_CODING	Single-strandednucleicacidbindingR3H
Kabuli_wild_4426659	1399936069	*Ca_kabuli_Chr04*	4426659	C/T	NA	INTERGENIC	NA
Kabuli_wild_4426796	1399936070	*Ca_kabuli_Chr04*	4426796	A/C	NA	INTERGENIC	NA
Kabuli_wild_4452018	1399936071	*Ca_kabuli_Chr04*	4452018	T/A	Gene_Ca_03812	DOWNSTREAM	Armadillo

SNP IDs	NCBI dbSNP accession IDs (NCBI_SS#)	Chromosomes	Physical positions (bp)	SNPs	Gene accession IDs	Sequence components of genome	Putative functions
Kabuli_wild_4452380	1399936072	*Ca_kabuli_Chr04*	4452380	C/G	Gene_Ca_03812	DOWNSTREAM	Armadillo
Kabuli_wild_4454305	1399936073	*Ca_kabuli_Chr04*	4454305	A/C	Gene_Ca_03813	DOWNSTREAM	Protein kinase,catalytic domain
Kabuli_wild_4454464	1399936074	*Ca_kabuli_Chr04*	4454464	A/G	NA	INTERGENIC	NA
Kabuli_wild_4454537	1399936075	*Ca_kabuli_Chr04*	4454537	C/T	NA	INTERGENIC	NA
Kabuli_wild_4454608	1399936076	*Ca_kabuli_Chr04*	4454608	C/T	NA	INTERGENIC	NA
Kabuli_wild_4461562	1399936077	*Ca_kabuli_Chr04*	4461562	G/C	Gene_Ca_03811	INTRON	TRM13/UPF0224family,U11-48K-likeCHHCZinc fingerdomain
Kabuli_wild_4468394	1399936078	*Ca_kabuli_Chr04*	4468394	C/T	Gene_Ca_03810	INTRON	TetratricopeptideTPR-1
Kabuli_wild_4535519	1399936079	*Ca_kabuli_Chr04*	4535519	T/G	NA	INTERGENIC	NA
Kabuli_wild_4535611	1399936080	*Ca_kabuli_Chr04*	4535611	A/T	NA	INTERGENIC	NA
Kabuli_wild_4536286	1399936081	*Ca_kabuli_Chr04*	4536286	G/C	NA	INTERGENIC	NA
Kabuli_wild_4536310	1399936082	*Ca_kabuli_Chr04*	4536310	A/G	NA	INTERGENIC	NA
Kabuli_wild_4578593	1399936083	*Ca_kabuli_Chr04*	4578593	G/T	NA	INTERGENIC	NA
Kabuli_wild_4578604	1399936084	*Ca_kabuli_Chr04*	4578604	C/A	NA	INTERGENIC	NA
Kabuli_wild_4578836	1399936085	*Ca_kabuli_Chr04*	4578836	C/T	NA	INTERGENIC	NA
Kabuli_wild_4579158	1399936086	*Ca_kabuli_Chr04*	4579158	A/G	NA	INTERGENIC	NA
Kabuli_wild_4579412	1399936087	*Ca_kabuli_Chr04*	4579412	C/T	NA	INTERGENIC	NA
Kabuli_wild_4605340	1399936088	*Ca_kabuli_Chr04*	4605340	A/C	NA	INTERGENIC	NA
Kabuli_wild_4628243	1399936089	*Ca_kabuli_Chr04*	4628243	G/A	Gene_Ca_03793	DOWNSTREAM	ASCH domain
Kabuli_wild_4628250	1399936090	*Ca_kabuli_Chr04*	4628250	T/C	Gene_Ca_03793	DOWNSTREAM	ASCH domain
Kabuli_wild_4630407	1399936091	*Ca_kabuli_Chr04*	4630407	G/A	NA	INTERGENIC	NA
Kabuli_wild_4633913	1399936092	*Ca_kabuli_Chr04*	4633913	T/G	Gene_Ca_03793	UPSTREAM	ASCH domain
Kabuli_wild_4634542	1399936093	*Ca_kabuli_Chr04*	4634542	A/T	NA	INTERGENIC	NA
Kabuli_wild_4634659	1399936094	*Ca_kabuli_Chr04*	4634659	G/A	NA	INTERGENIC	NA
Kabuli_wild_4634817	1399936095	*Ca_kabuli_Chr04*	4634817	C/T	NA	INTERGENIC	NA
Kabuli_wild_4670913	1399936096	*Ca_kabuli_Chr04*	4670913	A/G	Gene_Ca_03789	INTRON	EF-Hand1,calcium-bindingsite
Kabuli_wild_4675166	1399936097	*Ca_kabuli_Chr04*	4675166	A/T	NA	INTERGENIC	NA
Kabuli_wild_4675171	1399936098	*Ca_kabuli_Chr04*	4675171	T/A	NA	INTERGENIC	NA
Kabuli_wild_4675172	1399936099	*Ca_kabuli_Chr04*	4675172	T/A	NA	INTERGENIC	NA
Kabuli_wild_4675176	1399936100	*Ca_kabuli_Chr04*	4675176	T/G	NA	INTERGENIC	NA
Kabuli_wild_4675177	1399936101	*Ca_kabuli_Chr04*	4675177	T/A	NA	INTERGENIC	NA
Kabuli_wild_4675181	1399936102	*Ca_kabuli_Chr04*	4675181	A/C	NA	INTERGENIC	NA
Kabuli_wild_4675182	1399936103	*Ca_kabuli_Chr04*	4675182	T/G	NA	INTERGENIC	NA
Kabuli_wild_4679236	1399936104	*Ca_kabuli_Chr04*	4679236	A/G	Gene_Ca_03788	SYNONYMOUS_CODING	Pentatrico peptide repeat
Kabuli_wild_4680896	1399936105	*Ca_kabuli_Chr04*	4680896	A/G	Gene_Ca_03788	UPSTREAM	Pentatrico peptide repeat
Kabuli_wild_4746818	1399936106	*Ca_kabuli_Chr04*	4746818	T/C	Gene_Ca_03781	SYNONYMOUS_CODING	SET domain
Kabuli_wild_4799116	1399936107	*Ca_kabuli_Chr04*	4799116	C/T	Gene_Ca_03778	NON_SYNONYMOUS_CODING	SNF2-related
Kabuli_wild_4799122	1399936108	*Ca_kabuli_Chr04*	4799122	C/T	Gene_Ca_03778	NON_SYNONYMOUS_CODING	SNF2-related
Kabuli_wild_4799146	1399936109	*Ca_kabuli_Chr04*	4799146	C/T	Gene_Ca_03778	NON_SYNONYMOUS_CODING	SNF2-related
Kabuli_wild_4828832	1399936110	*Ca_kabuli_Chr04*	4828832	T/A	NA	INTERGENIC	NA
Kabuli_wild_4896153	1399936111	*Ca_kabuli_Chr04*	4896153	A/T	NA	INTERGENIC	NA
Kabuli_wild_4907987	1399936112	*Ca_kabuli_Chr04*	4907987	G/A	NA	INTERGENIC	NA
Kabuli_wild_4911083	1399936113	*Ca_kabuli_Chr04*	4911083	T/C	Gene_Ca_03767	INTRON	Phosphoribosyl amino imidazolecarboxylase,core
Kabuli_wild_4911130	1399936114	*Ca_kabuli_Chr04*	4911130	T/C	Gene_Ca_03767	INTRON	Phosphoribosyl amino imidazolecarboxylase,core
Kabuli_wild_4911209	1399936115	*Ca_kabuli_Chr04*	4911209	A/G	Gene_Ca_03767	SYNONYMOUS_CODING	Phosphoribosyl amino imidazolecarboxylase,core

SNP IDs	NCBI dbSNP accession IDs (NCBI_SS#)	Chromosomes	Physical positions (bp)	SNPs	Gene accession IDs	Sequence components of genome	Putative functions
Kabuli_wild_4911217	1399936116	*Ca_kabuli_Chr04*	4911217	A/C	Gene_Ca_03767	SYNONYMOUS_CODING	Phosphoribosyl amino imidazolecarboxylase,core
Kabuli_wild_4920706	1399936117	*Ca_kabuli_Chr04*	4920706	T/C	Gene_Ca_03766	NON_SYNONYMOUS_CODING	Zinc finger,PMZ-type
Kabuli_wild_4920712	1399936118	*Ca_kabuli_Chr04*	4920712	A/T	Gene_Ca_03766	NON_SYNONYMOUS_CODING	Zinc finger,PMZ-type
Kabuli_wild_4920719	1399936119	*Ca_kabuli_Chr04*	4920719	A/G	Gene_Ca_03766	NON_SYNONYMOUS_CODING	Zinc finger,PMZ-type
Kabuli_wild_4922410	1399936120	*Ca_kabuli_Chr04*	4922410	A/C	Gene_Ca_03766	DOWNSTREAM	Zinc finger,PMZ-type
Kabuli_wild_4928764	1399936121	*Ca_kabuli_Chr04*	4928764	T/C	NA	INTERGENIC	NA
Kabuli_wild_4928890	1399936122	*Ca_kabuli_Chr04*	4928890	A/G	NA	INTERGENIC	NA
Kabuli_wild_4953629	1399936123	*Ca_kabuli_Chr04*	4953629	C/A	NA	INTERGENIC	NA
Kabuli_wild_5038300	1399936124	*Ca_kabuli_Chr04*	5038300	G/C	NA	INTERGENIC	NA
Kabuli_wild_5050390	1399936125	*Ca_kabuli_Chr04*	5050390	T/C	NA	INTERGENIC	NA
Kabuli_wild_5155647	1399936126	*Ca_kabuli_Chr04*	5155647	C/A	NA	INTERGENIC	NA
Kabuli_wild_5186401	1399936127	*Ca_kabuli_Chr04*	5186401	A/G	Gene_Ca_03734	NON_SYNONYMOUS_CODING	Pyridoxalphosphate-dependent enzyme,beta subunit
Kabuli_wild_5204288	1399936128	*Ca_kabuli_Chr04*	5204288	G/A	Gene_Ca_03733	INTRON	Domain of unknown function DUF1981,SEC7 associated
Kabuli_wild_5204319	1399936129	*Ca_kabuli_Chr04*	5204319	A/G	Gene_Ca_03733	INTRON	Domain of unknown function DUF1981,SEC7 associated
Kabuli_wild_5222851	1399936130	*Ca_kabuli_Chr04*	5222851	C/T	Gene_Ca_03733	INTRON	Domain of unknown function DUF1981,SEC7 associated
Kabuli_wild_5223068	1399936131	*Ca_kabuli_Chr04*	5223068	A/G	Gene_Ca_03733	INTRON	Domain of unknown function DUF1981,SEC7 associated
Kabuli_wild_5223100	1399936132	*Ca_kabuli_Chr04*	5223100	G/A	Gene_Ca_03733	INTRON	Domain of unknown function DUF1981,SEC7 associated
Kabuli_wild_5232364	1399936133	*Ca_kabuli_Chr04*	5232364	T/C	NA	INTERGENIC	NA
Kabuli_wild_5258348	1399936134	*Ca_kabuli_Chr04*	5258348	T/A	Gene_Ca_03730	DOWNSTREAM	Protein kinase,catalytic domain
Kabuli_wild_5258351	1399936135	*Ca_kabuli_Chr04*	5258351	C/A	Gene_Ca_03730	DOWNSTREAM	Protein kinase,catalytic domain
Kabuli_wild_5258355	1399936136	*Ca_kabuli_Chr04*	5258355	C/A	Gene_Ca_03730	DOWNSTREAM	Protein kinase,catalytic domain
Kabuli_wild_5263199	1399936137	*Ca_kabuli_Chr04*	5263199	A/G	NA	INTERGENIC	NA
Kabuli_wild_5268995	1399936138	*Ca_kabuli_Chr04*	5268995	A/C	NA	INTERGENIC	NA
Kabuli_wild_5282131	1399936139	*Ca_kabuli_Chr04*	5282131	T/C	NA	INTERGENIC	NA
Kabuli_wild_5306231	1399936140	*Ca_kabuli_Chr04*	5306231	G/A	Gene_Ca_03727	DOWNSTREAM	IsopenicillinNsynthase
Kabuli_wild_5390317	1399936141	*Ca_kabuli_Chr04*	5390317	G/A	NA	INTERGENIC	NA
Kabuli_wild_5473174	1399936142	*Ca_kabuli_Chr04*	5473174	C/T	NA	INTERGENIC	NA
Kabuli_wild_5484220	1399936143	*Ca_kabuli_Chr04*	5484220	A/G	NA	INTERGENIC	NA
Kabuli_wild_5484236	1399936144	*Ca_kabuli_Chr04*	5484236	C/T	NA	INTERGENIC	NA
Kabuli_wild_5484238	1399936145	*Ca_kabuli_Chr04*	5484238	G/A	NA	INTERGENIC	NA
Kabuli_wild_5484257	1399936146	*Ca_kabuli_Chr04*	5484257	G/C	NA	INTERGENIC	NA
Kabuli_wild_5484259	1399936147	*Ca_kabuli_Chr04*	5484259	G/T	NA	INTERGENIC	NA
Kabuli_wild_5484979	1399936148	*Ca_kabuli_Chr04*	5484979	T/G	NA	INTERGENIC	NA
Kabuli_wild_5485013	1399936149	*Ca_kabuli_Chr04*	5485013	A/G	NA	INTERGENIC	NA
Kabuli_wild_5485029	1399936150	*Ca_kabuli_Chr04*	5485029	C/T	NA	INTERGENIC	NA
Kabuli_wild_5485031	1399936151	*Ca_kabuli_Chr04*	5485031	G/A	NA	INTERGENIC	NA
Kabuli_wild_5485050	1399936152	*Ca_kabuli_Chr04*	5485050	G/C	NA	INTERGENIC	NA
Kabuli_wild_5485052	1399936153	*Ca_kabuli_Chr04*	5485052	G/T	NA	INTERGENIC	NA
Kabuli_wild_5528418	1399936154	*Ca_kabuli_Chr04*	5528418	T/G	Gene_Ca_03702	SYNONYMOUS_CODING	Zinc finger, RING-type
Kabuli_wild_5528421	1399936155	*Ca_kabuli_Chr04*	5528421	T/G	Gene_Ca_03702	SYNONYMOUS_CODING	Zinc finger, RING-type
Kabuli_wild_5528469	1399936156	*Ca_kabuli_Chr04*	5528469	T/G	Gene_Ca_03702	SYNONYMOUS_CODING	Zinc finger, RING-type
Kabuli_wild_5561063	1399936157	*Ca_kabuli_Chr04*	5561063	A/G	Gene_Ca_03699	SYNONYMOUS_CODING	Heavymetal transport/detoxification protein
Kabuli_wild_5701873	1399936158	*Ca_kabuli_Chr04*	5701873	A/C	NA	INTERGENIC	NA
Kabuli_wild_5701900	1399936159	*Ca_kabuli_Chr04*	5701900	G/T	NA	INTERGENIC	NA

SNP IDs	NCBI dbSNP accession IDs (NCBI_SS#)	Chromosomes	Physical positions (bp)	SNPs	Gene accession IDs	Sequence components of genome	Putative functions
Kabuli_wild_5701902	1399936160	*Ca_kabuli_Chr04*	5701902	C/T	NA	INTERGENIC	NA
Kabuli_wild_5701907	1399936161	*Ca_kabuli_Chr04*	5701907	G/T	NA	INTERGENIC	NA
Kabuli_wild_5779698	1399936162	*Ca_kabuli_Chr04*	5779698	A/G	Gene_Ca_03672	DOWNSTREAM	Helicase,C-terminal
Kabuli_wild_5787722	1399936163	*Ca_kabuli_Chr04*	5787722	G/A	NA	INTERGENIC	NA
Kabuli_wild_5787727	1399936164	*Ca_kabuli_Chr04*	5787727	G/A	NA	INTERGENIC	NA
Kabuli_wild_5853798	1399936165	*Ca_kabuli_Chr04*	5853798	T/G	Gene_Ca_03664	INTRON	Zinc finger, RING-type
Kabuli_wild_5900290	1399936166	*Ca_kabuli_Chr04*	5900290	T/C	Gene_Ca_03661	DOWNSTREAM	Hypoxiainducedprotein,domain
Kabuli_wild_5905385	1399936167	*Ca_kabuli_Chr04*	5905385	G/A	NA	INTERGENIC	NA
Kabuli_wild_5907409	1399936168	*Ca_kabuli_Chr04*	5907409	T/G	Gene_Ca_03658	UPSTREAM	Dormancy auxin associated
Kabuli_wild_5907421	1399936169	*Ca_kabuli_Chr04*	5907421	T/G	Gene_Ca_03658	UPSTREAM	Dormancy auxin associated
Kabuli_wild_5907465	1399936170	*Ca_kabuli_Chr04*	5907465	C/A	Gene_Ca_03658	UPSTREAM	Dormancy auxin associated
Kabuli_wild_5999964	1399936171	*Ca_kabuli_Chr04*	5999964	T/C	Gene_Ca_03648	INTRON	WD40 repeat
Kabuli_wild_5999985	1399936172	*Ca_kabuli_Chr04*	5999985	T/C	Gene_Ca_03648	INTRON	WD40 repeat
Kabuli_wild_6000010	1399936173	*Ca_kabuli_Chr04*	6000010	T/A	Gene_Ca_03648	INTRON	WD40 repeat
Kabuli_wild_6000013	1399936174	*Ca_kabuli_Chr04*	6000013	A/C	Gene_Ca_03648	INTRON	WD40 repeat
Kabuli_wild_6000037	1399936175	*Ca_kabuli_Chr04*	6000037	T/G	Gene_Ca_03648	INTRON	WD40 repeat
Kabuli_wild_6000046	1399936176	*Ca_kabuli_Chr04*	6000046	T/C	Gene_Ca_03648	INTRON	WD40 repeat
Kabuli_wild_6041117	1399936177	*Ca_kabuli_Chr04*	6041117	C/T	NA	INTERGENIC	NA
Kabuli_wild_6041120	1399936178	*Ca_kabuli_Chr04*	6041120	G/T	NA	INTERGENIC	NA
Kabuli_wild_6041126	1399936179	*Ca_kabuli_Chr04*	6041126	A/G	NA	INTERGENIC	NA
Kabuli_wild_6041130	1399936180	*Ca_kabuli_Chr04*	6041130	C/A	NA	INTERGENIC	NA
Kabuli_wild_6041134	1399936181	*Ca_kabuli_Chr04*	6041134	A/C	NA	INTERGENIC	NA
Kabuli_wild_6134389	1399936182	*Ca_kabuli_Chr04*	6134389	A/C	NA	INTERGENIC	NA
Kabuli_wild_6157625	1399936183	*Ca_kabuli_Chr04*	6157625	T/C	Gene_Ca_03628	NON_SYNONYMOUS_CODING	PeptidaseS16,Lonprotease,C-terminal
Kabuli_wild_6201137	1399936184	*Ca_kabuli_Chr04*	6201137	T/C	NA	INTERGENIC	NA
Kabuli_wild_6201162	1399936185	*Ca_kabuli_Chr04*	6201162	A/C	NA	INTERGENIC	NA
Kabuli_wild_6264731	1399936186	*Ca_kabuli_Chr04*	6264731	G/A	NA	INTERGENIC	NA
Kabuli_wild_6281228	1399936187	*Ca_kabuli_Chr04*	6281228	A/C	NA	INTERGENIC	NA
Kabuli_wild_6387938	1399936188	*Ca_kabuli_Chr04*	6387938	T/G	NA	INTERGENIC	NA
Kabuli_wild_6403910	1399936189	*Ca_kabuli_Chr04*	6403910	A/C	Gene_Ca_03599	DOWNSTREAM	NA
Kabuli_wild_6408543	1399936190	*Ca_kabuli_Chr04*	6408543	T/G	NA	INTERGENIC	NA
Kabuli_wild_6544337	1399936191	*Ca_kabuli_Chr04*	6544337	A/C	NA	INTERGENIC	NA
Kabuli_wild_6544342	1399936192	*Ca_kabuli_Chr04*	6544342	A/C	NA	INTERGENIC	NA
Kabuli_wild_6680212	1399936193	*Ca_kabuli_Chr04*	6680212	C/T	Gene_Ca_03578	INTRON	NA
Kabuli_wild_6688218	1399936194	*Ca_kabuli_Chr04*	6688218	G/A	Gene_Ca_03578	DOWNSTREAM	NA
Kabuli_wild_6749550	1399936195	*Ca_kabuli_Chr04*	6749550	G/A	NA	INTERGENIC	NA
Kabuli_wild_6759998	1399936196	*Ca_kabuli_Chr04*	6759998	T/C	Gene_Ca_03565	INTRON	Pectinacetylesterase
Kabuli_wild_6761778	1399936197	*Ca_kabuli_Chr04*	6761778	C/A	Gene_Ca_03565	STOP_GAINED	Pectinacetylesterase
Kabuli_wild_6765884	1399936198	*Ca_kabuli_Chr04*	6765884	T/C	Gene_Ca_03564	NON_SYNONYMOUS_CODING	SANT domain,DNA binding
Kabuli_wild_6801632	1399936199	*Ca_kabuli_Chr04*	6801632	C/A	NA	INTERGENIC	NA
Kabuli_wild_6841854	1399936200	*Ca_kabuli_Chr04*	6841854	G/A	NA	INTERGENIC	NA
Kabuli_wild_6841856	1399936201	*Ca_kabuli_Chr04*	6841856	G/A	NA	INTERGENIC	NA
Kabuli_wild_6841857	1399936202	*Ca_kabuli_Chr04*	6841857	A/C	NA	INTERGENIC	NA
Kabuli_wild_6841858	1399936203	*Ca_kabuli_Chr04*	6841858	G/A	NA	INTERGENIC	NA

SNP IDs	NCBI dbSNP accession IDs (NCBI_SS#)	Chromosomes	Physical positions (bp)	SNPs	Gene accession IDs	Sequence components of genome	Putative functions
Kabuli_wild_6841859	1399936204	*Ca_kabuli_Chr04*	6841859	A/C	NA	INTERGENIC	NA
Kabuli_wild_6841860	1399936205	*Ca_kabuli_Chr04*	6841860	G/A	NA	INTERGENIC	NA
Kabuli_wild_6841862	1399936206	*Ca_kabuli_Chr04*	6841862	G/A	NA	INTERGENIC	NA
Kabuli_wild_6854530	1399936207	*Ca_kabuli_Chr04*	6854530	C/T	Gene_Ca_03554	INTRON	ABC transporter,trans membrane domain
Kabuli_wild_6854533	1399936208	*Ca_kabuli_Chr04*	6854533	C/A	Gene_Ca_03554	INTRON	ABC transporter,trans membrane domain
Kabuli_wild_6889964	1399936209	*Ca_kabuli_Chr04*	6889964	T/G	NA	INTERGENIC	NA
Kabuli_wild_6889974	1399936210	*Ca_kabuli_Chr04*	6889974	T/G	NA	INTERGENIC	NA
Kabuli_wild_6889994	1399936211	*Ca_kabuli_Chr04*	6889994	T/G	Gene_Ca_03550	NON_SYNONYMOUS_CODING	Pectinesterase,catalytic
Kabuli_wild_6890000	1399936212	*Ca_kabuli_Chr04*	6890000	A/G	Gene_Ca_03550	SYNONYMOUS_CODING	Pectinesterase,catalytic
Kabuli_wild_6890005	1399936213	*Ca_kabuli_Chr04*	6890005	C/T	Gene_Ca_03550	NON_SYNONYMOUS_CODING	Pectinesterase,catalytic
Kabuli_wild_6890014	1399936214	*Ca_kabuli_Chr04*	6890014	G/T	Gene_Ca_03550	NON_SYNONYMOUS_CODING	Pectinesterase,catalytic
Kabuli_wild_6895804	1399936215	*Ca_kabuli_Chr04*	6895804	G/A	Gene_Ca_03549	SYNONYMOUS_CODING	Protein kinase,catalytic domain
Kabuli_wild_6919896	1399936216	*Ca_kabuli_Chr04*	6919896	C/T	Gene_Ca_03548	NON_SYNONYMOUS_CODING	Transcription factor ,SBP-box
Kabuli_wild_6969799	1399936217	*Ca_kabuli_Chr04*	6969799	T/C	NA	INTERGENIC	NA
Kabuli_wild_7142498	1399936218	*Ca_kabuli_Chr04*	7142498	G/A	Gene_Ca_03523	DOWNSTREAM	Cytochromec-552/DMSOreductase-like,haem-binding domain
Kabuli_wild_7143238	1399936219	*Ca_kabuli_Chr04*	7143238	C/T	NA	INTERGENIC	NA
Kabuli_wild_7155826	1399936220	*Ca_kabuli_Chr04*	7155826	G/A	Gene_Ca_03519	UPSTREAM	Pectinesterase inhibitor
Kabuli_wild_7205223	1399936221	*Ca_kabuli_Chr04*	7205223	T/G	NA	INTERGENIC	NA
Kabuli_wild_7268040	1399936222	*Ca_kabuli_Chr04*	7268040	T/A	Gene_Ca_03508	INTRON	Zinc finger,RING-CH-type
Kabuli_wild_7301617	1399936223	*Ca_kabuli_Chr04*	7301617	C/T	NA	INTERGENIC	NA
Kabuli_wild_7335587	1399936224	*Ca_kabuli_Chr04*	7335587	C/T	Gene_Ca_03503	NON_SYNONYMOUS_CODING	BRCT
Kabuli_wild_7335668	1399936225	*Ca_kabuli_Chr04*	7335668	T/C	Gene_Ca_03503	NON_SYNONYMOUS_CODING	BRCT
Kabuli_wild_7349948	1399936226	*Ca_kabuli_Chr04*	7349948	T/C	Gene_Ca_03501	SYNONYMOUS_CODING	Lipase,class3
Kabuli_wild_7349958	1399936227	*Ca_kabuli_Chr04*	7349958	T/C	Gene_Ca_03501	NON_SYNONYMOUS_CODING	Lipase,class3
Kabuli_wild_7350192	1399936228	*Ca_kabuli_Chr04*	7350192	G/T	Gene_Ca_03501	NON_SYNONYMOUS_CODING	Lipase,class3
Kabuli_wild_7371708	1399936229	*Ca_kabuli_Chr04*	7371708	T/C	Gene_Ca_03500	DOWNSTREAM	Transcription initiation factor IID,18 kDa subunit
Kabuli_wild_7746286	1399936230	*Ca_kabuli_Chr04*	7746286	C/T	NA	INTERGENIC	NA
Kabuli_wild_7819865	1399936231	*Ca_kabuli_Chr04*	7819865	T/C	Gene_Ca_03457	INTRON	Glycosidehydrolase,family1
Kabuli_wild_7868480	1399936232	*Ca_kabuli_Chr04*	7868480	A/T	NA	INTERGENIC	NA
Kabuli_wild_7892628	1399936233	*Ca_kabuli_Chr04*	7892628	T/C	Gene_Ca_03447	INTRON	ATPase,AAA+type,core
Kabuli_wild_8050770	1399936234	*Ca_kabuli_Chr04*	8050770	T/C	Gene_Ca_03429	NON_SYNONYMOUS_CODING	Tubby,C-terminal
Kabuli_wild_8051306	1399936235	*Ca_kabuli_Chr04*	8051306	T/C	Gene_Ca_03429	INTRON	Tubby,C-terminal
Kabuli_wild_8067599	1399936236	*Ca_kabuli_Chr04*	8067599	C/G	Gene_Ca_03426	DOWNSTREAM	PeptidaseS1/S6,chymotrypsin/Hap
Kabuli_wild_8131433	1399936237	*Ca_kabuli_Chr04*	8131433	T/G	Gene_Ca_03419	INTRON	Metallo-dependentphosphatase
Kabuli_wild_8186660	1399936238	*Ca_kabuli_Chr04*	8186660	A/C	Gene_Ca_08308	NON_SYNONYMOUS_CODING	Signal transduction histidine kinase,phosphotransfer (Hpt) domain
Kabuli_wild_8198702	1399936239	*Ca_kabuli_Chr04*	8198702	T/G	Gene_Ca_08309	NON_SYNONYMOUS_CODING	WD40 repeat
Kabuli_wild_8269099	1399936240	*Ca_kabuli_Chr04*	8269099	T/G	Gene_Ca_08315	INTRON	Protein of unknown function DUF3595
Kabuli_wild_8269347	1399936241	*Ca_kabuli_Chr04*	8269347	C/G	Gene_Ca_08315	INTRON	Protein of unknown function DUF3595
Kabuli_wild_8369437	1399936242	*Ca_kabuli_Chr04*	8369437	C/A	Gene_Ca_08325	STOP_GAINED	NA
Kabuli_wild_8383890	1399936243	*Ca_kabuli_Chr04*	8383890	G/A	Gene_Ca_08327	INTRON	BSD
Kabuli_wild_8396837	1399936244	*Ca_kabuli_Chr04*	8396837	T/G	Gene_Ca_08328	DOWNSTREAM	Pentatrico peptide repeat
Kabuli_wild_8396861	1399936245	*Ca_kabuli_Chr04*	8396861	T/A	Gene_Ca_08328	DOWNSTREAM	Pentatrico peptide repeat
Kabuli_wild_8401109	1399936246	*Ca_kabuli_Chr04*	8401109	A/G	Gene_Ca_08329	INTRON	Chaperonin Cpn60
Kabuli_wild_8401126	1399936247	*Ca_kabuli_Chr04*	8401126	G/T	Gene_Ca_08329	INTRON	Chaperonin Cpn60

SNP IDs	NCBI dbSNP accession IDs (NCBI_SS#)	Chromosomes	Physical positions (bp)	SNPs	Gene accession IDs	Sequence components of genome	Putative functions
Kabuli_wild_8401143	1399936248	*Ca_kabuli_Chr04*	8401143	A/T	Gene_Ca_08329	SYNONYMOUS_CODING	Chaperonin Cpn60
Kabuli_wild_8401230	1399936249	*Ca_kabuli_Chr04*	8401230	A/G	Gene_Ca_08329	UPSTREAM	Chaperonin Cpn60
Kabuli_wild_8401248	1399936250	*Ca_kabuli_Chr04*	8401248	T/A	Gene_Ca_08329	UPSTREAM	Chaperonin Cpn60
Kabuli_wild_8404165	1399936251	*Ca_kabuli_Chr04*	8404165	C/A	NA	INTERGENIC	NA
Kabuli_wild_8471029	1399936252	*Ca_kabuli_Chr04*	8471029	G/A	NA	INTERGENIC	NA
Kabuli_wild_8473120	1399936253	*Ca_kabuli_Chr04*	8473120	G/A	Gene_Ca_08339	INTRON	Phospho fructokinase domain
Kabuli_wild_8473127	1399936254	*Ca_kabuli_Chr04*	8473127	G/A	Gene_Ca_08339	INTRON	Phospho fructokinase domain
Kabuli_wild_8473246	1399936255	*Ca_kabuli_Chr04*	8473246	T/C	Gene_Ca_08339	INTRON	Phospho fructokinase domain
Kabuli_wild_8473288	1399936256	*Ca_kabuli_Chr04*	8473288	A/G	Gene_Ca_08339	INTRON	Phospho fructokinase domain
Kabuli_wild_8473294	1399936257	*Ca_kabuli_Chr04*	8473294	G/A	Gene_Ca_08339	INTRON	Phospho fructokinase domain
Kabuli_wild_8474316	1399936258	*Ca_kabuli_Chr04*	8474316	T/C	Gene_Ca_08339	INTRON	Phospho fructokinase domain
Kabuli_wild_8475634	1399936259	*Ca_kabuli_Chr04*	8475634	A/C	Gene_Ca_08339	SYNONYMOUS_CODING	Phospho fructokinase domain
Kabuli_wild_8483482	1399936260	*Ca_kabuli_Chr04*	8483482	T/C	Gene_Ca_08340	SYNONYMOUS_CODING	WD40 repeat
Kabuli_wild_8483494	1399936261	*Ca_kabuli_Chr04*	8483494	T/C	Gene_Ca_08340	SYNONYMOUS_CODING	WD40 repeat
Kabuli_wild_8485918	1399936262	*Ca_kabuli_Chr04*	8485918	A/G	Gene_Ca_08340	NON_SYNONYMOUS_CODING	WD40 repeat
Kabuli_wild_8496641	1399936263	*Ca_kabuli_Chr04*	8496641	C/A	Gene_Ca_08341	SYNONYMOUS_CODING	WD40 repeat
Kabuli_wild_8496853	1399936264	*Ca_kabuli_Chr04*	8496853	A/G	Gene_Ca_08341	SYNONYMOUS_CODING	WD40 repeat
Kabuli_wild_8496910	1399936265	*Ca_kabuli_Chr04*	8496910	A/G	Gene_Ca_08341	SYNONYMOUS_CODING	WD40 repeat
Kabuli_wild_8510011	1399936266	*Ca_kabuli_Chr04*	8510011	A/C	Gene_Ca_08343	SYNONYMOUS_CODING	NA
Kabuli_wild_8512095	1399936267	*Ca_kabuli_Chr04*	8512095	G/A	Gene_Ca_08343	SYNONYMOUS_CODING	NA
Kabuli_wild_8512441	1399936268	*Ca_kabuli_Chr04*	8512441	T/C	Gene_Ca_08343	SYNONYMOUS_CODING	NA
Kabuli_wild_8518979	1399936269	*Ca_kabuli_Chr04*	8518979	G/A	NA	INTERGENIC	NA
Kabuli_wild_8570712	1399936270	*Ca_kabuli_Chr04*	8570712	T/G	NA	INTERGENIC	NA
Kabuli_wild_8570714	1399936271	*Ca_kabuli_Chr04*	8570714	T/G	NA	INTERGENIC	NA
Kabuli_wild_8570718	1399936272	*Ca_kabuli_Chr04*	8570718	G/T	NA	INTERGENIC	NA
Kabuli_wild_8601364	1399936273	*Ca_kabuli_Chr04*	8601364	T/G	Gene_Ca_08354	INTRON	Exo70 exocystcomplex subunit
Kabuli_wild_8627547	1399936274	*Ca_kabuli_Chr04*	8627547	G/A	Gene_Ca_08356	SYNONYMOUS_CODING	Shugoshin,C-terminal
Kabuli_wild_8656398	1399936275	*Ca_kabuli_Chr04*	8656398	G/A	Gene_Ca_08360	UPSTREAM	Disease resistance protein
Kabuli_wild_8656635	1399936276	*Ca_kabuli_Chr04*	8656635	C/T	Gene_Ca_08360	UPSTREAM	Disease resistance protein
Kabuli_wild_8659774	1399936277	*Ca_kabuli_Chr04*	8659774	A/G	NA	INTERGENIC	NA
Kabuli_wild_8660115	1399936278	*Ca_kabuli_Chr04*	8660115	A/T	NA	INTERGENIC	NA
Kabuli_wild_8668648	1399936279	*Ca_kabuli_Chr04*	8668648	G/A	NA	INTERGENIC	NA
Kabuli_wild_8668753	1399936280	*Ca_kabuli_Chr04*	8668753	A/G	NA	INTERGENIC	NA
Kabuli_wild_8668945	1399936281	*Ca_kabuli_Chr04*	8668945	T/C	NA	INTERGENIC	NA
Kabuli_wild_8669505	1399936282	*Ca_kabuli_Chr04*	8669505	G/T	NA	INTERGENIC	NA
Kabuli_wild_8669513	1399936283	*Ca_kabuli_Chr04*	8669513	G/A	NA	INTERGENIC	NA
Kabuli_wild_8669515	1399936284	*Ca_kabuli_Chr04*	8669515	C/T	NA	INTERGENIC	NA
Kabuli_wild_8681949	1399936285	*Ca_kabuli_Chr04*	8681949	G/A	NA	INTERGENIC	NA
Kabuli_wild_8682003	1399936286	*Ca_kabuli_Chr04*	8682003	A/G	NA	INTERGENIC	NA
Kabuli_wild_8682176	1399936287	*Ca_kabuli_Chr04*	8682176	C/A	NA	INTERGENIC	NA
Kabuli_wild_8682191	1399936288	*Ca_kabuli_Chr04*	8682191	C/T	NA	INTERGENIC	NA
Kabuli_wild_8686156	1399936289	*Ca_kabuli_Chr04*	8686156	C/A	NA	INTERGENIC	NA
Kabuli_wild_8686216	1399936290	*Ca_kabuli_Chr04*	8686216	T/A	NA	INTERGENIC	NA
Kabuli_wild_8695562	1399936291	*Ca_kabuli_Chr04*	8695562	C/T	Gene_Ca_08365	INTRON	Glycosidehydrolase,family13,N-terminal

SNP IDs	NCBI dbSNP accession IDs (NCBI_SS#)	Chromosomes	Physical positions (bp)	SNPs	Gene accession IDs	Sequence components of genome	Putative functions
Kabuli_wild_8721421	1399936292	*Ca_kabuli_Chr04*	8721421	C/A	Gene_Ca_08368	UPSTREAM	Multicopperoxidase,type1
Kabuli_wild_8751588	1399936293	*Ca_kabuli_Chr04*	8751588	A/C	NA	INTERGENIC	NA
Kabuli_wild_8751591	1399936294	*Ca_kabuli_Chr04*	8751591	A/C	NA	INTERGENIC	NA
Kabuli_wild_8751618	1399936295	*Ca_kabuli_Chr04*	8751618	A/C	Gene_Ca_08371	NON_SYNONYMOUS_CODING	No apical meristem (NAM) protein
Kabuli_wild_8751629	1399936296	*Ca_kabuli_Chr04*	8751629	T/C	Gene_Ca_08371	SYNONYMOUS_CODING	No apical meristem (NAM) protein
Kabuli_wild_8751638	1399936297	*Ca_kabuli_Chr04*	8751638	A/C	Gene_Ca_08371	SYNONYMOUS_CODING	No apical meristem (NAM) protein
Kabuli_wild_8782879	1399936298	*Ca_kabuli_Chr04*	8782879	G/C	NA	INTERGENIC	NA
Kabuli_wild_8791654	1399936299	*Ca_kabuli_Chr04*	8791654	A/G	Gene_Ca_08375	SYNONYMOUS_CODING	Kelchrepeat type2
Kabuli_wild_8805504	1399936300	*Ca_kabuli_Chr04*	8805504	A/G	NA	INTERGENIC	NA
Kabuli_wild_8805578	1399936301	*Ca_kabuli_Chr04*	8805578	A/C	NA	INTERGENIC	NA
Kabuli_wild_8805667	1399936302	*Ca_kabuli_Chr04*	8805667	C/T	NA	INTERGENIC	NA
Kabuli_wild_8805803	1399936303	*Ca_kabuli_Chr04*	8805803	G/A	NA	INTERGENIC	NA
Kabuli_wild_8805839	1399936304	*Ca_kabuli_Chr04*	8805839	G/A	NA	INTERGENIC	NA
Kabuli_wild_8805867	1399936305	*Ca_kabuli_Chr04*	8805867	A/C	NA	INTERGENIC	NA
Kabuli_wild_8809032	1399936306	*Ca_kabuli_Chr04*	8809032	C/T	Gene_Ca_08378	INTRON	K Homology
Kabuli_wild_8809064	1399936307	*Ca_kabuli_Chr04*	8809064	T/C	Gene_Ca_08378	SYNONYMOUS_CODING	K Homology
Kabuli_wild_8809456	1399936308	*Ca_kabuli_Chr04*	8809456	A/C	Gene_Ca_08378	UPSTREAM	K Homology
Kabuli_wild_8844846	1399936309	*Ca_kabuli_Chr04*	8844846	A/G	Gene_Ca_08381	SYNONYMOUS_CODING	Zinc finger, RING-type
Kabuli_wild_8848648	1399936310	*Ca_kabuli_Chr04*	8848648	C/A	Gene_Ca_08381	SYNONYMOUS_CODING	Zinc finger, RING-type
Kabuli_wild_8848652	1399936311	*Ca_kabuli_Chr04*	8848652	G/C	Gene_Ca_08381	NON_SYNONYMOUS_CODING	Zinc finger, RING-type
Kabuli_wild_8848653	1399936312	*Ca_kabuli_Chr04*	8848653	T/C	Gene_Ca_08381	NON_SYNONYMOUS_CODING	Zinc finger, RING-type
Kabuli_wild_8848654	1399936313	*Ca_kabuli_Chr04*	8848654	C/T	Gene_Ca_08381	SYNONYMOUS_CODING	Zinc finger, RING-type
Kabuli_wild_8852420	1399936314	*Ca_kabuli_Chr04*	8852420	C/T	Gene_Ca_08382	NON_SYNONYMOUS_CODING	NA
Kabuli_wild_8865427	1399936315	*Ca_kabuli_Chr04*	8865427	A/G	Gene_Ca_08384	DOWNSTREAM	PeptidaseM22,glycoprotease
Kabuli_wild_8919395	1399936316	*Ca_kabuli_Chr04*	8919395	A/G	NA	INTERGENIC	NA
Kabuli_wild_8926380	1399936317	*Ca_kabuli_Chr04*	8926380	A/G	Gene_Ca_08389	NON_SYNONYMOUS_CODING	Glycosyltransferase,group1
Kabuli_wild_8985998	1399936318	*Ca_kabuli_Chr04*	8985998	T/A	NA	INTERGENIC	NA
Kabuli_wild_8986002	1399936319	*Ca_kabuli_Chr04*	8986002	T/A	NA	INTERGENIC	NA
Kabuli_wild_8986008	1399936320	*Ca_kabuli_Chr04*	8986008	T/A	NA	INTERGENIC	NA
Kabuli_wild_8986018	1399936321	*Ca_kabuli_Chr04*	8986018	G/A	NA	INTERGENIC	NA
Kabuli_wild_8986024	1399936322	*Ca_kabuli_Chr04*	8986024	T/A	NA	INTERGENIC	NA
Kabuli_wild_8986026	1399936323	*Ca_kabuli_Chr04*	8986026	T/A	NA	INTERGENIC	NA
Kabuli_wild_8986031	1399936324	*Ca_kabuli_Chr04*	8986031	T/A	NA	INTERGENIC	NA
Kabuli_wild_9075395	1399936325	*Ca_kabuli_Chr04*	9075395	A/G	NA	INTERGENIC	NA
Kabuli_wild_9075398	1399936326	*Ca_kabuli_Chr04*	9075398	G/A	NA	INTERGENIC	NA
Kabuli_wild_9075459	1399936327	*Ca_kabuli_Chr04*	9075459	C/T	NA	INTERGENIC	NA
Kabuli_wild_9075520	1399936328	*Ca_kabuli_Chr04*	9075520	C/T	NA	INTERGENIC	NA
Kabuli_wild_9075543	1399936329	*Ca_kabuli_Chr04*	9075543	T/C	NA	INTERGENIC	NA
Kabuli_wild_9075565	1399936330	*Ca_kabuli_Chr04*	9075565	A/G	NA	INTERGENIC	NA
Kabuli_wild_9075671	1399936331	*Ca_kabuli_Chr04*	9075671	A/G	NA	INTERGENIC	NA
Kabuli_wild_9075677	1399936332	*Ca_kabuli_Chr04*	9075677	T/C	NA	INTERGENIC	NA
Kabuli_wild_9075682	1399936333	*Ca_kabuli_Chr04*	9075682	G/A	NA	INTERGENIC	NA
Kabuli_wild_9075737	1399936334	*Ca_kabuli_Chr04*	9075737	G/A	NA	INTERGENIC	NA
Kabuli_wild_9075744	1399936335	*Ca_kabuli_Chr04*	9075744	T/G	NA	INTERGENIC	NA

SNP IDs	NCBI dbSNP accession IDs (NCBI_SS#)	Chromosomes	Physical positions (bp)	SNPs	Gene accession IDs	Sequence components of genome	Putative functions
Kabuli_wild_9075746	1399936336	*Ca_kabuli_Chr04*	9075746	T/G	NA	INTERGENIC	NA
Kabuli_wild_9187887	1399936337	*Ca_kabuli_Chr04*	9187887	G/C	Gene_Ca_08410	NON_SYNONYMOUS_CODING	Vps51/Vps67
Kabuli_wild_9187973	1399936338	*Ca_kabuli_Chr04*	9187973	T/G	Gene_Ca_08410	SYNONYMOUS_CODING	Vps51/Vps68
Kabuli_wild_9189976	1399936339	*Ca_kabuli_Chr04*	9189976	T/A	Gene_Ca_08410	NON_SYNONYMOUS_CODING	Vps51/Vps69
Kabuli_wild_9257171	1399936340	*Ca_kabuli_Chr04*	9257171	G/A	NA	INTERGENIC	NA
Kabuli_wild_9331949	1399936341	*Ca_kabuli_Chr04*	9331949	C/T	NA	INTERGENIC	NA
Kabuli_wild_9421565	1399936342	*Ca_kabuli_Chr04*	9421565	T/C	Gene_Ca_08432	NON_SYNONYMOUS_CODING	PeptidaseM18
Kabuli_wild_9450986	1399936343	*Ca_kabuli_Chr04*	9450986	T/C	Gene_Ca_08434	NON_SYNONYMOUS_CODING	Myb,DNA-binding
Kabuli_wild_9451850	1399936344	*Ca_kabuli_Chr04*	9451850	T/G	NA	INTERGENIC	NA
Kabuli_wild_9451895	1399936345	*Ca_kabuli_Chr04*	9451895	T/A	NA	INTERGENIC	NA
Kabuli_wild_9470957	1399936346	*Ca_kabuli_Chr04*	9470957	C/G	Gene_Ca_08436	NON_SYNONYMOUS_CODING	Pathogenesis-related transcriptional factor/ERF, DNA-binding
Kabuli_wild_9491723	1399936347	*Ca_kabuli_Chr04*	9491723	A/T	Gene_Ca_08437	INTRON	Malectin
Kabuli_wild_9571887	1399936348	*Ca_kabuli_Chr04*	9571887	T/C	NA	INTERGENIC	NA
Kabuli_wild_9579517	1399936349	*Ca_kabuli_Chr04*	9579517	T/C	NA	INTERGENIC	NA
Kabuli_wild_9579519	1399936350	*Ca_kabuli_Chr04*	9579519	A/T	NA	INTERGENIC	NA
Kabuli_wild_9614905	1399936351	*Ca_kabuli_Chr04*	9614905	C/A	Gene_Ca_08443	NON_SYNONYMOUS_CODING	NA
Kabuli_wild_9706453	1399936352	*Ca_kabuli_Chr04*	9706453	T/G	NA	INTERGENIC	NA
Kabuli_wild_9803227	1399936353	*Ca_kabuli_Chr04*	9803227	C/T	Gene_Ca_08457	DOWNSTREAM	Peptidase,cysteine peptidase active site
Kabuli_wild_9803230	1399936354	*Ca_kabuli_Chr04*	9803230	C/T	Gene_Ca_08457	DOWNSTREAM	Peptidase,cysteine peptidase active site
Kabuli_wild_9803233	1399936355	*Ca_kabuli_Chr04*	9803233	C/T	Gene_Ca_08457	DOWNSTREAM	Peptidase,cysteine peptidase active site
Kabuli_wild_9803236	1399936356	*Ca_kabuli_Chr04*	9803236	C/T	Gene_Ca_08457	DOWNSTREAM	Peptidase,cysteine peptidase active site
Kabuli_wild_9803244	1399936357	*Ca_kabuli_Chr04*	9803244	C/T	Gene_Ca_08457	DOWNSTREAM	Peptidase,cysteine peptidase active site
Kabuli_wild_9824234	1399936358	*Ca_kabuli_Chr04*	9824234	C/T	NA	INTERGENIC	NA
Kabuli_wild_9824281	1399936359	*Ca_kabuli_Chr04*	9824281	A/G	NA	INTERGENIC	NA
Kabuli_wild_9836559	1399936360	*Ca_kabuli_Chr04*	9836559	G/T	Gene_Ca_08462	INTRON	VHS
Kabuli_wild_10007069	1399936361	*Ca_kabuli_Chr04*	10007069	A/G	NA	INTERGENIC	NA
Kabuli_wild_10116516	1399936362	*Ca_kabuli_Chr04*	10116516	T/G	Gene_Ca_08486	SYNONYMOUS_CODING	Pleckstrinhomologydomain
Kabuli_wild_10151943	1399936363	*Ca_kabuli_Chr04*	10151943	C/T	Gene_Ca_08487	NON_SYNONYMOUS_CODING	Zinc finger,GATA-type
Kabuli_wild_10184427	1399936364	*Ca_kabuli_Chr04*	10184427	C/A	Gene_Ca_08489	SYNONYMOUS_CODING	GCN5-related N-acetyltransferase (GNAT) domain
Kabuli_wild_10184428	1399936365	*Ca_kabuli_Chr04*	10184428	G/T	Gene_Ca_08489	NON_SYNONYMOUS_CODING	GCN5-related N-acetyltransferase (GNAT) domain
Kabuli_wild_10187627	1399936366	*Ca_kabuli_Chr04*	10187627	A/C	Gene_Ca_08489	INTRON	GCN5-related N-acetyltransferase (GNAT) domain
Kabuli_wild_10294380	1399936367	*Ca_kabuli_Chr04*	10294380	A/G	Gene_Ca_08496	INTRON	Carbamoyl-phosphatesynthase,GATase domain
Kabuli_wild_10305285	1399936368	*Ca_kabuli_Chr04*	10305285	C/A	Gene_Ca_08496	INTRON	Carbamoyl-phosphatesynthase,GATase domain
Kabuli_wild_10305286	1399936369	*Ca_kabuli_Chr04*	10305286	G/A	Gene_Ca_08496	INTRON	Carbamoyl-phosphatesynthase,GATase domain
Kabuli_wild_10411809	1399936370	*Ca_kabuli_Chr04*	10411809	G/A	NA	INTERGENIC	NA
Kabuli_wild_10418170	1399936371	*Ca_kabuli_Chr04*	10418170	T/G	Gene_Ca_04277	SYNONYMOUS_CODING	SANT domain,DNA binding
Kabuli_wild_10422142	1399936372	*Ca_kabuli_Chr04*	10422142	A/T	Gene_Ca_04277	DOWNSTREAM	NA
Kabuli_wild_10422469	1399936373	*Ca_kabuli_Chr04*	10422469	C/A	NA	INTERGENIC	NA
Kabuli_wild_10430574	1399936374	*Ca_kabuli_Chr04*	10430574	A/T	NA	INTERGENIC	NA
Kabuli_wild_10430588	1399936375	*Ca_kabuli_Chr04*	10430588	T/C	NA	INTERGENIC	NA
Kabuli_wild_10430595	1399936376	*Ca_kabuli_Chr04*	10430595	A/C	NA	INTERGENIC	NA
Kabuli_wild_10430603	1399936377	*Ca_kabuli_Chr04*	10430603	G/A	NA	INTERGENIC	NA
Kabuli_wild_10430613	1399936378	*Ca_kabuli_Chr04*	10430613	C/A	NA	INTERGENIC	NA
Kabuli_wild_10430617	1399936379	*Ca_kabuli_Chr04*	10430617	A/C	NA	INTERGENIC	NA

SNP IDs	NCBI dbSNP accession IDs (NCBI_SS#)	Chromosomes	Physical positions (bp)	SNPs	Gene accession IDs	Sequence components of genome	Putative functions
Kabuli_wild_10430622	1399936380	*Ca_kabuli_Chr04*	10430622	A/C	NA	INTERGENIC	NA
Kabuli_wild_10464927	1399936381	*Ca_kabuli_Chr04*	10464927	T/G	NA	INTERGENIC	NA
Kabuli_wild_10465328	1399936382	*Ca_kabuli_Chr04*	10465328	T/C	NA	INTERGENIC	NA
Kabuli_wild_10465981	1399936383	*Ca_kabuli_Chr04*	10465981	A/C	NA	INTERGENIC	NA
Kabuli_wild_10465982	1399936384	*Ca_kabuli_Chr04*	10465982	G/T	NA	INTERGENIC	NA
Kabuli_wild_10465985	1399936385	*Ca_kabuli_Chr04*	10465985	C/G	NA	INTERGENIC	NA
Kabuli_wild_10665122	1399936386	*Ca_kabuli_Chr04*	10665122	G/A	NA	INTERGENIC	NA
Kabuli_wild_10771276	1399936387	*Ca_kabuli_Chr04*	10771276	A/T	Gene_Ca_04314	INTRON	Vps51/Vps67
Kabuli_wild_10901282	1399936388	*Ca_kabuli_Chr04*	10901282	T/C	NA	INTERGENIC	NA
Kabuli_wild_10911500	1399936389	*Ca_kabuli_Chr04*	10911500	T/C	NA	INTERGENIC	NA
Kabuli_wild_10917717	1399936390	*Ca_kabuli_Chr04*	10917717	C/G	NA	INTERGENIC	NA
Kabuli_wild_10925490	1399936391	*Ca_kabuli_Chr04*	10925490	G/A	Gene_Ca_04326	SYNONYMOUS_CODING	Filamin/ABP280repeat-like
Kabuli_wild_10928963	1399936392	*Ca_kabuli_Chr04*	10928963	T/C	Gene_Ca_04326	INTRON	Filamin/ABP280repeat-like
Kabuli_wild_10931884	1399936393	*Ca_kabuli_Chr04*	10931884	A/G	Gene_Ca_04326	DOWNSTREAM	Filamin/ABP280repeat-like
Kabuli_wild_10931908	1399936394	*Ca_kabuli_Chr04*	10931908	C/T	Gene_Ca_04326	DOWNSTREAM	Filamin/ABP280repeat-like
Kabuli_wild_10932217	1399936395	*Ca_kabuli_Chr04*	10932217	A/G	Gene_Ca_04326	DOWNSTREAM	Filamin/ABP280repeat-like
Kabuli_wild_10971039	1399936396	*Ca_kabuli_Chr04*	10971039	C/A	Gene_Ca_04330	INTRON	WD40 repeat
Kabuli_wild_10989311	1399936397	*Ca_kabuli_Chr04*	10989311	C/T	Gene_Ca_04331	SYNONYMOUS_CODING	Protein kinase,catalytic domain
Kabuli_wild_10990000	1399936398	*Ca_kabuli_Chr04*	10990000	T/C	Gene_Ca_04331	INTRON	Protein kinase,catalytic domain
Kabuli_wild_10990049	1399936399	*Ca_kabuli_Chr04*	10990049	G/T	Gene_Ca_04331	INTRON	Protein kinase,catalytic domain
Kabuli_wild_10990058	1399936400	*Ca_kabuli_Chr04*	10990058	T/C	Gene_Ca_04331	INTRON	Protein kinase,catalytic domain
Kabuli_wild_10990265	1399936401	*Ca_kabuli_Chr04*	10990265	T/A	Gene_Ca_04331	INTRON	Protein kinase,catalytic domain
Kabuli_wild_10991842	1399936402	*Ca_kabuli_Chr04*	10991842	G/A	Gene_Ca_04331	INTRON	Protein kinase,catalytic domain
Kabuli_wild_10991910	1399936403	*Ca_kabuli_Chr04*	10991910	C/T	Gene_Ca_04331	INTRON	Protein kinase,catalytic domain
Kabuli_wild_11040108	1399936404	*Ca_kabuli_Chr04*	11040108	C/T	Gene_Ca_04335	DOWNSTREAM	Uncharacterised protein family Ycf19
Kabuli_wild_11051335	1399936405	*Ca_kabuli_Chr04*	11051335	C/T	Gene_Ca_04337	NON_SYNONYMOUS_CODING	No apical meristem (NAM) protein
Kabuli_wild_11059403	1399936406	*Ca_kabuli_Chr04*	11059403	T/G	NA	INTERGENIC	NA
Kabuli_wild_11059404	1399936407	*Ca_kabuli_Chr04*	11059404	T/G	NA	INTERGENIC	NA
Kabuli_wild_11059414	1399936408	*Ca_kabuli_Chr04*	11059414	T/G	NA	INTERGENIC	NA
Kabuli_wild_11091683	1399936409	*Ca_kabuli_Chr04*	11091683	T/A	NA	INTERGENIC	NA
Kabuli_wild_11096225	1399936410	*Ca_kabuli_Chr04*	11096225	A/G	Gene_Ca_04343	DOWNSTREAM	Protein of unknown function DUF1475
Kabuli_wild_11096290	1399936411	*Ca_kabuli_Chr04*	11096290	G/A	Gene_Ca_04343	DOWNSTREAM	Protein of unknown function DUF1475
Kabuli_wild_11113277	1399936412	*Ca_kabuli_Chr04*	11113277	A/C	Gene_Ca_04344	SYNONYMOUS_CODING	RNA recognition motif domain
Kabuli_wild_11142202	1399936413	*Ca_kabuli_Chr04*	11142202	A/G	NA	INTERGENIC	NA
Kabuli_wild_11142227	1399936414	*Ca_kabuli_Chr04*	11142227	C/T	NA	INTERGENIC	NA
Kabuli_wild_11230397	1399936415	*Ca_kabuli_Chr04*	11230397	G/C	Gene_Ca_04356	DOWNSTREAM	Choline/ethanolaminekinase
Kabuli_wild_11230400	1399936416	*Ca_kabuli_Chr04*	11230400	T/A	Gene_Ca_04356	DOWNSTREAM	Choline/ethanolaminekinase
Kabuli_wild_11230403	1399936417	*Ca_kabuli_Chr04*	11230403	G/T	Gene_Ca_04356	DOWNSTREAM	Choline/ethanolaminekinase
Kabuli_wild_11231127	1399936418	*Ca_kabuli_Chr04*	11231127	C/A	Gene_Ca_04356	DOWNSTREAM	Choline/ethanolaminekinase
Kabuli_wild_11231135	1399936419	*Ca_kabuli_Chr04*	11231135	C/T	Gene_Ca_04356	DOWNSTREAM	Choline/ethanolaminekinase
Kabuli_wild_11231137	1399936420	*Ca_kabuli_Chr04*	11231137	A/G	Gene_Ca_04356	DOWNSTREAM	Choline/ethanolaminekinase
Kabuli_wild_11231142	1399936421	*Ca_kabuli_Chr04*	11231142	A/G	Gene_Ca_04356	DOWNSTREAM	Choline/ethanolaminekinase
Kabuli_wild_11234130	1399936422	*Ca_kabuli_Chr04*	11234130	A/G	NA	INTERGENIC	NA
Kabuli_wild_11234397	1399936423	*Ca_kabuli_Chr04*	11234397	G/A	NA	INTERGENIC	NA

SNP IDs	NCBI dbSNP accession IDs (NCBI_SS#)	Chromosomes	Physical positions (bp)	SNPs	Gene accession IDs	Sequence components of genome	Putative functions
Kabuli_wild_11234418	1399936424	*Ca_kabuli_Chr04*	11234418	A/G	NA	INTERGENIC	NA
Kabuli_wild_11244334	1399936425	*Ca_kabuli_Chr04*	11244334	T/G	NA	INTERGENIC	NA
Kabuli_wild_11244395	1399936426	*Ca_kabuli_Chr04*	11244395	G/T	NA	INTERGENIC	NA
Kabuli_wild_11246093	1399936427	*Ca_kabuli_Chr04*	11246093	G/A	NA	INTERGENIC	NA
Kabuli_wild_11246164	1399936428	*Ca_kabuli_Chr04*	11246164	G/A	NA	INTERGENIC	NA
Kabuli_wild_11246173	1399936429	*Ca_kabuli_Chr04*	11246173	C/T	NA	INTERGENIC	NA
Kabuli_wild_11248605	1399936430	*Ca_kabuli_Chr04*	11248605	A/T	NA	INTERGENIC	NA
Kabuli_wild_11273328	1399936431	*Ca_kabuli_Chr04*	11273328	C/T	Gene_Ca_04359	INTRON	NA
Kabuli_wild_11273405	1399936432	*Ca_kabuli_Chr04*	11273405	A/T	Gene_Ca_04359	INTRON	NA
Kabuli_wild_11274281	1399936433	*Ca_kabuli_Chr04*	11274281	T/C	Gene_Ca_04359	SYNONYMOUS_CODING	NA
Kabuli_wild_11275171	1399936434	*Ca_kabuli_Chr04*	11275171	T/C	Gene_Ca_04359	SYNONYMOUS_CODING	NA
Kabuli_wild_11276225	1399936435	*Ca_kabuli_Chr04*	11276225	C/T	Gene_Ca_04359	SYNONYMOUS_CODING	NA
Kabuli_wild_11276413	1399936436	*Ca_kabuli_Chr04*	11276413	G/C	Gene_Ca_04359	INTRON	NA
Kabuli_wild_11276484	1399936437	*Ca_kabuli_Chr04*	11276484	T/C	Gene_Ca_04359	SYNONYMOUS_CODING	NA
Kabuli_wild_11277138	1399936438	*Ca_kabuli_Chr04*	11277138	A/G	Gene_Ca_04359	SYNONYMOUS_CODING	NA
Kabuli_wild_11277181	1399936439	*Ca_kabuli_Chr04*	11277181	G/T	Gene_Ca_04359	NON_SYNONYMOUS_CODING	NA
Kabuli_wild_11277297	1399936440	*Ca_kabuli_Chr04*	11277297	G/A	Gene_Ca_04359	SYNONYMOUS_CODING	NA
Kabuli_wild_11277574	1399936441	*Ca_kabuli_Chr04*	11277574	G/C	Gene_Ca_04359	NON_SYNONYMOUS_CODING	NA
Kabuli_wild_11305745	1399936442	*Ca_kabuli_Chr04*	11305745	G/T	Gene_Ca_04362	DOWNSTREAM	NA
Kabuli_wild_11328191	1399936443	*Ca_kabuli_Chr04*	11328191	T/C	Gene_Ca_04366	DOWNSTREAM	Protein kinase,catalytic domain
Kabuli_wild_11328194	1399936444	*Ca_kabuli_Chr04*	11328194	A/C	Gene_Ca_04366	DOWNSTREAM	Protein kinase,catalytic domain
Kabuli_wild_11328227	1399936445	*Ca_kabuli_Chr04*	11328227	T/G	Gene_Ca_04366	DOWNSTREAM	Protein kinase,catalytic domain
Kabuli_wild_11334343	1399936446	*Ca_kabuli_Chr04*	11334343	A/T	NA	INTERGENIC	NA
Kabuli_wild_11334350	1399936447	*Ca_kabuli_Chr04*	11334350	A/G	NA	INTERGENIC	NA
Kabuli_wild_11343230	1399936448	*Ca_kabuli_Chr04*	11343230	G/C	Gene_Ca_04368	INTRON	Zinc finger,DHHC-type,palmitoyltransferase
Kabuli_wild_11343257	1399936449	*Ca_kabuli_Chr04*	11343257	T/C	Gene_Ca_04368	INTRON	Zinc finger,DHHC-type,palmitoyltransferase
Kabuli_wild_11379077	1399936450	*Ca_kabuli_Chr04*	11379077	T/C	NA	INTERGENIC	NA
Kabuli_wild_11398682	1399936451	*Ca_kabuli_Chr04*	11398682	G/A	Gene_Ca_04373	UPSTREAM	RibosomalproteinL3
Kabuli_wild_11398699	1399936452	*Ca_kabuli_Chr04*	11398699	A/G	Gene_Ca_04373	UPSTREAM	RibosomalproteinL4
Kabuli_wild_11398889	1399936453	*Ca_kabuli_Chr04*	11398889	A/G	Gene_Ca_04373	UPSTREAM	RibosomalproteinL5
Kabuli_wild_11435380	1399936454	*Ca_kabuli_Chr04*	11435380	C/T	Gene_Ca_04379	UPSTREAM	Thioredoxin
Kabuli_wild_11435651	1399936455	*Ca_kabuli_Chr04*	11435651	A/G	Gene_Ca_04379	UPSTREAM	Thioredoxin
Kabuli_wild_11441604	1399936456	*Ca_kabuli_Chr04*	11441604	T/A	NA	INTERGENIC	NA
Kabuli_wild_11441673	1399936457	*Ca_kabuli_Chr04*	11441673	G/A	NA	INTERGENIC	NA
Kabuli_wild_11441735	1399936458	*Ca_kabuli_Chr04*	11441735	G/T	NA	INTERGENIC	NA
Kabuli_wild_11442061	1399936459	*Ca_kabuli_Chr04*	11442061	C/T	NA	INTERGENIC	NA
Kabuli_wild_11465057	1399936460	*Ca_kabuli_Chr04*	11465057	C/G	NA	INTERGENIC	NA
Kabuli_wild_11465075	1399936461	*Ca_kabuli_Chr04*	11465075	T/C	NA	INTERGENIC	NA
Kabuli_wild_11465113	1399936462	*Ca_kabuli_Chr04*	11465113	C/T	NA	INTERGENIC	NA
Kabuli_wild_11490100	1399936463	*Ca_kabuli_Chr04*	11490100	C/T	Gene_Ca_04384	SYNONYMOUS_CODING	Tubby,C-terminal
Kabuli_wild_11490125	1399936464	*Ca_kabuli_Chr04*	11490125	G/T	Gene_Ca_04384	SYNONYMOUS_CODING	Tubby,C-terminal
Kabuli_wild_11490154	1399936465	*Ca_kabuli_Chr04*	11490154	A/T	Gene_Ca_04384	SYNONYMOUS_CODING	Tubby,C-terminal
Kabuli_wild_11490496	1399936466	*Ca_kabuli_Chr04*	11490496	G/A	Gene_Ca_04384	SYNONYMOUS_CODING	Tubby,C-terminal
Kabuli_wild_11491864	1399936467	*Ca_kabuli_Chr04*	11491864	A/C	Gene_Ca_04384	UPSTREAM	Tubby,C-terminal

SNP IDs	NCBI dbSNP accession IDs (NCBI_SS#)	Chromosomes	Physical positions (bp)	SNPs	Gene accession IDs	Sequence components of genome	Putative functions
Kabuli_wild_11517517	1399936468	*Ca_kabuli_Chr04*	11517517	C/A	NA	INTERGENIC	NA
Kabuli_wild_11517582	1399936469	*Ca_kabuli_Chr04*	11517582	C/T	NA	INTERGENIC	NA
Kabuli_wild_11572051	1399936470	*Ca_kabuli_Chr04*	11572051	C/T	NA	INTERGENIC	NA
Kabuli_wild_11620964	1399936471	*Ca_kabuli_Chr04*	11620964	C/T	Gene_Ca_04388	SYNONYMOUS_CODING	Ubiquitininteractingmotif
Kabuli_wild_11646453	1399936472	*Ca_kabuli_Chr04*	11646453	G/A	Gene_Ca_04392	NON_SYNONYMOUS_CODING	Protein synthesis factor, GTP-binding
Kabuli_wild_11658314	1399936473	*Ca_kabuli_Chr04*	11658314	A/G	NA	INTERGENIC	NA
Kabuli_wild_11689611	1399936474	*Ca_kabuli_Chr04*	11689611	C/T	Gene_Ca_04395	NON_SYNONYMOUS_CODING	Basic-leucine zipper (bZIP) Transcription factor
Kabuli_wild_11752855	1399936475	*Ca_kabuli_Chr04*	11752855	A/G	Gene_Ca_04400	NON_SYNONYMOUS_CODING	NA
Kabuli_wild_11752862	1399936476	*Ca_kabuli_Chr04*	11752862	A/G	Gene_Ca_04400	SYNONYMOUS_CODING	NA
Kabuli_wild_11772023	1399936477	*Ca_kabuli_Chr04*	11772023	G/T	Gene_Ca_04401	NON_SYNONYMOUS_CODING	Zinc finger,FYVE-type
Kabuli_wild_11774322	1399936478	*Ca_kabuli_Chr04*	11774322	A/G	Gene_Ca_04401	SYNONYMOUS_CODING	Zinc finger,FYVE-type
Kabuli_wild_11774328	1399936479	*Ca_kabuli_Chr04*	11774328	A/G	Gene_Ca_04401	SYNONYMOUS_CODING	Zinc finger,FYVE-type
Kabuli_wild_11847494	1399936480	*Ca_kabuli_Chr04*	11847494	C/T	NA	INTERGENIC	NA
Kabuli_wild_11852425	1399936481	*Ca_kabuli_Chr04*	11852425	C/G	NA	INTERGENIC	NA
Kabuli_wild_11926352	1399936482	*Ca_kabuli_Chr04*	11926352	G/C	Gene_Ca_04414	INTRON	ATPase,AAA+type,core
Kabuli_wild_11931643	1399936483	*Ca_kabuli_Chr04*	11931643	T/G	Gene_Ca_04415	INTRON	ATPase,AAA+type,core
Kabuli_wild_12004082	1399936484	*Ca_kabuli_Chr04*	12004082	A/T	NA	INTERGENIC	NA
Kabuli_wild_12004205	1399936485	*Ca_kabuli_Chr04*	12004205	A/C	NA	INTERGENIC	NA
Kabuli_wild_12004209	1399936486	*Ca_kabuli_Chr04*	12004209	C/T	NA	INTERGENIC	NA
Kabuli_wild_12093051	1399936487	*Ca_kabuli_Chr04*	12093051	C/T	Gene_Ca_04433	SYNONYMOUS_CODING	Fattyacidhydroxylase
Kabuli_wild_12131914	1399936488	*Ca_kabuli_Chr04*	12131914	T/C	Gene_Ca_04435	SYNONYMOUS_CODING	Glycosidehydrolase,family28
Kabuli_wild_12165713	1399936489	*Ca_kabuli_Chr04*	12165713	A/G	Gene_Ca_04439	DOWNSTREAM	Protein of unknown function DUF819
Kabuli_wild_12167328	1399936490	*Ca_kabuli_Chr04*	12167328	A/G	NA	INTERGENIC	NA
Kabuli_wild_12167568	1399936491	*Ca_kabuli_Chr04*	12167568	G/A	NA	INTERGENIC	NA
Kabuli_wild_12234364	1399936492	*Ca_kabuli_Chr04*	12234364	A/C	Gene_Ca_04445	DOWNSTREAM	DNAmethylase,N-6adenine-specific,conservedsite
Kabuli_wild_12249494	1399936493	*Ca_kabuli_Chr04*	12249494	T/C	Gene_Ca_04448	INTRON	Protein kinase,catalytic domain
Kabuli_wild_12420390	1399936494	*Ca_kabuli_Chr04*	12420390	A/T	NA	INTERGENIC	NA
Kabuli_wild_12462644	1399936495	*Ca_kabuli_Chr04*	12462644	C/T	Gene_Ca_04469	INTRON	Zinc finger,CCHC-type
Kabuli_wild_12484911	1399936496	*Ca_kabuli_Chr04*	12484911	A/T	NA	INTERGENIC	NA
Kabuli_wild_12506440	1399936497	*Ca_kabuli_Chr04*	12506440	A/G	NA	INTERGENIC	NA
Kabuli_wild_12506450	1399936498	*Ca_kabuli_Chr04*	12506450	T/A	NA	INTERGENIC	NA
Kabuli_wild_12514706	1399936499	*Ca_kabuli_Chr04*	12514706	G/A	NA	INTERGENIC	NA
Kabuli_wild_12514724	1399936500	*Ca_kabuli_Chr04*	12514724	T/C	NA	INTERGENIC	NA
Kabuli_wild_12514726	1399936501	*Ca_kabuli_Chr04*	12514726	C/T	NA	INTERGENIC	NA
Kabuli_wild_12514728	1399936502	*Ca_kabuli_Chr04*	12514728	G/A	NA	INTERGENIC	NA
Kabuli_wild_12525313	1399936503	*Ca_kabuli_Chr04*	12525313	C/G	Gene_Ca_04475	SYNONYMOUS_CODING	Cytochromeb561,eukaryote
Kabuli_wild_12525328	1399936504	*Ca_kabuli_Chr04*	12525328	A/G	Gene_Ca_04475	SYNONYMOUS_CODING	Cytochromeb561,eukaryote
Kabuli_wild_12525563	1399936505	*Ca_kabuli_Chr04*	12525563	A/G	Gene_Ca_04475	INTRON	Cytochromeb561,eukaryote
Kabuli_wild_12525639	1399936506	*Ca_kabuli_Chr04*	12525639	T/A	Gene_Ca_04475	INTRON	Cytochromeb561,eukaryote
Kabuli_wild_12534847	1399936507	*Ca_kabuli_Chr04*	12534847	T/C	Gene_Ca_04476	DOWNSTREAM	Remorin,C-terminal
Kabuli_wild_12558541	1399936508	*Ca_kabuli_Chr04*	12558541	T/C	Gene_Ca_04478	INTRON	NUDIX hydrolase domain
Kabuli_wild_12558680	1399936509	*Ca_kabuli_Chr04*	12558680	C/T	Gene_Ca_04478	INTRON	NUDIX hydrolase domain
Kabuli_wild_12568599	1399936510	*Ca_kabuli_Chr04*	12568599	A/G	Gene_Ca_04479	SYNONYMOUS_CODING	Homoserine kinase
Kabuli_wild_12568602	1399936511	*Ca_kabuli_Chr04*	12568602	C/T	Gene_Ca_04479	SYNONYMOUS_CODING	Homoserine kinase

SNP IDs	NCBI dbSNP accession IDs (NCBI_SS#)	Chromosomes	Physical positions (bp)	SNPs	Gene accession IDs	Sequence components of genome	Putative functions
Kabuli_wild_12568707	1399936512	*Ca_kabuli_Chr04*	12568707	A/G	Gene_Ca_04479	SYNONYMOUS_CODING	Homoserine kinase
Kabuli_wild_12568845	1399936513	*Ca_kabuli_Chr04*	12568845	G/T	Gene_Ca_04479	SYNONYMOUS_CODING	Homoserine kinase
Kabuli_wild_12568905	1399936514	*Ca_kabuli_Chr04*	12568905	G/A	Gene_Ca_04479	SYNONYMOUS_CODING	Homoserine kinase
Kabuli_wild_12568944	1399936515	*Ca_kabuli_Chr04*	12568944	A/G	Gene_Ca_04479	SYNONYMOUS_CODING	Homoserine kinase
Kabuli_wild_12569022	1399936516	*Ca_kabuli_Chr04*	12569022	A/C	Gene_Ca_04479	SYNONYMOUS_CODING	Homoserine kinase
Kabuli_wild_12574650	1399936517	*Ca_kabuli_Chr04*	12574650	G/A	Gene_Ca_04480	NON_SYNONYMOUS_CODING	DNA mismatch repair protein MutS,C-terminal domain
Kabuli_wild_12578259	1399936518	*Ca_kabuli_Chr04*	12578259	A/G	Gene_Ca_04480	SYNONYMOUS_CODING	DNA mismatch repair protein MutS,C-terminal domain
Kabuli_wild_12578350	1399936519	*Ca_kabuli_Chr04*	12578350	A/G	Gene_Ca_04480	INTRON	DNA mismatch repair protein MutS,C-terminal domain
Kabuli_wild_12581371	1399936520	*Ca_kabuli_Chr04*	12581371	G/T	Gene_Ca_04480	SYNONYMOUS_CODING	DNA mismatch repair protein MutS,C-terminal domain
Kabuli_wild_12740280	1399936521	*Ca_kabuli_Chr04*	12740280	G/A	Gene_Ca_04493	NON_SYNONYMOUS_CODING	IQ motif,EF-handbinding site
Kabuli_wild_12740351	1399936522	*Ca_kabuli_Chr04*	12740351	T/G	Gene_Ca_04493	SYNONYMOUS_CODING	IQ motif,EF-handbinding site
Kabuli_wild_12811599	1399936523	*Ca_kabuli_Chr04*	12811599	C/A	NA	INTERGENIC	NA
Kabuli_wild_12869023	1399936524	*Ca_kabuli_Chr04*	12869023	C/T	NA	INTERGENIC	NA
Kabuli_wild_12973657	1399936525	*Ca_kabuli_Chr04*	12973657	C/A	Gene_Ca_04519	NON_SYNONYMOUS_CODING	GDP-fucoseproteinO-fucosyltransferase
Kabuli_wild_12982420	1399936526	*Ca_kabuli_Chr04*	12982420	G/A	Gene_Ca_04520	INTRON	Alpha-D-phosphohexomutase
Kabuli_wild_13179829	1399936527	*Ca_kabuli_Chr04*	13179829	T/C	NA	INTERGENIC	NA
Kabuli_wild_13203008	1399936528	*Ca_kabuli_Chr04*	13203008	A/T	Gene_Ca_04544	DOWNSTREAM	BromodomainTranscription factor
Kabuli_wild_13203164	1399936529	*Ca_kabuli_Chr04*	13203164	G/T	Gene_Ca_04544	DOWNSTREAM	BromodomainTranscription factor
Kabuli_wild_13229035	1399936530	*Ca_kabuli_Chr04*	13229035	C/T	Gene_Ca_04545	DOWNSTREAM	Argonaute/Dicerprotein,PAZ
Kabuli_wild_13252015	1399936531	*Ca_kabuli_Chr04*	13252015	A/T	Gene_Ca_04547	UPSTREAM	NA
Kabuli_wild_13252077	1399936532	*Ca_kabuli_Chr04*	13252077	C/G	Gene_Ca_04547	UPSTREAM	NA
Kabuli_wild_13252212	1399936533	*Ca_kabuli_Chr04*	13252212	G/A	Gene_Ca_04547	UPSTREAM	NA
Kabuli_wild_13282984	1399936534	*Ca_kabuli_Chr04*	13282984	A/C	NA	INTERGENIC	NA
Kabuli_wild_13310630	1399936535	*Ca_kabuli_Chr04*	13310630	G/A	NA	INTERGENIC	NA
Kabuli_wild_13376428	1399936536	*Ca_kabuli_Chr04*	13376428	A/C	Gene_Ca_04560	NON_SYNONYMOUS_CODING	Domain of unknown function DUF828
Kabuli_wild_13376464	1399936537	*Ca_kabuli_Chr04*	13376464	G/A	Gene_Ca_04560	NON_SYNONYMOUS_CODING	Domain of unknown function DUF828
Kabuli_wild_13575422	1399936538	*Ca_kabuli_Chr04*	13575422	T/G	Gene_Ca_04575	INTRON	Plantlipidtransferprotein/Parallergen
Kabuli_wild_13576867	1399936539	*Ca_kabuli_Chr04*	13576867	T/G	Gene_Ca_04575	DOWNSTREAM	Plantlipidtransferprotein/Parallergen
Kabuli_wild_13586365	1399936540	*Ca_kabuli_Chr04*	13586365	A/C	Gene_Ca_04577	UPSTREAM	Biopterin transport-related protein BT1
Kabuli_wild_13588928	1399936541	*Ca_kabuli_Chr04*	13588928	T/C	Gene_Ca_04577	INTRON	Biopterin transport-related protein BT1
Kabuli_wild_13588956	1399936542	*Ca_kabuli_Chr04*	13588956	C/T	Gene_Ca_04577	SYNONYMOUS_CODING	Biopterin transport-related protein BT1
Kabuli_wild_13646794	1399936543	*Ca_kabuli_Chr04*	13646794	T/A	NA	INTERGENIC	NA
Kabuli_wild_13660846	1399936544	*Ca_kabuli_Chr04*	13660846	G/A	NA	INTERGENIC	NA
Kabuli_wild_13687456	1399936545	*Ca_kabuli_Chr04*	13687456	G/A	Gene_Ca_04589	INTRON	Frigida-like
Kabuli_wild_13687570	1399936546	*Ca_kabuli_Chr04*	13687570	G/C	Gene_Ca_04589	INTRON	Frigida-like
Kabuli_wild_13687578	1399936547	*Ca_kabuli_Chr04*	13687578	G/A	Gene_Ca_04589	INTRON	Frigida-like
Kabuli_wild_13687728	1399936548	*Ca_kabuli_Chr04*	13687728	C/T	Gene_Ca_04589	INTRON	Frigida-like
Kabuli_wild_13698262	1399936549	*Ca_kabuli_Chr04*	13698262	T/C	NA	INTERGENIC	NA
Kabuli_wild_13698283	1399936550	*Ca_kabuli_Chr04*	13698283	T/A	NA	INTERGENIC	NA
Kabuli_wild_13699663	1399936551	*Ca_kabuli_Chr04*	13699663	G/A	NA	INTERGENIC	NA
Kabuli_wild_13702641	1399936552	*Ca_kabuli_Chr04*	13702641	G/A	Gene_Ca_04591	INTRON	XSdomain
Kabuli_wild_13713367	1399936553	*Ca_kabuli_Chr04*	13713367	G/C	Gene_Ca_04593	UPSTREAM	NA
Kabuli_wild_13713380	1399936554	*Ca_kabuli_Chr04*	13713380	G/T	Gene_Ca_04593	UPSTREAM	NA
Kabuli_wild_13724666	1399936555	*Ca_kabuli_Chr04*	13724666	G/C	Gene_Ca_04596	NON_SYNONYMOUS_CODING	Zinc finger,PHD-type

SNP IDs	NCBI dbSNP accession IDs (NCBI_SS#)	Chromosomes	Physical positions (bp)	SNPs	Gene accession IDs	Sequence components of genome	Putative functions
Kabuli_wild_13787448	1399936556	*Ca_kabuli_Chr04*	13787448	A/C	NA	INTERGENIC	NA
Kabuli_wild_13787649	1399936557	*Ca_kabuli_Chr04*	13787649	T/C	Gene_Ca_04604	DOWNSTREAM	Copperamineoxidase
Kabuli_wild_13787720	1399936558	*Ca_kabuli_Chr04*	13787720	G/C	Gene_Ca_04604	DOWNSTREAM	Copperamineoxidase
Kabuli_wild_13823643	1399936559	*Ca_kabuli_Chr04*	13823643	A/G	NA	INTERGENIC	NA
Kabuli_wild_13838706	1399936560	*Ca_kabuli_Chr04*	13838706	G/C	Gene_Ca_04608	SYNONYMOUS_CODING	NA
Kabuli_wild_13838796	1399936561	*Ca_kabuli_Chr04*	13838796	A/G	Gene_Ca_04608	SYNONYMOUS_CODING	NA
Kabuli_wild_13839288	1399936562	*Ca_kabuli_Chr04*	13839288	G/A	Gene_Ca_04608	SYNONYMOUS_CODING	NA
Kabuli_wild_13839294	1399936563	*Ca_kabuli_Chr04*	13839294	A/G	Gene_Ca_04608	SYNONYMOUS_CODING	NA
Kabuli_wild_13839357	1399936564	*Ca_kabuli_Chr04*	13839357	T/C	Gene_Ca_04608	SYNONYMOUS_CODING	NA
Kabuli_wild_13840191	1399936565	*Ca_kabuli_Chr04*	13840191	T/A	Gene_Ca_04608	SYNONYMOUS_CODING	NA
Kabuli_wild_13840227	1399936566	*Ca_kabuli_Chr04*	13840227	A/C	Gene_Ca_04608	SYNONYMOUS_CODING	NA
Kabuli_wild_13840251	1399936567	*Ca_kabuli_Chr04*	13840251	A/G	Gene_Ca_04608	SYNONYMOUS_CODING	NA
Kabuli_wild_13840484	1399936568	*Ca_kabuli_Chr04*	13840484	T/C	Gene_Ca_04608	SYNONYMOUS_CODING	NA
Kabuli_wild_13841340	1399936569	*Ca_kabuli_Chr04*	13841340	G/T	Gene_Ca_04608	NON_SYNONYMOUS_CODING	NA
Kabuli_wild_13845581	1399936570	*Ca_kabuli_Chr04*	13845581	A/T	Gene_Ca_04608	UPSTREAM	NA
Kabuli_wild_13845639	1399936571	*Ca_kabuli_Chr04*	13845639	A/C	Gene_Ca_04608	UPSTREAM	NA
Kabuli_wild_13845675	1399936572	*Ca_kabuli_Chr04*	13845675	T/A	NA	INTERGENIC	NA
Kabuli_wild_13858799	1399936573	*Ca_kabuli_Chr04*	13858799	C/T	Gene_Ca_04609	INTRON	Sugar/inositol transporter
Kabuli_wild_13858801	1399936574	*Ca_kabuli_Chr04*	13858801	A/C	Gene_Ca_04609	INTRON	Sugar/inositol transporter
Kabuli_wild_13858972	1399936575	*Ca_kabuli_Chr04*	13858972	A/G	Gene_Ca_04609	SYNONYMOUS_CODING	Sugar/inositol transporter
Kabuli_wild_13897588	1399936576	*Ca_kabuli_Chr04*	13897588	C/T	NA	INTERGENIC	NA
Kabuli_wild_13899990	1399936577	*Ca_kabuli_Chr04*	13899990	T/C	NA	INTERGENIC	NA
Kabuli_wild_13900013	1399936578	*Ca_kabuli_Chr04*	13900013	A/G	NA	INTERGENIC	NA
Kabuli_wild_13923215	1399936579	*Ca_kabuli_Chr04*	13923215	T/A	NA	INTERGENIC	NA
Kabuli_wild_13923411	1399936580	*Ca_kabuli_Chr04*	13923411	T/A	NA	INTERGENIC	NA
Kabuli_wild_13949513	1399936581	*Ca_kabuli_Chr04*	13949513	A/C	NA	INTERGENIC	NA
Kabuli_wild_13949580	1399936582	*Ca_kabuli_Chr04*	13949580	C/G	NA	INTERGENIC	NA
Kabuli_wild_13954832	1399936583	*Ca_kabuli_Chr04*	13954832	G/A	Gene_Ca_04617	DOWNSTREAM	Glycosyltransferase,group1
Kabuli_wild_13968291	1399936584	*Ca_kabuli_Chr04*	13968291	C/T	Gene_Ca_04621	DOWNSTREAM	Leucine-rich repeat, ribonuclease inhibitor subtype
Kabuli_wild_13968354	1399936585	*Ca_kabuli_Chr04*	13968354	A/G	Gene_Ca_04621	DOWNSTREAM	Leucine-rich repeat, ribonuclease inhibitor subtype
Kabuli_wild_13968397	1399936586	*Ca_kabuli_Chr04*	13968397	A/G	Gene_Ca_04621	DOWNSTREAM	Leucine-rich repeat, ribonuclease inhibitor subtype
Kabuli_wild_13982970	1399936587	*Ca_kabuli_Chr04*	13982970	G/A	NA	INTERGENIC	NA
Kabuli_wild_13985087	1399936588	*Ca_kabuli_Chr04*	13985087	A/G	Gene_Ca_04623	INTRON	Protein of unknown function DUF171
Kabuli_wild_14031548	1399936589	*Ca_kabuli_Chr04*	14031548	A/G	Gene_Ca_04628	DOWNSTREAM	NA
Kabuli_wild_14056908	1399936590	*Ca_kabuli_Chr04*	14056908	A/G	NA	INTERGENIC	NA
Kabuli_wild_14073004	1399936591	*Ca_kabuli_Chr04*	14073004	C/A	NA	INTERGENIC	NA
Kabuli_wild_14073044	1399936592	*Ca_kabuli_Chr04*	14073044	C/T	NA	INTERGENIC	NA
Kabuli_wild_14073045	1399936593	*Ca_kabuli_Chr04*	14073045	G/A	NA	INTERGENIC	NA
Kabuli_wild_14073056	1399936594	*Ca_kabuli_Chr04*	14073056	C/T	NA	INTERGENIC	NA
Kabuli_wild_14073059	1399936595	*Ca_kabuli_Chr04*	14073059	C/T	NA	INTERGENIC	NA
Kabuli_wild_14082157	1399936596	*Ca_kabuli_Chr04*	14082157	C/A	Gene_Ca_04635	INTRON	PDZ-bindingprotein,CRIPT
Kabuli_wild_14082158	1399936597	*Ca_kabuli_Chr04*	14082158	T/G	Gene_Ca_04635	INTRON	PDZ-bindingprotein,CRIPT
Kabuli_wild_14082186	1399936598	*Ca_kabuli_Chr04*	14082186	G/A	Gene_Ca_04635	INTRON	PDZ-bindingprotein,CRIPT
Kabuli_wild_14082210	1399936599	*Ca_kabuli_Chr04*	14082210	T/C	Gene_Ca_04635	INTRON	PDZ-bindingprotein,CRIPT

SNP IDs	NCBI dbSNP accession IDs (NCBI_SS#)	Chromosomes	Physical positions (bp)	SNPs	Gene accession IDs	Sequence components of genome	Putative functions
Kabuli_wild_14082285	1399936600	*Ca_kabuli_Chr04*	14082285	C/G	Gene_Ca_04635	DOWNSTREAM	PDZ-bindingprotein,CRIPT
Kabuli_wild_14093027	1399936601	*Ca_kabuli_Chr04*	14093027	A/G	Gene_Ca_04636	SYNONYMOUS_CODING	Ubiquitin
Kabuli_wild_14093096	1399936602	*Ca_kabuli_Chr04*	14093096	A/C	Gene_Ca_04636	SYNONYMOUS_CODING	Ubiquitin
Kabuli_wild_14093105	1399936603	*Ca_kabuli_Chr04*	14093105	C/A	Gene_Ca_04636	SYNONYMOUS_CODING	Ubiquitin
Kabuli_wild_14093108	1399936604	*Ca_kabuli_Chr04*	14093108	A/T	Gene_Ca_04636	SYNONYMOUS_CODING	Ubiquitin
Kabuli_wild_14093110	1399936605	*Ca_kabuli_Chr04*	14093110	G/A	Gene_Ca_04636	NON_SYNONYMOUS_CODING	Ubiquitin
Kabuli_wild_14093111	1399936606	*Ca_kabuli_Chr04*	14093111	G/A	Gene_Ca_04636	SYNONYMOUS_CODING	Ubiquitin
Kabuli_wild_14093116	1399936607	*Ca_kabuli_Chr04*	14093116	G/A	Gene_Ca_04636	NON_SYNONYMOUS_CODING	Ubiquitin
Kabuli_wild_14093117	1399936608	*Ca_kabuli_Chr04*	14093117	G/A	Gene_Ca_04636	SYNONYMOUS_CODING	Ubiquitin
Kabuli_wild_14093120	1399936609	*Ca_kabuli_Chr04*	14093120	T/G	Gene_Ca_04636	SYNONYMOUS_CODING	Ubiquitin
Kabuli_wild_14093132	1399936610	*Ca_kabuli_Chr04*	14093132	G/A	Gene_Ca_04636	SYNONYMOUS_CODING	Ubiquitin
Kabuli_wild_14093144	1399936611	*Ca_kabuli_Chr04*	14093144	A/T	Gene_Ca_04636	SYNONYMOUS_CODING	Ubiquitin
Kabuli_wild_14093147	1399936612	*Ca_kabuli_Chr04*	14093147	T/A	Gene_Ca_04636	NON_SYNONYMOUS_CODING	Ubiquitin
Kabuli_wild_14093156	1399936613	*Ca_kabuli_Chr04*	14093156	G/A	Gene_Ca_04636	SYNONYMOUS_CODING	Ubiquitin
Kabuli_wild_14093159	1399936614	*Ca_kabuli_Chr04*	14093159	A/G	Gene_Ca_04636	SYNONYMOUS_CODING	Ubiquitin
Kabuli_wild_14104096	1399936615	*Ca_kabuli_Chr04*	14104096	A/C	NA	INTERGENIC	NA
Kabuli_wild_14112911	1399936616	*Ca_kabuli_Chr04*	14112911	C/A	Gene_Ca_04640	UPSTREAM	Ubiquitin
Kabuli_wild_14113281	1399936617	*Ca_kabuli_Chr04*	14113281	G/C	Gene_Ca_04640	UPSTREAM	Ubiquitin
Kabuli_wild_14132659	1399936618	*Ca_kabuli_Chr04*	14132659	A/C	NA	INTERGENIC	NA
Kabuli_wild_14132677	1399936619	*Ca_kabuli_Chr04*	14132677	T/G	NA	INTERGENIC	NA
Kabuli_wild_14132711	1399936620	*Ca_kabuli_Chr04*	14132711	A/G	NA	INTERGENIC	NA
Kabuli_wild_14132715	1399936621	*Ca_kabuli_Chr04*	14132715	A/T	NA	INTERGENIC	NA
Kabuli_wild_14132717	1399936622	*Ca_kabuli_Chr04*	14132717	G/A	NA	INTERGENIC	NA
Kabuli_wild_14132719	1399936623	*Ca_kabuli_Chr04*	14132719	A/C	NA	INTERGENIC	NA
Kabuli_wild_14132724	1399936624	*Ca_kabuli_Chr04*	14132724	A/G	NA	INTERGENIC	NA
Kabuli_wild_14132729	1399936625	*Ca_kabuli_Chr04*	14132729	T/A	NA	INTERGENIC	NA
Kabuli_wild_14132733	1399936626	*Ca_kabuli_Chr04*	14132733	G/C	NA	INTERGENIC	NA
Kabuli_wild_14132734	1399936627	*Ca_kabuli_Chr04*	14132734	T/G	NA	INTERGENIC	NA
Kabuli_wild_14132747	1399936628	*Ca_kabuli_Chr04*	14132747	G/T	NA	INTERGENIC	NA
Kabuli_wild_14132749	1399936629	*Ca_kabuli_Chr04*	14132749	A/T	NA	INTERGENIC	NA
Kabuli_wild_14132752	1399936630	*Ca_kabuli_Chr04*	14132752	T/A	NA	INTERGENIC	NA
Kabuli_wild_14132760	1399936631	*Ca_kabuli_Chr04*	14132760	A/C	NA	INTERGENIC	NA
Kabuli_wild_14132781	1399936632	*Ca_kabuli_Chr04*	14132781	A/C	NA	INTERGENIC	NA
Kabuli_wild_14149357	1399936633	*Ca_kabuli_Chr04*	14149357	G/A	Gene_Ca_04645	NON_SYNONYMOUS_CODING	Protein kinase,catalytic domain
Kabuli_wild_14149588	1399936634	*Ca_kabuli_Chr04*	14149588	A/G	Gene_Ca_04645	NON_SYNONYMOUS_CODING	Protein kinase,catalytic domain
Kabuli_wild_14149589	1399936635	*Ca_kabuli_Chr04*	14149589	G/A	Gene_Ca_04645	NON_SYNONYMOUS_CODING	Protein kinase,catalytic domain
Kabuli_wild_14155419	1399936636	*Ca_kabuli_Chr04*	14155419	C/T	Gene_Ca_04646	NON_SYNONYMOUS_CODING	Protein kinase,catalytic domain
Kabuli_wild_14155978	1399936637	*Ca_kabuli_Chr04*	14155978	A/G	Gene_Ca_04646	SYNONYMOUS_CODING	Protein kinase,catalytic domain
Kabuli_wild_14156014	1399936638	*Ca_kabuli_Chr04*	14156014	C/T	Gene_Ca_04646	SYNONYMOUS_CODING	Protein kinase,catalytic domain
Kabuli_wild_14163122	1399936639	*Ca_kabuli_Chr04*	14163122	T/C	NA	INTERGENIC	NA
Kabuli_wild_14177500	1399936640	*Ca_kabuli_Chr04*	14177500	A/C	NA	INTERGENIC	NA
Kabuli_wild_14181589	1399936641	*Ca_kabuli_Chr04*	14181589	A/C	NA	INTERGENIC	NA
Kabuli_wild_14189356	1399936642	*Ca_kabuli_Chr04*	14189356	A/C	Gene_Ca_04652	NON_SYNONYMOUS_CODING	Protein kinase,catalytic domain
Kabuli_wild_14199331	1399936643	*Ca_kabuli_Chr04*	14199331	A/C	Gene_Ca_04653	SYNONYMOUS_CODING	Protein kinase,catalytic domain

SNP IDs	NCBI dbSNP accession IDs (NCBI_SS#)	Chromosomes	Physical positions (bp)	SNPs	Gene accession IDs	Sequence components of genome	Putative functions
Kabuli_wild_14270758	1399936644	*Ca_kabuli_Chr04*	14270758	C/T	NA	INTERGENIC	NA
Kabuli_wild_14270763	1399936645	*Ca_kabuli_Chr04*	14270763	G/A	NA	INTERGENIC	NA
Kabuli_wild_14270765	1399936646	*Ca_kabuli_Chr04*	14270765	A/C	NA	INTERGENIC	NA
Kabuli_wild_14270766	1399936647	*Ca_kabuli_Chr04*	14270766	G/A	NA	INTERGENIC	NA
Kabuli_wild_14270827	1399936648	*Ca_kabuli_Chr04*	14270827	C/T	NA	INTERGENIC	NA
Kabuli_wild_14270830	1399936649	*Ca_kabuli_Chr04*	14270830	G/A	NA	INTERGENIC	NA
Kabuli_wild_14270847	1399936650	*Ca_kabuli_Chr04*	14270847	G/T	NA	INTERGENIC	NA
Kabuli_wild_14271784	1399936651	*Ca_kabuli_Chr04*	14271784	C/T	NA	INTERGENIC	NA
Kabuli_wild_14271793	1399936652	*Ca_kabuli_Chr04*	14271793	A/G	NA	INTERGENIC	NA
Kabuli_wild_14399908	1399936653	*Ca_kabuli_Chr04*	14399908	A/G	Gene_Ca_05664	DOWNSTREAM	Transposase,Ptta/En/Spm,plant
Kabuli_wild_14515543	1399936654	*Ca_kabuli_Chr04*	14515543	T/A	NA	INTERGENIC	NA
Kabuli_wild_14657743	1399936655	*Ca_kabuli_Chr04*	14657743	C/T	Gene_Ca_05639	INTRON	Protein of unknown function DUF566
Kabuli_wild_14699291	1399936656	*Ca_kabuli_Chr04*	14699291	C/A	Gene_Ca_05634	NON_SYNONYMOUS_CODING	NA
Kabuli_wild_14748051	1399936657	*Ca_kabuli_Chr04*	14748051	A/C	Gene_Ca_05628	SYNONYMOUS_CODING	Protein of unknown function DUF620
Kabuli_wild_14770435	1399936658	*Ca_kabuli_Chr04*	14770435	G/A	Gene_Ca_05627	NON_SYNONYMOUS_CODING	Peptidase C48, SUMO/Sentrin/Ubl1
Kabuli_wild_14770448	1399936659	*Ca_kabuli_Chr04*	14770448	C/T	Gene_Ca_05627	NON_SYNONYMOUS_CODING	Peptidase C48, SUMO/Sentrin/Ubl1
Kabuli_wild_14785557	1399936660	*Ca_kabuli_Chr04*	14785557	A/C	Gene_Ca_05626	DOWNSTREAM	WD40 repeat
Kabuli_wild_14831204	1399936661	*Ca_kabuli_Chr04*	14831204	A/T	NA	INTERGENIC	NA
Kabuli_wild_14834479	1399936662	*Ca_kabuli_Chr04*	14834479	T/C	NA	INTERGENIC	NA
Kabuli_wild_14870641	1399936663	*Ca_kabuli_Chr04*	14870641	G/A	Gene_Ca_05614	SYNONYMOUS_CODING	Aminoglycoside phosphotransferase
Kabuli_wild_14870658	1399936664	*Ca_kabuli_Chr04*	14870658	T/G	Gene_Ca_05614	NON_SYNONYMOUS_CODING	Aminoglycoside phosphotransferase
Kabuli_wild_14985754	1399936665	*Ca_kabuli_Chr04*	14985754	C/A	NA	INTERGENIC	NA
Kabuli_wild_15240678	1399936666	*Ca_kabuli_Chr04*	15240678	T/C	NA	INTERGENIC	NA
Kabuli_wild_15357695	1399936667	*Ca_kabuli_Chr04*	15357695	A/G	NA	INTERGENIC	NA
Kabuli_wild_15394470	1399936668	*Ca_kabuli_Chr04*	15394470	T/C	NA	INTERGENIC	NA
Kabuli_wild_15542597	1399936669	*Ca_kabuli_Chr04*	15542597	A/G	Gene_Ca_05547	SYNONYMOUS_CODING	Zinc finger,CCCH-type
Kabuli_wild_15552690	1399936670	*Ca_kabuli_Chr04*	15552690	C/T	NA	INTERGENIC	NA
Kabuli_wild_15697128	1399936671	*Ca_kabuli_Chr04*	15697128	A/G	Gene_Ca_05536	NON_SYNONYMOUS_CODING	Phox/Bem1p
Kabuli_wild_15697303	1399936672	*Ca_kabuli_Chr04*	15697303	G/T	Gene_Ca_05536	SYNONYMOUS_CODING	Phox/Bem1p
Kabuli_wild_15697492	1399936673	*Ca_kabuli_Chr04*	15697492	G/A	Gene_Ca_05536	SYNONYMOUS_CODING	Phox/Bem1p
Kabuli_wild_15698179	1399936674	*Ca_kabuli_Chr04*	15698179	G/A	NA	INTERGENIC	NA
Kabuli_wild_15698212	1399936675	*Ca_kabuli_Chr04*	15698212	G/T	NA	INTERGENIC	NA
Kabuli_wild_15698233	1399936676	*Ca_kabuli_Chr04*	15698233	A/C	NA	INTERGENIC	NA
Kabuli_wild_15698305	1399936677	*Ca_kabuli_Chr04*	15698305	T/C	NA	INTERGENIC	NA
Kabuli_wild_15698420	1399936678	*Ca_kabuli_Chr04*	15698420	G/C	NA	INTERGENIC	NA
Kabuli_wild_15698444	1399936679	*Ca_kabuli_Chr04*	15698444	C/T	NA	INTERGENIC	NA
Kabuli_wild_15698456	1399936680	*Ca_kabuli_Chr04*	15698456	T/C	NA	INTERGENIC	NA
Kabuli_wild_15698486	1399936681	*Ca_kabuli_Chr04*	15698486	T/C	NA	INTERGENIC	NA
Kabuli_wild_15700603	1399936682	*Ca_kabuli_Chr04*	15700603	A/T	Gene_Ca_05535	DOWNSTREAM	WD40 repeat
Kabuli_wild_15736619	1399936683	*Ca_kabuli_Chr04*	15736619	T/G	Gene_Ca_05530	INTRON	Cytidylyltransferase
Kabuli_wild_15736633	1399936684	*Ca_kabuli_Chr04*	15736633	T/G	Gene_Ca_05530	INTRON	Cytidylyltransferase
Kabuli_wild_15752567	1399936685	*Ca_kabuli_Chr04*	15752567	G/C	Gene_Ca_05529	NON_SYNONYMOUS_CODING	Glycosyltransferase,family20
Kabuli_wild_15766601	1399936686	*Ca_kabuli_Chr04*	15766601	G/T	NA	INTERGENIC	NA
Kabuli_wild_15766606	1399936687	*Ca_kabuli_Chr04*	15766606	T/A	NA	INTERGENIC	NA

SNP IDs	NCBI dbSNP accession IDs (NCBI_SS#)	Chromosomes	Physical positions (bp)	SNPs	Gene accession IDs	Sequence components of genome	Putative functions
Kabuli_wild_15766610	1399936688	*Ca_kabuli_Chr04*	15766610	T/G	NA	INTERGENIC	NA
Kabuli_wild_15766626	1399936689	*Ca_kabuli_Chr04*	15766626	G/A	NA	INTERGENIC	NA
Kabuli_wild_15766631	1399936690	*Ca_kabuli_Chr04*	15766631	G/T	NA	INTERGENIC	NA
Kabuli_wild_15766640	1399936691	*Ca_kabuli_Chr04*	15766640	T/G	NA	INTERGENIC	NA
Kabuli_wild_15772275	1399936692	*Ca_kabuli_Chr04*	15772275	A/G	Gene_Ca_05528	SYNONYMOUS_CODING	Reticulon
Kabuli_wild_15825621	1399936693	*Ca_kabuli_Chr04*	15825621	A/T	Gene_Ca_05524	INTRON	Protein of unknown function DUF789
Kabuli_wild_15860359	1399936694	*Ca_kabuli_Chr04*	15860359	C/A	NA	INTERGENIC	NA
Kabuli_wild_15860388	1399936695	*Ca_kabuli_Chr04*	15860388	A/G	NA	INTERGENIC	NA
Kabuli_wild_15900602	1399936696	*Ca_kabuli_Chr04*	15900602	G/A	NA	INTERGENIC	NA
Kabuli_wild_15914525	1399936697	*Ca_kabuli_Chr04*	15914525	T/C	Gene_Ca_05516	SYNONYMOUS_CODING	Protein kinase,catalytic domain
Kabuli_wild_15925936	1399936698	*Ca_kabuli_Chr04*	15925936	T/G	NA	INTERGENIC	NA
Kabuli_wild_15926160	1399936699	*Ca_kabuli_Chr04*	15926160	A/G	NA	INTERGENIC	NA
Kabuli_wild_15934607	1399936700	*Ca_kabuli_Chr04*	15934607	A/T	Gene_Ca_05513	INTRON	Beta-lactamase-like
Kabuli_wild_15934901	1399936701	*Ca_kabuli_Chr04*	15934901	T/G	NA	INTERGENIC	NA
Kabuli_wild_15942209	1399936702	*Ca_kabuli_Chr04*	15942209	C/T	Gene_Ca_05512	INTRON	Heat shock protein DnaJ,N-terminal
Kabuli_wild_15942388	1399936703	*Ca_kabuli_Chr04*	15942388	C/T	Gene_Ca_05512	INTRON	Heat shock protein DnaJ,N-terminal
Kabuli_wild_15957070	1399936704	*Ca_kabuli_Chr04*	15957070	T/A	Gene_Ca_05511	DOWNSTREAM	Zinc finger,C2H2-type
Kabuli_wild_16045870	1399936705	*Ca_kabuli_Chr04*	16045870	G/A	Gene_Ca_05504	INTRON	Glutamyl/glutaminyl-tRNAsynthetase,classIc
Kabuli_wild_16046928	1399936706	*Ca_kabuli_Chr04*	16046928	T/C	Gene_Ca_05504	SYNONYMOUS_CODING	Glutamyl/glutaminyl-tRNAsynthetase,classIc
Kabuli_wild_16078354	1399936707	*Ca_kabuli_Chr04*	16078354	T/C	NA	INTERGENIC	NA
Kabuli_wild_16148668	1399936708	*Ca_kabuli_Chr04*	16148668	A/C	NA	INTERGENIC	NA
Kabuli_wild_16149795	1399936709	*Ca_kabuli_Chr04*	16149795	C/T	NA	INTERGENIC	NA
Kabuli_wild_16149998	1399936710	*Ca_kabuli_Chr04*	16149998	T/C	NA	INTERGENIC	NA
Kabuli_wild_16150106	1399936711	*Ca_kabuli_Chr04*	16150106	T/C	NA	INTERGENIC	NA
Kabuli_wild_16184259	1399936712	*Ca_kabuli_Chr04*	16184259	T/G	Gene_Ca_05493	INTRON	Domain of unknown function DUF292,eukaryotic
Kabuli_wild_16184265	1399936713	*Ca_kabuli_Chr04*	16184265	A/T	Gene_Ca_05493	INTRON	Domain of unknown function DUF292,eukaryotic
Kabuli_wild_16184275	1399936714	*Ca_kabuli_Chr04*	16184275	T/C	Gene_Ca_05493	INTRON	Domain of unknown function DUF292,eukaryotic
Kabuli_wild_16184284	1399936715	*Ca_kabuli_Chr04*	16184284	T/C	Gene_Ca_05493	INTRON	Domain of unknown function DUF292,eukaryotic
Kabuli_wild_16184343	1399936716	*Ca_kabuli_Chr04*	16184343	A/G	Gene_Ca_05493	INTRON	Domain of unknown function DUF292,eukaryotic
Kabuli_wild_16278600	1399936717	*Ca_kabuli_Chr04*	16278600	C/T	Gene_Ca_05487	NON_SYNONYMOUS_CODING	Zinc finger,B-box
Kabuli_wild_16278671	1399936718	*Ca_kabuli_Chr04*	16278671	T/A	Gene_Ca_05487	SYNONYMOUS_CODING	Zinc finger,B-box
Kabuli_wild_16571288	1399936719	*Ca_kabuli_Chr04*	16571288	T/C	Gene_Ca_05467	INTRON	ATPase,AAA+type,core
Kabuli_wild_16622514	1399936720	*Ca_kabuli_Chr04*	16622514	C/A	Gene_Ca_05461	NON_SYNONYMOUS_CODING	KOW
Kabuli_wild_16628529	1399936721	*Ca_kabuli_Chr04*	16628529	C/A	NA	INTERGENIC	NA
Kabuli_wild_16628540	1399936722	*Ca_kabuli_Chr04*	16628540	G/A	NA	INTERGENIC	NA
Kabuli_wild_16628559	1399936723	*Ca_kabuli_Chr04*	16628559	T/G	NA	INTERGENIC	NA
Kabuli_wild_16628568	1399936724	*Ca_kabuli_Chr04*	16628568	T/G	NA	INTERGENIC	NA
Kabuli_wild_16628591	1399936725	*Ca_kabuli_Chr04*	16628591	G/T	NA	INTERGENIC	NA
Kabuli_wild_16628594	1399936726	*Ca_kabuli_Chr04*	16628594	T/G	NA	INTERGENIC	NA
Kabuli_wild_16629559	1399936727	*Ca_kabuli_Chr04*	16629559	T/G	NA	INTERGENIC	NA
Kabuli_wild_16629560	1399936728	*Ca_kabuli_Chr04*	16629560	G/T	NA	INTERGENIC	NA
Kabuli_wild_16629638	1399936729	*Ca_kabuli_Chr04*	16629638	C/A	NA	INTERGENIC	NA
Kabuli_wild_16669866	1399936730	*Ca_kabuli_Chr04*	16669866	C/G	Gene_Ca_05457	NON_SYNONYMOUS_CODING	Inner centromere protein, ARK-binding domain
Kabuli_wild_16749740	1399936731	*Ca_kabuli_Chr04*	16749740	A/G	Gene_Ca_05450	UPSTREAM	RNA recognition motif domain

SNP IDs	NCBI dbSNP accession IDs (NCBI_SS#)	Chromosomes	Physical positions (bp)	SNPs	Gene accession IDs	Sequence components of genome	Putative functions
Kabuli_wild_16750416	1399936732	*Ca_kabuli_Chr04*	16750416	A/G	Gene_Ca_05450	UPSTREAM	RNA recognition motif domain
Kabuli_wild_16789476	1399936733	*Ca_kabuli_Chr04*	16789476	G/A	Gene_Ca_05446	SYNONYMOUS_CODING	Haemerythrin/HHEcation-bindingmotif
Kabuli_wild_16926614	1399936734	*Ca_kabuli_Chr04*	16926614	A/T	Gene_Ca_05430	NON_SYNONYMOUS_CODING	Zinc finger,C2H2-type
Kabuli_wild_16975510	1399936735	*Ca_kabuli_Chr04*	16975510	A/G	Gene_Ca_05427	INTRON	Nucleotide-diphospho-sugartransferase,predicted
Kabuli_wild_17028368	1399936736	*Ca_kabuli_Chr04*	17028368	G/A	NA	INTERGENIC	NA
Kabuli_wild_17032673	1399936737	*Ca_kabuli_Chr04*	17032673	C/T	NA	INTERGENIC	NA
Kabuli_wild_17032676	1399936738	*Ca_kabuli_Chr04*	17032676	T/C	NA	INTERGENIC	NA
Kabuli_wild_17038805	1399936739	*Ca_kabuli_Chr04*	17038805	C/G	Gene_Ca_05419	INTRON	D-isomerspecific2-hydroxyaciddehydrogenase,catalytic domain
Kabuli_wild_17039135	1399936740	*Ca_kabuli_Chr04*	17039135	T/C	Gene_Ca_05419	NON_SYNONYMOUS_CODING	D-isomerspecific2-hydroxyaciddehydrogenase,catalytic domain
Kabuli_wild_17059519	1399936741	*Ca_kabuli_Chr04*	17059519	G/T	Gene_Ca_05417	SYNONYMOUS_CODING	Phospholipase-like,arabidopsis
Kabuli_wild_17097879	1399936742	*Ca_kabuli_Chr04*	17097879	A/G	NA	INTERGENIC	NA
Kabuli_wild_17097928	1399936743	*Ca_kabuli_Chr04*	17097928	A/G	NA	INTERGENIC	NA
Kabuli_wild_17120563	1399936744	*Ca_kabuli_Chr04*	17120563	C/T	Gene_Ca_05412	INTRON	Monooxygenase,FAD-binding
Kabuli_wild_17147875	1399936745	*Ca_kabuli_Chr04*	17147875	C/T	Gene_Ca_05409	DOWNSTREAM	NA
Kabuli_wild_17147926	1399936746	*Ca_kabuli_Chr04*	17147926	T/C	Gene_Ca_05409	DOWNSTREAM	NA
Kabuli_wild_17187560	1399936747	*Ca_kabuli_Chr04*	17187560	C/G	NA	INTERGENIC	NA
Kabuli_wild_17191562	1399936748	*Ca_kabuli_Chr04*	17191562	T/A	Gene_Ca_05405	SYNONYMOUS_CODING	Protein phosphatase 2C, manganese/magnesium aspartate binding site
Kabuli_wild_17199316	1399936749	*Ca_kabuli_Chr04*	17199316	T/C	Gene_Ca_05404	SYNONYMOUS_CODING	Domain of unknown function DUF632
Kabuli_wild_17199477	1399936750	*Ca_kabuli_Chr04*	17199477	G/A	Gene_Ca_05404	SYNONYMOUS_CODING	Domain of unknown function DUF632
Kabuli_wild_17199573	1399936751	*Ca_kabuli_Chr04*	17199573	A/G	Gene_Ca_05404	SYNONYMOUS_CODING	Domain of unknown function DUF632
Kabuli_wild_17202770	1399936752	*Ca_kabuli_Chr04*	17202770	A/G	NA	INTERGENIC	NA
Kabuli_wild_17225332	1399936753	*Ca_kabuli_Chr04*	17225332	C/A	Gene_Ca_05400	NON_SYNONYMOUS_CODING	HEAT
Kabuli_wild_17225334	1399936754	*Ca_kabuli_Chr04*	17225334	G/A	Gene_Ca_05400	SYNONYMOUS_CODING	HEAT
Kabuli_wild_17237119	1399936755	*Ca_kabuli_Chr04*	17237119	G/A	Gene_Ca_05400	INTRON	HEAT
Kabuli_wild_17264248	1399936756	*Ca_kabuli_Chr04*	17264248	C/A	Gene_Ca_05398	SYNONYMOUS_CODING	Major facilitator superfamily MFS-1
Kabuli_wild_17264592	1399936757	*Ca_kabuli_Chr04*	17264592	G/A	Gene_Ca_05398	INTRON	Major facilitator superfamily MFS-1
Kabuli_wild_17264621	1399936758	*Ca_kabuli_Chr04*	17264621	C/G	Gene_Ca_05398	INTRON	Major facilitator superfamily MFS-1
Kabuli_wild_17264637	1399936759	*Ca_kabuli_Chr04*	17264637	C/T	Gene_Ca_05398	INTRON	Major facilitator superfamily MFS-1
Kabuli_wild_17273774	1399936760	*Ca_kabuli_Chr04*	17273774	C/T	NA	INTERGENIC	NA
Kabuli_wild_17275870	1399936761	*Ca_kabuli_Chr04*	17275870	C/T	NA	INTERGENIC	NA
Kabuli_wild_17275977	1399936762	*Ca_kabuli_Chr04*	17275977	A/G	NA	INTERGENIC	NA
Kabuli_wild_17281374	1399936763	*Ca_kabuli_Chr04*	17281374	G/A	NA	INTERGENIC	NA
Kabuli_wild_17282119	1399936764	*Ca_kabuli_Chr04*	17282119	T/C	NA	INTERGENIC	NA
Kabuli_wild_17282148	1399936765	*Ca_kabuli_Chr04*	17282148	G/C	NA	INTERGENIC	NA
Kabuli_wild_17282336	1399936766	*Ca_kabuli_Chr04*	17282336	G/T	NA	INTERGENIC	NA
Kabuli_wild_17282345	1399936767	*Ca_kabuli_Chr04*	17282345	G/C	NA	INTERGENIC	NA
Kabuli_wild_17289594	1399936768	*Ca_kabuli_Chr04*	17289594	G/C	Gene_Ca_05393	UPSTREAM	Ankyrinrepeat-containingdomain
Kabuli_wild_17297498	1399936769	*Ca_kabuli_Chr04*	17297498	G/T	Gene_Ca_05392	INTRON	Ankyrinrepeat-containingdomain
Kabuli_wild_17297563	1399936770	*Ca_kabuli_Chr04*	17297563	A/G	Gene_Ca_05392	INTRON	Ankyrinrepeat-containingdomain
Kabuli_wild_17297567	1399936771	*Ca_kabuli_Chr04*	17297567	G/A	Gene_Ca_05392	INTRON	Ankyrinrepeat-containingdomain
Kabuli_wild_17309656	1399936772	*Ca_kabuli_Chr04*	17309656	A/C	Gene_Ca_05391	SYNONYMOUS_CODING	Phosphatidylinositol3-/4-kinase,catalytic
Kabuli_wild_17345436	1399936773	*Ca_kabuli_Chr04*	17345436	G/T	Gene_Ca_05389	INTRON	Fibrillarin
Kabuli_wild_17345483	1399936774	*Ca_kabuli_Chr04*	17345483	C/A	Gene_Ca_05389	INTRON	Fibrillarin
Kabuli_wild_17345500	1399936775	*Ca_kabuli_Chr04*	17345500	G/A	Gene_Ca_05389	SYNONYMOUS_CODING	Fibrillarin

SNP IDs	NCBI dbSNP accession IDs (NCBI_SS#)	Chromosomes	Physical positions (bp)	SNPs	Gene accession IDs	Sequence components of genome	Putative functions
Kabuli_wild_17358736	1399936776	*Ca_kabuli_Chr04*	17358736	C/T	Gene_Ca_05388	INTRON	Protein kinase,catalytic domain
Kabuli_wild_17361143	1399936777	*Ca_kabuli_Chr04*	17361143	T/A	Gene_Ca_05388	INTRON	Protein kinase,catalytic domain
Kabuli_wild_17396325	1399936778	*Ca_kabuli_Chr04*	17396325	A/G	Gene_Ca_05386	INTRON	Zinc finger, RING-type
Kabuli_wild_17396349	1399936779	*Ca_kabuli_Chr04*	17396349	T/G	Gene_Ca_05386	INTRON	Zinc finger, RING-type
Kabuli_wild_17396352	1399936780	*Ca_kabuli_Chr04*	17396352	T/G	Gene_Ca_05386	INTRON	Zinc finger, RING-type
Kabuli_wild_17429456	1399936781	*Ca_kabuli_Chr04*	17429456	A/T	Gene_Ca_05384	SYNONYMOUS_CODING	Transcription factor jumonji/aspartylbeta-hydroxylase
Kabuli_wild_17429457	1399936782	*Ca_kabuli_Chr04*	17429457	A/T	Gene_Ca_05384	SYNONYMOUS_CODING	Transcription factor jumonji/aspartylbeta-hydroxylase
Kabuli_wild_17429468	1399936783	*Ca_kabuli_Chr04*	17429468	T/C	Gene_Ca_05384	SYNONYMOUS_CODING	Transcription factor jumonji/aspartylbeta-hydroxylase
Kabuli_wild_17429699	1399936784	*Ca_kabuli_Chr04*	17429699	G/C	Gene_Ca_05384	SYNONYMOUS_CODING	Transcription factor jumonji/aspartylbeta-hydroxylase
Kabuli_wild_17429735	1399936785	*Ca_kabuli_Chr04*	17429735	A/G	Gene_Ca_05384	SYNONYMOUS_CODING	Transcription factor jumonji/aspartylbeta-hydroxylase
Kabuli_wild_17429742	1399936786	*Ca_kabuli_Chr04*	17429742	A/G	Gene_Ca_05384	SYNONYMOUS_CODING	Transcription factor jumonji/aspartylbeta-hydroxylase
Kabuli_wild_17429747	1399936787	*Ca_kabuli_Chr04*	17429747	G/A	Gene_Ca_05384	SYNONYMOUS_CODING	Transcription factor jumonji/aspartylbeta-hydroxylase
Kabuli_wild_17432425	1399936788	*Ca_kabuli_Chr04*	17432425	G/T	Gene_Ca_05384	INTRON	Transcription factor jumonji/aspartylbeta-hydroxylase
Kabuli_wild_17432500	1399936789	*Ca_kabuli_Chr04*	17432500	G/C	Gene_Ca_05384	INTRON	Transcription factor jumonji/aspartylbeta-hydroxylase
Kabuli_wild_17433782	1399936790	*Ca_kabuli_Chr04*	17433782	G/A	Gene_Ca_05384	INTRON	Transcription factor jumonji/aspartylbeta-hydroxylase
Kabuli_wild_17433914	1399936791	*Ca_kabuli_Chr04*	17433914	G/A	Gene_Ca_05384	INTRON	Transcription factor jumonji/aspartylbeta-hydroxylase
Kabuli_wild_17444578	1399936792	*Ca_kabuli_Chr04*	17444578	A/C	Gene_Ca_05384	DOWNSTREAM	Transcription factor jumonji/aspartylbeta-hydroxylase
Kabuli_wild_17472992	1399936793	*Ca_kabuli_Chr04*	17472992	T/G	NA	INTERGENIC	NA
Kabuli_wild_17473234	1399936794	*Ca_kabuli_Chr04*	17473234	C/A	NA	INTERGENIC	NA
Kabuli_wild_17473500	1399936795	*Ca_kabuli_Chr04*	17473500	T/C	NA	INTERGENIC	NA
Kabuli_wild_17603613	1399936796	*Ca_kabuli_Chr04*	17603613	T/C	NA	INTERGENIC	NA
Kabuli_wild_17609694	1399936797	*Ca_kabuli_Chr04*	17609694	G/A	Gene_Ca_05370	DOWNSTREAM	Prefoldinsubunit
Kabuli_wild_17620966	1399936798	*Ca_kabuli_Chr04*	17620966	A/G	Gene_Ca_05368	INTRON	Dihydroorotatedehydrogenase, conserved site
Kabuli_wild_17620969	1399936799	*Ca_kabuli_Chr04*	17620969	A/T	Gene_Ca_05368	INTRON	Dihydroorotatedehydrogenase, conserved site
Kabuli_wild_17620973	1399936800	*Ca_kabuli_Chr04*	17620973	G/T	Gene_Ca_05368	INTRON	Dihydroorotatedehydrogenase, conserved site
Kabuli_wild_17621035	1399936801	*Ca_kabuli_Chr04*	17621035	G/T	Gene_Ca_05368	INTRON	Dihydroorotatedehydrogenase, conserved site
Kabuli_wild_17621609	1399936802	*Ca_kabuli_Chr04*	17621609	G/A	Gene_Ca_05368	NON_SYNONYMOUS_CODING	Dihydroorotatedehydrogenase, conserved site
Kabuli_wild_17647558	1399936803	*Ca_kabuli_Chr04*	17647558	G/A	NA	INTERGENIC	NA
Kabuli_wild_17685058	1399936804	*Ca_kabuli_Chr04*	17685058	T/G	Gene_Ca_05358	DOWNSTREAM	GRAM
Kabuli_wild_17685113	1399936805	*Ca_kabuli_Chr04*	17685113	C/A	Gene_Ca_05358	DOWNSTREAM	GRAM
Kabuli_wild_17718494	1399936806	*Ca_kabuli_Chr04*	17718494	C/T	NA	INTERGENIC	NA
Kabuli_wild_17718536	1399936807	*Ca_kabuli_Chr04*	17718536	C/A	NA	INTERGENIC	NA
Kabuli_wild_17718540	1399936808	*Ca_kabuli_Chr04*	17718540	C/T	NA	INTERGENIC	NA
Kabuli_wild_17718922	1399936809	*Ca_kabuli_Chr04*	17718922	T/G	NA	INTERGENIC	NA
Kabuli_wild_17718997	1399936810	*Ca_kabuli_Chr04*	17718997	C/A	NA	INTERGENIC	NA
Kabuli_wild_17728434	1399936811	*Ca_kabuli_Chr04*	17728434	T/A	Gene_Ca_05352	INTRON	SNF2-related
Kabuli_wild_17728462	1399936812	*Ca_kabuli_Chr04*	17728462	G/A	Gene_Ca_05352	INTRON	SNF2-related
Kabuli_wild_17729988	1399936813	*Ca_kabuli_Chr04*	17729988	A/C	Gene_Ca_05352	INTRON	SNF2-related
Kabuli_wild_17748171	1399936814	*Ca_kabuli_Chr04*	17748171	A/T	NA	INTERGENIC	NA
Kabuli_wild_17748696	1399936815	*Ca_kabuli_Chr04*	17748696	G/A	Gene_Ca_05350	DOWNSTREAM	Leucine-rich repeat
Kabuli_wild_17752013	1399936816	*Ca_kabuli_Chr04*	17752013	C/T	NA	INTERGENIC	NA
Kabuli_wild_17760671	1399936817	*Ca_kabuli_Chr04*	17760671	G/A	Gene_Ca_05349	INTRON	PeptidaseS9,prolyloligopeptidase,catalytic domain
Kabuli_wild_17760759	1399936818	*Ca_kabuli_Chr04*	17760759	G/A	Gene_Ca_05349	INTRON	PeptidaseS9,prolyloligopeptidase,catalytic domain
Kabuli_wild_17760841	1399936819	*Ca_kabuli_Chr04*	17760841	A/G	Gene_Ca_05349	INTRON	PeptidaseS9,prolyloligopeptidase,catalytic domain

SNP IDs	NCBI dbSNP accession IDs (NCBI_SS#)	Chromosomes	Physical positions (bp)	SNPs	Gene accession IDs	Sequence components of genome	Putative functions
Kabuli_wild_17760853	1399936820	*Ca_kabuli_Chr04*	17760853	T/G	Gene_Ca_05349	INTRON	PeptidaseS9,prolyloligopeptidase,catalytic domain
Kabuli_wild_17771708	1399936821	*Ca_kabuli_Chr04*	17771708	C/G	Gene_Ca_05348	UPSTREAM	NA
Kabuli_wild_17784792	1399936822	*Ca_kabuli_Chr04*	17784792	T/A	Gene_Ca_05346	INTRON	Protein of unknown function DUF639
Kabuli_wild_17826472	1399936823	*Ca_kabuli_Chr04*	17826472	G/A	NA	INTERGENIC	NA
Kabuli_wild_17826533	1399936824	*Ca_kabuli_Chr04*	17826533	A/C	NA	INTERGENIC	NA
Kabuli_wild_17826543	1399936825	*Ca_kabuli_Chr04*	17826543	T/C	NA	INTERGENIC	NA
Kabuli_wild_17826580	1399936826	*Ca_kabuli_Chr04*	17826580	C/G	NA	INTERGENIC	NA
Kabuli_wild_17826601	1399936827	*Ca_kabuli_Chr04*	17826601	T/C	NA	INTERGENIC	NA
Kabuli_wild_17840078	1399936828	*Ca_kabuli_Chr04*	17840078	G/A	Gene_Ca_18620	SYNONYMOUS_CODING	Uncharacterised protein family UPF0497,trans-membrane plant
Kabuli_wild_17847854	1399936829	*Ca_kabuli_Chr04*	17847854	T/C	Gene_Ca_18621	INTRON	Methionyl-tRNAsynthetase
Kabuli_wild_17847919	1399936830	*Ca_kabuli_Chr04*	17847919	C/T	Gene_Ca_18621	SYNONYMOUS_CODING	Methionyl-tRNAsynthetase
Kabuli_wild_17847929	1399936831	*Ca_kabuli_Chr04*	17847929	G/A	Gene_Ca_18621	SYNONYMOUS_CODING	Methionyl-tRNAsynthetase
Kabuli_wild_17856307	1399936832	*Ca_kabuli_Chr04*	17856307	T/C	Gene_Ca_18622	SYNONYMOUS_CODING	IKI3
Kabuli_wild_17856311	1399936833	*Ca_kabuli_Chr04*	17856311	T/G	Gene_Ca_18622	SYNONYMOUS_CODING	IKI3
Kabuli_wild_17856334	1399936834	*Ca_kabuli_Chr04*	17856334	A/G	Gene_Ca_18622	SYNONYMOUS_CODING	IKI3
Kabuli_wild_17857753	1399936835	*Ca_kabuli_Chr04*	17857753	C/T	Gene_Ca_18622	SYNONYMOUS_CODING	IKI3
Kabuli_wild_17858122	1399936836	*Ca_kabuli_Chr04*	17858122	T/G	Gene_Ca_18622	INTRON	IKI3
Kabuli_wild_17858133	1399936837	*Ca_kabuli_Chr04*	17858133	C/T	Gene_Ca_18622	INTRON	IKI3
Kabuli_wild_17858135	1399936838	*Ca_kabuli_Chr04*	17858135	G/A	Gene_Ca_18622	INTRON	IKI3
Kabuli_wild_17858140	1399936839	*Ca_kabuli_Chr04*	17858140	A/C	Gene_Ca_18622	INTRON	IKI3
Kabuli_wild_17858143	1399936840	*Ca_kabuli_Chr04*	17858143	A/T	Gene_Ca_18622	INTRON	IKI3
Kabuli_wild_17858144	1399936841	*Ca_kabuli_Chr04*	17858144	G/C	Gene_Ca_18622	INTRON	IKI3
Kabuli_wild_17858152	1399936842	*Ca_kabuli_Chr04*	17858152	A/T	Gene_Ca_18622	INTRON	IKI3
Kabuli_wild_17858159	1399936843	*Ca_kabuli_Chr04*	17858159	C/A	Gene_Ca_18622	INTRON	IKI3
Kabuli_wild_17858226	1399936844	*Ca_kabuli_Chr04*	17858226	C/T	Gene_Ca_18622	INTRON	IKI3
Kabuli_wild_17871763	1399936845	*Ca_kabuli_Chr04*	17871763	T/C	NA	INTERGENIC	NA
Kabuli_wild_17871804	1399936846	*Ca_kabuli_Chr04*	17871804	T/C	NA	INTERGENIC	NA
Kabuli_wild_17871828	1399936847	*Ca_kabuli_Chr04*	17871828	A/C	NA	INTERGENIC	NA
Kabuli_wild_17871915	1399936848	*Ca_kabuli_Chr04*	17871915	T/A	NA	INTERGENIC	NA
Kabuli_wild_17871919	1399936849	*Ca_kabuli_Chr04*	17871919	A/G	NA	INTERGENIC	NA
Kabuli_wild_17913682	1399936850	*Ca_kabuli_Chr04*	17913682	C/A	NA	INTERGENIC	NA
Kabuli_wild_17913698	1399936851	*Ca_kabuli_Chr04*	17913698	C/T	NA	INTERGENIC	NA
Kabuli_wild_17913719	1399936852	*Ca_kabuli_Chr04*	17913719	G/C	NA	INTERGENIC	NA
Kabuli_wild_17913726	1399936853	*Ca_kabuli_Chr04*	17913726	G/A	NA	INTERGENIC	NA
Kabuli_wild_17913731	1399936854	*Ca_kabuli_Chr04*	17913731	A/C	NA	INTERGENIC	NA
Kabuli_wild_17920613	1399936855	*Ca_kabuli_Chr04*	17920613	T/G	Gene_Ca_18632	SYNONYMOUS_CODING	ABC transporter,trans membrane domain
Kabuli_wild_17920616	1399936856	*Ca_kabuli_Chr04*	17920616	C/G	Gene_Ca_18632	SYNONYMOUS_CODING	ABC transporter,trans membrane domain
Kabuli_wild_17920619	1399936857	*Ca_kabuli_Chr04*	17920619	C/G	Gene_Ca_18632	NON_SYNONYMOUS_CODING	ABC transporter,trans membrane domain
Kabuli_wild_18013299	1399936858	*Ca_kabuli_Chr04*	18013299	G/A	NA	INTERGENIC	NA
Kabuli_wild_18020942	1399936859	*Ca_kabuli_Chr04*	18020942	A/C	NA	INTERGENIC	NA
Kabuli_wild_18031975	1399936860	*Ca_kabuli_Chr04*	18031975	T/C	Gene_Ca_18645	NON_SYNONYMOUS_CODING	NA
Kabuli_wild_18037013	1399936861	*Ca_kabuli_Chr04*	18037013	T/C	NA	INTERGENIC	NA
Kabuli_wild_18070293	1399936862	*Ca_kabuli_Chr04*	18070293	G/T	Gene_Ca_18648	NON_SYNONYMOUS_CODING	Ankyrinrepeat
Kabuli_wild_18071114	1399936863	*Ca_kabuli_Chr04*	18071114	C/A	Gene_Ca_18648	NON_SYNONYMOUS_CODING	Ankyrinrepeat

SNP IDs	NCBI dbSNP accession IDs (NCBI_SS#)	Chromosomes	Physical positions (bp)	SNPs	Gene accession IDs	Sequence components of genome	Putative functions
Kabuli_wild_18071324	1399936864	*Ca_kabuli_Chr04*	18071324	G/A	Gene_Ca_18648	NON_SYNONYMOUS_CODING	Ankyrinrepeat
Kabuli_wild_18089423	1399936865	*Ca_kabuli_Chr04*	18089423	G/C	Gene_Ca_18650	SYNONYMOUS_CODING	NA
Kabuli_wild_18117833	1399936866	*Ca_kabuli_Chr04*	18117833	T/C	NA	INTERGENIC	NA
Kabuli_wild_18223721	1399936867	*Ca_kabuli_Chr04*	18223721	A/G	Gene_Ca_18659	INTRON	SET domain
Kabuli_wild_18228846	1399936868	*Ca_kabuli_Chr04*	18228846	G/A	Gene_Ca_18659	INTRON	SET domain
Kabuli_wild_18231382	1399936869	*Ca_kabuli_Chr04*	18231382	T/C	Gene_Ca_18659	INTRON	SET domain
Kabuli_wild_18231407	1399936870	*Ca_kabuli_Chr04*	18231407	C/T	Gene_Ca_18659	INTRON	SET domain
Kabuli_wild_18231443	1399936871	*Ca_kabuli_Chr04*	18231443	T/C	Gene_Ca_18659	INTRON	SET domain
Kabuli_wild_18231463	1399936872	*Ca_kabuli_Chr04*	18231463	T/C	Gene_Ca_18659	INTRON	SET domain
Kabuli_wild_18231466	1399936873	*Ca_kabuli_Chr04*	18231466	T/C	Gene_Ca_18659	INTRON	SET domain
Kabuli_wild_18231651	1399936874	*Ca_kabuli_Chr04*	18231651	A/G	Gene_Ca_18659	INTRON	SET domain
Kabuli_wild_18241936	1399936875	*Ca_kabuli_Chr04*	18241936	T/C	Gene_Ca_18659	INTRON	SET domain
Kabuli_wild_18241995	1399936876	*Ca_kabuli_Chr04*	18241995	G/C	Gene_Ca_18659	INTRON	SET domain
Kabuli_wild_18242020	1399936877	*Ca_kabuli_Chr04*	18242020	C/G	Gene_Ca_18659	INTRON	SET domain
Kabuli_wild_18427384	1399936878	*Ca_kabuli_Chr04*	18427384	C/T	Gene_Ca_21626	INTRON	LisHdimerisationmotif
Kabuli_wild_18427385	1399936879	*Ca_kabuli_Chr04*	18427385	G/T	Gene_Ca_21626	INTRON	LisHdimerisationmotif
Kabuli_wild_18429139	1399936880	*Ca_kabuli_Chr04*	18429139	C/T	Gene_Ca_21625	DOWNSTREAM	Pre-mRNAcleavagecomplexIIClp1
Kabuli_wild_18489456	1399936881	*Ca_kabuli_Chr04*	18489456	G/C	Gene_Ca_21622	UPSTREAM	Ubi Aprenyltransferase family
Kabuli_wild_18545489	1399936882	*Ca_kabuli_Chr04*	18545489	A/T	Gene_Ca_21616	INTRON	Protein kinase,catalytic domain
Kabuli_wild_18546209	1399936883	*Ca_kabuli_Chr04*	18546209	T/G	Gene_Ca_21616	SYNONYMOUS_CODING	Protein kinase,catalytic domain
Kabuli_wild_18628582	1399936884	*Ca_kabuli_Chr04*	18628582	A/G	Gene_Ca_21604	SYNONYMOUS_CODING	Glycosyltransferase,family48
Kabuli_wild_18710941	1399936885	*Ca_kabuli_Chr04*	18710941	T/G	Gene_Ca_13978	INTRON	NA
Kabuli_wild_18719417	1399936886	*Ca_kabuli_Chr04*	18719417	A/G	Gene_Ca_13978	INTRON	NA
Kabuli_wild_18764659	1399936887	*Ca_kabuli_Chr04*	18764659	A/C	NA	INTERGENIC	NA
Kabuli_wild_18812610	1399936888	*Ca_kabuli_Chr04*	18812610	T/C	NA	INTERGENIC	NA
Kabuli_wild_18916472	1399936889	*Ca_kabuli_Chr04*	18916472	G/A	NA	INTERGENIC	NA
Kabuli_wild_18923641	1399936890	*Ca_kabuli_Chr04*	18923641	G/T	Gene_Ca_13991	INTRON	Nucleotide-sugartransporter
Kabuli_wild_19024699	1399936891	*Ca_kabuli_Chr04*	19024699	T/C	NA	INTERGENIC	NA
Kabuli_wild_19227691	1399936892	*Ca_kabuli_Chr04*	19227691	G/C	Gene_Ca_14021	NON_SYNONYMOUS_CODING	Protein kinase,catalytic domain
Kabuli_wild_19263967	1399936893	*Ca_kabuli_Chr04*	19263967	T/G	Gene_Ca_14024	INTRON	NA
Kabuli_wild_19267581	1399936894	*Ca_kabuli_Chr04*	19267581	A/G	Gene_Ca_14024	INTRON	NA
Kabuli_wild_19540632	1399936895	*Ca_kabuli_Chr04*	19540632	A/C	Gene_Ca_14045	NON_SYNONYMOUS_CODING	Spo11/DNAtopoisomeraseVI,subunitA
Kabuli_wild_19579442	1399936896	*Ca_kabuli_Chr04*	19579442	C/A	NA	INTERGENIC	NA
Kabuli_wild_19668502	1399936897	*Ca_kabuli_Chr04*	19668502	T/G	Gene_Ca_14053	SYNONYMOUS_CODING	Pentatrico peptide repeat
Kabuli_wild_19713311	1399936898	*Ca_kabuli_Chr04*	19713311	T/G	Gene_Ca_14059	SYNONYMOUS_CODING	NA
Kabuli_wild_19734934	1399936899	*Ca_kabuli_Chr04*	19734934	A/G	Gene_Ca_14061	SYNONYMOUS_CODING	Heat shock protein DnaJ,N-terminal
Kabuli_wild_19869729	1399936900	*Ca_kabuli_Chr04*	19869729	G/T	Gene_Ca_17123	NON_SYNONYMOUS_CODING	Target SNARE coiled-coil domain
Kabuli_wild_20161601	1399936901	*Ca_kabuli_Chr04*	20161601	A/G	Gene_Ca_17138	SYNONYMOUS_CODING	BTB/POZ-like
Kabuli_wild_20193257	1399936902	*Ca_kabuli_Chr04*	20193257	A/G	NA	INTERGENIC	NA
Kabuli_wild_20308992	1399936903	*Ca_kabuli_Chr04*	20308992	C/A	Gene_Ca_17144	SYNONYMOUS_CODING	Phenylalanine/histidineammonia-lyase
Kabuli_wild_20398685	1399936904	*Ca_kabuli_Chr04*	20398685	A/C	Gene_Ca_17149	NON_SYNONYMOUS_CODING	NA
Kabuli_wild_20599050	1399936905	*Ca_kabuli_Chr04*	20599050	A/G	NA	INTERGENIC	NA
Kabuli_wild_20599051	1399936906	*Ca_kabuli_Chr04*	20599051	G/A	NA	INTERGENIC	NA
Kabuli_wild_20599058	1399936907	*Ca_kabuli_Chr04*	20599058	T/C	NA	INTERGENIC	NA

SNP IDs	NCBI dbSNP accession IDs (NCBI_SS#)	Chromosomes	Physical positions (bp)	SNPs	Gene accession IDs	Sequence components of genome	Putative functions
Kabuli_wild_20599081	1399936908	*Ca_kabuli_Chr04*	20599081	C/T	NA	INTERGENIC	NA
Kabuli_wild_20599091	1399936909	*Ca_kabuli_Chr04*	20599091	T/G	NA	INTERGENIC	NA
Kabuli_wild_20656170	1399936910	*Ca_kabuli_Chr04*	20656170	T/G	Gene_Ca_17165	SYNONYMOUS_CODING	NucleoporinNup120/160
Kabuli_wild_20657494	1399936911	*Ca_kabuli_Chr04*	20657494	C/A	Gene_Ca_17165	NON_SYNONYMOUS_CODING	NucleoporinNup120/160
Kabuli_wild_20669174	1399936912	*Ca_kabuli_Chr04*	20669174	T/C	Gene_Ca_17165	INTRON	NucleoporinNup120/160
Kabuli_wild_20669442	1399936913	*Ca_kabuli_Chr04*	20669442	G/A	Gene_Ca_17165	INTRON	NucleoporinNup120/160
Kabuli_wild_20696952	1399936914	*Ca_kabuli_Chr04*	20696952	G/T	Gene_Ca_17166	SYNONYMOUS_CODING	ABC transporter,trans membrane domain
Kabuli_wild_20774180	1399936915	*Ca_kabuli_Chr04*	20774180	C/T	Gene_Ca_17170	INTRON	NA
Kabuli_wild_20774211	1399936916	*Ca_kabuli_Chr04*	20774211	C/T	Gene_Ca_17170	INTRON	NA
Kabuli_wild_20774238	1399936917	*Ca_kabuli_Chr04*	20774238	G/T	Gene_Ca_17170	INTRON	NA
Kabuli_wild_20774255	1399936918	*Ca_kabuli_Chr04*	20774255	C/A	Gene_Ca_17170	INTRON	NA
Kabuli_wild_20905036	1399936919	*Ca_kabuli_Chr04*	20905036	T/A	Gene_Ca_19045	INTRON	Serine/threoninedehydratase,pyridoxal-phosphate-bindingsite
Kabuli_wild_20905105	1399936920	*Ca_kabuli_Chr04*	20905105	G/A	Gene_Ca_19045	INTRON	Serine/threoninedehydratase,pyridoxal-phosphate-bindingsite
Kabuli_wild_20943719	1399936921	*Ca_kabuli_Chr04*	20943719	T/C	Gene_Ca_19046	NON_SYNONYMOUS_CODING	NA
Kabuli_wild_21385308	1399936922	*Ca_kabuli_Chr04*	21385308	A/T	Gene_Ca_19066	SYNONYMOUS_CODING	PeptidaseA1
Kabuli_wild_21687162	1399936923	*Ca_kabuli_Chr04*	21687162	T/C	Gene_Ca_19074	SYNONYMOUS_CODING	PeptidaseA1
Kabuli_wild_21835446	1399936924	*Ca_kabuli_Chr04*	21835446	T/A	NA	INTERGENIC	NA
Kabuli_wild_21835448	1399936925	*Ca_kabuli_Chr04*	21835448	G/A	NA	INTERGENIC	NA
Kabuli_wild_21835453	1399936926	*Ca_kabuli_Chr04*	21835453	C/A	NA	INTERGENIC	NA
Kabuli_wild_21835454	1399936927	*Ca_kabuli_Chr04*	21835454	C/A	NA	INTERGENIC	NA
Kabuli_wild_21835457	1399936928	*Ca_kabuli_Chr04*	21835457	T/A	NA	INTERGENIC	NA
Kabuli_wild_21835459	1399936929	*Ca_kabuli_Chr04*	21835459	T/A	NA	INTERGENIC	NA
Kabuli_wild_21931523	1399936930	*Ca_kabuli_Chr04*	21931523	T/C	NA	INTERGENIC	NA
Kabuli_wild_21931541	1399936931	*Ca_kabuli_Chr04*	21931541	C/T	NA	INTERGENIC	NA
Kabuli_wild_21931558	1399936932	*Ca_kabuli_Chr04*	21931558	G/A	NA	INTERGENIC	NA
Kabuli_wild_21931559	1399936933	*Ca_kabuli_Chr04*	21931559	G/A	NA	INTERGENIC	NA
Kabuli_wild_22081566	1399936934	*Ca_kabuli_Chr04*	22081566	C/T	Gene_Ca_20137	NON_SYNONYMOUS_CODING	HhH-GPDdomain
Kabuli_wild_22124841	1399936935	*Ca_kabuli_Chr04*	22124841	T/C	Gene_Ca_20135	NON_SYNONYMOUS_CODING	NA
Kabuli_wild_22266662	1399936936	*Ca_kabuli_Chr04*	22266662	C/T	NA	INTERGENIC	NA
Kabuli_wild_22293495	1399936937	*Ca_kabuli_Chr04*	22293495	T/C	NA	INTERGENIC	NA
Kabuli_wild_22375357	1399936938	*Ca_kabuli_Chr04*	22375357	T/G	Gene_Ca_20122	INTRON	Ribosomal protein 60S
Kabuli_wild_22384143	1399936939	*Ca_kabuli_Chr04*	22384143	G/A	Gene_Ca_20121	SYNONYMOUS_CODING	NA
Kabuli_wild_22725220	1399936940	*Ca_kabuli_Chr04*	22725220	C/T	Gene_Ca_14476	DOWNSTREAM	Protein of unknown function DUF593
Kabuli_wild_22725533	1399936941	*Ca_kabuli_Chr04*	22725533	T/C	NA	INTERGENIC	NA
Kabuli_wild_22850596	1399936942	*Ca_kabuli_Chr04*	22850596	C/G	Gene_Ca_14467	NON_SYNONYMOUS_CODING	NA
Kabuli_wild_22859422	1399936943	*Ca_kabuli_Chr04*	22859422	G/A	NA	INTERGENIC	NA
Kabuli_wild_22860281	1399936944	*Ca_kabuli_Chr04*	22860281	A/C	NA	INTERGENIC	NA
Kabuli_wild_22860656	1399936945	*Ca_kabuli_Chr04*	22860656	C/A	NA	INTERGENIC	NA
Kabuli_wild_22865389	1399936946	*Ca_kabuli_Chr04*	22865389	C/T	NA	INTERGENIC	NA
Kabuli_wild_22865390	1399936947	*Ca_kabuli_Chr04*	22865390	G/A	NA	INTERGENIC	NA
Kabuli_wild_22865436	1399936948	*Ca_kabuli_Chr04*	22865436	A/T	NA	INTERGENIC	NA
Kabuli_wild_22904152	1399936949	*Ca_kabuli_Chr04*	22904152	G/T	NA	INTERGENIC	NA
Kabuli_wild_22904166	1399936950	*Ca_kabuli_Chr04*	22904166	T/G	NA	INTERGENIC	NA
Kabuli_wild_22908360	1399936951	*Ca_kabuli_Chr04*	22908360	A/G	Gene_Ca_14463	SYNONYMOUS_CODING	Pentatrico peptide repeat

SNP IDs	NCBI dbSNP accession IDs (NCBI_SS#)	Chromosomes	Physical positions (bp)	SNPs	Gene accession IDs	Sequence components of genome	Putative functions
Kabuli_wild_22971608	1399936952	*Ca_kabuli_Chr04*	22971608	T/A	Gene_Ca_14460	NON_SYNONYMOUS_CODING	NA
Kabuli_wild_22971614	1399936953	*Ca_kabuli_Chr04*	22971614	C/T	Gene_Ca_14460	SYNONYMOUS_CODING	NA
Kabuli_wild_22971623	1399936954	*Ca_kabuli_Chr04*	22971623	C/T	Gene_Ca_14460	SYNONYMOUS_CODING	NA
Kabuli_wild_22971660	1399936955	*Ca_kabuli_Chr04*	22971660	G/A	Gene_Ca_14460	NON_SYNONYMOUS_CODING	NA
Kabuli_wild_22971744	1399936956	*Ca_kabuli_Chr04*	22971744	A/C	Gene_Ca_14460	NON_SYNONYMOUS_CODING	NA
Kabuli_wild_22971746	1399936957	*Ca_kabuli_Chr04*	22971746	G/A	Gene_Ca_14460	SYNONYMOUS_CODING	NA
Kabuli_wild_22971766	1399936958	*Ca_kabuli_Chr04*	22971766	A/G	Gene_Ca_14460	NON_SYNONYMOUS_CODING	NA
Kabuli_wild_22971847	1399936959	*Ca_kabuli_Chr04*	22971847	C/A	Gene_Ca_14460	NON_SYNONYMOUS_CODING	NA
Kabuli_wild_22971853	1399936960	*Ca_kabuli_Chr04*	22971853	C/A	Gene_Ca_14460	NON_SYNONYMOUS_CODING	NA
Kabuli_wild_22971884	1399936961	*Ca_kabuli_Chr04*	22971884	G/A	Gene_Ca_14460	SYNONYMOUS_CODING	NA
Kabuli_wild_23314665	1399936962	*Ca_kabuli_Chr04*	23314665	A/C	NA	INTERGENIC	NA
Kabuli_wild_23337312	1399936963	*Ca_kabuli_Chr04*	23337312	T/G	NA	INTERGENIC	NA
Kabuli_wild_23361115	1399936964	*Ca_kabuli_Chr04*	23361115	G/T	NA	INTERGENIC	NA
Kabuli_wild_23362978	1399936965	*Ca_kabuli_Chr04*	23362978	G/A	Gene_Ca_14445	SYNONYMOUS_CODING	Exodeoxyribonuclease IIIxth
Kabuli_wild_23481919	1399936966	*Ca_kabuli_Chr04*	23481919	G/A	NA	INTERGENIC	NA
Kabuli_wild_23481927	1399936967	*Ca_kabuli_Chr04*	23481927	G/T	NA	INTERGENIC	NA
Kabuli_wild_23481962	1399936968	*Ca_kabuli_Chr04*	23481962	G/A	NA	INTERGENIC	NA
Kabuli_wild_23481990	1399936969	*Ca_kabuli_Chr04*	23481990	A/C	NA	INTERGENIC	NA
Kabuli_wild_23481998	1399936970	*Ca_kabuli_Chr04*	23481998	T/C	NA	INTERGENIC	NA
Kabuli_wild_23482033	1399936971	*Ca_kabuli_Chr04*	23482033	A/G	NA	INTERGENIC	NA
Kabuli_wild_23482042	1399936972	*Ca_kabuli_Chr04*	23482042	G/A	NA	INTERGENIC	NA
Kabuli_wild_23482051	1399936973	*Ca_kabuli_Chr04*	23482051	G/A	NA	INTERGENIC	NA
Kabuli_wild_23486812	1399936974	*Ca_kabuli_Chr04*	23486812	A/G	NA	INTERGENIC	NA
Kabuli_wild_23486824	1399936975	*Ca_kabuli_Chr04*	23486824	T/C	NA	INTERGENIC	NA
Kabuli_wild_23486859	1399936976	*Ca_kabuli_Chr04*	23486859	A/G	NA	INTERGENIC	NA
Kabuli_wild_23486868	1399936977	*Ca_kabuli_Chr04*	23486868	G/A	NA	INTERGENIC	NA
Kabuli_wild_23486877	1399936978	*Ca_kabuli_Chr04*	23486877	G/A	NA	INTERGENIC	NA
Kabuli_wild_23663942	1399936979	*Ca_kabuli_Chr04*	23663942	C/T	NA	INTERGENIC	NA
Kabuli_wild_23718271	1399936980	*Ca_kabuli_Chr04*	23718271	C/T	NA	INTERGENIC	NA
Kabuli_wild_23812574	1399936981	*Ca_kabuli_Chr04*	23812574	T/G	Gene_Ca_14420	NON_SYNONYMOUS_CODING	NA
Kabuli_wild_24013394	1399936982	*Ca_kabuli_Chr04*	24013394	A/C	Gene_Ca_14412	UPSTREAM	Aminotransferase-like,plant mobile domain
Kabuli_wild_24120866	1399936983	*Ca_kabuli_Chr04*	24120866	A/G	NA	INTERGENIC	NA
Kabuli_wild_24179449	1399936984	*Ca_kabuli_Chr04*	24179449	T/G	Gene_Ca_14403	DOWNSTREAM	NA
Kabuli_wild_24261779	1399936985	*Ca_kabuli_Chr04*	24261779	C/A	NA	INTERGENIC	NA
Kabuli_wild_24587085	1399936986	*Ca_kabuli_Chr04*	24587085	A/G	NA	INTERGENIC	NA
Kabuli_wild_24587112	1399936987	*Ca_kabuli_Chr04*	24587112	C/T	NA	INTERGENIC	NA
Kabuli_wild_24614582	1399936988	*Ca_kabuli_Chr04*	24614582	G/T	NA	INTERGENIC	NA
Kabuli_wild_24729302	1399936989	*Ca_kabuli_Chr04*	24729302	T/C	Gene_Ca_20853	INTRON	Protein kinase,catalytic domain
Kabuli_wild_24732990	1399936990	*Ca_kabuli_Chr04*	24732990	T/C	Gene_Ca_20853	INTRON	Protein kinase,catalytic domain
Kabuli_wild_24732991	1399936991	*Ca_kabuli_Chr04*	24732991	G/T	Gene_Ca_20853	INTRON	Protein kinase,catalytic domain
Kabuli_wild_24771737	1399936992	*Ca_kabuli_Chr04*	24771737	T/A	Gene_Ca_20851	NON_SYNONYMOUS_CODING	NA
Kabuli_wild_24782714	1399936993	*Ca_kabuli_Chr04*	24782714	C/A	Gene_Ca_20850	NON_SYNONYMOUS_CODING	NA
Kabuli_wild_24782715	1399936994	*Ca_kabuli_Chr04*	24782715	A/G	Gene_Ca_20850	NON_SYNONYMOUS_CODING	NA
Kabuli_wild_24856115	1399936995	*Ca_kabuli_Chr04*	24856115	A/C	Gene_Ca_20847	NON_SYNONYMOUS_CODING	Protein kinase,catalytic domain

SNP IDs	NCBI dbSNP accession IDs (NCBI_SS#)	Chromosomes	Physical positions (bp)	SNPs	Gene accession IDs	Sequence components of genome	Putative functions
Kabuli_wild_24857501	1399936996	*Ca_kabuli_Chr04*	24857501	A/G	Gene_Ca_20847	INTRON	Protein kinase,catalytic domain
Kabuli_wild_24861477	1399936997	*Ca_kabuli_Chr04*	24861477	T/G	Gene_Ca_20847	INTRON	Protein kinase,catalytic domain
Kabuli_wild_24861500	1399936998	*Ca_kabuli_Chr04*	24861500	A/C	Gene_Ca_20847	INTRON	Protein kinase,catalytic domain
Kabuli_wild_24979942	1399936999	*Ca_kabuli_Chr04*	24979942	A/T	Gene_Ca_20842	DOWNSTREAM	Lipase,GDSL
Kabuli_wild_25318255	1399937000	*Ca_kabuli_Chr04*	25318255	T/C	NA	INTERGENIC	NA
Kabuli_wild_25401714	1399937001	*Ca_kabuli_Chr04*	25401714	T/C	NA	INTERGENIC	NA
Kabuli_wild_25446684	1399937002	*Ca_kabuli_Chr04*	25446684	T/C	NA	INTERGENIC	NA
Kabuli_wild_25455156	1399937003	*Ca_kabuli_Chr04*	25455156	G/A	NA	INTERGENIC	NA
Kabuli_wild_25455204	1399937004	*Ca_kabuli_Chr04*	25455204	A/G	NA	INTERGENIC	NA
Kabuli_wild_25455208	1399937005	*Ca_kabuli_Chr04*	25455208	C/A	NA	INTERGENIC	NA
Kabuli_wild_25463680	1399937006	*Ca_kabuli_Chr04*	25463680	A/C	NA	INTERGENIC	NA
Kabuli_wild_25592484	1399937007	*Ca_kabuli_Chr04*	25592484	A/C	NA	INTERGENIC	NA
Kabuli_wild_25592487	1399937008	*Ca_kabuli_Chr04*	25592487	A/G	NA	INTERGENIC	NA
Kabuli_wild_25729905	1399937009	*Ca_kabuli_Chr04*	25729905	C/A	NA	INTERGENIC	NA
Kabuli_wild_25729977	1399937010	*Ca_kabuli_Chr04*	25729977	C/T	NA	INTERGENIC	NA
Kabuli_wild_25747256	1399937011	*Ca_kabuli_Chr04*	25747256	T/C	NA	INTERGENIC	NA
Kabuli_wild_25747262	1399937012	*Ca_kabuli_Chr04*	25747262	G/A	NA	INTERGENIC	NA
Kabuli_wild_25747274	1399937013	*Ca_kabuli_Chr04*	25747274	T/C	NA	INTERGENIC	NA
Kabuli_wild_25747275	1399937014	*Ca_kabuli_Chr04*	25747275	G/A	NA	INTERGENIC	NA
Kabuli_wild_25747281	1399937015	*Ca_kabuli_Chr04*	25747281	T/C	NA	INTERGENIC	NA
Kabuli_wild_25747295	1399937016	*Ca_kabuli_Chr04*	25747295	G/A	NA	INTERGENIC	NA
Kabuli_wild_25747296	1399937017	*Ca_kabuli_Chr04*	25747296	T/C	NA	INTERGENIC	NA
Kabuli_wild_25747301	1399937018	*Ca_kabuli_Chr04*	25747301	C/G	NA	INTERGENIC	NA
Kabuli_wild_25747313	1399937019	*Ca_kabuli_Chr04*	25747313	T/G	NA	INTERGENIC	NA
Kabuli_wild_25747353	1399937020	*Ca_kabuli_Chr04*	25747353	A/C	NA	INTERGENIC	NA
Kabuli_wild_25809665	1399937021	*Ca_kabuli_Chr04*	25809665	T/C	Gene_Ca_16599	INTRON	Aminoglycoside phosphotransferase
Kabuli_wild_25810557	1399937022	*Ca_kabuli_Chr04*	25810557	T/C	Gene_Ca_16599	INTRON	Aminoglycoside phosphotransferase
Kabuli_wild_25840345	1399937023	*Ca_kabuli_Chr04*	25840345	T/A	Gene_Ca_16601	INTRON	Ankyrinrepeat
Kabuli_wild_25855756	1399937024	*Ca_kabuli_Chr04*	25855756	C/A	NA	INTERGENIC	NA
Kabuli_wild_25873290	1399937025	*Ca_kabuli_Chr04*	25873290	A/G	Gene_Ca_16604	INTRON	Zinc finger, RING-type
Kabuli_wild_25921165	1399937026	*Ca_kabuli_Chr04*	25921165	T/C	Gene_Ca_16606	INTRON	Thiolase
Kabuli_wild_26000444	1399937027	*Ca_kabuli_Chr04*	26000444	C/T	NA	INTERGENIC	NA
Kabuli_wild_26000454	1399937028	*Ca_kabuli_Chr04*	26000454	C/T	NA	INTERGENIC	NA
Kabuli_wild_26000483	1399937029	*Ca_kabuli_Chr04*	26000483	T/A	NA	INTERGENIC	NA
Kabuli_wild_26000532	1399937030	*Ca_kabuli_Chr04*	26000532	C/T	NA	INTERGENIC	NA
Kabuli_wild_26002991	1399937031	*Ca_kabuli_Chr04*	26002991	C/A	Gene_Ca_16610	INTRON	Aldo/ketoreductase
Kabuli_wild_26008744	1399937032	*Ca_kabuli_Chr04*	26008744	G/A	NA	INTERGENIC	NA
Kabuli_wild_26008745	1399937033	*Ca_kabuli_Chr04*	26008745	G/A	NA	INTERGENIC	NA
Kabuli_wild_26008748	1399937034	*Ca_kabuli_Chr04*	26008748	C/T	NA	INTERGENIC	NA
Kabuli_wild_26008768	1399937035	*Ca_kabuli_Chr04*	26008768	A/G	NA	INTERGENIC	NA
Kabuli_wild_26008772	1399937036	*Ca_kabuli_Chr04*	26008772	C/T	NA	INTERGENIC	NA
Kabuli_wild_26008816	1399937037	*Ca_kabuli_Chr04*	26008816	A/C	NA	INTERGENIC	NA
Kabuli_wild_26008835	1399937038	*Ca_kabuli_Chr04*	26008835	C/T	NA	INTERGENIC	NA
Kabuli_wild_26009438	1399937039	*Ca_kabuli_Chr04*	26009438	G/T	NA	INTERGENIC	NA

SNP IDs	NCBI dbSNP accession IDs (NCBI_SS#)	Chromosomes	Physical positions (bp)	SNPs	Gene accession IDs	Sequence components of genome	Putative functions
Kabuli_wild_26009439	1399937040	*Ca_kabuli_Chr04*	26009439	G/T	NA	INTERGENIC	NA
Kabuli_wild_26028734	1399937041	*Ca_kabuli_Chr04*	26028734	G/A	NA	INTERGENIC	NA
Kabuli_wild_26028738	1399937042	*Ca_kabuli_Chr04*	26028738	T/G	NA	INTERGENIC	NA
Kabuli_wild_26028770	1399937043	*Ca_kabuli_Chr04*	26028770	C/T	NA	INTERGENIC	NA
Kabuli_wild_26089832	1399937044	*Ca_kabuli_Chr04*	26089832	C/T	NA	INTERGENIC	NA
Kabuli_wild_26221888	1399937045	*Ca_kabuli_Chr04*	26221888	T/C	NA	INTERGENIC	NA
Kabuli_wild_26222180	1399937046	*Ca_kabuli_Chr04*	26222180	A/G	NA	INTERGENIC	NA
Kabuli_wild_26242006	1399937047	*Ca_kabuli_Chr04*	26242006	A/G	Gene_Ca_16616	SYNONYMOUS_CODING	NA
Kabuli_wild_26358597	1399937048	*Ca_kabuli_Chr04*	26358597	C/T	NA	INTERGENIC	NA
Kabuli_wild_26470555	1399937049	*Ca_kabuli_Chr04*	26470555	G/A	NA	INTERGENIC	NA
Kabuli_wild_26511506	1399937050	*Ca_kabuli_Chr04*	26511506	A/T	NA	INTERGENIC	NA
Kabuli_wild_26511507	1399937051	*Ca_kabuli_Chr04*	26511507	G/C	NA	INTERGENIC	NA
Kabuli_wild_26511513	1399937052	*Ca_kabuli_Chr04*	26511513	G/C	NA	INTERGENIC	NA
Kabuli_wild_26511514	1399937053	*Ca_kabuli_Chr04*	26511514	G/A	NA	INTERGENIC	NA
Kabuli_wild_26511520	1399937054	*Ca_kabuli_Chr04*	26511520	G/A	NA	INTERGENIC	NA
Kabuli_wild_26511526	1399937055	*Ca_kabuli_Chr04*	26511526	G/A	NA	INTERGENIC	NA
Kabuli_wild_26511530	1399937056	*Ca_kabuli_Chr04*	26511530	A/T	NA	INTERGENIC	NA
Kabuli_wild_26511538	1399937057	*Ca_kabuli_Chr04*	26511538	G/A	NA	INTERGENIC	NA
Kabuli_wild_26511551	1399937058	*Ca_kabuli_Chr04*	26511551	G/A	NA	INTERGENIC	NA
Kabuli_wild_26511563	1399937059	*Ca_kabuli_Chr04*	26511563	G/A	NA	INTERGENIC	NA
Kabuli_wild_26511571	1399937060	*Ca_kabuli_Chr04*	26511571	A/G	NA	INTERGENIC	NA
Kabuli_wild_26511577	1399937061	*Ca_kabuli_Chr04*	26511577	A/T	NA	INTERGENIC	NA
Kabuli_wild_26529606	1399937062	*Ca_kabuli_Chr04*	26529606	C/T	Gene_Ca_25003	INTRON	RNA recognition motif domain
Kabuli_wild_26532763	1399937063	*Ca_kabuli_Chr04*	26532763	T/G	Gene_Ca_25003	INTRON	RNA recognition motif domain
Kabuli_wild_26537053	1399937064	*Ca_kabuli_Chr04*	26537053	C/T	Gene_Ca_25003	DOWNSTREAM	RNA recognition motif domain
Kabuli_wild_26537258	1399937065	*Ca_kabuli_Chr04*	26537258	A/C	Gene_Ca_25003	DOWNSTREAM	RNA recognition motif domain
Kabuli_wild_26544731	1399937066	*Ca_kabuli_Chr04*	26544731	T/G	Gene_Ca_25002	NON_SYNONYMOUS_CODING	C2 calcium-dependent membrane targeting
Kabuli_wild_26815871	1399937067	*Ca_kabuli_Chr04*	26815871	A/T	NA	INTERGENIC	NA
Kabuli_wild_26834570	1399937068	*Ca_kabuli_Chr04*	26834570	C/A	Gene_Ca_20439	NON_SYNONYMOUS_CODING	Ammoniumtransporter
Kabuli_wild_26915621	1399937069	*Ca_kabuli_Chr04*	26915621	G/A	NA	INTERGENIC	NA
Kabuli_wild_26915626	1399937070	*Ca_kabuli_Chr04*	26915626	C/T	NA	INTERGENIC	NA
Kabuli_wild_26915638	1399937071	*Ca_kabuli_Chr04*	26915638	G/A	NA	INTERGENIC	NA
Kabuli_wild_26989204	1399937072	*Ca_kabuli_Chr04*	26989204	A/C	Gene_Ca_20453	NON_SYNONYMOUS_CODING	RibosomalproteinS3,C-terminal
Kabuli_wild_27078914	1399937073	*Ca_kabuli_Chr04*	27078914	C/A	NA	INTERGENIC	NA
Kabuli_wild_27078987	1399937074	*Ca_kabuli_Chr04*	27078987	A/G	NA	INTERGENIC	NA
Kabuli_wild_27095371	1399937075	*Ca_kabuli_Chr04*	27095371	A/G	NA	INTERGENIC	NA
Kabuli_wild_27095401	1399937076	*Ca_kabuli_Chr04*	27095401	A/T	NA	INTERGENIC	NA
Kabuli_wild_27102817	1399937077	*Ca_kabuli_Chr04*	27102817	A/C	NA	INTERGENIC	NA
Kabuli_wild_27102833	1399937078	*Ca_kabuli_Chr04*	27102833	G/T	NA	INTERGENIC	NA
Kabuli_wild_27228499	1399937079	*Ca_kabuli_Chr04*	27228499	T/C	Gene_Ca_20460	NON_SYNONYMOUS_CODING	Protein of unknown function DUF3527
Kabuli_wild_27262893	1399937080	*Ca_kabuli_Chr04*	27262893	G/A	NA	INTERGENIC	NA
Kabuli_wild_27268238	1399937081	*Ca_kabuli_Chr04*	27268238	T/A	NA	INTERGENIC	NA
Kabuli_wild_27268301	1399937082	*Ca_kabuli_Chr04*	27268301	G/A	Gene_Ca_20462	UPSTREAM	General substrate transporter
Kabuli_wild_27274063	1399937083	*Ca_kabuli_Chr04*	27274063	G/A	NA	INTERGENIC	NA

SNP IDs	NCBI dbSNP accession IDs (NCBI_SS#)	Chromosomes	Physical positions (bp)	SNPs	Gene accession IDs	Sequence components of genome	Putative functions
Kabuli_wild_27388419	1399937084	*Ca_kabuli_Chr04*	27388419	A/G	NA	INTERGENIC	NA
Kabuli_wild_27388431	1399937085	*Ca_kabuli_Chr04*	27388431	T/C	NA	INTERGENIC	NA
Kabuli_wild_27493010	1399937086	*Ca_kabuli_Chr04*	27493010	G/T	Gene_Ca_21317	SYNONYMOUS_CODING	Protein of unknown function DUF716,TMEM45
Kabuli_wild_27613432	1399937087	*Ca_kabuli_Chr04*	27613432	G/A	Gene_Ca_21314	INTRON	Peptidase C48, SUMO/Sentrin/Ubl1
Kabuli_wild_27613450	1399937088	*Ca_kabuli_Chr04*	27613450	C/T	Gene_Ca_21314	INTRON	Peptidase C48, SUMO/Sentrin/Ubl1
Kabuli_wild_27613452	1399937089	*Ca_kabuli_Chr04*	27613452	G/A	Gene_Ca_21314	INTRON	Peptidase C48, SUMO/Sentrin/Ubl1
Kabuli_wild_27617803	1399937090	*Ca_kabuli_Chr04*	27617803	A/G	NA	INTERGENIC	NA
Kabuli_wild_27625368	1399937091	*Ca_kabuli_Chr04*	27625368	T/A	Gene_Ca_21312	INTRON	Protein of unknown function DUF810
Kabuli_wild_27626559	1399937092	*Ca_kabuli_Chr04*	27626559	G/A	Gene_Ca_21312	SYNONYMOUS_CODING	Protein of unknown function DUF810
Kabuli_wild_27626999	1399937093	*Ca_kabuli_Chr04*	27626999	C/T	Gene_Ca_21312	SYNONYMOUS_CODING	Protein of unknown function DUF810
Kabuli_wild_27661241	1399937094	*Ca_kabuli_Chr04*	27661241	T/G	Gene_Ca_21309	SYNONYMOUS_CODING	NA
Kabuli_wild_27661326	1399937095	*Ca_kabuli_Chr04*	27661326	A/G	Gene_Ca_21309	NON_SYNONYMOUS_CODING	NA
Kabuli_wild_27669829	1399937096	*Ca_kabuli_Chr04*	27669829	C/G	NA	INTERGENIC	NA
Kabuli_wild_27777968	1399937097	*Ca_kabuli_Chr04*	27777968	A/C	Gene_Ca_21303	INTRON	MATH
Kabuli_wild_27786162	1399937098	*Ca_kabuli_Chr04*	27786162	G/C	Gene_Ca_21303	INTRON	MATH
Kabuli_wild_27786356	1399937099	*Ca_kabuli_Chr04*	27786356	G/C	Gene_Ca_21303	INTRON	MATH
Kabuli_wild_27970989	1399937100	*Ca_kabuli_Chr04*	27970989	T/G	Gene_Ca_21299	SYNONYMOUS_CODING	GAF
Kabuli_wild_27971211	1399937101	*Ca_kabuli_Chr04*	27971211	A/T	Gene_Ca_21299	NON_SYNONYMOUS_CODING	GAF
Kabuli_wild_28061002	1399937102	*Ca_kabuli_Chr04*	28061002	A/C	Gene_Ca_23178	SYNONYMOUS_CODING	NA
Kabuli_wild_28092609	1399937103	*Ca_kabuli_Chr04*	28092609	G/A	Gene_Ca_23175	NON_SYNONYMOUS_CODING	Crotonase,core
Kabuli_wild_28092724	1399937104	*Ca_kabuli_Chr04*	28092724	T/A	Gene_Ca_23175	NON_SYNONYMOUS_CODING	Crotonase,core
Kabuli_wild_28092725	1399937105	*Ca_kabuli_Chr04*	28092725	T/G	Gene_Ca_23175	NON_SYNONYMOUS_CODING	Crotonase,core
Kabuli_wild_28092727	1399937106	*Ca_kabuli_Chr04*	28092727	T/A	Gene_Ca_23175	NON_SYNONYMOUS_CODING	Crotonase,core
Kabuli_wild_28246738	1399937107	*Ca_kabuli_Chr04*	28246738	T/C	NA	INTERGENIC	NA
Kabuli_wild_28246744	1399937108	*Ca_kabuli_Chr04*	28246744	C/T	NA	INTERGENIC	NA
Kabuli_wild_28246755	1399937109	*Ca_kabuli_Chr04*	28246755	C/T	NA	INTERGENIC	NA
Kabuli_wild_28289900	1399937110	*Ca_kabuli_Chr04*	28289900	C/A	NA	INTERGENIC	NA
Kabuli_wild_28299238	1399937111	*Ca_kabuli_Chr04*	28299238	G/A	Gene_Ca_23164	DOWNSTREAM	Cryptochrome/DNAphotolyase,class1
Kabuli_wild_28300544	1399937112	*Ca_kabuli_Chr04*	28300544	C/A	Gene_Ca_23165	DOWNSTREAM	General substrate transporter
Kabuli_wild_28301853	1399937113	*Ca_kabuli_Chr04*	28301853	T/C	Gene_Ca_23165	DOWNSTREAM	General substrate transporter
Kabuli_wild_28376900	1399937114	*Ca_kabuli_Chr04*	28376900	T/C	Gene_Ca_23162	SYNONYMOUS_CODING	Lactate/malatedehydrogenase,N-terminal
Kabuli_wild_28423079	1399937115	*Ca_kabuli_Chr04*	28423079	T/C	NA	INTERGENIC	NA
Kabuli_wild_28423140	1399937116	*Ca_kabuli_Chr04*	28423140	A/G	NA	INTERGENIC	NA
Kabuli_wild_28423188	1399937117	*Ca_kabuli_Chr04*	28423188	A/G	NA	INTERGENIC	NA
Kabuli_wild_28431706	1399937118	*Ca_kabuli_Chr04*	28431706	G/A	NA	INTERGENIC	NA
Kabuli_wild_28516385	1399937119	*Ca_kabuli_Chr04*	28516385	T/G	Gene_Ca_26231	SYNONYMOUS_CODING	Protein of unknown function DUF668
Kabuli_wild_28516428	1399937120	*Ca_kabuli_Chr04*	28516428	T/G	Gene_Ca_26231	NON_SYNONYMOUS_CODING	Protein of unknown function DUF668
Kabuli_wild_28570657	1399937121	*Ca_kabuli_Chr04*	28570657	C/T	NA	INTERGENIC	NA
Kabuli_wild_28669453	1399937122	*Ca_kabuli_Chr04*	28669453	C/T	NA	INTERGENIC	NA
Kabuli_wild_28669477	1399937123	*Ca_kabuli_Chr04*	28669477	A/C	NA	INTERGENIC	NA
Kabuli_wild_28669515	1399937124	*Ca_kabuli_Chr04*	28669515	T/G	NA	INTERGENIC	NA
Kabuli_wild_28790085	1399937125	*Ca_kabuli_Chr04*	28790085	A/G	NA	INTERGENIC	NA
Kabuli_wild_28891107	1399937126	*Ca_kabuli_Chr04*	28891107	G/A	NA	INTERGENIC	NA
Kabuli_wild_28911272	1399937127	*Ca_kabuli_Chr04*	28911272	T/A	NA	INTERGENIC	NA

SNP IDs	NCBI dbSNP accession IDs (NCBI_SS#)	Chromosomes	Physical positions (bp)	SNPs	Gene accession IDs	Sequence components of genome	Putative functions
Kabuli_wild_28911274	1399937128	*Ca_kabuli_Chr04*	28911274	G/A	NA	INTERGENIC	NA
Kabuli_wild_29582219	1399937129	*Ca_kabuli_Chr04*	29582219	A/C	NA	INTERGENIC	NA
Kabuli_wild_29582227	1399937130	*Ca_kabuli_Chr04*	29582227	G/A	NA	INTERGENIC	NA
Kabuli_wild_29582228	1399937131	*Ca_kabuli_Chr04*	29582228	G/A	NA	INTERGENIC	NA
Kabuli_wild_29582232	1399937132	*Ca_kabuli_Chr04*	29582232	C/T	NA	INTERGENIC	NA
Kabuli_wild_29582235	1399937133	*Ca_kabuli_Chr04*	29582235	T/A	NA	INTERGENIC	NA
Kabuli_wild_29582249	1399937134	*Ca_kabuli_Chr04*	29582249	C/T	NA	INTERGENIC	NA
Kabuli_wild_29582252	1399937135	*Ca_kabuli_Chr04*	29582252	G/A	NA	INTERGENIC	NA
Kabuli_wild_29582255	1399937136	*Ca_kabuli_Chr04*	29582255	C/T	NA	INTERGENIC	NA
Kabuli_wild_29582257	1399937137	*Ca_kabuli_Chr04*	29582257	G/A	NA	INTERGENIC	NA
Kabuli_wild_29582268	1399937138	*Ca_kabuli_Chr04*	29582268	C/G	NA	INTERGENIC	NA
Kabuli_wild_29582271	1399937139	*Ca_kabuli_Chr04*	29582271	C/T	NA	INTERGENIC	NA
Kabuli_wild_29582273	1399937140	*Ca_kabuli_Chr04*	29582273	G/A	NA	INTERGENIC	NA
Kabuli_wild_29582282	1399937141	*Ca_kabuli_Chr04*	29582282	C/A	NA	INTERGENIC	NA
Kabuli_wild_29582285	1399937142	*Ca_kabuli_Chr04*	29582285	C/A	NA	INTERGENIC	NA
Kabuli_wild_29582286	1399937143	*Ca_kabuli_Chr04*	29582286	T/A	NA	INTERGENIC	NA
Kabuli_wild_29582289	1399937144	*Ca_kabuli_Chr04*	29582289	G/A	NA	INTERGENIC	NA
Kabuli_wild_29582294	1399937145	*Ca_kabuli_Chr04*	29582294	G/A	NA	INTERGENIC	NA
Kabuli_wild_29582296	1399937146	*Ca_kabuli_Chr04*	29582296	C/T	NA	INTERGENIC	NA
Kabuli_wild_29582301	1399937147	*Ca_kabuli_Chr04*	29582301	G/T	NA	INTERGENIC	NA
Kabuli_wild_29671563	1399937148	*Ca_kabuli_Chr04*	29671563	G/A	NA	INTERGENIC	NA
Kabuli_wild_29671606	1399937149	*Ca_kabuli_Chr04*	29671606	G/A	NA	INTERGENIC	NA
Kabuli_wild_29683718	1399937150	*Ca_kabuli_Chr04*	29683718	A/T	Gene_Ca_14166	NON_SYNONYMOUS_CODING	Kinesin,motordomain
Kabuli_wild_29855493	1399937151	*Ca_kabuli_Chr04*	29855493	T/G	NA	INTERGENIC	NA
Kabuli_wild_29923380	1399937152	*Ca_kabuli_Chr04*	29923380	G/A	Gene_Ca_14177	SYNONYMOUS_CODING	Plantperoxidase
Kabuli_wild_29958113	1399937153	*Ca_kabuli_Chr04*	29958113	G/A	NA	INTERGENIC	NA
Kabuli_wild_29958122	1399937154	*Ca_kabuli_Chr04*	29958122	T/G	NA	INTERGENIC	NA
Kabuli_wild_29972268	1399937155	*Ca_kabuli_Chr04*	29972268	T/G	NA	INTERGENIC	NA
Kabuli_wild_30028206	1399937156	*Ca_kabuli_Chr04*	30028206	C/A	NA	INTERGENIC	NA
Kabuli_wild_30131105	1399937157	*Ca_kabuli_Chr04*	30131105	T/C	NA	INTERGENIC	NA
Kabuli_wild_30153390	1399937158	*Ca_kabuli_Chr04*	30153390	G/A	Gene_Ca_14185	DOWNSTREAM	Plastid lipid-associated protein/fibrillin conserved domain
Kabuli_wild_30153448	1399937159	*Ca_kabuli_Chr04*	30153448	T/C	Gene_Ca_14185	DOWNSTREAM	Plastid lipid-associated protein/fibrillin conserved domain
Kabuli_wild_30153505	1399937160	*Ca_kabuli_Chr04*	30153505	C/G	Gene_Ca_14185	DOWNSTREAM	Plastid lipid-associated protein/fibrillin conserved domain
Kabuli_wild_30156596	1399937161	*Ca_kabuli_Chr04*	30156596	C/G	NA	INTERGENIC	NA
Kabuli_wild_30156624	1399937162	*Ca_kabuli_Chr04*	30156624	A/T	NA	INTERGENIC	NA
Kabuli_wild_30258044	1399937163	*Ca_kabuli_Chr04*	30258044	A/T	Gene_Ca_14190	DOWNSTREAM	Zinc finger,CCCH-type
Kabuli_wild_30258045	1399937164	*Ca_kabuli_Chr04*	30258045	A/T	Gene_Ca_14190	DOWNSTREAM	Zinc finger,CCCH-type
Kabuli_wild_30258056	1399937165	*Ca_kabuli_Chr04*	30258056	A/G	Gene_Ca_14190	DOWNSTREAM	Zinc finger,CCCH-type
Kabuli_wild_30258484	1399937166	*Ca_kabuli_Chr04*	30258484	G/C	Gene_Ca_14190	DOWNSTREAM	Zinc finger,CCCH-type
Kabuli_wild_30258567	1399937167	*Ca_kabuli_Chr04*	30258567	C/A	Gene_Ca_14190	DOWNSTREAM	Zinc finger,CCCH-type
Kabuli_wild_30258596	1399937168	*Ca_kabuli_Chr04*	30258596	G/C	Gene_Ca_14190	DOWNSTREAM	Zinc finger,CCCH-type
Kabuli_wild_30258604	1399937169	*Ca_kabuli_Chr04*	30258604	C/A	Gene_Ca_14190	DOWNSTREAM	Zinc finger,CCCH-type
Kabuli_wild_30258607	1399937170	*Ca_kabuli_Chr04*	30258607	C/A	Gene_Ca_14190	DOWNSTREAM	Zinc finger,CCCH-type
Kabuli_wild_30258613	1399937171	*Ca_kabuli_Chr04*	30258613	C/A	Gene_Ca_14190	DOWNSTREAM	Zinc finger,CCCH-type

SNP IDs	NCBI dbSNP accession IDs (NCBI_SS#)	Chromosomes	Physical positions (bp)	SNPs	Gene accession IDs	Sequence components of genome	Putative functions
Kabuli_wild_30258621	1399937172	*Ca_kabuli_Chr04*	30258621	T/G	Gene_Ca_14190	DOWNSTREAM	Zinc finger,CCCH-type
Kabuli_wild_30260485	1399937173	*Ca_kabuli_Chr04*	30260485	C/A	Gene_Ca_14189	DOWNSTREAM	Late embryo genesis abundant protein, group1
Kabuli_wild_30281991	1399937174	*Ca_kabuli_Chr04*	30281991	A/G	NA	INTERGENIC	NA
Kabuli_wild_30282333	1399937175	*Ca_kabuli_Chr04*	30282333	C/A	NA	INTERGENIC	NA
Kabuli_wild_30300334	1399937176	*Ca_kabuli_Chr04*	30300334	T/C	Gene_Ca_14192	SYNONYMOUS_CODING	SNF2-related
Kabuli_wild_30301002	1399937177	*Ca_kabuli_Chr04*	30301002	T/G	Gene_Ca_14192	SYNONYMOUS_CODING	SNF2-related
Kabuli_wild_30308719	1399937178	*Ca_kabuli_Chr04*	30308719	A/C	Gene_Ca_14192	INTRON	SNF2-related
Kabuli_wild_30308788	1399937179	*Ca_kabuli_Chr04*	30308788	T/C	Gene_Ca_14192	INTRON	SNF2-related
Kabuli_wild_30308812	1399937180	*Ca_kabuli_Chr04*	30308812	G/A	Gene_Ca_14192	INTRON	SNF2-related
Kabuli_wild_30334005	1399937181	*Ca_kabuli_Chr04*	30334005	G/A	NA	INTERGENIC	NA
Kabuli_wild_30463063	1399937182	*Ca_kabuli_Chr04*	30463063	T/C	NA	INTERGENIC	NA
Kabuli_wild_30463117	1399937183	*Ca_kabuli_Chr04*	30463117	C/A	NA	INTERGENIC	NA
Kabuli_wild_30566722	1399937184	*Ca_kabuli_Chr04*	30566722	C/T	NA	INTERGENIC	NA
Kabuli_wild_30579785	1399937185	*Ca_kabuli_Chr04*	30579785	T/C	Gene_Ca_14206	UPSTREAM	D111/G-patch
Kabuli_wild_30579795	1399937186	*Ca_kabuli_Chr04*	30579795	G/A	Gene_Ca_14206	UPSTREAM	D111/G-patch
Kabuli_wild_30579835	1399937187	*Ca_kabuli_Chr04*	30579835	G/T	Gene_Ca_14206	UPSTREAM	D111/G-patch
Kabuli_wild_30580184	1399937188	*Ca_kabuli_Chr04*	30580184	G/A	NA	INTERGENIC	NA
Kabuli_wild_30599096	1399937189	*Ca_kabuli_Chr04*	30599096	C/A	Gene_Ca_14207	SYNONYMOUS_CODING	Protein kinase,catalytic domain
Kabuli_wild_30599188	1399937190	*Ca_kabuli_Chr04*	30599188	C/T	Gene_Ca_14207	INTRON	Protein kinase,catalytic domain
Kabuli_wild_30668893	1399937191	*Ca_kabuli_Chr04*	30668893	A/C	NA	INTERGENIC	NA
Kabuli_wild_30851027	1399937192	*Ca_kabuli_Chr04*	30851027	A/T	Gene_Ca_14212	INTRON	Basic-leucine zipper (bZIP) Transcription factor
Kabuli_wild_30851411	1399937193	*Ca_kabuli_Chr04*	30851411	T/G	Gene_Ca_14212	INTRON	Basic-leucine zipper (bZIP) Transcription factor
Kabuli_wild_30851840	1399937194	*Ca_kabuli_Chr04*	30851840	A/T	Gene_Ca_14212	INTRON	Basic-leucine zipper (bZIP) Transcription factor
Kabuli_wild_30852212	1399937195	*Ca_kabuli_Chr04*	30852212	C/G	Gene_Ca_14212	INTRON	Basic-leucine zipper (bZIP) Transcription factor
Kabuli_wild_30852224	1399937196	*Ca_kabuli_Chr04*	30852224	T/G	Gene_Ca_14212	INTRON	Basic-leucine zipper (bZIP) Transcription factor
Kabuli_wild_30861487	1399937197	*Ca_kabuli_Chr04*	30861487	C/A	NA	INTERGENIC	NA
Kabuli_wild_30861508	1399937198	*Ca_kabuli_Chr04*	30861508	T/C	NA	INTERGENIC	NA
Kabuli_wild_30861515	1399937199	*Ca_kabuli_Chr04*	30861515	T/C	NA	INTERGENIC	NA
Kabuli_wild_30861537	1399937200	*Ca_kabuli_Chr04*	30861537	T/C	NA	INTERGENIC	NA
Kabuli_wild_30931586	1399937201	*Ca_kabuli_Chr04*	30931586	G/T	NA	INTERGENIC	NA
Kabuli_wild_30950605	1399937202	*Ca_kabuli_Chr04*	30950605	A/G	NA	INTERGENIC	NA
Kabuli_wild_30998138	1399937203	*Ca_kabuli_Chr04*	30998138	A/G	Gene_Ca_14216	DOWNSTREAM	Transposase,Ptta/En/Spm,plant
Kabuli_wild_30998185	1399937204	*Ca_kabuli_Chr04*	30998185	C/T	Gene_Ca_14216	DOWNSTREAM	Transposase,Ptta/En/Spm,plant
Kabuli_wild_31218647	1399937205	*Ca_kabuli_Chr04*	31218647	T/C	Gene_Ca_14226	INTRON	Ovariantumour,otubain
Kabuli_wild_31221226	1399937206	*Ca_kabuli_Chr04*	31221226	T/A	Gene_Ca_14226	UPSTREAM	Ovariantumour,otubain
Kabuli_wild_31221253	1399937207	*Ca_kabuli_Chr04*	31221253	G/A	Gene_Ca_14226	UPSTREAM	Ovariantumour,otubain
Kabuli_wild_31221264	1399937208	*Ca_kabuli_Chr04*	31221264	T/G	Gene_Ca_14226	UPSTREAM	Ovariantumour,otubain
Kabuli_wild_31224001	1399937209	*Ca_kabuli_Chr04*	31224001	G/A	NA	INTERGENIC	NA
Kabuli_wild_31262596	1399937210	*Ca_kabuli_Chr04*	31262596	T/A	NA	INTERGENIC	NA
Kabuli_wild_31263205	1399937211	*Ca_kabuli_Chr04*	31263205	A/T	Gene_Ca_14230	SYNONYMOUS_CODING	Pentatrico peptide repeat
Kabuli_wild_31591279	1399937212	*Ca_kabuli_Chr04*	31591279	G/A	NA	INTERGENIC	NA
Kabuli_wild_31594126	1399937213	*Ca_kabuli_Chr04*	31594126	A/C	NA	INTERGENIC	NA
Kabuli_wild_31618922	1399937214	*Ca_kabuli_Chr04*	31618922	T/C	NA	INTERGENIC	NA
Kabuli_wild_31618949	1399937215	*Ca_kabuli_Chr04*	31618949	T/A	NA	INTERGENIC	NA

SNP IDs	NCBI dbSNP accession IDs (NCBI_SS#)	Chromosomes	Physical positions (bp)	SNPs	Gene accession IDs	Sequence components of genome	Putative functions
Kabuli_wild_31918275	1399937216	*Ca_kabuli_Chr04*	31918275	A/G	NA	INTERGENIC	NA
Kabuli_wild_31987297	1399937217	*Ca_kabuli_Chr04*	31987297	G/T	Gene_Ca_15457	INTRON	Protein of unknown function DUF2363
Kabuli_wild_31987890	1399937218	*Ca_kabuli_Chr04*	31987890	A/C	Gene_Ca_15457	UPSTREAM	Protein of unknown function DUF2363
Kabuli_wild_32042488	1399937219	*Ca_kabuli_Chr04*	32042488	T/G	Gene_Ca_15459	SYNONYMOUS_CODING	Domain of unknown function DUF828
Kabuli_wild_32068961	1399937220	*Ca_kabuli_Chr04*	32068961	A/G	Gene_Ca_15461	SYNONYMOUS_CODING	Ankyrinrepeat
Kabuli_wild_32073549	1399937221	*Ca_kabuli_Chr04*	32073549	G/C	NA	INTERGENIC	NA
Kabuli_wild_32073550	1399937222	*Ca_kabuli_Chr04*	32073550	C/G	NA	INTERGENIC	NA
Kabuli_wild_32073552	1399937223	*Ca_kabuli_Chr04*	32073552	T/C	NA	INTERGENIC	NA
Kabuli_wild_32162032	1399937224	*Ca_kabuli_Chr04*	32162032	C/T	NA	INTERGENIC	NA
Kabuli_wild_32162075	1399937225	*Ca_kabuli_Chr04*	32162075	T/C	NA	INTERGENIC	NA
Kabuli_wild_32162081	1399937226	*Ca_kabuli_Chr04*	32162081	G/T	NA	INTERGENIC	NA
Kabuli_wild_32166557	1399937227	*Ca_kabuli_Chr04*	32166557	T/A	NA	INTERGENIC	NA
Kabuli_wild_32166559	1399937228	*Ca_kabuli_Chr04*	32166559	C/G	NA	INTERGENIC	NA
Kabuli_wild_32183307	1399937229	*Ca_kabuli_Chr04*	32183307	C/G	Gene_Ca_15464	SYNONYMOUS_CODING	Pentatrico peptide repeat
Kabuli_wild_32183366	1399937230	*Ca_kabuli_Chr04*	32183366	A/G	Gene_Ca_15464	NON_SYNONYMOUS_CODING	Pentatrico peptide repeat
Kabuli_wild_32184523	1399937231	*Ca_kabuli_Chr04*	32184523	T/G	Gene_Ca_15464	SYNONYMOUS_CODING	Pentatrico peptide repeat
Kabuli_wild_32184628	1399937232	*Ca_kabuli_Chr04*	32184628	A/G	Gene_Ca_15464	SYNONYMOUS_CODING	Pentatrico peptide repeat
Kabuli_wild_32212899	1399937233	*Ca_kabuli_Chr04*	32212899	A/G	NA	INTERGENIC	NA
Kabuli_wild_32297764	1399937234	*Ca_kabuli_Chr04*	32297764	A/G	Gene_Ca_15471	UPSTREAM	NA
Kabuli_wild_32303394	1399937235	*Ca_kabuli_Chr04*	32303394	C/T	NA	INTERGENIC	NA
Kabuli_wild_32405927	1399937236	*Ca_kabuli_Chr04*	32405927	T/C	NA	INTERGENIC	NA
Kabuli_wild_32429222	1399937237	*Ca_kabuli_Chr04*	32429222	A/C	Gene_Ca_15476	SYNONYMOUS_CODING	Armadillo
Kabuli_wild_32429228	1399937238	*Ca_kabuli_Chr04*	32429228	A/T	Gene_Ca_15476	NON_SYNONYMOUS_CODING	Armadillo
Kabuli_wild_32521346	1399937239	*Ca_kabuli_Chr04*	32521346	T/C	Gene_Ca_15480	INTRON	NA
Kabuli_wild_32602658	1399937240	*Ca_kabuli_Chr04*	32602658	C/T	NA	INTERGENIC	NA
Kabuli_wild_32604510	1399937241	*Ca_kabuli_Chr04*	32604510	A/C	Gene_Ca_15484	NON_SYNONYMOUS_CODING	Pentatrico peptide repeat
Kabuli_wild_32604538	1399937242	*Ca_kabuli_Chr04*	32604538	T/C	Gene_Ca_15484	SYNONYMOUS_CODING	Pentatrico peptide repeat
Kabuli_wild_32625733	1399937243	*Ca_kabuli_Chr04*	32625733	T/G	Gene_Ca_15485	INTRON	PeptidaseS9,prolyloligopeptidase,catalytic domain
Kabuli_wild_32635638	1399937244	*Ca_kabuli_Chr04*	32635638	A/G	NA	INTERGENIC	NA
Kabuli_wild_32636315	1399937245	*Ca_kabuli_Chr04*	32636315	T/C	NA	INTERGENIC	NA
Kabuli_wild_32636428	1399937246	*Ca_kabuli_Chr04*	32636428	C/A	NA	INTERGENIC	NA
Kabuli_wild_32636459	1399937247	*Ca_kabuli_Chr04*	32636459	A/G	NA	INTERGENIC	NA
Kabuli_wild_32636690	1399937248	*Ca_kabuli_Chr04*	32636690	C/T	NA	INTERGENIC	NA
Kabuli_wild_32637114	1399937249	*Ca_kabuli_Chr04*	32637114	A/C	NA	INTERGENIC	NA
Kabuli_wild_32639118	1399937250	*Ca_kabuli_Chr04*	32639118	A/C	Gene_Ca_15486	UPSTREAM	Pentatrico peptide repeat
Kabuli_wild_32639121	1399937251	*Ca_kabuli_Chr04*	32639121	C/T	Gene_Ca_15486	UPSTREAM	Pentatrico peptide repeat
Kabuli_wild_32639172	1399937252	*Ca_kabuli_Chr04*	32639172	A/C	Gene_Ca_15486	UPSTREAM	Pentatrico peptide repeat
Kabuli_wild_32639188	1399937253	*Ca_kabuli_Chr04*	32639188	C/G	Gene_Ca_15486	UPSTREAM	Pentatrico peptide repeat
Kabuli_wild_32639315	1399937254	*Ca_kabuli_Chr04*	32639315	A/G	Gene_Ca_15486	UPSTREAM	Pentatrico peptide repeat
Kabuli_wild_32639317	1399937255	*Ca_kabuli_Chr04*	32639317	A/C	Gene_Ca_15486	UPSTREAM	Pentatrico peptide repeat
Kabuli_wild_32639654	1399937256	*Ca_kabuli_Chr04*	32639654	T/C	Gene_Ca_15486	UPSTREAM	Pentatrico peptide repeat
Kabuli_wild_32639681	1399937257	*Ca_kabuli_Chr04*	32639681	C/T	NA	INTERGENIC	NA
Kabuli_wild_32647877	1399937258	*Ca_kabuli_Chr04*	32647877	A/C	Gene_Ca_15487	NON_SYNONYMOUS_CODING	NA
Kabuli_wild_32793291	1399937259	*Ca_kabuli_Chr04*	32793291	A/T	NA	INTERGENIC	NA

SNP IDs	NCBI dbSNP accession IDs (NCBI_SS#)	Chromosomes	Physical positions (bp)	SNPs	Gene accession IDs	Sequence components of genome	Putative functions
Kabuli_wild_32841664	1399937260	*Ca_kabuli_Chr04*	32841664	A/C	NA	INTERGENIC	NA
Kabuli_wild_32853834	1399937261	*Ca_kabuli_Chr04*	32853834	T/C	Gene_Ca_15495	INTRON	DNAtopoisomeraseI,C-terminal
Kabuli_wild_32853838	1399937262	*Ca_kabuli_Chr04*	32853838	G/T	Gene_Ca_15495	INTRON	DNAtopoisomeraseI,C-terminal
Kabuli_wild_33231667	1399937263	*Ca_kabuli_Chr04*	33231667	C/T	NA	INTERGENIC	NA
Kabuli_wild_33231669	1399937264	*Ca_kabuli_Chr04*	33231669	G/A	NA	INTERGENIC	NA
Kabuli_wild_33231699	1399937265	*Ca_kabuli_Chr04*	33231699	G/A	NA	INTERGENIC	NA
Kabuli_wild_33276702	1399937266	*Ca_kabuli_Chr04*	33276702	C/A	Gene_Ca_24020	SYNONYMOUS_CODING	Putativeesterase
Kabuli_wild_33316940	1399937267	*Ca_kabuli_Chr04*	33316940	C/A	NA	INTERGENIC	NA
Kabuli_wild_33374107	1399937268	*Ca_kabuli_Chr04*	33374107	G/A	NA	INTERGENIC	NA
Kabuli_wild_33374112	1399937269	*Ca_kabuli_Chr04*	33374112	T/C	NA	INTERGENIC	NA
Kabuli_wild_33374135	1399937270	*Ca_kabuli_Chr04*	33374135	C/T	NA	INTERGENIC	NA
Kabuli_wild_33374152	1399937271	*Ca_kabuli_Chr04*	33374152	T/A	NA	INTERGENIC	NA
Kabuli_wild_33374153	1399937272	*Ca_kabuli_Chr04*	33374153	T/C	NA	INTERGENIC	NA
Kabuli_wild_33509759	1399937273	*Ca_kabuli_Chr04*	33509759	A/C	NA	INTERGENIC	NA
Kabuli_wild_33509762	1399937274	*Ca_kabuli_Chr04*	33509762	G/A	NA	INTERGENIC	NA
Kabuli_wild_33509803	1399937275	*Ca_kabuli_Chr04*	33509803	C/T	NA	INTERGENIC	NA
Kabuli_wild_33509808	1399937276	*Ca_kabuli_Chr04*	33509808	G/A	NA	INTERGENIC	NA
Kabuli_wild_33509823	1399937277	*Ca_kabuli_Chr04*	33509823	G/A	NA	INTERGENIC	NA
Kabuli_wild_33626927	1399937278	*Ca_kabuli_Chr04*	33626927	C/A	NA	INTERGENIC	NA
Kabuli_wild_33626928	1399937279	*Ca_kabuli_Chr04*	33626928	C/T	NA	INTERGENIC	NA
Kabuli_wild_33626948	1399937280	*Ca_kabuli_Chr04*	33626948	C/T	NA	INTERGENIC	NA
Kabuli_wild_33626981	1399937281	*Ca_kabuli_Chr04*	33626981	T/A	NA	INTERGENIC	NA
Kabuli_wild_33641757	1399937282	*Ca_kabuli_Chr04*	33641757	C/T	Gene_Ca_24030	INTRON	SNF2-related
Kabuli_wild_33820657	1399937283	*Ca_kabuli_Chr04*	33820657	A/C	NA	INTERGENIC	NA
Kabuli_wild_33828673	1399937284	*Ca_kabuli_Chr04*	33828673	T/C	Gene_Ca_20369	INTRON	Uricase
Kabuli_wild_33828705	1399937285	*Ca_kabuli_Chr04*	33828705	T/A	Gene_Ca_20369	INTRON	Uricase
Kabuli_wild_33874313	1399937286	*Ca_kabuli_Chr04*	33874313	G/A	NA	INTERGENIC	NA
Kabuli_wild_33874327	1399937287	*Ca_kabuli_Chr04*	33874327	T/C	NA	INTERGENIC	NA
Kabuli_wild_33874346	1399937288	*Ca_kabuli_Chr04*	33874346	T/C	NA	INTERGENIC	NA
Kabuli_wild_33874375	1399937289	*Ca_kabuli_Chr04*	33874375	G/C	NA	INTERGENIC	NA
Kabuli_wild_33919432	1399937290	*Ca_kabuli_Chr04*	33919432	A/T	Gene_Ca_20365	SYNONYMOUS_CODING	NA
Kabuli_wild_33928810	1399937291	*Ca_kabuli_Chr04*	33928810	T/G	Gene_Ca_20365	INTRON	PeptidaseC19,ubiquitincarboxyl-terminalhydrolase2
Kabuli_wild_33929777	1399937292	*Ca_kabuli_Chr04*	33929777	C/T	Gene_Ca_20365	INTRON	PeptidaseC19,ubiquitincarboxyl-terminalhydrolase2
Kabuli_wild_34110771	1399937293	*Ca_kabuli_Chr04*	34110771	G/A	Gene_Ca_20361	DOWNSTREAM	NA
Kabuli_wild_34112210	1399937294	*Ca_kabuli_Chr04*	34112210	C/T	Gene_Ca_20362	DOWNSTREAM	NA
Kabuli_wild_34155076	1399937295	*Ca_kabuli_Chr04*	34155076	G/A	Gene_Ca_20360	NON_SYNONYMOUS_CODING	Peptidase C48, SUMO/Sentrin/Ubl1
Kabuli_wild_34155084	1399937296	*Ca_kabuli_Chr04*	34155084	C/T	Gene_Ca_20360	SYNONYMOUS_CODING	Peptidase C48, SUMO/Sentrin/Ubl1
Kabuli_wild_34155141	1399937297	*Ca_kabuli_Chr04*	34155141	C/T	Gene_Ca_20360	SYNONYMOUS_CODING	Peptidase C48, SUMO/Sentrin/Ubl1
Kabuli_wild_34155157	1399937298	*Ca_kabuli_Chr04*	34155157	G/C	Gene_Ca_20360	NON_SYNONYMOUS_CODING	Peptidase C48, SUMO/Sentrin/Ubl1
Kabuli_wild_34155160	1399937299	*Ca_kabuli_Chr04*	34155160	G/T	Gene_Ca_20360	NON_SYNONYMOUS_CODING	Peptidase C48, SUMO/Sentrin/Ubl1
Kabuli_wild_34222783	1399937300	*Ca_kabuli_Chr04*	34222783	T/C	NA	INTERGENIC	NA
Kabuli_wild_34222792	1399937301	*Ca_kabuli_Chr04*	34222792	G/C	NA	INTERGENIC	NA
Kabuli_wild_34354519	1399937302	*Ca_kabuli_Chr04*	34354519	G/T	NA	INTERGENIC	NA
Kabuli_wild_34487064	1399937303	*Ca_kabuli_Chr04*	34487064	T/G	Gene_Ca_20350	INTRON	Domain of unknown function DUF676,hydrolase-like

SNP IDs	NCBI dbSNP accession IDs (NCBI_SS#)	Chromosomes	Physical positions (bp)	SNPs	Gene accession IDs	Sequence components of genome	Putative functions
Kabuli_wild_34784285	1399937304	*Ca_kabuli_Chr04*	34784285	G/A	Gene_Ca_19990	NON_SYNONYMOUS_CODING	RibosomalproteinL22/L17
Kabuli_wild_34797959	1399937305	*Ca_kabuli_Chr04*	34797959	A/G	NA	INTERGENIC	NA
Kabuli_wild_34798597	1399937306	*Ca_kabuli_Chr04*	34798597	C/A	Gene_Ca_19992	INTRON	Pentatrico peptide repeat
Kabuli_wild_34798602	1399937307	*Ca_kabuli_Chr04*	34798602	G/A	Gene_Ca_19992	INTRON	Pentatrico peptide repeat
Kabuli_wild_34798657	1399937308	*Ca_kabuli_Chr04*	34798657	C/A	Gene_Ca_19992	INTRON	Pentatrico peptide repeat
Kabuli_wild_34915986	1399937309	*Ca_kabuli_Chr04*	34915986	C/T	Gene_Ca_19997	INTRON	Inosine/uridine-preferringnucleosidehydrolasedomain
Kabuli_wild_34916032	1399937310	*Ca_kabuli_Chr04*	34916032	C/T	Gene_Ca_19997	INTRON	Inosine/uridine-preferringnucleosidehydrolasedomain
Kabuli_wild_34916994	1399937311	*Ca_kabuli_Chr04*	34916994	G/A	Gene_Ca_19997	INTRON	Inosine/uridine-preferringnucleosidehydrolasedomain
Kabuli_wild_34924575	1399937312	*Ca_kabuli_Chr04*	34924575	C/T	Gene_Ca_19998	NON_SYNONYMOUS_CODING	Metallo-dependentphosphatase
Kabuli_wild_34955953	1399937313	*Ca_kabuli_Chr04*	34955953	A/G	Gene_Ca_19999	NON_SYNONYMOUS_CODING	Peptidase C48, SUMO/Sentrin/Ubl1
Kabuli_wild_34955985	1399937314	*Ca_kabuli_Chr04*	34955985	C/T	Gene_Ca_19999	SYNONYMOUS_CODING	Peptidase C48, SUMO/Sentrin/Ubl1
Kabuli_wild_34956006	1399937315	*Ca_kabuli_Chr04*	34956006	C/T	Gene_Ca_19999	SYNONYMOUS_CODING	Peptidase C48, SUMO/Sentrin/Ubl1
Kabuli_wild_34956023	1399937316	*Ca_kabuli_Chr04*	34956023	C/T	Gene_Ca_19999	NON_SYNONYMOUS_CODING	Peptidase C48, SUMO/Sentrin/Ubl1
Kabuli_wild_34956031	1399937317	*Ca_kabuli_Chr04*	34956031	G/A	Gene_Ca_19999	NON_SYNONYMOUS_CODING	Peptidase C48, SUMO/Sentrin/Ubl1
Kabuli_wild_34956034	1399937318	*Ca_kabuli_Chr04*	34956034	G/A	Gene_Ca_19999	NON_SYNONYMOUS_CODING	Peptidase C48, SUMO/Sentrin/Ubl1
Kabuli_wild_34956035	1399937319	*Ca_kabuli_Chr04*	34956035	C/T	Gene_Ca_19999	NON_SYNONYMOUS_CODING	Peptidase C48, SUMO/Sentrin/Ubl1
Kabuli_wild_34956083	1399937320	*Ca_kabuli_Chr04*	34956083	C/T	Gene_Ca_19999	NON_SYNONYMOUS_CODING	Peptidase C48, SUMO/Sentrin/Ubl1
Kabuli_wild_34956093	1399937321	*Ca_kabuli_Chr04*	34956093	C/T	Gene_Ca_19999	SYNONYMOUS_CODING	Peptidase C48, SUMO/Sentrin/Ubl1
Kabuli_wild_34956098	1399937322	*Ca_kabuli_Chr04*	34956098	G/A	Gene_Ca_19999	SYNONYMOUS_CODING	Peptidase C48, SUMO/Sentrin/Ubl1
Kabuli_wild_34956113	1399937323	*Ca_kabuli_Chr04*	34956113	C/T	Gene_Ca_19999	NON_SYNONYMOUS_CODING	Peptidase C48, SUMO/Sentrin/Ubl1
Kabuli_wild_35141194	1399937324	*Ca_kabuli_Chr04*	35141194	A/G	Gene_Ca_20010	INTRON	Glycosidehydrolase,family9
Kabuli_wild_35141281	1399937325	*Ca_kabuli_Chr04*	35141281	A/T	Gene_Ca_20010	NON_SYNONYMOUS_CODING	Glycosidehydrolase,family9
Kabuli_wild_35141309	1399937326	*Ca_kabuli_Chr04*	35141309	A/C	Gene_Ca_20010	SYNONYMOUS_CODING	Glycosidehydrolase,family9
Kabuli_wild_35141327	1399937327	*Ca_kabuli_Chr04*	35141327	G/T	Gene_Ca_20010	NON_SYNONYMOUS_CODING	Glycosidehydrolase,family9
Kabuli_wild_35141340	1399937328	*Ca_kabuli_Chr04*	35141340	G/A	Gene_Ca_20010	INTRON	Glycosidehydrolase,family9
Kabuli_wild_35141353	1399937329	*Ca_kabuli_Chr04*	35141353	C/G	Gene_Ca_20010	INTRON	Glycosidehydrolase,family9
Kabuli_wild_35141359	1399937330	*Ca_kabuli_Chr04*	35141359	C/G	Gene_Ca_20010	INTRON	Glycosidehydrolase,family9
Kabuli_wild_35141375	1399937331	*Ca_kabuli_Chr04*	35141375	A/G	Gene_Ca_20010	INTRON	Glycosidehydrolase,family9
Kabuli_wild_35141403	1399937332	*Ca_kabuli_Chr04*	35141403	C/T	Gene_Ca_20010	INTRON	Glycosidehydrolase,family9
Kabuli_wild_35168915	1399937333	*Ca_kabuli_Chr04*	35168915	C/T	Gene_Ca_20011	INTRON	NA
Kabuli_wild_35168939	1399937334	*Ca_kabuli_Chr04*	35168939	C/T	Gene_Ca_20011	INTRON	NA
Kabuli_wild_35168985	1399937335	*Ca_kabuli_Chr04*	35168985	C/T	Gene_Ca_20011	INTRON	NA
Kabuli_wild_35168995	1399937336	*Ca_kabuli_Chr04*	35168995	C/A	Gene_Ca_20011	INTRON	NA
Kabuli_wild_35171506	1399937337	*Ca_kabuli_Chr04*	35171506	C/T	Gene_Ca_20011	NON_SYNONYMOUS_CODING	NA
Kabuli_wild_35171719	1399937338	*Ca_kabuli_Chr04*	35171719	T/C	Gene_Ca_20011	INTRON	NA
Kabuli_wild_35229659	1399937339	*Ca_kabuli_Chr04*	35229659	T/C	Gene_Ca_20015	INTRON	NA
Kabuli_wild_35229850	1399937340	*Ca_kabuli_Chr04*	35229850	A/T	Gene_Ca_20015	INTRON	NA
Kabuli_wild_35229966	1399937341	*Ca_kabuli_Chr04*	35229966	C/T	Gene_Ca_20015	NON_SYNONYMOUS_CODING	NA
Kabuli_wild_35230106	1399937342	*Ca_kabuli_Chr04*	35230106	G/A	NA	INTERGENIC	NA
Kabuli_wild_35231891	1399937343	*Ca_kabuli_Chr04*	35231891	C/T	NA	INTERGENIC	NA
Kabuli_wild_35304102	1399937344	*Ca_kabuli_Chr04*	35304102	C/T	Gene_Ca_20017	SYNONYMOUS_CODING	NA
Kabuli_wild_35304108	1399937345	*Ca_kabuli_Chr04*	35304108	C/A	Gene_Ca_20017	NON_SYNONYMOUS_CODING	NA
Kabuli_wild_35304327	1399937346	*Ca_kabuli_Chr04*	35304327	G/A	Gene_Ca_20017	SYNONYMOUS_CODING	NA
Kabuli_wild_35309709	1399937347	*Ca_kabuli_Chr04*	35309709	A/G	Gene_Ca_20017	INTRON	NA

SNP IDs	NCBI dbSNP accession IDs (NCBI_SS#)	Chromosomes	Physical positions (bp)	SNPs	Gene accession IDs	Sequence components of genome	Putative functions
Kabuli_wild_35311815	1399937348	*Ca_kabuli_Chr04*	35311815	A/C	Gene_Ca_20017	SYNONYMOUS_CODING	NA
Kabuli_wild_35337282	1399937349	*Ca_kabuli_Chr04*	35337282	A/G	Gene_Ca_20018	INTRON	IQ motif,EF-handbinding site
Kabuli_wild_35344323	1399937350	*Ca_kabuli_Chr04*	35344323	C/T	Gene_Ca_20018	INTRON	IQ motif,EF-handbinding site
Kabuli_wild_35344336	1399937351	*Ca_kabuli_Chr04*	35344336	A/C	Gene_Ca_20018	INTRON	IQ motif,EF-handbinding site
Kabuli_wild_35344339	1399937352	*Ca_kabuli_Chr04*	35344339	A/T	Gene_Ca_20018	INTRON	IQ motif,EF-handbinding site
Kabuli_wild_35344353	1399937353	*Ca_kabuli_Chr04*	35344353	T/C	Gene_Ca_20018	INTRON	IQ motif,EF-handbinding site
Kabuli_wild_35344356	1399937354	*Ca_kabuli_Chr04*	35344356	A/G	Gene_Ca_20018	INTRON	IQ motif,EF-handbinding site
Kabuli_wild_35346354	1399937355	*Ca_kabuli_Chr04*	35346354	C/A	Gene_Ca_20018	INTRON	IQ motif,EF-handbinding site
Kabuli_wild_35346364	1399937356	*Ca_kabuli_Chr04*	35346364	C/T	Gene_Ca_20018	INTRON	IQ motif,EF-handbinding site
Kabuli_wild_35346386	1399937357	*Ca_kabuli_Chr04*	35346386	C/T	Gene_Ca_20018	INTRON	IQ motif,EF-handbinding site
Kabuli_wild_35346417	1399937358	*Ca_kabuli_Chr04*	35346417	C/T	Gene_Ca_20018	INTRON	IQ motif,EF-handbinding site
Kabuli_wild_35346465	1399937359	*Ca_kabuli_Chr04*	35346465	G/T	Gene_Ca_20018	INTRON	IQ motif,EF-handbinding site
Kabuli_wild_35346558	1399937360	*Ca_kabuli_Chr04*	35346558	G/T	Gene_Ca_20018	INTRON	IQ motif,EF-handbinding site
Kabuli_wild_35346563	1399937361	*Ca_kabuli_Chr04*	35346563	G/T	Gene_Ca_20018	INTRON	IQ motif,EF-handbinding site
Kabuli_wild_35346576	1399937362	*Ca_kabuli_Chr04*	35346576	C/G	Gene_Ca_20018	INTRON	IQ motif,EF-handbinding site
Kabuli_wild_35346624	1399937363	*Ca_kabuli_Chr04*	35346624	G/A	Gene_Ca_20018	INTRON	IQ motif,EF-handbinding site
Kabuli_wild_35346785	1399937364	*Ca_kabuli_Chr04*	35346785	G/A	Gene_Ca_20018	INTRON	IQ motif,EF-handbinding site
Kabuli_wild_35346804	1399937365	*Ca_kabuli_Chr04*	35346804	G/A	Gene_Ca_20018	INTRON	IQ motif,EF-handbinding site
Kabuli_wild_35346808	1399937366	*Ca_kabuli_Chr04*	35346808	G/A	Gene_Ca_20018	INTRON	IQ motif,EF-handbinding site
Kabuli_wild_35381267	1399937367	*Ca_kabuli_Chr04*	35381267	T/C	Gene_Ca_20020	SYNONYMOUS_CODING	Peptidase C48, SUMO/Sentrin/Ubl1
Kabuli_wild_35381289	1399937368	*Ca_kabuli_Chr04*	35381289	T/G	Gene_Ca_20020	NON_SYNONYMOUS_CODING	Peptidase C48, SUMO/Sentrin/Ubl1
Kabuli_wild_35393505	1399937369	*Ca_kabuli_Chr04*	35393505	G/T	Gene_Ca_20021	SYNONYMOUS_CODING	Transcription factor ,TCP
Kabuli_wild_35393643	1399937370	*Ca_kabuli_Chr04*	35393643	G/A	Gene_Ca_20021	SYNONYMOUS_CODING	Transcription factor ,TCP
Kabuli_wild_35455062	1399937371	*Ca_kabuli_Chr04*	35455062	A/T	NA	INTERGENIC	NA
Kabuli_wild_35455095	1399937372	*Ca_kabuli_Chr04*	35455095	A/C	NA	INTERGENIC	NA
Kabuli_wild_35455708	1399937373	*Ca_kabuli_Chr04*	35455708	G/A	NA	INTERGENIC	NA
Kabuli_wild_35469224	1399937374	*Ca_kabuli_Chr04*	35469224	C/T	Gene_Ca_22431	SYNONYMOUS_CODING	Sugar/inositol transporter
Kabuli_wild_35469304	1399937375	*Ca_kabuli_Chr04*	35469304	G/A	Gene_Ca_22431	NON_SYNONYMOUS_CODING	Sugar/inositol transporter
Kabuli_wild_35469308	1399937376	*Ca_kabuli_Chr04*	35469308	T/G	Gene_Ca_22431	NON_SYNONYMOUS_CODING	Sugar/inositol transporter
Kabuli_wild_35483917	1399937377	*Ca_kabuli_Chr04*	35483917	A/C	NA	INTERGENIC	NA
Kabuli_wild_35483942	1399937378	*Ca_kabuli_Chr04*	35483942	C/A	NA	INTERGENIC	NA
Kabuli_wild_35483950	1399937379	*Ca_kabuli_Chr04*	35483950	G/C	NA	INTERGENIC	NA
Kabuli_wild_35487471	1399937380	*Ca_kabuli_Chr04*	35487471	A/C	NA	INTERGENIC	NA
Kabuli_wild_35487496	1399937381	*Ca_kabuli_Chr04*	35487496	C/A	NA	INTERGENIC	NA
Kabuli_wild_35487504	1399937382	*Ca_kabuli_Chr04*	35487504	G/C	NA	INTERGENIC	NA
Kabuli_wild_35488920	1399937383	*Ca_kabuli_Chr04*	35488920	C/T	NA	INTERGENIC	NA
Kabuli_wild_35528758	1399937384	*Ca_kabuli_Chr04*	35528758	C/A	Gene_Ca_22434	INTRON	NA
Kabuli_wild_35528763	1399937385	*Ca_kabuli_Chr04*	35528763	G/A	Gene_Ca_22434	INTRON	NA
Kabuli_wild_35528893	1399937386	*Ca_kabuli_Chr04*	35528893	A/T	Gene_Ca_22434	INTRON	NA
Kabuli_wild_35529030	1399937387	*Ca_kabuli_Chr04*	35529030	G/A	Gene_Ca_22434	INTRON	NA
Kabuli_wild_35529076	1399937388	*Ca_kabuli_Chr04*	35529076	G/A	Gene_Ca_22434	INTRON	NA
Kabuli_wild_35534777	1399937389	*Ca_kabuli_Chr04*	35534777	G/C	Gene_Ca_22434	INTRON	NA
Kabuli_wild_35534834	1399937390	*Ca_kabuli_Chr04*	35534834	G/A	Gene_Ca_22434	INTRON	NA
Kabuli_wild_35534877	1399937391	*Ca_kabuli_Chr04*	35534877	T/C	Gene_Ca_22434	INTRON	NA

SNP IDs	NCBI dbSNP accession IDs (NCBI_SS#)	Chromosomes	Physical positions (bp)	SNPs	Gene accession IDs	Sequence components of genome	Putative functions
Kabuli_wild_35534891	1399937392	*Ca_kabuli_Chr04*	35534891	C/T	Gene_Ca_22434	INTRON	NA
Kabuli_wild_35543068	1399937393	*Ca_kabuli_Chr04*	35543068	G/T	Gene_Ca_22434	INTRON	NA
Kabuli_wild_35574088	1399937394	*Ca_kabuli_Chr04*	35574088	C/G	NA	INTERGENIC	NA
Kabuli_wild_35574112	1399937395	*Ca_kabuli_Chr04*	35574112	G/A	NA	INTERGENIC	NA
Kabuli_wild_35634712	1399937396	*Ca_kabuli_Chr04*	35634712	G/A	Gene_Ca_22441	DOWNSTREAM	Translation elongation factor EFG/EF2, C-terminal
Kabuli_wild_35634751	1399937397	*Ca_kabuli_Chr04*	35634751	G/A	Gene_Ca_22441	DOWNSTREAM	Translation elongation factor EFG/EF2, C-terminal
Kabuli_wild_35634873	1399937398	*Ca_kabuli_Chr04*	35634873	G/T	Gene_Ca_22441	DOWNSTREAM	Translation elongation factor EFG/EF2, C-terminal
Kabuli_wild_35635574	1399937399	*Ca_kabuli_Chr04*	35635574	C/T	NA	INTERGENIC	NA
Kabuli_wild_35635586	1399937400	*Ca_kabuli_Chr04*	35635586	A/C	NA	INTERGENIC	NA
Kabuli_wild_35635605	1399937401	*Ca_kabuli_Chr04*	35635605	C/T	NA	INTERGENIC	NA
Kabuli_wild_35635610	1399937402	*Ca_kabuli_Chr04*	35635610	C/T	NA	INTERGENIC	NA
Kabuli_wild_35635651	1399937403	*Ca_kabuli_Chr04*	35635651	T/C	NA	INTERGENIC	NA
Kabuli_wild_35780668	1399937404	*Ca_kabuli_Chr04*	35780668	G/C	NA	INTERGENIC	NA
Kabuli_wild_35961593	1399937405	*Ca_kabuli_Chr04*	35961593	A/C	NA	INTERGENIC	NA
Kabuli_wild_35977422	1399937406	*Ca_kabuli_Chr04*	35977422	G/A	NA	INTERGENIC	NA
Kabuli_wild_36005847	1399937407	*Ca_kabuli_Chr04*	36005847	T/G	NA	INTERGENIC	NA
Kabuli_wild_36010756	1399937408	*Ca_kabuli_Chr04*	36010756	T/G	NA	INTERGENIC	NA
Kabuli_wild_36155087	1399937409	*Ca_kabuli_Chr04*	36155087	G/A	Gene_Ca_14852	DOWNSTREAM	Cellular retinaldehyde-binding/triplefunction, C-terminal
Kabuli_wild_36222862	1399937410	*Ca_kabuli_Chr04*	36222862	G/T	Gene_Ca_14848	NON_SYNONYMOUS_CODING	ABC transporter-like
Kabuli_wild_36277383	1399937411	*Ca_kabuli_Chr04*	36277383	C/T	Gene_Ca_14843	NON_SYNONYMOUS_CODING	Helicase,C-terminal
Kabuli_wild_36311877	1399937412	*Ca_kabuli_Chr04*	36311877	C/T	NA	INTERGENIC	NA
Kabuli_wild_36311927	1399937413	*Ca_kabuli_Chr04*	36311927	A/G	NA	INTERGENIC	NA
Kabuli_wild_36311949	1399937414	*Ca_kabuli_Chr04*	36311949	A/T	NA	INTERGENIC	NA
Kabuli_wild_36311957	1399937415	*Ca_kabuli_Chr04*	36311957	C/T	NA	INTERGENIC	NA
Kabuli_wild_36311969	1399937416	*Ca_kabuli_Chr04*	36311969	G/A	NA	INTERGENIC	NA
Kabuli_wild_36445345	1399937417	*Ca_kabuli_Chr04*	36445345	G/A	Gene_Ca_14833	SYNONYMOUS_CODING	Armadillo
Kabuli_wild_36445367	1399937418	*Ca_kabuli_Chr04*	36445367	A/G	Gene_Ca_14833	NON_SYNONYMOUS_CODING	Armadillo
Kabuli_wild_36449101	1399937419	*Ca_kabuli_Chr04*	36449101	C/T	Gene_Ca_14833	INTRON	Armadillo
Kabuli_wild_36461836	1399937420	*Ca_kabuli_Chr04*	36461836	A/G	NA	INTERGENIC	NA
Kabuli_wild_36466198	1399937421	*Ca_kabuli_Chr04*	36466198	G/A	NA	INTERGENIC	NA
Kabuli_wild_36466237	1399937422	*Ca_kabuli_Chr04*	36466237	A/G	NA	INTERGENIC	NA
Kabuli_wild_36467928	1399937423	*Ca_kabuli_Chr04*	36467928	C/T	NA	INTERGENIC	NA
Kabuli_wild_36467943	1399937424	*Ca_kabuli_Chr04*	36467943	A/G	NA	INTERGENIC	NA
Kabuli_wild_36467964	1399937425	*Ca_kabuli_Chr04*	36467964	G/A	NA	INTERGENIC	NA
Kabuli_wild_36467967	1399937426	*Ca_kabuli_Chr04*	36467967	T/C	NA	INTERGENIC	NA
Kabuli_wild_36467980	1399937427	*Ca_kabuli_Chr04*	36467980	C/A	NA	INTERGENIC	NA
Kabuli_wild_36467988	1399937428	*Ca_kabuli_Chr04*	36467988	C/T	NA	INTERGENIC	NA
Kabuli_wild_36467990	1399937429	*Ca_kabuli_Chr04*	36467990	T/G	NA	INTERGENIC	NA
Kabuli_wild_36468003	1399937430	*Ca_kabuli_Chr04*	36468003	T/A	NA	INTERGENIC	NA
Kabuli_wild_36468053	1399937431	*Ca_kabuli_Chr04*	36468053	A/G	NA	INTERGENIC	NA
Kabuli_wild_36488955	1399937432	*Ca_kabuli_Chr04*	36488955	T/G	Gene_Ca_14828	NON_SYNONYMOUS_CODING	NA
Kabuli_wild_36490201	1399937433	*Ca_kabuli_Chr04*	36490201	T/C	Gene_Ca_14828	INTRON	NA
Kabuli_wild_36537118	1399937434	*Ca_kabuli_Chr04*	36537118	C/T	Gene_Ca_14825	SYNONYMOUS_CODING	Transcriptional factor B3
Kabuli_wild_36537124	1399937435	*Ca_kabuli_Chr04*	36537124	C/T	Gene_Ca_14825	SYNONYMOUS_CODING	Transcriptional factor B3

SNP IDs	NCBI dbSNP accession IDs (NCBI_SS#)	Chromosomes	Physical positions (bp)	SNPs	Gene accession IDs	Sequence components of genome	Putative functions
Kabuli_wild_36537145	1399937436	*Ca_kabuli_Chr04*	36537145	T/C	Gene_Ca_14825	SYNONYMOUS_CODING	Transcriptional factor B3
Kabuli_wild_36537170	1399937437	*Ca_kabuli_Chr04*	36537170	C/T	Gene_Ca_14825	NON_SYNONYMOUS_CODING	Transcriptional factor B3
Kabuli_wild_36541075	1399937438	*Ca_kabuli_Chr04*	36541075	G/A	NA	INTERGENIC	NA
Kabuli_wild_36541083	1399937439	*Ca_kabuli_Chr04*	36541083	A/C	NA	INTERGENIC	NA
Kabuli_wild_36541087	1399937440	*Ca_kabuli_Chr04*	36541087	T/C	NA	INTERGENIC	NA
Kabuli_wild_36541088	1399937441	*Ca_kabuli_Chr04*	36541088	G/T	NA	INTERGENIC	NA
Kabuli_wild_36639333	1399937442	*Ca_kabuli_Chr04*	36639333	A/T	Gene_Ca_14816	DOWNSTREAM	FAD dependent oxidoreductase
Kabuli_wild_36639382	1399937443	*Ca_kabuli_Chr04*	36639382	T/C	Gene_Ca_14816	DOWNSTREAM	FAD dependent oxidoreductase
Kabuli_wild_36639442	1399937444	*Ca_kabuli_Chr04*	36639442	G/A	Gene_Ca_14816	DOWNSTREAM	FAD dependent oxidoreductase
Kabuli_wild_36643459	1399937445	*Ca_kabuli_Chr04*	36643459	A/G	Gene_Ca_14816	INTRON	FAD dependent oxidoreductase
Kabuli_wild_36670226	1399937446	*Ca_kabuli_Chr04*	36670226	A/G	Gene_Ca_14814	INTRON	ATPase,AAA+type,core
Kabuli_wild_36670248	1399937447	*Ca_kabuli_Chr04*	36670248	G/A	Gene_Ca_14814	INTRON	ATPase,AAA+type,core
Kabuli_wild_36673677	1399937448	*Ca_kabuli_Chr04*	36673677	C/A	Gene_Ca_14813	DOWNSTREAM	Domain of unknown function DUF862,eukaryotic
Kabuli_wild_36682211	1399937449	*Ca_kabuli_Chr04*	36682211	G/A	Gene_Ca_14812	SYNONYMOUS_CODING	NA
Kabuli_wild_36682213	1399937450	*Ca_kabuli_Chr04*	36682213	C/T	Gene_Ca_14812	NON_SYNONYMOUS_CODING	NA
Kabuli_wild_36682215	1399937451	*Ca_kabuli_Chr04*	36682215	G/A	Gene_Ca_14812	NON_SYNONYMOUS_CODING	NA
Kabuli_wild_36682231	1399937452	*Ca_kabuli_Chr04*	36682231	G/T	Gene_Ca_14812	NON_SYNONYMOUS_CODING	NA
Kabuli_wild_36682253	1399937453	*Ca_kabuli_Chr04*	36682253	T/G	Gene_Ca_14812	SYNONYMOUS_CODING	NA
Kabuli_wild_36709731	1399937454	*Ca_kabuli_Chr04*	36709731	G/A	NA	INTERGENIC	NA
Kabuli_wild_36712600	1399937455	*Ca_kabuli_Chr04*	36712600	A/T	NA	INTERGENIC	NA
Kabuli_wild_36712662	1399937456	*Ca_kabuli_Chr04*	36712662	A/C	NA	INTERGENIC	NA
Kabuli_wild_36712806	1399937457	*Ca_kabuli_Chr04*	36712806	C/T	NA	INTERGENIC	NA
Kabuli_wild_36712838	1399937458	*Ca_kabuli_Chr04*	36712838	C/T	NA	INTERGENIC	NA
Kabuli_wild_36712840	1399937459	*Ca_kabuli_Chr04*	36712840	C/G	NA	INTERGENIC	NA
Kabuli_wild_36754121	1399937460	*Ca_kabuli_Chr04*	36754121	T/C	Gene_Ca_14808	INTRON	Phospho fructokinase domain
Kabuli_wild_36754290	1399937461	*Ca_kabuli_Chr04*	36754290	A/T	Gene_Ca_14808	INTRON	Phospho fructokinase domain
Kabuli_wild_36754327	1399937462	*Ca_kabuli_Chr04*	36754327	G/A	Gene_Ca_14808	INTRON	Phospho fructokinase domain
Kabuli_wild_36754328	1399937463	*Ca_kabuli_Chr04*	36754328	C/T	Gene_Ca_14808	INTRON	Phospho fructokinase domain
Kabuli_wild_36754343	1399937464	*Ca_kabuli_Chr04*	36754343	A/C	Gene_Ca_14808	INTRON	Phospho fructokinase domain
Kabuli_wild_36754348	1399937465	*Ca_kabuli_Chr04*	36754348	T/C	Gene_Ca_14808	INTRON	Phospho fructokinase domain
Kabuli_wild_36754377	1399937466	*Ca_kabuli_Chr04*	36754377	A/T	Gene_Ca_14808	INTRON	Phospho fructokinase domain
Kabuli_wild_36754378	1399937467	*Ca_kabuli_Chr04*	36754378	T/C	Gene_Ca_14808	INTRON	Phospho fructokinase domain
Kabuli_wild_36754453	1399937468	*Ca_kabuli_Chr04*	36754453	A/G	Gene_Ca_14808	SYNONYMOUS_CODING	Phospho fructokinase domain
Kabuli_wild_36754468	1399937469	*Ca_kabuli_Chr04*	36754468	G/A	Gene_Ca_14808	SYNONYMOUS_CODING	Phospho fructokinase domain
Kabuli_wild_36754542	1399937470	*Ca_kabuli_Chr04*	36754542	A/T	Gene_Ca_14808	INTRON	Phospho fructokinase domain
Kabuli_wild_36755322	1399937471	*Ca_kabuli_Chr04*	36755322	G/C	Gene_Ca_14808	INTRON	Phospho fructokinase domain
Kabuli_wild_36755324	1399937472	*Ca_kabuli_Chr04*	36755324	G/A	Gene_Ca_14808	INTRON	Phospho fructokinase domain
Kabuli_wild_36797039	1399937473	*Ca_kabuli_Chr04*	36797039	T/C	NA	INTERGENIC	NA
Kabuli_wild_36799551	1399937474	*Ca_kabuli_Chr04*	36799551	C/T	Gene_Ca_14807	SYNONYMOUS_CODING	RNA recognition motif domain
Kabuli_wild_36831199	1399937475	*Ca_kabuli_Chr04*	36831199	G/A	Gene_Ca_14805	NON_SYNONYMOUS_CODING	NA
Kabuli_wild_36831267	1399937476	*Ca_kabuli_Chr04*	36831267	A/C	Gene_Ca_14805	SYNONYMOUS_CODING	NA
Kabuli_wild_36955706	1399937477	*Ca_kabuli_Chr04*	36955706	G/T	Gene_Ca_14797	NON_SYNONYMOUS_CODING	Zinc finger,C2H2-type
Kabuli_wild_36975360	1399937478	*Ca_kabuli_Chr04*	36975360	G/A	Gene_Ca_14796	INTRON	Helicase,C-terminal
Kabuli_wild_36975361	1399937479	*Ca_kabuli_Chr04*	36975361	C/A	Gene_Ca_14796	INTRON	Helicase,C-terminal

SNP IDs	NCBI dbSNP accession IDs (NCBI_SS#)	Chromosomes	Physical positions (bp)	SNPs	Gene accession IDs	Sequence components of genome	Putative functions
Kabuli_wild_36975381	1399937480	*Ca_kabuli_Chr04*	36975381	A/G	Gene_Ca_14796	INTRON	Helicase,C-terminal
Kabuli_wild_36977109	1399937481	*Ca_kabuli_Chr04*	36977109	G/A	Gene_Ca_14796	NON_SYNONYMOUS_CODING	Helicase,C-terminal
Kabuli_wild_36977113	1399937482	*Ca_kabuli_Chr04*	36977113	G/A	Gene_Ca_14796	NON_SYNONYMOUS_CODING	Helicase,C-terminal
Kabuli_wild_36977131	1399937483	*Ca_kabuli_Chr04*	36977131	A/G	NA	INTERGENIC	NA
Kabuli_wild_36977192	1399937484	*Ca_kabuli_Chr04*	36977192	C/T	NA	INTERGENIC	NA
Kabuli_wild_36977194	1399937485	*Ca_kabuli_Chr04*	36977194	G/A	NA	INTERGENIC	NA
Kabuli_wild_36977280	1399937486	*Ca_kabuli_Chr04*	36977280	A/C	NA	INTERGENIC	NA
Kabuli_wild_36977283	1399937487	*Ca_kabuli_Chr04*	36977283	G/A	NA	INTERGENIC	NA
Kabuli_wild_36977287	1399937488	*Ca_kabuli_Chr04*	36977287	G/C	NA	INTERGENIC	NA
Kabuli_wild_36979879	1399937489	*Ca_kabuli_Chr04*	36979879	C/T	NA	INTERGENIC	NA
Kabuli_wild_36979926	1399937490	*Ca_kabuli_Chr04*	36979926	G/T	NA	INTERGENIC	NA
Kabuli_wild_36984727	1399937491	*Ca_kabuli_Chr04*	36984727	C/G	Gene_Ca_14795	INTRON	SNF2-related
Kabuli_wild_36984795	1399937492	*Ca_kabuli_Chr04*	36984795	T/A	Gene_Ca_14795	INTRON	SNF2-related
Kabuli_wild_36997937	1399937493	*Ca_kabuli_Chr04*	36997937	T/C	NA	INTERGENIC	NA
Kabuli_wild_36998201	1399937494	*Ca_kabuli_Chr04*	36998201	A/C	NA	INTERGENIC	NA
Kabuli_wild_36998231	1399937495	*Ca_kabuli_Chr04*	36998231	C/T	NA	INTERGENIC	NA
Kabuli_wild_36998252	1399937496	*Ca_kabuli_Chr04*	36998252	C/A	NA	INTERGENIC	NA
Kabuli_wild_37006188	1399937497	*Ca_kabuli_Chr04*	37006188	G/A	NA	INTERGENIC	NA
Kabuli_wild_37010855	1399937498	*Ca_kabuli_Chr04*	37010855	C/A	NA	INTERGENIC	NA
Kabuli_wild_37063558	1399937499	*Ca_kabuli_Chr04*	37063558	A/C	NA	INTERGENIC	NA
Kabuli_wild_37064588	1399937500	*Ca_kabuli_Chr04*	37064588	A/G	NA	INTERGENIC	NA
Kabuli_wild_37064630	1399937501	*Ca_kabuli_Chr04*	37064630	G/A	NA	INTERGENIC	NA
Kabuli_wild_37160622	1399937502	*Ca_kabuli_Chr04*	37160622	T/C	NA	INTERGENIC	NA
Kabuli_wild_37160625	1399937503	*Ca_kabuli_Chr04*	37160625	G/A	NA	INTERGENIC	NA
Kabuli_wild_37160654	1399937504	*Ca_kabuli_Chr04*	37160654	C/T	NA	INTERGENIC	NA
Kabuli_wild_37183865	1399937505	*Ca_kabuli_Chr04*	37183865	A/T	Gene_Ca_15153	NON_SYNONYMOUS_CODING	RNA-dependent RNA polymerase, eukaryotic-type
Kabuli_wild_37185576	1399937506	*Ca_kabuli_Chr04*	37185576	T/A	Gene_Ca_15153	INTRON	RNA-dependent RNA polymerase, eukaryotic-type
Kabuli_wild_37185577	1399937507	*Ca_kabuli_Chr04*	37185577	T/A	Gene_Ca_15153	INTRON	RNA-dependent RNA polymerase, eukaryotic-type
Kabuli_wild_37185578	1399937508	*Ca_kabuli_Chr04*	37185578	T/A	Gene_Ca_15153	INTRON	RNA-dependent RNA polymerase, eukaryotic-type
Kabuli_wild_37237211	1399937509	*Ca_kabuli_Chr04*	37237211	G/A	NA	INTERGENIC	NA
Kabuli_wild_37237276	1399937510	*Ca_kabuli_Chr04*	37237276	T/C	NA	INTERGENIC	NA
Kabuli_wild_37241235	1399937511	*Ca_kabuli_Chr04*	37241235	A/G	Gene_Ca_15151	SYNONYMOUS_CODING	Signal transduction response regulator, receiverdomain
Kabuli_wild_37241262	1399937512	*Ca_kabuli_Chr04*	37241262	G/A	Gene_Ca_15151	SYNONYMOUS_CODING	Signal transduction response regulator, receiverdomain
Kabuli_wild_37241962	1399937513	*Ca_kabuli_Chr04*	37241962	T/C	Gene_Ca_15151	INTRON	Signal transduction response regulator, receiverdomain
Kabuli_wild_37260701	1399937514	*Ca_kabuli_Chr04*	37260701	A/T	Gene_Ca_15149	INTRON	Dynamincentral domain
Kabuli_wild_37260841	1399937515	*Ca_kabuli_Chr04*	37260841	T/A	Gene_Ca_15149	INTRON	Dynamincentral domain
Kabuli_wild_37260873	1399937516	*Ca_kabuli_Chr04*	37260873	A/G	Gene_Ca_15149	SYNONYMOUS_CODING	Dynamincentral domain
Kabuli_wild_37264628	1399937517	*Ca_kabuli_Chr04*	37264628	G/A	Gene_Ca_15149	INTRON	Dynamincentral domain
Kabuli_wild_37264685	1399937518	*Ca_kabuli_Chr04*	37264685	T/C	Gene_Ca_15149	INTRON	Dynamincentral domain
Kabuli_wild_37274931	1399937519	*Ca_kabuli_Chr04*	37274931	C/T	Gene_Ca_15148	SYNONYMOUS_CODING	MyosinII heavychain-like
Kabuli_wild_37275816	1399937520	*Ca_kabuli_Chr04*	37275816	G/A	Gene_Ca_15148	INTRON	MyosinII heavychain-like
Kabuli_wild_37286591	1399937521	*Ca_kabuli_Chr04*	37286591	T/A	Gene_Ca_15147	INTRON	NA
Kabuli_wild_37286625	1399937522	*Ca_kabuli_Chr04*	37286625	C/A	Gene_Ca_15147	INTRON	NA
Kabuli_wild_37286632	1399937523	*Ca_kabuli_Chr04*	37286632	G/A	Gene_Ca_15147	INTRON	NA

SNP IDs	NCBI dbSNP accession IDs (NCBI_SS#)	Chromosomes	Physical positions (bp)	SNPs	Gene accession IDs	Sequence components of genome	Putative functions
Kabuli_wild_37286663	1399937524	*Ca_kabuli_Chr04*	37286663	A/G	Gene_Ca_15147	INTRON	NA
Kabuli_wild_37289370	1399937525	*Ca_kabuli_Chr04*	37289370	C/T	Gene_Ca_15147	DOWNSTREAM	NA
Kabuli_wild_37293752	1399937526	*Ca_kabuli_Chr04*	37293752	G/A	NA	INTERGENIC	NA
Kabuli_wild_37293769	1399937527	*Ca_kabuli_Chr04*	37293769	C/A	NA	INTERGENIC	NA
Kabuli_wild_37293773	1399937528	*Ca_kabuli_Chr04*	37293773	A/T	NA	INTERGENIC	NA
Kabuli_wild_37293780	1399937529	*Ca_kabuli_Chr04*	37293780	T/A	NA	INTERGENIC	NA
Kabuli_wild_37293825	1399937530	*Ca_kabuli_Chr04*	37293825	C/G	NA	INTERGENIC	NA
Kabuli_wild_37294720	1399937531	*Ca_kabuli_Chr04*	37294720	A/G	NA	INTERGENIC	NA
Kabuli_wild_37316574	1399937532	*Ca_kabuli_Chr04*	37316574	T/A	Gene_Ca_15144	SYNONYMOUS_CODING	Zinc finger, RING-type
Kabuli_wild_37316697	1399937533	*Ca_kabuli_Chr04*	37316697	A/C	Gene_Ca_15144	NON_SYNONYMOUS_CODING	Zinc finger, RING-type
Kabuli_wild_37316700	1399937534	*Ca_kabuli_Chr04*	37316700	C/A	Gene_Ca_15144	NON_SYNONYMOUS_CODING	Zinc finger, RING-type
Kabuli_wild_37316713	1399937535	*Ca_kabuli_Chr04*	37316713	A/T	Gene_Ca_15144	NON_SYNONYMOUS_CODING	Zinc finger, RING-type
Kabuli_wild_37316738	1399937536	*Ca_kabuli_Chr04*	37316738	C/T	Gene_Ca_15144	NON_SYNONYMOUS_CODING	Zinc finger, RING-type
Kabuli_wild_37316748	1399937537	*Ca_kabuli_Chr04*	37316748	T/A	Gene_Ca_15144	SYNONYMOUS_CODING	Zinc finger, RING-type
Kabuli_wild_37349071	1399937538	*Ca_kabuli_Chr04*	37349071	T/C	Gene_Ca_15141	INTRON	MethyltransferaseTRM13
Kabuli_wild_37349212	1399937539	*Ca_kabuli_Chr04*	37349212	A/C	Gene_Ca_15141	SYNONYMOUS_CODING	MethyltransferaseTRM13
Kabuli_wild_37349239	1399937540	*Ca_kabuli_Chr04*	37349239	C/G	Gene_Ca_15141	INTRON	MethyltransferaseTRM13
Kabuli_wild_37349304	1399937541	*Ca_kabuli_Chr04*	37349304	T/C	Gene_Ca_15141	INTRON	MethyltransferaseTRM13
Kabuli_wild_37349321	1399937542	*Ca_kabuli_Chr04*	37349321	T/C	Gene_Ca_15141	INTRON	MethyltransferaseTRM13
Kabuli_wild_37349331	1399937543	*Ca_kabuli_Chr04*	37349331	C/T	Gene_Ca_15141	INTRON	MethyltransferaseTRM13
Kabuli_wild_37356865	1399937544	*Ca_kabuli_Chr04*	37356865	C/A	Gene_Ca_15140	SYNONYMOUS_CODING	HEAT
Kabuli_wild_37356889	1399937545	*Ca_kabuli_Chr04*	37356889	C/T	Gene_Ca_15140	INTRON	HEAT
Kabuli_wild_37356892	1399937546	*Ca_kabuli_Chr04*	37356892	C/T	Gene_Ca_15140	INTRON	HEAT
Kabuli_wild_37356919	1399937547	*Ca_kabuli_Chr04*	37356919	A/T	Gene_Ca_15140	INTRON	HEAT
Kabuli_wild_37356951	1399937548	*Ca_kabuli_Chr04*	37356951	C/T	Gene_Ca_15140	INTRON	HEAT
Kabuli_wild_37357109	1399937549	*Ca_kabuli_Chr04*	37357109	T/C	Gene_Ca_15140	INTRON	HEAT
Kabuli_wild_37362545	1399937550	*Ca_kabuli_Chr04*	37362545	A/C	Gene_Ca_15140	NON_SYNONYMOUS_CODING	HEAT
Kabuli_wild_37362558	1399937551	*Ca_kabuli_Chr04*	37362558	G/A	Gene_Ca_15140	SYNONYMOUS_CODING	HEAT
Kabuli_wild_37362599	1399937552	*Ca_kabuli_Chr04*	37362599	G/A	Gene_Ca_15140	INTRON	HEAT
Kabuli_wild_37364980	1399937553	*Ca_kabuli_Chr04*	37364980	G/A	Gene_Ca_15140	SYNONYMOUS_CODING	HEAT
Kabuli_wild_37365148	1399937554	*Ca_kabuli_Chr04*	37365148	A/G	Gene_Ca_15140	SYNONYMOUS_CODING	HEAT
Kabuli_wild_37367903	1399937555	*Ca_kabuli_Chr04*	37367903	G/A	Gene_Ca_15139	SYNONYMOUS_CODING	Ankyrinrepeat
Kabuli_wild_37371262	1399937556	*Ca_kabuli_Chr04*	37371262	G/A	Gene_Ca_15139	SYNONYMOUS_CODING	Ankyrinrepeat
Kabuli_wild_37371263	1399937557	*Ca_kabuli_Chr04*	37371263	G/A	Gene_Ca_15139	NON_SYNONYMOUS_CODING	Ankyrinrepeat
Kabuli_wild_37433459	1399937558	*Ca_kabuli_Chr04*	37433459	G/T	Gene_Ca_15133	DOWNSTREAM	Integrase,catalyticcore
Kabuli_wild_37450206	1399937559	*Ca_kabuli_Chr04*	37450206	A/C	Gene_Ca_15131	SYNONYMOUS_CODING	Oxoglutarate/iron-dependent oxygenase
Kabuli_wild_37450294	1399937560	*Ca_kabuli_Chr04*	37450294	T/C	Gene_Ca_15131	SYNONYMOUS_CODING	Oxoglutarate/iron-dependent oxygenase
Kabuli_wild_37450326	1399937561	*Ca_kabuli_Chr04*	37450326	A/T	Gene_Ca_15131	SYNONYMOUS_CODING	Oxoglutarate/iron-dependent oxygenase
Kabuli_wild_37453449	1399937562	*Ca_kabuli_Chr04*	37453449	A/G	NA	INTERGENIC	NA
Kabuli_wild_37453493	1399937563	*Ca_kabuli_Chr04*	37453493	C/T	NA	INTERGENIC	NA
Kabuli_wild_37453497	1399937564	*Ca_kabuli_Chr04*	37453497	C/G	NA	INTERGENIC	NA
Kabuli_wild_37453586	1399937565	*Ca_kabuli_Chr04*	37453586	C/A	NA	INTERGENIC	NA
Kabuli_wild_37453590	1399937566	*Ca_kabuli_Chr04*	37453590	C/A	NA	INTERGENIC	NA
Kabuli_wild_37454296	1399937567	*Ca_kabuli_Chr04*	37454296	A/G	NA	INTERGENIC	NA

SNP IDs	NCBI dbSNP accession IDs (NCBI_SS#)	Chromosomes	Physical positions (bp)	SNPs	Gene accession IDs	Sequence components of genome	Putative functions
Kabuli_wild_37454374	1399937568	*Ca_kabuli_Chr04*	37454374	C/T	NA	INTERGENIC	NA
Kabuli_wild_37454428	1399937569	*Ca_kabuli_Chr04*	37454428	A/C	NA	INTERGENIC	NA
Kabuli_wild_37454567	1399937570	*Ca_kabuli_Chr04*	37454567	G/A	NA	INTERGENIC	NA
Kabuli_wild_37454571	1399937571	*Ca_kabuli_Chr04*	37454571	T/C	NA	INTERGENIC	NA
Kabuli_wild_37454593	1399937572	*Ca_kabuli_Chr04*	37454593	G/A	NA	INTERGENIC	NA
Kabuli_wild_37454603	1399937573	*Ca_kabuli_Chr04*	37454603	T/C	NA	INTERGENIC	NA
Kabuli_wild_37454636	1399937574	*Ca_kabuli_Chr04*	37454636	G/A	NA	INTERGENIC	NA
Kabuli_wild_37492422	1399937575	*Ca_kabuli_Chr04*	37492422	C/A	Gene_Ca_15129	DOWNSTREAM	Caleosin
Kabuli_wild_37549871	1399937576	*Ca_kabuli_Chr04*	37549871	A/T	Gene_Ca_15125	INTRON	Mediator complex subunit Med23
Kabuli_wild_37549883	1399937577	*Ca_kabuli_Chr04*	37549883	T/C	Gene_Ca_15125	INTRON	Mediator complex subunit Med23
Kabuli_wild_37549987	1399937578	*Ca_kabuli_Chr04*	37549987	C/T	Gene_Ca_15125	INTRON	Mediator complex subunit Med23
Kabuli_wild_37550122	1399937579	*Ca_kabuli_Chr04*	37550122	G/A	Gene_Ca_15125	INTRON	Mediator complex subunit Med23
Kabuli_wild_37550161	1399937580	*Ca_kabuli_Chr04*	37550161	T/G	Gene_Ca_15125	INTRON	Mediator complex subunit Med23
Kabuli_wild_37552973	1399937581	*Ca_kabuli_Chr04*	37552973	C/T	Gene_Ca_15125	INTRON	Mediator complex subunit Med23
Kabuli_wild_37558880	1399937582	*Ca_kabuli_Chr04*	37558880	C/T	Gene_Ca_15125	INTRON	Mediator complex subunit Med23
Kabuli_wild_37558881	1399937583	*Ca_kabuli_Chr04*	37558881	G/T	Gene_Ca_15125	INTRON	Mediator complex subunit Med23
Kabuli_wild_37575621	1399937584	*Ca_kabuli_Chr04*	37575621	G/A	Gene_Ca_15125	INTRON	Mediator complex subunit Med23
Kabuli_wild_37575636	1399937585	*Ca_kabuli_Chr04*	37575636	C/T	Gene_Ca_15125	INTRON	Mediator complex subunit Med23
Kabuli_wild_37575670	1399937586	*Ca_kabuli_Chr04*	37575670	T/C	Gene_Ca_15125	INTRON	Mediator complex subunit Med23
Kabuli_wild_37575709	1399937587	*Ca_kabuli_Chr04*	37575709	C/T	Gene_Ca_15125	INTRON	Mediator complex subunit Med23
Kabuli_wild_37576713	1399937588	*Ca_kabuli_Chr04*	37576713	T/A	Gene_Ca_15125	INTRON	Mediator complex subunit Med23
Kabuli_wild_37612154	1399937589	*Ca_kabuli_Chr04*	37612154	A/G	Gene_Ca_15121	SYNONYMOUS_CODING	Glycosidehydrolase,family17
Kabuli_wild_37630404	1399937590	*Ca_kabuli_Chr04*	37630404	A/G	NA	INTERGENIC	NA
Kabuli_wild_37630445	1399937591	*Ca_kabuli_Chr04*	37630445	A/G	NA	INTERGENIC	NA
Kabuli_wild_37630520	1399937592	*Ca_kabuli_Chr04*	37630520	G/C	NA	INTERGENIC	NA
Kabuli_wild_37630521	1399937593	*Ca_kabuli_Chr04*	37630521	G/A	NA	INTERGENIC	NA
Kabuli_wild_37630527	1399937594	*Ca_kabuli_Chr04*	37630527	A/G	NA	INTERGENIC	NA
Kabuli_wild_37632801	1399937595	*Ca_kabuli_Chr04*	37632801	C/T	Gene_Ca_15118	DOWNSTREAM	NA
Kabuli_wild_37632972	1399937596	*Ca_kabuli_Chr04*	37632972	T/C	Gene_Ca_15118	DOWNSTREAM	NA
Kabuli_wild_37632973	1399937597	*Ca_kabuli_Chr04*	37632973	A/G	Gene_Ca_15118	DOWNSTREAM	NA
Kabuli_wild_37633044	1399937598	*Ca_kabuli_Chr04*	37633044	C/T	Gene_Ca_15118	DOWNSTREAM	NA
Kabuli_wild_37635217	1399937599	*Ca_kabuli_Chr04*	37635217	C/T	Gene_Ca_15118	UPSTREAM	NA
Kabuli_wild_37635300	1399937600	*Ca_kabuli_Chr04*	37635300	T/A	Gene_Ca_15118	UPSTREAM	NA
Kabuli_wild_37685824	1399937601	*Ca_kabuli_Chr04*	37685824	T/C	Gene_Ca_15113	SYNONYMOUS_CODING	Armadillo
Kabuli_wild_37685842	1399937602	*Ca_kabuli_Chr04*	37685842	T/G	Gene_Ca_15113	SYNONYMOUS_CODING	Armadillo
Kabuli_wild_37699140	1399937603	*Ca_kabuli_Chr04*	37699140	C/T	Gene_Ca_15111	SYNONYMOUS_CODING	ATP-graspfold
Kabuli_wild_37699141	1399937604	*Ca_kabuli_Chr04*	37699141	T/G	Gene_Ca_15111	SYNONYMOUS_CODING	ATP-graspfold
Kabuli_wild_37699176	1399937605	*Ca_kabuli_Chr04*	37699176	G/A	Gene_Ca_15111	SYNONYMOUS_CODING	ATP-graspfold
Kabuli_wild_37703649	1399937606	*Ca_kabuli_Chr04*	37703649	A/C	Gene_Ca_15110	UPSTREAM	Cytochromecoxidase subunit IIC-terminal
Kabuli_wild_37703653	1399937607	*Ca_kabuli_Chr04*	37703653	A/C	Gene_Ca_15110	UPSTREAM	Cytochromecoxidase subunit IIC-terminal
Kabuli_wild_37708260	1399937608	*Ca_kabuli_Chr04*	37708260	A/G	NA	INTERGENIC	NA
Kabuli_wild_37708334	1399937609	*Ca_kabuli_Chr04*	37708334	T/A	NA	INTERGENIC	NA
Kabuli_wild_37708348	1399937610	*Ca_kabuli_Chr04*	37708348	T/G	NA	INTERGENIC	NA
Kabuli_wild_37753479	1399937611	*Ca_kabuli_Chr04*	37753479	C/T	Gene_Ca_15104	INTRON	NA

SNP IDs	NCBI dbSNP accession IDs (NCBI_SS#)	Chromosomes	Physical positions (bp)	SNPs	Gene accession IDs	Sequence components of genome	Putative functions
Kabuli_wild_37754999	1399937612	*Ca_kabuli_Chr04*	37754999	C/A	Gene_Ca_15104	INTRON	NA
Kabuli_wild_37772619	1399937613	*Ca_kabuli_Chr04*	37772619	G/A	NA	INTERGENIC	NA
Kabuli_wild_37797651	1399937614	*Ca_kabuli_Chr04*	37797651	G/C	NA	INTERGENIC	NA
Kabuli_wild_37797676	1399937615	*Ca_kabuli_Chr04*	37797676	C/T	NA	INTERGENIC	NA
Kabuli_wild_37806689	1399937616	*Ca_kabuli_Chr04*	37806689	T/C	Gene_Ca_15095	DOWNSTREAM	NUDIX hydrolase domain
Kabuli_wild_37812917	1399937617	*Ca_kabuli_Chr04*	37812917	G/C	NA	INTERGENIC	NA
Kabuli_wild_37859069	1399937618	*Ca_kabuli_Chr04*	37859069	T/G	Gene_Ca_15089	SYNONYMOUS_CODING	Auxin efflux carrier
Kabuli_wild_37897261	1399937619	*Ca_kabuli_Chr04*	37897261	T/G	NA	INTERGENIC	NA
Kabuli_wild_37897266	1399937620	*Ca_kabuli_Chr04*	37897266	A/T	NA	INTERGENIC	NA
Kabuli_wild_37897351	1399937621	*Ca_kabuli_Chr04*	37897351	A/C	NA	INTERGENIC	NA
Kabuli_wild_37898404	1399937622	*Ca_kabuli_Chr04*	37898404	A/G	NA	INTERGENIC	NA
Kabuli_wild_37901584	1399937623	*Ca_kabuli_Chr04*	37901584	C/T	NA	INTERGENIC	NA
Kabuli_wild_38008062	1399937624	*Ca_kabuli_Chr04*	38008062	T/A	NA	INTERGENIC	NA
Kabuli_wild_38008082	1399937625	*Ca_kabuli_Chr04*	38008082	T/C	NA	INTERGENIC	NA
Kabuli_wild_38008174	1399937626	*Ca_kabuli_Chr04*	38008174	A/G	NA	INTERGENIC	NA
Kabuli_wild_38008186	1399937627	*Ca_kabuli_Chr04*	38008186	A/C	NA	INTERGENIC	NA
Kabuli_wild_38008204	1399937628	*Ca_kabuli_Chr04*	38008204	G/A	NA	INTERGENIC	NA
Kabuli_wild_38008205	1399937629	*Ca_kabuli_Chr04*	38008205	A/G	NA	INTERGENIC	NA
Kabuli_wild_38008213	1399937630	*Ca_kabuli_Chr04*	38008213	C/T	NA	INTERGENIC	NA
Kabuli_wild_38008227	1399937631	*Ca_kabuli_Chr04*	38008227	G/T	NA	INTERGENIC	NA
Kabuli_wild_38008243	1399937632	*Ca_kabuli_Chr04*	38008243	C/T	NA	INTERGENIC	NA
Kabuli_wild_38020224	1399937633	*Ca_kabuli_Chr04*	38020224	C/T	Gene_Ca_13143	SYNONYMOUS_CODING	Aminoacid/polyaminetransporterI
Kabuli_wild_38029077	1399937634	*Ca_kabuli_Chr04*	38029077	G/A	Gene_Ca_13142	INTRON	ChaperoninTCP-1,conservedsite
Kabuli_wild_38029136	1399937635	*Ca_kabuli_Chr04*	38029136	G/T	Gene_Ca_13142	NON_SYNONYMOUS_CODING	ChaperoninTCP-1,conservedsite
Kabuli_wild_38029208	1399937636	*Ca_kabuli_Chr04*	38029208	G/A	Gene_Ca_13142	SYNONYMOUS_CODING	ChaperoninTCP-1,conservedsite
Kabuli_wild_38029215	1399937637	*Ca_kabuli_Chr04*	38029215	A/T	Gene_Ca_13142	INTRON	ChaperoninTCP-1,conservedsite
Kabuli_wild_38029358	1399937638	*Ca_kabuli_Chr04*	38029358	A/G	Gene_Ca_13142	INTRON	ChaperoninTCP-1,conservedsite
Kabuli_wild_38029380	1399937639	*Ca_kabuli_Chr04*	38029380	G/A	Gene_Ca_13142	INTRON	ChaperoninTCP-1,conservedsite
Kabuli_wild_38038865	1399937640	*Ca_kabuli_Chr04*	38038865	C/T	Gene_Ca_13141	NON_SYNONYMOUS_CODING	Protein of unknown function DUF3326
Kabuli_wild_38038959	1399937641	*Ca_kabuli_Chr04*	38038959	A/G	Gene_Ca_13141	INTRON	Protein of unknown function DUF3326
Kabuli_wild_38039037	1399937642	*Ca_kabuli_Chr04*	38039037	C/T	Gene_Ca_13141	INTRON	Protein of unknown function DUF3326
Kabuli_wild_38039046	1399937643	*Ca_kabuli_Chr04*	38039046	C/T	Gene_Ca_13141	INTRON	Protein of unknown function DUF3326
Kabuli_wild_38039076	1399937644	*Ca_kabuli_Chr04*	38039076	A/G	Gene_Ca_13141	INTRON	Protein of unknown function DUF3326
Kabuli_wild_38039088	1399937645	*Ca_kabuli_Chr04*	38039088	T/C	Gene_Ca_13141	INTRON	Protein of unknown function DUF3326
Kabuli_wild_38039251	1399937646	*Ca_kabuli_Chr04*	38039251	G/C	Gene_Ca_13141	INTRON	Protein of unknown function DUF3326
Kabuli_wild_38039252	1399937647	*Ca_kabuli_Chr04*	38039252	T/A	Gene_Ca_13141	INTRON	Protein of unknown function DUF3326
Kabuli_wild_38039255	1399937648	*Ca_kabuli_Chr04*	38039255	T/C	Gene_Ca_13141	INTRON	Protein of unknown function DUF3326
Kabuli_wild_38039289	1399937649	*Ca_kabuli_Chr04*	38039289	G/A	Gene_Ca_13141	INTRON	Protein of unknown function DUF3326
Kabuli_wild_38039297	1399937650	*Ca_kabuli_Chr04*	38039297	C/T	Gene_Ca_13141	NON_SYNONYMOUS_CODING	Protein of unknown function DUF3326
Kabuli_wild_38142776	1399937651	*Ca_kabuli_Chr04*	38142776	G/A	NA	INTERGENIC	NA
Kabuli_wild_38153183	1399937652	*Ca_kabuli_Chr04*	38153183	C/A	Gene_Ca_13138	SYNONYMOUS_CODING	Tetrahydrofolate dehydrogenase/cyclohydrolase
Kabuli_wild_38153195	1399937653	*Ca_kabuli_Chr04*	38153195	T/C	Gene_Ca_13138	SYNONYMOUS_CODING	Tetrahydrofolate dehydrogenase/cyclohydrolase
Kabuli_wild_38153198	1399937654	*Ca_kabuli_Chr04*	38153198	G/T	Gene_Ca_13138	SYNONYMOUS_CODING	Tetrahydrofolate dehydrogenase/cyclohydrolase
Kabuli_wild_38153216	1399937655	*Ca_kabuli_Chr04*	38153216	T/C	Gene_Ca_13138	SYNONYMOUS_CODING	Tetrahydrofolate dehydrogenase/cyclohydrolase

SNP IDs	NCBI dbSNP accession IDs (NCBI_SS#)	Chromosomes	Physical positions (bp)	SNPs	Gene accession IDs	Sequence components of genome	Putative functions
Kabuli_wild_38153275	1399937656	*Ca_kabuli_Chr04*	38153275	C/A	Gene_Ca_13138	INTRON	Tetrahydrofolate dehydrogenase/cyclohydrolase
Kabuli_wild_38153277	1399937657	*Ca_kabuli_Chr04*	38153277	A/G	Gene_Ca_13138	INTRON	Tetrahydrofolate dehydrogenase/cyclohydrolase
Kabuli_wild_38156918	1399937658	*Ca_kabuli_Chr04*	38156918	A/T	NA	INTERGENIC	NA
Kabuli_wild_38157210	1399937659	*Ca_kabuli_Chr04*	38157210	G/T	NA	INTERGENIC	NA
Kabuli_wild_38157279	1399937660	*Ca_kabuli_Chr04*	38157279	T/A	NA	INTERGENIC	NA
Kabuli_wild_38179583	1399937661	*Ca_kabuli_Chr04*	38179583	A/C	NA	INTERGENIC	NA
Kabuli_wild_38179614	1399937662	*Ca_kabuli_Chr04*	38179614	G/C	NA	INTERGENIC	NA
Kabuli_wild_38179621	1399937663	*Ca_kabuli_Chr04*	38179621	A/T	NA	INTERGENIC	NA
Kabuli_wild_38179624	1399937664	*Ca_kabuli_Chr04*	38179624	A/C	NA	INTERGENIC	NA
Kabuli_wild_38181292	1399937665	*Ca_kabuli_Chr04*	38181292	G/A	Gene_Ca_13135	NON_SYNONYMOUS_CODING	NA
Kabuli_wild_38195044	1399937666	*Ca_kabuli_Chr04*	38195044	G/C	Gene_Ca_13134	INTRON	Domain of unknown function DUF250
Kabuli_wild_38195346	1399937667	*Ca_kabuli_Chr04*	38195346	G/A	Gene_Ca_13134	SYNONYMOUS_CODING	Domain of unknown function DUF250
Kabuli_wild_38195411	1399937668	*Ca_kabuli_Chr04*	38195411	T/A	Gene_Ca_13134	NON_SYNONYMOUS_CODING	Domain of unknown function DUF250
Kabuli_wild_38195438	1399937669	*Ca_kabuli_Chr04*	38195438	C/T	Gene_Ca_13134	NON_SYNONYMOUS_CODING	Domain of unknown function DUF250
Kabuli_wild_38215267	1399937670	*Ca_kabuli_Chr04*	38215267	G/A	Gene_Ca_13132	INTRON	AMP-dependentsynthetase/ligase
Kabuli_wild_38221009	1399937671	*Ca_kabuli_Chr04*	38221009	A/G	Gene_Ca_13131	SYNONYMOUS_CODING	Lipase,GDSL
Kabuli_wild_38288823	1399937672	*Ca_kabuli_Chr04*	38288823	G/T	Gene_Ca_13128	SYNONYMOUS_CODING	Protein of unknown function DUF869,plant
Kabuli_wild_38288877	1399937673	*Ca_kabuli_Chr04*	38288877	C/G	Gene_Ca_13128	NON_SYNONYMOUS_CODING	Protein of unknown function DUF869,plant
Kabuli_wild_38288997	1399937674	*Ca_kabuli_Chr04*	38288997	T/A	Gene_Ca_13128	SYNONYMOUS_CODING	Protein of unknown function DUF869,plant
Kabuli_wild_38289052	1399937675	*Ca_kabuli_Chr04*	38289052	G/A	Gene_Ca_13128	SYNONYMOUS_CODING	Protein of unknown function DUF869,plant
Kabuli_wild_38289066	1399937676	*Ca_kabuli_Chr04*	38289066	G/A	Gene_Ca_13128	SYNONYMOUS_CODING	Protein of unknown function DUF869,plant
Kabuli_wild_38343633	1399937677	*Ca_kabuli_Chr04*	38343633	A/T	NA	INTERGENIC	NA
Kabuli_wild_38343687	1399937678	*Ca_kabuli_Chr04*	38343687	C/T	NA	INTERGENIC	NA
Kabuli_wild_38343750	1399937679	*Ca_kabuli_Chr04*	38343750	C/T	NA	INTERGENIC	NA
Kabuli_wild_38343796	1399937680	*Ca_kabuli_Chr04*	38343796	C/T	NA	INTERGENIC	NA
Kabuli_wild_38343837	1399937681	*Ca_kabuli_Chr04*	38343837	A/G	NA	INTERGENIC	NA
Kabuli_wild_38358067	1399937682	*Ca_kabuli_Chr04*	38358067	A/G	Gene_Ca_13120	NON_SYNONYMOUS_CODING	C2 calcium-dependent membrane targeting
Kabuli_wild_38358398	1399937683	*Ca_kabuli_Chr04*	38358398	A/G	Gene_Ca_13120	SYNONYMOUS_CODING	C2 calcium-dependent membrane targeting
Kabuli_wild_38358677	1399937684	*Ca_kabuli_Chr04*	38358677	G/A	Gene_Ca_13120	SYNONYMOUS_CODING	C2 calcium-dependent membrane targeting
Kabuli_wild_38358887	1399937685	*Ca_kabuli_Chr04*	38358887	G/A	Gene_Ca_13120	SYNONYMOUS_CODING	C2 calcium-dependent membrane targeting
Kabuli_wild_38359395	1399937686	*Ca_kabuli_Chr04*	38359395	A/C	Gene_Ca_13120	NON_SYNONYMOUS_CODING	C2 calcium-dependent membrane targeting
Kabuli_wild_38362132	1399937687	*Ca_kabuli_Chr04*	38362132	T/A	Gene_Ca_13120	SYNONYMOUS_CODING	C2 calcium-dependent membrane targeting
Kabuli_wild_38362575	1399937688	*Ca_kabuli_Chr04*	38362575	T/C	Gene_Ca_13120	NON_SYNONYMOUS_CODING	C2 calcium-dependent membrane targeting
Kabuli_wild_38370848	1399937689	*Ca_kabuli_Chr04*	38370848	A/G	NA	INTERGENIC	NA
Kabuli_wild_38370864	1399937690	*Ca_kabuli_Chr04*	38370864	G/A	NA	INTERGENIC	NA
Kabuli_wild_38370905	1399937691	*Ca_kabuli_Chr04*	38370905	A/G	NA	INTERGENIC	NA
Kabuli_wild_38370921	1399937692	*Ca_kabuli_Chr04*	38370921	T/G	NA	INTERGENIC	NA
Kabuli_wild_38370925	1399937693	*Ca_kabuli_Chr04*	38370925	G/A	NA	INTERGENIC	NA
Kabuli_wild_38370926	1399937694	*Ca_kabuli_Chr04*	38370926	T/C	NA	INTERGENIC	NA
Kabuli_wild_38370941	1399937695	*Ca_kabuli_Chr04*	38370941	A/G	NA	INTERGENIC	NA
Kabuli_wild_38370966	1399937696	*Ca_kabuli_Chr04*	38370966	T/C	NA	INTERGENIC	NA
Kabuli_wild_38382889	1399937697	*Ca_kabuli_Chr04*	38382889	C/A	Gene_Ca_13119	NON_SYNONYMOUS_CODING	Aminoacid transporter,transmembrane
Kabuli_wild_38382890	1399937698	*Ca_kabuli_Chr04*	38382890	C/G	Gene_Ca_13119	SYNONYMOUS_CODING	Aminoacid transporter,transmembrane
Kabuli_wild_38405123	1399937699	*Ca_kabuli_Chr04*	38405123	C/T	Gene_Ca_13117	INTRON	Protein of unknown function DUF248, methyltransferase

SNP IDs	NCBI dbSNP accession IDs (NCBI_SS#)	Chromosomes	Physical positions (bp)	SNPs	Gene accession IDs	Sequence components of genome	Putative functions
Kabuli_wild_38405153	1399937700	*Ca_kabuli_Chr04*	38405153	C/T	Gene_Ca_13117	INTRON	Protein of unknown function DUF248, methyltransferase
Kabuli_wild_38421798	1399937701	*Ca_kabuli_Chr04*	38421798	A/C	Gene_Ca_13115	INTRON	Zinc finger,CCCH-type
Kabuli_wild_38422037	1399937702	*Ca_kabuli_Chr04*	38422037	C/T	Gene_Ca_13115	INTRON	Zinc finger,CCCH-type
Kabuli_wild_38425675	1399937703	*Ca_kabuli_Chr04*	38425675	A/G	Gene_Ca_13115	SYNONYMOUS_CODING	Zinc finger,CCCH-type
Kabuli_wild_38425720	1399937704	*Ca_kabuli_Chr04*	38425720	T/A	Gene_Ca_13115	NON_SYNONYMOUS_CODING	Zinc finger,CCCH-type
Kabuli_wild_38434000	1399937705	*Ca_kabuli_Chr04*	38434000	T/C	Gene_Ca_13114	SYNONYMOUS_CODING	Protein kinase,catalytic domain
Kabuli_wild_38473653	1399937706	*Ca_kabuli_Chr04*	38473653	T/C	Gene_Ca_13112	INTRON	NA
Kabuli_wild_38474108	1399937707	*Ca_kabuli_Chr04*	38474108	T/C	Gene_Ca_13112	INTRON	NA
Kabuli_wild_38474123	1399937708	*Ca_kabuli_Chr04*	38474123	A/T	Gene_Ca_13112	INTRON	NA
Kabuli_wild_38474192	1399937709	*Ca_kabuli_Chr04*	38474192	T/C	Gene_Ca_13112	INTRON	NA
Kabuli_wild_38475310	1399937710	*Ca_kabuli_Chr04*	38475310	A/C	Gene_Ca_13112	INTRON	NA
Kabuli_wild_38475424	1399937711	*Ca_kabuli_Chr04*	38475424	C/G	Gene_Ca_13112	INTRON	NA
Kabuli_wild_38475425	1399937712	*Ca_kabuli_Chr04*	38475425	A/G	Gene_Ca_13112	INTRON	NA
Kabuli_wild_38475442	1399937713	*Ca_kabuli_Chr04*	38475442	A/G	Gene_Ca_13112	INTRON	NA
Kabuli_wild_38475449	1399937714	*Ca_kabuli_Chr04*	38475449	T/A	Gene_Ca_13112	INTRON	NA
Kabuli_wild_38475517	1399937715	*Ca_kabuli_Chr04*	38475517	G/A	Gene_Ca_13112	INTRON	NA
Kabuli_wild_38475547	1399937716	*Ca_kabuli_Chr04*	38475547	C/T	Gene_Ca_13112	INTRON	NA
Kabuli_wild_38536170	1399937717	*Ca_kabuli_Chr04*	38536170	G/A	NA	INTERGENIC	NA
Kabuli_wild_38536332	1399937718	*Ca_kabuli_Chr04*	38536332	A/C	NA	INTERGENIC	NA
Kabuli_wild_38540201	1399937719	*Ca_kabuli_Chr04*	38540201	C/T	NA	INTERGENIC	NA
Kabuli_wild_38540247	1399937720	*Ca_kabuli_Chr04*	38540247	G/A	NA	INTERGENIC	NA
Kabuli_wild_38554024	1399937721	*Ca_kabuli_Chr04*	38554024	T/C	NA	INTERGENIC	NA
Kabuli_wild_38612245	1399937722	*Ca_kabuli_Chr04*	38612245	C/T	NA	INTERGENIC	NA
Kabuli_wild_38620579	1399937723	*Ca_kabuli_Chr04*	38620579	C/T	NA	INTERGENIC	NA
Kabuli_wild_38620620	1399937724	*Ca_kabuli_Chr04*	38620620	A/T	NA	INTERGENIC	NA
Kabuli_wild_38659377	1399937725	*Ca_kabuli_Chr04*	38659377	T/C	NA	INTERGENIC	NA
Kabuli_wild_38659398	1399937726	*Ca_kabuli_Chr04*	38659398	G/A	NA	INTERGENIC	NA
Kabuli_wild_38659534	1399937727	*Ca_kabuli_Chr04*	38659534	T/A	NA	INTERGENIC	NA
Kabuli_wild_38659607	1399937728	*Ca_kabuli_Chr04*	38659607	T/C	NA	INTERGENIC	NA
Kabuli_wild_38679987	1399937729	*Ca_kabuli_Chr04*	38679987	A/G	Gene_Ca_13099	INTRON	LIM binding protein
Kabuli_wild_38680371	1399937730	*Ca_kabuli_Chr04*	38680371	G/A	Gene_Ca_13099	STOP_GAINED	LIM binding protein
Kabuli_wild_38721154	1399937731	*Ca_kabuli_Chr04*	38721154	A/G	Gene_Ca_13098	INTRON	Exostosin-like
Kabuli_wild_38721160	1399937732	*Ca_kabuli_Chr04*	38721160	A/G	Gene_Ca_13098	INTRON	Exostosin-like
Kabuli_wild_38721207	1399937733	*Ca_kabuli_Chr04*	38721207	A/C	Gene_Ca_13098	INTRON	Exostosin-like
Kabuli_wild_38756507	1399937734	*Ca_kabuli_Chr04*	38756507	C/T	Gene_Ca_13097	SYNONYMOUS_CODING	Auxin efflux carrier
Kabuli_wild_38756510	1399937735	*Ca_kabuli_Chr04*	38756510	C/G	Gene_Ca_13097	NON_SYNONYMOUS_CODING	Auxin efflux carrier
Kabuli_wild_38759640	1399937736	*Ca_kabuli_Chr04*	38759640	A/C	Gene_Ca_13097	INTRON	Auxin efflux carrier
Kabuli_wild_38770348	1399937737	*Ca_kabuli_Chr04*	38770348	T/A	Gene_Ca_13096	NON_SYNONYMOUS_CODING	Fattyaciddesaturase,type2
Kabuli_wild_38770370	1399937738	*Ca_kabuli_Chr04*	38770370	G/C	Gene_Ca_13096	NON_SYNONYMOUS_CODING	Fattyaciddesaturase,type2
Kabuli_wild_38770450	1399937739	*Ca_kabuli_Chr04*	38770450	C/G	Gene_Ca_13096	SYNONYMOUS_CODING	Fattyaciddesaturase,type2
Kabuli_wild_38770510	1399937740	*Ca_kabuli_Chr04*	38770510	G/A	Gene_Ca_13096	SYNONYMOUS_CODING	Fattyaciddesaturase,type2
Kabuli_wild_38770582	1399937741	*Ca_kabuli_Chr04*	38770582	G/A	Gene_Ca_13096	SYNONYMOUS_CODING	Fattyaciddesaturase,type2
Kabuli_wild_38770606	1399937742	*Ca_kabuli_Chr04*	38770606	G/A	Gene_Ca_13096	SYNONYMOUS_CODING	Fattyaciddesaturase,type2
Kabuli_wild_38785535	1399937743	*Ca_kabuli_Chr04*	38785535	G/A	NA	INTERGENIC	NA

SNP IDs	NCBI dbSNP accession IDs (NCBI_SS#)	Chromosomes	Physical positions (bp)	SNPs	Gene accession IDs	Sequence components of genome	Putative functions
Kabuli_wild_38829959	1399937744	*Ca_kabuli_Chr04*	38829959	T/G	NA	INTERGENIC	NA
Kabuli_wild_38831427	1399937745	*Ca_kabuli_Chr04*	38831427	C/T	Gene_Ca_13091	DOWNSTREAM	NA
Kabuli_wild_38833280	1399937746	*Ca_kabuli_Chr04*	38833280	C/T	Gene_Ca_13092	DOWNSTREAM	Ribosomalbiogenesis,methyltransferase,EMG1/NEP1
Kabuli_wild_38833304	1399937747	*Ca_kabuli_Chr04*	38833304	C/A	Gene_Ca_13092	DOWNSTREAM	Ribosomalbiogenesis,methyltransferase,EMG1/NEP2
Kabuli_wild_38833333	1399937748	*Ca_kabuli_Chr04*	38833333	A/C	Gene_Ca_13092	DOWNSTREAM	Ribosomalbiogenesis,methyltransferase,EMG1/NEP3
Kabuli_wild_38833521	1399937749	*Ca_kabuli_Chr04*	38833521	G/A	Gene_Ca_13092	DOWNSTREAM	Ribosomalbiogenesis,methyltransferase,EMG1/NEP4
Kabuli_wild_38833525	1399937750	*Ca_kabuli_Chr04*	38833525	A/C	Gene_Ca_13092	DOWNSTREAM	Ribosomalbiogenesis,methyltransferase,EMG1/NEP5
Kabuli_wild_38833535	1399937751	*Ca_kabuli_Chr04*	38833535	A/G	NA	INTERGENIC	NA
Kabuli_wild_38833778	1399937752	*Ca_kabuli_Chr04*	38833778	T/C	NA	INTERGENIC	NA
Kabuli_wild_38833779	1399937753	*Ca_kabuli_Chr04*	38833779	T/A	NA	INTERGENIC	NA
Kabuli_wild_38833810	1399937754	*Ca_kabuli_Chr04*	38833810	A/G	NA	INTERGENIC	NA
Kabuli_wild_38833843	1399937755	*Ca_kabuli_Chr04*	38833843	A/T	NA	INTERGENIC	NA
Kabuli_wild_38833859	1399937756	*Ca_kabuli_Chr04*	38833859	C/G	NA	INTERGENIC	NA
Kabuli_wild_38834383	1399937757	*Ca_kabuli_Chr04*	38834383	G/A	NA	INTERGENIC	NA
Kabuli_wild_38834405	1399937758	*Ca_kabuli_Chr04*	38834405	G/A	NA	INTERGENIC	NA
Kabuli_wild_38834423	1399937759	*Ca_kabuli_Chr04*	38834423	G/C	NA	INTERGENIC	NA
Kabuli_wild_38834431	1399937760	*Ca_kabuli_Chr04*	38834431	G/A	NA	INTERGENIC	NA
Kabuli_wild_38834474	1399937761	*Ca_kabuli_Chr04*	38834474	G/A	NA	INTERGENIC	NA
Kabuli_wild_38834477	1399937762	*Ca_kabuli_Chr04*	38834477	A/G	NA	INTERGENIC	NA
Kabuli_wild_38836356	1399937763	*Ca_kabuli_Chr04*	38836356	T/C	NA	INTERGENIC	NA
Kabuli_wild_38836381	1399937764	*Ca_kabuli_Chr04*	38836381	T/A	NA	INTERGENIC	NA
Kabuli_wild_38836398	1399937765	*Ca_kabuli_Chr04*	38836398	C/T	NA	INTERGENIC	NA
Kabuli_wild_38836436	1399937766	*Ca_kabuli_Chr04*	38836436	G/T	NA	INTERGENIC	NA
Kabuli_wild_38836459	1399937767	*Ca_kabuli_Chr04*	38836459	T/C	NA	INTERGENIC	NA
Kabuli_wild_38836488	1399937768	*Ca_kabuli_Chr04*	38836488	A/G	NA	INTERGENIC	NA
Kabuli_wild_38836649	1399937769	*Ca_kabuli_Chr04*	38836649	C/T	Gene_Ca_13091	SYNONYMOUS_CODING	NA
Kabuli_wild_38888779	1399937770	*Ca_kabuli_Chr04*	38888779	C/T	Gene_Ca_13087	SYNONYMOUS_CODING	Protein kinase,catalytic domain
Kabuli_wild_38888791	1399937771	*Ca_kabuli_Chr04*	38888791	G/C	Gene_Ca_13087	SYNONYMOUS_CODING	Protein kinase,catalytic domain
Kabuli_wild_38889023	1399937772	*Ca_kabuli_Chr04*	38889023	C/T	Gene_Ca_13087	NON_SYNONYMOUS_CODING	Protein kinase,catalytic domain
Kabuli_wild_38918394	1399937773	*Ca_kabuli_Chr04*	38918394	A/T	Gene_Ca_13086	SYNONYMOUS_CODING	General substrate transporter
Kabuli_wild_38918396	1399937774	*Ca_kabuli_Chr04*	38918396	T/C	Gene_Ca_13086	NON_SYNONYMOUS_CODING	General substrate transporter
Kabuli_wild_38918424	1399937775	*Ca_kabuli_Chr04*	38918424	G/A	Gene_Ca_13086	SYNONYMOUS_CODING	General substrate transporter
Kabuli_wild_38918430	1399937776	*Ca_kabuli_Chr04*	38918430	C/A	Gene_Ca_13086	SYNONYMOUS_CODING	General substrate transporter
Kabuli_wild_38918541	1399937777	*Ca_kabuli_Chr04*	38918541	T/C	Gene_Ca_13086	SYNONYMOUS_CODING	General substrate transporter
Kabuli_wild_38918547	1399937778	*Ca_kabuli_Chr04*	38918547	C/T	Gene_Ca_13086	SYNONYMOUS_CODING	General substrate transporter
Kabuli_wild_38918628	1399937779	*Ca_kabuli_Chr04*	38918628	C/T	Gene_Ca_13086	SYNONYMOUS_CODING	General substrate transporter
Kabuli_wild_38918649	1399937780	*Ca_kabuli_Chr04*	38918649	G/C	Gene_Ca_13086	SYNONYMOUS_CODING	General substrate transporter
Kabuli_wild_38918724	1399937781	*Ca_kabuli_Chr04*	38918724	A/C	Gene_Ca_13086	SYNONYMOUS_CODING	General substrate transporter
Kabuli_wild_38921338	1399937782	*Ca_kabuli_Chr04*	38921338	C/T	Gene_Ca_13086	DOWNSTREAM	General substrate transporter
Kabuli_wild_38921373	1399937783	*Ca_kabuli_Chr04*	38921373	G/T	Gene_Ca_13086	DOWNSTREAM	General substrate transporter
Kabuli_wild_38921378	1399937784	*Ca_kabuli_Chr04*	38921378	A/G	Gene_Ca_13086	DOWNSTREAM	General substrate transporter
Kabuli_wild_38921411	1399937785	*Ca_kabuli_Chr04*	38921411	T/C	Gene_Ca_13086	DOWNSTREAM	General substrate transporter
Kabuli_wild_38921413	1399937786	*Ca_kabuli_Chr04*	38921413	G/A	Gene_Ca_13086	DOWNSTREAM	General substrate transporter
Kabuli_wild_38921434	1399937787	*Ca_kabuli_Chr04*	38921434	T/C	Gene_Ca_13086	DOWNSTREAM	General substrate transporter

SNP IDs	NCBI dbSNP accession IDs (NCBI_SS#)	Chromosomes	Physical positions (bp)	SNPs	Gene accession IDs	Sequence components of genome	Putative functions
Kabuli_wild_38921589	1399937788	*Ca_kabuli_Chr04*	38921589	C/T	NA	INTERGENIC	NA
Kabuli_wild_38921614	1399937789	*Ca_kabuli_Chr04*	38921614	G/T	NA	INTERGENIC	NA
Kabuli_wild_38921615	1399937790	*Ca_kabuli_Chr04*	38921615	A/C	NA	INTERGENIC	NA
Kabuli_wild_38921628	1399937791	*Ca_kabuli_Chr04*	38921628	T/A	NA	INTERGENIC	NA
Kabuli_wild_38921678	1399937792	*Ca_kabuli_Chr04*	38921678	G/A	NA	INTERGENIC	NA
Kabuli_wild_38921685	1399937793	*Ca_kabuli_Chr04*	38921685	A/G	NA	INTERGENIC	NA
Kabuli_wild_38921721	1399937794	*Ca_kabuli_Chr04*	38921721	G/A	NA	INTERGENIC	NA
Kabuli_wild_38921728	1399937795	*Ca_kabuli_Chr04*	38921728	A/T	NA	INTERGENIC	NA
Kabuli_wild_38929681	1399937796	*Ca_kabuli_Chr04*	38929681	A/G	Gene_Ca_13085	SYNONYMOUS_CODING	NA
Kabuli_wild_38948414	1399937797	*Ca_kabuli_Chr04*	38948414	C/A	Gene_Ca_13084	NON_SYNONYMOUS_CODING	Pathogenesis-related transcriptional factor/ERF, DNA-binding
Kabuli_wild_38961521	1399937798	*Ca_kabuli_Chr04*	38961521	G/A	Gene_Ca_13083	INTRON	Ubiquitin-conjugating enzyme, E2
Kabuli_wild_38976822	1399937799	*Ca_kabuli_Chr04*	38976822	C/A	Gene_Ca_13081	INTRON	Folylpolyglutamatesynthetase
Kabuli_wild_38976835	1399937800	*Ca_kabuli_Chr04*	38976835	G/T	Gene_Ca_13081	INTRON	Folylpolyglutamatesynthetase
Kabuli_wild_38976865	1399937801	*Ca_kabuli_Chr04*	38976865	T/C	Gene_Ca_13081	INTRON	Folylpolyglutamatesynthetase
Kabuli_wild_38978462	1399937802	*Ca_kabuli_Chr04*	38978462	A/G	Gene_Ca_13081	SYNONYMOUS_CODING	Folylpolyglutamatesynthetase
Kabuli_wild_38979193	1399937803	*Ca_kabuli_Chr04*	38979193	G/T	Gene_Ca_13081	INTRON	Folylpolyglutamatesynthetase
Kabuli_wild_38979228	1399937804	*Ca_kabuli_Chr04*	38979228	G/T	Gene_Ca_13081	INTRON	Folylpolyglutamatesynthetase
Kabuli_wild_38979508	1399937805	*Ca_kabuli_Chr04*	38979508	G/A	Gene_Ca_13081	INTRON	Folylpolyglutamatesynthetase
Kabuli_wild_39031801	1399937806	*Ca_kabuli_Chr04*	39031801	C/T	Gene_Ca_13078	NON_SYNONYMOUS_CODING	RNA recognition motif domain
Kabuli_wild_39031823	1399937807	*Ca_kabuli_Chr04*	39031823	G/A	Gene_Ca_13078	SYNONYMOUS_CODING	RNA recognition motif domain
Kabuli_wild_39031970	1399937808	*Ca_kabuli_Chr04*	39031970	T/G	Gene_Ca_13078	SYNONYMOUS_CODING	RNA recognition motif domain
Kabuli_wild_39032138	1399937809	*Ca_kabuli_Chr04*	39032138	A/T	Gene_Ca_13078	INTRON	RNA recognition motif domain
Kabuli_wild_39033052	1399937810	*Ca_kabuli_Chr04*	39033052	A/G	Gene_Ca_13078	INTRON	RNA recognition motif domain
Kabuli_wild_39033073	1399937811	*Ca_kabuli_Chr04*	39033073	G/C	Gene_Ca_13078	INTRON	RNA recognition motif domain
Kabuli_wild_39033838	1399937812	*Ca_kabuli_Chr04*	39033838	C/A	Gene_Ca_13078	INTRON	RNA recognition motif domain
Kabuli_wild_39042921	1399937813	*Ca_kabuli_Chr04*	39042921	T/C	Gene_Ca_13077	INTRON	PeptidaseM41
Kabuli_wild_39042938	1399937814	*Ca_kabuli_Chr04*	39042938	T/G	Gene_Ca_13077	INTRON	PeptidaseM41
Kabuli_wild_39057991	1399937815	*Ca_kabuli_Chr04*	39057991	T/C	NA	INTERGENIC	NA
Kabuli_wild_39058011	1399937816	*Ca_kabuli_Chr04*	39058011	G/C	NA	INTERGENIC	NA
Kabuli_wild_39081951	1399937817	*Ca_kabuli_Chr04*	39081951	A/G	Gene_Ca_13074	INTRON	Cyclicnucleotide-binding domain
Kabuli_wild_39086017	1399937818	*Ca_kabuli_Chr04*	39086017	T/A	Gene_Ca_13074	DOWNSTREAM	Cyclicnucleotide-binding domain
Kabuli_wild_39086269	1399937819	*Ca_kabuli_Chr04*	39086269	A/G	Gene_Ca_13074	DOWNSTREAM	Cyclicnucleotide-binding domain
Kabuli_wild_39110473	1399937820	*Ca_kabuli_Chr04*	39110473	T/A	Gene_Ca_13071	DOWNSTREAM	WD40 repeat
Kabuli_wild_39113327	1399937821	*Ca_kabuli_Chr04*	39113327	G/A	NA	INTERGENIC	NA
Kabuli_wild_39113330	1399937822	*Ca_kabuli_Chr04*	39113330	G/A	NA	INTERGENIC	NA
Kabuli_wild_39113401	1399937823	*Ca_kabuli_Chr04*	39113401	A/T	NA	INTERGENIC	NA
Kabuli_wild_39116656	1399937824	*Ca_kabuli_Chr04*	39116656	G/A	Gene_Ca_13071	UPSTREAM	WD40 repeat
Kabuli_wild_39116795	1399937825	*Ca_kabuli_Chr04*	39116795	G/C	Gene_Ca_13071	UPSTREAM	WD40 repeat
Kabuli_wild_39136826	1399937826	*Ca_kabuli_Chr04*	39136826	C/T	Gene_Ca_13069	DOWNSTREAM	Protein phosphatase 2C, manganese/magnesium aspartate binding site
Kabuli_wild_39268707	1399937827	*Ca_kabuli_Chr04*	39268707	A/C	NA	INTERGENIC	NA
Kabuli_wild_39268770	1399937828	*Ca_kabuli_Chr04*	39268770	C/G	NA	INTERGENIC	NA
Kabuli_wild_39306891	1399937829	*Ca_kabuli_Chr04*	39306891	G/A	NA	INTERGENIC	NA
Kabuli_wild_39306900	1399937830	*Ca_kabuli_Chr04*	39306900	G/A	NA	INTERGENIC	NA
Kabuli_wild_39306915	1399937831	*Ca_kabuli_Chr04*	39306915	G/T	NA	INTERGENIC	NA

SNP IDs	NCBI dbSNP accession IDs (NCBI_SS#)	Chromosomes	Physical positions (bp)	SNPs	Gene accession IDs	Sequence components of genome	Putative functions
Kabuli_wild_39306938	1399937832	*Ca_kabuli_Chr04*	39306938	T/C	NA	INTERGENIC	NA
Kabuli_wild_39379990	1399937833	*Ca_kabuli_Chr04*	39379990	T/C	NA	INTERGENIC	NA
Kabuli_wild_39380084	1399937834	*Ca_kabuli_Chr04*	39380084	T/C	NA	INTERGENIC	NA
Kabuli_wild_39380114	1399937835	*Ca_kabuli_Chr04*	39380114	G/T	NA	INTERGENIC	NA
Kabuli_wild_39635683	1399937836	*Ca_kabuli_Chr04*	39635683	G/A	NA	INTERGENIC	NA
Kabuli_wild_39635718	1399937837	*Ca_kabuli_Chr04*	39635718	C/T	NA	INTERGENIC	NA
Kabuli_wild_39635720	1399937838	*Ca_kabuli_Chr04*	39635720	G/A	NA	INTERGENIC	NA
Kabuli_wild_39635721	1399937839	*Ca_kabuli_Chr04*	39635721	A/G	NA	INTERGENIC	NA
Kabuli_wild_39635742	1399937840	*Ca_kabuli_Chr04*	39635742	A/T	NA	INTERGENIC	NA
Kabuli_wild_39635746	1399937841	*Ca_kabuli_Chr04*	39635746	A/T	NA	INTERGENIC	NA
Kabuli_wild_39650872	1399937842	*Ca_kabuli_Chr04*	39650872	T/C	NA	INTERGENIC	NA
Kabuli_wild_39659750	1399937843	*Ca_kabuli_Chr04*	39659750	G/A	Gene_Ca_14935	DOWNSTREAM	Protein kinase,catalytic domain
Kabuli_wild_39659757	1399937844	*Ca_kabuli_Chr04*	39659757	C/T	Gene_Ca_14935	DOWNSTREAM	Protein kinase,catalytic domain
Kabuli_wild_39715087	1399937845	*Ca_kabuli_Chr04*	39715087	A/G	Gene_Ca_14929	SYNONYMOUS_CODING	Glutamineamidotransferase,class-II
Kabuli_wild_39719606	1399937846	*Ca_kabuli_Chr04*	39719606	C/G	Gene_Ca_14929	SYNONYMOUS_CODING	Glutamineamidotransferase,class-II
Kabuli_wild_39719747	1399937847	*Ca_kabuli_Chr04*	39719747	G/T	Gene_Ca_14929	SYNONYMOUS_CODING	Glutamineamidotransferase,class-II
Kabuli_wild_39779563	1399937848	*Ca_kabuli_Chr04*	39779563	G/A	Gene_Ca_14927	NON_SYNONYMOUS_CODING	Protein of unknown function DUF81
Kabuli_wild_39803305	1399937849	*Ca_kabuli_Chr04*	39803305	T/C	Gene_Ca_14925	SYNONYMOUS_CODING	SANT domain,DNA binding
Kabuli_wild_39964930	1399937850	*Ca_kabuli_Chr04*	39964930	T/C	Gene_Ca_14914	SYNONYMOUS_CODING	Exostosin-like
Kabuli_wild_40010119	1399937851	*Ca_kabuli_Chr04*	40010119	A/C	Gene_Ca_14911	NON_SYNONYMOUS_CODING	Pathogenesis-related transcriptional factor/ERF, DNA-binding
Kabuli_wild_40010165	1399937852	*Ca_kabuli_Chr04*	40010165	A/C	Gene_Ca_14911	NON_SYNONYMOUS_CODING	Pathogenesis-related transcriptional factor/ERF, DNA-binding
Kabuli_wild_40010177	1399937853	*Ca_kabuli_Chr04*	40010177	G/A	Gene_Ca_14911	SYNONYMOUS_CODING	Pathogenesis-related transcriptional factor/ERF, DNA-binding
Kabuli_wild_40010201	1399937854	*Ca_kabuli_Chr04*	40010201	A/G	Gene_Ca_14911	SYNONYMOUS_CODING	Pathogenesis-related transcriptional factor/ERF, DNA-binding
Kabuli_wild_40060063	1399937855	*Ca_kabuli_Chr04*	40060063	G/T	Gene_Ca_14910	SYNONYMOUS_CODING	Nuclearpore complex protein, Nucleoporin Nup85-like
Kabuli_wild_40061195	1399937856	*Ca_kabuli_Chr04*	40061195	T/C	Gene_Ca_14910	INTRON	Nuclearpore complex protein, Nucleoporin Nup85-like
Kabuli_wild_40061262	1399937857	*Ca_kabuli_Chr04*	40061262	A/C	Gene_Ca_14910	INTRON	Nuclearpore complex protein, Nucleoporin Nup85-like
Kabuli_wild_40065505	1399937858	*Ca_kabuli_Chr04*	40065505	T/A	Gene_Ca_14910	INTRON	Nuclearpore complex protein, Nucleoporin Nup85-like
Kabuli_wild_40134460	1399937859	*Ca_kabuli_Chr04*	40134460	A/G	Gene_Ca_14906	INTRON	Exoribonuclease,phosphorolytic domain1
Kabuli_wild_40134518	1399937860	*Ca_kabuli_Chr04*	40134518	T/C	Gene_Ca_14906	INTRON	Exoribonuclease,phosphorolytic domain1
Kabuli_wild_40149132	1399937861	*Ca_kabuli_Chr04*	40149132	G/C	Gene_Ca_14905	INTRON	NA
Kabuli_wild_40149324	1399937862	*Ca_kabuli_Chr04*	40149324	G/C	Gene_Ca_14905	INTRON	NA
Kabuli_wild_40166667	1399937863	*Ca_kabuli_Chr04*	40166667	G/C	Gene_Ca_14903	NON_SYNONYMOUS_CODING	PeptidaseS16,lonN-terminal
Kabuli_wild_40169176	1399937864	*Ca_kabuli_Chr04*	40169176	T/G	Gene_Ca_14903	UPSTREAM	PeptidaseS16,lonN-terminal
Kabuli_wild_40185606	1399937865	*Ca_kabuli_Chr04*	40185606	A/G	Gene_Ca_14901	NON_SYNONYMOUS_CODING	Aminoacid transporter,transmembrane
Kabuli_wild_40185699	1399937866	*Ca_kabuli_Chr04*	40185699	G/C	Gene_Ca_14901	INTRON	Aminoacid transporter,transmembrane
Kabuli_wild_40199692	1399937867	*Ca_kabuli_Chr04*	40199692	A/C	Gene_Ca_14900	INTRON	Cofprotein
Kabuli_wild_40218375	1399937868	*Ca_kabuli_Chr04*	40218375	G/T	NA	INTERGENIC	NA
Kabuli_wild_40218410	1399937869	*Ca_kabuli_Chr04*	40218410	G/A	NA	INTERGENIC	NA
Kabuli_wild_40267713	1399937870	*Ca_kabuli_Chr04*	40267713	G/T	NA	INTERGENIC	NA
Kabuli_wild_40267719	1399937871	*Ca_kabuli_Chr04*	40267719	G/A	NA	INTERGENIC	NA
Kabuli_wild_40279513	1399937872	*Ca_kabuli_Chr04*	40279513	G/T	NA	INTERGENIC	NA
Kabuli_wild_40279578	1399937873	*Ca_kabuli_Chr04*	40279578	G/C	NA	INTERGENIC	NA
Kabuli_wild_40313721	1399937874	*Ca_kabuli_Chr04*	40313721	A/G	NA	INTERGENIC	NA
Kabuli_wild_40349170	1399937875	*Ca_kabuli_Chr04*	40349170	C/T	Gene_Ca_14888	UPSTREAM	RibosomalproteinL28

SNP IDs	NCBI dbSNP accession IDs (NCBI_SS#)	Chromosomes	Physical positions (bp)	SNPs	Gene accession IDs	Sequence components of genome	Putative functions
Kabuli_wild_40368278	1399937876	*Ca_kabuli_Chr04*	40368278	T/C	NA	INTERGENIC	NA
Kabuli_wild_40368765	1399937877	*Ca_kabuli_Chr04*	40368765	G/A	NA	INTERGENIC	NA
Kabuli_wild_40375361	1399937878	*Ca_kabuli_Chr04*	40375361	A/G	NA	INTERGENIC	NA
Kabuli_wild_40375386	1399937879	*Ca_kabuli_Chr04*	40375386	C/T	NA	INTERGENIC	NA
Kabuli_wild_40387959	1399937880	*Ca_kabuli_Chr04*	40387959	A/G	NA	INTERGENIC	NA
Kabuli_wild_40429387	1399937881	*Ca_kabuli_Chr04*	40429387	G/A	NA	INTERGENIC	NA
Kabuli_wild_40471412	1399937882	*Ca_kabuli_Chr04*	40471412	G/C	NA	INTERGENIC	NA
Kabuli_wild_40472097	1399937883	*Ca_kabuli_Chr04*	40472097	A/G	NA	INTERGENIC	NA
Kabuli_wild_40481316	1399937884	*Ca_kabuli_Chr04*	40481316	G/T	Gene_Ca_14877	SYNONYMOUS_CODING	Alpha/betahydrolasefold-1
Kabuli_wild_40481332	1399937885	*Ca_kabuli_Chr04*	40481332	G/T	Gene_Ca_14877	SYNONYMOUS_CODING	Alpha/betahydrolasefold-1
Kabuli_wild_40513733	1399937886	*Ca_kabuli_Chr04*	40513733	C/T	Gene_Ca_14874	SYNONYMOUS_CODING	IQ motif,EF-handbinding site
Kabuli_wild_40544727	1399937887	*Ca_kabuli_Chr04*	40544727	C/T	NA	INTERGENIC	NA
Kabuli_wild_40554370	1399937888	*Ca_kabuli_Chr04*	40554370	G/A	Gene_Ca_14870	NON_SYNONYMOUS_CODING	NA
Kabuli_wild_40583205	1399937889	*Ca_kabuli_Chr04*	40583205	G/T	Gene_Ca_14868	STOP_GAINED	Zinc finger, RING-type
Kabuli_wild_40583206	1399937890	*Ca_kabuli_Chr04*	40583206	A/T	Gene_Ca_14868	NON_SYNONYMOUS_CODING	Zinc finger, RING-type
Kabuli_wild_40583211	1399937891	*Ca_kabuli_Chr04*	40583211	G/T	Gene_Ca_14868	STOP_GAINED	Zinc finger, RING-type
Kabuli_wild_40583214	1399937892	*Ca_kabuli_Chr04*	40583214	G/A	Gene_Ca_14868	NON_SYNONYMOUS_CODING	Zinc finger, RING-type
Kabuli_wild_40583216	1399937893	*Ca_kabuli_Chr04*	40583216	C/A	Gene_Ca_14868	NON_SYNONYMOUS_CODING	Zinc finger, RING-type
Kabuli_wild_40583217	1399937894	*Ca_kabuli_Chr04*	40583217	G/A	Gene_Ca_14868	NON_SYNONYMOUS_CODING	Zinc finger, RING-type
Kabuli_wild_40583219	1399937895	*Ca_kabuli_Chr04*	40583219	G/A	Gene_Ca_14868	SYNONYMOUS_CODING	Zinc finger, RING-type
Kabuli_wild_40594404	1399937896	*Ca_kabuli_Chr04*	40594404	G/A	NA	INTERGENIC	NA
Kabuli_wild_40594445	1399937897	*Ca_kabuli_Chr04*	40594445	A/T	NA	INTERGENIC	NA
Kabuli_wild_40594470	1399937898	*Ca_kabuli_Chr04*	40594470	A/C	NA	INTERGENIC	NA
Kabuli_wild_40597192	1399937899	*Ca_kabuli_Chr04*	40597192	G/A	NA	INTERGENIC	NA
Kabuli_wild_40597464	1399937900	*Ca_kabuli_Chr04*	40597464	G/A	NA	INTERGENIC	NA
Kabuli_wild_40597534	1399937901	*Ca_kabuli_Chr04*	40597534	A/G	NA	INTERGENIC	NA
Kabuli_wild_40705818	1399937902	*Ca_kabuli_Chr04*	40705818	C/A	NA	INTERGENIC	NA
Kabuli_wild_40723208	1399937903	*Ca_kabuli_Chr04*	40723208	C/G	NA	INTERGENIC	NA
Kabuli_wild_40723212	1399937904	*Ca_kabuli_Chr04*	40723212	G/A	NA	INTERGENIC	NA
Kabuli_wild_40734572	1399937905	*Ca_kabuli_Chr04*	40734572	T/G	Gene_Ca_23909	INTRON	PeptidaseS54,rhomboid
Kabuli_wild_40734765	1399937906	*Ca_kabuli_Chr04*	40734765	G/A	Gene_Ca_23909	SYNONYMOUS_CODING	PeptidaseS54,rhomboid
Kabuli_wild_40738927	1399937907	*Ca_kabuli_Chr04*	40738927	C/T	Gene_Ca_23908	SYNONYMOUS_CODING	Pentatrico peptide repeat
Kabuli_wild_40795060	1399937908	*Ca_kabuli_Chr04*	40795060	G/A	Gene_Ca_23903	DOWNSTREAM	BURP
Kabuli_wild_40795085	1399937909	*Ca_kabuli_Chr04*	40795085	C/G	Gene_Ca_23903	DOWNSTREAM	BURP
Kabuli_wild_40800814	1399937910	*Ca_kabuli_Chr04*	40800814	A/G	Gene_Ca_23902	SYNONYMOUS_CODING	DNA-directed DNA polymerase,family A, palm domain
Kabuli_wild_40801783	1399937911	*Ca_kabuli_Chr04*	40801783	T/C	Gene_Ca_23902	SYNONYMOUS_CODING	DNA-directed DNA polymerase,family A, palm domain
Kabuli_wild_40801790	1399937912	*Ca_kabuli_Chr04*	40801790	T/C	Gene_Ca_23902	NON_SYNONYMOUS_CODING	DNA-directed DNA polymerase,family A, palm domain
Kabuli_wild_40801791	1399937913	*Ca_kabuli_Chr04*	40801791	C/G	Gene_Ca_23902	NON_SYNONYMOUS_CODING	DNA-directed DNA polymerase,family A, palm domain
Kabuli_wild_40805740	1399937914	*Ca_kabuli_Chr04*	40805740	C/A	Gene_Ca_23902	SYNONYMOUS_CODING	DNA-directed DNA polymerase,family A, palm domain
Kabuli_wild_40805952	1399937915	*Ca_kabuli_Chr04*	40805952	A/C	Gene_Ca_23902	NON_SYNONYMOUS_CODING	DNA-directed DNA polymerase,family A, palm domain
Kabuli_wild_40806993	1399937916	*Ca_kabuli_Chr04*	40806993	A/G	Gene_Ca_23902	SYNONYMOUS_CODING	DNA-directed DNA polymerase,family A, palm domain
Kabuli_wild_40806995	1399937917	*Ca_kabuli_Chr04*	40806995	C/T	Gene_Ca_23902	SYNONYMOUS_CODING	DNA-directed DNA polymerase,family A, palm domain
Kabuli_wild_40807053	1399937918	*Ca_kabuli_Chr04*	40807053	G/T	Gene_Ca_23902	SYNONYMOUS_CODING	DNA-directed DNA polymerase,family A, palm domain
Kabuli_wild_40839949	1399937919	*Ca_kabuli_Chr04*	40839949	G/A	NA	INTERGENIC	NA

SNP IDs	NCBI dbSNP accession IDs (NCBI_SS#)	Chromosomes	Physical positions (bp)	SNPs	Gene accession IDs	Sequence components of genome	Putative functions
Kabuli_wild_40839967	1399937920	*Ca_kabuli_Chr04*	40839967	G/A	NA	INTERGENIC	NA
Kabuli_wild_40852381	1399937921	*Ca_kabuli_Chr04*	40852381	C/A	Gene_Ca_23897	SYNONYMOUS_CODING	Globin, truncated bacterial-like
Kabuli_wild_40852475	1399937922	*Ca_kabuli_Chr04*	40852475	T/C	Gene_Ca_23897	INTRON	Globin, truncated bacterial-like
Kabuli_wild_40912528	1399937923	*Ca_kabuli_Chr04*	40912528	T/C	Gene_Ca_11017	UPSTREAM	Beta-lactamase-related
Kabuli_wild_40912537	1399937924	*Ca_kabuli_Chr04*	40912537	G/T	Gene_Ca_11017	UPSTREAM	Beta-lactamase-related
Kabuli_wild_40912677	1399937925	*Ca_kabuli_Chr04*	40912677	A/G	Gene_Ca_11017	UPSTREAM	Beta-lactamase-related
Kabuli_wild_40912681	1399937926	*Ca_kabuli_Chr04*	40912681	A/G	Gene_Ca_11017	UPSTREAM	Beta-lactamase-related
Kabuli_wild_40912714	1399937927	*Ca_kabuli_Chr04*	40912714	C/G	Gene_Ca_11017	UPSTREAM	Beta-lactamase-related
Kabuli_wild_40912731	1399937928	*Ca_kabuli_Chr04*	40912731	C/T	Gene_Ca_11017	UPSTREAM	Beta-lactamase-related
Kabuli_wild_40917776	1399937929	*Ca_kabuli_Chr04*	40917776	C/A	NA	INTERGENIC	NA
Kabuli_wild_40917777	1399937930	*Ca_kabuli_Chr04*	40917777	T/A	NA	INTERGENIC	NA
Kabuli_wild_40917807	1399937931	*Ca_kabuli_Chr04*	40917807	C/T	NA	INTERGENIC	NA
Kabuli_wild_40941283	1399937932	*Ca_kabuli_Chr04*	40941283	A/G	Gene_Ca_11014	SYNONYMOUS_CODING	Proteasome component (PCI) domain
Kabuli_wild_41032564	1399937933	*Ca_kabuli_Chr04*	41032564	A/G	Gene_Ca_11007	INTRON	GHMPkinase
Kabuli_wild_41032590	1399937934	*Ca_kabuli_Chr04*	41032590	C/A	Gene_Ca_11007	INTRON	GHMPkinase
Kabuli_wild_41032599	1399937935	*Ca_kabuli_Chr04*	41032599	T/C	Gene_Ca_11007	INTRON	GHMPkinase
Kabuli_wild_41094082	1399937936	*Ca_kabuli_Chr04*	41094082	T/C	Gene_Ca_10999	NON_SYNONYMOUS_CODING	Protein kinase,catalytic domain
Kabuli_wild_41094554	1399937937	*Ca_kabuli_Chr04*	41094554	A/C	Gene_Ca_10999	INTRON	Protein kinase,catalytic domain
Kabuli_wild_41117822	1399937938	*Ca_kabuli_Chr04*	41117822	C/A	Gene_Ca_10997	INTRON	DNAtopoisomerase,typeIA
Kabuli_wild_41140101	1399937939	*Ca_kabuli_Chr04*	41140101	T/C	Gene_Ca_10994	NON_SYNONYMOUS_CODING	NA
Kabuli_wild_41141086	1399937940	*Ca_kabuli_Chr04*	41141086	G/T	Gene_Ca_10994	NON_SYNONYMOUS_CODING	NA
Kabuli_wild_41142288	1399937941	*Ca_kabuli_Chr04*	41142288	T/C	Gene_Ca_10994	NON_SYNONYMOUS_CODING	NA
Kabuli_wild_41142290	1399937942	*Ca_kabuli_Chr04*	41142290	A/G	Gene_Ca_10994	SYNONYMOUS_CODING	NA
Kabuli_wild_41142299	1399937943	*Ca_kabuli_Chr04*	41142299	T/C	Gene_Ca_10994	SYNONYMOUS_CODING	NA
Kabuli_wild_41144277	1399937944	*Ca_kabuli_Chr04*	41144277	T/A	Gene_Ca_10994	NON_SYNONYMOUS_CODING	NA
Kabuli_wild_41144411	1399937945	*Ca_kabuli_Chr04*	41144411	A/G	Gene_Ca_10994	NON_SYNONYMOUS_CODING	NA
Kabuli_wild_41145767	1399937946	*Ca_kabuli_Chr04*	41145767	C/T	Gene_Ca_10994	SYNONYMOUS_CODING	NA
Kabuli_wild_41145875	1399937947	*Ca_kabuli_Chr04*	41145875	G/A	Gene_Ca_10994	SYNONYMOUS_CODING	NA
Kabuli_wild_41207354	1399937948	*Ca_kabuli_Chr04*	41207354	A/C	Gene_Ca_10990	INTRON	Cystathioninebeta-synthase,core
Kabuli_wild_41207356	1399937949	*Ca_kabuli_Chr04*	41207356	C/T	Gene_Ca_10990	INTRON	Cystathioninebeta-synthase,core
Kabuli_wild_41209697	1399937950	*Ca_kabuli_Chr04*	41209697	C/A	Gene_Ca_10990	INTRON	Cystathioninebeta-synthase,core
Kabuli_wild_41209711	1399937951	*Ca_kabuli_Chr04*	41209711	A/C	Gene_Ca_10990	INTRON	Cystathioninebeta-synthase,core
Kabuli_wild_41215850	1399937952	*Ca_kabuli_Chr04*	41215850	A/C	NA	INTERGENIC	NA
Kabuli_wild_41229605	1399937953	*Ca_kabuli_Chr04*	41229605	C/T	NA	INTERGENIC	NA
Kabuli_wild_41264870	1399937954	*Ca_kabuli_Chr04*	41264870	A/T	Gene_Ca_10983	NON_SYNONYMOUS_CODING	Pentatrico peptide repeat
Kabuli_wild_41292152	1399937955	*Ca_kabuli_Chr04*	41292152	T/C	Gene_Ca_10980	NON_SYNONYMOUS_CODING	ATPase, P-type, H+ transporting proton pump
Kabuli_wild_41292870	1399937956	*Ca_kabuli_Chr04*	41292870	A/C	Gene_Ca_10980	NON_SYNONYMOUS_CODING	ATPase, P-type, H+ transporting proton pump
Kabuli_wild_41427086	1399937957	*Ca_kabuli_Chr04*	41427086	C/A	Gene_Ca_10964	UPSTREAM	Pentatrico peptide repeat
Kabuli_wild_41445209	1399937958	*Ca_kabuli_Chr04*	41445209	A/G	Gene_Ca_10963	NON_SYNONYMOUS_CODING	PeptidaseC19,ubiquitincarboxyl-terminalhydrolase2
Kabuli_wild_41445225	1399937959	*Ca_kabuli_Chr04*	41445225	A/T	Gene_Ca_10963	INTRON	PeptidaseC19,ubiquitincarboxyl-terminalhydrolase2
Kabuli_wild_41512840	1399937960	*Ca_kabuli_Chr04*	41512840	G/A	Gene_Ca_10956	INTRON	Skb1 methyltransferase
Kabuli_wild_41568682	1399937961	*Ca_kabuli_Chr04*	41568682	A/C	NA	INTERGENIC	NA
Kabuli_wild_41572031	1399937962	*Ca_kabuli_Chr04*	41572031	G/A	Gene_Ca_10953	NON_SYNONYMOUS_CODING	Skb1 methyltransferase
Kabuli_wild_41600485	1399937963	*Ca_kabuli_Chr04*	41600485	A/C	NA	INTERGENIC	NA

SNP IDs	NCBI dbSNP accession IDs (NCBI_SS#)	Chromosomes	Physical positions (bp)	SNPs	Gene accession IDs	Sequence components of genome	Putative functions
Kabuli_wild_41629852	1399937964	*Ca_kabuli_Chr04*	41629852	T/A	NA	INTERGENIC	NA
Kabuli_wild_41629907	1399937965	*Ca_kabuli_Chr04*	41629907	C/A	NA	INTERGENIC	NA
Kabuli_wild_41648927	1399937966	*Ca_kabuli_Chr04*	41648927	C/T	Gene_Ca_10946	SYNONYMOUS_CODING	NA
Kabuli_wild_41648998	1399937967	*Ca_kabuli_Chr04*	41648998	A/G	Gene_Ca_10946	SYNONYMOUS_CODING	NA
Kabuli_wild_41698291	1399937968	*Ca_kabuli_Chr04*	41698291	A/G	NA	INTERGENIC	NA
Kabuli_wild_41698386	1399937969	*Ca_kabuli_Chr04*	41698386	A/G	NA	INTERGENIC	NA
Kabuli_wild_41702800	1399937970	*Ca_kabuli_Chr04*	41702800	C/A	Gene_Ca_10942	NON_SYNONYMOUS_CODING	Enhancerofpolycomb-like,N-terminal
Kabuli_wild_41702801	1399937971	*Ca_kabuli_Chr04*	41702801	C/G	Gene_Ca_10942	NON_SYNONYMOUS_CODING	Enhancerofpolycomb-like,N-terminal
Kabuli_wild_41721187	1399937972	*Ca_kabuli_Chr04*	41721187	G/A	Gene_Ca_10941	SYNONYMOUS_CODING	NA
Kabuli_wild_41721289	1399937973	*Ca_kabuli_Chr04*	41721289	G/A	Gene_Ca_10941	SYNONYMOUS_CODING	NA
Kabuli_wild_41722384	1399937974	*Ca_kabuli_Chr04*	41722384	G/A	Gene_Ca_10941	SYNONYMOUS_CODING	NA
Kabuli_wild_41722408	1399937975	*Ca_kabuli_Chr04*	41722408	C/T	Gene_Ca_10941	SYNONYMOUS_CODING	NA
Kabuli_wild_41739416	1399937976	*Ca_kabuli_Chr04*	41739416	G/A	Gene_Ca_10940	INTRON	Protein of unknown function DUF490
Kabuli_wild_41739587	1399937977	*Ca_kabuli_Chr04*	41739587	A/G	Gene_Ca_10940	INTRON	Protein of unknown function DUF490
Kabuli_wild_41772690	1399937978	*Ca_kabuli_Chr04*	41772690	C/T	Gene_Ca_10937	DOWNSTREAM	Cyclin,C-terminal
Kabuli_wild_41772794	1399937979	*Ca_kabuli_Chr04*	41772794	T/C	Gene_Ca_10938	DOWNSTREAM	Proteinphosphatase2A,regulatoryBsubunit,B56
Kabuli_wild_41772796	1399937980	*Ca_kabuli_Chr04*	41772796	T/A	Gene_Ca_10938	DOWNSTREAM	Proteinphosphatase2A,regulatoryBsubunit,B56
Kabuli_wild_41774272	1399937981	*Ca_kabuli_Chr04*	41774272	A/G	Gene_Ca_10937	INTRON	Cyclin,C-terminal
Kabuli_wild_41864826	1399937982	*Ca_kabuli_Chr04*	41864826	G/A	Gene_Ca_10932	SYNONYMOUS_CODING	WD40 repeat
Kabuli_wild_41864846	1399937983	*Ca_kabuli_Chr04*	41864846	C/G	Gene_Ca_10932	NON_SYNONYMOUS_CODING	WD40 repeat
Kabuli_wild_41864916	1399937984	*Ca_kabuli_Chr04*	41864916	A/T	Gene_Ca_10932	SYNONYMOUS_CODING	WD40 repeat
Kabuli_wild_41865090	1399937985	*Ca_kabuli_Chr04*	41865090	T/C	Gene_Ca_10932	SYNONYMOUS_CODING	WD40 repeat
Kabuli_wild_41865128	1399937986	*Ca_kabuli_Chr04*	41865128	G/A	Gene_Ca_10932	STOP_GAINED	WD40 repeat
Kabuli_wild_41956050	1399937987	*Ca_kabuli_Chr04*	41956050	C/G	NA	INTERGENIC	NA
Kabuli_wild_41956085	1399937988	*Ca_kabuli_Chr04*	41956085	T/C	NA	INTERGENIC	NA
Kabuli_wild_41987193	1399937989	*Ca_kabuli_Chr04*	41987193	A/G	Gene_Ca_10923	INTRON	HistoneH2A
Kabuli_wild_42092379	1399937990	*Ca_kabuli_Chr04*	42092379	T/G	Gene_Ca_10914	INTRON	Tesmin/TSO1-like,CXC
Kabuli_wild_42092381	1399937991	*Ca_kabuli_Chr04*	42092381	A/G	Gene_Ca_10914	INTRON	Tesmin/TSO1-like,CXC
Kabuli_wild_42092385	1399937992	*Ca_kabuli_Chr04*	42092385	G/A	Gene_Ca_10914	INTRON	Tesmin/TSO1-like,CXC
Kabuli_wild_42105701	1399937993	*Ca_kabuli_Chr04*	42105701	A/T	Gene_Ca_10912	INTRON	ATPase,AAA+type,core
Kabuli_wild_42105737	1399937994	*Ca_kabuli_Chr04*	42105737	C/T	Gene_Ca_10912	INTRON	ATPase,AAA+type,core
Kabuli_wild_42143775	1399937995	*Ca_kabuli_Chr04*	42143775	A/G	Gene_Ca_10910	INTRON	Glycosidehydrolase,family28
Kabuli_wild_42143779	1399937996	*Ca_kabuli_Chr04*	42143779	G/A	Gene_Ca_10910	INTRON	Glycosidehydrolase,family28
Kabuli_wild_42143805	1399937997	*Ca_kabuli_Chr04*	42143805	T/A	Gene_Ca_10910	INTRON	Glycosidehydrolase,family28
Kabuli_wild_42143836	1399937998	*Ca_kabuli_Chr04*	42143836	A/G	Gene_Ca_10910	INTRON	Glycosidehydrolase,family28
Kabuli_wild_42246527	1399937999	*Ca_kabuli_Chr04*	42246527	C/A	Gene_Ca_10904	UPSTREAM	NA
Kabuli_wild_42246565	1399938000	*Ca_kabuli_Chr04*	42246565	T/G	Gene_Ca_10904	UPSTREAM	NA
Kabuli_wild_42246591	1399938001	*Ca_kabuli_Chr04*	42246591	G/T	Gene_Ca_10904	UPSTREAM	NA
Kabuli_wild_42246594	1399938002	*Ca_kabuli_Chr04*	42246594	C/T	Gene_Ca_10904	UPSTREAM	NA
Kabuli_wild_42246796	1399938003	*Ca_kabuli_Chr04*	42246796	A/G	Gene_Ca_10904	UPSTREAM	NA
Kabuli_wild_42273231	1399938004	*Ca_kabuli_Chr04*	42273231	C/T	Gene_Ca_10903	INTRON	Methyltransferasetype11
Kabuli_wild_42273252	1399938005	*Ca_kabuli_Chr04*	42273252	G/A	Gene_Ca_10903	INTRON	Methyltransferasetype11
Kabuli_wild_42273291	1399938006	*Ca_kabuli_Chr04*	42273291	G/A	Gene_Ca_10903	INTRON	Methyltransferasetype11
Kabuli_wild_42273294	1399938007	*Ca_kabuli_Chr04*	42273294	C/T	Gene_Ca_10903	INTRON	Methyltransferasetype11

SNP IDs	NCBI dbSNP accession IDs (NCBI_SS#)	Chromosomes	Physical positions (bp)	SNPs	Gene accession IDs	Sequence components of genome	Putative functions
Kabuli_wild_42499699	1399938008	*Ca_kabuli_Chr04*	42499699	A/T	Gene_Ca_10898	NON_SYNONYMOUS_CODING	Protein kinase,catalytic domain
Kabuli_wild_42592151	1399938009	*Ca_kabuli_Chr04*	42592151	C/T	NA	INTERGENIC	NA
Kabuli_wild_42592198	1399938010	*Ca_kabuli_Chr04*	42592198	G/A	NA	INTERGENIC	NA
Kabuli_wild_42592212	1399938011	*Ca_kabuli_Chr04*	42592212	A/G	NA	INTERGENIC	NA
Kabuli_wild_42592218	1399938012	*Ca_kabuli_Chr04*	42592218	C/T	NA	INTERGENIC	NA
Kabuli_wild_42592232	1399938013	*Ca_kabuli_Chr04*	42592232	G/A	NA	INTERGENIC	NA
Kabuli_wild_42715882	1399938014	*Ca_kabuli_Chr04*	42715882	A/G	Gene_Ca_10890	NON_SYNONYMOUS_CODING	Tetra tricopeptide repeat-containing
Kabuli_wild_42768142	1399938015	*Ca_kabuli_Chr04*	42768142	T/G	NA	INTERGENIC	NA
Kabuli_wild_42807483	1399938016	*Ca_kabuli_Chr04*	42807483	G/A	NA	INTERGENIC	NA
Kabuli_wild_42807518	1399938017	*Ca_kabuli_Chr04*	42807518	A/G	NA	INTERGENIC	NA
Kabuli_wild_43319846	1399938018	*Ca_kabuli_Chr04*	43319846	A/G	Gene_Ca_23683	SYNONYMOUS_CODING	Cyclin,C-terminal
Kabuli_wild_43324547	1399938019	*Ca_kabuli_Chr04*	43324547	G/C	NA	INTERGENIC	NA
Kabuli_wild_43324746	1399938020	*Ca_kabuli_Chr04*	43324746	C/T	NA	INTERGENIC	NA
Kabuli_wild_43431770	1399938021	*Ca_kabuli_Chr04*	43431770	C/T	NA	INTERGENIC	NA
Kabuli_wild_43431772	1399938022	*Ca_kabuli_Chr04*	43431772	T/A	NA	INTERGENIC	NA
Kabuli_wild_43431815	1399938023	*Ca_kabuli_Chr04*	43431815	T/G	NA	INTERGENIC	NA
Kabuli_wild_43447256	1399938024	*Ca_kabuli_Chr04*	43447256	G/A	NA	INTERGENIC	NA
Kabuli_wild_43447262	1399938025	*Ca_kabuli_Chr04*	43447262	A/T	NA	INTERGENIC	NA
Kabuli_wild_43475977	1399938026	*Ca_kabuli_Chr04*	43475977	T/C	NA	INTERGENIC	NA
Kabuli_wild_43476035	1399938027	*Ca_kabuli_Chr04*	43476035	T/A	NA	INTERGENIC	NA
Kabuli_wild_43476193	1399938028	*Ca_kabuli_Chr04*	43476193	T/C	Gene_Ca_23692	SYNONYMOUS_CODING	Pollen Olee1allergen/extensin
Kabuli_wild_43510426	1399938029	*Ca_kabuli_Chr04*	43510426	G/A	NA	INTERGENIC	NA
Kabuli_wild_43653225	1399938030	*Ca_kabuli_Chr04*	43653225	A/G	NA	INTERGENIC	NA
Kabuli_wild_43653226	1399938031	*Ca_kabuli_Chr04*	43653226	C/T	NA	INTERGENIC	NA
Kabuli_wild_43662224	1399938032	*Ca_kabuli_Chr04*	43662224	A/C	NA	INTERGENIC	NA
Kabuli_wild_43676507	1399938033	*Ca_kabuli_Chr04*	43676507	T/A	Gene_Ca_09052	SYNONYMOUS_CODING	Leucine-rich repeat
Kabuli_wild_43677006	1399938034	*Ca_kabuli_Chr04*	43677006	T/G	Gene_Ca_09052	NON_SYNONYMOUS_CODING	Leucine-rich repeat
Kabuli_wild_43722727	1399938035	*Ca_kabuli_Chr04*	43722727	A/G	NA	INTERGENIC	NA
Kabuli_wild_43722728	1399938036	*Ca_kabuli_Chr04*	43722728	T/C	NA	INTERGENIC	NA
Kabuli_wild_43726240	1399938037	*Ca_kabuli_Chr04*	43726240	A/T	NA	INTERGENIC	NA
Kabuli_wild_43726406	1399938038	*Ca_kabuli_Chr04*	43726406	C/T	NA	INTERGENIC	NA
Kabuli_wild_43730671	1399938039	*Ca_kabuli_Chr04*	43730671	A/T	NA	INTERGENIC	NA
Kabuli_wild_43730719	1399938040	*Ca_kabuli_Chr04*	43730719	C/T	NA	INTERGENIC	NA
Kabuli_wild_43763791	1399938041	*Ca_kabuli_Chr04*	43763791	A/G	NA	INTERGENIC	NA
Kabuli_wild_43807399	1399938042	*Ca_kabuli_Chr04*	43807399	C/T	Gene_Ca_09060	SYNONYMOUS_CODING	WD40 repeat
Kabuli_wild_43807460	1399938043	*Ca_kabuli_Chr04*	43807460	C/T	Gene_Ca_09060	INTRON	WD40 repeat
Kabuli_wild_43840650	1399938044	*Ca_kabuli_Chr04*	43840650	C/T	NA	INTERGENIC	NA
Kabuli_wild_43889033	1399938045	*Ca_kabuli_Chr04*	43889033	A/T	Gene_Ca_09068	NON_SYNONYMOUS_CODING	Homeobox
Kabuli_wild_43979065	1399938046	*Ca_kabuli_Chr04*	43979065	A/G	NA	INTERGENIC	NA
Kabuli_wild_44059060	1399938047	*Ca_kabuli_Chr04*	44059060	C/T	Gene_Ca_09080	NON_SYNONYMOUS_CODING	Signal transduction response regulator, receiverdomain
Kabuli_wild_44090715	1399938048	*Ca_kabuli_Chr04*	44090715	A/T	Gene_Ca_09082	INTRON	Armadillo
Kabuli_wild_44239883	1399938049	*Ca_kabuli_Chr04*	44239883	A/T	Gene_Ca_09098	UPSTREAM	Crotonase,core
Kabuli_wild_44250159	1399938050	*Ca_kabuli_Chr04*	44250159	C/T	NA	INTERGENIC	NA
Kabuli_wild_44261184	1399938051	*Ca_kabuli_Chr04*	44261184	A/C	Gene_Ca_09100	SYNONYMOUS_CODING	Actin-bindingFH2/DRFautoregulatory

SNP IDs	NCBI dbSNP accession IDs (NCBI_SS#)	Chromosomes	Physical positions (bp)	SNPs	Gene accession IDs	Sequence components of genome	Putative functions
Kabuli_wild_44261211	1399938052	*Ca_kabuli_Chr04*	44261211	A/C	Gene_Ca_09100	SYNONYMOUS_CODING	Actin-bindingFH2/DRFautoregulatory
Kabuli_wild_44261535	1399938053	*Ca_kabuli_Chr04*	44261535	A/C	Gene_Ca_09100	SYNONYMOUS_CODING	Actin-bindingFH2/DRFautoregulatory
Kabuli_wild_44266692	1399938054	*Ca_kabuli_Chr04*	44266692	T/A	Gene_Ca_09100	DOWNSTREAM	Actin-bindingFH2/DRFautoregulatory
Kabuli_wild_44294229	1399938055	*Ca_kabuli_Chr04*	44294229	T/G	NA	INTERGENIC	NA
Kabuli_wild_44353343	1399938056	*Ca_kabuli_Chr04*	44353343	G/A	Gene_Ca_09105	INTRON	Protein of unknown function DUF1077,TMEM85
Kabuli_wild_44353354	1399938057	*Ca_kabuli_Chr04*	44353354	C/T	Gene_Ca_09105	INTRON	Protein of unknown function DUF1077,TMEM85
Kabuli_wild_44375761	1399938058	*Ca_kabuli_Chr04*	44375761	T/C	Gene_Ca_09108	SYNONYMOUS_CODING	NA
Kabuli_wild_44375779	1399938059	*Ca_kabuli_Chr04*	44375779	T/C	Gene_Ca_09108	SYNONYMOUS_CODING	NA
Kabuli_wild_44375831	1399938060	*Ca_kabuli_Chr04*	44375831	T/C	Gene_Ca_09108	INTRON	NA
Kabuli_wild_44382515	1399938061	*Ca_kabuli_Chr04*	44382515	A/G	NA	INTERGENIC	NA
Kabuli_wild_44382602	1399938062	*Ca_kabuli_Chr04*	44382602	A/G	NA	INTERGENIC	NA
Kabuli_wild_44382614	1399938063	*Ca_kabuli_Chr04*	44382614	C/A	NA	INTERGENIC	NA
Kabuli_wild_44382671	1399938064	*Ca_kabuli_Chr04*	44382671	C/G	NA	INTERGENIC	NA
Kabuli_wild_44382677	1399938065	*Ca_kabuli_Chr04*	44382677	C/T	NA	INTERGENIC	NA
Kabuli_wild_44386579	1399938066	*Ca_kabuli_Chr04*	44386579	A/G	Gene_Ca_09109	SYNONYMOUS_CODING	Protein phosphatase 2C, manganese/magnesium aspartate binding site
Kabuli_wild_44388391	1399938067	*Ca_kabuli_Chr04*	44388391	G/C	Gene_Ca_09109	SYNONYMOUS_CODING	Protein phosphatase 2C, manganese/magnesium aspartate binding site
Kabuli_wild_44388394	1399938068	*Ca_kabuli_Chr04*	44388394	C/T	Gene_Ca_09109	SYNONYMOUS_CODING	Protein phosphatase 2C, manganese/magnesium aspartate binding site
Kabuli_wild_44388671	1399938069	*Ca_kabuli_Chr04*	44388671	C/G	NA	INTERGENIC	NA
Kabuli_wild_44431319	1399938070	*Ca_kabuli_Chr04*	44431319	A/C	Gene_Ca_09114	INTRON	PeptidaseC19,ubiquitincarboxyl-terminalhydrolase2
Kabuli_wild_44439492	1399938071	*Ca_kabuli_Chr04*	44439492	G/T	NA	INTERGENIC	NA
Kabuli_wild_44439495	1399938072	*Ca_kabuli_Chr04*	44439495	G/A	NA	INTERGENIC	NA
Kabuli_wild_44439503	1399938073	*Ca_kabuli_Chr04*	44439503	C/A	NA	INTERGENIC	NA
Kabuli_wild_44446468	1399938074	*Ca_kabuli_Chr04*	44446468	G/A	NA	INTERGENIC	NA
Kabuli_wild_44446503	1399938075	*Ca_kabuli_Chr04*	44446503	T/G	NA	INTERGENIC	NA
Kabuli_wild_44446575	1399938076	*Ca_kabuli_Chr04*	44446575	G/A	NA	INTERGENIC	NA
Kabuli_wild_44472237	1399938077	*Ca_kabuli_Chr04*	44472237	T/A	Gene_Ca_09119	UPSTREAM	NA
Kabuli_wild_44472278	1399938078	*Ca_kabuli_Chr04*	44472278	A/G	Gene_Ca_09119	UPSTREAM	NA
Kabuli_wild_44590652	1399938079	*Ca_kabuli_Chr04*	44590652	T/C	Gene_Ca_09131	SYNONYMOUS_CODING	Protein phosphatase 2C, manganese/magnesium aspartate binding site
Kabuli_wild_44590678	1399938080	*Ca_kabuli_Chr04*	44590678	G/A	Gene_Ca_09131	NON_SYNONYMOUS_CODING	Protein phosphatase 2C, manganese/magnesium aspartate binding site
Kabuli_wild_44590697	1399938081	*Ca_kabuli_Chr04*	44590697	A/C	Gene_Ca_09131	SYNONYMOUS_CODING	Protein phosphatase 2C, manganese/magnesium aspartate binding site
Kabuli_wild_44591405	1399938082	*Ca_kabuli_Chr04*	44591405	G/T	Gene_Ca_09131	INTRON	Protein phosphatase 2C, manganese/magnesium aspartate binding site
Kabuli_wild_44754355	1399938083	*Ca_kabuli_Chr04*	44754355	C/T	Gene_Ca_09143	INTRON	F-boxdomain,cyclin-like
Kabuli_wild_44802364	1399938084	*Ca_kabuli_Chr04*	44802364	A/C	Gene_Ca_09145	INTRON	PhosphoribosyltransferaseC-terminal
Kabuli_wild_44802374	1399938085	*Ca_kabuli_Chr04*	44802374	G/A	Gene_Ca_09145	INTRON	PhosphoribosyltransferaseC-terminal
Kabuli_wild_44803694	1399938086	*Ca_kabuli_Chr04*	44803694	T/A	Gene_Ca_09145	INTRON	PhosphoribosyltransferaseC-terminal
Kabuli_wild_44811519	1399938087	*Ca_kabuli_Chr04*	44811519	G/A	Gene_Ca_09145	UPSTREAM	PhosphoribosyltransferaseC-terminal
Kabuli_wild_44814857	1399938088	*Ca_kabuli_Chr04*	44814857	A/G	NA	INTERGENIC	NA
Kabuli_wild_44814981	1399938089	*Ca_kabuli_Chr04*	44814981	T/C	NA	INTERGENIC	NA
Kabuli_wild_44814982	1399938090	*Ca_kabuli_Chr04*	44814982	A/G	NA	INTERGENIC	NA
Kabuli_wild_44824103	1399938091	*Ca_kabuli_Chr04*	44824103	G/T	NA	INTERGENIC	NA
Kabuli_wild_44824105	1399938092	*Ca_kabuli_Chr04*	44824105	T/G	NA	INTERGENIC	NA
Kabuli_wild_44832121	1399938093	*Ca_kabuli_Chr04*	44832121	G/C	Gene_Ca_09147	DOWNSTREAM	Dilute
Kabuli_wild_44832144	1399938094	*Ca_kabuli_Chr04*	44832144	T/C	Gene_Ca_09147	DOWNSTREAM	Dilute
Kabuli_wild_44906128	1399938095	*Ca_kabuli_Chr04*	44906128	C/A	Gene_Ca_09153	INTRON	Protein of unknown function DUF803

SNP IDs	NCBI dbSNP accession IDs (NCBI_SS#)	Chromosomes	Physical positions (bp)	SNPs	Gene accession IDs	Sequence components of genome	Putative functions
Kabuli_wild_44906255	1399938096	*Ca_kabuli_Chr04*	44906255	A/G	Gene_Ca_09153	SYNONYMOUS_CODING	Protein of unknown function DUF803
Kabuli_wild_44947524	1399938097	*Ca_kabuli_Chr04*	44947524	T/C	Gene_Ca_09157	NON_SYNONYMOUS_CODING	Glycosidehydrolase,family32
Kabuli_wild_44979108	1399938098	*Ca_kabuli_Chr04*	44979108	G/A	NA	INTERGENIC	NA
Kabuli_wild_45019764	1399938099	*Ca_kabuli_Chr04*	45019764	T/C	NA	INTERGENIC	NA
Kabuli_wild_45019778	1399938100	*Ca_kabuli_Chr04*	45019778	T/C	NA	INTERGENIC	NA
Kabuli_wild_45019805	1399938101	*Ca_kabuli_Chr04*	45019805	C/A	NA	INTERGENIC	NA
Kabuli_wild_45019807	1399938102	*Ca_kabuli_Chr04*	45019807	C/T	NA	INTERGENIC	NA
Kabuli_wild_45019814	1399938103	*Ca_kabuli_Chr04*	45019814	C/A	NA	INTERGENIC	NA
Kabuli_wild_45019820	1399938104	*Ca_kabuli_Chr04*	45019820	C/T	NA	INTERGENIC	NA
Kabuli_wild_45019824	1399938105	*Ca_kabuli_Chr04*	45019824	A/C	NA	INTERGENIC	NA
Kabuli_wild_45019825	1399938106	*Ca_kabuli_Chr04*	45019825	A/T	NA	INTERGENIC	NA
Kabuli_wild_45019826	1399938107	*Ca_kabuli_Chr04*	45019826	A/G	NA	INTERGENIC	NA
Kabuli_wild_45019848	1399938108	*Ca_kabuli_Chr04*	45019848	C/T	NA	INTERGENIC	NA
Kabuli_wild_45019867	1399938109	*Ca_kabuli_Chr04*	45019867	C/G	NA	INTERGENIC	NA
Kabuli_wild_45022634	1399938110	*Ca_kabuli_Chr04*	45022634	G/C	Gene_Ca_09165	INTRON	Mrp,conservedsite
Kabuli_wild_45035589	1399938111	*Ca_kabuli_Chr04*	45035589	G/T	NA	INTERGENIC	NA
Kabuli_wild_45035745	1399938112	*Ca_kabuli_Chr04*	45035745	A/G	NA	INTERGENIC	NA
Kabuli_wild_45089570	1399938113	*Ca_kabuli_Chr04*	45089570	T/C	Gene_Ca_09169	DOWNSTREAM	GlycinecleavagesystemP-protein,N-terminal
Kabuli_wild_45104767	1399938114	*Ca_kabuli_Chr04*	45104767	G/A	NA	INTERGENIC	NA
Kabuli_wild_45104779	1399938115	*Ca_kabuli_Chr04*	45104779	C/A	NA	INTERGENIC	NA
Kabuli_wild_45141973	1399938116	*Ca_kabuli_Chr04*	45141973	A/C	NA	INTERGENIC	NA
Kabuli_wild_45165191	1399938117	*Ca_kabuli_Chr04*	45165191	T/C	Gene_Ca_09179	DOWNSTREAM	Cation/H+exchanger
Kabuli_wild_45165231	1399938118	*Ca_kabuli_Chr04*	45165231	A/C	Gene_Ca_09179	DOWNSTREAM	Cation/H+exchanger
Kabuli_wild_45165240	1399938119	*Ca_kabuli_Chr04*	45165240	T/C	Gene_Ca_09179	DOWNSTREAM	Cation/H+exchanger
Kabuli_wild_45204033	1399938120	*Ca_kabuli_Chr04*	45204033	A/G	Gene_Ca_09182	INTRON	NA
Kabuli_wild_45204213	1399938121	*Ca_kabuli_Chr04*	45204213	G/A	Gene_Ca_09182	NON_SYNONYMOUS_CODING	NA
Kabuli_wild_45206209	1399938122	*Ca_kabuli_Chr04*	45206209	G/C	Gene_Ca_09183	UPSTREAM	SAC3/GANP/Nin1/mts3/eIF-3p25
Kabuli_wild_45220084	1399938123	*Ca_kabuli_Chr04*	45220084	G/T	Gene_Ca_09184	SYNONYMOUS_CODING	K Homology
Kabuli_wild_45220916	1399938124	*Ca_kabuli_Chr04*	45220916	T/C	Gene_Ca_09184	NON_SYNONYMOUS_CODING	K Homology
Kabuli_wild_45224997	1399938125	*Ca_kabuli_Chr04*	45224997	G/C	NA	INTERGENIC	NA
Kabuli_wild_45225059	1399938126	*Ca_kabuli_Chr04*	45225059	G/A	NA	INTERGENIC	NA
Kabuli_wild_45225063	1399938127	*Ca_kabuli_Chr04*	45225063	T/C	NA	INTERGENIC	NA
Kabuli_wild_45225077	1399938128	*Ca_kabuli_Chr04*	45225077	A/T	NA	INTERGENIC	NA
Kabuli_wild_45225093	1399938129	*Ca_kabuli_Chr04*	45225093	G/A	NA	INTERGENIC	NA
Kabuli_wild_45225145	1399938130	*Ca_kabuli_Chr04*	45225145	T/A	NA	INTERGENIC	NA
Kabuli_wild_45225155	1399938131	*Ca_kabuli_Chr04*	45225155	C/A	NA	INTERGENIC	NA
Kabuli_wild_45225165	1399938132	*Ca_kabuli_Chr04*	45225165	T/C	NA	INTERGENIC	NA
Kabuli_wild_45231839	1399938133	*Ca_kabuli_Chr04*	45231839	T/C	Gene_Ca_09187	DOWNSTREAM	Phosphorylated adapter RNA export protein,RNA-binding domain
Kabuli_wild_45231893	1399938134	*Ca_kabuli_Chr04*	45231893	G/T	Gene_Ca_09187	DOWNSTREAM	Phosphorylated adapter RNA export protein,RNA-binding domain
Kabuli_wild_45292787	1399938135	*Ca_kabuli_Chr04*	45292787	C/T	Gene_Ca_09192	SYNONYMOUS_CODING	NA
Kabuli_wild_45292832	1399938136	*Ca_kabuli_Chr04*	45292832	G/A	Gene_Ca_09192	SYNONYMOUS_CODING	NA
Kabuli_wild_45292885	1399938137	*Ca_kabuli_Chr04*	45292885	C/A	Gene_Ca_09192	NON_SYNONYMOUS_CODING	NA
Kabuli_wild_45292918	1399938138	*Ca_kabuli_Chr04*	45292918	T/G	Gene_Ca_09192	UPSTREAM	NA
Kabuli_wild_45292960	1399938139	*Ca_kabuli_Chr04*	45292960	A/T	Gene_Ca_09192	UPSTREAM	NA

SNP IDs	NCBI dbSNP accession IDs (NCBI_SS#)	Chromosomes	Physical positions (bp)	SNPs	Gene accession IDs	Sequence components of genome	Putative functions
Kabuli_wild_45292969	1399938140	*Ca_kabuli_Chr04*	45292969	T/A	Gene_Ca_09192	UPSTREAM	NA
Kabuli_wild_45292981	1399938141	*Ca_kabuli_Chr04*	45292981	G/T	Gene_Ca_09192	UPSTREAM	NA
Kabuli_wild_45615407	1399938142	*Ca_kabuli_Chr04*	45615407	T/C	Gene_Ca_09217	INTRON	WW/Rsp5/WWP
Kabuli_wild_45615443	1399938143	*Ca_kabuli_Chr04*	45615443	G/C	Gene_Ca_09217	INTRON	WW/Rsp5/WWP
Kabuli_wild_45643913	1399938144	*Ca_kabuli_Chr04*	45643913	G/A	NA	INTERGENIC	NA
Kabuli_wild_45645922	1399938145	*Ca_kabuli_Chr04*	45645922	G/A	NA	INTERGENIC	NA
Kabuli_wild_45645935	1399938146	*Ca_kabuli_Chr04*	45645935	C/A	NA	INTERGENIC	NA
Kabuli_wild_45645944	1399938147	*Ca_kabuli_Chr04*	45645944	G/A	NA	INTERGENIC	NA
Kabuli_wild_45645951	1399938148	*Ca_kabuli_Chr04*	45645951	G/A	NA	INTERGENIC	NA
Kabuli_wild_45645957	1399938149	*Ca_kabuli_Chr04*	45645957	A/G	NA	INTERGENIC	NA
Kabuli_wild_45645964	1399938150	*Ca_kabuli_Chr04*	45645964	T/A	NA	INTERGENIC	NA
Kabuli_wild_45645972	1399938151	*Ca_kabuli_Chr04*	45645972	T/G	NA	INTERGENIC	NA
Kabuli_wild_45645973	1399938152	*Ca_kabuli_Chr04*	45645973	G/T	NA	INTERGENIC	NA
Kabuli_wild_45645975	1399938153	*Ca_kabuli_Chr04*	45645975	G/T	NA	INTERGENIC	NA
Kabuli_wild_45645996	1399938154	*Ca_kabuli_Chr04*	45645996	A/G	NA	INTERGENIC	NA
Kabuli_wild_45645999	1399938155	*Ca_kabuli_Chr04*	45645999	C/G	NA	INTERGENIC	NA
Kabuli_wild_45708895	1399938156	*Ca_kabuli_Chr04*	45708895	C/A	NA	INTERGENIC	NA
Kabuli_wild_45736291	1399938157	*Ca_kabuli_Chr04*	45736291	G/C	Gene_Ca_18387	NON_SYNONYMOUS_CODING	Pathogenesis-related transcriptional factor/ERF, DNA-binding
Kabuli_wild_45736334	1399938158	*Ca_kabuli_Chr04*	45736334	G/A	Gene_Ca_18387	SYNONYMOUS_CODING	Pathogenesis-related transcriptional factor/ERF, DNA-binding
Kabuli_wild_45752844	1399938159	*Ca_kabuli_Chr04*	45752844	G/A	Gene_Ca_18386	UPSTREAM	NA
Kabuli_wild_45752869	1399938160	*Ca_kabuli_Chr04*	45752869	G/T	Gene_Ca_18386	UPSTREAM	NA
Kabuli_wild_45752874	1399938161	*Ca_kabuli_Chr04*	45752874	A/T	Gene_Ca_18386	UPSTREAM	NA
Kabuli_wild_45753229	1399938162	*Ca_kabuli_Chr04*	45753229	C/T	Gene_Ca_18386	UPSTREAM	NA
Kabuli_wild_45817472	1399938163	*Ca_kabuli_Chr04*	45817472	G/A	Gene_Ca_18379	SYNONYMOUS_CODING	NA
Kabuli_wild_45869030	1399938164	*Ca_kabuli_Chr04*	45869030	T/C	NA	INTERGENIC	NA
Kabuli_wild_45918350	1399938165	*Ca_kabuli_Chr04*	45918350	T/C	NA	INTERGENIC	NA
Kabuli_wild_45918351	1399938166	*Ca_kabuli_Chr04*	45918351	A/C	NA	INTERGENIC	NA
Kabuli_wild_45935594	1399938167	*Ca_kabuli_Chr04*	45935594	A/C	Gene_Ca_18367	NON_SYNONYMOUS_CODING	RNA recognition motif domain
Kabuli_wild_46079203	1399938168	*Ca_kabuli_Chr04*	46079203	T/A	Gene_Ca_18362	UPSTREAM	Protein of unknown function DUF566
Kabuli_wild_46148985	1399938169	*Ca_kabuli_Chr04*	46148985	A/T	Gene_Ca_18357	INTRON	Zinc finger, RING-type
Kabuli_wild_46149013	1399938170	*Ca_kabuli_Chr04*	46149013	A/G	Gene_Ca_18357	INTRON	Zinc finger, RING-type
Kabuli_wild_46150670	1399938171	*Ca_kabuli_Chr04*	46150670	T/C	Gene_Ca_18357	INTRON	Zinc finger, RING-type
Kabuli_wild_46202862	1399938172	*Ca_kabuli_Chr04*	46202862	A/G	NA	INTERGENIC	NA
Kabuli_wild_46392506	1399938173	*Ca_kabuli_Chr04*	46392506	C/T	NA	INTERGENIC	NA
Kabuli_wild_46612244	1399938174	*Ca_kabuli_Chr04*	46612244	C/T	Gene_Ca_19257	DOWNSTREAM	Glycosidehydrolase,family13
Kabuli_wild_46613068	1399938175	*Ca_kabuli_Chr04*	46613068	T/G	Gene_Ca_19257	DOWNSTREAM	Glycosidehydrolase,family13
Kabuli_wild_46668821	1399938176	*Ca_kabuli_Chr04*	46668821	T/C	Gene_Ca_19252	NON_SYNONYMOUS_CODING	Proteinphosphatase2C-like
Kabuli_wild_46694919	1399938177	*Ca_kabuli_Chr04*	46694919	T/A	NA	INTERGENIC	NA
Kabuli_wild_46695008	1399938178	*Ca_kabuli_Chr04*	46695008	T/C	NA	INTERGENIC	NA
Kabuli_wild_46695043	1399938179	*Ca_kabuli_Chr04*	46695043	A/C	NA	INTERGENIC	NA
Kabuli_wild_46705167	1399938180	*Ca_kabuli_Chr04*	46705167	A/G	Gene_Ca_19247	NON_SYNONYMOUS_CODING	Patatin/PhospholipaseA2-related
Kabuli_wild_46705178	1399938181	*Ca_kabuli_Chr04*	46705178	A/T	Gene_Ca_19247	SYNONYMOUS_CODING	Patatin/PhospholipaseA2-related
Kabuli_wild_46705225	1399938182	*Ca_kabuli_Chr04*	46705225	A/C	Gene_Ca_19247	INTRON	Patatin/PhospholipaseA2-related
Kabuli_wild_46713907	1399938183	*Ca_kabuli_Chr04*	46713907	T/G	Gene_Ca_19246	SYNONYMOUS_CODING	PGAP1-like

SNP IDs	NCBI dbSNP accession IDs (NCBI_SS#)	Chromosomes	Physical positions (bp)	SNPs	Gene accession IDs	Sequence components of genome	Putative functions
Kabuli_wild_46713972	1399938184	*Ca_kabuli_Chr04*	46713972	C/T	Gene_Ca_19246	NON_SYNONYMOUS_CODING	PGAP1-like
Kabuli_wild_46715350	1399938185	*Ca_kabuli_Chr04*	46715350	T/C	Gene_Ca_19246	SYNONYMOUS_CODING	PGAP1-like
Kabuli_wild_46717153	1399938186	*Ca_kabuli_Chr04*	46717153	C/T	Gene_Ca_19246	INTRON	PGAP1-like
Kabuli_wild_46717200	1399938187	*Ca_kabuli_Chr04*	46717200	C/T	Gene_Ca_19246	INTRON	PGAP1-like
Kabuli_wild_46717508	1399938188	*Ca_kabuli_Chr04*	46717508	G/A	Gene_Ca_19246	INTRON	PGAP1-like
Kabuli_wild_46733965	1399938189	*Ca_kabuli_Chr04*	46733965	A/G	NA	INTERGENIC	NA
Kabuli_wild_46763540	1399938190	*Ca_kabuli_Chr04*	46763540	C/T	Gene_Ca_19242	SYNONYMOUS_CODING	RNA recognition motif domain
Kabuli_wild_46763555	1399938191	*Ca_kabuli_Chr04*	46763555	C/T	Gene_Ca_19242	SYNONYMOUS_CODING	RNA recognition motif domain
Kabuli_wild_46763696	1399938192	*Ca_kabuli_Chr04*	46763696	C/A	Gene_Ca_19242	INTRON	RNA recognition motif domain
Kabuli_wild_46763841	1399938193	*Ca_kabuli_Chr04*	46763841	A/C	Gene_Ca_19242	INTRON	RNA recognition motif domain
Kabuli_wild_46763868	1399938194	*Ca_kabuli_Chr04*	46763868	T/C	Gene_Ca_19242	INTRON	RNA recognition motif domain
Kabuli_wild_47172795	1399938195	*Ca_kabuli_Chr04*	47172795	T/C	Gene_Ca_10864	NON_SYNONYMOUS_CODING	Domain of unknown function DUF547
Kabuli_wild_47184837	1399938196	*Ca_kabuli_Chr04*	47184837	G/A	NA	INTERGENIC	NA
Kabuli_wild_47223016	1399938197	*Ca_kabuli_Chr04*	47223016	T/C	Gene_Ca_10859	INTRON	Xanthine/uracil/vitamin C permease
Kabuli_wild_47223047	1399938198	*Ca_kabuli_Chr04*	47223047	A/G	Gene_Ca_10859	INTRON	Xanthine/uracil/vitamin C permease
Kabuli_wild_47223635	1399938199	*Ca_kabuli_Chr04*	47223635	G/A	Gene_Ca_10859	SYNONYMOUS_CODING	Xanthine/uracil/vitamin C permease
Kabuli_wild_47223668	1399938200	*Ca_kabuli_Chr04*	47223668	T/G	Gene_Ca_10859	SYNONYMOUS_CODING	Xanthine/uracil/vitamin C permease
Kabuli_wild_47296164	1399938201	*Ca_kabuli_Chr04*	47296164	A/C	NA	INTERGENIC	NA
Kabuli_wild_47296232	1399938202	*Ca_kabuli_Chr04*	47296232	A/G	NA	INTERGENIC	NA
Kabuli_wild_47351262	1399938203	*Ca_kabuli_Chr04*	47351262	G/A	Gene_Ca_10849	INTRON	Exocyst complex,component Exoc1
Kabuli_wild_47351308	1399938204	*Ca_kabuli_Chr04*	47351308	T/C	Gene_Ca_10849	SYNONYMOUS_CODING	Exocyst complex,component Exoc1
Kabuli_wild_47379992	1399938205	*Ca_kabuli_Chr04*	47379992	C/T	NA	INTERGENIC	NA
Kabuli_wild_47405685	1399938206	*Ca_kabuli_Chr04*	47405685	T/A	NA	INTERGENIC	NA
Kabuli_wild_47419514	1399938207	*Ca_kabuli_Chr04*	47419514	A/G	NA	INTERGENIC	NA
Kabuli_wild_47419517	1399938208	*Ca_kabuli_Chr04*	47419517	C/T	NA	INTERGENIC	NA
Kabuli_wild_47428580	1399938209	*Ca_kabuli_Chr04*	47428580	C/T	Gene_Ca_10845	INTRON	Multiantimicrobial extrusion protein
Kabuli_wild_47428599	1399938210	*Ca_kabuli_Chr04*	47428599	C/T	Gene_Ca_10845	INTRON	Multiantimicrobial extrusion protein
Kabuli_wild_47428733	1399938211	*Ca_kabuli_Chr04*	47428733	A/G	Gene_Ca_10845	SYNONYMOUS_CODING	Multiantimicrobial extrusion protein
Kabuli_wild_47450409	1399938212	*Ca_kabuli_Chr04*	47450409	C/T	Gene_Ca_10842	DOWNSTREAM	Zinc finger,LIM-type
Kabuli_wild_47572228	1399938213	*Ca_kabuli_Chr04*	47572228	G/A	NA	INTERGENIC	NA
Kabuli_wild_47572276	1399938214	*Ca_kabuli_Chr04*	47572276	C/T	NA	INTERGENIC	NA
Kabuli_wild_47578556	1399938215	*Ca_kabuli_Chr04*	47578556	T/G	Gene_Ca_10832	INTRON	MT-A70
Kabuli_wild_47578596	1399938216	*Ca_kabuli_Chr04*	47578596	C/T	Gene_Ca_10832	INTRON	MT-A70
Kabuli_wild_47588760	1399938217	*Ca_kabuli_Chr04*	47588760	G/A	Gene_Ca_10830	INTRON	Phospholipid/glycerolacyltransferase
Kabuli_wild_47592406	1399938218	*Ca_kabuli_Chr04*	47592406	A/C	Gene_Ca_10830	INTRON	Phospholipid/glycerolacyltransferase
Kabuli_wild_47592455	1399938219	*Ca_kabuli_Chr04*	47592455	C/T	Gene_Ca_10830	INTRON	Phospholipid/glycerolacyltransferase
Kabuli_wild_47612395	1399938220	*Ca_kabuli_Chr04*	47612395	C/A	Gene_Ca_10828	UPSTREAM	Reticulon
Kabuli_wild_47612396	1399938221	*Ca_kabuli_Chr04*	47612396	G/T	Gene_Ca_10828	UPSTREAM	Reticulon
Kabuli_wild_47622112	1399938222	*Ca_kabuli_Chr04*	47622112	G/T	NA	INTERGENIC	NA
Kabuli_wild_47672832	1399938223	*Ca_kabuli_Chr04*	47672832	G/A	NA	INTERGENIC	NA
Kabuli_wild_47672986	1399938224	*Ca_kabuli_Chr04*	47672986	T/C	Gene_Ca_10821	NON_SYNONYMOUS_CODING	GYF
Kabuli_wild_47675596	1399938225	*Ca_kabuli_Chr04*	47675596	A/C	Gene_Ca_10821	SYNONYMOUS_CODING	GYF
Kabuli_wild_47675818	1399938226	*Ca_kabuli_Chr04*	47675818	T/A	Gene_Ca_10821	SYNONYMOUS_CODING	GYF
Kabuli_wild_47711051	1399938227	*Ca_kabuli_Chr04*	47711051	T/C	Gene_Ca_10818	STOP_LOST	RibosomalproteinL13e

SNP IDs	NCBI dbSNP accession IDs (NCBI_SS#)	Chromosomes	Physical positions (bp)	SNPs	Gene accession IDs	Sequence components of genome	Putative functions
Kabuli_wild_47798580	1399938228	*Ca_kabuli_Chr04*	47798580	A/G	Gene_Ca_10814	UPSTREAM	Domain of unknown function DUF250
Kabuli_wild_48008987	1399938229	*Ca_kabuli_Chr04*	48008987	T/C	Gene_Ca_10788	SYNONYMOUS_CODING	Heat shock protein DnaJ,N-terminal
Kabuli_wild_48082684	1399938230	*Ca_kabuli_Chr04*	48082684	G/C	NA	INTERGENIC	NA
Kabuli_wild_48098508	1399938231	*Ca_kabuli_Chr04*	48098508	A/T	NA	INTERGENIC	NA
Kabuli_wild_48103153	1399938232	*Ca_kabuli_Chr04*	48103153	A/C	Gene_Ca_10777	SYNONYMOUS_CODING	Plantperoxidase
Kabuli_wild_48151187	1399938233	*Ca_kabuli_Chr04*	48151187	T/C	NA	INTERGENIC	NA
Kabuli_wild_48228061	1399938234	*Ca_kabuli_Chr04*	48228061	G/A	NA	INTERGENIC	NA
Kabuli_wild_48228110	1399938235	*Ca_kabuli_Chr04*	48228110	C/A	NA	INTERGENIC	NA
Kabuli_wild_48238919	1399938236	*Ca_kabuli_Chr04*	48238919	C/T	Gene_Ca_10764	NON_SYNONYMOUS_CODING	NA
Kabuli_wild_48239048	1399938237	*Ca_kabuli_Chr04*	48239048	A/G	Gene_Ca_10764	INTRON	NA
Kabuli_wild_48263603	1399938238	*Ca_kabuli_Chr04*	48263603	G/A	Gene_Ca_10761	SYNONYMOUS_CODING	DNA-directed RNA polymerase,subunit 2, domain 6
Kabuli_wild_48269138	1399938239	*Ca_kabuli_Chr04*	48269138	G/C	Gene_Ca_10760	INTRON	Amineoxidase
Kabuli_wild_48275016	1399938240	*Ca_kabuli_Chr04*	48275016	C/T	Gene_Ca_10760	SYNONYMOUS_CODING	Amineoxidase
Kabuli_wild_48305147	1399938241	*Ca_kabuli_Chr04*	48305147	C/G	NA	INTERGENIC	NA
Kabuli_wild_48322502	1399938242	*Ca_kabuli_Chr04*	48322502	C/T	Gene_Ca_10754	DOWNSTREAM	Cytochrome P450
Kabuli_wild_48322507	1399938243	*Ca_kabuli_Chr04*	48322507	C/T	Gene_Ca_10754	DOWNSTREAM	Amineoxidase
Kabuli_wild_48331069	1399938244	*Ca_kabuli_Chr04*	48331069	C/A	Gene_Ca_10753	NON_SYNONYMOUS_CODING	RibosomalproteinL13
Kabuli_wild_48345604	1399938245	*Ca_kabuli_Chr04*	48345604	A/G	Gene_Ca_10752	INTRON	IQ motif,EF-handbinding site
Kabuli_wild_48345608	1399938246	*Ca_kabuli_Chr04*	48345608	G/T	Gene_Ca_10752	INTRON	IQ motif,EF-handbinding site
Kabuli_wild_48345655	1399938247	*Ca_kabuli_Chr04*	48345655	A/C	Gene_Ca_10752	INTRON	IQ motif,EF-handbinding site
Kabuli_wild_48355358	1399938248	*Ca_kabuli_Chr04*	48355358	A/T	Gene_Ca_10751	INTRON	Exonuclease
Kabuli_wild_48355468	1399938249	*Ca_kabuli_Chr04*	48355468	G/T	Gene_Ca_10751	INTRON	Exonuclease
Kabuli_wild_48364170	1399938250	*Ca_kabuli_Chr04*	48364170	G/A	Gene_Ca_10750	INTRON	Helicase,C-terminal
Kabuli_wild_48365208	1399938251	*Ca_kabuli_Chr04*	48365208	C/A	Gene_Ca_10750	DOWNSTREAM	Helicase,C-terminal
Kabuli_wild_48365388	1399938252	*Ca_kabuli_Chr04*	48365388	A/G	Gene_Ca_10750	DOWNSTREAM	Helicase,C-terminal
Kabuli_wild_48382666	1399938253	*Ca_kabuli_Chr04*	48382666	C/G	Gene_Ca_10748	NON_SYNONYMOUS_CODING	AUX/IAAprotein
Kabuli_wild_48455591	1399938254	*Ca_kabuli_Chr04*	48455591	T/G	Gene_Ca_10747	NON_SYNONYMOUS_CODING	Protein of unknown function DUF869,plant
Kabuli_wild_48457876	1399938255	*Ca_kabuli_Chr04*	48457876	C/T	Gene_Ca_10747	INTRON	Protein of unknown function DUF869,plant
Kabuli_wild_48457943	1399938256	*Ca_kabuli_Chr04*	48457943	C/T	Gene_Ca_10747	INTRON	Protein of unknown function DUF869,plant
Kabuli_wild_48464493	1399938257	*Ca_kabuli_Chr04*	48464493	T/C	Gene_Ca_10746	NON_SYNONYMOUS_CODING	Zinc finger,CCHC-type
Kabuli_wild_48467653	1399938258	*Ca_kabuli_Chr04*	48467653	G/A	Gene_Ca_10746	NON_SYNONYMOUS_CODING	Zinc finger,CCHC-type
Kabuli_wild_48470477	1399938259	*Ca_kabuli_Chr04*	48470477	A/G	Gene_Ca_10746	INTRON	Zinc finger,CCHC-type
Kabuli_wild_48475447	1399938260	*Ca_kabuli_Chr04*	48475447	T/C	NA	INTERGENIC	NA
Kabuli_wild_48475461	1399938261	*Ca_kabuli_Chr04*	48475461	G/A	NA	INTERGENIC	NA
Kabuli_wild_48478018	1399938262	*Ca_kabuli_Chr04*	48478018	G/A	NA	INTERGENIC	NA
Kabuli_wild_48478058	1399938263	*Ca_kabuli_Chr04*	48478058	C/T	NA	INTERGENIC	NA
Kabuli_wild_48478303	1399938264	*Ca_kabuli_Chr04*	48478303	G/A	Gene_Ca_10745	SYNONYMOUS_CODING	Appr-1-pprocessing
Kabuli_wild_48478359	1399938265	*Ca_kabuli_Chr04*	48478359	T/A	Gene_Ca_10745	NON_SYNONYMOUS_CODING	Appr-1-pprocessing
Kabuli_wild_48478524	1399938266	*Ca_kabuli_Chr04*	48478524	C/T	Gene_Ca_10745	NON_SYNONYMOUS_CODING	Appr-1-pprocessing
Kabuli_wild_48484453	1399938267	*Ca_kabuli_Chr04*	48484453	A/T	NA	INTERGENIC	NA
Kabuli_wild_48497714	1399938268	*Ca_kabuli_Chr04*	48497714	A/G	NA	INTERGENIC	NA
Kabuli_wild_48497725	1399938269	*Ca_kabuli_Chr04*	48497725	A/C	NA	INTERGENIC	NA
Kabuli_wild_48497731	1399938270	*Ca_kabuli_Chr04*	48497731	A/C	NA	INTERGENIC	NA
Kabuli_wild_48497756	1399938271	*Ca_kabuli_Chr04*	48497756	A/G	NA	INTERGENIC	NA

SNP IDs	NCBI dbSNP accession IDs (NCBI_SS#)	Chromosomes	Physical positions (bp)	SNPs	Gene accession IDs	Sequence components of genome	Putative functions
Kabuli_wild_48497765	1399938272	*Ca_kabuli_Chr04*	48497765	G/A	NA	INTERGENIC	NA
Kabuli_wild_48497839	1399938273	*Ca_kabuli_Chr04*	48497839	G/A	NA	INTERGENIC	NA
Kabuli_wild_48497926	1399938274	*Ca_kabuli_Chr04*	48497926	T/A	NA	INTERGENIC	NA
Kabuli_wild_48497934	1399938275	*Ca_kabuli_Chr04*	48497934	T/C	NA	INTERGENIC	NA
Kabuli_wild_48498133	1399938276	*Ca_kabuli_Chr04*	48498133	C/T	Gene_Ca_10741	DOWNSTREAM	Eukaryotic initiation factor 3, gamma subunit
Kabuli_wild_48498166	1399938277	*Ca_kabuli_Chr04*	48498166	A/G	Gene_Ca_10741	DOWNSTREAM	Eukaryotic initiation factor 3, gamma subunit
Kabuli_wild_48498181	1399938278	*Ca_kabuli_Chr04*	48498181	G/C	Gene_Ca_10742	DOWNSTREAM	TB2/DP1/HVA22-relatedprotein
Kabuli_wild_48498312	1399938279	*Ca_kabuli_Chr04*	48498312	T/C	Gene_Ca_10742	DOWNSTREAM	TB2/DP1/HVA22-relatedprotein
Kabuli_wild_48498331	1399938280	*Ca_kabuli_Chr04*	48498331	T/A	Gene_Ca_10742	DOWNSTREAM	TB2/DP1/HVA22-relatedprotein
Kabuli_wild_48498334	1399938281	*Ca_kabuli_Chr04*	48498334	T/G	Gene_Ca_10742	DOWNSTREAM	TB2/DP1/HVA22-relatedprotein
Kabuli_wild_48498368	1399938282	*Ca_kabuli_Chr04*	48498368	C/T	Gene_Ca_10742	DOWNSTREAM	TB2/DP1/HVA22-relatedprotein
Kabuli_wild_48501662	1399938283	*Ca_kabuli_Chr04*	48501662	A/T	Gene_Ca_10741	SYNONYMOUS_CODING	Eukaryotic initiation factor 3, gamma subunit
Kabuli_wild_48502554	1399938284	*Ca_kabuli_Chr04*	48502554	G/T	Gene_Ca_10741	INTRON	Eukaryotic initiation factor 3, gamma subunit
Kabuli_wild_48502687	1399938285	*Ca_kabuli_Chr04*	48502687	G/A	Gene_Ca_10741	INTRON	Eukaryotic initiation factor 3, gamma subunit
Kabuli_wild_48502714	1399938286	*Ca_kabuli_Chr04*	48502714	T/C	Gene_Ca_10741	INTRON	Eukaryotic initiation factor 3, gamma subunit
Kabuli_wild_48502718	1399938287	*Ca_kabuli_Chr04*	48502718	G/C	Gene_Ca_10741	INTRON	Eukaryotic initiation factor 3, gamma subunit
Kabuli_wild_48678692	1399938288	*Ca_kabuli_Chr04*	48678692	T/C	Gene_Ca_23018	SYNONYMOUS_CODING	Transcription factor IIS,N-terminal
Kabuli_wild_48688766	1399938289	*Ca_kabuli_Chr04*	48688766	A/G	Gene_Ca_23019	SYNONYMOUS_CODING	C2 calcium-dependent membrane targeting
Kabuli_wild_48705487	1399938290	*Ca_kabuli_Chr04*	48705487	T/C	Gene_Ca_23020	INTRON	ABC transporter,trans membrane domain
Kabuli_wild_48714847	1399938291	*Ca_kabuli_Chr04*	48714847	A/T	Gene_Ca_23021	INTRON	Vacuolar protein sorting-associated protein 35
Kabuli_wild_48714912	1399938292	*Ca_kabuli_Chr04*	48714912	G/A	Gene_Ca_23021	INTRON	Vacuolar protein sorting-associated protein 35
Kabuli_wild_48715028	1399938293	*Ca_kabuli_Chr04*	48715028	A/G	Gene_Ca_23021	INTRON	Vacuolar protein sorting-associated protein 35
Kabuli_wild_48720266	1399938294	*Ca_kabuli_Chr04*	48720266	C/T	NA	INTERGENIC	NA
Kabuli_wild_48720330	1399938295	*Ca_kabuli_Chr04*	48720330	C/T	NA	INTERGENIC	NA
Kabuli_wild_48765860	1399938296	*Ca_kabuli_Chr04*	48765860	C/A	Gene_Ca_23023	INTRON	Lipase,GDSL
Kabuli_wild_48784151	1399938297	*Ca_kabuli_Chr04*	48784151	T/G	NA	INTERGENIC	NA
Kabuli_wild_48784176	1399938298	*Ca_kabuli_Chr04*	48784176	T/C	NA	INTERGENIC	NA
Kabuli_wild_48784178	1399938299	*Ca_kabuli_Chr04*	48784178	C/G	NA	INTERGENIC	NA
Kabuli_wild_48784179	1399938300	*Ca_kabuli_Chr04*	48784179	G/A	NA	INTERGENIC	NA
Kabuli_wild_48784189	1399938301	*Ca_kabuli_Chr04*	48784189	A/T	NA	INTERGENIC	NA
Kabuli_wild_48784202	1399938302	*Ca_kabuli_Chr04*	48784202	A/C	NA	INTERGENIC	NA
Kabuli_wild_48784204	1399938303	*Ca_kabuli_Chr04*	48784204	G/C	NA	INTERGENIC	NA
Kabuli_wild_48936654	1399938304	*Ca_kabuli_Chr04*	48936654	A/C	NA	INTERGENIC	NA
Kabuli_wild_48936819	1399938305	*Ca_kabuli_Chr04*	48936819	C/T	NA	INTERGENIC	NA
Kabuli_wild_49012756	1399938306	*Ca_kabuli_Chr04*	49012756	C/T	Gene_Ca_20771	DOWNSTREAM	Glycosidehydrolase,family13
Kabuli_wild_49080099	1399938307	*Ca_kabuli_Chr04*	49080099	A/G	Gene_Ca_20777	NON_SYNONYMOUS_CODING	3-betahydroxysteroiddehydrogenase/isomerase
Kabuli_wild_131558	1399938308	*Ca_kabuli_Chr05*	131558	A/C	Gene_Ca_18168	DOWNSTREAM	NA
Kabuli_wild_154084	1399938309	*Ca_kabuli_Chr05*	154084	T/G	NA	INTERGENIC	NA
Kabuli_wild_154095	1399938310	*Ca_kabuli_Chr05*	154095	C/G	NA	INTERGENIC	NA
Kabuli_wild_154097	1399938311	*Ca_kabuli_Chr05*	154097	A/T	NA	INTERGENIC	NA
Kabuli_wild_154128	1399938312	*Ca_kabuli_Chr05*	154128	G/T	NA	INTERGENIC	NA
Kabuli_wild_154162	1399938313	*Ca_kabuli_Chr05*	154162	C/T	NA	INTERGENIC	NA
Kabuli_wild_166259	1399938314	*Ca_kabuli_Chr05*	166259	A/T	Gene_Ca_18171	NON_SYNONYMOUS_CODING	No apical meristem (NAM) protein
Kabuli_wild_166261	1399938315	*Ca_kabuli_Chr05*	166261	G/T	Gene_Ca_18171	SYNONYMOUS_CODING	No apical meristem (NAM) protein

SNP IDs	NCBI dbSNP accession IDs (NCBI_SS#)	Chromosomes	Physical positions (bp)	SNPs	Gene accession IDs	Sequence components of genome	Putative functions
Kabuli_wild_166341	1399938316	*Ca_kabuli_Chr05*	166341	C/A	Gene_Ca_18171	NON_SYNONYMOUS_CODING	No apical meristem (NAM) protein
Kabuli_wild_166381	1399938317	*Ca_kabuli_Chr05*	166381	A/G	Gene_Ca_18171	SYNONYMOUS_CODING	No apical meristem (NAM) protein
Kabuli_wild_210649	1399938318	*Ca_kabuli_Chr05*	210649	C/A	Gene_Ca_18176	DOWNSTREAM	F-boxdomain,cyclin-like
Kabuli_wild_222637	1399938319	*Ca_kabuli_Chr05*	222637	C/T	NA	INTERGENIC	NA
Kabuli_wild_222641	1399938320	*Ca_kabuli_Chr05*	222641	C/T	NA	INTERGENIC	NA
Kabuli_wild_222651	1399938321	*Ca_kabuli_Chr05*	222651	A/C	NA	INTERGENIC	NA
Kabuli_wild_222653	1399938322	*Ca_kabuli_Chr05*	222653	A/C	NA	INTERGENIC	NA
Kabuli_wild_222657	1399938323	*Ca_kabuli_Chr05*	222657	G/A	NA	INTERGENIC	NA
Kabuli_wild_223384	1399938324	*Ca_kabuli_Chr05*	223384	C/G	Gene_Ca_18177	SYNONYMOUS_CODING	ZF-HD homeobox protein,Cys/His-rich dimerisation domain
Kabuli_wild_234247	1399938325	*Ca_kabuli_Chr05*	234247	T/C	Gene_Ca_18178	INTRON	Protein of unknown function DUF647
Kabuli_wild_234302	1399938326	*Ca_kabuli_Chr05*	234302	T/A	Gene_Ca_18178	INTRON	Protein of unknown function DUF647
Kabuli_wild_234303	1399938327	*Ca_kabuli_Chr05*	234303	C/A	Gene_Ca_18178	INTRON	Protein of unknown function DUF647
Kabuli_wild_234305	1399938328	*Ca_kabuli_Chr05*	234305	T/A	Gene_Ca_18178	INTRON	Protein of unknown function DUF647
Kabuli_wild_234311	1399938329	*Ca_kabuli_Chr05*	234311	T/A	Gene_Ca_18178	INTRON	Protein of unknown function DUF647
Kabuli_wild_234319	1399938330	*Ca_kabuli_Chr05*	234319	T/A	Gene_Ca_18178	INTRON	Protein of unknown function DUF647
Kabuli_wild_234323	1399938331	*Ca_kabuli_Chr05*	234323	T/A	Gene_Ca_18178	INTRON	Protein of unknown function DUF647
Kabuli_wild_234410	1399938332	*Ca_kabuli_Chr05*	234410	A/C	Gene_Ca_18178	NON_SYNONYMOUS_CODING	Protein of unknown function DUF647
Kabuli_wild_235757	1399938333	*Ca_kabuli_Chr05*	235757	T/G	Gene_Ca_18178	SYNONYMOUS_CODING	Protein of unknown function DUF647
Kabuli_wild_235850	1399938334	*Ca_kabuli_Chr05*	235850	A/C	Gene_Ca_18178	SYNONYMOUS_CODING	Protein of unknown function DUF647
Kabuli_wild_245850	1399938335	*Ca_kabuli_Chr05*	245850	T/G	Gene_Ca_18179	INTRON	HEAT
Kabuli_wild_245863	1399938336	*Ca_kabuli_Chr05*	245863	A/T	Gene_Ca_18179	INTRON	HEAT
Kabuli_wild_245896	1399938337	*Ca_kabuli_Chr05*	245896	A/T	Gene_Ca_18179	INTRON	HEAT
Kabuli_wild_246466	1399938338	*Ca_kabuli_Chr05*	246466	A/T	Gene_Ca_18179	INTRON	HEAT
Kabuli_wild_272863	1399938339	*Ca_kabuli_Chr05*	272863	A/T	Gene_Ca_18181	INTRON	Mitoticcheckpoint
Kabuli_wild_272972	1399938340	*Ca_kabuli_Chr05*	272972	T/A	Gene_Ca_18181	INTRON	Mitoticcheckpoint
Kabuli_wild_277040	1399938341	*Ca_kabuli_Chr05*	277040	G/A	Gene_Ca_18181	SYNONYMOUS_CODING	Mitoticcheckpoint
Kabuli_wild_314007	1399938342	*Ca_kabuli_Chr05*	314007	G/C	Gene_Ca_18187	NON_SYNONYMOUS_CODING	Dynamincentral domain
Kabuli_wild_438442	1399938343	*Ca_kabuli_Chr05*	438442	T/C	Gene_Ca_18196	INTRON	NA
Kabuli_wild_438445	1399938344	*Ca_kabuli_Chr05*	438445	C/A	Gene_Ca_18196	INTRON	NA
Kabuli_wild_438448	1399938345	*Ca_kabuli_Chr05*	438448	C/A	Gene_Ca_18196	INTRON	NA
Kabuli_wild_443309	1399938346	*Ca_kabuli_Chr05*	443309	A/T	NA	INTERGENIC	NA
Kabuli_wild_468414	1399938347	*Ca_kabuli_Chr05*	468414	C/A	Gene_Ca_18200	NON_SYNONYMOUS_CODING	Inositolpolyphosphate-relatedphosphatase
Kabuli_wild_469397	1399938348	*Ca_kabuli_Chr05*	469397	G/A	Gene_Ca_18200	SYNONYMOUS_CODING	Inositolpolyphosphate-relatedphosphatase
Kabuli_wild_665998	1399938349	*Ca_kabuli_Chr05*	665998	A/G	Gene_Ca_23223	SYNONYMOUS_CODING	NA
Kabuli_wild_665999	1399938350	*Ca_kabuli_Chr05*	665999	C/A	Gene_Ca_23223	NON_SYNONYMOUS_CODING	NA
Kabuli_wild_666057	1399938351	*Ca_kabuli_Chr05*	666057	C/T	Gene_Ca_23223	SYNONYMOUS_CODING	NA
Kabuli_wild_666078	1399938352	*Ca_kabuli_Chr05*	666078	T/A	Gene_Ca_23223	SYNONYMOUS_CODING	NA
Kabuli_wild_666103	1399938353	*Ca_kabuli_Chr05*	666103	A/T	Gene_Ca_23223	NON_SYNONYMOUS_CODING	NA
Kabuli_wild_666123	1399938354	*Ca_kabuli_Chr05*	666123	G/A	Gene_Ca_23223	SYNONYMOUS_CODING	NA
Kabuli_wild_805859	1399938355	*Ca_kabuli_Chr05*	805859	C/T	NA	INTERGENIC	NA
Kabuli_wild_805860	1399938356	*Ca_kabuli_Chr05*	805860	A/G	NA	INTERGENIC	NA
Kabuli_wild_805896	1399938357	*Ca_kabuli_Chr05*	805896	T/C	NA	INTERGENIC	NA
Kabuli_wild_805929	1399938358	*Ca_kabuli_Chr05*	805929	G/A	NA	INTERGENIC	NA
Kabuli_wild_805961	1399938359	*Ca_kabuli_Chr05*	805961	T/G	NA	INTERGENIC	NA

SNP IDs	NCBI dbSNP accession IDs (NCBI_SS#)	Chromosomes	Physical positions (bp)	SNPs	Gene accession IDs	Sequence components of genome	Putative functions
Kabuli_wild_870867	1399938360	*Ca_kabuli_Chr05*	870867	A/G	NA	INTERGENIC	NA
Kabuli_wild_870909	1399938361	*Ca_kabuli_Chr05*	870909	G/A	NA	INTERGENIC	NA
Kabuli_wild_925554	1399938362	*Ca_kabuli_Chr05*	925554	T/C	NA	INTERGENIC	NA
Kabuli_wild_1141746	1399938363	*Ca_kabuli_Chr05*	1141746	C/T	Gene_Ca_23234	NON_SYNONYMOUS_CODING	NA
Kabuli_wild_1141753	1399938364	*Ca_kabuli_Chr05*	1141753	T/C	Gene_Ca_23234	SYNONYMOUS_CODING	NA
Kabuli_wild_1141755	1399938365	*Ca_kabuli_Chr05*	1141755	C/T	Gene_Ca_23234	NON_SYNONYMOUS_CODING	NA
Kabuli_wild_1141758	1399938366	*Ca_kabuli_Chr05*	1141758	G/A	Gene_Ca_23234	NON_SYNONYMOUS_CODING	NA
Kabuli_wild_1141772	1399938367	*Ca_kabuli_Chr05*	1141772	G/T	Gene_Ca_23234	NON_SYNONYMOUS_CODING	NA
Kabuli_wild_1141785	1399938368	*Ca_kabuli_Chr05*	1141785	C/T	Gene_Ca_23234	NON_SYNONYMOUS_CODING	NA
Kabuli_wild_1220867	1399938369	*Ca_kabuli_Chr05*	1220867	A/G	Gene_Ca_26284	INTRON	YbaK/aminoacyl-tRNAsynthetase-associateddomain
Kabuli_wild_1221219	1399938370	*Ca_kabuli_Chr05*	1221219	A/C	Gene_Ca_26284	INTRON	YbaK/aminoacyl-tRNAsynthetase-associateddomain
Kabuli_wild_1221260	1399938371	*Ca_kabuli_Chr05*	1221260	A/T	Gene_Ca_26284	INTRON	YbaK/aminoacyl-tRNAsynthetase-associateddomain
Kabuli_wild_1252388	1399938372	*Ca_kabuli_Chr05*	1252388	A/T	Gene_Ca_26282	INTRON	MORNmotif
Kabuli_wild_1270830	1399938373	*Ca_kabuli_Chr05*	1270830	T/C	Gene_Ca_26280	SYNONYMOUS_CODING	1-aminocyclopropane-1-carboxylatesynthase
Kabuli_wild_1272581	1399938374	*Ca_kabuli_Chr05*	1272581	T/C	Gene_Ca_26280	UPSTREAM	1-aminocyclopropane-1-carboxylatesynthase
Kabuli_wild_1280977	1399938375	*Ca_kabuli_Chr05*	1280977	T/G	Gene_Ca_26279	NON_SYNONYMOUS_CODING	Transcription facto rGRAS
Kabuli_wild_1281017	1399938376	*Ca_kabuli_Chr05*	1281017	G/A	Gene_Ca_26279	SYNONYMOUS_CODING	Transcription facto rGRAS
Kabuli_wild_1281021	1399938377	*Ca_kabuli_Chr05*	1281021	C/T	Gene_Ca_26279	SYNONYMOUS_CODING	Transcription facto rGRAS
Kabuli_wild_1418656	1399938378	*Ca_kabuli_Chr05*	1418656	C/T	NA	INTERGENIC	NA
Kabuli_wild_1495676	1399938379	*Ca_kabuli_Chr05*	1495676	C/A	Gene_Ca_26446	NON_SYNONYMOUS_CODING	Peptidase C48, SUMO/Sentrin/Ubl1
Kabuli_wild_1495701	1399938380	*Ca_kabuli_Chr05*	1495701	T/C	Gene_Ca_26446	SYNONYMOUS_CODING	Peptidase C48, SUMO/Sentrin/Ubl1
Kabuli_wild_1495735	1399938381	*Ca_kabuli_Chr05*	1495735	C/A	Gene_Ca_26446	NON_SYNONYMOUS_CODING	Peptidase C48, SUMO/Sentrin/Ubl1
Kabuli_wild_1495742	1399938382	*Ca_kabuli_Chr05*	1495742	G/A	Gene_Ca_26446	NON_SYNONYMOUS_CODING	Peptidase C48, SUMO/Sentrin/Ubl1
Kabuli_wild_1524631	1399938383	*Ca_kabuli_Chr05*	1524631	T/C	NA	INTERGENIC	NA
Kabuli_wild_1524651	1399938384	*Ca_kabuli_Chr05*	1524651	T/A	NA	INTERGENIC	NA
Kabuli_wild_1534820	1399938385	*Ca_kabuli_Chr05*	1534820	C/G	NA	INTERGENIC	NA
Kabuli_wild_1534883	1399938386	*Ca_kabuli_Chr05*	1534883	C/T	NA	INTERGENIC	NA
Kabuli_wild_1534907	1399938387	*Ca_kabuli_Chr05*	1534907	T/C	NA	INTERGENIC	NA
Kabuli_wild_1534935	1399938388	*Ca_kabuli_Chr05*	1534935	C/T	NA	INTERGENIC	NA
Kabuli_wild_1534937	1399938389	*Ca_kabuli_Chr05*	1534937	G/A	NA	INTERGENIC	NA
Kabuli_wild_1575600	1399938390	*Ca_kabuli_Chr05*	1575600	C/A	Gene_Ca_26447	NON_SYNONYMOUS_CODING	NA
Kabuli_wild_1585268	1399938391	*Ca_kabuli_Chr05*	1585268	C/A	NA	INTERGENIC	NA
Kabuli_wild_1666880	1399938392	*Ca_kabuli_Chr05*	1666880	G/A	NA	INTERGENIC	NA
Kabuli_wild_2300030	1399938393	*Ca_kabuli_Chr05*	2300030	C/A	Gene_Ca_26508	NON_SYNONYMOUS_CODING	Peptidase C48, SUMO/Sentrin/Ubl1
Kabuli_wild_2380051	1399938394	*Ca_kabuli_Chr05*	2380051	A/G	Gene_Ca_18664	INTRON	NA
Kabuli_wild_2472198	1399938395	*Ca_kabuli_Chr05*	2472198	C/T	Gene_Ca_18665	NON_SYNONYMOUS_CODING	NA
Kabuli_wild_2519949	1399938396	*Ca_kabuli_Chr05*	2519949	C/T	NA	INTERGENIC	NA
Kabuli_wild_2519960	1399938397	*Ca_kabuli_Chr05*	2519960	G/A	NA	INTERGENIC	NA
Kabuli_wild_2519961	1399938398	*Ca_kabuli_Chr05*	2519961	G/A	NA	INTERGENIC	NA
Kabuli_wild_2519977	1399938399	*Ca_kabuli_Chr05*	2519977	C/T	NA	INTERGENIC	NA
Kabuli_wild_2519991	1399938400	*Ca_kabuli_Chr05*	2519991	C/T	NA	INTERGENIC	NA
Kabuli_wild_2519996	1399938401	*Ca_kabuli_Chr05*	2519996	G/T	NA	INTERGENIC	NA
Kabuli_wild_2520015	1399938402	*Ca_kabuli_Chr05*	2520015	C/G	NA	INTERGENIC	NA
Kabuli_wild_2520037	1399938403	*Ca_kabuli_Chr05*	2520037	A/G	NA	INTERGENIC	NA

SNP IDs	NCBI dbSNP accession IDs (NCBI_SS#)	Chromosomes	Physical positions (bp)	SNPs	Gene accession IDs	Sequence components of genome	Putative functions
Kabuli_wild_2520048	1399938404	*Ca_kabuli_Chr05*	2520048	T/C	NA	INTERGENIC	NA
Kabuli_wild_2520049	1399938405	*Ca_kabuli_Chr05*	2520049	C/T	NA	INTERGENIC	NA
Kabuli_wild_2520050	1399938406	*Ca_kabuli_Chr05*	2520050	G/C	NA	INTERGENIC	NA
Kabuli_wild_2520051	1399938407	*Ca_kabuli_Chr05*	2520051	G/T	NA	INTERGENIC	NA
Kabuli_wild_2520055	1399938408	*Ca_kabuli_Chr05*	2520055	C/T	NA	INTERGENIC	NA
Kabuli_wild_2520058	1399938409	*Ca_kabuli_Chr05*	2520058	C/T	NA	INTERGENIC	NA
Kabuli_wild_2520061	1399938410	*Ca_kabuli_Chr05*	2520061	C/A	NA	INTERGENIC	NA
Kabuli_wild_2520067	1399938411	*Ca_kabuli_Chr05*	2520067	C/A	NA	INTERGENIC	NA
Kabuli_wild_2520068	1399938412	*Ca_kabuli_Chr05*	2520068	T/A	NA	INTERGENIC	NA
Kabuli_wild_2520069	1399938413	*Ca_kabuli_Chr05*	2520069	C/T	NA	INTERGENIC	NA
Kabuli_wild_2520070	1399938414	*Ca_kabuli_Chr05*	2520070	C/T	NA	INTERGENIC	NA
Kabuli_wild_2520093	1399938415	*Ca_kabuli_Chr05*	2520093	G/A	NA	INTERGENIC	NA
Kabuli_wild_2520094	1399938416	*Ca_kabuli_Chr05*	2520094	G/A	NA	INTERGENIC	NA
Kabuli_wild_2520101	1399938417	*Ca_kabuli_Chr05*	2520101	C/T	NA	INTERGENIC	NA
Kabuli_wild_2520109	1399938418	*Ca_kabuli_Chr05*	2520109	G/A	NA	INTERGENIC	NA
Kabuli_wild_2520114	1399938419	*Ca_kabuli_Chr05*	2520114	G/A	NA	INTERGENIC	NA
Kabuli_wild_2520136	1399938420	*Ca_kabuli_Chr05*	2520136	T/C	NA	INTERGENIC	NA
Kabuli_wild_2613423	1399938421	*Ca_kabuli_Chr05*	2613423	T/G	NA	INTERGENIC	NA
Kabuli_wild_2948530	1399938422	*Ca_kabuli_Chr05*	2948530	G/T	Gene_Ca_18681	INTRON	NA
Kabuli_wild_2948531	1399938423	*Ca_kabuli_Chr05*	2948531	T/C	Gene_Ca_18681	INTRON	NA
Kabuli_wild_2948549	1399938424	*Ca_kabuli_Chr05*	2948549	C/A	Gene_Ca_18681	INTRON	NA
Kabuli_wild_2948592	1399938425	*Ca_kabuli_Chr05*	2948592	T/C	Gene_Ca_18681	INTRON	NA
Kabuli_wild_2948616	1399938426	*Ca_kabuli_Chr05*	2948616	G/A	Gene_Ca_18681	INTRON	NA
Kabuli_wild_2948688	1399938427	*Ca_kabuli_Chr05*	2948688	C/T	Gene_Ca_18681	INTRON	NA
Kabuli_wild_2948689	1399938428	*Ca_kabuli_Chr05*	2948689	A/G	Gene_Ca_18681	INTRON	NA
Kabuli_wild_2948693	1399938429	*Ca_kabuli_Chr05*	2948693	G/T	Gene_Ca_18681	INTRON	NA
Kabuli_wild_2948716	1399938430	*Ca_kabuli_Chr05*	2948716	G/A	Gene_Ca_18681	INTRON	NA
Kabuli_wild_2948726	1399938431	*Ca_kabuli_Chr05*	2948726	C/T	Gene_Ca_18681	INTRON	NA
Kabuli_wild_2948736	1399938432	*Ca_kabuli_Chr05*	2948736	A/G	Gene_Ca_18681	INTRON	NA
Kabuli_wild_3348310	1399938433	*Ca_kabuli_Chr05*	3348310	A/G	NA	INTERGENIC	NA
Kabuli_wild_3348328	1399938434	*Ca_kabuli_Chr05*	3348328	C/A	NA	INTERGENIC	NA
Kabuli_wild_3348366	1399938435	*Ca_kabuli_Chr05*	3348366	G/C	NA	INTERGENIC	NA
Kabuli_wild_3353013	1399938436	*Ca_kabuli_Chr05*	3353013	T/G	NA	INTERGENIC	NA
Kabuli_wild_3353052	1399938437	*Ca_kabuli_Chr05*	3353052	A/G	NA	INTERGENIC	NA
Kabuli_wild_3353055	1399938438	*Ca_kabuli_Chr05*	3353055	G/A	NA	INTERGENIC	NA
Kabuli_wild_3353056	1399938439	*Ca_kabuli_Chr05*	3353056	C/A	NA	INTERGENIC	NA
Kabuli_wild_3643160	1399938440	*Ca_kabuli_Chr05*	3643160	C/T	NA	INTERGENIC	NA
Kabuli_wild_3643216	1399938441	*Ca_kabuli_Chr05*	3643216	C/T	NA	INTERGENIC	NA
Kabuli_wild_3643241	1399938442	*Ca_kabuli_Chr05*	3643241	G/A	NA	INTERGENIC	NA
Kabuli_wild_3838136	1399938443	*Ca_kabuli_Chr05*	3838136	A/G	NA	INTERGENIC	NA
Kabuli_wild_3838149	1399938444	*Ca_kabuli_Chr05*	3838149	C/A	NA	INTERGENIC	NA
Kabuli_wild_4042797	1399938445	*Ca_kabuli_Chr05*	4042797	A/C	NA	INTERGENIC	NA
Kabuli_wild_4122342	1399938446	*Ca_kabuli_Chr05*	4122342	T/C	NA	INTERGENIC	NA
Kabuli_wild_4122395	1399938447	*Ca_kabuli_Chr05*	4122395	T/C	NA	INTERGENIC	NA

SNP IDs	NCBI dbSNP accession IDs (NCBI_SS#)	Chromosomes	Physical positions (bp)	SNPs	Gene accession IDs	Sequence components of genome	Putative functions
Kabuli_wild_4200223	1399938448	*Ca_kabuli_Chr05*	4200223	A/C	NA	INTERGENIC	NA
Kabuli_wild_4890611	1399938449	*Ca_kabuli_Chr05*	4890611	C/T	NA	INTERGENIC	NA
Kabuli_wild_4928256	1399938450	*Ca_kabuli_Chr05*	4928256	A/G	NA	INTERGENIC	NA
Kabuli_wild_4928311	1399938451	*Ca_kabuli_Chr05*	4928311	T/C	NA	INTERGENIC	NA
Kabuli_wild_4928373	1399938452	*Ca_kabuli_Chr05*	4928373	A/C	NA	INTERGENIC	NA
Kabuli_wild_4928382	1399938453	*Ca_kabuli_Chr05*	4928382	G/T	NA	INTERGENIC	NA
Kabuli_wild_4938033	1399938454	*Ca_kabuli_Chr05*	4938033	C/T	NA	INTERGENIC	NA
Kabuli_wild_4938039	1399938455	*Ca_kabuli_Chr05*	4938039	A/G	NA	INTERGENIC	NA
Kabuli_wild_5133643	1399938456	*Ca_kabuli_Chr05*	5133643	A/T	Gene_Ca_25551	INTRON	Zinc finger,U1-type
Kabuli_wild_5133644	1399938457	*Ca_kabuli_Chr05*	5133644	A/G	Gene_Ca_25551	INTRON	Zinc finger,U1-type
Kabuli_wild_5404114	1399938458	*Ca_kabuli_Chr05*	5404114	A/G	NA	INTERGENIC	NA
Kabuli_wild_5404199	1399938459	*Ca_kabuli_Chr05*	5404199	T/C	NA	INTERGENIC	NA
Kabuli_wild_5404210	1399938460	*Ca_kabuli_Chr05*	5404210	A/G	NA	INTERGENIC	NA
Kabuli_wild_5404261	1399938461	*Ca_kabuli_Chr05*	5404261	G/A	NA	INTERGENIC	NA
Kabuli_wild_5647938	1399938462	*Ca_kabuli_Chr05*	5647938	A/C	NA	INTERGENIC	NA
Kabuli_wild_5854899	1399938463	*Ca_kabuli_Chr05*	5854899	T/G	NA	INTERGENIC	NA
Kabuli_wild_6082113	1399938464	*Ca_kabuli_Chr05*	6082113	C/A	Gene_Ca_20732	DOWNSTREAM	Transposase,Ptta/En/Spm,plant
Kabuli_wild_6186221	1399938465	*Ca_kabuli_Chr05*	6186221	C/T	Gene_Ca_20736	SYNONYMOUS_CODING	NA
Kabuli_wild_6190494	1399938466	*Ca_kabuli_Chr05*	6190494	T/C	NA	INTERGENIC	NA
Kabuli_wild_6225392	1399938467	*Ca_kabuli_Chr05*	6225392	G/A	NA	INTERGENIC	NA
Kabuli_wild_6225397	1399938468	*Ca_kabuli_Chr05*	6225397	G/A	NA	INTERGENIC	NA
Kabuli_wild_6225410	1399938469	*Ca_kabuli_Chr05*	6225410	A/T	NA	INTERGENIC	NA
Kabuli_wild_6225415	1399938470	*Ca_kabuli_Chr05*	6225415	C/G	NA	INTERGENIC	NA
Kabuli_wild_6225426	1399938471	*Ca_kabuli_Chr05*	6225426	T/A	NA	INTERGENIC	NA
Kabuli_wild_6290453	1399938472	*Ca_kabuli_Chr05*	6290453	G/A	Gene_Ca_20739	INTRON	RNA recognition motif domain
Kabuli_wild_6300337	1399938473	*Ca_kabuli_Chr05*	6300337	C/A	Gene_Ca_20739	INTRON	RNA recognition motif domain
Kabuli_wild_6300502	1399938474	*Ca_kabuli_Chr05*	6300502	C/A	Gene_Ca_20739	INTRON	RNA recognition motif domain
Kabuli_wild_6317014	1399938475	*Ca_kabuli_Chr05*	6317014	C/T	NA	INTERGENIC	NA
Kabuli_wild_6480629	1399938476	*Ca_kabuli_Chr05*	6480629	A/G	NA	INTERGENIC	NA
Kabuli_wild_6539792	1399938477	*Ca_kabuli_Chr05*	6539792	T/A	Gene_Ca_20752	DOWNSTREAM	NA
Kabuli_wild_6539848	1399938478	*Ca_kabuli_Chr05*	6539848	C/A	Gene_Ca_20752	DOWNSTREAM	NA
Kabuli_wild_6654053	1399938479	*Ca_kabuli_Chr05*	6654053	C/A	Gene_Ca_20754	DOWNSTREAM	NA
Kabuli_wild_6654072	1399938480	*Ca_kabuli_Chr05*	6654072	C/T	Gene_Ca_20754	DOWNSTREAM	NA
Kabuli_wild_6805221	1399938481	*Ca_kabuli_Chr05*	6805221	T/A	NA	INTERGENIC	NA
Kabuli_wild_6931865	1399938482	*Ca_kabuli_Chr05*	6931865	A/G	NA	INTERGENIC	NA
Kabuli_wild_7138725	1399938483	*Ca_kabuli_Chr05*	7138725	T/C	NA	INTERGENIC	NA
Kabuli_wild_7214400	1399938484	*Ca_kabuli_Chr05*	7214400	G/A	Gene_Ca_21567	NON_SYNONYMOUS_CODING	Peptidase C48, SUMO/Sentrin/Ubl1
Kabuli_wild_7316705	1399938485	*Ca_kabuli_Chr05*	7316705	A/T	Gene_Ca_21573	UPSTREAM	NA
Kabuli_wild_7316706	1399938486	*Ca_kabuli_Chr05*	7316706	T/C	Gene_Ca_21573	UPSTREAM	NA
Kabuli_wild_7316754	1399938487	*Ca_kabuli_Chr05*	7316754	T/C	Gene_Ca_21573	UPSTREAM	NA
Kabuli_wild_7316809	1399938488	*Ca_kabuli_Chr05*	7316809	T/C	Gene_Ca_21572	DOWNSTREAM	NA
Kabuli_wild_7424999	1399938489	*Ca_kabuli_Chr05*	7424999	G/A	NA	INTERGENIC	NA
Kabuli_wild_7425006	1399938490	*Ca_kabuli_Chr05*	7425006	G/T	NA	INTERGENIC	NA
Kabuli_wild_7425116	1399938491	*Ca_kabuli_Chr05*	7425116	C/T	NA	INTERGENIC	NA

SNP IDs	NCBI dbSNP accession IDs (NCBI_SS#)	Chromosomes	Physical positions (bp)	SNPs	Gene accession IDs	Sequence components of genome	Putative functions
Kabuli_wild_7664421	1399938492	*Ca_kabuli_Chr05*	7664421	T/C	Gene_Ca_18734	NON_SYNONYMOUS_CODING	Regulatorofnonsense-mediateddecay,UPF3
Kabuli_wild_7775298	1399938493	*Ca_kabuli_Chr05*	7775298	A/T	NA	INTERGENIC	NA
Kabuli_wild_7775456	1399938494	*Ca_kabuli_Chr05*	7775456	T/C	NA	INTERGENIC	NA
Kabuli_wild_7775472	1399938495	*Ca_kabuli_Chr05*	7775472	G/A	NA	INTERGENIC	NA
Kabuli_wild_7775497	1399938496	*Ca_kabuli_Chr05*	7775497	G/C	NA	INTERGENIC	NA
Kabuli_wild_7775516	1399938497	*Ca_kabuli_Chr05*	7775516	A/G	NA	INTERGENIC	NA
Kabuli_wild_7775543	1399938498	*Ca_kabuli_Chr05*	7775543	T/C	NA	INTERGENIC	NA
Kabuli_wild_7775915	1399938499	*Ca_kabuli_Chr05*	7775915	A/T	NA	INTERGENIC	NA
Kabuli_wild_7775916	1399938500	*Ca_kabuli_Chr05*	7775916	C/G	NA	INTERGENIC	NA
Kabuli_wild_7775918	1399938501	*Ca_kabuli_Chr05*	7775918	C/T	NA	INTERGENIC	NA
Kabuli_wild_7789021	1399938502	*Ca_kabuli_Chr05*	7789021	G/T	NA	INTERGENIC	NA
Kabuli_wild_7789065	1399938503	*Ca_kabuli_Chr05*	7789065	A/G	NA	INTERGENIC	NA
Kabuli_wild_7826274	1399938504	*Ca_kabuli_Chr05*	7826274	A/G	NA	INTERGENIC	NA
Kabuli_wild_7826338	1399938505	*Ca_kabuli_Chr05*	7826338	T/G	NA	INTERGENIC	NA
Kabuli_wild_7826356	1399938506	*Ca_kabuli_Chr05*	7826356	G/A	NA	INTERGENIC	NA
Kabuli_wild_7826366	1399938507	*Ca_kabuli_Chr05*	7826366	G/A	NA	INTERGENIC	NA
Kabuli_wild_7848711	1399938508	*Ca_kabuli_Chr05*	7848711	C/T	NA	INTERGENIC	NA
Kabuli_wild_7920989	1399938509	*Ca_kabuli_Chr05*	7920989	G/A	NA	INTERGENIC	NA
Kabuli_wild_8052248	1399938510	*Ca_kabuli_Chr05*	8052248	C/T	NA	INTERGENIC	NA
Kabuli_wild_8112387	1399938511	*Ca_kabuli_Chr05*	8112387	C/G	Gene_Ca_18714	NON_SYNONYMOUS_CODING	NA
Kabuli_wild_8263483	1399938512	*Ca_kabuli_Chr05*	8263483	A/G	NA	INTERGENIC	NA
Kabuli_wild_8272359	1399938513	*Ca_kabuli_Chr05*	8272359	G/A	Gene_Ca_18709	NON_SYNONYMOUS_CODING	Protein kinase,catalytic domain
Kabuli_wild_8308830	1399938514	*Ca_kabuli_Chr05*	8308830	T/A	NA	INTERGENIC	NA
Kabuli_wild_8486712	1399938515	*Ca_kabuli_Chr05*	8486712	A/C	NA	INTERGENIC	NA
Kabuli_wild_8535619	1399938516	*Ca_kabuli_Chr05*	8535619	A/C	NA	INTERGENIC	NA
Kabuli_wild_8814637	1399938517	*Ca_kabuli_Chr05*	8814637	T/C	Gene_Ca_20335	NON_SYNONYMOUS_CODING	Chromodomain
Kabuli_wild_9077465	1399938518	*Ca_kabuli_Chr05*	9077465	C/T	Gene_Ca_19962	NON_SYNONYMOUS_CODING	Prohibitin
Kabuli_wild_9121616	1399938519	*Ca_kabuli_Chr05*	9121616	T/C	NA	INTERGENIC	NA
Kabuli_wild_9226404	1399938520	*Ca_kabuli_Chr05*	9226404	A/C	Gene_Ca_19969	INTRON	FMN-dependentdehydrogenase
Kabuli_wild_9483731	1399938521	*Ca_kabuli_Chr05*	9483731	A/T	NA	INTERGENIC	NA
Kabuli_wild_9519382	1399938522	*Ca_kabuli_Chr05*	9519382	C/A	Gene_Ca_23779	DOWNSTREAM	NA
Kabuli_wild_9519390	1399938523	*Ca_kabuli_Chr05*	9519390	G/A	Gene_Ca_23779	DOWNSTREAM	NA
Kabuli_wild_9600407	1399938524	*Ca_kabuli_Chr05*	9600407	C/T	NA	INTERGENIC	NA
Kabuli_wild_9609066	1399938525	*Ca_kabuli_Chr05*	9609066	A/C	Gene_Ca_23788	SYNONYMOUS_CODING	Pyridine nucleotide-disulphideoxido reductase,NAD-binding region
Kabuli_wild_9620998	1399938526	*Ca_kabuli_Chr05*	9620998	C/A	NA	INTERGENIC	NA
Kabuli_wild_9728591	1399938527	*Ca_kabuli_Chr05*	9728591	A/G	NA	INTERGENIC	NA
Kabuli_wild_9728992	1399938528	*Ca_kabuli_Chr05*	9728992	A/T	NA	INTERGENIC	NA
Kabuli_wild_9729039	1399938529	*Ca_kabuli_Chr05*	9729039	G/A	NA	INTERGENIC	NA
Kabuli_wild_9729050	1399938530	*Ca_kabuli_Chr05*	9729050	T/G	NA	INTERGENIC	NA
Kabuli_wild_9729058	1399938531	*Ca_kabuli_Chr05*	9729058	C/T	NA	INTERGENIC	NA
Kabuli_wild_9729070	1399938532	*Ca_kabuli_Chr05*	9729070	G/A	NA	INTERGENIC	NA
Kabuli_wild_9868796	1399938533	*Ca_kabuli_Chr05*	9868796	T/C	Gene_Ca_20504	INTRON	Ankyrinrepeat
Kabuli_wild_9869005	1399938534	*Ca_kabuli_Chr05*	9869005	T/G	Gene_Ca_20504	SYNONYMOUS_CODING	Ankyrinrepeat
Kabuli_wild_9961380	1399938535	*Ca_kabuli_Chr05*	9961380	C/T	Gene_Ca_20508	NON_SYNONYMOUS_CODING	DNA-binding WRKY

SNP IDs	NCBI dbSNP accession IDs (NCBI_SS#)	Chromosomes	Physical positions (bp)	SNPs	Gene accession IDs	Sequence components of genome	Putative functions
Kabuli_wild_9965848	1399938536	*Ca_kabuli_Chr05*	9965848	T/C	NA	INTERGENIC	NA
Kabuli_wild_10004427	1399938537	*Ca_kabuli_Chr05*	10004427	T/G	Gene_Ca_20512	SYNONYMOUS_CODING	NA
Kabuli_wild_10004430	1399938538	*Ca_kabuli_Chr05*	10004430	T/G	Gene_Ca_20512	SYNONYMOUS_CODING	NA
Kabuli_wild_10004451	1399938539	*Ca_kabuli_Chr05*	10004451	T/G	Gene_Ca_20512	SYNONYMOUS_CODING	NA
Kabuli_wild_10072358	1399938540	*Ca_kabuli_Chr05*	10072358	G/A	NA	INTERGENIC	NA
Kabuli_wild_10072403	1399938541	*Ca_kabuli_Chr05*	10072403	G/C	NA	INTERGENIC	NA
Kabuli_wild_10072460	1399938542	*Ca_kabuli_Chr05*	10072460	G/A	NA	INTERGENIC	NA
Kabuli_wild_10293447	1399938543	*Ca_kabuli_Chr05*	10293447	C/A	Gene_Ca_21242	NON_SYNONYMOUS_CODING	Protein kinase,catalytic domain
Kabuli_wild_10318833	1399938544	*Ca_kabuli_Chr05*	10318833	A/G	NA	INTERGENIC	NA
Kabuli_wild_10365031	1399938545	*Ca_kabuli_Chr05*	10365031	C/T	Gene_Ca_21234	SYNONYMOUS_CODING	Dual specificity phosphatase, catalytic domain
Kabuli_wild_10406562	1399938546	*Ca_kabuli_Chr05*	10406562	C/A	NA	INTERGENIC	NA
Kabuli_wild_10406602	1399938547	*Ca_kabuli_Chr05*	10406602	C/T	NA	INTERGENIC	NA
Kabuli_wild_10406636	1399938548	*Ca_kabuli_Chr05*	10406636	A/G	NA	INTERGENIC	NA
Kabuli_wild_10545265	1399938549	*Ca_kabuli_Chr05*	10545265	A/C	Gene_Ca_21227	INTRON	D-isomerspecific2-hydroxyaciddehydrogenase,catalytic domain
Kabuli_wild_10675252	1399938550	*Ca_kabuli_Chr05*	10675252	A/G	NA	INTERGENIC	NA
Kabuli_wild_10675279	1399938551	*Ca_kabuli_Chr05*	10675279	C/G	NA	INTERGENIC	NA
Kabuli_wild_10675294	1399938552	*Ca_kabuli_Chr05*	10675294	A/G	NA	INTERGENIC	NA
Kabuli_wild_10675421	1399938553	*Ca_kabuli_Chr05*	10675421	A/G	NA	INTERGENIC	NA
Kabuli_wild_10858050	1399938554	*Ca_kabuli_Chr05*	10858050	A/G	NA	INTERGENIC	NA
Kabuli_wild_10858490	1399938555	*Ca_kabuli_Chr05*	10858490	C/T	NA	INTERGENIC	NA
Kabuli_wild_10918368	1399938556	*Ca_kabuli_Chr05*	10918368	A/G	Gene_Ca_23750	NON_SYNONYMOUS_CODING	MoeA,C-terminal,domainIV
Kabuli_wild_10919284	1399938557	*Ca_kabuli_Chr05*	10919284	A/G	Gene_Ca_23750	INTRON	MoeA,C-terminal,domainIV
Kabuli_wild_10919289	1399938558	*Ca_kabuli_Chr05*	10919289	C/A	Gene_Ca_23750	INTRON	MoeA,C-terminal,domainIV
Kabuli_wild_10919332	1399938559	*Ca_kabuli_Chr05*	10919332	T/G	Gene_Ca_23750	INTRON	MoeA,C-terminal,domainIV
Kabuli_wild_11191786	1399938560	*Ca_kabuli_Chr05*	11191786	T/C	NA	INTERGENIC	NA
Kabuli_wild_11222728	1399938561	*Ca_kabuli_Chr05*	11222728	T/C	NA	INTERGENIC	NA
Kabuli_wild_11230617	1399938562	*Ca_kabuli_Chr05*	11230617	C/G	NA	INTERGENIC	NA
Kabuli_wild_11230627	1399938563	*Ca_kabuli_Chr05*	11230627	A/G	NA	INTERGENIC	NA
Kabuli_wild_11230648	1399938564	*Ca_kabuli_Chr05*	11230648	G/A	NA	INTERGENIC	NA
Kabuli_wild_11230671	1399938565	*Ca_kabuli_Chr05*	11230671	T/C	NA	INTERGENIC	NA
Kabuli_wild_11230677	1399938566	*Ca_kabuli_Chr05*	11230677	C/T	NA	INTERGENIC	NA
Kabuli_wild_11263571	1399938567	*Ca_kabuli_Chr05*	11263571	C/G	NA	INTERGENIC	NA
Kabuli_wild_11263574	1399938568	*Ca_kabuli_Chr05*	11263574	C/A	NA	INTERGENIC	NA
Kabuli_wild_11317487	1399938569	*Ca_kabuli_Chr05*	11317487	C/T	NA	INTERGENIC	NA
Kabuli_wild_11317502	1399938570	*Ca_kabuli_Chr05*	11317502	C/A	NA	INTERGENIC	NA
Kabuli_wild_11385780	1399938571	*Ca_kabuli_Chr05*	11385780	A/C	Gene_Ca_23356	NON_SYNONYMOUS_CODING	RNA recognition motif domain
Kabuli_wild_11385874	1399938572	*Ca_kabuli_Chr05*	11385874	A/G	Gene_Ca_23356	SYNONYMOUS_CODING	RNA recognition motif domain
Kabuli_wild_11409971	1399938573	*Ca_kabuli_Chr05*	11409971	A/G	Gene_Ca_23355	NON_SYNONYMOUS_CODING	Peptidase C48, SUMO/Sentrin/Ubl1
Kabuli_wild_11637017	1399938574	*Ca_kabuli_Chr05*	11637017	C/T	NA	INTERGENIC	NA
Kabuli_wild_11637056	1399938575	*Ca_kabuli_Chr05*	11637056	G/A	NA	INTERGENIC	NA
Kabuli_wild_11777112	1399938576	*Ca_kabuli_Chr05*	11777112	C/T	NA	INTERGENIC	NA
Kabuli_wild_11777128	1399938577	*Ca_kabuli_Chr05*	11777128	A/T	NA	INTERGENIC	NA
Kabuli_wild_11777145	1399938578	*Ca_kabuli_Chr05*	11777145	A/G	NA	INTERGENIC	NA
Kabuli_wild_11835392	1399938579	*Ca_kabuli_Chr05*	11835392	T/C	NA	INTERGENIC	NA

SNP IDs	NCBI dbSNP accession IDs (NCBI_SS#)	Chromosomes	Physical positions (bp)	SNPs	Gene accession IDs	Sequence components of genome	Putative functions
Kabuli_wild_11835482	1399938580	*Ca_kabuli_Chr05*	11835482	C/T	NA	INTERGENIC	NA
Kabuli_wild_11848105	1399938581	*Ca_kabuli_Chr05*	11848105	A/T	Gene_Ca_17111	INTRON	ABC transporter,trans membrane domain
Kabuli_wild_11853090	1399938582	*Ca_kabuli_Chr05*	11853090	C/T	Gene_Ca_17111	INTRON	ABC transporter,trans membrane domain
Kabuli_wild_11864959	1399938583	*Ca_kabuli_Chr05*	11864959	T/C	Gene_Ca_17110	NON_SYNONYMOUS_CODING	Zinc finger,CCHC-type
Kabuli_wild_11983063	1399938584	*Ca_kabuli_Chr05*	11983063	A/C	Gene_Ca_17105	UPSTREAM	Pentatrico peptide repeat
Kabuli_wild_11983126	1399938585	*Ca_kabuli_Chr05*	11983126	A/T	Gene_Ca_17105	UPSTREAM	Pentatrico peptide repeat
Kabuli_wild_11983214	1399938586	*Ca_kabuli_Chr05*	11983214	A/C	Gene_Ca_17105	UPSTREAM	Pentatrico peptide repeat
Kabuli_wild_12157678	1399938587	*Ca_kabuli_Chr05*	12157678	A/G	NA	INTERGENIC	NA
Kabuli_wild_12157688	1399938588	*Ca_kabuli_Chr05*	12157688	C/T	NA	INTERGENIC	NA
Kabuli_wild_12157699	1399938589	*Ca_kabuli_Chr05*	12157699	G/T	NA	INTERGENIC	NA
Kabuli_wild_12157728	1399938590	*Ca_kabuli_Chr05*	12157728	C/T	NA	INTERGENIC	NA
Kabuli_wild_12157729	1399938591	*Ca_kabuli_Chr05*	12157729	G/A	NA	INTERGENIC	NA
Kabuli_wild_12157783	1399938592	*Ca_kabuli_Chr05*	12157783	T/C	NA	INTERGENIC	NA
Kabuli_wild_12157787	1399938593	*Ca_kabuli_Chr05*	12157787	A/C	NA	INTERGENIC	NA
Kabuli_wild_12162160	1399938594	*Ca_kabuli_Chr05*	12162160	G/A	NA	INTERGENIC	NA
Kabuli_wild_12162205	1399938595	*Ca_kabuli_Chr05*	12162205	A/C	NA	INTERGENIC	NA
Kabuli_wild_12162213	1399938596	*Ca_kabuli_Chr05*	12162213	C/T	NA	INTERGENIC	NA
Kabuli_wild_12210122	1399938597	*Ca_kabuli_Chr05*	12210122	G/A	NA	INTERGENIC	NA
Kabuli_wild_12247539	1399938598	*Ca_kabuli_Chr05*	12247539	C/A	NA	INTERGENIC	NA
Kabuli_wild_12461965	1399938599	*Ca_kabuli_Chr05*	12461965	G/A	NA	INTERGENIC	NA
Kabuli_wild_12463017	1399938600	*Ca_kabuli_Chr05*	12463017	G/A	NA	INTERGENIC	NA
Kabuli_wild_12480948	1399938601	*Ca_kabuli_Chr05*	12480948	T/G	Gene_Ca_17080	INTRON	Choline/ethanolaminekinase
Kabuli_wild_12553469	1399938602	*Ca_kabuli_Chr05*	12553469	A/C	Gene_Ca_17074	UPSTREAM	NA
Kabuli_wild_12553470	1399938603	*Ca_kabuli_Chr05*	12553470	G/C	Gene_Ca_17074	UPSTREAM	NA
Kabuli_wild_12590478	1399938604	*Ca_kabuli_Chr05*	12590478	T/C	Gene_Ca_17072	SYNONYMOUS_CODING	Glycerophosphoryldiester phosphodiesterase
Kabuli_wild_12590512	1399938605	*Ca_kabuli_Chr05*	12590512	G/A	Gene_Ca_17072	SYNONYMOUS_CODING	Glycerophosphoryldiester phosphodiesterase
Kabuli_wild_12648059	1399938606	*Ca_kabuli_Chr05*	12648059	A/C	Gene_Ca_17067	NON_SYNONYMOUS_CODING	Protein kinase,catalytic domain
Kabuli_wild_12662138	1399938607	*Ca_kabuli_Chr05*	12662138	G/T	Gene_Ca_17066	INTRON	Protein kinase,catalytic domain
Kabuli_wild_13982000	1399938608	*Ca_kabuli_Chr05*	13982000	T/G	Gene_Ca_20821	NON_SYNONYMOUS_CODING	Protein kinase,catalytic domain
Kabuli_wild_13982041	1399938609	*Ca_kabuli_Chr05*	13982041	C/T	Gene_Ca_20821	SYNONYMOUS_CODING	Protein kinase,catalytic domain
Kabuli_wild_14108868	1399938610	*Ca_kabuli_Chr05*	14108868	C/T	Gene_Ca_20826	DOWNSTREAM	Short-chaindehydrogenase/reductaseSDR
Kabuli_wild_14425390	1399938611	*Ca_kabuli_Chr05*	14425390	C/T	NA	INTERGENIC	NA
Kabuli_wild_14425420	1399938612	*Ca_kabuli_Chr05*	14425420	G/A	NA	INTERGENIC	NA
Kabuli_wild_14425421	1399938613	*Ca_kabuli_Chr05*	14425421	C/T	NA	INTERGENIC	NA
Kabuli_wild_15476405	1399938614	*Ca_kabuli_Chr05*	15476405	T/A	Gene_Ca_21644	DOWNSTREAM	NA
Kabuli_wild_15524860	1399938615	*Ca_kabuli_Chr05*	15524860	A/G	NA	INTERGENIC	NA
Kabuli_wild_15524885	1399938616	*Ca_kabuli_Chr05*	15524885	C/G	NA	INTERGENIC	NA
Kabuli_wild_15524904	1399938617	*Ca_kabuli_Chr05*	15524904	C/A	NA	INTERGENIC	NA
Kabuli_wild_15524907	1399938618	*Ca_kabuli_Chr05*	15524907	G/C	NA	INTERGENIC	NA
Kabuli_wild_15780122	1399938619	*Ca_kabuli_Chr05*	15780122	T/A	Gene_Ca_26865	INTRON	RNA recognition motif domain
Kabuli_wild_15952395	1399938620	*Ca_kabuli_Chr05*	15952395	C/T	NA	INTERGENIC	NA
Kabuli_wild_16152881	1399938621	*Ca_kabuli_Chr05*	16152881	C/T	NA	INTERGENIC	NA
Kabuli_wild_16152933	1399938622	*Ca_kabuli_Chr05*	16152933	A/C	NA	INTERGENIC	NA
Kabuli_wild_16152934	1399938623	*Ca_kabuli_Chr05*	16152934	G/A	NA	INTERGENIC	NA

SNP IDs	NCBI dbSNP accession IDs (NCBI_SS#)	Chromosomes	Physical positions (bp)	SNPs	Gene accession IDs	Sequence components of genome	Putative functions
Kabuli_wild_16152935	1399938624	*Ca_kabuli_Chr05*	16152935	C/T	NA	INTERGENIC	NA
Kabuli_wild_16227319	1399938625	*Ca_kabuli_Chr05*	16227319	G/T	Gene_Ca_17915	SYNONYMOUS_CODING	Peptidase C48, SUMO/Sentrin/Ubl1
Kabuli_wild_16227342	1399938626	*Ca_kabuli_Chr05*	16227342	T/G	Gene_Ca_17915	NON_SYNONYMOUS_CODING	Peptidase C48, SUMO/Sentrin/Ubl1
Kabuli_wild_16227353	1399938627	*Ca_kabuli_Chr05*	16227353	G/A	Gene_Ca_17915	NON_SYNONYMOUS_CODING	Peptidase C48, SUMO/Sentrin/Ubl1
Kabuli_wild_16227355	1399938628	*Ca_kabuli_Chr05*	16227355	C/T	Gene_Ca_17915	SYNONYMOUS_CODING	Peptidase C48, SUMO/Sentrin/Ubl1
Kabuli_wild_16227357	1399938629	*Ca_kabuli_Chr05*	16227357	C/A	Gene_Ca_17915	NON_SYNONYMOUS_CODING	Peptidase C48, SUMO/Sentrin/Ubl1
Kabuli_wild_16227358	1399938630	*Ca_kabuli_Chr05*	16227358	G/A	Gene_Ca_17915	SYNONYMOUS_CODING	Peptidase C48, SUMO/Sentrin/Ubl1
Kabuli_wild_16227371	1399938631	*Ca_kabuli_Chr05*	16227371	C/T	Gene_Ca_17915	NON_SYNONYMOUS_CODING	Peptidase C48, SUMO/Sentrin/Ubl1
Kabuli_wild_16227401	1399938632	*Ca_kabuli_Chr05*	16227401	G/A	Gene_Ca_17915	NON_SYNONYMOUS_CODING	Peptidase C48, SUMO/Sentrin/Ubl1
Kabuli_wild_16227402	1399938633	*Ca_kabuli_Chr05*	16227402	C/T	Gene_Ca_17915	NON_SYNONYMOUS_CODING	Peptidase C48, SUMO/Sentrin/Ubl1
Kabuli_wild_16227404	1399938634	*Ca_kabuli_Chr05*	16227404	G/A	Gene_Ca_17915	NON_SYNONYMOUS_CODING	Peptidase C48, SUMO/Sentrin/Ubl1
Kabuli_wild_16227449	1399938635	*Ca_kabuli_Chr05*	16227449	G/A	Gene_Ca_17915	NON_SYNONYMOUS_CODING	Peptidase C48, SUMO/Sentrin/Ubl1
Kabuli_wild_16227460	1399938636	*Ca_kabuli_Chr05*	16227460	A/T	Gene_Ca_17915	SYNONYMOUS_CODING	Peptidase C48, SUMO/Sentrin/Ubl1
Kabuli_wild_16227466	1399938637	*Ca_kabuli_Chr05*	16227466	C/T	Gene_Ca_17915	SYNONYMOUS_CODING	Peptidase C48, SUMO/Sentrin/Ubl1
Kabuli_wild_16227468	1399938638	*Ca_kabuli_Chr05*	16227468	G/A	Gene_Ca_17915	SYNONYMOUS_CODING	Peptidase C48, SUMO/Sentrin/Ubl1
Kabuli_wild_16227485	1399938639	*Ca_kabuli_Chr05*	16227485	G/T	Gene_Ca_17915	NON_SYNONYMOUS_CODING	Peptidase C48, SUMO/Sentrin/Ubl1
Kabuli_wild_16426569	1399938640	*Ca_kabuli_Chr05*	16426569	G/A	NA	INTERGENIC	NA
Kabuli_wild_16426575	1399938641	*Ca_kabuli_Chr05*	16426575	G/A	NA	INTERGENIC	NA
Kabuli_wild_16426642	1399938642	*Ca_kabuli_Chr05*	16426642	G/A	NA	INTERGENIC	NA
Kabuli_wild_16426656	1399938643	*Ca_kabuli_Chr05*	16426656	A/G	NA	INTERGENIC	NA
Kabuli_wild_16426662	1399938644	*Ca_kabuli_Chr05*	16426662	C/T	NA	INTERGENIC	NA
Kabuli_wild_16751536	1399938645	*Ca_kabuli_Chr05*	16751536	C/T	NA	INTERGENIC	NA
Kabuli_wild_16821369	1399938646	*Ca_kabuli_Chr05*	16821369	T/G	NA	INTERGENIC	NA
Kabuli_wild_16848621	1399938647	*Ca_kabuli_Chr05*	16848621	G/A	Gene_Ca_17946	DOWNSTREAM	NA
Kabuli_wild_16929860	1399938648	*Ca_kabuli_Chr05*	16929860	T/A	Gene_Ca_17949	INTRON	tRNA-dihydrouridinesynthase
Kabuli_wild_16929897	1399938649	*Ca_kabuli_Chr05*	16929897	A/G	Gene_Ca_17949	INTRON	tRNA-dihydrouridinesynthase
Kabuli_wild_16929903	1399938650	*Ca_kabuli_Chr05*	16929903	C/G	Gene_Ca_17949	INTRON	tRNA-dihydrouridinesynthase
Kabuli_wild_17037918	1399938651	*Ca_kabuli_Chr05*	17037918	T/G	NA	INTERGENIC	NA
Kabuli_wild_17037994	1399938652	*Ca_kabuli_Chr05*	17037994	T/G	NA	INTERGENIC	NA
Kabuli_wild_17044340	1399938653	*Ca_kabuli_Chr05*	17044340	G/A	NA	INTERGENIC	NA
Kabuli_wild_17045235	1399938654	*Ca_kabuli_Chr05*	17045235	T/C	Gene_Ca_17956	DOWNSTREAM	UDP-glucuronosyl/UDP-glucosyltransferase
Kabuli_wild_17045244	1399938655	*Ca_kabuli_Chr05*	17045244	A/G	Gene_Ca_17956	DOWNSTREAM	UDP-glucuronosyl/UDP-glucosyltransferase
Kabuli_wild_17239611	1399938656	*Ca_kabuli_Chr05*	17239611	C/T	NA	INTERGENIC	NA
Kabuli_wild_17239612	1399938657	*Ca_kabuli_Chr05*	17239612	T/C	NA	INTERGENIC	NA
Kabuli_wild_17494672	1399938658	*Ca_kabuli_Chr05*	17494672	C/T	NA	INTERGENIC	NA
Kabuli_wild_17494676	1399938659	*Ca_kabuli_Chr05*	17494676	A/G	NA	INTERGENIC	NA
Kabuli_wild_17494697	1399938660	*Ca_kabuli_Chr05*	17494697	C/T	NA	INTERGENIC	NA
Kabuli_wild_17494699	1399938661	*Ca_kabuli_Chr05*	17494699	C/A	NA	INTERGENIC	NA
Kabuli_wild_17494700	1399938662	*Ca_kabuli_Chr05*	17494700	T/C	NA	INTERGENIC	NA
Kabuli_wild_17494702	1399938663	*Ca_kabuli_Chr05*	17494702	G/A	NA	INTERGENIC	NA
Kabuli_wild_17494714	1399938664	*Ca_kabuli_Chr05*	17494714	A/G	NA	INTERGENIC	NA
Kabuli_wild_18044808	1399938665	*Ca_kabuli_Chr05*	18044808	C/G	NA	INTERGENIC	NA
Kabuli_wild_18288260	1399938666	*Ca_kabuli_Chr05*	18288260	A/G	NA	INTERGENIC	NA
Kabuli_wild_18664706	1399938667	*Ca_kabuli_Chr05*	18664706	C/T	Gene_Ca_24483	NON_SYNONYMOUS_CODING	NA

SNP IDs	NCBI dbSNP accession IDs (NCBI_SS#)	Chromosomes	Physical positions (bp)	SNPs	Gene accession IDs	Sequence components of genome	Putative functions
Kabuli_wild_18664816	1399938668	*Ca_kabuli_Chr05*	18664816	A/T	Gene_Ca_24483	NON_SYNONYMOUS_CODING	NA
Kabuli_wild_18665097	1399938669	*Ca_kabuli_Chr05*	18665097	C/T	Gene_Ca_24483	SYNONYMOUS_CODING	NA
Kabuli_wild_18682788	1399938670	*Ca_kabuli_Chr05*	18682788	A/C	NA	INTERGENIC	NA
Kabuli_wild_18682792	1399938671	*Ca_kabuli_Chr05*	18682792	T/C	NA	INTERGENIC	NA
Kabuli_wild_18682793	1399938672	*Ca_kabuli_Chr05*	18682793	A/G	NA	INTERGENIC	NA
Kabuli_wild_18682794	1399938673	*Ca_kabuli_Chr05*	18682794	A/G	NA	INTERGENIC	NA
Kabuli_wild_18682812	1399938674	*Ca_kabuli_Chr05*	18682812	A/G	NA	INTERGENIC	NA
Kabuli_wild_18682814	1399938675	*Ca_kabuli_Chr05*	18682814	T/A	NA	INTERGENIC	NA
Kabuli_wild_18682822	1399938676	*Ca_kabuli_Chr05*	18682822	A/G	NA	INTERGENIC	NA
Kabuli_wild_18682827	1399938677	*Ca_kabuli_Chr05*	18682827	C/G	NA	INTERGENIC	NA
Kabuli_wild_18682830	1399938678	*Ca_kabuli_Chr05*	18682830	G/A	NA	INTERGENIC	NA
Kabuli_wild_18682834	1399938679	*Ca_kabuli_Chr05*	18682834	A/G	NA	INTERGENIC	NA
Kabuli_wild_18682840	1399938680	*Ca_kabuli_Chr05*	18682840	T/C	NA	INTERGENIC	NA
Kabuli_wild_18682843	1399938681	*Ca_kabuli_Chr05*	18682843	G/C	NA	INTERGENIC	NA
Kabuli_wild_18682849	1399938682	*Ca_kabuli_Chr05*	18682849	T/C	NA	INTERGENIC	NA
Kabuli_wild_18684334	1399938683	*Ca_kabuli_Chr05*	18684334	C/T	NA	INTERGENIC	NA
Kabuli_wild_18971085	1399938684	*Ca_kabuli_Chr05*	18971085	G/A	NA	INTERGENIC	NA
Kabuli_wild_18971088	1399938685	*Ca_kabuli_Chr05*	18971088	C/A	NA	INTERGENIC	NA
Kabuli_wild_18971091	1399938686	*Ca_kabuli_Chr05*	18971091	T/G	NA	INTERGENIC	NA
Kabuli_wild_18971101	1399938687	*Ca_kabuli_Chr05*	18971101	T/G	NA	INTERGENIC	NA
Kabuli_wild_18971263	1399938688	*Ca_kabuli_Chr05*	18971263	G/C	NA	INTERGENIC	NA
Kabuli_wild_19383304	1399938689	*Ca_kabuli_Chr05*	19383304	A/C	NA	INTERGENIC	NA
Kabuli_wild_19421820	1399938690	*Ca_kabuli_Chr05*	19421820	C/T	Gene_Ca_23514	NON_SYNONYMOUS_CODING	Peptidase C48, SUMO/Sentrin/Ubl1
Kabuli_wild_19421868	1399938691	*Ca_kabuli_Chr05*	19421868	C/T	Gene_Ca_23514	NON_SYNONYMOUS_CODING	Peptidase C48, SUMO/Sentrin/Ubl1
Kabuli_wild_19421946	1399938692	*Ca_kabuli_Chr05*	19421946	T/C	Gene_Ca_23514	NON_SYNONYMOUS_CODING	Peptidase C48, SUMO/Sentrin/Ubl1
Kabuli_wild_19423339	1399938693	*Ca_kabuli_Chr05*	19423339	G/A	NA	INTERGENIC	NA
Kabuli_wild_19423342	1399938694	*Ca_kabuli_Chr05*	19423342	G/A	NA	INTERGENIC	NA
Kabuli_wild_19423358	1399938695	*Ca_kabuli_Chr05*	19423358	G/A	NA	INTERGENIC	NA
Kabuli_wild_19423382	1399938696	*Ca_kabuli_Chr05*	19423382	G/C	NA	INTERGENIC	NA
Kabuli_wild_19828124	1399938697	*Ca_kabuli_Chr05*	19828124	G/A	NA	INTERGENIC	NA
Kabuli_wild_19828135	1399938698	*Ca_kabuli_Chr05*	19828135	A/G	NA	INTERGENIC	NA
Kabuli_wild_19828142	1399938699	*Ca_kabuli_Chr05*	19828142	A/G	NA	INTERGENIC	NA
Kabuli_wild_19828151	1399938700	*Ca_kabuli_Chr05*	19828151	C/A	NA	INTERGENIC	NA
Kabuli_wild_19828154	1399938701	*Ca_kabuli_Chr05*	19828154	C/T	NA	INTERGENIC	NA
Kabuli_wild_19828163	1399938702	*Ca_kabuli_Chr05*	19828163	T/C	NA	INTERGENIC	NA
Kabuli_wild_19828166	1399938703	*Ca_kabuli_Chr05*	19828166	C/T	NA	INTERGENIC	NA
Kabuli_wild_19828167	1399938704	*Ca_kabuli_Chr05*	19828167	A/C	NA	INTERGENIC	NA
Kabuli_wild_19828172	1399938705	*Ca_kabuli_Chr05*	19828172	T/G	NA	INTERGENIC	NA
Kabuli_wild_19828190	1399938706	*Ca_kabuli_Chr05*	19828190	T/C	NA	INTERGENIC	NA
Kabuli_wild_19828203	1399938707	*Ca_kabuli_Chr05*	19828203	C/A	NA	INTERGENIC	NA
Kabuli_wild_19828226	1399938708	*Ca_kabuli_Chr05*	19828226	G/A	NA	INTERGENIC	NA
Kabuli_wild_19884124	1399938709	*Ca_kabuli_Chr05*	19884124	A/G	Gene_Ca_22815	INTRON	Vps16,C-terminal
Kabuli_wild_19884132	1399938710	*Ca_kabuli_Chr05*	19884132	C/T	Gene_Ca_22815	INTRON	Vps16,C-terminal
Kabuli_wild_20354352	1399938711	*Ca_kabuli_Chr05*	20354352	T/C	NA	INTERGENIC	NA

SNP IDs	NCBI dbSNP accession IDs (NCBI_SS#)	Chromosomes	Physical positions (bp)	SNPs	Gene accession IDs	Sequence components of genome	Putative functions
Kabuli_wild_20460859	1399938712	*Ca_kabuli_Chr05*	20460859	T/G	Gene_Ca_18208	NON_SYNONYMOUS_CODING	TetratricopeptideTPR-1
Kabuli_wild_20493370	1399938713	*Ca_kabuli_Chr05*	20493370	C/T	Gene_Ca_18211	DOWNSTREAM	NA
Kabuli_wild_20493429	1399938714	*Ca_kabuli_Chr05*	20493429	A/G	Gene_Ca_18211	DOWNSTREAM	NA
Kabuli_wild_20546385	1399938715	*Ca_kabuli_Chr05*	20546385	T/G	NA	INTERGENIC	NA
Kabuli_wild_20546402	1399938716	*Ca_kabuli_Chr05*	20546402	T/C	NA	INTERGENIC	NA
Kabuli_wild_20644984	1399938717	*Ca_kabuli_Chr05*	20644984	C/A	Gene_Ca_18219	NON_SYNONYMOUS_CODING	Amidase
Kabuli_wild_21163371	1399938718	*Ca_kabuli_Chr05*	21163371	G/A	NA	INTERGENIC	NA
Kabuli_wild_21385948	1399938719	*Ca_kabuli_Chr05*	21385948	T/C	Gene_Ca_18243	SYNONYMOUS_CODING	Signal transduction response regulator, receiverdomain
Kabuli_wild_21495508	1399938720	*Ca_kabuli_Chr05*	21495508	G/A	NA	INTERGENIC	NA
Kabuli_wild_21495562	1399938721	*Ca_kabuli_Chr05*	21495562	C/T	NA	INTERGENIC	NA
Kabuli_wild_21495583	1399938722	*Ca_kabuli_Chr05*	21495583	C/A	NA	INTERGENIC	NA
Kabuli_wild_21495631	1399938723	*Ca_kabuli_Chr05*	21495631	C/T	NA	INTERGENIC	NA
Kabuli_wild_21495672	1399938724	*Ca_kabuli_Chr05*	21495672	G/A	NA	INTERGENIC	NA
Kabuli_wild_21495674	1399938725	*Ca_kabuli_Chr05*	21495674	G/A	NA	INTERGENIC	NA
Kabuli_wild_21495679	1399938726	*Ca_kabuli_Chr05*	21495679	G/A	NA	INTERGENIC	NA
Kabuli_wild_21495707	1399938727	*Ca_kabuli_Chr05*	21495707	A/G	NA	INTERGENIC	NA
Kabuli_wild_22065518	1399938728	*Ca_kabuli_Chr05*	22065518	C/T	Gene_Ca_21161	INTRON	NA
Kabuli_wild_22065859	1399938729	*Ca_kabuli_Chr05*	22065859	A/G	Gene_Ca_21161	SYNONYMOUS_CODING	NA
Kabuli_wild_22115925	1399938730	*Ca_kabuli_Chr05*	22115925	T/G	NA	INTERGENIC	NA
Kabuli_wild_22116961	1399938731	*Ca_kabuli_Chr05*	22116961	T/G	NA	INTERGENIC	NA
Kabuli_wild_22302338	1399938732	*Ca_kabuli_Chr05*	22302338	G/A	Gene_Ca_21151	NON_SYNONYMOUS_CODING	Ribosome60SbiogenesisN-terminal
Kabuli_wild_22302350	1399938733	*Ca_kabuli_Chr05*	22302350	C/A	Gene_Ca_21151	NON_SYNONYMOUS_CODING	Ribosome60SbiogenesisN-terminal
Kabuli_wild_22344868	1399938734	*Ca_kabuli_Chr05*	22344868	A/G	Gene_Ca_21149	SYNONYMOUS_CODING	NA
Kabuli_wild_22526689	1399938735	*Ca_kabuli_Chr05*	22526689	A/G	Gene_Ca_20087	INTRON	NA
Kabuli_wild_22627341	1399938736	*Ca_kabuli_Chr05*	22627341	A/C	Gene_Ca_20079	SYNONYMOUS_CODING	Bromodomain
Kabuli_wild_22657146	1399938737	*Ca_kabuli_Chr05*	22657146	T/C	Gene_Ca_20077	DOWNSTREAM	NA
Kabuli_wild_22679380	1399938738	*Ca_kabuli_Chr05*	22679380	C/A	NA	INTERGENIC	NA
Kabuli_wild_22679381	1399938739	*Ca_kabuli_Chr05*	22679381	G/T	NA	INTERGENIC	NA
Kabuli_wild_22690192	1399938740	*Ca_kabuli_Chr05*	22690192	A/G	NA	INTERGENIC	NA
Kabuli_wild_22690194	1399938741	*Ca_kabuli_Chr05*	22690194	T/G	NA	INTERGENIC	NA
Kabuli_wild_22699980	1399938742	*Ca_kabuli_Chr05*	22699980	A/G	Gene_Ca_20068	DOWNSTREAM	ATPase,F0complex,subunitB,mitochondrial
Kabuli_wild_22699982	1399938743	*Ca_kabuli_Chr05*	22699982	T/G	Gene_Ca_20068	DOWNSTREAM	ATPase,F0complex,subunitB,mitochondrial
Kabuli_wild_23049683	1399938744	*Ca_kabuli_Chr05*	23049683	C/A	Gene_Ca_21972	DOWNSTREAM	Target SNARE coiled-coildomain
Kabuli_wild_23151998	1399938745	*Ca_kabuli_Chr05*	23151998	G/T	Gene_Ca_21977	INTRON	Zinc finger,PHD-type
Kabuli_wild_23152038	1399938746	*Ca_kabuli_Chr05*	23152038	A/C	Gene_Ca_21977	INTRON	Zinc finger,PHD-type
Kabuli_wild_23157944	1399938747	*Ca_kabuli_Chr05*	23157944	A/C	Gene_Ca_21977	INTRON	Zinc finger,PHD-type
Kabuli_wild_23161492	1399938748	*Ca_kabuli_Chr05*	23161492	A/T	Gene_Ca_21977	SYNONYMOUS_CODING	Zinc finger,PHD-type
Kabuli_wild_23162705	1399938749	*Ca_kabuli_Chr05*	23162705	T/G	NA	INTERGENIC	NA
Kabuli_wild_23162727	1399938750	*Ca_kabuli_Chr05*	23162727	A/G	NA	INTERGENIC	NA
Kabuli_wild_23262157	1399938751	*Ca_kabuli_Chr05*	23262157	T/C	NA	INTERGENIC	NA
Kabuli_wild_23323541	1399938752	*Ca_kabuli_Chr05*	23323541	A/T	NA	INTERGENIC	NA
Kabuli_wild_23560225	1399938753	*Ca_kabuli_Chr05*	23560225	G/A	NA	INTERGENIC	NA
Kabuli_wild_23561851	1399938754	*Ca_kabuli_Chr05*	23561851	A/G	Gene_Ca_17690	INTRON	NA
Kabuli_wild_23562152	1399938755	*Ca_kabuli_Chr05*	23562152	T/G	Gene_Ca_17690	INTRON	NA

SNP IDs	NCBI dbSNP accession IDs (NCBI_SS#)	Chromosomes	Physical positions (bp)	SNPs	Gene accession IDs	Sequence components of genome	Putative functions
Kabuli_wild_23562203	1399938756	*Ca_kabuli_Chr05*	23562203	C/T	Gene_Ca_17690	NON_SYNONYMOUS_CODING	NA
Kabuli_wild_23562467	1399938757	*Ca_kabuli_Chr05*	23562467	A/G	Gene_Ca_17690	NON_SYNONYMOUS_CODING	NA
Kabuli_wild_23562482	1399938758	*Ca_kabuli_Chr05*	23562482	G/A	Gene_Ca_17690	NON_SYNONYMOUS_CODING	NA
Kabuli_wild_23562595	1399938759	*Ca_kabuli_Chr05*	23562595	G/A	Gene_Ca_17690	NON_SYNONYMOUS_CODING	NA
Kabuli_wild_23562827	1399938760	*Ca_kabuli_Chr05*	23562827	G/A	Gene_Ca_17690	INTRON	NA
Kabuli_wild_23564746	1399938761	*Ca_kabuli_Chr05*	23564746	A/T	NA	INTERGENIC	NA
Kabuli_wild_23664529	1399938762	*Ca_kabuli_Chr05*	23664529	T/C	NA	INTERGENIC	NA
Kabuli_wild_23674814	1399938763	*Ca_kabuli_Chr05*	23674814	C/G	NA	INTERGENIC	NA
Kabuli_wild_23674815	1399938764	*Ca_kabuli_Chr05*	23674815	C/T	NA	INTERGENIC	NA
Kabuli_wild_23674845	1399938765	*Ca_kabuli_Chr05*	23674845	G/C	NA	INTERGENIC	NA
Kabuli_wild_23709142	1399938766	*Ca_kabuli_Chr05*	23709142	T/C	NA	INTERGENIC	NA
Kabuli_wild_24090515	1399938767	*Ca_kabuli_Chr05*	24090515	A/C	Gene_Ca_17656	DOWNSTREAM	Protein of unknown function DUF3437
Kabuli_wild_24090523	1399938768	*Ca_kabuli_Chr05*	24090523	A/C	Gene_Ca_17656	DOWNSTREAM	Protein of unknown function DUF3437
Kabuli_wild_24090533	1399938769	*Ca_kabuli_Chr05*	24090533	C/T	Gene_Ca_17656	DOWNSTREAM	Protein of unknown function DUF3437
Kabuli_wild_24090749	1399938770	*Ca_kabuli_Chr05*	24090749	A/C	Gene_Ca_17656	DOWNSTREAM	Protein of unknown function DUF3437
Kabuli_wild_24107319	1399938771	*Ca_kabuli_Chr05*	24107319	A/G	Gene_Ca_17656	INTRON	Protein of unknown function DUF3437
Kabuli_wild_24153138	1399938772	*Ca_kabuli_Chr05*	24153138	G/A	Gene_Ca_17653	NON_SYNONYMOUS_CODING	Protein of unknown function DUF3437
Kabuli_wild_24153158	1399938773	*Ca_kabuli_Chr05*	24153158	C/T	Gene_Ca_17653	SYNONYMOUS_CODING	Protein of unknown function DUF3437
Kabuli_wild_24153199	1399938774	*Ca_kabuli_Chr05*	24153199	C/T	Gene_Ca_17653	NON_SYNONYMOUS_CODING	Protein of unknown function DUF3437
Kabuli_wild_24153202	1399938775	*Ca_kabuli_Chr05*	24153202	A/C	Gene_Ca_17653	NON_SYNONYMOUS_CODING	Protein of unknown function DUF3437
Kabuli_wild_24153205	1399938776	*Ca_kabuli_Chr05*	24153205	T/C	Gene_Ca_17653	NON_SYNONYMOUS_CODING	Protein of unknown function DUF3437
Kabuli_wild_24153273	1399938777	*Ca_kabuli_Chr05*	24153273	T/C	Gene_Ca_17653	NON_SYNONYMOUS_CODING	Protein of unknown function DUF3437
Kabuli_wild_24165982	1399938778	*Ca_kabuli_Chr05*	24165982	G/A	Gene_Ca_17652	INTRON	Protein of unknown function DUF3437
Kabuli_wild_24165995	1399938779	*Ca_kabuli_Chr05*	24165995	A/G	Gene_Ca_17652	INTRON	Protein of unknown function DUF3437
Kabuli_wild_24166003	1399938780	*Ca_kabuli_Chr05*	24166003	T/C	Gene_Ca_17652	INTRON	Protein of unknown function DUF3437
Kabuli_wild_24166015	1399938781	*Ca_kabuli_Chr05*	24166015	G/T	Gene_Ca_17652	INTRON	Protein of unknown function DUF3437
Kabuli_wild_24166046	1399938782	*Ca_kabuli_Chr05*	24166046	G/A	Gene_Ca_17652	INTRON	Protein of unknown function DUF3437
Kabuli_wild_24166047	1399938783	*Ca_kabuli_Chr05*	24166047	C/T	Gene_Ca_17652	INTRON	Protein of unknown function DUF3437
Kabuli_wild_24166082	1399938784	*Ca_kabuli_Chr05*	24166082	G/A	Gene_Ca_17652	INTRON	Protein of unknown function DUF3437
Kabuli_wild_24166095	1399938785	*Ca_kabuli_Chr05*	24166095	G/A	Gene_Ca_17652	INTRON	Protein of unknown function DUF3437
Kabuli_wild_24166103	1399938786	*Ca_kabuli_Chr05*	24166103	C/T	Gene_Ca_17652	INTRON	Protein of unknown function DUF3437
Kabuli_wild_24176199	1399938787	*Ca_kabuli_Chr05*	24176199	G/A	Gene_Ca_17652	INTRON	Protein of unknown function DUF3437
Kabuli_wild_24181228	1399938788	*Ca_kabuli_Chr05*	24181228	C/A	Gene_Ca_17652	INTRON	Protein of unknown function DUF3437
Kabuli_wild_24181232	1399938789	*Ca_kabuli_Chr05*	24181232	A/C	Gene_Ca_17652	INTRON	Protein of unknown function DUF3437
Kabuli_wild_24245308	1399938790	*Ca_kabuli_Chr05*	24245308	C/A	Gene_Ca_15567	INTRON	Protein of unknown function DUF3437
Kabuli_wild_24245312	1399938791	*Ca_kabuli_Chr05*	24245312	A/C	Gene_Ca_15567	INTRON	Protein of unknown function DUF3437
Kabuli_wild_24453088	1399938792	*Ca_kabuli_Chr05*	24453088	A/C	Gene_Ca_15581	SYNONYMOUS_CODING	Neutral/alkalinenonlysosomalceramidase
Kabuli_wild_24576960	1399938793	*Ca_kabuli_Chr05*	24576960	C/T	NA	INTERGENIC	NA
Kabuli_wild_24576970	1399938794	*Ca_kabuli_Chr05*	24576970	A/G	NA	INTERGENIC	NA
Kabuli_wild_24576993	1399938795	*Ca_kabuli_Chr05*	24576993	T/G	NA	INTERGENIC	NA
Kabuli_wild_24724942	1399938796	*Ca_kabuli_Chr05*	24724942	T/A	NA	INTERGENIC	NA
Kabuli_wild_24822661	1399938797	*Ca_kabuli_Chr05*	24822661	T/C	NA	INTERGENIC	NA
Kabuli_wild_24830048	1399938798	*Ca_kabuli_Chr05*	24830048	G/C	NA	INTERGENIC	NA
Kabuli_wild_24843015	1399938799	*Ca_kabuli_Chr05*	24843015	G/T	NA	INTERGENIC	NA

SNP IDs	NCBI dbSNP accession IDs (NCBI_SS#)	Chromosomes	Physical positions (bp)	SNPs	Gene accession IDs	Sequence components of genome	Putative functions
Kabuli_wild_25006250	1399938800	*Ca_kabuli_Chr05*	25006250	C/A	Gene_Ca_15616	UPSTREAM	RNA-processing protein, HAThelix
Kabuli_wild_25092157	1399938801	*Ca_kabuli_Chr05*	25092157	T/C	Gene_Ca_15620	INTRON	Villinheadpiece
Kabuli_wild_25121200	1399938802	*Ca_kabuli_Chr05*	25121200	A/G	Gene_Ca_15623	DOWNSTREAM	Zinc finger, RING-type
Kabuli_wild_25399115	1399938803	*Ca_kabuli_Chr05*	25399115	T/G	Gene_Ca_09042	SYNONYMOUS_CODING	Zinc finger,CCCH-type
Kabuli_wild_25399443	1399938804	*Ca_kabuli_Chr05*	25399443	C/A	Gene_Ca_09042	NON_SYNONYMOUS_CODING	Zinc finger,CCCH-type
Kabuli_wild_25506629	1399938805	*Ca_kabuli_Chr05*	25506629	G/A	NA	INTERGENIC	NA
Kabuli_wild_25678975	1399938806	*Ca_kabuli_Chr05*	25678975	G/T	NA	INTERGENIC	NA
Kabuli_wild_25687210	1399938807	*Ca_kabuli_Chr05*	25687210	A/C	Gene_Ca_09027	SYNONYMOUS_CODING	Clathrin,heavychain/VPS,7-foldrepeat
Kabuli_wild_25697487	1399938808	*Ca_kabuli_Chr05*	25697487	C/G	NA	INTERGENIC	NA
Kabuli_wild_25805020	1399938809	*Ca_kabuli_Chr05*	25805020	A/G	Gene_Ca_09020	INTRON	Peptidase C48, SUMO/Sentrin/Ubl1
Kabuli_wild_25805073	1399938810	*Ca_kabuli_Chr05*	25805073	A/C	Gene_Ca_09020	INTRON	Peptidase C48, SUMO/Sentrin/Ubl1
Kabuli_wild_26025356	1399938811	*Ca_kabuli_Chr05*	26025356	A/T	Gene_Ca_09010	DOWNSTREAM	Protein kinase,catalytic domain
Kabuli_wild_26083559	1399938812	*Ca_kabuli_Chr05*	26083559	T/G	NA	INTERGENIC	NA
Kabuli_wild_26121156	1399938813	*Ca_kabuli_Chr05*	26121156	G/A	Gene_Ca_09005	NON_SYNONYMOUS_CODING	NA
Kabuli_wild_26124240	1399938814	*Ca_kabuli_Chr05*	26124240	T/C	Gene_Ca_09005	INTRON	NA
Kabuli_wild_26238262	1399938815	*Ca_kabuli_Chr05*	26238262	G/A	Gene_Ca_08997	DOWNSTREAM	NAD-dependentepimerase/dehydratase
Kabuli_wild_26384636	1399938816	*Ca_kabuli_Chr05*	26384636	C/G	NA	INTERGENIC	NA
Kabuli_wild_26474910	1399938817	*Ca_kabuli_Chr05*	26474910	A/C	Gene_Ca_08972	INTRON	NA
Kabuli_wild_26496197	1399938818	*Ca_kabuli_Chr05*	26496197	A/G	Gene_Ca_08970	NON_SYNONYMOUS_CODING	Aminotransferaseclass-III
Kabuli_wild_26569443	1399938819	*Ca_kabuli_Chr05*	26569443	A/C	Gene_Ca_08965	SYNONYMOUS_CODING	NA
Kabuli_wild_26615934	1399938820	*Ca_kabuli_Chr05*	26615934	C/T	Gene_Ca_08961	NON_SYNONYMOUS_CODING	Armadillo
Kabuli_wild_26626855	1399938821	*Ca_kabuli_Chr05*	26626855	T/G	Gene_Ca_08960	NON_SYNONYMOUS_CODING	Aminoacyl-tRNAsynthetase,classI,conservedsite
Kabuli_wild_26679306	1399938822	*Ca_kabuli_Chr05*	26679306	C/T	NA	INTERGENIC	NA
Kabuli_wild_26679390	1399938823	*Ca_kabuli_Chr05*	26679390	C/T	NA	INTERGENIC	NA
Kabuli_wild_26679836	1399938824	*Ca_kabuli_Chr05*	26679836	C/T	NA	INTERGENIC	NA
Kabuli_wild_26696412	1399938825	*Ca_kabuli_Chr05*	26696412	C/A	NA	INTERGENIC	NA
Kabuli_wild_26696441	1399938826	*Ca_kabuli_Chr05*	26696441	A/T	NA	INTERGENIC	NA
Kabuli_wild_26696533	1399938827	*Ca_kabuli_Chr05*	26696533	C/T	NA	INTERGENIC	NA
Kabuli_wild_26696547	1399938828	*Ca_kabuli_Chr05*	26696547	C/T	NA	INTERGENIC	NA
Kabuli_wild_26696578	1399938829	*Ca_kabuli_Chr05*	26696578	G/A	NA	INTERGENIC	NA
Kabuli_wild_26696624	1399938830	*Ca_kabuli_Chr05*	26696624	G/A	NA	INTERGENIC	NA
Kabuli_wild_26785123	1399938831	*Ca_kabuli_Chr05*	26785123	C/A	NA	INTERGENIC	NA
Kabuli_wild_26785140	1399938832	*Ca_kabuli_Chr05*	26785140	G/A	NA	INTERGENIC	NA
Kabuli_wild_26997335	1399938833	*Ca_kabuli_Chr05*	26997335	T/G	NA	INTERGENIC	NA
Kabuli_wild_26999793	1399938834	*Ca_kabuli_Chr05*	26999793	C/T	NA	INTERGENIC	NA
Kabuli_wild_27000104	1399938835	*Ca_kabuli_Chr05*	27000104	C/A	NA	INTERGENIC	NA
Kabuli_wild_27000127	1399938836	*Ca_kabuli_Chr05*	27000127	C/G	NA	INTERGENIC	NA
Kabuli_wild_27000278	1399938837	*Ca_kabuli_Chr05*	27000278	T/A	NA	INTERGENIC	NA
Kabuli_wild_27000280	1399938838	*Ca_kabuli_Chr05*	27000280	T/G	NA	INTERGENIC	NA
Kabuli_wild_27117953	1399938839	*Ca_kabuli_Chr05*	27117953	A/G	Gene_Ca_08929	INTRON	Basic-leucine zipper (bZIP) Transcription factor
Kabuli_wild_27150550	1399938840	*Ca_kabuli_Chr05*	27150550	A/G	Gene_Ca_08924	INTRON	Transcriptional factor B3
Kabuli_wild_27189958	1399938841	*Ca_kabuli_Chr05*	27189958	C/T	Gene_Ca_08921	DOWNSTREAM	GlutathioneS-transferase,N-terminal
Kabuli_wild_27190008	1399938842	*Ca_kabuli_Chr05*	27190008	G/A	Gene_Ca_08921	DOWNSTREAM	GlutathioneS-transferase,N-terminal
Kabuli_wild_27334056	1399938843	*Ca_kabuli_Chr05*	27334056	A/C	NA	INTERGENIC	NA

SNP IDs	NCBI dbSNP accession IDs (NCBI_SS#)	Chromosomes	Physical positions (bp)	SNPs	Gene accession IDs	Sequence components of genome	Putative functions
Kabuli_wild_27334057	1399938844	*Ca_kabuli_Chr05*	27334057	G/C	NA	INTERGENIC	NA
Kabuli_wild_27334061	1399938845	*Ca_kabuli_Chr05*	27334061	G/C	NA	INTERGENIC	NA
Kabuli_wild_27361579	1399938846	*Ca_kabuli_Chr05*	27361579	T/G	Gene_Ca_08907	SYNONYMOUS_CODING	BTB/POZ-like
Kabuli_wild_27361668	1399938847	*Ca_kabuli_Chr05*	27361668	G/T	Gene_Ca_08907	SYNONYMOUS_CODING	BTB/POZ-like
Kabuli_wild_27362570	1399938848	*Ca_kabuli_Chr05*	27362570	G/T	Gene_Ca_08907	NON_SYNONYMOUS_CODING	BTB/POZ-like
Kabuli_wild_27362573	1399938849	*Ca_kabuli_Chr05*	27362573	A/G	Gene_Ca_08907	NON_SYNONYMOUS_CODING	BTB/POZ-like
Kabuli_wild_27362584	1399938850	*Ca_kabuli_Chr05*	27362584	T/G	Gene_Ca_08907	SYNONYMOUS_CODING	BTB/POZ-like
Kabuli_wild_27452484	1399938851	*Ca_kabuli_Chr05*	27452484	C/G	Gene_Ca_08906	SYNONYMOUS_CODING	Protein kinase,catalytic domain
Kabuli_wild_27552106	1399938852	*Ca_kabuli_Chr05*	27552106	A/C	NA	INTERGENIC	NA
Kabuli_wild_27568016	1399938853	*Ca_kabuli_Chr05*	27568016	G/A	Gene_Ca_08898	NON_SYNONYMOUS_CODING	NA
Kabuli_wild_27568028	1399938854	*Ca_kabuli_Chr05*	27568028	A/T	Gene_Ca_08898	NON_SYNONYMOUS_CODING	NA
Kabuli_wild_27568029	1399938855	*Ca_kabuli_Chr05*	27568029	G/A	Gene_Ca_08898	NON_SYNONYMOUS_CODING	NA
Kabuli_wild_27568033	1399938856	*Ca_kabuli_Chr05*	27568033	G/A	Gene_Ca_08898	SYNONYMOUS_CODING	NA
Kabuli_wild_27568059	1399938857	*Ca_kabuli_Chr05*	27568059	C/T	Gene_Ca_08898	NON_SYNONYMOUS_CODING	NA
Kabuli_wild_27568064	1399938858	*Ca_kabuli_Chr05*	27568064	A/C	Gene_Ca_08898	NON_SYNONYMOUS_CODING	NA
Kabuli_wild_27568081	1399938859	*Ca_kabuli_Chr05*	27568081	G/C	Gene_Ca_08898	SYNONYMOUS_CODING	NA
Kabuli_wild_27568082	1399938860	*Ca_kabuli_Chr05*	27568082	G/A	Gene_Ca_08898	NON_SYNONYMOUS_CODING	NA
Kabuli_wild_27568091	1399938861	*Ca_kabuli_Chr05*	27568091	G/A	Gene_Ca_08898	NON_SYNONYMOUS_CODING	NA
Kabuli_wild_27568095	1399938862	*Ca_kabuli_Chr05*	27568095	C/A	Gene_Ca_08898	NON_SYNONYMOUS_CODING	NA
Kabuli_wild_27568097	1399938863	*Ca_kabuli_Chr05*	27568097	G/A	Gene_Ca_08898	NON_SYNONYMOUS_CODING	NA
Kabuli_wild_27568098	1399938864	*Ca_kabuli_Chr05*	27568098	C/T	Gene_Ca_08898	NON_SYNONYMOUS_CODING	NA
Kabuli_wild_27568107	1399938865	*Ca_kabuli_Chr05*	27568107	C/G	Gene_Ca_08898	NON_SYNONYMOUS_CODING	NA
Kabuli_wild_27568118	1399938866	*Ca_kabuli_Chr05*	27568118	A/G	Gene_Ca_08898	NON_SYNONYMOUS_CODING	NA
Kabuli_wild_27568133	1399938867	*Ca_kabuli_Chr05*	27568133	G/T	Gene_Ca_08898	NON_SYNONYMOUS_CODING	NA
Kabuli_wild_27568141	1399938868	*Ca_kabuli_Chr05*	27568141	C/T	Gene_Ca_08898	SYNONYMOUS_CODING	NA
Kabuli_wild_27568156	1399938869	*Ca_kabuli_Chr05*	27568156	C/G	Gene_Ca_08898	NON_SYNONYMOUS_CODING	NA
Kabuli_wild_27568176	1399938870	*Ca_kabuli_Chr05*	27568176	T/C	Gene_Ca_08898	NON_SYNONYMOUS_CODING	NA
Kabuli_wild_27568177	1399938871	*Ca_kabuli_Chr05*	27568177	G/C	Gene_Ca_08898	NON_SYNONYMOUS_CODING	NA
Kabuli_wild_27568180	1399938872	*Ca_kabuli_Chr05*	27568180	C/T	Gene_Ca_08898	SYNONYMOUS_CODING	NA
Kabuli_wild_27605037	1399938873	*Ca_kabuli_Chr05*	27605037	A/T	Gene_Ca_08894	DOWNSTREAM	Uncharacterised protein familyYcf54
Kabuli_wild_27605063	1399938874	*Ca_kabuli_Chr05*	27605063	T/A	Gene_Ca_08894	DOWNSTREAM	Uncharacterised protein familyYcf54
Kabuli_wild_27605081	1399938875	*Ca_kabuli_Chr05*	27605081	C/T	Gene_Ca_08894	DOWNSTREAM	Uncharacterised protein familyYcf54
Kabuli_wild_27605095	1399938876	*Ca_kabuli_Chr05*	27605095	G/C	Gene_Ca_08894	DOWNSTREAM	Uncharacterised protein familyYcf54
Kabuli_wild_27631258	1399938877	*Ca_kabuli_Chr05*	27631258	A/G	Gene_Ca_08891	NON_SYNONYMOUS_CODING	Helicase,C-terminal
Kabuli_wild_27692818	1399938878	*Ca_kabuli_Chr05*	27692818	C/T	Gene_Ca_08882	UPSTREAM	NA
Kabuli_wild_27692822	1399938879	*Ca_kabuli_Chr05*	27692822	C/T	Gene_Ca_08882	UPSTREAM	NA
Kabuli_wild_27692823	1399938880	*Ca_kabuli_Chr05*	27692823	A/T	Gene_Ca_08882	UPSTREAM	NA
Kabuli_wild_27692831	1399938881	*Ca_kabuli_Chr05*	27692831	A/T	Gene_Ca_08882	UPSTREAM	NA
Kabuli_wild_27692836	1399938882	*Ca_kabuli_Chr05*	27692836	G/T	Gene_Ca_08882	UPSTREAM	NA
Kabuli_wild_27694205	1399938883	*Ca_kabuli_Chr05*	27694205	C/A	NA	INTERGENIC	NA
Kabuli_wild_27694215	1399938884	*Ca_kabuli_Chr05*	27694215	A/G	NA	INTERGENIC	NA
Kabuli_wild_27694216	1399938885	*Ca_kabuli_Chr05*	27694216	C/G	NA	INTERGENIC	NA
Kabuli_wild_27697470	1399938886	*Ca_kabuli_Chr05*	27697470	C/A	Gene_Ca_08882	DOWNSTREAM	NA
Kabuli_wild_27749174	1399938887	*Ca_kabuli_Chr05*	27749174	A/T	Gene_Ca_08872	NON_SYNONYMOUS_CODING	AUX/IAAprotein

SNP IDs	NCBI dbSNP accession IDs (NCBI_SS#)	Chromosomes	Physical positions (bp)	SNPs	Gene accession IDs	Sequence components of genome	Putative functions
Kabuli_wild_27786025	1399938888	*Ca_kabuli_Chr05*	27786025	T/C	Gene_Ca_08871	INTRON	NA
Kabuli_wild_27820210	1399938889	*Ca_kabuli_Chr05*	27820210	A/C	NA	INTERGENIC	NA
Kabuli_wild_27838003	1399938890	*Ca_kabuli_Chr05*	27838003	C/T	NA	INTERGENIC	NA
Kabuli_wild_27895972	1399938891	*Ca_kabuli_Chr05*	27895972	C/A	Gene_Ca_13427	NON_SYNONYMOUS_CODING	NA
Kabuli_wild_27896939	1399938892	*Ca_kabuli_Chr05*	27896939	C/T	Gene_Ca_13427	INTRON	NA
Kabuli_wild_27932017	1399938893	*Ca_kabuli_Chr05*	27932017	G/A	NA	INTERGENIC	NA
Kabuli_wild_27939128	1399938894	*Ca_kabuli_Chr05*	27939128	A/G	NA	INTERGENIC	NA
Kabuli_wild_27939134	1399938895	*Ca_kabuli_Chr05*	27939134	A/T	NA	INTERGENIC	NA
Kabuli_wild_27939135	1399938896	*Ca_kabuli_Chr05*	27939135	G/C	NA	INTERGENIC	NA
Kabuli_wild_27939162	1399938897	*Ca_kabuli_Chr05*	27939162	G/A	NA	INTERGENIC	NA
Kabuli_wild_27974632	1399938898	*Ca_kabuli_Chr05*	27974632	G/T	Gene_Ca_13421	INTRON	ABC-1
Kabuli_wild_27974691	1399938899	*Ca_kabuli_Chr05*	27974691	G/T	Gene_Ca_13421	INTRON	ABC-1
Kabuli_wild_27989926	1399938900	*Ca_kabuli_Chr05*	27989926	G/A	Gene_Ca_13419	NON_SYNONYMOUS_CODING	Helicase-associateddomain
Kabuli_wild_27989938	1399938901	*Ca_kabuli_Chr05*	27989938	A/G	Gene_Ca_13419	NON_SYNONYMOUS_CODING	Helicase-associateddomain
Kabuli_wild_28016628	1399938902	*Ca_kabuli_Chr05*	28016628	C/T	NA	INTERGENIC	NA
Kabuli_wild_28016669	1399938903	*Ca_kabuli_Chr05*	28016669	A/G	NA	INTERGENIC	NA
Kabuli_wild_28071467	1399938904	*Ca_kabuli_Chr05*	28071467	A/G	NA	INTERGENIC	NA
Kabuli_wild_28071547	1399938905	*Ca_kabuli_Chr05*	28071547	G/A	NA	INTERGENIC	NA
Kabuli_wild_28115151	1399938906	*Ca_kabuli_Chr05*	28115151	A/T	Gene_Ca_13409	INTRON	NA
Kabuli_wild_28116988	1399938907	*Ca_kabuli_Chr05*	28116988	A/T	Gene_Ca_13409	DOWNSTREAM	NA
Kabuli_wild_28117011	1399938908	*Ca_kabuli_Chr05*	28117011	C/T	Gene_Ca_13409	DOWNSTREAM	NA
Kabuli_wild_28143644	1399938909	*Ca_kabuli_Chr05*	28143644	T/C	NA	INTERGENIC	NA
Kabuli_wild_28143679	1399938910	*Ca_kabuli_Chr05*	28143679	T/C	NA	INTERGENIC	NA
Kabuli_wild_28164985	1399938911	*Ca_kabuli_Chr05*	28164985	G/C	Gene_Ca_13404	INTRON	Clathrin,heavychain/VPS,7-foldrepeat
Kabuli_wild_28166322	1399938912	*Ca_kabuli_Chr05*	28166322	T/G	Gene_Ca_13404	NON_SYNONYMOUS_CODING	NA
Kabuli_wild_28168910	1399938913	*Ca_kabuli_Chr05*	28168910	A/G	NA	INTERGENIC	NA
Kabuli_wild_28169475	1399938914	*Ca_kabuli_Chr05*	28169475	T/C	NA	INTERGENIC	NA
Kabuli_wild_28175039	1399938915	*Ca_kabuli_Chr05*	28175039	A/G	Gene_Ca_13403	NON_SYNONYMOUS_CODING	Clathrin,heavychain/VPS,7-foldrepeat
Kabuli_wild_28266046	1399938916	*Ca_kabuli_Chr05*	28266046	T/G	Gene_Ca_13399	SYNONYMOUS_CODING	NA
Kabuli_wild_28266074	1399938917	*Ca_kabuli_Chr05*	28266074	T/C	Gene_Ca_13399	NON_SYNONYMOUS_CODING	NA
Kabuli_wild_28271638	1399938918	*Ca_kabuli_Chr05*	28271638	G/C	Gene_Ca_13398	INTRON	Bacterial surface antigen(D15)
Kabuli_wild_28271732	1399938919	*Ca_kabuli_Chr05*	28271732	C/T	Gene_Ca_13398	INTRON	Bacterial surface antigen(D15)
Kabuli_wild_28271742	1399938920	*Ca_kabuli_Chr05*	28271742	G/A	Gene_Ca_13398	INTRON	Bacterial surface antigen(D15)
Kabuli_wild_28271767	1399938921	*Ca_kabuli_Chr05*	28271767	G/C	Gene_Ca_13398	INTRON	Bacterial surface antigen(D15)
Kabuli_wild_28327832	1399938922	*Ca_kabuli_Chr05*	28327832	T/G	NA	INTERGENIC	NA
Kabuli_wild_28327856	1399938923	*Ca_kabuli_Chr05*	28327856	T/C	NA	INTERGENIC	NA
Kabuli_wild_28327868	1399938924	*Ca_kabuli_Chr05*	28327868	G/A	NA	INTERGENIC	NA
Kabuli_wild_28327870	1399938925	*Ca_kabuli_Chr05*	28327870	T/C	NA	INTERGENIC	NA
Kabuli_wild_28327883	1399938926	*Ca_kabuli_Chr05*	28327883	G/A	NA	INTERGENIC	NA
Kabuli_wild_28335455	1399938927	*Ca_kabuli_Chr05*	28335455	A/C	Gene_Ca_13389	SYNONYMOUS_CODING	Histidinephosphatasesuperfamily,clade-2
Kabuli_wild_28346497	1399938928	*Ca_kabuli_Chr05*	28346497	C/T	Gene_Ca_13388	SYNONYMOUS_CODING	Lateral organ boundaries, LOB
Kabuli_wild_28353952	1399938929	*Ca_kabuli_Chr05*	28353952	G/C	Gene_Ca_13387	INTRON	Glycosidehydrolase,family85
Kabuli_wild_28353953	1399938930	*Ca_kabuli_Chr05*	28353953	A/G	Gene_Ca_13387	INTRON	Glycosidehydrolase,family85
Kabuli_wild_28360017	1399938931	*Ca_kabuli_Chr05*	28360017	G/A	Gene_Ca_13386	DOWNSTREAM	RNAhelicase,ATP-dependent,DEAD-box,conservedsite

SNP IDs	NCBI dbSNP accession IDs (NCBI_SS#)	Chromosomes	Physical positions (bp)	SNPs	Gene accession IDs	Sequence components of genome	Putative functions
Kabuli_wild_28360492	1399938932	*Ca_kabuli_Chr05*	28360492	C/T	Gene_Ca_13387	UPSTREAM	Glycosidehydrolase,family85
Kabuli_wild_28360495	1399938933	*Ca_kabuli_Chr05*	28360495	C/T	Gene_Ca_13387	UPSTREAM	Glycosidehydrolase,family85
Kabuli_wild_28360585	1399938934	*Ca_kabuli_Chr05*	28360585	A/G	NA	INTERGENIC	NA
Kabuli_wild_28383374	1399938935	*Ca_kabuli_Chr05*	28383374	G/T	Gene_Ca_13385	INTRON	Autophagy-relatedprotein27
Kabuli_wild_28402498	1399938936	*Ca_kabuli_Chr05*	28402498	A/G	NA	INTERGENIC	NA
Kabuli_wild_28562734	1399938937	*Ca_kabuli_Chr05*	28562734	G/A	Gene_Ca_13369	DOWNSTREAM	Prephenatedehydrogenase
Kabuli_wild_28581814	1399938938	*Ca_kabuli_Chr05*	28581814	A/G	NA	INTERGENIC	NA
Kabuli_wild_28581815	1399938939	*Ca_kabuli_Chr05*	28581815	C/G	NA	INTERGENIC	NA
Kabuli_wild_28581827	1399938940	*Ca_kabuli_Chr05*	28581827	T/C	NA	INTERGENIC	NA
Kabuli_wild_28581866	1399938941	*Ca_kabuli_Chr05*	28581866	T/C	NA	INTERGENIC	NA
Kabuli_wild_28582525	1399938942	*Ca_kabuli_Chr05*	28582525	C/T	NA	INTERGENIC	NA
Kabuli_wild_28582763	1399938943	*Ca_kabuli_Chr05*	28582763	A/G	NA	INTERGENIC	NA
Kabuli_wild_28582806	1399938944	*Ca_kabuli_Chr05*	28582806	T/C	NA	INTERGENIC	NA
Kabuli_wild_28583375	1399938945	*Ca_kabuli_Chr05*	28583375	C/G	Gene_Ca_13367	INTRON	Sel1-like
Kabuli_wild_28583402	1399938946	*Ca_kabuli_Chr05*	28583402	G/A	Gene_Ca_13367	INTRON	Sel1-like
Kabuli_wild_28683045	1399938947	*Ca_kabuli_Chr05*	28683045	G/A	Gene_Ca_13361	SYNONYMOUS_CODING	BTB/POZ-like
Kabuli_wild_28717362	1399938948	*Ca_kabuli_Chr05*	28717362	C/T	Gene_Ca_13358	NON_SYNONYMOUS_CODING	Oligo peptide transporter
Kabuli_wild_28717384	1399938949	*Ca_kabuli_Chr05*	28717384	C/T	Gene_Ca_13358	SYNONYMOUS_CODING	Oligo peptide transporter
Kabuli_wild_28721236	1399938950	*Ca_kabuli_Chr05*	28721236	C/T	NA	INTERGENIC	NA
Kabuli_wild_28721237	1399938951	*Ca_kabuli_Chr05*	28721237	A/G	NA	INTERGENIC	NA
Kabuli_wild_28721383	1399938952	*Ca_kabuli_Chr05*	28721383	A/C	NA	INTERGENIC	NA
Kabuli_wild_28721386	1399938953	*Ca_kabuli_Chr05*	28721386	T/C	NA	INTERGENIC	NA
Kabuli_wild_28746139	1399938954	*Ca_kabuli_Chr05*	28746139	A/G	Gene_Ca_13355	NON_SYNONYMOUS_CODING	Haloaciddehalogenase-likehydrolase
Kabuli_wild_28757467	1399938955	*Ca_kabuli_Chr05*	28757467	A/T	Gene_Ca_13354	NON_SYNONYMOUS_CODING	Protein of unknown function DUF827,plant
Kabuli_wild_28757485	1399938956	*Ca_kabuli_Chr05*	28757485	A/C	Gene_Ca_13354	NON_SYNONYMOUS_CODING	Protein of unknown function DUF827,plant
Kabuli_wild_28757507	1399938957	*Ca_kabuli_Chr05*	28757507	A/T	Gene_Ca_13354	NON_SYNONYMOUS_CODING	Protein of unknown function DUF827,plant
Kabuli_wild_28758723	1399938958	*Ca_kabuli_Chr05*	28758723	A/G	Gene_Ca_13354	INTRON	Protein of unknown function DUF827,plant
Kabuli_wild_28817832	1399938959	*Ca_kabuli_Chr05*	28817832	A/G	Gene_Ca_13351	DOWNSTREAM	Domain of unknown function DUF250
Kabuli_wild_28827467	1399938960	*Ca_kabuli_Chr05*	28827467	A/G	NA	INTERGENIC	NA
Kabuli_wild_28843579	1399938961	*Ca_kabuli_Chr05*	28843579	A/G	Gene_Ca_13348	INTRON	Peptidoglycan-bindingLysinsubgroup
Kabuli_wild_28843674	1399938962	*Ca_kabuli_Chr05*	28843674	T/C	Gene_Ca_13348	SYNONYMOUS_CODING	Peptidoglycan-bindingLysinsubgroup
Kabuli_wild_28854486	1399938963	*Ca_kabuli_Chr05*	28854486	C/T	Gene_Ca_13347	DOWNSTREAM	Transcription factor Tfb4
Kabuli_wild_28886131	1399938964	*Ca_kabuli_Chr05*	28886131	T/C	NA	INTERGENIC	NA
Kabuli_wild_28905203	1399938965	*Ca_kabuli_Chr05*	28905203	A/C	Gene_Ca_13344	NON_SYNONYMOUS_CODING	Drug/metabolitetransporter
Kabuli_wild_29007844	1399938966	*Ca_kabuli_Chr05*	29007844	T/C	Gene_Ca_23834	SYNONYMOUS_CODING	CamphorresistanceCrcBprotein
Kabuli_wild_29007847	1399938967	*Ca_kabuli_Chr05*	29007847	A/C	Gene_Ca_23834	SYNONYMOUS_CODING	CamphorresistanceCrcBprotein
Kabuli_wild_29007879	1399938968	*Ca_kabuli_Chr05*	29007879	T/C	Gene_Ca_23834	NON_SYNONYMOUS_CODING	CamphorresistanceCrcBprotein
Kabuli_wild_29007882	1399938969	*Ca_kabuli_Chr05*	29007882	A/C	Gene_Ca_23834	NON_SYNONYMOUS_CODING	CamphorresistanceCrcBprotein
Kabuli_wild_29007884	1399938970	*Ca_kabuli_Chr05*	29007884	A/C	Gene_Ca_23834	NON_SYNONYMOUS_CODING	CamphorresistanceCrcBprotein
Kabuli_wild_29007886	1399938971	*Ca_kabuli_Chr05*	29007886	C/A	Gene_Ca_23834	SYNONYMOUS_CODING	CamphorresistanceCrcBprotein
Kabuli_wild_29007895	1399938972	*Ca_kabuli_Chr05*	29007895	T/C	Gene_Ca_23834	SYNONYMOUS_CODING	CamphorresistanceCrcBprotein
Kabuli_wild_29080060	1399938973	*Ca_kabuli_Chr05*	29080060	C/T	Gene_Ca_23829	SYNONYMOUS_CODING	Protein kinase,catalytic domain
Kabuli_wild_29114055	1399938974	*Ca_kabuli_Chr05*	29114055	A/T	Gene_Ca_23826	NON_SYNONYMOUS_CODING	Oxysterol-bindingprotein
Kabuli_wild_29222381	1399938975	*Ca_kabuli_Chr05*	29222381	T/C	Gene_Ca_16618	NON_SYNONYMOUS_CODING	NA

SNP IDs	NCBI dbSNP accession IDs (NCBI_SS#)	Chromosomes	Physical positions (bp)	SNPs	Gene accession IDs	Sequence components of genome	Putative functions
Kabuli_wild_29285397	1399938976	*Ca_kabuli_Chr05*	29285397	T/A	Gene_Ca_16623	SYNONYMOUS_CODING	Carboxyltransferase
Kabuli_wild_29340206	1399938977	*Ca_kabuli_Chr05*	29340206	C/T	NA	INTERGENIC	NA
Kabuli_wild_29354269	1399938978	*Ca_kabuli_Chr05*	29354269	A/T	NA	INTERGENIC	NA
Kabuli_wild_29437139	1399938979	*Ca_kabuli_Chr05*	29437139	C/T	NA	INTERGENIC	NA
Kabuli_wild_29437146	1399938980	*Ca_kabuli_Chr05*	29437146	C/A	NA	INTERGENIC	NA
Kabuli_wild_29437162	1399938981	*Ca_kabuli_Chr05*	29437162	A/C	NA	INTERGENIC	NA
Kabuli_wild_29437171	1399938982	*Ca_kabuli_Chr05*	29437171	A/T	NA	INTERGENIC	NA
Kabuli_wild_29437181	1399938983	*Ca_kabuli_Chr05*	29437181	A/C	NA	INTERGENIC	NA
Kabuli_wild_29437193	1399938984	*Ca_kabuli_Chr05*	29437193	C/A	NA	INTERGENIC	NA
Kabuli_wild_29468456	1399938985	*Ca_kabuli_Chr05*	29468456	T/C	NA	INTERGENIC	NA
Kabuli_wild_29528904	1399938986	*Ca_kabuli_Chr05*	29528904	T/G	Gene_Ca_16640	NON_SYNONYMOUS_CODING	Sugar/inositol transporter
Kabuli_wild_29567676	1399938987	*Ca_kabuli_Chr05*	29567676	T/C	NA	INTERGENIC	NA
Kabuli_wild_29567820	1399938988	*Ca_kabuli_Chr05*	29567820	T/C	NA	INTERGENIC	NA
Kabuli_wild_29576716	1399938989	*Ca_kabuli_Chr05*	29576716	A/C	NA	INTERGENIC	NA
Kabuli_wild_29608657	1399938990	*Ca_kabuli_Chr05*	29608657	T/C	NA	INTERGENIC	NA
Kabuli_wild_29641659	1399938991	*Ca_kabuli_Chr05*	29641659	T/C	NA	INTERGENIC	NA
Kabuli_wild_29658280	1399938992	*Ca_kabuli_Chr05*	29658280	A/C	Gene_Ca_16650	INTRON	Protein of unknown function DUF248, methyltransferase
Kabuli_wild_29788248	1399938993	*Ca_kabuli_Chr05*	29788248	T/G	Gene_Ca_16661	NON_SYNONYMOUS_CODING	Fork head-associated (FHA) domain
Kabuli_wild_29824218	1399938994	*Ca_kabuli_Chr05*	29824218	T/G	NA	INTERGENIC	NA
Kabuli_wild_29999414	1399938995	*Ca_kabuli_Chr05*	29999414	C/T	NA	INTERGENIC	NA
Kabuli_wild_29999433	1399938996	*Ca_kabuli_Chr05*	29999433	C/A	NA	INTERGENIC	NA
Kabuli_wild_29999439	1399938997	*Ca_kabuli_Chr05*	29999439	A/G	NA	INTERGENIC	NA
Kabuli_wild_29999465	1399938998	*Ca_kabuli_Chr05*	29999465	T/G	NA	INTERGENIC	NA
Kabuli_wild_29999476	1399938999	*Ca_kabuli_Chr05*	29999476	G/A	NA	INTERGENIC	NA
Kabuli_wild_30035732	1399939000	*Ca_kabuli_Chr05*	30035732	T/A	Gene_Ca_04680	STOP_GAINED	Sugar/inositol transporter
Kabuli_wild_30035763	1399939001	*Ca_kabuli_Chr05*	30035763	A/T	Gene_Ca_04680	SYNONYMOUS_CODING	Sugar/inositol transporter
Kabuli_wild_30049265	1399939002	*Ca_kabuli_Chr05*	30049265	G/A	NA	INTERGENIC	NA
Kabuli_wild_30053540	1399939003	*Ca_kabuli_Chr05*	30053540	C/G	Gene_Ca_04681	UPSTREAM	Glycosyltransferase,family13
Kabuli_wild_30060160	1399939004	*Ca_kabuli_Chr05*	30060160	C/T	NA	INTERGENIC	NA
Kabuli_wild_30086232	1399939005	*Ca_kabuli_Chr05*	30086232	A/C	NA	INTERGENIC	NA
Kabuli_wild_30090906	1399939006	*Ca_kabuli_Chr05*	30090906	A/G	NA	INTERGENIC	NA
Kabuli_wild_30091063	1399939007	*Ca_kabuli_Chr05*	30091063	G/A	NA	INTERGENIC	NA
Kabuli_wild_30124077	1399939008	*Ca_kabuli_Chr05*	30124077	G/C	Gene_Ca_04688	NON_SYNONYMOUS_CODING	NA
Kabuli_wild_30124086	1399939009	*Ca_kabuli_Chr05*	30124086	C/G	Gene_Ca_04688	SYNONYMOUS_CODING	NA
Kabuli_wild_30191389	1399939010	*Ca_kabuli_Chr05*	30191389	C/A	NA	INTERGENIC	NA
Kabuli_wild_30248447	1399939011	*Ca_kabuli_Chr05*	30248447	C/A	NA	INTERGENIC	NA
Kabuli_wild_30367349	1399939012	*Ca_kabuli_Chr05*	30367349	G/T	Gene_Ca_04714	NON_SYNONYMOUS_CODING	HECT
Kabuli_wild_30373542	1399939013	*Ca_kabuli_Chr05*	30373542	A/G	Gene_Ca_04715	NON_SYNONYMOUS_CODING	Armadillo
Kabuli_wild_30373783	1399939014	*Ca_kabuli_Chr05*	30373783	T/A	Gene_Ca_04715	SYNONYMOUS_CODING	Armadillo
Kabuli_wild_30376670	1399939015	*Ca_kabuli_Chr05*	30376670	C/A	Gene_Ca_04715	INTRON	Armadillo
Kabuli_wild_30439348	1399939016	*Ca_kabuli_Chr05*	30439348	A/C	Gene_Ca_04723	DOWNSTREAM	PollenOlee1allergen/extensin
Kabuli_wild_30444264	1399939017	*Ca_kabuli_Chr05*	30444264	G/A	NA	INTERGENIC	NA
Kabuli_wild_30456581	1399939018	*Ca_kabuli_Chr05*	30456581	T/C	Gene_Ca_04727	UPSTREAM	Plantneutralinvertase
Kabuli_wild_30562524	1399939019	*Ca_kabuli_Chr05*	30562524	G/T	NA	INTERGENIC	NA

SNP IDs	NCBI dbSNP accession IDs (NCBI_SS#)	Chromosomes	Physical positions (bp)	SNPs	Gene accession IDs	Sequence components of genome	Putative functions
Kabuli_wild_30577985	1399939020	*Ca_kabuli_Chr05*	30577985	T/G	Gene_Ca_04750	SYNONYMOUS_CODING	Zinc finger,CCCH-type
Kabuli_wild_30623408	1399939021	*Ca_kabuli_Chr05*	30623408	C/T	Gene_Ca_04755	NON_SYNONYMOUS_CODING	Ironhydrogenase,smallsubunit-like
Kabuli_wild_30632697	1399939022	*Ca_kabuli_Chr05*	30632697	A/G	Gene_Ca_04756	INTRON	NA
Kabuli_wild_30656058	1399939023	*Ca_kabuli_Chr05*	30656058	A/T	Gene_Ca_04757	INTRON	Kinesin,motordomain
Kabuli_wild_30690885	1399939024	*Ca_kabuli_Chr05*	30690885	A/G	NA	INTERGENIC	NA
Kabuli_wild_30690891	1399939025	*Ca_kabuli_Chr05*	30690891	T/G	NA	INTERGENIC	NA
Kabuli_wild_30691510	1399939026	*Ca_kabuli_Chr05*	30691510	A/G	NA	INTERGENIC	NA
Kabuli_wild_30691515	1399939027	*Ca_kabuli_Chr05*	30691515	G/A	NA	INTERGENIC	NA
Kabuli_wild_30691543	1399939028	*Ca_kabuli_Chr05*	30691543	A/G	NA	INTERGENIC	NA
Kabuli_wild_30697113	1399939029	*Ca_kabuli_Chr05*	30697113	T/C	Gene_Ca_04764	DOWNSTREAM	Zinc finger, RING-type
Kabuli_wild_30697511	1399939030	*Ca_kabuli_Chr05*	30697511	G/A	NA	INTERGENIC	NA
Kabuli_wild_30796451	1399939031	*Ca_kabuli_Chr05*	30796451	C/T	NA	INTERGENIC	NA
Kabuli_wild_30811250	1399939032	*Ca_kabuli_Chr05*	30811250	A/T	NA	INTERGENIC	NA
Kabuli_wild_30868695	1399939033	*Ca_kabuli_Chr05*	30868695	G/C	Gene_Ca_04783	UPSTREAM	Glycosidehydrolase,family17
Kabuli_wild_30868696	1399939034	*Ca_kabuli_Chr05*	30868696	A/G	Gene_Ca_04783	UPSTREAM	Glycosidehydrolase,family17
Kabuli_wild_31025764	1399939035	*Ca_kabuli_Chr05*	31025764	G/T	Gene_Ca_04800	DOWNSTREAM	RNA recognition motif domain
Kabuli_wild_31036938	1399939036	*Ca_kabuli_Chr05*	31036938	A/G	NA	INTERGENIC	NA
Kabuli_wild_31108863	1399939037	*Ca_kabuli_Chr05*	31108863	C/A	Gene_Ca_04812	INTRON	Zinc finger,DHHC-type,palmitoyltransferase
Kabuli_wild_31126125	1399939038	*Ca_kabuli_Chr05*	31126125	C/T	NA	INTERGENIC	NA
Kabuli_wild_31131830	1399939039	*Ca_kabuli_Chr05*	31131830	T/C	Gene_Ca_04815	INTRON	NA
Kabuli_wild_31132568	1399939040	*Ca_kabuli_Chr05*	31132568	A/T	Gene_Ca_04815	SYNONYMOUS_CODING	NA
Kabuli_wild_31141947	1399939041	*Ca_kabuli_Chr05*	31141947	A/C	NA	INTERGENIC	NA
Kabuli_wild_31157892	1399939042	*Ca_kabuli_Chr05*	31157892	A/G	NA	INTERGENIC	NA
Kabuli_wild_31158252	1399939043	*Ca_kabuli_Chr05*	31158252	C/T	NA	INTERGENIC	NA
Kabuli_wild_31162533	1399939044	*Ca_kabuli_Chr05*	31162533	G/A	NA	INTERGENIC	NA
Kabuli_wild_31164387	1399939045	*Ca_kabuli_Chr05*	31164387	G/C	Gene_Ca_04820	INTRON	Armadillo
Kabuli_wild_31164539	1399939046	*Ca_kabuli_Chr05*	31164539	T/C	Gene_Ca_04820	SYNONYMOUS_CODING	Armadillo
Kabuli_wild_31165002	1399939047	*Ca_kabuli_Chr05*	31165002	A/C	Gene_Ca_04820	NON_SYNONYMOUS_CODING	Armadillo
Kabuli_wild_31165033	1399939048	*Ca_kabuli_Chr05*	31165033	A/C	Gene_Ca_04820	NON_SYNONYMOUS_CODING	Armadillo
Kabuli_wild_31165035	1399939049	*Ca_kabuli_Chr05*	31165035	T/C	Gene_Ca_04820	NON_SYNONYMOUS_CODING	Armadillo
Kabuli_wild_31165039	1399939050	*Ca_kabuli_Chr05*	31165039	T/C	Gene_Ca_04820	SYNONYMOUS_CODING	Armadillo
Kabuli_wild_31165048	1399939051	*Ca_kabuli_Chr05*	31165048	T/C	Gene_Ca_04820	SYNONYMOUS_CODING	Armadillo
Kabuli_wild_31165057	1399939052	*Ca_kabuli_Chr05*	31165057	A/G	Gene_Ca_04820	SYNONYMOUS_CODING	Armadillo
Kabuli_wild_31176844	1399939053	*Ca_kabuli_Chr05*	31176844	G/A	Gene_Ca_04822	INTRON	EPS15homology(EH)
Kabuli_wild_31194974	1399939054	*Ca_kabuli_Chr05*	31194974	T/C	Gene_Ca_04824	DOWNSTREAM	NADH:flavinoxidoreductase/NADHoxidase,N-terminal
Kabuli_wild_31215109	1399939055	*Ca_kabuli_Chr05*	31215109	C/T	NA	INTERGENIC	NA
Kabuli_wild_31313858	1399939056	*Ca_kabuli_Chr05*	31313858	T/C	Gene_Ca_04834	NON_SYNONYMOUS_CODING	Phox/Bem1p
Kabuli_wild_31314012	1399939057	*Ca_kabuli_Chr05*	31314012	A/C	Gene_Ca_04834	SYNONYMOUS_CODING	Catalase
Kabuli_wild_31314056	1399939058	*Ca_kabuli_Chr05*	31314056	A/C	Gene_Ca_04834	SYNONYMOUS_CODING	NA
Kabuli_wild_31330773	1399939059	*Ca_kabuli_Chr05*	31330773	C/A	Gene_Ca_04835	INTRON	NA
Kabuli_wild_31390319	1399939060	*Ca_kabuli_Chr05*	31390319	C/T	Gene_Ca_04841	INTRON	NA
Kabuli_wild_31687891	1399939061	*Ca_kabuli_Chr05*	31687891	A/C	Gene_Ca_04871	NON_SYNONYMOUS_CODING	RNA-bindingproteinLupusLa
Kabuli_wild_31694063	1399939062	*Ca_kabuli_Chr05*	31694063	T/G	NA	INTERGENIC	NA
Kabuli_wild_31704649	1399939063	*Ca_kabuli_Chr05*	31704649	T/G	Gene_Ca_04873	NON_SYNONYMOUS_CODING	PeptidaseS8/S53,subtilisin/kexin/sedolisin

SNP IDs	NCBI dbSNP accession IDs (NCBI_SS#)	Chromosomes	Physical positions (bp)	SNPs	Gene accession IDs	Sequence components of genome	Putative functions
Kabuli_wild_31750242	1399939064	*Ca_kabuli_Chr05*	31750242	A/G	NA	INTERGENIC	NA
Kabuli_wild_31935674	1399939065	*Ca_kabuli_Chr05*	31935674	T/C	Gene_Ca_04898	NON_SYNONYMOUS_CODING	Protein of unknown function DUF869,plant
Kabuli_wild_32011781	1399939066	*Ca_kabuli_Chr05*	32011781	G/T	Gene_Ca_04904	INTRON	Kinesin,motordomain
Kabuli_wild_32011792	1399939067	*Ca_kabuli_Chr05*	32011792	T/G	Gene_Ca_04904	INTRON	Kinesin,motordomain
Kabuli_wild_32171632	1399939068	*Ca_kabuli_Chr05*	32171632	G/A	Gene_Ca_04917	INTRON	Tetrapyrrolemethylase
Kabuli_wild_32291118	1399939069	*Ca_kabuli_Chr05*	32291118	G/A	NA	INTERGENIC	NA
Kabuli_wild_32291124	1399939070	*Ca_kabuli_Chr05*	32291124	G/A	NA	INTERGENIC	NA
Kabuli_wild_32291133	1399939071	*Ca_kabuli_Chr05*	32291133	T/A	NA	INTERGENIC	NA
Kabuli_wild_32291134	1399939072	*Ca_kabuli_Chr05*	32291134	A/G	Gene_Ca_04927	START_LOST	Aminotransferase-like,plant mobile domain
Kabuli_wild_32291136	1399939073	*Ca_kabuli_Chr05*	32291136	G/T	Gene_Ca_04927	START_LOST	Aminotransferase-like,plant mobile domain
Kabuli_wild_32291157	1399939074	*Ca_kabuli_Chr05*	32291157	G/A	Gene_Ca_04927	SYNONYMOUS_CODING	Aminotransferase-like,plant mobile domain
Kabuli_wild_32291158	1399939075	*Ca_kabuli_Chr05*	32291158	C/T	Gene_Ca_04927	STOP_GAINED	Aminotransferase-like,plant mobile domain
Kabuli_wild_32291160	1399939076	*Ca_kabuli_Chr05*	32291160	G/A	Gene_Ca_04927	SYNONYMOUS_CODING	Aminotransferase-like,plant mobile domain
Kabuli_wild_32291166	1399939077	*Ca_kabuli_Chr05*	32291166	T/G	Gene_Ca_04927	NON_SYNONYMOUS_CODING	Aminotransferase-like,plant mobile domain
Kabuli_wild_32357511	1399939078	*Ca_kabuli_Chr05*	32357511	G/A	Gene_Ca_04934	NON_SYNONYMOUS_CODING	Pseudouridinesynthase/archaeosinetransglycosylase
Kabuli_wild_32368485	1399939079	*Ca_kabuli_Chr05*	32368485	G/C	NA	INTERGENIC	NA
Kabuli_wild_32383745	1399939080	*Ca_kabuli_Chr05*	32383745	G/A	NA	INTERGENIC	NA
Kabuli_wild_32383807	1399939081	*Ca_kabuli_Chr05*	32383807	G/A	NA	INTERGENIC	NA
Kabuli_wild_32383828	1399939082	*Ca_kabuli_Chr05*	32383828	G/A	NA	INTERGENIC	NA
Kabuli_wild_32383871	1399939083	*Ca_kabuli_Chr05*	32383871	G/C	NA	INTERGENIC	NA
Kabuli_wild_32383875	1399939084	*Ca_kabuli_Chr05*	32383875	T/G	NA	INTERGENIC	NA
Kabuli_wild_32399391	1399939085	*Ca_kabuli_Chr05*	32399391	T/A	NA	INTERGENIC	NA
Kabuli_wild_32516773	1399939086	*Ca_kabuli_Chr05*	32516773	T/A	Gene_Ca_04958	INTRON	NA
Kabuli_wild_32524646	1399939087	*Ca_kabuli_Chr05*	32524646	T/C	Gene_Ca_04959	INTRON	NA
Kabuli_wild_32598221	1399939088	*Ca_kabuli_Chr05*	32598221	C/A	Gene_Ca_04965	DOWNSTREAM	Epidermalgrowthfactor-like,type3
Kabuli_wild_32598281	1399939089	*Ca_kabuli_Chr05*	32598281	C/A	Gene_Ca_04965	DOWNSTREAM	Epidermalgrowthfactor-like,type3
Kabuli_wild_32816626	1399939090	*Ca_kabuli_Chr05*	32816626	G/T	NA	INTERGENIC	NA
Kabuli_wild_32840522	1399939091	*Ca_kabuli_Chr05*	32840522	T/C	Gene_Ca_04983	NON_SYNONYMOUS_CODING	Quinate/shikimate5-dehydrogenase/glutamyl-tRNAreductase
Kabuli_wild_32840541	1399939092	*Ca_kabuli_Chr05*	32840541	T/G	Gene_Ca_04983	NON_SYNONYMOUS_CODING	Quinate/shikimate5-dehydrogenase/glutamyl-tRNAreductase
Kabuli_wild_32859504	1399939093	*Ca_kabuli_Chr05*	32859504	T/G	Gene_Ca_04985	INTRON	Protein of unknown function DUF3453
Kabuli_wild_32859515	1399939094	*Ca_kabuli_Chr05*	32859515	T/G	Gene_Ca_04985	INTRON	Protein of unknown function DUF3453
Kabuli_wild_32860087	1399939095	*Ca_kabuli_Chr05*	32860087	C/T	Gene_Ca_04985	INTRON	Protein of unknown function DUF3453
Kabuli_wild_32978975	1399939096	*Ca_kabuli_Chr05*	32978975	A/T	Gene_Ca_04995	DOWNSTREAM	Cation/H+exchanger
Kabuli_wild_33052329	1399939097	*Ca_kabuli_Chr05*	33052329	C/G	NA	INTERGENIC	NA
Kabuli_wild_33236167	1399939098	*Ca_kabuli_Chr05*	33236167	C/T	Gene_Ca_05015	NON_SYNONYMOUS_CODING	Aminoacyl-tRNAsynthetase,classI,conservedsite
Kabuli_wild_33255823	1399939099	*Ca_kabuli_Chr05*	33255823	T/G	NA	INTERGENIC	NA
Kabuli_wild_33422813	1399939100	*Ca_kabuli_Chr05*	33422813	C/T	NA	INTERGENIC	NA
Kabuli_wild_33523676	1399939101	*Ca_kabuli_Chr05*	33523676	T/C	NA	INTERGENIC	NA
Kabuli_wild_33523686	1399939102	*Ca_kabuli_Chr05*	33523686	G/A	NA	INTERGENIC	NA
Kabuli_wild_33532807	1399939103	*Ca_kabuli_Chr05*	33532807	A/G	NA	INTERGENIC	NA
Kabuli_wild_33574724	1399939104	*Ca_kabuli_Chr05*	33574724	T/C	NA	INTERGENIC	NA
Kabuli_wild_33627132	1399939105	*Ca_kabuli_Chr05*	33627132	T/C	NA	INTERGENIC	NA
Kabuli_wild_33631108	1399939106	*Ca_kabuli_Chr05*	33631108	A/G	NA	INTERGENIC	NA
Kabuli_wild_33631266	1399939107	*Ca_kabuli_Chr05*	33631266	T/A	NA	INTERGENIC	NA

SNP IDs	NCBI dbSNP accession IDs (NCBI_SS#)	Chromosomes	Physical positions (bp)	SNPs	Gene accession IDs	Sequence components of genome	Putative functions
Kabuli_wild_33631274	1399939108	*Ca_kabuli_Chr05*	33631274	C/A	NA	INTERGENIC	NA
Kabuli_wild_33673894	1399939109	*Ca_kabuli_Chr05*	33673894	T/C	NA	INTERGENIC	NA
Kabuli_wild_33675481	1399939110	*Ca_kabuli_Chr05*	33675481	C/G	Gene_Ca_01888	NON_SYNONYMOUS_CODING	VHS
Kabuli_wild_33812213	1399939111	*Ca_kabuli_Chr05*	33812213	A/C	Gene_Ca_01876	INTRON	NA
Kabuli_wild_33812363	1399939112	*Ca_kabuli_Chr05*	33812363	T/A	Gene_Ca_01876	INTRON	NA
Kabuli_wild_33818742	1399939113	*Ca_kabuli_Chr05*	33818742	T/A	Gene_Ca_01875	NON_SYNONYMOUS_CODING	RNA recognition motif domain
Kabuli_wild_33818823	1399939114	*Ca_kabuli_Chr05*	33818823	A/G	Gene_Ca_01875	NON_SYNONYMOUS_CODING	RNA recognition motif domain
Kabuli_wild_33892596	1399939115	*Ca_kabuli_Chr05*	33892596	A/C	Gene_Ca_01864	NON_SYNONYMOUS_CODING	Proteasome component (PCI) domain
Kabuli_wild_33937307	1399939116	*Ca_kabuli_Chr05*	33937307	G/A	Gene_Ca_01859	DOWNSTREAM	Tetrapyrrolemethylase
Kabuli_wild_33937316	1399939117	*Ca_kabuli_Chr05*	33937316	G/A	Gene_Ca_01859	DOWNSTREAM	Tetrapyrrolemethylase
Kabuli_wild_33963525	1399939118	*Ca_kabuli_Chr05*	33963525	G/C	NA	INTERGENIC	NA
Kabuli_wild_33986253	1399939119	*Ca_kabuli_Chr05*	33986253	C/A	Gene_Ca_01852	SYNONYMOUS_CODING	Protein kinase,catalytic domain
Kabuli_wild_34041827	1399939120	*Ca_kabuli_Chr05*	34041827	A/C	NA	INTERGENIC	NA
Kabuli_wild_34129856	1399939121	*Ca_kabuli_Chr05*	34129856	A/G	Gene_Ca_01835	NON_SYNONYMOUS_CODING	ATPase, P-type, H+ transporting proton pump
Kabuli_wild_34129900	1399939122	*Ca_kabuli_Chr05*	34129900	A/C	Gene_Ca_01835	NON_SYNONYMOUS_CODING	ATPase, P-type, H+ transporting proton pump
Kabuli_wild_34189654	1399939123	*Ca_kabuli_Chr05*	34189654	A/T	Gene_Ca_01828	NON_SYNONYMOUS_CODING	Domain of unknown function DUF2296
Kabuli_wild_34190000	1399939124	*Ca_kabuli_Chr05*	34190000	A/G	Gene_Ca_01828	SYNONYMOUS_CODING	Domain of unknown function DUF2296
Kabuli_wild_34202075	1399939125	*Ca_kabuli_Chr05*	34202075	T/G	Gene_Ca_01827	NON_SYNONYMOUS_CODING	PeptidaseC19,ubiquitincarboxyl-terminalhydrolase2
Kabuli_wild_34244641	1399939126	*Ca_kabuli_Chr05*	34244641	A/G	Gene_Ca_01824	INTRON	WD40 repeat
Kabuli_wild_34334936	1399939127	*Ca_kabuli_Chr05*	34334936	A/G	Gene_Ca_01813	SYNONYMOUS_CODING	RibosomalproteinL10/acidicP0
Kabuli_wild_34411801	1399939128	*Ca_kabuli_Chr05*	34411801	T/C	NA	INTERGENIC	NA
Kabuli_wild_34441581	1399939129	*Ca_kabuli_Chr05*	34441581	A/C	Gene_Ca_01800	NON_SYNONYMOUS_CODING	Transcription elongation factor S-II, centraldomain
Kabuli_wild_34482017	1399939130	*Ca_kabuli_Chr05*	34482017	T/G	Gene_Ca_01795	SYNONYMOUS_CODING	Glycosyltransferase,family8
Kabuli_wild_34531222	1399939131	*Ca_kabuli_Chr05*	34531222	A/G	Gene_Ca_01787	INTRON	ABC transporter-like
Kabuli_wild_34535758	1399939132	*Ca_kabuli_Chr05*	34535758	T/C	Gene_Ca_01787	NON_SYNONYMOUS_CODING	ABC transporter-like
Kabuli_wild_34535893	1399939133	*Ca_kabuli_Chr05*	34535893	G/T	Gene_Ca_01787	UPSTREAM	ABC transporter-like
Kabuli_wild_34603263	1399939134	*Ca_kabuli_Chr05*	34603263	A/C	Gene_Ca_01780	INTRON	RanGTPase
Kabuli_wild_34603284	1399939135	*Ca_kabuli_Chr05*	34603284	A/G	Gene_Ca_01780	INTRON	RanGTPase
Kabuli_wild_34646956	1399939136	*Ca_kabuli_Chr05*	34646956	A/G	Gene_Ca_01776	UPSTREAM	Phospholipase/carboxylesterase
Kabuli_wild_34807540	1399939137	*Ca_kabuli_Chr05*	34807540	A/T	Gene_Ca_01760	NON_SYNONYMOUS_CODING	F-boxdomain,cyclin-like
Kabuli_wild_34808850	1399939138	*Ca_kabuli_Chr05*	34808850	T/G	NA	INTERGENIC	NA
Kabuli_wild_34824749	1399939139	*Ca_kabuli_Chr05*	34824749	A/T	Gene_Ca_01757	DOWNSTREAM	Protein kinase,catalytic domain
Kabuli_wild_34845398	1399939140	*Ca_kabuli_Chr05*	34845398	T/G	NA	INTERGENIC	NA
Kabuli_wild_34857308	1399939141	*Ca_kabuli_Chr05*	34857308	T/G	Gene_Ca_01754	INTRON	AUX/IAAprotein
Kabuli_wild_34882263	1399939142	*Ca_kabuli_Chr05*	34882263	T/G	Gene_Ca_01752	DOWNSTREAM	Ubiquitin supe rgroup
Kabuli_wild_34974703	1399939143	*Ca_kabuli_Chr05*	34974703	C/T	NA	INTERGENIC	NA
Kabuli_wild_35191371	1399939144	*Ca_kabuli_Chr05*	35191371	C/G	Gene_Ca_01716	NON_SYNONYMOUS_CODING	Proteasome component (PCI) domain
Kabuli_wild_35302645	1399939145	*Ca_kabuli_Chr05*	35302645	A/C	NA	INTERGENIC	NA
Kabuli_wild_35312501	1399939146	*Ca_kabuli_Chr05*	35312501	G/A	Gene_Ca_01705	DOWNSTREAM	Pentatrico peptide repeat
Kabuli_wild_35500751	1399939147	*Ca_kabuli_Chr05*	35500751	T/C	Gene_Ca_01682	SYNONYMOUS_CODING	Sterilealpha motif domain
Kabuli_wild_35532133	1399939148	*Ca_kabuli_Chr05*	35532133	A/C	Gene_Ca_01678	INTRON	BRCT
Kabuli_wild_35555184	1399939149	*Ca_kabuli_Chr05*	35555184	A/G	Gene_Ca_01676	UPSTREAM	Pentatrico peptide repeat
Kabuli_wild_35617798	1399939150	*Ca_kabuli_Chr05*	35617798	A/C	NA	INTERGENIC	NA
Kabuli_wild_35690996	1399939151	*Ca_kabuli_Chr05*	35690996	G/A	NA	INTERGENIC	NA

SNP IDs	NCBI dbSNP accession IDs (NCBI_SS#)	Chromosomes	Physical positions (bp)	SNPs	Gene accession IDs	Sequence components of genome	Putative functions
Kabuli_wild_35715548	1399939152	*Ca_kabuli_Chr05*	35715548	T/C	NA	INTERGENIC	NA
Kabuli_wild_35715571	1399939153	*Ca_kabuli_Chr05*	35715571	A/C	NA	INTERGENIC	NA
Kabuli_wild_35773044	1399939154	*Ca_kabuli_Chr05*	35773044	A/G	NA	INTERGENIC	NA
Kabuli_wild_35789373	1399939155	*Ca_kabuli_Chr05*	35789373	T/A	Gene_Ca_01646	INTRON	Protein kinase,catalytic domain
Kabuli_wild_35839233	1399939156	*Ca_kabuli_Chr05*	35839233	G/A	Gene_Ca_01642	SYNONYMOUS_CODING	Mitochondrial carrier protein
Kabuli_wild_35844295	1399939157	*Ca_kabuli_Chr05*	35844295	G/T	NA	INTERGENIC	NA
Kabuli_wild_35877505	1399939158	*Ca_kabuli_Chr05*	35877505	T/C	Gene_Ca_01638	SYNONYMOUS_CODING	Mitochondrial carrier protein
Kabuli_wild_35937842	1399939159	*Ca_kabuli_Chr05*	35937842	G/T	Gene_Ca_01631	NON_SYNONYMOUS_CODING	NADH:ubiquinone oxidoreductase-like,20 kDa subunit
Kabuli_wild_35937912	1399939160	*Ca_kabuli_Chr05*	35937912	C/A	Gene_Ca_01631	NON_SYNONYMOUS_CODING	NADH:ubiquinone oxidoreductase-like,20 kDa subunit
Kabuli_wild_35957499	1399939161	*Ca_kabuli_Chr05*	35957499	T/C	Gene_Ca_01628	INTRON	Phosphorylated adapter RNA export protein,RNA-binding domain
Kabuli_wild_35967605	1399939162	*Ca_kabuli_Chr05*	35967605	T/C	Gene_Ca_01627	INTRON	PeptidaseC13,legumain
Kabuli_wild_36011980	1399939163	*Ca_kabuli_Chr05*	36011980	T/A	Gene_Ca_01621	DOWNSTREAM	Domain of unknown function DUF239,plant
Kabuli_wild_36128655	1399939164	*Ca_kabuli_Chr05*	36128655	G/T	Gene_Ca_01611	INTRON	NA
Kabuli_wild_36131156	1399939165	*Ca_kabuli_Chr05*	36131156	A/C	Gene_Ca_01611	INTRON	NA
Kabuli_wild_36238630	1399939166	*Ca_kabuli_Chr05*	36238630	G/A	Gene_Ca_01601	INTRON	ATPase,P-type,K/Mg/Cd/Cu/Zn/Na/Ca/Na/H-transporter
Kabuli_wild_36238655	1399939167	*Ca_kabuli_Chr05*	36238655	G/A	Gene_Ca_01601	INTRON	ATPase,P-type,K/Mg/Cd/Cu/Zn/Na/Ca/Na/H-transporter
Kabuli_wild_36238656	1399939168	*Ca_kabuli_Chr05*	36238656	G/A	Gene_Ca_01601	INTRON	ATPase,P-type,K/Mg/Cd/Cu/Zn/Na/Ca/Na/H-transporter
Kabuli_wild_36238663	1399939169	*Ca_kabuli_Chr05*	36238663	C/A	Gene_Ca_01601	INTRON	ATPase,P-type,K/Mg/Cd/Cu/Zn/Na/Ca/Na/H-transporter
Kabuli_wild_36248623	1399939170	*Ca_kabuli_Chr05*	36248623	A/C	Gene_Ca_01601	INTRON	ATPase,P-type,K/Mg/Cd/Cu/Zn/Na/Ca/Na/H-transporter
Kabuli_wild_36408719	1399939171	*Ca_kabuli_Chr05*	36408719	G/A	Gene_Ca_01583	DOWNSTREAM	Ankyrinrepeat
Kabuli_wild_36534056	1399939172	*Ca_kabuli_Chr05*	36534056	G/C	Gene_Ca_01566	INTRON	MRG
Kabuli_wild_36534063	1399939173	*Ca_kabuli_Chr05*	36534063	C/T	Gene_Ca_01566	INTRON	MRG
Kabuli_wild_36613606	1399939174	*Ca_kabuli_Chr05*	36613606	T/G	Gene_Ca_01559	NON_SYNONYMOUS_CODING	Protein kinase,catalytic domain
Kabuli_wild_36619837	1399939175	*Ca_kabuli_Chr05*	36619837	A/G	Gene_Ca_01558	INTRON	Kinesin,motordomain
Kabuli_wild_36621872	1399939176	*Ca_kabuli_Chr05*	36621872	A/T	Gene_Ca_01558	NON_SYNONYMOUS_CODING	Kinesin,motordomain
Kabuli_wild_36643373	1399939177	*Ca_kabuli_Chr05*	36643373	G/T	NA	INTERGENIC	NA
Kabuli_wild_36672147	1399939178	*Ca_kabuli_Chr05*	36672147	C/T	Gene_Ca_01555	NON_SYNONYMOUS_CODING	RNA recognition motif domain
Kabuli_wild_36673950	1399939179	*Ca_kabuli_Chr05*	36673950	C/A	NA	INTERGENIC	NA
Kabuli_wild_36735459	1399939180	*Ca_kabuli_Chr05*	36735459	A/T	Gene_Ca_01550	NON_SYNONYMOUS_CODING	Helix-loop-helixDNA-binding domain
Kabuli_wild_36751625	1399939181	*Ca_kabuli_Chr05*	36751625	C/T	Gene_Ca_01548	DOWNSTREAM	Basic-leucine zipper (bZIP) Transcription factor
Kabuli_wild_36751851	1399939182	*Ca_kabuli_Chr05*	36751851	T/A	Gene_Ca_01548	DOWNSTREAM	Basic-leucine zipper (bZIP) Transcription factor
Kabuli_wild_36757408	1399939183	*Ca_kabuli_Chr05*	36757408	G/A	Gene_Ca_01548	UPSTREAM	Basic-leucine zipper (bZIP) Transcription factor
Kabuli_wild_36797187	1399939184	*Ca_kabuli_Chr05*	36797187	G/T	NA	INTERGENIC	NA
Kabuli_wild_36800139	1399939185	*Ca_kabuli_Chr05*	36800139	G/A	NA	INTERGENIC	NA
Kabuli_wild_36978227	1399939186	*Ca_kabuli_Chr05*	36978227	T/G	NA	INTERGENIC	NA
Kabuli_wild_36999074	1399939187	*Ca_kabuli_Chr05*	36999074	G/C	Gene_Ca_01518	NON_SYNONYMOUS_CODING	Protein kinase,catalytic domain
Kabuli_wild_37012335	1399939188	*Ca_kabuli_Chr05*	37012335	T/G	NA	INTERGENIC	NA
Kabuli_wild_37012371	1399939189	*Ca_kabuli_Chr05*	37012371	A/T	NA	INTERGENIC	NA
Kabuli_wild_37016704	1399939190	*Ca_kabuli_Chr05*	37016704	C/G	NA	INTERGENIC	NA
Kabuli_wild_37039246	1399939191	*Ca_kabuli_Chr05*	37039246	A/T	NA	INTERGENIC	NA
Kabuli_wild_37039300	1399939192	*Ca_kabuli_Chr05*	37039300	A/G	NA	INTERGENIC	NA
Kabuli_wild_37040045	1399939193	*Ca_kabuli_Chr05*	37040045	G/A	NA	INTERGENIC	NA
Kabuli_wild_37066540	1399939194	*Ca_kabuli_Chr05*	37066540	G/A	Gene_Ca_01509	SYNONYMOUS_CODING	Aminoacyl-tRNAsynthetase,classII(G/H/P/S),conserveddomain
Kabuli_wild_37067293	1399939195	*Ca_kabuli_Chr05*	37067293	A/G	Gene_Ca_01509	INTRON	Aminoacyl-tRNAsynthetase,classII(G/H/P/S),conserveddomain

SNP IDs	NCBI dbSNP accession IDs (NCBI_SS#)	Chromosomes	Physical positions (bp)	SNPs	Gene accession IDs	Sequence components of genome	Putative functions
Kabuli_wild_37082797	1399939196	*Ca_kabuli_Chr05*	37082797	G/T	Gene_Ca_01507	SYNONYMOUS_CODING	GRAM
Kabuli_wild_37083275	1399939197	*Ca_kabuli_Chr05*	37083275	G/T	Gene_Ca_01507	INTRON	GRAM
Kabuli_wild_37083276	1399939198	*Ca_kabuli_Chr05*	37083276	T/G	Gene_Ca_01507	INTRON	GRAM
Kabuli_wild_37113187	1399939199	*Ca_kabuli_Chr05*	37113187	C/A	Gene_Ca_01505	INTRON	WD40 repeat
Kabuli_wild_37126371	1399939200	*Ca_kabuli_Chr05*	37126371	A/G	NA	INTERGENIC	NA
Kabuli_wild_37174786	1399939201	*Ca_kabuli_Chr05*	37174786	T/C	Gene_Ca_01500	SYNONYMOUS_CODING	Rho GTPase-activating protein domain
Kabuli_wild_37197321	1399939202	*Ca_kabuli_Chr05*	37197321	C/A	Gene_Ca_01498	DOWNSTREAM	NA
Kabuli_wild_37207855	1399939203	*Ca_kabuli_Chr05*	37207855	G/T	Gene_Ca_01496	NON_SYNONYMOUS_CODING	PeptidaseC19,ubiquitincarboxyl-terminalhydrolase2
Kabuli_wild_37260632	1399939204	*Ca_kabuli_Chr05*	37260632	T/C	NA	INTERGENIC	NA
Kabuli_wild_37274572	1399939205	*Ca_kabuli_Chr05*	37274572	C/T	NA	INTERGENIC	NA
Kabuli_wild_37274583	1399939206	*Ca_kabuli_Chr05*	37274583	C/T	NA	INTERGENIC	NA
Kabuli_wild_37274616	1399939207	*Ca_kabuli_Chr05*	37274616	C/G	NA	INTERGENIC	NA
Kabuli_wild_37309935	1399939208	*Ca_kabuli_Chr05*	37309935	T/G	Gene_Ca_01488	NON_SYNONYMOUS_CODING	GDP-fucoseproteinO-fucosyltransferase
Kabuli_wild_37339094	1399939209	*Ca_kabuli_Chr05*	37339094	C/A	Gene_Ca_01485	NON_SYNONYMOUS_CODING	NA
Kabuli_wild_37375575	1399939210	*Ca_kabuli_Chr05*	37375575	C/G	Gene_Ca_01480	INTRON	Flavodoxin
Kabuli_wild_37425779	1399939211	*Ca_kabuli_Chr05*	37425779	T/C	Gene_Ca_01477	NON_SYNONYMOUS_CODING	Double-strandedRNA-binding
Kabuli_wild_37460979	1399939212	*Ca_kabuli_Chr05*	37460979	C/A	NA	INTERGENIC	NA
Kabuli_wild_37460980	1399939213	*Ca_kabuli_Chr05*	37460980	A/G	NA	INTERGENIC	NA
Kabuli_wild_37472968	1399939214	*Ca_kabuli_Chr05*	37472968	A/G	NA	INTERGENIC	NA
Kabuli_wild_37555846	1399939215	*Ca_kabuli_Chr05*	37555846	T/A	Gene_Ca_01465	INTRON	Domain of unknown function FMP27,domain-4
Kabuli_wild_37555877	1399939216	*Ca_kabuli_Chr05*	37555877	A/G	Gene_Ca_01465	INTRON	Domain of unknown function FMP27,domain-4
Kabuli_wild_37578999	1399939217	*Ca_kabuli_Chr05*	37578999	T/G	Gene_Ca_01463	INTRON	Protein of unknown function DUF914,eukaryotic
Kabuli_wild_37596144	1399939218	*Ca_kabuli_Chr05*	37596144	T/A	Gene_Ca_01462	SYNONYMOUS_CODING	Ubiquitin-associated/translation elongation factor EF1B, N-terminal
Kabuli_wild_37604030	1399939219	*Ca_kabuli_Chr05*	37604030	T/C	NA	INTERGENIC	NA
Kabuli_wild_37874725	1399939220	*Ca_kabuli_Chr05*	37874725	A/C	Gene_Ca_01436	UPSTREAM	NA
Kabuli_wild_37886413	1399939221	*Ca_kabuli_Chr05*	37886413	C/G	Gene_Ca_01435	DOWNSTREAM	NA
Kabuli_wild_37886417	1399939222	*Ca_kabuli_Chr05*	37886417	T/G	Gene_Ca_01435	DOWNSTREAM	NA
Kabuli_wild_37899098	1399939223	*Ca_kabuli_Chr05*	37899098	T/C	NA	INTERGENIC	NA
Kabuli_wild_37899183	1399939224	*Ca_kabuli_Chr05*	37899183	A/C	NA	INTERGENIC	NA
Kabuli_wild_37911818	1399939225	*Ca_kabuli_Chr05*	37911818	A/C	Gene_Ca_01431	UPSTREAM	NA
Kabuli_wild_37911819	1399939226	*Ca_kabuli_Chr05*	37911819	G/A	Gene_Ca_01431	UPSTREAM	NA
Kabuli_wild_38018552	1399939227	*Ca_kabuli_Chr05*	38018552	A/T	NA	INTERGENIC	NA
Kabuli_wild_38018558	1399939228	*Ca_kabuli_Chr05*	38018558	G/C	NA	INTERGENIC	NA
Kabuli_wild_38018575	1399939229	*Ca_kabuli_Chr05*	38018575	C/T	NA	INTERGENIC	NA
Kabuli_wild_38057582	1399939230	*Ca_kabuli_Chr05*	38057582	C/T	Gene_Ca_01417	NON_SYNONYMOUS_CODING	Zinc finger,DHHC-type,palmitoyltransferase
Kabuli_wild_38062576	1399939231	*Ca_kabuli_Chr05*	38062576	G/A	Gene_Ca_01415	UPSTREAM	Mitochondrial inner membrane translocase complex,Tim 8/9/10/13-Zinc finger-like
Kabuli_wild_38066502	1399939232	*Ca_kabuli_Chr05*	38066502	C/G	NA	INTERGENIC	NA
Kabuli_wild_38066557	1399939233	*Ca_kabuli_Chr05*	38066557	C/G	NA	INTERGENIC	NA
Kabuli_wild_38080226	1399939234	*Ca_kabuli_Chr05*	38080226	A/G	Gene_Ca_01411	SYNONYMOUS_CODING	Protein kinase,catalytic domain
Kabuli_wild_38080349	1399939235	*Ca_kabuli_Chr05*	38080349	C/T	Gene_Ca_01411	SYNONYMOUS_CODING	Protein kinase,catalytic domain
Kabuli_wild_38092606	1399939236	*Ca_kabuli_Chr05*	38092606	G/A	NA	INTERGENIC	NA
Kabuli_wild_38092685	1399939237	*Ca_kabuli_Chr05*	38092685	T/A	NA	INTERGENIC	NA
Kabuli_wild_38092732	1399939238	*Ca_kabuli_Chr05*	38092732	C/T	NA	INTERGENIC	NA
Kabuli_wild_38092735	1399939239	*Ca_kabuli_Chr05*	38092735	C/T	NA	INTERGENIC	NA

SNP IDs	NCBI dbSNP accession IDs (NCBI_SS#)	Chromosomes	Physical positions (bp)	SNPs	Gene accession IDs	Sequence components of genome	Putative functions
Kabuli_wild_38092793	1399939240	*Ca_kabuli_Chr05*	38092793	C/T	NA	INTERGENIC	NA
Kabuli_wild_38102941	1399939241	*Ca_kabuli_Chr05*	38102941	T/G	Gene_Ca_01408	SYNONYMOUS_CODING	NA
Kabuli_wild_38108396	1399939242	*Ca_kabuli_Chr05*	38108396	G/A	Gene_Ca_01407	NON_SYNONYMOUS_CODING	NA
Kabuli_wild_38110219	1399939243	*Ca_kabuli_Chr05*	38110219	T/G	NA	INTERGENIC	NA
Kabuli_wild_38183662	1399939244	*Ca_kabuli_Chr05*	38183662	T/A	Gene_Ca_01400	INTRON	NA
Kabuli_wild_38216058	1399939245	*Ca_kabuli_Chr05*	38216058	T/C	NA	INTERGENIC	NA
Kabuli_wild_38220843	1399939246	*Ca_kabuli_Chr05*	38220843	G/A	NA	INTERGENIC	NA
Kabuli_wild_38248788	1399939247	*Ca_kabuli_Chr05*	38248788	T/G	Gene_Ca_01398	SYNONYMOUS_CODING	Dual specificity phosphatase, catalytic domain
Kabuli_wild_38307036	1399939248	*Ca_kabuli_Chr05*	38307036	G/A	Gene_Ca_01392	SYNONYMOUS_CODING	Glycosidehydrolase,family38,core
Kabuli_wild_38347201	1399939249	*Ca_kabuli_Chr05*	38347201	A/C	Gene_Ca_01389	SYNONYMOUS_CODING	WD40 repeat
Kabuli_wild_38347202	1399939250	*Ca_kabuli_Chr05*	38347202	G/T	Gene_Ca_01389	NON_SYNONYMOUS_CODING	WD40 repeat
Kabuli_wild_38455563	1399939251	*Ca_kabuli_Chr05*	38455563	A/C	Gene_Ca_07405	SYNONYMOUS_CODING	Telomerel ength regulation protein,conserved domain
Kabuli_wild_38483396	1399939252	*Ca_kabuli_Chr05*	38483396	G/T	Gene_Ca_07407	DOWNSTREAM	Elongatorprotein3/MiaB/NifB
Kabuli_wild_38509657	1399939253	*Ca_kabuli_Chr05*	38509657	A/C	Gene_Ca_07410	INTRON	Transcriptional factor B3
Kabuli_wild_38538904	1399939254	*Ca_kabuli_Chr05*	38538904	A/G	Gene_Ca_07411	INTRON	Glycosyltransferase,family48
Kabuli_wild_38551438	1399939255	*Ca_kabuli_Chr05*	38551438	C/T	Gene_Ca_07411	DOWNSTREAM	Glycosyltransferase,family48
Kabuli_wild_38636510	1399939256	*Ca_kabuli_Chr05*	38636510	A/G	NA	INTERGENIC	NA
Kabuli_wild_38783578	1399939257	*Ca_kabuli_Chr05*	38783578	C/A	Gene_Ca_07428	NON_SYNONYMOUS_CODING	Mediator complex subunit Med28
Kabuli_wild_38783604	1399939258	*Ca_kabuli_Chr05*	38783604	C/A	Gene_Ca_07428	NON_SYNONYMOUS_CODING	Mediator complex subunit Med28
Kabuli_wild_38857904	1399939259	*Ca_kabuli_Chr05*	38857904	T/C	Gene_Ca_07437	DOWNSTREAM	Cytochrome P450
Kabuli_wild_38892558	1399939260	*Ca_kabuli_Chr05*	38892558	T/G	NA	INTERGENIC	NA
Kabuli_wild_38892604	1399939261	*Ca_kabuli_Chr05*	38892604	A/C	NA	INTERGENIC	NA
Kabuli_wild_38892618	1399939262	*Ca_kabuli_Chr05*	38892618	A/G	NA	INTERGENIC	NA
Kabuli_wild_38898971	1399939263	*Ca_kabuli_Chr05*	38898971	G/A	NA	INTERGENIC	NA
Kabuli_wild_38909701	1399939264	*Ca_kabuli_Chr05*	38909701	C/T	Gene_Ca_07443	NON_SYNONYMOUS_CODING	Droughtinduced19/RING finger protein114
Kabuli_wild_38931963	1399939265	*Ca_kabuli_Chr05*	38931963	C/T	NA	INTERGENIC	NA
Kabuli_wild_38931980	1399939266	*Ca_kabuli_Chr05*	38931980	G/A	NA	INTERGENIC	NA
Kabuli_wild_38937344	1399939267	*Ca_kabuli_Chr05*	38937344	A/C	Gene_Ca_07446	NON_SYNONYMOUS_CODING	ChaperoninClpA/B
Kabuli_wild_38939524	1399939268	*Ca_kabuli_Chr05*	38939524	A/C	Gene_Ca_07446	NON_SYNONYMOUS_CODING	ChaperoninClpA/B
Kabuli_wild_38977439	1399939269	*Ca_kabuli_Chr05*	38977439	A/C	NA	INTERGENIC	NA
Kabuli_wild_39283856	1399939270	*Ca_kabuli_Chr05*	39283856	G/A	Gene_Ca_07474	INTRON	NA
Kabuli_wild_39326933	1399939271	*Ca_kabuli_Chr05*	39326933	G/T	NA	INTERGENIC	NA
Kabuli_wild_39364522	1399939272	*Ca_kabuli_Chr05*	39364522	T/C	NA	INTERGENIC	NA
Kabuli_wild_39364569	1399939273	*Ca_kabuli_Chr05*	39364569	G/A	NA	INTERGENIC	NA
Kabuli_wild_39364571	1399939274	*Ca_kabuli_Chr05*	39364571	C/A	NA	INTERGENIC	NA
Kabuli_wild_39364667	1399939275	*Ca_kabuli_Chr05*	39364667	T/A	NA	INTERGENIC	NA
Kabuli_wild_39364695	1399939276	*Ca_kabuli_Chr05*	39364695	A/C	NA	INTERGENIC	NA
Kabuli_wild_39364700	1399939277	*Ca_kabuli_Chr05*	39364700	C/A	NA	INTERGENIC	NA
Kabuli_wild_39364720	1399939278	*Ca_kabuli_Chr05*	39364720	A/T	NA	INTERGENIC	NA
Kabuli_wild_39364741	1399939279	*Ca_kabuli_Chr05*	39364741	A/T	NA	INTERGENIC	NA
Kabuli_wild_39364752	1399939280	*Ca_kabuli_Chr05*	39364752	G/T	NA	INTERGENIC	NA
Kabuli_wild_39364841	1399939281	*Ca_kabuli_Chr05*	39364841	A/T	NA	INTERGENIC	NA
Kabuli_wild_39364848	1399939282	*Ca_kabuli_Chr05*	39364848	C/T	NA	INTERGENIC	NA
Kabuli_wild_39364856	1399939283	*Ca_kabuli_Chr05*	39364856	A/G	NA	INTERGENIC	NA

SNP IDs	NCBI dbSNP accession IDs (NCBI_SS#)	Chromosomes	Physical positions (bp)	SNPs	Gene accession IDs	Sequence components of genome	Putative functions
Kabuli_wild_39364895	1399939284	*Ca_kabuli_Chr05*	39364895	A/C	NA	INTERGENIC	NA
Kabuli_wild_39366353	1399939285	*Ca_kabuli_Chr05*	39366353	C/G	NA	INTERGENIC	NA
Kabuli_wild_39385592	1399939286	*Ca_kabuli_Chr05*	39385592	C/G	NA	INTERGENIC	NA
Kabuli_wild_39385603	1399939287	*Ca_kabuli_Chr05*	39385603	T/G	NA	INTERGENIC	NA
Kabuli_wild_39385630	1399939288	*Ca_kabuli_Chr05*	39385630	C/T	NA	INTERGENIC	NA
Kabuli_wild_39385676	1399939289	*Ca_kabuli_Chr05*	39385676	G/A	NA	INTERGENIC	NA
Kabuli_wild_39385682	1399939290	*Ca_kabuli_Chr05*	39385682	A/T	NA	INTERGENIC	NA
Kabuli_wild_39385688	1399939291	*Ca_kabuli_Chr05*	39385688	T/C	NA	INTERGENIC	NA
Kabuli_wild_39410329	1399939292	*Ca_kabuli_Chr05*	39410329	T/G	Gene_Ca_07478	INTRON	Glycosyltransferase,family3
Kabuli_wild_39421069	1399939293	*Ca_kabuli_Chr05*	39421069	T/G	Gene_Ca_07479	INTRON	Phoxhomologousdomain
Kabuli_wild_39489155	1399939294	*Ca_kabuli_Chr05*	39489155	A/G	Gene_Ca_07485	UPSTREAM	NA
Kabuli_wild_39489160	1399939295	*Ca_kabuli_Chr05*	39489160	C/A	Gene_Ca_07485	UPSTREAM	NA
Kabuli_wild_39489173	1399939296	*Ca_kabuli_Chr05*	39489173	C/A	Gene_Ca_07485	UPSTREAM	NA
Kabuli_wild_39489179	1399939297	*Ca_kabuli_Chr05*	39489179	C/A	Gene_Ca_07485	UPSTREAM	NA
Kabuli_wild_39489212	1399939298	*Ca_kabuli_Chr05*	39489212	G/A	Gene_Ca_07485	UPSTREAM	NA
Kabuli_wild_39489217	1399939299	*Ca_kabuli_Chr05*	39489217	C/G	Gene_Ca_07485	UPSTREAM	NA
Kabuli_wild_39543929	1399939300	*Ca_kabuli_Chr05*	39543929	C/T	Gene_Ca_07489	NON_SYNONYMOUS_CODING	Phosphatidylinositol3-/4-kinase,catalytic
Kabuli_wild_39576823	1399939301	*Ca_kabuli_Chr05*	39576823	C/T	Gene_Ca_07489	INTRON	Phosphatidylinositol3-/4-kinase,catalytic
Kabuli_wild_39576836	1399939302	*Ca_kabuli_Chr05*	39576836	T/A	Gene_Ca_07489	INTRON	Phosphatidylinositol3-/4-kinase,catalytic
Kabuli_wild_39576839	1399939303	*Ca_kabuli_Chr05*	39576839	C/T	Gene_Ca_07489	INTRON	Phosphatidylinositol3-/4-kinase,catalytic
Kabuli_wild_39576841	1399939304	*Ca_kabuli_Chr05*	39576841	G/A	Gene_Ca_07489	INTRON	Phosphatidylinositol3-/4-kinase,catalytic
Kabuli_wild_39576864	1399939305	*Ca_kabuli_Chr05*	39576864	C/A	Gene_Ca_07489	INTRON	Phosphatidylinositol3-/4-kinase,catalytic
Kabuli_wild_39576870	1399939306	*Ca_kabuli_Chr05*	39576870	C/T	Gene_Ca_07489	INTRON	Phosphatidylinositol3-/4-kinase,catalytic
Kabuli_wild_39576874	1399939307	*Ca_kabuli_Chr05*	39576874	G/T	Gene_Ca_07489	INTRON	Phosphatidylinositol3-/4-kinase,catalytic
Kabuli_wild_39576969	1399939308	*Ca_kabuli_Chr05*	39576969	C/T	Gene_Ca_07489	INTRON	Phosphatidylinositol3-/4-kinase,catalytic
Kabuli_wild_39597485	1399939309	*Ca_kabuli_Chr05*	39597485	C/T	Gene_Ca_07489	INTRON	Phosphatidylinositol3-/4-kinase,catalytic
Kabuli_wild_39608204	1399939310	*Ca_kabuli_Chr05*	39608204	A/G	Gene_Ca_07489	SYNONYMOUS_CODING	Phosphatidylinositol3-/4-kinase,catalytic
Kabuli_wild_39608209	1399939311	*Ca_kabuli_Chr05*	39608209	G/T	Gene_Ca_07489	INTRON	Phosphatidylinositol3-/4-kinase,catalytic
Kabuli_wild_39760214	1399939312	*Ca_kabuli_Chr05*	39760214	T/A	NA	INTERGENIC	NA
Kabuli_wild_39776123	1399939313	*Ca_kabuli_Chr05*	39776123	C/T	NA	INTERGENIC	NA
Kabuli_wild_39776818	1399939314	*Ca_kabuli_Chr05*	39776818	C/T	NA	INTERGENIC	NA
Kabuli_wild_39776917	1399939315	*Ca_kabuli_Chr05*	39776917	T/C	NA	INTERGENIC	NA
Kabuli_wild_39776919	1399939316	*Ca_kabuli_Chr05*	39776919	A/G	NA	INTERGENIC	NA
Kabuli_wild_39776920	1399939317	*Ca_kabuli_Chr05*	39776920	G/A	NA	INTERGENIC	NA
Kabuli_wild_39776938	1399939318	*Ca_kabuli_Chr05*	39776938	C/G	NA	INTERGENIC	NA
Kabuli_wild_39776940	1399939319	*Ca_kabuli_Chr05*	39776940	T/G	NA	INTERGENIC	NA
Kabuli_wild_39831632	1399939320	*Ca_kabuli_Chr05*	39831632	T/A	Gene_Ca_07508	SYNONYMOUS_CODING	Glycosyltransferase,family20
Kabuli_wild_39847512	1399939321	*Ca_kabuli_Chr05*	39847512	C/T	NA	INTERGENIC	NA
Kabuli_wild_39869398	1399939322	*Ca_kabuli_Chr05*	39869398	C/T	Gene_Ca_07512	SYNONYMOUS_CODING	Xanthine/uracil/vitamin C permease
Kabuli_wild_39943275	1399939323	*Ca_kabuli_Chr05*	39943275	T/C	NA	INTERGENIC	NA
Kabuli_wild_39943298	1399939324	*Ca_kabuli_Chr05*	39943298	T/C	NA	INTERGENIC	NA
Kabuli_wild_39985332	1399939325	*Ca_kabuli_Chr05*	39985332	A/G	NA	INTERGENIC	NA
Kabuli_wild_40018264	1399939326	*Ca_kabuli_Chr05*	40018264	A/C	NA	INTERGENIC	NA
Kabuli_wild_40025376	1399939327	*Ca_kabuli_Chr05*	40025376	A/G	Gene_Ca_07531	INTRON	Argonaute/Dicerprotein,PAZ

SNP IDs	NCBI dbSNP accession IDs (NCBI_SS#)	Chromosomes	Physical positions (bp)	SNPs	Gene accession IDs	Sequence components of genome	Putative functions
Kabuli_wild_40025377	1399939328	*Ca_kabuli_Chr05*	40025377	T/A	Gene_Ca_07531	INTRON	Argonaute/Dicerprotein,PAZ
Kabuli_wild_40159359	1399939329	*Ca_kabuli_Chr05*	40159359	C/T	Gene_Ca_07540	SYNONYMOUS_CODING	Nse1 non-SMC component of SMC5-6 complex
Kabuli_wild_40162755	1399939330	*Ca_kabuli_Chr05*	40162755	T/C	NA	INTERGENIC	NA
Kabuli_wild_40164176	1399939331	*Ca_kabuli_Chr05*	40164176	C/G	NA	INTERGENIC	NA
Kabuli_wild_40164221	1399939332	*Ca_kabuli_Chr05*	40164221	C/T	NA	INTERGENIC	NA
Kabuli_wild_40184049	1399939333	*Ca_kabuli_Chr05*	40184049	G/A	NA	INTERGENIC	NA
Kabuli_wild_40213494	1399939334	*Ca_kabuli_Chr05*	40213494	T/C	NA	INTERGENIC	NA
Kabuli_wild_40213545	1399939335	*Ca_kabuli_Chr05*	40213545	G/A	NA	INTERGENIC	NA
Kabuli_wild_40464449	1399939336	*Ca_kabuli_Chr05*	40464449	T/A	NA	INTERGENIC	NA
Kabuli_wild_40464608	1399939337	*Ca_kabuli_Chr05*	40464608	A/G	NA	INTERGENIC	NA
Kabuli_wild_40465463	1399939338	*Ca_kabuli_Chr05*	40465463	A/C	Gene_Ca_07566	INTRON	Methionine synthase, vitamin-B12 independent
Kabuli_wild_40505573	1399939339	*Ca_kabuli_Chr05*	40505573	T/C	Gene_Ca_07571	INTRON	Zinc finger,CCHC-type
Kabuli_wild_40505592	1399939340	*Ca_kabuli_Chr05*	40505592	T/C	Gene_Ca_07571	NON_SYNONYMOUS_CODING	Zinc finger,CCHC-type
Kabuli_wild_40507018	1399939341	*Ca_kabuli_Chr05*	40507018	T/A	Gene_Ca_07571	INTRON	Zinc finger,CCHC-type
Kabuli_wild_40527587	1399939342	*Ca_kabuli_Chr05*	40527587	G/T	NA	INTERGENIC	NA
Kabuli_wild_40532433	1399939343	*Ca_kabuli_Chr05*	40532433	G/T	NA	INTERGENIC	NA
Kabuli_wild_40532435	1399939344	*Ca_kabuli_Chr05*	40532435	A/G	NA	INTERGENIC	NA
Kabuli_wild_40532449	1399939345	*Ca_kabuli_Chr05*	40532449	T/G	NA	INTERGENIC	NA
Kabuli_wild_40555799	1399939346	*Ca_kabuli_Chr05*	40555799	T/C	NA	INTERGENIC	NA
Kabuli_wild_40632788	1399939347	*Ca_kabuli_Chr05*	40632788	C/T	NA	INTERGENIC	NA
Kabuli_wild_40666174	1399939348	*Ca_kabuli_Chr05*	40666174	C/T	Gene_Ca_07586	INTRON	Sugar/inositol transporter
Kabuli_wild_40666176	1399939349	*Ca_kabuli_Chr05*	40666176	G/T	Gene_Ca_07586	INTRON	Sugar/inositol transporter
Kabuli_wild_40666178	1399939350	*Ca_kabuli_Chr05*	40666178	A/T	Gene_Ca_07586	INTRON	Sugar/inositol transporter
Kabuli_wild_40700318	1399939351	*Ca_kabuli_Chr05*	40700318	C/A	Gene_Ca_07589	NON_SYNONYMOUS_CODING	Glycosidehydrolase,family17
Kabuli_wild_40734317	1399939352	*Ca_kabuli_Chr05*	40734317	T/C	Gene_Ca_07594	INTRON	NA
Kabuli_wild_40736291	1399939353	*Ca_kabuli_Chr05*	40736291	C/G	Gene_Ca_07594	INTRON	NA
Kabuli_wild_40828566	1399939354	*Ca_kabuli_Chr05*	40828566	A/C	NA	INTERGENIC	NA
Kabuli_wild_40828567	1399939355	*Ca_kabuli_Chr05*	40828567	G/C	NA	INTERGENIC	NA
Kabuli_wild_40841690	1399939356	*Ca_kabuli_Chr05*	40841690	C/T	Gene_Ca_07604	NON_SYNONYMOUS_CODING	Oligo peptide transporterOPTsuperfamily
Kabuli_wild_40883438	1399939357	*Ca_kabuli_Chr05*	40883438	C/A	NA	INTERGENIC	NA
Kabuli_wild_41063657	1399939358	*Ca_kabuli_Chr05*	41063657	C/T	NA	INTERGENIC	NA
Kabuli_wild_41063696	1399939359	*Ca_kabuli_Chr05*	41063696	G/A	NA	INTERGENIC	NA
Kabuli_wild_41066644	1399939360	*Ca_kabuli_Chr05*	41066644	C/G	NA	INTERGENIC	NA
Kabuli_wild_41066664	1399939361	*Ca_kabuli_Chr05*	41066664	T/C	NA	INTERGENIC	NA
Kabuli_wild_41066667	1399939362	*Ca_kabuli_Chr05*	41066667	C/A	NA	INTERGENIC	NA
Kabuli_wild_41197909	1399939363	*Ca_kabuli_Chr05*	41197909	A/T	NA	INTERGENIC	NA
Kabuli_wild_41197924	1399939364	*Ca_kabuli_Chr05*	41197924	A/T	NA	INTERGENIC	NA
Kabuli_wild_41201628	1399939365	*Ca_kabuli_Chr05*	41201628	A/T	NA	INTERGENIC	NA
Kabuli_wild_41228098	1399939366	*Ca_kabuli_Chr05*	41228098	T/C	Gene_Ca_07639	NON_SYNONYMOUS_CODING	NA
Kabuli_wild_41282635	1399939367	*Ca_kabuli_Chr05*	41282635	A/G	NA	INTERGENIC	NA
Kabuli_wild_41283253	1399939368	*Ca_kabuli_Chr05*	41283253	T/C	Gene_Ca_07642	NON_SYNONYMOUS_CODING	Protein of unknown function DUF1296
Kabuli_wild_41283453	1399939369	*Ca_kabuli_Chr05*	41283453	A/C	Gene_Ca_07642	NON_SYNONYMOUS_CODING	Protein of unknown function DUF1296
Kabuli_wild_41407105	1399939370	*Ca_kabuli_Chr05*	41407105	A/G	NA	INTERGENIC	NA
Kabuli_wild_41407253	1399939371	*Ca_kabuli_Chr05*	41407253	T/C	NA	INTERGENIC	NA

SNP IDs	NCBI dbSNP accession IDs (NCBI_SS#)	Chromosomes	Physical positions (bp)	SNPs	Gene accession IDs	Sequence components of genome	Putative functions
Kabuli_wild_41678144	1399939372	*Ca_kabuli_Chr05*	41678144	C/T	Gene_Ca_19215	SYNONYMOUS_CODING	Argonaute/Dicerprotein,PAZ
Kabuli_wild_41863617	1399939373	*Ca_kabuli_Chr05*	41863617	G/T	NA	INTERGENIC	NA
Kabuli_wild_41887872	1399939374	*Ca_kabuli_Chr05*	41887872	C/A	Gene_Ca_11425	DOWNSTREAM	NA
Kabuli_wild_41887879	1399939375	*Ca_kabuli_Chr05*	41887879	C/A	Gene_Ca_11425	DOWNSTREAM	NA
Kabuli_wild_41902363	1399939376	*Ca_kabuli_Chr05*	41902363	G/A	Gene_Ca_11423	NON_SYNONYMOUS_CODING	PeptidaseC19,ubiquitincarboxyl-terminalhydrolase2
Kabuli_wild_41945308	1399939377	*Ca_kabuli_Chr05*	41945308	G/A	Gene_Ca_11418	NON_SYNONYMOUS_CODING	Helicase,C-terminal
Kabuli_wild_42004600	1399939378	*Ca_kabuli_Chr05*	42004600	T/G	Gene_Ca_11412	INTRON	Rab-GAP/TBCdomain
Kabuli_wild_42004602	1399939379	*Ca_kabuli_Chr05*	42004602	A/G	Gene_Ca_11412	INTRON	Rab-GAP/TBCdomain
Kabuli_wild_42044863	1399939380	*Ca_kabuli_Chr05*	42044863	T/G	Gene_Ca_11405	SYNONYMOUS_CODING	Rhodanese-like
Kabuli_wild_42225634	1399939381	*Ca_kabuli_Chr05*	42225634	G/A	NA	INTERGENIC	NA
Kabuli_wild_42282252	1399939382	*Ca_kabuli_Chr05*	42282252	C/T	Gene_Ca_11387	INTRON	AMP-dependentsynthetase/ligase
Kabuli_wild_42413430	1399939383	*Ca_kabuli_Chr05*	42413430	A/C	Gene_Ca_11377	INTRON	Zinc finger,Sec23/Sec24-type
Kabuli_wild_42436972	1399939384	*Ca_kabuli_Chr05*	42436972	A/G	Gene_Ca_11373	UPSTREAM	DNAtopoisomerase,typeIA
Kabuli_wild_42436976	1399939385	*Ca_kabuli_Chr05*	42436976	A/T	Gene_Ca_11373	UPSTREAM	DNAtopoisomerase,typeIA
Kabuli_wild_42528744	1399939386	*Ca_kabuli_Chr05*	42528744	T/G	Gene_Ca_11361	NON_SYNONYMOUS_CODING	IQ motif,EF-handbinding site
Kabuli_wild_42528934	1399939387	*Ca_kabuli_Chr05*	42528934	A/G	Gene_Ca_11361	NON_SYNONYMOUS_CODING	IQ motif,EF-handbinding site
Kabuli_wild_42540636	1399939388	*Ca_kabuli_Chr05*	42540636	G/C	Gene_Ca_11358	NON_SYNONYMOUS_CODING	Signal transduction response regulator, receiverdomain
Kabuli_wild_42749499	1399939389	*Ca_kabuli_Chr05*	42749499	C/T	Gene_Ca_11334	INTRON	NA
Kabuli_wild_42863366	1399939390	*Ca_kabuli_Chr05*	42863366	C/T	NA	INTERGENIC	NA
Kabuli_wild_42863405	1399939391	*Ca_kabuli_Chr05*	42863405	G/T	NA	INTERGENIC	NA
Kabuli_wild_42883163	1399939392	*Ca_kabuli_Chr05*	42883163	T/C	NA	INTERGENIC	NA
Kabuli_wild_42910653	1399939393	*Ca_kabuli_Chr05*	42910653	A/G	NA	INTERGENIC	NA
Kabuli_wild_42924167	1399939394	*Ca_kabuli_Chr05*	42924167	G/A	NA	INTERGENIC	NA
Kabuli_wild_42928986	1399939395	*Ca_kabuli_Chr05*	42928986	T/G	Gene_Ca_11315	SYNONYMOUS_CODING	SANT domain,DNA binding
Kabuli_wild_42928995	1399939396	*Ca_kabuli_Chr05*	42928995	T/G	Gene_Ca_11315	SYNONYMOUS_CODING	SANT domain,DNA binding
Kabuli_wild_42929087	1399939397	*Ca_kabuli_Chr05*	42929087	A/C	Gene_Ca_11315	UPSTREAM	SANT domain,DNA binding
Kabuli_wild_42977337	1399939398	*Ca_kabuli_Chr05*	42977337	C/A	NA	INTERGENIC	NA
Kabuli_wild_43012119	1399939399	*Ca_kabuli_Chr05*	43012119	A/C	NA	INTERGENIC	NA
Kabuli_wild_43037863	1399939400	*Ca_kabuli_Chr05*	43037863	G/A	Gene_Ca_11305	NON_SYNONYMOUS_CODING	RegulatorofGproteinsignalling
Kabuli_wild_43056159	1399939401	*Ca_kabuli_Chr05*	43056159	G/T	Gene_Ca_11302	NON_SYNONYMOUS_CODING	NA
Kabuli_wild_43067905	1399939402	*Ca_kabuli_Chr05*	43067905	A/C	Gene_Ca_11300	SYNONYMOUS_CODING	Membraneinsertionprotein,OxaA/YidC
Kabuli_wild_43080493	1399939403	*Ca_kabuli_Chr05*	43080493	G/A	Gene_Ca_11299	NON_SYNONYMOUS_CODING	Regulatorofchromosomecondensation,RCC1
Kabuli_wild_43167549	1399939404	*Ca_kabuli_Chr05*	43167549	C/T	Gene_Ca_12742	INTRON	SDA1
Kabuli_wild_43231220	1399939405	*Ca_kabuli_Chr05*	43231220	T/C	Gene_Ca_12734	SYNONYMOUS_CODING	Protein kinase,catalytic domain
Kabuli_wild_43275472	1399939406	*Ca_kabuli_Chr05*	43275472	T/C	NA	INTERGENIC	NA
Kabuli_wild_43366590	1399939407	*Ca_kabuli_Chr05*	43366590	G/T	Gene_Ca_12719	NON_SYNONYMOUS_CODING	Aminotransferase-like,plant mobile domain
Kabuli_wild_43710359	1399939408	*Ca_kabuli_Chr05*	43710359	T/G	Gene_Ca_12681	DOWNSTREAM	Sulphate transporter/antisigma-factor antagonist STAS
Kabuli_wild_43710362	1399939409	*Ca_kabuli_Chr05*	43710362	A/T	Gene_Ca_12681	DOWNSTREAM	Sulphate transporter/antisigma-factor antagonist STAS
Kabuli_wild_43710366	1399939410	*Ca_kabuli_Chr05*	43710366	G/T	Gene_Ca_12681	DOWNSTREAM	Sulphate transporter/antisigma-factor antagonist STAS
Kabuli_wild_43710372	1399939411	*Ca_kabuli_Chr05*	43710372	A/T	Gene_Ca_12681	DOWNSTREAM	Sulphate transporter/antisigma-factor antagonist STAS
Kabuli_wild_43710391	1399939412	*Ca_kabuli_Chr05*	43710391	A/G	Gene_Ca_12681	DOWNSTREAM	Sulphate transporter/antisigma-factor antagonist STAS
Kabuli_wild_43742448	1399939413	*Ca_kabuli_Chr05*	43742448	C/T	NA	INTERGENIC	NA
Kabuli_wild_43742456	1399939414	*Ca_kabuli_Chr05*	43742456	G/A	NA	INTERGENIC	NA
Kabuli_wild_43759059	1399939415	*Ca_kabuli_Chr05*	43759059	A/C	Gene_Ca_12676	NON_SYNONYMOUS_CODING	C2 calcium-dependent membrane targeting

SNP IDs	NCBI dbSNP accession IDs (NCBI_SS#)	Chromosomes	Physical positions (bp)	SNPs	Gene accession IDs	Sequence components of genome	Putative functions
Kabuli_wild_43786846	1399939416	*Ca_kabuli_Chr05*	43786846	T/C	Gene_Ca_12674	NON_SYNONYMOUS_CODING	RNApolymeraseII,largesubunit,CTD
Kabuli_wild_43789957	1399939417	*Ca_kabuli_Chr05*	43789957	G/T	NA	INTERGENIC	NA
Kabuli_wild_43904489	1399939418	*Ca_kabuli_Chr05*	43904489	T/A	NA	INTERGENIC	NA
Kabuli_wild_43919009	1399939419	*Ca_kabuli_Chr05*	43919009	C/T	NA	INTERGENIC	NA
Kabuli_wild_43919017	1399939420	*Ca_kabuli_Chr05*	43919017	T/G	NA	INTERGENIC	NA
Kabuli_wild_43919022	1399939421	*Ca_kabuli_Chr05*	43919022	G/C	NA	INTERGENIC	NA
Kabuli_wild_43919028	1399939422	*Ca_kabuli_Chr05*	43919028	A/G	NA	INTERGENIC	NA
Kabuli_wild_43952287	1399939423	*Ca_kabuli_Chr05*	43952287	C/T	Gene_Ca_12660	NON_SYNONYMOUS_CODING	No apical meristem (NAM) protein
Kabuli_wild_43952312	1399939424	*Ca_kabuli_Chr05*	43952312	T/C	Gene_Ca_12660	NON_SYNONYMOUS_CODING	No apical meristem (NAM) protein
Kabuli_wild_43952345	1399939425	*Ca_kabuli_Chr05*	43952345	C/T	Gene_Ca_12660	NON_SYNONYMOUS_CODING	No apical meristem (NAM) protein
Kabuli_wild_44098507	1399939426	*Ca_kabuli_Chr05*	44098507	T/A	Gene_Ca_12648	STOP_GAINED	Tetrapyrrolebiosynthesis,porphobilinogensynthase
Kabuli_wild_44109260	1399939427	*Ca_kabuli_Chr05*	44109260	T/G	Gene_Ca_12646	SYNONYMOUS_CODING	NA
Kabuli_wild_44159237	1399939428	*Ca_kabuli_Chr05*	44159237	C/G	NA	INTERGENIC	NA
Kabuli_wild_44299577	1399939429	*Ca_kabuli_Chr05*	44299577	A/C	Gene_Ca_03876	SYNONYMOUS_CODING	Aminoacid transporter,transmembrane
Kabuli_wild_44373968	1399939430	*Ca_kabuli_Chr05*	44373968	G/T	Gene_Ca_03885	NON_SYNONYMOUS_CODING	NA
Kabuli_wild_44378178	1399939431	*Ca_kabuli_Chr05*	44378178	C/A	Gene_Ca_03885	INTRON	NA
Kabuli_wild_44422711	1399939432	*Ca_kabuli_Chr05*	44422711	C/T	Gene_Ca_03889	INTRON	SET domain
Kabuli_wild_44555718	1399939433	*Ca_kabuli_Chr05*	44555718	A/C	NA	INTERGENIC	NA
Kabuli_wild_44656921	1399939434	*Ca_kabuli_Chr05*	44656921	G/A	NA	INTERGENIC	NA
Kabuli_wild_44667297	1399939435	*Ca_kabuli_Chr05*	44667297	C/T	NA	INTERGENIC	NA
Kabuli_wild_44760347	1399939436	*Ca_kabuli_Chr05*	44760347	G/T	Gene_Ca_03920	NON_SYNONYMOUS_CODING	Transcription facto rGRAS
Kabuli_wild_44760403	1399939437	*Ca_kabuli_Chr05*	44760403	A/G	Gene_Ca_03920	SYNONYMOUS_CODING	Transcription facto rGRAS
Kabuli_wild_44760469	1399939438	*Ca_kabuli_Chr05*	44760469	C/T	Gene_Ca_03920	SYNONYMOUS_CODING	Transcription facto rGRAS
Kabuli_wild_44769623	1399939439	*Ca_kabuli_Chr05*	44769623	T/G	NA	INTERGENIC	NA
Kabuli_wild_44772326	1399939440	*Ca_kabuli_Chr05*	44772326	G/A	NA	INTERGENIC	NA
Kabuli_wild_44772443	1399939441	*Ca_kabuli_Chr05*	44772443	C/T	NA	INTERGENIC	NA
Kabuli_wild_44887105	1399939442	*Ca_kabuli_Chr05*	44887105	T/G	Gene_Ca_03932	INTRON	Sec1-likeprotein
Kabuli_wild_44887234	1399939443	*Ca_kabuli_Chr05*	44887234	A/C	Gene_Ca_03932	INTRON	Sec1-likeprotein
Kabuli_wild_44887241	1399939444	*Ca_kabuli_Chr05*	44887241	T/C	Gene_Ca_03932	INTRON	Sec1-likeprotein
Kabuli_wild_44890500	1399939445	*Ca_kabuli_Chr05*	44890500	A/G	Gene_Ca_03933	UPSTREAM	Protein of unknown function DUF300
Kabuli_wild_44891401	1399939446	*Ca_kabuli_Chr05*	44891401	G/A	Gene_Ca_03933	UPSTREAM	Protein of unknown function DUF301
Kabuli_wild_44891426	1399939447	*Ca_kabuli_Chr05*	44891426	G/C	Gene_Ca_03933	UPSTREAM	Protein of unknown function DUF301
Kabuli_wild_44911842	1399939448	*Ca_kabuli_Chr05*	44911842	C/T	NA	INTERGENIC	NA
Kabuli_wild_44918947	1399939449	*Ca_kabuli_Chr05*	44918947	C/A	Gene_Ca_03937	DOWNSTREAM	Glycolipidtransferproteindomain
Kabuli_wild_44938814	1399939450	*Ca_kabuli_Chr05*	44938814	A/T	NA	INTERGENIC	NA
Kabuli_wild_44939033	1399939451	*Ca_kabuli_Chr05*	44939033	G/A	NA	INTERGENIC	NA
Kabuli_wild_44939058	1399939452	*Ca_kabuli_Chr05*	44939058	C/G	NA	INTERGENIC	NA
Kabuli_wild_44975251	1399939453	*Ca_kabuli_Chr05*	44975251	A/C	Gene_Ca_03944	NON_SYNONYMOUS_CODING	Glycosyltransferase,family31
Kabuli_wild_44979963	1399939454	*Ca_kabuli_Chr05*	44979963	T/G	NA	INTERGENIC	NA
Kabuli_wild_45087140	1399939455	*Ca_kabuli_Chr05*	45087140	C/A	NA	INTERGENIC	NA
Kabuli_wild_45087146	1399939456	*Ca_kabuli_Chr05*	45087146	C/G	NA	INTERGENIC	NA
Kabuli_wild_45087156	1399939457	*Ca_kabuli_Chr05*	45087156	A/G	NA	INTERGENIC	NA
Kabuli_wild_45087170	1399939458	*Ca_kabuli_Chr05*	45087170	G/A	NA	INTERGENIC	NA
Kabuli_wild_45087202	1399939459	*Ca_kabuli_Chr05*	45087202	G/C	NA	INTERGENIC	NA

SNP IDs	NCBI dbSNP accession IDs (NCBI_SS#)	Chromosomes	Physical positions (bp)	SNPs	Gene accession IDs	Sequence components of genome	Putative functions
Kabuli_wild_45087209	1399939460	*Ca_kabuli_Chr05*	45087209	T/C	NA	INTERGENIC	NA
Kabuli_wild_45087219	1399939461	*Ca_kabuli_Chr05*	45087219	T/C	NA	INTERGENIC	NA
Kabuli_wild_45087261	1399939462	*Ca_kabuli_Chr05*	45087261	C/T	NA	INTERGENIC	NA
Kabuli_wild_45185408	1399939463	*Ca_kabuli_Chr05*	45185408	T/C	Gene_Ca_03961	INTRON	Short-chain dehydrogenase/reductase SDR
Kabuli_wild_45254112	1399939464	*Ca_kabuli_Chr05*	45254112	T/G	Gene_Ca_03969	NON_SYNONYMOUS_CODING	Zinc finger,C2H2-typematrin
Kabuli_wild_45337307	1399939465	*Ca_kabuli_Chr05*	45337307	C/T	Gene_Ca_03976	SYNONYMOUS_CODING	Pectinesterase,catalytic
Kabuli_wild_45407044	1399939466	*Ca_kabuli_Chr05*	45407044	A/C	Gene_Ca_03983	INTRON	NA
Kabuli_wild_45428234	1399939467	*Ca_kabuli_Chr05*	45428234	T/G	NA	INTERGENIC	NA
Kabuli_wild_45429486	1399939468	*Ca_kabuli_Chr05*	45429486	A/C	Gene_Ca_03986	DOWNSTREAM	Longin
Kabuli_wild_45480175	1399939469	*Ca_kabuli_Chr05*	45480175	T/G	Gene_Ca_03993	DOWNSTREAM	NA
Kabuli_wild_45501379	1399939470	*Ca_kabuli_Chr05*	45501379	C/T	Gene_Ca_03996	UPSTREAM	K Homology
Kabuli_wild_45507463	1399939471	*Ca_kabuli_Chr05*	45507463	A/G	NA	INTERGENIC	NA
Kabuli_wild_45646693	1399939472	*Ca_kabuli_Chr05*	45646693	C/A	Gene_Ca_04010	NON_SYNONYMOUS_CODING	SecretorypathwaySec39
Kabuli_wild_45694453	1399939473	*Ca_kabuli_Chr05*	45694453	T/G	Gene_Ca_04017	SYNONYMOUS_CODING	ATPase,AAA+type,core
Kabuli_wild_45704206	1399939474	*Ca_kabuli_Chr05*	45704206	C/T	NA	INTERGENIC	NA
Kabuli_wild_45704207	1399939475	*Ca_kabuli_Chr05*	45704207	G/T	NA	INTERGENIC	NA
Kabuli_wild_45704298	1399939476	*Ca_kabuli_Chr05*	45704298	C/A	NA	INTERGENIC	NA
Kabuli_wild_45710476	1399939477	*Ca_kabuli_Chr05*	45710476	A/G	Gene_Ca_04018	UPSTREAM	ATPase,AAA+type,core
Kabuli_wild_45710509	1399939478	*Ca_kabuli_Chr05*	45710509	G/A	Gene_Ca_04018	UPSTREAM	ATPase,AAA+type,core
Kabuli_wild_45726955	1399939479	*Ca_kabuli_Chr05*	45726955	A/C	Gene_Ca_04021	INTRON	Cytochromeb5
Kabuli_wild_45750121	1399939480	*Ca_kabuli_Chr05*	45750121	A/T	Gene_Ca_04024	INTRON	Glucosamine/galactosamine-6-phosphateisomerase
Kabuli_wild_45831932	1399939481	*Ca_kabuli_Chr05*	45831932	A/G	Gene_Ca_04031	INTRON	NA
Kabuli_wild_45842438	1399939482	*Ca_kabuli_Chr05*	45842438	A/C	NA	INTERGENIC	NA
Kabuli_wild_45845081	1399939483	*Ca_kabuli_Chr05*	45845081	A/C	Gene_Ca_04032	UPSTREAM	SSXT
Kabuli_wild_45971233	1399939484	*Ca_kabuli_Chr05*	45971233	A/T	NA	INTERGENIC	NA
Kabuli_wild_46018939	1399939485	*Ca_kabuli_Chr05*	46018939	C/T	NA	INTERGENIC	NA
Kabuli_wild_46022566	1399939486	*Ca_kabuli_Chr05*	46022566	C/T	NA	INTERGENIC	NA
Kabuli_wild_46022598	1399939487	*Ca_kabuli_Chr05*	46022598	T/C	NA	INTERGENIC	NA
Kabuli_wild_46056900	1399939488	*Ca_kabuli_Chr05*	46056900	A/C	NA	INTERGENIC	NA
Kabuli_wild_46077201	1399939489	*Ca_kabuli_Chr05*	46077201	T/A	Gene_Ca_04056	INTRON	Signal transduction response regulator, receiverdomain
Kabuli_wild_46077302	1399939490	*Ca_kabuli_Chr05*	46077302	G/A	Gene_Ca_04056	NON_SYNONYMOUS_CODING	Signal transduction response regulator, receiverdomain
Kabuli_wild_46130289	1399939491	*Ca_kabuli_Chr05*	46130289	A/C	NA	INTERGENIC	NA
Kabuli_wild_46156213	1399939492	*Ca_kabuli_Chr05*	46156213	A/C	NA	INTERGENIC	NA
Kabuli_wild_46176443	1399939493	*Ca_kabuli_Chr05*	46176443	G/A	NA	INTERGENIC	NA
Kabuli_wild_46197746	1399939494	*Ca_kabuli_Chr05*	46197746	T/G	Gene_Ca_04072	INTRON	Band7protein
Kabuli_wild_46240664	1399939495	*Ca_kabuli_Chr05*	46240664	T/G	NA	INTERGENIC	NA
Kabuli_wild_46252605	1399939496	*Ca_kabuli_Chr05*	46252605	C/A	Gene_Ca_04077	DOWNSTREAM	NA
Kabuli_wild_46252643	1399939497	*Ca_kabuli_Chr05*	46252643	T/A	Gene_Ca_04077	DOWNSTREAM	NA
Kabuli_wild_46260828	1399939498	*Ca_kabuli_Chr05*	46260828	A/C	Gene_Ca_04079	SYNONYMOUS_CODING	Mitochondrial inner membrane translocase complex,subunitTim17/22
Kabuli_wild_46301639	1399939499	*Ca_kabuli_Chr05*	46301639	A/G	Gene_Ca_04083	SYNONYMOUS_CODING	NA
Kabuli_wild_46328627	1399939500	*Ca_kabuli_Chr05*	46328627	A/G	Gene_Ca_04088	DOWNSTREAM	Alpha/betahydrolasefold-1
Kabuli_wild_46328895	1399939501	*Ca_kabuli_Chr05*	46328895	T/C	Gene_Ca_04088	DOWNSTREAM	Protein of unknown function DUF2044,membrane
Kabuli_wild_46422869	1399939502	*Ca_kabuli_Chr05*	46422869	A/T	NA	INTERGENIC	NA
Kabuli_wild_46526963	1399939503	*Ca_kabuli_Chr05*	46526963	A/C	Gene_Ca_04110	SYNONYMOUS_CODING	Pentatrico peptide repeat

SNP IDs	NCBI dbSNP accession IDs (NCBI_SS#)	Chromosomes	Physical positions (bp)	SNPs	Gene accession IDs	Sequence components of genome	Putative functions
Kabuli_wild_46537319	1399939504	*Ca_kabuli_Chr05*	46537319	T/C	NA	INTERGENIC	NA
Kabuli_wild_46632867	1399939505	*Ca_kabuli_Chr05*	46632867	G/C	Gene_Ca_04122	DOWNSTREAM	ABC-1
Kabuli_wild_46632887	1399939506	*Ca_kabuli_Chr05*	46632887	T/C	Gene_Ca_04122	DOWNSTREAM	ABC-1
Kabuli_wild_46704196	1399939507	*Ca_kabuli_Chr05*	46704196	G/A	Gene_Ca_04129	INTRON	NA
Kabuli_wild_46713780	1399939508	*Ca_kabuli_Chr05*	46713780	A/C	Gene_Ca_04129	SYNONYMOUS_CODING	NA
Kabuli_wild_46713781	1399939509	*Ca_kabuli_Chr05*	46713781	G/T	Gene_Ca_04129	NON_SYNONYMOUS_CODING	NA
Kabuli_wild_47068061	1399939510	*Ca_kabuli_Chr05*	47068061	C/T	NA	INTERGENIC	NA
Kabuli_wild_47073011	1399939511	*Ca_kabuli_Chr05*	47073011	A/T	NA	INTERGENIC	NA
Kabuli_wild_47083588	1399939512	*Ca_kabuli_Chr05*	47083588	C/T	Gene_Ca_04166	NON_SYNONYMOUS_CODING	Autophagy-relatedprotein13
Kabuli_wild_47151831	1399939513	*Ca_kabuli_Chr05*	47151831	A/T	Gene_Ca_04172	SYNONYMOUS_CODING	WD40 repeat
Kabuli_wild_47252496	1399939514	*Ca_kabuli_Chr05*	47252496	T/A	Gene_Ca_04182	INTRON	GDP-fucoseproteinO-fucosyltransferase
Kabuli_wild_47298345	1399939515	*Ca_kabuli_Chr05*	47298345	A/G	Gene_Ca_04186	INTRON	Phosphatidylinositol3-/4-kinase,catalytic
Kabuli_wild_47329979	1399939516	*Ca_kabuli_Chr05*	47329979	T/G	Gene_Ca_04190	NON_SYNONYMOUS_CODING	Glycosyltransferase,family48
Kabuli_wild_47362666	1399939517	*Ca_kabuli_Chr05*	47362666	T/G	Gene_Ca_04193	DOWNSTREAM	NAD(P)H-quinoneoxidoreductasesubunitO
Kabuli_wild_47435931	1399939518	*Ca_kabuli_Chr05*	47435931	A/G	Gene_Ca_04206	DOWNSTREAM	Pentatrico peptide repeat
Kabuli_wild_47436163	1399939519	*Ca_kabuli_Chr05*	47436163	T/A	Gene_Ca_04206	DOWNSTREAM	Pentatrico peptide repeat
Kabuli_wild_47515887	1399939520	*Ca_kabuli_Chr05*	47515887	G/C	NA	INTERGENIC	NA
Kabuli_wild_47628599	1399939521	*Ca_kabuli_Chr05*	47628599	T/C	Gene_Ca_04223	INTRON	Regulatorofchromosomecondensation,RCC1
Kabuli_wild_47635816	1399939522	*Ca_kabuli_Chr05*	47635816	A/G	NA	INTERGENIC	NA
Kabuli_wild_47641458	1399939523	*Ca_kabuli_Chr05*	47641458	C/T	Gene_Ca_04224	NON_SYNONYMOUS_CODING	Heat shock protein DnaJ,N-terminal
Kabuli_wild_47641567	1399939524	*Ca_kabuli_Chr05*	47641567	A/G	Gene_Ca_04224	NON_SYNONYMOUS_CODING	Heat shock protein DnaJ,N-terminal
Kabuli_wild_47641628	1399939525	*Ca_kabuli_Chr05*	47641628	T/C	Gene_Ca_04224	SYNONYMOUS_CODING	Heat shock protein DnaJ,N-terminal
Kabuli_wild_47643992	1399939526	*Ca_kabuli_Chr05*	47643992	G/T	Gene_Ca_04224	INTRON	Heat shock protein DnaJ,N-terminal
Kabuli_wild_47670649	1399939527	*Ca_kabuli_Chr05*	47670649	G/A	Gene_Ca_04227	NON_SYNONYMOUS_CODING	Aminoacid transporter,transmembrane
Kabuli_wild_47684226	1399939528	*Ca_kabuli_Chr05*	47684226	A/G	NA	INTERGENIC	NA
Kabuli_wild_47684281	1399939529	*Ca_kabuli_Chr05*	47684281	G/C	NA	INTERGENIC	NA
Kabuli_wild_47718830	1399939530	*Ca_kabuli_Chr05*	47718830	A/G	Gene_Ca_04232	INTRON	Glycosidehydrolase,family35
Kabuli_wild_47759752	1399939531	*Ca_kabuli_Chr05*	47759752	T/A	Gene_Ca_04237	INTRON	Protein kinase,catalytic domain
Kabuli_wild_47759789	1399939532	*Ca_kabuli_Chr05*	47759789	A/C	Gene_Ca_04237	INTRON	Protein kinase,catalytic domain
Kabuli_wild_47770909	1399939533	*Ca_kabuli_Chr05*	47770909	T/G	Gene_Ca_04238	INTRON	Amidase
Kabuli_wild_47812041	1399939534	*Ca_kabuli_Chr05*	47812041	C/T	NA	INTERGENIC	NA
Kabuli_wild_47829331	1399939535	*Ca_kabuli_Chr05*	47829331	T/A	Gene_Ca_04244	UPSTREAM	NA
Kabuli_wild_47965279	1399939536	*Ca_kabuli_Chr05*	47965279	G/A	Gene_Ca_04258	NON_SYNONYMOUS_CODING	NA
Kabuli_wild_47994780	1399939537	*Ca_kabuli_Chr05*	47994780	A/C	Gene_Ca_04259	INTRON	Cofprotein
Kabuli_wild_48063074	1399939538	*Ca_kabuli_Chr05*	48063074	T/A	NA	INTERGENIC	NA
Kabuli_wild_48069833	1399939539	*Ca_kabuli_Chr05*	48069833	G/A	NA	INTERGENIC	NA
Kabuli_wild_146795	1399939540	*Ca_kabuli_Chr06*	146795	C/G	NA	INTERGENIC	NA
Kabuli_wild_146797	1399939541	*Ca_kabuli_Chr06*	146797	C/T	NA	INTERGENIC	NA
Kabuli_wild_152513	1399939542	*Ca_kabuli_Chr06*	152513	C/T	NA	INTERGENIC	NA
Kabuli_wild_166787	1399939543	*Ca_kabuli_Chr06*	166787	T/A	NA	INTERGENIC	NA
Kabuli_wild_166793	1399939544	*Ca_kabuli_Chr06*	166793	G/A	NA	INTERGENIC	NA
Kabuli_wild_166800	1399939545	*Ca_kabuli_Chr06*	166800	G/T	NA	INTERGENIC	NA
Kabuli_wild_166806	1399939546	*Ca_kabuli_Chr06*	166806	C/T	NA	INTERGENIC	NA
Kabuli_wild_166822	1399939547	*Ca_kabuli_Chr06*	166822	G/C	NA	INTERGENIC	NA

SNP IDs	NCBI dbSNP accession IDs (NCBI_SS#)	Chromosomes	Physical positions (bp)	SNPs	Gene accession IDs	Sequence components of genome	Putative functions
Kabuli_wild_166823	1399939548	*Ca_kabuli_Chr06*	166823	G/A	NA	INTERGENIC	NA
Kabuli_wild_166916	1399939549	*Ca_kabuli_Chr06*	166916	C/T	NA	INTERGENIC	NA
Kabuli_wild_166917	1399939550	*Ca_kabuli_Chr06*	166917	G/A	NA	INTERGENIC	NA
Kabuli_wild_166932	1399939551	*Ca_kabuli_Chr06*	166932	G/A	NA	INTERGENIC	NA
Kabuli_wild_166934	1399939552	*Ca_kabuli_Chr06*	166934	G/A	NA	INTERGENIC	NA
Kabuli_wild_166938	1399939553	*Ca_kabuli_Chr06*	166938	G/T	NA	INTERGENIC	NA
Kabuli_wild_166943	1399939554	*Ca_kabuli_Chr06*	166943	G/A	NA	INTERGENIC	NA
Kabuli_wild_166959	1399939555	*Ca_kabuli_Chr06*	166959	G/A	NA	INTERGENIC	NA
Kabuli_wild_204368	1399939556	*Ca_kabuli_Chr06*	204368	G/A	Gene_Ca_13250	INTRON	Flavin-containingamineoxidase
Kabuli_wild_204412	1399939557	*Ca_kabuli_Chr06*	204412	C/G	Gene_Ca_13250	INTRON	Flavin-containingamineoxidase
Kabuli_wild_204438	1399939558	*Ca_kabuli_Chr06*	204438	C/T	Gene_Ca_13250	INTRON	Flavin-containingamineoxidase
Kabuli_wild_324301	1399939559	*Ca_kabuli_Chr06*	324301	C/T	Gene_Ca_13257	NON_SYNONYMOUS_CODING	Zinc finger,FYVE-type
Kabuli_wild_375684	1399939560	*Ca_kabuli_Chr06*	375684	T/G	Gene_Ca_13262	SYNONYMOUS_CODING	Pyridoxalphosphate-dependentdecarboxylase
Kabuli_wild_386250	1399939561	*Ca_kabuli_Chr06*	386250	A/G	NA	INTERGENIC	NA
Kabuli_wild_386289	1399939562	*Ca_kabuli_Chr06*	386289	G/A	NA	INTERGENIC	NA
Kabuli_wild_474489	1399939563	*Ca_kabuli_Chr06*	474489	C/A	NA	INTERGENIC	NA
Kabuli_wild_505875	1399939564	*Ca_kabuli_Chr06*	505875	T/C	Gene_Ca_13271	NON_SYNONYMOUS_CODING	NA
Kabuli_wild_551130	1399939565	*Ca_kabuli_Chr06*	551130	T/G	Gene_Ca_13276	NON_SYNONYMOUS_CODING	Nicastrin
Kabuli_wild_580616	1399939566	*Ca_kabuli_Chr06*	580616	G/C	Gene_Ca_13279	DOWNSTREAM	PeptidaseA1
Kabuli_wild_647322	1399939567	*Ca_kabuli_Chr06*	647322	A/C	Gene_Ca_13283	SYNONYMOUS_CODING	NA
Kabuli_wild_697138	1399939568	*Ca_kabuli_Chr06*	697138	C/T	NA	INTERGENIC	NA
Kabuli_wild_697147	1399939569	*Ca_kabuli_Chr06*	697147	A/G	NA	INTERGENIC	NA
Kabuli_wild_697168	1399939570	*Ca_kabuli_Chr06*	697168	C/T	NA	INTERGENIC	NA
Kabuli_wild_948945	1399939571	*Ca_kabuli_Chr06*	948945	A/G	Gene_Ca_13307	NON_SYNONYMOUS_CODING	Protein kinase,catalytic domain
Kabuli_wild_961805	1399939572	*Ca_kabuli_Chr06*	961805	C/T	NA	INTERGENIC	NA
Kabuli_wild_961875	1399939573	*Ca_kabuli_Chr06*	961875	T/C	NA	INTERGENIC	NA
Kabuli_wild_979827	1399939574	*Ca_kabuli_Chr06*	979827	C/A	NA	INTERGENIC	NA
Kabuli_wild_997997	1399939575	*Ca_kabuli_Chr06*	997997	T/A	NA	INTERGENIC	NA
Kabuli_wild_998014	1399939576	*Ca_kabuli_Chr06*	998014	G/A	NA	INTERGENIC	NA
Kabuli_wild_998031	1399939577	*Ca_kabuli_Chr06*	998031	A/T	NA	INTERGENIC	NA
Kabuli_wild_1034087	1399939578	*Ca_kabuli_Chr06*	1034087	A/T	NA	INTERGENIC	NA
Kabuli_wild_1208372	1399939579	*Ca_kabuli_Chr06*	1208372	A/G	Gene_Ca_13327	INTRON	Short-chaindehydrogenase/reductaseSDR
Kabuli_wild_1220460	1399939580	*Ca_kabuli_Chr06*	1220460	G/C	NA	INTERGENIC	NA
Kabuli_wild_1290413	1399939581	*Ca_kabuli_Chr06*	1290413	C/G	Gene_Ca_13334	INTRON	Regulatorofchromosomecondensation,RCC1
Kabuli_wild_1315583	1399939582	*Ca_kabuli_Chr06*	1315583	G/C	Gene_Ca_13338	INTRON	Aspartate/glutamate/uridylatekinase
Kabuli_wild_1362779	1399939583	*Ca_kabuli_Chr06*	1362779	C/A	NA	INTERGENIC	NA
Kabuli_wild_1400132	1399939584	*Ca_kabuli_Chr06*	1400132	T/A	NA	INTERGENIC	NA
Kabuli_wild_1400135	1399939585	*Ca_kabuli_Chr06*	1400135	C/A	NA	INTERGENIC	NA
Kabuli_wild_1400170	1399939586	*Ca_kabuli_Chr06*	1400170	C/A	NA	INTERGENIC	NA
Kabuli_wild_1400182	1399939587	*Ca_kabuli_Chr06*	1400182	G/T	NA	INTERGENIC	NA
Kabuli_wild_1400202	1399939588	*Ca_kabuli_Chr06*	1400202	C/A	NA	INTERGENIC	NA
Kabuli_wild_1626712	1399939589	*Ca_kabuli_Chr06*	1626712	C/T	NA	INTERGENIC	NA
Kabuli_wild_1626723	1399939590	*Ca_kabuli_Chr06*	1626723	T/C	NA	INTERGENIC	NA
Kabuli_wild_1815245	1399939591	*Ca_kabuli_Chr06*	1815245	T/C	Gene_Ca_10308	DOWNSTREAM	Zinc finger,LIM-type

SNP IDs	NCBI dbSNP accession IDs (NCBI_SS#)	Chromosomes	Physical positions (bp)	SNPs	Gene accession IDs	Sequence components of genome	Putative functions
Kabuli_wild_1815278	1399939592	*Ca_kabuli_Chr06*	1815278	G/A	Gene_Ca_10308	DOWNSTREAM	Zinc finger,LIM-type
Kabuli_wild_1817599	1399939593	*Ca_kabuli_Chr06*	1817599	C/T	Gene_Ca_10308	INTRON	Zinc finger,LIM-type
Kabuli_wild_1817602	1399939594	*Ca_kabuli_Chr06*	1817602	A/T	Gene_Ca_10308	INTRON	Zinc finger,LIM-type
Kabuli_wild_1817607	1399939595	*Ca_kabuli_Chr06*	1817607	T/C	Gene_Ca_10308	INTRON	Zinc finger,LIM-type
Kabuli_wild_1817616	1399939596	*Ca_kabuli_Chr06*	1817616	G/C	Gene_Ca_10308	INTRON	Zinc finger,LIM-type
Kabuli_wild_1818205	1399939597	*Ca_kabuli_Chr06*	1818205	T/G	Gene_Ca_10308	INTRON	Zinc finger,LIM-type
Kabuli_wild_1818225	1399939598	*Ca_kabuli_Chr06*	1818225	A/G	Gene_Ca_10308	INTRON	Zinc finger,LIM-type
Kabuli_wild_1818234	1399939599	*Ca_kabuli_Chr06*	1818234	C/T	Gene_Ca_10308	INTRON	Zinc finger,LIM-type
Kabuli_wild_1878470	1399939600	*Ca_kabuli_Chr06*	1878470	T/C	Gene_Ca_10314	SYNONYMOUS_CODING	Transferase
Kabuli_wild_1878506	1399939601	*Ca_kabuli_Chr06*	1878506	G/A	Gene_Ca_10314	SYNONYMOUS_CODING	Transferase
Kabuli_wild_1932175	1399939602	*Ca_kabuli_Chr06*	1932175	G/A	Gene_Ca_10319	INTRON	Major intrinsic protein
Kabuli_wild_1966137	1399939603	*Ca_kabuli_Chr06*	1966137	T/C	Gene_Ca_10321	DOWNSTREAM	NA
Kabuli_wild_1978864	1399939604	*Ca_kabuli_Chr06*	1978864	A/C	Gene_Ca_10322	INTRON	NA
Kabuli_wild_1978867	1399939605	*Ca_kabuli_Chr06*	1978867	A/T	Gene_Ca_10322	INTRON	NA
Kabuli_wild_2037620	1399939606	*Ca_kabuli_Chr06*	2037620	T/G	NA	INTERGENIC	NA
Kabuli_wild_2045611	1399939607	*Ca_kabuli_Chr06*	2045611	G/C	Gene_Ca_10325	NON_SYNONYMOUS_CODING	Pyridoxalphosphate-dependent enzyme,beta subunit
Kabuli_wild_2049344	1399939608	*Ca_kabuli_Chr06*	2049344	A/G	NA	INTERGENIC	NA
Kabuli_wild_2061459	1399939609	*Ca_kabuli_Chr06*	2061459	A/G	NA	INTERGENIC	NA
Kabuli_wild_2079137	1399939610	*Ca_kabuli_Chr06*	2079137	T/G	NA	INTERGENIC	NA
Kabuli_wild_2079186	1399939611	*Ca_kabuli_Chr06*	2079186	G/T	NA	INTERGENIC	NA
Kabuli_wild_2083805	1399939612	*Ca_kabuli_Chr06*	2083805	G/A	Gene_Ca_10330	SYNONYMOUS_CODING	K+ potassium transporter
Kabuli_wild_2083813	1399939613	*Ca_kabuli_Chr06*	2083813	T/C	Gene_Ca_10330	NON_SYNONYMOUS_CODING	K+ potassium transporter
Kabuli_wild_2126667	1399939614	*Ca_kabuli_Chr06*	2126667	C/T	NA	INTERGENIC	NA
Kabuli_wild_2140189	1399939615	*Ca_kabuli_Chr06*	2140189	G/T	Gene_Ca_10336	NON_SYNONYMOUS_CODING	Leucine-rich repeat
Kabuli_wild_2140406	1399939616	*Ca_kabuli_Chr06*	2140406	G/A	Gene_Ca_10336	SYNONYMOUS_CODING	Leucine-rich repeat
Kabuli_wild_2172005	1399939617	*Ca_kabuli_Chr06*	2172005	C/T	Gene_Ca_10341	NON_SYNONYMOUS_CODING	Sel1-like
Kabuli_wild_2175820	1399939618	*Ca_kabuli_Chr06*	2175820	C/T	NA	INTERGENIC	NA
Kabuli_wild_2176110	1399939619	*Ca_kabuli_Chr06*	2176110	C/T	NA	INTERGENIC	NA
Kabuli_wild_2176221	1399939620	*Ca_kabuli_Chr06*	2176221	C/A	NA	INTERGENIC	NA
Kabuli_wild_2193379	1399939621	*Ca_kabuli_Chr06*	2193379	C/A	NA	INTERGENIC	NA
Kabuli_wild_2193748	1399939622	*Ca_kabuli_Chr06*	2193748	T/A	Gene_Ca_10344	INTRON	Auxin efflux carrier
Kabuli_wild_2193763	1399939623	*Ca_kabuli_Chr06*	2193763	A/C	Gene_Ca_10344	INTRON	Auxin efflux carrier
Kabuli_wild_2193803	1399939624	*Ca_kabuli_Chr06*	2193803	G/A	Gene_Ca_10344	SYNONYMOUS_CODING	Auxin efflux carrier
Kabuli_wild_2214131	1399939625	*Ca_kabuli_Chr06*	2214131	T/A	Gene_Ca_10348	INTRON	Amidase
Kabuli_wild_2214178	1399939626	*Ca_kabuli_Chr06*	2214178	T/C	Gene_Ca_10348	INTRON	Amidase
Kabuli_wild_2214247	1399939627	*Ca_kabuli_Chr06*	2214247	A/G	Gene_Ca_10348	INTRON	Amidase
Kabuli_wild_2223544	1399939628	*Ca_kabuli_Chr06*	2223544	A/T	Gene_Ca_10351	DOWNSTREAM	Pentatrico peptide repeat
Kabuli_wild_2223578	1399939629	*Ca_kabuli_Chr06*	2223578	T/G	Gene_Ca_10351	DOWNSTREAM	Pentatrico peptide repeat
Kabuli_wild_2223664	1399939630	*Ca_kabuli_Chr06*	2223664	A/C	Gene_Ca_10351	DOWNSTREAM	Pentatrico peptide repeat
Kabuli_wild_2233314	1399939631	*Ca_kabuli_Chr06*	2233314	T/G	Gene_Ca_10352	NON_SYNONYMOUS_CODING	Homeobox
Kabuli_wild_2233318	1399939632	*Ca_kabuli_Chr06*	2233318	T/C	Gene_Ca_10352	NON_SYNONYMOUS_CODING	Homeobox
Kabuli_wild_2244901	1399939633	*Ca_kabuli_Chr06*	2244901	A/C	NA	INTERGENIC	NA
Kabuli_wild_2275505	1399939634	*Ca_kabuli_Chr06*	2275505	A/G	Gene_Ca_10356	NON_SYNONYMOUS_CODING	NA
Kabuli_wild_2275524	1399939635	*Ca_kabuli_Chr06*	2275524	G/A	Gene_Ca_10356	SYNONYMOUS_CODING	NA

SNP IDs	NCBI dbSNP accession IDs (NCBI_SS#)	Chromosomes	Physical positions (bp)	SNPs	Gene accession IDs	Sequence components of genome	Putative functions
Kabuli_wild_2275553	1399939636	*Ca_kabuli_Chr06*	2275553	A/G	Gene_Ca_10356	NON_SYNONYMOUS_CODING	NA
Kabuli_wild_2275824	1399939637	*Ca_kabuli_Chr06*	2275824	C/T	Gene_Ca_10356	SYNONYMOUS_CODING	NA
Kabuli_wild_2275825	1399939638	*Ca_kabuli_Chr06*	2275825	T/A	Gene_Ca_10356	NON_SYNONYMOUS_CODING	NA
Kabuli_wild_2280734	1399939639	*Ca_kabuli_Chr06*	2280734	T/A	Gene_Ca_10356	INTRON	NA
Kabuli_wild_2280752	1399939640	*Ca_kabuli_Chr06*	2280752	A/G	Gene_Ca_10356	INTRON	NA
Kabuli_wild_2286237	1399939641	*Ca_kabuli_Chr06*	2286237	C/T	Gene_Ca_10358	DOWNSTREAM	3-oxo-5-alpha-steroid4-dehydrogenase,C-terminal
Kabuli_wild_2294911	1399939642	*Ca_kabuli_Chr06*	2294911	T/G	Gene_Ca_10360	DOWNSTREAM	Protein kinase,catalytic domain
Kabuli_wild_2303123	1399939643	*Ca_kabuli_Chr06*	2303123	T/G	Gene_Ca_10360	INTRON	Protein kinase,catalytic domain
Kabuli_wild_2313683	1399939644	*Ca_kabuli_Chr06*	2313683	A/C	Gene_Ca_10361	NON_SYNONYMOUS_CODING	HistoneH1/H5
Kabuli_wild_2313710	1399939645	*Ca_kabuli_Chr06*	2313710	T/G	Gene_Ca_10361	SYNONYMOUS_CODING	HistoneH1/H5
Kabuli_wild_2313828	1399939646	*Ca_kabuli_Chr06*	2313828	G/A	Gene_Ca_10361	NON_SYNONYMOUS_CODING	HistoneH1/H5
Kabuli_wild_2314029	1399939647	*Ca_kabuli_Chr06*	2314029	T/C	Gene_Ca_10361	NON_SYNONYMOUS_CODING	HistoneH1/H5
Kabuli_wild_2420148	1399939648	*Ca_kabuli_Chr06*	2420148	T/C	NA	INTERGENIC	NA
Kabuli_wild_2462481	1399939649	*Ca_kabuli_Chr06*	2462481	C/T	NA	INTERGENIC	NA
Kabuli_wild_2470723	1399939650	*Ca_kabuli_Chr06*	2470723	C/T	Gene_Ca_10378	NON_SYNONYMOUS_CODING	Double-strandedRNA-binding
Kabuli_wild_2502485	1399939651	*Ca_kabuli_Chr06*	2502485	A/G	NA	INTERGENIC	NA
Kabuli_wild_2527768	1399939652	*Ca_kabuli_Chr06*	2527768	A/G	NA	INTERGENIC	NA
Kabuli_wild_2540711	1399939653	*Ca_kabuli_Chr06*	2540711	C/T	Gene_Ca_10386	NON_SYNONYMOUS_CODING	Phox/Bem1p
Kabuli_wild_2540749	1399939654	*Ca_kabuli_Chr06*	2540749	C/T	Gene_Ca_10386	SYNONYMOUS_CODING	Phox/Bem1p
Kabuli_wild_2541032	1399939655	*Ca_kabuli_Chr06*	2541032	G/A	Gene_Ca_10386	NON_SYNONYMOUS_CODING	Phox/Bem1p
Kabuli_wild_2541088	1399939656	*Ca_kabuli_Chr06*	2541088	T/A	Gene_Ca_10386	SYNONYMOUS_CODING	Phox/Bem1p
Kabuli_wild_2542942	1399939657	*Ca_kabuli_Chr06*	2542942	T/A	NA	INTERGENIC	NA
Kabuli_wild_2542981	1399939658	*Ca_kabuli_Chr06*	2542981	C/T	NA	INTERGENIC	NA
Kabuli_wild_2543062	1399939659	*Ca_kabuli_Chr06*	2543062	A/G	NA	INTERGENIC	NA
Kabuli_wild_2543096	1399939660	*Ca_kabuli_Chr06*	2543096	C/T	NA	INTERGENIC	NA
Kabuli_wild_2548412	1399939661	*Ca_kabuli_Chr06*	2548412	A/G	Gene_Ca_10387	SYNONYMOUS_CODING	Zinc finger, RING-type
Kabuli_wild_2549991	1399939662	*Ca_kabuli_Chr06*	2549991	T/C	Gene_Ca_10387	SYNONYMOUS_CODING	Zinc finger, RING-type
Kabuli_wild_2550154	1399939663	*Ca_kabuli_Chr06*	2550154	A/C	Gene_Ca_10387	SYNONYMOUS_CODING	Zinc finger, RING-type
Kabuli_wild_2625793	1399939664	*Ca_kabuli_Chr06*	2625793	C/G	Gene_Ca_10393	INTRON	THO complex,subunitTHOC2,N-terminal
Kabuli_wild_2639558	1399939665	*Ca_kabuli_Chr06*	2639558	T/A	NA	INTERGENIC	NA
Kabuli_wild_2639606	1399939666	*Ca_kabuli_Chr06*	2639606	A/G	NA	INTERGENIC	NA
Kabuli_wild_2639607	1399939667	*Ca_kabuli_Chr06*	2639607	T/C	NA	INTERGENIC	NA
Kabuli_wild_2639644	1399939668	*Ca_kabuli_Chr06*	2639644	T/C	NA	INTERGENIC	NA
Kabuli_wild_2639716	1399939669	*Ca_kabuli_Chr06*	2639716	T/C	NA	INTERGENIC	NA
Kabuli_wild_2639768	1399939670	*Ca_kabuli_Chr06*	2639768	A/C	NA	INTERGENIC	NA
Kabuli_wild_2639770	1399939671	*Ca_kabuli_Chr06*	2639770	A/C	NA	INTERGENIC	NA
Kabuli_wild_2639800	1399939672	*Ca_kabuli_Chr06*	2639800	A/G	NA	INTERGENIC	NA
Kabuli_wild_2696209	1399939673	*Ca_kabuli_Chr06*	2696209	T/C	NA	INTERGENIC	NA
Kabuli_wild_2696212	1399939674	*Ca_kabuli_Chr06*	2696212	T/C	NA	INTERGENIC	NA
Kabuli_wild_2696213	1399939675	*Ca_kabuli_Chr06*	2696213	T/A	NA	INTERGENIC	NA
Kabuli_wild_2714897	1399939676	*Ca_kabuli_Chr06*	2714897	C/A	NA	INTERGENIC	NA
Kabuli_wild_2717150	1399939677	*Ca_kabuli_Chr06*	2717150	G/A	Gene_Ca_10406	DOWNSTREAM	Heat shock factor(HSF)-type,DNA-binding
Kabuli_wild_2719151	1399939678	*Ca_kabuli_Chr06*	2719151	A/G	NA	INTERGENIC	NA
Kabuli_wild_2740765	1399939679	*Ca_kabuli_Chr06*	2740765	A/G	Gene_Ca_10407	DOWNSTREAM	Glycosyltransferase,family20

SNP IDs	NCBI dbSNP accession IDs (NCBI_SS#)	Chromosomes	Physical positions (bp)	SNPs	Gene accession IDs	Sequence components of genome	Putative functions
Kabuli_wild_2740784	1399939680	*Ca_kabuli_Chr06*	2740784	T/C	Gene_Ca_10407	DOWNSTREAM	Glycosyltransferase,family20
Kabuli_wild_2789573	1399939681	*Ca_kabuli_Chr06*	2789573	A/G	Gene_Ca_10413	INTRON	MyosinII heavychain-like
Kabuli_wild_2802942	1399939682	*Ca_kabuli_Chr06*	2802942	C/A	Gene_Ca_10415	SYNONYMOUS_CODING	NA
Kabuli_wild_2811345	1399939683	*Ca_kabuli_Chr06*	2811345	A/G	Gene_Ca_10416	NON_SYNONYMOUS_CODING	Nucleoporin,Nup155-like
Kabuli_wild_2812710	1399939684	*Ca_kabuli_Chr06*	2812710	T/A	Gene_Ca_10416	SYNONYMOUS_CODING	Nucleoporin,Nup155-like
Kabuli_wild_2812719	1399939685	*Ca_kabuli_Chr06*	2812719	G/A	Gene_Ca_10416	SYNONYMOUS_CODING	Nucleoporin,Nup155-like
Kabuli_wild_2812794	1399939686	*Ca_kabuli_Chr06*	2812794	A/C	Gene_Ca_10416	SYNONYMOUS_CODING	Nucleoporin,Nup155-like
Kabuli_wild_2813118	1399939687	*Ca_kabuli_Chr06*	2813118	G/C	Gene_Ca_10416	SYNONYMOUS_CODING	Nucleoporin,Nup155-like
Kabuli_wild_2813244	1399939688	*Ca_kabuli_Chr06*	2813244	G/A	Gene_Ca_10416	SYNONYMOUS_CODING	Nucleoporin,Nup155-like
Kabuli_wild_2821836	1399939689	*Ca_kabuli_Chr06*	2821836	G/T	Gene_Ca_10417	INTRON	HEAT
Kabuli_wild_2855691	1399939690	*Ca_kabuli_Chr06*	2855691	C/G	Gene_Ca_10421	INTRON	4Fe-4S binding domain
Kabuli_wild_2864713	1399939691	*Ca_kabuli_Chr06*	2864713	G/A	NA	INTERGENIC	NA
Kabuli_wild_2884970	1399939692	*Ca_kabuli_Chr06*	2884970	T/G	Gene_Ca_10426	DOWNSTREAM	XAP5protein
Kabuli_wild_2884974	1399939693	*Ca_kabuli_Chr06*	2884974	T/C	Gene_Ca_10426	DOWNSTREAM	XAP5protein
Kabuli_wild_2919355	1399939694	*Ca_kabuli_Chr06*	2919355	A/C	Gene_Ca_10430	INTRON	UBX
Kabuli_wild_2925908	1399939695	*Ca_kabuli_Chr06*	2925908	A/G	NA	INTERGENIC	NA
Kabuli_wild_2926006	1399939696	*Ca_kabuli_Chr06*	2926006	T/C	NA	INTERGENIC	NA
Kabuli_wild_2927271	1399939697	*Ca_kabuli_Chr06*	2927271	G/A	NA	INTERGENIC	NA
Kabuli_wild_2927386	1399939698	*Ca_kabuli_Chr06*	2927386	C/G	Gene_Ca_10432	UPSTREAM	Glycosidehydrolase,family17
Kabuli_wild_2927387	1399939699	*Ca_kabuli_Chr06*	2927387	T/C	Gene_Ca_10432	UPSTREAM	Glycosidehydrolase,family17
Kabuli_wild_2930459	1399939700	*Ca_kabuli_Chr06*	2930459	C/T	NA	INTERGENIC	NA
Kabuli_wild_2930504	1399939701	*Ca_kabuli_Chr06*	2930504	T/C	NA	INTERGENIC	NA
Kabuli_wild_2932051	1399939702	*Ca_kabuli_Chr06*	2932051	G/A	Gene_Ca_10433	DOWNSTREAM	F-boxdomain,cyclin-like
Kabuli_wild_2932143	1399939703	*Ca_kabuli_Chr06*	2932143	C/A	Gene_Ca_10433	DOWNSTREAM	F-boxdomain,cyclin-like
Kabuli_wild_2932168	1399939704	*Ca_kabuli_Chr06*	2932168	A/C	Gene_Ca_10433	DOWNSTREAM	F-boxdomain,cyclin-like
Kabuli_wild_2932179	1399939705	*Ca_kabuli_Chr06*	2932179	T/G	Gene_Ca_10433	DOWNSTREAM	F-boxdomain,cyclin-like
Kabuli_wild_2972301	1399939706	*Ca_kabuli_Chr06*	2972301	T/C	Gene_Ca_10439	INTRON	Alpha-D-phosphohexomutase
Kabuli_wild_2972319	1399939707	*Ca_kabuli_Chr06*	2972319	C/T	Gene_Ca_10439	INTRON	Alpha-D-phosphohexomutase
Kabuli_wild_2973756	1399939708	*Ca_kabuli_Chr06*	2973756	A/C	Gene_Ca_10439	INTRON	Alpha-D-phosphohexomutase
Kabuli_wild_3060502	1399939709	*Ca_kabuli_Chr06*	3060502	G/A	NA	INTERGENIC	NA
Kabuli_wild_3060508	1399939710	*Ca_kabuli_Chr06*	3060508	G/A	NA	INTERGENIC	NA
Kabuli_wild_3060542	1399939711	*Ca_kabuli_Chr06*	3060542	C/T	NA	INTERGENIC	NA
Kabuli_wild_3060544	1399939712	*Ca_kabuli_Chr06*	3060544	G/A	NA	INTERGENIC	NA
Kabuli_wild_3283199	1399939713	*Ca_kabuli_Chr06*	3283199	T/A	Gene_Ca_25241	INTRON	Aldehydedehydrogenasedomain
Kabuli_wild_3288441	1399939714	*Ca_kabuli_Chr06*	3288441	A/G	NA	INTERGENIC	NA
Kabuli_wild_3288444	1399939715	*Ca_kabuli_Chr06*	3288444	G/A	NA	INTERGENIC	NA
Kabuli_wild_3288481	1399939716	*Ca_kabuli_Chr06*	3288481	C/A	NA	INTERGENIC	NA
Kabuli_wild_3288505	1399939717	*Ca_kabuli_Chr06*	3288505	G/T	NA	INTERGENIC	NA
Kabuli_wild_3297086	1399939718	*Ca_kabuli_Chr06*	3297086	G/A	Gene_Ca_25243	INTRON	Peptidyl-tRNAhydrolase
Kabuli_wild_3298952	1399939719	*Ca_kabuli_Chr06*	3298952	A/T	Gene_Ca_25243	INTRON	Peptidyl-tRNAhydrolase
Kabuli_wild_3395024	1399939720	*Ca_kabuli_Chr06*	3395024	G/A	NA	INTERGENIC	NA
Kabuli_wild_3395075	1399939721	*Ca_kabuli_Chr06*	3395075	T/G	NA	INTERGENIC	NA
Kabuli_wild_3395253	1399939722	*Ca_kabuli_Chr06*	3395253	A/G	NA	INTERGENIC	NA
Kabuli_wild_3395370	1399939723	*Ca_kabuli_Chr06*	3395370	C/T	NA	INTERGENIC	NA

SNP IDs	NCBI dbSNP accession IDs (NCBI_SS#)	Chromosomes	Physical positions (bp)	SNPs	Gene accession IDs	Sequence components of genome	Putative functions
Kabuli_wild_3397650	1399939724	*Ca_kabuli_Chr06*	3397650	A/G	Gene_Ca_05972	DOWNSTREAM	Domain of unknown function DUF292,eukaryotic
Kabuli_wild_3400331	1399939725	*Ca_kabuli_Chr06*	3400331	A/C	Gene_Ca_05972	NON_SYNONYMOUS_CODING	Domain of unknown function DUF292,eukaryotic
Kabuli_wild_3482411	1399939726	*Ca_kabuli_Chr06*	3482411	A/G	NA	INTERGENIC	NA
Kabuli_wild_3505662	1399939727	*Ca_kabuli_Chr06*	3505662	C/A	Gene_Ca_05959	NON_SYNONYMOUS_CODING	Oligo peptide transporter
Kabuli_wild_3530094	1399939728	*Ca_kabuli_Chr06*	3530094	T/C	Gene_Ca_05957	INTRON	FAD-dependent pyridine nucleotide-disulphideoxidoreductase
Kabuli_wild_3530137	1399939729	*Ca_kabuli_Chr06*	3530137	A/G	Gene_Ca_05957	INTRON	FAD-dependent pyridine nucleotide-disulphideoxidoreductase
Kabuli_wild_3548114	1399939730	*Ca_kabuli_Chr06*	3548114	A/C	NA	INTERGENIC	NA
Kabuli_wild_3548121	1399939731	*Ca_kabuli_Chr06*	3548121	C/A	NA	INTERGENIC	NA
Kabuli_wild_3711483	1399939732	*Ca_kabuli_Chr06*	3711483	C/T	NA	INTERGENIC	NA
Kabuli_wild_3711486	1399939733	*Ca_kabuli_Chr06*	3711486	T/C	NA	INTERGENIC	NA
Kabuli_wild_3729810	1399939734	*Ca_kabuli_Chr06*	3729810	T/C	Gene_Ca_05932	INTRON	Malicoxidoreductase
Kabuli_wild_3786843	1399939735	*Ca_kabuli_Chr06*	3786843	A/G	NA	INTERGENIC	NA
Kabuli_wild_3787239	1399939736	*Ca_kabuli_Chr06*	3787239	C/A	NA	INTERGENIC	NA
Kabuli_wild_3829070	1399939737	*Ca_kabuli_Chr06*	3829070	C/A	Gene_Ca_05920	NON_SYNONYMOUS_CODING	Glycosidehydrolase,family17
Kabuli_wild_3962516	1399939738	*Ca_kabuli_Chr06*	3962516	A/C	Gene_Ca_05905	DOWNSTREAM	Snf7
Kabuli_wild_3985519	1399939739	*Ca_kabuli_Chr06*	3985519	G/A	Gene_Ca_05902	UPSTREAM	NA
Kabuli_wild_3992419	1399939740	*Ca_kabuli_Chr06*	3992419	C/T	Gene_Ca_05900	DOWNSTREAM	NA
Kabuli_wild_4112085	1399939741	*Ca_kabuli_Chr06*	4112085	T/C	Gene_Ca_05885	UPSTREAM	PhospholipaseA2,eukaryotic
Kabuli_wild_4226924	1399939742	*Ca_kabuli_Chr06*	4226924	G/T	NA	INTERGENIC	NA
Kabuli_wild_4226947	1399939743	*Ca_kabuli_Chr06*	4226947	A/T	NA	INTERGENIC	NA
Kabuli_wild_4226949	1399939744	*Ca_kabuli_Chr06*	4226949	T/C	NA	INTERGENIC	NA
Kabuli_wild_4226956	1399939745	*Ca_kabuli_Chr06*	4226956	G/A	NA	INTERGENIC	NA
Kabuli_wild_4228814	1399939746	*Ca_kabuli_Chr06*	4228814	A/T	NA	INTERGENIC	NA
Kabuli_wild_4228884	1399939747	*Ca_kabuli_Chr06*	4228884	T/C	NA	INTERGENIC	NA
Kabuli_wild_4229046	1399939748	*Ca_kabuli_Chr06*	4229046	T/A	NA	INTERGENIC	NA
Kabuli_wild_4229069	1399939749	*Ca_kabuli_Chr06*	4229069	C/T	NA	INTERGENIC	NA
Kabuli_wild_4287714	1399939750	*Ca_kabuli_Chr06*	4287714	A/G	NA	INTERGENIC	NA
Kabuli_wild_4288557	1399939751	*Ca_kabuli_Chr06*	4288557	G/C	Gene_Ca_05873	DOWNSTREAM	Protein kinase,catalytic domain
Kabuli_wild_4288560	1399939752	*Ca_kabuli_Chr06*	4288560	C/T	Gene_Ca_05873	DOWNSTREAM	Protein kinase,catalytic domain
Kabuli_wild_4290347	1399939753	*Ca_kabuli_Chr06*	4290347	A/G	Gene_Ca_05873	DOWNSTREAM	Protein kinase,catalytic domain
Kabuli_wild_4344604	1399939754	*Ca_kabuli_Chr06*	4344604	T/C	Gene_Ca_05866	SYNONYMOUS_CODING	Srchomology-3domain
Kabuli_wild_4350822	1399939755	*Ca_kabuli_Chr06*	4350822	A/T	Gene_Ca_05866	NON_SYNONYMOUS_CODING	Srchomology-3domain
Kabuli_wild_4350867	1399939756	*Ca_kabuli_Chr06*	4350867	A/C	Gene_Ca_05866	NON_SYNONYMOUS_CODING	Srchomology-3domain
Kabuli_wild_4350883	1399939757	*Ca_kabuli_Chr06*	4350883	A/C	Gene_Ca_05866	SYNONYMOUS_CODING	Srchomology-3domain
Kabuli_wild_4350889	1399939758	*Ca_kabuli_Chr06*	4350889	A/C	Gene_Ca_05866	SYNONYMOUS_CODING	Srchomology-3domain
Kabuli_wild_4418397	1399939759	*Ca_kabuli_Chr06*	4418397	T/C	NA	INTERGENIC	NA
Kabuli_wild_4418403	1399939760	*Ca_kabuli_Chr06*	4418403	G/T	NA	INTERGENIC	NA
Kabuli_wild_4440034	1399939761	*Ca_kabuli_Chr06*	4440034	T/G	NA	INTERGENIC	NA
Kabuli_wild_4524443	1399939762	*Ca_kabuli_Chr06*	4524443	G/A	Gene_Ca_05849	DOWNSTREAM	RAG1-activatingprotein-1-related
Kabuli_wild_4562263	1399939763	*Ca_kabuli_Chr06*	4562263	C/A	NA	INTERGENIC	NA
Kabuli_wild_4562264	1399939764	*Ca_kabuli_Chr06*	4562264	C/G	NA	INTERGENIC	NA
Kabuli_wild_4593891	1399939765	*Ca_kabuli_Chr06*	4593891	C/A	NA	INTERGENIC	NA
Kabuli_wild_4601303	1399939766	*Ca_kabuli_Chr06*	4601303	C/A	Gene_Ca_05837	NON_SYNONYMOUS_CODING	ATPase,AAA+type,core
Kabuli_wild_4624907	1399939767	*Ca_kabuli_Chr06*	4624907	A/C	NA	INTERGENIC	NA

SNP IDs	NCBI dbSNP accession IDs (NCBI_SS#)	Chromosomes	Physical positions (bp)	SNPs	Gene accession IDs	Sequence components of genome	Putative functions
Kabuli_wild_4637595	1399939768	*Ca_kabuli_Chr06*	4637595	A/G	NA	INTERGENIC	NA
Kabuli_wild_4678523	1399939769	*Ca_kabuli_Chr06*	4678523	A/C	NA	INTERGENIC	NA
Kabuli_wild_4743563	1399939770	*Ca_kabuli_Chr06*	4743563	C/G	Gene_Ca_05822	DOWNSTREAM	AMP-dependentsynthetase/ligase
Kabuli_wild_4743608	1399939771	*Ca_kabuli_Chr06*	4743608	C/T	Gene_Ca_05822	DOWNSTREAM	AMP-dependentsynthetase/ligase
Kabuli_wild_4807908	1399939772	*Ca_kabuli_Chr06*	4807908	G/C	NA	INTERGENIC	NA
Kabuli_wild_4807958	1399939773	*Ca_kabuli_Chr06*	4807958	C/G	NA	INTERGENIC	NA
Kabuli_wild_4861184	1399939774	*Ca_kabuli_Chr06*	4861184	T/G	Gene_Ca_05808	SYNONYMOUS_CODING	NA
Kabuli_wild_4861500	1399939775	*Ca_kabuli_Chr06*	4861500	T/C	Gene_Ca_05808	NON_SYNONYMOUS_CODING	NA
Kabuli_wild_4981078	1399939776	*Ca_kabuli_Chr06*	4981078	G/T	NA	INTERGENIC	NA
Kabuli_wild_4994876	1399939777	*Ca_kabuli_Chr06*	4994876	T/A	Gene_Ca_05794	NON_SYNONYMOUS_CODING	K+ potassium transporter
Kabuli_wild_5185380	1399939778	*Ca_kabuli_Chr06*	5185380	A/G	NA	INTERGENIC	NA
Kabuli_wild_5341441	1399939779	*Ca_kabuli_Chr06*	5341441	C/A	Gene_Ca_05766	NON_SYNONYMOUS_CODING	ARID/BRIGHTDNA-binding domain
Kabuli_wild_5341510	1399939780	*Ca_kabuli_Chr06*	5341510	C/A	Gene_Ca_05766	NON_SYNONYMOUS_CODING	ARID/BRIGHTDNA-binding domain
Kabuli_wild_5366145	1399939781	*Ca_kabuli_Chr06*	5366145	T/A	NA	INTERGENIC	NA
Kabuli_wild_5366191	1399939782	*Ca_kabuli_Chr06*	5366191	T/C	NA	INTERGENIC	NA
Kabuli_wild_5392185	1399939783	*Ca_kabuli_Chr06*	5392185	T/C	Gene_Ca_05761	SYNONYMOUS_CODING	PeptidaseM1,alanineaminopeptidase/leukotrieneA4hydrolase
Kabuli_wild_5396888	1399939784	*Ca_kabuli_Chr06*	5396888	T/G	Gene_Ca_05761	INTRON	PeptidaseM1,alanineaminopeptidase/leukotrieneA4hydrolase
Kabuli_wild_5396890	1399939785	*Ca_kabuli_Chr06*	5396890	T/G	Gene_Ca_05761	INTRON	PeptidaseM1,alanineaminopeptidase/leukotrieneA4hydrolase
Kabuli_wild_5396897	1399939786	*Ca_kabuli_Chr06*	5396897	T/G	Gene_Ca_05761	INTRON	PeptidaseM1,alanineaminopeptidase/leukotrieneA4hydrolase
Kabuli_wild_5396905	1399939787	*Ca_kabuli_Chr06*	5396905	C/T	Gene_Ca_05761	NON_SYNONYMOUS_CODING	PeptidaseM1,alanineaminopeptidase/leukotrieneA4hydrolase
Kabuli_wild_5453512	1399939788	*Ca_kabuli_Chr06*	5453512	A/G	Gene_Ca_05756	NON_SYNONYMOUS_CODING	Protein kinase,catalytic domain
Kabuli_wild_5498898	1399939789	*Ca_kabuli_Chr06*	5498898	C/T	Gene_Ca_05751	SYNONYMOUS_CODING	GMPsynthase,C-terminal
Kabuli_wild_5567368	1399939790	*Ca_kabuli_Chr06*	5567368	A/G	Gene_Ca_05742	DOWNSTREAM	Haloaciddehalogenase-likehydrolase
Kabuli_wild_5589373	1399939791	*Ca_kabuli_Chr06*	5589373	T/C	NA	INTERGENIC	NA
Kabuli_wild_5589991	1399939792	*Ca_kabuli_Chr06*	5589991	T/C	NA	INTERGENIC	NA
Kabuli_wild_5590085	1399939793	*Ca_kabuli_Chr06*	5590085	G/A	NA	INTERGENIC	NA
Kabuli_wild_5616566	1399939794	*Ca_kabuli_Chr06*	5616566	C/A	NA	INTERGENIC	NA
Kabuli_wild_5628956	1399939795	*Ca_kabuli_Chr06*	5628956	T/G	NA	INTERGENIC	NA
Kabuli_wild_5694397	1399939796	*Ca_kabuli_Chr06*	5694397	G/T	Gene_Ca_05725	INTRON	Ribosomal protein S1 ,RNA-binding domain
Kabuli_wild_5704845	1399939797	*Ca_kabuli_Chr06*	5704845	T/C	NA	INTERGENIC	NA
Kabuli_wild_5706649	1399939798	*Ca_kabuli_Chr06*	5706649	C/T	NA	INTERGENIC	NA
Kabuli_wild_6110805	1399939799	*Ca_kabuli_Chr06*	6110805	T/C	Gene_Ca_05684	NON_SYNONYMOUS_CODING	PeptidaseS10,serinecarboxypeptidase
Kabuli_wild_6193105	1399939800	*Ca_kabuli_Chr06*	6193105	G/C	Gene_Ca_05679	STOP_GAINED	NA
Kabuli_wild_6295624	1399939801	*Ca_kabuli_Chr06*	6295624	A/G	Gene_Ca_16900	INTRON	Exostosin-like
Kabuli_wild_6415818	1399939802	*Ca_kabuli_Chr06*	6415818	A/G	NA	INTERGENIC	NA
Kabuli_wild_6517785	1399939803	*Ca_kabuli_Chr06*	6517785	T/C	NA	INTERGENIC	NA
Kabuli_wild_6607761	1399939804	*Ca_kabuli_Chr06*	6607761	G/A	Gene_Ca_16921	SYNONYMOUS_CODING	Membrane attack complex component/perforin (MACPF) domain
Kabuli_wild_6764394	1399939805	*Ca_kabuli_Chr06*	6764394	A/G	Gene_Ca_16937	DOWNSTREAM	Phox/Bem1p
Kabuli_wild_6779147	1399939806	*Ca_kabuli_Chr06*	6779147	G/C	Gene_Ca_16938	NON_SYNONYMOUS_CODING	Protein of unknown function DUF869,plant
Kabuli_wild_6802259	1399939807	*Ca_kabuli_Chr06*	6802259	C/T	Gene_Ca_16940	INTRON	Pyridine nucleotide-disulphideoxido reductase,NAD-binding region
Kabuli_wild_6815594	1399939808	*Ca_kabuli_Chr06*	6815594	G/A	Gene_Ca_16941	DOWNSTREAM	NA
Kabuli_wild_6871350	1399939809	*Ca_kabuli_Chr06*	6871350	A/T	Gene_Ca_16949	SYNONYMOUS_CODING	Aminoacid transporter,transmembrane
Kabuli_wild_6877088	1399939810	*Ca_kabuli_Chr06*	6877088	C/A	Gene_Ca_16950	NON_SYNONYMOUS_CODING	Heat shock protein DnaJ,N-terminal
Kabuli_wild_6877163	1399939811	*Ca_kabuli_Chr06*	6877163	T/C	Gene_Ca_16950	NON_SYNONYMOUS_CODING	Heat shock protein DnaJ,N-terminal

SNP IDs	NCBI dbSNP accession IDs (NCBI_SS#)	Chromosomes	Physical positions (bp)	SNPs	Gene accession IDs	Sequence components of genome	Putative functions
Kabuli_wild_6877284	1399939812	*Ca_kabuli_Chr06*	6877284	C/T	Gene_Ca_16950	SYNONYMOUS_CODING	Heat shock protein DnaJ,N-terminal
Kabuli_wild_6913622	1399939813	*Ca_kabuli_Chr06*	6913622	A/C	NA	INTERGENIC	NA
Kabuli_wild_6913695	1399939814	*Ca_kabuli_Chr06*	6913695	G/A	NA	INTERGENIC	NA
Kabuli_wild_6913844	1399939815	*Ca_kabuli_Chr06*	6913844	C/T	NA	INTERGENIC	NA
Kabuli_wild_6913879	1399939816	*Ca_kabuli_Chr06*	6913879	C/T	NA	INTERGENIC	NA
Kabuli_wild_6939908	1399939817	*Ca_kabuli_Chr06*	6939908	A/G	Gene_Ca_09689	NON_SYNONYMOUS_CODING	Oxoglutarate/iron-dependent oxygenase
Kabuli_wild_7026481	1399939818	*Ca_kabuli_Chr06*	7026481	G/C	Gene_Ca_09682	DOWNSTREAM	Cation/H+exchanger
Kabuli_wild_7040216	1399939819	*Ca_kabuli_Chr06*	7040216	C/T	NA	INTERGENIC	NA
Kabuli_wild_7072268	1399939820	*Ca_kabuli_Chr06*	7072268	T/C	Gene_Ca_09675	INTRON	Glycosyltransferase,family48
Kabuli_wild_7349517	1399939821	*Ca_kabuli_Chr06*	7349517	A/C	Gene_Ca_09645	DOWNSTREAM	NA
Kabuli_wild_7398761	1399939822	*Ca_kabuli_Chr06*	7398761	C/G	Gene_Ca_09641	NON_SYNONYMOUS_CODING	Zinc finger, RING-type
Kabuli_wild_7406219	1399939823	*Ca_kabuli_Chr06*	7406219	A/G	NA	INTERGENIC	NA
Kabuli_wild_7449180	1399939824	*Ca_kabuli_Chr06*	7449180	T/G	Gene_Ca_09636	INTRON	PeptidaseC19,ubiquitincarboxyl-terminalhydrolase2
Kabuli_wild_7566842	1399939825	*Ca_kabuli_Chr06*	7566842	G/A	Gene_Ca_09627	SYNONYMOUS_CODING	NA
Kabuli_wild_7571950	1399939826	*Ca_kabuli_Chr06*	7571950	T/C	Gene_Ca_09627	NON_SYNONYMOUS_CODING	NA
Kabuli_wild_7575102	1399939827	*Ca_kabuli_Chr06*	7575102	A/T	Gene_Ca_09627	NON_SYNONYMOUS_CODING	NA
Kabuli_wild_7581799	1399939828	*Ca_kabuli_Chr06*	7581799	T/C	NA	INTERGENIC	NA
Kabuli_wild_7581956	1399939829	*Ca_kabuli_Chr06*	7581956	C/G	NA	INTERGENIC	NA
Kabuli_wild_7582726	1399939830	*Ca_kabuli_Chr06*	7582726	G/T	NA	INTERGENIC	NA
Kabuli_wild_7600817	1399939831	*Ca_kabuli_Chr06*	7600817	G/A	Gene_Ca_09624	INTRON	Domain of unknown function DUF699,exodeoxyribonucleaseValphachain
Kabuli_wild_7603134	1399939832	*Ca_kabuli_Chr06*	7603134	C/T	Gene_Ca_09624	INTRON	Domain of unknown function DUF699,exodeoxyribonuclease V alpha chain
Kabuli_wild_7603178	1399939833	*Ca_kabuli_Chr06*	7603178	A/C	Gene_Ca_09624	INTRON	Domain of unknown function DUF699,exodeoxyribonucleaseValphachain
Kabuli_wild_7603195	1399939834	*Ca_kabuli_Chr06*	7603195	G/A	Gene_Ca_09624	NON_SYNONYMOUS_CODING	Domain of unknown function DUF699,exodeoxyribonucleaseValphachain
Kabuli_wild_7609380	1399939835	*Ca_kabuli_Chr06*	7609380	G/A	Gene_Ca_09623	DOWNSTREAM	NA
Kabuli_wild_7609453	1399939836	*Ca_kabuli_Chr06*	7609453	T/G	Gene_Ca_09623	DOWNSTREAM	NA
Kabuli_wild_7609573	1399939837	*Ca_kabuli_Chr06*	7609573	G/A	Gene_Ca_09623	DOWNSTREAM	NA
Kabuli_wild_7618391	1399939838	*Ca_kabuli_Chr06*	7618391	C/G	NA	INTERGENIC	NA
Kabuli_wild_7647907	1399939839	*Ca_kabuli_Chr06*	7647907	T/G	Gene_Ca_09618	SYNONYMOUS_CODING	RasGTPase
Kabuli_wild_7647931	1399939840	*Ca_kabuli_Chr06*	7647931	G/A	Gene_Ca_09618	SYNONYMOUS_CODING	RasGTPase
Kabuli_wild_7661924	1399939841	*Ca_kabuli_Chr06*	7661924	T/C	NA	INTERGENIC	NA
Kabuli_wild_7661930	1399939842	*Ca_kabuli_Chr06*	7661930	T/G	NA	INTERGENIC	NA
Kabuli_wild_7667301	1399939843	*Ca_kabuli_Chr06*	7667301	T/C	NA	INTERGENIC	NA
Kabuli_wild_7667328	1399939844	*Ca_kabuli_Chr06*	7667328	A/T	NA	INTERGENIC	NA
Kabuli_wild_7694609	1399939845	*Ca_kabuli_Chr06*	7694609	A/T	NA	INTERGENIC	NA
Kabuli_wild_7694696	1399939846	*Ca_kabuli_Chr06*	7694696	T/C	NA	INTERGENIC	NA
Kabuli_wild_7704483	1399939847	*Ca_kabuli_Chr06*	7704483	G/A	NA	INTERGENIC	NA
Kabuli_wild_7705311	1399939848	*Ca_kabuli_Chr06*	7705311	G/A	Gene_Ca_09609	DOWNSTREAM	NA
Kabuli_wild_7719800	1399939849	*Ca_kabuli_Chr06*	7719800	C/A	NA	INTERGENIC	NA
Kabuli_wild_7719829	1399939850	*Ca_kabuli_Chr06*	7719829	C/T	NA	INTERGENIC	NA
Kabuli_wild_7719857	1399939851	*Ca_kabuli_Chr06*	7719857	G/T	NA	INTERGENIC	NA
Kabuli_wild_7719877	1399939852	*Ca_kabuli_Chr06*	7719877	T/C	NA	INTERGENIC	NA
Kabuli_wild_7744730	1399939853	*Ca_kabuli_Chr06*	7744730	A/G	Gene_Ca_09604	INTRON	ABC-1
Kabuli_wild_7745641	1399939854	*Ca_kabuli_Chr06*	7745641	A/G	Gene_Ca_09604	INTRON	ABC-1
Kabuli_wild_7747254	1399939855	*Ca_kabuli_Chr06*	7747254	G/A	Gene_Ca_09604	SYNONYMOUS_CODING	ABC-1

SNP IDs	NCBI dbSNP accession IDs (NCBI_SS#)	Chromosomes	Physical positions (bp)	SNPs	Gene accession IDs	Sequence components of genome	Putative functions
Kabuli_wild_7770357	1399939856	*Ca_kabuli_Chr06*	7770357	C/T	NA	INTERGENIC	NA
Kabuli_wild_7822666	1399939857	*Ca_kabuli_Chr06*	7822666	C/A	NA	INTERGENIC	NA
Kabuli_wild_7829485	1399939858	*Ca_kabuli_Chr06*	7829485	G/A	NA	INTERGENIC	NA
Kabuli_wild_7829627	1399939859	*Ca_kabuli_Chr06*	7829627	T/G	NA	INTERGENIC	NA
Kabuli_wild_7831214	1399939860	*Ca_kabuli_Chr06*	7831214	C/T	Gene_Ca_09595	SYNONYMOUS_CODING	C2 calcium-dependent membrane targeting
Kabuli_wild_7831409	1399939861	*Ca_kabuli_Chr06*	7831409	C/T	Gene_Ca_09595	SYNONYMOUS_CODING	C2 calcium-dependent membrane targeting
Kabuli_wild_7831874	1399939862	*Ca_kabuli_Chr06*	7831874	A/G	Gene_Ca_09595	SYNONYMOUS_CODING	C2 calcium-dependent membrane targeting
Kabuli_wild_7831899	1399939863	*Ca_kabuli_Chr06*	7831899	G/A	Gene_Ca_09595	NON_SYNONYMOUS_CODING	C2 calcium-dependent membrane targeting
Kabuli_wild_7832023	1399939864	*Ca_kabuli_Chr06*	7832023	C/T	Gene_Ca_09595	NON_SYNONYMOUS_CODING	C2 calcium-dependent membrane targeting
Kabuli_wild_7832029	1399939865	*Ca_kabuli_Chr06*	7832029	C/T	Gene_Ca_09595	NON_SYNONYMOUS_CODING	C2 calcium-dependent membrane targeting
Kabuli_wild_7834595	1399939866	*Ca_kabuli_Chr06*	7834595	C/T	Gene_Ca_09595	SYNONYMOUS_CODING	C2 calcium-dependent membrane targeting
Kabuli_wild_7834895	1399939867	*Ca_kabuli_Chr06*	7834895	G/T	Gene_Ca_09595	SYNONYMOUS_CODING	C2 calcium-dependent membrane targeting
Kabuli_wild_7834922	1399939868	*Ca_kabuli_Chr06*	7834922	G/A	Gene_Ca_09595	SYNONYMOUS_CODING	C2 calcium-dependent membrane targeting
Kabuli_wild_7834976	1399939869	*Ca_kabuli_Chr06*	7834976	G/A	Gene_Ca_09595	SYNONYMOUS_CODING	C2 calcium-dependent membrane targeting
Kabuli_wild_7835024	1399939870	*Ca_kabuli_Chr06*	7835024	T/A	Gene_Ca_09595	SYNONYMOUS_CODING	C2 calcium-dependent membrane targeting
Kabuli_wild_7835427	1399939871	*Ca_kabuli_Chr06*	7835427	G/A	Gene_Ca_09595	NON_SYNONYMOUS_CODING	C2 calcium-dependent membrane targeting
Kabuli_wild_7835510	1399939872	*Ca_kabuli_Chr06*	7835510	A/G	Gene_Ca_09595	SYNONYMOUS_CODING	C2 calcium-dependent membrane targeting
Kabuli_wild_7835738	1399939873	*Ca_kabuli_Chr06*	7835738	C/T	Gene_Ca_09595	SYNONYMOUS_CODING	C2 calcium-dependent membrane targeting
Kabuli_wild_7835984	1399939874	*Ca_kabuli_Chr06*	7835984	T/A	Gene_Ca_09595	NON_SYNONYMOUS_CODING	C2 calcium-dependent membrane targeting
Kabuli_wild_7851000	1399939875	*Ca_kabuli_Chr06*	7851000	G/C	Gene_Ca_09593	UPSTREAM	Peptidyl-prolylcis-transisomerase,FKBP-type,domain
Kabuli_wild_7852466	1399939876	*Ca_kabuli_Chr06*	7852466	T/C	NA	INTERGENIC	NA
Kabuli_wild_7852481	1399939877	*Ca_kabuli_Chr06*	7852481	G/A	NA	INTERGENIC	NA
Kabuli_wild_7876049	1399939878	*Ca_kabuli_Chr06*	7876049	A/C	Gene_Ca_09590	DOWNSTREAM	1-aminocyclopropane-1-carboxylatesynthase
Kabuli_wild_7926416	1399939879	*Ca_kabuli_Chr06*	7926416	C/A	Gene_Ca_09581	DOWNSTREAM	Acyl-CoAoxidase,C-terminal
Kabuli_wild_7929263	1399939880	*Ca_kabuli_Chr06*	7929263	C/T	NA	INTERGENIC	NA
Kabuli_wild_7929338	1399939881	*Ca_kabuli_Chr06*	7929338	T/C	NA	INTERGENIC	NA
Kabuli_wild_7929339	1399939882	*Ca_kabuli_Chr06*	7929339	G/A	NA	INTERGENIC	NA
Kabuli_wild_7929348	1399939883	*Ca_kabuli_Chr06*	7929348	C/T	NA	INTERGENIC	NA
Kabuli_wild_7929384	1399939884	*Ca_kabuli_Chr06*	7929384	T/C	NA	INTERGENIC	NA
Kabuli_wild_7929607	1399939885	*Ca_kabuli_Chr06*	7929607	A/C	NA	INTERGENIC	NA
Kabuli_wild_7929628	1399939886	*Ca_kabuli_Chr06*	7929628	C/T	NA	INTERGENIC	NA
Kabuli_wild_7929647	1399939887	*Ca_kabuli_Chr06*	7929647	A/C	NA	INTERGENIC	NA
Kabuli_wild_7939281	1399939888	*Ca_kabuli_Chr06*	7939281	T/G	Gene_Ca_09580	NON_SYNONYMOUS_CODING	NA
Kabuli_wild_7939345	1399939889	*Ca_kabuli_Chr06*	7939345	T/G	Gene_Ca_09580	NON_SYNONYMOUS_CODING	NA
Kabuli_wild_7939465	1399939890	*Ca_kabuli_Chr06*	7939465	A/G	Gene_Ca_09580	INTRON	NA
Kabuli_wild_7994732	1399939891	*Ca_kabuli_Chr06*	7994732	G/T	NA	INTERGENIC	NA
Kabuli_wild_8006728	1399939892	*Ca_kabuli_Chr06*	8006728	C/A	Gene_Ca_09573	UPSTREAM	Major facilitator superfamily MFS-1
Kabuli_wild_8006729	1399939893	*Ca_kabuli_Chr06*	8006729	G/T	Gene_Ca_09573	UPSTREAM	Major facilitator superfamily MFS-1
Kabuli_wild_8011685	1399939894	*Ca_kabuli_Chr06*	8011685	G/T	NA	INTERGENIC	NA
Kabuli_wild_8029226	1399939895	*Ca_kabuli_Chr06*	8029226	C/T	Gene_Ca_09571	SYNONYMOUS_CODING	NA
Kabuli_wild_8029310	1399939896	*Ca_kabuli_Chr06*	8029310	G/C	Gene_Ca_09571	SYNONYMOUS_CODING	NA
Kabuli_wild_8029340	1399939897	*Ca_kabuli_Chr06*	8029340	G/A	Gene_Ca_09571	SYNONYMOUS_CODING	NA
Kabuli_wild_8052987	1399939898	*Ca_kabuli_Chr06*	8052987	G/C	Gene_Ca_09568	INTRON	TLDc
Kabuli_wild_8070427	1399939899	*Ca_kabuli_Chr06*	8070427	T/C	NA	INTERGENIC	NA

SNP IDs	NCBI dbSNP accession IDs (NCBI_SS#)	Chromosomes	Physical positions (bp)	SNPs	Gene accession IDs	Sequence components of genome	Putative functions
Kabuli_wild_8076394	1399939900	*Ca_kabuli_Chr06*	8076394	T/C	NA	INTERGENIC	NA
Kabuli_wild_8076446	1399939901	*Ca_kabuli_Chr06*	8076446	G/A	NA	INTERGENIC	NA
Kabuli_wild_8081800	1399939902	*Ca_kabuli_Chr06*	8081800	C/G	NA	INTERGENIC	NA
Kabuli_wild_8084950	1399939903	*Ca_kabuli_Chr06*	8084950	T/A	Gene_Ca_09563	DOWNSTREAM	Mitochondrial carrier protein
Kabuli_wild_8090739	1399939904	*Ca_kabuli_Chr06*	8090739	T/A	Gene_Ca_09562	NON_SYNONYMOUS_CODING	IQ motif,EF-handbinding site
Kabuli_wild_8090918	1399939905	*Ca_kabuli_Chr06*	8090918	A/C	Gene_Ca_09562	NON_SYNONYMOUS_CODING	IQ motif,EF-handbinding site
Kabuli_wild_8090980	1399939906	*Ca_kabuli_Chr06*	8090980	A/G	Gene_Ca_09562	SYNONYMOUS_CODING	IQ motif,EF-handbinding site
Kabuli_wild_8091050	1399939907	*Ca_kabuli_Chr06*	8091050	A/G	Gene_Ca_09562	NON_SYNONYMOUS_CODING	IQ motif,EF-handbinding site
Kabuli_wild_8094879	1399939908	*Ca_kabuli_Chr06*	8094879	T/A	Gene_Ca_09562	DOWNSTREAM	IQ motif,EF-handbinding site
Kabuli_wild_8095458	1399939909	*Ca_kabuli_Chr06*	8095458	C/T	NA	INTERGENIC	NA
Kabuli_wild_8134381	1399939910	*Ca_kabuli_Chr06*	8134381	T/C	Gene_Ca_09555	DOWNSTREAM	ATPase, P-type, H+ transporting proton pump
Kabuli_wild_8139446	1399939911	*Ca_kabuli_Chr06*	8139446	C/A	NA	INTERGENIC	NA
Kabuli_wild_8166317	1399939912	*Ca_kabuli_Chr06*	8166317	T/C	NA	INTERGENIC	NA
Kabuli_wild_8166339	1399939913	*Ca_kabuli_Chr06*	8166339	T/C	NA	INTERGENIC	NA
Kabuli_wild_8166435	1399939914	*Ca_kabuli_Chr06*	8166435	A/G	NA	INTERGENIC	NA
Kabuli_wild_8166462	1399939915	*Ca_kabuli_Chr06*	8166462	A/T	NA	INTERGENIC	NA
Kabuli_wild_8170583	1399939916	*Ca_kabuli_Chr06*	8170583	C/T	Gene_Ca_09552	INTRON	Enhancerofpolycomb-like,N-terminal
Kabuli_wild_8170633	1399939917	*Ca_kabuli_Chr06*	8170633	A/G	Gene_Ca_09552	INTRON	Enhancerofpolycomb-like,N-terminal
Kabuli_wild_8179815	1399939918	*Ca_kabuli_Chr06*	8179815	T/C	NA	INTERGENIC	NA
Kabuli_wild_8221204	1399939919	*Ca_kabuli_Chr06*	8221204	C/A	Gene_Ca_09549	NON_SYNONYMOUS_CODING	DNA-dependentATPaseMCM
Kabuli_wild_8221205	1399939920	*Ca_kabuli_Chr06*	8221205	G/T	Gene_Ca_09549	NON_SYNONYMOUS_CODING	DNA-dependentATPaseMCM
Kabuli_wild_8223986	1399939921	*Ca_kabuli_Chr06*	8223986	C/G	Gene_Ca_09549	NON_SYNONYMOUS_CODING	DNA-dependentATPaseMCM
Kabuli_wild_8237286	1399939922	*Ca_kabuli_Chr06*	8237286	A/G	Gene_Ca_09547	INTRON	RNA recognition motif domain
Kabuli_wild_8246187	1399939923	*Ca_kabuli_Chr06*	8246187	A/C	Gene_Ca_09546	DOWNSTREAM	NA
Kabuli_wild_8655336	1399939924	*Ca_kabuli_Chr06*	8655336	T/G	NA	INTERGENIC	NA
Kabuli_wild_8655742	1399939925	*Ca_kabuli_Chr06*	8655742	A/C	NA	INTERGENIC	NA
Kabuli_wild_8710064	1399939926	*Ca_kabuli_Chr06*	8710064	A/T	Gene_Ca_16763	UPSTREAM	Actin-like
Kabuli_wild_8711737	1399939927	*Ca_kabuli_Chr06*	8711737	A/T	Gene_Ca_16763	SYNONYMOUS_CODING	Actin-like
Kabuli_wild_8787557	1399939928	*Ca_kabuli_Chr06*	8787557	T/C	Gene_Ca_16756	NON_SYNONYMOUS_CODING	Protein kinase,catalytic domain
Kabuli_wild_8787562	1399939929	*Ca_kabuli_Chr06*	8787562	C/T	Gene_Ca_16756	NON_SYNONYMOUS_CODING	Protein kinase,catalytic domain
Kabuli_wild_8787564	1399939930	*Ca_kabuli_Chr06*	8787564	G/T	Gene_Ca_16756	SYNONYMOUS_CODING	Protein kinase,catalytic domain
Kabuli_wild_8787568	1399939931	*Ca_kabuli_Chr06*	8787568	G/T	Gene_Ca_16756	STOP_GAINED	Protein kinase,catalytic domain
Kabuli_wild_8787572	1399939932	*Ca_kabuli_Chr06*	8787572	T/A	Gene_Ca_16756	NON_SYNONYMOUS_CODING	Protein kinase,catalytic domain
Kabuli_wild_8787573	1399939933	*Ca_kabuli_Chr06*	8787573	G/A	Gene_Ca_16756	SYNONYMOUS_CODING	Protein kinase,catalytic domain
Kabuli_wild_8968068	1399939934	*Ca_kabuli_Chr06*	8968068	G/A	Gene_Ca_16741	INTRON	Phosphatidylinositol-4-phosphate5-kinase,core
Kabuli_wild_9227767	1399939935	*Ca_kabuli_Chr06*	9227767	C/A	NA	INTERGENIC	NA
Kabuli_wild_9228004	1399939936	*Ca_kabuli_Chr06*	9228004	C/A	NA	INTERGENIC	NA
Kabuli_wild_9229496	1399939937	*Ca_kabuli_Chr06*	9229496	T/G	NA	INTERGENIC	NA
Kabuli_wild_9324122	1399939938	*Ca_kabuli_Chr06*	9324122	A/C	Gene_Ca_08667	NON_SYNONYMOUS_CODING	Homeobox
Kabuli_wild_9523228	1399939939	*Ca_kabuli_Chr06*	9523228	T/C	Gene_Ca_08648	INTRON	RNA-processing protein, HATthelix
Kabuli_wild_9542944	1399939940	*Ca_kabuli_Chr06*	9542944	A/G	NA	INTERGENIC	NA
Kabuli_wild_9814378	1399939941	*Ca_kabuli_Chr06*	9814378	G/T	Gene_Ca_08611	INTRON	ProteinTransporter,Pam16
Kabuli_wild_9833725	1399939942	*Ca_kabuli_Chr06*	9833725	C/T	Gene_Ca_08608	INTRON	WD40 repeat
Kabuli_wild_9833832	1399939943	*Ca_kabuli_Chr06*	9833832	T/A	Gene_Ca_08608	INTRON	WD40 repeat

SNP IDs	NCBI dbSNP accession IDs (NCBI_SS#)	Chromosomes	Physical positions (bp)	SNPs	Gene accession IDs	Sequence components of genome	Putative functions
Kabuli_wild_9834183	1399939944	*Ca_kabuli_Chr06*	9834183	C/T	Gene_Ca_08608	NON_SYNONYMOUS_CODING	WD40 repeat
Kabuli_wild_9834184	1399939945	*Ca_kabuli_Chr06*	9834184	A/T	Gene_Ca_08608	SYNONYMOUS_CODING	WD40 repeat
Kabuli_wild_9834187	1399939946	*Ca_kabuli_Chr06*	9834187	A/T	Gene_Ca_08608	NON_SYNONYMOUS_CODING	WD40 repeat
Kabuli_wild_9834198	1399939947	*Ca_kabuli_Chr06*	9834198	C/A	Gene_Ca_08608	NON_SYNONYMOUS_CODING	WD40 repeat
Kabuli_wild_9834199	1399939948	*Ca_kabuli_Chr06*	9834199	T/C	Gene_Ca_08608	SYNONYMOUS_CODING	WD40 repeat
Kabuli_wild_9841836	1399939949	*Ca_kabuli_Chr06*	9841836	G/A	NA	INTERGENIC	NA
Kabuli_wild_9872634	1399939950	*Ca_kabuli_Chr06*	9872634	A/G	Gene_Ca_08602	SYNONYMOUS_CODING	Xanthine/uracil/vitamin C permease
Kabuli_wild_9872657	1399939951	*Ca_kabuli_Chr06*	9872657	C/T	Gene_Ca_08602	NON_SYNONYMOUS_CODING	Xanthine/uracil/vitamin C permease
Kabuli_wild_9872988	1399939952	*Ca_kabuli_Chr06*	9872988	A/T	Gene_Ca_08602	INTRON	Xanthine/uracil/vitamin C permease
Kabuli_wild_9959686	1399939953	*Ca_kabuli_Chr06*	9959686	T/C	Gene_Ca_08594	SYNONYMOUS_CODING	PreproteintranslocaseSec,Sec61-betasubunit
Kabuli_wild_10013651	1399939954	*Ca_kabuli_Chr06*	10013651	C/T	NA	INTERGENIC	NA
Kabuli_wild_10020177	1399939955	*Ca_kabuli_Chr06*	10020177	C/G	NA	INTERGENIC	NA
Kabuli_wild_10020187	1399939956	*Ca_kabuli_Chr06*	10020187	A/G	NA	INTERGENIC	NA
Kabuli_wild_10020438	1399939957	*Ca_kabuli_Chr06*	10020438	G/C	NA	INTERGENIC	NA
Kabuli_wild_10020464	1399939958	*Ca_kabuli_Chr06*	10020464	C/G	NA	INTERGENIC	NA
Kabuli_wild_10020475	1399939959	*Ca_kabuli_Chr06*	10020475	A/T	NA	INTERGENIC	NA
Kabuli_wild_10020491	1399939960	*Ca_kabuli_Chr06*	10020491	A/G	NA	INTERGENIC	NA
Kabuli_wild_10025113	1399939961	*Ca_kabuli_Chr06*	10025113	C/G	NA	INTERGENIC	NA
Kabuli_wild_10025115	1399939962	*Ca_kabuli_Chr06*	10025115	A/C	NA	INTERGENIC	NA
Kabuli_wild_10028504	1399939963	*Ca_kabuli_Chr06*	10028504	A/G	Gene_Ca_08589	DOWNSTREAM	Mitochondrialsubstrate/solutecarrier
Kabuli_wild_10028508	1399939964	*Ca_kabuli_Chr06*	10028508	G/C	Gene_Ca_08589	DOWNSTREAM	Mitochondrialsubstrate/solutecarrier
Kabuli_wild_10045644	1399939965	*Ca_kabuli_Chr06*	10045644	A/C	Gene_Ca_08587	SYNONYMOUS_CODING	AMP-dependentsynthetase/ligase
Kabuli_wild_10045844	1399939966	*Ca_kabuli_Chr06*	10045844	A/G	Gene_Ca_08587	UPSTREAM	AMP-dependentsynthetase/ligase
Kabuli_wild_10088984	1399939967	*Ca_kabuli_Chr06*	10088984	A/G	NA	INTERGENIC	NA
Kabuli_wild_10100863	1399939968	*Ca_kabuli_Chr06*	10100863	C/G	Gene_Ca_08581	SYNONYMOUS_CODING	HEAT
Kabuli_wild_10111488	1399939969	*Ca_kabuli_Chr06*	10111488	C/G	Gene_Ca_08580	INTRON	Peptidyl-tRNAhydrolase
Kabuli_wild_10111520	1399939970	*Ca_kabuli_Chr06*	10111520	G/C	Gene_Ca_08580	INTRON	Peptidyl-tRNAhydrolase
Kabuli_wild_10111870	1399939971	*Ca_kabuli_Chr06*	10111870	A/G	Gene_Ca_08580	INTRON	Peptidyl-tRNAhydrolase
Kabuli_wild_10111927	1399939972	*Ca_kabuli_Chr06*	10111927	G/T	Gene_Ca_08580	INTRON	Peptidyl-tRNAhydrolase
Kabuli_wild_10116403	1399939973	*Ca_kabuli_Chr06*	10116403	T/A	Gene_Ca_08580	INTRON	Peptidyl-tRNAhydrolase
Kabuli_wild_10116475	1399939974	*Ca_kabuli_Chr06*	10116475	A/C	Gene_Ca_08580	INTRON	Peptidyl-tRNAhydrolase
Kabuli_wild_10116608	1399939975	*Ca_kabuli_Chr06*	10116608	G/T	Gene_Ca_08580	INTRON	Peptidyl-tRNAhydrolase
Kabuli_wild_10116647	1399939976	*Ca_kabuli_Chr06*	10116647	C/T	Gene_Ca_08580	INTRON	Peptidyl-tRNAhydrolase
Kabuli_wild_10119556	1399939977	*Ca_kabuli_Chr06*	10119556	G/C	Gene_Ca_08580	SYNONYMOUS_CODING	Peptidyl-tRNAhydrolase
Kabuli_wild_10134177	1399939978	*Ca_kabuli_Chr06*	10134177	A/G	NA	INTERGENIC	NA
Kabuli_wild_10137249	1399939979	*Ca_kabuli_Chr06*	10137249	A/G	Gene_Ca_08579	INTRON	NA
Kabuli_wild_10137277	1399939980	*Ca_kabuli_Chr06*	10137277	T/G	Gene_Ca_08579	INTRON	NA
Kabuli_wild_10151973	1399939981	*Ca_kabuli_Chr06*	10151973	A/G	Gene_Ca_08577	INTRON	NA
Kabuli_wild_10158620	1399939982	*Ca_kabuli_Chr06*	10158620	C/A	Gene_Ca_08576	NON_SYNONYMOUS_CODING	Pheophorbideaoxygenase
Kabuli_wild_10158850	1399939983	*Ca_kabuli_Chr06*	10158850	T/C	Gene_Ca_08576	INTRON	Protein of unknown function DUF616
Kabuli_wild_10188400	1399939984	*Ca_kabuli_Chr06*	10188400	G/T	Gene_Ca_08570	UPSTREAM	AMP-dependentsynthetase/ligase
Kabuli_wild_10192015	1399939985	*Ca_kabuli_Chr06*	10192015	A/G	NA	INTERGENIC	NA
Kabuli_wild_10192017	1399939986	*Ca_kabuli_Chr06*	10192017	C/T	NA	INTERGENIC	NA
Kabuli_wild_10192247	1399939987	*Ca_kabuli_Chr06*	10192247	A/G	NA	INTERGENIC	NA

SNP IDs	NCBI dbSNP accession IDs (NCBI_SS#)	Chromosomes	Physical positions (bp)	SNPs	Gene accession IDs	Sequence components of genome	Putative functions
Kabuli_wild_10192296	1399939988	*Ca_kabuli_Chr06*	10192296	G/T	NA	INTERGENIC	NA
Kabuli_wild_10215262	1399939989	*Ca_kabuli_Chr06*	10215262	T/G	NA	INTERGENIC	NA
Kabuli_wild_10230617	1399939990	*Ca_kabuli_Chr06*	10230617	G/A	Gene_Ca_08566	INTRON	Metallo-dependentphosphatase
Kabuli_wild_10230657	1399939991	*Ca_kabuli_Chr06*	10230657	G/A	Gene_Ca_08566	INTRON	Metallo-dependentphosphatase
Kabuli_wild_10231092	1399939992	*Ca_kabuli_Chr06*	10231092	T/G	Gene_Ca_08566	SYNONYMOUS_CODING	Metallo-dependentphosphatase
Kabuli_wild_10231095	1399939993	*Ca_kabuli_Chr06*	10231095	G/T	Gene_Ca_08566	SYNONYMOUS_CODING	Metallo-dependentphosphatase
Kabuli_wild_10231188	1399939994	*Ca_kabuli_Chr06*	10231188	T/G	Gene_Ca_08566	SYNONYMOUS_CODING	Metallo-dependentphosphatase
Kabuli_wild_10231199	1399939995	*Ca_kabuli_Chr06*	10231199	A/G	Gene_Ca_08566	INTRON	Metallo-dependentphosphatase
Kabuli_wild_10234241	1399939996	*Ca_kabuli_Chr06*	10234241	A/G	Gene_Ca_08566	INTRON	Metallo-dependentphosphatase
Kabuli_wild_10259614	1399939997	*Ca_kabuli_Chr06*	10259614	C/T	NA	INTERGENIC	NA
Kabuli_wild_10260274	1399939998	*Ca_kabuli_Chr06*	10260274	C/T	Gene_Ca_08563	SYNONYMOUS_CODING	Mediatorcomplex,subunitMed10
Kabuli_wild_10314993	1399939999	*Ca_kabuli_Chr06*	10314993	C/G	NA	INTERGENIC	NA
Kabuli_wild_10394743	1399940000	*Ca_kabuli_Chr06*	10394743	G/A	Gene_Ca_08553	INTRON	Smallubiquitin-relatedmodifier,SUMO
Kabuli_wild_10395273	1399940001	*Ca_kabuli_Chr06*	10395273	T/G	Gene_Ca_08553	INTRON	Smallubiquitin-relatedmodifier,SUMO
Kabuli_wild_10395356	1399940002	*Ca_kabuli_Chr06*	10395356	T/A	Gene_Ca_08553	INTRON	Smallubiquitin-relatedmodifier,SUMO
Kabuli_wild_10395379	1399940003	*Ca_kabuli_Chr06*	10395379	T/C	Gene_Ca_08553	INTRON	Smallubiquitin-relatedmodifier,SUMO
Kabuli_wild_10395411	1399940004	*Ca_kabuli_Chr06*	10395411	G/A	Gene_Ca_08553	INTRON	Smallubiquitin-relatedmodifier,SUMO
Kabuli_wild_10441705	1399940005	*Ca_kabuli_Chr06*	10441705	T/C	Gene_Ca_08551	UPSTREAM	NA
Kabuli_wild_10441736	1399940006	*Ca_kabuli_Chr06*	10441736	A/C	Gene_Ca_08551	NON_SYNONYMOUS_CODING	NA
Kabuli_wild_10442148	1399940007	*Ca_kabuli_Chr06*	10442148	A/C	Gene_Ca_08551	SYNONYMOUS_CODING	NA
Kabuli_wild_10445185	1399940008	*Ca_kabuli_Chr06*	10445185	C/T	Gene_Ca_08551	INTRON	NA
Kabuli_wild_10445274	1399940009	*Ca_kabuli_Chr06*	10445274	G/A	Gene_Ca_08551	INTRON	NA
Kabuli_wild_10456774	1399940010	*Ca_kabuli_Chr06*	10456774	A/G	Gene_Ca_08551	SYNONYMOUS_CODING	NA
Kabuli_wild_10456854	1399940011	*Ca_kabuli_Chr06*	10456854	G/T	Gene_Ca_08551	SYNONYMOUS_CODING	NA
Kabuli_wild_10494055	1399940012	*Ca_kabuli_Chr06*	10494055	T/A	Gene_Ca_08547	INTRON	D-galactoside/L-rhamnosebindingSUELlectindomain
Kabuli_wild_10494057	1399940013	*Ca_kabuli_Chr06*	10494057	C/A	Gene_Ca_08547	INTRON	D-galactoside/L-rhamnosebindingSUELlectindomain
Kabuli_wild_10494134	1399940014	*Ca_kabuli_Chr06*	10494134	C/T	Gene_Ca_08547	INTRON	D-galactoside/L-rhamnosebindingSUELlectindomain
Kabuli_wild_10495747	1399940015	*Ca_kabuli_Chr06*	10495747	G/T	Gene_Ca_08547	INTRON	D-galactoside/L-rhamnosebindingSUELlectindomain
Kabuli_wild_10510996	1399940016	*Ca_kabuli_Chr06*	10510996	T/C	NA	INTERGENIC	NA
Kabuli_wild_10520958	1399940017	*Ca_kabuli_Chr06*	10520958	C/T	Gene_Ca_08543	INTRON	RNA recognition motif domain
Kabuli_wild_10522298	1399940018	*Ca_kabuli_Chr06*	10522298	A/G	Gene_Ca_08543	SYNONYMOUS_CODING	RNA recognition motif domain
Kabuli_wild_10531399	1399940019	*Ca_kabuli_Chr06*	10531399	C/T	Gene_Ca_08542	INTRON	RNA recognition motif domain
Kabuli_wild_10532178	1399940020	*Ca_kabuli_Chr06*	10532178	G/T	Gene_Ca_08542	NON_SYNONYMOUS_CODING	RNA recognition motif domain
Kabuli_wild_10532182	1399940021	*Ca_kabuli_Chr06*	10532182	A/T	Gene_Ca_08542	NON_SYNONYMOUS_CODING	RNA recognition motif domain
Kabuli_wild_10532739	1399940022	*Ca_kabuli_Chr06*	10532739	A/G	Gene_Ca_08542	SYNONYMOUS_CODING	RNA recognition motif domain
Kabuli_wild_10539918	1399940023	*Ca_kabuli_Chr06*	10539918	A/C	NA	INTERGENIC	NA
Kabuli_wild_10539977	1399940024	*Ca_kabuli_Chr06*	10539977	C/T	NA	INTERGENIC	NA
Kabuli_wild_10539994	1399940025	*Ca_kabuli_Chr06*	10539994	A/C	NA	INTERGENIC	NA
Kabuli_wild_10567518	1399940026	*Ca_kabuli_Chr06*	10567518	A/G	NA	INTERGENIC	NA
Kabuli_wild_10574535	1399940027	*Ca_kabuli_Chr06*	10574535	T/C	NA	INTERGENIC	NA
Kabuli_wild_10588030	1399940028	*Ca_kabuli_Chr06*	10588030	C/T	Gene_Ca_08539	DOWNSTREAM	Lateembryogenesisabundantprotein,group6
Kabuli_wild_10613546	1399940029	*Ca_kabuli_Chr06*	10613546	A/G	Gene_Ca_08536	SYNONYMOUS_CODING	Heat shock factor(HSF)-type,DNA-binding
Kabuli_wild_10613612	1399940030	*Ca_kabuli_Chr06*	10613612	A/G	Gene_Ca_08536	SYNONYMOUS_CODING	Heat shock factor(HSF)-type,DNA-binding
Kabuli_wild_10631937	1399940031	*Ca_kabuli_Chr06*	10631937	C/T	NA	INTERGENIC	NA

SNP IDs	NCBI dbSNP accession IDs (NCBI_SS#)	Chromosomes	Physical positions (bp)	SNPs	Gene accession IDs	Sequence components of genome	Putative functions
Kabuli_wild_10632293	1399940032	*Ca_kabuli_Chr06*	10632293	A/G	NA	INTERGENIC	NA
Kabuli_wild_10641737	1399940033	*Ca_kabuli_Chr06*	10641737	C/A	NA	INTERGENIC	NA
Kabuli_wild_10641752	1399940034	*Ca_kabuli_Chr06*	10641752	A/G	NA	INTERGENIC	NA
Kabuli_wild_10641757	1399940035	*Ca_kabuli_Chr06*	10641757	T/G	NA	INTERGENIC	NA
Kabuli_wild_10641758	1399940036	*Ca_kabuli_Chr06*	10641758	T/A	NA	INTERGENIC	NA
Kabuli_wild_10641769	1399940037	*Ca_kabuli_Chr06*	10641769	T/C	NA	INTERGENIC	NA
Kabuli_wild_10641770	1399940038	*Ca_kabuli_Chr06*	10641770	G/A	NA	INTERGENIC	NA
Kabuli_wild_10641771	1399940039	*Ca_kabuli_Chr06*	10641771	A/G	NA	INTERGENIC	NA
Kabuli_wild_10641773	1399940040	*Ca_kabuli_Chr06*	10641773	C/T	NA	INTERGENIC	NA
Kabuli_wild_10641800	1399940041	*Ca_kabuli_Chr06*	10641800	A/G	NA	INTERGENIC	NA
Kabuli_wild_10641802	1399940042	*Ca_kabuli_Chr06*	10641802	C/A	NA	INTERGENIC	NA
Kabuli_wild_10667559	1399940043	*Ca_kabuli_Chr06*	10667559	T/C	NA	INTERGENIC	NA
Kabuli_wild_10670368	1399940044	*Ca_kabuli_Chr06*	10670368	G/A	Gene_Ca_08530	INTRON	Aspartate/glutamate/uridylatekinase
Kabuli_wild_10670409	1399940045	*Ca_kabuli_Chr06*	10670409	G/A	Gene_Ca_08530	INTRON	Aspartate/glutamate/uridylatekinase
Kabuli_wild_10670482	1399940046	*Ca_kabuli_Chr06*	10670482	C/T	Gene_Ca_08530	INTRON	Aspartate/glutamate/uridylatekinase
Kabuli_wild_10670582	1399940047	*Ca_kabuli_Chr06*	10670582	G/A	Gene_Ca_08530	INTRON	Aspartate/glutamate/uridylatekinase
Kabuli_wild_10670694	1399940048	*Ca_kabuli_Chr06*	10670694	G/A	Gene_Ca_08530	INTRON	Aspartate/glutamate/uridylatekinase
Kabuli_wild_10670773	1399940049	*Ca_kabuli_Chr06*	10670773	A/C	Gene_Ca_08530	INTRON	Aspartate/glutamate/uridylatekinase
Kabuli_wild_10670959	1399940050	*Ca_kabuli_Chr06*	10670959	T/C	Gene_Ca_08530	INTRON	Aspartate/glutamate/uridylatekinase
Kabuli_wild_10671444	1399940051	*Ca_kabuli_Chr06*	10671444	A/C	Gene_Ca_08530	INTRON	Aspartate/glutamate/uridylatekinase
Kabuli_wild_10671458	1399940052	*Ca_kabuli_Chr06*	10671458	T/C	Gene_Ca_08530	INTRON	Aspartate/glutamate/uridylatekinase
Kabuli_wild_10672468	1399940053	*Ca_kabuli_Chr06*	10672468	C/T	Gene_Ca_08530	INTRON	Aspartate/glutamate/uridylatekinase
Kabuli_wild_10677469	1399940054	*Ca_kabuli_Chr06*	10677469	A/G	Gene_Ca_08530	INTRON	Aspartate/glutamate/uridylatekinase
Kabuli_wild_10677501	1399940055	*Ca_kabuli_Chr06*	10677501	C/T	Gene_Ca_08530	INTRON	Aspartate/glutamate/uridylatekinase
Kabuli_wild_10682201	1399940056	*Ca_kabuli_Chr06*	10682201	C/G	Gene_Ca_08530	DOWNSTREAM	Aspartate/glutamate/uridylatekinase
Kabuli_wild_10682221	1399940057	*Ca_kabuli_Chr06*	10682221	G/T	Gene_Ca_08530	DOWNSTREAM	Aspartate/glutamate/uridylatekinase
Kabuli_wild_10683193	1399940058	*Ca_kabuli_Chr06*	10683193	C/T	NA	INTERGENIC	NA
Kabuli_wild_10684042	1399940059	*Ca_kabuli_Chr06*	10684042	T/C	NA	INTERGENIC	NA
Kabuli_wild_10696986	1399940060	*Ca_kabuli_Chr06*	10696986	A/T	Gene_Ca_08528	INTRON	Protein kinase,catalytic domain
Kabuli_wild_10696990	1399940061	*Ca_kabuli_Chr06*	10696990	T/C	Gene_Ca_08528	INTRON	Protein kinase,catalytic domain
Kabuli_wild_10697064	1399940062	*Ca_kabuli_Chr06*	10697064	T/A	Gene_Ca_08528	INTRON	Protein kinase,catalytic domain
Kabuli_wild_10716933	1399940063	*Ca_kabuli_Chr06*	10716933	T/G	NA	INTERGENIC	NA
Kabuli_wild_10716968	1399940064	*Ca_kabuli_Chr06*	10716968	G/A	Gene_Ca_08526	SYNONYMOUS_CODING	NA
Kabuli_wild_10716986	1399940065	*Ca_kabuli_Chr06*	10716986	G/A	Gene_Ca_08526	SYNONYMOUS_CODING	NA
Kabuli_wild_10718770	1399940066	*Ca_kabuli_Chr06*	10718770	G/A	Gene_Ca_08526	SYNONYMOUS_CODING	NA
Kabuli_wild_10734364	1399940067	*Ca_kabuli_Chr06*	10734364	A/G	Gene_Ca_08524	INTRON	Tic22-like
Kabuli_wild_10743996	1399940068	*Ca_kabuli_Chr06*	10743996	A/T	NA	INTERGENIC	NA
Kabuli_wild_10744029	1399940069	*Ca_kabuli_Chr06*	10744029	C/A	NA	INTERGENIC	NA
Kabuli_wild_10748707	1399940070	*Ca_kabuli_Chr06*	10748707	C/A	Gene_Ca_08523	NON_SYNONYMOUS_CODING	NA
Kabuli_wild_10778506	1399940071	*Ca_kabuli_Chr06*	10778506	G/A	Gene_Ca_08519	INTRON	Kinesin,motordomain
Kabuli_wild_10779623	1399940072	*Ca_kabuli_Chr06*	10779623	T/C	Gene_Ca_08519	SYNONYMOUS_CODING	Kinesin,motordomain
Kabuli_wild_10779714	1399940073	*Ca_kabuli_Chr06*	10779714	C/A	Gene_Ca_08519	SYNONYMOUS_CODING	Kinesin,motordomain
Kabuli_wild_10779837	1399940074	*Ca_kabuli_Chr06*	10779837	G/A	Gene_Ca_08519	NON_SYNONYMOUS_CODING	Kinesin,motordomain
Kabuli_wild_10780228	1399940075	*Ca_kabuli_Chr06*	10780228	C/T	Gene_Ca_08519	INTRON	Kinesin,motordomain

SNP IDs	NCBI dbSNP accession IDs (NCBI_SS#)	Chromosomes	Physical positions (bp)	SNPs	Gene accession IDs	Sequence components of genome	Putative functions
Kabuli_wild_10780240	1399940076	*Ca_kabuli_Chr06*	10780240	T/C	Gene_Ca_08519	INTRON	Kinesin,motordomain
Kabuli_wild_10781522	1399940077	*Ca_kabuli_Chr06*	10781522	G/T	Gene_Ca_08519	INTRON	Kinesin,motordomain
Kabuli_wild_10785480	1399940078	*Ca_kabuli_Chr06*	10785480	T/G	NA	INTERGENIC	NA
Kabuli_wild_10822140	1399940079	*Ca_kabuli_Chr06*	10822140	G/A	NA	INTERGENIC	NA
Kabuli_wild_10822184	1399940080	*Ca_kabuli_Chr06*	10822184	G/A	NA	INTERGENIC	NA
Kabuli_wild_10870187	1399940081	*Ca_kabuli_Chr06*	10870187	T/G	Gene_Ca_08511	NON_SYNONYMOUS_CODING	IQ motif,EF-handbinding site
Kabuli_wild_10870534	1399940082	*Ca_kabuli_Chr06*	10870534	A/G	Gene_Ca_08511	SYNONYMOUS_CODING	IQ motif,EF-handbinding site
Kabuli_wild_10873180	1399940083	*Ca_kabuli_Chr06*	10873180	G/A	Gene_Ca_08510	DOWNSTREAM	Nucleartransportfactor2
Kabuli_wild_10880791	1399940084	*Ca_kabuli_Chr06*	10880791	C/T	Gene_Ca_08509	INTRON	Signal transduction response regulator, receiverdomain
Kabuli_wild_10880819	1399940085	*Ca_kabuli_Chr06*	10880819	T/G	Gene_Ca_08509	INTRON	Signal transduction response regulator, receiverdomain
Kabuli_wild_10880962	1399940086	*Ca_kabuli_Chr06*	10880962	G/T	Gene_Ca_08509	INTRON	Signal transduction response regulator, receiverdomain
Kabuli_wild_10880985	1399940087	*Ca_kabuli_Chr06*	10880985	C/T	Gene_Ca_08509	INTRON	Signal transduction response regulator, receiverdomain
Kabuli_wild_10881002	1399940088	*Ca_kabuli_Chr06*	10881002	A/G	Gene_Ca_08509	INTRON	Signal transduction response regulator, receiverdomain
Kabuli_wild_10900193	1399940089	*Ca_kabuli_Chr06*	10900193	A/G	Gene_Ca_08507	INTRON	WD40 repeat
Kabuli_wild_10900225	1399940090	*Ca_kabuli_Chr06*	10900225	T/C	Gene_Ca_08507	INTRON	WD40 repeat
Kabuli_wild_10900678	1399940091	*Ca_kabuli_Chr06*	10900678	G/T	Gene_Ca_08507	INTRON	WD40 repeat
Kabuli_wild_10991652	1399940092	*Ca_kabuli_Chr06*	10991652	C/A	Gene_Ca_20467	NON_SYNONYMOUS_CODING	Zinc finger,MYND-type
Kabuli_wild_10991717	1399940093	*Ca_kabuli_Chr06*	10991717	T/A	Gene_Ca_20467	NON_SYNONYMOUS_CODING	Zinc finger,MYND-type
Kabuli_wild_10991737	1399940094	*Ca_kabuli_Chr06*	10991737	A/C	Gene_Ca_20467	SYNONYMOUS_CODING	Zinc finger,MYND-type
Kabuli_wild_11011847	1399940095	*Ca_kabuli_Chr06*	11011847	C/G	Gene_Ca_20469	NON_SYNONYMOUS_CODING	Rubberelongationfactor
Kabuli_wild_11053302	1399940096	*Ca_kabuli_Chr06*	11053302	T/C	Gene_Ca_20472	INTRON	PeptidaseM28
Kabuli_wild_11131010	1399940097	*Ca_kabuli_Chr06*	11131010	T/A	Gene_Ca_20479	INTRON	Glycosidehydrolase,family63
Kabuli_wild_11131464	1399940098	*Ca_kabuli_Chr06*	11131464	G/A	Gene_Ca_20479	INTRON	Glycosidehydrolase,family63
Kabuli_wild_11188803	1399940099	*Ca_kabuli_Chr06*	11188803	G/A	Gene_Ca_20485	NON_SYNONYMOUS_CODING	Protein kinase,catalytic domain
Kabuli_wild_11189051	1399940100	*Ca_kabuli_Chr06*	11189051	A/G	Gene_Ca_20485	SYNONYMOUS_CODING	Protein kinase,catalytic domain
Kabuli_wild_11219633	1399940101	*Ca_kabuli_Chr06*	11219633	A/C	Gene_Ca_20488	INTRON	Pre-mRNAcleavagecomplexIIClp1
Kabuli_wild_11229102	1399940102	*Ca_kabuli_Chr06*	11229102	C/T	Gene_Ca_20489	SYNONYMOUS_CODING	Ankyrinrepeat
Kabuli_wild_11229144	1399940103	*Ca_kabuli_Chr06*	11229144	C/G	Gene_Ca_20489	NON_SYNONYMOUS_CODING	Ankyrinrepeat
Kabuli_wild_11229302	1399940104	*Ca_kabuli_Chr06*	11229302	G/A	Gene_Ca_20489	SYNONYMOUS_CODING	Ankyrinrepeat
Kabuli_wild_11229379	1399940105	*Ca_kabuli_Chr06*	11229379	C/G	Gene_Ca_20489	NON_SYNONYMOUS_CODING	Ankyrinrepeat
Kabuli_wild_11229388	1399940106	*Ca_kabuli_Chr06*	11229388	C/T	Gene_Ca_20489	NON_SYNONYMOUS_CODING	Ankyrinrepeat
Kabuli_wild_11300549	1399940107	*Ca_kabuli_Chr06*	11300549	A/C	Gene_Ca_20493	SYNONYMOUS_CODING	Signalrecognitionparticle,SRP72subunit,RNA-binding
Kabuli_wild_11302033	1399940108	*Ca_kabuli_Chr06*	11302033	T/C	Gene_Ca_20493	NON_SYNONYMOUS_CODING	NA
Kabuli_wild_11302037	1399940109	*Ca_kabuli_Chr06*	11302037	A/C	Gene_Ca_20493	NON_SYNONYMOUS_CODING	NA
Kabuli_wild_11314537	1399940110	*Ca_kabuli_Chr06*	11314537	G/A	Gene_Ca_20494	SYNONYMOUS_CODING	Pentatrico peptide repeat
Kabuli_wild_11314581	1399940111	*Ca_kabuli_Chr06*	11314581	C/T	Gene_Ca_20494	NON_SYNONYMOUS_CODING	Pentatrico peptide repeat
Kabuli_wild_11508816	1399940112	*Ca_kabuli_Chr06*	11508816	A/C	Gene_Ca_23431	NON_SYNONYMOUS_CODING	Heat shock protein DnaJ,N-terminal
Kabuli_wild_11508817	1399940113	*Ca_kabuli_Chr06*	11508817	G/A	Gene_Ca_23431	NON_SYNONYMOUS_CODING	Heat shock protein DnaJ,N-terminal
Kabuli_wild_11508824	1399940114	*Ca_kabuli_Chr06*	11508824	T/G	Gene_Ca_23431	STOP_GAINED	Heat shock protein DnaJ,N-terminal
Kabuli_wild_11508828	1399940115	*Ca_kabuli_Chr06*	11508828	C/T	Gene_Ca_23431	NON_SYNONYMOUS_CODING	Heat shock protein DnaJ,N-terminal
Kabuli_wild_11514852	1399940116	*Ca_kabuli_Chr06*	11514852	A/T	Gene_Ca_23432	NON_SYNONYMOUS_CODING	NA
Kabuli_wild_11514868	1399940117	*Ca_kabuli_Chr06*	11514868	G/A	Gene_Ca_23432	NON_SYNONYMOUS_CODING	NA
Kabuli_wild_11514875	1399940118	*Ca_kabuli_Chr06*	11514875	C/T	Gene_Ca_23432	NON_SYNONYMOUS_CODING	NA
Kabuli_wild_11514950	1399940119	*Ca_kabuli_Chr06*	11514950	C/T	Gene_Ca_23432	NON_SYNONYMOUS_CODING	NA

SNP IDs	NCBI dbSNP accession IDs (NCBI_SS#)	Chromosomes	Physical positions (bp)	SNPs	Gene accession IDs	Sequence components of genome	Putative functions
Kabuli_wild_11514952	1399940120	*Ca_kabuli_Chr06*	11514952	G/A	Gene_Ca_23432	NON_SYNONYMOUS_CODING	NA
Kabuli_wild_11543618	1399940121	*Ca_kabuli_Chr06*	11543618	T/G	NA	INTERGENIC	NA
Kabuli_wild_11665233	1399940122	*Ca_kabuli_Chr06*	11665233	G/A	Gene_Ca_25061	SYNONYMOUS_CODING	Amineoxidase
Kabuli_wild_11665338	1399940123	*Ca_kabuli_Chr06*	11665338	T/G	Gene_Ca_25061	SYNONYMOUS_CODING	Amineoxidase
Kabuli_wild_11839188	1399940124	*Ca_kabuli_Chr06*	11839188	T/C	NA	INTERGENIC	NA
Kabuli_wild_11992488	1399940125	*Ca_kabuli_Chr06*	11992488	C/T	NA	INTERGENIC	NA
Kabuli_wild_12003393	1399940126	*Ca_kabuli_Chr06*	12003393	G/A	NA	INTERGENIC	NA
Kabuli_wild_12029759	1399940127	*Ca_kabuli_Chr06*	12029759	C/T	NA	INTERGENIC	NA
Kabuli_wild_12324310	1399940128	*Ca_kabuli_Chr06*	12324310	G/A	NA	INTERGENIC	NA
Kabuli_wild_12324315	1399940129	*Ca_kabuli_Chr06*	12324315	C/T	NA	INTERGENIC	NA
Kabuli_wild_12324367	1399940130	*Ca_kabuli_Chr06*	12324367	C/G	NA	INTERGENIC	NA
Kabuli_wild_12324389	1399940131	*Ca_kabuli_Chr06*	12324389	G/A	NA	INTERGENIC	NA
Kabuli_wild_12437130	1399940132	*Ca_kabuli_Chr06*	12437130	C/A	Gene_Ca_05025	NON_SYNONYMOUS_CODING	AUX/IAAprotein
Kabuli_wild_12437275	1399940133	*Ca_kabuli_Chr06*	12437275	A/T	Gene_Ca_05025	NON_SYNONYMOUS_CODING	AUX/IAAprotein
Kabuli_wild_12437284	1399940134	*Ca_kabuli_Chr06*	12437284	A/C	Gene_Ca_05025	NON_SYNONYMOUS_CODING	AUX/IAAprotein
Kabuli_wild_12437285	1399940135	*Ca_kabuli_Chr06*	12437285	A/G	Gene_Ca_05025	SYNONYMOUS_CODING	AUX/IAAprotein
Kabuli_wild_12437286	1399940136	*Ca_kabuli_Chr06*	12437286	C/G	Gene_Ca_05025	NON_SYNONYMOUS_CODING	AUX/IAAprotein
Kabuli_wild_12437288	1399940137	*Ca_kabuli_Chr06*	12437288	G/A	Gene_Ca_05025	SYNONYMOUS_CODING	AUX/IAAprotein
Kabuli_wild_12437319	1399940138	*Ca_kabuli_Chr06*	12437319	C/T	Gene_Ca_05025	STOP_GAINED	AUX/IAAprotein
Kabuli_wild_12437330	1399940139	*Ca_kabuli_Chr06*	12437330	A/C	Gene_Ca_05025	SYNONYMOUS_CODING	AUX/IAAprotein
Kabuli_wild_12490253	1399940140	*Ca_kabuli_Chr06*	12490253	C/A	Gene_Ca_05028	NON_SYNONYMOUS_CODING	Zinc finger,C3HC-like
Kabuli_wild_12532947	1399940141	*Ca_kabuli_Chr06*	12532947	A/T	NA	INTERGENIC	NA
Kabuli_wild_12535733	1399940142	*Ca_kabuli_Chr06*	12535733	C/G	Gene_Ca_05031	NON_SYNONYMOUS_CODING	Sugar/inositol transporter
Kabuli_wild_12642779	1399940143	*Ca_kabuli_Chr06*	12642779	G/T	Gene_Ca_05040	UPSTREAM	Integrase,catalyticcore
Kabuli_wild_12799890	1399940144	*Ca_kabuli_Chr06*	12799890	T/G	NA	INTERGENIC	NA
Kabuli_wild_12893335	1399940145	*Ca_kabuli_Chr06*	12893335	C/A	NA	INTERGENIC	NA
Kabuli_wild_12893424	1399940146	*Ca_kabuli_Chr06*	12893424	C/T	NA	INTERGENIC	NA
Kabuli_wild_12905457	1399940147	*Ca_kabuli_Chr06*	12905457	G/A	Gene_Ca_05064	SYNONYMOUS_CODING	Protein kinase,catalytic domain
Kabuli_wild_12905458	1399940148	*Ca_kabuli_Chr06*	12905458	T/A	Gene_Ca_05064	SYNONYMOUS_CODING	Protein kinase,catalytic domain
Kabuli_wild_12906599	1399940149	*Ca_kabuli_Chr06*	12906599	G/A	Gene_Ca_05064	INTRON	Protein kinase,catalytic domain
Kabuli_wild_12906669	1399940150	*Ca_kabuli_Chr06*	12906669	G/A	Gene_Ca_05064	INTRON	Protein kinase,catalytic domain
Kabuli_wild_12906680	1399940151	*Ca_kabuli_Chr06*	12906680	T/C	Gene_Ca_05064	INTRON	Protein kinase,catalytic domain
Kabuli_wild_12973765	1399940152	*Ca_kabuli_Chr06*	12973765	A/G	Gene_Ca_05070	UPSTREAM	RibosomalproteinS17e
Kabuli_wild_12976473	1399940153	*Ca_kabuli_Chr06*	12976473	A/T	Gene_Ca_05070	DOWNSTREAM	RibosomalproteinS17e
Kabuli_wild_12976513	1399940154	*Ca_kabuli_Chr06*	12976513	G/A	Gene_Ca_05070	DOWNSTREAM	RibosomalproteinS17e
Kabuli_wild_12977129	1399940155	*Ca_kabuli_Chr06*	12977129	A/G	Gene_Ca_05070	DOWNSTREAM	RibosomalproteinS17e
Kabuli_wild_12977196	1399940156	*Ca_kabuli_Chr06*	12977196	A/G	Gene_Ca_05070	DOWNSTREAM	RibosomalproteinS17e
Kabuli_wild_12977204	1399940157	*Ca_kabuli_Chr06*	12977204	T/G	Gene_Ca_05070	DOWNSTREAM	RibosomalproteinS17e
Kabuli_wild_12985896	1399940158	*Ca_kabuli_Chr06*	12985896	A/C	NA	INTERGENIC	NA
Kabuli_wild_12985908	1399940159	*Ca_kabuli_Chr06*	12985908	C/G	NA	INTERGENIC	NA
Kabuli_wild_12985910	1399940160	*Ca_kabuli_Chr06*	12985910	C/T	NA	INTERGENIC	NA
Kabuli_wild_12986776	1399940161	*Ca_kabuli_Chr06*	12986776	A/G	NA	INTERGENIC	NA
Kabuli_wild_12986833	1399940162	*Ca_kabuli_Chr06*	12986833	A/C	NA	INTERGENIC	NA
Kabuli_wild_13136955	1399940163	*Ca_kabuli_Chr06*	13136955	G/A	Gene_Ca_05086	INTRON	Cyclicnucleotide-binding domain

SNP IDs	NCBI dbSNP accession IDs (NCBI_SS#)	Chromosomes	Physical positions (bp)	SNPs	Gene accession IDs	Sequence components of genome	Putative functions
Kabuli_wild_13188119	1399940164	*Ca_kabuli_Chr06*	13188119	A/C	NA	INTERGENIC	NA
Kabuli_wild_13228589	1399940165	*Ca_kabuli_Chr06*	13228589	T/C	NA	INTERGENIC	NA
Kabuli_wild_13228714	1399940166	*Ca_kabuli_Chr06*	13228714	G/A	NA	INTERGENIC	NA
Kabuli_wild_13243036	1399940167	*Ca_kabuli_Chr06*	13243036	T/C	Gene_Ca_05097	SYNONYMOUS_CODING	Zinc finger,CCCH-type
Kabuli_wild_13326910	1399940168	*Ca_kabuli_Chr06*	13326910	A/C	Gene_Ca_05104	SYNONYMOUS_CODING	Zinc finger, RING-type
Kabuli_wild_13340012	1399940169	*Ca_kabuli_Chr06*	13340012	A/G	NA	INTERGENIC	NA
Kabuli_wild_13340762	1399940170	*Ca_kabuli_Chr06*	13340762	G/A	NA	INTERGENIC	NA
Kabuli_wild_13413848	1399940171	*Ca_kabuli_Chr06*	13413848	C/G	NA	INTERGENIC	NA
Kabuli_wild_13413912	1399940172	*Ca_kabuli_Chr06*	13413912	T/C	NA	INTERGENIC	NA
Kabuli_wild_13462423	1399940173	*Ca_kabuli_Chr06*	13462423	G/C	Gene_Ca_05116	SYNONYMOUS_CODING	Homeobox
Kabuli_wild_13462428	1399940174	*Ca_kabuli_Chr06*	13462428	C/T	Gene_Ca_05116	SYNONYMOUS_CODING	Homeobox
Kabuli_wild_13462461	1399940175	*Ca_kabuli_Chr06*	13462461	G/T	Gene_Ca_05116	SYNONYMOUS_CODING	Homeobox
Kabuli_wild_13462467	1399940176	*Ca_kabuli_Chr06*	13462467	G/T	Gene_Ca_05116	SYNONYMOUS_CODING	Homeobox
Kabuli_wild_13462480	1399940177	*Ca_kabuli_Chr06*	13462480	G/T	Gene_Ca_05116	SYNONYMOUS_CODING	Homeobox
Kabuli_wild_13462485	1399940178	*Ca_kabuli_Chr06*	13462485	T/C	Gene_Ca_05116	SYNONYMOUS_CODING	Homeobox
Kabuli_wild_13462943	1399940179	*Ca_kabuli_Chr06*	13462943	A/G	Gene_Ca_05116	NON_SYNONYMOUS_CODING	Homeobox
Kabuli_wild_13483841	1399940180	*Ca_kabuli_Chr06*	13483841	T/G	Gene_Ca_05119	UPSTREAM	MitochondrialmatrixMmp37
Kabuli_wild_13565990	1399940181	*Ca_kabuli_Chr06*	13565990	C/T	Gene_Ca_05127	NON_SYNONYMOUS_CODING	HECT
Kabuli_wild_13566007	1399940182	*Ca_kabuli_Chr06*	13566007	C/T	Gene_Ca_05127	SYNONYMOUS_CODING	HECT
Kabuli_wild_13566012	1399940183	*Ca_kabuli_Chr06*	13566012	T/G	Gene_Ca_05127	NON_SYNONYMOUS_CODING	HECT
Kabuli_wild_13566041	1399940184	*Ca_kabuli_Chr06*	13566041	C/T	Gene_Ca_05127	NON_SYNONYMOUS_CODING	HECT
Kabuli_wild_13566110	1399940185	*Ca_kabuli_Chr06*	13566110	A/G	Gene_Ca_05127	NON_SYNONYMOUS_CODING	HECT
Kabuli_wild_13566136	1399940186	*Ca_kabuli_Chr06*	13566136	A/C	Gene_Ca_05127	SYNONYMOUS_CODING	HECT
Kabuli_wild_13572221	1399940187	*Ca_kabuli_Chr06*	13572221	A/C	Gene_Ca_05127	INTRON	HECT
Kabuli_wild_13572236	1399940188	*Ca_kabuli_Chr06*	13572236	A/T	Gene_Ca_05127	INTRON	HECT
Kabuli_wild_13576963	1399940189	*Ca_kabuli_Chr06*	13576963	T/A	Gene_Ca_05127	INTRON	HECT
Kabuli_wild_13584845	1399940190	*Ca_kabuli_Chr06*	13584845	T/C	NA	INTERGENIC	NA
Kabuli_wild_13591590	1399940191	*Ca_kabuli_Chr06*	13591590	T/G	NA	INTERGENIC	NA
Kabuli_wild_13593600	1399940192	*Ca_kabuli_Chr06*	13593600	T/C	NA	INTERGENIC	NA
Kabuli_wild_13619538	1399940193	*Ca_kabuli_Chr06*	13619538	C/T	Gene_Ca_05131	NON_SYNONYMOUS_CODING	HECT
Kabuli_wild_13619555	1399940194	*Ca_kabuli_Chr06*	13619555	C/T	Gene_Ca_05131	SYNONYMOUS_CODING	HECT
Kabuli_wild_13619560	1399940195	*Ca_kabuli_Chr06*	13619560	T/G	Gene_Ca_05131	NON_SYNONYMOUS_CODING	HECT
Kabuli_wild_13619589	1399940196	*Ca_kabuli_Chr06*	13619589	C/T	Gene_Ca_05131	NON_SYNONYMOUS_CODING	HECT
Kabuli_wild_13619658	1399940197	*Ca_kabuli_Chr06*	13619658	A/G	Gene_Ca_05131	NON_SYNONYMOUS_CODING	HECT
Kabuli_wild_13619684	1399940198	*Ca_kabuli_Chr06*	13619684	A/C	Gene_Ca_05131	SYNONYMOUS_CODING	HECT
Kabuli_wild_13622292	1399940199	*Ca_kabuli_Chr06*	13622292	C/T	Gene_Ca_05131	NON_SYNONYMOUS_CODING	HECT
Kabuli_wild_13622301	1399940200	*Ca_kabuli_Chr06*	13622301	A/G	Gene_Ca_05131	NON_SYNONYMOUS_CODING	HECT
Kabuli_wild_13625388	1399940201	*Ca_kabuli_Chr06*	13625388	A/G	Gene_Ca_05132	DOWNSTREAM	E3ubiquitinligase,Domain of unknown function DUF908
Kabuli_wild_13668916	1399940202	*Ca_kabuli_Chr06*	13668916	T/A	NA	INTERGENIC	NA
Kabuli_wild_13668995	1399940203	*Ca_kabuli_Chr06*	13668995	A/G	NA	INTERGENIC	NA
Kabuli_wild_13669006	1399940204	*Ca_kabuli_Chr06*	13669006	G/C	NA	INTERGENIC	NA
Kabuli_wild_13669018	1399940205	*Ca_kabuli_Chr06*	13669018	G/A	NA	INTERGENIC	NA
Kabuli_wild_13669042	1399940206	*Ca_kabuli_Chr06*	13669042	A/G	NA	INTERGENIC	NA
Kabuli_wild_13669048	1399940207	*Ca_kabuli_Chr06*	13669048	T/A	NA	INTERGENIC	NA

SNP IDs	NCBI dbSNP accession IDs (NCBI_SS#)	Chromosomes	Physical positions (bp)	SNPs	Gene accession IDs	Sequence components of genome	Putative functions
Kabuli_wild_13669049	1399940208	*Ca_kabuli_Chr06*	13669049	C/T	NA	INTERGENIC	NA
Kabuli_wild_13669052	1399940209	*Ca_kabuli_Chr06*	13669052	G/C	NA	INTERGENIC	NA
Kabuli_wild_13714741	1399940210	*Ca_kabuli_Chr06*	13714741	C/T	NA	INTERGENIC	NA
Kabuli_wild_13820285	1399940211	*Ca_kabuli_Chr06*	13820285	C/T	NA	INTERGENIC	NA
Kabuli_wild_13827452	1399940212	*Ca_kabuli_Chr06*	13827452	C/G	Gene_Ca_05146	INTRON	Aspartate carbamoyltransferase, eukaryotic
Kabuli_wild_13827484	1399940213	*Ca_kabuli_Chr06*	13827484	C/T	Gene_Ca_05146	SYNONYMOUS_CODING	Aspartate carbamoyltransferase, eukaryotic
Kabuli_wild_13913539	1399940214	*Ca_kabuli_Chr06*	13913539	A/G	Gene_Ca_05156	INTRON	DNA-dependentATPaseMCM
Kabuli_wild_13921930	1399940215	*Ca_kabuli_Chr06*	13921930	A/C	NA	INTERGENIC	NA
Kabuli_wild_13938232	1399940216	*Ca_kabuli_Chr06*	13938232	A/G	Gene_Ca_05159	DOWNSTREAM	Domain of unknown function DUF1084
Kabuli_wild_13942036	1399940217	*Ca_kabuli_Chr06*	13942036	A/C	Gene_Ca_05159	INTRON	Domain of unknown function DUF1085
Kabuli_wild_13948732	1399940218	*Ca_kabuli_Chr06*	13948732	G/T	NA	INTERGENIC	NA
Kabuli_wild_13948778	1399940219	*Ca_kabuli_Chr06*	13948778	A/C	NA	INTERGENIC	NA
Kabuli_wild_13986559	1399940220	*Ca_kabuli_Chr06*	13986559	A/G	NA	INTERGENIC	NA
Kabuli_wild_14017464	1399940221	*Ca_kabuli_Chr06*	14017464	T/A	NA	INTERGENIC	NA
Kabuli_wild_14031802	1399940222	*Ca_kabuli_Chr06*	14031802	A/T	NA	INTERGENIC	NA
Kabuli_wild_14042859	1399940223	*Ca_kabuli_Chr06*	14042859	A/T	Gene_Ca_05169	UPSTREAM	Glycosyltransferase,group1
Kabuli_wild_14067312	1399940224	*Ca_kabuli_Chr06*	14067312	A/C	NA	INTERGENIC	NA
Kabuli_wild_14067326	1399940225	*Ca_kabuli_Chr06*	14067326	C/T	NA	INTERGENIC	NA
Kabuli_wild_14112542	1399940226	*Ca_kabuli_Chr06*	14112542	A/G	Gene_Ca_05177	INTRON	Stressup-regulatedNod19
Kabuli_wild_14148771	1399940227	*Ca_kabuli_Chr06*	14148771	A/G	Gene_Ca_05181	DOWNSTREAM	Disease resistance protein
Kabuli_wild_14155543	1399940228	*Ca_kabuli_Chr06*	14155543	C/T	NA	INTERGENIC	NA
Kabuli_wild_14155643	1399940229	*Ca_kabuli_Chr06*	14155643	A/C	NA	INTERGENIC	NA
Kabuli_wild_14155662	1399940230	*Ca_kabuli_Chr06*	14155662	T/C	NA	INTERGENIC	NA
Kabuli_wild_14160332	1399940231	*Ca_kabuli_Chr06*	14160332	A/G	Gene_Ca_05182	INTRON	N-acetylglucosaminylphosphatidylinositoldeacetylase
Kabuli_wild_14161084	1399940232	*Ca_kabuli_Chr06*	14161084	G/C	Gene_Ca_05182	INTRON	N-acetylglucosaminylphosphatidylinositoldeacetylase
Kabuli_wild_14161141	1399940233	*Ca_kabuli_Chr06*	14161141	A/G	Gene_Ca_05182	INTRON	N-acetylglucosaminylphosphatidylinositoldeacetylase
Kabuli_wild_14169755	1399940234	*Ca_kabuli_Chr06*	14169755	A/G	Gene_Ca_05183	DOWNSTREAM	Tubulin
Kabuli_wild_14169779	1399940235	*Ca_kabuli_Chr06*	14169779	A/G	Gene_Ca_05183	DOWNSTREAM	Tubulin
Kabuli_wild_14169808	1399940236	*Ca_kabuli_Chr06*	14169808	C/G	Gene_Ca_05183	DOWNSTREAM	Tubulin
Kabuli_wild_14200997	1399940237	*Ca_kabuli_Chr06*	14200997	G/A	NA	INTERGENIC	NA
Kabuli_wild_14201001	1399940238	*Ca_kabuli_Chr06*	14201001	T/C	NA	INTERGENIC	NA
Kabuli_wild_14204400	1399940239	*Ca_kabuli_Chr06*	14204400	T/C	NA	INTERGENIC	NA
Kabuli_wild_14204469	1399940240	*Ca_kabuli_Chr06*	14204469	G/A	NA	INTERGENIC	NA
Kabuli_wild_14211297	1399940241	*Ca_kabuli_Chr06*	14211297	G/T	NA	INTERGENIC	NA
Kabuli_wild_14254394	1399940242	*Ca_kabuli_Chr06*	14254394	A/G	NA	INTERGENIC	NA
Kabuli_wild_14309042	1399940243	*Ca_kabuli_Chr06*	14309042	C/T	NA	INTERGENIC	NA
Kabuli_wild_14330533	1399940244	*Ca_kabuli_Chr06*	14330533	T/C	NA	INTERGENIC	NA
Kabuli_wild_14332018	1399940245	*Ca_kabuli_Chr06*	14332018	A/C	NA	INTERGENIC	NA
Kabuli_wild_14332404	1399940246	*Ca_kabuli_Chr06*	14332404	A/G	NA	INTERGENIC	NA
Kabuli_wild_14343605	1399940247	*Ca_kabuli_Chr06*	14343605	A/G	Gene_Ca_05204	INTRON	DNA-binding WRKY
Kabuli_wild_14343619	1399940248	*Ca_kabuli_Chr06*	14343619	C/A	Gene_Ca_05204	INTRON	DNA-binding WRKY
Kabuli_wild_14343645	1399940249	*Ca_kabuli_Chr06*	14343645	G/A	Gene_Ca_05204	INTRON	DNA-binding WRKY
Kabuli_wild_14353598	1399940250	*Ca_kabuli_Chr06*	14353598	C/T	NA	INTERGENIC	NA
Kabuli_wild_14353624	1399940251	*Ca_kabuli_Chr06*	14353624	G/A	NA	INTERGENIC	NA

SNP IDs	NCBI dbSNP accession IDs (NCBI_SS#)	Chromosomes	Physical positions (bp)	SNPs	Gene accession IDs	Sequence components of genome	Putative functions
Kabuli_wild_14371493	1399940252	*Ca_kabuli_Chr06*	14371493	C/T	Gene_Ca_05207	SYNONYMOUS_CODING	Sec1-likeprotein
Kabuli_wild_14371530	1399940253	*Ca_kabuli_Chr06*	14371530	C/T	Gene_Ca_05207	SYNONYMOUS_CODING	Sec1-likeprotein
Kabuli_wild_14375135	1399940254	*Ca_kabuli_Chr06*	14375135	G/A	NA	INTERGENIC	NA
Kabuli_wild_14375141	1399940255	*Ca_kabuli_Chr06*	14375141	G/C	NA	INTERGENIC	NA
Kabuli_wild_14375172	1399940256	*Ca_kabuli_Chr06*	14375172	A/G	NA	INTERGENIC	NA
Kabuli_wild_14375196	1399940257	*Ca_kabuli_Chr06*	14375196	G/A	NA	INTERGENIC	NA
Kabuli_wild_14375207	1399940258	*Ca_kabuli_Chr06*	14375207	C/T	NA	INTERGENIC	NA
Kabuli_wild_14416981	1399940259	*Ca_kabuli_Chr06*	14416981	A/G	Gene_Ca_05213	SYNONYMOUS_CODING	Domain of unknown function DUF676,hydrolase-like
Kabuli_wild_14416997	1399940260	*Ca_kabuli_Chr06*	14416997	A/G	Gene_Ca_05213	NON_SYNONYMOUS_CODING	Domain of unknown function DUF676,hydrolase-like
Kabuli_wild_14419832	1399940261	*Ca_kabuli_Chr06*	14419832	C/T	Gene_Ca_05213	SYNONYMOUS_CODING	Domain of unknown function DUF676,hydrolase-like
Kabuli_wild_14526540	1399940262	*Ca_kabuli_Chr06*	14526540	A/T	NA	INTERGENIC	NA
Kabuli_wild_14589480	1399940263	*Ca_kabuli_Chr06*	14589480	T/C	NA	INTERGENIC	NA
Kabuli_wild_14589520	1399940264	*Ca_kabuli_Chr06*	14589520	G/C	NA	INTERGENIC	NA
Kabuli_wild_14589744	1399940265	*Ca_kabuli_Chr06*	14589744	C/T	Gene_Ca_05228	INTRON	Dynamin,GTPasedomain
Kabuli_wild_14589807	1399940266	*Ca_kabuli_Chr06*	14589807	T/G	Gene_Ca_05228	SYNONYMOUS_CODING	Dynamin,GTPasedomain
Kabuli_wild_14589873	1399940267	*Ca_kabuli_Chr06*	14589873	T/C	Gene_Ca_05228	SYNONYMOUS_CODING	Dynamin,GTPasedomain
Kabuli_wild_14589944	1399940268	*Ca_kabuli_Chr06*	14589944	G/A	Gene_Ca_05228	INTRON	Dynamin,GTPasedomain
Kabuli_wild_14606892	1399940269	*Ca_kabuli_Chr06*	14606892	A/C	NA	INTERGENIC	NA
Kabuli_wild_14628213	1399940270	*Ca_kabuli_Chr06*	14628213	A/T	Gene_Ca_05233	SYNONYMOUS_CODING	Glycosi dehydrolase,family 3, N-terminal
Kabuli_wild_14628215	1399940271	*Ca_kabuli_Chr06*	14628215	T/C	Gene_Ca_05233	NON_SYNONYMOUS_CODING	Glycosi dehydrolase,family 3, N-terminal
Kabuli_wild_14628216	1399940272	*Ca_kabuli_Chr06*	14628216	A/C	Gene_Ca_05233	SYNONYMOUS_CODING	Glycosi dehydrolase,family 3, N-terminal
Kabuli_wild_14753113	1399940273	*Ca_kabuli_Chr06*	14753113	C/A	NA	INTERGENIC	NA
Kabuli_wild_14753122	1399940274	*Ca_kabuli_Chr06*	14753122	C/T	NA	INTERGENIC	NA
Kabuli_wild_14753145	1399940275	*Ca_kabuli_Chr06*	14753145	C/A	NA	INTERGENIC	NA
Kabuli_wild_14753158	1399940276	*Ca_kabuli_Chr06*	14753158	A/G	NA	INTERGENIC	NA
Kabuli_wild_14753190	1399940277	*Ca_kabuli_Chr06*	14753190	G/A	NA	INTERGENIC	NA
Kabuli_wild_14753191	1399940278	*Ca_kabuli_Chr06*	14753191	C/A	NA	INTERGENIC	NA
Kabuli_wild_14762493	1399940279	*Ca_kabuli_Chr06*	14762493	A/G	Gene_Ca_05249	NON_SYNONYMOUS_CODING	K+ potassium transporter
Kabuli_wild_14771602	1399940280	*Ca_kabuli_Chr06*	14771602	T/G	Gene_Ca_05250	INTRON	ATPase,P-type,K/Mg/Cd/Cu/Zn/Na/Ca/Na/H-transporter
Kabuli_wild_14773716	1399940281	*Ca_kabuli_Chr06*	14773716	G/A	Gene_Ca_05250	INTRON	ATPase,P-type,K/Mg/Cd/Cu/Zn/Na/Ca/Na/H-transporter
Kabuli_wild_14786015	1399940282	*Ca_kabuli_Chr06*	14786015	T/G	Gene_Ca_05251	UPSTREAM	NA
Kabuli_wild_14857680	1399940283	*Ca_kabuli_Chr06*	14857680	G/A	NA	INTERGENIC	NA
Kabuli_wild_14857709	1399940284	*Ca_kabuli_Chr06*	14857709	A/G	NA	INTERGENIC	NA
Kabuli_wild_14857722	1399940285	*Ca_kabuli_Chr06*	14857722	C/A	NA	INTERGENIC	NA
Kabuli_wild_14861487	1399940286	*Ca_kabuli_Chr06*	14861487	T/A	Gene_Ca_05260	UPSTREAM	Integrase,catalyticcore
Kabuli_wild_14871864	1399940287	*Ca_kabuli_Chr06*	14871864	T/A	NA	INTERGENIC	NA
Kabuli_wild_14884604	1399940288	*Ca_kabuli_Chr06*	14884604	T/G	NA	INTERGENIC	NA
Kabuli_wild_14901725	1399940289	*Ca_kabuli_Chr06*	14901725	T/C	Gene_Ca_05264	SYNONYMOUS_CODING	ABC transporter-like
Kabuli_wild_14983391	1399940290	*Ca_kabuli_Chr06*	14983391	C/T	Gene_Ca_05273	SYNONYMOUS_CODING	BTB/POZ-like
Kabuli_wild_15051094	1399940291	*Ca_kabuli_Chr06*	15051094	T/C	NA	INTERGENIC	NA
Kabuli_wild_15056637	1399940292	*Ca_kabuli_Chr06*	15056637	C/A	Gene_Ca_05286	INTRON	Protein of unknown function DUF3506
Kabuli_wild_15061717	1399940293	*Ca_kabuli_Chr06*	15061717	G/A	Gene_Ca_05286	INTRON	Protein of unknown function DUF3507
Kabuli_wild_15123739	1399940294	*Ca_kabuli_Chr06*	15123739	C/T	Gene_Ca_05293	INTRON	Dynamincentral domain
Kabuli_wild_15185722	1399940295	*Ca_kabuli_Chr06*	15185722	A/C	NA	INTERGENIC	NA

SNP IDs	NCBI dbSNP accession IDs (NCBI_SS#)	Chromosomes	Physical positions (bp)	SNPs	Gene accession IDs	Sequence components of genome	Putative functions
Kabuli_wild_15289797	1399940296	*Ca_kabuli_Chr06*	15289797	A/C	Gene_Ca_05311	UPSTREAM	Phox/Bem1p
Kabuli_wild_15294329	1399940297	*Ca_kabuli_Chr06*	15294329	A/C	Gene_Ca_05312	UPSTREAM	Histidine phosphatase superfamily,clade-1
Kabuli_wild_15294331	1399940298	*Ca_kabuli_Chr06*	15294331	C/T	Gene_Ca_05312	UPSTREAM	Histidinephosphatasesuperfamily,clade-2
Kabuli_wild_15294332	1399940299	*Ca_kabuli_Chr06*	15294332	T/G	Gene_Ca_05312	UPSTREAM	Histidinephosphatasesuperfamily,clade-3
Kabuli_wild_15301137	1399940300	*Ca_kabuli_Chr06*	15301137	C/T	NA	INTERGENIC	NA
Kabuli_wild_15313655	1399940301	*Ca_kabuli_Chr06*	15313655	G/T	Gene_Ca_05316	NON_SYNONYMOUS_CODING	RegulatorofK+conductance,N-terminal
Kabuli_wild_15463289	1399940302	*Ca_kabuli_Chr06*	15463289	A/G	NA	INTERGENIC	NA
Kabuli_wild_15488309	1399940303	*Ca_kabuli_Chr06*	15488309	G/A	Gene_Ca_05332	INTRON	Kinesin,motordomain
Kabuli_wild_15500046	1399940304	*Ca_kabuli_Chr06*	15500046	C/T	NA	INTERGENIC	NA
Kabuli_wild_15537908	1399940305	*Ca_kabuli_Chr06*	15537908	A/C	Gene_Ca_05338	SYNONYMOUS_CODING	NA
Kabuli_wild_15543286	1399940306	*Ca_kabuli_Chr06*	15543286	G/A	Gene_Ca_05339	NON_SYNONYMOUS_CODING	Tetra tricopeptide repeat
Kabuli_wild_15543293	1399940307	*Ca_kabuli_Chr06*	15543293	T/G	Gene_Ca_05339	NON_SYNONYMOUS_CODING	Tetra tricopeptide repeat
Kabuli_wild_15543295	1399940308	*Ca_kabuli_Chr06*	15543295	C/G	Gene_Ca_05339	NON_SYNONYMOUS_CODING	Tetra tricopeptide repeat
Kabuli_wild_15582726	1399940309	*Ca_kabuli_Chr06*	15582726	T/C	Gene_Ca_05341	INTRON	Terpenesynthase-like
Kabuli_wild_15589885	1399940310	*Ca_kabuli_Chr06*	15589885	G/T	Gene_Ca_05341	INTRON	Terpenesynthase-like
Kabuli_wild_15644412	1399940311	*Ca_kabuli_Chr06*	15644412	A/T	Gene_Ca_05343	INTRON	Terpenesynthase-like
Kabuli_wild_15644796	1399940312	*Ca_kabuli_Chr06*	15644796	A/T	Gene_Ca_05343	INTRON	Terpenesynthase-like
Kabuli_wild_15861278	1399940313	*Ca_kabuli_Chr06*	15861278	G/A	Gene_Ca_22020	UPSTREAM	ABC-1
Kabuli_wild_15923061	1399940314	*Ca_kabuli_Chr06*	15923061	A/G	Gene_Ca_22026	NON_SYNONYMOUS_CODING	Leucine-rich repeat,cysteine-containingsubtype
Kabuli_wild_16113521	1399940315	*Ca_kabuli_Chr06*	16113521	G/T	NA	INTERGENIC	NA
Kabuli_wild_16113522	1399940316	*Ca_kabuli_Chr06*	16113522	A/T	NA	INTERGENIC	NA
Kabuli_wild_16113523	1399940317	*Ca_kabuli_Chr06*	16113523	T/C	NA	INTERGENIC	NA
Kabuli_wild_16148277	1399940318	*Ca_kabuli_Chr06*	16148277	A/C	Gene_Ca_19676	INTRON	Calmodulinbindingprotein-like
Kabuli_wild_16189692	1399940319	*Ca_kabuli_Chr06*	16189692	A/C	Gene_Ca_19671	SYNONYMOUS_CODING	S1/P1nuclease
Kabuli_wild_16189693	1399940320	*Ca_kabuli_Chr06*	16189693	G/T	Gene_Ca_19671	SYNONYMOUS_CODING	S1/P1nuclease
Kabuli_wild_16196582	1399940321	*Ca_kabuli_Chr06*	16196582	G/C	Gene_Ca_19670	SYNONYMOUS_CODING	Sugar/inositol transporter
Kabuli_wild_16332377	1399940322	*Ca_kabuli_Chr06*	16332377	G/A	Gene_Ca_19665	INTRON	Protein of unknown function DUF707
Kabuli_wild_16352068	1399940323	*Ca_kabuli_Chr06*	16352068	T/C	Gene_Ca_19662	UPSTREAM	Armadillo
Kabuli_wild_16460495	1399940324	*Ca_kabuli_Chr06*	16460495	A/C	Gene_Ca_19651	SYNONYMOUS_CODING	NA
Kabuli_wild_16460501	1399940325	*Ca_kabuli_Chr06*	16460501	A/C	Gene_Ca_19651	SYNONYMOUS_CODING	NA
Kabuli_wild_16635061	1399940326	*Ca_kabuli_Chr06*	16635061	C/T	Gene_Ca_06293	INTRON	Double-strandedRNA-binding
Kabuli_wild_16646713	1399940327	*Ca_kabuli_Chr06*	16646713	T/G	Gene_Ca_06294	DOWNSTREAM	Pentatrico peptide repeat
Kabuli_wild_16667858	1399940328	*Ca_kabuli_Chr06*	16667858	C/T	Gene_Ca_06297	INTRON	NA
Kabuli_wild_16719918	1399940329	*Ca_kabuli_Chr06*	16719918	C/A	Gene_Ca_06303	NON_SYNONYMOUS_CODING	DNA mismatch repair protein MutS,C-terminal domain
Kabuli_wild_16719919	1399940330	*Ca_kabuli_Chr06*	16719919	T/G	Gene_Ca_06303	SYNONYMOUS_CODING	DNA mismatch repair protein MutS,C-terminal domain
Kabuli_wild_16741897	1399940331	*Ca_kabuli_Chr06*	16741897	T/C	Gene_Ca_06306	INTRON	Zinc finger, RING-type
Kabuli_wild_16931756	1399940332	*Ca_kabuli_Chr06*	16931756	C/A	Gene_Ca_06319	NON_SYNONYMOUS_CODING	WD40 repeat
Kabuli_wild_16955695	1399940333	*Ca_kabuli_Chr06*	16955695	A/G	NA	INTERGENIC	NA
Kabuli_wild_17096214	1399940334	*Ca_kabuli_Chr06*	17096214	C/T	Gene_Ca_06327	INTRON	Mitochondrialsubstrate/solutecarrier
Kabuli_wild_17096224	1399940335	*Ca_kabuli_Chr06*	17096224	C/T	Gene_Ca_06327	INTRON	Mitochondrialsubstrate/solutecarrier
Kabuli_wild_17129574	1399940336	*Ca_kabuli_Chr06*	17129574	A/G	NA	INTERGENIC	NA
Kabuli_wild_17175334	1399940337	*Ca_kabuli_Chr06*	17175334	C/A	Gene_Ca_06337	DOWNSTREAM	Droughtinduced19/RING finger protein114
Kabuli_wild_17258886	1399940338	*Ca_kabuli_Chr06*	17258886	T/G	Gene_Ca_06343	NON_SYNONYMOUS_CODING	Homeobox
Kabuli_wild_17262283	1399940339	*Ca_kabuli_Chr06*	17262283	C/T	Gene_Ca_06343	SYNONYMOUS_CODING	Homeobox

SNP IDs	NCBI dbSNP accession IDs (NCBI_SS#)	Chromosomes	Physical positions (bp)	SNPs	Gene accession IDs	Sequence components of genome	Putative functions
Kabuli_wild_17263126	1399940340	*Ca_kabuli_Chr06*	17263126	G/A	Gene_Ca_06343	SYNONYMOUS_CODING	Homeobox
Kabuli_wild_17369613	1399940341	*Ca_kabuli_Chr06*	17369613	A/G	Gene_Ca_06352	SYNONYMOUS_CODING	Transcriptional factor B3
Kabuli_wild_17396315	1399940342	*Ca_kabuli_Chr06*	17396315	A/G	Gene_Ca_06354	SYNONYMOUS_CODING	O-methyltransferase,family2
Kabuli_wild_17445945	1399940343	*Ca_kabuli_Chr06*	17445945	T/G	NA	INTERGENIC	NA
Kabuli_wild_17477741	1399940344	*Ca_kabuli_Chr06*	17477741	A/G	Gene_Ca_06361	SYNONYMOUS_CODING	NA
Kabuli_wild_17478220	1399940345	*Ca_kabuli_Chr06*	17478220	G/A	Gene_Ca_06361	SYNONYMOUS_CODING	NA
Kabuli_wild_17480486	1399940346	*Ca_kabuli_Chr06*	17480486	G/C	Gene_Ca_06361	SYNONYMOUS_CODING	NA
Kabuli_wild_17481288	1399940347	*Ca_kabuli_Chr06*	17481288	T/C	NA	INTERGENIC	NA
Kabuli_wild_17481307	1399940348	*Ca_kabuli_Chr06*	17481307	C/A	NA	INTERGENIC	NA
Kabuli_wild_17481392	1399940349	*Ca_kabuli_Chr06*	17481392	A/G	Gene_Ca_06362	DOWNSTREAM	Protein of unknown function DUF701,zinc-bindingputative
Kabuli_wild_17481403	1399940350	*Ca_kabuli_Chr06*	17481403	T/G	Gene_Ca_06362	DOWNSTREAM	Protein of unknown function DUF701,zinc-bindingputative
Kabuli_wild_17482626	1399940351	*Ca_kabuli_Chr06*	17482626	A/G	Gene_Ca_06362	DOWNSTREAM	Protein of unknown function DUF701,zinc-bindingputative
Kabuli_wild_17482640	1399940352	*Ca_kabuli_Chr06*	17482640	C/T	Gene_Ca_06362	DOWNSTREAM	Protein of unknown function DUF701,zinc-bindingputative
Kabuli_wild_17482649	1399940353	*Ca_kabuli_Chr06*	17482649	A/G	Gene_Ca_06362	DOWNSTREAM	Protein of unknown function DUF701,zinc-bindingputative
Kabuli_wild_17482697	1399940354	*Ca_kabuli_Chr06*	17482697	A/G	Gene_Ca_06362	DOWNSTREAM	Protein of unknown function DUF701,zinc-bindingputative
Kabuli_wild_17482718	1399940355	*Ca_kabuli_Chr06*	17482718	G/A	Gene_Ca_06362	DOWNSTREAM	Protein of unknown function DUF701,zinc-bindingputative
Kabuli_wild_17573309	1399940356	*Ca_kabuli_Chr06*	17573309	A/G	Gene_Ca_06374	INTRON	Apoptosisinhibitory5
Kabuli_wild_17659067	1399940357	*Ca_kabuli_Chr06*	17659067	A/C	NA	INTERGENIC	NA
Kabuli_wild_17726996	1399940358	*Ca_kabuli_Chr06*	17726996	A/C	NA	INTERGENIC	NA
Kabuli_wild_17741078	1399940359	*Ca_kabuli_Chr06*	17741078	G/A	Gene_Ca_06393	SYNONYMOUS_CODING	WW/Rsp5/WWP
Kabuli_wild_17766868	1399940360	*Ca_kabuli_Chr06*	17766868	A/G	NA	INTERGENIC	NA
Kabuli_wild_18027391	1399940361	*Ca_kabuli_Chr06*	18027391	G/A	Gene_Ca_06422	INTRON	Myb,DNA-binding
Kabuli_wild_18048738	1399940362	*Ca_kabuli_Chr06*	18048738	T/A	NA	INTERGENIC	NA
Kabuli_wild_18048770	1399940363	*Ca_kabuli_Chr06*	18048770	C/T	NA	INTERGENIC	NA
Kabuli_wild_18094691	1399940364	*Ca_kabuli_Chr06*	18094691	C/A	Gene_Ca_06428	UPSTREAM	NA
Kabuli_wild_18125422	1399940365	*Ca_kabuli_Chr06*	18125422	T/G	NA	INTERGENIC	NA
Kabuli_wild_18125613	1399940366	*Ca_kabuli_Chr06*	18125613	C/A	NA	INTERGENIC	NA
Kabuli_wild_18241362	1399940367	*Ca_kabuli_Chr06*	18241362	C/A	NA	INTERGENIC	NA
Kabuli_wild_18241363	1399940368	*Ca_kabuli_Chr06*	18241363	G/T	NA	INTERGENIC	NA
Kabuli_wild_18389661	1399940369	*Ca_kabuli_Chr06*	18389661	A/C	Gene_Ca_06455	INTRON	Glycosyltransferase,family10
Kabuli_wild_18389704	1399940370	*Ca_kabuli_Chr06*	18389704	T/G	Gene_Ca_06455	INTRON	Glycosyltransferase,family10
Kabuli_wild_18444725	1399940371	*Ca_kabuli_Chr06*	18444725	T/C	NA	INTERGENIC	NA
Kabuli_wild_18528804	1399940372	*Ca_kabuli_Chr06*	18528804	T/G	Gene_Ca_06465	NON_SYNONYMOUS_CODING	U box domain
Kabuli_wild_18796322	1399940373	*Ca_kabuli_Chr06*	18796322	T/C	NA	INTERGENIC	NA
Kabuli_wild_18798685	1399940374	*Ca_kabuli_Chr06*	18798685	T/G	Gene_Ca_06490	INTRON	Heat shock protein DnaJ,N-terminal
Kabuli_wild_18853331	1399940375	*Ca_kabuli_Chr06*	18853331	G/A	Gene_Ca_06494	SYNONYMOUS_CODING	Auxin efflux carrier
Kabuli_wild_18916616	1399940376	*Ca_kabuli_Chr06*	18916616	A/C	Gene_Ca_06499	INTRON	Uridinekinase
Kabuli_wild_18987220	1399940377	*Ca_kabuli_Chr06*	18987220	T/G	NA	INTERGENIC	NA
Kabuli_wild_19125254	1399940378	*Ca_kabuli_Chr06*	19125254	G/A	NA	INTERGENIC	NA
Kabuli_wild_19143420	1399940379	*Ca_kabuli_Chr06*	19143420	A/G	NA	INTERGENIC	NA
Kabuli_wild_19198359	1399940380	*Ca_kabuli_Chr06*	19198359	T/C	Gene_Ca_06528	INTRON	GlutathioneS-transferase,C-terminal
Kabuli_wild_19308544	1399940381	*Ca_kabuli_Chr06*	19308544	C/T	Gene_Ca_06537	INTRON	Zinc finger,ZPR1-type
Kabuli_wild_19308589	1399940382	*Ca_kabuli_Chr06*	19308589	A/C	Gene_Ca_06537	INTRON	Zinc finger,ZPR1-type
Kabuli_wild_19450845	1399940383	*Ca_kabuli_Chr06*	19450845	T/A	Gene_Ca_06550	INTRON	ATPase,AAA+type,core

SNP IDs	NCBI dbSNP accession IDs (NCBI_SS#)	Chromosomes	Physical positions (bp)	SNPs	Gene accession IDs	Sequence components of genome	Putative functions
Kabuli_wild_19551459	1399940384	*Ca_kabuli_Chr06*	19551459	G/A	Gene_Ca_06563	INTRON	Asparaginesynthase
Kabuli_wild_19551504	1399940385	*Ca_kabuli_Chr06*	19551504	G/C	Gene_Ca_06563	INTRON	Asparaginesynthase
Kabuli_wild_19551561	1399940386	*Ca_kabuli_Chr06*	19551561	G/A	Gene_Ca_06563	INTRON	Asparaginesynthase
Kabuli_wild_19557154	1399940387	*Ca_kabuli_Chr06*	19557154	A/G	Gene_Ca_06563	INTRON	Asparaginesynthase
Kabuli_wild_20505641	1399940388	*Ca_kabuli_Chr06*	20505641	T/A	Gene_Ca_16381	SYNONYMOUS_CODING	RNA recognition motif domain
Kabuli_wild_20766377	1399940389	*Ca_kabuli_Chr06*	20766377	C/A	Gene_Ca_16355	NON_SYNONYMOUS_CODING	Helix-loop-helixDNA-binding domain
Kabuli_wild_20766386	1399940390	*Ca_kabuli_Chr06*	20766386	C/T	Gene_Ca_16355	NON_SYNONYMOUS_CODING	Cullin,N-terminal
Kabuli_wild_20830353	1399940391	*Ca_kabuli_Chr06*	20830353	T/G	NA	INTERGENIC	NA
Kabuli_wild_20917713	1399940392	*Ca_kabuli_Chr06*	20917713	A/C	Gene_Ca_16338	DOWNSTREAM	Domain of unknown function DUF1692
Kabuli_wild_21025673	1399940393	*Ca_kabuli_Chr06*	21025673	C/T	NA	INTERGENIC	NA
Kabuli_wild_21025677	1399940394	*Ca_kabuli_Chr06*	21025677	T/A	NA	INTERGENIC	NA
Kabuli_wild_21025706	1399940395	*Ca_kabuli_Chr06*	21025706	C/T	NA	INTERGENIC	NA
Kabuli_wild_21025742	1399940396	*Ca_kabuli_Chr06*	21025742	T/G	NA	INTERGENIC	NA
Kabuli_wild_21025759	1399940397	*Ca_kabuli_Chr06*	21025759	T/A	NA	INTERGENIC	NA
Kabuli_wild_21025797	1399940398	*Ca_kabuli_Chr06*	21025797	T/A	NA	INTERGENIC	NA
Kabuli_wild_21025889	1399940399	*Ca_kabuli_Chr06*	21025889	C/T	NA	INTERGENIC	NA
Kabuli_wild_21025894	1399940400	*Ca_kabuli_Chr06*	21025894	G/T	NA	INTERGENIC	NA
Kabuli_wild_21135764	1399940401	*Ca_kabuli_Chr06*	21135764	C/T	Gene_Ca_19889	NON_SYNONYMOUS_CODING	NA
Kabuli_wild_21136106	1399940402	*Ca_kabuli_Chr06*	21136106	A/T	Gene_Ca_19889	NON_SYNONYMOUS_CODING	NA
Kabuli_wild_21139038	1399940403	*Ca_kabuli_Chr06*	21139038	C/G	NA	INTERGENIC	NA
Kabuli_wild_21165999	1399940404	*Ca_kabuli_Chr06*	21165999	G/T	Gene_Ca_19887	DOWNSTREAM	RibosomalproteinL1
Kabuli_wild_21237395	1399940405	*Ca_kabuli_Chr06*	21237395	T/G	Gene_Ca_19881	DOWNSTREAM	Protein kinase,catalytic domain
Kabuli_wild_21238707	1399940406	*Ca_kabuli_Chr06*	21238707	G/T	NA	INTERGENIC	NA
Kabuli_wild_21276479	1399940407	*Ca_kabuli_Chr06*	21276479	G/T	NA	INTERGENIC	NA
Kabuli_wild_21323378	1399940408	*Ca_kabuli_Chr06*	21323378	A/G	Gene_Ca_19873	INTRON	Phosphoenolpyruvatecarboxylase,activesite
Kabuli_wild_21323438	1399940409	*Ca_kabuli_Chr06*	21323438	T/C	Gene_Ca_19873	SYNONYMOUS_CODING	Phosphoenolpyruvatecarboxylase,activesite
Kabuli_wild_21385915	1399940410	*Ca_kabuli_Chr06*	21385915	C/A	NA	INTERGENIC	NA
Kabuli_wild_21478816	1399940411	*Ca_kabuli_Chr06*	21478816	C/T	NA	INTERGENIC	NA
Kabuli_wild_21478820	1399940412	*Ca_kabuli_Chr06*	21478820	T/G	NA	INTERGENIC	NA
Kabuli_wild_21483425	1399940413	*Ca_kabuli_Chr06*	21483425	T/G	NA	INTERGENIC	NA
Kabuli_wild_21483429	1399940414	*Ca_kabuli_Chr06*	21483429	C/T	NA	INTERGENIC	NA
Kabuli_wild_21483494	1399940415	*Ca_kabuli_Chr06*	21483494	T/G	NA	INTERGENIC	NA
Kabuli_wild_21659607	1399940416	*Ca_kabuli_Chr06*	21659607	A/G	Gene_Ca_11274	NON_SYNONYMOUS_CODING	Zinc finger,C2H2-type
Kabuli_wild_21718674	1399940417	*Ca_kabuli_Chr06*	21718674	G/T	Gene_Ca_11268	NON_SYNONYMOUS_CODING	Myb,DNA-binding
Kabuli_wild_21718891	1399940418	*Ca_kabuli_Chr06*	21718891	A/C	Gene_Ca_11268	SYNONYMOUS_CODING	Myb,DNA-binding
Kabuli_wild_21763468	1399940419	*Ca_kabuli_Chr06*	21763468	G/A	Gene_Ca_11263	UPSTREAM	Pentatrico peptide repeat
Kabuli_wild_21940793	1399940420	*Ca_kabuli_Chr06*	21940793	T/C	NA	INTERGENIC	NA
Kabuli_wild_22017180	1399940421	*Ca_kabuli_Chr06*	22017180	T/C	Gene_Ca_11238	NON_SYNONYMOUS_CODING	Glycosyltransferase,family48
Kabuli_wild_22038857	1399940422	*Ca_kabuli_Chr06*	22038857	A/G	Gene_Ca_11236	UPSTREAM	NA
Kabuli_wild_22137464	1399940423	*Ca_kabuli_Chr06*	22137464	G/A	Gene_Ca_11225	UPSTREAM	Cytochrome P450
Kabuli_wild_22192498	1399940424	*Ca_kabuli_Chr06*	22192498	A/C	Gene_Ca_11219	NON_SYNONYMOUS_CODING	Exostosin-like
Kabuli_wild_22192503	1399940425	*Ca_kabuli_Chr06*	22192503	G/A	Gene_Ca_11219	SYNONYMOUS_CODING	Exostosin-like
Kabuli_wild_22194387	1399940426	*Ca_kabuli_Chr06*	22194387	C/A	Gene_Ca_11219	STOP_GAINED	Exostosin-like
Kabuli_wild_22208093	1399940427	*Ca_kabuli_Chr06*	22208093	G/A	NA	INTERGENIC	NA

SNP IDs	NCBI dbSNP accession IDs (NCBI_SS#)	Chromosomes	Physical positions (bp)	SNPs	Gene accession IDs	Sequence components of genome	Putative functions
Kabuli_wild_22208125	1399940428	*Ca_kabuli_Chr06*	22208125	G/A	NA	INTERGENIC	NA
Kabuli_wild_22211777	1399940429	*Ca_kabuli_Chr06*	22211777	T/C	NA	INTERGENIC	NA
Kabuli_wild_22211823	1399940430	*Ca_kabuli_Chr06*	22211823	T/C	NA	INTERGENIC	NA
Kabuli_wild_22217157	1399940431	*Ca_kabuli_Chr06*	22217157	T/G	Gene_Ca_11217	DOWNSTREAM	ATPase,AAA+type,core
Kabuli_wild_22217158	1399940432	*Ca_kabuli_Chr06*	22217158	A/G	Gene_Ca_11217	DOWNSTREAM	ATPase,AAA+type,core
Kabuli_wild_22225288	1399940433	*Ca_kabuli_Chr06*	22225288	T/G	NA	INTERGENIC	NA
Kabuli_wild_22236515	1399940434	*Ca_kabuli_Chr06*	22236515	G/C	NA	INTERGENIC	NA
Kabuli_wild_22252149	1399940435	*Ca_kabuli_Chr06*	22252149	G/A	Gene_Ca_11212	DOWNSTREAM	Transcription facto rGRAS
Kabuli_wild_22263388	1399940436	*Ca_kabuli_Chr06*	22263388	A/G	Gene_Ca_11210	DOWNSTREAM	Zinc finger, RING-type
Kabuli_wild_22352976	1399940437	*Ca_kabuli_Chr06*	22352976	T/C	NA	INTERGENIC	NA
Kabuli_wild_22370242	1399940438	*Ca_kabuli_Chr06*	22370242	C/T	NA	INTERGENIC	NA
Kabuli_wild_22446556	1399940439	*Ca_kabuli_Chr06*	22446556	G/A	Gene_Ca_11195	NON_SYNONYMOUS_CODING	Peptidase C48, SUMO/Sentrin/Ubl1
Kabuli_wild_22446562	1399940440	*Ca_kabuli_Chr06*	22446562	G/A	Gene_Ca_11195	NON_SYNONYMOUS_CODING	Peptidase C48, SUMO/Sentrin/Ubl1
Kabuli_wild_22446571	1399940441	*Ca_kabuli_Chr06*	22446571	G/A	Gene_Ca_11195	NON_SYNONYMOUS_CODING	Peptidase C48, SUMO/Sentrin/Ubl1
Kabuli_wild_22446610	1399940442	*Ca_kabuli_Chr06*	22446610	G/A	Gene_Ca_11195	NON_SYNONYMOUS_CODING	Peptidase C48, SUMO/Sentrin/Ubl1
Kabuli_wild_22446646	1399940443	*Ca_kabuli_Chr06*	22446646	G/A	Gene_Ca_11195	NON_SYNONYMOUS_CODING	Peptidase C48, SUMO/Sentrin/Ubl1
Kabuli_wild_22446649	1399940444	*Ca_kabuli_Chr06*	22446649	C/T	Gene_Ca_11195	NON_SYNONYMOUS_CODING	Peptidase C48, SUMO/Sentrin/Ubl1
Kabuli_wild_22482366	1399940445	*Ca_kabuli_Chr06*	22482366	A/C	Gene_Ca_11192	INTRON	Nitrite/sulphitereductase,hemoproteinbeta-component,ferrodoxin-like
Kabuli_wild_22881205	1399940446	*Ca_kabuli_Chr06*	22881205	A/G	Gene_Ca_11157	INTRON	Inorganicpolyphosphate/ATP-NADkinase,predicted
Kabuli_wild_23023346	1399940447	*Ca_kabuli_Chr06*	23023346	A/C	NA	INTERGENIC	NA
Kabuli_wild_23039097	1399940448	*Ca_kabuli_Chr06*	23039097	C/T	Gene_Ca_11144	DOWNSTREAM	NA
Kabuli_wild_23039125	1399940449	*Ca_kabuli_Chr06*	23039125	G/A	Gene_Ca_11144	DOWNSTREAM	NA
Kabuli_wild_23039145	1399940450	*Ca_kabuli_Chr06*	23039145	A/G	Gene_Ca_11144	DOWNSTREAM	NA
Kabuli_wild_23039147	1399940451	*Ca_kabuli_Chr06*	23039147	A/G	Gene_Ca_11144	DOWNSTREAM	NA
Kabuli_wild_23039165	1399940452	*Ca_kabuli_Chr06*	23039165	T/C	Gene_Ca_11144	DOWNSTREAM	NA
Kabuli_wild_23039776	1399940453	*Ca_kabuli_Chr06*	23039776	G/A	NA	INTERGENIC	NA
Kabuli_wild_23043274	1399940454	*Ca_kabuli_Chr06*	23043274	T/C	Gene_Ca_11143	NON_SYNONYMOUS_CODING	Glycosidehydrolase,family17
Kabuli_wild_23043295	1399940455	*Ca_kabuli_Chr06*	23043295	T/G	Gene_Ca_11143	SYNONYMOUS_CODING	Glycosidehydrolase,family17
Kabuli_wild_23043309	1399940456	*Ca_kabuli_Chr06*	23043309	T/A	Gene_Ca_11143	NON_SYNONYMOUS_CODING	Glycosidehydrolase,family17
Kabuli_wild_23043338	1399940457	*Ca_kabuli_Chr06*	23043338	C/A	Gene_Ca_11143	SYNONYMOUS_CODING	Glycosidehydrolase,family17
Kabuli_wild_23076791	1399940458	*Ca_kabuli_Chr06*	23076791	C/T	NA	INTERGENIC	NA
Kabuli_wild_23076819	1399940459	*Ca_kabuli_Chr06*	23076819	G/A	NA	INTERGENIC	NA
Kabuli_wild_23076839	1399940460	*Ca_kabuli_Chr06*	23076839	A/G	NA	INTERGENIC	NA
Kabuli_wild_23076841	1399940461	*Ca_kabuli_Chr06*	23076841	A/G	NA	INTERGENIC	NA
Kabuli_wild_23076859	1399940462	*Ca_kabuli_Chr06*	23076859	T/C	NA	INTERGENIC	NA
Kabuli_wild_23380698	1399940463	*Ca_kabuli_Chr06*	23380698	T/G	Gene_Ca_11121	SYNONYMOUS_CODING	Ropnucleotideexchanger,PRONE
Kabuli_wild_23549267	1399940464	*Ca_kabuli_Chr06*	23549267	T/G	NA	INTERGENIC	NA
Kabuli_wild_23580007	1399940465	*Ca_kabuli_Chr06*	23580007	A/G	Gene_Ca_11098	UPSTREAM	NA
Kabuli_wild_23696243	1399940466	*Ca_kabuli_Chr06*	23696243	C/G	Gene_Ca_11083	NON_SYNONYMOUS_CODING	ATPase,AAA+type,core
Kabuli_wild_23705409	1399940467	*Ca_kabuli_Chr06*	23705409	A/G	Gene_Ca_11083	INTRON	ATPase,AAA+type,core
Kabuli_wild_23766943	1399940468	*Ca_kabuli_Chr06*	23766943	C/T	Gene_Ca_11079	INTRON	Tetra tricopeptide repeat-containing
Kabuli_wild_23767700	1399940469	*Ca_kabuli_Chr06*	23767700	C/A	Gene_Ca_11079	NON_SYNONYMOUS_CODING	Tetra tricopeptide repeat-containing
Kabuli_wild_23809179	1399940470	*Ca_kabuli_Chr06*	23809179	C/T	Gene_Ca_11078	NON_SYNONYMOUS_CODING	Alanyl-tRNAsynthetase,classIIc
Kabuli_wild_23973410	1399940471	*Ca_kabuli_Chr06*	23973410	G/A	Gene_Ca_11064	INTRON	RNA recognition motif domain

SNP IDs	NCBI dbSNP accession IDs (NCBI_SS#)	Chromosomes	Physical positions (bp)	SNPs	Gene accession IDs	Sequence components of genome	Putative functions
Kabuli_wild_23993044	1399940472	*Ca_kabuli_Chr06*	23993044	T/C	Gene_Ca_11063	INTRON	NA
Kabuli_wild_24007185	1399940473	*Ca_kabuli_Chr06*	24007185	C/T	NA	INTERGENIC	NA
Kabuli_wild_24235632	1399940474	*Ca_kabuli_Chr06*	24235632	C/T	NA	INTERGENIC	NA
Kabuli_wild_24235675	1399940475	*Ca_kabuli_Chr06*	24235675	C/T	NA	INTERGENIC	NA
Kabuli_wild_24403316	1399940476	*Ca_kabuli_Chr06*	24403316	G/T	Gene_Ca_11029	NON_SYNONYMOUS_CODING	PeptidaseS8/S53,subtilisin/kexin/sedolisin
Kabuli_wild_24455026	1399940477	*Ca_kabuli_Chr06*	24455026	A/G	Gene_Ca_11025	INTRON	Haemperoxidase,plant/fungal/bacterial
Kabuli_wild_24516532	1399940478	*Ca_kabuli_Chr06*	24516532	T/C	Gene_Ca_27822	SYNONYMOUS_CODING	NA
Kabuli_wild_24586237	1399940479	*Ca_kabuli_Chr06*	24586237	G/T	NA	INTERGENIC	NA
Kabuli_wild_24661895	1399940480	*Ca_kabuli_Chr06*	24661895	G/A	NA	INTERGENIC	NA
Kabuli_wild_24661916	1399940481	*Ca_kabuli_Chr06*	24661916	C/T	NA	INTERGENIC	NA
Kabuli_wild_24661937	1399940482	*Ca_kabuli_Chr06*	24661937	C/T	NA	INTERGENIC	NA
Kabuli_wild_24661945	1399940483	*Ca_kabuli_Chr06*	24661945	T/A	NA	INTERGENIC	NA
Kabuli_wild_24661986	1399940484	*Ca_kabuli_Chr06*	24661986	G/A	NA	INTERGENIC	NA
Kabuli_wild_24661999	1399940485	*Ca_kabuli_Chr06*	24661999	G/A	NA	INTERGENIC	NA
Kabuli_wild_24662035	1399940486	*Ca_kabuli_Chr06*	24662035	T/G	NA	INTERGENIC	NA
Kabuli_wild_24662615	1399940487	*Ca_kabuli_Chr06*	24662615	T/C	NA	INTERGENIC	NA
Kabuli_wild_24965983	1399940488	*Ca_kabuli_Chr06*	24965983	T/A	NA	INTERGENIC	NA
Kabuli_wild_25106229	1399940489	*Ca_kabuli_Chr06*	25106229	C/T	Gene_Ca_25128	INTRON	NA
Kabuli_wild_25106238	1399940490	*Ca_kabuli_Chr06*	25106238	G/T	Gene_Ca_25128	INTRON	NA
Kabuli_wild_25106241	1399940491	*Ca_kabuli_Chr06*	25106241	G/T	Gene_Ca_25128	INTRON	NA
Kabuli_wild_25118039	1399940492	*Ca_kabuli_Chr06*	25118039	C/T	NA	INTERGENIC	NA
Kabuli_wild_25574947	1399940493	*Ca_kabuli_Chr06*	25574947	C/T	Gene_Ca_24008	SYNONYMOUS_CODING	NA
Kabuli_wild_25574994	1399940494	*Ca_kabuli_Chr06*	25574994	T/C	Gene_Ca_24008	NON_SYNONYMOUS_CODING	NA
Kabuli_wild_25575016	1399940495	*Ca_kabuli_Chr06*	25575016	G/A	Gene_Ca_24008	SYNONYMOUS_CODING	NA
Kabuli_wild_25575045	1399940496	*Ca_kabuli_Chr06*	25575045	C/A	Gene_Ca_24008	NON_SYNONYMOUS_CODING	NA
Kabuli_wild_25575062	1399940497	*Ca_kabuli_Chr06*	25575062	G/A	Gene_Ca_24008	NON_SYNONYMOUS_CODING	NA
Kabuli_wild_25575078	1399940498	*Ca_kabuli_Chr06*	25575078	C/T	Gene_Ca_24008	NON_SYNONYMOUS_CODING	NA
Kabuli_wild_25575097	1399940499	*Ca_kabuli_Chr06*	25575097	C/T	Gene_Ca_24008	SYNONYMOUS_CODING	NA
Kabuli_wild_25604922	1399940500	*Ca_kabuli_Chr06*	25604922	A/G	NA	INTERGENIC	NA
Kabuli_wild_25604931	1399940501	*Ca_kabuli_Chr06*	25604931	C/A	NA	INTERGENIC	NA
Kabuli_wild_25604936	1399940502	*Ca_kabuli_Chr06*	25604936	C/T	NA	INTERGENIC	NA
Kabuli_wild_25604942	1399940503	*Ca_kabuli_Chr06*	25604942	C/A	NA	INTERGENIC	NA
Kabuli_wild_25604943	1399940504	*Ca_kabuli_Chr06*	25604943	T/G	NA	INTERGENIC	NA
Kabuli_wild_25604954	1399940505	*Ca_kabuli_Chr06*	25604954	C/T	NA	INTERGENIC	NA
Kabuli_wild_25604959	1399940506	*Ca_kabuli_Chr06*	25604959	G/A	NA	INTERGENIC	NA
Kabuli_wild_25604962	1399940507	*Ca_kabuli_Chr06*	25604962	G/A	NA	INTERGENIC	NA
Kabuli_wild_25604963	1399940508	*Ca_kabuli_Chr06*	25604963	C/T	NA	INTERGENIC	NA
Kabuli_wild_25604987	1399940509	*Ca_kabuli_Chr06*	25604987	G/C	NA	INTERGENIC	NA
Kabuli_wild_25604988	1399940510	*Ca_kabuli_Chr06*	25604988	C/T	NA	INTERGENIC	NA
Kabuli_wild_25605006	1399940511	*Ca_kabuli_Chr06*	25605006	C/A	NA	INTERGENIC	NA
Kabuli_wild_25605009	1399940512	*Ca_kabuli_Chr06*	25605009	C/T	NA	INTERGENIC	NA
Kabuli_wild_25605022	1399940513	*Ca_kabuli_Chr06*	25605022	C/T	NA	INTERGENIC	NA
Kabuli_wild_25605023	1399940514	*Ca_kabuli_Chr06*	25605023	C/A	NA	INTERGENIC	NA
Kabuli_wild_25605038	1399940515	*Ca_kabuli_Chr06*	25605038	T/C	NA	INTERGENIC	NA

SNP IDs	NCBI dbSNP accession IDs (NCBI_SS#)	Chromosomes	Physical positions (bp)	SNPs	Gene accession IDs	Sequence components of genome	Putative functions
Kabuli_wild_25632536	1399940516	*Ca_kabuli_Chr06*	25632536	C/A	NA	INTERGENIC	NA
Kabuli_wild_25850673	1399940517	*Ca_kabuli_Chr06*	25850673	A/G	Gene_Ca_24011	NON_SYNONYMOUS_CODING	NA
Kabuli_wild_25850736	1399940518	*Ca_kabuli_Chr06*	25850736	C/T	Gene_Ca_24011	NON_SYNONYMOUS_CODING	NA
Kabuli_wild_25850759	1399940519	*Ca_kabuli_Chr06*	25850759	C/A	Gene_Ca_24011	NON_SYNONYMOUS_CODING	NA
Kabuli_wild_25850768	1399940520	*Ca_kabuli_Chr06*	25850768	G/A	Gene_Ca_24011	NON_SYNONYMOUS_CODING	NA
Kabuli_wild_25850773	1399940521	*Ca_kabuli_Chr06*	25850773	A/G	Gene_Ca_24011	SYNONYMOUS_CODING	NA
Kabuli_wild_25850777	1399940522	*Ca_kabuli_Chr06*	25850777	G/A	Gene_Ca_24011	NON_SYNONYMOUS_CODING	NA
Kabuli_wild_25850780	1399940523	*Ca_kabuli_Chr06*	25850780	G/A	Gene_Ca_24011	NON_SYNONYMOUS_CODING	NA
Kabuli_wild_25850783	1399940524	*Ca_kabuli_Chr06*	25850783	G/A	Gene_Ca_24011	NON_SYNONYMOUS_CODING	NA
Kabuli_wild_25850813	1399940525	*Ca_kabuli_Chr06*	25850813	C/T	Gene_Ca_24011	NON_SYNONYMOUS_CODING	NA
Kabuli_wild_25850816	1399940526	*Ca_kabuli_Chr06*	25850816	C/A	Gene_Ca_24011	NON_SYNONYMOUS_CODING	NA
Kabuli_wild_25850827	1399940527	*Ca_kabuli_Chr06*	25850827	C/T	Gene_Ca_24011	SYNONYMOUS_CODING	NA
Kabuli_wild_25850866	1399940528	*Ca_kabuli_Chr06*	25850866	C/G	Gene_Ca_24011	SYNONYMOUS_CODING	NA
Kabuli_wild_26349434	1399940529	*Ca_kabuli_Chr06*	26349434	G/A	NA	INTERGENIC	NA
Kabuli_wild_26349451	1399940530	*Ca_kabuli_Chr06*	26349451	T/C	NA	INTERGENIC	NA
Kabuli_wild_26353512	1399940531	*Ca_kabuli_Chr06*	26353512	C/T	NA	INTERGENIC	NA
Kabuli_wild_26353740	1399940532	*Ca_kabuli_Chr06*	26353740	G/C	NA	INTERGENIC	NA
Kabuli_wild_26357149	1399940533	*Ca_kabuli_Chr06*	26357149	C/G	NA	INTERGENIC	NA
Kabuli_wild_26360367	1399940534	*Ca_kabuli_Chr06*	26360367	C/A	Gene_Ca_16679	SYNONYMOUS_CODING	Oxysterol-bindingprotein
Kabuli_wild_26371255	1399940535	*Ca_kabuli_Chr06*	26371255	G/C	NA	INTERGENIC	NA
Kabuli_wild_26383440	1399940536	*Ca_kabuli_Chr06*	26383440	C/A	NA	INTERGENIC	NA
Kabuli_wild_26383500	1399940537	*Ca_kabuli_Chr06*	26383500	G/C	NA	INTERGENIC	NA
Kabuli_wild_26385432	1399940538	*Ca_kabuli_Chr06*	26385432	T/C	NA	INTERGENIC	NA
Kabuli_wild_26426239	1399940539	*Ca_kabuli_Chr06*	26426239	C/A	NA	INTERGENIC	NA
Kabuli_wild_26426252	1399940540	*Ca_kabuli_Chr06*	26426252	A/G	NA	INTERGENIC	NA
Kabuli_wild_26426283	1399940541	*Ca_kabuli_Chr06*	26426283	G/A	NA	INTERGENIC	NA
Kabuli_wild_26439443	1399940542	*Ca_kabuli_Chr06*	26439443	C/T	NA	INTERGENIC	NA
Kabuli_wild_26439474	1399940543	*Ca_kabuli_Chr06*	26439474	C/T	NA	INTERGENIC	NA
Kabuli_wild_26527161	1399940544	*Ca_kabuli_Chr06*	26527161	C/G	Gene_Ca_16688	INTRON	Zinc finger, RING-type
Kabuli_wild_26549168	1399940545	*Ca_kabuli_Chr06*	26549168	T/G	Gene_Ca_16689	INTRON	Armadillo
Kabuli_wild_26551586	1399940546	*Ca_kabuli_Chr06*	26551586	C/A	Gene_Ca_16689	SYNONYMOUS_CODING	Armadillo
Kabuli_wild_26559728	1399940547	*Ca_kabuli_Chr06*	26559728	G/A	NA	INTERGENIC	NA
Kabuli_wild_26567360	1399940548	*Ca_kabuli_Chr06*	26567360	A/G	Gene_Ca_16690	SYNONYMOUS_CODING	Zinc finger,Dof-type
Kabuli_wild_26616292	1399940549	*Ca_kabuli_Chr06*	26616292	G/T	Gene_Ca_16692	NON_SYNONYMOUS_CODING	NA
Kabuli_wild_26616317	1399940550	*Ca_kabuli_Chr06*	26616317	A/G	Gene_Ca_16692	INTRON	NA
Kabuli_wild_26698265	1399940551	*Ca_kabuli_Chr06*	26698265	A/C	Gene_Ca_16698	SYNONYMOUS_CODING	Cellular retinaldehyde-binding/triplefunction, C-terminal
Kabuli_wild_26769087	1399940552	*Ca_kabuli_Chr06*	26769087	C/A	Gene_Ca_16707	NON_SYNONYMOUS_CODING	RasGTPase
Kabuli_wild_26800873	1399940553	*Ca_kabuli_Chr06*	26800873	A/T	NA	INTERGENIC	NA
Kabuli_wild_26912205	1399940554	*Ca_kabuli_Chr06*	26912205	T/G	Gene_Ca_16724	UPSTREAM	Plantdiseaseresistanceresponseprotein
Kabuli_wild_27156217	1399940555	*Ca_kabuli_Chr06*	27156217	T/G	Gene_Ca_14630	INTRON	CCTdomain
Kabuli_wild_27310315	1399940556	*Ca_kabuli_Chr06*	27310315	C/T	Gene_Ca_14618	INTRON	Pathogenesis-related transcriptional factor/ERF, DNA-binding
Kabuli_wild_27310316	1399940557	*Ca_kabuli_Chr06*	27310316	A/T	Gene_Ca_14618	INTRON	Pathogenesis-related transcriptional factor/ERF, DNA-binding
Kabuli_wild_27310325	1399940558	*Ca_kabuli_Chr06*	27310325	G/T	Gene_Ca_14618	INTRON	Pathogenesis-related transcriptional factor/ERF, DNA-binding
Kabuli_wild_27310327	1399940559	*Ca_kabuli_Chr06*	27310327	A/T	Gene_Ca_14618	INTRON	Pathogenesis-related transcriptional factor/ERF, DNA-binding

SNP IDs	NCBI dbSNP accession IDs (NCBI_SS#)	Chromosomes	Physical positions (bp)	SNPs	Gene accession IDs	Sequence components of genome	Putative functions
Kabuli_wild_27310328	1399940560	*Ca_kabuli_Chr06*	27310328	T/A	Gene_Ca_14618	INTRON	Pathogenesis-related transcriptional factor/ERF, DNA-binding
Kabuli_wild_27310333	1399940561	*Ca_kabuli_Chr06*	27310333	C/T	Gene_Ca_14618	INTRON	Pathogenesis-related transcriptional factor/ERF, DNA-binding
Kabuli_wild_27310336	1399940562	*Ca_kabuli_Chr06*	27310336	A/T	Gene_Ca_14618	INTRON	Pathogenesis-related transcriptional factor/ERF, DNA-binding
Kabuli_wild_27310341	1399940563	*Ca_kabuli_Chr06*	27310341	G/T	Gene_Ca_14618	INTRON	Pathogenesis-related transcriptional factor/ERF, DNA-binding
Kabuli_wild_27310342	1399940564	*Ca_kabuli_Chr06*	27310342	A/T	Gene_Ca_14618	INTRON	Pathogenesis-related transcriptional factor/ERF, DNA-binding
Kabuli_wild_27310358	1399940565	*Ca_kabuli_Chr06*	27310358	A/T	Gene_Ca_14618	INTRON	Pathogenesis-related transcriptional factor/ERF, DNA-binding
Kabuli_wild_27310360	1399940566	*Ca_kabuli_Chr06*	27310360	C/T	Gene_Ca_14618	INTRON	Pathogenesis-related transcriptional factor/ERF, DNA-binding
Kabuli_wild_27312785	1399940567	*Ca_kabuli_Chr06*	27312785	A/C	Gene_Ca_14618	SYNONYMOUS_CODING	Pathogenesis-related transcriptional factor/ERF, DNA-binding
Kabuli_wild_27331165	1399940568	*Ca_kabuli_Chr06*	27331165	T/C	Gene_Ca_14617	NON_SYNONYMOUS_CODING	RNA recognition motif domain
Kabuli_wild_27383744	1399940569	*Ca_kabuli_Chr06*	27383744	G/T	NA	INTERGENIC	NA
Kabuli_wild_27383745	1399940570	*Ca_kabuli_Chr06*	27383745	G/T	NA	INTERGENIC	NA
Kabuli_wild_27383746	1399940571	*Ca_kabuli_Chr06*	27383746	A/T	NA	INTERGENIC	NA
Kabuli_wild_27515293	1399940572	*Ca_kabuli_Chr06*	27515293	G/A	Gene_Ca_14606	SYNONYMOUS_CODING	Tetraspanin,subgroup
Kabuli_wild_27536125	1399940573	*Ca_kabuli_Chr06*	27536125	T/C	Gene_Ca_14604	INTRON	Zinc finger, RING-type
Kabuli_wild_27652790	1399940574	*Ca_kabuli_Chr06*	27652790	A/G	Gene_Ca_14592	DOWNSTREAM	CytochromeB561-related
Kabuli_wild_27655605	1399940575	*Ca_kabuli_Chr06*	27655605	A/G	Gene_Ca_14592	NON_SYNONYMOUS_CODING	CytochromeB561-related
Kabuli_wild_27710401	1399940576	*Ca_kabuli_Chr06*	27710401	G/T	Gene_Ca_14588	UPSTREAM	PreproteintranslocaseSec,Sec61-betasubunit
Kabuli_wild_27710528	1399940577	*Ca_kabuli_Chr06*	27710528	T/G	Gene_Ca_14589	UPSTREAM	NA
Kabuli_wild_27710531	1399940578	*Ca_kabuli_Chr06*	27710531	T/G	Gene_Ca_14589	UPSTREAM	NA
Kabuli_wild_27717504	1399940579	*Ca_kabuli_Chr06*	27717504	C/G	Gene_Ca_14587	NON_SYNONYMOUS_CODING	Zinc finger,C2H2-type
Kabuli_wild_27787738	1399940580	*Ca_kabuli_Chr06*	27787738	T/C	NA	INTERGENIC	NA
Kabuli_wild_27986719	1399940581	*Ca_kabuli_Chr06*	27986719	A/G	Gene_Ca_14567	NON_SYNONYMOUS_CODING	RibosomalproteinL11
Kabuli_wild_28021899	1399940582	*Ca_kabuli_Chr06*	28021899	C/T	Gene_Ca_14563	NON_SYNONYMOUS_CODING	DNA-directedDNApolymerase,familyB,exonucleasedomain
Kabuli_wild_28044830	1399940583	*Ca_kabuli_Chr06*	28044830	C/A	Gene_Ca_14563	NON_SYNONYMOUS_CODING	DNA-directedDNApolymerase,familyB,exonucleasedomain
Kabuli_wild_28052463	1399940584	*Ca_kabuli_Chr06*	28052463	T/G	Gene_Ca_14562	SYNONYMOUS_CODING	Vacuola rfusion protein MON1
Kabuli_wild_28177387	1399940585	*Ca_kabuli_Chr06*	28177387	C/T	Gene_Ca_26122	INTRON	SIT4phosphatase-associatedproteinfamily
Kabuli_wild_28261211	1399940586	*Ca_kabuli_Chr06*	28261211	C/A	Gene_Ca_26119	INTRON	Heparansulphate6-sulfotransferase
Kabuli_wild_28284375	1399940587	*Ca_kabuli_Chr06*	28284375	T/C	NA	INTERGENIC	NA
Kabuli_wild_28325392	1399940588	*Ca_kabuli_Chr06*	28325392	C/T	Gene_Ca_25968	NON_SYNONYMOUS_CODING	RibonucleaseT2
Kabuli_wild_28372130	1399940589	*Ca_kabuli_Chr06*	28372130	C/T	NA	INTERGENIC	NA
Kabuli_wild_28518596	1399940590	*Ca_kabuli_Chr06*	28518596	C/A	NA	INTERGENIC	NA
Kabuli_wild_28518597	1399940591	*Ca_kabuli_Chr06*	28518597	A/T	NA	INTERGENIC	NA
Kabuli_wild_28518603	1399940592	*Ca_kabuli_Chr06*	28518603	G/A	NA	INTERGENIC	NA
Kabuli_wild_28728293	1399940593	*Ca_kabuli_Chr06*	28728293	A/C	Gene_Ca_17478	NON_SYNONYMOUS_CODING	Chaperonin Cpn60
Kabuli_wild_28755308	1399940594	*Ca_kabuli_Chr06*	28755308	A/T	Gene_Ca_17477	INTRON	NA
Kabuli_wild_28755310	1399940595	*Ca_kabuli_Chr06*	28755310	C/T	Gene_Ca_17477	INTRON	NA
Kabuli_wild_28757139	1399940596	*Ca_kabuli_Chr06*	28757139	C/A	Gene_Ca_17477	INTRON	NA
Kabuli_wild_28757204	1399940597	*Ca_kabuli_Chr06*	28757204	G/A	Gene_Ca_17477	INTRON	NA
Kabuli_wild_28757208	1399940598	*Ca_kabuli_Chr06*	28757208	C/A	Gene_Ca_17477	INTRON	NA
Kabuli_wild_28887188	1399940599	*Ca_kabuli_Chr06*	28887188	G/T	NA	INTERGENIC	NA
Kabuli_wild_28947529	1399940600	*Ca_kabuli_Chr06*	28947529	A/C	Gene_Ca_17471	NON_SYNONYMOUS_CODING	Glyoxalase/bleomycin resistance protein/dioxygenase
Kabuli_wild_29085558	1399940601	*Ca_kabuli_Chr06*	29085558	T/C	NA	INTERGENIC	NA
Kabuli_wild_29163022	1399940602	*Ca_kabuli_Chr06*	29163022	G/C	NA	INTERGENIC	NA
Kabuli_wild_29163640	1399940603	*Ca_kabuli_Chr06*	29163640	A/C	NA	INTERGENIC	NA

SNP IDs	NCBI dbSNP accession IDs (NCBI_SS#)	Chromosomes	Physical positions (bp)	SNPs	Gene accession IDs	Sequence components of genome	Putative functions
Kabuli_wild_29163642	1399940604	*Ca_kabuli_Chr06*	29163642	G/A	NA	INTERGENIC	NA
Kabuli_wild_29163667	1399940605	*Ca_kabuli_Chr06*	29163667	C/A	NA	INTERGENIC	NA
Kabuli_wild_29205260	1399940606	*Ca_kabuli_Chr06*	29205260	A/G	Gene_Ca_17457	INTRON	NA
Kabuli_wild_29205940	1399940607	*Ca_kabuli_Chr06*	29205940	A/G	Gene_Ca_17457	NON_SYNONYMOUS_CODING	DNAligase,ATP-dependent,N-terminal
Kabuli_wild_29485220	1399940608	*Ca_kabuli_Chr06*	29485220	C/T	NA	INTERGENIC	NA
Kabuli_wild_29495856	1399940609	*Ca_kabuli_Chr06*	29495856	C/T	Gene_Ca_17443	SYNONYMOUS_CODING	NA
Kabuli_wild_30048584	1399940610	*Ca_kabuli_Chr06*	30048584	A/G	NA	INTERGENIC	NA
Kabuli_wild_30048627	1399940611	*Ca_kabuli_Chr06*	30048627	C/A	NA	INTERGENIC	NA
Kabuli_wild_30073799	1399940612	*Ca_kabuli_Chr06*	30073799	C/T	Gene_Ca_24051	SYNONYMOUS_CODING	NA
Kabuli_wild_30257284	1399940613	*Ca_kabuli_Chr06*	30257284	A/C	Gene_Ca_16442	INTRON	Peptidase C48, SUMO/Sentrin/Ubl1
Kabuli_wild_30676031	1399940614	*Ca_kabuli_Chr06*	30676031	G/T	Gene_Ca_16465	SYNONYMOUS_CODING	Translation elongation factor EFG/EF2, C-terminal
Kabuli_wild_30676153	1399940615	*Ca_kabuli_Chr06*	30676153	A/G	Gene_Ca_16465	NON_SYNONYMOUS_CODING	Translation elongation factor EFG/EF2, C-terminal
Kabuli_wild_30676248	1399940616	*Ca_kabuli_Chr06*	30676248	C/T	Gene_Ca_16465	SYNONYMOUS_CODING	Translation elongation factor EFG/EF2, C-terminal
Kabuli_wild_30756237	1399940617	*Ca_kabuli_Chr06*	30756237	A/T	NA	INTERGENIC	NA
Kabuli_wild_30756238	1399940618	*Ca_kabuli_Chr06*	30756238	T/G	NA	INTERGENIC	NA
Kabuli_wild_30756242	1399940619	*Ca_kabuli_Chr06*	30756242	A/C	NA	INTERGENIC	NA
Kabuli_wild_30926084	1399940620	*Ca_kabuli_Chr06*	30926084	G/A	Gene_Ca_16485	NON_SYNONYMOUS_CODING	NA
Kabuli_wild_30927221	1399940621	*Ca_kabuli_Chr06*	30927221	C/G	Gene_Ca_16485	NON_SYNONYMOUS_CODING	NA
Kabuli_wild_31024935	1399940622	*Ca_kabuli_Chr06*	31024935	A/C	Gene_Ca_16493	NON_SYNONYMOUS_CODING	Zinc finger,Sec23/Sec24-type
Kabuli_wild_31048949	1399940623	*Ca_kabuli_Chr06*	31048949	T/C	Gene_Ca_16497	SYNONYMOUS_CODING	Late embryo genesis abundant protein, group2
Kabuli_wild_31049015	1399940624	*Ca_kabuli_Chr06*	31049015	A/C	Gene_Ca_16497	NON_SYNONYMOUS_CODING	Late embryo genesis abundant protein, group2
Kabuli_wild_31057806	1399940625	*Ca_kabuli_Chr06*	31057806	C/T	NA	INTERGENIC	NA
Kabuli_wild_31324962	1399940626	*Ca_kabuli_Chr06*	31324962	A/C	NA	INTERGENIC	NA
Kabuli_wild_31324976	1399940627	*Ca_kabuli_Chr06*	31324976	A/G	NA	INTERGENIC	NA
Kabuli_wild_31356463	1399940628	*Ca_kabuli_Chr06*	31356463	C/T	NA	INTERGENIC	NA
Kabuli_wild_31717567	1399940629	*Ca_kabuli_Chr06*	31717567	G/A	NA	INTERGENIC	NA
Kabuli_wild_31717574	1399940630	*Ca_kabuli_Chr06*	31717574	T/C	NA	INTERGENIC	NA
Kabuli_wild_31719586	1399940631	*Ca_kabuli_Chr06*	31719586	C/A	NA	INTERGENIC	NA
Kabuli_wild_31719620	1399940632	*Ca_kabuli_Chr06*	31719620	A/C	NA	INTERGENIC	NA
Kabuli_wild_31747956	1399940633	*Ca_kabuli_Chr06*	31747956	C/T	Gene_Ca_15193	DOWNSTREAM	NA
Kabuli_wild_31747964	1399940634	*Ca_kabuli_Chr06*	31747964	G/C	Gene_Ca_15193	DOWNSTREAM	NA
Kabuli_wild_31747979	1399940635	*Ca_kabuli_Chr06*	31747979	A/G	Gene_Ca_15193	DOWNSTREAM	NA
Kabuli_wild_31747986	1399940636	*Ca_kabuli_Chr06*	31747986	T/G	Gene_Ca_15193	DOWNSTREAM	NA
Kabuli_wild_31748011	1399940637	*Ca_kabuli_Chr06*	31748011	C/T	Gene_Ca_15193	DOWNSTREAM	NA
Kabuli_wild_31748022	1399940638	*Ca_kabuli_Chr06*	31748022	G/T	Gene_Ca_15193	DOWNSTREAM	NA
Kabuli_wild_31895264	1399940639	*Ca_kabuli_Chr06*	31895264	C/T	Gene_Ca_15200	INTRON	Metallo-dependentphosphatase
Kabuli_wild_31900781	1399940640	*Ca_kabuli_Chr06*	31900781	G/T	Gene_Ca_15200	INTRON	Metallo-dependentphosphatase
Kabuli_wild_31900785	1399940641	*Ca_kabuli_Chr06*	31900785	T/G	Gene_Ca_15200	INTRON	Metallo-dependentphosphatase
Kabuli_wild_31900790	1399940642	*Ca_kabuli_Chr06*	31900790	A/C	Gene_Ca_15200	INTRON	Metallo-dependentphosphatase
Kabuli_wild_31902063	1399940643	*Ca_kabuli_Chr06*	31902063	A/C	Gene_Ca_15200	SYNONYMOUS_CODING	Metallo-dependentphosphatase
Kabuli_wild_31992907	1399940644	*Ca_kabuli_Chr06*	31992907	A/G	Gene_Ca_15204	INTRON	NA
Kabuli_wild_32048545	1399940645	*Ca_kabuli_Chr06*	32048545	C/T	Gene_Ca_15208	INTRON	MD-2-relatedlipid-recognition
Kabuli_wild_32048566	1399940646	*Ca_kabuli_Chr06*	32048566	C/T	Gene_Ca_15208	INTRON	MD-2-relatedlipid-recognition
Kabuli_wild_32140428	1399940647	*Ca_kabuli_Chr06*	32140428	C/A	Gene_Ca_15214	NON_SYNONYMOUS_CODING	BRCT

SNP IDs	NCBI dbSNP accession IDs (NCBI_SS#)	Chromosomes	Physical positions (bp)	SNPs	Gene accession IDs	Sequence components of genome	Putative functions
Kabuli_wild_32140429	1399940648	*Ca_kabuli_Chr06*	32140429	G/T	Gene_Ca_15214	SYNONYMOUS_CODING	BRCT
Kabuli_wild_32201419	1399940649	*Ca_kabuli_Chr06*	32201419	A/C	Gene_Ca_15217	NON_SYNONYMOUS_CODING	NA
Kabuli_wild_32261594	1399940650	*Ca_kabuli_Chr06*	32261594	A/C	NA	INTERGENIC	NA
Kabuli_wild_32262807	1399940651	*Ca_kabuli_Chr06*	32262807	T/C	NA	INTERGENIC	NA
Kabuli_wild_32262855	1399940652	*Ca_kabuli_Chr06*	32262855	C/T	NA	INTERGENIC	NA
Kabuli_wild_32328468	1399940653	*Ca_kabuli_Chr06*	32328468	T/C	Gene_Ca_15224	NON_SYNONYMOUS_CODING	DNA-binding WRKY
Kabuli_wild_32328749	1399940654	*Ca_kabuli_Chr06*	32328749	A/C	Gene_Ca_15224	SYNONYMOUS_CODING	DNA-binding WRKY
Kabuli_wild_32682315	1399940655	*Ca_kabuli_Chr06*	32682315	C/A	Gene_Ca_22218	INTRON	Protease-associateddomain,PA
Kabuli_wild_32682316	1399940656	*Ca_kabuli_Chr06*	32682316	G/T	Gene_Ca_22218	INTRON	Protease-associateddomain,PA
Kabuli_wild_32717450	1399940657	*Ca_kabuli_Chr06*	32717450	G/A	NA	INTERGENIC	NA
Kabuli_wild_32717665	1399940658	*Ca_kabuli_Chr06*	32717665	C/A	NA	INTERGENIC	NA
Kabuli_wild_32825547	1399940659	*Ca_kabuli_Chr06*	32825547	A/G	NA	INTERGENIC	NA
Kabuli_wild_32825556	1399940660	*Ca_kabuli_Chr06*	32825556	C/A	NA	INTERGENIC	NA
Kabuli_wild_32825561	1399940661	*Ca_kabuli_Chr06*	32825561	C/T	NA	INTERGENIC	NA
Kabuli_wild_32825567	1399940662	*Ca_kabuli_Chr06*	32825567	C/A	NA	INTERGENIC	NA
Kabuli_wild_32825576	1399940663	*Ca_kabuli_Chr06*	32825576	G/C	NA	INTERGENIC	NA
Kabuli_wild_32825587	1399940664	*Ca_kabuli_Chr06*	32825587	G/A	NA	INTERGENIC	NA
Kabuli_wild_32825619	1399940665	*Ca_kabuli_Chr06*	32825619	T/C	NA	INTERGENIC	NA
Kabuli_wild_32825623	1399940666	*Ca_kabuli_Chr06*	32825623	G/A	NA	INTERGENIC	NA
Kabuli_wild_32825631	1399940667	*Ca_kabuli_Chr06*	32825631	C/A	NA	INTERGENIC	NA
Kabuli_wild_32825634	1399940668	*Ca_kabuli_Chr06*	32825634	C/T	NA	INTERGENIC	NA
Kabuli_wild_32914927	1399940669	*Ca_kabuli_Chr06*	32914927	G/C	Gene_Ca_22229	INTRON	PeptidaseM48
Kabuli_wild_33039319	1399940670	*Ca_kabuli_Chr06*	33039319	C/T	NA	INTERGENIC	NA
Kabuli_wild_33039370	1399940671	*Ca_kabuli_Chr06*	33039370	G/A	NA	INTERGENIC	NA
Kabuli_wild_33039394	1399940672	*Ca_kabuli_Chr06*	33039394	T/C	NA	INTERGENIC	NA
Kabuli_wild_33068889	1399940673	*Ca_kabuli_Chr06*	33068889	A/G	NA	INTERGENIC	NA
Kabuli_wild_33068917	1399940674	*Ca_kabuli_Chr06*	33068917	G/C	NA	INTERGENIC	NA
Kabuli_wild_33068924	1399940675	*Ca_kabuli_Chr06*	33068924	C/A	NA	INTERGENIC	NA
Kabuli_wild_33229649	1399940676	*Ca_kabuli_Chr06*	33229649	A/G	NA	INTERGENIC	NA
Kabuli_wild_33502994	1399940677	*Ca_kabuli_Chr06*	33502994	C/A	NA	INTERGENIC	NA
Kabuli_wild_33503007	1399940678	*Ca_kabuli_Chr06*	33503007	T/C	NA	INTERGENIC	NA
Kabuli_wild_33503018	1399940679	*Ca_kabuli_Chr06*	33503018	C/T	NA	INTERGENIC	NA
Kabuli_wild_33503028	1399940680	*Ca_kabuli_Chr06*	33503028	C/T	NA	INTERGENIC	NA
Kabuli_wild_33503032	1399940681	*Ca_kabuli_Chr06*	33503032	A/C	NA	INTERGENIC	NA
Kabuli_wild_33503051	1399940682	*Ca_kabuli_Chr06*	33503051	G/C	NA	INTERGENIC	NA
Kabuli_wild_33503057	1399940683	*Ca_kabuli_Chr06*	33503057	A/T	NA	INTERGENIC	NA
Kabuli_wild_33503063	1399940684	*Ca_kabuli_Chr06*	33503063	C/G	NA	INTERGENIC	NA
Kabuli_wild_33503065	1399940685	*Ca_kabuli_Chr06*	33503065	C/A	NA	INTERGENIC	NA
Kabuli_wild_33503071	1399940686	*Ca_kabuli_Chr06*	33503071	A/G	NA	INTERGENIC	NA
Kabuli_wild_33598926	1399940687	*Ca_kabuli_Chr06*	33598926	G/A	Gene_Ca_22069	INTRON	NA
Kabuli_wild_33704792	1399940688	*Ca_kabuli_Chr06*	33704792	C/T	NA	INTERGENIC	NA
Kabuli_wild_33704807	1399940689	*Ca_kabuli_Chr06*	33704807	C/A	NA	INTERGENIC	NA
Kabuli_wild_33704820	1399940690	*Ca_kabuli_Chr06*	33704820	T/C	NA	INTERGENIC	NA
Kabuli_wild_33733252	1399940691	*Ca_kabuli_Chr06*	33733252	T/G	Gene_Ca_22063	NON_SYNONYMOUS_CODING	Zinc finger, RING-type

SNP IDs	NCBI dbSNP accession IDs (NCBI_SS#)	Chromosomes	Physical positions (bp)	SNPs	Gene accession IDs	Sequence components of genome	Putative functions
Kabuli_wild_33733259	1399940692	*Ca_kabuli_Chr06*	33733259	A/G	Gene_Ca_22063	SYNONYMOUS_CODING	Zinc finger, RING-type
Kabuli_wild_34247329	1399940693	*Ca_kabuli_Chr06*	34247329	A/C	NA	INTERGENIC	NA
Kabuli_wild_34407309	1399940694	*Ca_kabuli_Chr06*	34407309	T/C	NA	INTERGENIC	NA
Kabuli_wild_34481256	1399940695	*Ca_kabuli_Chr06*	34481256	C/T	Gene_Ca_15818	INTRON	NA
Kabuli_wild_34481266	1399940696	*Ca_kabuli_Chr06*	34481266	A/G	Gene_Ca_15818	INTRON	NA
Kabuli_wild_34596792	1399940697	*Ca_kabuli_Chr06*	34596792	A/G	NA	INTERGENIC	NA
Kabuli_wild_34744433	1399940698	*Ca_kabuli_Chr06*	34744433	A/G	NA	INTERGENIC	NA
Kabuli_wild_34744438	1399940699	*Ca_kabuli_Chr06*	34744438	T/G	NA	INTERGENIC	NA
Kabuli_wild_34744480	1399940700	*Ca_kabuli_Chr06*	34744480	C/T	NA	INTERGENIC	NA
Kabuli_wild_34744665	1399940701	*Ca_kabuli_Chr06*	34744665	G/A	NA	INTERGENIC	NA
Kabuli_wild_34744670	1399940702	*Ca_kabuli_Chr06*	34744670	A/G	NA	INTERGENIC	NA
Kabuli_wild_34744681	1399940703	*Ca_kabuli_Chr06*	34744681	T/G	NA	INTERGENIC	NA
Kabuli_wild_34771344	1399940704	*Ca_kabuli_Chr06*	34771344	A/C	Gene_Ca_15807	SYNONYMOUS_CODING	NA
Kabuli_wild_34773450	1399940705	*Ca_kabuli_Chr06*	34773450	C/A	Gene_Ca_15807	INTRON	NA
Kabuli_wild_34773517	1399940706	*Ca_kabuli_Chr06*	34773517	C/T	Gene_Ca_15807	INTRON	NA
Kabuli_wild_35129588	1399940707	*Ca_kabuli_Chr06*	35129588	G/A	Gene_Ca_15788	SYNONYMOUS_CODING	Zinc finger,PHD-type
Kabuli_wild_35596064	1399940708	*Ca_kabuli_Chr06*	35596064	T/C	NA	INTERGENIC	NA
Kabuli_wild_35596070	1399940709	*Ca_kabuli_Chr06*	35596070	G/A	NA	INTERGENIC	NA
Kabuli_wild_35596161	1399940710	*Ca_kabuli_Chr06*	35596161	C/T	NA	INTERGENIC	NA
Kabuli_wild_35596184	1399940711	*Ca_kabuli_Chr06*	35596184	A/G	NA	INTERGENIC	NA
Kabuli_wild_35596195	1399940712	*Ca_kabuli_Chr06*	35596195	G/C	NA	INTERGENIC	NA
Kabuli_wild_35596197	1399940713	*Ca_kabuli_Chr06*	35596197	G/A	NA	INTERGENIC	NA
Kabuli_wild_35596207	1399940714	*Ca_kabuli_Chr06*	35596207	G/T	NA	INTERGENIC	NA
Kabuli_wild_35596218	1399940715	*Ca_kabuli_Chr06*	35596218	T/C	NA	INTERGENIC	NA
Kabuli_wild_35596220	1399940716	*Ca_kabuli_Chr06*	35596220	G/A	NA	INTERGENIC	NA
Kabuli_wild_35596230	1399940717	*Ca_kabuli_Chr06*	35596230	T/A	NA	INTERGENIC	NA
Kabuli_wild_35962309	1399940718	*Ca_kabuli_Chr06*	35962309	C/T	NA	INTERGENIC	NA
Kabuli_wild_35962320	1399940719	*Ca_kabuli_Chr06*	35962320	T/C	NA	INTERGENIC	NA
Kabuli_wild_35962360	1399940720	*Ca_kabuli_Chr06*	35962360	T/C	NA	INTERGENIC	NA
Kabuli_wild_35962364	1399940721	*Ca_kabuli_Chr06*	35962364	T/G	NA	INTERGENIC	NA
Kabuli_wild_35962365	1399940722	*Ca_kabuli_Chr06*	35962365	T/C	NA	INTERGENIC	NA
Kabuli_wild_35962366	1399940723	*Ca_kabuli_Chr06*	35962366	G/A	NA	INTERGENIC	NA
Kabuli_wild_36228879	1399940724	*Ca_kabuli_Chr06*	36228879	A/G	Gene_Ca_23958	INTRON	Peptidyl-prolylcis-transisomerase,FKBP-type,domain
Kabuli_wild_36378706	1399940725	*Ca_kabuli_Chr06*	36378706	A/G	Gene_Ca_23965	INTRON	UvrABCcomplex,subunitB
Kabuli_wild_36411933	1399940726	*Ca_kabuli_Chr06*	36411933	G/A	NA	INTERGENIC	NA
Kabuli_wild_36574836	1399940727	*Ca_kabuli_Chr06*	36574836	A/G	NA	INTERGENIC	NA
Kabuli_wild_36774699	1399940728	*Ca_kabuli_Chr06*	36774699	G/T	NA	INTERGENIC	NA
Kabuli_wild_36774702	1399940729	*Ca_kabuli_Chr06*	36774702	A/G	NA	INTERGENIC	NA
Kabuli_wild_36774717	1399940730	*Ca_kabuli_Chr06*	36774717	T/C	NA	INTERGENIC	NA
Kabuli_wild_36774718	1399940731	*Ca_kabuli_Chr06*	36774718	C/G	NA	INTERGENIC	NA
Kabuli_wild_36790428	1399940732	*Ca_kabuli_Chr06*	36790428	G/A	Gene_Ca_19812	NON_SYNONYMOUS_CODING	NA
Kabuli_wild_36790441	1399940733	*Ca_kabuli_Chr06*	36790441	C/A	Gene_Ca_19812	NON_SYNONYMOUS_CODING	NA
Kabuli_wild_36790455	1399940734	*Ca_kabuli_Chr06*	36790455	C/T	Gene_Ca_19812	NON_SYNONYMOUS_CODING	NA
Kabuli_wild_36790458	1399940735	*Ca_kabuli_Chr06*	36790458	G/A	Gene_Ca_19812	NON_SYNONYMOUS_CODING	NA

SNP IDs	NCBI dbSNP accession IDs (NCBI_SS#)	Chromosomes	Physical positions (bp)	SNPs	Gene accession IDs	Sequence components of genome	Putative functions
Kabuli_wild_36790467	1399940736	*Ca_kabuli_Chr06*	36790467	G/A	Gene_Ca_19812	NON_SYNONYMOUS_CODING	NA
Kabuli_wild_36889465	1399940737	*Ca_kabuli_Chr06*	36889465	C/T	Gene_Ca_19808	NON_SYNONYMOUS_CODING	NA
Kabuli_wild_36892197	1399940738	*Ca_kabuli_Chr06*	36892197	G/A	NA	INTERGENIC	NA
Kabuli_wild_37139418	1399940739	*Ca_kabuli_Chr06*	37139418	C/T	Gene_Ca_19803	NON_SYNONYMOUS_CODING	Peptidase C48, SUMO/Sentrin/Ubl1
Kabuli_wild_37139420	1399940740	*Ca_kabuli_Chr06*	37139420	A/G	Gene_Ca_19803	NON_SYNONYMOUS_CODING	Peptidase C48, SUMO/Sentrin/Ubl1
Kabuli_wild_37139425	1399940741	*Ca_kabuli_Chr06*	37139425	C/T	Gene_Ca_19803	SYNONYMOUS_CODING	Peptidase C48, SUMO/Sentrin/Ubl1
Kabuli_wild_37139426	1399940742	*Ca_kabuli_Chr06*	37139426	C/T	Gene_Ca_19803	NON_SYNONYMOUS_CODING	Peptidase C48, SUMO/Sentrin/Ubl1
Kabuli_wild_37139453	1399940743	*Ca_kabuli_Chr06*	37139453	C/T	Gene_Ca_19803	NON_SYNONYMOUS_CODING	Peptidase C48, SUMO/Sentrin/Ubl1
Kabuli_wild_37139466	1399940744	*Ca_kabuli_Chr06*	37139466	G/C	Gene_Ca_19803	SYNONYMOUS_CODING	Peptidase C48, SUMO/Sentrin/Ubl1
Kabuli_wild_37139484	1399940745	*Ca_kabuli_Chr06*	37139484	T/C	Gene_Ca_19803	NON_SYNONYMOUS_CODING	Peptidase C48, SUMO/Sentrin/Ubl1
Kabuli_wild_37139499	1399940746	*Ca_kabuli_Chr06*	37139499	C/G	Gene_Ca_19803	NON_SYNONYMOUS_CODING	Peptidase C48, SUMO/Sentrin/Ubl1
Kabuli_wild_37139502	1399940747	*Ca_kabuli_Chr06*	37139502	C/G	Gene_Ca_19803	NON_SYNONYMOUS_CODING	Peptidase C48, SUMO/Sentrin/Ubl1
Kabuli_wild_37139514	1399940748	*Ca_kabuli_Chr06*	37139514	C/T	Gene_Ca_19803	NON_SYNONYMOUS_CODING	Peptidase C48, SUMO/Sentrin/Ubl1
Kabuli_wild_37139604	1399940749	*Ca_kabuli_Chr06*	37139604	G/T	Gene_Ca_19803	NON_SYNONYMOUS_CODING	Peptidase C48, SUMO/Sentrin/Ubl1
Kabuli_wild_37139610	1399940750	*Ca_kabuli_Chr06*	37139610	T/G	Gene_Ca_19803	NON_SYNONYMOUS_CODING	Peptidase C48, SUMO/Sentrin/Ubl1
Kabuli_wild_37221699	1399940751	*Ca_kabuli_Chr06*	37221699	C/G	NA	INTERGENIC	NA
Kabuli_wild_37476426	1399940752	*Ca_kabuli_Chr06*	37476426	C/T	NA	INTERGENIC	NA
Kabuli_wild_37476465	1399940753	*Ca_kabuli_Chr06*	37476465	G/A	NA	INTERGENIC	NA
Kabuli_wild_37476516	1399940754	*Ca_kabuli_Chr06*	37476516	G/T	NA	INTERGENIC	NA
Kabuli_wild_37484467	1399940755	*Ca_kabuli_Chr06*	37484467	C/T	NA	INTERGENIC	NA
Kabuli_wild_37484468	1399940756	*Ca_kabuli_Chr06*	37484468	G/T	NA	INTERGENIC	NA
Kabuli_wild_37484470	1399940757	*Ca_kabuli_Chr06*	37484470	C/A	NA	INTERGENIC	NA
Kabuli_wild_37484489	1399940758	*Ca_kabuli_Chr06*	37484489	C/G	NA	INTERGENIC	NA
Kabuli_wild_37484509	1399940759	*Ca_kabuli_Chr06*	37484509	T/C	NA	INTERGENIC	NA
Kabuli_wild_37484513	1399940760	*Ca_kabuli_Chr06*	37484513	T/C	NA	INTERGENIC	NA
Kabuli_wild_37692569	1399940761	*Ca_kabuli_Chr06*	37692569	C/A	NA	INTERGENIC	NA
Kabuli_wild_37943905	1399940762	*Ca_kabuli_Chr06*	37943905	C/T	Gene_Ca_19549	DOWNSTREAM	NA
Kabuli_wild_38136025	1399940763	*Ca_kabuli_Chr06*	38136025	A/G	NA	INTERGENIC	NA
Kabuli_wild_38194797	1399940764	*Ca_kabuli_Chr06*	38194797	C/T	Gene_Ca_19560	SYNONYMOUS_CODING	Transposase,Ptta/En/Spm,plant
Kabuli_wild_38194819	1399940765	*Ca_kabuli_Chr06*	38194819	G/T	Gene_Ca_19560	NON_SYNONYMOUS_CODING	Transposase,Ptta/En/Spm,plant
Kabuli_wild_38308827	1399940766	*Ca_kabuli_Chr06*	38308827	T/C	Gene_Ca_19565	INTRON	Neutral/alkalinenonlysosomalceramidase
Kabuli_wild_38514183	1399940767	*Ca_kabuli_Chr06*	38514183	A/C	NA	INTERGENIC	NA
Kabuli_wild_38816345	1399940768	*Ca_kabuli_Chr06*	38816345	C/T	Gene_Ca_15898	SYNONYMOUS_CODING	Transcription facto rGRAS
Kabuli_wild_38838320	1399940769	*Ca_kabuli_Chr06*	38838320	A/G	Gene_Ca_15899	NON_SYNONYMOUS_CODING	Calcium-bindingEF-hand
Kabuli_wild_38840273	1399940770	*Ca_kabuli_Chr06*	38840273	G/A	Gene_Ca_15899	DOWNSTREAM	Calcium-bindingEF-hand
Kabuli_wild_38840279	1399940771	*Ca_kabuli_Chr06*	38840279	G/A	Gene_Ca_15899	DOWNSTREAM	Calcium-bindingEF-hand
Kabuli_wild_38840286	1399940772	*Ca_kabuli_Chr06*	38840286	C/G	Gene_Ca_15899	DOWNSTREAM	Calcium-bindingEF-hand
Kabuli_wild_38840313	1399940773	*Ca_kabuli_Chr06*	38840313	C/T	Gene_Ca_15899	DOWNSTREAM	Calcium-bindingEF-hand
Kabuli_wild_38881909	1399940774	*Ca_kabuli_Chr06*	38881909	C/A	NA	INTERGENIC	NA
Kabuli_wild_38886735	1399940775	*Ca_kabuli_Chr06*	38886735	C/A	NA	INTERGENIC	NA
Kabuli_wild_38994879	1399940776	*Ca_kabuli_Chr06*	38994879	A/G	Gene_Ca_15903	NON_SYNONYMOUS_CODING	NA
Kabuli_wild_38994925	1399940777	*Ca_kabuli_Chr06*	38994925	G/T	Gene_Ca_15903	NON_SYNONYMOUS_CODING	NA
Kabuli_wild_38994954	1399940778	*Ca_kabuli_Chr06*	38994954	A/G	Gene_Ca_15903	NON_SYNONYMOUS_CODING	NA
Kabuli_wild_38994962	1399940779	*Ca_kabuli_Chr06*	38994962	G/A	Gene_Ca_15903	SYNONYMOUS_CODING	NA

SNP IDs	NCBI dbSNP accession IDs (NCBI_SS#)	Chromosomes	Physical positions (bp)	SNPs	Gene accession IDs	Sequence components of genome	Putative functions
Kabuli_wild_39107166	1399940780	*Ca_kabuli_Chr06*	39107166	A/G	NA	INTERGENIC	NA
Kabuli_wild_39107177	1399940781	*Ca_kabuli_Chr06*	39107177	G/A	NA	INTERGENIC	NA
Kabuli_wild_39107187	1399940782	*Ca_kabuli_Chr06*	39107187	C/T	NA	INTERGENIC	NA
Kabuli_wild_39317016	1399940783	*Ca_kabuli_Chr06*	39317016	T/G	Gene_Ca_15914	INTRON	PAP/25A-associated
Kabuli_wild_39380625	1399940784	*Ca_kabuli_Chr06*	39380625	A/C	Gene_Ca_15918	INTRON	Resolvase,hollidayjunction-type,YqgF-like
Kabuli_wild_39505343	1399940785	*Ca_kabuli_Chr06*	39505343	T/A	NA	INTERGENIC	NA
Kabuli_wild_39724016	1399940786	*Ca_kabuli_Chr06*	39724016	A/C	NA	INTERGENIC	NA
Kabuli_wild_39724022	1399940787	*Ca_kabuli_Chr06*	39724022	A/G	NA	INTERGENIC	NA
Kabuli_wild_39863065	1399940788	*Ca_kabuli_Chr06*	39863065	C/T	NA	INTERGENIC	NA
Kabuli_wild_39863077	1399940789	*Ca_kabuli_Chr06*	39863077	C/T	NA	INTERGENIC	NA
Kabuli_wild_39863085	1399940790	*Ca_kabuli_Chr06*	39863085	C/T	NA	INTERGENIC	NA
Kabuli_wild_39864744	1399940791	*Ca_kabuli_Chr06*	39864744	A/G	NA	INTERGENIC	NA
Kabuli_wild_39864787	1399940792	*Ca_kabuli_Chr06*	39864787	C/A	NA	INTERGENIC	NA
Kabuli_wild_39864799	1399940793	*Ca_kabuli_Chr06*	39864799	C/T	NA	INTERGENIC	NA
Kabuli_wild_39908314	1399940794	*Ca_kabuli_Chr06*	39908314	T/G	NA	INTERGENIC	NA
Kabuli_wild_40047823	1399940795	*Ca_kabuli_Chr06*	40047823	G/A	Gene_Ca_15938	SYNONYMOUS_CODING	Chromodomain
Kabuli_wild_40047893	1399940796	*Ca_kabuli_Chr06*	40047893	C/T	Gene_Ca_15938	NON_SYNONYMOUS_CODING	Chromodomain
Kabuli_wild_40128644	1399940797	*Ca_kabuli_Chr06*	40128644	T/C	Gene_Ca_15942	UPSTREAM	PhosphotransferaseKptA/Tpt1
Kabuli_wild_40166834	1399940798	*Ca_kabuli_Chr06*	40166834	T/C	NA	INTERGENIC	NA
Kabuli_wild_40166840	1399940799	*Ca_kabuli_Chr06*	40166840	T/G	NA	INTERGENIC	NA
Kabuli_wild_40468356	1399940800	*Ca_kabuli_Chr06*	40468356	T/A	Gene_Ca_21602	INTRON	NA
Kabuli_wild_40477285	1399940801	*Ca_kabuli_Chr06*	40477285	G/A	Gene_Ca_21600	NON_SYNONYMOUS_CODING	Nitroreductase-like
Kabuli_wild_40657695	1399940802	*Ca_kabuli_Chr06*	40657695	A/C	Gene_Ca_21596	SYNONYMOUS_CODING	PeptidaseM22,glycoprotease
Kabuli_wild_40735186	1399940803	*Ca_kabuli_Chr06*	40735186	C/A	Gene_Ca_21592	UPSTREAM	DNArepairproteinRadA
Kabuli_wild_40767566	1399940804	*Ca_kabuli_Chr06*	40767566	A/G	NA	INTERGENIC	NA
Kabuli_wild_40821354	1399940805	*Ca_kabuli_Chr06*	40821354	C/T	Gene_Ca_21586	INTRON	Transketolase,N-terminal
Kabuli_wild_40924233	1399940806	*Ca_kabuli_Chr06*	40924233	A/G	Gene_Ca_21582	INTRON	ExocystcomplexcomponentSec6
Kabuli_wild_40935441	1399940807	*Ca_kabuli_Chr06*	40935441	A/T	Gene_Ca_21581	INTRON	ExocystcomplexcomponentSec6
Kabuli_wild_40947803	1399940808	*Ca_kabuli_Chr06*	40947803	C/A	Gene_Ca_21580	INTRON	ExocystcomplexcomponentSec6
Kabuli_wild_41099629	1399940809	*Ca_kabuli_Chr06*	41099629	A/G	NA	INTERGENIC	NA
Kabuli_wild_41099664	1399940810	*Ca_kabuli_Chr06*	41099664	G/A	NA	INTERGENIC	NA
Kabuli_wild_41099671	1399940811	*Ca_kabuli_Chr06*	41099671	A/G	NA	INTERGENIC	NA
Kabuli_wild_41121287	1399940812	*Ca_kabuli_Chr06*	41121287	G/A	Gene_Ca_23143	SYNONYMOUS_CODING	NA
Kabuli_wild_41121330	1399940813	*Ca_kabuli_Chr06*	41121330	G/A	Gene_Ca_23143	NON_SYNONYMOUS_CODING	NA
Kabuli_wild_41121378	1399940814	*Ca_kabuli_Chr06*	41121378	G/A	Gene_Ca_23143	NON_SYNONYMOUS_CODING	NA
Kabuli_wild_41121397	1399940815	*Ca_kabuli_Chr06*	41121397	G/A	Gene_Ca_23143	SYNONYMOUS_CODING	NA
Kabuli_wild_41121412	1399940816	*Ca_kabuli_Chr06*	41121412	C/T	Gene_Ca_23143	NON_SYNONYMOUS_CODING	NA
Kabuli_wild_41214997	1399940817	*Ca_kabuli_Chr06*	41214997	C/T	Gene_Ca_23146	DOWNSTREAM	NA
Kabuli_wild_41448947	1399940818	*Ca_kabuli_Chr06*	41448947	C/A	NA	INTERGENIC	NA
Kabuli_wild_41448969	1399940819	*Ca_kabuli_Chr06*	41448969	A/T	NA	INTERGENIC	NA
Kabuli_wild_41448988	1399940820	*Ca_kabuli_Chr06*	41448988	T/C	NA	INTERGENIC	NA
Kabuli_wild_41682943	1399940821	*Ca_kabuli_Chr06*	41682943	T/C	NA	INTERGENIC	NA
Kabuli_wild_41682966	1399940822	*Ca_kabuli_Chr06*	41682966	T/C	NA	INTERGENIC	NA
Kabuli_wild_41682983	1399940823	*Ca_kabuli_Chr06*	41682983	T/C	NA	INTERGENIC	NA

SNP IDs	NCBI dbSNP accession IDs (NCBI_SS#)	Chromosomes	Physical positions (bp)	SNPs	Gene accession IDs	Sequence components of genome	Putative functions
Kabuli_wild_41697812	1399940824	*Ca_kabuli_Chr06*	41697812	T/G	Gene_Ca_21940	INTRON	RibonucleaseII/R
Kabuli_wild_41708154	1399940825	*Ca_kabuli_Chr06*	41708154	T/G	NA	INTERGENIC	NA
Kabuli_wild_41708270	1399940826	*Ca_kabuli_Chr06*	41708270	G/C	NA	INTERGENIC	NA
Kabuli_wild_41708295	1399940827	*Ca_kabuli_Chr06*	41708295	G/C	NA	INTERGENIC	NA
Kabuli_wild_41708299	1399940828	*Ca_kabuli_Chr06*	41708299	C/T	NA	INTERGENIC	NA
Kabuli_wild_41708306	1399940829	*Ca_kabuli_Chr06*	41708306	G/A	NA	INTERGENIC	NA
Kabuli_wild_41708309	1399940830	*Ca_kabuli_Chr06*	41708309	G/A	NA	INTERGENIC	NA
Kabuli_wild_41708328	1399940831	*Ca_kabuli_Chr06*	41708328	A/G	NA	INTERGENIC	NA
Kabuli_wild_41708474	1399940832	*Ca_kabuli_Chr06*	41708474	G/A	NA	INTERGENIC	NA
Kabuli_wild_41708523	1399940833	*Ca_kabuli_Chr06*	41708523	T/G	NA	INTERGENIC	NA
Kabuli_wild_41708530	1399940834	*Ca_kabuli_Chr06*	41708530	C/A	NA	INTERGENIC	NA
Kabuli_wild_41907459	1399940835	*Ca_kabuli_Chr06*	41907459	C/G	NA	INTERGENIC	NA
Kabuli_wild_42004813	1399940836	*Ca_kabuli_Chr06*	42004813	C/T	Gene_Ca_21951	SYNONYMOUS_CODING	TGS
Kabuli_wild_42004818	1399940837	*Ca_kabuli_Chr06*	42004818	C/G	Gene_Ca_21951	SYNONYMOUS_CODING	TGS
Kabuli_wild_42004829	1399940838	*Ca_kabuli_Chr06*	42004829	T/G	Gene_Ca_21951	SYNONYMOUS_CODING	TGS
Kabuli_wild_42004842	1399940839	*Ca_kabuli_Chr06*	42004842	C/A	Gene_Ca_21951	NON_SYNONYMOUS_CODING	TGS
Kabuli_wild_42004851	1399940840	*Ca_kabuli_Chr06*	42004851	A/G	Gene_Ca_21951	NON_SYNONYMOUS_CODING	TGS
Kabuli_wild_42161857	1399940841	*Ca_kabuli_Chr06*	42161857	C/G	NA	INTERGENIC	NA
Kabuli_wild_42161866	1399940842	*Ca_kabuli_Chr06*	42161866	T/G	NA	INTERGENIC	NA
Kabuli_wild_42161887	1399940843	*Ca_kabuli_Chr06*	42161887	T/C	NA	INTERGENIC	NA
Kabuli_wild_42171905	1399940844	*Ca_kabuli_Chr06*	42171905	A/C	Gene_Ca_21956	SYNONYMOUS_CODING	NA
Kabuli_wild_42171916	1399940845	*Ca_kabuli_Chr06*	42171916	T/C	Gene_Ca_21956	SYNONYMOUS_CODING	NA
Kabuli_wild_42207407	1399940846	*Ca_kabuli_Chr06*	42207407	T/C	NA	INTERGENIC	NA
Kabuli_wild_42207459	1399940847	*Ca_kabuli_Chr06*	42207459	T/C	NA	INTERGENIC	NA
Kabuli_wild_42521162	1399940848	*Ca_kabuli_Chr06*	42521162	G/T	Gene_Ca_23238	INTRON	UDP-glucuronosyl/UDP-glucosyltransferase
Kabuli_wild_42663847	1399940849	*Ca_kabuli_Chr06*	42663847	A/T	Gene_Ca_23243	SYNONYMOUS_CODING	Thiolase
Kabuli_wild_42840647	1399940850	*Ca_kabuli_Chr06*	42840647	C/G	NA	INTERGENIC	NA
Kabuli_wild_42840687	1399940851	*Ca_kabuli_Chr06*	42840687	T/C	NA	INTERGENIC	NA
Kabuli_wild_42840729	1399940852	*Ca_kabuli_Chr06*	42840729	G/A	NA	INTERGENIC	NA
Kabuli_wild_42840747	1399940853	*Ca_kabuli_Chr06*	42840747	C/T	NA	INTERGENIC	NA
Kabuli_wild_42855091	1399940854	*Ca_kabuli_Chr06*	42855091	T/G	Gene_Ca_23252	NON_SYNONYMOUS_CODING	BTB/POZ-like
Kabuli_wild_42905012	1399940855	*Ca_kabuli_Chr06*	42905012	G/A	Gene_Ca_23254	SYNONYMOUS_CODING	Peptidase C48, SUMO/Sentrin/Ubl1
Kabuli_wild_42905035	1399940856	*Ca_kabuli_Chr06*	42905035	T/A	Gene_Ca_23254	NON_SYNONYMOUS_CODING	Peptidase C48, SUMO/Sentrin/Ubl1
Kabuli_wild_42905039	1399940857	*Ca_kabuli_Chr06*	42905039	C/T	Gene_Ca_23254	NON_SYNONYMOUS_CODING	Peptidase C48, SUMO/Sentrin/Ubl1
Kabuli_wild_42905058	1399940858	*Ca_kabuli_Chr06*	42905058	C/G	Gene_Ca_23254	NON_SYNONYMOUS_CODING	Peptidase C48, SUMO/Sentrin/Ubl1
Kabuli_wild_42905059	1399940859	*Ca_kabuli_Chr06*	42905059	A/G	Gene_Ca_23254	SYNONYMOUS_CODING	Peptidase C48, SUMO/Sentrin/Ubl1
Kabuli_wild_42905073	1399940860	*Ca_kabuli_Chr06*	42905073	C/T	Gene_Ca_23254	NON_SYNONYMOUS_CODING	Peptidase C48, SUMO/Sentrin/Ubl1
Kabuli_wild_42905086	1399940861	*Ca_kabuli_Chr06*	42905086	T/C	Gene_Ca_23254	SYNONYMOUS_CODING	Peptidase C48, SUMO/Sentrin/Ubl1
Kabuli_wild_42905090	1399940862	*Ca_kabuli_Chr06*	42905090	A/G	Gene_Ca_23254	SYNONYMOUS_CODING	Peptidase C48, SUMO/Sentrin/Ubl1
Kabuli_wild_42905094	1399940863	*Ca_kabuli_Chr06*	42905094	C/T	Gene_Ca_23254	NON_SYNONYMOUS_CODING	Peptidase C48, SUMO/Sentrin/Ubl1
Kabuli_wild_42905115	1399940864	*Ca_kabuli_Chr06*	42905115	G/T	Gene_Ca_23254	NON_SYNONYMOUS_CODING	Peptidase C48, SUMO/Sentrin/Ubl1
Kabuli_wild_42905116	1399940865	*Ca_kabuli_Chr06*	42905116	A/T	Gene_Ca_23254	SYNONYMOUS_CODING	Peptidase C48, SUMO/Sentrin/Ubl1
Kabuli_wild_42923969	1399940866	*Ca_kabuli_Chr06*	42923969	A/C	Gene_Ca_23255	DOWNSTREAM	Zinc finger,RING-type
Kabuli_wild_43148784	1399940867	*Ca_kabuli_Chr06*	43148784	C/G	NA	INTERGENIC	NA

SNP IDs	NCBI dbSNP accession IDs (NCBI_SS#)	Chromosomes	Physical positions (bp)	SNPs	Gene accession IDs	Sequence components of genome	Putative functions
Kabuli_wild_43148793	1399940868	*Ca_kabuli_Chr06*	43148793	T/G	NA	INTERGENIC	NA
Kabuli_wild_43150118	1399940869	*Ca_kabuli_Chr06*	43150118	G/T	NA	INTERGENIC	NA
Kabuli_wild_43150146	1399940870	*Ca_kabuli_Chr06*	43150146	A/T	NA	INTERGENIC	NA
Kabuli_wild_43878892	1399940871	*Ca_kabuli_Chr06*	43878892	G/T	NA	INTERGENIC	NA
Kabuli_wild_43879105	1399940872	*Ca_kabuli_Chr06*	43879105	C/T	NA	INTERGENIC	NA
Kabuli_wild_43905486	1399940873	*Ca_kabuli_Chr06*	43905486	G/T	NA	INTERGENIC	NA
Kabuli_wild_43905509	1399940874	*Ca_kabuli_Chr06*	43905509	A/T	NA	INTERGENIC	NA
Kabuli_wild_43905518	1399940875	*Ca_kabuli_Chr06*	43905518	G/A	NA	INTERGENIC	NA
Kabuli_wild_43905519	1399940876	*Ca_kabuli_Chr06*	43905519	G/A	NA	INTERGENIC	NA
Kabuli_wild_43907424	1399940877	*Ca_kabuli_Chr06*	43907424	G/T	NA	INTERGENIC	NA
Kabuli_wild_43907445	1399940878	*Ca_kabuli_Chr06*	43907445	T/C	NA	INTERGENIC	NA
Kabuli_wild_44022191	1399940879	*Ca_kabuli_Chr06*	44022191	T/G	Gene_Ca_24870	NON_SYNONYMOUS_CODING	Protein of unknown function DUF1421
Kabuli_wild_44293637	1399940880	*Ca_kabuli_Chr06*	44293637	C/T	NA	INTERGENIC	NA
Kabuli_wild_44293647	1399940881	*Ca_kabuli_Chr06*	44293647	T/A	NA	INTERGENIC	NA
Kabuli_wild_44293648	1399940882	*Ca_kabuli_Chr06*	44293648	T/A	NA	INTERGENIC	NA
Kabuli_wild_44293649	1399940883	*Ca_kabuli_Chr06*	44293649	A/G	NA	INTERGENIC	NA
Kabuli_wild_44293651	1399940884	*Ca_kabuli_Chr06*	44293651	G/T	NA	INTERGENIC	NA
Kabuli_wild_44293673	1399940885	*Ca_kabuli_Chr06*	44293673	C/T	NA	INTERGENIC	NA
Kabuli_wild_44293675	1399940886	*Ca_kabuli_Chr06*	44293675	G/A	NA	INTERGENIC	NA
Kabuli_wild_44293681	1399940887	*Ca_kabuli_Chr06*	44293681	T/A	NA	INTERGENIC	NA
Kabuli_wild_44530888	1399940888	*Ca_kabuli_Chr06*	44530888	C/A	Gene_Ca_24241	NON_SYNONYMOUS_CODING	Aspartate/glutamate/uridylatekinase
Kabuli_wild_44530889	1399940889	*Ca_kabuli_Chr06*	44530889	A/G	Gene_Ca_24241	SYNONYMOUS_CODING	Aspartate/glutamate/uridylatekinase
Kabuli_wild_44707313	1399940890	*Ca_kabuli_Chr06*	44707313	T/A	NA	INTERGENIC	NA
Kabuli_wild_44711166	1399940891	*Ca_kabuli_Chr06*	44711166	A/G	NA	INTERGENIC	NA
Kabuli_wild_44711168	1399940892	*Ca_kabuli_Chr06*	44711168	T/G	NA	INTERGENIC	NA
Kabuli_wild_44711216	1399940893	*Ca_kabuli_Chr06*	44711216	G/A	NA	INTERGENIC	NA
Kabuli_wild_44711238	1399940894	*Ca_kabuli_Chr06*	44711238	C/G	NA	INTERGENIC	NA
Kabuli_wild_44792457	1399940895	*Ca_kabuli_Chr06*	44792457	G/A	Gene_Ca_13807	NON_SYNONYMOUS_CODING	NA
Kabuli_wild_44792461	1399940896	*Ca_kabuli_Chr06*	44792461	C/G	Gene_Ca_13807	NON_SYNONYMOUS_CODING	NA
Kabuli_wild_44792553	1399940897	*Ca_kabuli_Chr06*	44792553	G/A	Gene_Ca_13807	SYNONYMOUS_CODING	NA
Kabuli_wild_44792566	1399940898	*Ca_kabuli_Chr06*	44792566	G/C	Gene_Ca_13807	SYNONYMOUS_CODING	NA
Kabuli_wild_44814082	1399940899	*Ca_kabuli_Chr06*	44814082	G/A	NA	INTERGENIC	NA
Kabuli_wild_44944917	1399940900	*Ca_kabuli_Chr06*	44944917	G/A	Gene_Ca_13813	NON_SYNONYMOUS_CODING	Carbohydrate/puinekinase,PfkB,conservedsite
Kabuli_wild_44959884	1399940901	*Ca_kabuli_Chr06*	44959884	A/C	NA	INTERGENIC	NA
Kabuli_wild_44959886	1399940902	*Ca_kabuli_Chr06*	44959886	G/T	NA	INTERGENIC	NA
Kabuli_wild_44959905	1399940903	*Ca_kabuli_Chr06*	44959905	A/C	NA	INTERGENIC	NA
Kabuli_wild_45178044	1399940904	*Ca_kabuli_Chr06*	45178044	G/A	NA	INTERGENIC	NA
Kabuli_wild_45178060	1399940905	*Ca_kabuli_Chr06*	45178060	G/A	NA	INTERGENIC	NA
Kabuli_wild_45178061	1399940906	*Ca_kabuli_Chr06*	45178061	C/T	NA	INTERGENIC	NA
Kabuli_wild_45178077	1399940907	*Ca_kabuli_Chr06*	45178077	C/A	NA	INTERGENIC	NA
Kabuli_wild_45293271	1399940908	*Ca_kabuli_Chr06*	45293271	T/C	NA	INTERGENIC	NA
Kabuli_wild_45500961	1399940909	*Ca_kabuli_Chr06*	45500961	T/A	Gene_Ca_13841	NON_SYNONYMOUS_CODING	Ankyrinrepeat
Kabuli_wild_45505942	1399940910	*Ca_kabuli_Chr06*	45505942	A/G	Gene_Ca_13841	NON_SYNONYMOUS_CODING	Ankyrinrepeat
Kabuli_wild_45524526	1399940911	*Ca_kabuli_Chr06*	45524526	G/C	NA	INTERGENIC	NA

SNP IDs	NCBI dbSNP accession IDs (NCBI_SS#)	Chromosomes	Physical positions (bp)	SNPs	Gene accession IDs	Sequence components of genome	Putative functions
Kabuli_wild_45538329	1399940912	*Ca_kabuli_Chr06*	45538329	A/G	Gene_Ca_13846	INTRON	Thiolase
Kabuli_wild_45550031	1399940913	*Ca_kabuli_Chr06*	45550031	C/A	NA	INTERGENIC	NA
Kabuli_wild_45554040	1399940914	*Ca_kabuli_Chr06*	45554040	G/A	Gene_Ca_13847	DOWNSTREAM	C2 calcium-dependent membrane targeting
Kabuli_wild_45637276	1399940915	*Ca_kabuli_Chr06*	45637276	G/A	Gene_Ca_13850	NON_SYNONYMOUS_CODING	NA
Kabuli_wild_45637284	1399940916	*Ca_kabuli_Chr06*	45637284	A/G	Gene_Ca_13850	SYNONYMOUS_CODING	NA
Kabuli_wild_45637292	1399940917	*Ca_kabuli_Chr06*	45637292	C/A	Gene_Ca_13850	STOP_GAINED	NA
Kabuli_wild_45637293	1399940918	*Ca_kabuli_Chr06*	45637293	T/C	Gene_Ca_13850	SYNONYMOUS_CODING	NA
Kabuli_wild_45637308	1399940919	*Ca_kabuli_Chr06*	45637308	G/T	Gene_Ca_13850	SYNONYMOUS_CODING	NA
Kabuli_wild_45637309	1399940920	*Ca_kabuli_Chr06*	45637309	G/A	Gene_Ca_13850	NON_SYNONYMOUS_CODING	NA
Kabuli_wild_45637315	1399940921	*Ca_kabuli_Chr06*	45637315	C/A	Gene_Ca_13850	NON_SYNONYMOUS_CODING	NA
Kabuli_wild_45637322	1399940922	*Ca_kabuli_Chr06*	45637322	C/A	Gene_Ca_13850	NON_SYNONYMOUS_CODING	NA
Kabuli_wild_45637328	1399940923	*Ca_kabuli_Chr06*	45637328	A/G	Gene_Ca_13850	SYNONYMOUS_CODING	NA
Kabuli_wild_45637331	1399940924	*Ca_kabuli_Chr06*	45637331	T/G	Gene_Ca_13850	NON_SYNONYMOUS_CODING	NA
Kabuli_wild_45693275	1399940925	*Ca_kabuli_Chr06*	45693275	G/A	NA	INTERGENIC	NA
Kabuli_wild_45978263	1399940926	*Ca_kabuli_Chr06*	45978263	G/A	NA	INTERGENIC	NA
Kabuli_wild_45982145	1399940927	*Ca_kabuli_Chr06*	45982145	T/C	Gene_Ca_13868	NON_SYNONYMOUS_CODING	NA
Kabuli_wild_46113631	1399940928	*Ca_kabuli_Chr06*	46113631	T/G	Gene_Ca_13877	SYNONYMOUS_CODING	NA
Kabuli_wild_46113656	1399940929	*Ca_kabuli_Chr06*	46113656	T/G	Gene_Ca_13877	SYNONYMOUS_CODING	NA
Kabuli_wild_46113696	1399940930	*Ca_kabuli_Chr06*	46113696	C/G	Gene_Ca_13877	SYNONYMOUS_CODING	NA
Kabuli_wild_46113717	1399940931	*Ca_kabuli_Chr06*	46113717	T/C	Gene_Ca_13877	SYNONYMOUS_CODING	NA
Kabuli_wild_46157942	1399940932	*Ca_kabuli_Chr06*	46157942	A/C	Gene_Ca_13881	NON_SYNONYMOUS_CODING	Aminoacyl-tRNAsynthetase,classI,conservedsite
Kabuli_wild_46157943	1399940933	*Ca_kabuli_Chr06*	46157943	G/T	Gene_Ca_13881	NON_SYNONYMOUS_CODING	Aminoacyl-tRNAsynthetase,classI,conservedsite
Kabuli_wild_46268499	1399940934	*Ca_kabuli_Chr06*	46268499	C/A	NA	INTERGENIC	NA
Kabuli_wild_46734668	1399940935	*Ca_kabuli_Chr06*	46734668	A/C	NA	INTERGENIC	NA
Kabuli_wild_46734706	1399940936	*Ca_kabuli_Chr06*	46734706	C/G	NA	INTERGENIC	NA
Kabuli_wild_46734717	1399940937	*Ca_kabuli_Chr06*	46734717	A/G	NA	INTERGENIC	NA
Kabuli_wild_46734725	1399940938	*Ca_kabuli_Chr06*	46734725	G/A	NA	INTERGENIC	NA
Kabuli_wild_46734726	1399940939	*Ca_kabuli_Chr06*	46734726	T/C	NA	INTERGENIC	NA
Kabuli_wild_46734782	1399940940	*Ca_kabuli_Chr06*	46734782	C/A	NA	INTERGENIC	NA
Kabuli_wild_46734817	1399940941	*Ca_kabuli_Chr06*	46734817	G/T	NA	INTERGENIC	NA
Kabuli_wild_46765659	1399940942	*Ca_kabuli_Chr06*	46765659	G/A	Gene_Ca_17737	NON_SYNONYMOUS_CODING	Peptidase C48, SUMO/Sentrin/Ubl1
Kabuli_wild_46765661	1399940943	*Ca_kabuli_Chr06*	46765661	A/T	Gene_Ca_17737	SYNONYMOUS_CODING	Peptidase C48, SUMO/Sentrin/Ubl1
Kabuli_wild_46765670	1399940944	*Ca_kabuli_Chr06*	46765670	T/A	Gene_Ca_17737	NON_SYNONYMOUS_CODING	Peptidase C48, SUMO/Sentrin/Ubl1
Kabuli_wild_46765673	1399940945	*Ca_kabuli_Chr06*	46765673	G/A	Gene_Ca_17737	SYNONYMOUS_CODING	Peptidase C48, SUMO/Sentrin/Ubl1
Kabuli_wild_46765696	1399940946	*Ca_kabuli_Chr06*	46765696	C/T	Gene_Ca_17737	NON_SYNONYMOUS_CODING	Peptidase C48, SUMO/Sentrin/Ubl1
Kabuli_wild_46765704	1399940947	*Ca_kabuli_Chr06*	46765704	G/A	Gene_Ca_17737	NON_SYNONYMOUS_CODING	Peptidase C48, SUMO/Sentrin/Ubl1
Kabuli_wild_46765725	1399940948	*Ca_kabuli_Chr06*	46765725	C/T	Gene_Ca_17737	NON_SYNONYMOUS_CODING	Peptidase C48, SUMO/Sentrin/Ubl1
Kabuli_wild_46765741	1399940949	*Ca_kabuli_Chr06*	46765741	G/A	Gene_Ca_17737	NON_SYNONYMOUS_CODING	Peptidase C48, SUMO/Sentrin/Ubl1
Kabuli_wild_46765753	1399940950	*Ca_kabuli_Chr06*	46765753	A/G	Gene_Ca_17737	NON_SYNONYMOUS_CODING	Peptidase C48, SUMO/Sentrin/Ubl1
Kabuli_wild_46765778	1399940951	*Ca_kabuli_Chr06*	46765778	C/A	Gene_Ca_17737	SYNONYMOUS_CODING	Peptidase C48, SUMO/Sentrin/Ubl1
Kabuli_wild_46896903	1399940952	*Ca_kabuli_Chr06*	46896903	C/T	NA	INTERGENIC	NA
Kabuli_wild_46896910	1399940953	*Ca_kabuli_Chr06*	46896910	A/C	NA	INTERGENIC	NA
Kabuli_wild_46898225	1399940954	*Ca_kabuli_Chr06*	46898225	A/C	NA	INTERGENIC	NA
Kabuli_wild_46898305	1399940955	*Ca_kabuli_Chr06*	46898305	T/G	NA	INTERGENIC	NA

SNP IDs	NCBI dbSNP accession IDs (NCBI_SS#)	Chromosomes	Physical positions (bp)	SNPs	Gene accession IDs	Sequence components of genome	Putative functions
Kabuli_wild_46898316	1399940956	*Ca_kabuli_Chr06*	46898316	T/G	NA	INTERGENIC	NA
Kabuli_wild_46898317	1399940957	*Ca_kabuli_Chr06*	46898317	C/T	NA	INTERGENIC	NA
Kabuli_wild_46898661	1399940958	*Ca_kabuli_Chr06*	46898661	C/A	NA	INTERGENIC	NA
Kabuli_wild_46898699	1399940959	*Ca_kabuli_Chr06*	46898699	G/T	NA	INTERGENIC	NA
Kabuli_wild_46899229	1399940960	*Ca_kabuli_Chr06*	46899229	G/A	NA	INTERGENIC	NA
Kabuli_wild_46979240	1399940961	*Ca_kabuli_Chr06*	46979240	C/T	Gene_Ca_17728	UPSTREAM	Pentatrico peptide repeat
Kabuli_wild_46979267	1399940962	*Ca_kabuli_Chr06*	46979267	C/A	Gene_Ca_17728	UPSTREAM	Pentatrico peptide repeat
Kabuli_wild_46998962	1399940963	*Ca_kabuli_Chr06*	46998962	G/A	NA	INTERGENIC	NA
Kabuli_wild_46998967	1399940964	*Ca_kabuli_Chr06*	46998967	A/G	NA	INTERGENIC	NA
Kabuli_wild_46998973	1399940965	*Ca_kabuli_Chr06*	46998973	C/T	NA	INTERGENIC	NA
Kabuli_wild_46999001	1399940966	*Ca_kabuli_Chr06*	46999001	C/T	NA	INTERGENIC	NA
Kabuli_wild_46999004	1399940967	*Ca_kabuli_Chr06*	46999004	C/T	NA	INTERGENIC	NA
Kabuli_wild_46999007	1399940968	*Ca_kabuli_Chr06*	46999007	T/C	NA	INTERGENIC	NA
Kabuli_wild_46999033	1399940969	*Ca_kabuli_Chr06*	46999033	G/A	NA	INTERGENIC	NA
Kabuli_wild_46999035	1399940970	*Ca_kabuli_Chr06*	46999035	A/T	NA	INTERGENIC	NA
Kabuli_wild_46999052	1399940971	*Ca_kabuli_Chr06*	46999052	A/G	NA	INTERGENIC	NA
Kabuli_wild_47024720	1399940972	*Ca_kabuli_Chr06*	47024720	A/G	NA	INTERGENIC	NA
Kabuli_wild_47906599	1399940973	*Ca_kabuli_Chr06*	47906599	A/G	Gene_Ca_23445	INTRON	Zinc finger,CCHC-type
Kabuli_wild_47906615	1399940974	*Ca_kabuli_Chr06*	47906615	A/T	Gene_Ca_23445	INTRON	Zinc finger,CCHC-type
Kabuli_wild_47906629	1399940975	*Ca_kabuli_Chr06*	47906629	T/G	Gene_Ca_23445	INTRON	Zinc finger,CCHC-type
Kabuli_wild_47906647	1399940976	*Ca_kabuli_Chr06*	47906647	T/C	Gene_Ca_23445	INTRON	Zinc finger,CCHC-type
Kabuli_wild_47906677	1399940977	*Ca_kabuli_Chr06*	47906677	G/A	Gene_Ca_23445	INTRON	Zinc finger,CCHC-type
Kabuli_wild_47906680	1399940978	*Ca_kabuli_Chr06*	47906680	C/T	Gene_Ca_23445	INTRON	Zinc finger,CCHC-type
Kabuli_wild_47906683	1399940979	*Ca_kabuli_Chr06*	47906683	G/A	Gene_Ca_23445	INTRON	Zinc finger,CCHC-type
Kabuli_wild_48088550	1399940980	*Ca_kabuli_Chr06*	48088550	G/A	NA	INTERGENIC	NA
Kabuli_wild_48088561	1399940981	*Ca_kabuli_Chr06*	48088561	C/T	NA	INTERGENIC	NA
Kabuli_wild_48088592	1399940982	*Ca_kabuli_Chr06*	48088592	C/T	NA	INTERGENIC	NA
Kabuli_wild_48088621	1399940983	*Ca_kabuli_Chr06*	48088621	G/A	NA	INTERGENIC	NA
Kabuli_wild_48251190	1399940984	*Ca_kabuli_Chr06*	48251190	T/G	Gene_Ca_24603	INTRON	Queuine/othertRNA-ribosyltransferase
Kabuli_wild_48251264	1399940985	*Ca_kabuli_Chr06*	48251264	T/C	Gene_Ca_24603	INTRON	Queuine/othertRNA-ribosyltransferase
Kabuli_wild_48255380	1399940986	*Ca_kabuli_Chr06*	48255380	A/C	Gene_Ca_24604	DOWNSTREAM	DNA/RNAhelicase,DEAD/DEAHboxtype,N-terminal
Kabuli_wild_48255392	1399940987	*Ca_kabuli_Chr06*	48255392	G/A	Gene_Ca_24604	DOWNSTREAM	DNA/RNAhelicase,DEAD/DEAHboxtype,N-terminal
Kabuli_wild_48255538	1399940988	*Ca_kabuli_Chr06*	48255538	A/G	Gene_Ca_24604	DOWNSTREAM	DNA/RNAhelicase,DEAD/DEAHboxtype,N-terminal
Kabuli_wild_48255558	1399940989	*Ca_kabuli_Chr06*	48255558	C/T	Gene_Ca_24604	DOWNSTREAM	DNA/RNAhelicase,DEAD/DEAHboxtype,N-terminal
Kabuli_wild_48255559	1399940990	*Ca_kabuli_Chr06*	48255559	A/G	Gene_Ca_24604	DOWNSTREAM	DNA/RNAhelicase,DEAD/DEAHboxtype,N-terminal
Kabuli_wild_48255568	1399940991	*Ca_kabuli_Chr06*	48255568	A/G	Gene_Ca_24604	DOWNSTREAM	DNA/RNAhelicase,DEAD/DEAHboxtype,N-terminal
Kabuli_wild_48255599	1399940992	*Ca_kabuli_Chr06*	48255599	C/A	Gene_Ca_24604	DOWNSTREAM	DNA/RNAhelicase,DEAD/DEAHboxtype,N-terminal
Kabuli_wild_48314052	1399940993	*Ca_kabuli_Chr06*	48314052	T/A	Gene_Ca_24606	SYNONYMOUS_CODING	NA
Kabuli_wild_48314122	1399940994	*Ca_kabuli_Chr06*	48314122	C/G	Gene_Ca_24606	NON_SYNONYMOUS_CODING	NA
Kabuli_wild_48314744	1399940995	*Ca_kabuli_Chr06*	48314744	T/C	Gene_Ca_24606	DOWNSTREAM	NA
Kabuli_wild_48314834	1399940996	*Ca_kabuli_Chr06*	48314834	C/T	Gene_Ca_24606	DOWNSTREAM	NA
Kabuli_wild_48315189	1399940997	*Ca_kabuli_Chr06*	48315189	C/T	Gene_Ca_24606	DOWNSTREAM	NA
Kabuli_wild_48315194	1399940998	*Ca_kabuli_Chr06*	48315194	C/A	Gene_Ca_24606	DOWNSTREAM	NA
Kabuli_wild_48315278	1399940999	*Ca_kabuli_Chr06*	48315278	G/A	Gene_Ca_24606	DOWNSTREAM	NA

SNP IDs	NCBI dbSNP accession IDs (NCBI_SS#)	Chromosomes	Physical positions (bp)	SNPs	Gene accession IDs	Sequence components of genome	Putative functions
Kabuli_wild_48315340	1399941000	*Ca_kabuli_Chr06*	48315340	C/A	Gene_Ca_24606	DOWNSTREAM	NA
Kabuli_wild_48315343	1399941001	*Ca_kabuli_Chr06*	48315343	G/T	Gene_Ca_24606	DOWNSTREAM	NA
Kabuli_wild_48709584	1399941002	*Ca_kabuli_Chr06*	48709584	T/C	NA	INTERGENIC	NA
Kabuli_wild_48709664	1399941003	*Ca_kabuli_Chr06*	48709664	C/T	NA	INTERGENIC	NA
Kabuli_wild_48792532	1399941004	*Ca_kabuli_Chr06*	48792532	A/G	Gene_Ca_20702	SYNONYMOUS_CODING	NA
Kabuli_wild_48792602	1399941005	*Ca_kabuli_Chr06*	48792602	C/T	Gene_Ca_20702	SYNONYMOUS_CODING	NA
Kabuli_wild_48792604	1399941006	*Ca_kabuli_Chr06*	48792604	A/G	Gene_Ca_20702	SYNONYMOUS_CODING	NA
Kabuli_wild_48792609	1399941007	*Ca_kabuli_Chr06*	48792609	G/T	Gene_Ca_20702	SYNONYMOUS_CODING	NA
Kabuli_wild_48799366	1399941008	*Ca_kabuli_Chr06*	48799366	T/A	NA	INTERGENIC	NA
Kabuli_wild_48799368	1399941009	*Ca_kabuli_Chr06*	48799368	A/T	NA	INTERGENIC	NA
Kabuli_wild_48799372	1399941010	*Ca_kabuli_Chr06*	48799372	C/T	NA	INTERGENIC	NA
Kabuli_wild_48799388	1399941011	*Ca_kabuli_Chr06*	48799388	C/A	NA	INTERGENIC	NA
Kabuli_wild_48799407	1399941012	*Ca_kabuli_Chr06*	48799407	T/C	NA	INTERGENIC	NA
Kabuli_wild_48799413	1399941013	*Ca_kabuli_Chr06*	48799413	T/C	NA	INTERGENIC	NA
Kabuli_wild_48799427	1399941014	*Ca_kabuli_Chr06*	48799427	G/T	NA	INTERGENIC	NA
Kabuli_wild_48864625	1399941015	*Ca_kabuli_Chr06*	48864625	G/A	NA	INTERGENIC	NA
Kabuli_wild_48864639	1399941016	*Ca_kabuli_Chr06*	48864639	A/C	NA	INTERGENIC	NA
Kabuli_wild_48864699	1399941017	*Ca_kabuli_Chr06*	48864699	T/C	NA	INTERGENIC	NA
Kabuli_wild_48869449	1399941018	*Ca_kabuli_Chr06*	48869449	A/G	Gene_Ca_20706	UPSTREAM	Integrase,catalyticcore
Kabuli_wild_48869452	1399941019	*Ca_kabuli_Chr06*	48869452	T/C	Gene_Ca_20706	UPSTREAM	Integrase,catalyticcore
Kabuli_wild_48869512	1399941020	*Ca_kabuli_Chr06*	48869512	G/C	Gene_Ca_20706	UPSTREAM	Integrase,catalyticcore
Kabuli_wild_48869529	1399941021	*Ca_kabuli_Chr06*	48869529	T/C	Gene_Ca_20706	UPSTREAM	Integrase,catalyticcore
Kabuli_wild_48869539	1399941022	*Ca_kabuli_Chr06*	48869539	A/G	Gene_Ca_20706	UPSTREAM	Integrase,catalyticcore
Kabuli_wild_48869577	1399941023	*Ca_kabuli_Chr06*	48869577	C/T	Gene_Ca_20706	UPSTREAM	Integrase,catalyticcore
Kabuli_wild_48897552	1399941024	*Ca_kabuli_Chr06*	48897552	G/A	NA	INTERGENIC	NA
Kabuli_wild_48940386	1399941025	*Ca_kabuli_Chr06*	48940386	A/C	Gene_Ca_20710	SYNONYMOUS_CODING	Clathrinadaptorcomplex,smallchain
Kabuli_wild_49044772	1399941026	*Ca_kabuli_Chr06*	49044772	A/C	NA	INTERGENIC	NA
Kabuli_wild_49044780	1399941027	*Ca_kabuli_Chr06*	49044780	G/A	NA	INTERGENIC	NA
Kabuli_wild_49044785	1399941028	*Ca_kabuli_Chr06*	49044785	C/T	NA	INTERGENIC	NA
Kabuli_wild_49044788	1399941029	*Ca_kabuli_Chr06*	49044788	T/A	NA	INTERGENIC	NA
Kabuli_wild_49044794	1399941030	*Ca_kabuli_Chr06*	49044794	T/A	NA	INTERGENIC	NA
Kabuli_wild_49044802	1399941031	*Ca_kabuli_Chr06*	49044802	C/T	NA	INTERGENIC	NA
Kabuli_wild_49044805	1399941032	*Ca_kabuli_Chr06*	49044805	G/A	NA	INTERGENIC	NA
Kabuli_wild_49044808	1399941033	*Ca_kabuli_Chr06*	49044808	C/T	NA	INTERGENIC	NA
Kabuli_wild_49044810	1399941034	*Ca_kabuli_Chr06*	49044810	G/A	NA	INTERGENIC	NA
Kabuli_wild_49044814	1399941035	*Ca_kabuli_Chr06*	49044814	G/T	NA	INTERGENIC	NA
Kabuli_wild_49044821	1399941036	*Ca_kabuli_Chr06*	49044821	C/G	NA	INTERGENIC	NA
Kabuli_wild_49044824	1399941037	*Ca_kabuli_Chr06*	49044824	C/T	NA	INTERGENIC	NA
Kabuli_wild_49044826	1399941038	*Ca_kabuli_Chr06*	49044826	G/A	NA	INTERGENIC	NA
Kabuli_wild_49044835	1399941039	*Ca_kabuli_Chr06*	49044835	C/A	NA	INTERGENIC	NA
Kabuli_wild_49044838	1399941040	*Ca_kabuli_Chr06*	49044838	C/A	NA	INTERGENIC	NA
Kabuli_wild_49044839	1399941041	*Ca_kabuli_Chr06*	49044839	T/A	NA	INTERGENIC	NA
Kabuli_wild_49044842	1399941042	*Ca_kabuli_Chr06*	49044842	G/A	NA	INTERGENIC	NA
Kabuli_wild_49044847	1399941043	*Ca_kabuli_Chr06*	49044847	G/A	NA	INTERGENIC	NA

SNP IDs	NCBI dbSNP accession IDs (NCBI_SS#)	Chromosomes	Physical positions (bp)	SNPs	Gene accession IDs	Sequence components of genome	Putative functions
Kabuli_wild_49044850	1399941044	*Ca_kabuli_Chr06*	49044850	C/A	NA	INTERGENIC	NA
Kabuli_wild_49044915	1399941045	*Ca_kabuli_Chr06*	49044915	A/G	NA	INTERGENIC	NA
Kabuli_wild_49044928	1399941046	*Ca_kabuli_Chr06*	49044928	C/A	NA	INTERGENIC	NA
Kabuli_wild_49044937	1399941047	*Ca_kabuli_Chr06*	49044937	A/G	NA	INTERGENIC	NA
Kabuli_wild_49044944	1399941048	*Ca_kabuli_Chr06*	49044944	A/G	NA	INTERGENIC	NA
Kabuli_wild_49044947	1399941049	*Ca_kabuli_Chr06*	49044947	C/G	NA	INTERGENIC	NA
Kabuli_wild_49044966	1399941050	*Ca_kabuli_Chr06*	49044966	G/T	NA	INTERGENIC	NA
Kabuli_wild_49044968	1399941051	*Ca_kabuli_Chr06*	49044968	G/T	NA	INTERGENIC	NA
Kabuli_wild_49101908	1399941052	*Ca_kabuli_Chr06*	49101908	C/A	NA	INTERGENIC	NA
Kabuli_wild_49167340	1399941053	*Ca_kabuli_Chr06*	49167340	C/T	NA	INTERGENIC	NA
Kabuli_wild_49167341	1399941054	*Ca_kabuli_Chr06*	49167341	T/G	NA	INTERGENIC	NA
Kabuli_wild_49190196	1399941055	*Ca_kabuli_Chr06*	49190196	C/T	Gene_Ca_20723	SYNONYMOUS_CODING	Peptidase C48, SUMO/Sentrin/Ubl1
Kabuli_wild_49190223	1399941056	*Ca_kabuli_Chr06*	49190223	T/A	Gene_Ca_20723	SYNONYMOUS_CODING	Peptidase C48, SUMO/Sentrin/Ubl1
Kabuli_wild_49190255	1399941057	*Ca_kabuli_Chr06*	49190255	G/A	Gene_Ca_20723	SYNONYMOUS_CODING	Peptidase C48, SUMO/Sentrin/Ubl1
Kabuli_wild_49190264	1399941058	*Ca_kabuli_Chr06*	49190264	G/A	Gene_Ca_20723	SYNONYMOUS_CODING	Peptidase C48, SUMO/Sentrin/Ubl1
Kabuli_wild_49190269	1399941059	*Ca_kabuli_Chr06*	49190269	G/C	Gene_Ca_20723	NON_SYNONYMOUS_CODING	Peptidase C48, SUMO/Sentrin/Ubl1
Kabuli_wild_49190272	1399941060	*Ca_kabuli_Chr06*	49190272	G/T	Gene_Ca_20723	NON_SYNONYMOUS_CODING	Peptidase C48, SUMO/Sentrin/Ubl1
Kabuli_wild_49222235	1399941061	*Ca_kabuli_Chr06*	49222235	G/C	NA	INTERGENIC	NA
Kabuli_wild_49222251	1399941062	*Ca_kabuli_Chr06*	49222251	G/A	NA	INTERGENIC	NA
Kabuli_wild_49222268	1399941063	*Ca_kabuli_Chr06*	49222268	T/A	NA	INTERGENIC	NA
Kabuli_wild_49222275	1399941064	*Ca_kabuli_Chr06*	49222275	A/C	NA	INTERGENIC	NA
Kabuli_wild_49222290	1399941065	*Ca_kabuli_Chr06*	49222290	C/T	NA	INTERGENIC	NA
Kabuli_wild_49222291	1399941066	*Ca_kabuli_Chr06*	49222291	G/A	NA	INTERGENIC	NA
Kabuli_wild_49377275	1399941067	*Ca_kabuli_Chr06*	49377275	G/A	Gene_Ca_13522	DOWNSTREAM	Leucine-rich repeat
Kabuli_wild_49377353	1399941068	*Ca_kabuli_Chr06*	49377353	A/C	Gene_Ca_13522	DOWNSTREAM	Leucine-rich repeat
Kabuli_wild_49445785	1399941069	*Ca_kabuli_Chr06*	49445785	A/G	NA	INTERGENIC	NA
Kabuli_wild_49524179	1399941070	*Ca_kabuli_Chr06*	49524179	A/T	NA	INTERGENIC	NA
Kabuli_wild_49565934	1399941071	*Ca_kabuli_Chr06*	49565934	C/T	Gene_Ca_13509	START_LOST	RibosomalproteinL37e
Kabuli_wild_49629674	1399941072	*Ca_kabuli_Chr06*	49629674	G/A	NA	INTERGENIC	NA
Kabuli_wild_49724431	1399941073	*Ca_kabuli_Chr06*	49724431	C/A	NA	INTERGENIC	NA
Kabuli_wild_49724472	1399941074	*Ca_kabuli_Chr06*	49724472	C/G	Gene_Ca_13494	DOWNSTREAM	NA
Kabuli_wild_49724476	1399941075	*Ca_kabuli_Chr06*	49724476	C/T	Gene_Ca_13494	DOWNSTREAM	NA
Kabuli_wild_49724558	1399941076	*Ca_kabuli_Chr06*	49724558	C/T	Gene_Ca_13494	DOWNSTREAM	NA
Kabuli_wild_49724574	1399941077	*Ca_kabuli_Chr06*	49724574	A/C	Gene_Ca_13494	DOWNSTREAM	NA
Kabuli_wild_49724585	1399941078	*Ca_kabuli_Chr06*	49724585	A/T	Gene_Ca_13494	DOWNSTREAM	NA
Kabuli_wild_49726711	1399941079	*Ca_kabuli_Chr06*	49726711	C/T	Gene_Ca_13495	DOWNSTREAM	Peptidase C48, SUMO/Sentrin/Ubl1
Kabuli_wild_49726723	1399941080	*Ca_kabuli_Chr06*	49726723	G/A	Gene_Ca_13495	DOWNSTREAM	Peptidase C48, SUMO/Sentrin/Ubl1
Kabuli_wild_49726729	1399941081	*Ca_kabuli_Chr06*	49726729	G/A	Gene_Ca_13495	DOWNSTREAM	Peptidase C48, SUMO/Sentrin/Ubl1
Kabuli_wild_49726734	1399941082	*Ca_kabuli_Chr06*	49726734	G/C	Gene_Ca_13495	DOWNSTREAM	Peptidase C48, SUMO/Sentrin/Ubl1
Kabuli_wild_49830299	1399941083	*Ca_kabuli_Chr06*	49830299	A/G	Gene_Ca_13490	NON_SYNONYMOUS_CODING	Heat shock protein Hsp70
Kabuli_wild_49846303	1399941084	*Ca_kabuli_Chr06*	49846303	T/G	Gene_Ca_13488	SYNONYMOUS_CODING	Heat shock protein Hsp70
Kabuli_wild_49940602	1399941085	*Ca_kabuli_Chr06*	49940602	C/A	Gene_Ca_13485	NON_SYNONYMOUS_CODING	Pentatrico peptide repeat
Kabuli_wild_49940603	1399941086	*Ca_kabuli_Chr06*	49940603	A/G	Gene_Ca_13485	SYNONYMOUS_CODING	Pentatrico peptide repeat
Kabuli_wild_50152053	1399941087	*Ca_kabuli_Chr06*	50152053	C/T	NA	INTERGENIC	NA

SNP IDs	NCBI dbSNP accession IDs (NCBI_SS#)	Chromosomes	Physical positions (bp)	SNPs	Gene accession IDs	Sequence components of genome	Putative functions
Kabuli_wild_50152082	1399941088	*Ca_kabuli_Chr06*	50152082	G/A	NA	INTERGENIC	NA
Kabuli_wild_50152092	1399941089	*Ca_kabuli_Chr06*	50152092	T/A	NA	INTERGENIC	NA
Kabuli_wild_50152113	1399941090	*Ca_kabuli_Chr06*	50152113	G/A	NA	INTERGENIC	NA
Kabuli_wild_50152124	1399941091	*Ca_kabuli_Chr06*	50152124	C/T	NA	INTERGENIC	NA
Kabuli_wild_50152775	1399941092	*Ca_kabuli_Chr06*	50152775	C/T	NA	INTERGENIC	NA
Kabuli_wild_50152787	1399941093	*Ca_kabuli_Chr06*	50152787	G/A	NA	INTERGENIC	NA
Kabuli_wild_50152840	1399941094	*Ca_kabuli_Chr06*	50152840	C/T	NA	INTERGENIC	NA
Kabuli_wild_50152841	1399941095	*Ca_kabuli_Chr06*	50152841	G/T	NA	INTERGENIC	NA
Kabuli_wild_50356255	1399941096	*Ca_kabuli_Chr06*	50356255	G/A	NA	INTERGENIC	NA
Kabuli_wild_50477672	1399941097	*Ca_kabuli_Chr06*	50477672	A/C	Gene_Ca_13459	SYNONYMOUS_CODING	NA
Kabuli_wild_50617932	1399941098	*Ca_kabuli_Chr06*	50617932	G/A	Gene_Ca_13450	SYNONYMOUS_CODING	NA
Kabuli_wild_50649899	1399941099	*Ca_kabuli_Chr06*	50649899	C/T	NA	INTERGENIC	NA
Kabuli_wild_50723595	1399941100	*Ca_kabuli_Chr06*	50723595	T/C	NA	INTERGENIC	NA
Kabuli_wild_50890769	1399941101	*Ca_kabuli_Chr06*	50890769	G/A	NA	INTERGENIC	NA
Kabuli_wild_51157939	1399941102	*Ca_kabuli_Chr06*	51157939	A/C	Gene_Ca_19183	NON_SYNONYMOUS_CODING	NA
Kabuli_wild_51471573	1399941103	*Ca_kabuli_Chr06*	51471573	T/C	Gene_Ca_19165	NON_SYNONYMOUS_CODING	NA
Kabuli_wild_51606991	1399941104	*Ca_kabuli_Chr06*	51606991	T/A	Gene_Ca_25851	NON_SYNONYMOUS_CODING	RibosomalproteinL1
Kabuli_wild_51617288	1399941105	*Ca_kabuli_Chr06*	51617288	C/A	NA	INTERGENIC	NA
Kabuli_wild_51692501	1399941106	*Ca_kabuli_Chr06*	51692501	C/T	NA	INTERGENIC	NA
Kabuli_wild_51692515	1399941107	*Ca_kabuli_Chr06*	51692515	G/C	NA	INTERGENIC	NA
Kabuli_wild_51692534	1399941108	*Ca_kabuli_Chr06*	51692534	A/G	NA	INTERGENIC	NA
Kabuli_wild_51692540	1399941109	*Ca_kabuli_Chr06*	51692540	G/A	NA	INTERGENIC	NA
Kabuli_wild_51692548	1399941110	*Ca_kabuli_Chr06*	51692548	C/T	NA	INTERGENIC	NA
Kabuli_wild_51692550	1399941111	*Ca_kabuli_Chr06*	51692550	T/A	NA	INTERGENIC	NA
Kabuli_wild_51692553	1399941112	*Ca_kabuli_Chr06*	51692553	T/A	NA	INTERGENIC	NA
Kabuli_wild_52007211	1399941113	*Ca_kabuli_Chr06*	52007211	T/C	NA	INTERGENIC	NA
Kabuli_wild_52007225	1399941114	*Ca_kabuli_Chr06*	52007225	T/C	NA	INTERGENIC	NA
Kabuli_wild_52007228	1399941115	*Ca_kabuli_Chr06*	52007228	C/T	NA	INTERGENIC	NA
Kabuli_wild_52007238	1399941116	*Ca_kabuli_Chr06*	52007238	A/T	NA	INTERGENIC	NA
Kabuli_wild_52007266	1399941117	*Ca_kabuli_Chr06*	52007266	C/A	NA	INTERGENIC	NA
Kabuli_wild_52007306	1399941118	*Ca_kabuli_Chr06*	52007306	G/T	NA	INTERGENIC	NA
Kabuli_wild_52007318	1399941119	*Ca_kabuli_Chr06*	52007318	G/A	NA	INTERGENIC	NA
Kabuli_wild_52007353	1399941120	*Ca_kabuli_Chr06*	52007353	G/A	NA	INTERGENIC	NA
Kabuli_wild_52007380	1399941121	*Ca_kabuli_Chr06*	52007380	A/C	NA	INTERGENIC	NA
Kabuli_wild_52007392	1399941122	*Ca_kabuli_Chr06*	52007392	C/A	NA	INTERGENIC	NA
Kabuli_wild_52007427	1399941123	*Ca_kabuli_Chr06*	52007427	T/G	NA	INTERGENIC	NA
Kabuli_wild_52007440	1399941124	*Ca_kabuli_Chr06*	52007440	C/T	NA	INTERGENIC	NA
Kabuli_wild_52007464	1399941125	*Ca_kabuli_Chr06*	52007464	C/G	NA	INTERGENIC	NA
Kabuli_wild_52007471	1399941126	*Ca_kabuli_Chr06*	52007471	C/T	NA	INTERGENIC	NA
Kabuli_wild_52012401	1399941127	*Ca_kabuli_Chr06*	52012401	C/T	NA	INTERGENIC	NA
Kabuli_wild_52021996	1399941128	*Ca_kabuli_Chr06*	52021996	G/A	Gene_Ca_17498	DOWNSTREAM	AuxinresponsiveSAURprotein
Kabuli_wild_52065393	1399941129	*Ca_kabuli_Chr06*	52065393	C/A	NA	INTERGENIC	NA
Kabuli_wild_52065395	1399941130	*Ca_kabuli_Chr06*	52065395	G/T	NA	INTERGENIC	NA
Kabuli_wild_52065527	1399941131	*Ca_kabuli_Chr06*	52065527	T/A	NA	INTERGENIC	NA

SNP IDs	NCBI dbSNP accession IDs (NCBI_SS#)	Chromosomes	Physical positions (bp)	SNPs	Gene accession IDs	Sequence components of genome	Putative functions
Kabuli_wild_52164571	1399941132	*Ca_kabuli_Chr06*	52164571	C/A	Gene_Ca_17518	INTRON	ATPase,P-type,K/Mg/Cd/Cu/Zn/Na/Ca/Na/H-transporter
Kabuli_wild_52416534	1399941133	*Ca_kabuli_Chr06*	52416534	T/C	NA	INTERGENIC	NA
Kabuli_wild_52567474	1399941134	*Ca_kabuli_Chr06*	52567474	A/C	Gene_Ca_17540	NON_SYNONYMOUS_CODING	Transferase
Kabuli_wild_52596616	1399941135	*Ca_kabuli_Chr06*	52596616	T/C	Gene_Ca_17541	INTRON	Protein phosphatase 2C, manganese/magnesium aspartate binding site
Kabuli_wild_52597243	1399941136	*Ca_kabuli_Chr06*	52597243	G/A	Gene_Ca_17541	INTRON	Protein phosphatase 2C, manganese/magnesium aspartate binding site
Kabuli_wild_52630806	1399941137	*Ca_kabuli_Chr06*	52630806	A/C	NA	INTERGENIC	NA
Kabuli_wild_52684148	1399941138	*Ca_kabuli_Chr06*	52684148	T/G	Gene_Ca_17547	SYNONYMOUS_CODING	HhH-GPDdomain
Kabuli_wild_52851852	1399941139	*Ca_kabuli_Chr06*	52851852	G/C	NA	INTERGENIC	NA
Kabuli_wild_52858359	1399941140	*Ca_kabuli_Chr06*	52858359	A/G	NA	INTERGENIC	NA
Kabuli_wild_52890682	1399941141	*Ca_kabuli_Chr06*	52890682	G/C	NA	INTERGENIC	NA
Kabuli_wild_52899485	1399941142	*Ca_kabuli_Chr06*	52899485	A/T	NA	INTERGENIC	NA
Kabuli_wild_52993231	1399941143	*Ca_kabuli_Chr06*	52993231	A/G	NA	INTERGENIC	NA
Kabuli_wild_53097803	1399941144	*Ca_kabuli_Chr06*	53097803	A/G	Gene_Ca_22909	DOWNSTREAM	IQ motif,EF-handbinding site
Kabuli_wild_53097833	1399941145	*Ca_kabuli_Chr06*	53097833	G/C	Gene_Ca_22909	DOWNSTREAM	IQ motif,EF-handbinding site
Kabuli_wild_53124182	1399941146	*Ca_kabuli_Chr06*	53124182	G/T	Gene_Ca_22912	NON_SYNONYMOUS_CODING	3-Oxoacyl-[acyl-carrier-protein(ACP)]synthaseIIIC-terminal
Kabuli_wild_53348993	1399941147	*Ca_kabuli_Chr06*	53348993	C/A	Gene_Ca_18877	INTRON	Sodium/sulphatesymporter
Kabuli_wild_53349034	1399941148	*Ca_kabuli_Chr06*	53349034	G/A	Gene_Ca_18877	INTRON	Sodium/sulphatesymporter
Kabuli_wild_53382949	1399941149	*Ca_kabuli_Chr06*	53382949	C/T	Gene_Ca_18880	INTRON	GTP-bindingprotein,HSR1-related
Kabuli_wild_53383076	1399941150	*Ca_kabuli_Chr06*	53383076	C/T	Gene_Ca_18880	INTRON	GTP-bindingprotein,HSR1-related
Kabuli_wild_53461883	1399941151	*Ca_kabuli_Chr06*	53461883	A/G	NA	INTERGENIC	NA
Kabuli_wild_53461929	1399941152	*Ca_kabuli_Chr06*	53461929	C/A	NA	INTERGENIC	NA
Kabuli_wild_53541031	1399941153	*Ca_kabuli_Chr06*	53541031	G/A	Gene_Ca_18891	DOWNSTREAM	Zinc/ironpermease
Kabuli_wild_53541094	1399941154	*Ca_kabuli_Chr06*	53541094	C/G	Gene_Ca_18890	UPSTREAM	Ctrcoppertransporter
Kabuli_wild_53541111	1399941155	*Ca_kabuli_Chr06*	53541111	T/G	Gene_Ca_18890	UPSTREAM	Ctrcoppertransporter
Kabuli_wild_53551878	1399941156	*Ca_kabuli_Chr06*	53551878	C/A	NA	INTERGENIC	NA
Kabuli_wild_53558430	1399941157	*Ca_kabuli_Chr06*	53558430	A/C	NA	INTERGENIC	NA
Kabuli_wild_53566496	1399941158	*Ca_kabuli_Chr06*	53566496	G/A	Gene_Ca_18894	NON_SYNONYMOUS_CODING	Pathogenesis-related transcriptional factor/ERF, DNA-binding
Kabuli_wild_53625264	1399941159	*Ca_kabuli_Chr06*	53625264	A/C	Gene_Ca_18898	SYNONYMOUS_CODING	Leucine-rich repeat
Kabuli_wild_53683746	1399941160	*Ca_kabuli_Chr06*	53683746	C/T	Gene_Ca_18901	INTRON	NA
Kabuli_wild_53683753	1399941161	*Ca_kabuli_Chr06*	53683753	T/C	Gene_Ca_18901	INTRON	NA
Kabuli_wild_53683780	1399941162	*Ca_kabuli_Chr06*	53683780	T/A	Gene_Ca_18901	INTRON	NA
Kabuli_wild_53683876	1399941163	*Ca_kabuli_Chr06*	53683876	C/T	Gene_Ca_18901	INTRON	NA
Kabuli_wild_53683885	1399941164	*Ca_kabuli_Chr06*	53683885	C/T	Gene_Ca_18901	INTRON	NA
Kabuli_wild_53683891	1399941165	*Ca_kabuli_Chr06*	53683891	C/A	Gene_Ca_18901	INTRON	NA
Kabuli_wild_53692784	1399941166	*Ca_kabuli_Chr06*	53692784	G/A	NA	INTERGENIC	NA
Kabuli_wild_53769160	1399941167	*Ca_kabuli_Chr06*	53769160	C/T	NA	INTERGENIC	NA
Kabuli_wild_53840760	1399941168	*Ca_kabuli_Chr06*	53840760	T/C	Gene_Ca_18914	INTRON	Glycosidehydrolase,family1
Kabuli_wild_54092038	1399941169	*Ca_kabuli_Chr06*	54092038	T/C	NA	INTERGENIC	NA
Kabuli_wild_54151902	1399941170	*Ca_kabuli_Chr06*	54151902	T/G	Gene_Ca_16270	DOWNSTREAM	Pentatrico peptide repeat
Kabuli_wild_54151966	1399941171	*Ca_kabuli_Chr06*	54151966	G/C	Gene_Ca_16270	DOWNSTREAM	Pentatrico peptide repeat
Kabuli_wild_54151969	1399941172	*Ca_kabuli_Chr06*	54151969	T/G	Gene_Ca_16270	DOWNSTREAM	Pentatrico peptide repeat
Kabuli_wild_54157694	1399941173	*Ca_kabuli_Chr06*	54157694	T/C	NA	INTERGENIC	NA
Kabuli_wild_54157719	1399941174	*Ca_kabuli_Chr06*	54157719	G/T	NA	INTERGENIC	NA
Kabuli_wild_54203356	1399941175	*Ca_kabuli_Chr06*	54203356	G/A	Gene_Ca_16276	NON_SYNONYMOUS_CODING	NmrA-like

SNP IDs	NCBI dbSNP accession IDs (NCBI_SS#)	Chromosomes	Physical positions (bp)	SNPs	Gene accession IDs	Sequence components of genome	Putative functions
Kabuli_wild_54203400	1399941176	*Ca_kabuli_Chr06*	54203400	C/T	Gene_Ca_16276	SYNONYMOUS_CODING	NmrA-like
Kabuli_wild_54208203	1399941177	*Ca_kabuli_Chr06*	54208203	G/T	NA	INTERGENIC	NA
Kabuli_wild_54229449	1399941178	*Ca_kabuli_Chr06*	54229449	G/T	Gene_Ca_16279	DOWNSTREAM	NA
Kabuli_wild_54237764	1399941179	*Ca_kabuli_Chr06*	54237764	A/C	Gene_Ca_16281	UPSTREAM	Pentatrico peptide repeat
Kabuli_wild_54241792	1399941180	*Ca_kabuli_Chr06*	54241792	A/G	Gene_Ca_16281	DOWNSTREAM	Pentatrico peptide repeat
Kabuli_wild_54250702	1399941181	*Ca_kabuli_Chr06*	54250702	C/T	Gene_Ca_16283	NON_SYNONYMOUS_CODING	Pumilio RNA-binding repeat
Kabuli_wild_54250743	1399941182	*Ca_kabuli_Chr06*	54250743	G/A	Gene_Ca_16283	NON_SYNONYMOUS_CODING	Pumilio RNA-binding repeat
Kabuli_wild_54333329	1399941183	*Ca_kabuli_Chr06*	54333329	G/A	NA	INTERGENIC	NA
Kabuli_wild_54368267	1399941184	*Ca_kabuli_Chr06*	54368267	G/A	Gene_Ca_16294	DOWNSTREAM	Transcription facto rGRAS
Kabuli_wild_54869600	1399941185	*Ca_kabuli_Chr06*	54869600	C/A	Gene_Ca_21207	NON_SYNONYMOUS_CODING	Peptidase C48, SUMO/Sentrin/Ubl1
Kabuli_wild_54869625	1399941186	*Ca_kabuli_Chr06*	54869625	T/G	Gene_Ca_21207	SYNONYMOUS_CODING	Peptidase C48, SUMO/Sentrin/Ubl1
Kabuli_wild_54869683	1399941187	*Ca_kabuli_Chr06*	54869683	G/A	Gene_Ca_21207	NON_SYNONYMOUS_CODING	Peptidase C48, SUMO/Sentrin/Ubl1
Kabuli_wild_55024071	1399941188	*Ca_kabuli_Chr06*	55024071	C/T	Gene_Ca_21213	NON_SYNONYMOUS_CODING	NA
Kabuli_wild_55024093	1399941189	*Ca_kabuli_Chr06*	55024093	G/C	Gene_Ca_21213	NON_SYNONYMOUS_CODING	NA
Kabuli_wild_55024108	1399941190	*Ca_kabuli_Chr06*	55024108	C/T	Gene_Ca_21213	SYNONYMOUS_CODING	NA
Kabuli_wild_55024112	1399941191	*Ca_kabuli_Chr06*	55024112	C/G	Gene_Ca_21213	NON_SYNONYMOUS_CODING	NA
Kabuli_wild_55024130	1399941192	*Ca_kabuli_Chr06*	55024130	T/A	Gene_Ca_21213	NON_SYNONYMOUS_CODING	NA
Kabuli_wild_55024140	1399941193	*Ca_kabuli_Chr06*	55024140	G/A	Gene_Ca_21213	NON_SYNONYMOUS_CODING	NA
Kabuli_wild_55024146	1399941194	*Ca_kabuli_Chr06*	55024146	C/T	Gene_Ca_21213	NON_SYNONYMOUS_CODING	NA
Kabuli_wild_55024159	1399941195	*Ca_kabuli_Chr06*	55024159	T/A	Gene_Ca_21213	SYNONYMOUS_CODING	NA
Kabuli_wild_55024198	1399941196	*Ca_kabuli_Chr06*	55024198	T/G	Gene_Ca_21213	SYNONYMOUS_CODING	NA
Kabuli_wild_55074979	1399941197	*Ca_kabuli_Chr06*	55074979	T/A	NA	INTERGENIC	NA
Kabuli_wild_55083311	1399941198	*Ca_kabuli_Chr06*	55083311	T/A	Gene_Ca_21214	INTRON	Oligo peptide transporter
Kabuli_wild_55083721	1399941199	*Ca_kabuli_Chr06*	55083721	T/A	Gene_Ca_21214	SYNONYMOUS_CODING	Oligo peptide transporter
Kabuli_wild_55332515	1399941200	*Ca_kabuli_Chr06*	55332515	G/A	Gene_Ca_21219	SYNONYMOUS_CODING	Homeobox
Kabuli_wild_55378104	1399941201	*Ca_kabuli_Chr06*	55378104	A/C	NA	INTERGENIC	NA
Kabuli_wild_55683478	1399941202	*Ca_kabuli_Chr06*	55683478	T/A	NA	INTERGENIC	NA
Kabuli_wild_55922843	1399941203	*Ca_kabuli_Chr06*	55922843	T/A	Gene_Ca_19023	NON_SYNONYMOUS_CODING	Proteinofunknown functionwound-induced
Kabuli_wild_55942105	1399941204	*Ca_kabuli_Chr06*	55942105	A/G	Gene_Ca_19025	UPSTREAM	Zinc finger, RING-type
Kabuli_wild_55953320	1399941205	*Ca_kabuli_Chr06*	55953320	T/A	Gene_Ca_19026	NON_SYNONYMOUS_CODING	Proteinofunknown functionwound-induced
Kabuli_wild_56137857	1399941206	*Ca_kabuli_Chr06*	56137857	G/T	NA	INTERGENIC	NA
Kabuli_wild_56137878	1399941207	*Ca_kabuli_Chr06*	56137878	T/C	NA	INTERGENIC	NA
Kabuli_wild_56248243	1399941208	*Ca_kabuli_Chr06*	56248243	C/A	NA	INTERGENIC	NA
Kabuli_wild_56248258	1399941209	*Ca_kabuli_Chr06*	56248258	A/C	NA	INTERGENIC	NA
Kabuli_wild_56505281	1399941210	*Ca_kabuli_Chr06*	56505281	T/C	NA	INTERGENIC	NA
Kabuli_wild_56538737	1399941211	*Ca_kabuli_Chr06*	56538737	T/C	Gene_Ca_17391	NON_SYNONYMOUS_CODING	NA
Kabuli_wild_56553930	1399941212	*Ca_kabuli_Chr06*	56553930	G/A	Gene_Ca_17393	NON_SYNONYMOUS_CODING	TetratricopeptideTPR-1
Kabuli_wild_56564038	1399941213	*Ca_kabuli_Chr06*	56564038	T/A	NA	INTERGENIC	NA
Kabuli_wild_56564249	1399941214	*Ca_kabuli_Chr06*	56564249	G/C	NA	INTERGENIC	NA
Kabuli_wild_56587094	1399941215	*Ca_kabuli_Chr06*	56587094	C/G	Gene_Ca_17396	DOWNSTREAM	Prolinedehydrogenase
Kabuli_wild_56593121	1399941216	*Ca_kabuli_Chr06*	56593121	T/G	Gene_Ca_17397	SYNONYMOUS_CODING	Helicase,C-terminal
Kabuli_wild_56624316	1399941217	*Ca_kabuli_Chr06*	56624316	A/T	Gene_Ca_17398	INTRON	Prolinedehydrogenase
Kabuli_wild_56624515	1399941218	*Ca_kabuli_Chr06*	56624515	C/T	Gene_Ca_17398	SYNONYMOUS_CODING	Prolinedehydrogenase
Kabuli_wild_56624674	1399941219	*Ca_kabuli_Chr06*	56624674	T/C	Gene_Ca_17398	INTRON	Prolinedehydrogenase

SNP IDs	NCBI dbSNP accession IDs (NCBI_SS#)	Chromosomes	Physical positions (bp)	SNPs	Gene accession IDs	Sequence components of genome	Putative functions
Kabuli_wild_56624718	1399941220	*Ca_kabuli_Chr06*	56624718	C/A	Gene_Ca_17398	INTRON	Prolinedehydrogenase
Kabuli_wild_56674138	1399941221	*Ca_kabuli_Chr06*	56674138	G/C	NA	INTERGENIC	NA
Kabuli_wild_56674158	1399941222	*Ca_kabuli_Chr06*	56674158	G/C	NA	INTERGENIC	NA
Kabuli_wild_56694898	1399941223	*Ca_kabuli_Chr06*	56694898	T/C	Gene_Ca_17408	NON_SYNONYMOUS_CODING	ATPase,AAA+type,core
Kabuli_wild_56706490	1399941224	*Ca_kabuli_Chr06*	56706490	A/T	Gene_Ca_17409	INTRON	Rab-GAP/TBCdomain
Kabuli_wild_56793021	1399941225	*Ca_kabuli_Chr06*	56793021	G/T	NA	INTERGENIC	NA
Kabuli_wild_56793037	1399941226	*Ca_kabuli_Chr06*	56793037	A/T	NA	INTERGENIC	NA
Kabuli_wild_56793077	1399941227	*Ca_kabuli_Chr06*	56793077	G/A	NA	INTERGENIC	NA
Kabuli_wild_56793122	1399941228	*Ca_kabuli_Chr06*	56793122	C/T	NA	INTERGENIC	NA
Kabuli_wild_56822060	1399941229	*Ca_kabuli_Chr06*	56822060	G/A	Gene_Ca_17420	INTRON	Domain of unknown function DUF231,plant
Kabuli_wild_56822084	1399941230	*Ca_kabuli_Chr06*	56822084	G/T	Gene_Ca_17420	INTRON	Domain of unknown function DUF231,plant
Kabuli_wild_56840030	1399941231	*Ca_kabuli_Chr06*	56840030	A/C	NA	INTERGENIC	NA
Kabuli_wild_56840063	1399941232	*Ca_kabuli_Chr06*	56840063	T/A	NA	INTERGENIC	NA
Kabuli_wild_56854753	1399941233	*Ca_kabuli_Chr06*	56854753	T/C	Gene_Ca_17422	SYNONYMOUS_CODING	NA
Kabuli_wild_56918016	1399941234	*Ca_kabuli_Chr06*	56918016	C/T	NA	INTERGENIC	NA
Kabuli_wild_56950487	1399941235	*Ca_kabuli_Chr06*	56950487	G/T	NA	INTERGENIC	NA
Kabuli_wild_56959697	1399941236	*Ca_kabuli_Chr06*	56959697	G/A	NA	INTERGENIC	NA
Kabuli_wild_56980286	1399941237	*Ca_kabuli_Chr06*	56980286	T/C	Gene_Ca_17429	INTRON	GDP-fucoseproteinO-fucosyltransferase
Kabuli_wild_56980313	1399941238	*Ca_kabuli_Chr06*	56980313	T/C	Gene_Ca_17429	INTRON	GDP-fucoseproteinO-fucosyltransferase
Kabuli_wild_56980321	1399941239	*Ca_kabuli_Chr06*	56980321	T/C	Gene_Ca_17429	INTRON	GDP-fucoseproteinO-fucosyltransferase
Kabuli_wild_57198837	1399941240	*Ca_kabuli_Chr06*	57198837	G/A	Gene_Ca_17442	INTRON	NA
Kabuli_wild_57272593	1399941241	*Ca_kabuli_Chr06*	57272593	C/A	NA	INTERGENIC	NA
Kabuli_wild_57272601	1399941242	*Ca_kabuli_Chr06*	57272601	T/C	NA	INTERGENIC	NA
Kabuli_wild_57272604	1399941243	*Ca_kabuli_Chr06*	57272604	A/G	NA	INTERGENIC	NA
Kabuli_wild_57272605	1399941244	*Ca_kabuli_Chr06*	57272605	C/T	NA	INTERGENIC	NA
Kabuli_wild_57272607	1399941245	*Ca_kabuli_Chr06*	57272607	G/A	NA	INTERGENIC	NA
Kabuli_wild_57272608	1399941246	*Ca_kabuli_Chr06*	57272608	C/A	NA	INTERGENIC	NA
Kabuli_wild_57277440	1399941247	*Ca_kabuli_Chr06*	57277440	C/T	Gene_Ca_13709	DOWNSTREAM	Integrase,catalyticcore
Kabuli_wild_57382220	1399941248	*Ca_kabuli_Chr06*	57382220	G/A	Gene_Ca_13705	NON_SYNONYMOUS_CODING	Zinc finger, RING-type
Kabuli_wild_57425447	1399941249	*Ca_kabuli_Chr06*	57425447	T/C	Gene_Ca_13701	INTRON	NA
Kabuli_wild_57425496	1399941250	*Ca_kabuli_Chr06*	57425496	T/C	Gene_Ca_13701	INTRON	NA
Kabuli_wild_57436320	1399941251	*Ca_kabuli_Chr06*	57436320	T/G	Gene_Ca_13700	NON_SYNONYMOUS_CODING	Bromodomain
Kabuli_wild_57437904	1399941252	*Ca_kabuli_Chr06*	57437904	A/G	Gene_Ca_13700	SYNONYMOUS_CODING	Bromodomain
Kabuli_wild_57438007	1399941253	*Ca_kabuli_Chr06*	57438007	C/T	Gene_Ca_13700	NON_SYNONYMOUS_CODING	Bromodomain
Kabuli_wild_57449892	1399941254	*Ca_kabuli_Chr06*	57449892	A/G	Gene_Ca_13699	NON_SYNONYMOUS_CODING	NA
Kabuli_wild_57533087	1399941255	*Ca_kabuli_Chr06*	57533087	G/A	NA	INTERGENIC	NA
Kabuli_wild_57579768	1399941256	*Ca_kabuli_Chr06*	57579768	C/T	Gene_Ca_13686	SYNONYMOUS_CODING	RNA recognition motif domain
Kabuli_wild_57579770	1399941257	*Ca_kabuli_Chr06*	57579770	G/T	Gene_Ca_13686	SYNONYMOUS_CODING	RNA recognition motif domain
Kabuli_wild_57603257	1399941258	*Ca_kabuli_Chr06*	57603257	T/A	Gene_Ca_13685	INTRON	3-betahydroxysteroiddehydrogenase/isomerase
Kabuli_wild_57625134	1399941259	*Ca_kabuli_Chr06*	57625134	G/A	Gene_Ca_13683	INTRON	Calreticulin/calnexin
Kabuli_wild_57625320	1399941260	*Ca_kabuli_Chr06*	57625320	G/A	Gene_Ca_13683	SYNONYMOUS_CODING	Calreticulin/calnexin
Kabuli_wild_57632486	1399941261	*Ca_kabuli_Chr06*	57632486	C/T	Gene_Ca_13682	SYNONYMOUS_CODING	Protein of unknown function DUF936,plant
Kabuli_wild_57632584	1399941262	*Ca_kabuli_Chr06*	57632584	T/C	Gene_Ca_13682	INTRON	Protein of unknown function DUF936,plant
Kabuli_wild_57634387	1399941263	*Ca_kabuli_Chr06*	57634387	G/T	Gene_Ca_13682	NON_SYNONYMOUS_CODING	Protein of unknown function DUF936,plant

SNP IDs	NCBI dbSNP accession IDs (NCBI_SS#)	Chromosomes	Physical positions (bp)	SNPs	Gene accession IDs	Sequence components of genome	Putative functions
Kabuli_wild_57634677	1399941264	*Ca_kabuli_Chr06*	57634677	G/A	Gene_Ca_13682	SYNONYMOUS_CODING	Protein of unknown function DUF936,plant
Kabuli_wild_57637075	1399941265	*Ca_kabuli_Chr06*	57637075	C/T	NA	INTERGENIC	NA
Kabuli_wild_57637083	1399941266	*Ca_kabuli_Chr06*	57637083	T/G	NA	INTERGENIC	NA
Kabuli_wild_57637091	1399941267	*Ca_kabuli_Chr06*	57637091	G/A	NA	INTERGENIC	NA
Kabuli_wild_57667515	1399941268	*Ca_kabuli_Chr06*	57667515	A/T	NA	INTERGENIC	NA
Kabuli_wild_57704057	1399941269	*Ca_kabuli_Chr06*	57704057	C/T	NA	INTERGENIC	NA
Kabuli_wild_57704802	1399941270	*Ca_kabuli_Chr06*	57704802	T/C	Gene_Ca_13673	UPSTREAM	Triggerfactor,ribosome-binding,bacterial
Kabuli_wild_57704994	1399941271	*Ca_kabuli_Chr06*	57704994	A/G	Gene_Ca_13673	UPSTREAM	Triggerfactor,ribosome-binding,bacterial
Kabuli_wild_57705011	1399941272	*Ca_kabuli_Chr06*	57705011	G/A	Gene_Ca_13673	UPSTREAM	Triggerfactor,ribosome-binding,bacterial
Kabuli_wild_57707716	1399941273	*Ca_kabuli_Chr06*	57707716	C/T	Gene_Ca_13674	DOWNSTREAM	Orn/Lys/Argdecarboxylase,majordomain
Kabuli_wild_57707724	1399941274	*Ca_kabuli_Chr06*	57707724	A/C	Gene_Ca_13674	DOWNSTREAM	Orn/Lys/Argdecarboxylase,majordomain
Kabuli_wild_57707727	1399941275	*Ca_kabuli_Chr06*	57707727	T/G	Gene_Ca_13674	DOWNSTREAM	Orn/Lys/Argdecarboxylase,majordomain
Kabuli_wild_57707846	1399941276	*Ca_kabuli_Chr06*	57707846	G/A	NA	INTERGENIC	NA
Kabuli_wild_57707848	1399941277	*Ca_kabuli_Chr06*	57707848	A/G	NA	INTERGENIC	NA
Kabuli_wild_57707912	1399941278	*Ca_kabuli_Chr06*	57707912	G/C	NA	INTERGENIC	NA
Kabuli_wild_57708019	1399941279	*Ca_kabuli_Chr06*	57708019	A/G	NA	INTERGENIC	NA
Kabuli_wild_57708101	1399941280	*Ca_kabuli_Chr06*	57708101	T/G	NA	INTERGENIC	NA
Kabuli_wild_57708130	1399941281	*Ca_kabuli_Chr06*	57708130	T/C	NA	INTERGENIC	NA
Kabuli_wild_57708198	1399941282	*Ca_kabuli_Chr06*	57708198	C/T	NA	INTERGENIC	NA
Kabuli_wild_57710133	1399941283	*Ca_kabuli_Chr06*	57710133	C/G	NA	INTERGENIC	NA
Kabuli_wild_57720161	1399941284	*Ca_kabuli_Chr06*	57720161	A/T	Gene_Ca_13671	SYNONYMOUS_CODING	Transcriptional factor B3
Kabuli_wild_57720446	1399941285	*Ca_kabuli_Chr06*	57720446	T/C	Gene_Ca_13671	SYNONYMOUS_CODING	Transcriptional factor B3
Kabuli_wild_57725358	1399941286	*Ca_kabuli_Chr06*	57725358	T/G	NA	INTERGENIC	NA
Kabuli_wild_57746989	1399941287	*Ca_kabuli_Chr06*	57746989	C/T	Gene_Ca_13666	NON_SYNONYMOUS_CODING	NA
Kabuli_wild_57747101	1399941288	*Ca_kabuli_Chr06*	57747101	C/T	Gene_Ca_13666	SYNONYMOUS_CODING	NA
Kabuli_wild_57748565	1399941289	*Ca_kabuli_Chr06*	57748565	A/G	Gene_Ca_13666	SYNONYMOUS_CODING	NA
Kabuli_wild_57748602	1399941290	*Ca_kabuli_Chr06*	57748602	A/T	Gene_Ca_13666	NON_SYNONYMOUS_CODING	NA
Kabuli_wild_57753076	1399941291	*Ca_kabuli_Chr06*	57753076	T/C	Gene_Ca_13665	UPSTREAM	Phosphatidylinositol3-/4-kinase,catalytic
Kabuli_wild_57753193	1399941292	*Ca_kabuli_Chr06*	57753193	C/T	Gene_Ca_13665	UPSTREAM	Phosphatidylinositol3-/4-kinase,catalytic
Kabuli_wild_57756158	1399941293	*Ca_kabuli_Chr06*	57756158	A/G	Gene_Ca_13665	NON_SYNONYMOUS_CODING	Phosphatidylinositol3-/4-kinase,catalytic
Kabuli_wild_57756699	1399941294	*Ca_kabuli_Chr06*	57756699	G/A	Gene_Ca_13665	INTRON	Phosphatidylinositol3-/4-kinase,catalytic
Kabuli_wild_57760176	1399941295	*Ca_kabuli_Chr06*	57760176	C/T	Gene_Ca_13665	INTRON	Phosphatidylinositol3-/4-kinase,catalytic
Kabuli_wild_57760216	1399941296	*Ca_kabuli_Chr06*	57760216	G/C	Gene_Ca_13665	NON_SYNONYMOUS_CODING	Phosphatidylinositol3-/4-kinase,catalytic
Kabuli_wild_57760227	1399941297	*Ca_kabuli_Chr06*	57760227	C/T	Gene_Ca_13665	SYNONYMOUS_CODING	Phosphatidylinositol3-/4-kinase,catalytic
Kabuli_wild_57760360	1399941298	*Ca_kabuli_Chr06*	57760360	A/G	Gene_Ca_13665	INTRON	Phosphatidylinositol3-/4-kinase,catalytic
Kabuli_wild_57761758	1399941299	*Ca_kabuli_Chr06*	57761758	G/A	Gene_Ca_13665	NON_SYNONYMOUS_CODING	Phosphatidylinositol3-/4-kinase,catalytic
Kabuli_wild_57766444	1399941300	*Ca_kabuli_Chr06*	57766444	C/T	NA	INTERGENIC	NA
Kabuli_wild_57766570	1399941301	*Ca_kabuli_Chr06*	57766570	C/T	NA	INTERGENIC	NA
Kabuli_wild_57766588	1399941302	*Ca_kabuli_Chr06*	57766588	G/A	NA	INTERGENIC	NA
Kabuli_wild_57766598	1399941303	*Ca_kabuli_Chr06*	57766598	G/A	NA	INTERGENIC	NA
Kabuli_wild_57801985	1399941304	*Ca_kabuli_Chr06*	57801985	A/C	Gene_Ca_13661	INTRON	Proteasomeassemblychaperone2
Kabuli_wild_57831040	1399941305	*Ca_kabuli_Chr06*	57831040	T/C	NA	INTERGENIC	NA
Kabuli_wild_57831049	1399941306	*Ca_kabuli_Chr06*	57831049	C/T	NA	INTERGENIC	NA
Kabuli_wild_57831050	1399941307	*Ca_kabuli_Chr06*	57831050	G/T	NA	INTERGENIC	NA

SNP IDs	NCBI dbSNP accession IDs (NCBI_SS#)	Chromosomes	Physical positions (bp)	SNPs	Gene accession IDs	Sequence components of genome	Putative functions
Kabuli_wild_57835093	1399941308	*Ca_kabuli_Chr06*	57835093	T/A	Gene_Ca_13657	DOWNSTREAM	ColdacclimationWCOR413
Kabuli_wild_57835120	1399941309	*Ca_kabuli_Chr06*	57835120	G/A	Gene_Ca_13657	DOWNSTREAM	ColdacclimationWCOR413
Kabuli_wild_57835132	1399941310	*Ca_kabuli_Chr06*	57835132	G/A	Gene_Ca_13657	DOWNSTREAM	ColdacclimationWCOR413
Kabuli_wild_57835899	1399941311	*Ca_kabuli_Chr06*	57835899	C/T	NA	INTERGENIC	NA
Kabuli_wild_57835917	1399941312	*Ca_kabuli_Chr06*	57835917	G/C	NA	INTERGENIC	NA
Kabuli_wild_57836024	1399941313	*Ca_kabuli_Chr06*	57836024	G/A	NA	INTERGENIC	NA
Kabuli_wild_57836237	1399941314	*Ca_kabuli_Chr06*	57836237	A/G	NA	INTERGENIC	NA
Kabuli_wild_57836240	1399941315	*Ca_kabuli_Chr06*	57836240	T/A	NA	INTERGENIC	NA
Kabuli_wild_57836284	1399941316	*Ca_kabuli_Chr06*	57836284	G/A	NA	INTERGENIC	NA
Kabuli_wild_57836314	1399941317	*Ca_kabuli_Chr06*	57836314	T/A	NA	INTERGENIC	NA
Kabuli_wild_57836316	1399941318	*Ca_kabuli_Chr06*	57836316	G/A	NA	INTERGENIC	NA
Kabuli_wild_57839564	1399941319	*Ca_kabuli_Chr06*	57839564	T/C	NA	INTERGENIC	NA
Kabuli_wild_57853458	1399941320	*Ca_kabuli_Chr06*	57853458	C/T	Gene_Ca_13655	SYNONYMOUS_CODING	Protein kinase,catalytic domain
Kabuli_wild_57870893	1399941321	*Ca_kabuli_Chr06*	57870893	C/A	NA	INTERGENIC	NA
Kabuli_wild_57877446	1399941322	*Ca_kabuli_Chr06*	57877446	C/A	NA	INTERGENIC	NA
Kabuli_wild_57882514	1399941323	*Ca_kabuli_Chr06*	57882514	C/T	NA	INTERGENIC	NA
Kabuli_wild_57882773	1399941324	*Ca_kabuli_Chr06*	57882773	G/T	NA	INTERGENIC	NA
Kabuli_wild_57894369	1399941325	*Ca_kabuli_Chr06*	57894369	G/T	Gene_Ca_13650	DOWNSTREAM	NA
Kabuli_wild_57894377	1399941326	*Ca_kabuli_Chr06*	57894377	A/G	Gene_Ca_13650	DOWNSTREAM	NA
Kabuli_wild_57894495	1399941327	*Ca_kabuli_Chr06*	57894495	C/G	NA	INTERGENIC	NA
Kabuli_wild_57894585	1399941328	*Ca_kabuli_Chr06*	57894585	T/G	NA	INTERGENIC	NA
Kabuli_wild_57894590	1399941329	*Ca_kabuli_Chr06*	57894590	G/C	NA	INTERGENIC	NA
Kabuli_wild_57899764	1399941330	*Ca_kabuli_Chr06*	57899764	A/T	Gene_Ca_13648	DOWNSTREAM	Domain of unknown function DUF547
Kabuli_wild_57904540	1399941331	*Ca_kabuli_Chr06*	57904540	T/G	NA	INTERGENIC	NA
Kabuli_wild_57931382	1399941332	*Ca_kabuli_Chr06*	57931382	A/T	Gene_Ca_13645	SYNONYMOUS_CODING	NmrA-like
Kabuli_wild_57943585	1399941333	*Ca_kabuli_Chr06*	57943585	A/T	Gene_Ca_13643	UPSTREAM	NA
Kabuli_wild_57943626	1399941334	*Ca_kabuli_Chr06*	57943626	G/A	Gene_Ca_13643	UPSTREAM	NA
Kabuli_wild_57943628	1399941335	*Ca_kabuli_Chr06*	57943628	A/C	Gene_Ca_13643	UPSTREAM	NA
Kabuli_wild_57960875	1399941336	*Ca_kabuli_Chr06*	57960875	G/A	Gene_Ca_13640	INTRON	WD40 repeat
Kabuli_wild_57960878	1399941337	*Ca_kabuli_Chr06*	57960878	G/A	Gene_Ca_13640	INTRON	WD40 repeat
Kabuli_wild_57968924	1399941338	*Ca_kabuli_Chr06*	57968924	A/T	Gene_Ca_13640	INTRON	WD40 repeat
Kabuli_wild_57994242	1399941339	*Ca_kabuli_Chr06*	57994242	G/A	NA	INTERGENIC	NA
Kabuli_wild_58029408	1399941340	*Ca_kabuli_Chr06*	58029408	G/A	NA	INTERGENIC	NA
Kabuli_wild_58036471	1399941341	*Ca_kabuli_Chr06*	58036471	C/T	Gene_Ca_13632	UPSTREAM	BURP
Kabuli_wild_58053528	1399941342	*Ca_kabuli_Chr06*	58053528	C/A	Gene_Ca_13631	INTRON	PeptidaseS14,ClpP
Kabuli_wild_58053568	1399941343	*Ca_kabuli_Chr06*	58053568	G/A	Gene_Ca_13631	SYNONYMOUS_CODING	PeptidaseS14,ClpP
Kabuli_wild_58053878	1399941344	*Ca_kabuli_Chr06*	58053878	T/A	NA	INTERGENIC	NA
Kabuli_wild_58110541	1399941345	*Ca_kabuli_Chr06*	58110541	T/C	NA	INTERGENIC	NA
Kabuli_wild_58110605	1399941346	*Ca_kabuli_Chr06*	58110605	C/T	NA	INTERGENIC	NA
Kabuli_wild_58159915	1399941347	*Ca_kabuli_Chr06*	58159915	T/C	Gene_Ca_13624	SYNONYMOUS_CODING	Clathrin/coatomeradaptor,adaptin-like,N-terminal
Kabuli_wild_58165739	1399941348	*Ca_kabuli_Chr06*	58165739	T/A	NA	INTERGENIC	NA
Kabuli_wild_58174702	1399941349	*Ca_kabuli_Chr06*	58174702	T/C	Gene_Ca_13623	INTRON	Glycosidehydrolase,family17
Kabuli_wild_58174737	1399941350	*Ca_kabuli_Chr06*	58174737	A/C	Gene_Ca_13623	INTRON	Glycosidehydrolase,family17
Kabuli_wild_58187822	1399941351	*Ca_kabuli_Chr06*	58187822	T/G	Gene_Ca_13622	INTRON	Adenylatecyclase-associatedCAP

SNP IDs	NCBI dbSNP accession IDs (NCBI_SS#)	Chromosomes	Physical positions (bp)	SNPs	Gene accession IDs	Sequence components of genome	Putative functions
Kabuli_wild_58187957	1399941352	*Ca_kabuli_Chr06*	58187957	A/G	Gene_Ca_13622	SYNONYMOUS_CODING	Adenylatecyclase-associatedCAP
Kabuli_wild_58188477	1399941353	*Ca_kabuli_Chr06*	58188477	A/T	Gene_Ca_13622	INTRON	Adenylatecyclase-associatedCAP
Kabuli_wild_58190725	1399941354	*Ca_kabuli_Chr06*	58190725	G/A	Gene_Ca_13622	INTRON	Adenylatecyclase-associatedCAP
Kabuli_wild_58190730	1399941355	*Ca_kabuli_Chr06*	58190730	G/A	Gene_Ca_13622	INTRON	Adenylatecyclase-associatedCAP
Kabuli_wild_58224023	1399941356	*Ca_kabuli_Chr06*	58224023	G/T	NA	INTERGENIC	NA
Kabuli_wild_58227006	1399941357	*Ca_kabuli_Chr06*	58227006	T/C	NA	INTERGENIC	NA
Kabuli_wild_58237503	1399941358	*Ca_kabuli_Chr06*	58237503	A/T	NA	INTERGENIC	NA
Kabuli_wild_58243505	1399941359	*Ca_kabuli_Chr06*	58243505	G/A	Gene_Ca_15368	SYNONYMOUS_CODING	Zinc finger,C2HC5-type
Kabuli_wild_58243652	1399941360	*Ca_kabuli_Chr06*	58243652	G/A	Gene_Ca_15368	INTRON	Mitochodrialtranscriptionterminationfactor-related
Kabuli_wild_58244128	1399941361	*Ca_kabuli_Chr06*	58244128	T/C	Gene_Ca_15368	INTRON	Mitochodrialtranscriptionterminationfactor-related
Kabuli_wild_58244129	1399941362	*Ca_kabuli_Chr06*	58244129	G/A	Gene_Ca_15368	INTRON	Mitochodrialtranscriptionterminationfactor-related
Kabuli_wild_58326344	1399941363	*Ca_kabuli_Chr06*	58326344	A/T	NA	INTERGENIC	NA
Kabuli_wild_58380367	1399941364	*Ca_kabuli_Chr06*	58380367	T/C	Gene_Ca_15376	SYNONYMOUS_CODING	Ribosoma lRNA methyltransferase RrmJ/FtsJ
Kabuli_wild_58380394	1399941365	*Ca_kabuli_Chr06*	58380394	G/A	Gene_Ca_15376	SYNONYMOUS_CODING	Ribosoma lRNA methyltransferase RrmJ/FtsJ
Kabuli_wild_58380563	1399941366	*Ca_kabuli_Chr06*	58380563	G/A	Gene_Ca_15376	NON_SYNONYMOUS_CODING	Ribosoma lRNA methyltransferase RrmJ/FtsJ
Kabuli_wild_58434138	1399941367	*Ca_kabuli_Chr06*	58434138	C/T	Gene_Ca_15379	STOP_GAINED	NA
Kabuli_wild_58434217	1399941368	*Ca_kabuli_Chr06*	58434217	C/G	Gene_Ca_15379	NON_SYNONYMOUS_CODING	NA
Kabuli_wild_58452843	1399941369	*Ca_kabuli_Chr06*	58452843	A/C	NA	INTERGENIC	NA
Kabuli_wild_58569569	1399941370	*Ca_kabuli_Chr06*	58569569	T/C	Gene_Ca_15398	SYNONYMOUS_CODING	Pre-mRNAprocessingribonucleoprotein,snoRNA-binding domain
Kabuli_wild_58569576	1399941371	*Ca_kabuli_Chr06*	58569576	G/T	Gene_Ca_15398	NON_SYNONYMOUS_CODING	Pre-mRNAprocessingribonucleoprotein,snoRNA-binding domain
Kabuli_wild_58571660	1399941372	*Ca_kabuli_Chr06*	58571660	T/C	Gene_Ca_15398	SYNONYMOUS_CODING	Pre-mRNAprocessingribonucleoprotein,snoRNA-binding domain
Kabuli_wild_58596821	1399941373	*Ca_kabuli_Chr06*	58596821	T/G	NA	INTERGENIC	NA
Kabuli_wild_58596878	1399941374	*Ca_kabuli_Chr06*	58596878	C/A	NA	INTERGENIC	NA
Kabuli_wild_58618628	1399941375	*Ca_kabuli_Chr06*	58618628	A/C	NA	INTERGENIC	NA
Kabuli_wild_58618921	1399941376	*Ca_kabuli_Chr06*	58618921	T/C	NA	INTERGENIC	NA
Kabuli_wild_58623095	1399941377	*Ca_kabuli_Chr06*	58623095	A/G	Gene_Ca_15407	INTRON	ATPase,V0/A0complex,116kDasubunit
Kabuli_wild_58623096	1399941378	*Ca_kabuli_Chr06*	58623096	A/T	Gene_Ca_15407	INTRON	ATPase,V0/A0complex,116kDasubunit
Kabuli_wild_58624183	1399941379	*Ca_kabuli_Chr06*	58624183	G/A	Gene_Ca_15407	INTRON	ATPase,V0/A0complex,116kDasubunit
Kabuli_wild_58624233	1399941380	*Ca_kabuli_Chr06*	58624233	C/A	Gene_Ca_15407	INTRON	ATPase,V0/A0complex,116kDasubunit
Kabuli_wild_58624299	1399941381	*Ca_kabuli_Chr06*	58624299	A/G	Gene_Ca_15407	INTRON	ATPase,V0/A0complex,116kDasubunit
Kabuli_wild_58650318	1399941382	*Ca_kabuli_Chr06*	58650318	A/C	Gene_Ca_15410	SYNONYMOUS_CODING	WD40 repeat
Kabuli_wild_58650459	1399941383	*Ca_kabuli_Chr06*	58650459	A/T	Gene_Ca_15410	NON_SYNONYMOUS_CODING	WD40 repeat
Kabuli_wild_58650491	1399941384	*Ca_kabuli_Chr06*	58650491	A/G	Gene_Ca_15410	NON_SYNONYMOUS_CODING	WD40 repeat
Kabuli_wild_58652881	1399941385	*Ca_kabuli_Chr06*	58652881	A/G	Gene_Ca_15410	SYNONYMOUS_CODING	WD40 repeat
Kabuli_wild_58672317	1399941386	*Ca_kabuli_Chr06*	58672317	A/G	NA	INTERGENIC	NA
Kabuli_wild_58675749	1399941387	*Ca_kabuli_Chr06*	58675749	T/C	Gene_Ca_15413	SYNONYMOUS_CODING	Kinesin,motordomain
Kabuli_wild_58676507	1399941388	*Ca_kabuli_Chr06*	58676507	A/G	Gene_Ca_15413	SYNONYMOUS_CODING	Kinesin,motordomain
Kabuli_wild_58676517	1399941389	*Ca_kabuli_Chr06*	58676517	A/C	Gene_Ca_15413	NON_SYNONYMOUS_CODING	Kinesin,motordomain
Kabuli_wild_58682397	1399941390	*Ca_kabuli_Chr06*	58682397	G/A	Gene_Ca_15413	INTRON	Kinesin,motordomain
Kabuli_wild_58732423	1399941391	*Ca_kabuli_Chr06*	58732423	C/T	Gene_Ca_15417	INTRON	NA
Kabuli_wild_58732424	1399941392	*Ca_kabuli_Chr06*	58732424	G/T	Gene_Ca_15417	INTRON	Multiantimicrobial extrusion protein
Kabuli_wild_58732451	1399941393	*Ca_kabuli_Chr06*	58732451	T/C	Gene_Ca_15417	SYNONYMOUS_CODING	Multiantimicrobial extrusion protein
Kabuli_wild_58732475	1399941394	*Ca_kabuli_Chr06*	58732475	G/A	Gene_Ca_15417	SYNONYMOUS_CODING	Multiantimicrobial extrusion protein
Kabuli_wild_58745356	1399941395	*Ca_kabuli_Chr06*	58745356	A/G	Gene_Ca_15419	SYNONYMOUS_CODING	Multiantimicrobial extrusion protein

SNP IDs	NCBI dbSNP accession IDs (NCBI_SS#)	Chromosomes	Physical positions (bp)	SNPs	Gene accession IDs	Sequence components of genome	Putative functions
Kabuli_wild_58745426	1399941396	*Ca_kabuli_Chr06*	58745426	T/C	Gene_Ca_15419	INTRON	Multiantimicrobial extrusion protein
Kabuli_wild_58745473	1399941397	*Ca_kabuli_Chr06*	58745473	A/G	Gene_Ca_15419	SYNONYMOUS_CODING	Multiantimicrobial extrusion protein
Kabuli_wild_58745583	1399941398	*Ca_kabuli_Chr06*	58745583	C/T	Gene_Ca_15419	NON_SYNONYMOUS_CODING	Multiantimicrobial extrusion protein
Kabuli_wild_58761879	1399941399	*Ca_kabuli_Chr06*	58761879	G/A	NA	INTERGENIC	NA
Kabuli_wild_58779344	1399941400	*Ca_kabuli_Chr06*	58779344	A/G	Gene_Ca_15421	INTRON	Srchomology-3domain
Kabuli_wild_58779389	1399941401	*Ca_kabuli_Chr06*	58779389	T/G	Gene_Ca_15421	SYNONYMOUS_CODING	Srchomology-3domain
Kabuli_wild_58820632	1399941402	*Ca_kabuli_Chr06*	58820632	A/C	NA	INTERGENIC	NA
Kabuli_wild_58844717	1399941403	*Ca_kabuli_Chr06*	58844717	T/C	NA	INTERGENIC	NA
Kabuli_wild_58844776	1399941404	*Ca_kabuli_Chr06*	58844776	C/G	NA	INTERGENIC	NA
Kabuli_wild_58878604	1399941405	*Ca_kabuli_Chr06*	58878604	C/G	Gene_Ca_15428	SYNONYMOUS_CODING	Ornithine/DAP/Argdecarboxylase
Kabuli_wild_58879570	1399941406	*Ca_kabuli_Chr06*	58879570	T/C	Gene_Ca_15428	SYNONYMOUS_CODING	Ornithine/DAP/Argdecarboxylase
Kabuli_wild_58903346	1399941407	*Ca_kabuli_Chr06*	58903346	G/A	Gene_Ca_15431	INTRON	Aldo/ketoreductase
Kabuli_wild_58903381	1399941408	*Ca_kabuli_Chr06*	58903381	G/A	Gene_Ca_15431	INTRON	Aldo/ketoreductase
Kabuli_wild_58903395	1399941409	*Ca_kabuli_Chr06*	58903395	A/G	Gene_Ca_15431	INTRON	Aldo/ketoreductase
Kabuli_wild_58903416	1399941410	*Ca_kabuli_Chr06*	58903416	T/G	Gene_Ca_15431	INTRON	Aldo/ketoreductase
Kabuli_wild_58903497	1399941411	*Ca_kabuli_Chr06*	58903497	G/C	Gene_Ca_15431	INTRON	Aldo/ketoreductase
Kabuli_wild_58903538	1399941412	*Ca_kabuli_Chr06*	58903538	G/A	Gene_Ca_15431	INTRON	Aldo/ketoreductase
Kabuli_wild_58907318	1399941413	*Ca_kabuli_Chr06*	58907318	C/A	Gene_Ca_15431	SYNONYMOUS_CODING	Aldo/ketoreductase
Kabuli_wild_58907544	1399941414	*Ca_kabuli_Chr06*	58907544	T/C	Gene_Ca_15431	INTRON	Aldo/ketoreductase
Kabuli_wild_58907606	1399941415	*Ca_kabuli_Chr06*	58907606	A/G	Gene_Ca_15431	INTRON	Aldo/ketoreductase
Kabuli_wild_58908024	1399941416	*Ca_kabuli_Chr06*	58908024	G/A	Gene_Ca_15431	SYNONYMOUS_CODING	Aldo/ketoreductase
Kabuli_wild_58908069	1399941417	*Ca_kabuli_Chr06*	58908069	G/A	Gene_Ca_15431	SYNONYMOUS_CODING	Aldo/ketoreductase
Kabuli_wild_58951079	1399941418	*Ca_kabuli_Chr06*	58951079	T/C	Gene_Ca_18874	SYNONYMOUS_CODING	Clathrin/coatomeradaptor,adaptin-like,N-terminal
Kabuli_wild_58953219	1399941419	*Ca_kabuli_Chr06*	58953219	A/G	Gene_Ca_18874	INTRON	Clathrin/coatomeradaptor,adaptin-like,N-terminal
Kabuli_wild_58984925	1399941420	*Ca_kabuli_Chr06*	58984925	A/C	NA	INTERGENIC	NA
Kabuli_wild_59003792	1399941421	*Ca_kabuli_Chr06*	59003792	A/C	Gene_Ca_18871	INTRON	RNA recognition motif domain
Kabuli_wild_59064798	1399941422	*Ca_kabuli_Chr06*	59064798	G/T	NA	INTERGENIC	NA
Kabuli_wild_59064815	1399941423	*Ca_kabuli_Chr06*	59064815	C/T	NA	INTERGENIC	NA
Kabuli_wild_59081169	1399941424	*Ca_kabuli_Chr06*	59081169	C/T	Gene_Ca_18866	SYNONYMOUS_CODING	Glycosyltransferase,family2
Kabuli_wild_59107007	1399941425	*Ca_kabuli_Chr06*	59107007	G/T	Gene_Ca_18863	NON_SYNONYMOUS_CODING	RNA-binding,CRMdomain
Kabuli_wild_59107027	1399941426	*Ca_kabuli_Chr06*	59107027	A/T	Gene_Ca_18863	NON_SYNONYMOUS_CODING	RNA-binding,CRMdomain
Kabuli_wild_59135944	1399941427	*Ca_kabuli_Chr06*	59135944	A/C	Gene_Ca_18861	SYNONYMOUS_CODING	Protein kinase,catalytic domain
Kabuli_wild_59178844	1399941428	*Ca_kabuli_Chr06*	59178844	A/G	NA	INTERGENIC	NA
Kabuli_wild_59178848	1399941429	*Ca_kabuli_Chr06*	59178848	G/C	NA	INTERGENIC	NA
Kabuli_wild_59193994	1399941430	*Ca_kabuli_Chr06*	59193994	G/T	Gene_Ca_18855	SYNONYMOUS_CODING	NA
Kabuli_wild_59194015	1399941431	*Ca_kabuli_Chr06*	59194015	C/G	Gene_Ca_18855	SYNONYMOUS_CODING	NA
Kabuli_wild_59194081	1399941432	*Ca_kabuli_Chr06*	59194081	T/A	Gene_Ca_18855	SYNONYMOUS_CODING	NA
Kabuli_wild_59219099	1399941433	*Ca_kabuli_Chr06*	59219099	T/C	Gene_Ca_18853	SYNONYMOUS_CODING	ErgosterolbiosynthesisERG4/ERG24
Kabuli_wild_59222759	1399941434	*Ca_kabuli_Chr06*	59222759	T/C	NA	INTERGENIC	NA
Kabuli_wild_59222848	1399941435	*Ca_kabuli_Chr06*	59222848	C/T	NA	INTERGENIC	NA
Kabuli_wild_59296368	1399941436	*Ca_kabuli_Chr06*	59296368	C/T	Gene_Ca_18847	NON_SYNONYMOUS_CODING	YTHdomain
Kabuli_wild_59360004	1399941437	*Ca_kabuli_Chr06*	59360004	A/T	NA	INTERGENIC	NA
Kabuli_wild_59407761	1399941438	*Ca_kabuli_Chr06*	59407761	G/A	NA	INTERGENIC	NA
Kabuli_wild_59407904	1399941439	*Ca_kabuli_Chr06*	59407904	A/T	NA	INTERGENIC	NA

SNP IDs	NCBI dbSNP accession IDs (NCBI_SS#)	Chromosomes	Physical positions (bp)	SNPs	Gene accession IDs	Sequence components of genome	Putative functions
Kabuli_wild_16881	1399941440	*Ca_kabuli_Chr07*	16881	A/G	Gene_Ca_20246	NON_SYNONYMOUS_CODING	RNAhelicase,ATP-dependent,DEAD-box,conservedsite
Kabuli_wild_122600	1399941441	*Ca_kabuli_Chr07*	122600	T/G	Gene_Ca_20232	SYNONYMOUS_CODING	MyosinII heavychain-like
Kabuli_wild_343562	1399941442	*Ca_kabuli_Chr07*	343562	T/A	Gene_Ca_24406	UPSTREAM	Retroviralaspartylprotease
Kabuli_wild_343588	1399941443	*Ca_kabuli_Chr07*	343588	C/T	Gene_Ca_24406	UPSTREAM	Retroviralaspartylprotease
Kabuli_wild_343627	1399941444	*Ca_kabuli_Chr07*	343627	C/T	Gene_Ca_24406	UPSTREAM	Retroviralaspartylprotease
Kabuli_wild_393001	1399941445	*Ca_kabuli_Chr07*	393001	C/T	Gene_Ca_24407	NON_SYNONYMOUS_CODING	Domain of unknown function DUF1767
Kabuli_wild_419280	1399941446	*Ca_kabuli_Chr07*	419280	C/T	NA	INTERGENIC	NA
Kabuli_wild_419283	1399941447	*Ca_kabuli_Chr07*	419283	G/A	NA	INTERGENIC	NA
Kabuli_wild_419301	1399941448	*Ca_kabuli_Chr07*	419301	C/T	NA	INTERGENIC	NA
Kabuli_wild_419316	1399941449	*Ca_kabuli_Chr07*	419316	A/G	NA	INTERGENIC	NA
Kabuli_wild_419334	1399941450	*Ca_kabuli_Chr07*	419334	A/C	NA	INTERGENIC	NA
Kabuli_wild_419355	1399941451	*Ca_kabuli_Chr07*	419355	G/A	NA	INTERGENIC	NA
Kabuli_wild_419364	1399941452	*Ca_kabuli_Chr07*	419364	T/C	NA	INTERGENIC	NA
Kabuli_wild_419385	1399941453	*Ca_kabuli_Chr07*	419385	T/G	NA	INTERGENIC	NA
Kabuli_wild_419462	1399941454	*Ca_kabuli_Chr07*	419462	G/A	NA	INTERGENIC	NA
Kabuli_wild_419628	1399941455	*Ca_kabuli_Chr07*	419628	A/C	NA	INTERGENIC	NA
Kabuli_wild_419692	1399941456	*Ca_kabuli_Chr07*	419692	G/A	NA	INTERGENIC	NA
Kabuli_wild_528521	1399941457	*Ca_kabuli_Chr07*	528521	G/A	Gene_Ca_24410	DOWNSTREAM	NA
Kabuli_wild_601475	1399941458	*Ca_kabuli_Chr07*	601475	G/C	NA	INTERGENIC	NA
Kabuli_wild_601496	1399941459	*Ca_kabuli_Chr07*	601496	C/T	NA	INTERGENIC	NA
Kabuli_wild_601502	1399941460	*Ca_kabuli_Chr07*	601502	G/T	NA	INTERGENIC	NA
Kabuli_wild_601513	1399941461	*Ca_kabuli_Chr07*	601513	C/G	NA	INTERGENIC	NA
Kabuli_wild_601516	1399941462	*Ca_kabuli_Chr07*	601516	G/A	NA	INTERGENIC	NA
Kabuli_wild_601521	1399941463	*Ca_kabuli_Chr07*	601521	G/A	NA	INTERGENIC	NA
Kabuli_wild_601538	1399941464	*Ca_kabuli_Chr07*	601538	C/A	NA	INTERGENIC	NA
Kabuli_wild_601545	1399941465	*Ca_kabuli_Chr07*	601545	C/T	NA	INTERGENIC	NA
Kabuli_wild_601551	1399941466	*Ca_kabuli_Chr07*	601551	T/C	NA	INTERGENIC	NA
Kabuli_wild_601560	1399941467	*Ca_kabuli_Chr07*	601560	C/G	NA	INTERGENIC	NA
Kabuli_wild_601563	1399941468	*Ca_kabuli_Chr07*	601563	T/A	NA	INTERGENIC	NA
Kabuli_wild_601586	1399941469	*Ca_kabuli_Chr07*	601586	T/C	NA	INTERGENIC	NA
Kabuli_wild_601601	1399941470	*Ca_kabuli_Chr07*	601601	C/T	NA	INTERGENIC	NA
Kabuli_wild_601612	1399941471	*Ca_kabuli_Chr07*	601612	G/A	NA	INTERGENIC	NA
Kabuli_wild_601622	1399941472	*Ca_kabuli_Chr07*	601622	G/C	NA	INTERGENIC	NA
Kabuli_wild_601643	1399941473	*Ca_kabuli_Chr07*	601643	G/T	NA	INTERGENIC	NA
Kabuli_wild_601647	1399941474	*Ca_kabuli_Chr07*	601647	C/T	NA	INTERGENIC	NA
Kabuli_wild_601657	1399941475	*Ca_kabuli_Chr07*	601657	T/C	NA	INTERGENIC	NA
Kabuli_wild_601709	1399941476	*Ca_kabuli_Chr07*	601709	A/C	NA	INTERGENIC	NA
Kabuli_wild_601714	1399941477	*Ca_kabuli_Chr07*	601714	A/T	NA	INTERGENIC	NA
Kabuli_wild_601718	1399941478	*Ca_kabuli_Chr07*	601718	G/C	NA	INTERGENIC	NA
Kabuli_wild_601719	1399941479	*Ca_kabuli_Chr07*	601719	G/T	NA	INTERGENIC	NA
Kabuli_wild_610723	1399941480	*Ca_kabuli_Chr07*	610723	G/T	Gene_Ca_24417	NON_SYNONYMOUS_CODING	Transcription facto rGRAS
Kabuli_wild_610737	1399941481	*Ca_kabuli_Chr07*	610737	C/A	Gene_Ca_24417	NON_SYNONYMOUS_CODING	Transcription facto rGRAS
Kabuli_wild_674791	1399941482	*Ca_kabuli_Chr07*	674791	A/T	Gene_Ca_03417	INTRON	HECT
Kabuli_wild_676785	1399941483	*Ca_kabuli_Chr07*	676785	G/T	Gene_Ca_03417	DOWNSTREAM	HECT

SNP IDs	NCBI dbSNP accession IDs (NCBI_SS#)	Chromosomes	Physical positions (bp)	SNPs	Gene accession IDs	Sequence components of genome	Putative functions
Kabuli_wild_676786	1399941484	*Ca_kabuli_Chr07*	676786	G/A	Gene_Ca_03417	DOWNSTREAM	HECT
Kabuli_wild_676790	1399941485	*Ca_kabuli_Chr07*	676790	T/C	Gene_Ca_03417	DOWNSTREAM	HECT
Kabuli_wild_767708	1399941486	*Ca_kabuli_Chr07*	767708	G/A	Gene_Ca_03402	STOP_GAINED	EPS15homology(EH)
Kabuli_wild_781409	1399941487	*Ca_kabuli_Chr07*	781409	A/G	NA	INTERGENIC	NA
Kabuli_wild_807110	1399941488	*Ca_kabuli_Chr07*	807110	T/C	Gene_Ca_03397	INTRON	Phospho fructokinase domain
Kabuli_wild_815526	1399941489	*Ca_kabuli_Chr07*	815526	C/A	Gene_Ca_03396	NON_SYNONYMOUS_CODING	NA
Kabuli_wild_831159	1399941490	*Ca_kabuli_Chr07*	831159	T/A	Gene_Ca_03395	DOWNSTREAM	PeptidaseS8/S53,subtilisin/kexin/sedolisin
Kabuli_wild_838581	1399941491	*Ca_kabuli_Chr07*	838581	C/A	NA	INTERGENIC	NA
Kabuli_wild_838651	1399941492	*Ca_kabuli_Chr07*	838651	C/A	NA	INTERGENIC	NA
Kabuli_wild_841988	1399941493	*Ca_kabuli_Chr07*	841988	T/C	Gene_Ca_03393	INTRON	Domain of unknown function DUF221
Kabuli_wild_865096	1399941494	*Ca_kabuli_Chr07*	865096	A/C	Gene_Ca_03392	INTRON	Aminoacyl-tRNAsynthetase,classIa
Kabuli_wild_873110	1399941495	*Ca_kabuli_Chr07*	873110	A/C	NA	INTERGENIC	NA
Kabuli_wild_975236	1399941496	*Ca_kabuli_Chr07*	975236	T/A	Gene_Ca_03381	INTRON	Multicopperoxidase,type1
Kabuli_wild_1096379	1399941497	*Ca_kabuli_Chr07*	1096379	C/T	Gene_Ca_03366	SYNONYMOUS_CODING	IQ motif,EF-handbinding site
Kabuli_wild_1096440	1399941498	*Ca_kabuli_Chr07*	1096440	T/C	Gene_Ca_03366	NON_SYNONYMOUS_CODING	IQ motif,EF-handbinding site
Kabuli_wild_1153267	1399941499	*Ca_kabuli_Chr07*	1153267	A/C	NA	INTERGENIC	NA
Kabuli_wild_1174240	1399941500	*Ca_kabuli_Chr07*	1174240	T/C	Gene_Ca_03356	INTRON	Helicase,C-terminal
Kabuli_wild_1184557	1399941501	*Ca_kabuli_Chr07*	1184557	A/C	Gene_Ca_03354	NON_SYNONYMOUS_CODING	Ubiquitin/SUMO-activatingenzymeE1
Kabuli_wild_1293601	1399941502	*Ca_kabuli_Chr07*	1293601	C/G	Gene_Ca_03339	SYNONYMOUS_CODING	Protein kinase,catalytic domain
Kabuli_wild_1350605	1399941503	*Ca_kabuli_Chr07*	1350605	C/T	Gene_Ca_03334	NON_SYNONYMOUS_CODING	PeptidaseC19,ubiquitincarboxyl-terminalhydrolase2
Kabuli_wild_1414206	1399941504	*Ca_kabuli_Chr07*	1414206	C/A	NA	INTERGENIC	NA
Kabuli_wild_1505965	1399941505	*Ca_kabuli_Chr07*	1505965	A/G	Gene_Ca_03315	INTRON	Pentatrico peptide repeat
Kabuli_wild_1511252	1399941506	*Ca_kabuli_Chr07*	1511252	C/T	Gene_Ca_03315	INTRON	Pentatrico peptide repeat
Kabuli_wild_1585622	1399941507	*Ca_kabuli_Chr07*	1585622	T/C	Gene_Ca_03309	UPSTREAM	RibonucleaseH2,subunitC
Kabuli_wild_1704780	1399941508	*Ca_kabuli_Chr07*	1704780	T/G	NA	INTERGENIC	NA
Kabuli_wild_1705197	1399941509	*Ca_kabuli_Chr07*	1705197	A/C	Gene_Ca_03296	NON_SYNONYMOUS_CODING	Cytochromecoxidase,subunitVIb
Kabuli_wild_1707283	1399941510	*Ca_kabuli_Chr07*	1707283	G/T	Gene_Ca_03296	NON_SYNONYMOUS_CODING	Cytochromecoxidase,subunitVIb
Kabuli_wild_1991578	1399941511	*Ca_kabuli_Chr07*	1991578	A/G	Gene_Ca_03275	NON_SYNONYMOUS_CODING	Zinc finger,B-box
Kabuli_wild_2045614	1399941512	*Ca_kabuli_Chr07*	2045614	T/C	NA	INTERGENIC	NA
Kabuli_wild_2075694	1399941513	*Ca_kabuli_Chr07*	2075694	A/C	NA	INTERGENIC	NA
Kabuli_wild_2103626	1399941514	*Ca_kabuli_Chr07*	2103626	C/T	Gene_Ca_03262	INTRON	Calmodulinbindingprotein-like
Kabuli_wild_2121515	1399941515	*Ca_kabuli_Chr07*	2121515	T/A	NA	INTERGENIC	NA
Kabuli_wild_2170700	1399941516	*Ca_kabuli_Chr07*	2170700	T/G	Gene_Ca_03253	INTRON	Porin,eukaryotictype
Kabuli_wild_2219829	1399941517	*Ca_kabuli_Chr07*	2219829	T/C	NA	INTERGENIC	NA
Kabuli_wild_2391350	1399941518	*Ca_kabuli_Chr07*	2391350	T/G	Gene_Ca_03236	INTRON	WD40 repeat
Kabuli_wild_2450980	1399941519	*Ca_kabuli_Chr07*	2450980	A/C	NA	INTERGENIC	NA
Kabuli_wild_2609831	1399941520	*Ca_kabuli_Chr07*	2609831	C/A	NA	INTERGENIC	NA
Kabuli_wild_2684213	1399941521	*Ca_kabuli_Chr07*	2684213	A/G	Gene_Ca_03210	INTRON	LisHdimerisationmotif
Kabuli_wild_2707564	1399941522	*Ca_kabuli_Chr07*	2707564	T/C	NA	INTERGENIC	NA
Kabuli_wild_2734680	1399941523	*Ca_kabuli_Chr07*	2734680	A/G	Gene_Ca_03205	INTRON	Protein kinase,catalytic domain
Kabuli_wild_2745652	1399941524	*Ca_kabuli_Chr07*	2745652	T/A	NA	INTERGENIC	NA
Kabuli_wild_2745656	1399941525	*Ca_kabuli_Chr07*	2745656	A/C	NA	INTERGENIC	NA
Kabuli_wild_2745665	1399941526	*Ca_kabuli_Chr07*	2745665	A/C	NA	INTERGENIC	NA
Kabuli_wild_2757247	1399941527	*Ca_kabuli_Chr07*	2757247	A/G	Gene_Ca_03202	NON_SYNONYMOUS_CODING	Transcription factor jumonji/aspartylbeta-hydroxylase

SNP IDs	NCBI dbSNP accession IDs (NCBI_SS#)	Chromosomes	Physical positions (bp)	SNPs	Gene accession IDs	Sequence components of genome	Putative functions
Kabuli_wild_2757253	1399941528	*Ca_kabuli_Chr07*	2757253	T/G	Gene_Ca_03202	NON_SYNONYMOUS_CODING	Transcription factor jumonji/aspartylbeta-hydroxylase
Kabuli_wild_2757255	1399941529	*Ca_kabuli_Chr07*	2757255	G/C	Gene_Ca_03202	NON_SYNONYMOUS_CODING	Transcription factor jumonji/aspartylbeta-hydroxylase
Kabuli_wild_2762396	1399941530	*Ca_kabuli_Chr07*	2762396	G/A	NA	INTERGENIC	NA
Kabuli_wild_2832369	1399941531	*Ca_kabuli_Chr07*	2832369	C/T	Gene_Ca_03193	SYNONYMOUS_CODING	Zinc finger,GATA-type
Kabuli_wild_2857630	1399941532	*Ca_kabuli_Chr07*	2857630	T/G	Gene_Ca_03190	INTRON	Protein kinase,catalytic domain
Kabuli_wild_2864694	1399941533	*Ca_kabuli_Chr07*	2864694	G/T	Gene_Ca_03189	NON_SYNONYMOUS_CODING	Zinc finger,CCCH-type
Kabuli_wild_2909176	1399941534	*Ca_kabuli_Chr07*	2909176	C/A	NA	INTERGENIC	NA
Kabuli_wild_2915375	1399941535	*Ca_kabuli_Chr07*	2915375	C/G	Gene_Ca_03185	UPSTREAM	DNArecombinationandrepairproteinRecA
Kabuli_wild_2915420	1399941536	*Ca_kabuli_Chr07*	2915420	C/T	Gene_Ca_03185	UPSTREAM	DNArecombinationandrepairproteinRecA
Kabuli_wild_3023154	1399941537	*Ca_kabuli_Chr07*	3023154	T/C	Gene_Ca_03170	INTRON	NA
Kabuli_wild_3070531	1399941538	*Ca_kabuli_Chr07*	3070531	A/C	NA	INTERGENIC	NA
Kabuli_wild_3093979	1399941539	*Ca_kabuli_Chr07*	3093979	A/G	Gene_Ca_03163	SYNONYMOUS_CODING	IQ motif,EF-handbinding site
Kabuli_wild_3094087	1399941540	*Ca_kabuli_Chr07*	3094087	A/G	Gene_Ca_03163	SYNONYMOUS_CODING	IQ motif,EF-handbinding site
Kabuli_wild_3109250	1399941541	*Ca_kabuli_Chr07*	3109250	T/C	Gene_Ca_03162	DOWNSTREAM	Peptidoglycan-bindingLysinsubgroup
Kabuli_wild_3109352	1399941542	*Ca_kabuli_Chr07*	3109352	T/C	Gene_Ca_03162	DOWNSTREAM	Peptidoglycan-bindingLysinsubgroup
Kabuli_wild_3130069	1399941543	*Ca_kabuli_Chr07*	3130069	A/G	Gene_Ca_03158	NON_SYNONYMOUS_CODING	PhotosystemIIPsbX
Kabuli_wild_3130070	1399941544	*Ca_kabuli_Chr07*	3130070	A/G	Gene_Ca_03158	NON_SYNONYMOUS_CODING	PhotosystemIIPsbX
Kabuli_wild_3143409	1399941545	*Ca_kabuli_Chr07*	3143409	C/T	NA	INTERGENIC	NA
Kabuli_wild_3186262	1399941546	*Ca_kabuli_Chr07*	3186262	T/G	NA	INTERGENIC	NA
Kabuli_wild_3186341	1399941547	*Ca_kabuli_Chr07*	3186341	C/T	NA	INTERGENIC	NA
Kabuli_wild_3297683	1399941548	*Ca_kabuli_Chr07*	3297683	C/T	Gene_Ca_03139	SYNONYMOUS_CODING	Domain of unknown function DUF231,plant
Kabuli_wild_3315157	1399941549	*Ca_kabuli_Chr07*	3315157	A/G	NA	INTERGENIC	NA
Kabuli_wild_3356399	1399941550	*Ca_kabuli_Chr07*	3356399	G/A	Gene_Ca_03128	INTRON	AUX/IAAprotein
Kabuli_wild_3357452	1399941551	*Ca_kabuli_Chr07*	3357452	G/A	Gene_Ca_03128	NON_SYNONYMOUS_CODING	AUX/IAAprotein
Kabuli_wild_3374547	1399941552	*Ca_kabuli_Chr07*	3374547	G/T	Gene_Ca_03127	INTRON	NUDIX hydrolase domain
Kabuli_wild_3374551	1399941553	*Ca_kabuli_Chr07*	3374551	C/T	Gene_Ca_03127	INTRON	NUDIX hydrolase domain
Kabuli_wild_3504900	1399941554	*Ca_kabuli_Chr07*	3504900	G/A	NA	INTERGENIC	NA
Kabuli_wild_3505012	1399941555	*Ca_kabuli_Chr07*	3505012	A/C	NA	INTERGENIC	NA
Kabuli_wild_3518048	1399941556	*Ca_kabuli_Chr07*	3518048	G/A	Gene_Ca_03116	SYNONYMOUS_CODING	Synaptojanin,N-terminal
Kabuli_wild_3519510	1399941557	*Ca_kabuli_Chr07*	3519510	A/G	Gene_Ca_03116	INTRON	Synaptojanin,N-terminal
Kabuli_wild_3519701	1399941558	*Ca_kabuli_Chr07*	3519701	A/C	Gene_Ca_03116	INTRON	Synaptojanin,N-terminal
Kabuli_wild_3520759	1399941559	*Ca_kabuli_Chr07*	3520759	C/T	Gene_Ca_03116	INTRON	Synaptojanin,N-terminal
Kabuli_wild_3521150	1399941560	*Ca_kabuli_Chr07*	3521150	A/G	Gene_Ca_03116	INTRON	Synaptojanin,N-terminal
Kabuli_wild_3521167	1399941561	*Ca_kabuli_Chr07*	3521167	C/G	Gene_Ca_03116	INTRON	Synaptojanin,N-terminal
Kabuli_wild_3521192	1399941562	*Ca_kabuli_Chr07*	3521192	C/A	Gene_Ca_03116	INTRON	Synaptojanin,N-terminal
Kabuli_wild_3521257	1399941563	*Ca_kabuli_Chr07*	3521257	G/A	Gene_Ca_03116	INTRON	Synaptojanin,N-terminal
Kabuli_wild_3536194	1399941564	*Ca_kabuli_Chr07*	3536194	C/T	NA	INTERGENIC	NA
Kabuli_wild_3536233	1399941565	*Ca_kabuli_Chr07*	3536233	T/C	NA	INTERGENIC	NA
Kabuli_wild_3543765	1399941566	*Ca_kabuli_Chr07*	3543765	A/G	NA	INTERGENIC	NA
Kabuli_wild_3543768	1399941567	*Ca_kabuli_Chr07*	3543768	T/C	NA	INTERGENIC	NA
Kabuli_wild_3557570	1399941568	*Ca_kabuli_Chr07*	3557570	C/T	Gene_Ca_03113	INTRON	Zinc finger,FYVE-type
Kabuli_wild_3560182	1399941569	*Ca_kabuli_Chr07*	3560182	G/A	Gene_Ca_03113	NON_SYNONYMOUS_CODING	Zinc finger,FYVE-type
Kabuli_wild_3572963	1399941570	*Ca_kabuli_Chr07*	3572963	T/C	Gene_Ca_03112	INTRON	Sec34-likeprotein
Kabuli_wild_3573456	1399941571	*Ca_kabuli_Chr07*	3573456	G/A	Gene_Ca_03112	INTRON	Sec34-likeprotein

SNP IDs	NCBI dbSNP accession IDs (NCBI_SS#)	Chromosomes	Physical positions (bp)	SNPs	Gene accession IDs	Sequence components of genome	Putative functions
Kabuli_wild_3573477	1399941572	*Ca_kabuli_Chr07*	3573477	C/G	Gene_Ca_03112	INTRON	Sec34-likeprotein
Kabuli_wild_3581123	1399941573	*Ca_kabuli_Chr07*	3581123	G/A	Gene_Ca_03112	INTRON	Sec34-likeprotein
Kabuli_wild_3600826	1399941574	*Ca_kabuli_Chr07*	3600826	C/T	NA	INTERGENIC	NA
Kabuli_wild_3600833	1399941575	*Ca_kabuli_Chr07*	3600833	T/G	NA	INTERGENIC	NA
Kabuli_wild_3600866	1399941576	*Ca_kabuli_Chr07*	3600866	T/C	NA	INTERGENIC	NA
Kabuli_wild_3600867	1399941577	*Ca_kabuli_Chr07*	3600867	C/T	NA	INTERGENIC	NA
Kabuli_wild_3600987	1399941578	*Ca_kabuli_Chr07*	3600987	A/C	NA	INTERGENIC	NA
Kabuli_wild_3628086	1399941579	*Ca_kabuli_Chr07*	3628086	G/A	Gene_Ca_03106	INTRON	Saccharopinedehydrogenase/Homospermidinesynthase
Kabuli_wild_3634037	1399941580	*Ca_kabuli_Chr07*	3634037	C/T	Gene_Ca_03106	INTRON	Saccharopinedehydrogenase/Homospermidinesynthase
Kabuli_wild_3634052	1399941581	*Ca_kabuli_Chr07*	3634052	C/T	Gene_Ca_03106	INTRON	Saccharopinedehydrogenase/Homospermidinesynthase
Kabuli_wild_3634427	1399941582	*Ca_kabuli_Chr07*	3634427	G/C	Gene_Ca_03106	SYNONYMOUS_CODING	Saccharopinedehydrogenase/Homospermidinesynthase
Kabuli_wild_3634430	1399941583	*Ca_kabuli_Chr07*	3634430	A/T	Gene_Ca_03106	NON_SYNONYMOUS_CODING	Saccharopinedehydrogenase/Homospermidinesynthase
Kabuli_wild_3634459	1399941584	*Ca_kabuli_Chr07*	3634459	T/C	Gene_Ca_03106	NON_SYNONYMOUS_CODING	Saccharopinedehydrogenase/Homospermidinesynthase
Kabuli_wild_3634478	1399941585	*Ca_kabuli_Chr07*	3634478	A/G	Gene_Ca_03106	SYNONYMOUS_CODING	Saccharopinedehydrogenase/Homospermidinesynthase
Kabuli_wild_3634497	1399941586	*Ca_kabuli_Chr07*	3634497	A/G	Gene_Ca_03106	NON_SYNONYMOUS_CODING	Saccharopinedehydrogenase/Homospermidinesynthase
Kabuli_wild_3635307	1399941587	*Ca_kabuli_Chr07*	3635307	G/A	Gene_Ca_03106	INTRON	Saccharopinedehydrogenase/Homospermidinesynthase
Kabuli_wild_3635309	1399941588	*Ca_kabuli_Chr07*	3635309	C/T	Gene_Ca_03106	INTRON	Saccharopinedehydrogenase/Homospermidinesynthase
Kabuli_wild_3635318	1399941589	*Ca_kabuli_Chr07*	3635318	T/A	Gene_Ca_03106	INTRON	Saccharopinedehydrogenase/Homospermidinesynthase
Kabuli_wild_3635325	1399941590	*Ca_kabuli_Chr07*	3635325	T/C	Gene_Ca_03106	INTRON	Saccharopinedehydrogenase/Homospermidinesynthase
Kabuli_wild_3635369	1399941591	*Ca_kabuli_Chr07*	3635369	G/T	Gene_Ca_03106	INTRON	Saccharopinedehydrogenase/Homospermidinesynthase
Kabuli_wild_3650893	1399941592	*Ca_kabuli_Chr07*	3650893	T/A	Gene_Ca_03103	INTRON	SEC7-like
Kabuli_wild_3651051	1399941593	*Ca_kabuli_Chr07*	3651051	T/C	Gene_Ca_03103	INTRON	SEC7-like
Kabuli_wild_3665651	1399941594	*Ca_kabuli_Chr07*	3665651	G/C	Gene_Ca_03103	SYNONYMOUS_CODING	SEC7-like
Kabuli_wild_3672192	1399941595	*Ca_kabuli_Chr07*	3672192	T/C	Gene_Ca_03102	INTRON	Zinc finger,FYVE-type
Kabuli_wild_3672236	1399941596	*Ca_kabuli_Chr07*	3672236	G/A	Gene_Ca_03102	INTRON	Zinc finger,FYVE-type
Kabuli_wild_3796826	1399941597	*Ca_kabuli_Chr07*	3796826	A/G	Gene_Ca_03085	INTRON	SNF2-related
Kabuli_wild_3802188	1399941598	*Ca_kabuli_Chr07*	3802188	T/A	NA	INTERGENIC	NA
Kabuli_wild_3817112	1399941599	*Ca_kabuli_Chr07*	3817112	C/T	Gene_Ca_03082	NON_SYNONYMOUS_CODING	ABC transporter,trans membrane domain
Kabuli_wild_3817733	1399941600	*Ca_kabuli_Chr07*	3817733	T/G	Gene_Ca_03082	NON_SYNONYMOUS_CODING	ABC transporter,trans membrane domain
Kabuli_wild_3817740	1399941601	*Ca_kabuli_Chr07*	3817740	G/T	Gene_Ca_03082	NON_SYNONYMOUS_CODING	ABC transporter,trans membrane domain
Kabuli_wild_3817743	1399941602	*Ca_kabuli_Chr07*	3817743	C/A	Gene_Ca_03082	SYNONYMOUS_CODING	ABC transporter,trans membrane domain
Kabuli_wild_3867250	1399941603	*Ca_kabuli_Chr07*	3867250	A/G	Gene_Ca_03077	NON_SYNONYMOUS_CODING	NA
Kabuli_wild_3867273	1399941604	*Ca_kabuli_Chr07*	3867273	A/T	Gene_Ca_03077	SYNONYMOUS_CODING	NA
Kabuli_wild_3867279	1399941605	*Ca_kabuli_Chr07*	3867279	T/C	Gene_Ca_03077	SYNONYMOUS_CODING	NA
Kabuli_wild_3867377	1399941606	*Ca_kabuli_Chr07*	3867377	A/G	Gene_Ca_03077	NON_SYNONYMOUS_CODING	NA
Kabuli_wild_3883678	1399941607	*Ca_kabuli_Chr07*	3883678	C/T	Gene_Ca_03076	DOWNSTREAM	Protein kinase,catalytic domain
Kabuli_wild_3916849	1399941608	*Ca_kabuli_Chr07*	3916849	A/C	NA	INTERGENIC	NA
Kabuli_wild_4017780	1399941609	*Ca_kabuli_Chr07*	4017780	T/C	Gene_Ca_03061	INTRON	NA
Kabuli_wild_4017838	1399941610	*Ca_kabuli_Chr07*	4017838	T/G	Gene_Ca_03061	INTRON	NA
Kabuli_wild_4017851	1399941611	*Ca_kabuli_Chr07*	4017851	T/C	Gene_Ca_03061	INTRON	NA
Kabuli_wild_4017920	1399941612	*Ca_kabuli_Chr07*	4017920	A/C	Gene_Ca_03061	INTRON	NA
Kabuli_wild_4028506	1399941613	*Ca_kabuli_Chr07*	4028506	T/C	NA	INTERGENIC	NA
Kabuli_wild_4095293	1399941614	*Ca_kabuli_Chr07*	4095293	A/G	Gene_Ca_03057	NON_SYNONYMOUS_CODING	Galectin,carbohydraterecognitiondomain
Kabuli_wild_4183185	1399941615	*Ca_kabuli_Chr07*	4183185	G/A	Gene_Ca_03046	INTRON	Cas1p-like

SNP IDs	NCBI dbSNP accession IDs (NCBI_SS#)	Chromosomes	Physical positions (bp)	SNPs	Gene accession IDs	Sequence components of genome	Putative functions
Kabuli_wild_4194809	1399941616	*Ca_kabuli_Chr07*	4194809	T/A	Gene_Ca_03043	DOWNSTREAM	NA
Kabuli_wild_4194890	1399941617	*Ca_kabuli_Chr07*	4194890	C/A	Gene_Ca_03043	DOWNSTREAM	NA
Kabuli_wild_4194891	1399941618	*Ca_kabuli_Chr07*	4194891	C/G	Gene_Ca_03043	DOWNSTREAM	NA
Kabuli_wild_4198812	1399941619	*Ca_kabuli_Chr07*	4198812	A/C	Gene_Ca_03043	UPSTREAM	NA
Kabuli_wild_4209425	1399941620	*Ca_kabuli_Chr07*	4209425	C/T	NA	INTERGENIC	NA
Kabuli_wild_4209465	1399941621	*Ca_kabuli_Chr07*	4209465	A/C	NA	INTERGENIC	NA
Kabuli_wild_4300584	1399941622	*Ca_kabuli_Chr07*	4300584	C/T	Gene_Ca_03028	DOWNSTREAM	Integrase,catalyticcore
Kabuli_wild_4300592	1399941623	*Ca_kabuli_Chr07*	4300592	G/A	Gene_Ca_03028	DOWNSTREAM	Integrase,catalyticcore
Kabuli_wild_4300645	1399941624	*Ca_kabuli_Chr07*	4300645	C/T	Gene_Ca_03028	DOWNSTREAM	Integrase,catalyticcore
Kabuli_wild_4300650	1399941625	*Ca_kabuli_Chr07*	4300650	T/C	Gene_Ca_03028	DOWNSTREAM	Integrase,catalyticcore
Kabuli_wild_4305424	1399941626	*Ca_kabuli_Chr07*	4305424	G/T	NA	INTERGENIC	NA
Kabuli_wild_4431138	1399941627	*Ca_kabuli_Chr07*	4431138	A/G	Gene_Ca_03015	NON_SYNONYMOUS_CODING	NA
Kabuli_wild_4461990	1399941628	*Ca_kabuli_Chr07*	4461990	A/C	Gene_Ca_03012	DOWNSTREAM	NA
Kabuli_wild_4498899	1399941629	*Ca_kabuli_Chr07*	4498899	G/A	NA	INTERGENIC	NA
Kabuli_wild_4520617	1399941630	*Ca_kabuli_Chr07*	4520617	C/A	Gene_Ca_03001	NON_SYNONYMOUS_CODING	Pyridine nucleotide-disulphideoxido reductase,NAD-binding region
Kabuli_wild_4552790	1399941631	*Ca_kabuli_Chr07*	4552790	T/C	NA	INTERGENIC	NA
Kabuli_wild_4573236	1399941632	*Ca_kabuli_Chr07*	4573236	G/A	NA	INTERGENIC	NA
Kabuli_wild_4625723	1399941633	*Ca_kabuli_Chr07*	4625723	C/T	Gene_Ca_02989	SYNONYMOUS_CODING	Signal transduction response regulator, receiverdomain
Kabuli_wild_4652083	1399941634	*Ca_kabuli_Chr07*	4652083	G/A	Gene_Ca_02988	INTRON	Protein of unknown function DUF789
Kabuli_wild_4707650	1399941635	*Ca_kabuli_Chr07*	4707650	T/G	Gene_Ca_02979	UPSTREAM	WD40 repeat
Kabuli_wild_4749212	1399941636	*Ca_kabuli_Chr07*	4749212	T/G	NA	INTERGENIC	NA
Kabuli_wild_4764034	1399941637	*Ca_kabuli_Chr07*	4764034	G/T	NA	INTERGENIC	NA
Kabuli_wild_4834177	1399941638	*Ca_kabuli_Chr07*	4834177	G/T	Gene_Ca_02961	UPSTREAM	Lateembryogenesisabundantprotein,group3
Kabuli_wild_4884236	1399941639	*Ca_kabuli_Chr07*	4884236	A/C	NA	INTERGENIC	NA
Kabuli_wild_4981846	1399941640	*Ca_kabuli_Chr07*	4981846	T/C	NA	INTERGENIC	NA
Kabuli_wild_4981870	1399941641	*Ca_kabuli_Chr07*	4981870	C/A	NA	INTERGENIC	NA
Kabuli_wild_4981871	1399941642	*Ca_kabuli_Chr07*	4981871	C/G	NA	INTERGENIC	NA
Kabuli_wild_5057515	1399941643	*Ca_kabuli_Chr07*	5057515	G/T	Gene_Ca_06859	NON_SYNONYMOUS_CODING	Peptidase C48, SUMO/Sentrin/Ubl1
Kabuli_wild_5106231	1399941644	*Ca_kabuli_Chr07*	5106231	G/A	Gene_Ca_06855	DOWNSTREAM	Transcriptionalcoactivator/pterindehydratase
Kabuli_wild_5122426	1399941645	*Ca_kabuli_Chr07*	5122426	A/G	NA	INTERGENIC	NA
Kabuli_wild_5122536	1399941646	*Ca_kabuli_Chr07*	5122536	T/G	NA	INTERGENIC	NA
Kabuli_wild_5383564	1399941647	*Ca_kabuli_Chr07*	5383564	G/C	NA	INTERGENIC	NA
Kabuli_wild_5383566	1399941648	*Ca_kabuli_Chr07*	5383566	A/T	NA	INTERGENIC	NA
Kabuli_wild_5383671	1399941649	*Ca_kabuli_Chr07*	5383671	G/T	NA	INTERGENIC	NA
Kabuli_wild_5383695	1399941650	*Ca_kabuli_Chr07*	5383695	T/G	NA	INTERGENIC	NA
Kabuli_wild_5383767	1399941651	*Ca_kabuli_Chr07*	5383767	G/A	NA	INTERGENIC	NA
Kabuli_wild_5384447	1399941652	*Ca_kabuli_Chr07*	5384447	G/C	Gene_Ca_06832	DOWNSTREAM	Pentatrico peptide repeat
Kabuli_wild_5406524	1399941653	*Ca_kabuli_Chr07*	5406524	T/G	NA	INTERGENIC	NA
Kabuli_wild_5421847	1399941654	*Ca_kabuli_Chr07*	5421847	A/C	Gene_Ca_06826	INTRON	SNF2-related
Kabuli_wild_5421861	1399941655	*Ca_kabuli_Chr07*	5421861	A/T	Gene_Ca_06826	INTRON	SNF2-related
Kabuli_wild_5464193	1399941656	*Ca_kabuli_Chr07*	5464193	G/A	NA	INTERGENIC	NA
Kabuli_wild_5464941	1399941657	*Ca_kabuli_Chr07*	5464941	G/A	Gene_Ca_06820	DOWNSTREAM	F-boxdomain,cyclin-like
Kabuli_wild_5464942	1399941658	*Ca_kabuli_Chr07*	5464942	A/G	Gene_Ca_06820	DOWNSTREAM	F-boxdomain,cyclin-like
Kabuli_wild_5467296	1399941659	*Ca_kabuli_Chr07*	5467296	T/A	NA	INTERGENIC	NA

SNP IDs	NCBI dbSNP accession IDs (NCBI_SS#)	Chromosomes	Physical positions (bp)	SNPs	Gene accession IDs	Sequence components of genome	Putative functions
Kabuli_wild_5467645	1399941660	*Ca_kabuli_Chr07*	5467645	G/A	NA	INTERGENIC	NA
Kabuli_wild_5467744	1399941661	*Ca_kabuli_Chr07*	5467744	G/C	NA	INTERGENIC	NA
Kabuli_wild_5474255	1399941662	*Ca_kabuli_Chr07*	5474255	C/T	NA	INTERGENIC	NA
Kabuli_wild_5500202	1399941663	*Ca_kabuli_Chr07*	5500202	A/G	Gene_Ca_06816	INTRON	SNF2-related
Kabuli_wild_5504454	1399941664	*Ca_kabuli_Chr07*	5504454	T/C	Gene_Ca_06816	INTRON	SNF2-related
Kabuli_wild_5508162	1399941665	*Ca_kabuli_Chr07*	5508162	A/C	Gene_Ca_06816	SYNONYMOUS_CODING	SNF2-related
Kabuli_wild_5509712	1399941666	*Ca_kabuli_Chr07*	5509712	T/G	Gene_Ca_06816	NON_SYNONYMOUS_CODING	SNF2-related
Kabuli_wild_5520720	1399941667	*Ca_kabuli_Chr07*	5520720	T/G	Gene_Ca_06814	NON_SYNONYMOUS_CODING	Heat shock protein DnaJ,N-terminal
Kabuli_wild_5520934	1399941668	*Ca_kabuli_Chr07*	5520934	T/C	Gene_Ca_06814	NON_SYNONYMOUS_CODING	Heat shock protein DnaJ,N-terminal
Kabuli_wild_5521596	1399941669	*Ca_kabuli_Chr07*	5521596	T/C	Gene_Ca_06814	NON_SYNONYMOUS_CODING	Heat shock protein DnaJ,N-terminal
Kabuli_wild_5521659	1399941670	*Ca_kabuli_Chr07*	5521659	G/T	Gene_Ca_06814	NON_SYNONYMOUS_CODING	Heat shock protein DnaJ,N-terminal
Kabuli_wild_5529110	1399941671	*Ca_kabuli_Chr07*	5529110	A/G	NA	INTERGENIC	NA
Kabuli_wild_5529573	1399941672	*Ca_kabuli_Chr07*	5529573	C/G	NA	INTERGENIC	NA
Kabuli_wild_5529577	1399941673	*Ca_kabuli_Chr07*	5529577	T/G	NA	INTERGENIC	NA
Kabuli_wild_5529729	1399941674	*Ca_kabuli_Chr07*	5529729	A/G	NA	INTERGENIC	NA
Kabuli_wild_5529844	1399941675	*Ca_kabuli_Chr07*	5529844	C/A	NA	INTERGENIC	NA
Kabuli_wild_5529888	1399941676	*Ca_kabuli_Chr07*	5529888	A/G	NA	INTERGENIC	NA
Kabuli_wild_5537851	1399941677	*Ca_kabuli_Chr07*	5537851	G/C	Gene_Ca_06811	INTRON	ProtamineP1
Kabuli_wild_5537880	1399941678	*Ca_kabuli_Chr07*	5537880	G/T	Gene_Ca_06811	INTRON	ProtamineP2
Kabuli_wild_5542812	1399941679	*Ca_kabuli_Chr07*	5542812	A/C	NA	INTERGENIC	NA
Kabuli_wild_5561607	1399941680	*Ca_kabuli_Chr07*	5561607	G/T	Gene_Ca_06808	INTRON	Inosine/uridine-preferringnucleosidehydrolasedomain
Kabuli_wild_5568114	1399941681	*Ca_kabuli_Chr07*	5568114	C/A	Gene_Ca_06807	UPSTREAM	Metridin-likeShKtoxin
Kabuli_wild_5577258	1399941682	*Ca_kabuli_Chr07*	5577258	T/G	NA	INTERGENIC	NA
Kabuli_wild_5577311	1399941683	*Ca_kabuli_Chr07*	5577311	T/C	Gene_Ca_06805	SYNONYMOUS_CODING	NA
Kabuli_wild_5577321	1399941684	*Ca_kabuli_Chr07*	5577321	C/A	Gene_Ca_06805	NON_SYNONYMOUS_CODING	NA
Kabuli_wild_5577439	1399941685	*Ca_kabuli_Chr07*	5577439	A/G	Gene_Ca_06805	NON_SYNONYMOUS_CODING	NA
Kabuli_wild_5577467	1399941686	*Ca_kabuli_Chr07*	5577467	G/T	Gene_Ca_06805	SYNONYMOUS_CODING	NA
Kabuli_wild_5581968	1399941687	*Ca_kabuli_Chr07*	5581968	G/C	NA	INTERGENIC	NA
Kabuli_wild_5658445	1399941688	*Ca_kabuli_Chr07*	5658445	G/T	NA	INTERGENIC	NA
Kabuli_wild_5671874	1399941689	*Ca_kabuli_Chr07*	5671874	G/T	NA	INTERGENIC	NA
Kabuli_wild_5671902	1399941690	*Ca_kabuli_Chr07*	5671902	G/T	NA	INTERGENIC	NA
Kabuli_wild_5672156	1399941691	*Ca_kabuli_Chr07*	5672156	T/C	NA	INTERGENIC	NA
Kabuli_wild_5678992	1399941692	*Ca_kabuli_Chr07*	5678992	T/C	Gene_Ca_06789	INTRON	RNApolymeraseIspecifictranscriptioninitiationfactorRRN3
Kabuli_wild_5683412	1399941693	*Ca_kabuli_Chr07*	5683412	T/A	Gene_Ca_06789	INTRON	RNApolymeraseIspecifictranscriptioninitiationfactorRRN3
Kabuli_wild_5713977	1399941694	*Ca_kabuli_Chr07*	5713977	T/C	Gene_Ca_06787	DOWNSTREAM	Copperamineoxidase
Kabuli_wild_5730386	1399941695	*Ca_kabuli_Chr07*	5730386	C/G	Gene_Ca_06785	DOWNSTREAM	Alpha/betahydrolasefold-1
Kabuli_wild_5735547	1399941696	*Ca_kabuli_Chr07*	5735547	A/G	Gene_Ca_06783	INTRON	Protein kinase,catalytic domain
Kabuli_wild_5737305	1399941697	*Ca_kabuli_Chr07*	5737305	G/A	Gene_Ca_06783	SYNONYMOUS_CODING	Protein kinase,catalytic domain
Kabuli_wild_5756173	1399941698	*Ca_kabuli_Chr07*	5756173	T/C	NA	INTERGENIC	NA
Kabuli_wild_5756269	1399941699	*Ca_kabuli_Chr07*	5756269	C/T	NA	INTERGENIC	NA
Kabuli_wild_5756832	1399941700	*Ca_kabuli_Chr07*	5756832	A/C	NA	INTERGENIC	NA
Kabuli_wild_5774218	1399941701	*Ca_kabuli_Chr07*	5774218	T/A	Gene_Ca_06781	INTRON	Dienelactonehydrolase
Kabuli_wild_5774219	1399941702	*Ca_kabuli_Chr07*	5774219	T/A	Gene_Ca_06781	INTRON	Dienelactonehydrolase
Kabuli_wild_5774220	1399941703	*Ca_kabuli_Chr07*	5774220	T/A	Gene_Ca_06781	INTRON	Dienelactonehydrolase

SNP IDs	NCBI dbSNP accession IDs (NCBI_SS#)	Chromosomes	Physical positions (bp)	SNPs	Gene accession IDs	Sequence components of genome	Putative functions
Kabuli_wild_5774221	1399941704	*Ca_kabuli_Chr07*	5774221	A/T	Gene_Ca_06781	INTRON	Dienelactonehydrolase
Kabuli_wild_5774230	1399941705	*Ca_kabuli_Chr07*	5774230	G/A	Gene_Ca_06781	INTRON	Dienelactonehydrolase
Kabuli_wild_5774257	1399941706	*Ca_kabuli_Chr07*	5774257	C/T	Gene_Ca_06781	INTRON	Dienelactonehydrolase
Kabuli_wild_5774310	1399941707	*Ca_kabuli_Chr07*	5774310	A/G	Gene_Ca_06781	INTRON	Dienelactonehydrolase
Kabuli_wild_5801997	1399941708	*Ca_kabuli_Chr07*	5801997	T/A	NA	INTERGENIC	NA
Kabuli_wild_5802607	1399941709	*Ca_kabuli_Chr07*	5802607	T/G	Gene_Ca_06778	NON_SYNONYMOUS_CODING	RNA recognition motif domain
Kabuli_wild_5802631	1399941710	*Ca_kabuli_Chr07*	5802631	C/A	Gene_Ca_06778	NON_SYNONYMOUS_CODING	RNA recognition motif domain
Kabuli_wild_5802916	1399941711	*Ca_kabuli_Chr07*	5802916	A/C	NA	INTERGENIC	NA
Kabuli_wild_5810043	1399941712	*Ca_kabuli_Chr07*	5810043	A/C	NA	INTERGENIC	NA
Kabuli_wild_5811097	1399941713	*Ca_kabuli_Chr07*	5811097	G/A	NA	INTERGENIC	NA
Kabuli_wild_5836421	1399941714	*Ca_kabuli_Chr07*	5836421	A/T	NA	INTERGENIC	NA
Kabuli_wild_5846477	1399941715	*Ca_kabuli_Chr07*	5846477	A/G	NA	INTERGENIC	NA
Kabuli_wild_5849904	1399941716	*Ca_kabuli_Chr07*	5849904	C/A	Gene_Ca_06774	DOWNSTREAM	SNARE associated Golgi protein
Kabuli_wild_5849936	1399941717	*Ca_kabuli_Chr07*	5849936	C/T	Gene_Ca_06774	DOWNSTREAM	SNARE associated Golgi protein
Kabuli_wild_5850058	1399941718	*Ca_kabuli_Chr07*	5850058	T/C	Gene_Ca_06774	DOWNSTREAM	SNARE associated Golgi protein
Kabuli_wild_5854491	1399941719	*Ca_kabuli_Chr07*	5854491	T/G	NA	INTERGENIC	NA
Kabuli_wild_5854598	1399941720	*Ca_kabuli_Chr07*	5854598	C/T	NA	INTERGENIC	NA
Kabuli_wild_5925751	1399941721	*Ca_kabuli_Chr07*	5925751	T/C	Gene_Ca_06765	UPSTREAM	Domain of unknown function DUF298
Kabuli_wild_5928979	1399941722	*Ca_kabuli_Chr07*	5928979	T/G	Gene_Ca_06764	INTRON	ATPase, P-type, H+ transporting proton pump
Kabuli_wild_5929009	1399941723	*Ca_kabuli_Chr07*	5929009	G/C	Gene_Ca_06764	INTRON	ATPase, P-type, H+ transporting proton pump
Kabuli_wild_5946113	1399941724	*Ca_kabuli_Chr07*	5946113	G/A	NA	INTERGENIC	NA
Kabuli_wild_5946536	1399941725	*Ca_kabuli_Chr07*	5946536	G/C	NA	INTERGENIC	NA
Kabuli_wild_5947944	1399941726	*Ca_kabuli_Chr07*	5947944	A/G	NA	INTERGENIC	NA
Kabuli_wild_5948771	1399941727	*Ca_kabuli_Chr07*	5948771	A/G	Gene_Ca_06762	INTRON	DNA-dependentATPaseMCM
Kabuli_wild_5950352	1399941728	*Ca_kabuli_Chr07*	5950352	T/A	Gene_Ca_06762	INTRON	DNA-dependentATPaseMCM
Kabuli_wild_5950492	1399941729	*Ca_kabuli_Chr07*	5950492	T/C	Gene_Ca_06762	SYNONYMOUS_CODING	DNA-dependentATPaseMCM
Kabuli_wild_5957696	1399941730	*Ca_kabuli_Chr07*	5957696	T/C	Gene_Ca_06761	INTRON	RibonucleaseIII
Kabuli_wild_5963609	1399941731	*Ca_kabuli_Chr07*	5963609	C/A	Gene_Ca_06761	INTRON	RibonucleaseIII
Kabuli_wild_5963611	1399941732	*Ca_kabuli_Chr07*	5963611	C/G	Gene_Ca_06761	INTRON	RibonucleaseIII
Kabuli_wild_6012089	1399941733	*Ca_kabuli_Chr07*	6012089	T/C	NA	INTERGENIC	NA
Kabuli_wild_6012363	1399941734	*Ca_kabuli_Chr07*	6012363	T/A	NA	INTERGENIC	NA
Kabuli_wild_6012561	1399941735	*Ca_kabuli_Chr07*	6012561	A/C	NA	INTERGENIC	NA
Kabuli_wild_6012611	1399941736	*Ca_kabuli_Chr07*	6012611	A/C	NA	INTERGENIC	NA
Kabuli_wild_6023906	1399941737	*Ca_kabuli_Chr07*	6023906	G/A	Gene_Ca_06757	INTRON	Sugar/inositol transporter
Kabuli_wild_6029990	1399941738	*Ca_kabuli_Chr07*	6029990	C/G	NA	INTERGENIC	NA
Kabuli_wild_6066322	1399941739	*Ca_kabuli_Chr07*	6066322	G/A	Gene_Ca_06752	DOWNSTREAM	Phox/Bem1p
Kabuli_wild_6069979	1399941740	*Ca_kabuli_Chr07*	6069979	T/C	Gene_Ca_06752	INTRON	Phox/Bem1p
Kabuli_wild_6074408	1399941741	*Ca_kabuli_Chr07*	6074408	T/A	Gene_Ca_06752	SYNONYMOUS_CODING	Phox/Bem1p
Kabuli_wild_6075124	1399941742	*Ca_kabuli_Chr07*	6075124	T/G	Gene_Ca_06752	NON_SYNONYMOUS_CODING	Phox/Bem1p
Kabuli_wild_6075218	1399941743	*Ca_kabuli_Chr07*	6075218	C/G	Gene_Ca_06752	START_LOST	Phox/Bem1p
Kabuli_wild_6096238	1399941744	*Ca_kabuli_Chr07*	6096238	C/A	NA	INTERGENIC	NA
Kabuli_wild_6108535	1399941745	*Ca_kabuli_Chr07*	6108535	G/T	NA	INTERGENIC	NA
Kabuli_wild_6110019	1399941746	*Ca_kabuli_Chr07*	6110019	T/C	NA	INTERGENIC	NA
Kabuli_wild_6132021	1399941747	*Ca_kabuli_Chr07*	6132021	G/A	Gene_Ca_06744	INTRON	Ubiquitin

SNP IDs	NCBI dbSNP accession IDs (NCBI_SS#)	Chromosomes	Physical positions (bp)	SNPs	Gene accession IDs	Sequence components of genome	Putative functions
Kabuli_wild_6232777	1399941748	*Ca_kabuli_Chr07*	6232777	T/C	NA	INTERGENIC	NA
Kabuli_wild_6299248	1399941749	*Ca_kabuli_Chr07*	6299248	A/G	NA	INTERGENIC	NA
Kabuli_wild_6299249	1399941750	*Ca_kabuli_Chr07*	6299249	C/G	NA	INTERGENIC	NA
Kabuli_wild_6307554	1399941751	*Ca_kabuli_Chr07*	6307554	A/G	NA	INTERGENIC	NA
Kabuli_wild_6393366	1399941752	*Ca_kabuli_Chr07*	6393366	G/A	Gene_Ca_06712	INTRON	Metallo-dependentphosphatase
Kabuli_wild_6467903	1399941753	*Ca_kabuli_Chr07*	6467903	C/T	Gene_Ca_06705	SYNONYMOUS_CODING	Mak10subunit,NatCN(alpha)-terminalacetyltransferase
Kabuli_wild_6540601	1399941754	*Ca_kabuli_Chr07*	6540601	G/C	Gene_Ca_06700	INTRON	Rap1-interactingfactor1N-terminal
Kabuli_wild_6540602	1399941755	*Ca_kabuli_Chr07*	6540602	G/A	Gene_Ca_06700	INTRON	Rap1-interactingfactor1N-terminal
Kabuli_wild_6540624	1399941756	*Ca_kabuli_Chr07*	6540624	A/G	Gene_Ca_06700	INTRON	Rap1-interactingfactor1N-terminal
Kabuli_wild_6553205	1399941757	*Ca_kabuli_Chr07*	6553205	A/T	NA	INTERGENIC	NA
Kabuli_wild_6566263	1399941758	*Ca_kabuli_Chr07*	6566263	A/G	NA	INTERGENIC	NA
Kabuli_wild_6566322	1399941759	*Ca_kabuli_Chr07*	6566322	T/C	NA	INTERGENIC	NA
Kabuli_wild_6573160	1399941760	*Ca_kabuli_Chr07*	6573160	G/A	NA	INTERGENIC	NA
Kabuli_wild_6573169	1399941761	*Ca_kabuli_Chr07*	6573169	C/T	NA	INTERGENIC	NA
Kabuli_wild_6573175	1399941762	*Ca_kabuli_Chr07*	6573175	G/T	NA	INTERGENIC	NA
Kabuli_wild_6573218	1399941763	*Ca_kabuli_Chr07*	6573218	C/T	NA	INTERGENIC	NA
Kabuli_wild_6644942	1399941764	*Ca_kabuli_Chr07*	6644942	T/G	NA	INTERGENIC	NA
Kabuli_wild_6680973	1399941765	*Ca_kabuli_Chr07*	6680973	T/G	Gene_Ca_06692	NON_SYNONYMOUS_CODING	Glycosyltransferase,family17
Kabuli_wild_6680976	1399941766	*Ca_kabuli_Chr07*	6680976	C/G	Gene_Ca_06692	NON_SYNONYMOUS_CODING	Glycosyltransferase,family17
Kabuli_wild_6682439	1399941767	*Ca_kabuli_Chr07*	6682439	T/A	NA	INTERGENIC	NA
Kabuli_wild_6682443	1399941768	*Ca_kabuli_Chr07*	6682443	C/T	NA	INTERGENIC	NA
Kabuli_wild_6682448	1399941769	*Ca_kabuli_Chr07*	6682448	T/C	NA	INTERGENIC	NA
Kabuli_wild_6682453	1399941770	*Ca_kabuli_Chr07*	6682453	G/T	NA	INTERGENIC	NA
Kabuli_wild_6682454	1399941771	*Ca_kabuli_Chr07*	6682454	A/G	NA	INTERGENIC	NA
Kabuli_wild_6682455	1399941772	*Ca_kabuli_Chr07*	6682455	G/T	NA	INTERGENIC	NA
Kabuli_wild_6699937	1399941773	*Ca_kabuli_Chr07*	6699937	A/G	Gene_Ca_06691	INTRON	WD40 repeat
Kabuli_wild_6703849	1399941774	*Ca_kabuli_Chr07*	6703849	G/T	Gene_Ca_06691	NON_SYNONYMOUS_CODING	WD40 repeat
Kabuli_wild_6780836	1399941775	*Ca_kabuli_Chr07*	6780836	A/C	Gene_Ca_06684	SYNONYMOUS_CODING	Glycosidehydrolase,family17
Kabuli_wild_6792606	1399941776	*Ca_kabuli_Chr07*	6792606	T/G	Gene_Ca_06683	DOWNSTREAM	NA
Kabuli_wild_6814752	1399941777	*Ca_kabuli_Chr07*	6814752	A/G	Gene_Ca_06681	INTRON	YABBYprotein
Kabuli_wild_6859511	1399941778	*Ca_kabuli_Chr07*	6859511	C/G	Gene_Ca_06677	DOWNSTREAM	Protein kinase,catalytic domain
Kabuli_wild_6884304	1399941779	*Ca_kabuli_Chr07*	6884304	T/A	NA	INTERGENIC	NA
Kabuli_wild_6963056	1399941780	*Ca_kabuli_Chr07*	6963056	T/C	Gene_Ca_06667	UPSTREAM	Protein kinase,catalytic domain
Kabuli_wild_7026516	1399941781	*Ca_kabuli_Chr07*	7026516	T/C	NA	INTERGENIC	NA
Kabuli_wild_7119653	1399941782	*Ca_kabuli_Chr07*	7119653	C/G	Gene_Ca_06647	DOWNSTREAM	Pentatrico peptide repeat
Kabuli_wild_7146159	1399941783	*Ca_kabuli_Chr07*	7146159	C/G	NA	INTERGENIC	NA
Kabuli_wild_7265386	1399941784	*Ca_kabuli_Chr07*	7265386	T/A	Gene_Ca_06635	DOWNSTREAM	Cyclicnucleotide-binding domain
Kabuli_wild_7423419	1399941785	*Ca_kabuli_Chr07*	7423419	G/A	Gene_Ca_06617	NON_SYNONYMOUS_CODING	Heat shock protein DnaJ,N-terminal
Kabuli_wild_7424203	1399941786	*Ca_kabuli_Chr07*	7424203	G/A	Gene_Ca_06617	NON_SYNONYMOUS_CODING	Heat shock protein DnaJ,N-terminal
Kabuli_wild_7428102	1399941787	*Ca_kabuli_Chr07*	7428102	T/C	Gene_Ca_06617	NON_SYNONYMOUS_CODING	Heat shock protein DnaJ,N-terminal
Kabuli_wild_7432208	1399941788	*Ca_kabuli_Chr07*	7432208	T/G	Gene_Ca_06617	NON_SYNONYMOUS_CODING	Heat shock protein DnaJ,N-terminal
Kabuli_wild_7576668	1399941789	*Ca_kabuli_Chr07*	7576668	A/C	NA	INTERGENIC	NA
Kabuli_wild_7576690	1399941790	*Ca_kabuli_Chr07*	7576690	C/T	NA	INTERGENIC	NA
Kabuli_wild_7580101	1399941791	*Ca_kabuli_Chr07*	7580101	T/G	NA	INTERGENIC	NA

SNP IDs	NCBI dbSNP accession IDs (NCBI_SS#)	Chromosomes	Physical positions (bp)	SNPs	Gene accession IDs	Sequence components of genome	Putative functions
Kabuli_wild_7580158	1399941792	*Ca_kabuli_Chr07*	7580158	T/C	NA	INTERGENIC	NA
Kabuli_wild_7706816	1399941793	*Ca_kabuli_Chr07*	7706816	A/G	Gene_Ca_06588	DOWNSTREAM	Protein of unknown function DUF3353
Kabuli_wild_7737850	1399941794	*Ca_kabuli_Chr07*	7737850	T/G	NA	INTERGENIC	NA
Kabuli_wild_7737855	1399941795	*Ca_kabuli_Chr07*	7737855	T/C	NA	INTERGENIC	NA
Kabuli_wild_7737899	1399941796	*Ca_kabuli_Chr07*	7737899	C/T	NA	INTERGENIC	NA
Kabuli_wild_7756452	1399941797	*Ca_kabuli_Chr07*	7756452	C/T	Gene_Ca_13243	NON_SYNONYMOUS_CODING	SNF2-related
Kabuli_wild_7756455	1399941798	*Ca_kabuli_Chr07*	7756455	C/T	Gene_Ca_13243	NON_SYNONYMOUS_CODING	SNF2-related
Kabuli_wild_7756566	1399941799	*Ca_kabuli_Chr07*	7756566	G/A	Gene_Ca_13243	INTRON	SNF2-related
Kabuli_wild_7781888	1399941800	*Ca_kabuli_Chr07*	7781888	A/G	Gene_Ca_13241	INTRON	Peptidyl-argininedeiminase,Porphyromonas-type
Kabuli_wild_7809179	1399941801	*Ca_kabuli_Chr07*	7809179	T/G	Gene_Ca_13237	INTRON	PeptidaseS49
Kabuli_wild_7809192	1399941802	*Ca_kabuli_Chr07*	7809192	A/C	Gene_Ca_13237	INTRON	PeptidaseS49
Kabuli_wild_7983809	1399941803	*Ca_kabuli_Chr07*	7983809	G/A	Gene_Ca_13220	NON_SYNONYMOUS_CODING	ABC transporter,trans membrane domain
Kabuli_wild_8061808	1399941804	*Ca_kabuli_Chr07*	8061808	A/G	Gene_Ca_13209	SYNONYMOUS_CODING	Nucleicacidbinding,OB-fold,tRNA/helicase-type
Kabuli_wild_8169478	1399941805	*Ca_kabuli_Chr07*	8169478	A/G	Gene_Ca_13198	UPSTREAM	X8
Kabuli_wild_8169947	1399941806	*Ca_kabuli_Chr07*	8169947	T/C	NA	INTERGENIC	NA
Kabuli_wild_8187181	1399941807	*Ca_kabuli_Chr07*	8187181	G/A	Gene_Ca_13195	SYNONYMOUS_CODING	ChaperoninClpA/B
Kabuli_wild_8188004	1399941808	*Ca_kabuli_Chr07*	8188004	C/G	Gene_Ca_13195	NON_SYNONYMOUS_CODING	ChaperoninClpA/B
Kabuli_wild_8249693	1399941809	*Ca_kabuli_Chr07*	8249693	A/T	Gene_Ca_13190	SYNONYMOUS_CODING	Zinc finger,C2H2-type
Kabuli_wild_8261062	1399941810	*Ca_kabuli_Chr07*	8261062	C/A	NA	INTERGENIC	NA
Kabuli_wild_8282670	1399941811	*Ca_kabuli_Chr07*	8282670	T/G	Gene_Ca_13185	NON_SYNONYMOUS_CODING	NA
Kabuli_wild_8388062	1399941812	*Ca_kabuli_Chr07*	8388062	A/G	Gene_Ca_13170	DOWNSTREAM	NA
Kabuli_wild_8404852	1399941813	*Ca_kabuli_Chr07*	8404852	T/A	NA	INTERGENIC	NA
Kabuli_wild_8434392	1399941814	*Ca_kabuli_Chr07*	8434392	C/A	NA	INTERGENIC	NA
Kabuli_wild_8490611	1399941815	*Ca_kabuli_Chr07*	8490611	A/C	Gene_Ca_13158	DOWNSTREAM	HNHendonuclease
Kabuli_wild_8569632	1399941816	*Ca_kabuli_Chr07*	8569632	A/G	NA	INTERGENIC	NA
Kabuli_wild_8583890	1399941817	*Ca_kabuli_Chr07*	8583890	T/C	Gene_Ca_15359	SYNONYMOUS_CODING	Ubiquitin-conjugating enzyme, E2
Kabuli_wild_8609982	1399941818	*Ca_kabuli_Chr07*	8609982	A/C	NA	INTERGENIC	NA
Kabuli_wild_8626875	1399941819	*Ca_kabuli_Chr07*	8626875	A/T	NA	INTERGENIC	NA
Kabuli_wild_8723939	1399941820	*Ca_kabuli_Chr07*	8723939	A/G	Gene_Ca_15344	UPSTREAM	NA
Kabuli_wild_8835601	1399941821	*Ca_kabuli_Chr07*	8835601	A/G	NA	INTERGENIC	NA
Kabuli_wild_8835602	1399941822	*Ca_kabuli_Chr07*	8835602	G/A	NA	INTERGENIC	NA
Kabuli_wild_8835610	1399941823	*Ca_kabuli_Chr07*	8835610	A/G	NA	INTERGENIC	NA
Kabuli_wild_8835631	1399941824	*Ca_kabuli_Chr07*	8835631	T/G	NA	INTERGENIC	NA
Kabuli_wild_8835659	1399941825	*Ca_kabuli_Chr07*	8835659	T/G	NA	INTERGENIC	NA
Kabuli_wild_8835705	1399941826	*Ca_kabuli_Chr07*	8835705	T/A	NA	INTERGENIC	NA
Kabuli_wild_8835718	1399941827	*Ca_kabuli_Chr07*	8835718	T/G	NA	INTERGENIC	NA
Kabuli_wild_8835757	1399941828	*Ca_kabuli_Chr07*	8835757	T/C	NA	INTERGENIC	NA
Kabuli_wild_8848940	1399941829	*Ca_kabuli_Chr07*	8848940	A/C	Gene_Ca_15327	INTRON	ATPase, P-type, H+ transporting proton pump
Kabuli_wild_8849200	1399941830	*Ca_kabuli_Chr07*	8849200	A/C	Gene_Ca_15327	INTRON	ATPase, P-type, H+ transporting proton pump
Kabuli_wild_8895450	1399941831	*Ca_kabuli_Chr07*	8895450	T/G	Gene_Ca_15325	SYNONYMOUS_CODING	Domain of unknown function DUF629
Kabuli_wild_8937376	1399941832	*Ca_kabuli_Chr07*	8937376	A/G	Gene_Ca_15321	SYNONYMOUS_CODING	WWdomainbindingprotein11
Kabuli_wild_8937379	1399941833	*Ca_kabuli_Chr07*	8937379	T/C	Gene_Ca_15321	SYNONYMOUS_CODING	WWdomainbindingprotein11
Kabuli_wild_9028286	1399941834	*Ca_kabuli_Chr07*	9028286	G/A	Gene_Ca_15313	INTRON	Exportin-1/Importin-beta-like
Kabuli_wild_9128044	1399941835	*Ca_kabuli_Chr07*	9128044	A/G	NA	INTERGENIC	NA

SNP IDs	NCBI dbSNP accession IDs (NCBI_SS#)	Chromosomes	Physical positions (bp)	SNPs	Gene accession IDs	Sequence components of genome	Putative functions
Kabuli_wild_9128050	1399941836	*Ca_kabuli_Chr07*	9128050	C/A	NA	INTERGENIC	NA
Kabuli_wild_9147366	1399941837	*Ca_kabuli_Chr07*	9147366	T/G	Gene_Ca_15301	INTRON	Transcription factor E2F/dimerisationpartner(TDP)
Kabuli_wild_9147367	1399941838	*Ca_kabuli_Chr07*	9147367	A/T	Gene_Ca_15301	INTRON	Transcription factor E2F/dimerisationpartner(TDP)
Kabuli_wild_9147386	1399941839	*Ca_kabuli_Chr07*	9147386	A/C	Gene_Ca_15301	INTRON	Transcription factor E2F/dimerisationpartner(TDP)
Kabuli_wild_9147502	1399941840	*Ca_kabuli_Chr07*	9147502	C/T	Gene_Ca_15301	INTRON	Transcription factor E2F/dimerisationpartner(TDP)
Kabuli_wild_9151709	1399941841	*Ca_kabuli_Chr07*	9151709	T/G	NA	INTERGENIC	NA
Kabuli_wild_9151755	1399941842	*Ca_kabuli_Chr07*	9151755	C/T	NA	INTERGENIC	NA
Kabuli_wild_9152949	1399941843	*Ca_kabuli_Chr07*	9152949	T/C	NA	INTERGENIC	NA
Kabuli_wild_9152984	1399941844	*Ca_kabuli_Chr07*	9152984	C/G	NA	INTERGENIC	NA
Kabuli_wild_9153062	1399941845	*Ca_kabuli_Chr07*	9153062	G/A	NA	INTERGENIC	NA
Kabuli_wild_9153104	1399941846	*Ca_kabuli_Chr07*	9153104	C/A	NA	INTERGENIC	NA
Kabuli_wild_9214422	1399941847	*Ca_kabuli_Chr07*	9214422	C/T	NA	INTERGENIC	NA
Kabuli_wild_9214457	1399941848	*Ca_kabuli_Chr07*	9214457	T/A	NA	INTERGENIC	NA
Kabuli_wild_9223172	1399941849	*Ca_kabuli_Chr07*	9223172	C/T	NA	INTERGENIC	NA
Kabuli_wild_9223204	1399941850	*Ca_kabuli_Chr07*	9223204	A/T	NA	INTERGENIC	NA
Kabuli_wild_9223209	1399941851	*Ca_kabuli_Chr07*	9223209	C/T	NA	INTERGENIC	NA
Kabuli_wild_9266762	1399941852	*Ca_kabuli_Chr07*	9266762	A/G	Gene_Ca_17334	INTRON	Lipoxygenase,C-terminal
Kabuli_wild_9268649	1399941853	*Ca_kabuli_Chr07*	9268649	G/C	NA	INTERGENIC	NA
Kabuli_wild_9268684	1399941854	*Ca_kabuli_Chr07*	9268684	A/G	NA	INTERGENIC	NA
Kabuli_wild_9281622	1399941855	*Ca_kabuli_Chr07*	9281622	G/T	Gene_Ca_17333	DOWNSTREAM	WD40 repeat
Kabuli_wild_9281669	1399941856	*Ca_kabuli_Chr07*	9281669	T/C	Gene_Ca_17333	DOWNSTREAM	WD40 repeat
Kabuli_wild_9301825	1399941857	*Ca_kabuli_Chr07*	9301825	G/C	Gene_Ca_17330	NON_SYNONYMOUS_CODING	rRNAsmallsubunitmethyltransferaseG
Kabuli_wild_9301881	1399941858	*Ca_kabuli_Chr07*	9301881	C/T	Gene_Ca_17330	UPSTREAM	rRNAsmallsubunitmethyltransferaseG
Kabuli_wild_9348281	1399941859	*Ca_kabuli_Chr07*	9348281	T/C	NA	INTERGENIC	NA
Kabuli_wild_9350506	1399941860	*Ca_kabuli_Chr07*	9350506	T/G	NA	INTERGENIC	NA
Kabuli_wild_9350535	1399941861	*Ca_kabuli_Chr07*	9350535	A/C	NA	INTERGENIC	NA
Kabuli_wild_9351783	1399941862	*Ca_kabuli_Chr07*	9351783	C/G	Gene_Ca_17325	INTRON	Glycosidehydrolase,family47
Kabuli_wild_9351863	1399941863	*Ca_kabuli_Chr07*	9351863	G/A	Gene_Ca_17325	NON_SYNONYMOUS_CODING	Glycosidehydrolase,family47
Kabuli_wild_9475275	1399941864	*Ca_kabuli_Chr07*	9475275	T/G	NA	INTERGENIC	NA
Kabuli_wild_9523902	1399941865	*Ca_kabuli_Chr07*	9523902	C/T	NA	INTERGENIC	NA
Kabuli_wild_9528073	1399941866	*Ca_kabuli_Chr07*	9528073	G/C	NA	INTERGENIC	NA
Kabuli_wild_9612558	1399941867	*Ca_kabuli_Chr07*	9612558	A/C	NA	INTERGENIC	NA
Kabuli_wild_9671210	1399941868	*Ca_kabuli_Chr07*	9671210	T/C	NA	INTERGENIC	NA
Kabuli_wild_9696620	1399941869	*Ca_kabuli_Chr07*	9696620	A/G	Gene_Ca_17285	DOWNSTREAM	NA
Kabuli_wild_9744436	1399941870	*Ca_kabuli_Chr07*	9744436	G/T	NA	INTERGENIC	NA
Kabuli_wild_9897698	1399941871	*Ca_kabuli_Chr07*	9897698	T/G	NA	INTERGENIC	NA
Kabuli_wild_9914745	1399941872	*Ca_kabuli_Chr07*	9914745	T/C	NA	INTERGENIC	NA
Kabuli_wild_9914751	1399941873	*Ca_kabuli_Chr07*	9914751	G/A	NA	INTERGENIC	NA
Kabuli_wild_9914763	1399941874	*Ca_kabuli_Chr07*	9914763	T/G	NA	INTERGENIC	NA
Kabuli_wild_9914764	1399941875	*Ca_kabuli_Chr07*	9914764	G/A	NA	INTERGENIC	NA
Kabuli_wild_9914784	1399941876	*Ca_kabuli_Chr07*	9914784	G/A	NA	INTERGENIC	NA
Kabuli_wild_9914785	1399941877	*Ca_kabuli_Chr07*	9914785	T/C	NA	INTERGENIC	NA
Kabuli_wild_9914790	1399941878	*Ca_kabuli_Chr07*	9914790	C/G	NA	INTERGENIC	NA
Kabuli_wild_9914794	1399941879	*Ca_kabuli_Chr07*	9914794	G/A	NA	INTERGENIC	NA

SNP IDs	NCBI dbSNP accession IDs (NCBI_SS#)	Chromosomes	Physical positions (bp)	SNPs	Gene accession IDs	Sequence components of genome	Putative functions
Kabuli_wild_9914802	1399941880	*Ca_kabuli_Chr07*	9914802	T/G	NA	INTERGENIC	NA
Kabuli_wild_9914804	1399941881	*Ca_kabuli_Chr07*	9914804	C/A	NA	INTERGENIC	NA
Kabuli_wild_10049904	1399941882	*Ca_kabuli_Chr07*	10049904	G/A	NA	INTERGENIC	NA
Kabuli_wild_10115618	1399941883	*Ca_kabuli_Chr07*	10115618	T/G	Gene_Ca_12774	NON_SYNONYMOUS_CODING	Pathogenesis-related transcriptional factor/ERF, DNA-binding
Kabuli_wild_10207510	1399941884	*Ca_kabuli_Chr07*	10207510	A/T	Gene_Ca_12782	DOWNSTREAM	RasGTPase
Kabuli_wild_10208029	1399941885	*Ca_kabuli_Chr07*	10208029	T/C	Gene_Ca_12782	DOWNSTREAM	RasGTPase
Kabuli_wild_10208267	1399941886	*Ca_kabuli_Chr07*	10208267	G/A	Gene_Ca_12782	DOWNSTREAM	RasGTPase
Kabuli_wild_10216831	1399941887	*Ca_kabuli_Chr07*	10216831	A/G	NA	INTERGENIC	NA
Kabuli_wild_10223027	1399941888	*Ca_kabuli_Chr07*	10223027	G/T	NA	INTERGENIC	NA
Kabuli_wild_10223040	1399941889	*Ca_kabuli_Chr07*	10223040	T/A	NA	INTERGENIC	NA
Kabuli_wild_10223068	1399941890	*Ca_kabuli_Chr07*	10223068	G/T	NA	INTERGENIC	NA
Kabuli_wild_10223069	1399941891	*Ca_kabuli_Chr07*	10223069	G/T	NA	INTERGENIC	NA
Kabuli_wild_10223071	1399941892	*Ca_kabuli_Chr07*	10223071	T/A	NA	INTERGENIC	NA
Kabuli_wild_10223074	1399941893	*Ca_kabuli_Chr07*	10223074	G/T	NA	INTERGENIC	NA
Kabuli_wild_10223075	1399941894	*Ca_kabuli_Chr07*	10223075	C/T	NA	INTERGENIC	NA
Kabuli_wild_10231755	1399941895	*Ca_kabuli_Chr07*	10231755	C/T	NA	INTERGENIC	NA
Kabuli_wild_10276530	1399941896	*Ca_kabuli_Chr07*	10276530	A/G	NA	INTERGENIC	NA
Kabuli_wild_10283596	1399941897	*Ca_kabuli_Chr07*	10283596	G/A	NA	INTERGENIC	NA
Kabuli_wild_10322726	1399941898	*Ca_kabuli_Chr07*	10322726	T/C	Gene_Ca_12794	SYNONYMOUS_CODING	tRNA/rRNAmethyltransferase,SpoU
Kabuli_wild_10429442	1399941899	*Ca_kabuli_Chr07*	10429442	G/A	Gene_Ca_12802	SYNONYMOUS_CODING	MORNmotif
Kabuli_wild_10435983	1399941900	*Ca_kabuli_Chr07*	10435983	C/A	Gene_Ca_12803	NON_SYNONYMOUS_CODING	CaseinkinaseII,regulatorysubunit
Kabuli_wild_10483883	1399941901	*Ca_kabuli_Chr07*	10483883	T/G	Gene_Ca_12810	INTRON	Protein of unknown function DUF702
Kabuli_wild_10484200	1399941902	*Ca_kabuli_Chr07*	10484200	G/A	Gene_Ca_12810	INTRON	Protein of unknown function DUF702
Kabuli_wild_10485397	1399941903	*Ca_kabuli_Chr07*	10485397	C/G	Gene_Ca_12810	NON_SYNONYMOUS_CODING	Protein of unknown function DUF702
Kabuli_wild_10521449	1399941904	*Ca_kabuli_Chr07*	10521449	C/A	Gene_Ca_12811	NON_SYNONYMOUS_CODING	Zinc finger, RING-type
Kabuli_wild_10640289	1399941905	*Ca_kabuli_Chr07*	10640289	C/G	Gene_Ca_12821	DOWNSTREAM	Expansin/pollenallergen,DPBBdomain
Kabuli_wild_10688189	1399941906	*Ca_kabuli_Chr07*	10688189	T/G	Gene_Ca_12825	INTRON	Signal transduction response regulator, receiverdomain
Kabuli_wild_10703569	1399941907	*Ca_kabuli_Chr07*	10703569	A/T	NA	INTERGENIC	NA
Kabuli_wild_10703595	1399941908	*Ca_kabuli_Chr07*	10703595	T/A	NA	INTERGENIC	NA
Kabuli_wild_10703685	1399941909	*Ca_kabuli_Chr07*	10703685	G/T	NA	INTERGENIC	NA
Kabuli_wild_10703704	1399941910	*Ca_kabuli_Chr07*	10703704	G/A	NA	INTERGENIC	NA
Kabuli_wild_10712162	1399941911	*Ca_kabuli_Chr07*	10712162	T/G	NA	INTERGENIC	NA
Kabuli_wild_10712198	1399941912	*Ca_kabuli_Chr07*	10712198	T/C	NA	INTERGENIC	NA
Kabuli_wild_10712203	1399941913	*Ca_kabuli_Chr07*	10712203	C/A	NA	INTERGENIC	NA
Kabuli_wild_10712231	1399941914	*Ca_kabuli_Chr07*	10712231	G/A	NA	INTERGENIC	NA
Kabuli_wild_10775843	1399941915	*Ca_kabuli_Chr07*	10775843	A/C	NA	INTERGENIC	NA
Kabuli_wild_10775857	1399941916	*Ca_kabuli_Chr07*	10775857	G/A	NA	INTERGENIC	NA
Kabuli_wild_10775859	1399941917	*Ca_kabuli_Chr07*	10775859	C/T	NA	INTERGENIC	NA
Kabuli_wild_10775860	1399941918	*Ca_kabuli_Chr07*	10775860	A/G	NA	INTERGENIC	NA
Kabuli_wild_10775881	1399941919	*Ca_kabuli_Chr07*	10775881	C/T	NA	INTERGENIC	NA
Kabuli_wild_10780680	1399941920	*Ca_kabuli_Chr07*	10780680	C/G	NA	INTERGENIC	NA
Kabuli_wild_10780682	1399941921	*Ca_kabuli_Chr07*	10780682	G/A	NA	INTERGENIC	NA
Kabuli_wild_10780686	1399941922	*Ca_kabuli_Chr07*	10780686	T/C	NA	INTERGENIC	NA
Kabuli_wild_10780716	1399941923	*Ca_kabuli_Chr07*	10780716	T/C	NA	INTERGENIC	NA

SNP IDs	NCBI dbSNP accession IDs (NCBI_SS#)	Chromosomes	Physical positions (bp)	SNPs	Gene accession IDs	Sequence components of genome	Putative functions
Kabuli_wild_10797204	1399941924	*Ca_kabuli_Chr07*	10797204	T/C	Gene_Ca_12832	INTRON	Protein kinase,catalytic domain
Kabuli_wild_10816523	1399941925	*Ca_kabuli_Chr07*	10816523	T/G	NA	INTERGENIC	NA
Kabuli_wild_10900910	1399941926	*Ca_kabuli_Chr07*	10900910	T/C	NA	INTERGENIC	NA
Kabuli_wild_11048796	1399941927	*Ca_kabuli_Chr07*	11048796	C/A	Gene_Ca_09380	STOP_GAINED	Reverse transcriptase, RNA-dependent DNA polymerase
Kabuli_wild_11105847	1399941928	*Ca_kabuli_Chr07*	11105847	C/G	Gene_Ca_09376	NON_SYNONYMOUS_CODING	Hexokinase
Kabuli_wild_11183385	1399941929	*Ca_kabuli_Chr07*	11183385	G/A	Gene_Ca_09372	NON_SYNONYMOUS_CODING	Glycosidehydrolase,family16
Kabuli_wild_11192703	1399941930	*Ca_kabuli_Chr07*	11192703	A/C	Gene_Ca_09371	SYNONYMOUS_CODING	Homeobox
Kabuli_wild_11261924	1399941931	*Ca_kabuli_Chr07*	11261924	G/A	NA	INTERGENIC	NA
Kabuli_wild_11393795	1399941932	*Ca_kabuli_Chr07*	11393795	A/G	NA	INTERGENIC	NA
Kabuli_wild_11545846	1399941933	*Ca_kabuli_Chr07*	11545846	A/G	Gene_Ca_09342	NON_SYNONYMOUS_CODING	Lateral organ boundaries, LOB
Kabuli_wild_11568963	1399941934	*Ca_kabuli_Chr07*	11568963	T/A	NA	INTERGENIC	NA
Kabuli_wild_11630509	1399941935	*Ca_kabuli_Chr07*	11630509	A/G	NA	INTERGENIC	NA
Kabuli_wild_11696995	1399941936	*Ca_kabuli_Chr07*	11696995	A/G	NA	INTERGENIC	NA
Kabuli_wild_11725204	1399941937	*Ca_kabuli_Chr07*	11725204	A/G	Gene_Ca_09325	DOWNSTREAM	NA
Kabuli_wild_11731021	1399941938	*Ca_kabuli_Chr07*	11731021	G/C	Gene_Ca_09324	UPSTREAM	NA
Kabuli_wild_11734921	1399941939	*Ca_kabuli_Chr07*	11734921	T/G	NA	INTERGENIC	NA
Kabuli_wild_11735368	1399941940	*Ca_kabuli_Chr07*	11735368	G/C	NA	INTERGENIC	NA
Kabuli_wild_11803091	1399941941	*Ca_kabuli_Chr07*	11803091	T/G	NA	INTERGENIC	NA
Kabuli_wild_11858425	1399941942	*Ca_kabuli_Chr07*	11858425	C/T	NA	INTERGENIC	NA
Kabuli_wild_11886954	1399941943	*Ca_kabuli_Chr07*	11886954	A/G	NA	INTERGENIC	NA
Kabuli_wild_12010426	1399941944	*Ca_kabuli_Chr07*	12010426	T/C	NA	INTERGENIC	NA
Kabuli_wild_12010432	1399941945	*Ca_kabuli_Chr07*	12010432	G/A	NA	INTERGENIC	NA
Kabuli_wild_12010457	1399941946	*Ca_kabuli_Chr07*	12010457	C/T	NA	INTERGENIC	NA
Kabuli_wild_12010471	1399941947	*Ca_kabuli_Chr07*	12010471	C/T	NA	INTERGENIC	NA
Kabuli_wild_12010483	1399941948	*Ca_kabuli_Chr07*	12010483	C/T	NA	INTERGENIC	NA
Kabuli_wild_12010489	1399941949	*Ca_kabuli_Chr07*	12010489	C/T	NA	INTERGENIC	NA
Kabuli_wild_12010491	1399941950	*Ca_kabuli_Chr07*	12010491	G/T	NA	INTERGENIC	NA
Kabuli_wild_12010494	1399941951	*Ca_kabuli_Chr07*	12010494	G/A	NA	INTERGENIC	NA
Kabuli_wild_12010499	1399941952	*Ca_kabuli_Chr07*	12010499	C/A	NA	INTERGENIC	NA
Kabuli_wild_12010504	1399941953	*Ca_kabuli_Chr07*	12010504	G/A	NA	INTERGENIC	NA
Kabuli_wild_12010547	1399941954	*Ca_kabuli_Chr07*	12010547	G/A	NA	INTERGENIC	NA
Kabuli_wild_12030080	1399941955	*Ca_kabuli_Chr07*	12030080	A/C	Gene_Ca_09301	SYNONYMOUS_CODING	PeptidaseA1
Kabuli_wild_12071764	1399941956	*Ca_kabuli_Chr07*	12071764	T/C	Gene_Ca_09297	INTRON	tRNA-dihydrouridinesynthase
Kabuli_wild_12149119	1399941957	*Ca_kabuli_Chr07*	12149119	A/G	Gene_Ca_09294	INTRON	Kinesin,motordomain
Kabuli_wild_12192653	1399941958	*Ca_kabuli_Chr07*	12192653	G/A	Gene_Ca_09289	DOWNSTREAM	Ovariantumour,otubain
Kabuli_wild_12200656	1399941959	*Ca_kabuli_Chr07*	12200656	G/A	NA	INTERGENIC	NA
Kabuli_wild_12244259	1399941960	*Ca_kabuli_Chr07*	12244259	G/A	Gene_Ca_09285	INTRON	PhospholipaseC,phosphatidylinositol-specific,Xdomain
Kabuli_wild_12384170	1399941961	*Ca_kabuli_Chr07*	12384170	T/C	Gene_Ca_09276	DOWNSTREAM	Nicotinate phosphoribosyltransferase-related
Kabuli_wild_12390774	1399941962	*Ca_kabuli_Chr07*	12390774	G/A	Gene_Ca_09275	SYNONYMOUS_CODING	Pathogenesis-related transcriptional factor/ERF, DNA-binding
Kabuli_wild_12476679	1399941963	*Ca_kabuli_Chr07*	12476679	A/C	Gene_Ca_09269	NON_SYNONYMOUS_CODING	CTLH,C-terminalLisHmotif
Kabuli_wild_12481556	1399941964	*Ca_kabuli_Chr07*	12481556	G/C	NA	INTERGENIC	NA
Kabuli_wild_12482953	1399941965	*Ca_kabuli_Chr07*	12482953	A/C	NA	INTERGENIC	NA
Kabuli_wild_12483290	1399941966	*Ca_kabuli_Chr07*	12483290	A/C	NA	INTERGENIC	NA
Kabuli_wild_12718820	1399941967	*Ca_kabuli_Chr07*	12718820	C/G	NA	INTERGENIC	NA

SNP IDs	NCBI dbSNP accession IDs (NCBI_SS#)	Chromosomes	Physical positions (bp)	SNPs	Gene accession IDs	Sequence components of genome	Putative functions
Kabuli_wild_12718853	1399941968	*Ca_kabuli_Chr07*	12718853	G/A	NA	INTERGENIC	NA
Kabuli_wild_12730600	1399941969	*Ca_kabuli_Chr07*	12730600	C/A	NA	INTERGENIC	NA
Kabuli_wild_12737516	1399941970	*Ca_kabuli_Chr07*	12737516	T/C	NA	INTERGENIC	NA
Kabuli_wild_12893655	1399941971	*Ca_kabuli_Chr07*	12893655	C/T	NA	INTERGENIC	NA
Kabuli_wild_13020754	1399941972	*Ca_kabuli_Chr07*	13020754	G/A	Gene_Ca_09225	INTRON	Vacuolar protein sorting-associated protein
Kabuli_wild_13059710	1399941973	*Ca_kabuli_Chr07*	13059710	C/A	Gene_Ca_09222	NON_SYNONYMOUS_CODING	BTB/POZ-like
Kabuli_wild_13059712	1399941974	*Ca_kabuli_Chr07*	13059712	C/A	Gene_Ca_09222	NON_SYNONYMOUS_CODING	BTB/POZ-like
Kabuli_wild_13059852	1399941975	*Ca_kabuli_Chr07*	13059852	G/A	Gene_Ca_09222	SYNONYMOUS_CODING	BTB/POZ-like
Kabuli_wild_13069842	1399941976	*Ca_kabuli_Chr07*	13069842	C/A	Gene_Ca_09221	NON_SYNONYMOUS_CODING	ColdacclimationWCOR413
Kabuli_wild_13514074	1399941977	*Ca_kabuli_Chr07*	13514074	C/T	Gene_Ca_16037	SYNONYMOUS_CODING	Protein of unknown function DUF677
Kabuli_wild_13581088	1399941978	*Ca_kabuli_Chr07*	13581088	T/C	Gene_Ca_16043	SYNONYMOUS_CODING	Transcription facto rGRAS
Kabuli_wild_13581091	1399941979	*Ca_kabuli_Chr07*	13581091	T/C	Gene_Ca_16043	SYNONYMOUS_CODING	Transcription facto rGRAS
Kabuli_wild_13605188	1399941980	*Ca_kabuli_Chr07*	13605188	A/C	Gene_Ca_16044	SYNONYMOUS_CODING	Zinc finger,C2H2-type
Kabuli_wild_13605191	1399941981	*Ca_kabuli_Chr07*	13605191	A/C	Gene_Ca_16044	SYNONYMOUS_CODING	Zinc finger,C2H2-type
Kabuli_wild_13605200	1399941982	*Ca_kabuli_Chr07*	13605200	A/C	Gene_Ca_16044	SYNONYMOUS_CODING	Zinc finger,C2H2-type
Kabuli_wild_13605574	1399941983	*Ca_kabuli_Chr07*	13605574	T/G	Gene_Ca_16044	NON_SYNONYMOUS_CODING	Zinc finger,C2H2-type
Kabuli_wild_13605578	1399941984	*Ca_kabuli_Chr07*	13605578	C/G	Gene_Ca_16044	SYNONYMOUS_CODING	Zinc finger,C2H2-type
Kabuli_wild_13832484	1399941985	*Ca_kabuli_Chr07*	13832484	G/T	Gene_Ca_16060	INTRON	Sugar/inositol transporter
Kabuli_wild_13903980	1399941986	*Ca_kabuli_Chr07*	13903980	T/G	Gene_Ca_16064	NON_SYNONYMOUS_CODING	Tubby,C-terminal
Kabuli_wild_13949259	1399941987	*Ca_kabuli_Chr07*	13949259	A/G	Gene_Ca_16067	NON_SYNONYMOUS_CODING	Heat shock protein DnaJ,N-terminal
Kabuli_wild_14051146	1399941988	*Ca_kabuli_Chr07*	14051146	G/T	Gene_Ca_16074	INTRON	NA
Kabuli_wild_14051162	1399941989	*Ca_kabuli_Chr07*	14051162	C/T	Gene_Ca_16074	INTRON	NA
Kabuli_wild_14051168	1399941990	*Ca_kabuli_Chr07*	14051168	C/T	Gene_Ca_16074	INTRON	NA
Kabuli_wild_14051191	1399941991	*Ca_kabuli_Chr07*	14051191	G/A	Gene_Ca_16074	INTRON	NA
Kabuli_wild_14161235	1399941992	*Ca_kabuli_Chr07*	14161235	A/G	Gene_Ca_23031	NON_SYNONYMOUS_CODING	NA
Kabuli_wild_14281993	1399941993	*Ca_kabuli_Chr07*	14281993	G/T	Gene_Ca_23042	NON_SYNONYMOUS_CODING	Protein of unknown function DUF702
Kabuli_wild_14321726	1399941994	*Ca_kabuli_Chr07*	14321726	T/C	NA	INTERGENIC	NA
Kabuli_wild_14328583	1399941995	*Ca_kabuli_Chr07*	14328583	G/A	NA	INTERGENIC	NA
Kabuli_wild_14328602	1399941996	*Ca_kabuli_Chr07*	14328602	T/G	NA	INTERGENIC	NA
Kabuli_wild_14328649	1399941997	*Ca_kabuli_Chr07*	14328649	T/C	NA	INTERGENIC	NA
Kabuli_wild_14328993	1399941998	*Ca_kabuli_Chr07*	14328993	C/G	NA	INTERGENIC	NA
Kabuli_wild_14329077	1399941999	*Ca_kabuli_Chr07*	14329077	A/C	NA	INTERGENIC	NA
Kabuli_wild_14329126	1399942000	*Ca_kabuli_Chr07*	14329126	C/T	NA	INTERGENIC	NA
Kabuli_wild_14329167	1399942001	*Ca_kabuli_Chr07*	14329167	A/T	NA	INTERGENIC	NA
Kabuli_wild_14329175	1399942002	*Ca_kabuli_Chr07*	14329175	G/A	NA	INTERGENIC	NA
Kabuli_wild_14329184	1399942003	*Ca_kabuli_Chr07*	14329184	T/C	NA	INTERGENIC	NA
Kabuli_wild_14329196	1399942004	*Ca_kabuli_Chr07*	14329196	G/A	NA	INTERGENIC	NA
Kabuli_wild_14329217	1399942005	*Ca_kabuli_Chr07*	14329217	T/C	NA	INTERGENIC	NA
Kabuli_wild_14345799	1399942006	*Ca_kabuli_Chr07*	14345799	T/A	Gene_Ca_23045	NON_SYNONYMOUS_CODING	D-galactoside/L-rhamnosebindingSUELlectindomain
Kabuli_wild_14470549	1399942007	*Ca_kabuli_Chr07*	14470549	C/G	Gene_Ca_09992	NON_SYNONYMOUS_CODING	Fork head-associated (FHA) domain
Kabuli_wild_14492116	1399942008	*Ca_kabuli_Chr07*	14492116	A/G	NA	INTERGENIC	NA
Kabuli_wild_14492267	1399942009	*Ca_kabuli_Chr07*	14492267	G/T	NA	INTERGENIC	NA
Kabuli_wild_14492285	1399942010	*Ca_kabuli_Chr07*	14492285	T/C	NA	INTERGENIC	NA
Kabuli_wild_14492289	1399942011	*Ca_kabuli_Chr07*	14492289	C/T	NA	INTERGENIC	NA

SNP IDs	NCBI dbSNP accession IDs (NCBI_SS#)	Chromosomes	Physical positions (bp)	SNPs	Gene accession IDs	Sequence components of genome	Putative functions
Kabuli_wild_14492341	1399942012	*Ca_kabuli_Chr07*	14492341	C/G	NA	INTERGENIC	NA
Kabuli_wild_14559055	1399942013	*Ca_kabuli_Chr07*	14559055	A/C	Gene_Ca_09987	INTRON	Protein of unknown function DUF726
Kabuli_wild_14573229	1399942014	*Ca_kabuli_Chr07*	14573229	C/G	Gene_Ca_09985	INTRON	PeptidaseS28
Kabuli_wild_14573245	1399942015	*Ca_kabuli_Chr07*	14573245	T/C	Gene_Ca_09985	INTRON	PeptidaseS28
Kabuli_wild_14573373	1399942016	*Ca_kabuli_Chr07*	14573373	C/A	Gene_Ca_09985	INTRON	PeptidaseS28
Kabuli_wild_14685011	1399942017	*Ca_kabuli_Chr07*	14685011	G/C	Gene_Ca_09976	DOWNSTREAM	Zinc finger,RanBP2-type
Kabuli_wild_14685017	1399942018	*Ca_kabuli_Chr07*	14685017	C/T	Gene_Ca_09976	DOWNSTREAM	Zinc finger,RanBP2-type
Kabuli_wild_14685024	1399942019	*Ca_kabuli_Chr07*	14685024	A/G	Gene_Ca_09976	DOWNSTREAM	Zinc finger,RanBP2-type
Kabuli_wild_14712518	1399942020	*Ca_kabuli_Chr07*	14712518	C/A	NA	INTERGENIC	NA
Kabuli_wild_14712550	1399942021	*Ca_kabuli_Chr07*	14712550	G/A	NA	INTERGENIC	NA
Kabuli_wild_14712568	1399942022	*Ca_kabuli_Chr07*	14712568	C/A	NA	INTERGENIC	NA
Kabuli_wild_14712572	1399942023	*Ca_kabuli_Chr07*	14712572	A/G	NA	INTERGENIC	NA
Kabuli_wild_14712589	1399942024	*Ca_kabuli_Chr07*	14712589	T/C	NA	INTERGENIC	NA
Kabuli_wild_14712596	1399942025	*Ca_kabuli_Chr07*	14712596	T/C	NA	INTERGENIC	NA
Kabuli_wild_14712601	1399942026	*Ca_kabuli_Chr07*	14712601	C/T	NA	INTERGENIC	NA
Kabuli_wild_14712608	1399942027	*Ca_kabuli_Chr07*	14712608	T/A	NA	INTERGENIC	NA
Kabuli_wild_14712617	1399942028	*Ca_kabuli_Chr07*	14712617	T/C	NA	INTERGENIC	NA
Kabuli_wild_14748257	1399942029	*Ca_kabuli_Chr07*	14748257	T/A	NA	INTERGENIC	NA
Kabuli_wild_14776054	1399942030	*Ca_kabuli_Chr07*	14776054	T/G	NA	INTERGENIC	NA
Kabuli_wild_14782627	1399942031	*Ca_kabuli_Chr07*	14782627	G/C	Gene_Ca_09968	SYNONYMOUS_CODING	Heat shock protein DnaJ,N-terminal
Kabuli_wild_14948610	1399942032	*Ca_kabuli_Chr07*	14948610	C/A	Gene_Ca_09958	INTRON	Senescence/spartin-associated
Kabuli_wild_14997794	1399942033	*Ca_kabuli_Chr07*	14997794	G/A	Gene_Ca_09954	NON_SYNONYMOUS_CODING	RNAhelicase,ATP-dependent,DEAD-box,conservedsite
Kabuli_wild_15039606	1399942034	*Ca_kabuli_Chr07*	15039606	T/A	Gene_Ca_09950	DOWNSTREAM	Pentatrico peptide repeat
Kabuli_wild_15070091	1399942035	*Ca_kabuli_Chr07*	15070091	T/A	Gene_Ca_09947	INTRON	Uncharacterised protein familyCys-rich
Kabuli_wild_15125638	1399942036	*Ca_kabuli_Chr07*	15125638	G/C	Gene_Ca_09942	NON_SYNONYMOUS_CODING	PeptidaseA1
Kabuli_wild_15399369	1399942037	*Ca_kabuli_Chr07*	15399369	A/C	Gene_Ca_09920	SYNONYMOUS_CODING	Glycosidehydrolase,family19,catalytic
Kabuli_wild_15399421	1399942038	*Ca_kabuli_Chr07*	15399421	T/C	Gene_Ca_09920	NON_SYNONYMOUS_CODING	Glycosidehydrolase,family19,catalytic
Kabuli_wild_15441347	1399942039	*Ca_kabuli_Chr07*	15441347	T/G	NA	INTERGENIC	NA
Kabuli_wild_15462772	1399942040	*Ca_kabuli_Chr07*	15462772	A/T	Gene_Ca_09916	NON_SYNONYMOUS_CODING	AMP-dependentsynthetase/ligase
Kabuli_wild_15462807	1399942041	*Ca_kabuli_Chr07*	15462807	G/C	Gene_Ca_09916	SYNONYMOUS_CODING	AMP-dependentsynthetase/ligase
Kabuli_wild_15463169	1399942042	*Ca_kabuli_Chr07*	15463169	G/A	Gene_Ca_09916	SYNONYMOUS_CODING	AMP-dependentsynthetase/ligase
Kabuli_wild_15463183	1399942043	*Ca_kabuli_Chr07*	15463183	G/T	Gene_Ca_09916	NON_SYNONYMOUS_CODING	AMP-dependentsynthetase/ligase
Kabuli_wild_15529918	1399942044	*Ca_kabuli_Chr07*	15529918	T/A	Gene_Ca_09913	SYNONYMOUS_CODING	AMP-dependentsynthetase/ligase
Kabuli_wild_15529953	1399942045	*Ca_kabuli_Chr07*	15529953	C/G	Gene_Ca_09913	SYNONYMOUS_CODING	AMP-dependentsynthetase/ligase
Kabuli_wild_15530315	1399942046	*Ca_kabuli_Chr07*	15530315	G/A	Gene_Ca_09913	SYNONYMOUS_CODING	AMP-dependentsynthetase/ligase
Kabuli_wild_15530329	1399942047	*Ca_kabuli_Chr07*	15530329	G/T	Gene_Ca_09913	NON_SYNONYMOUS_CODING	AMP-dependentsynthetase/ligase
Kabuli_wild_15568017	1399942048	*Ca_kabuli_Chr07*	15568017	C/A	NA	INTERGENIC	NA
Kabuli_wild_15568046	1399942049	*Ca_kabuli_Chr07*	15568046	G/A	NA	INTERGENIC	NA
Kabuli_wild_15568060	1399942050	*Ca_kabuli_Chr07*	15568060	G/T	NA	INTERGENIC	NA
Kabuli_wild_15622251	1399942051	*Ca_kabuli_Chr07*	15622251	C/T	Gene_Ca_09907	INTRON	Ubiquitin-associated/translation elongation factor EF1B, N-terminal
Kabuli_wild_15624645	1399942052	*Ca_kabuli_Chr07*	15624645	T/G	Gene_Ca_09907	SYNONYMOUS_CODING	Ubiquitin-associated/translation elongation factor EF1B, N-terminal
Kabuli_wild_15826588	1399942053	*Ca_kabuli_Chr07*	15826588	A/C	Gene_Ca_09889	SYNONYMOUS_CODING	Lipid-bindingSTART
Kabuli_wild_15830468	1399942054	*Ca_kabuli_Chr07*	15830468	C/T	Gene_Ca_09889	UPSTREAM	Lipid-bindingSTART
Kabuli_wild_15966680	1399942055	*Ca_kabuli_Chr07*	15966680	A/G	Gene_Ca_09880	SYNONYMOUS_CODING	Sugar/inositol transporter

SNP IDs	NCBI dbSNP accession IDs (NCBI_SS#)	Chromosomes	Physical positions (bp)	SNPs	Gene accession IDs	Sequence components of genome	Putative functions
Kabuli_wild_15966709	1399942056	*Ca_kabuli_Chr07*	15966709	T/C	Gene_Ca_09880	NON_SYNONYMOUS_CODING	Sugar/inositol transporter
Kabuli_wild_15966714	1399942057	*Ca_kabuli_Chr07*	15966714	G/A	Gene_Ca_09880	NON_SYNONYMOUS_CODING	Sugar/inositol transporter
Kabuli_wild_15966727	1399942058	*Ca_kabuli_Chr07*	15966727	T/C	Gene_Ca_09880	NON_SYNONYMOUS_CODING	Sugar/inositol transporter
Kabuli_wild_15966762	1399942059	*Ca_kabuli_Chr07*	15966762	G/A	Gene_Ca_09880	NON_SYNONYMOUS_CODING	Sugar/inositol transporter
Kabuli_wild_15966798	1399942060	*Ca_kabuli_Chr07*	15966798	C/A	Gene_Ca_09880	SYNONYMOUS_CODING	Sugar/inositol transporter
Kabuli_wild_16289794	1399942061	*Ca_kabuli_Chr07*	16289794	G/T	Gene_Ca_09857	NON_SYNONYMOUS_CODING	Tafazzin
Kabuli_wild_16324985	1399942062	*Ca_kabuli_Chr07*	16324985	T/C	Gene_Ca_09853	INTRON	Drug/metabolitetransporter
Kabuli_wild_16526921	1399942063	*Ca_kabuli_Chr07*	16526921	A/G	Gene_Ca_20554	NON_SYNONYMOUS_CODING	Sugar/inositol transporter
Kabuli_wild_16624789	1399942064	*Ca_kabuli_Chr07*	16624789	A/C	Gene_Ca_20562	NON_SYNONYMOUS_CODING	Nodulin-like
Kabuli_wild_16626047	1399942065	*Ca_kabuli_Chr07*	16626047	T/G	Gene_Ca_20562	SYNONYMOUS_CODING	Nodulin-like
Kabuli_wild_16663311	1399942066	*Ca_kabuli_Chr07*	16663311	A/C	Gene_Ca_20568	SYNONYMOUS_CODING	Tetra tricopeptide repeat-containing
Kabuli_wild_16665577	1399942067	*Ca_kabuli_Chr07*	16665577	A/G	Gene_Ca_20568	SYNONYMOUS_CODING	Tetra tricopeptide repeat-containing
Kabuli_wild_16735437	1399942068	*Ca_kabuli_Chr07*	16735437	G/A	Gene_Ca_20573	UPSTREAM	Brevisradix-likedomain
Kabuli_wild_16821274	1399942069	*Ca_kabuli_Chr07*	16821274	A/C	Gene_Ca_20581	NON_SYNONYMOUS_CODING	NA
Kabuli_wild_16936123	1399942070	*Ca_kabuli_Chr07*	16936123	C/A	Gene_Ca_15885	NON_SYNONYMOUS_CODING	H/ACAribonucleoproteincomplex,subunitNop10
Kabuli_wild_17133005	1399942071	*Ca_kabuli_Chr07*	17133005	G/A	NA	INTERGENIC	NA
Kabuli_wild_17133013	1399942072	*Ca_kabuli_Chr07*	17133013	C/A	NA	INTERGENIC	NA
Kabuli_wild_17133036	1399942073	*Ca_kabuli_Chr07*	17133036	T/C	NA	INTERGENIC	NA
Kabuli_wild_17133046	1399942074	*Ca_kabuli_Chr07*	17133046	T/G	NA	INTERGENIC	NA
Kabuli_wild_17133061	1399942075	*Ca_kabuli_Chr07*	17133061	C/T	NA	INTERGENIC	NA
Kabuli_wild_17133076	1399942076	*Ca_kabuli_Chr07*	17133076	A/G	NA	INTERGENIC	NA
Kabuli_wild_17133111	1399942077	*Ca_kabuli_Chr07*	17133111	T/G	NA	INTERGENIC	NA
Kabuli_wild_17140664	1399942078	*Ca_kabuli_Chr07*	17140664	C/T	NA	INTERGENIC	NA
Kabuli_wild_17223116	1399942079	*Ca_kabuli_Chr07*	17223116	A/C	Gene_Ca_15866	SYNONYMOUS_CODING	NA
Kabuli_wild_17228904	1399942080	*Ca_kabuli_Chr07*	17228904	T/G	NA	INTERGENIC	NA
Kabuli_wild_17318119	1399942081	*Ca_kabuli_Chr07*	17318119	A/G	Gene_Ca_15859	SYNONYMOUS_CODING	NA
Kabuli_wild_17318174	1399942082	*Ca_kabuli_Chr07*	17318174	G/T	Gene_Ca_15859	NON_SYNONYMOUS_CODING	NA
Kabuli_wild_17588267	1399942083	*Ca_kabuli_Chr07*	17588267	A/C	Gene_Ca_15850	INTRON	SET domain
Kabuli_wild_17766914	1399942084	*Ca_kabuli_Chr07*	17766914	C/A	NA	INTERGENIC	NA
Kabuli_wild_17766952	1399942085	*Ca_kabuli_Chr07*	17766952	T/A	NA	INTERGENIC	NA
Kabuli_wild_17779414	1399942086	*Ca_kabuli_Chr07*	17779414	A/G	Gene_Ca_15839	NON_SYNONYMOUS_CODING	Bromodomain
Kabuli_wild_17879307	1399942087	*Ca_kabuli_Chr07*	17879307	T/G	NA	INTERGENIC	NA
Kabuli_wild_17879391	1399942088	*Ca_kabuli_Chr07*	17879391	G/C	NA	INTERGENIC	NA
Kabuli_wild_18005844	1399942089	*Ca_kabuli_Chr07*	18005844	G/A	NA	INTERGENIC	NA
Kabuli_wild_18011500	1399942090	*Ca_kabuli_Chr07*	18011500	A/C	Gene_Ca_15826	INTRON	Protein kinase,catalytic domain
Kabuli_wild_18011604	1399942091	*Ca_kabuli_Chr07*	18011604	G/C	Gene_Ca_15826	NON_SYNONYMOUS_CODING	Basic-leucine zipper (bZIP) Transcription factor
Kabuli_wild_18090289	1399942092	*Ca_kabuli_Chr07*	18090289	T/A	NA	INTERGENIC	NA
Kabuli_wild_18319049	1399942093	*Ca_kabuli_Chr07*	18319049	T/G	NA	INTERGENIC	NA
Kabuli_wild_18319073	1399942094	*Ca_kabuli_Chr07*	18319073	T/G	NA	INTERGENIC	NA
Kabuli_wild_18319079	1399942095	*Ca_kabuli_Chr07*	18319079	C/A	NA	INTERGENIC	NA
Kabuli_wild_18365218	1399942096	*Ca_kabuli_Chr07*	18365218	G/A	NA	INTERGENIC	NA
Kabuli_wild_18398083	1399942097	*Ca_kabuli_Chr07*	18398083	T/G	NA	INTERGENIC	NA
Kabuli_wild_18462115	1399942098	*Ca_kabuli_Chr07*	18462115	G/C	Gene_Ca_22127	INTRON	NA
Kabuli_wild_18469422	1399942099	*Ca_kabuli_Chr07*	18469422	C/T	Gene_Ca_22127	NON_SYNONYMOUS_CODING	NA

SNP IDs	NCBI dbSNP accession IDs (NCBI_SS#)	Chromosomes	Physical positions (bp)	SNPs	Gene accession IDs	Sequence components of genome	Putative functions
Kabuli_wild_18638565	1399942100	*Ca_kabuli_Chr07*	18638565	A/G	Gene_Ca_12302	NON_SYNONYMOUS_CODING	NA
Kabuli_wild_18642538	1399942101	*Ca_kabuli_Chr07*	18642538	A/G	Gene_Ca_12302	NON_SYNONYMOUS_CODING	NA
Kabuli_wild_18658563	1399942102	*Ca_kabuli_Chr07*	18658563	C/G	Gene_Ca_12303	SYNONYMOUS_CODING	Phosphatetransporter
Kabuli_wild_18696645	1399942103	*Ca_kabuli_Chr07*	18696645	G/A	NA	INTERGENIC	NA
Kabuli_wild_18728531	1399942104	*Ca_kabuli_Chr07*	18728531	G/C	Gene_Ca_12306	INTRON	GRAM
Kabuli_wild_18910468	1399942105	*Ca_kabuli_Chr07*	18910468	A/G	Gene_Ca_12312	DOWNSTREAM	WD40 repeat
Kabuli_wild_18946770	1399942106	*Ca_kabuli_Chr07*	18946770	A/C	Gene_Ca_12313	SYNONYMOUS_CODING	Mitochondrialbrownfatuncouplingprotein
Kabuli_wild_19042183	1399942107	*Ca_kabuli_Chr07*	19042183	C/T	Gene_Ca_12316	INTRON	Kinesin,motordomain
Kabuli_wild_19189784	1399942108	*Ca_kabuli_Chr07*	19189784	C/A	Gene_Ca_12326	UPSTREAM	Xanthine/uracil/vitamin C permease
Kabuli_wild_19195625	1399942109	*Ca_kabuli_Chr07*	19195625	A/C	Gene_Ca_12326	NON_SYNONYMOUS_CODING	Xanthine/uracil/vitamin C permease
Kabuli_wild_19217501	1399942110	*Ca_kabuli_Chr07*	19217501	A/C	Gene_Ca_12328	NON_SYNONYMOUS_CODING	Pathogenesis-related transcriptional factor/ERF, DNA-binding
Kabuli_wild_19241112	1399942111	*Ca_kabuli_Chr07*	19241112	T/C	Gene_Ca_12331	NON_SYNONYMOUS_CODING	WD40 repeat
Kabuli_wild_19252437	1399942112	*Ca_kabuli_Chr07*	19252437	T/C	Gene_Ca_12332	INTRON	Dehydrogenase,E1component
Kabuli_wild_19365654	1399942113	*Ca_kabuli_Chr07*	19365654	G/A	NA	INTERGENIC	NA
Kabuli_wild_19418115	1399942114	*Ca_kabuli_Chr07*	19418115	T/C	NA	INTERGENIC	NA
Kabuli_wild_19472689	1399942115	*Ca_kabuli_Chr07*	19472689	T/A	NA	INTERGENIC	NA
Kabuli_wild_19519093	1399942116	*Ca_kabuli_Chr07*	19519093	T/C	Gene_Ca_12347	INTRON	tRNA-dihydrouridinesynthase
Kabuli_wild_19538108	1399942117	*Ca_kabuli_Chr07*	19538108	T/G	Gene_Ca_12348	INTRON	Phosphoenolpyruvatecarboxykinase,ATP-utilising
Kabuli_wild_19576442	1399942118	*Ca_kabuli_Chr07*	19576442	T/C	NA	INTERGENIC	NA
Kabuli_wild_19576447	1399942119	*Ca_kabuli_Chr07*	19576447	C/T	NA	INTERGENIC	NA
Kabuli_wild_19576457	1399942120	*Ca_kabuli_Chr07*	19576457	G/A	NA	INTERGENIC	NA
Kabuli_wild_19576472	1399942121	*Ca_kabuli_Chr07*	19576472	C/A	NA	INTERGENIC	NA
Kabuli_wild_19787497	1399942122	*Ca_kabuli_Chr07*	19787497	C/A	NA	INTERGENIC	NA
Kabuli_wild_19818757	1399942123	*Ca_kabuli_Chr07*	19818757	G/C	NA	INTERGENIC	NA
Kabuli_wild_19818759	1399942124	*Ca_kabuli_Chr07*	19818759	G/C	NA	INTERGENIC	NA
Kabuli_wild_19818761	1399942125	*Ca_kabuli_Chr07*	19818761	C/T	NA	INTERGENIC	NA
Kabuli_wild_19818770	1399942126	*Ca_kabuli_Chr07*	19818770	T/C	NA	INTERGENIC	NA
Kabuli_wild_19818789	1399942127	*Ca_kabuli_Chr07*	19818789	C/A	NA	INTERGENIC	NA
Kabuli_wild_19818793	1399942128	*Ca_kabuli_Chr07*	19818793	T/C	NA	INTERGENIC	NA
Kabuli_wild_19989645	1399942129	*Ca_kabuli_Chr07*	19989645	G/C	NA	INTERGENIC	NA
Kabuli_wild_20031805	1399942130	*Ca_kabuli_Chr07*	20031805	T/C	NA	INTERGENIC	NA
Kabuli_wild_20032205	1399942131	*Ca_kabuli_Chr07*	20032205	A/C	Gene_Ca_12371	SYNONYMOUS_CODING	General substrate transporter
Kabuli_wild_20097908	1399942132	*Ca_kabuli_Chr07*	20097908	A/G	Gene_Ca_12375	NON_SYNONYMOUS_CODING	Target SNARE coiled-coil domain
Kabuli_wild_20175669	1399942133	*Ca_kabuli_Chr07*	20175669	T/C	Gene_Ca_12380	UPSTREAM	NA
Kabuli_wild_20213416	1399942134	*Ca_kabuli_Chr07*	20213416	A/G	NA	INTERGENIC	NA
Kabuli_wild_20252133	1399942135	*Ca_kabuli_Chr07*	20252133	T/C	Gene_Ca_12383	DOWNSTREAM	Protein of unknown function DUF220
Kabuli_wild_20252134	1399942136	*Ca_kabuli_Chr07*	20252134	G/A	Gene_Ca_12383	DOWNSTREAM	Protein of unknown function DUF220
Kabuli_wild_20252135	1399942137	*Ca_kabuli_Chr07*	20252135	A/G	Gene_Ca_12383	DOWNSTREAM	Protein of unknown function DUF220
Kabuli_wild_20252144	1399942138	*Ca_kabuli_Chr07*	20252144	G/A	Gene_Ca_12383	DOWNSTREAM	Protein of unknown function DUF220
Kabuli_wild_20252145	1399942139	*Ca_kabuli_Chr07*	20252145	G/A	Gene_Ca_12383	DOWNSTREAM	Protein of unknown function DUF220
Kabuli_wild_20252179	1399942140	*Ca_kabuli_Chr07*	20252179	T/G	Gene_Ca_12383	DOWNSTREAM	Protein of unknown function DUF220
Kabuli_wild_20252181	1399942141	*Ca_kabuli_Chr07*	20252181	C/T	Gene_Ca_12383	DOWNSTREAM	Protein of unknown function DUF220
Kabuli_wild_20252192	1399942142	*Ca_kabuli_Chr07*	20252192	G/A	Gene_Ca_12383	DOWNSTREAM	Protein of unknown function DUF220
Kabuli_wild_20252201	1399942143	*Ca_kabuli_Chr07*	20252201	C/A	Gene_Ca_12383	DOWNSTREAM	Protein of unknown function DUF220

SNP IDs	NCBI dbSNP accession IDs (NCBI_SS#)	Chromosomes	Physical positions (bp)	SNPs	Gene accession IDs	Sequence components of genome	Putative functions
Kabuli_wild_20252219	1399942144	*Ca_kabuli_Chr07*	20252219	C/T	Gene_Ca_12383	DOWNSTREAM	Protein of unknown function DUF220
Kabuli_wild_20252221	1399942145	*Ca_kabuli_Chr07*	20252221	T/C	Gene_Ca_12383	DOWNSTREAM	Protein of unknown function DUF220
Kabuli_wild_20252233	1399942146	*Ca_kabuli_Chr07*	20252233	C/T	Gene_Ca_12383	DOWNSTREAM	Protein of unknown function DUF220
Kabuli_wild_20252309	1399942147	*Ca_kabuli_Chr07*	20252309	T/C	Gene_Ca_12383	DOWNSTREAM	Protein of unknown function DUF220
Kabuli_wild_20252334	1399942148	*Ca_kabuli_Chr07*	20252334	A/T	Gene_Ca_12383	DOWNSTREAM	Protein of unknown function DUF220
Kabuli_wild_20252340	1399942149	*Ca_kabuli_Chr07*	20252340	A/G	Gene_Ca_12383	DOWNSTREAM	Protein of unknown function DUF220
Kabuli_wild_20414703	1399942150	*Ca_kabuli_Chr07*	20414703	C/A	Gene_Ca_12394	DOWNSTREAM	DNA-directedRNApolymerase,bacteriophagetype
Kabuli_wild_20414704	1399942151	*Ca_kabuli_Chr07*	20414704	G/A	Gene_Ca_12394	DOWNSTREAM	DNA-directedRNApolymerase,bacteriophagetype
Kabuli_wild_20425287	1399942152	*Ca_kabuli_Chr07*	20425287	A/G	Gene_Ca_12394	NON_SYNONYMOUS_CODING	DNA-directedRNApolymerase,bacteriophagetype
Kabuli_wild_20452278	1399942153	*Ca_kabuli_Chr07*	20452278	A/G	Gene_Ca_12396	SYNONYMOUS_CODING	Protein kinase,catalytic domain
Kabuli_wild_20452457	1399942154	*Ca_kabuli_Chr07*	20452457	G/A	Gene_Ca_12396	NON_SYNONYMOUS_CODING	Protein kinase,catalytic domain
Kabuli_wild_20496303	1399942155	*Ca_kabuli_Chr07*	20496303	C/T	Gene_Ca_12400	SYNONYMOUS_CODING	Pathogenesis-related transcriptional factor/ERF, DNA-binding
Kabuli_wild_20496378	1399942156	*Ca_kabuli_Chr07*	20496378	G/A	Gene_Ca_12400	SYNONYMOUS_CODING	Pathogenesis-related transcriptional factor/ERF, DNA-binding
Kabuli_wild_20496438	1399942157	*Ca_kabuli_Chr07*	20496438	C/T	Gene_Ca_12400	SYNONYMOUS_CODING	Pathogenesis-related transcriptional factor/ERF, DNA-binding
Kabuli_wild_20498552	1399942158	*Ca_kabuli_Chr07*	20498552	T/C	Gene_Ca_12400	INTRON	Pathogenesis-related transcriptional factor/ERF, DNA-binding
Kabuli_wild_20498626	1399942159	*Ca_kabuli_Chr07*	20498626	G/T	Gene_Ca_12400	INTRON	Pathogenesis-related transcriptional factor/ERF, DNA-binding
Kabuli_wild_20540780	1399942160	*Ca_kabuli_Chr07*	20540780	C/T	Gene_Ca_12403	DOWNSTREAM	Protein kinase,catalytic domain
Kabuli_wild_20540791	1399942161	*Ca_kabuli_Chr07*	20540791	G/A	Gene_Ca_12403	DOWNSTREAM	Protein kinase,catalytic domain
Kabuli_wild_20543112	1399942162	*Ca_kabuli_Chr07*	20543112	T/C	NA	INTERGENIC	NA
Kabuli_wild_20543119	1399942163	*Ca_kabuli_Chr07*	20543119	T/C	NA	INTERGENIC	NA
Kabuli_wild_20550563	1399942164	*Ca_kabuli_Chr07*	20550563	G/A	NA	INTERGENIC	NA
Kabuli_wild_20550575	1399942165	*Ca_kabuli_Chr07*	20550575	C/G	NA	INTERGENIC	NA
Kabuli_wild_20550619	1399942166	*Ca_kabuli_Chr07*	20550619	A/G	NA	INTERGENIC	NA
Kabuli_wild_20552041	1399942167	*Ca_kabuli_Chr07*	20552041	G/A	Gene_Ca_12405	SYNONYMOUS_CODING	SGT1
Kabuli_wild_20552053	1399942168	*Ca_kabuli_Chr07*	20552053	C/G	Gene_Ca_12405	SYNONYMOUS_CODING	SGT1
Kabuli_wild_20556466	1399942169	*Ca_kabuli_Chr07*	20556466	G/C	NA	INTERGENIC	NA
Kabuli_wild_20556576	1399942170	*Ca_kabuli_Chr07*	20556576	C/T	NA	INTERGENIC	NA
Kabuli_wild_20560421	1399942171	*Ca_kabuli_Chr07*	20560421	G/A	NA	INTERGENIC	NA
Kabuli_wild_20563931	1399942172	*Ca_kabuli_Chr07*	20563931	G/A	Gene_Ca_12406	SYNONYMOUS_CODING	KIP1-like
Kabuli_wild_20564024	1399942173	*Ca_kabuli_Chr07*	20564024	G/A	Gene_Ca_12406	SYNONYMOUS_CODING	KIP1-like
Kabuli_wild_20564266	1399942174	*Ca_kabuli_Chr07*	20564266	G/A	Gene_Ca_12406	NON_SYNONYMOUS_CODING	KIP1-like
Kabuli_wild_20564269	1399942175	*Ca_kabuli_Chr07*	20564269	C/T	Gene_Ca_12406	NON_SYNONYMOUS_CODING	KIP1-like
Kabuli_wild_20564297	1399942176	*Ca_kabuli_Chr07*	20564297	A/G	Gene_Ca_12406	SYNONYMOUS_CODING	KIP1-like
Kabuli_wild_20579963	1399942177	*Ca_kabuli_Chr07*	20579963	G/T	NA	INTERGENIC	NA
Kabuli_wild_20579974	1399942178	*Ca_kabuli_Chr07*	20579974	A/T	NA	INTERGENIC	NA
Kabuli_wild_20586045	1399942179	*Ca_kabuli_Chr07*	20586045	G/T	Gene_Ca_12409	DOWNSTREAM	Integrase,catalyticcore
Kabuli_wild_20605373	1399942180	*Ca_kabuli_Chr07*	20605373	A/C	Gene_Ca_12411	DOWNSTREAM	NA
Kabuli_wild_20605384	1399942181	*Ca_kabuli_Chr07*	20605384	C/G	Gene_Ca_12411	DOWNSTREAM	NA
Kabuli_wild_20605389	1399942182	*Ca_kabuli_Chr07*	20605389	A/G	Gene_Ca_12411	DOWNSTREAM	NA
Kabuli_wild_20610728	1399942183	*Ca_kabuli_Chr07*	20610728	A/C	Gene_Ca_12413	DOWNSTREAM	MechanosensitiveionchannelMscS
Kabuli_wild_20610732	1399942184	*Ca_kabuli_Chr07*	20610732	T/G	Gene_Ca_12413	DOWNSTREAM	NA
Kabuli_wild_20613547	1399942185	*Ca_kabuli_Chr07*	20613547	A/G	NA	INTERGENIC	NA
Kabuli_wild_20613659	1399942186	*Ca_kabuli_Chr07*	20613659	T/A	NA	INTERGENIC	NA
Kabuli_wild_20613718	1399942187	*Ca_kabuli_Chr07*	20613718	C/T	NA	INTERGENIC	NA

SNP IDs	NCBI dbSNP accession IDs (NCBI_SS#)	Chromosomes	Physical positions (bp)	SNPs	Gene accession IDs	Sequence components of genome	Putative functions
Kabuli_wild_20632901	1399942188	*Ca_kabuli_Chr07*	20632901	C/T	NA	INTERGENIC	NA
Kabuli_wild_20633040	1399942189	*Ca_kabuli_Chr07*	20633040	A/G	NA	INTERGENIC	NA
Kabuli_wild_20637152	1399942190	*Ca_kabuli_Chr07*	20637152	G/A	Gene_Ca_12415	SYNONYMOUS_CODING	Protein synthesis factor, GTP-binding
Kabuli_wild_20917740	1399942191	*Ca_kabuli_Chr07*	20917740	A/G	NA	INTERGENIC	NA
Kabuli_wild_20917742	1399942192	*Ca_kabuli_Chr07*	20917742	C/A	NA	INTERGENIC	NA
Kabuli_wild_20917743	1399942193	*Ca_kabuli_Chr07*	20917743	T/G	NA	INTERGENIC	NA
Kabuli_wild_20917747	1399942194	*Ca_kabuli_Chr07*	20917747	A/G	NA	INTERGENIC	NA
Kabuli_wild_21073385	1399942195	*Ca_kabuli_Chr07*	21073385	A/C	Gene_Ca_14490	NON_SYNONYMOUS_CODING	Alcohol dehydrogenase superfamily, zinc-containing
Kabuli_wild_21099782	1399942196	*Ca_kabuli_Chr07*	21099782	G/T	NA	INTERGENIC	NA
Kabuli_wild_21401261	1399942197	*Ca_kabuli_Chr07*	21401261	C/T	Gene_Ca_14503	NON_SYNONYMOUS_CODING	Aldehydedehydrogenasedomain
Kabuli_wild_21402075	1399942198	*Ca_kabuli_Chr07*	21402075	A/G	Gene_Ca_14503	SYNONYMOUS_CODING	Aldehydedehydrogenasedomain
Kabuli_wild_21402117	1399942199	*Ca_kabuli_Chr07*	21402117	A/C	Gene_Ca_14503	INTRON	Aldehydedehydrogenasedomain
Kabuli_wild_21595525	1399942200	*Ca_kabuli_Chr07*	21595525	C/T	NA	INTERGENIC	NA
Kabuli_wild_21616285	1399942201	*Ca_kabuli_Chr07*	21616285	A/G	Gene_Ca_14512	INTRON	Clathrin/coatomeradaptor,adaptin-like,N-terminal
Kabuli_wild_21616287	1399942202	*Ca_kabuli_Chr07*	21616287	T/G	Gene_Ca_14512	INTRON	Clathrin/coatomeradaptor,adaptin-like,N-terminal
Kabuli_wild_21616290	1399942203	*Ca_kabuli_Chr07*	21616290	A/G	Gene_Ca_14512	INTRON	Clathrin/coatomeradaptor,adaptin-like,N-terminal
Kabuli_wild_21616294	1399942204	*Ca_kabuli_Chr07*	21616294	T/G	Gene_Ca_14512	INTRON	Clathrin/coatomeradaptor,adaptin-like,N-terminal
Kabuli_wild_21633103	1399942205	*Ca_kabuli_Chr07*	21633103	C/G	Gene_Ca_14515	UPSTREAM	Clathrin/coatomeradaptor,adaptin-like,N-terminal
Kabuli_wild_21639234	1399942206	*Ca_kabuli_Chr07*	21639234	G/A	Gene_Ca_14515	INTRON	Clathrin/coatomeradaptor,adaptin-like,N-terminal
Kabuli_wild_21639238	1399942207	*Ca_kabuli_Chr07*	21639238	G/A	Gene_Ca_14515	INTRON	Clathrin/coatomeradaptor,adaptin-like,N-terminal
Kabuli_wild_21639244	1399942208	*Ca_kabuli_Chr07*	21639244	T/G	Gene_Ca_14515	INTRON	Clathrin/coatomeradaptor,adaptin-like,N-terminal
Kabuli_wild_21639251	1399942209	*Ca_kabuli_Chr07*	21639251	T/G	Gene_Ca_14515	INTRON	Clathrin/coatomeradaptor,adaptin-like,N-terminal
Kabuli_wild_21639254	1399942210	*Ca_kabuli_Chr07*	21639254	T/G	Gene_Ca_14515	INTRON	Clathrin/coatomeradaptor,adaptin-like,N-terminal
Kabuli_wild_21639375	1399942211	*Ca_kabuli_Chr07*	21639375	T/G	Gene_Ca_14515	INTRON	Clathrin/coatomeradaptor,adaptin-like,N-terminal
Kabuli_wild_21644279	1399942212	*Ca_kabuli_Chr07*	21644279	C/T	Gene_Ca_14515	DOWNSTREAM	Clathrin/coatomeradaptor,adaptin-like,N-terminal
Kabuli_wild_21644342	1399942213	*Ca_kabuli_Chr07*	21644342	G/A	Gene_Ca_14515	DOWNSTREAM	Clathrin/coatomeradaptor,adaptin-like,N-terminal
Kabuli_wild_21690733	1399942214	*Ca_kabuli_Chr07*	21690733	T/C	NA	INTERGENIC	NA
Kabuli_wild_21713147	1399942215	*Ca_kabuli_Chr07*	21713147	T/G	Gene_Ca_14519	INTRON	NA
Kabuli_wild_21744514	1399942216	*Ca_kabuli_Chr07*	21744514	T/C	NA	INTERGENIC	NA
Kabuli_wild_21756162	1399942217	*Ca_kabuli_Chr07*	21756162	A/G	NA	INTERGENIC	NA
Kabuli_wild_21756166	1399942218	*Ca_kabuli_Chr07*	21756166	T/C	NA	INTERGENIC	NA
Kabuli_wild_22698940	1399942219	*Ca_kabuli_Chr07*	22698940	G/A	Gene_Ca_19532	SYNONYMOUS_CODING	Kinesin,motordomain
Kabuli_wild_22721332	1399942220	*Ca_kabuli_Chr07*	22721332	G/C	Gene_Ca_19530	NON_SYNONYMOUS_CODING	Haloaciddehalogenase-likehydrolase
Kabuli_wild_22721356	1399942221	*Ca_kabuli_Chr07*	22721356	A/C	Gene_Ca_19530	SYNONYMOUS_CODING	Haloaciddehalogenase-likehydrolase
Kabuli_wild_22738152	1399942222	*Ca_kabuli_Chr07*	22738152	C/G	Gene_Ca_19530	INTRON	Haloaciddehalogenase-likehydrolase
Kabuli_wild_22738482	1399942223	*Ca_kabuli_Chr07*	22738482	A/G	Gene_Ca_19530	INTRON	Haloaciddehalogenase-likehydrolase
Kabuli_wild_22803861	1399942224	*Ca_kabuli_Chr07*	22803861	A/C	NA	INTERGENIC	NA
Kabuli_wild_22803872	1399942225	*Ca_kabuli_Chr07*	22803872	C/T	NA	INTERGENIC	NA
Kabuli_wild_22803878	1399942226	*Ca_kabuli_Chr07*	22803878	G/T	NA	INTERGENIC	NA
Kabuli_wild_22803926	1399942227	*Ca_kabuli_Chr07*	22803926	C/T	NA	INTERGENIC	NA
Kabuli_wild_22808687	1399942228	*Ca_kabuli_Chr07*	22808687	C/T	NA	INTERGENIC	NA
Kabuli_wild_22808696	1399942229	*Ca_kabuli_Chr07*	22808696	A/G	NA	INTERGENIC	NA
Kabuli_wild_22808717	1399942230	*Ca_kabuli_Chr07*	22808717	C/T	NA	INTERGENIC	NA
Kabuli_wild_22808718	1399942231	*Ca_kabuli_Chr07*	22808718	A/C	NA	INTERGENIC	NA

SNP IDs	NCBI dbSNP accession IDs (NCBI_SS#)	Chromosomes	Physical positions (bp)	SNPs	Gene accession IDs	Sequence components of genome	Putative functions
Kabuli_wild_22808736	1399942232	*Ca_kabuli_Chr07*	22808736	A/G	NA	INTERGENIC	NA
Kabuli_wild_22839776	1399942233	*Ca_kabuli_Chr07*	22839776	G/A	NA	INTERGENIC	NA
Kabuli_wild_22973144	1399942234	*Ca_kabuli_Chr07*	22973144	G/A	Gene_Ca_19520	INTRON	F-boxdomain,cyclin-like
Kabuli_wild_22973145	1399942235	*Ca_kabuli_Chr07*	22973145	C/A	Gene_Ca_19520	INTRON	F-boxdomain,cyclin-like
Kabuli_wild_22987768	1399942236	*Ca_kabuli_Chr07*	22987768	G/A	NA	INTERGENIC	NA
Kabuli_wild_22987777	1399942237	*Ca_kabuli_Chr07*	22987777	A/T	NA	INTERGENIC	NA
Kabuli_wild_22987782	1399942238	*Ca_kabuli_Chr07*	22987782	G/A	NA	INTERGENIC	NA
Kabuli_wild_22987803	1399942239	*Ca_kabuli_Chr07*	22987803	C/T	NA	INTERGENIC	NA
Kabuli_wild_22987809	1399942240	*Ca_kabuli_Chr07*	22987809	C/T	NA	INTERGENIC	NA
Kabuli_wild_23243057	1399942241	*Ca_kabuli_Chr07*	23243057	T/C	NA	INTERGENIC	NA
Kabuli_wild_23243077	1399942242	*Ca_kabuli_Chr07*	23243077	T/C	NA	INTERGENIC	NA
Kabuli_wild_23574823	1399942243	*Ca_kabuli_Chr07*	23574823	C/T	NA	INTERGENIC	NA
Kabuli_wild_23575629	1399942244	*Ca_kabuli_Chr07*	23575629	G/A	Gene_Ca_26017	NON_SYNONYMOUS_CODING	Phosphatidylinositol3-/4-kinase,catalytic
Kabuli_wild_23616050	1399942245	*Ca_kabuli_Chr07*	23616050	A/G	Gene_Ca_26014	INTRON	Translin
Kabuli_wild_24021690	1399942246	*Ca_kabuli_Chr07*	24021690	T/A	Gene_Ca_19916	NON_SYNONYMOUS_CODING	ZF-HD homeobox protein,Cys/His-rich dimerisation domain
Kabuli_wild_24181321	1399942247	*Ca_kabuli_Chr07*	24181321	G/A	Gene_Ca_19912	NON_SYNONYMOUS_CODING	Zinc finger,RING-CH-type
Kabuli_wild_24190633	1399942248	*Ca_kabuli_Chr07*	24190633	G/A	NA	INTERGENIC	NA
Kabuli_wild_24190636	1399942249	*Ca_kabuli_Chr07*	24190636	G/A	NA	INTERGENIC	NA
Kabuli_wild_24190645	1399942250	*Ca_kabuli_Chr07*	24190645	A/T	NA	INTERGENIC	NA
Kabuli_wild_24190650	1399942251	*Ca_kabuli_Chr07*	24190650	G/A	NA	INTERGENIC	NA
Kabuli_wild_24190671	1399942252	*Ca_kabuli_Chr07*	24190671	C/T	NA	INTERGENIC	NA
Kabuli_wild_24190691	1399942253	*Ca_kabuli_Chr07*	24190691	G/C	NA	INTERGENIC	NA
Kabuli_wild_24190700	1399942254	*Ca_kabuli_Chr07*	24190700	G/C	NA	INTERGENIC	NA
Kabuli_wild_24190706	1399942255	*Ca_kabuli_Chr07*	24190706	A/G	NA	INTERGENIC	NA
Kabuli_wild_24190722	1399942256	*Ca_kabuli_Chr07*	24190722	C/G	NA	INTERGENIC	NA
Kabuli_wild_24245096	1399942257	*Ca_kabuli_Chr07*	24245096	C/T	NA	INTERGENIC	NA
Kabuli_wild_24590545	1399942258	*Ca_kabuli_Chr07*	24590545	G/A	NA	INTERGENIC	NA
Kabuli_wild_24590587	1399942259	*Ca_kabuli_Chr07*	24590587	G/A	NA	INTERGENIC	NA
Kabuli_wild_24590598	1399942260	*Ca_kabuli_Chr07*	24590598	C/T	NA	INTERGENIC	NA
Kabuli_wild_24867394	1399942261	*Ca_kabuli_Chr07*	24867394	T/C	NA	INTERGENIC	NA
Kabuli_wild_24867409	1399942262	*Ca_kabuli_Chr07*	24867409	C/T	NA	INTERGENIC	NA
Kabuli_wild_24867420	1399942263	*Ca_kabuli_Chr07*	24867420	G/A	NA	INTERGENIC	NA
Kabuli_wild_24867435	1399942264	*Ca_kabuli_Chr07*	24867435	G/C	NA	INTERGENIC	NA
Kabuli_wild_24867436	1399942265	*Ca_kabuli_Chr07*	24867436	T/A	NA	INTERGENIC	NA
Kabuli_wild_24867442	1399942266	*Ca_kabuli_Chr07*	24867442	C/T	NA	INTERGENIC	NA
Kabuli_wild_24867451	1399942267	*Ca_kabuli_Chr07*	24867451	G/T	NA	INTERGENIC	NA
Kabuli_wild_24867458	1399942268	*Ca_kabuli_Chr07*	24867458	C/A	NA	INTERGENIC	NA
Kabuli_wild_24867491	1399942269	*Ca_kabuli_Chr07*	24867491	G/A	NA	INTERGENIC	NA
Kabuli_wild_24882334	1399942270	*Ca_kabuli_Chr07*	24882334	C/A	NA	INTERGENIC	NA
Kabuli_wild_25218341	1399942271	*Ca_kabuli_Chr07*	25218341	A/C	NA	INTERGENIC	NA
Kabuli_wild_25218387	1399942272	*Ca_kabuli_Chr07*	25218387	A/C	NA	INTERGENIC	NA
Kabuli_wild_25218407	1399942273	*Ca_kabuli_Chr07*	25218407	C/T	NA	INTERGENIC	NA
Kabuli_wild_25313174	1399942274	*Ca_kabuli_Chr07*	25313174	T/C	NA	INTERGENIC	NA
Kabuli_wild_25313195	1399942275	*Ca_kabuli_Chr07*	25313195	C/T	NA	INTERGENIC	NA

SNP IDs	NCBI dbSNP accession IDs (NCBI_SS#)	Chromosomes	Physical positions (bp)	SNPs	Gene accession IDs	Sequence components of genome	Putative functions
Kabuli_wild_25313199	1399942276	*Ca_kabuli_Chr07*	25313199	G/C	NA	INTERGENIC	NA
Kabuli_wild_25313215	1399942277	*Ca_kabuli_Chr07*	25313215	A/T	NA	INTERGENIC	NA
Kabuli_wild_25362272	1399942278	*Ca_kabuli_Chr07*	25362272	T/C	Gene_Ca_23606	UPSTREAM	RNA-binding,CRMdomain
Kabuli_wild_25428518	1399942279	*Ca_kabuli_Chr07*	25428518	T/C	NA	INTERGENIC	NA
Kabuli_wild_25428548	1399942280	*Ca_kabuli_Chr07*	25428548	G/T	NA	INTERGENIC	NA
Kabuli_wild_25428566	1399942281	*Ca_kabuli_Chr07*	25428566	T/C	NA	INTERGENIC	NA
Kabuli_wild_25428660	1399942282	*Ca_kabuli_Chr07*	25428660	G/A	NA	INTERGENIC	NA
Kabuli_wild_25485247	1399942283	*Ca_kabuli_Chr07*	25485247	A/C	NA	INTERGENIC	NA
Kabuli_wild_25569657	1399942284	*Ca_kabuli_Chr07*	25569657	T/C	Gene_Ca_23609	INTRON	Zinc finger,PHD-type
Kabuli_wild_25569665	1399942285	*Ca_kabuli_Chr07*	25569665	T/G	Gene_Ca_23609	INTRON	Zinc finger,PHD-type
Kabuli_wild_25774486	1399942286	*Ca_kabuli_Chr07*	25774486	T/C	NA	INTERGENIC	NA
Kabuli_wild_25777137	1399942287	*Ca_kabuli_Chr07*	25777137	T/G	NA	INTERGENIC	NA
Kabuli_wild_25777161	1399942288	*Ca_kabuli_Chr07*	25777161	T/A	NA	INTERGENIC	NA
Kabuli_wild_25777197	1399942289	*Ca_kabuli_Chr07*	25777197	C/T	NA	INTERGENIC	NA
Kabuli_wild_25777198	1399942290	*Ca_kabuli_Chr07*	25777198	T/C	NA	INTERGENIC	NA
Kabuli_wild_25830689	1399942291	*Ca_kabuli_Chr07*	25830689	C/T	Gene_Ca_23632	NON_SYNONYMOUS_CODING	NA
Kabuli_wild_25891658	1399942292	*Ca_kabuli_Chr07*	25891658	C/T	Gene_Ca_23633	STOP_GAINED	Peptidase C48, SUMO/Sentrin/Ubl1
Kabuli_wild_26066416	1399942293	*Ca_kabuli_Chr07*	26066416	T/C	NA	INTERGENIC	NA
Kabuli_wild_26070513	1399942294	*Ca_kabuli_Chr07*	26070513	G/T	NA	INTERGENIC	NA
Kabuli_wild_26070515	1399942295	*Ca_kabuli_Chr07*	26070515	A/G	NA	INTERGENIC	NA
Kabuli_wild_26094562	1399942296	*Ca_kabuli_Chr07*	26094562	G/T	NA	INTERGENIC	NA
Kabuli_wild_26094567	1399942297	*Ca_kabuli_Chr07*	26094567	G/A	NA	INTERGENIC	NA
Kabuli_wild_26137315	1399942298	*Ca_kabuli_Chr07*	26137315	T/C	Gene_Ca_23640	SYNONYMOUS_CODING	IQ motif,EF-handbinding site
Kabuli_wild_26144246	1399942299	*Ca_kabuli_Chr07*	26144246	A/C	Gene_Ca_23640	INTRON	IQ motif,EF-handbinding site
Kabuli_wild_26146779	1399942300	*Ca_kabuli_Chr07*	26146779	C/T	Gene_Ca_23640	INTRON	IQ motif,EF-handbinding site
Kabuli_wild_26226146	1399942301	*Ca_kabuli_Chr07*	26226146	G/C	NA	INTERGENIC	NA
Kabuli_wild_26256883	1399942302	*Ca_kabuli_Chr07*	26256883	T/C	Gene_Ca_23644	UPSTREAM	NA
Kabuli_wild_26332655	1399942303	*Ca_kabuli_Chr07*	26332655	A/T	NA	INTERGENIC	NA
Kabuli_wild_26692128	1399942304	*Ca_kabuli_Chr07*	26692128	A/C	NA	INTERGENIC	NA
Kabuli_wild_26692130	1399942305	*Ca_kabuli_Chr07*	26692130	C/T	NA	INTERGENIC	NA
Kabuli_wild_26692131	1399942306	*Ca_kabuli_Chr07*	26692131	T/G	NA	INTERGENIC	NA
Kabuli_wild_26692194	1399942307	*Ca_kabuli_Chr07*	26692194	C/A	Gene_Ca_16250	DOWNSTREAM	NA
Kabuli_wild_26692195	1399942308	*Ca_kabuli_Chr07*	26692195	G/T	Gene_Ca_16250	DOWNSTREAM	NA
Kabuli_wild_26694365	1399942309	*Ca_kabuli_Chr07*	26694365	G/A	NA	INTERGENIC	NA
Kabuli_wild_26694440	1399942310	*Ca_kabuli_Chr07*	26694440	C/A	NA	INTERGENIC	NA
Kabuli_wild_26694441	1399942311	*Ca_kabuli_Chr07*	26694441	G/A	NA	INTERGENIC	NA
Kabuli_wild_26697772	1399942312	*Ca_kabuli_Chr07*	26697772	C/A	NA	INTERGENIC	NA
Kabuli_wild_26697867	1399942313	*Ca_kabuli_Chr07*	26697867	A/G	NA	INTERGENIC	NA
Kabuli_wild_26701547	1399942314	*Ca_kabuli_Chr07*	26701547	C/T	NA	INTERGENIC	NA
Kabuli_wild_26897883	1399942315	*Ca_kabuli_Chr07*	26897883	C/T	NA	INTERGENIC	NA
Kabuli_wild_26897884	1399942316	*Ca_kabuli_Chr07*	26897884	G/A	NA	INTERGENIC	NA
Kabuli_wild_26897901	1399942317	*Ca_kabuli_Chr07*	26897901	G/A	NA	INTERGENIC	NA
Kabuli_wild_26897910	1399942318	*Ca_kabuli_Chr07*	26897910	A/G	NA	INTERGENIC	NA
Kabuli_wild_26897920	1399942319	*Ca_kabuli_Chr07*	26897920	A/G	NA	INTERGENIC	NA

SNP IDs	NCBI dbSNP accession IDs (NCBI_SS#)	Chromosomes	Physical positions (bp)	SNPs	Gene accession IDs	Sequence components of genome	Putative functions
Kabuli_wild_26897923	1399942320	*Ca_kabuli_Chr07*	26897923	G/A	NA	INTERGENIC	NA
Kabuli_wild_26897948	1399942321	*Ca_kabuli_Chr07*	26897948	A/T	NA	INTERGENIC	NA
Kabuli_wild_26897958	1399942322	*Ca_kabuli_Chr07*	26897958	G/T	NA	INTERGENIC	NA
Kabuli_wild_26940917	1399942323	*Ca_kabuli_Chr07*	26940917	G/A	Gene_Ca_16240	NON_SYNONYMOUS_CODING	Plastocyanin-like
Kabuli_wild_26940967	1399942324	*Ca_kabuli_Chr07*	26940967	A/G	Gene_Ca_16240	SYNONYMOUS_CODING	Plastocyanin-like
Kabuli_wild_26940979	1399942325	*Ca_kabuli_Chr07*	26940979	A/G	Gene_Ca_16240	SYNONYMOUS_CODING	Plastocyanin-like
Kabuli_wild_26940985	1399942326	*Ca_kabuli_Chr07*	26940985	T/G	Gene_Ca_16240	SYNONYMOUS_CODING	Plastocyanin-like
Kabuli_wild_27050259	1399942327	*Ca_kabuli_Chr07*	27050259	C/G	Gene_Ca_16234	NON_SYNONYMOUS_CODING	Dynamin,GTPasedomain
Kabuli_wild_27174078	1399942328	*Ca_kabuli_Chr07*	27174078	G/C	Gene_Ca_16229	NON_SYNONYMOUS_CODING	Lactate/malatedehydrogenase,N-terminal
Kabuli_wild_27176481	1399942329	*Ca_kabuli_Chr07*	27176481	C/A	Gene_Ca_16229	NON_SYNONYMOUS_CODING	Lactate/malatedehydrogenase,N-terminal
Kabuli_wild_27210848	1399942330	*Ca_kabuli_Chr07*	27210848	A/T	NA	INTERGENIC	NA
Kabuli_wild_27210853	1399942331	*Ca_kabuli_Chr07*	27210853	A/G	NA	INTERGENIC	NA
Kabuli_wild_27210858	1399942332	*Ca_kabuli_Chr07*	27210858	C/T	NA	INTERGENIC	NA
Kabuli_wild_27210871	1399942333	*Ca_kabuli_Chr07*	27210871	C/A	NA	INTERGENIC	NA
Kabuli_wild_27210881	1399942334	*Ca_kabuli_Chr07*	27210881	C/T	NA	INTERGENIC	NA
Kabuli_wild_27210894	1399942335	*Ca_kabuli_Chr07*	27210894	T/C	NA	INTERGENIC	NA
Kabuli_wild_27210918	1399942336	*Ca_kabuli_Chr07*	27210918	C/A	NA	INTERGENIC	NA
Kabuli_wild_27210919	1399942337	*Ca_kabuli_Chr07*	27210919	C/T	NA	INTERGENIC	NA
Kabuli_wild_27210935	1399942338	*Ca_kabuli_Chr07*	27210935	G/T	NA	INTERGENIC	NA
Kabuli_wild_27210940	1399942339	*Ca_kabuli_Chr07*	27210940	G/A	NA	INTERGENIC	NA
Kabuli_wild_27210988	1399942340	*Ca_kabuli_Chr07*	27210988	T/C	NA	INTERGENIC	NA
Kabuli_wild_27210989	1399942341	*Ca_kabuli_Chr07*	27210989	C/T	NA	INTERGENIC	NA
Kabuli_wild_27210992	1399942342	*Ca_kabuli_Chr07*	27210992	C/A	NA	INTERGENIC	NA
Kabuli_wild_27211004	1399942343	*Ca_kabuli_Chr07*	27211004	T/C	NA	INTERGENIC	NA
Kabuli_wild_27211036	1399942344	*Ca_kabuli_Chr07*	27211036	G/A	NA	INTERGENIC	NA
Kabuli_wild_27211051	1399942345	*Ca_kabuli_Chr07*	27211051	C/A	NA	INTERGENIC	NA
Kabuli_wild_27211057	1399942346	*Ca_kabuli_Chr07*	27211057	A/G	NA	INTERGENIC	NA
Kabuli_wild_27232405	1399942347	*Ca_kabuli_Chr07*	27232405	G/T	NA	INTERGENIC	NA
Kabuli_wild_27350825	1399942348	*Ca_kabuli_Chr07*	27350825	T/G	Gene_Ca_16224	SYNONYMOUS_CODING	NA
Kabuli_wild_27350830	1399942349	*Ca_kabuli_Chr07*	27350830	T/C	Gene_Ca_16224	NON_SYNONYMOUS_CODING	NA
Kabuli_wild_27350854	1399942350	*Ca_kabuli_Chr07*	27350854	T/A	Gene_Ca_16224	NON_SYNONYMOUS_CODING	NA
Kabuli_wild_27350880	1399942351	*Ca_kabuli_Chr07*	27350880	G/A	Gene_Ca_16224	SYNONYMOUS_CODING	NA
Kabuli_wild_27633960	1399942352	*Ca_kabuli_Chr07*	27633960	C/A	NA	INTERGENIC	NA
Kabuli_wild_27648163	1399942353	*Ca_kabuli_Chr07*	27648163	T/G	Gene_Ca_16215	UPSTREAM	NA
Kabuli_wild_27648165	1399942354	*Ca_kabuli_Chr07*	27648165	T/C	Gene_Ca_16215	UPSTREAM	NA
Kabuli_wild_27648216	1399942355	*Ca_kabuli_Chr07*	27648216	G/T	Gene_Ca_16215	UPSTREAM	NA
Kabuli_wild_27648224	1399942356	*Ca_kabuli_Chr07*	27648224	C/G	Gene_Ca_16215	UPSTREAM	NA
Kabuli_wild_27711878	1399942357	*Ca_kabuli_Chr07*	27711878	G/A	NA	INTERGENIC	NA
Kabuli_wild_27711879	1399942358	*Ca_kabuli_Chr07*	27711879	C/T	NA	INTERGENIC	NA
Kabuli_wild_27735234	1399942359	*Ca_kabuli_Chr07*	27735234	A/G	Gene_Ca_16212	INTRON	Cyclin,C-terminal
Kabuli_wild_27743487	1399942360	*Ca_kabuli_Chr07*	27743487	T/A	Gene_Ca_16212	INTRON	Cyclin,C-terminal
Kabuli_wild_27899782	1399942361	*Ca_kabuli_Chr07*	27899782	T/C	NA	INTERGENIC	NA
Kabuli_wild_27971176	1399942362	*Ca_kabuli_Chr07*	27971176	T/C	NA	INTERGENIC	NA
Kabuli_wild_27971192	1399942363	*Ca_kabuli_Chr07*	27971192	T/C	NA	INTERGENIC	NA

SNP IDs	NCBI dbSNP accession IDs (NCBI_SS#)	Chromosomes	Physical positions (bp)	SNPs	Gene accession IDs	Sequence components of genome	Putative functions
Kabuli_wild_27971193	1399942364	*Ca_kabuli_Chr07*	27971193	T/C	NA	INTERGENIC	NA
Kabuli_wild_27971195	1399942365	*Ca_kabuli_Chr07*	27971195	A/C	NA	INTERGENIC	NA
Kabuli_wild_28414473	1399942366	*Ca_kabuli_Chr07*	28414473	G/A	Gene_Ca_18928	DOWNSTREAM	Acidphosphatase(ClassB)
Kabuli_wild_28503199	1399942367	*Ca_kabuli_Chr07*	28503199	G/A	Gene_Ca_18937	UPSTREAM	NA
Kabuli_wild_28520368	1399942368	*Ca_kabuli_Chr07*	28520368	A/C	NA	INTERGENIC	NA
Kabuli_wild_28520385	1399942369	*Ca_kabuli_Chr07*	28520385	A/C	NA	INTERGENIC	NA
Kabuli_wild_28567015	1399942370	*Ca_kabuli_Chr07*	28567015	A/T	NA	INTERGENIC	NA
Kabuli_wild_28620851	1399942371	*Ca_kabuli_Chr07*	28620851	C/T	Gene_Ca_18949	INTRON	Heat shock protein DnaJ, cysteine-richdomain
Kabuli_wild_28931212	1399942372	*Ca_kabuli_Chr07*	28931212	A/C	NA	INTERGENIC	NA
Kabuli_wild_28931223	1399942373	*Ca_kabuli_Chr07*	28931223	C/A	NA	INTERGENIC	NA
Kabuli_wild_29148471	1399942374	*Ca_kabuli_Chr07*	29148471	C/T	Gene_Ca_11791	NON_SYNONYMOUS_CODING	NA
Kabuli_wild_29150927	1399942375	*Ca_kabuli_Chr07*	29150927	A/C	Gene_Ca_11791	INTRON	NA
Kabuli_wild_29153994	1399942376	*Ca_kabuli_Chr07*	29153994	T/C	Gene_Ca_11791	SYNONYMOUS_CODING	NA
Kabuli_wild_29157286	1399942377	*Ca_kabuli_Chr07*	29157286	A/G	Gene_Ca_11791	SYNONYMOUS_CODING	NA
Kabuli_wild_29168980	1399942378	*Ca_kabuli_Chr07*	29168980	C/T	NA	INTERGENIC	NA
Kabuli_wild_29168982	1399942379	*Ca_kabuli_Chr07*	29168982	A/G	NA	INTERGENIC	NA
Kabuli_wild_29190659	1399942380	*Ca_kabuli_Chr07*	29190659	T/G	Gene_Ca_11786	NON_SYNONYMOUS_CODING	Glutamyl/glutaminyl-tRNAsynthetase,classIc
Kabuli_wild_29223723	1399942381	*Ca_kabuli_Chr07*	29223723	T/C	Gene_Ca_11784	UPSTREAM	Protein of unknown function DUF3550/UPF0682
Kabuli_wild_29225348	1399942382	*Ca_kabuli_Chr07*	29225348	G/C	NA	INTERGENIC	NA
Kabuli_wild_29225351	1399942383	*Ca_kabuli_Chr07*	29225351	T/G	NA	INTERGENIC	NA
Kabuli_wild_29225385	1399942384	*Ca_kabuli_Chr07*	29225385	T/G	NA	INTERGENIC	NA
Kabuli_wild_29229965	1399942385	*Ca_kabuli_Chr07*	29229965	A/G	NA	INTERGENIC	NA
Kabuli_wild_29313051	1399942386	*Ca_kabuli_Chr07*	29313051	T/C	NA	INTERGENIC	NA
Kabuli_wild_29313058	1399942387	*Ca_kabuli_Chr07*	29313058	T/C	NA	INTERGENIC	NA
Kabuli_wild_29313059	1399942388	*Ca_kabuli_Chr07*	29313059	G/A	NA	INTERGENIC	NA
Kabuli_wild_29313067	1399942389	*Ca_kabuli_Chr07*	29313067	G/T	NA	INTERGENIC	NA
Kabuli_wild_29313080	1399942390	*Ca_kabuli_Chr07*	29313080	T/C	NA	INTERGENIC	NA
Kabuli_wild_29313257	1399942391	*Ca_kabuli_Chr07*	29313257	G/A	NA	INTERGENIC	NA
Kabuli_wild_29435190	1399942392	*Ca_kabuli_Chr07*	29435190	T/C	NA	INTERGENIC	NA
Kabuli_wild_29449916	1399942393	*Ca_kabuli_Chr07*	29449916	T/G	NA	INTERGENIC	NA
Kabuli_wild_29450027	1399942394	*Ca_kabuli_Chr07*	29450027	A/T	NA	INTERGENIC	NA
Kabuli_wild_29482556	1399942395	*Ca_kabuli_Chr07*	29482556	G/A	Gene_Ca_11768	UPSTREAM	Protein kinase,catalytic domain
Kabuli_wild_29484304	1399942396	*Ca_kabuli_Chr07*	29484304	G/A	Gene_Ca_11768	UPSTREAM	Protein kinase,catalytic domain
Kabuli_wild_29548425	1399942397	*Ca_kabuli_Chr07*	29548425	A/G	Gene_Ca_11767	INTRON	Profilin/allergen
Kabuli_wild_29607943	1399942398	*Ca_kabuli_Chr07*	29607943	C/T	NA	INTERGENIC	NA
Kabuli_wild_29613699	1399942399	*Ca_kabuli_Chr07*	29613699	C/T	Gene_Ca_11764	SYNONYMOUS_CODING	F-boxdomain,cyclin-like
Kabuli_wild_29625884	1399942400	*Ca_kabuli_Chr07*	29625884	T/C	Gene_Ca_11763	UPSTREAM	NA
Kabuli_wild_29704336	1399942401	*Ca_kabuli_Chr07*	29704336	G/A	Gene_Ca_11752	NON_SYNONYMOUS_CODING	NA
Kabuli_wild_29705457	1399942402	*Ca_kabuli_Chr07*	29705457	G/T	Gene_Ca_11752	SYNONYMOUS_CODING	NA
Kabuli_wild_29705499	1399942403	*Ca_kabuli_Chr07*	29705499	T/C	Gene_Ca_11752	NON_SYNONYMOUS_CODING	NA
Kabuli_wild_29705531	1399942404	*Ca_kabuli_Chr07*	29705531	T/G	Gene_Ca_11752	NON_SYNONYMOUS_CODING	NA
Kabuli_wild_29796564	1399942405	*Ca_kabuli_Chr07*	29796564	G/T	Gene_Ca_11746	NON_SYNONYMOUS_CODING	Spc97/Spc98
Kabuli_wild_29801148	1399942406	*Ca_kabuli_Chr07*	29801148	A/G	Gene_Ca_11746	NON_SYNONYMOUS_CODING	Spc97/Spc98
Kabuli_wild_29801154	1399942407	*Ca_kabuli_Chr07*	29801154	T/C	Gene_Ca_11746	NON_SYNONYMOUS_CODING	Spc97/Spc98

SNP IDs	NCBI dbSNP accession IDs (NCBI_SS#)	Chromosomes	Physical positions (bp)	SNPs	Gene accession IDs	Sequence components of genome	Putative functions
Kabuli_wild_29837035	1399942408	*Ca_kabuli_Chr07*	29837035	G/A	NA	INTERGENIC	NA
Kabuli_wild_29838073	1399942409	*Ca_kabuli_Chr07*	29838073	C/T	NA	INTERGENIC	NA
Kabuli_wild_29848248	1399942410	*Ca_kabuli_Chr07*	29848248	T/C	Gene_Ca_11739	INTRON	Lipase,class3
Kabuli_wild_29848433	1399942411	*Ca_kabuli_Chr07*	29848433	C/T	Gene_Ca_11739	SYNONYMOUS_CODING	Lipase,class3
Kabuli_wild_29849509	1399942412	*Ca_kabuli_Chr07*	29849509	C/T	Gene_Ca_11739	NON_SYNONYMOUS_CODING	Lipase,class3
Kabuli_wild_29849544	1399942413	*Ca_kabuli_Chr07*	29849544	A/G	Gene_Ca_11739	SYNONYMOUS_CODING	Lipase,class3
Kabuli_wild_29850432	1399942414	*Ca_kabuli_Chr07*	29850432	T/A	Gene_Ca_11739	INTRON	Lipase,class3
Kabuli_wild_29850452	1399942415	*Ca_kabuli_Chr07*	29850452	A/C	Gene_Ca_11739	INTRON	Lipase,class3
Kabuli_wild_29850460	1399942416	*Ca_kabuli_Chr07*	29850460	C/T	Gene_Ca_11739	INTRON	Lipase,class3
Kabuli_wild_29850470	1399942417	*Ca_kabuli_Chr07*	29850470	T/C	Gene_Ca_11739	INTRON	Lipase,class3
Kabuli_wild_29884718	1399942418	*Ca_kabuli_Chr07*	29884718	C/A	Gene_Ca_11736	SYNONYMOUS_CODING	TetratricopeptideTPR-1
Kabuli_wild_29884719	1399942419	*Ca_kabuli_Chr07*	29884719	G/T	Gene_Ca_11736	NON_SYNONYMOUS_CODING	TetratricopeptideTPR-1
Kabuli_wild_29925008	1399942420	*Ca_kabuli_Chr07*	29925008	G/A	NA	INTERGENIC	NA
Kabuli_wild_30004767	1399942421	*Ca_kabuli_Chr07*	30004767	T/C	NA	INTERGENIC	NA
Kabuli_wild_30004934	1399942422	*Ca_kabuli_Chr07*	30004934	A/C	NA	INTERGENIC	NA
Kabuli_wild_30026017	1399942423	*Ca_kabuli_Chr07*	30026017	C/T	Gene_Ca_11727	INTRON	Glycosyltransferase,family2
Kabuli_wild_30026039	1399942424	*Ca_kabuli_Chr07*	30026039	T/G	Gene_Ca_11727	INTRON	Glycosyltransferase,family2
Kabuli_wild_30029793	1399942425	*Ca_kabuli_Chr07*	30029793	A/C	NA	INTERGENIC	NA
Kabuli_wild_30029820	1399942426	*Ca_kabuli_Chr07*	30029820	T/C	NA	INTERGENIC	NA
Kabuli_wild_30038061	1399942427	*Ca_kabuli_Chr07*	30038061	A/C	Gene_Ca_11725	UPSTREAM	PlantECmetallothionein-likeprotein,family15
Kabuli_wild_30060299	1399942428	*Ca_kabuli_Chr07*	30060299	C/A	Gene_Ca_11722	SYNONYMOUS_CODING	ATPase,P-type,K/Mg/Cd/Cu/Zn/Na/Ca/Na/H-transporter
Kabuli_wild_30085847	1399942429	*Ca_kabuli_Chr07*	30085847	C/T	Gene_Ca_11718	NON_SYNONYMOUS_CODING	Domain of unknown function DUF828
Kabuli_wild_30085883	1399942430	*Ca_kabuli_Chr07*	30085883	T/G	Gene_Ca_11718	NON_SYNONYMOUS_CODING	Domain of unknown function DUF828
Kabuli_wild_30085906	1399942431	*Ca_kabuli_Chr07*	30085906	C/T	NA	INTERGENIC	NA
Kabuli_wild_30085910	1399942432	*Ca_kabuli_Chr07*	30085910	T/A	NA	INTERGENIC	NA
Kabuli_wild_30099003	1399942433	*Ca_kabuli_Chr07*	30099003	C/T	NA	INTERGENIC	NA
Kabuli_wild_30107340	1399942434	*Ca_kabuli_Chr07*	30107340	G/A	NA	INTERGENIC	NA
Kabuli_wild_30403618	1399942435	*Ca_kabuli_Chr07*	30403618	G/A	Gene_Ca_11689	DOWNSTREAM	Integrase,catalyticcore
Kabuli_wild_30403619	1399942436	*Ca_kabuli_Chr07*	30403619	C/A	Gene_Ca_11689	DOWNSTREAM	Integrase,catalyticcore
Kabuli_wild_30403633	1399942437	*Ca_kabuli_Chr07*	30403633	G/A	Gene_Ca_11689	DOWNSTREAM	Integrase,catalyticcore
Kabuli_wild_30691280	1399942438	*Ca_kabuli_Chr07*	30691280	A/C	Gene_Ca_23327	NON_SYNONYMOUS_CODING	NA
Kabuli_wild_30691318	1399942439	*Ca_kabuli_Chr07*	30691318	A/C	Gene_Ca_23327	NON_SYNONYMOUS_CODING	NA
Kabuli_wild_30691324	1399942440	*Ca_kabuli_Chr07*	30691324	A/C	Gene_Ca_23327	NON_SYNONYMOUS_CODING	NA
Kabuli_wild_30768315	1399942441	*Ca_kabuli_Chr07*	30768315	G/A	NA	INTERGENIC	NA
Kabuli_wild_30809971	1399942442	*Ca_kabuli_Chr07*	30809971	T/A	Gene_Ca_23321	NON_SYNONYMOUS_CODING	PeptidaseM16,N-terminal
Kabuli_wild_30850950	1399942443	*Ca_kabuli_Chr07*	30850950	A/C	NA	INTERGENIC	NA
Kabuli_wild_31190059	1399942444	*Ca_kabuli_Chr07*	31190059	G/A	Gene_Ca_10139	SYNONYMOUS_CODING	RNApolymeraseII-associated,Paf1
Kabuli_wild_31193786	1399942445	*Ca_kabuli_Chr07*	31193786	T/G	Gene_Ca_10139	SYNONYMOUS_CODING	RNApolymeraseII-associated,Paf1
Kabuli_wild_31298240	1399942446	*Ca_kabuli_Chr07*	31298240	T/C	Gene_Ca_10130	INTRON	PeptidaseM16,zinc-bindingsite
Kabuli_wild_31314421	1399942447	*Ca_kabuli_Chr07*	31314421	T/A	NA	INTERGENIC	NA
Kabuli_wild_31324279	1399942448	*Ca_kabuli_Chr07*	31324279	A/G	NA	INTERGENIC	NA
Kabuli_wild_31342254	1399942449	*Ca_kabuli_Chr07*	31342254	T/C	Gene_Ca_10124	UPSTREAM	Pentatrico peptide repeat
Kabuli_wild_31395704	1399942450	*Ca_kabuli_Chr07*	31395704	C/T	Gene_Ca_10119	DOWNSTREAM	Peptidase C48, SUMO/Sentrin/Ubl1
Kabuli_wild_31395721	1399942451	*Ca_kabuli_Chr07*	31395721	C/T	NA	INTERGENIC	NA

SNP IDs	NCBI dbSNP accession IDs (NCBI_SS#)	Chromosomes	Physical positions (bp)	SNPs	Gene accession IDs	Sequence components of genome	Putative functions
Kabuli_wild_31395736	1399942452	*Ca_kabuli_Chr07*	31395736	G/A	NA	INTERGENIC	NA
Kabuli_wild_31395740	1399942453	*Ca_kabuli_Chr07*	31395740	C/T	NA	INTERGENIC	NA
Kabuli_wild_31395773	1399942454	*Ca_kabuli_Chr07*	31395773	G/A	NA	INTERGENIC	NA
Kabuli_wild_31395788	1399942455	*Ca_kabuli_Chr07*	31395788	C/T	NA	INTERGENIC	NA
Kabuli_wild_31401807	1399942456	*Ca_kabuli_Chr07*	31401807	T/C	NA	INTERGENIC	NA
Kabuli_wild_31442425	1399942457	*Ca_kabuli_Chr07*	31442425	A/C	Gene_Ca_10114	INTRON	PWWP
Kabuli_wild_31461273	1399942458	*Ca_kabuli_Chr07*	31461273	T/C	Gene_Ca_10112	INTRON	Glutamate/phenylalanine/leucine/valinedehydrogenase,C-terminal
Kabuli_wild_31487441	1399942459	*Ca_kabuli_Chr07*	31487441	C/T	Gene_Ca_10109	SYNONYMOUS_CODING	Occludin/RNApolymeraseIIelongationfactor,ELLdomain
Kabuli_wild_31612697	1399942460	*Ca_kabuli_Chr07*	31612697	T/A	Gene_Ca_10100	NON_SYNONYMOUS_CODING	ABC transporter,trans membrane domain
Kabuli_wild_31639701	1399942461	*Ca_kabuli_Chr07*	31639701	C/T	Gene_Ca_10099	INTRON	Domain of unknown function DUF250
Kabuli_wild_31830238	1399942462	*Ca_kabuli_Chr07*	31830238	A/G	NA	INTERGENIC	NA
Kabuli_wild_31851096	1399942463	*Ca_kabuli_Chr07*	31851096	T/A	Gene_Ca_10079	NON_SYNONYMOUS_CODING	Prenyltransferase/squaleneoxidase
Kabuli_wild_31873079	1399942464	*Ca_kabuli_Chr07*	31873079	G/T	Gene_Ca_10078	DOWNSTREAM	Chaperonin Cpn60
Kabuli_wild_31996345	1399942465	*Ca_kabuli_Chr07*	31996345	A/C	NA	INTERGENIC	NA
Kabuli_wild_32017221	1399942466	*Ca_kabuli_Chr07*	32017221	C/T	NA	INTERGENIC	NA
Kabuli_wild_32017228	1399942467	*Ca_kabuli_Chr07*	32017228	G/A	NA	INTERGENIC	NA
Kabuli_wild_32017238	1399942468	*Ca_kabuli_Chr07*	32017238	C/T	NA	INTERGENIC	NA
Kabuli_wild_32017252	1399942469	*Ca_kabuli_Chr07*	32017252	G/A	NA	INTERGENIC	NA
Kabuli_wild_32017286	1399942470	*Ca_kabuli_Chr07*	32017286	T/G	NA	INTERGENIC	NA
Kabuli_wild_32017288	1399942471	*Ca_kabuli_Chr07*	32017288	G/T	NA	INTERGENIC	NA
Kabuli_wild_32105467	1399942472	*Ca_kabuli_Chr07*	32105467	G/A	NA	INTERGENIC	NA
Kabuli_wild_32127288	1399942473	*Ca_kabuli_Chr07*	32127288	T/A	NA	INTERGENIC	NA
Kabuli_wild_32283585	1399942474	*Ca_kabuli_Chr07*	32283585	T/G	Gene_Ca_10045	INTRON	vonWille brand factor,typeA
Kabuli_wild_32288840	1399942475	*Ca_kabuli_Chr07*	32288840	G/C	Gene_Ca_10045	NON_SYNONYMOUS_CODING	vonWille brand factor,typeA
Kabuli_wild_32309372	1399942476	*Ca_kabuli_Chr07*	32309372	T/G	Gene_Ca_10045	INTRON	vonWille brand factor,typeA
Kabuli_wild_32309376	1399942477	*Ca_kabuli_Chr07*	32309376	C/G	Gene_Ca_10045	INTRON	vonWille brand factor,typeA
Kabuli_wild_32309377	1399942478	*Ca_kabuli_Chr07*	32309377	C/T	Gene_Ca_10045	INTRON	vonWille brand factor,typeA
Kabuli_wild_32309397	1399942479	*Ca_kabuli_Chr07*	32309397	A/T	Gene_Ca_10045	INTRON	vonWille brand factor,typeA
Kabuli_wild_32309411	1399942480	*Ca_kabuli_Chr07*	32309411	G/T	Gene_Ca_10045	INTRON	vonWille brand factor,typeA
Kabuli_wild_32309415	1399942481	*Ca_kabuli_Chr07*	32309415	C/T	Gene_Ca_10045	INTRON	vonWille brand factor,typeA
Kabuli_wild_32309429	1399942482	*Ca_kabuli_Chr07*	32309429	C/T	Gene_Ca_10045	INTRON	vonWille brand factor,typeA
Kabuli_wild_32309908	1399942483	*Ca_kabuli_Chr07*	32309908	T/G	Gene_Ca_10045	NON_SYNONYMOUS_CODING	vonWille brand factor,typeA
Kabuli_wild_32374244	1399942484	*Ca_kabuli_Chr07*	32374244	A/T	NA	INTERGENIC	NA
Kabuli_wild_32399759	1399942485	*Ca_kabuli_Chr07*	32399759	C/T	Gene_Ca_10038	SYNONYMOUS_CODING	LsmADdomain
Kabuli_wild_32404932	1399942486	*Ca_kabuli_Chr07*	32404932	T/C	NA	INTERGENIC	NA
Kabuli_wild_32409413	1399942487	*Ca_kabuli_Chr07*	32409413	A/G	NA	INTERGENIC	NA
Kabuli_wild_32409453	1399942488	*Ca_kabuli_Chr07*	32409453	G/T	NA	INTERGENIC	NA
Kabuli_wild_32451377	1399942489	*Ca_kabuli_Chr07*	32451377	A/C	Gene_Ca_10034	INTRON	Protein of unknown function DUF1296
Kabuli_wild_32492991	1399942490	*Ca_kabuli_Chr07*	32492991	C/A	NA	INTERGENIC	NA
Kabuli_wild_32522201	1399942491	*Ca_kabuli_Chr07*	32522201	A/G	Gene_Ca_10028	SYNONYMOUS_CODING	SANT domain,DNA binding
Kabuli_wild_32522203	1399942492	*Ca_kabuli_Chr07*	32522203	A/G	Gene_Ca_10028	NON_SYNONYMOUS_CODING	SANT domain,DNA binding
Kabuli_wild_32522207	1399942493	*Ca_kabuli_Chr07*	32522207	T/G	Gene_Ca_10028	SYNONYMOUS_CODING	SANT domain,DNA binding
Kabuli_wild_32522213	1399942494	*Ca_kabuli_Chr07*	32522213	A/G	Gene_Ca_10028	SYNONYMOUS_CODING	SANT domain,DNA binding
Kabuli_wild_32522230	1399942495	*Ca_kabuli_Chr07*	32522230	T/G	Gene_Ca_10028	NON_SYNONYMOUS_CODING	SANT domain,DNA binding

SNP IDs	NCBI dbSNP accession IDs (NCBI_SS#)	Chromosomes	Physical positions (bp)	SNPs	Gene accession IDs	Sequence components of genome	Putative functions
Kabuli_wild_32528503	1399942496	*Ca_kabuli_Chr07*	32528503	T/C	NA	INTERGENIC	NA
Kabuli_wild_32528545	1399942497	*Ca_kabuli_Chr07*	32528545	A/G	NA	INTERGENIC	NA
Kabuli_wild_32528578	1399942498	*Ca_kabuli_Chr07*	32528578	G/A	NA	INTERGENIC	NA
Kabuli_wild_32557548	1399942499	*Ca_kabuli_Chr07*	32557548	A/G	NA	INTERGENIC	NA
Kabuli_wild_32557599	1399942500	*Ca_kabuli_Chr07*	32557599	G/A	NA	INTERGENIC	NA
Kabuli_wild_32568197	1399942501	*Ca_kabuli_Chr07*	32568197	A/C	NA	INTERGENIC	NA
Kabuli_wild_32568209	1399942502	*Ca_kabuli_Chr07*	32568209	T/G	NA	INTERGENIC	NA
Kabuli_wild_32568244	1399942503	*Ca_kabuli_Chr07*	32568244	T/G	NA	INTERGENIC	NA
Kabuli_wild_32568247	1399942504	*Ca_kabuli_Chr07*	32568247	T/A	NA	INTERGENIC	NA
Kabuli_wild_32669570	1399942505	*Ca_kabuli_Chr07*	32669570	A/G	NA	INTERGENIC	NA
Kabuli_wild_32674163	1399942506	*Ca_kabuli_Chr07*	32674163	G/C	NA	INTERGENIC	NA
Kabuli_wild_32754633	1399942507	*Ca_kabuli_Chr07*	32754633	T/G	Gene_Ca_10017	INTRON	NA
Kabuli_wild_32755614	1399942508	*Ca_kabuli_Chr07*	32755614	A/C	Gene_Ca_10017	SYNONYMOUS_CODING	NA
Kabuli_wild_32755615	1399942509	*Ca_kabuli_Chr07*	32755615	G/T	Gene_Ca_10017	NON_SYNONYMOUS_CODING	NA
Kabuli_wild_32755662	1399942510	*Ca_kabuli_Chr07*	32755662	G/T	Gene_Ca_10017	NON_SYNONYMOUS_CODING	NA
Kabuli_wild_32782823	1399942511	*Ca_kabuli_Chr07*	32782823	A/G	Gene_Ca_10015	SYNONYMOUS_CODING	TranscriptioninitiationfactorTFIID
Kabuli_wild_32784877	1399942512	*Ca_kabuli_Chr07*	32784877	C/A	Gene_Ca_10015	NON_SYNONYMOUS_CODING	TranscriptioninitiationfactorTFIID
Kabuli_wild_32784903	1399942513	*Ca_kabuli_Chr07*	32784903	T/G	Gene_Ca_10015	SYNONYMOUS_CODING	TranscriptioninitiationfactorTFIID
Kabuli_wild_32785429	1399942514	*Ca_kabuli_Chr07*	32785429	A/G	Gene_Ca_10015	INTRON	TranscriptioninitiationfactorTFIID
Kabuli_wild_32785432	1399942515	*Ca_kabuli_Chr07*	32785432	T/C	Gene_Ca_10015	INTRON	TranscriptioninitiationfactorTFIID
Kabuli_wild_32799037	1399942516	*Ca_kabuli_Chr07*	32799037	A/G	NA	INTERGENIC	NA
Kabuli_wild_32807457	1399942517	*Ca_kabuli_Chr07*	32807457	T/A	NA	INTERGENIC	NA
Kabuli_wild_32807499	1399942518	*Ca_kabuli_Chr07*	32807499	A/G	NA	INTERGENIC	NA
Kabuli_wild_32874840	1399942519	*Ca_kabuli_Chr07*	32874840	C/T	NA	INTERGENIC	NA
Kabuli_wild_32874847	1399942520	*Ca_kabuli_Chr07*	32874847	C/T	NA	INTERGENIC	NA
Kabuli_wild_32875799	1399942521	*Ca_kabuli_Chr07*	32875799	T/A	Gene_Ca_10011	SYNONYMOUS_CODING	Germin
Kabuli_wild_32932232	1399942522	*Ca_kabuli_Chr07*	32932232	T/G	NA	INTERGENIC	NA
Kabuli_wild_32932285	1399942523	*Ca_kabuli_Chr07*	32932285	T/A	NA	INTERGENIC	NA
Kabuli_wild_32942072	1399942524	*Ca_kabuli_Chr07*	32942072	G/A	NA	INTERGENIC	NA
Kabuli_wild_32942199	1399942525	*Ca_kabuli_Chr07*	32942199	G/A	NA	INTERGENIC	NA
Kabuli_wild_32942951	1399942526	*Ca_kabuli_Chr07*	32942951	T/G	Gene_Ca_10003	DOWNSTREAM	Zinc finger, RING-type
Kabuli_wild_32948171	1399942527	*Ca_kabuli_Chr07*	32948171	A/C	NA	INTERGENIC	NA
Kabuli_wild_32948203	1399942528	*Ca_kabuli_Chr07*	32948203	G/A	NA	INTERGENIC	NA
Kabuli_wild_32948246	1399942529	*Ca_kabuli_Chr07*	32948246	T/G	NA	INTERGENIC	NA
Kabuli_wild_32989796	1399942530	*Ca_kabuli_Chr07*	32989796	T/A	Gene_Ca_10000	SYNONYMOUS_CODING	Protein kinase,catalytic domain
Kabuli_wild_32989838	1399942531	*Ca_kabuli_Chr07*	32989838	C/T	Gene_Ca_10000	SYNONYMOUS_CODING	Protein kinase,catalytic domain
Kabuli_wild_32989902	1399942532	*Ca_kabuli_Chr07*	32989902	C/A	Gene_Ca_10000	INTRON	Protein kinase,catalytic domain
Kabuli_wild_32989903	1399942533	*Ca_kabuli_Chr07*	32989903	A/G	Gene_Ca_10000	INTRON	Protein kinase,catalytic domain
Kabuli_wild_33026091	1399942534	*Ca_kabuli_Chr07*	33026091	T/G	Gene_Ca_09997	INTRON	Lipoxygenase
Kabuli_wild_33026150	1399942535	*Ca_kabuli_Chr07*	33026150	T/G	Gene_Ca_09997	INTRON	Lipoxygenase
Kabuli_wild_33065611	1399942536	*Ca_kabuli_Chr07*	33065611	G/T	NA	INTERGENIC	NA
Kabuli_wild_33089682	1399942537	*Ca_kabuli_Chr07*	33089682	A/G	Gene_Ca_16140	SYNONYMOUS_CODING	NA
Kabuli_wild_33099276	1399942538	*Ca_kabuli_Chr07*	33099276	C/A	NA	INTERGENIC	NA
Kabuli_wild_33099299	1399942539	*Ca_kabuli_Chr07*	33099299	T/C	NA	INTERGENIC	NA

SNP IDs	NCBI dbSNP accession IDs (NCBI_SS#)	Chromosomes	Physical positions (bp)	SNPs	Gene accession IDs	Sequence components of genome	Putative functions
Kabuli_wild_33099304	1399942540	*Ca_kabuli_Chr07*	33099304	C/T	NA	INTERGENIC	NA
Kabuli_wild_33099313	1399942541	*Ca_kabuli_Chr07*	33099313	A/G	NA	INTERGENIC	NA
Kabuli_wild_33115488	1399942542	*Ca_kabuli_Chr07*	33115488	C/G	Gene_Ca_16142	SYNONYMOUS_CODING	MyosinII heavychain-like
Kabuli_wild_33121640	1399942543	*Ca_kabuli_Chr07*	33121640	C/A	NA	INTERGENIC	NA
Kabuli_wild_33121674	1399942544	*Ca_kabuli_Chr07*	33121674	G/C	NA	INTERGENIC	NA
Kabuli_wild_33122473	1399942545	*Ca_kabuli_Chr07*	33122473	A/C	NA	INTERGENIC	NA
Kabuli_wild_33139304	1399942546	*Ca_kabuli_Chr07*	33139304	A/T	NA	INTERGENIC	NA
Kabuli_wild_33182932	1399942547	*Ca_kabuli_Chr07*	33182932	G/A	Gene_Ca_16149	SYNONYMOUS_CODING	Lipase,class3
Kabuli_wild_33198651	1399942548	*Ca_kabuli_Chr07*	33198651	T/C	NA	INTERGENIC	NA
Kabuli_wild_33251859	1399942549	*Ca_kabuli_Chr07*	33251859	A/G	NA	INTERGENIC	NA
Kabuli_wild_33267485	1399942550	*Ca_kabuli_Chr07*	33267485	T/C	NA	INTERGENIC	NA
Kabuli_wild_33267496	1399942551	*Ca_kabuli_Chr07*	33267496	T/A	NA	INTERGENIC	NA
Kabuli_wild_33333090	1399942552	*Ca_kabuli_Chr07*	33333090	C/T	Gene_Ca_16158	INTRON	RNA recognition motif domain
Kabuli_wild_33335978	1399942553	*Ca_kabuli_Chr07*	33335978	C/A	Gene_Ca_16158	NON_SYNONYMOUS_CODING	RNA recognition motif domain
Kabuli_wild_33358969	1399942554	*Ca_kabuli_Chr07*	33358969	T/C	Gene_Ca_16161	INTRON	Nuclearporeprotein84/107
Kabuli_wild_33364329	1399942555	*Ca_kabuli_Chr07*	33364329	C/T	NA	INTERGENIC	NA
Kabuli_wild_33364354	1399942556	*Ca_kabuli_Chr07*	33364354	G/A	NA	INTERGENIC	NA
Kabuli_wild_33378427	1399942557	*Ca_kabuli_Chr07*	33378427	C/T	Gene_Ca_16163	INTRON	Spt20family
Kabuli_wild_33411577	1399942558	*Ca_kabuli_Chr07*	33411577	G/C	Gene_Ca_16164	INTRON	Zinc finger,CCCH-type
Kabuli_wild_33411746	1399942559	*Ca_kabuli_Chr07*	33411746	C/A	Gene_Ca_16164	INTRON	Zinc finger,CCCH-type
Kabuli_wild_33460918	1399942560	*Ca_kabuli_Chr07*	33460918	G/A	Gene_Ca_16167	SYNONYMOUS_CODING	ABC transporter,trans membrane domain
Kabuli_wild_33461145	1399942561	*Ca_kabuli_Chr07*	33461145	A/T	Gene_Ca_16167	NON_SYNONYMOUS_CODING	ABC transporter,trans membrane domain
Kabuli_wild_33461168	1399942562	*Ca_kabuli_Chr07*	33461168	A/C	Gene_Ca_16167	UPSTREAM	ABC transporter,trans membrane domain
Kabuli_wild_33463300	1399942563	*Ca_kabuli_Chr07*	33463300	C/G	NA	INTERGENIC	NA
Kabuli_wild_33465781	1399942564	*Ca_kabuli_Chr07*	33465781	G/T	NA	INTERGENIC	NA
Kabuli_wild_33465800	1399942565	*Ca_kabuli_Chr07*	33465800	T/A	NA	INTERGENIC	NA
Kabuli_wild_33465802	1399942566	*Ca_kabuli_Chr07*	33465802	C/A	NA	INTERGENIC	NA
Kabuli_wild_33465805	1399942567	*Ca_kabuli_Chr07*	33465805	T/C	NA	INTERGENIC	NA
Kabuli_wild_33465818	1399942568	*Ca_kabuli_Chr07*	33465818	G/A	NA	INTERGENIC	NA
Kabuli_wild_33465828	1399942569	*Ca_kabuli_Chr07*	33465828	C/A	NA	INTERGENIC	NA
Kabuli_wild_33465855	1399942570	*Ca_kabuli_Chr07*	33465855	A/C	NA	INTERGENIC	NA
Kabuli_wild_33480940	1399942571	*Ca_kabuli_Chr07*	33480940	C/T	NA	INTERGENIC	NA
Kabuli_wild_33573605	1399942572	*Ca_kabuli_Chr07*	33573605	C/T	Gene_Ca_16177	SYNONYMOUS_CODING	CCAAT-binding factor
Kabuli_wild_33573655	1399942573	*Ca_kabuli_Chr07*	33573655	A/C	Gene_Ca_16177	NON_SYNONYMOUS_CODING	CCAAT-binding factor
Kabuli_wild_33619268	1399942574	*Ca_kabuli_Chr07*	33619268	G/C	NA	INTERGENIC	NA
Kabuli_wild_33624168	1399942575	*Ca_kabuli_Chr07*	33624168	A/G	NA	INTERGENIC	NA
Kabuli_wild_33624175	1399942576	*Ca_kabuli_Chr07*	33624175	A/G	NA	INTERGENIC	NA
Kabuli_wild_33624207	1399942577	*Ca_kabuli_Chr07*	33624207	G/T	NA	INTERGENIC	NA
Kabuli_wild_33624227	1399942578	*Ca_kabuli_Chr07*	33624227	C/T	NA	INTERGENIC	NA
Kabuli_wild_33624249	1399942579	*Ca_kabuli_Chr07*	33624249	A/G	NA	INTERGENIC	NA
Kabuli_wild_33641799	1399942580	*Ca_kabuli_Chr07*	33641799	G/T	Gene_Ca_16180	SYNONYMOUS_CODING	Pathogenesis-related transcriptional factor/ERF, DNA-binding
Kabuli_wild_33641882	1399942581	*Ca_kabuli_Chr07*	33641882	A/T	Gene_Ca_16180	INTRON	Pathogenesis-related transcriptional factor/ERF, DNA-binding
Kabuli_wild_33674454	1399942582	*Ca_kabuli_Chr07*	33674454	T/C	Gene_Ca_16181	SYNONYMOUS_CODING	PeptidaseS8/S53,subtilisin/kexin/sedolisin
Kabuli_wild_33744521	1399942583	*Ca_kabuli_Chr07*	33744521	C/G	Gene_Ca_16182	INTRON	ARID/BRIGHTDNA-binding domain

SNP IDs	NCBI dbSNP accession IDs (NCBI_SS#)	Chromosomes	Physical positions (bp)	SNPs	Gene accession IDs	Sequence components of genome	Putative functions
Kabuli_wild_33753917	1399942584	*Ca_kabuli_Chr07*	33753917	A/G	Gene_Ca_16183	SYNONYMOUS_CODING	Initiation factor2B-related
Kabuli_wild_33761944	1399942585	*Ca_kabuli_Chr07*	33761944	G/A	Gene_Ca_16184	SYNONYMOUS_CODING	NA
Kabuli_wild_33762053	1399942586	*Ca_kabuli_Chr07*	33762053	T/G	Gene_Ca_16184	NON_SYNONYMOUS_CODING	NA
Kabuli_wild_33821658	1399942587	*Ca_kabuli_Chr07*	33821658	C/T	Gene_Ca_16190	INTRON	1-aminocyclopropane-1-carboxylatesynthase
Kabuli_wild_33835856	1399942588	*Ca_kabuli_Chr07*	33835856	A/G	Gene_Ca_16191	SYNONYMOUS_CODING	Pectinesterase,catalytic
Kabuli_wild_33865463	1399942589	*Ca_kabuli_Chr07*	33865463	C/T	Gene_Ca_16193	INTRON	ExocystcomplexcomponentSec10
Kabuli_wild_33865475	1399942590	*Ca_kabuli_Chr07*	33865475	G/A	Gene_Ca_16193	INTRON	ExocystcomplexcomponentSec10
Kabuli_wild_33867334	1399942591	*Ca_kabuli_Chr07*	33867334	C/T	Gene_Ca_16193	INTRON	ExocystcomplexcomponentSec10
Kabuli_wild_33867491	1399942592	*Ca_kabuli_Chr07*	33867491	T/C	Gene_Ca_16193	INTRON	ExocystcomplexcomponentSec10
Kabuli_wild_33867674	1399942593	*Ca_kabuli_Chr07*	33867674	C/T	Gene_Ca_16193	INTRON	ExocystcomplexcomponentSec10
Kabuli_wild_33868148	1399942594	*Ca_kabuli_Chr07*	33868148	T/C	Gene_Ca_16193	SYNONYMOUS_CODING	ExocystcomplexcomponentSec10
Kabuli_wild_33869367	1399942595	*Ca_kabuli_Chr07*	33869367	G/A	Gene_Ca_16193	SYNONYMOUS_CODING	ExocystcomplexcomponentSec10
Kabuli_wild_33879017	1399942596	*Ca_kabuli_Chr07*	33879017	T/A	Gene_Ca_16194	INTRON	Ubiquitin-conjugating enzyme, E2
Kabuli_wild_33906614	1399942597	*Ca_kabuli_Chr07*	33906614	C/T	NA	INTERGENIC	NA
Kabuli_wild_33910324	1399942598	*Ca_kabuli_Chr07*	33910324	C/A	NA	INTERGENIC	NA
Kabuli_wild_33910870	1399942599	*Ca_kabuli_Chr07*	33910870	C/A	NA	INTERGENIC	NA
Kabuli_wild_33912719	1399942600	*Ca_kabuli_Chr07*	33912719	T/G	NA	INTERGENIC	NA
Kabuli_wild_33912758	1399942601	*Ca_kabuli_Chr07*	33912758	T/C	NA	INTERGENIC	NA
Kabuli_wild_33912866	1399942602	*Ca_kabuli_Chr07*	33912866	C/T	NA	INTERGENIC	NA
Kabuli_wild_33912899	1399942603	*Ca_kabuli_Chr07*	33912899	T/A	NA	INTERGENIC	NA
Kabuli_wild_33912902	1399942604	*Ca_kabuli_Chr07*	33912902	A/T	NA	INTERGENIC	NA
Kabuli_wild_33912913	1399942605	*Ca_kabuli_Chr07*	33912913	A/T	NA	INTERGENIC	NA
Kabuli_wild_33912916	1399942606	*Ca_kabuli_Chr07*	33912916	G/T	NA	INTERGENIC	NA
Kabuli_wild_33912921	1399942607	*Ca_kabuli_Chr07*	33912921	T/G	NA	INTERGENIC	NA
Kabuli_wild_33912950	1399942608	*Ca_kabuli_Chr07*	33912950	C/T	NA	INTERGENIC	NA
Kabuli_wild_33912965	1399942609	*Ca_kabuli_Chr07*	33912965	G/T	NA	INTERGENIC	NA
Kabuli_wild_33912966	1399942610	*Ca_kabuli_Chr07*	33912966	T/C	NA	INTERGENIC	NA
Kabuli_wild_33913045	1399942611	*Ca_kabuli_Chr07*	33913045	G/T	NA	INTERGENIC	NA
Kabuli_wild_33913047	1399942612	*Ca_kabuli_Chr07*	33913047	C/T	NA	INTERGENIC	NA
Kabuli_wild_33913094	1399942613	*Ca_kabuli_Chr07*	33913094	A/G	NA	INTERGENIC	NA
Kabuli_wild_34079083	1399942614	*Ca_kabuli_Chr07*	34079083	C/T	Gene_Ca_16200	INTRON	NA
Kabuli_wild_34107603	1399942615	*Ca_kabuli_Chr07*	34107603	C/A	NA	INTERGENIC	NA
Kabuli_wild_34152124	1399942616	*Ca_kabuli_Chr07*	34152124	G/C	Gene_Ca_21006	NON_SYNONYMOUS_CODING	DNA-directed RNA polymerase,subunit 2, domain 6
Kabuli_wild_34152720	1399942617	*Ca_kabuli_Chr07*	34152720	G/T	Gene_Ca_21006	INTRON	DNA-directed RNA polymerase,subunit 2, domain 6
Kabuli_wild_34227428	1399942618	*Ca_kabuli_Chr07*	34227428	G/A	NA	INTERGENIC	NA
Kabuli_wild_34227786	1399942619	*Ca_kabuli_Chr07*	34227786	G/A	NA	INTERGENIC	NA
Kabuli_wild_34271486	1399942620	*Ca_kabuli_Chr07*	34271486	A/G	NA	INTERGENIC	NA
Kabuli_wild_34276965	1399942621	*Ca_kabuli_Chr07*	34276965	T/C	NA	INTERGENIC	NA
Kabuli_wild_34277010	1399942622	*Ca_kabuli_Chr07*	34277010	A/G	NA	INTERGENIC	NA
Kabuli_wild_34277014	1399942623	*Ca_kabuli_Chr07*	34277014	G/T	NA	INTERGENIC	NA
Kabuli_wild_34279357	1399942624	*Ca_kabuli_Chr07*	34279357	G/T	NA	INTERGENIC	NA
Kabuli_wild_34279673	1399942625	*Ca_kabuli_Chr07*	34279673	G/A	NA	INTERGENIC	NA
Kabuli_wild_34279715	1399942626	*Ca_kabuli_Chr07*	34279715	A/C	NA	INTERGENIC	NA
Kabuli_wild_34279725	1399942627	*Ca_kabuli_Chr07*	34279725	A/G	NA	INTERGENIC	NA

SNP IDs	NCBI dbSNP accession IDs (NCBI_SS#)	Chromosomes	Physical positions (bp)	SNPs	Gene accession IDs	Sequence components of genome	Putative functions
Kabuli_wild_34318860	1399942628	*Ca_kabuli_Chr07*	34318860	C/T	NA	INTERGENIC	NA
Kabuli_wild_34318862	1399942629	*Ca_kabuli_Chr07*	34318862	A/G	NA	INTERGENIC	NA
Kabuli_wild_34474614	1399942630	*Ca_kabuli_Chr07*	34474614	A/C	Gene_Ca_17602	NON_SYNONYMOUS_CODING	SSXT
Kabuli_wild_34475540	1399942631	*Ca_kabuli_Chr07*	34475540	G/A	Gene_Ca_17602	DOWNSTREAM	SSXT
Kabuli_wild_34796876	1399942632	*Ca_kabuli_Chr07*	34796876	C/T	NA	INTERGENIC	NA
Kabuli_wild_34801179	1399942633	*Ca_kabuli_Chr07*	34801179	T/C	NA	INTERGENIC	NA
Kabuli_wild_34801839	1399942634	*Ca_kabuli_Chr07*	34801839	A/T	Gene_Ca_17614	SYNONYMOUS_CODING	NA
Kabuli_wild_34857693	1399942635	*Ca_kabuli_Chr07*	34857693	C/T	Gene_Ca_17617	NON_SYNONYMOUS_CODING	Armadillo
Kabuli_wild_34879321	1399942636	*Ca_kabuli_Chr07*	34879321	T/G	Gene_Ca_17621	DOWNSTREAM	EF-Hand1,calcium-bindingsite
Kabuli_wild_34944888	1399942637	*Ca_kabuli_Chr07*	34944888	G/A	Gene_Ca_17626	NON_SYNONYMOUS_CODING	Phosphoribosyltransferase
Kabuli_wild_34947729	1399942638	*Ca_kabuli_Chr07*	34947729	C/A	Gene_Ca_17626	INTRON	Phosphoribosyltransferase
Kabuli_wild_34947743	1399942639	*Ca_kabuli_Chr07*	34947743	A/C	Gene_Ca_17626	INTRON	Phosphoribosyltransferase
Kabuli_wild_35016516	1399942640	*Ca_kabuli_Chr07*	35016516	G/C	Gene_Ca_17628	DOWNSTREAM	Pyridoxamine5'-phosphateoxidase
Kabuli_wild_35038291	1399942641	*Ca_kabuli_Chr07*	35038291	A/C	NA	INTERGENIC	NA
Kabuli_wild_35072425	1399942642	*Ca_kabuli_Chr07*	35072425	A/T	NA	INTERGENIC	NA
Kabuli_wild_35074237	1399942643	*Ca_kabuli_Chr07*	35074237	C/G	NA	INTERGENIC	NA
Kabuli_wild_35074280	1399942644	*Ca_kabuli_Chr07*	35074280	A/C	NA	INTERGENIC	NA
Kabuli_wild_35074362	1399942645	*Ca_kabuli_Chr07*	35074362	T/C	NA	INTERGENIC	NA
Kabuli_wild_35074547	1399942646	*Ca_kabuli_Chr07*	35074547	C/T	NA	INTERGENIC	NA
Kabuli_wild_35074548	1399942647	*Ca_kabuli_Chr07*	35074548	C/A	NA	INTERGENIC	NA
Kabuli_wild_35112785	1399942648	*Ca_kabuli_Chr07*	35112785	T/C	Gene_Ca_17637	SYNONYMOUS_CODING	Importin-beta,N-terminal
Kabuli_wild_35114049	1399942649	*Ca_kabuli_Chr07*	35114049	C/T	Gene_Ca_17637	NON_SYNONYMOUS_CODING	Importin-beta,N-terminal
Kabuli_wild_35134809	1399942650	*Ca_kabuli_Chr07*	35134809	A/C	Gene_Ca_17638	NON_SYNONYMOUS_CODING	Pathogenesis-related transcriptional factor/ERF, DNA-binding
Kabuli_wild_35134816	1399942651	*Ca_kabuli_Chr07*	35134816	A/C	Gene_Ca_17638	NON_SYNONYMOUS_CODING	Pathogenesis-related transcriptional factor/ERF, DNA-binding
Kabuli_wild_35134824	1399942652	*Ca_kabuli_Chr07*	35134824	A/C	Gene_Ca_17638	SYNONYMOUS_CODING	Pathogenesis-related transcriptional factor/ERF, DNA-binding
Kabuli_wild_35134840	1399942653	*Ca_kabuli_Chr07*	35134840	G/T	Gene_Ca_17638	NON_SYNONYMOUS_CODING	Pathogenesis-related transcriptional factor/ERF, DNA-binding
Kabuli_wild_35134863	1399942654	*Ca_kabuli_Chr07*	35134863	T/C	Gene_Ca_17638	SYNONYMOUS_CODING	Pathogenesis-related transcriptional factor/ERF, DNA-binding
Kabuli_wild_35181514	1399942655	*Ca_kabuli_Chr07*	35181514	A/T	Gene_Ca_17639	NON_SYNONYMOUS_CODING	Helix-loop-helixDNA-binding domain
Kabuli_wild_35181619	1399942656	*Ca_kabuli_Chr07*	35181619	T/G	Gene_Ca_17639	NON_SYNONYMOUS_CODING	Helix-loop-helixDNA-binding domain
Kabuli_wild_35181635	1399942657	*Ca_kabuli_Chr07*	35181635	G/C	Gene_Ca_17639	NON_SYNONYMOUS_CODING	Helix-loop-helixDNA-binding domain
Kabuli_wild_35181660	1399942658	*Ca_kabuli_Chr07*	35181660	G/A	Gene_Ca_17639	NON_SYNONYMOUS_CODING	Helix-loop-helixDNA-binding domain
Kabuli_wild_35181661	1399942659	*Ca_kabuli_Chr07*	35181661	T/C	Gene_Ca_17639	SYNONYMOUS_CODING	Helix-loop-helixDNA-binding domain
Kabuli_wild_35227794	1399942660	*Ca_kabuli_Chr07*	35227794	A/C	NA	INTERGENIC	NA
Kabuli_wild_35258029	1399942661	*Ca_kabuli_Chr07*	35258029	T/G	Gene_Ca_17644	INTRON	Dynamincentral domain
Kabuli_wild_35263711	1399942662	*Ca_kabuli_Chr07*	35263711	A/T	Gene_Ca_17645	UPSTREAM	NA
Kabuli_wild_35263712	1399942663	*Ca_kabuli_Chr07*	35263712	G/T	Gene_Ca_17645	UPSTREAM	NA
Kabuli_wild_35263736	1399942664	*Ca_kabuli_Chr07*	35263736	C/G	Gene_Ca_17645	UPSTREAM	NA
Kabuli_wild_35263758	1399942665	*Ca_kabuli_Chr07*	35263758	A/G	Gene_Ca_17645	UPSTREAM	NA
Kabuli_wild_35274368	1399942666	*Ca_kabuli_Chr07*	35274368	A/G	NA	INTERGENIC	NA
Kabuli_wild_35274680	1399942667	*Ca_kabuli_Chr07*	35274680	G/A	NA	INTERGENIC	NA
Kabuli_wild_35274828	1399942668	*Ca_kabuli_Chr07*	35274828	A/G	NA	INTERGENIC	NA
Kabuli_wild_35274858	1399942669	*Ca_kabuli_Chr07*	35274858	G/A	NA	INTERGENIC	NA
Kabuli_wild_35274859	1399942670	*Ca_kabuli_Chr07*	35274859	T/G	NA	INTERGENIC	NA
Kabuli_wild_35279675	1399942671	*Ca_kabuli_Chr07*	35279675	A/G	Gene_Ca_17646	NON_SYNONYMOUS_CODING	NA

SNP IDs	NCBI dbSNP accession IDs (NCBI_SS#)	Chromosomes	Physical positions (bp)	SNPs	Gene accession IDs	Sequence components of genome	Putative functions
Kabuli_wild_35280167	1399942672	*Ca_kabuli_Chr07*	35280167	A/G	Gene_Ca_17646	SYNONYMOUS_CODING	NA
Kabuli_wild_35280189	1399942673	*Ca_kabuli_Chr07*	35280189	G/A	Gene_Ca_17646	SYNONYMOUS_CODING	NA
Kabuli_wild_35322382	1399942674	*Ca_kabuli_Chr07*	35322382	T/C	Gene_Ca_17647	INTRON	PWWP
Kabuli_wild_35342271	1399942675	*Ca_kabuli_Chr07*	35342271	A/G	Gene_Ca_17648	DOWNSTREAM	Zinc finger, RING-type
Kabuli_wild_35576427	1399942676	*Ca_kabuli_Chr07*	35576427	C/A	Gene_Ca_17765	NON_SYNONYMOUS_CODING	Reticulon
Kabuli_wild_35619487	1399942677	*Ca_kabuli_Chr07*	35619487	G/A	NA	INTERGENIC	NA
Kabuli_wild_35619518	1399942678	*Ca_kabuli_Chr07*	35619518	G/C	NA	INTERGENIC	NA
Kabuli_wild_35619611	1399942679	*Ca_kabuli_Chr07*	35619611	G/A	NA	INTERGENIC	NA
Kabuli_wild_35622168	1399942680	*Ca_kabuli_Chr07*	35622168	A/G	Gene_Ca_17769	NON_SYNONYMOUS_CODING	Ketopantoate hydroxymethyltransferase
Kabuli_wild_35637090	1399942681	*Ca_kabuli_Chr07*	35637090	T/C	NA	INTERGENIC	NA
Kabuli_wild_35680423	1399942682	*Ca_kabuli_Chr07*	35680423	A/C	NA	INTERGENIC	NA
Kabuli_wild_35723912	1399942683	*Ca_kabuli_Chr07*	35723912	T/C	NA	INTERGENIC	NA
Kabuli_wild_35911606	1399942684	*Ca_kabuli_Chr07*	35911606	T/C	NA	INTERGENIC	NA
Kabuli_wild_35911612	1399942685	*Ca_kabuli_Chr07*	35911612	T/G	NA	INTERGENIC	NA
Kabuli_wild_35911615	1399942686	*Ca_kabuli_Chr07*	35911615	C/T	NA	INTERGENIC	NA
Kabuli_wild_35911617	1399942687	*Ca_kabuli_Chr07*	35911617	A/C	NA	INTERGENIC	NA
Kabuli_wild_35911620	1399942688	*Ca_kabuli_Chr07*	35911620	T/G	NA	INTERGENIC	NA
Kabuli_wild_35940587	1399942689	*Ca_kabuli_Chr07*	35940587	A/G	NA	INTERGENIC	NA
Kabuli_wild_35947529	1399942690	*Ca_kabuli_Chr07*	35947529	T/C	Gene_Ca_17800	NON_SYNONYMOUS_CODING	Transposase,Ptta/En/Spm,plant
Kabuli_wild_35947538	1399942691	*Ca_kabuli_Chr07*	35947538	G/A	Gene_Ca_17800	NON_SYNONYMOUS_CODING	Transposase,Ptta/En/Spm,plant
Kabuli_wild_35947550	1399942692	*Ca_kabuli_Chr07*	35947550	C/T	Gene_Ca_17800	NON_SYNONYMOUS_CODING	Transposase,Ptta/En/Spm,plant
Kabuli_wild_35947551	1399942693	*Ca_kabuli_Chr07*	35947551	A/G	Gene_Ca_17800	SYNONYMOUS_CODING	Transposase,Ptta/En/Spm,plant
Kabuli_wild_35959825	1399942694	*Ca_kabuli_Chr07*	35959825	G/A	NA	INTERGENIC	NA
Kabuli_wild_35959878	1399942695	*Ca_kabuli_Chr07*	35959878	A/G	NA	INTERGENIC	NA
Kabuli_wild_35972411	1399942696	*Ca_kabuli_Chr07*	35972411	G/A	NA	INTERGENIC	NA
Kabuli_wild_36681308	1399942697	*Ca_kabuli_Chr07*	36681308	G/A	Gene_Ca_13755	NON_SYNONYMOUS_CODING	NA
Kabuli_wild_36711954	1399942698	*Ca_kabuli_Chr07*	36711954	C/A	Gene_Ca_13756	NON_SYNONYMOUS_CODING	Mediatorcomplex,subunitMed12
Kabuli_wild_36715249	1399942699	*Ca_kabuli_Chr07*	36715249	T/C	Gene_Ca_13756	SYNONYMOUS_CODING	Mediatorcomplex,subunitMed12
Kabuli_wild_36745439	1399942700	*Ca_kabuli_Chr07*	36745439	T/G	Gene_Ca_13760	UPSTREAM	Oxoglutarate/iron-dependent oxygenase
Kabuli_wild_36916273	1399942701	*Ca_kabuli_Chr07*	36916273	T/A	NA	INTERGENIC	NA
Kabuli_wild_36916275	1399942702	*Ca_kabuli_Chr07*	36916275	T/G	NA	INTERGENIC	NA
Kabuli_wild_36916281	1399942703	*Ca_kabuli_Chr07*	36916281	T/G	NA	INTERGENIC	NA
Kabuli_wild_36933363	1399942704	*Ca_kabuli_Chr07*	36933363	A/T	NA	INTERGENIC	NA
Kabuli_wild_36933370	1399942705	*Ca_kabuli_Chr07*	36933370	C/G	NA	INTERGENIC	NA
Kabuli_wild_36998083	1399942706	*Ca_kabuli_Chr07*	36998083	G/A	Gene_Ca_13784	NON_SYNONYMOUS_CODING	Peptidase C48, SUMO/Sentrin/Ubl1
Kabuli_wild_36998096	1399942707	*Ca_kabuli_Chr07*	36998096	T/C	Gene_Ca_13784	SYNONYMOUS_CODING	Peptidase C48, SUMO/Sentrin/Ubl1
Kabuli_wild_36998104	1399942708	*Ca_kabuli_Chr07*	36998104	T/G	Gene_Ca_13784	NON_SYNONYMOUS_CODING	Peptidase C48, SUMO/Sentrin/Ubl1
Kabuli_wild_36998113	1399942709	*Ca_kabuli_Chr07*	36998113	C/T	Gene_Ca_13784	SYNONYMOUS_CODING	Peptidase C48, SUMO/Sentrin/Ubl1
Kabuli_wild_36998163	1399942710	*Ca_kabuli_Chr07*	36998163	G/A	Gene_Ca_13784	SYNONYMOUS_CODING	Peptidase C48, SUMO/Sentrin/Ubl1
Kabuli_wild_36998180	1399942711	*Ca_kabuli_Chr07*	36998180	C/T	Gene_Ca_13784	NON_SYNONYMOUS_CODING	Peptidase C48, SUMO/Sentrin/Ubl1
Kabuli_wild_37058979	1399942712	*Ca_kabuli_Chr07*	37058979	C/T	Gene_Ca_13790	NON_SYNONYMOUS_CODING	Hydroxymethylglutaryl-CoAlyase,activesite
Kabuli_wild_37114617	1399942713	*Ca_kabuli_Chr07*	37114617	C/T	NA	INTERGENIC	NA
Kabuli_wild_37187439	1399942714	*Ca_kabuli_Chr07*	37187439	A/G	Gene_Ca_13799	UPSTREAM	Short-chaindehydrogenase/reductaseSDR
Kabuli_wild_37626653	1399942715	*Ca_kabuli_Chr07*	37626653	A/C	Gene_Ca_22806	NON_SYNONYMOUS_CODING	Transposase,Ptta/En/Spm,plant

SNP IDs	NCBI dbSNP accession IDs (NCBI_SS#)	Chromosomes	Physical positions (bp)	SNPs	Gene accession IDs	Sequence components of genome	Putative functions
Kabuli_wild_37626665	1399942716	*Ca_kabuli_Chr07*	37626665	G/A	Gene_Ca_22806	SYNONYMOUS_CODING	Transposase,Ptta/En/Spm,plant
Kabuli_wild_37626666	1399942717	*Ca_kabuli_Chr07*	37626666	C/T	Gene_Ca_22806	NON_SYNONYMOUS_CODING	Transposase,Ptta/En/Spm,plant
Kabuli_wild_37626687	1399942718	*Ca_kabuli_Chr07*	37626687	A/G	Gene_Ca_22806	NON_SYNONYMOUS_CODING	Transposase,Ptta/En/Spm,plant
Kabuli_wild_37626729	1399942719	*Ca_kabuli_Chr07*	37626729	G/T	Gene_Ca_22806	NON_SYNONYMOUS_CODING	Transposase,Ptta/En/Spm,plant
Kabuli_wild_37626802	1399942720	*Ca_kabuli_Chr07*	37626802	G/A	Gene_Ca_22806	SYNONYMOUS_CODING	Transposase,Ptta/En/Spm,plant
Kabuli_wild_37626805	1399942721	*Ca_kabuli_Chr07*	37626805	A/G	Gene_Ca_22806	INTRON	Transposase,Ptta/En/Spm,plant
Kabuli_wild_37626809	1399942722	*Ca_kabuli_Chr07*	37626809	A/T	Gene_Ca_22806	INTRON	Transposase,Ptta/En/Spm,plant
Kabuli_wild_37626827	1399942723	*Ca_kabuli_Chr07*	37626827	A/G	Gene_Ca_22806	INTRON	Transposase,Ptta/En/Spm,plant
Kabuli_wild_37703912	1399942724	*Ca_kabuli_Chr07*	37703912	G/A	NA	INTERGENIC	NA
Kabuli_wild_37703913	1399942725	*Ca_kabuli_Chr07*	37703913	C/G	NA	INTERGENIC	NA
Kabuli_wild_37703921	1399942726	*Ca_kabuli_Chr07*	37703921	G/T	NA	INTERGENIC	NA
Kabuli_wild_37703925	1399942727	*Ca_kabuli_Chr07*	37703925	C/T	NA	INTERGENIC	NA
Kabuli_wild_37703936	1399942728	*Ca_kabuli_Chr07*	37703936	C/T	NA	INTERGENIC	NA
Kabuli_wild_37703965	1399942729	*Ca_kabuli_Chr07*	37703965	C/T	NA	INTERGENIC	NA
Kabuli_wild_37703966	1399942730	*Ca_kabuli_Chr07*	37703966	C/T	NA	INTERGENIC	NA
Kabuli_wild_37703967	1399942731	*Ca_kabuli_Chr07*	37703967	G/A	NA	INTERGENIC	NA
Kabuli_wild_37853290	1399942732	*Ca_kabuli_Chr07*	37853290	C/T	NA	INTERGENIC	NA
Kabuli_wild_37853314	1399942733	*Ca_kabuli_Chr07*	37853314	G/A	NA	INTERGENIC	NA
Kabuli_wild_37853315	1399942734	*Ca_kabuli_Chr07*	37853315	C/G	NA	INTERGENIC	NA
Kabuli_wild_37857870	1399942735	*Ca_kabuli_Chr07*	37857870	C/T	NA	INTERGENIC	NA
Kabuli_wild_37857887	1399942736	*Ca_kabuli_Chr07*	37857887	C/T	NA	INTERGENIC	NA
Kabuli_wild_37857928	1399942737	*Ca_kabuli_Chr07*	37857928	A/G	NA	INTERGENIC	NA
Kabuli_wild_37857936	1399942738	*Ca_kabuli_Chr07*	37857936	G/A	NA	INTERGENIC	NA
Kabuli_wild_37857951	1399942739	*Ca_kabuli_Chr07*	37857951	C/T	NA	INTERGENIC	NA
Kabuli_wild_37886451	1399942740	*Ca_kabuli_Chr07*	37886451	T/A	NA	INTERGENIC	NA
Kabuli_wild_37886519	1399942741	*Ca_kabuli_Chr07*	37886519	A/C	NA	INTERGENIC	NA
Kabuli_wild_38044503	1399942742	*Ca_kabuli_Chr07*	38044503	G/A	NA	INTERGENIC	NA
Kabuli_wild_38044536	1399942743	*Ca_kabuli_Chr07*	38044536	G/A	NA	INTERGENIC	NA
Kabuli_wild_38047374	1399942744	*Ca_kabuli_Chr07*	38047374	A/G	NA	INTERGENIC	NA
Kabuli_wild_38047378	1399942745	*Ca_kabuli_Chr07*	38047378	C/A	NA	INTERGENIC	NA
Kabuli_wild_38143361	1399942746	*Ca_kabuli_Chr07*	38143361	A/G	Gene_Ca_16396	INTRON	Fructosamine/Ketosamine-3-kinase
Kabuli_wild_38143452	1399942747	*Ca_kabuli_Chr07*	38143452	G/A	Gene_Ca_16396	INTRON	Fructosamine/Ketosamine-3-kinase
Kabuli_wild_38143524	1399942748	*Ca_kabuli_Chr07*	38143524	T/A	Gene_Ca_16396	INTRON	Fructosamine/Ketosamine-3-kinase
Kabuli_wild_38167486	1399942749	*Ca_kabuli_Chr07*	38167486	T/G	NA	INTERGENIC	NA
Kabuli_wild_38238977	1399942750	*Ca_kabuli_Chr07*	38238977	C/T	Gene_Ca_16402	INTRON	NA
Kabuli_wild_38409029	1399942751	*Ca_kabuli_Chr07*	38409029	T/G	NA	INTERGENIC	NA
Kabuli_wild_38460415	1399942752	*Ca_kabuli_Chr07*	38460415	T/G	NA	INTERGENIC	NA
Kabuli_wild_38460425	1399942753	*Ca_kabuli_Chr07*	38460425	G/A	NA	INTERGENIC	NA
Kabuli_wild_38460438	1399942754	*Ca_kabuli_Chr07*	38460438	C/A	NA	INTERGENIC	NA
Kabuli_wild_38460452	1399942755	*Ca_kabuli_Chr07*	38460452	C/T	NA	INTERGENIC	NA
Kabuli_wild_38460455	1399942756	*Ca_kabuli_Chr07*	38460455	G/A	NA	INTERGENIC	NA
Kabuli_wild_38460457	1399942757	*Ca_kabuli_Chr07*	38460457	A/G	NA	INTERGENIC	NA
Kabuli_wild_38460464	1399942758	*Ca_kabuli_Chr07*	38460464	G/A	NA	INTERGENIC	NA
Kabuli_wild_38707437	1399942759	*Ca_kabuli_Chr07*	38707437	T/C	NA	INTERGENIC	NA

SNP IDs	NCBI dbSNP accession IDs (NCBI_SS#)	Chromosomes	Physical positions (bp)	SNPs	Gene accession IDs	Sequence components of genome	Putative functions
Kabuli_wild_39181763	1399942760	*Ca_kabuli_Chr07*	39181763	T/G	Gene_Ca_19581	SYNONYMOUS_CODING	BEACHdomain
Kabuli_wild_39182564	1399942761	*Ca_kabuli_Chr07*	39182564	A/T	Gene_Ca_19581	NON_SYNONYMOUS_CODING	BEACHdomain
Kabuli_wild_39215009	1399942762	*Ca_kabuli_Chr07*	39215009	C/T	Gene_Ca_19581	INTRON	BEACHdomain
Kabuli_wild_39339495	1399942763	*Ca_kabuli_Chr07*	39339495	G/T	Gene_Ca_19588	NON_SYNONYMOUS_CODING	WRC
Kabuli_wild_39339498	1399942764	*Ca_kabuli_Chr07*	39339498	G/T	Gene_Ca_19588	NON_SYNONYMOUS_CODING	WRC
Kabuli_wild_39339499	1399942765	*Ca_kabuli_Chr07*	39339499	C/T	Gene_Ca_19588	NON_SYNONYMOUS_CODING	WRC
Kabuli_wild_39339500	1399942766	*Ca_kabuli_Chr07*	39339500	G/T	Gene_Ca_19588	SYNONYMOUS_CODING	WRC
Kabuli_wild_39339501	1399942767	*Ca_kabuli_Chr07*	39339501	G/C	Gene_Ca_19588	NON_SYNONYMOUS_CODING	WRC
Kabuli_wild_39339502	1399942768	*Ca_kabuli_Chr07*	39339502	C/A	Gene_Ca_19588	NON_SYNONYMOUS_CODING	WRC
Kabuli_wild_39339503	1399942769	*Ca_kabuli_Chr07*	39339503	C/A	Gene_Ca_19588	SYNONYMOUS_CODING	WRC
Kabuli_wild_39339508	1399942770	*Ca_kabuli_Chr07*	39339508	G/A	Gene_Ca_19588	NON_SYNONYMOUS_CODING	WRC
Kabuli_wild_39339509	1399942771	*Ca_kabuli_Chr07*	39339509	G/A	Gene_Ca_19588	SYNONYMOUS_CODING	WRC
Kabuli_wild_39339510	1399942772	*Ca_kabuli_Chr07*	39339510	T/G	Gene_Ca_19588	NON_SYNONYMOUS_CODING	WRC
Kabuli_wild_39348139	1399942773	*Ca_kabuli_Chr07*	39348139	C/T	Gene_Ca_19589	DOWNSTREAM	Pentatrico peptide repeat
Kabuli_wild_39399280	1399942774	*Ca_kabuli_Chr07*	39399280	T/C	Gene_Ca_19592	SYNONYMOUS_CODING	Domain of unknown function DUF296
Kabuli_wild_39630792	1399942775	*Ca_kabuli_Chr07*	39630792	G/A	NA	INTERGENIC	NA
Kabuli_wild_39630798	1399942776	*Ca_kabuli_Chr07*	39630798	T/G	NA	INTERGENIC	NA
Kabuli_wild_39630802	1399942777	*Ca_kabuli_Chr07*	39630802	G/A	NA	INTERGENIC	NA
Kabuli_wild_39630807	1399942778	*Ca_kabuli_Chr07*	39630807	A/G	NA	INTERGENIC	NA
Kabuli_wild_39630812	1399942779	*Ca_kabuli_Chr07*	39630812	T/C	NA	INTERGENIC	NA
Kabuli_wild_39680221	1399942780	*Ca_kabuli_Chr07*	39680221	C/A	Gene_Ca_19607	INTRON	Domain of unknown function DUF296
Kabuli_wild_39680222	1399942781	*Ca_kabuli_Chr07*	39680222	G/A	Gene_Ca_19607	INTRON	Domain of unknown function DUF296
Kabuli_wild_39944444	1399942782	*Ca_kabuli_Chr07*	39944444	T/C	Gene_Ca_25106	SYNONYMOUS_CODING	Mitochondrial carrier protein
Kabuli_wild_39944474	1399942783	*Ca_kabuli_Chr07*	39944474	T/C	Gene_Ca_25106	SYNONYMOUS_CODING	Mitochondrial carrier protein
Kabuli_wild_40092162	1399942784	*Ca_kabuli_Chr07*	40092162	T/G	Gene_Ca_24269	NON_SYNONYMOUS_CODING	NA
Kabuli_wild_40092181	1399942785	*Ca_kabuli_Chr07*	40092181	C/T	Gene_Ca_24269	NON_SYNONYMOUS_CODING	NA
Kabuli_wild_40092185	1399942786	*Ca_kabuli_Chr07*	40092185	A/G	Gene_Ca_24269	SYNONYMOUS_CODING	NA
Kabuli_wild_40092198	1399942787	*Ca_kabuli_Chr07*	40092198	G/A	Gene_Ca_24269	NON_SYNONYMOUS_CODING	NA
Kabuli_wild_40092201	1399942788	*Ca_kabuli_Chr07*	40092201	G/A	Gene_Ca_24269	NON_SYNONYMOUS_CODING	NA
Kabuli_wild_40092211	1399942789	*Ca_kabuli_Chr07*	40092211	C/T	Gene_Ca_24269	NON_SYNONYMOUS_CODING	NA
Kabuli_wild_40092213	1399942790	*Ca_kabuli_Chr07*	40092213	G/C	Gene_Ca_24269	NON_SYNONYMOUS_CODING	NA
Kabuli_wild_40092214	1399942791	*Ca_kabuli_Chr07*	40092214	T/C	Gene_Ca_24269	SYNONYMOUS_CODING	NA
Kabuli_wild_40092218	1399942792	*Ca_kabuli_Chr07*	40092218	A/G	Gene_Ca_24269	SYNONYMOUS_CODING	NA
Kabuli_wild_40092229	1399942793	*Ca_kabuli_Chr07*	40092229	C/A	Gene_Ca_24269	NON_SYNONYMOUS_CODING	NA
Kabuli_wild_40092237	1399942794	*Ca_kabuli_Chr07*	40092237	C/T	Gene_Ca_24269	SYNONYMOUS_CODING	NA
Kabuli_wild_40092250	1399942795	*Ca_kabuli_Chr07*	40092250	A/C	Gene_Ca_24269	NON_SYNONYMOUS_CODING	NA
Kabuli_wild_40092253	1399942796	*Ca_kabuli_Chr07*	40092253	C/T	Gene_Ca_24269	NON_SYNONYMOUS_CODING	NA
Kabuli_wild_40092256	1399942797	*Ca_kabuli_Chr07*	40092256	T/C	Gene_Ca_24269	NON_SYNONYMOUS_CODING	NA
Kabuli_wild_40092267	1399942798	*Ca_kabuli_Chr07*	40092267	G/C	Gene_Ca_24269	NON_SYNONYMOUS_CODING	NA
Kabuli_wild_40092270	1399942799	*Ca_kabuli_Chr07*	40092270	A/G	Gene_Ca_24269	NON_SYNONYMOUS_CODING	NA
Kabuli_wild_40092340	1399942800	*Ca_kabuli_Chr07*	40092340	T/C	Gene_Ca_24269	NON_SYNONYMOUS_CODING	NA
Kabuli_wild_40142149	1399942801	*Ca_kabuli_Chr07*	40142149	C/A	NA	INTERGENIC	NA
Kabuli_wild_40183449	1399942802	*Ca_kabuli_Chr07*	40183449	C/A	NA	INTERGENIC	NA
Kabuli_wild_40183452	1399942803	*Ca_kabuli_Chr07*	40183452	A/C	NA	INTERGENIC	NA

SNP IDs	NCBI dbSNP accession IDs (NCBI_SS#)	Chromosomes	Physical positions (bp)	SNPs	Gene accession IDs	Sequence components of genome	Putative functions
Kabuli_wild_40217527	1399942804	*Ca_kabuli_Chr07*	40217527	G/T	NA	INTERGENIC	NA
Kabuli_wild_40242765	1399942805	*Ca_kabuli_Chr07*	40242765	A/G	Gene_Ca_24261	INTRON	RNA recognition motif domain
Kabuli_wild_40260831	1399942806	*Ca_kabuli_Chr07*	40260831	A/C	NA	INTERGENIC	NA
Kabuli_wild_40260845	1399942807	*Ca_kabuli_Chr07*	40260845	A/T	NA	INTERGENIC	NA
Kabuli_wild_40260853	1399942808	*Ca_kabuli_Chr07*	40260853	G/T	NA	INTERGENIC	NA
Kabuli_wild_40260889	1399942809	*Ca_kabuli_Chr07*	40260889	C/G	NA	INTERGENIC	NA
Kabuli_wild_40260893	1399942810	*Ca_kabuli_Chr07*	40260893	G/T	NA	INTERGENIC	NA
Kabuli_wild_40323212	1399942811	*Ca_kabuli_Chr07*	40323212	G/A	Gene_Ca_20251	DOWNSTREAM	NA
Kabuli_wild_40323213	1399942812	*Ca_kabuli_Chr07*	40323213	C/A	Gene_Ca_20251	DOWNSTREAM	NA
Kabuli_wild_40323251	1399942813	*Ca_kabuli_Chr07*	40323251	T/A	Gene_Ca_20251	DOWNSTREAM	NA
Kabuli_wild_40323258	1399942814	*Ca_kabuli_Chr07*	40323258	T/G	Gene_Ca_20251	DOWNSTREAM	NA
Kabuli_wild_40340104	1399942815	*Ca_kabuli_Chr07*	40340104	C/A	NA	INTERGENIC	NA
Kabuli_wild_40340114	1399942816	*Ca_kabuli_Chr07*	40340114	A/G	NA	INTERGENIC	NA
Kabuli_wild_40340142	1399942817	*Ca_kabuli_Chr07*	40340142	T/G	NA	INTERGENIC	NA
Kabuli_wild_40340145	1399942818	*Ca_kabuli_Chr07*	40340145	C/T	NA	INTERGENIC	NA
Kabuli_wild_40385974	1399942819	*Ca_kabuli_Chr07*	40385974	T/C	Gene_Ca_20255	INTRON	WD40 repeat
Kabuli_wild_40429548	1399942820	*Ca_kabuli_Chr07*	40429548	A/G	NA	INTERGENIC	NA
Kabuli_wild_40429552	1399942821	*Ca_kabuli_Chr07*	40429552	C/T	NA	INTERGENIC	NA
Kabuli_wild_40429564	1399942822	*Ca_kabuli_Chr07*	40429564	G/T	NA	INTERGENIC	NA
Kabuli_wild_40429567	1399942823	*Ca_kabuli_Chr07*	40429567	G/A	NA	INTERGENIC	NA
Kabuli_wild_40429573	1399942824	*Ca_kabuli_Chr07*	40429573	G/T	NA	INTERGENIC	NA
Kabuli_wild_40429596	1399942825	*Ca_kabuli_Chr07*	40429596	G/A	NA	INTERGENIC	NA
Kabuli_wild_40429599	1399942826	*Ca_kabuli_Chr07*	40429599	G/A	NA	INTERGENIC	NA
Kabuli_wild_40429600	1399942827	*Ca_kabuli_Chr07*	40429600	C/T	NA	INTERGENIC	NA
Kabuli_wild_40429604	1399942828	*Ca_kabuli_Chr07*	40429604	C/T	NA	INTERGENIC	NA
Kabuli_wild_40429621	1399942829	*Ca_kabuli_Chr07*	40429621	G/T	NA	INTERGENIC	NA
Kabuli_wild_40429629	1399942830	*Ca_kabuli_Chr07*	40429629	G/T	NA	INTERGENIC	NA
Kabuli_wild_40429643	1399942831	*Ca_kabuli_Chr07*	40429643	G/T	NA	INTERGENIC	NA
Kabuli_wild_40429660	1399942832	*Ca_kabuli_Chr07*	40429660	C/T	NA	INTERGENIC	NA
Kabuli_wild_40429661	1399942833	*Ca_kabuli_Chr07*	40429661	C/A	NA	INTERGENIC	NA
Kabuli_wild_40429686	1399942834	*Ca_kabuli_Chr07*	40429686	T/C	NA	INTERGENIC	NA
Kabuli_wild_40429702	1399942835	*Ca_kabuli_Chr07*	40429702	G/C	NA	INTERGENIC	NA
Kabuli_wild_40456574	1399942836	*Ca_kabuli_Chr07*	40456574	T/C	NA	INTERGENIC	NA
Kabuli_wild_40555051	1399942837	*Ca_kabuli_Chr07*	40555051	G/A	NA	INTERGENIC	NA
Kabuli_wild_40555070	1399942838	*Ca_kabuli_Chr07*	40555070	C/T	NA	INTERGENIC	NA
Kabuli_wild_40555075	1399942839	*Ca_kabuli_Chr07*	40555075	C/T	NA	INTERGENIC	NA
Kabuli_wild_40555092	1399942840	*Ca_kabuli_Chr07*	40555092	A/G	NA	INTERGENIC	NA
Kabuli_wild_40555099	1399942841	*Ca_kabuli_Chr07*	40555099	G/A	NA	INTERGENIC	NA
Kabuli_wild_40779909	1399942842	*Ca_kabuli_Chr07*	40779909	G/A	NA	INTERGENIC	NA
Kabuli_wild_40779925	1399942843	*Ca_kabuli_Chr07*	40779925	G/A	NA	INTERGENIC	NA
Kabuli_wild_40779928	1399942844	*Ca_kabuli_Chr07*	40779928	G/T	NA	INTERGENIC	NA
Kabuli_wild_40779934	1399942845	*Ca_kabuli_Chr07*	40779934	G/A	NA	INTERGENIC	NA
Kabuli_wild_40872829	1399942846	*Ca_kabuli_Chr07*	40872829	C/T	NA	INTERGENIC	NA
Kabuli_wild_40889561	1399942847	*Ca_kabuli_Chr07*	40889561	T/C	NA	INTERGENIC	NA

SNP IDs	NCBI dbSNP accession IDs (NCBI_SS#)	Chromosomes	Physical positions (bp)	SNPs	Gene accession IDs	Sequence components of genome	Putative functions
Kabuli_wild_40889575	1399942848	*Ca_kabuli_Chr07*	40889575	C/T	NA	INTERGENIC	NA
Kabuli_wild_40889596	1399942849	*Ca_kabuli_Chr07*	40889596	A/C	NA	INTERGENIC	NA
Kabuli_wild_40889598	1399942850	*Ca_kabuli_Chr07*	40889598	C/G	NA	INTERGENIC	NA
Kabuli_wild_40889632	1399942851	*Ca_kabuli_Chr07*	40889632	C/T	NA	INTERGENIC	NA
Kabuli_wild_40889633	1399942852	*Ca_kabuli_Chr07*	40889633	G/A	NA	INTERGENIC	NA
Kabuli_wild_40889638	1399942853	*Ca_kabuli_Chr07*	40889638	C/A	NA	INTERGENIC	NA
Kabuli_wild_40889640	1399942854	*Ca_kabuli_Chr07*	40889640	G/A	NA	INTERGENIC	NA
Kabuli_wild_40889641	1399942855	*Ca_kabuli_Chr07*	40889641	C/T	NA	INTERGENIC	NA
Kabuli_wild_40889643	1399942856	*Ca_kabuli_Chr07*	40889643	G/A	NA	INTERGENIC	NA
Kabuli_wild_40889644	1399942857	*Ca_kabuli_Chr07*	40889644	C/T	NA	INTERGENIC	NA
Kabuli_wild_40889646	1399942858	*Ca_kabuli_Chr07*	40889646	G/A	NA	INTERGENIC	NA
Kabuli_wild_40889667	1399942859	*Ca_kabuli_Chr07*	40889667	C/T	NA	INTERGENIC	NA
Kabuli_wild_40889670	1399942860	*Ca_kabuli_Chr07*	40889670	C/T	NA	INTERGENIC	NA
Kabuli_wild_40889673	1399942861	*Ca_kabuli_Chr07*	40889673	C/A	NA	INTERGENIC	NA
Kabuli_wild_40889684	1399942862	*Ca_kabuli_Chr07*	40889684	C/T	NA	INTERGENIC	NA
Kabuli_wild_40889723	1399942863	*Ca_kabuli_Chr07*	40889723	C/G	NA	INTERGENIC	NA
Kabuli_wild_41208211	1399942864	*Ca_kabuli_Chr07*	41208211	G/A	NA	INTERGENIC	NA
Kabuli_wild_41208258	1399942865	*Ca_kabuli_Chr07*	41208258	C/T	NA	INTERGENIC	NA
Kabuli_wild_41282883	1399942866	*Ca_kabuli_Chr07*	41282883	A/T	NA	INTERGENIC	NA
Kabuli_wild_41282957	1399942867	*Ca_kabuli_Chr07*	41282957	G/A	NA	INTERGENIC	NA
Kabuli_wild_41282960	1399942868	*Ca_kabuli_Chr07*	41282960	A/C	NA	INTERGENIC	NA
Kabuli_wild_41282961	1399942869	*Ca_kabuli_Chr07*	41282961	G/T	NA	INTERGENIC	NA
Kabuli_wild_41325886	1399942870	*Ca_kabuli_Chr07*	41325886	A/G	Gene_Ca_18002	SYNONYMOUS_CODING	Orotidine5'-phosphatedecarboxylasedomain
Kabuli_wild_41387475	1399942871	*Ca_kabuli_Chr07*	41387475	A/C	Gene_Ca_17996	NON_SYNONYMOUS_CODING	WD40 repeat
Kabuli_wild_41425049	1399942872	*Ca_kabuli_Chr07*	41425049	G/A	NA	INTERGENIC	NA
Kabuli_wild_41657180	1399942873	*Ca_kabuli_Chr07*	41657180	G/T	Gene_Ca_17988	INTRON	RNA polymerase,alpha subunit
Kabuli_wild_41657241	1399942874	*Ca_kabuli_Chr07*	41657241	T/C	Gene_Ca_17988	NON_SYNONYMOUS_CODING	RNA polymerase,alpha subunit
Kabuli_wild_41666809	1399942875	*Ca_kabuli_Chr07*	41666809	A/T	Gene_Ca_17988	NON_SYNONYMOUS_CODING	RNA polymerase,alpha subunit
Kabuli_wild_41821410	1399942876	*Ca_kabuli_Chr07*	41821410	T/C	NA	INTERGENIC	NA
Kabuli_wild_42011888	1399942877	*Ca_kabuli_Chr07*	42011888	A/C	Gene_Ca_17974	SYNONYMOUS_CODING	Ribosomerecyclingfactor
Kabuli_wild_42272939	1399942878	*Ca_kabuli_Chr07*	42272939	G/C	NA	INTERGENIC	NA
Kabuli_wild_42291250	1399942879	*Ca_kabuli_Chr07*	42291250	C/A	NA	INTERGENIC	NA
Kabuli_wild_42291274	1399942880	*Ca_kabuli_Chr07*	42291274	G/A	NA	INTERGENIC	NA
Kabuli_wild_42291291	1399942881	*Ca_kabuli_Chr07*	42291291	C/T	NA	INTERGENIC	NA
Kabuli_wild_42354938	1399942882	*Ca_kabuli_Chr07*	42354938	C/T	NA	INTERGENIC	NA
Kabuli_wild_42354946	1399942883	*Ca_kabuli_Chr07*	42354946	G/A	NA	INTERGENIC	NA
Kabuli_wild_42354999	1399942884	*Ca_kabuli_Chr07*	42354999	C/T	NA	INTERGENIC	NA
Kabuli_wild_42355002	1399942885	*Ca_kabuli_Chr07*	42355002	T/G	NA	INTERGENIC	NA
Kabuli_wild_42355011	1399942886	*Ca_kabuli_Chr07*	42355011	G/T	NA	INTERGENIC	NA
Kabuli_wild_42355315	1399942887	*Ca_kabuli_Chr07*	42355315	C/G	NA	INTERGENIC	NA
Kabuli_wild_42474465	1399942888	*Ca_kabuli_Chr07*	42474465	T/A	NA	INTERGENIC	NA
Kabuli_wild_42558871	1399942889	*Ca_kabuli_Chr07*	42558871	A/G	Gene_Ca_20213	DOWNSTREAM	Cytochrome P450
Kabuli_wild_42587321	1399942890	*Ca_kabuli_Chr07*	42587321	A/G	NA	INTERGENIC	NA
Kabuli_wild_42846800	1399942891	*Ca_kabuli_Chr07*	42846800	G/A	Gene_Ca_20198	NON_SYNONYMOUS_CODING	NA

SNP IDs	NCBI dbSNP accession IDs (NCBI_SS#)	Chromosomes	Physical positions (bp)	SNPs	Gene accession IDs	Sequence components of genome	Putative functions
Kabuli_wild_42846801	1399942892	*Ca_kabuli_Chr07*	42846801	C/A	Gene_Ca_20198	NON_SYNONYMOUS_CODING	NA
Kabuli_wild_42846803	1399942893	*Ca_kabuli_Chr07*	42846803	G/A	Gene_Ca_20198	NON_SYNONYMOUS_CODING	NA
Kabuli_wild_42846818	1399942894	*Ca_kabuli_Chr07*	42846818	C/T	Gene_Ca_20198	SYNONYMOUS_CODING	NA
Kabuli_wild_42846867	1399942895	*Ca_kabuli_Chr07*	42846867	G/A	Gene_Ca_20198	NON_SYNONYMOUS_CODING	NA
Kabuli_wild_42846868	1399942896	*Ca_kabuli_Chr07*	42846868	G/A	Gene_Ca_20198	SYNONYMOUS_CODING	NA
Kabuli_wild_42913317	1399942897	*Ca_kabuli_Chr07*	42913317	T/G	NA	INTERGENIC	NA
Kabuli_wild_42915851	1399942898	*Ca_kabuli_Chr07*	42915851	G/A	NA	INTERGENIC	NA
Kabuli_wild_42915928	1399942899	*Ca_kabuli_Chr07*	42915928	G/T	NA	INTERGENIC	NA
Kabuli_wild_43080710	1399942900	*Ca_kabuli_Chr07*	43080710	G/A	Gene_Ca_20188	INTRON	NA
Kabuli_wild_43210821	1399942901	*Ca_kabuli_Chr07*	43210821	A/T	NA	INTERGENIC	NA
Kabuli_wild_43583019	1399942902	*Ca_kabuli_Chr07*	43583019	A/C	NA	INTERGENIC	NA
Kabuli_wild_43997059	1399942903	*Ca_kabuli_Chr07*	43997059	G/A	NA	INTERGENIC	NA
Kabuli_wild_44232172	1399942904	*Ca_kabuli_Chr07*	44232172	G/A	Gene_Ca_21369	NON_SYNONYMOUS_CODING	NA
Kabuli_wild_44232194	1399942905	*Ca_kabuli_Chr07*	44232194	C/T	Gene_Ca_21369	NON_SYNONYMOUS_CODING	NA
Kabuli_wild_44232204	1399942906	*Ca_kabuli_Chr07*	44232204	T/A	Gene_Ca_21369	SYNONYMOUS_CODING	NA
Kabuli_wild_44232208	1399942907	*Ca_kabuli_Chr07*	44232208	G/A	Gene_Ca_21369	NON_SYNONYMOUS_CODING	NA
Kabuli_wild_44232221	1399942908	*Ca_kabuli_Chr07*	44232221	C/T	Gene_Ca_21369	NON_SYNONYMOUS_CODING	NA
Kabuli_wild_44232232	1399942909	*Ca_kabuli_Chr07*	44232232	C/T	Gene_Ca_21369	NON_SYNONYMOUS_CODING	NA
Kabuli_wild_44232239	1399942910	*Ca_kabuli_Chr07*	44232239	G/A	Gene_Ca_21369	SYNONYMOUS_CODING	NA
Kabuli_wild_44232266	1399942911	*Ca_kabuli_Chr07*	44232266	C/T	Gene_Ca_21369	NON_SYNONYMOUS_CODING	NA
Kabuli_wild_44339918	1399942912	*Ca_kabuli_Chr07*	44339918	A/G	NA	INTERGENIC	NA
Kabuli_wild_44446034	1399942913	*Ca_kabuli_Chr07*	44446034	T/C	NA	INTERGENIC	NA
Kabuli_wild_44540553	1399942914	*Ca_kabuli_Chr07*	44540553	T/G	NA	INTERGENIC	NA
Kabuli_wild_44540581	1399942915	*Ca_kabuli_Chr07*	44540581	C/A	NA	INTERGENIC	NA
Kabuli_wild_44540596	1399942916	*Ca_kabuli_Chr07*	44540596	C/A	NA	INTERGENIC	NA
Kabuli_wild_44540610	1399942917	*Ca_kabuli_Chr07*	44540610	G/A	NA	INTERGENIC	NA
Kabuli_wild_44540616	1399942918	*Ca_kabuli_Chr07*	44540616	C/A	NA	INTERGENIC	NA
Kabuli_wild_44540629	1399942919	*Ca_kabuli_Chr07*	44540629	G/A	NA	INTERGENIC	NA
Kabuli_wild_44612651	1399942920	*Ca_kabuli_Chr07*	44612651	C/A	Gene_Ca_15697	NON_SYNONYMOUS_CODING	Ethylenein sensitive3
Kabuli_wild_44728766	1399942921	*Ca_kabuli_Chr07*	44728766	G/T	Gene_Ca_15700	NON_SYNONYMOUS_CODING	Inositolpolyphosphate-relatedphosphatase
Kabuli_wild_44747668	1399942922	*Ca_kabuli_Chr07*	44747668	C/T	Gene_Ca_15701	SYNONYMOUS_CODING	NA
Kabuli_wild_44765450	1399942923	*Ca_kabuli_Chr07*	44765450	C/T	Gene_Ca_15703	SYNONYMOUS_CODING	Transposase,Ptta/En/Spm,plant
Kabuli_wild_44765522	1399942924	*Ca_kabuli_Chr07*	44765522	A/C	Gene_Ca_15703	SYNONYMOUS_CODING	Transposase,Ptta/En/Spm,plant
Kabuli_wild_44765582	1399942925	*Ca_kabuli_Chr07*	44765582	C/A	Gene_Ca_15703	NON_SYNONYMOUS_CODING	Transposase,Ptta/En/Spm,plant
Kabuli_wild_44877324	1399942926	*Ca_kabuli_Chr07*	44877324	A/C	Gene_Ca_15708	NON_SYNONYMOUS_CODING	Zinc finger,C2H2-type
Kabuli_wild_44926768	1399942927	*Ca_kabuli_Chr07*	44926768	C/T	NA	INTERGENIC	NA
Kabuli_wild_44926783	1399942928	*Ca_kabuli_Chr07*	44926783	C/A	NA	INTERGENIC	NA
Kabuli_wild_44938708	1399942929	*Ca_kabuli_Chr07*	44938708	T/G	Gene_Ca_15711	SYNONYMOUS_CODING	Pathogenesis-related transcriptional factor/ERF, DNA-binding
Kabuli_wild_45280659	1399942930	*Ca_kabuli_Chr07*	45280659	T/A	Gene_Ca_15727	UPSTREAM	Protein kinase,catalytic domain
Kabuli_wild_45300692	1399942931	*Ca_kabuli_Chr07*	45300692	A/G	Gene_Ca_15729	INTRON	Double-strandedRNA-binding
Kabuli_wild_45739326	1399942932	*Ca_kabuli_Chr07*	45739326	G/A	NA	INTERGENIC	NA
Kabuli_wild_45739341	1399942933	*Ca_kabuli_Chr07*	45739341	C/A	NA	INTERGENIC	NA
Kabuli_wild_45764010	1399942934	*Ca_kabuli_Chr07*	45764010	T/C	Gene_Ca_15751	INTRON	RibosomalproteinL5
Kabuli_wild_45936581	1399942935	*Ca_kabuli_Chr07*	45936581	C/A	Gene_Ca_15757	NON_SYNONYMOUS_CODING	Dedicatorofcytokinesis

SNP IDs	NCBI dbSNP accession IDs (NCBI_SS#)	Chromosomes	Physical positions (bp)	SNPs	Gene accession IDs	Sequence components of genome	Putative functions
Kabuli_wild_46053109	1399942936	*Ca_kabuli_Chr07*	46053109	G/A	Gene_Ca_15758	NON_SYNONYMOUS_CODING	Peptidase C48, SUMO/Sentrin/Ubl1
Kabuli_wild_46053116	1399942937	*Ca_kabuli_Chr07*	46053116	C/T	Gene_Ca_15758	NON_SYNONYMOUS_CODING	Peptidase C48, SUMO/Sentrin/Ubl1
Kabuli_wild_46053134	1399942938	*Ca_kabuli_Chr07*	46053134	G/T	Gene_Ca_15758	NON_SYNONYMOUS_CODING	Peptidase C48, SUMO/Sentrin/Ubl1
Kabuli_wild_46053176	1399942939	*Ca_kabuli_Chr07*	46053176	G/A	Gene_Ca_15758	SYNONYMOUS_CODING	Peptidase C48, SUMO/Sentrin/Ubl1
Kabuli_wild_46053193	1399942940	*Ca_kabuli_Chr07*	46053193	G/A	Gene_Ca_15758	NON_SYNONYMOUS_CODING	Peptidase C48, SUMO/Sentrin/Ubl1
Kabuli_wild_46485934	1399942941	*Ca_kabuli_Chr07*	46485934	A/G	Gene_Ca_22756	INTRON	NA
Kabuli_wild_46493344	1399942942	*Ca_kabuli_Chr07*	46493344	A/G	NA	INTERGENIC	NA
Kabuli_wild_46549964	1399942943	*Ca_kabuli_Chr07*	46549964	T/G	NA	INTERGENIC	NA
Kabuli_wild_46550010	1399942944	*Ca_kabuli_Chr07*	46550010	T/C	NA	INTERGENIC	NA
Kabuli_wild_46747255	1399942945	*Ca_kabuli_Chr07*	46747255	A/C	Gene_Ca_19471	DOWNSTREAM	Protein of unknown function DUF616
Kabuli_wild_46747283	1399942946	*Ca_kabuli_Chr07*	46747283	T/C	Gene_Ca_19471	DOWNSTREAM	Protein of unknown function DUF617
Kabuli_wild_46747290	1399942947	*Ca_kabuli_Chr07*	46747290	G/T	Gene_Ca_19471	DOWNSTREAM	Protein of unknown function DUF618
Kabuli_wild_46747295	1399942948	*Ca_kabuli_Chr07*	46747295	G/C	Gene_Ca_19471	DOWNSTREAM	Protein of unknown function DUF619
Kabuli_wild_46747309	1399942949	*Ca_kabuli_Chr07*	46747309	C/T	Gene_Ca_19471	DOWNSTREAM	Protein of unknown function DUF620
Kabuli_wild_46747330	1399942950	*Ca_kabuli_Chr07*	46747330	G/A	Gene_Ca_19471	DOWNSTREAM	Protein of unknown function DUF621
Kabuli_wild_46825952	1399942951	*Ca_kabuli_Chr07*	46825952	A/G	Gene_Ca_19475	SYNONYMOUS_CODING	Glycosidehydrolase,family18, catalytic domain
Kabuli_wild_46825954	1399942952	*Ca_kabuli_Chr07*	46825954	T/G	Gene_Ca_19475	NON_SYNONYMOUS_CODING	Glycosidehydrolase,family18, catalytic domain
Kabuli_wild_46825957	1399942953	*Ca_kabuli_Chr07*	46825957	A/T	Gene_Ca_19475	NON_SYNONYMOUS_CODING	Glycosidehydrolase,family18, catalytic domain
Kabuli_wild_46825985	1399942954	*Ca_kabuli_Chr07*	46825985	T/G	Gene_Ca_19475	SYNONYMOUS_CODING	Glycosidehydrolase,family18, catalytic domain
Kabuli_wild_46825992	1399942955	*Ca_kabuli_Chr07*	46825992	T/G	Gene_Ca_19475	NON_SYNONYMOUS_CODING	Glycosidehydrolase,family18, catalytic domain
Kabuli_wild_46825997	1399942956	*Ca_kabuli_Chr07*	46825997	A/G	Gene_Ca_19475	SYNONYMOUS_CODING	Glycosidehydrolase,family18, catalytic domain
Kabuli_wild_46826008	1399942957	*Ca_kabuli_Chr07*	46826008	A/T	Gene_Ca_19475	NON_SYNONYMOUS_CODING	Glycosidehydrolase,family18, catalytic domain
Kabuli_wild_46826015	1399942958	*Ca_kabuli_Chr07*	46826015	G/A	Gene_Ca_19475	SYNONYMOUS_CODING	Glycosidehydrolase,family18, catalytic domain
Kabuli_wild_46826020	1399942959	*Ca_kabuli_Chr07*	46826020	G/A	Gene_Ca_19475	NON_SYNONYMOUS_CODING	Glycosidehydrolase,family18, catalytic domain
Kabuli_wild_46826028	1399942960	*Ca_kabuli_Chr07*	46826028	C/A	Gene_Ca_19475	NON_SYNONYMOUS_CODING	Glycosidehydrolase,family18, catalytic domain
Kabuli_wild_46826030	1399942961	*Ca_kabuli_Chr07*	46826030	T/C	Gene_Ca_19475	SYNONYMOUS_CODING	Glycosidehydrolase,family18, catalytic domain
Kabuli_wild_46826036	1399942962	*Ca_kabuli_Chr07*	46826036	T/A	Gene_Ca_19475	SYNONYMOUS_CODING	Glycosidehydrolase,family18, catalytic domain
Kabuli_wild_46936006	1399942963	*Ca_kabuli_Chr07*	46936006	G/A	NA	INTERGENIC	NA
Kabuli_wild_46936053	1399942964	*Ca_kabuli_Chr07*	46936053	G/A	NA	INTERGENIC	NA
Kabuli_wild_47029851	1399942965	*Ca_kabuli_Chr07*	47029851	A/C	NA	INTERGENIC	NA
Kabuli_wild_47029867	1399942966	*Ca_kabuli_Chr07*	47029867	G/T	NA	INTERGENIC	NA
Kabuli_wild_47029885	1399942967	*Ca_kabuli_Chr07*	47029885	A/G	NA	INTERGENIC	NA
Kabuli_wild_47127331	1399942968	*Ca_kabuli_Chr07*	47127331	T/G	NA	INTERGENIC	NA
Kabuli_wild_47127342	1399942969	*Ca_kabuli_Chr07*	47127342	T/A	NA	INTERGENIC	NA
Kabuli_wild_47127343	1399942970	*Ca_kabuli_Chr07*	47127343	C/T	NA	INTERGENIC	NA
Kabuli_wild_47239763	1399942971	*Ca_kabuli_Chr07*	47239763	A/G	Gene_Ca_19492	SYNONYMOUS_CODING	Helix-turn-helixmotif,lambda-likerepressor
Kabuli_wild_47335916	1399942972	*Ca_kabuli_Chr07*	47335916	C/G	NA	INTERGENIC	NA
Kabuli_wild_47335940	1399942973	*Ca_kabuli_Chr07*	47335940	G/A	NA	INTERGENIC	NA
Kabuli_wild_47336169	1399942974	*Ca_kabuli_Chr07*	47336169	T/A	NA	INTERGENIC	NA
Kabuli_wild_47395492	1399942975	*Ca_kabuli_Chr07*	47395492	T/C	NA	INTERGENIC	NA
Kabuli_wild_47524131	1399942976	*Ca_kabuli_Chr07*	47524131	C/A	Gene_Ca_19499	NON_SYNONYMOUS_CODING	Protein kinase,catalytic domain
Kabuli_wild_47524173	1399942977	*Ca_kabuli_Chr07*	47524173	A/C	Gene_Ca_19499	NON_SYNONYMOUS_CODING	Protein kinase,catalytic domain
Kabuli_wild_47694706	1399942978	*Ca_kabuli_Chr07*	47694706	A/C	Gene_Ca_22539	DOWNSTREAM	NA
Kabuli_wild_47839952	1399942979	*Ca_kabuli_Chr07*	47839952	G/T	Gene_Ca_22546	NON_SYNONYMOUS_CODING	Ubiquitin-conjugating enzyme, E2

SNP IDs	NCBI dbSNP accession IDs (NCBI_SS#)	Chromosomes	Physical positions (bp)	SNPs	Gene accession IDs	Sequence components of genome	Putative functions
Kabuli_wild_47840092	1399942980	*Ca_kabuli_Chr07*	47840092	A/C	Gene_Ca_22546	SYNONYMOUS_CODING	Ubiquitin-conjugating enzyme, E2
Kabuli_wild_47986994	1399942981	*Ca_kabuli_Chr07*	47986994	T/C	NA	INTERGENIC	NA
Kabuli_wild_48109885	1399942982	*Ca_kabuli_Chr07*	48109885	G/T	NA	INTERGENIC	NA
Kabuli_wild_48200821	1399942983	*Ca_kabuli_Chr07*	48200821	C/A	NA	INTERGENIC	NA
Kabuli_wild_48200822	1399942984	*Ca_kabuli_Chr07*	48200822	G/A	NA	INTERGENIC	NA
Kabuli_wild_48200823	1399942985	*Ca_kabuli_Chr07*	48200823	G/A	NA	INTERGENIC	NA
Kabuli_wild_48200841	1399942986	*Ca_kabuli_Chr07*	48200841	T/C	NA	INTERGENIC	NA
Kabuli_wild_48200848	1399942987	*Ca_kabuli_Chr07*	48200848	T/C	NA	INTERGENIC	NA
Kabuli_wild_48200865	1399942988	*Ca_kabuli_Chr07*	48200865	A/G	NA	INTERGENIC	NA
Kabuli_wild_48461030	1399942989	*Ca_kabuli_Chr07*	48461030	A/G	NA	INTERGENIC	NA
Kabuli_wild_48785119	1399942990	*Ca_kabuli_Chr07*	48785119	C/T	NA	INTERGENIC	NA
Kabuli_wild_54095	1399942991	*Ca_kabuli_Chr08*	54095	A/C	Gene_Ca_11935	SYNONYMOUS_CODING	Heat shock protein DnaJ,N-terminal
Kabuli_wild_171259	1399942992	*Ca_kabuli_Chr08*	171259	T/C	NA	INTERGENIC	NA
Kabuli_wild_184467	1399942993	*Ca_kabuli_Chr08*	184467	A/G	Gene_Ca_11925	INTRON	Spc97/Spc98
Kabuli_wild_281622	1399942994	*Ca_kabuli_Chr08*	281622	G/A	Gene_Ca_11908	DOWNSTREAM	EGF-likecalcium-binding
Kabuli_wild_281630	1399942995	*Ca_kabuli_Chr08*	281630	A/C	Gene_Ca_11908	DOWNSTREAM	EGF-likecalcium-binding
Kabuli_wild_325107	1399942996	*Ca_kabuli_Chr08*	325107	T/A	NA	INTERGENIC	NA
Kabuli_wild_340712	1399942997	*Ca_kabuli_Chr08*	340712	C/T	NA	INTERGENIC	NA
Kabuli_wild_340827	1399942998	*Ca_kabuli_Chr08*	340827	T/C	NA	INTERGENIC	NA
Kabuli_wild_356295	1399942999	*Ca_kabuli_Chr08*	356295	A/T	Gene_Ca_11899	INTRON	HEAT
Kabuli_wild_356306	1399943000	*Ca_kabuli_Chr08*	356306	A/G	Gene_Ca_11899	INTRON	HEAT
Kabuli_wild_356308	1399943001	*Ca_kabuli_Chr08*	356308	G/A	Gene_Ca_11899	INTRON	HEAT
Kabuli_wild_499819	1399943002	*Ca_kabuli_Chr08*	499819	A/C	Gene_Ca_11887	INTRON	Protein phosphatase 2C, manganese/magnesium aspartate binding site
Kabuli_wild_560746	1399943003	*Ca_kabuli_Chr08*	560746	A/G	Gene_Ca_11882	INTRON	Cystathioninebeta-synthase,core
Kabuli_wild_566094	1399943004	*Ca_kabuli_Chr08*	566094	A/G	NA	INTERGENIC	NA
Kabuli_wild_614299	1399943005	*Ca_kabuli_Chr08*	614299	C/A	Gene_Ca_11874	DOWNSTREAM	NA
Kabuli_wild_614301	1399943006	*Ca_kabuli_Chr08*	614301	C/A	Gene_Ca_11874	DOWNSTREAM	NA
Kabuli_wild_614324	1399943007	*Ca_kabuli_Chr08*	614324	G/A	Gene_Ca_11874	DOWNSTREAM	NA
Kabuli_wild_614327	1399943008	*Ca_kabuli_Chr08*	614327	T/C	Gene_Ca_11874	DOWNSTREAM	NA
Kabuli_wild_619444	1399943009	*Ca_kabuli_Chr08*	619444	A/C	Gene_Ca_11873	UPSTREAM	Survival protein SurE-like phosphatase/nucleotidase
Kabuli_wild_619605	1399943010	*Ca_kabuli_Chr08*	619605	T/C	Gene_Ca_11873	UPSTREAM	Survival protein SurE-like phosphatase/nucleotidase
Kabuli_wild_634862	1399943011	*Ca_kabuli_Chr08*	634862	C/T	NA	INTERGENIC	NA
Kabuli_wild_649491	1399943012	*Ca_kabuli_Chr08*	649491	A/C	Gene_Ca_11866	SYNONYMOUS_CODING	Protein of unknown function DUF1423,plant
Kabuli_wild_660610	1399943013	*Ca_kabuli_Chr08*	660610	T/G	NA	INTERGENIC	NA
Kabuli_wild_666010	1399943014	*Ca_kabuli_Chr08*	666010	C/T	NA	INTERGENIC	NA
Kabuli_wild_666069	1399943015	*Ca_kabuli_Chr08*	666069	G/T	NA	INTERGENIC	NA
Kabuli_wild_666088	1399943016	*Ca_kabuli_Chr08*	666088	T/C	NA	INTERGENIC	NA
Kabuli_wild_666274	1399943017	*Ca_kabuli_Chr08*	666274	T/G	NA	INTERGENIC	NA
Kabuli_wild_666284	1399943018	*Ca_kabuli_Chr08*	666284	T/A	NA	INTERGENIC	NA
Kabuli_wild_666288	1399943019	*Ca_kabuli_Chr08*	666288	A/T	NA	INTERGENIC	NA
Kabuli_wild_666294	1399943020	*Ca_kabuli_Chr08*	666294	G/A	NA	INTERGENIC	NA
Kabuli_wild_673820	1399943021	*Ca_kabuli_Chr08*	673820	A/G	Gene_Ca_11861	NON_SYNONYMOUS_CODING	KIP1-like
Kabuli_wild_673893	1399943022	*Ca_kabuli_Chr08*	673893	A/C	Gene_Ca_11861	NON_SYNONYMOUS_CODING	KIP1-like
Kabuli_wild_701803	1399943023	*Ca_kabuli_Chr08*	701803	C/T	Gene_Ca_11858	INTRON	Protein kinase,catalytic domain

SNP IDs	NCBI dbSNP accession IDs (NCBI_SS#)	Chromosomes	Physical positions (bp)	SNPs	Gene accession IDs	Sequence components of genome	Putative functions
Kabuli_wild_701811	1399943024	*Ca_kabuli_Chr08*	701811	T/G	Gene_Ca_11858	INTRON	NA
Kabuli_wild_701837	1399943025	*Ca_kabuli_Chr08*	701837	G/A	Gene_Ca_11858	INTRON	NA
Kabuli_wild_706482	1399943026	*Ca_kabuli_Chr08*	706482	G/C	NA	INTERGENIC	NA
Kabuli_wild_706514	1399943027	*Ca_kabuli_Chr08*	706514	T/C	NA	INTERGENIC	NA
Kabuli_wild_706555	1399943028	*Ca_kabuli_Chr08*	706555	G/A	NA	INTERGENIC	NA
Kabuli_wild_706614	1399943029	*Ca_kabuli_Chr08*	706614	C/T	NA	INTERGENIC	NA
Kabuli_wild_706793	1399943030	*Ca_kabuli_Chr08*	706793	A/G	NA	INTERGENIC	NA
Kabuli_wild_706800	1399943031	*Ca_kabuli_Chr08*	706800	G/A	NA	INTERGENIC	NA
Kabuli_wild_707395	1399943032	*Ca_kabuli_Chr08*	707395	A/G	NA	INTERGENIC	NA
Kabuli_wild_709063	1399943033	*Ca_kabuli_Chr08*	709063	T/C	Gene_Ca_11857	DOWNSTREAM	gh
Kabuli_wild_709094	1399943034	*Ca_kabuli_Chr08*	709094	T/C	Gene_Ca_11857	DOWNSTREAM	Pentatrico peptide repeat
Kabuli_wild_709124	1399943035	*Ca_kabuli_Chr08*	709124	A/T	Gene_Ca_11857	DOWNSTREAM	Pentatrico peptide repeat
Kabuli_wild_714967	1399943036	*Ca_kabuli_Chr08*	714967	A/G	NA	INTERGENIC	NA
Kabuli_wild_714974	1399943037	*Ca_kabuli_Chr08*	714974	G/A	NA	INTERGENIC	NA
Kabuli_wild_822302	1399943038	*Ca_kabuli_Chr08*	822302	A/G	Gene_Ca_11839	NON_SYNONYMOUS_CODING	Zinc finger,PHD-type
Kabuli_wild_839786	1399943039	*Ca_kabuli_Chr08*	839786	C/T	NA	INTERGENIC	NA
Kabuli_wild_875236	1399943040	*Ca_kabuli_Chr08*	875236	T/G	Gene_Ca_11831	INTRON	PeptidaseM16,C-terminal
Kabuli_wild_894184	1399943041	*Ca_kabuli_Chr08*	894184	C/A	NA	INTERGENIC	NA
Kabuli_wild_897714	1399943042	*Ca_kabuli_Chr08*	897714	A/G	NA	INTERGENIC	NA
Kabuli_wild_898089	1399943043	*Ca_kabuli_Chr08*	898089	C/T	NA	INTERGENIC	NA
Kabuli_wild_924715	1399943044	*Ca_kabuli_Chr08*	924715	A/G	NA	INTERGENIC	NA
Kabuli_wild_938347	1399943045	*Ca_kabuli_Chr08*	938347	G/A	NA	INTERGENIC	NA
Kabuli_wild_938404	1399943046	*Ca_kabuli_Chr08*	938404	T/C	NA	INTERGENIC	NA
Kabuli_wild_938428	1399943047	*Ca_kabuli_Chr08*	938428	T/A	NA	INTERGENIC	NA
Kabuli_wild_957405	1399943048	*Ca_kabuli_Chr08*	957405	A/G	Gene_Ca_11821	SYNONYMOUS_CODING	Chalcone/stilbenesynthase,N-terminal
Kabuli_wild_957462	1399943049	*Ca_kabuli_Chr08*	957462	C/T	Gene_Ca_11821	SYNONYMOUS_CODING	Chalcone/stilbenesynthase,N-terminal
Kabuli_wild_957486	1399943050	*Ca_kabuli_Chr08*	957486	T/A	Gene_Ca_11821	SYNONYMOUS_CODING	Chalcone/stilbenesynthase,N-terminal
Kabuli_wild_1011320	1399943051	*Ca_kabuli_Chr08*	1011320	G/T	NA	INTERGENIC	NA
Kabuli_wild_1131429	1399943052	*Ca_kabuli_Chr08*	1131429	C/A	NA	INTERGENIC	NA
Kabuli_wild_1194886	1399943053	*Ca_kabuli_Chr08*	1194886	G/A	Gene_Ca_15060	INTRON	Alpha/betahydrolasefold-1
Kabuli_wild_1222064	1399943054	*Ca_kabuli_Chr08*	1222064	G/A	Gene_Ca_15058	DOWNSTREAM	De-etiolatedprotein1,Det1
Kabuli_wild_1241739	1399943055	*Ca_kabuli_Chr08*	1241739	C/T	NA	INTERGENIC	NA
Kabuli_wild_1362691	1399943056	*Ca_kabuli_Chr08*	1362691	T/A	NA	INTERGENIC	NA
Kabuli_wild_1530812	1399943057	*Ca_kabuli_Chr08*	1530812	A/G	Gene_Ca_15018	INTRON	ATPase,V1/A1complex,subunitE
Kabuli_wild_1534461	1399943058	*Ca_kabuli_Chr08*	1534461	A/C	NA	INTERGENIC	NA
Kabuli_wild_1540926	1399943059	*Ca_kabuli_Chr08*	1540926	A/G	Gene_Ca_15017	UPSTREAM	NA
Kabuli_wild_1544916	1399943060	*Ca_kabuli_Chr08*	1544916	C/T	Gene_Ca_15016	UPSTREAM	NA
Kabuli_wild_1573877	1399943061	*Ca_kabuli_Chr08*	1573877	G/T	Gene_Ca_15012	NON_SYNONYMOUS_CODING	ATPase,AAA+type,core
Kabuli_wild_1615234	1399943062	*Ca_kabuli_Chr08*	1615234	G/A	NA	INTERGENIC	NA
Kabuli_wild_1708860	1399943063	*Ca_kabuli_Chr08*	1708860	T/C	Gene_Ca_02436	SYNONYMOUS_CODING	NA
Kabuli_wild_1782034	1399943064	*Ca_kabuli_Chr08*	1782034	C/G	NA	INTERGENIC	NA
Kabuli_wild_1794755	1399943065	*Ca_kabuli_Chr08*	1794755	C/A	Gene_Ca_02426	NON_SYNONYMOUS_CODING	ATPase,P-type,K/Mg/Cd/Cu/Zn/Na/Ca/Na/H-transporter
Kabuli_wild_1794756	1399943066	*Ca_kabuli_Chr08*	1794756	G/T	Gene_Ca_02426	SYNONYMOUS_CODING	ATPase,P-type,K/Mg/Cd/Cu/Zn/Na/Ca/Na/H-transporter
Kabuli_wild_1794759	1399943067	*Ca_kabuli_Chr08*	1794759	C/T	Gene_Ca_02426	SYNONYMOUS_CODING	ATPase,P-type,K/Mg/Cd/Cu/Zn/Na/Ca/Na/H-transporter

SNP IDs	NCBI dbSNP accession IDs (NCBI_SS#)	Chromosomes	Physical positions (bp)	SNPs	Gene accession IDs	Sequence components of genome	Putative functions
Kabuli_wild_1817388	1399943068	*Ca_kabuli_Chr08*	1817388	T/C	NA	INTERGENIC	NA
Kabuli_wild_1817503	1399943069	*Ca_kabuli_Chr08*	1817503	T/C	NA	INTERGENIC	NA
Kabuli_wild_1846603	1399943070	*Ca_kabuli_Chr08*	1846603	T/C	Gene_Ca_02422	DOWNSTREAM	Remorin,C-terminal
Kabuli_wild_1854899	1399943071	*Ca_kabuli_Chr08*	1854899	C/T	Gene_Ca_02420	NON_SYNONYMOUS_CODING	Thaumatin,pathogenesis-related
Kabuli_wild_1932975	1399943072	*Ca_kabuli_Chr08*	1932975	T/C	NA	INTERGENIC	NA
Kabuli_wild_1932981	1399943073	*Ca_kabuli_Chr08*	1932981	T/C	NA	INTERGENIC	NA
Kabuli_wild_1932990	1399943074	*Ca_kabuli_Chr08*	1932990	A/C	NA	INTERGENIC	NA
Kabuli_wild_1932993	1399943075	*Ca_kabuli_Chr08*	1932993	C/T	NA	INTERGENIC	NA
Kabuli_wild_1932997	1399943076	*Ca_kabuli_Chr08*	1932997	G/T	NA	INTERGENIC	NA
Kabuli_wild_1933026	1399943077	*Ca_kabuli_Chr08*	1933026	G/A	NA	INTERGENIC	NA
Kabuli_wild_1933035	1399943078	*Ca_kabuli_Chr08*	1933035	T/A	NA	INTERGENIC	NA
Kabuli_wild_1933039	1399943079	*Ca_kabuli_Chr08*	1933039	G/T	NA	INTERGENIC	NA
Kabuli_wild_1933052	1399943080	*Ca_kabuli_Chr08*	1933052	T/C	NA	INTERGENIC	NA
Kabuli_wild_1933053	1399943081	*Ca_kabuli_Chr08*	1933053	G/A	NA	INTERGENIC	NA
Kabuli_wild_1933064	1399943082	*Ca_kabuli_Chr08*	1933064	G/C	NA	INTERGENIC	NA
Kabuli_wild_1989343	1399943083	*Ca_kabuli_Chr08*	1989343	G/A	NA	INTERGENIC	NA
Kabuli_wild_2028423	1399943084	*Ca_kabuli_Chr08*	2028423	G/T	Gene_Ca_02400	INTRON	Protein of unknown function DUF544
Kabuli_wild_2088892	1399943085	*Ca_kabuli_Chr08*	2088892	T/G	Gene_Ca_02391	NON_SYNONYMOUS_CODING	Cell cycle regulated microtubule associated protein
Kabuli_wild_2090852	1399943086	*Ca_kabuli_Chr08*	2090852	A/G	Gene_Ca_02390	UPSTREAM	1-aminocyclopropane-1-carboxylatesynthase
Kabuli_wild_2092669	1399943087	*Ca_kabuli_Chr08*	2092669	T/G	Gene_Ca_02389	DOWNSTREAM	Protein kinase,catalytic domain
Kabuli_wild_2095607	1399943088	*Ca_kabuli_Chr08*	2095607	A/G	NA	INTERGENIC	NA
Kabuli_wild_2190581	1399943089	*Ca_kabuli_Chr08*	2190581	C/T	Gene_Ca_02376	INTRON	Sec1-likeprotein
Kabuli_wild_2250178	1399943090	*Ca_kabuli_Chr08*	2250178	A/C	Gene_Ca_02366	UPSTREAM	Squalene/phytoenesynthase
Kabuli_wild_2306291	1399943091	*Ca_kabuli_Chr08*	2306291	A/G	NA	INTERGENIC	NA
Kabuli_wild_2417474	1399943092	*Ca_kabuli_Chr08*	2417474	A/T	Gene_Ca_02345	SYNONYMOUS_CODING	NA
Kabuli_wild_2482834	1399943093	*Ca_kabuli_Chr08*	2482834	A/G	NA	INTERGENIC	NA
Kabuli_wild_2515582	1399943094	*Ca_kabuli_Chr08*	2515582	A/T	Gene_Ca_02333	INTRON	DNA mismatch repair protein MutS,C-terminal domain
Kabuli_wild_2524148	1399943095	*Ca_kabuli_Chr08*	2524148	A/G	NA	INTERGENIC	NA
Kabuli_wild_2524188	1399943096	*Ca_kabuli_Chr08*	2524188	A/G	NA	INTERGENIC	NA
Kabuli_wild_2543156	1399943097	*Ca_kabuli_Chr08*	2543156	A/G	Gene_Ca_02330	NON_SYNONYMOUS_CODING	NA
Kabuli_wild_2604759	1399943098	*Ca_kabuli_Chr08*	2604759	C/T	Gene_Ca_02321	INTRON	Polycombprotein,VEFS-Box
Kabuli_wild_2621973	1399943099	*Ca_kabuli_Chr08*	2621973	T/G	Gene_Ca_02319	SYNONYMOUS_CODING	Monooxygenase,FAD-binding
Kabuli_wild_2722673	1399943100	*Ca_kabuli_Chr08*	2722673	C/A	NA	INTERGENIC	NA
Kabuli_wild_2827984	1399943101	*Ca_kabuli_Chr08*	2827984	T/C	Gene_Ca_02297	INTRON	Pleckstrinhomologydomain
Kabuli_wild_2838179	1399943102	*Ca_kabuli_Chr08*	2838179	T/A	Gene_Ca_02297	INTRON	Pleckstrinhomologydomain
Kabuli_wild_2871037	1399943103	*Ca_kabuli_Chr08*	2871037	C/A	Gene_Ca_02297	INTRON	Pleckstrinhomologydomain
Kabuli_wild_2871064	1399943104	*Ca_kabuli_Chr08*	2871064	G/A	Gene_Ca_02297	INTRON	Pleckstrinhomologydomain
Kabuli_wild_2871095	1399943105	*Ca_kabuli_Chr08*	2871095	G/A	Gene_Ca_02297	INTRON	Pleckstrinhomologydomain
Kabuli_wild_2888353	1399943106	*Ca_kabuli_Chr08*	2888353	G/T	Gene_Ca_02296	INTRON	PeptidaseS16,Lonprotease,C-terminal
Kabuli_wild_2943631	1399943107	*Ca_kabuli_Chr08*	2943631	G/T	NA	INTERGENIC	NA
Kabuli_wild_3041947	1399943108	*Ca_kabuli_Chr08*	3041947	A/G	Gene_Ca_02278	SYNONYMOUS_CODING	Protein of unknown function DUF947
Kabuli_wild_3050452	1399943109	*Ca_kabuli_Chr08*	3050452	T/C	NA	INTERGENIC	NA
Kabuli_wild_3171324	1399943110	*Ca_kabuli_Chr08*	3171324	C/T	NA	INTERGENIC	NA
Kabuli_wild_3179582	1399943111	*Ca_kabuli_Chr08*	3179582	G/A	NA	INTERGENIC	NA

SNP IDs	NCBI dbSNP accession IDs (NCBI_SS#)	Chromosomes	Physical positions (bp)	SNPs	Gene accession IDs	Sequence components of genome	Putative functions
Kabuli_wild_3179631	1399943112	*Ca_kabuli_Chr08*	3179631	A/T	NA	INTERGENIC	NA
Kabuli_wild_3250707	1399943113	*Ca_kabuli_Chr08*	3250707	C/T	NA	INTERGENIC	NA
Kabuli_wild_3250734	1399943114	*Ca_kabuli_Chr08*	3250734	G/T	NA	INTERGENIC	NA
Kabuli_wild_3269085	1399943115	*Ca_kabuli_Chr08*	3269085	A/C	Gene_Ca_02251	SYNONYMOUS_CODING	Homeobox
Kabuli_wild_3269088	1399943116	*Ca_kabuli_Chr08*	3269088	A/C	Gene_Ca_02251	SYNONYMOUS_CODING	Homeobox
Kabuli_wild_3297410	1399943117	*Ca_kabuli_Chr08*	3297410	T/C	Gene_Ca_02249	NON_SYNONYMOUS_CODING	SANT domain,DNA binding
Kabuli_wild_3394931	1399943118	*Ca_kabuli_Chr08*	3394931	T/C	Gene_Ca_02240	DOWNSTREAM	BSD
Kabuli_wild_3438361	1399943119	*Ca_kabuli_Chr08*	3438361	A/G	Gene_Ca_02235	INTRON	Hly-III-related
Kabuli_wild_3497315	1399943120	*Ca_kabuli_Chr08*	3497315	G/A	Gene_Ca_02229	INTRON	RasGTPase
Kabuli_wild_3539690	1399943121	*Ca_kabuli_Chr08*	3539690	A/G	Gene_Ca_02223	DOWNSTREAM	BTB/POZ-like
Kabuli_wild_3539745	1399943122	*Ca_kabuli_Chr08*	3539745	T/C	Gene_Ca_02223	DOWNSTREAM	BTB/POZ-like
Kabuli_wild_3568302	1399943123	*Ca_kabuli_Chr08*	3568302	C/T	NA	INTERGENIC	NA
Kabuli_wild_3730369	1399943124	*Ca_kabuli_Chr08*	3730369	A/C	Gene_Ca_02204	DOWNSTREAM	Protein of unknown function DUF791
Kabuli_wild_3837306	1399943125	*Ca_kabuli_Chr08*	3837306	G/C	NA	INTERGENIC	NA
Kabuli_wild_3837321	1399943126	*Ca_kabuli_Chr08*	3837321	G/T	NA	INTERGENIC	NA
Kabuli_wild_3951767	1399943127	*Ca_kabuli_Chr08*	3951767	C/A	NA	INTERGENIC	NA
Kabuli_wild_3951768	1399943128	*Ca_kabuli_Chr08*	3951768	A/G	NA	INTERGENIC	NA
Kabuli_wild_3954880	1399943129	*Ca_kabuli_Chr08*	3954880	A/T	NA	INTERGENIC	NA
Kabuli_wild_3954883	1399943130	*Ca_kabuli_Chr08*	3954883	G/A	NA	INTERGENIC	NA
Kabuli_wild_3954885	1399943131	*Ca_kabuli_Chr08*	3954885	A/G	NA	INTERGENIC	NA
Kabuli_wild_3954889	1399943132	*Ca_kabuli_Chr08*	3954889	T/A	NA	INTERGENIC	NA
Kabuli_wild_3954892	1399943133	*Ca_kabuli_Chr08*	3954892	A/C	NA	INTERGENIC	NA
Kabuli_wild_4023068	1399943134	*Ca_kabuli_Chr08*	4023068	A/G	Gene_Ca_02168	NON_SYNONYMOUS_CODING	Pathogenesis-related transcriptional factor/ERF, DNA-binding
Kabuli_wild_4023125	1399943135	*Ca_kabuli_Chr08*	4023125	G/A	Gene_Ca_02168	NON_SYNONYMOUS_CODING	Pathogenesis-related transcriptional factor/ERF, DNA-binding
Kabuli_wild_4042166	1399943136	*Ca_kabuli_Chr08*	4042166	A/C	NA	INTERGENIC	NA
Kabuli_wild_4042424	1399943137	*Ca_kabuli_Chr08*	4042424	G/A	NA	INTERGENIC	NA
Kabuli_wild_4073528	1399943138	*Ca_kabuli_Chr08*	4073528	C/T	Gene_Ca_02163	NON_SYNONYMOUS_CODING	Zinc finger, RING-type
Kabuli_wild_4073603	1399943139	*Ca_kabuli_Chr08*	4073603	A/G	Gene_Ca_02163	NON_SYNONYMOUS_CODING	Zinc finger, RING-type
Kabuli_wild_4092418	1399943140	*Ca_kabuli_Chr08*	4092418	T/G	Gene_Ca_02161	DOWNSTREAM	Nicotinate phosphoribosyltransferase-related
Kabuli_wild_4097729	1399943141	*Ca_kabuli_Chr08*	4097729	C/T	Gene_Ca_02160	NON_SYNONYMOUS_CODING	DNA-directedDNApolymerase,familyB,exonucleasedomain
Kabuli_wild_4106644	1399943142	*Ca_kabuli_Chr08*	4106644	C/T	NA	INTERGENIC	NA
Kabuli_wild_4172356	1399943143	*Ca_kabuli_Chr08*	4172356	G/C	Gene_Ca_02150	SYNONYMOUS_CODING	Fork head-associated (FHA) domain
Kabuli_wild_4172368	1399943144	*Ca_kabuli_Chr08*	4172368	T/C	Gene_Ca_02150	SYNONYMOUS_CODING	Fork head-associated (FHA) domain
Kabuli_wild_4198214	1399943145	*Ca_kabuli_Chr08*	4198214	A/C	Gene_Ca_02148	NON_SYNONYMOUS_CODING	Splicing factor, suppressor of whiteapricot
Kabuli_wild_4344459	1399943146	*Ca_kabuli_Chr08*	4344459	C/A	NA	INTERGENIC	NA
Kabuli_wild_4346749	1399943147	*Ca_kabuli_Chr08*	4346749	A/G	Gene_Ca_02131	DOWNSTREAM	NA
Kabuli_wild_4347373	1399943148	*Ca_kabuli_Chr08*	4347373	G/T	Gene_Ca_02131	DOWNSTREAM	NA
Kabuli_wild_4347535	1399943149	*Ca_kabuli_Chr08*	4347535	A/C	Gene_Ca_02131	DOWNSTREAM	NA
Kabuli_wild_4351751	1399943150	*Ca_kabuli_Chr08*	4351751	G/A	NA	INTERGENIC	NA
Kabuli_wild_4406956	1399943151	*Ca_kabuli_Chr08*	4406956	C/G	Gene_Ca_02125	SYNONYMOUS_CODING	Lateral organ boundaries, LOB
Kabuli_wild_4421618	1399943152	*Ca_kabuli_Chr08*	4421618	G/A	Gene_Ca_02124	NON_SYNONYMOUS_CODING	Lateral organ boundaries, LOB
Kabuli_wild_4430584	1399943153	*Ca_kabuli_Chr08*	4430584	C/A	Gene_Ca_02123	NON_SYNONYMOUS_CODING	Misato Segment II,myosin-like
Kabuli_wild_4484606	1399943154	*Ca_kabuli_Chr08*	4484606	T/A	Gene_Ca_02117	INTRON	Zinc finger,LIM-type
Kabuli_wild_4512311	1399943155	*Ca_kabuli_Chr08*	4512311	T/C	NA	INTERGENIC	NA

SNP IDs	NCBI dbSNP accession IDs (NCBI_SS#)	Chromosomes	Physical positions (bp)	SNPs	Gene accession IDs	Sequence components of genome	Putative functions
Kabuli_wild_4549690	1399943156	*Ca_kabuli_Chr08*	4549690	C/A	NA	INTERGENIC	NA
Kabuli_wild_4549691	1399943157	*Ca_kabuli_Chr08*	4549691	T/G	NA	INTERGENIC	NA
Kabuli_wild_4556064	1399943158	*Ca_kabuli_Chr08*	4556064	T/C	NA	INTERGENIC	NA
Kabuli_wild_4565771	1399943159	*Ca_kabuli_Chr08*	4565771	T/G	NA	INTERGENIC	NA
Kabuli_wild_4611094	1399943160	*Ca_kabuli_Chr08*	4611094	C/T	Gene_Ca_02096	NON_SYNONYMOUS_CODING	SET domain
Kabuli_wild_4614681	1399943161	*Ca_kabuli_Chr08*	4614681	G/T	Gene_Ca_02096	NON_SYNONYMOUS_CODING	SET domain
Kabuli_wild_4659781	1399943162	*Ca_kabuli_Chr08*	4659781	C/T	NA	INTERGENIC	NA
Kabuli_wild_4756983	1399943163	*Ca_kabuli_Chr08*	4756983	G/A	Gene_Ca_02082	UPSTREAM	Glycosyltransferase,family8
Kabuli_wild_4773205	1399943164	*Ca_kabuli_Chr08*	4773205	A/C	Gene_Ca_02080	UPSTREAM	Coiled-coildomaincontainingprotein109,C-terminal
Kabuli_wild_4858262	1399943165	*Ca_kabuli_Chr08*	4858262	C/A	Gene_Ca_02071	NON_SYNONYMOUS_CODING	NADPH-dependent FMN reductase
Kabuli_wild_4899982	1399943166	*Ca_kabuli_Chr08*	4899982	A/C	NA	INTERGENIC	NA
Kabuli_wild_4899983	1399943167	*Ca_kabuli_Chr08*	4899983	G/A	NA	INTERGENIC	NA
Kabuli_wild_4980916	1399943168	*Ca_kabuli_Chr08*	4980916	C/T	NA	INTERGENIC	NA
Kabuli_wild_4980949	1399943169	*Ca_kabuli_Chr08*	4980949	A/T	NA	INTERGENIC	NA
Kabuli_wild_5091091	1399943170	*Ca_kabuli_Chr08*	5091091	A/G	Gene_Ca_02046	DOWNSTREAM	Peptidase C48, SUMO/Sentrin/Ubl1
Kabuli_wild_5091109	1399943171	*Ca_kabuli_Chr08*	5091109	T/C	Gene_Ca_02046	DOWNSTREAM	Peptidase C48, SUMO/Sentrin/Ubl1
Kabuli_wild_5091127	1399943172	*Ca_kabuli_Chr08*	5091127	G/T	Gene_Ca_02046	DOWNSTREAM	Peptidase C48, SUMO/Sentrin/Ubl1
Kabuli_wild_5091141	1399943173	*Ca_kabuli_Chr08*	5091141	C/T	Gene_Ca_02046	DOWNSTREAM	Peptidase C48, SUMO/Sentrin/Ubl1
Kabuli_wild_5091174	1399943174	*Ca_kabuli_Chr08*	5091174	G/A	Gene_Ca_02046	DOWNSTREAM	Peptidase C48, SUMO/Sentrin/Ubl1
Kabuli_wild_5091183	1399943175	*Ca_kabuli_Chr08*	5091183	C/T	Gene_Ca_02046	DOWNSTREAM	Peptidase C48, SUMO/Sentrin/Ubl1
Kabuli_wild_5091192	1399943176	*Ca_kabuli_Chr08*	5091192	C/T	Gene_Ca_02046	DOWNSTREAM	Peptidase C48, SUMO/Sentrin/Ubl1
Kabuli_wild_5115522	1399943177	*Ca_kabuli_Chr08*	5115522	G/C	NA	INTERGENIC	NA
Kabuli_wild_5134706	1399943178	*Ca_kabuli_Chr08*	5134706	A/C	NA	INTERGENIC	NA
Kabuli_wild_5184227	1399943179	*Ca_kabuli_Chr08*	5184227	C/G	Gene_Ca_02036	NON_SYNONYMOUS_CODING	Sugar/inositol transporter
Kabuli_wild_5197004	1399943180	*Ca_kabuli_Chr08*	5197004	G/T	NA	INTERGENIC	NA
Kabuli_wild_5200592	1399943181	*Ca_kabuli_Chr08*	5200592	A/G	NA	INTERGENIC	NA
Kabuli_wild_5271566	1399943182	*Ca_kabuli_Chr08*	5271566	G/A	Gene_Ca_02025	INTRON	Cytidylyltransferase
Kabuli_wild_5313768	1399943183	*Ca_kabuli_Chr08*	5313768	T/C	NA	INTERGENIC	NA
Kabuli_wild_5389322	1399943184	*Ca_kabuli_Chr08*	5389322	A/G	NA	INTERGENIC	NA
Kabuli_wild_5390267	1399943185	*Ca_kabuli_Chr08*	5390267	T/C	NA	INTERGENIC	NA
Kabuli_wild_5390602	1399943186	*Ca_kabuli_Chr08*	5390602	T/C	NA	INTERGENIC	NA
Kabuli_wild_5399503	1399943187	*Ca_kabuli_Chr08*	5399503	G/A	NA	INTERGENIC	NA
Kabuli_wild_5400188	1399943188	*Ca_kabuli_Chr08*	5400188	A/G	NA	INTERGENIC	NA
Kabuli_wild_5412489	1399943189	*Ca_kabuli_Chr08*	5412489	T/C	Gene_Ca_02004	SYNONYMOUS_CODING	Protein of unknown function DUF702
Kabuli_wild_5470357	1399943190	*Ca_kabuli_Chr08*	5470357	T/A	NA	INTERGENIC	NA
Kabuli_wild_5487769	1399943191	*Ca_kabuli_Chr08*	5487769	G/A	Gene_Ca_01994	UPSTREAM	Transcription factor ,MADS-box
Kabuli_wild_5538683	1399943192	*Ca_kabuli_Chr08*	5538683	T/C	Gene_Ca_01989	INTRON	Protein of unknown function DUF726
Kabuli_wild_5538695	1399943193	*Ca_kabuli_Chr08*	5538695	T/C	Gene_Ca_01989	INTRON	Protein of unknown function DUF726
Kabuli_wild_5538696	1399943194	*Ca_kabuli_Chr08*	5538696	A/C	Gene_Ca_01989	INTRON	Protein of unknown function DUF726
Kabuli_wild_5712347	1399943195	*Ca_kabuli_Chr08*	5712347	A/T	Gene_Ca_01977	INTRON	Protein of unknown function DUF869,plant
Kabuli_wild_5712379	1399943196	*Ca_kabuli_Chr08*	5712379	A/T	Gene_Ca_01977	INTRON	Protein of unknown function DUF869,plant
Kabuli_wild_5712396	1399943197	*Ca_kabuli_Chr08*	5712396	A/G	Gene_Ca_01977	NON_SYNONYMOUS_CODING	Protein of unknown function DUF869,plant
Kabuli_wild_5870664	1399943198	*Ca_kabuli_Chr08*	5870664	T/C	Gene_Ca_01965	NON_SYNONYMOUS_CODING	Armadillo
Kabuli_wild_5935435	1399943199	*Ca_kabuli_Chr08*	5935435	A/C	Gene_Ca_01957	SYNONYMOUS_CODING	Senescence/spartin-associated

SNP IDs	NCBI dbSNP accession IDs (NCBI_SS#)	Chromosomes	Physical positions (bp)	SNPs	Gene accession IDs	Sequence components of genome	Putative functions
Kabuli_wild_6084527	1399943200	*Ca_kabuli_Chr08*	6084527	G/A	NA	INTERGENIC	NA
Kabuli_wild_6084528	1399943201	*Ca_kabuli_Chr08*	6084528	C/T	NA	INTERGENIC	NA
Kabuli_wild_6084592	1399943202	*Ca_kabuli_Chr08*	6084592	C/T	NA	INTERGENIC	NA
Kabuli_wild_6084594	1399943203	*Ca_kabuli_Chr08*	6084594	G/T	NA	INTERGENIC	NA
Kabuli_wild_6084611	1399943204	*Ca_kabuli_Chr08*	6084611	G/A	NA	INTERGENIC	NA
Kabuli_wild_6084612	1399943205	*Ca_kabuli_Chr08*	6084612	C/T	NA	INTERGENIC	NA
Kabuli_wild_6148862	1399943206	*Ca_kabuli_Chr08*	6148862	T/C	Gene_Ca_01932	SYNONYMOUS_CODING	AconitaseA/isopropyl malatedehydratase small subunit, swivel
Kabuli_wild_6192944	1399943207	*Ca_kabuli_Chr08*	6192944	T/G	Gene_Ca_01927	SYNONYMOUS_CODING	Pentatrico peptide repeat
Kabuli_wild_6193078	1399943208	*Ca_kabuli_Chr08*	6193078	G/A	Gene_Ca_01927	NON_SYNONYMOUS_CODING	Pentatrico peptide repeat
Kabuli_wild_6346867	1399943209	*Ca_kabuli_Chr08*	6346867	T/G	NA	INTERGENIC	NA
Kabuli_wild_6372230	1399943210	*Ca_kabuli_Chr08*	6372230	G/A	NA	INTERGENIC	NA
Kabuli_wild_6476249	1399943211	*Ca_kabuli_Chr08*	6476249	T/C	NA	INTERGENIC	NA
Kabuli_wild_6503885	1399943212	*Ca_kabuli_Chr08*	6503885	A/C	Gene_Ca_10738	STOP_GAINED	PolyApolymerase,head domain
Kabuli_wild_6504013	1399943213	*Ca_kabuli_Chr08*	6504013	A/G	Gene_Ca_10738	NON_SYNONYMOUS_CODING	PolyApolymerase,head domain
Kabuli_wild_6544218	1399943214	*Ca_kabuli_Chr08*	6544218	C/T	Gene_Ca_10735	SYNONYMOUS_CODING	Zinc finger,C2H2-type
Kabuli_wild_6600844	1399943215	*Ca_kabuli_Chr08*	6600844	C/A	Gene_Ca_10728	NON_SYNONYMOUS_CODING	RabGTPaseactivator
Kabuli_wild_6664509	1399943216	*Ca_kabuli_Chr08*	6664509	T/A	NA	INTERGENIC	NA
Kabuli_wild_6748294	1399943217	*Ca_kabuli_Chr08*	6748294	T/C	Gene_Ca_10708	NON_SYNONYMOUS_CODING	Sugar/inositol transporter
Kabuli_wild_6780592	1399943218	*Ca_kabuli_Chr08*	6780592	T/G	Gene_Ca_10705	NON_SYNONYMOUS_CODING	ATPase-like,ATP-binding domain
Kabuli_wild_6835452	1399943219	*Ca_kabuli_Chr08*	6835452	T/C	Gene_Ca_10703	INTRON	NA
Kabuli_wild_6838416	1399943220	*Ca_kabuli_Chr08*	6838416	C/T	Gene_Ca_10703	INTRON	NA
Kabuli_wild_6838449	1399943221	*Ca_kabuli_Chr08*	6838449	T/C	Gene_Ca_10703	INTRON	NA
Kabuli_wild_6838503	1399943222	*Ca_kabuli_Chr08*	6838503	C/T	Gene_Ca_10703	INTRON	NA
Kabuli_wild_6841931	1399943223	*Ca_kabuli_Chr08*	6841931	G/A	Gene_Ca_10703	INTRON	NA
Kabuli_wild_6841966	1399943224	*Ca_kabuli_Chr08*	6841966	C/T	Gene_Ca_10703	INTRON	NA
Kabuli_wild_6845342	1399943225	*Ca_kabuli_Chr08*	6845342	C/T	Gene_Ca_10703	INTRON	NA
Kabuli_wild_6845375	1399943226	*Ca_kabuli_Chr08*	6845375	T/C	Gene_Ca_10703	INTRON	NA
Kabuli_wild_6845394	1399943227	*Ca_kabuli_Chr08*	6845394	G/A	Gene_Ca_10703	INTRON	NA
Kabuli_wild_6845429	1399943228	*Ca_kabuli_Chr08*	6845429	C/T	Gene_Ca_10703	INTRON	NA
Kabuli_wild_6942651	1399943229	*Ca_kabuli_Chr08*	6942651	G/T	Gene_Ca_10698	INTRON	ARID/BRIGHTDNA-binding domain
Kabuli_wild_6987609	1399943230	*Ca_kabuli_Chr08*	6987609	G/T	Gene_Ca_10694	NON_SYNONYMOUS_CODING	PeptidaseS8/S53,subtilisin/kexin/sedolisin
Kabuli_wild_6987615	1399943231	*Ca_kabuli_Chr08*	6987615	A/C	Gene_Ca_10694	NON_SYNONYMOUS_CODING	PeptidaseS8/S53,subtilisin/kexin/sedolisin
Kabuli_wild_7013179	1399943232	*Ca_kabuli_Chr08*	7013179	G/A	Gene_Ca_10690	INTRON	MyTH4domain
Kabuli_wild_7041901	1399943233	*Ca_kabuli_Chr08*	7041901	A/C	NA	INTERGENIC	NA
Kabuli_wild_7162826	1399943234	*Ca_kabuli_Chr08*	7162826	T/G	NA	INTERGENIC	NA
Kabuli_wild_7174066	1399943235	*Ca_kabuli_Chr08*	7174066	C/T	Gene_Ca_10671	SYNONYMOUS_CODING	F-boxdomain,cyclin-like
Kabuli_wild_7175086	1399943236	*Ca_kabuli_Chr08*	7175086	A/C	Gene_Ca_10671	INTRON	F-boxdomain,cyclin-like
Kabuli_wild_7228599	1399943237	*Ca_kabuli_Chr08*	7228599	T/G	NA	INTERGENIC	NA
Kabuli_wild_7228623	1399943238	*Ca_kabuli_Chr08*	7228623	C/G	NA	INTERGENIC	NA
Kabuli_wild_7228637	1399943239	*Ca_kabuli_Chr08*	7228637	G/A	NA	INTERGENIC	NA
Kabuli_wild_7228638	1399943240	*Ca_kabuli_Chr08*	7228638	C/A	NA	INTERGENIC	NA
Kabuli_wild_7357583	1399943241	*Ca_kabuli_Chr08*	7357583	T/C	NA	INTERGENIC	NA
Kabuli_wild_7357621	1399943242	*Ca_kabuli_Chr08*	7357621	A/G	NA	INTERGENIC	NA
Kabuli_wild_7563370	1399943243	*Ca_kabuli_Chr08*	7563370	T/C	Gene_Ca_10631	DOWNSTREAM	NA

SNP IDs	NCBI dbSNP accession IDs (NCBI_SS#)	Chromosomes	Physical positions (bp)	SNPs	Gene accession IDs	Sequence components of genome	Putative functions
Kabuli_wild_7563373	1399943244	*Ca_kabuli_Chr08*	7563373	T/A	Gene_Ca_10631	DOWNSTREAM	NA
Kabuli_wild_7563379	1399943245	*Ca_kabuli_Chr08*	7563379	A/C	Gene_Ca_10631	DOWNSTREAM	NA
Kabuli_wild_7563385	1399943246	*Ca_kabuli_Chr08*	7563385	C/G	Gene_Ca_10631	DOWNSTREAM	NA
Kabuli_wild_7563405	1399943247	*Ca_kabuli_Chr08*	7563405	T/C	NA	INTERGENIC	NA
Kabuli_wild_7563629	1399943248	*Ca_kabuli_Chr08*	7563629	G/C	NA	INTERGENIC	NA
Kabuli_wild_7563641	1399943249	*Ca_kabuli_Chr08*	7563641	G/C	NA	INTERGENIC	NA
Kabuli_wild_7665291	1399943250	*Ca_kabuli_Chr08*	7665291	T/C	Gene_Ca_10625	DOWNSTREAM	NA
Kabuli_wild_7691607	1399943251	*Ca_kabuli_Chr08*	7691607	G/T	Gene_Ca_10621	DOWNSTREAM	NA
Kabuli_wild_7694746	1399943252	*Ca_kabuli_Chr08*	7694746	C/A	NA	INTERGENIC	NA
Kabuli_wild_7694747	1399943253	*Ca_kabuli_Chr08*	7694747	A/G	NA	INTERGENIC	NA
Kabuli_wild_7733573	1399943254	*Ca_kabuli_Chr08*	7733573	A/C	NA	INTERGENIC	NA
Kabuli_wild_7733574	1399943255	*Ca_kabuli_Chr08*	7733574	G/A	NA	INTERGENIC	NA
Kabuli_wild_7733647	1399943256	*Ca_kabuli_Chr08*	7733647	G/T	NA	INTERGENIC	NA
Kabuli_wild_7733670	1399943257	*Ca_kabuli_Chr08*	7733670	C/T	NA	INTERGENIC	NA
Kabuli_wild_7775908	1399943258	*Ca_kabuli_Chr08*	7775908	G/T	NA	INTERGENIC	NA
Kabuli_wild_7776014	1399943259	*Ca_kabuli_Chr08*	7776014	G/T	NA	INTERGENIC	NA
Kabuli_wild_7776018	1399943260	*Ca_kabuli_Chr08*	7776018	C/T	NA	INTERGENIC	NA
Kabuli_wild_7776041	1399943261	*Ca_kabuli_Chr08*	7776041	A/C	NA	INTERGENIC	NA
Kabuli_wild_7776042	1399943262	*Ca_kabuli_Chr08*	7776042	C/T	NA	INTERGENIC	NA
Kabuli_wild_7776466	1399943263	*Ca_kabuli_Chr08*	7776466	G/A	NA	INTERGENIC	NA
Kabuli_wild_7776592	1399943264	*Ca_kabuli_Chr08*	7776592	T/C	NA	INTERGENIC	NA
Kabuli_wild_7776897	1399943265	*Ca_kabuli_Chr08*	7776897	A/T	NA	INTERGENIC	NA
Kabuli_wild_7776934	1399943266	*Ca_kabuli_Chr08*	7776934	G/T	NA	INTERGENIC	NA
Kabuli_wild_7776970	1399943267	*Ca_kabuli_Chr08*	7776970	T/G	NA	INTERGENIC	NA
Kabuli_wild_7777023	1399943268	*Ca_kabuli_Chr08*	7777023	G/A	NA	INTERGENIC	NA
Kabuli_wild_8017452	1399943269	*Ca_kabuli_Chr08*	8017452	T/G	Gene_Ca_11439	NON_SYNONYMOUS_CODING	NA
Kabuli_wild_8017477	1399943270	*Ca_kabuli_Chr08*	8017477	T/G	Gene_Ca_11439	SYNONYMOUS_CODING	NA
Kabuli_wild_8017481	1399943271	*Ca_kabuli_Chr08*	8017481	A/G	Gene_Ca_11439	SYNONYMOUS_CODING	NA
Kabuli_wild_8017500	1399943272	*Ca_kabuli_Chr08*	8017500	C/T	Gene_Ca_11439	SYNONYMOUS_CODING	NA
Kabuli_wild_8017501	1399943273	*Ca_kabuli_Chr08*	8017501	G/A	Gene_Ca_11439	SYNONYMOUS_CODING	NA
Kabuli_wild_8066005	1399943274	*Ca_kabuli_Chr08*	8066005	A/G	NA	INTERGENIC	NA
Kabuli_wild_8066006	1399943275	*Ca_kabuli_Chr08*	8066006	C/A	NA	INTERGENIC	NA
Kabuli_wild_8104884	1399943276	*Ca_kabuli_Chr08*	8104884	T/A	NA	INTERGENIC	NA
Kabuli_wild_8194232	1399943277	*Ca_kabuli_Chr08*	8194232	C/A	Gene_Ca_11454	NON_SYNONYMOUS_CODING	Aspartyl/Asparaginyl-tRNAsynthetase,classIIb
Kabuli_wild_8194234	1399943278	*Ca_kabuli_Chr08*	8194234	C/A	Gene_Ca_11454	NON_SYNONYMOUS_CODING	Aspartyl/Asparaginyl-tRNAsynthetase,classIIb
Kabuli_wild_8389117	1399943279	*Ca_kabuli_Chr08*	8389117	C/T	Gene_Ca_11471	NON_SYNONYMOUS_CODING	Transcription factor jumonji/aspartylbeta-hydroxylase
Kabuli_wild_8452552	1399943280	*Ca_kabuli_Chr08*	8452552	A/G	NA	INTERGENIC	NA
Kabuli_wild_8458383	1399943281	*Ca_kabuli_Chr08*	8458383	G/A	NA	INTERGENIC	NA
Kabuli_wild_8509413	1399943282	*Ca_kabuli_Chr08*	8509413	G/A	Gene_Ca_11483	NON_SYNONYMOUS_CODING	RNA recognition motif domain
Kabuli_wild_8518968	1399943283	*Ca_kabuli_Chr08*	8518968	G/T	Gene_Ca_11484	INTRON	Target SNARE coiled-coil domain
Kabuli_wild_8533479	1399943284	*Ca_kabuli_Chr08*	8533479	G/T	Gene_Ca_11486	DOWNSTREAM	Protein kinase,catalytic domain
Kabuli_wild_8533978	1399943285	*Ca_kabuli_Chr08*	8533978	G/A	Gene_Ca_11486	DOWNSTREAM	Protein kinase,catalytic domain
Kabuli_wild_8560326	1399943286	*Ca_kabuli_Chr08*	8560326	G/T	Gene_Ca_11489	UPSTREAM	Zinc finger, RING-type
Kabuli_wild_8560330	1399943287	*Ca_kabuli_Chr08*	8560330	C/A	Gene_Ca_11489	UPSTREAM	Zinc finger, RING-type

SNP IDs	NCBI dbSNP accession IDs (NCBI_SS#)	Chromosomes	Physical positions (bp)	SNPs	Gene accession IDs	Sequence components of genome	Putative functions
Kabuli_wild_8560331	1399943288	*Ca_kabuli_Chr08*	8560331	C/G	Gene_Ca_11489	UPSTREAM	Zinc finger, RING-type
Kabuli_wild_8560332	1399943289	*Ca_kabuli_Chr08*	8560332	A/C	Gene_Ca_11489	UPSTREAM	Zinc finger, RING-type
Kabuli_wild_8650188	1399943290	*Ca_kabuli_Chr08*	8650188	T/G	Gene_Ca_11495	SYNONYMOUS_CODING	Aminotransferase-like,plant mobile domain
Kabuli_wild_8661327	1399943291	*Ca_kabuli_Chr08*	8661327	T/C	Gene_Ca_11496	DOWNSTREAM	SNARE associated Golgi protein
Kabuli_wild_8670242	1399943292	*Ca_kabuli_Chr08*	8670242	T/G	Gene_Ca_11498	NON_SYNONYMOUS_CODING	Tetra tricopeptide repeat-containing
Kabuli_wild_8772118	1399943293	*Ca_kabuli_Chr08*	8772118	G/A	NA	INTERGENIC	NA
Kabuli_wild_8772124	1399943294	*Ca_kabuli_Chr08*	8772124	G/T	NA	INTERGENIC	NA
Kabuli_wild_8772131	1399943295	*Ca_kabuli_Chr08*	8772131	C/G	NA	INTERGENIC	NA
Kabuli_wild_8842371	1399943296	*Ca_kabuli_Chr08*	8842371	A/G	Gene_Ca_11514	NON_SYNONYMOUS_CODING	Helix-loop-helixDNA-binding domain
Kabuli_wild_8843831	1399943297	*Ca_kabuli_Chr08*	8843831	T/C	Gene_Ca_11514	DOWNSTREAM	Helix-loop-helixDNA-binding domain
Kabuli_wild_8853602	1399943298	*Ca_kabuli_Chr08*	8853602	G/A	NA	INTERGENIC	NA
Kabuli_wild_8853664	1399943299	*Ca_kabuli_Chr08*	8853664	A/G	NA	INTERGENIC	NA
Kabuli_wild_8856046	1399943300	*Ca_kabuli_Chr08*	8856046	C/T	NA	INTERGENIC	NA
Kabuli_wild_8856047	1399943301	*Ca_kabuli_Chr08*	8856047	C/T	NA	INTERGENIC	NA
Kabuli_wild_8856143	1399943302	*Ca_kabuli_Chr08*	8856143	G/A	NA	INTERGENIC	NA
Kabuli_wild_8856150	1399943303	*Ca_kabuli_Chr08*	8856150	A/G	NA	INTERGENIC	NA
Kabuli_wild_8864079	1399943304	*Ca_kabuli_Chr08*	8864079	C/T	Gene_Ca_11517	SYNONYMOUS_CODING	VQ
Kabuli_wild_8864157	1399943305	*Ca_kabuli_Chr08*	8864157	G/A	Gene_Ca_11517	SYNONYMOUS_CODING	VQ
Kabuli_wild_8888412	1399943306	*Ca_kabuli_Chr08*	8888412	A/G	NA	INTERGENIC	NA
Kabuli_wild_8888414	1399943307	*Ca_kabuli_Chr08*	8888414	G/T	NA	INTERGENIC	NA
Kabuli_wild_8888426	1399943308	*Ca_kabuli_Chr08*	8888426	T/C	NA	INTERGENIC	NA
Kabuli_wild_8888476	1399943309	*Ca_kabuli_Chr08*	8888476	A/C	NA	INTERGENIC	NA
Kabuli_wild_8888542	1399943310	*Ca_kabuli_Chr08*	8888542	T/C	NA	INTERGENIC	NA
Kabuli_wild_8903053	1399943311	*Ca_kabuli_Chr08*	8903053	T/C	Gene_Ca_11521	INTRON	Major facilitator superfamily MFS-1
Kabuli_wild_8904346	1399943312	*Ca_kabuli_Chr08*	8904346	C/A	Gene_Ca_11521	INTRON	Major facilitator superfamily MFS-1
Kabuli_wild_8904366	1399943313	*Ca_kabuli_Chr08*	8904366	A/G	Gene_Ca_11521	INTRON	Major facilitator superfamily MFS-1
Kabuli_wild_8911129	1399943314	*Ca_kabuli_Chr08*	8911129	G/A	Gene_Ca_11522	DOWNSTREAM	Starchsynthase,catalytic domain
Kabuli_wild_8911466	1399943315	*Ca_kabuli_Chr08*	8911466	A/G	Gene_Ca_11522	NON_SYNONYMOUS_CODING	Starchsynthase,catalytic domain
Kabuli_wild_8911478	1399943316	*Ca_kabuli_Chr08*	8911478	G/A	Gene_Ca_11522	SYNONYMOUS_CODING	Starchsynthase,catalytic domain
Kabuli_wild_8914018	1399943317	*Ca_kabuli_Chr08*	8914018	A/C	Gene_Ca_11522	INTRON	Starchsynthase,catalytic domain
Kabuli_wild_8932493	1399943318	*Ca_kabuli_Chr08*	8932493	A/C	Gene_Ca_11524	INTRON	Phosphoenolpyruvatecarboxykinase,ATP-utilising
Kabuli_wild_9097271	1399943319	*Ca_kabuli_Chr08*	9097271	T/A	Gene_Ca_11532	UPSTREAM	NA
Kabuli_wild_9097297	1399943320	*Ca_kabuli_Chr08*	9097297	A/C	Gene_Ca_11532	UPSTREAM	NA
Kabuli_wild_9097300	1399943321	*Ca_kabuli_Chr08*	9097300	C/G	Gene_Ca_11532	UPSTREAM	NA
Kabuli_wild_9097585	1399943322	*Ca_kabuli_Chr08*	9097585	A/C	NA	INTERGENIC	NA
Kabuli_wild_9099798	1399943323	*Ca_kabuli_Chr08*	9099798	G/T	NA	INTERGENIC	NA
Kabuli_wild_9100111	1399943324	*Ca_kabuli_Chr08*	9100111	C/T	NA	INTERGENIC	NA
Kabuli_wild_9100522	1399943325	*Ca_kabuli_Chr08*	9100522	G/A	Gene_Ca_11533	SYNONYMOUS_CODING	Glycosi dehydrolase,family 3, N-terminal
Kabuli_wild_9109896	1399943326	*Ca_kabuli_Chr08*	9109896	A/T	NA	INTERGENIC	NA
Kabuli_wild_9109907	1399943327	*Ca_kabuli_Chr08*	9109907	A/T	NA	INTERGENIC	NA
Kabuli_wild_9109920	1399943328	*Ca_kabuli_Chr08*	9109920	C/A	NA	INTERGENIC	NA
Kabuli_wild_9109924	1399943329	*Ca_kabuli_Chr08*	9109924	T/A	NA	INTERGENIC	NA
Kabuli_wild_9112172	1399943330	*Ca_kabuli_Chr08*	9112172	A/C	Gene_Ca_11534	SYNONYMOUS_CODING	Protein of unknown function DUF1399
Kabuli_wild_9112178	1399943331	*Ca_kabuli_Chr08*	9112178	G/A	Gene_Ca_11534	SYNONYMOUS_CODING	Protein of unknown function DUF1399

SNP IDs	NCBI dbSNP accession IDs (NCBI_SS#)	Chromosomes	Physical positions (bp)	SNPs	Gene accession IDs	Sequence components of genome	Putative functions
Kabuli_wild_9112181	1399943332	*Ca_kabuli_Chr08*	9112181	A/C	Gene_Ca_11534	SYNONYMOUS_CODING	Protein of unknown function DUF1399
Kabuli_wild_9115081	1399943333	*Ca_kabuli_Chr08*	9115081	A/G	NA	INTERGENIC	NA
Kabuli_wild_9115121	1399943334	*Ca_kabuli_Chr08*	9115121	T/G	NA	INTERGENIC	NA
Kabuli_wild_9118255	1399943335	*Ca_kabuli_Chr08*	9118255	A/C	Gene_Ca_11535	DOWNSTREAM	NA
Kabuli_wild_9118582	1399943336	*Ca_kabuli_Chr08*	9118582	C/T	Gene_Ca_11534	UPSTREAM	Protein of unknown function DUF1399
Kabuli_wild_9118667	1399943337	*Ca_kabuli_Chr08*	9118667	A/G	Gene_Ca_11534	UPSTREAM	Protein of unknown function DUF1399
Kabuli_wild_9143840	1399943338	*Ca_kabuli_Chr08*	9143840	C/A	NA	INTERGENIC	NA
Kabuli_wild_9151722	1399943339	*Ca_kabuli_Chr08*	9151722	G/T	Gene_Ca_11539	SYNONYMOUS_CODING	PeptidaseM3A/M3B,thimet/oligopeptidaseF
Kabuli_wild_9151805	1399943340	*Ca_kabuli_Chr08*	9151805	A/G	Gene_Ca_11539	INTRON	PeptidaseM3A/M3B,thimet/oligopeptidaseF
Kabuli_wild_9151855	1399943341	*Ca_kabuli_Chr08*	9151855	A/T	Gene_Ca_11539	INTRON	PeptidaseM3A/M3B,thimet/oligopeptidaseF
Kabuli_wild_9386682	1399943342	*Ca_kabuli_Chr08*	9386682	C/G	Gene_Ca_11559	NON_SYNONYMOUS_CODING	Initiation factor2B-related
Kabuli_wild_9388614	1399943343	*Ca_kabuli_Chr08*	9388614	A/C	Gene_Ca_11559	UPSTREAM	Initiation factor2B-related
Kabuli_wild_9388734	1399943344	*Ca_kabuli_Chr08*	9388734	T/A	Gene_Ca_11559	UPSTREAM	Initiation factor2B-related
Kabuli_wild_9389347	1399943345	*Ca_kabuli_Chr08*	9389347	G/A	Gene_Ca_11559	UPSTREAM	Initiation factor2B-related
Kabuli_wild_10004481	1399943346	*Ca_kabuli_Chr08*	10004481	T/C	Gene_Ca_18400	DOWNSTREAM	NA
Kabuli_wild_10022825	1399943347	*Ca_kabuli_Chr08*	10022825	G/A	NA	INTERGENIC	NA
Kabuli_wild_10022829	1399943348	*Ca_kabuli_Chr08*	10022829	T/G	NA	INTERGENIC	NA
Kabuli_wild_10022868	1399943349	*Ca_kabuli_Chr08*	10022868	C/A	NA	INTERGENIC	NA
Kabuli_wild_10031818	1399943350	*Ca_kabuli_Chr08*	10031818	A/C	NA	INTERGENIC	NA
Kabuli_wild_10155981	1399943351	*Ca_kabuli_Chr08*	10155981	A/C	Gene_Ca_18411	SYNONYMOUS_CODING	NADH:flavinoxidoreductase/NADHoxidase,N-terminal
Kabuli_wild_10257655	1399943352	*Ca_kabuli_Chr08*	10257655	T/G	Gene_Ca_18416	SYNONYMOUS_CODING	Zinc finger,PHD-type
Kabuli_wild_10258892	1399943353	*Ca_kabuli_Chr08*	10258892	G/T	Gene_Ca_18416	NON_SYNONYMOUS_CODING	Zinc finger,PHD-type
Kabuli_wild_10544361	1399943354	*Ca_kabuli_Chr08*	10544361	G/A	Gene_Ca_18436	INTRON	ChaperoninTCP-1,conservedsite
Kabuli_wild_10668346	1399943355	*Ca_kabuli_Chr08*	10668346	A/C	NA	INTERGENIC	NA
Kabuli_wild_10668405	1399943356	*Ca_kabuli_Chr08*	10668405	C/T	NA	INTERGENIC	NA
Kabuli_wild_10668422	1399943357	*Ca_kabuli_Chr08*	10668422	A/C	NA	INTERGENIC	NA
Kabuli_wild_10728226	1399943358	*Ca_kabuli_Chr08*	10728226	T/A	NA	INTERGENIC	NA
Kabuli_wild_10733404	1399943359	*Ca_kabuli_Chr08*	10733404	A/G	Gene_Ca_19724	SYNONYMOUS_CODING	Ankyrinrepeat
Kabuli_wild_10733503	1399943360	*Ca_kabuli_Chr08*	10733503	C/T	Gene_Ca_19724	SYNONYMOUS_CODING	Ankyrinrepeat
Kabuli_wild_10733661	1399943361	*Ca_kabuli_Chr08*	10733661	A/G	Gene_Ca_19724	NON_SYNONYMOUS_CODING	Ankyrinrepeat
Kabuli_wild_10733747	1399943362	*Ca_kabuli_Chr08*	10733747	C/T	Gene_Ca_19724	NON_SYNONYMOUS_CODING	Ankyrinrepeat
Kabuli_wild_10763656	1399943363	*Ca_kabuli_Chr08*	10763656	A/T	NA	INTERGENIC	NA
Kabuli_wild_10953767	1399943364	*Ca_kabuli_Chr08*	10953767	C/T	NA	INTERGENIC	NA
Kabuli_wild_10963827	1399943365	*Ca_kabuli_Chr08*	10963827	T/A	NA	INTERGENIC	NA
Kabuli_wild_10963856	1399943366	*Ca_kabuli_Chr08*	10963856	T/G	NA	INTERGENIC	NA
Kabuli_wild_11015666	1399943367	*Ca_kabuli_Chr08*	11015666	G/C	NA	INTERGENIC	NA
Kabuli_wild_11015678	1399943368	*Ca_kabuli_Chr08*	11015678	C/G	NA	INTERGENIC	NA
Kabuli_wild_11015680	1399943369	*Ca_kabuli_Chr08*	11015680	G/T	NA	INTERGENIC	NA
Kabuli_wild_11066644	1399943370	*Ca_kabuli_Chr08*	11066644	C/T	Gene_Ca_16843	SYNONYMOUS_CODING	Multiantimicrobial extrusion protein
Kabuli_wild_11066728	1399943371	*Ca_kabuli_Chr08*	11066728	G/A	Gene_Ca_16843	INTRON	Multiantimicrobial extrusion protein
Kabuli_wild_11066975	1399943372	*Ca_kabuli_Chr08*	11066975	T/C	Gene_Ca_16843	INTRON	Multiantimicrobial extrusion protein
Kabuli_wild_11067014	1399943373	*Ca_kabuli_Chr08*	11067014	G/A	Gene_Ca_16843	INTRON	Multiantimicrobial extrusion protein
Kabuli_wild_11067090	1399943374	*Ca_kabuli_Chr08*	11067090	T/C	Gene_Ca_16843	SYNONYMOUS_CODING	Multiantimicrobial extrusion protein
Kabuli_wild_11067096	1399943375	*Ca_kabuli_Chr08*	11067096	T/G	Gene_Ca_16843	SYNONYMOUS_CODING	Multiantimicrobial extrusion protein

SNP IDs	NCBI dbSNP accession IDs (NCBI_SS#)	Chromosomes	Physical positions (bp)	SNPs	Gene accession IDs	Sequence components of genome	Putative functions
Kabuli_wild_11087123	1399943376	*Ca_kabuli_Chr08*	11087123	C/T	NA	INTERGENIC	NA
Kabuli_wild_11087143	1399943377	*Ca_kabuli_Chr08*	11087143	A/G	NA	INTERGENIC	NA
Kabuli_wild_11087247	1399943378	*Ca_kabuli_Chr08*	11087247	T/C	NA	INTERGENIC	NA
Kabuli_wild_11087250	1399943379	*Ca_kabuli_Chr08*	11087250	T/C	NA	INTERGENIC	NA
Kabuli_wild_11087302	1399943380	*Ca_kabuli_Chr08*	11087302	A/T	NA	INTERGENIC	NA
Kabuli_wild_11102884	1399943381	*Ca_kabuli_Chr08*	11102884	T/G	Gene_Ca_16842	INTRON	Protein kinase,catalytic domain
Kabuli_wild_11102892	1399943382	*Ca_kabuli_Chr08*	11102892	T/A	Gene_Ca_16842	INTRON	Protein kinase,catalytic domain
Kabuli_wild_11186986	1399943383	*Ca_kabuli_Chr08*	11186986	A/C	Gene_Ca_16839	SYNONYMOUS_CODING	Zinc finger,RanBP2-type
Kabuli_wild_11231812	1399943384	*Ca_kabuli_Chr08*	11231812	A/G	Gene_Ca_16835	SYNONYMOUS_CODING	Protein kinase,catalytic domain
Kabuli_wild_11240258	1399943385	*Ca_kabuli_Chr08*	11240258	C/A	NA	INTERGENIC	NA
Kabuli_wild_11240598	1399943386	*Ca_kabuli_Chr08*	11240598	A/G	Gene_Ca_16834	NON_SYNONYMOUS_CODING	HistoneH1/H5
Kabuli_wild_11243256	1399943387	*Ca_kabuli_Chr08*	11243256	A/C	Gene_Ca_16834	SYNONYMOUS_CODING	HistoneH1/H6
Kabuli_wild_11243260	1399943388	*Ca_kabuli_Chr08*	11243260	T/C	Gene_Ca_16834	NON_SYNONYMOUS_CODING	HistoneH1/H7
Kabuli_wild_11243263	1399943389	*Ca_kabuli_Chr08*	11243263	A/C	Gene_Ca_16834	NON_SYNONYMOUS_CODING	HistoneH1/H8
Kabuli_wild_11266447	1399943390	*Ca_kabuli_Chr08*	11266447	G/A	NA	INTERGENIC	NA
Kabuli_wild_11310418	1399943391	*Ca_kabuli_Chr08*	11310418	G/A	Gene_Ca_16827	DOWNSTREAM	Pentatrico peptide repeat
Kabuli_wild_11310460	1399943392	*Ca_kabuli_Chr08*	11310460	G/A	Gene_Ca_16827	DOWNSTREAM	Pentatrico peptide repeat
Kabuli_wild_11310480	1399943393	*Ca_kabuli_Chr08*	11310480	T/A	Gene_Ca_16827	DOWNSTREAM	Pentatrico peptide repeat
Kabuli_wild_11332934	1399943394	*Ca_kabuli_Chr08*	11332934	C/T	NA	INTERGENIC	NA
Kabuli_wild_11332940	1399943395	*Ca_kabuli_Chr08*	11332940	T/C	NA	INTERGENIC	NA
Kabuli_wild_11333057	1399943396	*Ca_kabuli_Chr08*	11333057	C/G	NA	INTERGENIC	NA
Kabuli_wild_11334190	1399943397	*Ca_kabuli_Chr08*	11334190	A/T	NA	INTERGENIC	NA
Kabuli_wild_11334210	1399943398	*Ca_kabuli_Chr08*	11334210	C/T	NA	INTERGENIC	NA
Kabuli_wild_11349891	1399943399	*Ca_kabuli_Chr08*	11349891	T/A	NA	INTERGENIC	NA
Kabuli_wild_11349995	1399943400	*Ca_kabuli_Chr08*	11349995	A/G	NA	INTERGENIC	NA
Kabuli_wild_11403754	1399943401	*Ca_kabuli_Chr08*	11403754	G/A	Gene_Ca_16820	NON_SYNONYMOUS_CODING	SANT domain,DNA binding
Kabuli_wild_11403805	1399943402	*Ca_kabuli_Chr08*	11403805	G/A	Gene_Ca_16820	SYNONYMOUS_CODING	SANT domain,DNA binding
Kabuli_wild_11562814	1399943403	*Ca_kabuli_Chr08*	11562814	A/C	NA	INTERGENIC	NA
Kabuli_wild_11631836	1399943404	*Ca_kabuli_Chr08*	11631836	C/T	Gene_Ca_16809	NON_SYNONYMOUS_CODING	Ketose-bisphosphatealdolase,class-II
Kabuli_wild_11656670	1399943405	*Ca_kabuli_Chr08*	11656670	A/G	NA	INTERGENIC	NA
Kabuli_wild_11656675	1399943406	*Ca_kabuli_Chr08*	11656675	T/C	NA	INTERGENIC	NA
Kabuli_wild_11656687	1399943407	*Ca_kabuli_Chr08*	11656687	A/G	NA	INTERGENIC	NA
Kabuli_wild_11656700	1399943408	*Ca_kabuli_Chr08*	11656700	G/T	NA	INTERGENIC	NA
Kabuli_wild_11694220	1399943409	*Ca_kabuli_Chr08*	11694220	A/G	NA	INTERGENIC	NA
Kabuli_wild_11695661	1399943410	*Ca_kabuli_Chr08*	11695661	T/C	Gene_Ca_16803	SYNONYMOUS_CODING	Protein kinase,catalytic domain
Kabuli_wild_11702005	1399943411	*Ca_kabuli_Chr08*	11702005	A/C	Gene_Ca_16803	INTRON	Protein kinase,catalytic domain
Kabuli_wild_11702025	1399943412	*Ca_kabuli_Chr08*	11702025	C/T	Gene_Ca_16803	INTRON	Protein kinase,catalytic domain
Kabuli_wild_11721730	1399943413	*Ca_kabuli_Chr08*	11721730	G/T	NA	INTERGENIC	NA
Kabuli_wild_11735882	1399943414	*Ca_kabuli_Chr08*	11735882	A/C	Gene_Ca_16799	SYNONYMOUS_CODING	Heat shock protein DnaJ, cysteine-richdomain
Kabuli_wild_11760324	1399943415	*Ca_kabuli_Chr08*	11760324	G/C	Gene_Ca_16796	INTRON	NUDIX hydrolase domain
Kabuli_wild_12022984	1399943416	*Ca_kabuli_Chr08*	12022984	G/C	Gene_Ca_23847	NON_SYNONYMOUS_CODING	Peptidyl-tRNAhydrolase
Kabuli_wild_12076492	1399943417	*Ca_kabuli_Chr08*	12076492	A/G	Gene_Ca_23850	SYNONYMOUS_CODING	PutativeRNAmethylase
Kabuli_wild_12865072	1399943418	*Ca_kabuli_Chr08*	12865072	C/A	NA	INTERGENIC	NA
Kabuli_wild_12875187	1399943419	*Ca_kabuli_Chr08*	12875187	T/G	NA	INTERGENIC	NA

SNP IDs	NCBI dbSNP accession IDs (NCBI_SS#)	Chromosomes	Physical positions (bp)	SNPs	Gene accession IDs	Sequence components of genome	Putative functions
Kabuli_wild_12875217	1399943420	*Ca_kabuli_Chr08*	12875217	A/G	NA	INTERGENIC	NA
Kabuli_wild_13062576	1399943421	*Ca_kabuli_Chr08*	13062576	A/G	NA	INTERGENIC	NA
Kabuli_wild_13137386	1399943422	*Ca_kabuli_Chr08*	13137386	T/G	Gene_Ca_20103	INTRON	NA
Kabuli_wild_13137388	1399943423	*Ca_kabuli_Chr08*	13137388	T/G	Gene_Ca_20103	INTRON	NA
Kabuli_wild_13248268	1399943424	*Ca_kabuli_Chr08*	13248268	T/C	Gene_Ca_20108	SYNONYMOUS_CODING	C2 calcium-dependent membrane targeting
Kabuli_wild_13745335	1399943425	*Ca_kabuli_Chr08*	13745335	A/G	NA	INTERGENIC	NA
Kabuli_wild_13830309	1399943426	*Ca_kabuli_Chr08*	13830309	A/T	Gene_Ca_22724	NON_SYNONYMOUS_CODING	Arf GTPase activating protein
Kabuli_wild_13840865	1399943427	*Ca_kabuli_Chr08*	13840865	A/C	NA	INTERGENIC	NA
Kabuli_wild_13864026	1399943428	*Ca_kabuli_Chr08*	13864026	T/G	NA	INTERGENIC	NA
Kabuli_wild_13997371	1399943429	*Ca_kabuli_Chr08*	13997371	C/T	NA	INTERGENIC	NA
Kabuli_wild_13997386	1399943430	*Ca_kabuli_Chr08*	13997386	G/A	NA	INTERGENIC	NA
Kabuli_wild_13998102	1399943431	*Ca_kabuli_Chr08*	13998102	A/G	Gene_Ca_22740	UPSTREAM	NA
Kabuli_wild_14006709	1399943432	*Ca_kabuli_Chr08*	14006709	G/A	Gene_Ca_22742	INTRON	NA
Kabuli_wild_14007360	1399943433	*Ca_kabuli_Chr08*	14007360	T/A	Gene_Ca_22742	INTRON	NA
Kabuli_wild_14007431	1399943434	*Ca_kabuli_Chr08*	14007431	A/G	Gene_Ca_22742	INTRON	NA
Kabuli_wild_14124588	1399943435	*Ca_kabuli_Chr08*	14124588	C/A	NA	INTERGENIC	NA
Kabuli_wild_14129040	1399943436	*Ca_kabuli_Chr08*	14129040	G/A	Gene_Ca_13047	NON_SYNONYMOUS_CODING	Proteinphosphatase2C-like
Kabuli_wild_14325980	1399943437	*Ca_kabuli_Chr08*	14325980	C/A	Gene_Ca_13032	INTRON	Kinesin,motordomain
Kabuli_wild_14436678	1399943438	*Ca_kabuli_Chr08*	14436678	A/T	NA	INTERGENIC	NA
Kabuli_wild_14444330	1399943439	*Ca_kabuli_Chr08*	14444330	C/T	NA	INTERGENIC	NA
Kabuli_wild_14444424	1399943440	*Ca_kabuli_Chr08*	14444424	G/A	NA	INTERGENIC	NA
Kabuli_wild_14444438	1399943441	*Ca_kabuli_Chr08*	14444438	A/C	NA	INTERGENIC	NA
Kabuli_wild_14445205	1399943442	*Ca_kabuli_Chr08*	14445205	C/A	NA	INTERGENIC	NA
Kabuli_wild_14466020	1399943443	*Ca_kabuli_Chr08*	14466020	T/C	Gene_Ca_13020	INTRON	Zinc finger, RING-type
Kabuli_wild_14466050	1399943444	*Ca_kabuli_Chr08*	14466050	A/G	Gene_Ca_13020	INTRON	Zinc finger, RING-type
Kabuli_wild_14497891	1399943445	*Ca_kabuli_Chr08*	14497891	A/G	Gene_Ca_13019	INTRON	Protein of unknown function DUF639
Kabuli_wild_14497919	1399943446	*Ca_kabuli_Chr08*	14497919	C/A	Gene_Ca_13019	NON_SYNONYMOUS_CODING	Protein of unknown function DUF639
Kabuli_wild_14528966	1399943447	*Ca_kabuli_Chr08*	14528966	C/G	NA	INTERGENIC	NA
Kabuli_wild_14613302	1399943448	*Ca_kabuli_Chr08*	14613302	G/A	Gene_Ca_13006	INTRON	Endonuclease/exonuclease/phosphatase
Kabuli_wild_14626081	1399943449	*Ca_kabuli_Chr08*	14626081	G/A	NA	INTERGENIC	NA
Kabuli_wild_14626100	1399943450	*Ca_kabuli_Chr08*	14626100	T/C	NA	INTERGENIC	NA
Kabuli_wild_14629887	1399943451	*Ca_kabuli_Chr08*	14629887	G/A	NA	INTERGENIC	NA
Kabuli_wild_14629906	1399943452	*Ca_kabuli_Chr08*	14629906	T/C	NA	INTERGENIC	NA
Kabuli_wild_14640967	1399943453	*Ca_kabuli_Chr08*	14640967	A/C	Gene_Ca_13004	INTRON	Toprimdomain
Kabuli_wild_14715495	1399943454	*Ca_kabuli_Chr08*	14715495	T/G	Gene_Ca_12997	INTRON	HEAT
Kabuli_wild_14759784	1399943455	*Ca_kabuli_Chr08*	14759784	G/T	NA	INTERGENIC	NA
Kabuli_wild_14760059	1399943456	*Ca_kabuli_Chr08*	14760059	T/C	NA	INTERGENIC	NA
Kabuli_wild_14760479	1399943457	*Ca_kabuli_Chr08*	14760479	C/G	NA	INTERGENIC	NA
Kabuli_wild_14760497	1399943458	*Ca_kabuli_Chr08*	14760497	G/A	NA	INTERGENIC	NA
Kabuli_wild_14760535	1399943459	*Ca_kabuli_Chr08*	14760535	G/T	NA	INTERGENIC	NA
Kabuli_wild_14760802	1399943460	*Ca_kabuli_Chr08*	14760802	A/G	NA	INTERGENIC	NA
Kabuli_wild_14821416	1399943461	*Ca_kabuli_Chr08*	14821416	G/A	Gene_Ca_12987	SYNONYMOUS_CODING	Mrp,conservedsite
Kabuli_wild_14969330	1399943462	*Ca_kabuli_Chr08*	14969330	A/C	Gene_Ca_12976	INTRON	DNA mismatch repair protein MutS,C-terminal domain
Kabuli_wild_15000109	1399943463	*Ca_kabuli_Chr08*	15000109	A/G	Gene_Ca_12972	NON_SYNONYMOUS_CODING	Aldehydedehydrogenasedomain

SNP IDs	NCBI dbSNP accession IDs (NCBI_SS#)	Chromosomes	Physical positions (bp)	SNPs	Gene accession IDs	Sequence components of genome	Putative functions
Kabuli_wild_15034465	1399943464	*Ca_kabuli_Chr08*	15034465	T/C	NA	INTERGENIC	NA
Kabuli_wild_15131175	1399943465	*Ca_kabuli_Chr08*	15131175	G/A	Gene_Ca_12965	SYNONYMOUS_CODING	Sugar/inositol transporter
Kabuli_wild_15421674	1399943466	*Ca_kabuli_Chr08*	15421674	A/T	Gene_Ca_17898	DOWNSTREAM	Nucleosidephosphorylasedomain
Kabuli_wild_15473060	1399943467	*Ca_kabuli_Chr08*	15473060	T/C	NA	INTERGENIC	NA
Kabuli_wild_15528709	1399943468	*Ca_kabuli_Chr08*	15528709	T/C	Gene_Ca_17890	INTRON	Protein kinase,catalytic domain
Kabuli_wild_15528710	1399943469	*Ca_kabuli_Chr08*	15528710	T/G	Gene_Ca_17890	INTRON	Protein kinase,catalytic domain
Kabuli_wild_15528717	1399943470	*Ca_kabuli_Chr08*	15528717	C/G	Gene_Ca_17890	INTRON	Protein kinase,catalytic domain
Kabuli_wild_15528719	1399943471	*Ca_kabuli_Chr08*	15528719	A/T	Gene_Ca_17890	INTRON	Protein kinase,catalytic domain
Kabuli_wild_15528720	1399943472	*Ca_kabuli_Chr08*	15528720	A/T	Gene_Ca_17890	INTRON	Protein kinase,catalytic domain
Kabuli_wild_15528721	1399943473	*Ca_kabuli_Chr08*	15528721	C/G	Gene_Ca_17890	INTRON	Protein kinase,catalytic domain
Kabuli_wild_15528724	1399943474	*Ca_kabuli_Chr08*	15528724	C/A	Gene_Ca_17890	INTRON	Protein kinase,catalytic domain
Kabuli_wild_15555771	1399943475	*Ca_kabuli_Chr08*	15555771	A/C	Gene_Ca_17888	SYNONYMOUS_CODING	Protein of unknown function DUF1675
Kabuli_wild_15638443	1399943476	*Ca_kabuli_Chr08*	15638443	C/A	NA	INTERGENIC	NA
Kabuli_wild_15692070	1399943477	*Ca_kabuli_Chr08*	15692070	G/A	Gene_Ca_17876	INTRON	RNA-dependent RNA polymerase, eukaryotic-type
Kabuli_wild_15699180	1399943478	*Ca_kabuli_Chr08*	15699180	T/C	Gene_Ca_17876	INTRON	RNA-dependent RNA polymerase, eukaryotic-type
Kabuli_wild_15764874	1399943479	*Ca_kabuli_Chr08*	15764874	G/A	Gene_Ca_17866	SYNONYMOUS_CODING	Chromodomain
Kabuli_wild_15857158	1399943480	*Ca_kabuli_Chr08*	15857158	G/C	Gene_Ca_17859	DOWNSTREAM	Pectinesterase,catalytic
Kabuli_wild_15857192	1399943481	*Ca_kabuli_Chr08*	15857192	A/G	Gene_Ca_17859	DOWNSTREAM	Pectinesterase,catalytic
Kabuli_wild_15857359	1399943482	*Ca_kabuli_Chr08*	15857359	T/G	Gene_Ca_17859	DOWNSTREAM	Pectinesterase,catalytic
Kabuli_wild_15871698	1399943483	*Ca_kabuli_Chr08*	15871698	T/C	Gene_Ca_17857	NON_SYNONYMOUS_CODING	HEAT
Kabuli_wild_15873044	1399943484	*Ca_kabuli_Chr08*	15873044	A/G	Gene_Ca_17857	SYNONYMOUS_CODING	HEAT
Kabuli_wild_15873071	1399943485	*Ca_kabuli_Chr08*	15873071	A/C	Gene_Ca_17857	SYNONYMOUS_CODING	HEAT
Kabuli_wild_15873133	1399943486	*Ca_kabuli_Chr08*	15873133	A/G	Gene_Ca_17857	NON_SYNONYMOUS_CODING	HEAT
Kabuli_wild_16004511	1399943487	*Ca_kabuli_Chr08*	16004511	T/C	Gene_Ca_15508	DOWNSTREAM	Profilin/allergen
Kabuli_wild_16004976	1399943488	*Ca_kabuli_Chr08*	16004976	G/C	Gene_Ca_15508	DOWNSTREAM	Profilin/allergen
Kabuli_wild_16005046	1399943489	*Ca_kabuli_Chr08*	16005046	C/T	Gene_Ca_15508	DOWNSTREAM	Profilin/allergen
Kabuli_wild_16005078	1399943490	*Ca_kabuli_Chr08*	16005078	A/G	Gene_Ca_15508	DOWNSTREAM	Profilin/allergen
Kabuli_wild_16020239	1399943491	*Ca_kabuli_Chr08*	16020239	C/A	NA	INTERGENIC	NA
Kabuli_wild_16020277	1399943492	*Ca_kabuli_Chr08*	16020277	T/G	NA	INTERGENIC	NA
Kabuli_wild_16020323	1399943493	*Ca_kabuli_Chr08*	16020323	G/T	NA	INTERGENIC	NA
Kabuli_wild_16022287	1399943494	*Ca_kabuli_Chr08*	16022287	G/A	Gene_Ca_15512	UPSTREAM	Acidphosphatase(ClassB)
Kabuli_wild_16022432	1399943495	*Ca_kabuli_Chr08*	16022432	C/T	Gene_Ca_15512	UPSTREAM	Acidphosphatase(ClassB)
Kabuli_wild_16027974	1399943496	*Ca_kabuli_Chr08*	16027974	A/T	NA	INTERGENIC	NA
Kabuli_wild_16028040	1399943497	*Ca_kabuli_Chr08*	16028040	A/G	NA	INTERGENIC	NA
Kabuli_wild_16028062	1399943498	*Ca_kabuli_Chr08*	16028062	G/A	NA	INTERGENIC	NA
Kabuli_wild_16028064	1399943499	*Ca_kabuli_Chr08*	16028064	G/T	NA	INTERGENIC	NA
Kabuli_wild_16076161	1399943500	*Ca_kabuli_Chr08*	16076161	C/G	Gene_Ca_15517	NON_SYNONYMOUS_CODING	Gamma-glutamyltranspeptidase
Kabuli_wild_16076168	1399943501	*Ca_kabuli_Chr08*	16076168	A/G	Gene_Ca_15517	SYNONYMOUS_CODING	Gamma-glutamyltranspeptidase
Kabuli_wild_16076170	1399943502	*Ca_kabuli_Chr08*	16076170	T/C	Gene_Ca_15517	NON_SYNONYMOUS_CODING	Gamma-glutamyltranspeptidase
Kabuli_wild_16102414	1399943503	*Ca_kabuli_Chr08*	16102414	G/T	NA	INTERGENIC	NA
Kabuli_wild_16130914	1399943504	*Ca_kabuli_Chr08*	16130914	A/C	NA	INTERGENIC	NA
Kabuli_wild_16144279	1399943505	*Ca_kabuli_Chr08*	16144279	T/A	NA	INTERGENIC	NA
Kabuli_wild_16145275	1399943506	*Ca_kabuli_Chr08*	16145275	A/G	Gene_Ca_15527	INTRON	Domain of unknown function DUF292,eukaryotic
Kabuli_wild_16145370	1399943507	*Ca_kabuli_Chr08*	16145370	C/A	Gene_Ca_15527	INTRON	Domain of unknown function DUF292,eukaryotic

SNP IDs	NCBI dbSNP accession IDs (NCBI_SS#)	Chromosomes	Physical positions (bp)	SNPs	Gene accession IDs	Sequence components of genome	Putative functions
Kabuli_wild_16145376	1399943508	*Ca_kabuli_Chr08*	16145376	T/A	Gene_Ca_15527	INTRON	Domain of unknown function DUF292,eukaryotic
Kabuli_wild_16145385	1399943509	*Ca_kabuli_Chr08*	16145385	A/C	Gene_Ca_15527	INTRON	Domain of unknown function DUF292,eukaryotic
Kabuli_wild_16145411	1399943510	*Ca_kabuli_Chr08*	16145411	C/G	Gene_Ca_15527	NON_SYNONYMOUS_CODING	Domain of unknown function DUF292,eukaryotic
Kabuli_wild_16264310	1399943511	*Ca_kabuli_Chr08*	16264310	G/T	NA	INTERGENIC	NA
Kabuli_wild_16278758	1399943512	*Ca_kabuli_Chr08*	16278758	T/C	NA	INTERGENIC	NA
Kabuli_wild_16283660	1399943513	*Ca_kabuli_Chr08*	16283660	C/T	NA	INTERGENIC	NA
Kabuli_wild_16283661	1399943514	*Ca_kabuli_Chr08*	16283661	A/G	NA	INTERGENIC	NA
Kabuli_wild_16283722	1399943515	*Ca_kabuli_Chr08*	16283722	A/G	NA	INTERGENIC	NA
Kabuli_wild_16283738	1399943516	*Ca_kabuli_Chr08*	16283738	G/C	NA	INTERGENIC	NA
Kabuli_wild_16366536	1399943517	*Ca_kabuli_Chr08*	16366536	T/A	Gene_Ca_15558	SYNONYMOUS_CODING	Prenyltransferase/squaleneoxidase
Kabuli_wild_16411550	1399943518	*Ca_kabuli_Chr08*	16411550	A/G	Gene_Ca_15562	INTRON	NA
Kabuli_wild_16421407	1399943519	*Ca_kabuli_Chr08*	16421407	G/A	NA	INTERGENIC	NA
Kabuli_wild_16421441	1399943520	*Ca_kabuli_Chr08*	16421441	C/A	Gene_Ca_15563	NON_SYNONYMOUS_CODING	NA
Kabuli_wild_16421442	1399943521	*Ca_kabuli_Chr08*	16421442	C/G	Gene_Ca_15563	SYNONYMOUS_CODING	NA
Kabuli_wild_16421497	1399943522	*Ca_kabuli_Chr08*	16421497	C/T	Gene_Ca_15563	NON_SYNONYMOUS_CODING	NA
Kabuli_wild_16421595	1399943523	*Ca_kabuli_Chr08*	16421595	A/C	Gene_Ca_15563	SYNONYMOUS_CODING	NA
Kabuli_wild_16432907	1399943524	*Ca_kabuli_Chr08*	16432907	T/G	Gene_Ca_15563	INTRON	NA
Kabuli_wild_16472919	1399943525	*Ca_kabuli_Chr08*	16472919	T/A	NA	INTERGENIC	NA
Kabuli_wild_16472932	1399943526	*Ca_kabuli_Chr08*	16472932	T/C	NA	INTERGENIC	NA
Kabuli_wild_16472933	1399943527	*Ca_kabuli_Chr08*	16472933	C/G	NA	INTERGENIC	NA
Kabuli_wild_16472998	1399943528	*Ca_kabuli_Chr08*	16472998	C/T	NA	INTERGENIC	NA
Kabuli_wild_16473139	1399943529	*Ca_kabuli_Chr08*	16473139	C/T	NA	INTERGENIC	NA
Kabuli_wild_16473157	1399943530	*Ca_kabuli_Chr08*	16473157	C/T	NA	INTERGENIC	NA
Kabuli_wild_16473290	1399943531	*Ca_kabuli_Chr08*	16473290	A/G	NA	INTERGENIC	NA
Kabuli_wild_137301	1399943532	*Ca_kabuli_scaffold*	137301	G/A	NA	INTERGENIC	NA
Kabuli_wild_137309	1399943533	*Ca_kabuli_scaffold*	137309	T/G	NA	INTERGENIC	NA
Kabuli_wild_504248	1399943534	*Ca_kabuli_scaffold*	504248	G/A	NA	INTERGENIC	NA
Kabuli_wild_504253	1399943535	*Ca_kabuli_scaffold*	504253	C/T	NA	INTERGENIC	NA
Kabuli_wild_504258	1399943536	*Ca_kabuli_scaffold*	504258	C/T	NA	INTERGENIC	NA
Kabuli_wild_505926	1399943537	*Ca_kabuli_scaffold*	505926	C/T	NA	INTERGENIC	NA
Kabuli_wild_505931	1399943538	*Ca_kabuli_scaffold*	505931	C/T	NA	INTERGENIC	NA
Kabuli_wild_61952	1399943539	*Ca_kabuli_scaffold*	61952	G/A	Gene_Ca_27384	INTRON	NA
Kabuli_wild_62027	1399943540	*Ca_kabuli_scaffold*	62027	T/G	Gene_Ca_27384	INTRON	NA
Kabuli_wild_41096	1399943541	*Ca_kabuli_scaffold*	41096	G/A	NA	INTERGENIC	NA
Kabuli_wild_90	1399943542	*Ca_kabuli_scaffold*	90	C/T	NA	INTERGENIC	NA
Kabuli_wild_95	1399943543	*Ca_kabuli_scaffold*	95	A/G	NA	INTERGENIC	NA
Kabuli_wild_122	1399943544	*Ca_kabuli_scaffold*	122	G/A	NA	INTERGENIC	NA
Kabuli_wild_126	1399943545	*Ca_kabuli_scaffold*	126	T/C	NA	INTERGENIC	NA
Kabuli_wild_131	1399943546	*Ca_kabuli_scaffold*	131	A/C	NA	INTERGENIC	NA
Kabuli_wild_133	1399943547	*Ca_kabuli_scaffold*	133	C/T	NA	INTERGENIC	NA
Kabuli_wild_134	1399943548	*Ca_kabuli_scaffold*	134	C/T	NA	INTERGENIC	NA
Kabuli_wild_136	1399943549	*Ca_kabuli_scaffold*	136	G/A	NA	INTERGENIC	NA
Kabuli_wild_140	1399943550	*Ca_kabuli_scaffold*	140	A/C	NA	INTERGENIC	NA
Kabuli_wild_145	1399943551	*Ca_kabuli_scaffold*	145	T/G	NA	INTERGENIC	NA

SNP IDs	NCBI dbSNP accession IDs (NCBI_SS#)	Chromosomes	Physical positions (bp)	SNPs	Gene accession IDs	Sequence components of genome	Putative functions
Kabuli_wild_11903	1399943552	*Ca_kabuli_scaffold*	11903	A/T	NA	INTERGENIC	NA
Kabuli_wild_32269	1399943553	*Ca_kabuli_scaffold*	32269	A/G	NA	INTERGENIC	NA
Kabuli_wild_32303	1399943554	*Ca_kabuli_scaffold*	32303	T/C	NA	INTERGENIC	NA
Kabuli_wild_32310	1399943555	*Ca_kabuli_scaffold*	32310	C/T	NA	INTERGENIC	NA
Kabuli_wild_60399	1399943556	*Ca_kabuli_scaffold*	60399	G/A	Gene_Ca_22968	NON_SYNONYMOUS_CODING	Peptidase C48, SUMO/Sentrin/Ubl1
Kabuli_wild_60403	1399943557	*Ca_kabuli_scaffold*	60403	C/G	Gene_Ca_22968	NON_SYNONYMOUS_CODING	Peptidase C48, SUMO/Sentrin/Ubl1
Kabuli_wild_100399	1399943558	*Ca_kabuli_scaffold*	100399	C/A	Gene_Ca_22971	NON_SYNONYMOUS_CODING	NA
Kabuli_wild_100418	1399943559	*Ca_kabuli_scaffold*	100418	G/T	Gene_Ca_22971	SYNONYMOUS_CODING	NA
Kabuli_wild_100460	1399943560	*Ca_kabuli_scaffold*	100460	G/A	Gene_Ca_22971	SYNONYMOUS_CODING	NA
Kabuli_wild_100472	1399943561	*Ca_kabuli_scaffold*	100472	A/G	Gene_Ca_22971	SYNONYMOUS_CODING	NA
Kabuli_wild_100476	1399943562	*Ca_kabuli_scaffold*	100476	G/A	Gene_Ca_22971	NON_SYNONYMOUS_CODING	NA
Kabuli_wild_100480	1399943563	*Ca_kabuli_scaffold*	100480	C/T	Gene_Ca_22971	NON_SYNONYMOUS_CODING	NA
Kabuli_wild_100483	1399943564	*Ca_kabuli_scaffold*	100483	C/T	Gene_Ca_22971	NON_SYNONYMOUS_CODING	NA
Kabuli_wild_107748	1399943565	*Ca_kabuli_scaffold*	107748	C/T	Gene_Ca_22972	NON_SYNONYMOUS_CODING	Peptidase C48, SUMO/Sentrin/Ubl1
Kabuli_wild_107757	1399943566	*Ca_kabuli_scaffold*	107757	C/T	Gene_Ca_22972	NON_SYNONYMOUS_CODING	Peptidase C48, SUMO/Sentrin/Ubl1
Kabuli_wild_107774	1399943567	*Ca_kabuli_scaffold*	107774	C/T	Gene_Ca_22972	NON_SYNONYMOUS_CODING	Peptidase C48, SUMO/Sentrin/Ubl1
Kabuli_wild_107807	1399943568	*Ca_kabuli_scaffold*	107807	G/A	Gene_Ca_22972	NON_SYNONYMOUS_CODING	Peptidase C48, SUMO/Sentrin/Ubl1
Kabuli_wild_107823	1399943569	*Ca_kabuli_scaffold*	107823	G/A	Gene_Ca_22972	SYNONYMOUS_CODING	Peptidase C48, SUMO/Sentrin/Ubl1
Kabuli_wild_107828	1399943570	*Ca_kabuli_scaffold*	107828	T/C	Gene_Ca_22972	NON_SYNONYMOUS_CODING	Peptidase C48, SUMO/Sentrin/Ubl1
Kabuli_wild_107840	1399943571	*Ca_kabuli_scaffold*	107840	G/T	Gene_Ca_22972	NON_SYNONYMOUS_CODING	Peptidase C48, SUMO/Sentrin/Ubl1
Kabuli_wild_200573	1399943572	*Ca_kabuli_scaffold*	200573	G/C	NA	INTERGENIC	NA
Kabuli_wild_224114	1399943573	*Ca_kabuli_scaffold*	224114	A/G	NA	INTERGENIC	NA
Kabuli_wild_331890	1399943574	*Ca_kabuli_scaffold*	331890	A/G	NA	INTERGENIC	NA
Kabuli_wild_332909	1399943575	*Ca_kabuli_scaffold*	332909	C/T	NA	INTERGENIC	NA
Kabuli_wild_332921	1399943576	*Ca_kabuli_scaffold*	332921	G/A	NA	INTERGENIC	NA
Kabuli_wild_332963	1399943577	*Ca_kabuli_scaffold*	332963	C/T	NA	INTERGENIC	NA
Kabuli_wild_62781	1399943578	*Ca_kabuli_scaffold*	62781	C/T	Gene_Ca_28098	SYNONYMOUS_CODING	NA
Kabuli_wild_62807	1399943579	*Ca_kabuli_scaffold*	62807	C/T	Gene_Ca_28098	NON_SYNONYMOUS_CODING	NA
Kabuli_wild_167556	1399943580	*Ca_kabuli_scaffold*	167556	C/T	NA	INTERGENIC	NA
Kabuli_wild_226511	1399943581	*Ca_kabuli_scaffold*	226511	A/G	Gene_Ca_25864	SYNONYMOUS_CODING	NA
Kabuli_wild_167242	1399943582	*Ca_kabuli_scaffold*	167242	A/G	Gene_Ca_27246	SYNONYMOUS_CODING	NA
Kabuli_wild_69458	1399943583	*Ca_kabuli_scaffold*	69458	G/A	NA	INTERGENIC	NA
Kabuli_wild_69499	1399943584	*Ca_kabuli_scaffold*	69499	A/G	NA	INTERGENIC	NA
Kabuli_wild_69547	1399943585	*Ca_kabuli_scaffold*	69547	T/G	NA	INTERGENIC	NA
Kabuli_wild_288084	1399943586	*Ca_kabuli_scaffold*	288084	T/C	Gene_Ca_23621	DOWNSTREAM	Helix-loop-helixDNA-binding domain
Kabuli_wild_413685	1399943587	*Ca_kabuli_scaffold*	413685	G/T	NA	INTERGENIC	NA
Kabuli_wild_413691	1399943588	*Ca_kabuli_scaffold*	413691	G/A	NA	INTERGENIC	NA
Kabuli_wild_413713	1399943589	*Ca_kabuli_scaffold*	413713	C/A	NA	INTERGENIC	NA
Kabuli_wild_413719	1399943590	*Ca_kabuli_scaffold*	413719	A/G	NA	INTERGENIC	NA
Kabuli_wild_457392	1399943591	*Ca_kabuli_scaffold*	457392	C/A	Gene_Ca_23625	SYNONYMOUS_CODING	Armadillo
Kabuli_wild_457596	1399943592	*Ca_kabuli_scaffold*	457596	T/A	Gene_Ca_23625	INTRON	Armadillo
Kabuli_wild_520311	1399943593	*Ca_kabuli_scaffold*	520311	G/A	NA	INTERGENIC	NA
Kabuli_wild_520372	1399943594	*Ca_kabuli_scaffold*	520372	T/G	NA	INTERGENIC	NA
Kabuli_wild_520377	1399943595	*Ca_kabuli_scaffold*	520377	T/C	NA	INTERGENIC	NA

SNP IDs	NCBI dbSNP accession IDs (NCBI_SS#)	Chromosomes	Physical positions (bp)	SNPs	Gene accession IDs	Sequence components of genome	Putative functions
Kabuli_wild_537036	1399943596	*Ca_kabuli_scaffold*	537036	G/T	NA	INTERGENIC	NA
Kabuli_wild_537059	1399943597	*Ca_kabuli_scaffold*	537059	A/T	NA	INTERGENIC	NA
Kabuli_wild_537068	1399943598	*Ca_kabuli_scaffold*	537068	G/A	NA	INTERGENIC	NA
Kabuli_wild_538657	1399943599	*Ca_kabuli_scaffold*	538657	T/C	NA	INTERGENIC	NA
Kabuli_wild_538674	1399943600	*Ca_kabuli_scaffold*	538674	G/C	NA	INTERGENIC	NA
Kabuli_wild_538803	1399943601	*Ca_kabuli_scaffold*	538803	T/C	NA	INTERGENIC	NA
Kabuli_wild_538909	1399943602	*Ca_kabuli_scaffold*	538909	A/G	NA	INTERGENIC	NA
Kabuli_wild_538996	1399943603	*Ca_kabuli_scaffold*	538996	T/C	NA	INTERGENIC	NA
Kabuli_wild_539182	1399943604	*Ca_kabuli_scaffold*	539182	C/T	NA	INTERGENIC	NA
Kabuli_wild_539215	1399943605	*Ca_kabuli_scaffold*	539215	T/C	NA	INTERGENIC	NA
Kabuli_wild_541373	1399943606	*Ca_kabuli_scaffold*	541373	T/G	NA	INTERGENIC	NA
Kabuli_wild_386189	1399943607	*Ca_kabuli_scaffold*	386189	A/C	NA	INTERGENIC	NA
Kabuli_wild_497729	1399943608	*Ca_kabuli_scaffold*	497729	T/C	Gene_Ca_18973	NON_SYNONYMOUS_CODING	F-boxdomain,cyclin-like
Kabuli_wild_542951	1399943609	*Ca_kabuli_scaffold*	542951	C/G	Gene_Ca_18975	INTRON	NA
Kabuli_wild_548453	1399943610	*Ca_kabuli_scaffold*	548453	A/G	Gene_Ca_18975	INTRON	NA
Kabuli_wild_548499	1399943611	*Ca_kabuli_scaffold*	548499	C/A	Gene_Ca_18975	INTRON	NA
Kabuli_wild_715064	1399943612	*Ca_kabuli_scaffold*	715064	C/T	Gene_Ca_18984	NON_SYNONYMOUS_CODING	NA
Kabuli_wild_869557	1399943613	*Ca_kabuli_scaffold*	869557	T/A	NA	INTERGENIC	NA
Kabuli_wild_871303	1399943614	*Ca_kabuli_scaffold*	871303	A/C	NA	INTERGENIC	NA
Kabuli_wild_871346	1399943615	*Ca_kabuli_scaffold*	871346	G/A	NA	INTERGENIC	NA
Kabuli_wild_871507	1399943616	*Ca_kabuli_scaffold*	871507	C/A	NA	INTERGENIC	NA
Kabuli_wild_910472	1399943617	*Ca_kabuli_scaffold*	910472	C/T	NA	INTERGENIC	NA
Kabuli_wild_911602	1399943618	*Ca_kabuli_scaffold*	911602	T/G	Gene_Ca_18994	INTRON	NA
Kabuli_wild_961089	1399943619	*Ca_kabuli_scaffold*	961089	G/A	NA	INTERGENIC	NA
Kabuli_wild_962675	1399943620	*Ca_kabuli_scaffold*	962675	G/A	NA	INTERGENIC	NA
Kabuli_wild_968798	1399943621	*Ca_kabuli_scaffold*	968798	G/A	Gene_Ca_18995	SYNONYMOUS_CODING	NA
Kabuli_wild_992642	1399943622	*Ca_kabuli_scaffold*	992642	T/G	Gene_Ca_18996	NON_SYNONYMOUS_CODING	NA
Kabuli_wild_996604	1399943623	*Ca_kabuli_scaffold*	996604	T/C	Gene_Ca_18996	SYNONYMOUS_CODING	NA
Kabuli_wild_1002765	1399943624	*Ca_kabuli_scaffold*	1002765	C/G	Gene_Ca_18997	SYNONYMOUS_CODING	NA
Kabuli_wild_1002801	1399943625	*Ca_kabuli_scaffold*	1002801	A/G	Gene_Ca_18997	SYNONYMOUS_CODING	NA
Kabuli_wild_1014805	1399943626	*Ca_kabuli_scaffold*	1014805	C/T	NA	INTERGENIC	NA
Kabuli_wild_1014826	1399943627	*Ca_kabuli_scaffold*	1014826	C/T	NA	INTERGENIC	NA
Kabuli_wild_1014834	1399943628	*Ca_kabuli_scaffold*	1014834	T/A	NA	INTERGENIC	NA
Kabuli_wild_1014875	1399943629	*Ca_kabuli_scaffold*	1014875	G/A	NA	INTERGENIC	NA
Kabuli_wild_1028794	1399943630	*Ca_kabuli_scaffold*	1028794	C/A	NA	INTERGENIC	NA
Kabuli_wild_1028830	1399943631	*Ca_kabuli_scaffold*	1028830	T/A	NA	INTERGENIC	NA
Kabuli_wild_1039218	1399943632	*Ca_kabuli_scaffold*	1039218	T/C	NA	INTERGENIC	NA
Kabuli_wild_1039233	1399943633	*Ca_kabuli_scaffold*	1039233	A/T	NA	INTERGENIC	NA
Kabuli_wild_1040956	1399943634	*Ca_kabuli_scaffold*	1040956	A/C	NA	INTERGENIC	NA
Kabuli_wild_1041022	1399943635	*Ca_kabuli_scaffold*	1041022	A/C	NA	INTERGENIC	NA
Kabuli_wild_1049594	1399943636	*Ca_kabuli_scaffold*	1049594	G/A	Gene_Ca_19000	INTRON	SANT domain,DNA binding
Kabuli_wild_1050164	1399943637	*Ca_kabuli_scaffold*	1050164	C/T	Gene_Ca_19000	INTRON	SANT domain,DNA binding
Kabuli_wild_1050175	1399943638	*Ca_kabuli_scaffold*	1050175	G/C	Gene_Ca_19000	INTRON	SANT domain,DNA binding
Kabuli_wild_1052597	1399943639	*Ca_kabuli_scaffold*	1052597	T/C	Gene_Ca_19000	INTRON	SANT domain,DNA binding

SNP IDs	NCBI dbSNP accession IDs (NCBI_SS#)	Chromosomes	Physical positions (bp)	SNPs	Gene accession IDs	Sequence components of genome	Putative functions
Kabuli_wild_1052983	1399943640	*Ca_kabuli_scaffold*	1052983	G/A	Gene_Ca_19000	INTRON	SANT domain,DNA binding
Kabuli_wild_1053215	1399943641	*Ca_kabuli_scaffold*	1053215	G/A	Gene_Ca_19000	INTRON	SANT domain,DNA binding
Kabuli_wild_1053231	1399943642	*Ca_kabuli_scaffold*	1053231	C/A	Gene_Ca_19000	INTRON	SANT domain,DNA binding
Kabuli_wild_1053232	1399943643	*Ca_kabuli_scaffold*	1053232	G/T	Gene_Ca_19000	INTRON	SANT domain,DNA binding
Kabuli_wild_1053247	1399943644	*Ca_kabuli_scaffold*	1053247	G/A	Gene_Ca_19000	INTRON	SANT domain,DNA binding
Kabuli_wild_1053261	1399943645	*Ca_kabuli_scaffold*	1053261	T/C	Gene_Ca_19000	INTRON	SANT domain,DNA binding
Kabuli_wild_1053416	1399943646	*Ca_kabuli_scaffold*	1053416	G/C	Gene_Ca_19000	INTRON	SANT domain,DNA binding
Kabuli_wild_1055590	1399943647	*Ca_kabuli_scaffold*	1055590	G/A	Gene_Ca_19000	NON_SYNONYMOUS_CODING	SANT domain,DNA binding
Kabuli_wild_1055634	1399943648	*Ca_kabuli_scaffold*	1055634	G/A	Gene_Ca_19000	SYNONYMOUS_CODING	SANT domain,DNA binding
Kabuli_wild_137462	1399943649	*Ca_kabuli_scaffold*	137462	A/G	NA	INTERGENIC	NA
Kabuli_wild_66280	1399943650	*Ca_kabuli_scaffold*	66280	G/A	Gene_Ca_25409	NON_SYNONYMOUS_CODING	NA
Kabuli_wild_87412	1399943651	*Ca_kabuli_scaffold*	87412	G/A	Gene_Ca_25411	NON_SYNONYMOUS_CODING	NA
Kabuli_wild_120510	1399943652	*Ca_kabuli_scaffold*	120510	T/C	Gene_Ca_25412	NON_SYNONYMOUS_CODING	Protein kinase,catalytic domain
Kabuli_wild_39438	1399943653	*Ca_kabuli_scaffold*	39438	C/A	Gene_Ca_27600	DOWNSTREAM	NA
Kabuli_wild_39470	1399943654	*Ca_kabuli_scaffold*	39470	T/C	Gene_Ca_27600	DOWNSTREAM	NA
Kabuli_wild_261719	1399943655	*Ca_kabuli_scaffold*	261719	A/G	NA	INTERGENIC	NA
Kabuli_wild_191625	1399943656	*Ca_kabuli_scaffold*	191625	T/C	Gene_Ca_24567	NON_SYNONYMOUS_CODING	NA
Kabuli_wild_195777	1399943657	*Ca_kabuli_scaffold*	195777	A/G	NA	INTERGENIC	NA
Kabuli_wild_195828	1399943658	*Ca_kabuli_scaffold*	195828	G/C	NA	INTERGENIC	NA
Kabuli_wild_201219	1399943659	*Ca_kabuli_scaffold*	201219	C/G	NA	INTERGENIC	NA
Kabuli_wild_448866	1399943660	*Ca_kabuli_scaffold*	448866	G/A	Gene_Ca_24573	INTRON	NA
Kabuli_wild_49273	1399943661	*Ca_kabuli_scaffold*	49273	A/G	NA	INTERGENIC	NA
Kabuli_wild_46021	1399943662	*Ca_kabuli_scaffold*	46021	C/T	Gene_Ca_26577	SYNONYMOUS_CODING	Peptidase C48, SUMO/Sentrin/Ubl1
Kabuli_wild_46080	1399943663	*Ca_kabuli_scaffold*	46080	G/A	Gene_Ca_26577	SYNONYMOUS_CODING	Peptidase C48, SUMO/Sentrin/Ubl1
Kabuli_wild_46088	1399943664	*Ca_kabuli_scaffold*	46088	C/T	Gene_Ca_26577	NON_SYNONYMOUS_CODING	Peptidase C48, SUMO/Sentrin/Ubl1
Kabuli_wild_87968	1399943665	*Ca_kabuli_scaffold*	87968	G/T	NA	INTERGENIC	NA
Kabuli_wild_207676	1399943666	*Ca_kabuli_scaffold*	207676	A/G	NA	INTERGENIC	NA
Kabuli_wild_34108	1399943667	*Ca_kabuli_scaffold*	34108	C/T	NA	INTERGENIC	NA
Kabuli_wild_34115	1399943668	*Ca_kabuli_scaffold*	34115	G/A	NA	INTERGENIC	NA
Kabuli_wild_34160	1399943669	*Ca_kabuli_scaffold*	34160	G/T	NA	INTERGENIC	NA
Kabuli_wild_34164	1399943670	*Ca_kabuli_scaffold*	34164	C/T	NA	INTERGENIC	NA
Kabuli_wild_123866	1399943671	*Ca_kabuli_scaffold*	123866	G/A	Gene_Ca_26292	UPSTREAM	Zinc finger,CCHC-type
Kabuli_wild_123870	1399943672	*Ca_kabuli_scaffold*	123870	T/G	Gene_Ca_26292	UPSTREAM	NA
Kabuli_wild_123935	1399943673	*Ca_kabuli_scaffold*	123935	C/G	Gene_Ca_26292	UPSTREAM	NA
Kabuli_wild_123936	1399943674	*Ca_kabuli_scaffold*	123936	T/G	Gene_Ca_26292	UPSTREAM	NA
Kabuli_wild_376643	1399943675	*Ca_kabuli_scaffold*	376643	A/C	NA	INTERGENIC	NA
Kabuli_wild_66090	1399943676	*Ca_kabuli_scaffold*	66090	G/A	NA	INTERGENIC	NA
Kabuli_wild_8772	1399943677	*Ca_kabuli_scaffold*	8772	A/G	Gene_Ca_23383	INTRON	Peptidase C48, SUMO/Sentrin/Ubl1
Kabuli_wild_8799	1399943678	*Ca_kabuli_scaffold*	8799	G/A	Gene_Ca_23383	INTRON	Peptidase C48, SUMO/Sentrin/Ubl1
Kabuli_wild_182781	1399943679	*Ca_kabuli_scaffold*	182781	C/A	Gene_Ca_23388	NON_SYNONYMOUS_CODING	PeptidaseS8/S53,subtilisin/kexin/sedolisin
Kabuli_wild_182788	1399943680	*Ca_kabuli_scaffold*	182788	A/C	Gene_Ca_23388	SYNONYMOUS_CODING	PeptidaseS8/S53,subtilisin/kexin/sedolisin
Kabuli_wild_240767	1399943681	*Ca_kabuli_scaffold*	240767	G/T	Gene_Ca_23389	SYNONYMOUS_CODING	Transposase,Ptta/En/Spm,plant
Kabuli_wild_9982	1399943682	*Ca_kabuli_scaffold*	9982	C/A	Gene_Ca_22168	INTRON	Dakkinase
Kabuli_wild_16398	1399943683	*Ca_kabuli_scaffold*	16398	C/T	Gene_Ca_22169	NON_SYNONYMOUS_CODING	ZF-HD homeobox protein,Cys/His-rich dimerisation domain

SNP IDs	NCBI dbSNP accession IDs (NCBI_SS#)	Chromosomes	Physical positions (bp)	SNPs	Gene accession IDs	Sequence components of genome	Putative functions
Kabuli_wild_86009	1399943684	*Ca_kabuli_scaffold*	86009	A/C	NA	INTERGENIC	NA
Kabuli_wild_86027	1399943685	*Ca_kabuli_scaffold*	86027	C/T	NA	INTERGENIC	NA
Kabuli_wild_86048	1399943686	*Ca_kabuli_scaffold*	86048	C/G	NA	INTERGENIC	NA
Kabuli_wild_86053	1399943687	*Ca_kabuli_scaffold*	86053	C/T	NA	INTERGENIC	NA
Kabuli_wild_257162	1399943688	*Ca_kabuli_scaffold*	257162	T/A	NA	INTERGENIC	NA
Kabuli_wild_274006	1399943689	*Ca_kabuli_scaffold*	274006	A/G	Gene_Ca_22187	INTRON	RNA polymerase II subunit A
Kabuli_wild_205677	1399943690	*Ca_kabuli_scaffold*	205677	G/A	Gene_Ca_23069	SYNONYMOUS_CODING	Lipoxygenase
Kabuli_wild_321753	1399943691	*Ca_kabuli_scaffold*	321753	A/C	NA	INTERGENIC	NA
Kabuli_wild_366432	1399943692	*Ca_kabuli_scaffold*	366432	C/G	NA	INTERGENIC	NA
Kabuli_wild_500779	1399943693	*Ca_kabuli_scaffold*	500779	G/T	Gene_Ca_23079	SYNONYMOUS_CODING	NA
Kabuli_wild_506983	1399943694	*Ca_kabuli_scaffold*	506983	C/A	NA	INTERGENIC	NA
Kabuli_wild_640363	1399943695	*Ca_kabuli_scaffold*	640363	G/A	NA	INTERGENIC	NA
Kabuli_wild_640373	1399943696	*Ca_kabuli_scaffold*	640373	A/C	NA	INTERGENIC	NA
Kabuli_wild_640378	1399943697	*Ca_kabuli_scaffold*	640378	C/A	NA	INTERGENIC	NA
Kabuli_wild_640389	1399943698	*Ca_kabuli_scaffold*	640389	G/T	NA	INTERGENIC	NA
Kabuli_wild_640410	1399943699	*Ca_kabuli_scaffold*	640410	A/G	NA	INTERGENIC	NA
Kabuli_wild_640430	1399943700	*Ca_kabuli_scaffold*	640430	C/T	NA	INTERGENIC	NA
Kabuli_wild_640438	1399943701	*Ca_kabuli_scaffold*	640438	G/C	NA	INTERGENIC	NA
Kabuli_wild_640444	1399943702	*Ca_kabuli_scaffold*	640444	G/T	NA	INTERGENIC	NA
Kabuli_wild_640445	1399943703	*Ca_kabuli_scaffold*	640445	C/T	NA	INTERGENIC	NA
Kabuli_wild_640447	1399943704	*Ca_kabuli_scaffold*	640447	G/A	NA	INTERGENIC	NA
Kabuli_wild_640448	1399943705	*Ca_kabuli_scaffold*	640448	T/C	NA	INTERGENIC	NA
Kabuli_wild_640459	1399943706	*Ca_kabuli_scaffold*	640459	G/T	NA	INTERGENIC	NA
Kabuli_wild_640467	1399943707	*Ca_kabuli_scaffold*	640467	C/A	NA	INTERGENIC	NA
Kabuli_wild_640487	1399943708	*Ca_kabuli_scaffold*	640487	A/C	NA	INTERGENIC	NA
Kabuli_wild_645328	1399943709	*Ca_kabuli_scaffold*	645328	A/C	Gene_Ca_23085	UPSTREAM	Integrase,catalyticcore
Kabuli_wild_645344	1399943710	*Ca_kabuli_scaffold*	645344	G/T	Gene_Ca_23085	UPSTREAM	Integrase,catalyticcore
Kabuli_wild_645385	1399943711	*Ca_kabuli_scaffold*	645385	C/T	Gene_Ca_23085	UPSTREAM	Integrase,catalyticcore
Kabuli_wild_645393	1399943712	*Ca_kabuli_scaffold*	645393	G/A	Gene_Ca_23085	UPSTREAM	Integrase,catalyticcore
Kabuli_wild_645399	1399943713	*Ca_kabuli_scaffold*	645399	G/T	Gene_Ca_23085	UPSTREAM	Integrase,catalyticcore
Kabuli_wild_645400	1399943714	*Ca_kabuli_scaffold*	645400	C/T	Gene_Ca_23085	UPSTREAM	Integrase,catalyticcore
Kabuli_wild_645402	1399943715	*Ca_kabuli_scaffold*	645402	G/A	Gene_Ca_23085	UPSTREAM	Integrase,catalyticcore
Kabuli_wild_645403	1399943716	*Ca_kabuli_scaffold*	645403	T/C	Gene_Ca_23085	UPSTREAM	Integrase,catalyticcore
Kabuli_wild_645414	1399943717	*Ca_kabuli_scaffold*	645414	G/T	Gene_Ca_23085	UPSTREAM	Integrase,catalyticcore
Kabuli_wild_645426	1399943718	*Ca_kabuli_scaffold*	645426	G/T	Gene_Ca_23085	UPSTREAM	Integrase,catalyticcore
Kabuli_wild_645442	1399943719	*Ca_kabuli_scaffold*	645442	A/C	Gene_Ca_23085	UPSTREAM	Integrase,catalyticcore
Kabuli_wild_650955	1399943720	*Ca_kabuli_scaffold*	650955	T/C	NA	INTERGENIC	NA
Kabuli_wild_650958	1399943721	*Ca_kabuli_scaffold*	650958	G/A	NA	INTERGENIC	NA
Kabuli_wild_650968	1399943722	*Ca_kabuli_scaffold*	650968	G/A	NA	INTERGENIC	NA
Kabuli_wild_650981	1399943723	*Ca_kabuli_scaffold*	650981	T/C	NA	INTERGENIC	NA
Kabuli_wild_651018	1399943724	*Ca_kabuli_scaffold*	651018	C/T	NA	INTERGENIC	NA
Kabuli_wild_651020	1399943725	*Ca_kabuli_scaffold*	651020	C/T	NA	INTERGENIC	NA
Kabuli_wild_29042	1399943726	*Ca_kabuli_scaffold*	29042	A/G	Gene_Ca_24077	NON_SYNONYMOUS_CODING	Peptidoglycan-bindingLysinsubgroup
Kabuli_wild_144260	1399943727	*Ca_kabuli_scaffold*	144260	A/C	NA	INTERGENIC	NA

SNP IDs	NCBI dbSNP accession IDs (NCBI_SS#)	Chromosomes	Physical positions (bp)	SNPs	Gene accession IDs	Sequence components of genome	Putative functions
Kabuli_wild_278004	1399943728	*Ca_kabuli_scaffold*	278004	A/G	Gene_Ca_24088	INTRON	Translation initiation factor SUI1
Kabuli_wild_287933	1399943729	*Ca_kabuli_scaffold*	287933	T/C	NA	INTERGENIC	NA
Kabuli_wild_287956	1399943730	*Ca_kabuli_scaffold*	287956	T/A	NA	INTERGENIC	NA
Kabuli_wild_320224	1399943731	*Ca_kabuli_scaffold*	320224	G/T	Gene_Ca_21385	INTRON	NA
Kabuli_wild_374574	1399943732	*Ca_kabuli_scaffold*	374574	A/T	NA	INTERGENIC	NA
Kabuli_wild_440243	1399943733	*Ca_kabuli_scaffold*	440243	G/C	NA	INTERGENIC	NA
Kabuli_wild_533476	1399943734	*Ca_kabuli_scaffold*	533476	A/G	Gene_Ca_21397	SYNONYMOUS_CODING	Pectinesterase,catalytic
Kabuli_wild_562979	1399943735	*Ca_kabuli_scaffold*	562979	G/A	Gene_Ca_21399	INTRON	Zinc finger,FYVE-type
Kabuli_wild_568221	1399943736	*Ca_kabuli_scaffold*	568221	A/C	Gene_Ca_21399	SYNONYMOUS_CODING	Zinc finger,FYVE-type
Kabuli_wild_573779	1399943737	*Ca_kabuli_scaffold*	573779	A/T	NA	INTERGENIC	NA
Kabuli_wild_82135	1399943738	*Ca_kabuli_scaffold*	82135	G/A	NA	INTERGENIC	NA
Kabuli_wild_21714	1399943739	*Ca_kabuli_scaffold*	21714	C/T	Gene_Ca_26111	NON_SYNONYMOUS_CODING	NA
Kabuli_wild_26572	1399943740	*Ca_kabuli_scaffold*	26572	C/A	Gene_Ca_28117	SYNONYMOUS_CODING	NA
Kabuli_wild_26575	1399943741	*Ca_kabuli_scaffold*	26575	C/A	Gene_Ca_28117	SYNONYMOUS_CODING	NA
Kabuli_wild_26601	1399943742	*Ca_kabuli_scaffold*	26601	C/A	Gene_Ca_28117	SYNONYMOUS_CODING	NA
Kabuli_wild_26603	1399943743	*Ca_kabuli_scaffold*	26603	T/G	Gene_Ca_28117	NON_SYNONYMOUS_CODING	NA
Kabuli_wild_206399	1399943744	*Ca_kabuli_scaffold*	206399	C/T	Gene_Ca_22950	STOP_GAINED	NA
Kabuli_wild_206562	1399943745	*Ca_kabuli_scaffold*	206562	A/G	Gene_Ca_22950	NON_SYNONYMOUS_CODING	NA
Kabuli_wild_303784	1399943746	*Ca_kabuli_scaffold*	303784	A/C	Gene_Ca_22952	INTRON	Sulfatase
Kabuli_wild_305694	1399943747	*Ca_kabuli_scaffold*	305694	T/C	Gene_Ca_22952	INTRON	Sulfatase
Kabuli_wild_311419	1399943748	*Ca_kabuli_scaffold*	311419	A/G	Gene_Ca_22952	INTRON	Sulfatase
Kabuli_wild_332593	1399943749	*Ca_kabuli_scaffold*	332593	T/C	NA	INTERGENIC	NA
Kabuli_wild_332673	1399943750	*Ca_kabuli_scaffold*	332673	T/C	NA	INTERGENIC	NA
Kabuli_wild_12249	1399943751	*Ca_kabuli_scaffold*	12249	T/A	NA	INTERGENIC	NA
Kabuli_wild_12259	1399943752	*Ca_kabuli_scaffold*	12259	A/G	NA	INTERGENIC	NA
Kabuli_wild_12263	1399943753	*Ca_kabuli_scaffold*	12263	C/A	NA	INTERGENIC	NA
Kabuli_wild_12279	1399943754	*Ca_kabuli_scaffold*	12279	G/A	NA	INTERGENIC	NA
Kabuli_wild_12281	1399943755	*Ca_kabuli_scaffold*	12281	G/A	NA	INTERGENIC	NA
Kabuli_wild_137095	1399943756	*Ca_kabuli_scaffold*	137095	G/A	NA	INTERGENIC	NA
Kabuli_wild_241668	1399943757	*Ca_kabuli_scaffold*	241668	C/T	NA	INTERGENIC	NA
Kabuli_wild_241680	1399943758	*Ca_kabuli_scaffold*	241680	G/A	NA	INTERGENIC	NA
Kabuli_wild_241715	1399943759	*Ca_kabuli_scaffold*	241715	C/T	NA	INTERGENIC	NA
Kabuli_wild_28658	1399943760	*Ca_kabuli_scaffold*	28658	T/C	NA	INTERGENIC	NA
Kabuli_wild_28689	1399943761	*Ca_kabuli_scaffold*	28689	T/C	NA	INTERGENIC	NA
Kabuli_wild_28755	1399943762	*Ca_kabuli_scaffold*	28755	C/T	NA	INTERGENIC	NA
Kabuli_wild_28758	1399943763	*Ca_kabuli_scaffold*	28758	T/C	NA	INTERGENIC	NA
Kabuli_wild_28761	1399943764	*Ca_kabuli_scaffold*	28761	C/T	NA	INTERGENIC	NA
Kabuli_wild_28772	1399943765	*Ca_kabuli_scaffold*	28772	G/A	NA	INTERGENIC	NA
Kabuli_wild_28776	1399943766	*Ca_kabuli_scaffold*	28776	T/G	NA	INTERGENIC	NA
Kabuli_wild_28809	1399943767	*Ca_kabuli_scaffold*	28809	A/T	NA	INTERGENIC	NA
Kabuli_wild_28816	1399943768	*Ca_kabuli_scaffold*	28816	G/C	NA	INTERGENIC	NA
Kabuli_wild_52844	1399943769	*Ca_kabuli_scaffold*	52844	A/T	NA	INTERGENIC	NA
Kabuli_wild_52855	1399943770	*Ca_kabuli_scaffold*	52855	G/A	NA	INTERGENIC	NA
Kabuli_wild_52901	1399943771	*Ca_kabuli_scaffold*	52901	T/C	NA	INTERGENIC	NA

SNP IDs	NCBI dbSNP accession IDs (NCBI_SS#)	Chromosomes	Physical positions (bp)	SNPs	Gene accession IDs	Sequence components of genome	Putative functions
Kabuli_wild_52913	1399943772	*Ca_kabuli_scaffold*	52913	A/C	NA	INTERGENIC	NA
Kabuli_wild_61225	1399943773	*Ca_kabuli_scaffold*	61225	T/G	NA	INTERGENIC	NA
Kabuli_wild_61292	1399943774	*Ca_kabuli_scaffold*	61292	G/C	NA	INTERGENIC	NA
Kabuli_wild_61299	1399943775	*Ca_kabuli_scaffold*	61299	T/A	NA	INTERGENIC	NA
Kabuli_wild_65328	1399943776	*Ca_kabuli_scaffold*	65328	A/G	NA	INTERGENIC	NA
Kabuli_wild_162642	1399943777	*Ca_kabuli_scaffold*	162642	T/C	NA	INTERGENIC	NA
Kabuli_wild_263572	1399943778	*Ca_kabuli_scaffold*	263572	T/G	NA	INTERGENIC	NA
Kabuli_wild_263586	1399943779	*Ca_kabuli_scaffold*	263586	T/A	NA	INTERGENIC	NA
Kabuli_wild_334090	1399943780	*Ca_kabuli_scaffold*	334090	C/T	NA	INTERGENIC	NA
Kabuli_wild_334129	1399943781	*Ca_kabuli_scaffold*	334129	G/A	NA	INTERGENIC	NA
Kabuli_wild_334180	1399943782	*Ca_kabuli_scaffold*	334180	G/T	NA	INTERGENIC	NA
Kabuli_wild_408580	1399943783	*Ca_kabuli_scaffold*	408580	C/T	NA	INTERGENIC	NA
Kabuli_wild_408592	1399943784	*Ca_kabuli_scaffold*	408592	T/C	NA	INTERGENIC	NA
Kabuli_wild_408593	1399943785	*Ca_kabuli_scaffold*	408593	G/C	NA	INTERGENIC	NA
Kabuli_wild_408603	1399943786	*Ca_kabuli_scaffold*	408603	G/A	NA	INTERGENIC	NA
Kabuli_wild_408656	1399943787	*Ca_kabuli_scaffold*	408656	G/A	NA	INTERGENIC	NA
Kabuli_wild_593782	1399943788	*Ca_kabuli_scaffold*	593782	T/C	NA	INTERGENIC	NA
Kabuli_wild_593989	1399943789	*Ca_kabuli_scaffold*	593989	T/C	NA	INTERGENIC	NA
Kabuli_wild_860970	1399943790	*Ca_kabuli_scaffold*	860970	A/G	Gene_Ca_21452	INTRON	Helicase,C-terminal
Kabuli_wild_860975	1399943791	*Ca_kabuli_scaffold*	860975	G/A	Gene_Ca_21452	INTRON	Helicase,C-terminal
Kabuli_wild_860981	1399943792	*Ca_kabuli_scaffold*	860981	T/A	Gene_Ca_21452	INTRON	Helicase,C-terminal
Kabuli_wild_860983	1399943793	*Ca_kabuli_scaffold*	860983	A/T	Gene_Ca_21452	INTRON	Helicase,C-terminal
Kabuli_wild_860987	1399943794	*Ca_kabuli_scaffold*	860987	C/T	Gene_Ca_21452	INTRON	Helicase,C-terminal
Kabuli_wild_861003	1399943795	*Ca_kabuli_scaffold*	861003	C/A	Gene_Ca_21452	INTRON	Helicase,C-terminal
Kabuli_wild_861010	1399943796	*Ca_kabuli_scaffold*	861010	C/T	Gene_Ca_21452	INTRON	Helicase,C-terminal
Kabuli_wild_861011	1399943797	*Ca_kabuli_scaffold*	861011	C/T	Gene_Ca_21452	INTRON	Helicase,C-terminal
Kabuli_wild_861015	1399943798	*Ca_kabuli_scaffold*	861015	C/T	Gene_Ca_21452	INTRON	Helicase,C-terminal
Kabuli_wild_861022	1399943799	*Ca_kabuli_scaffold*	861022	T/C	Gene_Ca_21452	INTRON	Helicase,C-terminal
Kabuli_wild_861028	1399943800	*Ca_kabuli_scaffold*	861028	T/C	Gene_Ca_21452	INTRON	Helicase,C-terminal
Kabuli_wild_861053	1399943801	*Ca_kabuli_scaffold*	861053	G/A	Gene_Ca_21452	INTRON	Helicase,C-terminal
Kabuli_wild_861071	1399943802	*Ca_kabuli_scaffold*	861071	G/C	Gene_Ca_21452	INTRON	Helicase,C-terminal
Kabuli_wild_887160	1399943803	*Ca_kabuli_scaffold*	887160	A/C	NA	INTERGENIC	NA
Kabuli_wild_887173	1399943804	*Ca_kabuli_scaffold*	887173	C/T	NA	INTERGENIC	NA
Kabuli_wild_20778	1399943805	*Ca_kabuli_scaffold*	20778	G/A	NA	INTERGENIC	NA
Kabuli_wild_20938	1399943806	*Ca_kabuli_scaffold*	20938	C/T	NA	INTERGENIC	NA
Kabuli_wild_153328	1399943807	*Ca_kabuli_scaffold*	153328	T/C	NA	INTERGENIC	NA
Kabuli_wild_153348	1399943808	*Ca_kabuli_scaffold*	153348	T/A	NA	INTERGENIC	NA
Kabuli_wild_153361	1399943809	*Ca_kabuli_scaffold*	153361	A/G	NA	INTERGENIC	NA
Kabuli_wild_153364	1399943810	*Ca_kabuli_scaffold*	153364	A/G	NA	INTERGENIC	NA
Kabuli_wild_153371	1399943811	*Ca_kabuli_scaffold*	153371	C/T	NA	INTERGENIC	NA
Kabuli_wild_218244	1399943812	*Ca_kabuli_scaffold*	218244	T/G	Gene_Ca_22581	NON_SYNONYMOUS_CODING	SET domain
Kabuli_wild_218307	1399943813	*Ca_kabuli_scaffold*	218307	T/G	Gene_Ca_22581	NON_SYNONYMOUS_CODING	SET domain
Kabuli_wild_218336	1399943814	*Ca_kabuli_scaffold*	218336	T/G	Gene_Ca_22581	NON_SYNONYMOUS_CODING	SET domain
Kabuli_wild_218352	1399943815	*Ca_kabuli_scaffold*	218352	T/G	Gene_Ca_22581	NON_SYNONYMOUS_CODING	SET domain

SNP IDs	NCBI dbSNP accession IDs (NCBI_SS#)	Chromosomes	Physical positions (bp)	SNPs	Gene accession IDs	Sequence components of genome	Putative functions
Kabuli_wild_218356	1399943816	*Ca_kabuli_scaffold*	218356	G/T	Gene_Ca_22581	NON_SYNONYMOUS_CODING	SET domain
Kabuli_wild_218359	1399943817	*Ca_kabuli_scaffold*	218359	T/G	Gene_Ca_22581	NON_SYNONYMOUS_CODING	SET domain
Kabuli_wild_223318	1399943818	*Ca_kabuli_scaffold*	223318	C/T	Gene_Ca_22581	INTRON	SET domain
Kabuli_wild_463570	1399943819	*Ca_kabuli_scaffold*	463570	G/T	NA	INTERGENIC	NA
Kabuli_wild_18492	1399943820	*Ca_kabuli_scaffold*	18492	A/G	NA	INTERGENIC	NA
Kabuli_wild_266998	1399943821	*Ca_kabuli_scaffold*	266998	G/A	Gene_Ca_25631	NON_SYNONYMOUS_CODING	LETM1-like
Kabuli_wild_267060	1399943822	*Ca_kabuli_scaffold*	267060	T/G	Gene_Ca_25631	SYNONYMOUS_CODING	LETM1-like
Kabuli_wild_275743	1399943823	*Ca_kabuli_scaffold*	275743	T/A	Gene_Ca_25631	NON_SYNONYMOUS_CODING	LETM1-like
Kabuli_wild_100228	1399943824	*Ca_kabuli_scaffold*	100228	A/G	Gene_Ca_21065	SYNONYMOUS_CODING	RNA recognition motif domain
Kabuli_wild_100364	1399943825	*Ca_kabuli_scaffold*	100364	A/T	Gene_Ca_21065	INTRON	RNA recognition motif domain
Kabuli_wild_100529	1399943826	*Ca_kabuli_scaffold*	100529	T/C	Gene_Ca_21065	NON_SYNONYMOUS_CODING	RNA recognition motif domain
Kabuli_wild_103027	1399943827	*Ca_kabuli_scaffold*	103027	T/C	Gene_Ca_21065	SYNONYMOUS_CODING	RNA recognition motif domain
Kabuli_wild_106331	1399943828	*Ca_kabuli_scaffold*	106331	G/A	Gene_Ca_21065	INTRON	RNA recognition motif domain
Kabuli_wild_106349	1399943829	*Ca_kabuli_scaffold*	106349	C/T	Gene_Ca_21065	INTRON	RNA recognition motif domain
Kabuli_wild_142843	1399943830	*Ca_kabuli_scaffold*	142843	T/C	NA	INTERGENIC	NA
Kabuli_wild_151081	1399943831	*Ca_kabuli_scaffold*	151081	G/A	Gene_Ca_21070	NON_SYNONYMOUS_CODING	Zinc finger,DHHC-type,palmitoyltransferase
Kabuli_wild_151131	1399943832	*Ca_kabuli_scaffold*	151131	T/A	Gene_Ca_21070	SYNONYMOUS_CODING	Zinc finger,DHHC-type,palmitoyltransferase
Kabuli_wild_151177	1399943833	*Ca_kabuli_scaffold*	151177	A/G	Gene_Ca_21070	INTRON	Zinc finger,DHHC-type,palmitoyltransferase
Kabuli_wild_151192	1399943834	*Ca_kabuli_scaffold*	151192	T/C	Gene_Ca_21070	INTRON	Zinc finger,DHHC-type,palmitoyltransferase
Kabuli_wild_151197	1399943835	*Ca_kabuli_scaffold*	151197	T/A	Gene_Ca_21070	INTRON	Zinc finger,DHHC-type,palmitoyltransferase
Kabuli_wild_151199	1399943836	*Ca_kabuli_scaffold*	151199	T/A	Gene_Ca_21070	INTRON	Zinc finger,DHHC-type,palmitoyltransferase
Kabuli_wild_213380	1399943837	*Ca_kabuli_scaffold*	213380	C/G	Gene_Ca_21071	NON_SYNONYMOUS_CODING	Mitochondrialsubstrate/solutecarrier
Kabuli_wild_213398	1399943838	*Ca_kabuli_scaffold*	213398	T/G	Gene_Ca_21071	NON_SYNONYMOUS_CODING	Mitochondrialsubstrate/solutecarrier
Kabuli_wild_213404	1399943839	*Ca_kabuli_scaffold*	213404	G/T	Gene_Ca_21071	SYNONYMOUS_CODING	Mitochondrialsubstrate/solutecarrier
Kabuli_wild_213477	1399943840	*Ca_kabuli_scaffold*	213477	G/A	Gene_Ca_21071	INTRON	Mitochondrialsubstrate/solutecarrier
Kabuli_wild_213487	1399943841	*Ca_kabuli_scaffold*	213487	G/C	Gene_Ca_21071	INTRON	Mitochondrialsubstrate/solutecarrier
Kabuli_wild_213518	1399943842	*Ca_kabuli_scaffold*	213518	T/C	Gene_Ca_21071	INTRON	Mitochondrialsubstrate/solutecarrier
Kabuli_wild_229900	1399943843	*Ca_kabuli_scaffold*	229900	G/A	NA	INTERGENIC	NA
Kabuli_wild_229917	1399943844	*Ca_kabuli_scaffold*	229917	G/A	NA	INTERGENIC	NA
Kabuli_wild_321125	1399943845	*Ca_kabuli_scaffold*	321125	A/G	NA	INTERGENIC	NA
Kabuli_wild_321137	1399943846	*Ca_kabuli_scaffold*	321137	C/T	NA	INTERGENIC	NA
Kabuli_wild_321306	1399943847	*Ca_kabuli_scaffold*	321306	G/A	NA	INTERGENIC	NA
Kabuli_wild_321352	1399943848	*Ca_kabuli_scaffold*	321352	G/A	NA	INTERGENIC	NA
Kabuli_wild_321386	1399943849	*Ca_kabuli_scaffold*	321386	C/T	NA	INTERGENIC	NA
Kabuli_wild_326434	1399943850	*Ca_kabuli_scaffold*	326434	A/G	Gene_Ca_21078	INTRON	Protein of unknown function DUF3755
Kabuli_wild_326467	1399943851	*Ca_kabuli_scaffold*	326467	C/T	Gene_Ca_21078	INTRON	Protein of unknown function DUF3755
Kabuli_wild_326494	1399943852	*Ca_kabuli_scaffold*	326494	A/G	Gene_Ca_21078	INTRON	Protein of unknown function DUF3755
Kabuli_wild_326497	1399943853	*Ca_kabuli_scaffold*	326497	A/G	Gene_Ca_21078	INTRON	Protein of unknown function DUF3755
Kabuli_wild_352687	1399943854	*Ca_kabuli_scaffold*	352687	C/T	Gene_Ca_21079	INTRON	DEPdomain
Kabuli_wild_352712	1399943855	*Ca_kabuli_scaffold*	352712	G/A	Gene_Ca_21079	INTRON	DEPdomain
Kabuli_wild_352747	1399943856	*Ca_kabuli_scaffold*	352747	T/C	Gene_Ca_21079	INTRON	DEPdomain
Kabuli_wild_355222	1399943857	*Ca_kabuli_scaffold*	355222	G/A	Gene_Ca_21079	INTRON	DEPdomain
Kabuli_wild_469198	1399943858	*Ca_kabuli_scaffold*	469198	A/C	NA	INTERGENIC	NA
Kabuli_wild_512979	1399943859	*Ca_kabuli_scaffold*	512979	A/T	NA	INTERGENIC	NA

SNP IDs	NCBI dbSNP accession IDs (NCBI_SS#)	Chromosomes	Physical positions (bp)	SNPs	Gene accession IDs	Sequence components of genome	Putative functions
Kabuli_wild_512985	1399943860	*Ca_kabuli_scaffold*	512985	T/C	NA	INTERGENIC	NA
Kabuli_wild_513004	1399943861	*Ca_kabuli_scaffold*	513004	A/G	NA	INTERGENIC	NA
Kabuli_wild_534991	1399943862	*Ca_kabuli_scaffold*	534991	G/A	NA	INTERGENIC	NA
Kabuli_wild_535016	1399943863	*Ca_kabuli_scaffold*	535016	A/C	NA	INTERGENIC	NA
Kabuli_wild_535017	1399943864	*Ca_kabuli_scaffold*	535017	G/A	NA	INTERGENIC	NA
Kabuli_wild_535027	1399943865	*Ca_kabuli_scaffold*	535027	A/G	NA	INTERGENIC	NA
Kabuli_wild_535057	1399943866	*Ca_kabuli_scaffold*	535057	G/T	NA	INTERGENIC	NA
Kabuli_wild_535075	1399943867	*Ca_kabuli_scaffold*	535075	T/C	NA	INTERGENIC	NA
Kabuli_wild_535108	1399943868	*Ca_kabuli_scaffold*	535108	C/T	NA	INTERGENIC	NA
Kabuli_wild_535111	1399943869	*Ca_kabuli_scaffold*	535111	G/A	NA	INTERGENIC	NA
Kabuli_wild_295314	1399943870	*Ca_kabuli_scaffold*	295314	C/T	NA	INTERGENIC	NA
Kabuli_wild_10891	1399943871	*Ca_kabuli_scaffold*	10891	G/C	NA	INTERGENIC	NA
Kabuli_wild_10971	1399943872	*Ca_kabuli_scaffold*	10971	G/A	NA	INTERGENIC	NA
Kabuli_wild_11009	1399943873	*Ca_kabuli_scaffold*	11009	A/T	NA	INTERGENIC	NA
Kabuli_wild_11018	1399943874	*Ca_kabuli_scaffold*	11018	T/A	NA	INTERGENIC	NA
Kabuli_wild_73944	1399943875	*Ca_kabuli_scaffold*	73944	G/T	NA	INTERGENIC	NA
Kabuli_wild_137848	1399943876	*Ca_kabuli_scaffold*	137848	A/G	NA	INTERGENIC	NA
Kabuli_wild_137869	1399943877	*Ca_kabuli_scaffold*	137869	C/T	NA	INTERGENIC	NA
Kabuli_wild_239687	1399943878	*Ca_kabuli_scaffold*	239687	G/A	NA	INTERGENIC	NA
Kabuli_wild_303202	1399943879	*Ca_kabuli_scaffold*	303202	G/A	NA	INTERGENIC	NA
Kabuli_wild_303222	1399943880	*Ca_kabuli_scaffold*	303222	T/G	NA	INTERGENIC	NA
Kabuli_wild_327841	1399943881	*Ca_kabuli_scaffold*	327841	G/A	NA	INTERGENIC	NA
Kabuli_wild_327856	1399943882	*Ca_kabuli_scaffold*	327856	G/A	NA	INTERGENIC	NA
Kabuli_wild_96763	1399943883	*Ca_kabuli_scaffold*	96763	T/C	Gene_Ca_25945	SYNONYMOUS_CODING	NA
Kabuli_wild_183718	1399943884	*Ca_kabuli_scaffold*	183718	T/C	Gene_Ca_25948	INTRON	Short-chaindehydrogenase/reductaseSDR
Kabuli_wild_183720	1399943885	*Ca_kabuli_scaffold*	183720	C/T	Gene_Ca_25948	INTRON	Short-chaindehydrogenase/reductaseSDR
Kabuli_wild_183721	1399943886	*Ca_kabuli_scaffold*	183721	G/A	Gene_Ca_25948	INTRON	Short-chaindehydrogenase/reductaseSDR
Kabuli_wild_183727	1399943887	*Ca_kabuli_scaffold*	183727	C/T	Gene_Ca_25948	INTRON	Short-chaindehydrogenase/reductaseSDR
Kabuli_wild_183752	1399943888	*Ca_kabuli_scaffold*	183752	T/G	Gene_Ca_25948	INTRON	Short-chaindehydrogenase/reductaseSDR
Kabuli_wild_183755	1399943889	*Ca_kabuli_scaffold*	183755	T/G	Gene_Ca_25948	INTRON	Short-chaindehydrogenase/reductaseSDR
Kabuli_wild_183759	1399943890	*Ca_kabuli_scaffold*	183759	T/C	Gene_Ca_25948	INTRON	Short-chaindehydrogenase/reductaseSDR
Kabuli_wild_186119	1399943891	*Ca_kabuli_scaffold*	186119	T/C	Gene_Ca_25948	NON_SYNONYMOUS_CODING	Short-chaindehydrogenase/reductaseSDR
Kabuli_wild_186125	1399943892	*Ca_kabuli_scaffold*	186125	C/A	Gene_Ca_25948	NON_SYNONYMOUS_CODING	Short-chaindehydrogenase/reductaseSDR
Kabuli_wild_208050	1399943893	*Ca_kabuli_scaffold*	208050	A/G	Gene_Ca_25951	DOWNSTREAM	NA
Kabuli_wild_209964	1399943894	*Ca_kabuli_scaffold*	209964	G/T	NA	INTERGENIC	NA
Kabuli_wild_44014	1399943895	*Ca_kabuli_scaffold*	44014	T/G	NA	INTERGENIC	NA
Kabuli_wild_47952	1399943896	*Ca_kabuli_scaffold*	47952	A/G	NA	INTERGENIC	NA
Kabuli_wild_61454	1399943897	*Ca_kabuli_scaffold*	61454	T/C	Gene_Ca_21706	NON_SYNONYMOUS_CODING	Protein kinase,catalytic domain
Kabuli_wild_263899	1399943898	*Ca_kabuli_scaffold*	263899	G/A	Gene_Ca_21715	NON_SYNONYMOUS_CODING	RNA polymerase,alpha subunit
Kabuli_wild_335077	1399943899	*Ca_kabuli_scaffold*	335077	C/T	Gene_Ca_21719	INTRON	RNA polymerase,alpha subunit
Kabuli_wild_341322	1399943900	*Ca_kabuli_scaffold*	341322	C/T	Gene_Ca_21719	INTRON	RNA polymerase,alpha subunit
Kabuli_wild_414576	1399943901	*Ca_kabuli_scaffold*	414576	C/T	NA	INTERGENIC	NA
Kabuli_wild_414602	1399943902	*Ca_kabuli_scaffold*	414602	C/T	NA	INTERGENIC	NA
Kabuli_wild_457627	1399943903	*Ca_kabuli_scaffold*	457627	G/A	Gene_Ca_21723	INTRON	NA

SNP IDs	NCBI dbSNP accession IDs (NCBI_SS#)	Chromosomes	Physical positions (bp)	SNPs	Gene accession IDs	Sequence components of genome	Putative functions
Kabuli_wild_11965	1399943904	*Ca_kabuli_scaffold*	11965	A/G	NA	INTERGENIC	NA
Kabuli_wild_173228	1399943905	*Ca_kabuli_scaffold*	173228	T/C	Gene_Ca_24795	INTRON	Domain of unknown function DUF632
Kabuli_wild_279942	1399943906	*Ca_kabuli_scaffold*	279942	A/G	NA	INTERGENIC	NA
Kabuli_wild_280009	1399943907	*Ca_kabuli_scaffold*	280009	G/T	NA	INTERGENIC	NA
Kabuli_wild_280015	1399943908	*Ca_kabuli_scaffold*	280015	T/C	NA	INTERGENIC	NA
Kabuli_wild_280023	1399943909	*Ca_kabuli_scaffold*	280023	C/T	NA	INTERGENIC	NA
Kabuli_wild_280047	1399943910	*Ca_kabuli_scaffold*	280047	A/G	NA	INTERGENIC	NA
Kabuli_wild_280062	1399943911	*Ca_kabuli_scaffold*	280062	T/C	NA	INTERGENIC	NA
Kabuli_wild_408886	1399943912	*Ca_kabuli_scaffold*	408886	G/C	NA	INTERGENIC	NA
Kabuli_wild_38279	1399943913	*Ca_kabuli_scaffold*	38279	C/A	NA	INTERGENIC	NA
Kabuli_wild_58352	1399943914	*Ca_kabuli_scaffold*	58352	T/C	Gene_Ca_25163	NON_SYNONYMOUS_CODING	tRNA-binding domain
Kabuli_wild_186486	1399943915	*Ca_kabuli_scaffold*	186486	G/A	NA	INTERGENIC	NA
Kabuli_wild_186510	1399943916	*Ca_kabuli_scaffold*	186510	C/A	NA	INTERGENIC	NA
Kabuli_wild_186515	1399943917	*Ca_kabuli_scaffold*	186515	T/G	NA	INTERGENIC	NA
Kabuli_wild_186523	1399943918	*Ca_kabuli_scaffold*	186523	T/C	NA	INTERGENIC	NA
Kabuli_wild_186538	1399943919	*Ca_kabuli_scaffold*	186538	C/T	NA	INTERGENIC	NA
Kabuli_wild_186582	1399943920	*Ca_kabuli_scaffold*	186582	C/A	NA	INTERGENIC	NA
Kabuli_wild_186587	1399943921	*Ca_kabuli_scaffold*	186587	G/T	NA	INTERGENIC	NA
Kabuli_wild_186588	1399943922	*Ca_kabuli_scaffold*	186588	C/G	NA	INTERGENIC	NA
Kabuli_wild_186595	1399943923	*Ca_kabuli_scaffold*	186595	C/T	NA	INTERGENIC	NA
Kabuli_wild_186598	1399943924	*Ca_kabuli_scaffold*	186598	A/G	NA	INTERGENIC	NA
Kabuli_wild_186604	1399943925	*Ca_kabuli_scaffold*	186604	T/C	NA	INTERGENIC	NA
Kabuli_wild_186611	1399943926	*Ca_kabuli_scaffold*	186611	G/A	NA	INTERGENIC	NA
Kabuli_wild_186639	1399943927	*Ca_kabuli_scaffold*	186639	C/T	NA	INTERGENIC	NA
Kabuli_wild_186651	1399943928	*Ca_kabuli_scaffold*	186651	T/C	NA	INTERGENIC	NA
Kabuli_wild_186657	1399943929	*Ca_kabuli_scaffold*	186657	G/A	NA	INTERGENIC	NA
Kabuli_wild_186661	1399943930	*Ca_kabuli_scaffold*	186661	T/C	NA	INTERGENIC	NA
Kabuli_wild_242733	1399943931	*Ca_kabuli_scaffold*	242733	G/T	NA	INTERGENIC	NA
Kabuli_wild_242763	1399943932	*Ca_kabuli_scaffold*	242763	T/C	NA	INTERGENIC	NA
Kabuli_wild_399283	1399943933	*Ca_kabuli_scaffold*	399283	C/A	NA	INTERGENIC	NA
Kabuli_wild_399301	1399943934	*Ca_kabuli_scaffold*	399301	G/A	NA	INTERGENIC	NA
Kabuli_wild_399326	1399943935	*Ca_kabuli_scaffold*	399326	G/A	NA	INTERGENIC	NA
Kabuli_wild_399340	1399943936	*Ca_kabuli_scaffold*	399340	G/A	NA	INTERGENIC	NA
Kabuli_wild_188896	1399943937	*Ca_kabuli_scaffold*	188896	G/T	NA	INTERGENIC	NA
Kabuli_wild_188922	1399943938	*Ca_kabuli_scaffold*	188922	C/T	NA	INTERGENIC	NA
Kabuli_wild_188926	1399943939	*Ca_kabuli_scaffold*	188926	G/A	NA	INTERGENIC	NA
Kabuli_wild_424558	1399943940	*Ca_kabuli_scaffold*	424558	G/T	NA	INTERGENIC	NA
Kabuli_wild_586081	1399943941	*Ca_kabuli_scaffold*	586081	C/A	Gene_Ca_20922	NON_SYNONYMOUS_CODING	Ribokinase
Kabuli_wild_631125	1399943942	*Ca_kabuli_scaffold*	631125	G/A	NA	INTERGENIC	NA
Kabuli_wild_631140	1399943943	*Ca_kabuli_scaffold*	631140	G/A	NA	INTERGENIC	NA
Kabuli_wild_631160	1399943944	*Ca_kabuli_scaffold*	631160	G/A	NA	INTERGENIC	NA
Kabuli_wild_631174	1399943945	*Ca_kabuli_scaffold*	631174	C/T	NA	INTERGENIC	NA
Kabuli_wild_631175	1399943946	*Ca_kabuli_scaffold*	631175	C/A	NA	INTERGENIC	NA
Kabuli_wild_631185	1399943947	*Ca_kabuli_scaffold*	631185	G/A	NA	INTERGENIC	NA

SNP IDs	NCBI dbSNP accession IDs (NCBI_SS#)	Chromosomes	Physical positions (bp)	SNPs	Gene accession IDs	Sequence components of genome	Putative functions
Kabuli_wild_631239	1399943948	*Ca_kabuli_scaffold*	631239	T/C	NA	INTERGENIC	NA
Kabuli_wild_631260	1399943949	*Ca_kabuli_scaffold*	631260	G/A	NA	INTERGENIC	NA
Kabuli_wild_631264	1399943950	*Ca_kabuli_scaffold*	631264	C/T	NA	INTERGENIC	NA
Kabuli_wild_631265	1399943951	*Ca_kabuli_scaffold*	631265	G/A	NA	INTERGENIC	NA
Kabuli_wild_631271	1399943952	*Ca_kabuli_scaffold*	631271	T/G	NA	INTERGENIC	NA
Kabuli_wild_641243	1399943953	*Ca_kabuli_scaffold*	641243	A/G	NA	INTERGENIC	NA
Kabuli_wild_641252	1399943954	*Ca_kabuli_scaffold*	641252	A/G	NA	INTERGENIC	NA
Kabuli_wild_641266	1399943955	*Ca_kabuli_scaffold*	641266	C/T	NA	INTERGENIC	NA
Kabuli_wild_108985	1399943956	*Ca_kabuli_scaffold*	108985	G/T	NA	INTERGENIC	NA
Kabuli_wild_64030	1399943957	*Ca_kabuli_scaffold*	64030	A/G	Gene_Ca_27125	INTRON	NA
Kabuli_wild_92532	1399943958	*Ca_kabuli_scaffold*	92532	C/A	Gene_Ca_27126	INTRON	Vacuolar protein sorting-associated protein
Kabuli_wild_96281	1399943959	*Ca_kabuli_scaffold*	96281	G/A	Gene_Ca_27126	INTRON	Vacuolar protein sorting-associated protein
Kabuli_wild_96333	1399943960	*Ca_kabuli_scaffold*	96333	G/A	Gene_Ca_27126	INTRON	Vacuolar protein sorting-associated protein
Kabuli_wild_96363	1399943961	*Ca_kabuli_scaffold*	96363	T/G	Gene_Ca_27126	INTRON	Vacuolar protein sorting-associated protein
Kabuli_wild_96394	1399943962	*Ca_kabuli_scaffold*	96394	T/A	Gene_Ca_27126	INTRON	Vacuolar protein sorting-associated protein
Kabuli_wild_96397	1399943963	*Ca_kabuli_scaffold*	96397	G/A	Gene_Ca_27126	INTRON	Vacuolar protein sorting-associated protein
Kabuli_wild_96444	1399943964	*Ca_kabuli_scaffold*	96444	G/A	Gene_Ca_27126	INTRON	Vacuolar protein sorting-associated protein
Kabuli_wild_96456	1399943965	*Ca_kabuli_scaffold*	96456	T/C	Gene_Ca_27126	INTRON	Vacuolar protein sorting-associated protein
Kabuli_wild_96464	1399943966	*Ca_kabuli_scaffold*	96464	T/G	Gene_Ca_27126	INTRON	Vacuolar protein sorting-associated protein
Kabuli_wild_96532	1399943967	*Ca_kabuli_scaffold*	96532	G/T	Gene_Ca_27126	INTRON	Vacuolar protein sorting-associated protein
Kabuli_wild_96580	1399943968	*Ca_kabuli_scaffold*	96580	T/G	Gene_Ca_27126	INTRON	Vacuolar protein sorting-associated protein
Kabuli_wild_96589	1399943969	*Ca_kabuli_scaffold*	96589	A/T	Gene_Ca_27126	INTRON	Vacuolar protein sorting-associated protein
Kabuli_wild_99123	1399943970	*Ca_kabuli_scaffold*	99123	C/T	Gene_Ca_27127	DOWNSTREAM	Vacuolar protein sorting-associated protein Like-Smribonucleo protein (LSM) domain
Kabuli_wild_74397	1399943971	*Ca_kabuli_scaffold*	74397	G/A	NA	INTERGENIC	NA
Kabuli_wild_74399	1399943972	*Ca_kabuli_scaffold*	74399	C/T	NA	INTERGENIC	NA
Kabuli_wild_74424	1399943973	*Ca_kabuli_scaffold*	74424	T/C	NA	INTERGENIC	NA
Kabuli_wild_74430	1399943974	*Ca_kabuli_scaffold*	74430	A/G	NA	INTERGENIC	NA
Kabuli_wild_74432	1399943975	*Ca_kabuli_scaffold*	74432	G/A	NA	INTERGENIC	NA
Kabuli_wild_74448	1399943976	*Ca_kabuli_scaffold*	74448	T/G	NA	INTERGENIC	NA
Kabuli_wild_143338	1399943977	*Ca_kabuli_scaffold*	143338	T/C	NA	INTERGENIC	NA
Kabuli_wild_143353	1399943978	*Ca_kabuli_scaffold*	143353	C/G	NA	INTERGENIC	NA
Kabuli_wild_143356	1399943979	*Ca_kabuli_scaffold*	143356	A/G	NA	INTERGENIC	NA
Kabuli_wild_143400	1399943980	*Ca_kabuli_scaffold*	143400	G/A	NA	INTERGENIC	NA
Kabuli_wild_143406	1399943981	*Ca_kabuli_scaffold*	143406	G/C	NA	INTERGENIC	NA
Kabuli_wild_143409	1399943982	*Ca_kabuli_scaffold*	143409	G/A	NA	INTERGENIC	NA
Kabuli_wild_58349	1399943983	*Ca_kabuli_scaffold*	58349	C/A	Gene_Ca_27283	INTRON	Peptidase C48, SUMO/Sentrin/Ubl1
Kabuli_wild_62985	1399943984	*Ca_kabuli_scaffold*	62985	G/A	Gene_Ca_27283	STOP_GAINED	Peptidase C48, SUMO/Sentrin/Ubl1
Kabuli_wild_344503	1399943985	*Ca_kabuli_scaffold*	344503	G/T	NA	INTERGENIC	NA
Kabuli_wild_344515	1399943986	*Ca_kabuli_scaffold*	344515	A/G	NA	INTERGENIC	NA
Kabuli_wild_344519	1399943987	*Ca_kabuli_scaffold*	344519	A/T	NA	INTERGENIC	NA
Kabuli_wild_344533	1399943988	*Ca_kabuli_scaffold*	344533	C/T	NA	INTERGENIC	NA
Kabuli_wild_344556	1399943989	*Ca_kabuli_scaffold*	344556	T/G	NA	INTERGENIC	NA
Kabuli_wild_59198	1399943990	*Ca_kabuli_scaffold*	59198	T/C	NA	INTERGENIC	NA
Kabuli_wild_59850	1399943991	*Ca_kabuli_scaffold*	59850	G/A	Gene_Ca_26104	SYNONYMOUS_CODING	NA

SNP IDs	NCBI dbSNP accession IDs (NCBI_SS#)	Chromosomes	Physical positions (bp)	SNPs	Gene accession IDs	Sequence components of genome	Putative functions
Kabuli_wild_404	1399943992	*Ca_kabuli_scaffold*	404	G/A	NA	INTERGENIC	NA
Kabuli_wild_434	1399943993	*Ca_kabuli_scaffold*	434	C/T	NA	INTERGENIC	NA
Kabuli_wild_449	1399943994	*Ca_kabuli_scaffold*	449	C/T	NA	INTERGENIC	NA
Kabuli_wild_615	1399943995	*Ca_kabuli_scaffold*	615	G/T	NA	INTERGENIC	NA
Kabuli_wild_293799	1399943996	*Ca_kabuli_scaffold*	293799	G/A	Gene_Ca_21905	STOP_GAINED	NA
Kabuli_wild_355284	1399943997	*Ca_kabuli_scaffold*	355284	G/T	Gene_Ca_21906	NON_SYNONYMOUS_CODING	Mitochondrial carrier protein
Kabuli_wild_374106	1399943998	*Ca_kabuli_scaffold*	374106	G/T	NA	INTERGENIC	NA
Kabuli_wild_374149	1399943999	*Ca_kabuli_scaffold*	374149	G/C	NA	INTERGENIC	NA
Kabuli_wild_533416	1399944000	*Ca_kabuli_scaffold*	533416	G/A	Gene_Ca_21912	NON_SYNONYMOUS_CODING	Peptidase C48, SUMO/Sentrin/Ubl1
Kabuli_wild_533417	1399944001	*Ca_kabuli_scaffold*	533417	C/T	Gene_Ca_21912	NON_SYNONYMOUS_CODING	Peptidase C48, SUMO/Sentrin/Ubl1
Kabuli_wild_533419	1399944002	*Ca_kabuli_scaffold*	533419	G/A	Gene_Ca_21912	NON_SYNONYMOUS_CODING	Peptidase C48, SUMO/Sentrin/Ubl1
Kabuli_wild_533441	1399944003	*Ca_kabuli_scaffold*	533441	G/T	Gene_Ca_21912	NON_SYNONYMOUS_CODING	Peptidase C48, SUMO/Sentrin/Ubl1
Kabuli_wild_533453	1399944004	*Ca_kabuli_scaffold*	533453	C/T	Gene_Ca_21912	NON_SYNONYMOUS_CODING	Peptidase C48, SUMO/Sentrin/Ubl1
Kabuli_wild_533501	1399944005	*Ca_kabuli_scaffold*	533501	C/T	Gene_Ca_21912	NON_SYNONYMOUS_CODING	Peptidase C48, SUMO/Sentrin/Ubl1
Kabuli_wild_617358	1399944006	*Ca_kabuli_scaffold*	617358	C/A	NA	INTERGENIC	NA
Kabuli_wild_617373	1399944007	*Ca_kabuli_scaffold*	617373	T/C	NA	INTERGENIC	NA
Kabuli_wild_53841	1399944008	*Ca_kabuli_scaffold*	53841	T/G	Gene_Ca_27612	SYNONYMOUS_CODING	AMP-dependentsynthetase/ligase
Kabuli_wild_131234	1399944009	*Ca_kabuli_scaffold*	131234	T/C	Gene_Ca_25621	NON_SYNONYMOUS_CODING	Protein of unknown function DUF1997
Kabuli_wild_156278	1399944010	*Ca_kabuli_scaffold*	156278	C/T	NA	INTERGENIC	NA
Kabuli_wild_173215	1399944011	*Ca_kabuli_scaffold*	173215	T/C	Gene_Ca_26343	INTRON	NA
Kabuli_wild_142718	1399944012	*Ca_kabuli_scaffold*	142718	G/T	Gene_Ca_25190	UPSTREAM	NA
Kabuli_wild_351868	1399944013	*Ca_kabuli_scaffold*	351868	G/A	Gene_Ca_25198	NON_SYNONYMOUS_CODING	NA
Kabuli_wild_127726	1399944014	*Ca_kabuli_scaffold*	127726	C/T	Gene_Ca_26966	SYNONYMOUS_CODING	Peptidase C48, SUMO/Sentrin/Ubl1
Kabuli_wild_127790	1399944015	*Ca_kabuli_scaffold*	127790	C/T	Gene_Ca_26966	NON_SYNONYMOUS_CODING	Peptidase C48, SUMO/Sentrin/Ubl1
Kabuli_wild_4781	1399944016	*Ca_kabuli_scaffold*	4781	A/G	NA	INTERGENIC	NA
Kabuli_wild_101349	1399944017	*Ca_kabuli_scaffold*	101349	T/G	NA	INTERGENIC	NA
Kabuli_wild_101416	1399944018	*Ca_kabuli_scaffold*	101416	A/G	NA	INTERGENIC	NA
Kabuli_wild_101417	1399944019	*Ca_kabuli_scaffold*	101417	A/G	NA	INTERGENIC	NA
Kabuli_wild_297700	1399944020	*Ca_kabuli_scaffold*	297700	C/G	NA	INTERGENIC	NA
Kabuli_wild_30857	1399944021	*Ca_kabuli_scaffold*	30857	A/G	NA	INTERGENIC	NA
Kabuli_wild_6152	1399944022	*Ca_kabuli_scaffold*	6152	C/T	NA	INTERGENIC	NA
Kabuli_wild_6193	1399944023	*Ca_kabuli_scaffold*	6193	G/A	NA	INTERGENIC	NA
Kabuli_wild_6201	1399944024	*Ca_kabuli_scaffold*	6201	G/A	NA	INTERGENIC	NA
Kabuli_wild_6231	1399944025	*Ca_kabuli_scaffold*	6231	T/A	NA	INTERGENIC	NA
Kabuli_wild_6242	1399944026	*Ca_kabuli_scaffold*	6242	C/A	NA	INTERGENIC	NA
Kabuli_wild_6248	1399944027	*Ca_kabuli_scaffold*	6248	T/G	NA	INTERGENIC	NA
Kabuli_wild_6252	1399944028	*Ca_kabuli_scaffold*	6252	G/A	NA	INTERGENIC	NA
Kabuli_wild_6254	1399944029	*Ca_kabuli_scaffold*	6254	C/A	NA	INTERGENIC	NA
Kabuli_wild_49847	1399944030	*Ca_kabuli_scaffold*	49847	G/A	Gene_Ca_25655	SYNONYMOUS_CODING	NA
Kabuli_wild_119119	1399944031	*Ca_kabuli_scaffold*	119119	C/T	Gene_Ca_25659	NON_SYNONYMOUS_CODING	Peptidase C48, SUMO/Sentrin/Ubl1
Kabuli_wild_119155	1399944032	*Ca_kabuli_scaffold*	119155	C/T	Gene_Ca_25659	NON_SYNONYMOUS_CODING	NA
Kabuli_wild_119157	1399944033	*Ca_kabuli_scaffold*	119157	G/A	Gene_Ca_25659	SYNONYMOUS_CODING	NA
Kabuli_wild_119171	1399944034	*Ca_kabuli_scaffold*	119171	G/A	Gene_Ca_25659	NON_SYNONYMOUS_CODING	NA
Kabuli_wild_119265	1399944035	*Ca_kabuli_scaffold*	119265	C/G	Gene_Ca_25659	SYNONYMOUS_CODING	NA

SNP IDs	NCBI dbSNP accession IDs (NCBI_SS#)	Chromosomes	Physical positions (bp)	SNPs	Gene accession IDs	Sequence components of genome	Putative functions
Kabuli_wild_119267	1399944036	*Ca_kabuli_scaffold*	119267	G/C	Gene_Ca_25659	NON_SYNONYMOUS_CODING	NA
Kabuli_wild_119269	1399944037	*Ca_kabuli_scaffold*	119269	C/T	Gene_Ca_25659	NON_SYNONYMOUS_CODING	NA
Kabuli_wild_119278	1399944038	*Ca_kabuli_scaffold*	119278	T/C	Gene_Ca_25659	NON_SYNONYMOUS_CODING	NA
Kabuli_wild_119297	1399944039	*Ca_kabuli_scaffold*	119297	C/A	Gene_Ca_25659	STOP_GAINED	NA
Kabuli_wild_119301	1399944040	*Ca_kabuli_scaffold*	119301	T/C	Gene_Ca_25659	SYNONYMOUS_CODING	NA
Kabuli_wild_58481	1399944041	*Ca_kabuli_scaffold*	58481	T/A	Gene_Ca_22259	INTRON	NA
Kabuli_wild_58689	1399944042	*Ca_kabuli_scaffold*	58689	A/T	Gene_Ca_22259	NON_SYNONYMOUS_CODING	NA
Kabuli_wild_84118	1399944043	*Ca_kabuli_scaffold*	84118	G/A	NA	INTERGENIC	NA
Kabuli_wild_293872	1399944044	*Ca_kabuli_scaffold*	293872	A/G	Gene_Ca_22265	INTRON	Raffinosesynthase
Kabuli_wild_6987	1399944045	*Ca_kabuli_scaffold*	6987	C/T	NA	INTERGENIC	NA
Kabuli_wild_7000	1399944046	*Ca_kabuli_scaffold*	7000	C/T	NA	INTERGENIC	NA
Kabuli_wild_7006	1399944047	*Ca_kabuli_scaffold*	7006	C/A	NA	INTERGENIC	NA
Kabuli_wild_7011	1399944048	*Ca_kabuli_scaffold*	7011	C/T	NA	INTERGENIC	NA
Kabuli_wild_7063	1399944049	*Ca_kabuli_scaffold*	7063	G/A	NA	INTERGENIC	NA
Kabuli_wild_7069	1399944050	*Ca_kabuli_scaffold*	7069	C/T	NA	INTERGENIC	NA
Kabuli_wild_38270	1399944051	*Ca_kabuli_scaffold*	38270	A/G	Gene_Ca_27037	INTRON	SH2motif
Kabuli_wild_78657	1399944052	*Ca_kabuli_scaffold*	78657	A/T	NA	INTERGENIC	NA
Kabuli_wild_78792	1399944053	*Ca_kabuli_scaffold*	78792	A/T	NA	INTERGENIC	NA
Kabuli_wild_78803	1399944054	*Ca_kabuli_scaffold*	78803	G/C	NA	INTERGENIC	NA
Kabuli_wild_78829	1399944055	*Ca_kabuli_scaffold*	78829	G/A	NA	INTERGENIC	NA
Kabuli_wild_78861	1399944056	*Ca_kabuli_scaffold*	78861	A/C	NA	INTERGENIC	NA
Kabuli_wild_226835	1399944057	*Ca_kabuli_scaffold*	226835	C/A	Gene_Ca_24581	INTRON	ChaperoninTCP-1,conservedsite
Kabuli_wild_84629	1399944058	*Ca_kabuli_scaffold*	84629	G/T	NA	INTERGENIC	NA
Kabuli_wild_84922	1399944059	*Ca_kabuli_scaffold*	84922	A/G	NA	INTERGENIC	NA
Kabuli_wild_84924	1399944060	*Ca_kabuli_scaffold*	84924	A/G	NA	INTERGENIC	NA
Kabuli_wild_84952	1399944061	*Ca_kabuli_scaffold*	84952	T/G	NA	INTERGENIC	NA
Kabuli_wild_84970	1399944062	*Ca_kabuli_scaffold*	84970	T/C	NA	INTERGENIC	NA
Kabuli_wild_85006	1399944063	*Ca_kabuli_scaffold*	85006	G/A	NA	INTERGENIC	NA
Kabuli_wild_2940	1399944064	*Ca_kabuli_scaffold*	2940	C/T	NA	INTERGENIC	NA
Kabuli_wild_2949	1399944065	*Ca_kabuli_scaffold*	2949	G/T	NA	INTERGENIC	NA
Kabuli_wild_3151	1399944066	*Ca_kabuli_scaffold*	3151	G/T	NA	INTERGENIC	NA
Kabuli_wild_3158	1399944067	*Ca_kabuli_scaffold*	3158	C/G	NA	INTERGENIC	NA
Kabuli_wild_3262	1399944068	*Ca_kabuli_scaffold*	3262	T/G	NA	INTERGENIC	NA
Kabuli_wild_87388	1399944069	*Ca_kabuli_scaffold*	87388	A/C	NA	INTERGENIC	NA
Kabuli_wild_87547	1399944070	*Ca_kabuli_scaffold*	87547	C/T	NA	INTERGENIC	NA
Kabuli_wild_87640	1399944071	*Ca_kabuli_scaffold*	87640	A/G	NA	INTERGENIC	NA
Kabuli_wild_87646	1399944072	*Ca_kabuli_scaffold*	87646	A/G	NA	INTERGENIC	NA
Kabuli_wild_87658	1399944073	*Ca_kabuli_scaffold*	87658	T/C	NA	INTERGENIC	NA
Kabuli_wild_202514	1399944074	*Ca_kabuli_scaffold*	202514	T/G	NA	INTERGENIC	NA
Kabuli_wild_208067	1399944075	*Ca_kabuli_scaffold*	208067	C/A	NA	INTERGENIC	NA
Kabuli_wild_208768	1399944076	*Ca_kabuli_scaffold*	208768	G/A	NA	INTERGENIC	NA
Kabuli_wild_208786	1399944077	*Ca_kabuli_scaffold*	208786	G/C	NA	INTERGENIC	NA
Kabuli_wild_208793	1399944078	*Ca_kabuli_scaffold*	208793	G/C	NA	INTERGENIC	NA
Kabuli_wild_208821	1399944079	*Ca_kabuli_scaffold*	208821	G/T	NA	INTERGENIC	NA

SNP IDs	NCBI dbSNP accession IDs (NCBI_SS#)	Chromosomes	Physical positions (bp)	SNPs	Gene accession IDs	Sequence components of genome	Putative functions
Kabuli_wild_208904	1399944080	*Ca_kabuli_scaffold*	208904	A/G	NA	INTERGENIC	NA
Kabuli_wild_208911	1399944081	*Ca_kabuli_scaffold*	208911	A/G	NA	INTERGENIC	NA
Kabuli_wild_208913	1399944082	*Ca_kabuli_scaffold*	208913	C/T	NA	INTERGENIC	NA
Kabuli_wild_208914	1399944083	*Ca_kabuli_scaffold*	208914	C/G	NA	INTERGENIC	NA
Kabuli_wild_208932	1399944084	*Ca_kabuli_scaffold*	208932	G/T	NA	INTERGENIC	NA
Kabuli_wild_208933	1399944085	*Ca_kabuli_scaffold*	208933	G/A	NA	INTERGENIC	NA
Kabuli_wild_208939	1399944086	*Ca_kabuli_scaffold*	208939	G/A	NA	INTERGENIC	NA
Kabuli_wild_215419	1399944087	*Ca_kabuli_scaffold*	215419	G/A	Gene_Ca_23459	SYNONYMOUS_CODING	NA
Kabuli_wild_230606	1399944088	*Ca_kabuli_scaffold*	230606	A/G	NA	INTERGENIC	NA
Kabuli_wild_231895	1399944089	*Ca_kabuli_scaffold*	231895	T/A	NA	INTERGENIC	NA
Kabuli_wild_265276	1399944090	*Ca_kabuli_scaffold*	265276	C/A	NA	INTERGENIC	NA
Kabuli_wild_347361	1399944091	*Ca_kabuli_scaffold*	347361	C/T	NA	INTERGENIC	NA
Kabuli_wild_347384	1399944092	*Ca_kabuli_scaffold*	347384	G/A	NA	INTERGENIC	NA
Kabuli_wild_347422	1399944093	*Ca_kabuli_scaffold*	347422	C/A	NA	INTERGENIC	NA
Kabuli_wild_404388	1399944094	*Ca_kabuli_scaffold*	404388	C/T	Gene_Ca_23464	SYNONYMOUS_CODING	NA
Kabuli_wild_404429	1399944095	*Ca_kabuli_scaffold*	404429	T/C	Gene_Ca_23464	NON_SYNONYMOUS_CODING	NA
Kabuli_wild_404444	1399944096	*Ca_kabuli_scaffold*	404444	C/T	Gene_Ca_23464	NON_SYNONYMOUS_CODING	NA
Kabuli_wild_404447	1399944097	*Ca_kabuli_scaffold*	404447	A/G	Gene_Ca_23464	NON_SYNONYMOUS_CODING	NA
Kabuli_wild_404464	1399944098	*Ca_kabuli_scaffold*	404464	G/A	Gene_Ca_23464	NON_SYNONYMOUS_CODING	NA
Kabuli_wild_479497	1399944099	*Ca_kabuli_scaffold*	479497	C/T	NA	INTERGENIC	NA
Kabuli_wild_561844	1399944100	*Ca_kabuli_scaffold*	561844	T/C	Gene_Ca_23466	INTRON	Helicase,C-terminal
Kabuli_wild_1035	1399944101	*Ca_kabuli_scaffold*	1035	C/A	Gene_Ca_25745	DOWNSTREAM	Sugar/inositol transporter
Kabuli_wild_18278	1399944102	*Ca_kabuli_scaffold*	18278	T/G	Gene_Ca_25747	SYNONYMOUS_CODING	Transposase,Ptta/En/Spm,plant
Kabuli_wild_22190	1399944103	*Ca_kabuli_scaffold*	22190	A/C	NA	INTERGENIC	NA
Kabuli_wild_22250	1399944104	*Ca_kabuli_scaffold*	22250	C/A	NA	INTERGENIC	NA
Kabuli_wild_72208	1399944105	*Ca_kabuli_scaffold*	72208	C/T	NA	INTERGENIC	NA
Kabuli_wild_72305	1399944106	*Ca_kabuli_scaffold*	72305	G/A	NA	INTERGENIC	NA
Kabuli_wild_72309	1399944107	*Ca_kabuli_scaffold*	72309	G/A	NA	INTERGENIC	NA
Kabuli_wild_72417	1399944108	*Ca_kabuli_scaffold*	72417	C/A	NA	INTERGENIC	NA
Kabuli_wild_72434	1399944109	*Ca_kabuli_scaffold*	72434	T/A	NA	INTERGENIC	NA
Kabuli_wild_72437	1399944110	*Ca_kabuli_scaffold*	72437	G/A	NA	INTERGENIC	NA
Kabuli_wild_72440	1399944111	*Ca_kabuli_scaffold*	72440	T/A	NA	INTERGENIC	NA
Kabuli_wild_72449	1399944112	*Ca_kabuli_scaffold*	72449	G/A	NA	INTERGENIC	NA
Kabuli_wild_72469	1399944113	*Ca_kabuli_scaffold*	72469	A/G	NA	INTERGENIC	NA
Kabuli_wild_72491	1399944114	*Ca_kabuli_scaffold*	72491	T/C	NA	INTERGENIC	NA
Kabuli_wild_72495	1399944115	*Ca_kabuli_scaffold*	72495	G/A	NA	INTERGENIC	NA
Kabuli_wild_80165	1399944116	*Ca_kabuli_scaffold*	80165	A/G	NA	INTERGENIC	NA
Kabuli_wild_69751	1399944117	*Ca_kabuli_scaffold*	69751	C/G	NA	INTERGENIC	NA
Kabuli_wild_69760	1399944118	*Ca_kabuli_scaffold*	69760	T/G	NA	INTERGENIC	NA
Kabuli_wild_69779	1399944119	*Ca_kabuli_scaffold*	69779	G/C	NA	INTERGENIC	NA
Kabuli_wild_69826	1399944120	*Ca_kabuli_scaffold*	69826	C/A	NA	INTERGENIC	NA
Kabuli_wild_69839	1399944121	*Ca_kabuli_scaffold*	69839	T/A	NA	INTERGENIC	NA
Kabuli_wild_90875	1399944122	*Ca_kabuli_scaffold*	90875	G/A	NA	INTERGENIC	NA
Kabuli_wild_90950	1399944123	*Ca_kabuli_scaffold*	90950	C/A	NA	INTERGENIC	NA

SNP IDs	NCBI dbSNP accession IDs (NCBI_SS#)	Chromosomes	Physical positions (bp)	SNPs	Gene accession IDs	Sequence components of genome	Putative functions
Kabuli_wild_98176	1399944124	*Ca_kabuli_scaffold*	98176	G/T	NA	INTERGENIC	NA
Kabuli_wild_99281	1399944125	*Ca_kabuli_scaffold*	99281	A/T	NA	INTERGENIC	NA
Kabuli_wild_99357	1399944126	*Ca_kabuli_scaffold*	99357	C/A	NA	INTERGENIC	NA
Kabuli_wild_111084	1399944127	*Ca_kabuli_scaffold*	111084	A/C	NA	INTERGENIC	NA
Kabuli_wild_111160	1399944128	*Ca_kabuli_scaffold*	111160	C/A	NA	INTERGENIC	NA
Kabuli_wild_112316	1399944129	*Ca_kabuli_scaffold*	112316	A/G	NA	INTERGENIC	NA
Kabuli_wild_117703	1399944130	*Ca_kabuli_scaffold*	117703	T/A	NA	INTERGENIC	NA
Kabuli_wild_117836	1399944131	*Ca_kabuli_scaffold*	117836	A/C	NA	INTERGENIC	NA
Kabuli_wild_104877	1399944132	*Ca_kabuli_scaffold*	104877	T/C	NA	INTERGENIC	NA
Kabuli_wild_104878	1399944133	*Ca_kabuli_scaffold*	104878	T/G	NA	INTERGENIC	NA
Kabuli_wild_104899	1399944134	*Ca_kabuli_scaffold*	104899	A/G	NA	INTERGENIC	NA
Kabuli_wild_77506	1399944135	*Ca_kabuli_scaffold*	77506	C/T	NA	INTERGENIC	NA
Kabuli_wild_522764	1399944136	*Ca_kabuli_scaffold*	522764	T/G	Gene_Ca_20600	SYNONYMOUS_CODING	Oxidoreductase,molybdopterin-binding domain
Kabuli_wild_613764	1399944137	*Ca_kabuli_scaffold*	613764	G/A	NA	INTERGENIC	NA
Kabuli_wild_613811	1399944138	*Ca_kabuli_scaffold*	613811	G/A	NA	INTERGENIC	NA
Kabuli_wild_613825	1399944139	*Ca_kabuli_scaffold*	613825	T/C	NA	INTERGENIC	NA
Kabuli_wild_613850	1399944140	*Ca_kabuli_scaffold*	613850	C/T	NA	INTERGENIC	NA
Kabuli_wild_613855	1399944141	*Ca_kabuli_scaffold*	613855	C/A	NA	INTERGENIC	NA
Kabuli_wild_613861	1399944142	*Ca_kabuli_scaffold*	613861	C/T	NA	INTERGENIC	NA
Kabuli_wild_640416	1399944143	*Ca_kabuli_scaffold*	640416	G/T	NA	INTERGENIC	NA
Kabuli_wild_654585	1399944144	*Ca_kabuli_scaffold*	654585	T/G	Gene_Ca_20605	SYNONYMOUS_CODING	GDP-fucoseproteinO-fucosyltransferase
Kabuli_wild_826644	1399944145	*Ca_kabuli_scaffold*	826644	G/A	NA	INTERGENIC	NA
Kabuli_wild_826690	1399944146	*Ca_kabuli_scaffold*	826690	T/C	NA	INTERGENIC	NA
Kabuli_wild_875473	1399944147	*Ca_kabuli_scaffold*	875473	G/A	NA	INTERGENIC	NA
Kabuli_wild_2879	1399944148	*Ca_kabuli_scaffold*	2879	G/A	Gene_Ca_25379	NON_SYNONYMOUS_CODING	NA
Kabuli_wild_3224	1399944149	*Ca_kabuli_scaffold*	3224	G/A	NA	INTERGENIC	NA
Kabuli_wild_7091	1399944150	*Ca_kabuli_scaffold*	7091	T/C	NA	INTERGENIC	NA
Kabuli_wild_19032	1399944151	*Ca_kabuli_scaffold*	19032	T/C	NA	INTERGENIC	NA
Kabuli_wild_163591	1399944152	*Ca_kabuli_scaffold*	163591	C/A	NA	INTERGENIC	NA
Kabuli_wild_221602	1399944153	*Ca_kabuli_scaffold*	221602	C/T	Gene_Ca_25387	INTRON	Domain of unknown function DUF699,exodeoxyribonucleaseValphachain
Kabuli_wild_48847	1399944154	*Ca_kabuli_scaffold*	48847	C/T	Gene_Ca_19823	NON_SYNONYMOUS_CODING	Peptidase C48, SUMO/Sentrin/Ubl1
Kabuli_wild_123585	1399944155	*Ca_kabuli_scaffold*	123585	C/G	NA	INTERGENIC	NA
Kabuli_wild_179940	1399944156	*Ca_kabuli_scaffold*	179940	G/A	Gene_Ca_19830	INTRON	Xanthine/uracil/vitamin C permease
Kabuli_wild_179969	1399944157	*Ca_kabuli_scaffold*	179969	A/T	Gene_Ca_19830	INTRON	Xanthine/uracil/vitamin C permease
Kabuli_wild_179993	1399944158	*Ca_kabuli_scaffold*	179993	C/T	Gene_Ca_19830	INTRON	Xanthine/uracil/vitamin C permease
Kabuli_wild_179998	1399944159	*Ca_kabuli_scaffold*	179998	T/C	Gene_Ca_19830	INTRON	Xanthine/uracil/vitamin C permease
Kabuli_wild_181468	1399944160	*Ca_kabuli_scaffold*	181468	C/T	Gene_Ca_19830	INTRON	Xanthine/uracil/vitamin C permease
Kabuli_wild_211634	1399944161	*Ca_kabuli_scaffold*	211634	A/C	Gene_Ca_19830	INTRON	Xanthine/uracil/vitamin C permease
Kabuli_wild_212514	1399944162	*Ca_kabuli_scaffold*	212514	C/T	NA	INTERGENIC	NA
Kabuli_wild_212536	1399944163	*Ca_kabuli_scaffold*	212536	A/C	NA	INTERGENIC	NA
Kabuli_wild_234794	1399944164	*Ca_kabuli_scaffold*	234794	T/G	NA	INTERGENIC	NA
Kabuli_wild_255161	1399944165	*Ca_kabuli_scaffold*	255161	G/A	Gene_Ca_19833	INTRON	Villinheadpiece
Kabuli_wild_515736	1399944166	*Ca_kabuli_scaffold*	515736	C/T	NA	INTERGENIC	NA
Kabuli_wild_619172	1399944167	*Ca_kabuli_scaffold*	619172	G/A	NA	INTERGENIC	NA

SNP IDs	NCBI dbSNP accession IDs (NCBI_SS#)	Chromosomes	Physical positions (bp)	SNPs	Gene accession IDs	Sequence components of genome	Putative functions
Kabuli_wild_619186	1399944168	*Ca_kabuli_scaffold*	619186	C/T	NA	INTERGENIC	NA
Kabuli_wild_626052	1399944169	*Ca_kabuli_scaffold*	626052	T/C	Gene_Ca_19843	INTRON	Pyridinenucleotide-disulphideoxidoreductase,class-II
Kabuli_wild_631375	1399944170	*Ca_kabuli_scaffold*	631375	G/A	Gene_Ca_19843	INTRON	Pyridinenucleotide-disulphideoxidoreductase,class-II
Kabuli_wild_728010	1399944171	*Ca_kabuli_scaffold*	728010	T/A	NA	INTERGENIC	NA
Kabuli_wild_800511	1399944172	*Ca_kabuli_scaffold*	800511	C/T	NA	INTERGENIC	NA
Kabuli_wild_800522	1399944173	*Ca_kabuli_scaffold*	800522	C/T	NA	INTERGENIC	NA
Kabuli_wild_824906	1399944174	*Ca_kabuli_scaffold*	824906	C/T	NA	INTERGENIC	NA
Kabuli_wild_824993	1399944175	*Ca_kabuli_scaffold*	824993	T/A	NA	INTERGENIC	NA
Kabuli_wild_853159	1399944176	*Ca_kabuli_scaffold*	853159	C/T	NA	INTERGENIC	NA
Kabuli_wild_853161	1399944177	*Ca_kabuli_scaffold*	853161	T/A	NA	INTERGENIC	NA
Kabuli_wild_853187	1399944178	*Ca_kabuli_scaffold*	853187	C/T	NA	INTERGENIC	NA
Kabuli_wild_853204	1399944179	*Ca_kabuli_scaffold*	853204	G/A	NA	INTERGENIC	NA
Kabuli_wild_853215	1399944180	*Ca_kabuli_scaffold*	853215	T/A	NA	INTERGENIC	NA
Kabuli_wild_853217	1399944181	*Ca_kabuli_scaffold*	853217	C/T	NA	INTERGENIC	NA
Kabuli_wild_884733	1399944182	*Ca_kabuli_scaffold*	884733	C/T	NA	INTERGENIC	NA
Kabuli_wild_41422	1399944183	*Ca_kabuli_scaffold*	41422	T/G	Gene_Ca_20526	INTRON	Glycosylhydrolase,family13,catalytic domain
Kabuli_wild_59883	1399944184	*Ca_kabuli_scaffold*	59883	A/C	Gene_Ca_20530	SYNONYMOUS_CODING	Alanineracemase,N-terminal
Kabuli_wild_150359	1399944185	*Ca_kabuli_scaffold*	150359	T/C	NA	INTERGENIC	NA
Kabuli_wild_150395	1399944186	*Ca_kabuli_scaffold*	150395	G/C	NA	INTERGENIC	NA
Kabuli_wild_128675	1399944187	*Ca_kabuli_scaffold*	128675	A/T	Gene_Ca_25993	DOWNSTREAM	NA
Kabuli_wild_199050	1399944188	*Ca_kabuli_scaffold*	199050	A/T	Gene_Ca_25997	UPSTREAM	NA
Kabuli_wild_199067	1399944189	*Ca_kabuli_scaffold*	199067	A/T	Gene_Ca_25997	UPSTREAM	NA
Kabuli_wild_199068	1399944190	*Ca_kabuli_scaffold*	199068	G/T	Gene_Ca_25997	UPSTREAM	NA
Kabuli_wild_199073	1399944191	*Ca_kabuli_scaffold*	199073	G/A	Gene_Ca_25997	UPSTREAM	NA
Kabuli_wild_204540	1399944192	*Ca_kabuli_scaffold*	204540	G/A	NA	INTERGENIC	NA
Kabuli_wild_111887	1399944193	*Ca_kabuli_scaffold*	111887	A/C	Gene_Ca_27306	NON_SYNONYMOUS_CODING	NA
Kabuli_wild_111889	1399944194	*Ca_kabuli_scaffold*	111889	A/G	Gene_Ca_27306	SYNONYMOUS_CODING	NA
Kabuli_wild_111914	1399944195	*Ca_kabuli_scaffold*	111914	C/T	Gene_Ca_27306	NON_SYNONYMOUS_CODING	NA
Kabuli_wild_111924	1399944196	*Ca_kabuli_scaffold*	111924	C/T	Gene_Ca_27306	NON_SYNONYMOUS_CODING	NA
Kabuli_wild_111926	1399944197	*Ca_kabuli_scaffold*	111926	G/A	Gene_Ca_27306	NON_SYNONYMOUS_CODING	NA
Kabuli_wild_111930	1399944198	*Ca_kabuli_scaffold*	111930	T/C	Gene_Ca_27306	NON_SYNONYMOUS_CODING	NA
Kabuli_wild_111933	1399944199	*Ca_kabuli_scaffold*	111933	C/T	Gene_Ca_27306	NON_SYNONYMOUS_CODING	NA
Kabuli_wild_111936	1399944200	*Ca_kabuli_scaffold*	111936	C/A	Gene_Ca_27306	NON_SYNONYMOUS_CODING	NA
Kabuli_wild_111959	1399944201	*Ca_kabuli_scaffold*	111959	C/T	Gene_Ca_27306	NON_SYNONYMOUS_CODING	NA
Kabuli_wild_111964	1399944202	*Ca_kabuli_scaffold*	111964	C/T	Gene_Ca_27306	SYNONYMOUS_CODING	NA
Kabuli_wild_111972	1399944203	*Ca_kabuli_scaffold*	111972	A/T	Gene_Ca_27306	NON_SYNONYMOUS_CODING	NA
Kabuli_wild_111973	1399944204	*Ca_kabuli_scaffold*	111973	A/T	Gene_Ca_27306	NON_SYNONYMOUS_CODING	NA
Kabuli_wild_111976	1399944205	*Ca_kabuli_scaffold*	111976	C/T	Gene_Ca_27306	SYNONYMOUS_CODING	NA
Kabuli_wild_112005	1399944206	*Ca_kabuli_scaffold*	112005	G/A	Gene_Ca_27306	NON_SYNONYMOUS_CODING	NA
Kabuli_wild_112006	1399944207	*Ca_kabuli_scaffold*	112006	G/A	Gene_Ca_27306	SYNONYMOUS_CODING	NA
Kabuli_wild_112011	1399944208	*Ca_kabuli_scaffold*	112011	T/C	Gene_Ca_27306	NON_SYNONYMOUS_CODING	NA
Kabuli_wild_18213	1399944209	*Ca_kabuli_scaffold*	18213	G/A	NA	INTERGENIC	NA
Kabuli_wild_164796	1399944210	*Ca_kabuli_scaffold*	164796	G/T	Gene_Ca_26661	INTRON	NA
Kabuli_wild_164797	1399944211	*Ca_kabuli_scaffold*	164797	C/T	Gene_Ca_26661	INTRON	NA

SNP IDs	NCBI dbSNP accession IDs (NCBI_SS#)	Chromosomes	Physical positions (bp)	SNPs	Gene accession IDs	Sequence components of genome	Putative functions
Kabuli_wild_49263	1399944212	*Ca_kabuli_scaffold*	49263	G/A	NA	INTERGENIC	NA
Kabuli_wild_49266	1399944213	*Ca_kabuli_scaffold*	49266	T/G	NA	INTERGENIC	NA
Kabuli_wild_138929	1399944214	*Ca_kabuli_scaffold*	138929	G/A	NA	INTERGENIC	NA
Kabuli_wild_194932	1399944215	*Ca_kabuli_scaffold*	194932	C/T	NA	INTERGENIC	NA
Kabuli_wild_195000	1399944216	*Ca_kabuli_scaffold*	195000	C/T	NA	INTERGENIC	NA
Kabuli_wild_19215	1399944217	*Ca_kabuli_scaffold*	19215	C/A	NA	INTERGENIC	NA
Kabuli_wild_85993	1399944218	*Ca_kabuli_scaffold*	85993	T/A	NA	INTERGENIC	NA
Kabuli_wild_88389	1399944219	*Ca_kabuli_scaffold*	88389	T/A	NA	INTERGENIC	NA
Kabuli_wild_88419	1399944220	*Ca_kabuli_scaffold*	88419	A/T	NA	INTERGENIC	NA
Kabuli_wild_410571	1399944221	*Ca_kabuli_scaffold*	410571	C/A	NA	INTERGENIC	NA
Kabuli_wild_86592	1399944222	*Ca_kabuli_scaffold*	86592	G/A	Gene_Ca_26331	DOWNSTREAM	Peptidase C48, SUMO/Sentrin/Ubl1
Kabuli_wild_86595	1399944223	*Ca_kabuli_scaffold*	86595	G/A	Gene_Ca_26331	DOWNSTREAM	Peptidase C48, SUMO/Sentrin/Ubl1
Kabuli_wild_86610	1399944224	*Ca_kabuli_scaffold*	86610	C/T	Gene_Ca_26331	DOWNSTREAM	Peptidase C48, SUMO/Sentrin/Ubl1
Kabuli_wild_86674	1399944225	*Ca_kabuli_scaffold*	86674	C/T	Gene_Ca_26331	DOWNSTREAM	Peptidase C48, SUMO/Sentrin/Ubl1
Kabuli_wild_86676	1399944226	*Ca_kabuli_scaffold*	86676	G/A	Gene_Ca_26331	DOWNSTREAM	Peptidase C48, SUMO/Sentrin/Ubl1
Kabuli_wild_86677	1399944227	*Ca_kabuli_scaffold*	86677	C/T	Gene_Ca_26331	DOWNSTREAM	Peptidase C48, SUMO/Sentrin/Ubl1
Kabuli_wild_88124	1399944228	*Ca_kabuli_scaffold*	88124	G/A	Gene_Ca_26330	DOWNSTREAM	NA
Kabuli_wild_40521	1399944229	*Ca_kabuli_scaffold*	40521	A/G	NA	INTERGENIC	NA
Kabuli_wild_432453	1399944230	*Ca_kabuli_scaffold*	432453	T/C	NA	INTERGENIC	NA
Kabuli_wild_432471	1399944231	*Ca_kabuli_scaffold*	432471	A/G	NA	INTERGENIC	NA
Kabuli_wild_535266	1399944232	*Ca_kabuli_scaffold*	535266	A/C	Gene_Ca_21812	NON_SYNONYMOUS_CODING	Dual specificity phosphatase, catalytic domain
Kabuli_wild_535269	1399944233	*Ca_kabuli_scaffold*	535269	A/C	Gene_Ca_21812	NON_SYNONYMOUS_CODING	Dual specificity phosphatase, catalytic domain
Kabuli_wild_186066	1399944234	*Ca_kabuli_scaffold*	186066	G/T	NA	INTERGENIC	NA
Kabuli_wild_186103	1399944235	*Ca_kabuli_scaffold*	186103	A/G	NA	INTERGENIC	NA
Kabuli_wild_243142	1399944236	*Ca_kabuli_scaffold*	243142	A/C	NA	INTERGENIC	NA
Kabuli_wild_243466	1399944237	*Ca_kabuli_scaffold*	243466	C/G	NA	INTERGENIC	NA
Kabuli_wild_55475	1399944238	*Ca_kabuli_scaffold*	55475	G/A	NA	INTERGENIC	NA
Kabuli_wild_55479	1399944239	*Ca_kabuli_scaffold*	55479	G/T	NA	INTERGENIC	NA
Kabuli_wild_55589	1399944240	*Ca_kabuli_scaffold*	55589	A/T	NA	INTERGENIC	NA
Kabuli_wild_15569	1399944241	*Ca_kabuli_scaffold*	15569	A/C	Gene_Ca_24910	NON_SYNONYMOUS_CODING	NA
Kabuli_wild_15574	1399944242	*Ca_kabuli_scaffold*	15574	G/A	Gene_Ca_24910	SYNONYMOUS_CODING	NA
Kabuli_wild_15581	1399944243	*Ca_kabuli_scaffold*	15581	G/C	Gene_Ca_24910	NON_SYNONYMOUS_CODING	NA
Kabuli_wild_15588	1399944244	*Ca_kabuli_scaffold*	15588	C/T	Gene_Ca_24910	NON_SYNONYMOUS_CODING	NA
Kabuli_wild_15590	1399944245	*Ca_kabuli_scaffold*	15590	G/T	Gene_Ca_24910	NON_SYNONYMOUS_CODING	NA
Kabuli_wild_15593	1399944246	*Ca_kabuli_scaffold*	15593	G/A	Gene_Ca_24910	NON_SYNONYMOUS_CODING	NA
Kabuli_wild_15594	1399944247	*Ca_kabuli_scaffold*	15594	C/A	Gene_Ca_24910	NON_SYNONYMOUS_CODING	NA
Kabuli_wild_15614	1399944248	*Ca_kabuli_scaffold*	15614	C/T	Gene_Ca_24910	STOP_GAINED	NA
Kabuli_wild_15636	1399944249	*Ca_kabuli_scaffold*	15636	G/A	Gene_Ca_24910	NON_SYNONYMOUS_CODING	NA
Kabuli_wild_46534	1399944250	*Ca_kabuli_scaffold*	46534	T/C	Gene_Ca_24912	DOWNSTREAM	NA
Kabuli_wild_375902	1399944251	*Ca_kabuli_scaffold*	375902	T/A	Gene_Ca_24920	NON_SYNONYMOUS_CODING	NA
Kabuli_wild_375907	1399944252	*Ca_kabuli_scaffold*	375907	T/G	Gene_Ca_24920	SYNONYMOUS_CODING	NA
Kabuli_wild_375918	1399944253	*Ca_kabuli_scaffold*	375918	C/A	Gene_Ca_24920	SYNONYMOUS_CODING	NA
Kabuli_wild_375938	1399944254	*Ca_kabuli_scaffold*	375938	C/T	Gene_Ca_24920	NON_SYNONYMOUS_CODING	NA
Kabuli_wild_375941	1399944255	*Ca_kabuli_scaffold*	375941	C/T	Gene_Ca_24920	NON_SYNONYMOUS_CODING	NA

SNP IDs	NCBI dbSNP accession IDs (NCBI_SS#)	Chromosomes	Physical positions (bp)	SNPs	Gene accession IDs	Sequence components of genome	Putative functions
Kabuli_wild_375958	1399944256	*Ca_kabuli_scaffold*	375958	C/T	Gene_Ca_24920	STOP_GAINED	NA
Kabuli_wild_132970	1399944257	*Ca_kabuli_scaffold*	132970	A/G	NA	INTERGENIC	NA
Kabuli_wild_132976	1399944258	*Ca_kabuli_scaffold*	132976	T/C	NA	INTERGENIC	NA
Kabuli_wild_132977	1399944259	*Ca_kabuli_scaffold*	132977	T/C	NA	INTERGENIC	NA
Kabuli_wild_133000	1399944260	*Ca_kabuli_scaffold*	133000	A/G	NA	INTERGENIC	NA
Kabuli_wild_133028	1399944261	*Ca_kabuli_scaffold*	133028	C/T	NA	INTERGENIC	NA
Kabuli_wild_133030	1399944262	*Ca_kabuli_scaffold*	133030	T/A	NA	INTERGENIC	NA
Kabuli_wild_133035	1399944263	*Ca_kabuli_scaffold*	133035	G/A	NA	INTERGENIC	NA
Kabuli_wild_137400	1399944264	*Ca_kabuli_scaffold*	137400	A/G	NA	INTERGENIC	NA
Kabuli_wild_137406	1399944265	*Ca_kabuli_scaffold*	137406	T/C	NA	INTERGENIC	NA
Kabuli_wild_137407	1399944266	*Ca_kabuli_scaffold*	137407	T/C	NA	INTERGENIC	NA
Kabuli_wild_137430	1399944267	*Ca_kabuli_scaffold*	137430	A/G	NA	INTERGENIC	NA
Kabuli_wild_137458	1399944268	*Ca_kabuli_scaffold*	137458	C/T	NA	INTERGENIC	NA
Kabuli_wild_137460	1399944269	*Ca_kabuli_scaffold*	137460	T/A	NA	INTERGENIC	NA
Kabuli_wild_137465	1399944270	*Ca_kabuli_scaffold*	137465	G/A	NA	INTERGENIC	NA
Kabuli_wild_203691	1399944271	*Ca_kabuli_scaffold*	203691	T/A	Gene_Ca_25760	DOWNSTREAM	NA
Kabuli_wild_203755	1399944272	*Ca_kabuli_scaffold*	203755	A/G	Gene_Ca_25760	DOWNSTREAM	NA
Kabuli_wild_203756	1399944273	*Ca_kabuli_scaffold*	203756	C/T	Gene_Ca_25760	DOWNSTREAM	NA
Kabuli_wild_204193	1399944274	*Ca_kabuli_scaffold*	204193	G/A	Gene_Ca_25760	DOWNSTREAM	NA
Kabuli_wild_204783	1399944275	*Ca_kabuli_scaffold*	204783	A/G	Gene_Ca_25760	DOWNSTREAM	NA
Kabuli_wild_204785	1399944276	*Ca_kabuli_scaffold*	204785	G/A	Gene_Ca_25760	DOWNSTREAM	NA
Kabuli_wild_204952	1399944277	*Ca_kabuli_scaffold*	204952	G/A	Gene_Ca_25760	DOWNSTREAM	NA
Kabuli_wild_43160	1399944278	*Ca_kabuli_scaffold*	43160	G/A	Gene_Ca_25777	NON_SYNONYMOUS_CODING	NA
Kabuli_wild_43241	1399944279	*Ca_kabuli_scaffold*	43241	G/C	Gene_Ca_25777	NON_SYNONYMOUS_CODING	NA
Kabuli_wild_174761	1399944280	*Ca_kabuli_scaffold*	174761	G/C	NA	INTERGENIC	NA
Kabuli_wild_60906	1399944281	*Ca_kabuli_scaffold*	60906	T/G	NA	INTERGENIC	NA
Kabuli_wild_60950	1399944282	*Ca_kabuli_scaffold*	60950	T/C	NA	INTERGENIC	NA
Kabuli_wild_60951	1399944283	*Ca_kabuli_scaffold*	60951	T/C	NA	INTERGENIC	NA
Kabuli_wild_60952	1399944284	*Ca_kabuli_scaffold*	60952	T/C	NA	INTERGENIC	NA
Kabuli_wild_60956	1399944285	*Ca_kabuli_scaffold*	60956	T/C	NA	INTERGENIC	NA
Kabuli_wild_60957	1399944286	*Ca_kabuli_scaffold*	60957	T/C	NA	INTERGENIC	NA
Kabuli_wild_60959	1399944287	*Ca_kabuli_scaffold*	60959	T/C	NA	INTERGENIC	NA
Kabuli_wild_60960	1399944288	*Ca_kabuli_scaffold*	60960	T/C	NA	INTERGENIC	NA
Kabuli_wild_60962	1399944289	*Ca_kabuli_scaffold*	60962	T/G	NA	INTERGENIC	NA
Kabuli_wild_60963	1399944290	*Ca_kabuli_scaffold*	60963	T/G	NA	INTERGENIC	NA
Kabuli_wild_135964	1399944291	*Ca_kabuli_scaffold*	135964	G/A	Gene_Ca_26029	SYNONYMOUS_CODING	Peptidase C48, SUMO/Sentrin/Ubl1
Kabuli_wild_135969	1399944292	*Ca_kabuli_scaffold*	135969	C/T	Gene_Ca_26029	NON_SYNONYMOUS_CODING	Peptidase C48, SUMO/Sentrin/Ubl1
Kabuli_wild_135976	1399944293	*Ca_kabuli_scaffold*	135976	C/T	Gene_Ca_26029	SYNONYMOUS_CODING	Peptidase C48, SUMO/Sentrin/Ubl1
Kabuli_wild_135999	1399944294	*Ca_kabuli_scaffold*	135999	G/A	Gene_Ca_26029	NON_SYNONYMOUS_CODING	Peptidase C48, SUMO/Sentrin/Ubl1
Kabuli_wild_136004	1399944295	*Ca_kabuli_scaffold*	136004	C/T	Gene_Ca_26029	NON_SYNONYMOUS_CODING	Peptidase C48, SUMO/Sentrin/Ubl1
Kabuli_wild_136017	1399944296	*Ca_kabuli_scaffold*	136017	C/G	Gene_Ca_26029	NON_SYNONYMOUS_CODING	Peptidase C48, SUMO/Sentrin/Ubl1
Kabuli_wild_136030	1399944297	*Ca_kabuli_scaffold*	136030	G/A	Gene_Ca_26029	SYNONYMOUS_CODING	Peptidase C48, SUMO/Sentrin/Ubl1
Kabuli_wild_136034	1399944298	*Ca_kabuli_scaffold*	136034	G/A	Gene_Ca_26029	NON_SYNONYMOUS_CODING	Peptidase C48, SUMO/Sentrin/Ubl1
Kabuli_wild_136050	1399944299	*Ca_kabuli_scaffold*	136050	G/A	Gene_Ca_26029	SYNONYMOUS_CODING	Peptidase C48, SUMO/Sentrin/Ubl1

SNP IDs	NCBI dbSNP accession IDs (NCBI_SS#)	Chromosomes	Physical positions (bp)	SNPs	Gene accession IDs	Sequence components of genome	Putative functions
Kabuli_wild_136051	1399944300	*Ca_kabuli_scaffold*	136051	T/A	Gene_Ca_26029	NON_SYNONYMOUS_CODING	Peptidase C48, SUMO/Sentrin/Ubl1
Kabuli_wild_136073	1399944301	*Ca_kabuli_scaffold*	136073	G/A	Gene_Ca_26029	NON_SYNONYMOUS_CODING	Peptidase C48, SUMO/Sentrin/Ubl1
Kabuli_wild_136076	1399944302	*Ca_kabuli_scaffold*	136076	G/C	Gene_Ca_26029	NON_SYNONYMOUS_CODING	Peptidase C48, SUMO/Sentrin/Ubl1
Kabuli_wild_136077	1399944303	*Ca_kabuli_scaffold*	136077	C/G	Gene_Ca_26029	NON_SYNONYMOUS_CODING	Peptidase C48, SUMO/Sentrin/Ubl1
Kabuli_wild_136090	1399944304	*Ca_kabuli_scaffold*	136090	C/G	Gene_Ca_26029	SYNONYMOUS_CODING	Peptidase C48, SUMO/Sentrin/Ubl1
Kabuli_wild_136091	1399944305	*Ca_kabuli_scaffold*	136091	G/A	Gene_Ca_26029	NON_SYNONYMOUS_CODING	Peptidase C48, SUMO/Sentrin/Ubl1
Kabuli_wild_136092	1399944306	*Ca_kabuli_scaffold*	136092	A/G	Gene_Ca_26029	NON_SYNONYMOUS_CODING	Peptidase C48, SUMO/Sentrin/Ubl1
Kabuli_wild_136096	1399944307	*Ca_kabuli_scaffold*	136096	T/C	Gene_Ca_26029	SYNONYMOUS_CODING	Peptidase C48, SUMO/Sentrin/Ubl1
Kabuli_wild_136106	1399944308	*Ca_kabuli_scaffold*	136106	G/A	Gene_Ca_26029	NON_SYNONYMOUS_CODING	Peptidase C48, SUMO/Sentrin/Ubl1
Kabuli_wild_136109	1399944309	*Ca_kabuli_scaffold*	136109	G/T	Gene_Ca_26029	NON_SYNONYMOUS_CODING	Peptidase C48, SUMO/Sentrin/Ubl1
Kabuli_wild_136129	1399944310	*Ca_kabuli_scaffold*	136129	T/C	Gene_Ca_26029	SYNONYMOUS_CODING	Peptidase C48, SUMO/Sentrin/Ubl1
Kabuli_wild_468863	1399944311	*Ca_kabuli_scaffold*	468863	T/A	Gene_Ca_21282	NON_SYNONYMOUS_CODING	Protein kinase,catalytic domain
Kabuli_wild_563293	1399944312	*Ca_kabuli_scaffold*	563293	A/G	Gene_Ca_21286	DOWNSTREAM	NA
Kabuli_wild_563319	1399944313	*Ca_kabuli_scaffold*	563319	C/G	Gene_Ca_21286	DOWNSTREAM	NA
Kabuli_wild_563363	1399944314	*Ca_kabuli_scaffold*	563363	C/T	Gene_Ca_21286	DOWNSTREAM	NA
Kabuli_wild_618427	1399944315	*Ca_kabuli_scaffold*	618427	T/C	Gene_Ca_21291	SYNONYMOUS_CODING	ABC transporter,transmembrane domain
Kabuli_wild_637556	1399944316	*Ca_kabuli_scaffold*	637556	T/C	NA	INTERGENIC	NA
Kabuli_wild_771130	1399944317	*Ca_kabuli_scaffold*	771130	C/T	Gene_Ca_21296	NON_SYNONYMOUS_CODING	Peptidase C48, SUMO/Sentrin/Ubl1
Kabuli_wild_771163	1399944318	*Ca_kabuli_scaffold*	771163	G/A	Gene_Ca_21296	NON_SYNONYMOUS_CODING	Peptidase C48, SUMO/Sentrin/Ubl1
Kabuli_wild_771169	1399944319	*Ca_kabuli_scaffold*	771169	C/T	Gene_Ca_21296	NON_SYNONYMOUS_CODING	Peptidase C48, SUMO/Sentrin/Ubl1
Kabuli_wild_771170	1399944320	*Ca_kabuli_scaffold*	771170	G/A	Gene_Ca_21296	SYNONYMOUS_CODING	Peptidase C48, SUMO/Sentrin/Ubl1
Kabuli_wild_800641	1399944321	*Ca_kabuli_scaffold*	800641	C/T	NA	INTERGENIC	NA
Kabuli_wild_800650	1399944322	*Ca_kabuli_scaffold*	800650	C/T	NA	INTERGENIC	NA
Kabuli_wild_800669	1399944323	*Ca_kabuli_scaffold*	800669	A/G	NA	INTERGENIC	NA
Kabuli_wild_37627	1399944324	*Ca_kabuli_scaffold*	37627	C/G	NA	INTERGENIC	NA
Kabuli_wild_172869	1399944325	*Ca_kabuli_scaffold*	172869	T/G	NA	INTERGENIC	NA
Kabuli_wild_182445	1399944326	*Ca_kabuli_scaffold*	182445	T/C	NA	INTERGENIC	NA
Kabuli_wild_310000	1399944327	*Ca_kabuli_scaffold*	310000	A/T	NA	INTERGENIC	NA
Kabuli_wild_310014	1399944328	*Ca_kabuli_scaffold*	310014	G/A	NA	INTERGENIC	NA
Kabuli_wild_98240	1399944329	*Ca_kabuli_scaffold*	98240	A/G	Gene_Ca_27458	NON_SYNONYMOUS_CODING	Glycosidehydrolase,family17
Kabuli_wild_41774	1399944330	*Ca_kabuli_scaffold*	41774	G/A	Gene_Ca_24719	INTRON	Ionotropicglutamate receptor
Kabuli_wild_311350	1399944331	*Ca_kabuli_scaffold*	311350	C/T	NA	INTERGENIC	NA
Kabuli_wild_311394	1399944332	*Ca_kabuli_scaffold*	311394	A/C	NA	INTERGENIC	NA
Kabuli_wild_311411	1399944333	*Ca_kabuli_scaffold*	311411	G/A	NA	INTERGENIC	NA
Kabuli_wild_359376	1399944334	*Ca_kabuli_scaffold*	359376	A/C	Gene_Ca_24729	INTRON	NA
Kabuli_wild_51381	1399944335	*Ca_kabuli_scaffold*	51381	A/T	NA	INTERGENIC	NA
Kabuli_wild_51396	1399944336	*Ca_kabuli_scaffold*	51396	G/A	NA	INTERGENIC	NA
Kabuli_wild_51414	1399944337	*Ca_kabuli_scaffold*	51414	C/T	NA	INTERGENIC	NA
Kabuli_wild_51435	1399944338	*Ca_kabuli_scaffold*	51435	C/T	NA	INTERGENIC	NA
Kabuli_wild_51446	1399944339	*Ca_kabuli_scaffold*	51446	G/A	NA	INTERGENIC	NA
Kabuli_wild_51494	1399944340	*Ca_kabuli_scaffold*	51494	C/G	NA	INTERGENIC	NA
Kabuli_wild_51498	1399944341	*Ca_kabuli_scaffold*	51498	C/T	NA	INTERGENIC	NA
Kabuli_wild_51518	1399944342	*Ca_kabuli_scaffold*	51518	T/C	NA	INTERGENIC	NA
Kabuli_wild_51548	1399944343	*Ca_kabuli_scaffold*	51548	T/C	NA	INTERGENIC	NA

SNP IDs	NCBI dbSNP accession IDs (NCBI_SS#)	Chromosomes	Physical positions (bp)	SNPs	Gene accession IDs	Sequence components of genome	Putative functions
Kabuli_wild_51605	1399944344	*Ca_kabuli_scaffold*	51605	C/T	NA	INTERGENIC	NA
Kabuli_wild_51617	1399944345	*Ca_kabuli_scaffold*	51617	G/A	NA	INTERGENIC	NA
Kabuli_wild_51624	1399944346	*Ca_kabuli_scaffold*	51624	G/A	NA	INTERGENIC	NA
Kabuli_wild_51637	1399944347	*Ca_kabuli_scaffold*	51637	T/A	NA	INTERGENIC	NA
Kabuli_wild_174039	1399944348	*Ca_kabuli_scaffold*	174039	A/T	NA	INTERGENIC	NA
Kabuli_wild_153995	1399944349	*Ca_kabuli_scaffold*	153995	C/G	NA	INTERGENIC	NA
Kabuli_wild_154023	1399944350	*Ca_kabuli_scaffold*	154023	C/T	NA	INTERGENIC	NA
Kabuli_wild_41848	1399944351	*Ca_kabuli_scaffold*	41848	C/T	Gene_Ca_27825	NON_SYNONYMOUS_CODING	NA
Kabuli_wild_41878	1399944352	*Ca_kabuli_scaffold*	41878	C/T	Gene_Ca_27825	NON_SYNONYMOUS_CODING	NA
Kabuli_wild_22672	1399944353	*Ca_kabuli_scaffold*	22672	C/T	NA	INTERGENIC	NA
Kabuli_wild_22675	1399944354	*Ca_kabuli_scaffold*	22675	G/A	NA	INTERGENIC	NA
Kabuli_wild_22680	1399944355	*Ca_kabuli_scaffold*	22680	C/A	NA	INTERGENIC	NA
Kabuli_wild_22693	1399944356	*Ca_kabuli_scaffold*	22693	C/T	NA	INTERGENIC	NA
Kabuli_wild_22699	1399944357	*Ca_kabuli_scaffold*	22699	A/G	NA	INTERGENIC	NA
Kabuli_wild_22708	1399944358	*Ca_kabuli_scaffold*	22708	A/G	NA	INTERGENIC	NA
Kabuli_wild_22720	1399944359	*Ca_kabuli_scaffold*	22720	C/T	NA	INTERGENIC	NA
Kabuli_wild_22726	1399944360	*Ca_kabuli_scaffold*	22726	A/C	NA	INTERGENIC	NA
Kabuli_wild_22732	1399944361	*Ca_kabuli_scaffold*	22732	G/A	NA	INTERGENIC	NA
Kabuli_wild_22777	1399944362	*Ca_kabuli_scaffold*	22777	T/G	NA	INTERGENIC	NA
Kabuli_wild_188959	1399944363	*Ca_kabuli_scaffold*	188959	G/T	NA	INTERGENIC	NA
Kabuli_wild_88627	1399944364	*Ca_kabuli_scaffold*	88627	A/G	Gene_Ca_27561	SYNONYMOUS_CODING	Peptidase C48, SUMO/Sentrin/Ubl1
Kabuli_wild_88629	1399944365	*Ca_kabuli_scaffold*	88629	C/T	Gene_Ca_27561	SYNONYMOUS_CODING	Peptidase C48, SUMO/Sentrin/Ubl1
Kabuli_wild_88669	1399944366	*Ca_kabuli_scaffold*	88669	T/A	Gene_Ca_27561	NON_SYNONYMOUS_CODING	Peptidase C48, SUMO/Sentrin/Ubl1
Kabuli_wild_88670	1399944367	*Ca_kabuli_scaffold*	88670	C/T	Gene_Ca_27561	SYNONYMOUS_CODING	Peptidase C48, SUMO/Sentrin/Ubl1
Kabuli_wild_88694	1399944368	*Ca_kabuli_scaffold*	88694	G/A	Gene_Ca_27561	SYNONYMOUS_CODING	Peptidase C48, SUMO/Sentrin/Ubl1
Kabuli_wild_88721	1399944369	*Ca_kabuli_scaffold*	88721	C/T	Gene_Ca_27561	NON_SYNONYMOUS_CODING	Peptidase C48, SUMO/Sentrin/Ubl1
Kabuli_wild_88726	1399944370	*Ca_kabuli_scaffold*	88726	A/C	Gene_Ca_27561	NON_SYNONYMOUS_CODING	Peptidase C48, SUMO/Sentrin/Ubl1
Kabuli_wild_84915	1399944371	*Ca_kabuli_scaffold*	84915	T/A	NA	INTERGENIC	NA
Kabuli_wild_84943	1399944372	*Ca_kabuli_scaffold*	84943	A/G	NA	INTERGENIC	NA
Kabuli_wild_84945	1399944373	*Ca_kabuli_scaffold*	84945	T/C	NA	INTERGENIC	NA
Kabuli_wild_84953	1399944374	*Ca_kabuli_scaffold*	84953	T/G	NA	INTERGENIC	NA
Kabuli_wild_84969	1399944375	*Ca_kabuli_scaffold*	84969	G/A	NA	INTERGENIC	NA
Kabuli_wild_84972	1399944376	*Ca_kabuli_scaffold*	84972	C/A	NA	INTERGENIC	NA
Kabuli_wild_84974	1399944377	*Ca_kabuli_scaffold*	84974	T/G	NA	INTERGENIC	NA
Kabuli_wild_84988	1399944378	*Ca_kabuli_scaffold*	84988	T/G	NA	INTERGENIC	NA
Kabuli_wild_84994	1399944379	*Ca_kabuli_scaffold*	84994	G/A	NA	INTERGENIC	NA
Kabuli_wild_164620	1399944380	*Ca_kabuli_scaffold*	164620	C/T	NA	INTERGENIC	NA
Kabuli_wild_164787	1399944381	*Ca_kabuli_scaffold*	164787	C/T	NA	INTERGENIC	NA
Kabuli_wild_164789	1399944382	*Ca_kabuli_scaffold*	164789	T/C	NA	INTERGENIC	NA
Kabuli_wild_164983	1399944383	*Ca_kabuli_scaffold*	164983	G/A	NA	INTERGENIC	NA
Kabuli_wild_165014	1399944384	*Ca_kabuli_scaffold*	165014	G/A	NA	INTERGENIC	NA
Kabuli_wild_165285	1399944385	*Ca_kabuli_scaffold*	165285	A/G	Gene_Ca_24992	UPSTREAM	NA
Kabuli_wild_165303	1399944386	*Ca_kabuli_scaffold*	165303	G/A	Gene_Ca_24992	UPSTREAM	NA
Kabuli_wild_165312	1399944387	*Ca_kabuli_scaffold*	165312	A/T	Gene_Ca_24992	UPSTREAM	NA

SNP IDs	NCBI dbSNP accession IDs (NCBI_SS#)	Chromosomes	Physical positions (bp)	SNPs	Gene accession IDs	Sequence components of genome	Putative functions
Kabuli_wild_165582	1399944388	*Ca_kabuli_scaffold*	165582	G/A	Gene_Ca_24992	UPSTREAM	NA
Kabuli_wild_165583	1399944389	*Ca_kabuli_scaffold*	165583	T/C	Gene_Ca_24992	UPSTREAM	NA
Kabuli_wild_196556	1399944390	*Ca_kabuli_scaffold*	196556	G/A	Gene_Ca_24993	NON_SYNONYMOUS_CODING	NA
Kabuli_wild_219176	1399944391	*Ca_kabuli_scaffold*	219176	T/C	NA	INTERGENIC	NA
Kabuli_wild_83724	1399944392	*Ca_kabuli_scaffold*	83724	G/C	Gene_Ca_26955	NON_SYNONYMOUS_CODING	Transposase,Ptta/En/Spm,plant
Kabuli_wild_83732	1399944393	*Ca_kabuli_scaffold*	83732	C/A	Gene_Ca_26955	NON_SYNONYMOUS_CODING	Transposase,Ptta/En/Spm,plant
Kabuli_wild_83898	1399944394	*Ca_kabuli_scaffold*	83898	G/T	Gene_Ca_26955	NON_SYNONYMOUS_CODING	Transposase,Ptta/En/Spm,plant
Kabuli_wild_129171	1399944395	*Ca_kabuli_scaffold*	129171	C/T	NA	INTERGENIC	NA
Kabuli_wild_132825	1399944396	*Ca_kabuli_scaffold*	132825	C/T	NA	INTERGENIC	NA
Kabuli_wild_132843	1399944397	*Ca_kabuli_scaffold*	132843	C/T	NA	INTERGENIC	NA
Kabuli_wild_132849	1399944398	*Ca_kabuli_scaffold*	132849	A/C	NA	INTERGENIC	NA
Kabuli_wild_132858	1399944399	*Ca_kabuli_scaffold*	132858	G/A	NA	INTERGENIC	NA
Kabuli_wild_132859	1399944400	*Ca_kabuli_scaffold*	132859	C/T	NA	INTERGENIC	NA
Kabuli_wild_132873	1399944401	*Ca_kabuli_scaffold*	132873	G/A	NA	INTERGENIC	NA
Kabuli_wild_132896	1399944402	*Ca_kabuli_scaffold*	132896	C/T	NA	INTERGENIC	NA
Kabuli_wild_132907	1399944403	*Ca_kabuli_scaffold*	132907	A/C	NA	INTERGENIC	NA
Kabuli_wild_54813	1399944404	*Ca_kabuli_scaffold*	54813	T/C	NA	INTERGENIC	NA
Kabuli_wild_393535	1399944405	*Ca_kabuli_scaffold*	393535	G/T	NA	INTERGENIC	NA
Kabuli_wild_69075	1399944406	*Ca_kabuli_scaffold*	69075	A/C	Gene_Ca_26154	NON_SYNONYMOUS_CODING	PGAP1-like
Kabuli_wild_144442	1399944407	*Ca_kabuli_scaffold*	144442	G/A	NA	INTERGENIC	NA
Kabuli_wild_177270	1399944408	*Ca_kabuli_scaffold*	177270	T/C	NA	INTERGENIC	NA
Kabuli_wild_177306	1399944409	*Ca_kabuli_scaffold*	177306	T/C	NA	INTERGENIC	NA
Kabuli_wild_177340	1399944410	*Ca_kabuli_scaffold*	177340	C/T	NA	INTERGENIC	NA
Kabuli_wild_177354	1399944411	*Ca_kabuli_scaffold*	177354	G/A	NA	INTERGENIC	NA
Kabuli_wild_177388	1399944412	*Ca_kabuli_scaffold*	177388	C/G	NA	INTERGENIC	NA
Kabuli_wild_177685	1399944413	*Ca_kabuli_scaffold*	177685	T/C	NA	INTERGENIC	NA
Kabuli_wild_178945	1399944414	*Ca_kabuli_scaffold*	178945	T/C	NA	INTERGENIC	NA
Kabuli_wild_227608	1399944415	*Ca_kabuli_scaffold*	227608	A/T	NA	INTERGENIC	NA
Kabuli_wild_36686	1399944416	*Ca_kabuli_scaffold*	36686	A/T	Gene_Ca_27060	INTRON	NA
Kabuli_wild_36703	1399944417	*Ca_kabuli_scaffold*	36703	A/T	Gene_Ca_27060	INTRON	NA
Kabuli_wild_36704	1399944418	*Ca_kabuli_scaffold*	36704	T/G	Gene_Ca_27060	INTRON	NA
Kabuli_wild_36709	1399944419	*Ca_kabuli_scaffold*	36709	G/A	Gene_Ca_27060	INTRON	NA
Kabuli_wild_38767	1399944420	*Ca_kabuli_scaffold*	38767	C/T	Gene_Ca_27060	INTRON	NA
Kabuli_wild_107659	1399944421	*Ca_kabuli_scaffold*	107659	G/A	NA	INTERGENIC	NA
Kabuli_wild_107669	1399944422	*Ca_kabuli_scaffold*	107669	G/A	NA	INTERGENIC	NA
Kabuli_wild_107671	1399944423	*Ca_kabuli_scaffold*	107671	A/G	NA	INTERGENIC	NA
Kabuli_wild_107678	1399944424	*Ca_kabuli_scaffold*	107678	A/T	NA	INTERGENIC	NA
Kabuli_wild_107704	1399944425	*Ca_kabuli_scaffold*	107704	G/A	NA	INTERGENIC	NA
Kabuli_wild_3175	1399944426	*Ca_kabuli_scaffold*	3175	A/G	NA	INTERGENIC	NA
Kabuli_wild_3221	1399944427	*Ca_kabuli_scaffold*	3221	C/A	NA	INTERGENIC	NA
Kabuli_wild_3238	1399944428	*Ca_kabuli_scaffold*	3238	G/T	NA	INTERGENIC	NA
Kabuli_wild_3263	1399944429	*Ca_kabuli_scaffold*	3263	A/G	NA	INTERGENIC	NA
Kabuli_wild_3266	1399944430	*Ca_kabuli_scaffold*	3266	G/A	NA	INTERGENIC	NA
Kabuli_wild_3271	1399944431	*Ca_kabuli_scaffold*	3271	A/T	NA	INTERGENIC	NA

SNP IDs	NCBI dbSNP accession IDs (NCBI_SS#)	Chromosomes	Physical positions (bp)	SNPs	Gene accession IDs	Sequence components of genome	Putative functions
Kabuli_wild_3277	1399944432	*Ca_kabuli_scaffold*	3277	A/C	NA	INTERGENIC	NA
Kabuli_wild_3309	1399944433	*Ca_kabuli_scaffold*	3309	T/C	NA	INTERGENIC	NA
Kabuli_wild_130572	1399944434	*Ca_kabuli_scaffold*	130572	G/A	NA	INTERGENIC	NA
Kabuli_wild_130580	1399944435	*Ca_kabuli_scaffold*	130580	T/C	NA	INTERGENIC	NA
Kabuli_wild_130583	1399944436	*Ca_kabuli_scaffold*	130583	C/T	NA	INTERGENIC	NA
Kabuli_wild_130587	1399944437	*Ca_kabuli_scaffold*	130587	C/A	NA	INTERGENIC	NA
Kabuli_wild_130589	1399944438	*Ca_kabuli_scaffold*	130589	C/A	NA	INTERGENIC	NA
Kabuli_wild_130611	1399944439	*Ca_kabuli_scaffold*	130611	C/T	NA	INTERGENIC	NA
Kabuli_wild_130614	1399944440	*Ca_kabuli_scaffold*	130614	A/G	NA	INTERGENIC	NA
Kabuli_wild_130621	1399944441	*Ca_kabuli_scaffold*	130621	C/A	NA	INTERGENIC	NA
Kabuli_wild_130624	1399944442	*Ca_kabuli_scaffold*	130624	A/G	NA	INTERGENIC	NA
Kabuli_wild_130635	1399944443	*Ca_kabuli_scaffold*	130635	G/T	NA	INTERGENIC	NA
Kabuli_wild_130636	1399944444	*Ca_kabuli_scaffold*	130636	G/A	NA	INTERGENIC	NA
Kabuli_wild_130639	1399944445	*Ca_kabuli_scaffold*	130639	C/A	NA	INTERGENIC	NA
Kabuli_wild_130664	1399944446	*Ca_kabuli_scaffold*	130664	T/C	NA	INTERGENIC	NA
Kabuli_wild_130671	1399944447	*Ca_kabuli_scaffold*	130671	A/G	NA	INTERGENIC	NA
Kabuli_wild_139314	1399944448	*Ca_kabuli_scaffold*	139314	G/T	NA	INTERGENIC	NA
Kabuli_wild_195890	1399944449	*Ca_kabuli_scaffold*	195890	A/G	NA	INTERGENIC	NA
Kabuli_wild_296448	1399944450	*Ca_kabuli_scaffold*	296448	A/C	Gene_Ca_23551	INTRON	SecYprotein
Kabuli_wild_297325	1399944451	*Ca_kabuli_scaffold*	297325	G/C	Gene_Ca_23551	INTRON	SecYprotein
Kabuli_wild_178515	1399944452	*Ca_kabuli_scaffold*	178515	T/C	NA	INTERGENIC	NA
Kabuli_wild_246920	1399944453	*Ca_kabuli_scaffold*	246920	C/A	Gene_Ca_22286	NON_SYNONYMOUS_CODING	Peptidase C48, SUMO/Sentrin/Ubl1
Kabuli_wild_246922	1399944454	*Ca_kabuli_scaffold*	246922	G/A	Gene_Ca_22286	NON_SYNONYMOUS_CODING	Peptidase C48, SUMO/Sentrin/Ubl1
Kabuli_wild_246987	1399944455	*Ca_kabuli_scaffold*	246987	G/A	Gene_Ca_22286	SYNONYMOUS_CODING	Peptidase C48, SUMO/Sentrin/Ubl1
Kabuli_wild_246996	1399944456	*Ca_kabuli_scaffold*	246996	G/T	Gene_Ca_22286	SYNONYMOUS_CODING	Peptidase C48, SUMO/Sentrin/Ubl1
Kabuli_wild_247001	1399944457	*Ca_kabuli_scaffold*	247001	C/T	Gene_Ca_22286	NON_SYNONYMOUS_CODING	Peptidase C48, SUMO/Sentrin/Ubl1
Kabuli_wild_256286	1399944458	*Ca_kabuli_scaffold*	256286	G/T	Gene_Ca_22287	INTRON	Protein of unknown function DUF620
Kabuli_wild_321050	1399944459	*Ca_kabuli_scaffold*	321050	G/A	NA	INTERGENIC	NA
Kabuli_wild_441570	1399944460	*Ca_kabuli_scaffold*	441570	C/T	NA	INTERGENIC	NA
Kabuli_wild_441605	1399944461	*Ca_kabuli_scaffold*	441605	C/A	NA	INTERGENIC	NA
Kabuli_wild_49060	1399944462	*Ca_kabuli_scaffold*	49060	T/C	Gene_Ca_25591	SYNONYMOUS_CODING	NA
Kabuli_wild_49065	1399944463	*Ca_kabuli_scaffold*	49065	G/A	Gene_Ca_25591	STOP_GAINED	NA
Kabuli_wild_49076	1399944464	*Ca_kabuli_scaffold*	49076	G/T	Gene_Ca_25591	NON_SYNONYMOUS_CODING	NA
Kabuli_wild_272574	1399944465	*Ca_kabuli_scaffold*	272574	A/G	NA	INTERGENIC	NA
Kabuli_wild_273155	1399944466	*Ca_kabuli_scaffold*	273155	A/G	NA	INTERGENIC	NA
Kabuli_wild_273203	1399944467	*Ca_kabuli_scaffold*	273203	C/A	NA	INTERGENIC	NA
Kabuli_wild_273209	1399944468	*Ca_kabuli_scaffold*	273209	C/A	NA	INTERGENIC	NA
Kabuli_wild_273220	1399944469	*Ca_kabuli_scaffold*	273220	G/A	NA	INTERGENIC	NA
Kabuli_wild_273245	1399944470	*Ca_kabuli_scaffold*	273245	C/T	NA	INTERGENIC	NA
Kabuli_wild_285952	1399944471	*Ca_kabuli_scaffold*	285952	T/C	NA	INTERGENIC	NA
Kabuli_wild_285958	1399944472	*Ca_kabuli_scaffold*	285958	C/G	NA	INTERGENIC	NA
Kabuli_wild_285999	1399944473	*Ca_kabuli_scaffold*	285999	T/G	NA	INTERGENIC	NA
Kabuli_wild_355850	1399944474	*Ca_kabuli_scaffold*	355850	G/A	Gene_Ca_24364	NON_SYNONYMOUS_CODING	NA
Kabuli_wild_355857	1399944475	*Ca_kabuli_scaffold*	355857	C/T	Gene_Ca_24364	NON_SYNONYMOUS_CODING	NA

SNP IDs	NCBI dbSNP accession IDs (NCBI_SS#)	Chromosomes	Physical positions (bp)	SNPs	Gene accession IDs	Sequence components of genome	Putative functions
Kabuli_wild_355859	1399944476	*Ca_kabuli_scaffold*	355859	G/A	Gene_Ca_24364	NON_SYNONYMOUS_CODING	NA
Kabuli_wild_355875	1399944477	*Ca_kabuli_scaffold*	355875	G/T	Gene_Ca_24364	NON_SYNONYMOUS_CODING	NA
Kabuli_wild_355898	1399944478	*Ca_kabuli_scaffold*	355898	G/A	Gene_Ca_24364	NON_SYNONYMOUS_CODING	NA
Kabuli_wild_355932	1399944479	*Ca_kabuli_scaffold*	355932	C/A	Gene_Ca_24364	NON_SYNONYMOUS_CODING	NA
Kabuli_wild_378363	1399944480	*Ca_kabuli_scaffold*	378363	T/A	Gene_Ca_24364	INTRON	NA
Kabuli_wild_90740	1399944481	*Ca_kabuli_scaffold*	90740	G/T	Gene_Ca_25277	UPSTREAM	NA
Kabuli_wild_90813	1399944482	*Ca_kabuli_scaffold*	90813	C/T	Gene_Ca_25277	UPSTREAM	NA
Kabuli_wild_60078	1399944483	*Ca_kabuli_scaffold*	60078	C/T	NA	INTERGENIC	NA
Kabuli_wild_60099	1399944484	*Ca_kabuli_scaffold*	60099	C/T	NA	INTERGENIC	NA
Kabuli_wild_62060	1399944485	*Ca_kabuli_scaffold*	62060	G/A	NA	INTERGENIC	NA
Kabuli_wild_62073	1399944486	*Ca_kabuli_scaffold*	62073	T/C	NA	INTERGENIC	NA
Kabuli_wild_62246	1399944487	*Ca_kabuli_scaffold*	62246	A/G	NA	INTERGENIC	NA
Kabuli_wild_231515	1399944488	*Ca_kabuli_scaffold*	231515	G/T	NA	INTERGENIC	NA
Kabuli_wild_231580	1399944489	*Ca_kabuli_scaffold*	231580	G/A	NA	INTERGENIC	NA
Kabuli_wild_231603	1399944490	*Ca_kabuli_scaffold*	231603	G/A	NA	INTERGENIC	NA
Kabuli_wild_231808	1399944491	*Ca_kabuli_scaffold*	231808	C/T	NA	INTERGENIC	NA
Kabuli_wild_231819	1399944492	*Ca_kabuli_scaffold*	231819	A/C	NA	INTERGENIC	NA
Kabuli_wild_231837	1399944493	*Ca_kabuli_scaffold*	231837	G/A	NA	INTERGENIC	NA
Kabuli_wild_272150	1399944494	*Ca_kabuli_scaffold*	272150	A/G	NA	INTERGENIC	NA
Kabuli_wild_323122	1399944495	*Ca_kabuli_scaffold*	323122	T/C	NA	INTERGENIC	NA
Kabuli_wild_323128	1399944496	*Ca_kabuli_scaffold*	323128	G/A	NA	INTERGENIC	NA
Kabuli_wild_323140	1399944497	*Ca_kabuli_scaffold*	323140	T/C	NA	INTERGENIC	NA
Kabuli_wild_323141	1399944498	*Ca_kabuli_scaffold*	323141	G/A	NA	INTERGENIC	NA
Kabuli_wild_323147	1399944499	*Ca_kabuli_scaffold*	323147	T/C	NA	INTERGENIC	NA
Kabuli_wild_323161	1399944500	*Ca_kabuli_scaffold*	323161	G/A	NA	INTERGENIC	NA
Kabuli_wild_323162	1399944501	*Ca_kabuli_scaffold*	323162	T/C	NA	INTERGENIC	NA
Kabuli_wild_323167	1399944502	*Ca_kabuli_scaffold*	323167	C/G	NA	INTERGENIC	NA
Kabuli_wild_323171	1399944503	*Ca_kabuli_scaffold*	323171	G/A	NA	INTERGENIC	NA
Kabuli_wild_323181	1399944504	*Ca_kabuli_scaffold*	323181	C/A	NA	INTERGENIC	NA
Kabuli_wild_323219	1399944505	*Ca_kabuli_scaffold*	323219	A/C	NA	INTERGENIC	NA
Kabuli_wild_8479	1399944506	*Ca_kabuli_scaffold*	8479	A/C	Gene_Ca_28252	NON_SYNONYMOUS_CODING	NA
Kabuli_wild_6092	1399944507	*Ca_kabuli_scaffold*	6092	C/G	Gene_Ca_24670	INTRON	PeptidaseM48
Kabuli_wild_13769	1399944508	*Ca_kabuli_scaffold*	13769	T/G	NA	INTERGENIC	NA
Kabuli_wild_13809	1399944509	*Ca_kabuli_scaffold*	13809	C/T	NA	INTERGENIC	NA
Kabuli_wild_13811	1399944510	*Ca_kabuli_scaffold*	13811	G/A	NA	INTERGENIC	NA
Kabuli_wild_13812	1399944511	*Ca_kabuli_scaffold*	13812	C/A	NA	INTERGENIC	NA
Kabuli_wild_151121	1399944512	*Ca_kabuli_scaffold*	151121	C/A	NA	INTERGENIC	NA
Kabuli_wild_151123	1399944513	*Ca_kabuli_scaffold*	151123	G/A	NA	INTERGENIC	NA
Kabuli_wild_151124	1399944514	*Ca_kabuli_scaffold*	151124	C/G	NA	INTERGENIC	NA
Kabuli_wild_151138	1399944515	*Ca_kabuli_scaffold*	151138	C/T	NA	INTERGENIC	NA
Kabuli_wild_151149	1399944516	*Ca_kabuli_scaffold*	151149	T/C	NA	INTERGENIC	NA
Kabuli_wild_151196	1399944517	*Ca_kabuli_scaffold*	151196	C/T	NA	INTERGENIC	NA
Kabuli_wild_151201	1399944518	*Ca_kabuli_scaffold*	151201	G/C	NA	INTERGENIC	NA
Kabuli_wild_151205	1399944519	*Ca_kabuli_scaffold*	151205	C/T	NA	INTERGENIC	NA

SNP IDs	NCBI dbSNP accession IDs (NCBI_SS#)	Chromosomes	Physical positions (bp)	SNPs	Gene accession IDs	Sequence components of genome	Putative functions
Kabuli_wild_151219	1399944520	*Ca_kabuli_scaffold*	151219	A/G	NA	INTERGENIC	NA
Kabuli_wild_151256	1399944521	*Ca_kabuli_scaffold*	151256	A/G	NA	INTERGENIC	NA
Kabuli_wild_151273	1399944522	*Ca_kabuli_scaffold*	151273	A/G	NA	INTERGENIC	NA
Kabuli_wild_151278	1399944523	*Ca_kabuli_scaffold*	151278	G/A	NA	INTERGENIC	NA
Kabuli_wild_73537	1399944524	*Ca_kabuli_scaffold*	73537	T/G	Gene_Ca_26396	UPSTREAM	Disease resistance protein
Kabuli_wild_32546	1399944525	*Ca_kabuli_scaffold*	32546	T/C	Gene_Ca_22195	INTRON	Zinc finger,PHD-type
Kabuli_wild_34120	1399944526	*Ca_kabuli_scaffold*	34120	C/A	Gene_Ca_22195	STOP_GAINED	Zinc finger,PHD-type
Kabuli_wild_34145	1399944527	*Ca_kabuli_scaffold*	34145	T/G	Gene_Ca_22195	NON_SYNONYMOUS_CODING	Zinc finger,PHD-type
Kabuli_wild_42532	1399944528	*Ca_kabuli_scaffold*	42532	G/A	Gene_Ca_22196	SYNONYMOUS_CODING	Ubiquitin
Kabuli_wild_42739	1399944529	*Ca_kabuli_scaffold*	42739	C/T	Gene_Ca_22196	SYNONYMOUS_CODING	Ubiquitin
Kabuli_wild_42811	1399944530	*Ca_kabuli_scaffold*	42811	C/T	Gene_Ca_22196	SYNONYMOUS_CODING	Ubiquitin
Kabuli_wild_42877	1399944531	*Ca_kabuli_scaffold*	42877	T/C	Gene_Ca_22196	SYNONYMOUS_CODING	Ubiquitin
Kabuli_wild_43372	1399944532	*Ca_kabuli_scaffold*	43372	C/T	Gene_Ca_22196	SYNONYMOUS_CODING	Ubiquitin
Kabuli_wild_44242	1399944533	*Ca_kabuli_scaffold*	44242	C/T	Gene_Ca_22196	SYNONYMOUS_CODING	Ubiquitin
Kabuli_wild_70995	1399944534	*Ca_kabuli_scaffold*	70995	T/G	NA	INTERGENIC	NA
Kabuli_wild_71020	1399944535	*Ca_kabuli_scaffold*	71020	G/A	NA	INTERGENIC	NA
Kabuli_wild_71048	1399944536	*Ca_kabuli_scaffold*	71048	C/G	NA	INTERGENIC	NA
Kabuli_wild_85267	1399944537	*Ca_kabuli_scaffold*	85267	G/A	NA	INTERGENIC	NA
Kabuli_wild_85327	1399944538	*Ca_kabuli_scaffold*	85327	T/G	NA	INTERGENIC	NA
Kabuli_wild_85344	1399944539	*Ca_kabuli_scaffold*	85344	A/C	NA	INTERGENIC	NA
Kabuli_wild_85351	1399944540	*Ca_kabuli_scaffold*	85351	G/T	NA	INTERGENIC	NA
Kabuli_wild_85352	1399944541	*Ca_kabuli_scaffold*	85352	G/A	NA	INTERGENIC	NA
Kabuli_wild_92756	1399944542	*Ca_kabuli_scaffold*	92756	C/T	NA	INTERGENIC	NA
Kabuli_wild_92759	1399944543	*Ca_kabuli_scaffold*	92759	A/G	NA	INTERGENIC	NA
Kabuli_wild_92784	1399944544	*Ca_kabuli_scaffold*	92784	C/T	NA	INTERGENIC	NA
Kabuli_wild_92813	1399944545	*Ca_kabuli_scaffold*	92813	G/T	NA	INTERGENIC	NA
Kabuli_wild_92837	1399944546	*Ca_kabuli_scaffold*	92837	A/T	NA	INTERGENIC	NA
Kabuli_wild_149744	1399944547	*Ca_kabuli_scaffold*	149744	C/G	Gene_Ca_22199	INTRON	Protein kinase,catalytic domain
Kabuli_wild_235121	1399944548	*Ca_kabuli_scaffold*	235121	T/C	NA	INTERGENIC	NA
Kabuli_wild_242487	1399944549	*Ca_kabuli_scaffold*	242487	A/G	NA	INTERGENIC	NA
Kabuli_wild_242503	1399944550	*Ca_kabuli_scaffold*	242503	C/T	NA	INTERGENIC	NA
Kabuli_wild_242922	1399944551	*Ca_kabuli_scaffold*	242922	A/C	NA	INTERGENIC	NA
Kabuli_wild_254661	1399944552	*Ca_kabuli_scaffold*	254661	T/G	Gene_Ca_22205	INTRON	Fattyaciddesaturase,type1
Kabuli_wild_265077	1399944553	*Ca_kabuli_scaffold*	265077	T/C	NA	INTERGENIC	NA
Kabuli_wild_265085	1399944554	*Ca_kabuli_scaffold*	265085	G/A	NA	INTERGENIC	NA
Kabuli_wild_265101	1399944555	*Ca_kabuli_scaffold*	265101	A/C	NA	INTERGENIC	NA
Kabuli_wild_265105	1399944556	*Ca_kabuli_scaffold*	265105	T/C	NA	INTERGENIC	NA
Kabuli_wild_326938	1399944557	*Ca_kabuli_scaffold*	326938	G/T	NA	INTERGENIC	NA
Kabuli_wild_331058	1399944558	*Ca_kabuli_scaffold*	331058	T/C	NA	INTERGENIC	NA
Kabuli_wild_341184	1399944559	*Ca_kabuli_scaffold*	341184	A/G	Gene_Ca_22212	INTRON	Glycosidehydrolase,family1
Kabuli_wild_341202	1399944560	*Ca_kabuli_scaffold*	341202	T/G	Gene_Ca_22212	INTRON	Glycosidehydrolase,family1
Kabuli_wild_341206	1399944561	*Ca_kabuli_scaffold*	341206	G/A	Gene_Ca_22212	INTRON	Glycosidehydrolase,family1
Kabuli_wild_32402	1399944562	*Ca_kabuli_scaffold*	32402	C/G	Gene_Ca_27938	NON_SYNONYMOUS_CODING	RNA-binding,CRMdomain
Kabuli_wild_182706	1399944563	*Ca_kabuli_scaffold*	182706	T/C	Gene_Ca_26242	DOWNSTREAM	NA

SNP IDs	NCBI dbSNP accession IDs (NCBI_SS#)	Chromosomes	Physical positions (bp)	SNPs	Gene accession IDs	Sequence components of genome	Putative functions
Kabuli_wild_1387	1399944564	*Ca_kabuli_scaffold*	1387	G/A	NA	INTERGENIC	NA
Kabuli_wild_1390	1399944565	*Ca_kabuli_scaffold*	1390	G/A	NA	INTERGENIC	NA
Kabuli_wild_1450	1399944566	*Ca_kabuli_scaffold*	1450	G/A	NA	INTERGENIC	NA
Kabuli_wild_132402	1399944567	*Ca_kabuli_scaffold*	132402	T/A	Gene_Ca_23794	NON_SYNONYMOUS_CODING	NA
Kabuli_wild_132427	1399944568	*Ca_kabuli_scaffold*	132427	A/T	Gene_Ca_23794	SYNONYMOUS_CODING	NA
Kabuli_wild_197226	1399944569	*Ca_kabuli_scaffold*	197226	G/A	NA	INTERGENIC	NA
Kabuli_wild_197230	1399944570	*Ca_kabuli_scaffold*	197230	C/A	NA	INTERGENIC	NA
Kabuli_wild_197251	1399944571	*Ca_kabuli_scaffold*	197251	G/C	NA	INTERGENIC	NA
Kabuli_wild_197261	1399944572	*Ca_kabuli_scaffold*	197261	C/T	NA	INTERGENIC	NA
Kabuli_wild_197262	1399944573	*Ca_kabuli_scaffold*	197262	T/G	NA	INTERGENIC	NA
Kabuli_wild_197272	1399944574	*Ca_kabuli_scaffold*	197272	G/A	NA	INTERGENIC	NA
Kabuli_wild_197287	1399944575	*Ca_kabuli_scaffold*	197287	G/A	NA	INTERGENIC	NA
Kabuli_wild_250303	1399944576	*Ca_kabuli_scaffold*	250303	G/T	NA	INTERGENIC	NA
Kabuli_wild_73786	1399944577	*Ca_kabuli_scaffold*	73786	T/C	NA	INTERGENIC	NA
Kabuli_wild_103510	1399944578	*Ca_kabuli_scaffold*	103510	G/A	NA	INTERGENIC	NA
Kabuli_wild_103555	1399944579	*Ca_kabuli_scaffold*	103555	G/C	NA	INTERGENIC	NA
Kabuli_wild_103596	1399944580	*Ca_kabuli_scaffold*	103596	A/G	NA	INTERGENIC	NA
Kabuli_wild_1835	1399944581	*Ca_kabuli_scaffold*	1835	C/T	Gene_Ca_28102	DOWNSTREAM	NA
Kabuli_wild_1836	1399944582	*Ca_kabuli_scaffold*	1836	A/G	Gene_Ca_28102	DOWNSTREAM	NA
Kabuli_wild_1837	1399944583	*Ca_kabuli_scaffold*	1837	A/C	Gene_Ca_28102	DOWNSTREAM	NA
Kabuli_wild_1841	1399944584	*Ca_kabuli_scaffold*	1841	A/G	Gene_Ca_28102	DOWNSTREAM	NA
Kabuli_wild_1881	1399944585	*Ca_kabuli_scaffold*	1881	A/G	Gene_Ca_28102	DOWNSTREAM	NA
Kabuli_wild_1892	1399944586	*Ca_kabuli_scaffold*	1892	G/A	Gene_Ca_28102	DOWNSTREAM	NA
Kabuli_wild_43430	1399944587	*Ca_kabuli_scaffold*	43430	C/A	Gene_Ca_21726	NON_SYNONYMOUS_CODING	PeptidaseS8/S53,subtilisin/kexin/sedolisin
Kabuli_wild_141029	1399944588	*Ca_kabuli_scaffold*	141029	C/T	NA	INTERGENIC	NA
Kabuli_wild_179369	1399944589	*Ca_kabuli_scaffold*	179369	A/G	Gene_Ca_21734	INTRON	NA
Kabuli_wild_303193	1399944590	*Ca_kabuli_scaffold*	303193	T/C	NA	INTERGENIC	NA
Kabuli_wild_303201	1399944591	*Ca_kabuli_scaffold*	303201	G/C	NA	INTERGENIC	NA
Kabuli_wild_303246	1399944592	*Ca_kabuli_scaffold*	303246	A/G	NA	INTERGENIC	NA
Kabuli_wild_303248	1399944593	*Ca_kabuli_scaffold*	303248	C/T	NA	INTERGENIC	NA
Kabuli_wild_303259	1399944594	*Ca_kabuli_scaffold*	303259	C/G	NA	INTERGENIC	NA
Kabuli_wild_330997	1399944595	*Ca_kabuli_scaffold*	330997	G/A	NA	INTERGENIC	NA
Kabuli_wild_478826	1399944596	*Ca_kabuli_scaffold*	478826	C/A	NA	INTERGENIC	NA
Kabuli_wild_478875	1399944597	*Ca_kabuli_scaffold*	478875	A/T	NA	INTERGENIC	NA
Kabuli_wild_20603	1399944598	*Ca_kabuli_scaffold*	20603	C/T	NA	INTERGENIC	NA
Kabuli_wild_143174	1399944599	*Ca_kabuli_scaffold*	143174	A/G	NA	INTERGENIC	NA
Kabuli_wild_143182	1399944600	*Ca_kabuli_scaffold*	143182	C/T	NA	INTERGENIC	NA
Kabuli_wild_166729	1399944601	*Ca_kabuli_scaffold*	166729	C/T	Gene_Ca_25699	INTRON	NA
Kabuli_wild_166746	1399944602	*Ca_kabuli_scaffold*	166746	A/G	Gene_Ca_25699	INTRON	NA
Kabuli_wild_166747	1399944603	*Ca_kabuli_scaffold*	166747	A/G	Gene_Ca_25699	INTRON	NA
Kabuli_wild_166748	1399944604	*Ca_kabuli_scaffold*	166748	G/T	Gene_Ca_25699	INTRON	NA
Kabuli_wild_166756	1399944605	*Ca_kabuli_scaffold*	166756	G/T	Gene_Ca_25699	INTRON	NA
Kabuli_wild_166769	1399944606	*Ca_kabuli_scaffold*	166769	A/G	Gene_Ca_25699	INTRON	NA
Kabuli_wild_564	1399944607	*Ca_kabuli_scaffold*	564	T/C	NA	INTERGENIC	NA

SNP IDs	NCBI dbSNP accession IDs (NCBI_SS#)	Chromosomes	Physical positions (bp)	SNPs	Gene accession IDs	Sequence components of genome	Putative functions
Kabuli_wild_135286	1399944608	*Ca_kabuli_scaffold*	135286	C/A	NA	INTERGENIC	NA
Kabuli_wild_135290	1399944609	*Ca_kabuli_scaffold*	135290	G/A	NA	INTERGENIC	NA
Kabuli_wild_135647	1399944610	*Ca_kabuli_scaffold*	135647	A/G	NA	INTERGENIC	NA
Kabuli_wild_146831	1399944611	*Ca_kabuli_scaffold*	146831	C/G	NA	INTERGENIC	NA
Kabuli_wild_162366	1399944612	*Ca_kabuli_scaffold*	162366	C/A	NA	INTERGENIC	NA
Kabuli_wild_8270	1399944613	*Ca_kabuli_scaffold*	8270	C/T	NA	INTERGENIC	NA
Kabuli_wild_8303	1399944614	*Ca_kabuli_scaffold*	8303	A/G	NA	INTERGENIC	NA
Kabuli_wild_51898	1399944615	*Ca_kabuli_scaffold*	51898	C/T	NA	INTERGENIC	NA
Kabuli_wild_67375	1399944616	*Ca_kabuli_scaffold*	67375	T/C	Gene_Ca_26870	INTRON	PeptidaseM24,structural domain
Kabuli_wild_202586	1399944617	*Ca_kabuli_scaffold*	202586	T/C	Gene_Ca_21177	NON_SYNONYMOUS_CODING	Cholinetransporter-like
Kabuli_wild_202616	1399944618	*Ca_kabuli_scaffold*	202616	C/T	Gene_Ca_21177	NON_SYNONYMOUS_CODING	Cholinetransporter-like
Kabuli_wild_209786	1399944619	*Ca_kabuli_scaffold*	209786	A/C	NA	INTERGENIC	NA
Kabuli_wild_243637	1399944620	*Ca_kabuli_scaffold*	243637	C/T	Gene_Ca_21179	NON_SYNONYMOUS_CODING	Pectatelyase/Amballergen
Kabuli_wild_341790	1399944621	*Ca_kabuli_scaffold*	341790	T/A	Gene_Ca_21181	SYNONYMOUS_CODING	NA
Kabuli_wild_341837	1399944622	*Ca_kabuli_scaffold*	341837	C/T	Gene_Ca_21181	NON_SYNONYMOUS_CODING	NA
Kabuli_wild_341838	1399944623	*Ca_kabuli_scaffold*	341838	A/C	Gene_Ca_21181	SYNONYMOUS_CODING	NA
Kabuli_wild_341880	1399944624	*Ca_kabuli_scaffold*	341880	A/C	Gene_Ca_21181	SYNONYMOUS_CODING	NA
Kabuli_wild_457422	1399944625	*Ca_kabuli_scaffold*	457422	C/T	NA	INTERGENIC	NA
Kabuli_wild_647786	1399944626	*Ca_kabuli_scaffold*	647786	T/C	NA	INTERGENIC	NA
Kabuli_wild_647809	1399944627	*Ca_kabuli_scaffold*	647809	A/T	NA	INTERGENIC	NA
Kabuli_wild_647866	1399944628	*Ca_kabuli_scaffold*	647866	A/G	NA	INTERGENIC	NA
Kabuli_wild_647868	1399944629	*Ca_kabuli_scaffold*	647868	C/T	NA	INTERGENIC	NA
Kabuli_wild_660749	1399944630	*Ca_kabuli_scaffold*	660749	C/G	NA	INTERGENIC	NA
Kabuli_wild_7749	1399944631	*Ca_kabuli_scaffold*	7749	C/T	Gene_Ca_25562	INTRON	NA
Kabuli_wild_23996	1399944632	*Ca_kabuli_scaffold*	23996	A/T	Gene_Ca_25563	NON_SYNONYMOUS_CODING	Carbamoyl-phosphatesynthase,GATase domain
Kabuli_wild_123578	1399944633	*Ca_kabuli_scaffold*	123578	T/C	NA	INTERGENIC	NA
Kabuli_wild_123598	1399944634	*Ca_kabuli_scaffold*	123598	T/A	NA	INTERGENIC	NA
Kabuli_wild_80897	1399944635	*Ca_kabuli_scaffold*	80897	C/A	NA	INTERGENIC	NA
Kabuli_wild_77906	1399944636	*Ca_kabuli_scaffold*	77906	A/G	NA	INTERGENIC	NA
Kabuli_wild_188381	1399944637	*Ca_kabuli_scaffold*	188381	G/C	NA	INTERGENIC	NA
Kabuli_wild_188406	1399944638	*Ca_kabuli_scaffold*	188406	G/C	NA	INTERGENIC	NA
Kabuli_wild_188630	1399944639	*Ca_kabuli_scaffold*	188630	T/C	NA	INTERGENIC	NA
Kabuli_wild_91185	1399944640	*Ca_kabuli_scaffold*	91185	T/C	Gene_Ca_26760	NON_SYNONYMOUS_CODING	PWWP
Kabuli_wild_111844	1399944641	*Ca_kabuli_scaffold*	111844	A/C	NA	INTERGENIC	NA
Kabuli_wild_111894	1399944642	*Ca_kabuli_scaffold*	111894	A/T	NA	INTERGENIC	NA
Kabuli_wild_111949	1399944643	*Ca_kabuli_scaffold*	111949	T/C	NA	INTERGENIC	NA
Kabuli_wild_128200	1399944644	*Ca_kabuli_scaffold*	128200	A/T	Gene_Ca_26761	INTRON	Pathogenesis-related transcriptional factor/ERF, DNA-binding
Kabuli_wild_133155	1399944645	*Ca_kabuli_scaffold*	133155	T/G	NA	INTERGENIC	NA
Kabuli_wild_133189	1399944646	*Ca_kabuli_scaffold*	133189	G/A	NA	INTERGENIC	NA
Kabuli_wild_133190	1399944647	*Ca_kabuli_scaffold*	133190	T/C	NA	INTERGENIC	NA
Kabuli_wild_133201	1399944648	*Ca_kabuli_scaffold*	133201	T/C	NA	INTERGENIC	NA
Kabuli_wild_133208	1399944649	*Ca_kabuli_scaffold*	133208	C/T	NA	INTERGENIC	NA
Kabuli_wild_133234	1399944650	*Ca_kabuli_scaffold*	133234	C/A	NA	INTERGENIC	NA
Kabuli_wild_133235	1399944651	*Ca_kabuli_scaffold*	133235	A/G	NA	INTERGENIC	NA

SNP IDs	NCBI dbSNP accession IDs (NCBI_SS#)	Chromosomes	Physical positions (bp)	SNPs	Gene accession IDs	Sequence components of genome	Putative functions
Kabuli_wild_133397	1399944652	*Ca_kabuli_scaffold*	133397	G/T	NA	INTERGENIC	NA
Kabuli_wild_53652	1399944653	*Ca_kabuli_scaffold*	53652	G/A	NA	INTERGENIC	NA
Kabuli_wild_53657	1399944654	*Ca_kabuli_scaffold*	53657	A/T	NA	INTERGENIC	NA
Kabuli_wild_175426	1399944655	*Ca_kabuli_scaffold*	175426	G/A	Gene_Ca_27202	NON_SYNONYMOUS_CODING	Peptidase C48, SUMO/Sentrin/Ubl1
Kabuli_wild_175427	1399944656	*Ca_kabuli_scaffold*	175427	C/T	Gene_Ca_27202	NON_SYNONYMOUS_CODING	Peptidase C48, SUMO/Sentrin/Ubl1
Kabuli_wild_175430	1399944657	*Ca_kabuli_scaffold*	175430	C/T	Gene_Ca_27202	NON_SYNONYMOUS_CODING	Peptidase C48, SUMO/Sentrin/Ubl1
Kabuli_wild_175433	1399944658	*Ca_kabuli_scaffold*	175433	C/T	Gene_Ca_27202	NON_SYNONYMOUS_CODING	Peptidase C48, SUMO/Sentrin/Ubl1
Kabuli_wild_175435	1399944659	*Ca_kabuli_scaffold*	175435	G/A	Gene_Ca_27202	NON_SYNONYMOUS_CODING	Peptidase C48, SUMO/Sentrin/Ubl1
Kabuli_wild_175478	1399944660	*Ca_kabuli_scaffold*	175478	C/T	Gene_Ca_27202	NON_SYNONYMOUS_CODING	Peptidase C48, SUMO/Sentrin/Ubl1
Kabuli_wild_175493	1399944661	*Ca_kabuli_scaffold*	175493	G/A	Gene_Ca_27202	SYNONYMOUS_CODING	Peptidase C48, SUMO/Sentrin/Ubl1
Kabuli_wild_175507	1399944662	*Ca_kabuli_scaffold*	175507	G/C	Gene_Ca_27202	NON_SYNONYMOUS_CODING	Peptidase C48, SUMO/Sentrin/Ubl1
Kabuli_wild_175508	1399944663	*Ca_kabuli_scaffold*	175508	C/T	Gene_Ca_27202	NON_SYNONYMOUS_CODING	Peptidase C48, SUMO/Sentrin/Ubl1
Kabuli_wild_175510	1399944664	*Ca_kabuli_scaffold*	175510	G/A	Gene_Ca_27202	NON_SYNONYMOUS_CODING	Peptidase C48, SUMO/Sentrin/Ubl1
Kabuli_wild_175523	1399944665	*Ca_kabuli_scaffold*	175523	C/T	Gene_Ca_27202	NON_SYNONYMOUS_CODING	Peptidase C48, SUMO/Sentrin/Ubl1
Kabuli_wild_45669	1399944666	*Ca_kabuli_scaffold*	45669	C/T	NA	INTERGENIC	NA
Kabuli_wild_12383	1399944667	*Ca_kabuli_scaffold*	12383	T/G	Gene_Ca_26545	DOWNSTREAM	NA
Kabuli_wild_12394	1399944668	*Ca_kabuli_scaffold*	12394	C/A	Gene_Ca_26545	DOWNSTREAM	NA
Kabuli_wild_12414	1399944669	*Ca_kabuli_scaffold*	12414	C/T	Gene_Ca_26545	DOWNSTREAM	NA
Kabuli_wild_12417	1399944670	*Ca_kabuli_scaffold*	12417	C/T	Gene_Ca_26545	DOWNSTREAM	NA
Kabuli_wild_10450	1399944671	*Ca_kabuli_scaffold*	10450	T/G	Gene_Ca_26652	SYNONYMOUS_CODING	NA
Kabuli_wild_10520	1399944672	*Ca_kabuli_scaffold*	10520	G/T	Gene_Ca_26652	INTRON	NA
Kabuli_wild_7188	1399944673	*Ca_kabuli_scaffold*	7188	A/C	NA	INTERGENIC	NA
Kabuli_wild_143283	1399944674	*Ca_kabuli_scaffold*	143283	C/T	NA	INTERGENIC	NA
Kabuli_wild_143291	1399944675	*Ca_kabuli_scaffold*	143291	G/A	NA	INTERGENIC	NA
Kabuli_wild_143294	1399944676	*Ca_kabuli_scaffold*	143294	G/A	NA	INTERGENIC	NA
Kabuli_wild_143300	1399944677	*Ca_kabuli_scaffold*	143300	G/A	NA	INTERGENIC	NA
Kabuli_wild_143317	1399944678	*Ca_kabuli_scaffold*	143317	T/A	NA	INTERGENIC	NA
Kabuli_wild_143318	1399944679	*Ca_kabuli_scaffold*	143318	T/C	NA	INTERGENIC	NA
Kabuli_wild_143324	1399944680	*Ca_kabuli_scaffold*	143324	G/A	NA	INTERGENIC	NA
Kabuli_wild_143333	1399944681	*Ca_kabuli_scaffold*	143333	A/T	NA	INTERGENIC	NA
Kabuli_wild_143334	1399944682	*Ca_kabuli_scaffold*	143334	G/A	NA	INTERGENIC	NA
Kabuli_wild_143336	1399944683	*Ca_kabuli_scaffold*	143336	T/C	NA	INTERGENIC	NA
Kabuli_wild_143355	1399944684	*Ca_kabuli_scaffold*	143355	C/T	NA	INTERGENIC	NA
Kabuli_wild_143358	1399944685	*Ca_kabuli_scaffold*	143358	G/A	NA	INTERGENIC	NA
Kabuli_wild_143359	1399944686	*Ca_kabuli_scaffold*	143359	C/T	NA	INTERGENIC	NA
Kabuli_wild_143372	1399944687	*Ca_kabuli_scaffold*	143372	G/A	NA	INTERGENIC	NA
Kabuli_wild_143391	1399944688	*Ca_kabuli_scaffold*	143391	A/C	NA	INTERGENIC	NA
Kabuli_wild_76993	1399944689	*Ca_kabuli_scaffold*	76993	T/C	NA	INTERGENIC	NA
Kabuli_wild_136669	1399944690	*Ca_kabuli_scaffold*	136669	C/T	NA	INTERGENIC	NA
Kabuli_wild_136692	1399944691	*Ca_kabuli_scaffold*	136692	G/A	NA	INTERGENIC	NA
Kabuli_wild_101108	1399944692	*Ca_kabuli_scaffold*	101108	T/C	NA	INTERGENIC	NA
Kabuli_wild_247957	1399944693	*Ca_kabuli_scaffold*	247957	T/G	NA	INTERGENIC	NA
Kabuli_wild_247964	1399944694	*Ca_kabuli_scaffold*	247964	G/A	NA	INTERGENIC	NA
Kabuli_wild_247987	1399944695	*Ca_kabuli_scaffold*	247987	C/A	NA	INTERGENIC	NA

SNP IDs	NCBI dbSNP accession IDs (NCBI_SS#)	Chromosomes	Physical positions (bp)	SNPs	Gene accession IDs	Sequence components of genome	Putative functions
Kabuli_wild_265923	1399944696	*Ca_kabuli_scaffold*	265923	T/C	NA	INTERGENIC	NA
Kabuli_wild_266984	1399944697	*Ca_kabuli_scaffold*	266984	T/G	Gene_Ca_24532	SYNONYMOUS_CODING	Protein kinase,catalytic domain
Kabuli_wild_2592	1399944698	*Ca_kabuli_scaffold*	2592	G/A	NA	INTERGENIC	NA
Kabuli_wild_2668	1399944699	*Ca_kabuli_scaffold*	2668	C/T	NA	INTERGENIC	NA
Kabuli_wild_65242	1399944700	*Ca_kabuli_scaffold*	65242	T/G	Gene_Ca_22392	DOWNSTREAM	Transposase,MuDR,plant
Kabuli_wild_101520	1399944701	*Ca_kabuli_scaffold*	101520	C/G	NA	INTERGENIC	NA
Kabuli_wild_101524	1399944702	*Ca_kabuli_scaffold*	101524	C/T	NA	INTERGENIC	NA
Kabuli_wild_124019	1399944703	*Ca_kabuli_scaffold*	124019	T/G	NA	INTERGENIC	NA
Kabuli_wild_144592	1399944704	*Ca_kabuli_scaffold*	144592	T/C	NA	INTERGENIC	NA
Kabuli_wild_156836	1399944705	*Ca_kabuli_scaffold*	156836	G/A	NA	INTERGENIC	NA
Kabuli_wild_146774	1399944706	*Ca_kabuli_scaffold*	146774	C/T	NA	INTERGENIC	NA
Kabuli_wild_147198	1399944707	*Ca_kabuli_scaffold*	147198	T/C	Gene_Ca_23281	SYNONYMOUS_CODING	Pentatrico peptide repeat
Kabuli_wild_216656	1399944708	*Ca_kabuli_scaffold*	216656	A/G	NA	INTERGENIC	NA
Kabuli_wild_220106	1399944709	*Ca_kabuli_scaffold*	220106	C/T	Gene_Ca_23287	NON_SYNONYMOUS_CODING	Sel1-like
Kabuli_wild_20780	1399944710	*Ca_kabuli_scaffold*	20780	T/G	NA	INTERGENIC	NA
Kabuli_wild_20819	1399944711	*Ca_kabuli_scaffold*	20819	T/C	NA	INTERGENIC	NA
Kabuli_wild_20827	1399944712	*Ca_kabuli_scaffold*	20827	G/T	NA	INTERGENIC	NA
Kabuli_wild_20980	1399944713	*Ca_kabuli_scaffold*	20980	T/G	NA	INTERGENIC	NA
Kabuli_wild_143169	1399944714	*Ca_kabuli_scaffold*	143169	C/T	NA	INTERGENIC	NA
Kabuli_wild_143191	1399944715	*Ca_kabuli_scaffold*	143191	G/A	NA	INTERGENIC	NA
Kabuli_wild_143202	1399944716	*Ca_kabuli_scaffold*	143202	G/T	NA	INTERGENIC	NA
Kabuli_wild_266729	1399944717	*Ca_kabuli_scaffold*	266729	A/C	NA	INTERGENIC	NA
Kabuli_wild_266748	1399944718	*Ca_kabuli_scaffold*	266748	A/C	NA	INTERGENIC	NA
Kabuli_wild_266848	1399944719	*Ca_kabuli_scaffold*	266848	A/C	NA	INTERGENIC	NA
Kabuli_wild_17699	1399944720	*Ca_kabuli_scaffold*	17699	A/C	NA	INTERGENIC	NA
Kabuli_wild_139118	1399944721	*Ca_kabuli_scaffold*	139118	A/T	Gene_Ca_23923	SYNONYMOUS_CODING	Zinc finger,C2H2-type
Kabuli_wild_147549	1399944722	*Ca_kabuli_scaffold*	147549	G/A	Gene_Ca_23924	NON_SYNONYMOUS_CODING	Uncharacterised protein familyUPF0503
Kabuli_wild_227045	1399944723	*Ca_kabuli_scaffold*	227045	C/T	Gene_Ca_23926	INTRON	WD40 repeat
Kabuli_wild_17462	1399944724	*Ca_kabuli_scaffold*	17462	G/A	Gene_Ca_21478	NON_SYNONYMOUS_CODING	Peptidase C48, SUMO/Sentrin/Ubl1
Kabuli_wild_17475	1399944725	*Ca_kabuli_scaffold*	17475	C/T	Gene_Ca_21478	SYNONYMOUS_CODING	Peptidase C48, SUMO/Sentrin/Ubl1
Kabuli_wild_231381	1399944726	*Ca_kabuli_scaffold*	231381	C/T	NA	INTERGENIC	NA
Kabuli_wild_231396	1399944727	*Ca_kabuli_scaffold*	231396	G/A	NA	INTERGENIC	NA
Kabuli_wild_231411	1399944728	*Ca_kabuli_scaffold*	231411	C/T	NA	INTERGENIC	NA
Kabuli_wild_231413	1399944729	*Ca_kabuli_scaffold*	231413	G/T	NA	INTERGENIC	NA
Kabuli_wild_231437	1399944730	*Ca_kabuli_scaffold*	231437	G/A	NA	INTERGENIC	NA
Kabuli_wild_347479	1399944731	*Ca_kabuli_scaffold*	347479	T/A	Gene_Ca_21493	STOP_GAINED	Cyclin
Kabuli_wild_25929	1399944732	*Ca_kabuli_scaffold*	25929	G/A	NA	INTERGENIC	NA
Kabuli_wild_235362	1399944733	*Ca_kabuli_scaffold*	235362	T/A	Gene_Ca_19930	INTRON	NA
Kabuli_wild_252722	1399944734	*Ca_kabuli_scaffold*	252722	T/G	Gene_Ca_19931	SYNONYMOUS_CODING	Leucine-rich repeat,cysteine-containingsubtype
Kabuli_wild_310759	1399944735	*Ca_kabuli_scaffold*	310759	T/A	Gene_Ca_19934	INTRON	NA
Kabuli_wild_401905	1399944736	*Ca_kabuli_scaffold*	401905	A/C	NA	INTERGENIC	NA
Kabuli_wild_441898	1399944737	*Ca_kabuli_scaffold*	441898	G/A	Gene_Ca_19938	INTRON	Protein kinase,catalytic domain
Kabuli_wild_794439	1399944738	*Ca_kabuli_scaffold*	794439	G/T	Gene_Ca_19955	NON_SYNONYMOUS_CODING	PeptidaseS8/S53,subtilisin/kexin/sedolisin
Kabuli_wild_794441	1399944739	*Ca_kabuli_scaffold*	794441	C/T	Gene_Ca_19955	NON_SYNONYMOUS_CODING	PeptidaseS8/S53,subtilisin/kexin/sedolisin

SNP IDs	NCBI dbSNP accession IDs (NCBI_SS#)	Chromosomes	Physical positions (bp)	SNPs	Gene accession IDs	Sequence components of genome	Putative functions
Kabuli_wild_49136	1399944740	*Ca_kabuli_scaffold*	49136	G/A	Gene_Ca_26217	SYNONYMOUS_CODING	Acetyl-CoAcarboxylase,alphasubunit
Kabuli_wild_76829	1399944741	*Ca_kabuli_scaffold*	76829	C/A	NA	INTERGENIC	NA
Kabuli_wild_76847	1399944742	*Ca_kabuli_scaffold*	76847	G/C	NA	INTERGENIC	NA
Kabuli_wild_76848	1399944743	*Ca_kabuli_scaffold*	76848	G/A	NA	INTERGENIC	NA
Kabuli_wild_76850	1399944744	*Ca_kabuli_scaffold*	76850	T/C	NA	INTERGENIC	NA
Kabuli_wild_76857	1399944745	*Ca_kabuli_scaffold*	76857	T/C	NA	INTERGENIC	NA
Kabuli_wild_76879	1399944746	*Ca_kabuli_scaffold*	76879	T/C	NA	INTERGENIC	NA
Kabuli_wild_84830	1399944747	*Ca_kabuli_scaffold*	84830	T/C	NA	INTERGENIC	NA
Kabuli_wild_84841	1399944748	*Ca_kabuli_scaffold*	84841	T/C	NA	INTERGENIC	NA
Kabuli_wild_84905	1399944749	*Ca_kabuli_scaffold*	84905	T/C	NA	INTERGENIC	NA
Kabuli_wild_221686	1399944750	*Ca_kabuli_scaffold*	221686	C/A	Gene_Ca_23135	NON_SYNONYMOUS_CODING	Peptidase C48, SUMO/Sentrin/Ubl1
Kabuli_wild_221714	1399944751	*Ca_kabuli_scaffold*	221714	C/T	Gene_Ca_23135	NON_SYNONYMOUS_CODING	Peptidase C48, SUMO/Sentrin/Ubl1
Kabuli_wild_221728	1399944752	*Ca_kabuli_scaffold*	221728	C/T	Gene_Ca_23135	NON_SYNONYMOUS_CODING	Peptidase C48, SUMO/Sentrin/Ubl1
Kabuli_wild_221730	1399944753	*Ca_kabuli_scaffold*	221730	C/G	Gene_Ca_23135	SYNONYMOUS_CODING	Peptidase C48, SUMO/Sentrin/Ubl1
Kabuli_wild_221733	1399944754	*Ca_kabuli_scaffold*	221733	G/T	Gene_Ca_23135	NON_SYNONYMOUS_CODING	Peptidase C48, SUMO/Sentrin/Ubl1
Kabuli_wild_221735	1399944755	*Ca_kabuli_scaffold*	221735	G/A	Gene_Ca_23135	NON_SYNONYMOUS_CODING	Peptidase C48, SUMO/Sentrin/Ubl1
Kabuli_wild_221743	1399944756	*Ca_kabuli_scaffold*	221743	G/C	Gene_Ca_23135	NON_SYNONYMOUS_CODING	Peptidase C48, SUMO/Sentrin/Ubl1
Kabuli_wild_221748	1399944757	*Ca_kabuli_scaffold*	221748	C/A	Gene_Ca_23135	SYNONYMOUS_CODING	Peptidase C48, SUMO/Sentrin/Ubl1
Kabuli_wild_221758	1399944758	*Ca_kabuli_scaffold*	221758	G/A	Gene_Ca_23135	NON_SYNONYMOUS_CODING	Peptidase C48, SUMO/Sentrin/Ubl1
Kabuli_wild_221769	1399944759	*Ca_kabuli_scaffold*	221769	T/A	Gene_Ca_23135	NON_SYNONYMOUS_CODING	Peptidase C48, SUMO/Sentrin/Ubl1
Kabuli_wild_221772	1399944760	*Ca_kabuli_scaffold*	221772	G/A	Gene_Ca_23135	SYNONYMOUS_CODING	Peptidase C48, SUMO/Sentrin/Ubl1
Kabuli_wild_221778	1399944761	*Ca_kabuli_scaffold*	221778	G/A	Gene_Ca_23135	SYNONYMOUS_CODING	Peptidase C48, SUMO/Sentrin/Ubl1
Kabuli_wild_221779	1399944762	*Ca_kabuli_scaffold*	221779	G/A	Gene_Ca_23135	NON_SYNONYMOUS_CODING	Peptidase C48, SUMO/Sentrin/Ubl1
Kabuli_wild_221788	1399944763	*Ca_kabuli_scaffold*	221788	G/A	Gene_Ca_23135	NON_SYNONYMOUS_CODING	Peptidase C48, SUMO/Sentrin/Ubl1
Kabuli_wild_221792	1399944764	*Ca_kabuli_scaffold*	221792	C/A	Gene_Ca_23135	NON_SYNONYMOUS_CODING	Peptidase C48, SUMO/Sentrin/Ubl1
Kabuli_wild_221824	1399944765	*Ca_kabuli_scaffold*	221824	C/T	Gene_Ca_23135	NON_SYNONYMOUS_CODING	Peptidase C48, SUMO/Sentrin/Ubl1
Kabuli_wild_221827	1399944766	*Ca_kabuli_scaffold*	221827	C/A	Gene_Ca_23135	NON_SYNONYMOUS_CODING	Peptidase C48, SUMO/Sentrin/Ubl1
Kabuli_wild_221828	1399944767	*Ca_kabuli_scaffold*	221828	T/A	Gene_Ca_23135	NON_SYNONYMOUS_CODING	Peptidase C48, SUMO/Sentrin/Ubl1
Kabuli_wild_221843	1399944768	*Ca_kabuli_scaffold*	221843	C/T	Gene_Ca_23135	NON_SYNONYMOUS_CODING	Peptidase C48, SUMO/Sentrin/Ubl1
Kabuli_wild_255295	1399944769	*Ca_kabuli_scaffold*	255295	G/A	Gene_Ca_23136	INTRON	NA
Kabuli_wild_255299	1399944770	*Ca_kabuli_scaffold*	255299	C/T	Gene_Ca_23136	INTRON	NA
Kabuli_wild_255319	1399944771	*Ca_kabuli_scaffold*	255319	A/T	Gene_Ca_23136	INTRON	NA
Kabuli_wild_255333	1399944772	*Ca_kabuli_scaffold*	255333	T/C	Gene_Ca_23136	INTRON	NA
Kabuli_wild_255375	1399944773	*Ca_kabuli_scaffold*	255375	G/T	Gene_Ca_23136	INTRON	NA
Kabuli_wild_289080	1399944774	*Ca_kabuli_scaffold*	289080	C/A	NA	INTERGENIC	NA
Kabuli_wild_289081	1399944775	*Ca_kabuli_scaffold*	289081	A/G	NA	INTERGENIC	NA
Kabuli_wild_290071	1399944776	*Ca_kabuli_scaffold*	290071	C/T	Gene_Ca_23141	INTRON	Protein of unknown function DUF642
Kabuli_wild_55364	1399944777	*Ca_kabuli_scaffold*	55364	G/A	NA	INTERGENIC	NA
Kabuli_wild_55369	1399944778	*Ca_kabuli_scaffold*	55369	T/C	NA	INTERGENIC	NA
Kabuli_wild_55401	1399944779	*Ca_kabuli_scaffold*	55401	T/A	NA	INTERGENIC	NA
Kabuli_wild_36238	1399944780	*Ca_kabuli_scaffold*	36238	C/T	Gene_Ca_27000	INTRON	NA
Kabuli_wild_45190	1399944781	*Ca_kabuli_scaffold*	45190	C/T	Gene_Ca_27001	UPSTREAM	NA
Kabuli_wild_45192	1399944782	*Ca_kabuli_scaffold*	45192	C/T	Gene_Ca_27001	UPSTREAM	NA
Kabuli_wild_45193	1399944783	*Ca_kabuli_scaffold*	45193	G/A	Gene_Ca_27001	UPSTREAM	NA

SNP IDs	NCBI dbSNP accession IDs (NCBI_SS#)	Chromosomes	Physical positions (bp)	SNPs	Gene accession IDs	Sequence components of genome	Putative functions
Kabuli_wild_45215	1399944784	*Ca_kabuli_scaffold*	45215	C/A	Gene_Ca_27001	UPSTREAM	NA
Kabuli_wild_45228	1399944785	*Ca_kabuli_scaffold*	45228	C/T	Gene_Ca_27001	UPSTREAM	NA
Kabuli_wild_45229	1399944786	*Ca_kabuli_scaffold*	45229	A/T	Gene_Ca_27001	UPSTREAM	NA
Kabuli_wild_45231	1399944787	*Ca_kabuli_scaffold*	45231	C/T	Gene_Ca_27001	UPSTREAM	NA
Kabuli_wild_45232	1399944788	*Ca_kabuli_scaffold*	45232	A/T	Gene_Ca_27001	UPSTREAM	NA
Kabuli_wild_45234	1399944789	*Ca_kabuli_scaffold*	45234	C/T	Gene_Ca_27001	UPSTREAM	NA
Kabuli_wild_45236	1399944790	*Ca_kabuli_scaffold*	45236	G/A	Gene_Ca_27001	UPSTREAM	NA
Kabuli_wild_45237	1399944791	*Ca_kabuli_scaffold*	45237	C/T	Gene_Ca_27001	UPSTREAM	NA
Kabuli_wild_45243	1399944792	*Ca_kabuli_scaffold*	45243	C/T	Gene_Ca_27001	UPSTREAM	NA
Kabuli_wild_45244	1399944793	*Ca_kabuli_scaffold*	45244	C/T	Gene_Ca_27001	UPSTREAM	NA
Kabuli_wild_45245	1399944794	*Ca_kabuli_scaffold*	45245	G/A	Gene_Ca_27001	UPSTREAM	NA
Kabuli_wild_45246	1399944795	*Ca_kabuli_scaffold*	45246	A/G	Gene_Ca_27001	UPSTREAM	NA
Kabuli_wild_45250	1399944796	*Ca_kabuli_scaffold*	45250	T/C	Gene_Ca_27001	UPSTREAM	NA
Kabuli_wild_45254	1399944797	*Ca_kabuli_scaffold*	45254	C/T	Gene_Ca_27001	UPSTREAM	NA
Kabuli_wild_45269	1399944798	*Ca_kabuli_scaffold*	45269	G/C	Gene_Ca_27001	UPSTREAM	NA
Kabuli_wild_45279	1399944799	*Ca_kabuli_scaffold*	45279	G/C	Gene_Ca_27001	UPSTREAM	NA
Kabuli_wild_45342	1399944800	*Ca_kabuli_scaffold*	45342	T/G	Gene_Ca_27001	UPSTREAM	NA
Kabuli_wild_80072	1399944801	*Ca_kabuli_scaffold*	80072	T/G	NA	INTERGENIC	NA
Kabuli_wild_99074	1399944802	*Ca_kabuli_scaffold*	99074	T/G	NA	INTERGENIC	NA
Kabuli_wild_102725	1399944803	*Ca_kabuli_scaffold*	102725	C/T	NA	INTERGENIC	NA
Kabuli_wild_108277	1399944804	*Ca_kabuli_scaffold*	108277	T/G	NA	INTERGENIC	NA
Kabuli_wild_110753	1399944805	*Ca_kabuli_scaffold*	110753	G/A	NA	INTERGENIC	NA
Kabuli_wild_110757	1399944806	*Ca_kabuli_scaffold*	110757	T/C	NA	INTERGENIC	NA
Kabuli_wild_110759	1399944807	*Ca_kabuli_scaffold*	110759	C/T	NA	INTERGENIC	NA
Kabuli_wild_110765	1399944808	*Ca_kabuli_scaffold*	110765	C/T	NA	INTERGENIC	NA
Kabuli_wild_110772	1399944809	*Ca_kabuli_scaffold*	110772	G/T	NA	INTERGENIC	NA
Kabuli_wild_110785	1399944810	*Ca_kabuli_scaffold*	110785	C/T	NA	INTERGENIC	NA
Kabuli_wild_117216	1399944811	*Ca_kabuli_scaffold*	117216	A/T	NA	INTERGENIC	NA
Kabuli_wild_117287	1399944812	*Ca_kabuli_scaffold*	117287	T/G	NA	INTERGENIC	NA
Kabuli_wild_53203	1399944813	*Ca_kabuli_scaffold*	53203	G/T	Gene_Ca_25252	INTRON	SAGA-associatedfactor29
Kabuli_wild_150215	1399944814	*Ca_kabuli_scaffold*	150215	G/A	NA	INTERGENIC	NA
Kabuli_wild_150237	1399944815	*Ca_kabuli_scaffold*	150237	C/T	NA	INTERGENIC	NA
Kabuli_wild_31802	1399944816	*Ca_kabuli_scaffold*	31802	A/G	Gene_Ca_25554	INTRON	Glycosidehydrolase,family1
Kabuli_wild_68413	1399944817	*Ca_kabuli_scaffold*	68413	C/T	Gene_Ca_25556	NON_SYNONYMOUS_CODING	Peptidase C48, SUMO/Sentrin/Ubl1
Kabuli_wild_68450	1399944818	*Ca_kabuli_scaffold*	68450	A/C	Gene_Ca_25556	SYNONYMOUS_CODING	Peptidase C48, SUMO/Sentrin/Ubl1
Kabuli_wild_68497	1399944819	*Ca_kabuli_scaffold*	68497	C/T	Gene_Ca_25556	NON_SYNONYMOUS_CODING	Peptidase C48, SUMO/Sentrin/Ubl1
Kabuli_wild_88420	1399944820	*Ca_kabuli_scaffold*	88420	T/C	Gene_Ca_25557	INTRON	WD40 repeat
Kabuli_wild_88429	1399944821	*Ca_kabuli_scaffold*	88429	A/T	Gene_Ca_25557	INTRON	WD40 repeat
Kabuli_wild_137196	1399944822	*Ca_kabuli_scaffold*	137196	G/T	Gene_Ca_21752	SYNONYMOUS_CODING	Patatin/PhospholipaseA2-related
Kabuli_wild_161754	1399944823	*Ca_kabuli_scaffold*	161754	T/C	NA	INTERGENIC	NA
Kabuli_wild_161854	1399944824	*Ca_kabuli_scaffold*	161854	C/T	Gene_Ca_21754	UPSTREAM	NA
Kabuli_wild_300825	1399944825	*Ca_kabuli_scaffold*	300825	A/C	Gene_Ca_21757	NON_SYNONYMOUS_CODING	F-boxdomain,cyclin-like
Kabuli_wild_300872	1399944826	*Ca_kabuli_scaffold*	300872	T/C	Gene_Ca_21757	SYNONYMOUS_CODING	F-boxdomain,cyclin-like
Kabuli_wild_300940	1399944827	*Ca_kabuli_scaffold*	300940	C/T	Gene_Ca_21757	SYNONYMOUS_CODING	F-boxdomain,cyclin-like

SNP IDs	NCBI dbSNP accession IDs (NCBI_SS#)	Chromosomes	Physical positions (bp)	SNPs	Gene accession IDs	Sequence components of genome	Putative functions
Kabuli_wild_404240	1399944828	*Ca_kabuli_scaffold*	404240	T/C	NA	INTERGENIC	NA
Kabuli_wild_404254	1399944829	*Ca_kabuli_scaffold*	404254	G/C	NA	INTERGENIC	NA
Kabuli_wild_404275	1399944830	*Ca_kabuli_scaffold*	404275	G/T	NA	INTERGENIC	NA
Kabuli_wild_404282	1399944831	*Ca_kabuli_scaffold*	404282	C/T	NA	INTERGENIC	NA
Kabuli_wild_404364	1399944832	*Ca_kabuli_scaffold*	404364	T/C	NA	INTERGENIC	NA
Kabuli_wild_441731	1399944833	*Ca_kabuli_scaffold*	441731	T/C	NA	INTERGENIC	NA
Kabuli_wild_510949	1399944834	*Ca_kabuli_scaffold*	510949	A/C	Gene_Ca_21770	INTRON	Folylpolyglutamatesynthetase
Kabuli_wild_294383	1399944835	*Ca_kabuli_scaffold*	294383	C/A	NA	INTERGENIC	NA
Kabuli_wild_294399	1399944836	*Ca_kabuli_scaffold*	294399	G/A	NA	INTERGENIC	NA
Kabuli_wild_294411	1399944837	*Ca_kabuli_scaffold*	294411	T/A	NA	INTERGENIC	NA
Kabuli_wild_294436	1399944838	*Ca_kabuli_scaffold*	294436	C/G	NA	INTERGENIC	NA
Kabuli_wild_294438	1399944839	*Ca_kabuli_scaffold*	294438	G/A	NA	INTERGENIC	NA
Kabuli_wild_294463	1399944840	*Ca_kabuli_scaffold*	294463	G/A	NA	INTERGENIC	NA
Kabuli_wild_294511	1399944841	*Ca_kabuli_scaffold*	294511	C/T	NA	INTERGENIC	NA
Kabuli_wild_294679	1399944842	*Ca_kabuli_scaffold*	294679	C/A	NA	INTERGENIC	NA
Kabuli_wild_303328	1399944843	*Ca_kabuli_scaffold*	303328	C/T	Gene_Ca_25344	SYNONYMOUS_CODING	Peptidase C48, SUMO/Sentrin/Ubl1
Kabuli_wild_349605	1399944844	*Ca_kabuli_scaffold*	349605	T/G	NA	INTERGENIC	NA
Kabuli_wild_363426	1399944845	*Ca_kabuli_scaffold*	363426	G/C	NA	INTERGENIC	NA
Kabuli_wild_34907	1399944846	*Ca_kabuli_scaffold*	34907	C/T	NA	INTERGENIC	NA
Kabuli_wild_55478	1399944847	*Ca_kabuli_scaffold*	55478	G/A	NA	INTERGENIC	NA
Kabuli_wild_55489	1399944848	*Ca_kabuli_scaffold*	55489	C/T	NA	INTERGENIC	NA
Kabuli_wild_55519	1399944849	*Ca_kabuli_scaffold*	55519	G/A	NA	INTERGENIC	NA
Kabuli_wild_55520	1399944850	*Ca_kabuli_scaffold*	55520	C/T	NA	INTERGENIC	NA
Kabuli_wild_55523	1399944851	*Ca_kabuli_scaffold*	55523	T/C	NA	INTERGENIC	NA
Kabuli_wild_55549	1399944852	*Ca_kabuli_scaffold*	55549	G/A	NA	INTERGENIC	NA
Kabuli_wild_71998	1399944853	*Ca_kabuli_scaffold*	71998	T/A	NA	INTERGENIC	NA
Kabuli_wild_110896	1399944854	*Ca_kabuli_scaffold*	110896	C/A	NA	INTERGENIC	NA
Kabuli_wild_110931	1399944855	*Ca_kabuli_scaffold*	110931	A/G	NA	INTERGENIC	NA
Kabuli_wild_14084	1399944856	*Ca_kabuli_scaffold*	14084	G/A	NA	INTERGENIC	NA
Kabuli_wild_14122	1399944857	*Ca_kabuli_scaffold*	14122	T/C	NA	INTERGENIC	NA
Kabuli_wild_37368	1399944858	*Ca_kabuli_scaffold*	37368	G/A	NA	INTERGENIC	NA
Kabuli_wild_37379	1399944859	*Ca_kabuli_scaffold*	37379	A/G	NA	INTERGENIC	NA
Kabuli_wild_37390	1399944860	*Ca_kabuli_scaffold*	37390	A/G	NA	INTERGENIC	NA
Kabuli_wild_37404	1399944861	*Ca_kabuli_scaffold*	37404	A/G	NA	INTERGENIC	NA
Kabuli_wild_37554	1399944862	*Ca_kabuli_scaffold*	37554	G/A	NA	INTERGENIC	NA
Kabuli_wild_37592	1399944863	*Ca_kabuli_scaffold*	37592	T/C	NA	INTERGENIC	NA
Kabuli_wild_23769	1399944864	*Ca_kabuli_scaffold*	23769	A/C	NA	INTERGENIC	NA
Kabuli_wild_23773	1399944865	*Ca_kabuli_scaffold*	23773	T/C	NA	INTERGENIC	NA
Kabuli_wild_23774	1399944866	*Ca_kabuli_scaffold*	23774	A/G	NA	INTERGENIC	NA
Kabuli_wild_23775	1399944867	*Ca_kabuli_scaffold*	23775	A/G	NA	INTERGENIC	NA
Kabuli_wild_23793	1399944868	*Ca_kabuli_scaffold*	23793	A/G	NA	INTERGENIC	NA
Kabuli_wild_23795	1399944869	*Ca_kabuli_scaffold*	23795	T/A	NA	INTERGENIC	NA
Kabuli_wild_23803	1399944870	*Ca_kabuli_scaffold*	23803	A/G	NA	INTERGENIC	NA
Kabuli_wild_23808	1399944871	*Ca_kabuli_scaffold*	23808	C/G	NA	INTERGENIC	NA

SNP IDs	NCBI dbSNP accession IDs (NCBI_SS#)	Chromosomes	Physical positions (bp)	SNPs	Gene accession IDs	Sequence components of genome	Putative functions
Kabuli_wild_23811	1399944872	*Ca_kabuli_scaffold*	23811	G/A	NA	INTERGENIC	NA
Kabuli_wild_23815	1399944873	*Ca_kabuli_scaffold*	23815	A/G	NA	INTERGENIC	NA
Kabuli_wild_23816	1399944874	*Ca_kabuli_scaffold*	23816	T/A	NA	INTERGENIC	NA
Kabuli_wild_23821	1399944875	*Ca_kabuli_scaffold*	23821	T/C	NA	INTERGENIC	NA
Kabuli_wild_23824	1399944876	*Ca_kabuli_scaffold*	23824	G/C	NA	INTERGENIC	NA
Kabuli_wild_23830	1399944877	*Ca_kabuli_scaffold*	23830	T/C	NA	INTERGENIC	NA
Kabuli_wild_23912	1399944878	*Ca_kabuli_scaffold*	23912	C/A	NA	INTERGENIC	NA
Kabuli_wild_23913	1399944879	*Ca_kabuli_scaffold*	23913	A/G	NA	INTERGENIC	NA
Kabuli_wild_23914	1399944880	*Ca_kabuli_scaffold*	23914	A/T	NA	INTERGENIC	NA
Kabuli_wild_23924	1399944881	*Ca_kabuli_scaffold*	23924	T/C	NA	INTERGENIC	NA
Kabuli_wild_23925	1399944882	*Ca_kabuli_scaffold*	23925	T/C	NA	INTERGENIC	NA
Kabuli_wild_23929	1399944883	*Ca_kabuli_scaffold*	23929	G/A	NA	INTERGENIC	NA
Kabuli_wild_84761	1399944884	*Ca_kabuli_scaffold*	84761	A/C	NA	INTERGENIC	NA
Kabuli_wild_84765	1399944885	*Ca_kabuli_scaffold*	84765	T/C	NA	INTERGENIC	NA
Kabuli_wild_84766	1399944886	*Ca_kabuli_scaffold*	84766	A/G	NA	INTERGENIC	NA
Kabuli_wild_84767	1399944887	*Ca_kabuli_scaffold*	84767	A/G	NA	INTERGENIC	NA
Kabuli_wild_84785	1399944888	*Ca_kabuli_scaffold*	84785	A/G	NA	INTERGENIC	NA
Kabuli_wild_84787	1399944889	*Ca_kabuli_scaffold*	84787	T/A	NA	INTERGENIC	NA
Kabuli_wild_84795	1399944890	*Ca_kabuli_scaffold*	84795	A/G	NA	INTERGENIC	NA
Kabuli_wild_84800	1399944891	*Ca_kabuli_scaffold*	84800	C/G	NA	INTERGENIC	NA
Kabuli_wild_84803	1399944892	*Ca_kabuli_scaffold*	84803	G/A	NA	INTERGENIC	NA
Kabuli_wild_84807	1399944893	*Ca_kabuli_scaffold*	84807	A/T	NA	INTERGENIC	NA
Kabuli_wild_84813	1399944894	*Ca_kabuli_scaffold*	84813	T/C	NA	INTERGENIC	NA
Kabuli_wild_84816	1399944895	*Ca_kabuli_scaffold*	84816	G/C	NA	INTERGENIC	NA
Kabuli_wild_84822	1399944896	*Ca_kabuli_scaffold*	84822	T/C	NA	INTERGENIC	NA
Kabuli_wild_84904	1399944897	*Ca_kabuli_scaffold*	84904	C/A	NA	INTERGENIC	NA
Kabuli_wild_84906	1399944898	*Ca_kabuli_scaffold*	84906	A/T	NA	INTERGENIC	NA
Kabuli_wild_84916	1399944899	*Ca_kabuli_scaffold*	84916	T/C	NA	INTERGENIC	NA
Kabuli_wild_84917	1399944900	*Ca_kabuli_scaffold*	84917	T/C	NA	INTERGENIC	NA
Kabuli_wild_84921	1399944901	*Ca_kabuli_scaffold*	84921	G/A	NA	INTERGENIC	NA
Kabuli_wild_84937	1399944902	*Ca_kabuli_scaffold*	84937	A/C	NA	INTERGENIC	NA
Kabuli_wild_84939	1399944903	*Ca_kabuli_scaffold*	84939	C/T	NA	INTERGENIC	NA
Kabuli_wild_62540	1399944904	*Ca_kabuli_scaffold*	62540	A/G	NA	INTERGENIC	NA
Kabuli_wild_15458	1399944905	*Ca_kabuli_scaffold*	15458	A/C	NA	INTERGENIC	NA
Kabuli_wild_15459	1399944906	*Ca_kabuli_scaffold*	15459	C/T	NA	INTERGENIC	NA
Kabuli_wild_15462	1399944907	*Ca_kabuli_scaffold*	15462	A/G	NA	INTERGENIC	NA
Kabuli_wild_15471	1399944908	*Ca_kabuli_scaffold*	15471	C/T	NA	INTERGENIC	NA
Kabuli_wild_15472	1399944909	*Ca_kabuli_scaffold*	15472	C/A	NA	INTERGENIC	NA
Kabuli_wild_15473	1399944910	*Ca_kabuli_scaffold*	15473	C/A	NA	INTERGENIC	NA
Kabuli_wild_15482	1399944911	*Ca_kabuli_scaffold*	15482	A/G	NA	INTERGENIC	NA
Kabuli_wild_15487	1399944912	*Ca_kabuli_scaffold*	15487	C/T	NA	INTERGENIC	NA
Kabuli_wild_15488	1399944913	*Ca_kabuli_scaffold*	15488	A/C	NA	INTERGENIC	NA
Kabuli_wild_15518	1399944914	*Ca_kabuli_scaffold*	15518	A/G	NA	INTERGENIC	NA
Kabuli_wild_15522	1399944915	*Ca_kabuli_scaffold*	15522	G/A	NA	INTERGENIC	NA

SNP IDs	NCBI dbSNP accession IDs (NCBI_SS#)	Chromosomes	Physical positions (bp)	SNPs	Gene accession IDs	Sequence components of genome	Putative functions
Kabuli_wild_254901	1399944916	*Ca_kabuli_scaffold*	254901	C/T	NA	INTERGENIC	NA
Kabuli_wild_254942	1399944917	*Ca_kabuli_scaffold*	254942	A/T	NA	INTERGENIC	NA
Kabuli_wild_254950	1399944918	*Ca_kabuli_scaffold*	254950	G/A	NA	INTERGENIC	NA
Kabuli_wild_254959	1399944919	*Ca_kabuli_scaffold*	254959	T/C	NA	INTERGENIC	NA
Kabuli_wild_254971	1399944920	*Ca_kabuli_scaffold*	254971	G/A	NA	INTERGENIC	NA
Kabuli_wild_254992	1399944921	*Ca_kabuli_scaffold*	254992	T/C	NA	INTERGENIC	NA
Kabuli_wild_373755	1399944922	*Ca_kabuli_scaffold*	373755	G/T	NA	INTERGENIC	NA
Kabuli_wild_25241	1399944923	*Ca_kabuli_scaffold*	25241	T/C	NA	INTERGENIC	NA
Kabuli_wild_31470	1399944924	*Ca_kabuli_scaffold*	31470	T/C	Gene_Ca_27338	NON_SYNONYMOUS_CODING	Oligo peptide transporterOPTsuperfamily
Kabuli_wild_107613	1399944925	*Ca_kabuli_scaffold*	107613	C/G	NA	INTERGENIC	NA
Kabuli_wild_107635	1399944926	*Ca_kabuli_scaffold*	107635	A/C	NA	INTERGENIC	NA
Kabuli_wild_107636	1399944927	*Ca_kabuli_scaffold*	107636	T/C	NA	INTERGENIC	NA
Kabuli_wild_107661	1399944928	*Ca_kabuli_scaffold*	107661	A/C	NA	INTERGENIC	NA
Kabuli_wild_107670	1399944929	*Ca_kabuli_scaffold*	107670	A/C	NA	INTERGENIC	NA
Kabuli_wild_28716	1399944930	*Ca_kabuli_scaffold*	28716	G/C	NA	INTERGENIC	NA
Kabuli_wild_29183	1399944931	*Ca_kabuli_scaffold*	29183	G/A	NA	INTERGENIC	NA
Kabuli_wild_29190	1399944932	*Ca_kabuli_scaffold*	29190	G/T	NA	INTERGENIC	NA
Kabuli_wild_29192	1399944933	*Ca_kabuli_scaffold*	29192	A/C	NA	INTERGENIC	NA
Kabuli_wild_46559	1399944934	*Ca_kabuli_scaffold*	46559	A/G	Gene_Ca_19086	INTRON	TetratricopeptideTPR-1
Kabuli_wild_187837	1399944935	*Ca_kabuli_scaffold*	187837	G/A	NA	INTERGENIC	NA
Kabuli_wild_288904	1399944936	*Ca_kabuli_scaffold*	288904	C/T	NA	INTERGENIC	NA
Kabuli_wild_91908	1399944937	*Ca_kabuli_scaffold*	91908	C/T	NA	INTERGENIC	NA
Kabuli_wild_193441	1399944938	*Ca_kabuli_scaffold*	193441	C/T	NA	INTERGENIC	NA
Kabuli_wild_244994	1399944939	*Ca_kabuli_scaffold*	244994	A/T	Gene_Ca_25158	INTRON	Phospholipid/glycerolacyltransferase
Kabuli_wild_245034	1399944940	*Ca_kabuli_scaffold*	245034	C/T	Gene_Ca_25158	INTRON	Phospholipid/glycerolacyltransferase
Kabuli_wild_245036	1399944941	*Ca_kabuli_scaffold*	245036	T/A	Gene_Ca_25158	INTRON	Phospholipid/glycerolacyltransferase
Kabuli_wild_145231	1399944942	*Ca_kabuli_scaffold*	145231	A/G	NA	INTERGENIC	NA
Kabuli_wild_183054	1399944943	*Ca_kabuli_scaffold*	183054	T/C	NA	INTERGENIC	NA
Kabuli_wild_183063	1399944944	*Ca_kabuli_scaffold*	183063	G/A	NA	INTERGENIC	NA
Kabuli_wild_183069	1399944945	*Ca_kabuli_scaffold*	183069	C/A	NA	INTERGENIC	NA
Kabuli_wild_183074	1399944946	*Ca_kabuli_scaffold*	183074	C/G	NA	INTERGENIC	NA
Kabuli_wild_183078	1399944947	*Ca_kabuli_scaffold*	183078	T/G	NA	INTERGENIC	NA
Kabuli_wild_183091	1399944948	*Ca_kabuli_scaffold*	183091	T/C	NA	INTERGENIC	NA
Kabuli_wild_183112	1399944949	*Ca_kabuli_scaffold*	183112	T/C	NA	INTERGENIC	NA
Kabuli_wild_183135	1399944950	*Ca_kabuli_scaffold*	183135	T/C	NA	INTERGENIC	NA
Kabuli_wild_183143	1399944951	*Ca_kabuli_scaffold*	183143	T/A	NA	INTERGENIC	NA
Kabuli_wild_189129	1399944952	*Ca_kabuli_scaffold*	189129	C/T	Gene_Ca_24313	SYNONYMOUS_CODING	Aminotransferase,classI/classII
Kabuli_wild_191730	1399944953	*Ca_kabuli_scaffold*	191730	A/C	Gene_Ca_24313	INTRON	Aminotransferase,classI/classII
Kabuli_wild_197878	1399944954	*Ca_kabuli_scaffold*	197878	A/T	Gene_Ca_24313	NON_SYNONYMOUS_CODING	Aminotransferase,classI/classII
Kabuli_wild_26813	1399944955	*Ca_kabuli_scaffold*	26813	A/G	NA	INTERGENIC	NA
Kabuli_wild_26828	1399944956	*Ca_kabuli_scaffold*	26828	A/G	NA	INTERGENIC	NA
Kabuli_wild_26845	1399944957	*Ca_kabuli_scaffold*	26845	C/T	NA	INTERGENIC	NA
Kabuli_wild_26849	1399944958	*Ca_kabuli_scaffold*	26849	T/A	NA	INTERGENIC	NA
Kabuli_wild_180302	1399944959	*Ca_kabuli_scaffold*	180302	G/T	Gene_Ca_23946	NON_SYNONYMOUS_CODING	Peptidase C48, SUMO/Sentrin/Ubl1

SNP IDs	NCBI dbSNP accession IDs (NCBI_SS#)	Chromosomes	Physical positions (bp)	SNPs	Gene accession IDs	Sequence components of genome	Putative functions
Kabuli_wild_180348	1399944960	*Ca_kabuli_scaffold*	180348	C/T	Gene_Ca_23946	NON_SYNONYMOUS_CODING	Peptidase C48, SUMO/Sentrin/Ubl1
Kabuli_wild_180362	1399944961	*Ca_kabuli_scaffold*	180362	A/C	Gene_Ca_23946	NON_SYNONYMOUS_CODING	Peptidase C48, SUMO/Sentrin/Ubl1
Kabuli_wild_180364	1399944962	*Ca_kabuli_scaffold*	180364	G/T	Gene_Ca_23946	NON_SYNONYMOUS_CODING	Peptidase C48, SUMO/Sentrin/Ubl1
Kabuli_wild_311182	1399944963	*Ca_kabuli_scaffold*	311182	C/T	NA	INTERGENIC	NA
Kabuli_wild_311222	1399944964	*Ca_kabuli_scaffold*	311222	G/T	NA	INTERGENIC	NA
Kabuli_wild_372816	1399944965	*Ca_kabuli_scaffold*	372816	G/A	NA	INTERGENIC	NA
Kabuli_wild_372845	1399944966	*Ca_kabuli_scaffold*	372845	T/G	NA	INTERGENIC	NA
Kabuli_wild_372851	1399944967	*Ca_kabuli_scaffold*	372851	T/C	NA	INTERGENIC	NA
Kabuli_wild_372860	1399944968	*Ca_kabuli_scaffold*	372860	G/T	NA	INTERGENIC	NA
Kabuli_wild_372862	1399944969	*Ca_kabuli_scaffold*	372862	G/A	NA	INTERGENIC	NA
Kabuli_wild_372869	1399944970	*Ca_kabuli_scaffold*	372869	G/T	NA	INTERGENIC	NA
Kabuli_wild_372874	1399944971	*Ca_kabuli_scaffold*	372874	G/A	NA	INTERGENIC	NA
Kabuli_wild_372875	1399944972	*Ca_kabuli_scaffold*	372875	T/G	NA	INTERGENIC	NA
Kabuli_wild_634514	1399944973	*Ca_kabuli_scaffold*	634514	C/T	NA	INTERGENIC	NA
Kabuli_wild_634515	1399944974	*Ca_kabuli_scaffold*	634515	T/C	NA	INTERGENIC	NA
Kabuli_wild_634517	1399944975	*Ca_kabuli_scaffold*	634517	G/A	NA	INTERGENIC	NA
Kabuli_wild_634605	1399944976	*Ca_kabuli_scaffold*	634605	G/A	NA	INTERGENIC	NA
Kabuli_wild_15998	1399944977	*Ca_kabuli_scaffold*	15998	C/G	NA	INTERGENIC	NA
Kabuli_wild_16029	1399944978	*Ca_kabuli_scaffold*	16029	G/A	NA	INTERGENIC	NA
Kabuli_wild_16042	1399944979	*Ca_kabuli_scaffold*	16042	C/A	NA	INTERGENIC	NA
Kabuli_wild_16056	1399944980	*Ca_kabuli_scaffold*	16056	C/T	NA	INTERGENIC	NA
Kabuli_wild_16061	1399944981	*Ca_kabuli_scaffold*	16061	A/G	NA	INTERGENIC	NA
Kabuli_wild_16068	1399944982	*Ca_kabuli_scaffold*	16068	G/A	NA	INTERGENIC	NA
Kabuli_wild_91684	1399944983	*Ca_kabuli_scaffold*	91684	G/A	Gene_Ca_24332	SYNONYMOUS_CODING	NA
Kabuli_wild_91704	1399944984	*Ca_kabuli_scaffold*	91704	G/C	Gene_Ca_24332	SYNONYMOUS_CODING	NA
Kabuli_wild_156613	1399944985	*Ca_kabuli_scaffold*	156613	G/C	NA	INTERGENIC	NA
Kabuli_wild_217402	1399944986	*Ca_kabuli_scaffold*	217402	C/T	Gene_Ca_27984	SYNONYMOUS_CODING	NA
Kabuli_wild_91979	1399944987	*Ca_kabuli_scaffold*	91979	T/A	NA	INTERGENIC	NA
Kabuli_wild_137032	1399944988	*Ca_kabuli_scaffold*	137032	A/C	NA	INTERGENIC	NA
Kabuli_wild_309725	1399944989	*Ca_kabuli_scaffold*	309725	C/T	NA	INTERGENIC	NA
Kabuli_wild_309797	1399944990	*Ca_kabuli_scaffold*	309797	C/A	NA	INTERGENIC	NA
Kabuli_wild_309818	1399944991	*Ca_kabuli_scaffold*	309818	T/C	NA	INTERGENIC	NA
Kabuli_wild_309865	1399944992	*Ca_kabuli_scaffold*	309865	G/A	NA	INTERGENIC	NA
Kabuli_wild_360343	1399944993	*Ca_kabuli_scaffold*	360343	T/C	NA	INTERGENIC	NA
Kabuli_wild_360349	1399944994	*Ca_kabuli_scaffold*	360349	G/A	NA	INTERGENIC	NA
Kabuli_wild_360366	1399944995	*Ca_kabuli_scaffold*	360366	C/T	NA	INTERGENIC	NA
Kabuli_wild_360385	1399944996	*Ca_kabuli_scaffold*	360385	G/T	NA	INTERGENIC	NA
Kabuli_wild_360388	1399944997	*Ca_kabuli_scaffold*	360388	C/T	NA	INTERGENIC	NA
Kabuli_wild_360400	1399944998	*Ca_kabuli_scaffold*	360400	C/T	NA	INTERGENIC	NA
Kabuli_wild_360406	1399944999	*Ca_kabuli_scaffold*	360406	C/T	NA	INTERGENIC	NA
Kabuli_wild_360408	1399945000	*Ca_kabuli_scaffold*	360408	G/T	NA	INTERGENIC	NA
Kabuli_wild_360411	1399945001	*Ca_kabuli_scaffold*	360411	G/A	NA	INTERGENIC	NA
Kabuli_wild_360413	1399945002	*Ca_kabuli_scaffold*	360413	G/A	NA	INTERGENIC	NA
Kabuli_wild_360416	1399945003	*Ca_kabuli_scaffold*	360416	C/T	NA	INTERGENIC	NA

SNP IDs	NCBI dbSNP accession IDs (NCBI_SS#)	Chromosomes	Physical positions (bp)	SNPs	Gene accession IDs	Sequence components of genome	Putative functions
Kabuli_wild_360421	1399945004	*Ca_kabuli_scaffold*	360421	G/A	NA	INTERGENIC	NA
Kabuli_wild_360461	1399945005	*Ca_kabuli_scaffold*	360461	G/A	NA	INTERGENIC	NA
Kabuli_wild_608623	1399945006	*Ca_kabuli_scaffold*	608623	T/G	Gene_Ca_20172	INTRON	Basic-leucine zipper (bZIP) Transcription factor
Kabuli_wild_652382	1399945007	*Ca_kabuli_scaffold*	652382	G/A	NA	INTERGENIC	NA
Kabuli_wild_826128	1399945008	*Ca_kabuli_scaffold*	826128	T/A	Gene_Ca_20181	DOWNSTREAM	Cys/Metmetabolism,pyridoxalphosphate-dependentenzyme
Kabuli_wild_826136	1399945009	*Ca_kabuli_scaffold*	826136	T/C	Gene_Ca_20181	DOWNSTREAM	Cys/Metmetabolism,pyridoxalphosphate-dependentenzyme
Kabuli_wild_826162	1399945010	*Ca_kabuli_scaffold*	826162	A/C	Gene_Ca_20181	DOWNSTREAM	Cys/Metmetabolism,pyridoxalphosphate-dependentenzyme
Kabuli_wild_827749	1399945011	*Ca_kabuli_scaffold*	827749	G/A	NA	INTERGENIC	NA
Kabuli_wild_827776	1399945012	*Ca_kabuli_scaffold*	827776	G/T	NA	INTERGENIC	NA
Kabuli_wild_18543	1399945013	*Ca_kabuli_scaffold*	18543	T/C	NA	INTERGENIC	NA
Kabuli_wild_157238	1399945014	*Ca_kabuli_scaffold*	157238	G/C	Gene_Ca_23185	NON_SYNONYMOUS_CODING	Transcriptional factor B3
Kabuli_wild_161097	1399945015	*Ca_kabuli_scaffold*	161097	A/G	NA	INTERGENIC	NA
Kabuli_wild_253689	1399945016	*Ca_kabuli_scaffold*	253689	T/C	NA	INTERGENIC	NA
Kabuli_wild_253716	1399945017	*Ca_kabuli_scaffold*	253716	T/G	NA	INTERGENIC	NA
Kabuli_wild_253719	1399945018	*Ca_kabuli_scaffold*	253719	G/A	NA	INTERGENIC	NA
Kabuli_wild_253723	1399945019	*Ca_kabuli_scaffold*	253723	C/T	NA	INTERGENIC	NA
Kabuli_wild_253748	1399945020	*Ca_kabuli_scaffold*	253748	A/G	Gene_Ca_23190	SYNONYMOUS_CODING	NA
Kabuli_wild_542066	1399945021	*Ca_kabuli_scaffold*	542066	T/C	NA	INTERGENIC	NA
Kabuli_wild_578200	1399945022	*Ca_kabuli_scaffold*	578200	T/G	Gene_Ca_23199	SYNONYMOUS_CODING	AMP-dependentsynthetase/ligase
Kabuli_wild_78842	1399945023	*Ca_kabuli_scaffold*	78842	G/A	Gene_Ca_26805	SYNONYMOUS_CODING	NA
Kabuli_wild_78844	1399945024	*Ca_kabuli_scaffold*	78844	A/G	Gene_Ca_26805	NON_SYNONYMOUS_CODING	NA
Kabuli_wild_78864	1399945025	*Ca_kabuli_scaffold*	78864	C/T	Gene_Ca_26805	STOP_GAINED	NA
Kabuli_wild_78871	1399945026	*Ca_kabuli_scaffold*	78871	C/G	Gene_Ca_26805	NON_SYNONYMOUS_CODING	NA
Kabuli_wild_78883	1399945027	*Ca_kabuli_scaffold*	78883	T/G	Gene_Ca_26805	NON_SYNONYMOUS_CODING	NA
Kabuli_wild_78893	1399945028	*Ca_kabuli_scaffold*	78893	C/T	Gene_Ca_26805	SYNONYMOUS_CODING	NA
Kabuli_wild_78894	1399945029	*Ca_kabuli_scaffold*	78894	G/A	Gene_Ca_26805	NON_SYNONYMOUS_CODING	NA
Kabuli_wild_163272	1399945030	*Ca_kabuli_scaffold*	163272	T/G	NA	INTERGENIC	NA
Kabuli_wild_206072	1399945031	*Ca_kabuli_scaffold*	206072	T/G	NA	INTERGENIC	NA
Kabuli_wild_206087	1399945032	*Ca_kabuli_scaffold*	206087	T/G	NA	INTERGENIC	NA
Kabuli_wild_206088	1399945033	*Ca_kabuli_scaffold*	206088	A/G	NA	INTERGENIC	NA
Kabuli_wild_206093	1399945034	*Ca_kabuli_scaffold*	206093	G/A	NA	INTERGENIC	NA
Kabuli_wild_257403	1399945035	*Ca_kabuli_scaffold*	257403	C/T	Gene_Ca_26826	SYNONYMOUS_CODING	NA
Kabuli_wild_257423	1399945036	*Ca_kabuli_scaffold*	257423	C/A	Gene_Ca_26826	SYNONYMOUS_CODING	NA
Kabuli_wild_257470	1399945037	*Ca_kabuli_scaffold*	257470	C/T	Gene_Ca_26826	NON_SYNONYMOUS_CODING	NA
Kabuli_wild_257542	1399945038	*Ca_kabuli_scaffold*	257542	G/A	Gene_Ca_26826	NON_SYNONYMOUS_CODING	NA
Kabuli_wild_247644	1399945039	*Ca_kabuli_scaffold*	247644	G/C	NA	INTERGENIC	NA
Kabuli_wild_2605	1399945040	*Ca_kabuli_scaffold*	2605	C/T	NA	INTERGENIC	NA
Kabuli_wild_2611	1399945041	*Ca_kabuli_scaffold*	2611	A/C	NA	INTERGENIC	NA
Kabuli_wild_2685	1399945042	*Ca_kabuli_scaffold*	2685	T/C	NA	INTERGENIC	NA
Kabuli_wild_27318	1399945043	*Ca_kabuli_scaffold*	27318	C/A	Gene_Ca_24287	INTRON	Porin,eukaryotictype
Kabuli_wild_27319	1399945044	*Ca_kabuli_scaffold*	27319	G/T	Gene_Ca_24287	INTRON	Porin,eukaryotictype
Kabuli_wild_153667	1399945045	*Ca_kabuli_scaffold*	153667	G/A	Gene_Ca_24295	NON_SYNONYMOUS_CODING	NA
Kabuli_wild_153674	1399945046	*Ca_kabuli_scaffold*	153674	T/C	Gene_Ca_24295	NON_SYNONYMOUS_CODING	NA
Kabuli_wild_153680	1399945047	*Ca_kabuli_scaffold*	153680	C/A	Gene_Ca_24295	NON_SYNONYMOUS_CODING	NA

SNP IDs	NCBI dbSNP accession IDs (NCBI_SS#)	Chromosomes	Physical positions (bp)	SNPs	Gene accession IDs	Sequence components of genome	Putative functions
Kabuli_wild_153694	1399945048	*Ca_kabuli_scaffold*	153694	C/T	Gene_Ca_24295	NON_SYNONYMOUS_CODING	NA
Kabuli_wild_153697	1399945049	*Ca_kabuli_scaffold*	153697	G/A	Gene_Ca_24295	NON_SYNONYMOUS_CODING	NA
Kabuli_wild_153699	1399945050	*Ca_kabuli_scaffold*	153699	A/G	Gene_Ca_24295	SYNONYMOUS_CODING	NA
Kabuli_wild_153706	1399945051	*Ca_kabuli_scaffold*	153706	G/A	Gene_Ca_24295	NON_SYNONYMOUS_CODING	NA
Kabuli_wild_90139	1399945052	*Ca_kabuli_scaffold*	90139	T/C	NA	INTERGENIC	NA
Kabuli_wild_212326	1399945053	*Ca_kabuli_scaffold*	212326	G/A	NA	INTERGENIC	NA
Kabuli_wild_212393	1399945054	*Ca_kabuli_scaffold*	212393	G/A	NA	INTERGENIC	NA
Kabuli_wild_212410	1399945055	*Ca_kabuli_scaffold*	212410	G/T	NA	INTERGENIC	NA
Kabuli_wild_269230	1399945056	*Ca_kabuli_scaffold*	269230	A/C	NA	INTERGENIC	NA
Kabuli_wild_269242	1399945057	*Ca_kabuli_scaffold*	269242	A/G	NA	INTERGENIC	NA
Kabuli_wild_269261	1399945058	*Ca_kabuli_scaffold*	269261	T/A	NA	INTERGENIC	NA
Kabuli_wild_269266	1399945059	*Ca_kabuli_scaffold*	269266	G/T	NA	INTERGENIC	NA
Kabuli_wild_269281	1399945060	*Ca_kabuli_scaffold*	269281	T/G	NA	INTERGENIC	NA
Kabuli_wild_269299	1399945061	*Ca_kabuli_scaffold*	269299	T/C	NA	INTERGENIC	NA
Kabuli_wild_269308	1399945062	*Ca_kabuli_scaffold*	269308	T/C	NA	INTERGENIC	NA
Kabuli_wild_269314	1399945063	*Ca_kabuli_scaffold*	269314	G/A	NA	INTERGENIC	NA
Kabuli_wild_269332	1399945064	*Ca_kabuli_scaffold*	269332	C/T	NA	INTERGENIC	NA
Kabuli_wild_269335	1399945065	*Ca_kabuli_scaffold*	269335	G/A	NA	INTERGENIC	NA
Kabuli_wild_295417	1399945066	*Ca_kabuli_scaffold*	295417	C/T	Gene_Ca_24971	DOWNSTREAM	NA
Kabuli_wild_11511	1399945067	*Ca_kabuli_scaffold*	11511	T/C	NA	INTERGENIC	NA
Kabuli_wild_11850	1399945068	*Ca_kabuli_scaffold*	11850	T/C	NA	INTERGENIC	NA
Kabuli_wild_60880	1399945069	*Ca_kabuli_scaffold*	60880	G/A	NA	INTERGENIC	NA
Kabuli_wild_60887	1399945070	*Ca_kabuli_scaffold*	60887	C/T	NA	INTERGENIC	NA
Kabuli_wild_60898	1399945071	*Ca_kabuli_scaffold*	60898	C/T	NA	INTERGENIC	NA
Kabuli_wild_60928	1399945072	*Ca_kabuli_scaffold*	60928	G/A	NA	INTERGENIC	NA
Kabuli_wild_130103	1399945073	*Ca_kabuli_scaffold*	130103	C/T	NA	INTERGENIC	NA
Kabuli_wild_179070	1399945074	*Ca_kabuli_scaffold*	179070	G/C	NA	INTERGENIC	NA
Kabuli_wild_197212	1399945075	*Ca_kabuli_scaffold*	197212	A/C	Gene_Ca_24737	UPSTREAM	NA
Kabuli_wild_370639	1399945076	*Ca_kabuli_scaffold*	370639	T/A	NA	INTERGENIC	NA
Kabuli_wild_370725	1399945077	*Ca_kabuli_scaffold*	370725	G/A	NA	INTERGENIC	NA
Kabuli_wild_25019	1399945078	*Ca_kabuli_scaffold*	25019	G/A	Gene_Ca_25043	DOWNSTREAM	NA
Kabuli_wild_25722	1399945079	*Ca_kabuli_scaffold*	25722	G/C	NA	INTERGENIC	NA
Kabuli_wild_13360	1399945080	*Ca_kabuli_scaffold*	13360	G/A	NA	INTERGENIC	NA
Kabuli_wild_315093	1399945081	*Ca_kabuli_scaffold*	315093	A/G	Gene_Ca_23815	UPSTREAM	Photosystemantennaprotein-like
Kabuli_wild_315114	1399945082	*Ca_kabuli_scaffold*	315114	T/C	Gene_Ca_23815	UPSTREAM	Photosystemantennaprotein-like
Kabuli_wild_315135	1399945083	*Ca_kabuli_scaffold*	315135	T/G	Gene_Ca_23815	UPSTREAM	Photosystemantennaprotein-like
Kabuli_wild_315138	1399945084	*Ca_kabuli_scaffold*	315138	T/C	Gene_Ca_23815	UPSTREAM	Photosystemantennaprotein-like
Kabuli_wild_315141	1399945085	*Ca_kabuli_scaffold*	315141	G/A	Gene_Ca_23815	UPSTREAM	Photosystemantennaprotein-like
Kabuli_wild_317199	1399945086	*Ca_kabuli_scaffold*	317199	A/T	Gene_Ca_23814	DOWNSTREAM	RibosomalproteinS11
Kabuli_wild_317202	1399945087	*Ca_kabuli_scaffold*	317202	T/A	Gene_Ca_23814	DOWNSTREAM	RibosomalproteinS11
Kabuli_wild_332378	1399945088	*Ca_kabuli_scaffold*	332378	A/C	NA	INTERGENIC	NA
Kabuli_wild_332413	1399945089	*Ca_kabuli_scaffold*	332413	T/C	Gene_Ca_23816	SYNONYMOUS_CODING	RibosomalproteinS7
Kabuli_wild_332458	1399945090	*Ca_kabuli_scaffold*	332458	A/T	Gene_Ca_23816	NON_SYNONYMOUS_CODING	RibosomalproteinS7
Kabuli_wild_332464	1399945091	*Ca_kabuli_scaffold*	332464	G/T	Gene_Ca_23816	SYNONYMOUS_CODING	RibosomalproteinS7

SNP IDs	NCBI dbSNP accession IDs (NCBI_SS#)	Chromosomes	Physical positions (bp)	SNPs	Gene accession IDs	Sequence components of genome	Putative functions
Kabuli_wild_332465	1399945092	*Ca_kabuli_scaffold*	332465	T/A	Gene_Ca_23816	NON_SYNONYMOUS_CODING	RibosomalproteinS7
Kabuli_wild_332467	1399945093	*Ca_kabuli_scaffold*	332467	G/C	Gene_Ca_23816	SYNONYMOUS_CODING	RibosomalproteinS7
Kabuli_wild_332497	1399945094	*Ca_kabuli_scaffold*	332497	G/A	Gene_Ca_23816	SYNONYMOUS_CODING	RibosomalproteinS7
Kabuli_wild_357635	1399945095	*Ca_kabuli_scaffold*	357635	A/T	NA	INTERGENIC	NA
Kabuli_wild_357641	1399945096	*Ca_kabuli_scaffold*	357641	G/T	NA	INTERGENIC	NA
Kabuli_wild_357642	1399945097	*Ca_kabuli_scaffold*	357642	T/A	NA	INTERGENIC	NA
Kabuli_wild_357644	1399945098	*Ca_kabuli_scaffold*	357644	G/C	NA	INTERGENIC	NA
Kabuli_wild_357645	1399945099	*Ca_kabuli_scaffold*	357645	G/T	NA	INTERGENIC	NA
Kabuli_wild_357674	1399945100	*Ca_kabuli_scaffold*	357674	G/A	NA	INTERGENIC	NA
Kabuli_wild_359724	1399945101	*Ca_kabuli_scaffold*	359724	T/C	Gene_Ca_23817	UPSTREAM	RibosomalproteinS12,bacteria
Kabuli_wild_362008	1399945102	*Ca_kabuli_scaffold*	362008	A/G	NA	INTERGENIC	NA
Kabuli_wild_362059	1399945103	*Ca_kabuli_scaffold*	362059	G/T	NA	INTERGENIC	NA
Kabuli_wild_362065	1399945104	*Ca_kabuli_scaffold*	362065	C/A	NA	INTERGENIC	NA
Kabuli_wild_362114	1399945105	*Ca_kabuli_scaffold*	362114	A/C	NA	INTERGENIC	NA
Kabuli_wild_362118	1399945106	*Ca_kabuli_scaffold*	362118	C/A	NA	INTERGENIC	NA
Kabuli_wild_362123	1399945107	*Ca_kabuli_scaffold*	362123	G/A	NA	INTERGENIC	NA
Kabuli_wild_362547	1399945108	*Ca_kabuli_scaffold*	362547	A/G	NA	INTERGENIC	NA
Kabuli_wild_362549	1399945109	*Ca_kabuli_scaffold*	362549	T/G	NA	INTERGENIC	NA
Kabuli_wild_362553	1399945110	*Ca_kabuli_scaffold*	362553	T/C	NA	INTERGENIC	NA
Kabuli_wild_362554	1399945111	*Ca_kabuli_scaffold*	362554	T/A	NA	INTERGENIC	NA
Kabuli_wild_362555	1399945112	*Ca_kabuli_scaffold*	362555	T/G	NA	INTERGENIC	NA
Kabuli_wild_362556	1399945113	*Ca_kabuli_scaffold*	362556	C/A	NA	INTERGENIC	NA
Kabuli_wild_362574	1399945114	*Ca_kabuli_scaffold*	362574	T/C	NA	INTERGENIC	NA
Kabuli_wild_362581	1399945115	*Ca_kabuli_scaffold*	362581	A/G	NA	INTERGENIC	NA
Kabuli_wild_362582	1399945116	*Ca_kabuli_scaffold*	362582	T/C	NA	INTERGENIC	NA
Kabuli_wild_362594	1399945117	*Ca_kabuli_scaffold*	362594	G/T	NA	INTERGENIC	NA
Kabuli_wild_362607	1399945118	*Ca_kabuli_scaffold*	362607	G/A	NA	INTERGENIC	NA
Kabuli_wild_362610	1399945119	*Ca_kabuli_scaffold*	362610	C/T	NA	INTERGENIC	NA
Kabuli_wild_362625	1399945120	*Ca_kabuli_scaffold*	362625	G/T	NA	INTERGENIC	NA
Kabuli_wild_362626	1399945121	*Ca_kabuli_scaffold*	362626	C/G	NA	INTERGENIC	NA
Kabuli_wild_362794	1399945122	*Ca_kabuli_scaffold*	362794	C/T	NA	INTERGENIC	NA
Kabuli_wild_362796	1399945123	*Ca_kabuli_scaffold*	362796	C/T	NA	INTERGENIC	NA
Kabuli_wild_362814	1399945124	*Ca_kabuli_scaffold*	362814	C/T	NA	INTERGENIC	NA
Kabuli_wild_372183	1399945125	*Ca_kabuli_scaffold*	372183	C/T	NA	INTERGENIC	NA
Kabuli_wild_372186	1399945126	*Ca_kabuli_scaffold*	372186	A/C	NA	INTERGENIC	NA
Kabuli_wild_372188	1399945127	*Ca_kabuli_scaffold*	372188	C/G	NA	INTERGENIC	NA
Kabuli_wild_372209	1399945128	*Ca_kabuli_scaffold*	372209	G/C	NA	INTERGENIC	NA
Kabuli_wild_372215	1399945129	*Ca_kabuli_scaffold*	372215	A/G	NA	INTERGENIC	NA
Kabuli_wild_372243	1399945130	*Ca_kabuli_scaffold*	372243	A/T	NA	INTERGENIC	NA
Kabuli_wild_372244	1399945131	*Ca_kabuli_scaffold*	372244	C/T	NA	INTERGENIC	NA
Kabuli_wild_372250	1399945132	*Ca_kabuli_scaffold*	372250	G/A	NA	INTERGENIC	NA
Kabuli_wild_372253	1399945133	*Ca_kabuli_scaffold*	372253	G/T	NA	INTERGENIC	NA
Kabuli_wild_372255	1399945134	*Ca_kabuli_scaffold*	372255	G/A	NA	INTERGENIC	NA
Kabuli_wild_372959	1399945135	*Ca_kabuli_scaffold*	372959	A/C	NA	INTERGENIC	NA

SNP IDs	NCBI dbSNP accession IDs (NCBI_SS#)	Chromosomes	Physical positions (bp)	SNPs	Gene accession IDs	Sequence components of genome	Putative functions
Kabuli_wild_372970	1399945136	*Ca_kabuli_scaffold*	372970	T/A	NA	INTERGENIC	NA
Kabuli_wild_373019	1399945137	*Ca_kabuli_scaffold*	373019	A/T	NA	INTERGENIC	NA
Kabuli_wild_373022	1399945138	*Ca_kabuli_scaffold*	373022	A/T	NA	INTERGENIC	NA
Kabuli_wild_6755	1399945139	*Ca_kabuli_scaffold*	6755	G/A	Gene_Ca_25786	SYNONYMOUS_CODING	NA
Kabuli_wild_6770	1399945140	*Ca_kabuli_scaffold*	6770	C/T	Gene_Ca_25786	NON_SYNONYMOUS_CODING	NA
Kabuli_wild_187484	1399945141	*Ca_kabuli_scaffold*	187484	C/G	NA	INTERGENIC	NA
Kabuli_wild_12589	1399945142	*Ca_kabuli_scaffold*	12589	G/A	NA	INTERGENIC	NA
Kabuli_wild_339689	1399945143	*Ca_kabuli_scaffold*	339689	A/G	NA	INTERGENIC	NA
Kabuli_wild_339701	1399945144	*Ca_kabuli_scaffold*	339701	A/C	NA	INTERGENIC	NA
Kabuli_wild_359448	1399945145	*Ca_kabuli_scaffold*	359448	G/A	Gene_Ca_25944	NON_SYNONYMOUS_CODING	NA
Kabuli_wild_359465	1399945146	*Ca_kabuli_scaffold*	359465	T/A	Gene_Ca_25944	SYNONYMOUS_CODING	NA
Kabuli_wild_2860	1399945147	*Ca_kabuli_scaffold*	2860	G/T	NA	INTERGENIC	NA
Kabuli_wild_152249	1399945148	*Ca_kabuli_scaffold*	152249	T/A	NA	INTERGENIC	NA
Kabuli_wild_152281	1399945149	*Ca_kabuli_scaffold*	152281	C/A	NA	INTERGENIC	NA
Kabuli_wild_382375	1399945150	*Ca_kabuli_scaffold*	382375	G/A	Gene_Ca_25235	INTRON	Dual specificity phosphatase, catalytic domain
Kabuli_wild_478695	1399945151	*Ca_kabuli_scaffold*	478695	A/G	Gene_Ca_25238	INTRON	XerodermapigmentosumgroupGprotein
Kabuli_wild_497428	1399945152	*Ca_kabuli_scaffold*	497428	T/A	NA	INTERGENIC	NA
Kabuli_wild_497452	1399945153	*Ca_kabuli_scaffold*	497452	T/A	NA	INTERGENIC	NA
Kabuli_wild_497454	1399945154	*Ca_kabuli_scaffold*	497454	A/G	NA	INTERGENIC	NA
Kabuli_wild_7614	1399945155	*Ca_kabuli_scaffold*	7614	G/A	Gene_Ca_25929	NON_SYNONYMOUS_CODING	Zinc finger,Dof-type
Kabuli_wild_278864	1399945156	*Ca_kabuli_scaffold*	278864	C/T	NA	INTERGENIC	NA
Kabuli_wild_278914	1399945157	*Ca_kabuli_scaffold*	278914	C/G	NA	INTERGENIC	NA
Kabuli_wild_16480	1399945158	*Ca_kabuli_scaffold*	16480	G/T	NA	INTERGENIC	NA
Kabuli_wild_201291	1399945159	*Ca_kabuli_scaffold*	201291	T/G	NA	INTERGENIC	NA
Kabuli_wild_201293	1399945160	*Ca_kabuli_scaffold*	201293	C/T	NA	INTERGENIC	NA
Kabuli_wild_201326	1399945161	*Ca_kabuli_scaffold*	201326	C/T	NA	INTERGENIC	NA
Kabuli_wild_201365	1399945162	*Ca_kabuli_scaffold*	201365	G/A	NA	INTERGENIC	NA
Kabuli_wild_85264	1399945163	*Ca_kabuli_scaffold*	85264	A/T	NA	INTERGENIC	NA
Kabuli_wild_85318	1399945164	*Ca_kabuli_scaffold*	85318	T/C	NA	INTERGENIC	NA
Kabuli_wild_53157	1399945165	*Ca_kabuli_scaffold*	53157	T/C	NA	INTERGENIC	NA
Kabuli_wild_53192	1399945166	*Ca_kabuli_scaffold*	53192	C/T	NA	INTERGENIC	NA
Kabuli_wild_53196	1399945167	*Ca_kabuli_scaffold*	53196	C/T	NA	INTERGENIC	NA
Kabuli_wild_53229	1399945168	*Ca_kabuli_scaffold*	53229	A/G	NA	INTERGENIC	NA
Kabuli_wild_89238	1399945169	*Ca_kabuli_scaffold*	89238	T/C	NA	INTERGENIC	NA
Kabuli_wild_89239	1399945170	*Ca_kabuli_scaffold*	89239	C/T	NA	INTERGENIC	NA
Kabuli_wild_89248	1399945171	*Ca_kabuli_scaffold*	89248	C/T	NA	INTERGENIC	NA
Kabuli_wild_218012	1399945172	*Ca_kabuli_scaffold*	218012	A/G	NA	INTERGENIC	NA
Kabuli_wild_265215	1399945173	*Ca_kabuli_scaffold*	265215	A/C	NA	INTERGENIC	NA
Kabuli_wild_265226	1399945174	*Ca_kabuli_scaffold*	265226	T/C	NA	INTERGENIC	NA
Kabuli_wild_265251	1399945175	*Ca_kabuli_scaffold*	265251	C/T	NA	INTERGENIC	NA
Kabuli_wild_356107	1399945176	*Ca_kabuli_scaffold*	356107	C/A	NA	INTERGENIC	NA
Kabuli_wild_448604	1399945177	*Ca_kabuli_scaffold*	448604	G/A	NA	INTERGENIC	NA
Kabuli_wild_448743	1399945178	*Ca_kabuli_scaffold*	448743	T/C	NA	INTERGENIC	NA
Kabuli_wild_57173	1399945179	*Ca_kabuli_scaffold*	57173	C/T	NA	INTERGENIC	NA

SNP IDs	NCBI dbSNP accession IDs (NCBI_SS#)	Chromosomes	Physical positions (bp)	SNPs	Gene accession IDs	Sequence components of genome	Putative functions
Kabuli_wild_159740	1399945180	*Ca_kabuli_scaffold*	159740	T/C	NA	INTERGENIC	NA
Kabuli_wild_188888	1399945181	*Ca_kabuli_scaffold*	188888	G/A	Gene_Ca_25442	UPSTREAM	NA
Kabuli_wild_188894	1399945182	*Ca_kabuli_scaffold*	188894	A/G	Gene_Ca_25442	UPSTREAM	NA
Kabuli_wild_188925	1399945183	*Ca_kabuli_scaffold*	188925	C/T	Gene_Ca_25442	UPSTREAM	NA
Kabuli_wild_188955	1399945184	*Ca_kabuli_scaffold*	188955	G/A	Gene_Ca_25442	UPSTREAM	NA
Kabuli_wild_188973	1399945185	*Ca_kabuli_scaffold*	188973	C/T	Gene_Ca_25442	UPSTREAM	NA
Kabuli_wild_188981	1399945186	*Ca_kabuli_scaffold*	188981	A/T	Gene_Ca_25442	UPSTREAM	NA
Kabuli_wild_102989	1399945187	*Ca_kabuli_scaffold*	102989	A/G	NA	INTERGENIC	NA
Kabuli_wild_102998	1399945188	*Ca_kabuli_scaffold*	102998	A/G	NA	INTERGENIC	NA
Kabuli_wild_103001	1399945189	*Ca_kabuli_scaffold*	103001	A/G	NA	INTERGENIC	NA
Kabuli_wild_103040	1399945190	*Ca_kabuli_scaffold*	103040	A/G	NA	INTERGENIC	NA
Kabuli_wild_103050	1399945191	*Ca_kabuli_scaffold*	103050	C/T	NA	INTERGENIC	NA
Kabuli_wild_201617	1399945192	*Ca_kabuli_scaffold*	201617	T/C	NA	INTERGENIC	NA
Kabuli_wild_244209	1399945193	*Ca_kabuli_scaffold*	244209	T/C	NA	INTERGENIC	NA
Kabuli_wild_4363	1399945194	*Ca_kabuli_scaffold*	4363	A/G	NA	INTERGENIC	NA
Kabuli_wild_33106	1399945195	*Ca_kabuli_scaffold*	33106	A/T	NA	INTERGENIC	NA
Kabuli_wild_33108	1399945196	*Ca_kabuli_scaffold*	33108	T/G	NA	INTERGENIC	NA
Kabuli_wild_336860	1399945197	*Ca_kabuli_scaffold*	336860	C/A	NA	INTERGENIC	NA
Kabuli_wild_336894	1399945198	*Ca_kabuli_scaffold*	336894	T/C	NA	INTERGENIC	NA
Kabuli_wild_360756	1399945199	*Ca_kabuli_scaffold*	360756	T/C	NA	INTERGENIC	NA
Kabuli_wild_360762	1399945200	*Ca_kabuli_scaffold*	360762	C/A	NA	INTERGENIC	NA
Kabuli_wild_366195	1399945201	*Ca_kabuli_scaffold*	366195	T/C	NA	INTERGENIC	NA
Kabuli_wild_52238	1399945202	*Ca_kabuli_scaffold*	52238	A/C	NA	INTERGENIC	NA
Kabuli_wild_27214	1399945203	*Ca_kabuli_scaffold*	27214	G/A	NA	INTERGENIC	NA
Kabuli_wild_27274	1399945204	*Ca_kabuli_scaffold*	27274	A/G	NA	INTERGENIC	NA
Kabuli_wild_27285	1399945205	*Ca_kabuli_scaffold*	27285	T/C	NA	INTERGENIC	NA
Kabuli_wild_50239	1399945206	*Ca_kabuli_scaffold*	50239	G/A	NA	INTERGENIC	NA
Kabuli_wild_50240	1399945207	*Ca_kabuli_scaffold*	50240	C/A	NA	INTERGENIC	NA
Kabuli_wild_3930	1399945208	*Ca_kabuli_scaffold*	3930	A/G	Gene_Ca_27768	DOWNSTREAM	Domain of unknown function DUF828
Kabuli_wild_3933	1399945209	*Ca_kabuli_scaffold*	3933	A/G	Gene_Ca_27768	DOWNSTREAM	Domain of unknown function DUF828
Kabuli_wild_3955	1399945210	*Ca_kabuli_scaffold*	3955	G/A	Gene_Ca_27768	DOWNSTREAM	Domain of unknown function DUF828
Kabuli_wild_3970	1399945211	*Ca_kabuli_scaffold*	3970	C/A	Gene_Ca_27768	DOWNSTREAM	Domain of unknown function DUF828
Kabuli_wild_292	1399945212	*Ca_kabuli_scaffold*	292	A/G	NA	INTERGENIC	NA
Kabuli_wild_310	1399945213	*Ca_kabuli_scaffold*	310	A/C	NA	INTERGENIC	NA
Kabuli_wild_340	1399945214	*Ca_kabuli_scaffold*	340	T/C	NA	INTERGENIC	NA
Kabuli_wild_67115	1399945215	*Ca_kabuli_scaffold*	67115	C/T	Gene_Ca_26176	INTRON	PeptidaseS10,serinecarboxypeptidase
Kabuli_wild_16700	1399945216	*Ca_kabuli_scaffold*	16700	A/C	NA	INTERGENIC	NA
Kabuli_wild_129263	1399945217	*Ca_kabuli_scaffold*	129263	A/G	NA	INTERGENIC	NA
Kabuli_wild_129265	1399945218	*Ca_kabuli_scaffold*	129265	G/T	NA	INTERGENIC	NA
Kabuli_wild_129350	1399945219	*Ca_kabuli_scaffold*	129350	C/G	NA	INTERGENIC	NA
Kabuli_wild_48650	1399945220	*Ca_kabuli_scaffold*	48650	C/A	NA	INTERGENIC	NA
Kabuli_wild_48678	1399945221	*Ca_kabuli_scaffold*	48678	T/C	NA	INTERGENIC	NA
Kabuli_wild_53431	1399945222	*Ca_kabuli_scaffold*	53431	G/A	NA	INTERGENIC	NA
Kabuli_wild_53443	1399945223	*Ca_kabuli_scaffold*	53443	T/G	NA	INTERGENIC	NA

SNP IDs	NCBI dbSNP accession IDs (NCBI_SS#)	Chromosomes	Physical positions (bp)	SNPs	Gene accession IDs	Sequence components of genome	Putative functions
Kabuli_wild_53444	1399945224	*Ca_kabuli_scaffold*	53444	G/A	NA	INTERGENIC	NA
Kabuli_wild_53450	1399945225	*Ca_kabuli_scaffold*	53450	T/C	NA	INTERGENIC	NA
Kabuli_wild_53464	1399945226	*Ca_kabuli_scaffold*	53464	G/A	NA	INTERGENIC	NA
Kabuli_wild_53465	1399945227	*Ca_kabuli_scaffold*	53465	T/C	NA	INTERGENIC	NA
Kabuli_wild_53470	1399945228	*Ca_kabuli_scaffold*	53470	C/G	NA	INTERGENIC	NA
Kabuli_wild_53474	1399945229	*Ca_kabuli_scaffold*	53474	G/A	NA	INTERGENIC	NA
Kabuli_wild_104946	1399945230	*Ca_kabuli_scaffold*	104946	T/C	Gene_Ca_26625	INTRON	DNA mismatch repair protein MutS,C-terminal domain
Kabuli_wild_104963	1399945231	*Ca_kabuli_scaffold*	104963	T/A	Gene_Ca_26625	INTRON	DNA mismatch repair protein MutS,C-terminal domain
Kabuli_wild_81373	1399945232	*Ca_kabuli_scaffold*	81373	C/T	NA	INTERGENIC	NA
Kabuli_wild_34686	1399945233	*Ca_kabuli_scaffold*	34686	C/T	NA	INTERGENIC	NA
Kabuli_wild_41667	1399945234	*Ca_kabuli_scaffold*	41667	A/C	NA	INTERGENIC	NA
Kabuli_wild_41705	1399945235	*Ca_kabuli_scaffold*	41705	A/G	NA	INTERGENIC	NA
Kabuli_wild_62134	1399945236	*Ca_kabuli_scaffold*	62134	A/C	Gene_Ca_23368	INTRON	NA
Kabuli_wild_62141	1399945237	*Ca_kabuli_scaffold*	62141	C/T	Gene_Ca_23368	INTRON	NA
Kabuli_wild_62148	1399945238	*Ca_kabuli_scaffold*	62148	T/A	Gene_Ca_23368	INTRON	NA
Kabuli_wild_62166	1399945239	*Ca_kabuli_scaffold*	62166	A/T	Gene_Ca_23368	INTRON	NA
Kabuli_wild_168592	1399945240	*Ca_kabuli_scaffold*	168592	G/A	Gene_Ca_23370	INTRON	Zinc finger,FYVE-type
Kabuli_wild_246198	1399945241	*Ca_kabuli_scaffold*	246198	A/C	NA	INTERGENIC	NA
Kabuli_wild_246208	1399945242	*Ca_kabuli_scaffold*	246208	A/C	NA	INTERGENIC	NA
Kabuli_wild_246288	1399945243	*Ca_kabuli_scaffold*	246288	C/T	NA	INTERGENIC	NA
Kabuli_wild_246556	1399945244	*Ca_kabuli_scaffold*	246556	T/C	Gene_Ca_23373	SYNONYMOUS_CODING	NA
Kabuli_wild_528815	1399945245	*Ca_kabuli_scaffold*	528815	C/G	NA	INTERGENIC	NA
Kabuli_wild_29089	1399945246	*Ca_kabuli_scaffold*	29089	C/T	NA	INTERGENIC	NA
Kabuli_wild_29105	1399945247	*Ca_kabuli_scaffold*	29105	C/T	NA	INTERGENIC	NA
Kabuli_wild_29145	1399945248	*Ca_kabuli_scaffold*	29145	C/T	NA	INTERGENIC	NA
Kabuli_wild_13689	1399945249	*Ca_kabuli_scaffold*	13689	T/A	NA	INTERGENIC	NA
Kabuli_wild_13707	1399945250	*Ca_kabuli_scaffold*	13707	G/A	NA	INTERGENIC	NA
Kabuli_wild_8131	1399945251	*Ca_kabuli_scaffold*	8131	C/T	NA	INTERGENIC	NA
Kabuli_wild_13898	1399945252	*Ca_kabuli_scaffold*	13898	T/C	NA	INTERGENIC	NA
Kabuli_wild_13928	1399945253	*Ca_kabuli_scaffold*	13928	G/T	NA	INTERGENIC	NA
Kabuli_wild_13946	1399945254	*Ca_kabuli_scaffold*	13946	T/C	NA	INTERGENIC	NA
Kabuli_wild_28172	1399945255	*Ca_kabuli_scaffold*	28172	G/T	NA	INTERGENIC	NA
Kabuli_wild_44876	1399945256	*Ca_kabuli_scaffold*	44876	G/A	Gene_Ca_22411	SYNONYMOUS_CODING	Cation efflux protein
Kabuli_wild_45557	1399945257	*Ca_kabuli_scaffold*	45557	A/G	Gene_Ca_22411	SYNONYMOUS_CODING	Cation efflux protein
Kabuli_wild_71041	1399945258	*Ca_kabuli_scaffold*	71041	A/G	NA	INTERGENIC	NA
Kabuli_wild_78956	1399945259	*Ca_kabuli_scaffold*	78956	A/G	NA	INTERGENIC	NA
Kabuli_wild_129326	1399945260	*Ca_kabuli_scaffold*	129326	C/G	Gene_Ca_22416	SYNONYMOUS_CODING	Dehydrogenase,E1component
Kabuli_wild_129370	1399945261	*Ca_kabuli_scaffold*	129370	A/G	Gene_Ca_22416	SYNONYMOUS_CODING	Dehydrogenase,E1component
Kabuli_wild_130569	1399945262	*Ca_kabuli_scaffold*	130569	G/C	Gene_Ca_22416	SYNONYMOUS_CODING	Dehydrogenase,E1component
Kabuli_wild_131911	1399945263	*Ca_kabuli_scaffold*	131911	A/G	Gene_Ca_22416	INTRON	Dehydrogenase,E1component
Kabuli_wild_136796	1399945264	*Ca_kabuli_scaffold*	136796	A/G	Gene_Ca_22417	INTRON	Zinc finger, RING-type
Kabuli_wild_138379	1399945265	*Ca_kabuli_scaffold*	138379	T/G	Gene_Ca_22417	NON_SYNONYMOUS_CODING	Zinc finger, RING-type
Kabuli_wild_139268	1399945266	*Ca_kabuli_scaffold*	139268	G/A	Gene_Ca_22417	SYNONYMOUS_CODING	Zinc finger, RING-type
Kabuli_wild_154556	1399945267	*Ca_kabuli_scaffold*	154556	T/C	Gene_Ca_22418	INTRON	Glycosyltransferase,group1

SNP IDs	NCBI dbSNP accession IDs (NCBI_SS#)	Chromosomes	Physical positions (bp)	SNPs	Gene accession IDs	Sequence components of genome	Putative functions
Kabuli_wild_205934	1399945268	*Ca_kabuli_scaffold*	205934	T/C	Gene_Ca_22422	SYNONYMOUS_CODING	PeptidaseS8/S53,subtilisin/kexin/sedolisin
Kabuli_wild_341613	1399945269	*Ca_kabuli_scaffold*	341613	A/G	NA	INTERGENIC	NA
Kabuli_wild_108833	1399945270	*Ca_kabuli_scaffold*	108833	C/T	Gene_Ca_25689	SYNONYMOUS_CODING	Tetra tricopeptide repeat-containing
Kabuli_wild_108908	1399945271	*Ca_kabuli_scaffold*	108908	T/G	Gene_Ca_25689	INTRON	Tetra tricopeptide repeat-containing
Kabuli_wild_112026	1399945272	*Ca_kabuli_scaffold*	112026	A/C	Gene_Ca_25689	INTRON	Tetra tricopeptide repeat-containing
Kabuli_wild_12398	1399945273	*Ca_kabuli_scaffold*	12398	C/A	NA	INTERGENIC	NA
Kabuli_wild_36755	1399945274	*Ca_kabuli_scaffold*	36755	T/C	NA	INTERGENIC	NA
Kabuli_wild_39934	1399945275	*Ca_kabuli_scaffold*	39934	T/G	NA	INTERGENIC	NA
Kabuli_wild_39984	1399945276	*Ca_kabuli_scaffold*	39984	T/G	NA	INTERGENIC	NA
Kabuli_wild_39985	1399945277	*Ca_kabuli_scaffold*	39985	C/T	NA	INTERGENIC	NA
Kabuli_wild_39990	1399945278	*Ca_kabuli_scaffold*	39990	G/A	NA	INTERGENIC	NA
Kabuli_wild_39994	1399945279	*Ca_kabuli_scaffold*	39994	C/T	NA	INTERGENIC	NA
Kabuli_wild_158548	1399945280	*Ca_kabuli_scaffold*	158548	T/C	NA	INTERGENIC	NA
Kabuli_wild_158557	1399945281	*Ca_kabuli_scaffold*	158557	G/A	NA	INTERGENIC	NA
Kabuli_wild_158569	1399945282	*Ca_kabuli_scaffold*	158569	C/T	NA	INTERGENIC	NA
Kabuli_wild_158570	1399945283	*Ca_kabuli_scaffold*	158570	A/G	NA	INTERGENIC	NA
Kabuli_wild_158594	1399945284	*Ca_kabuli_scaffold*	158594	A/G	NA	INTERGENIC	NA
Kabuli_wild_158611	1399945285	*Ca_kabuli_scaffold*	158611	C/T	NA	INTERGENIC	NA
Kabuli_wild_158626	1399945286	*Ca_kabuli_scaffold*	158626	G/T	NA	INTERGENIC	NA
Kabuli_wild_158638	1399945287	*Ca_kabuli_scaffold*	158638	G/A	NA	INTERGENIC	NA
Kabuli_wild_166613	1399945288	*Ca_kabuli_scaffold*	166613	T/A	NA	INTERGENIC	NA
Kabuli_wild_166616	1399945289	*Ca_kabuli_scaffold*	166616	C/T	NA	INTERGENIC	NA
Kabuli_wild_166618	1399945290	*Ca_kabuli_scaffold*	166618	C/T	NA	INTERGENIC	NA
Kabuli_wild_166631	1399945291	*Ca_kabuli_scaffold*	166631	G/T	NA	INTERGENIC	NA
Kabuli_wild_172017	1399945292	*Ca_kabuli_scaffold*	172017	A/G	NA	INTERGENIC	NA
Kabuli_wild_172139	1399945293	*Ca_kabuli_scaffold*	172139	T/G	NA	INTERGENIC	NA
Kabuli_wild_172184	1399945294	*Ca_kabuli_scaffold*	172184	T/C	NA	INTERGENIC	NA
Kabuli_wild_5162	1399945295	*Ca_kabuli_scaffold*	5162	G/A	Gene_Ca_27907	NON_SYNONYMOUS_CODING	Peptidase C48, SUMO/Sentrin/Ubl1
Kabuli_wild_5229	1399945296	*Ca_kabuli_scaffold*	5229	G/A	Gene_Ca_27907	NON_SYNONYMOUS_CODING	Peptidase C48, SUMO/Sentrin/Ubl1
Kabuli_wild_5253	1399945297	*Ca_kabuli_scaffold*	5253	C/T	Gene_Ca_27907	NON_SYNONYMOUS_CODING	Peptidase C48, SUMO/Sentrin/Ubl1
Kabuli_wild_6347	1399945298	*Ca_kabuli_scaffold*	6347	G/A	NA	INTERGENIC	NA
Kabuli_wild_6426	1399945299	*Ca_kabuli_scaffold*	6426	G/A	NA	INTERGENIC	NA
Kabuli_wild_83811	1399945300	*Ca_kabuli_scaffold*	83811	G/C	NA	INTERGENIC	NA
Kabuli_wild_83850	1399945301	*Ca_kabuli_scaffold*	83850	G/A	NA	INTERGENIC	NA
Kabuli_wild_27261	1399945302	*Ca_kabuli_scaffold*	27261	G/A	NA	INTERGENIC	NA
Kabuli_wild_27279	1399945303	*Ca_kabuli_scaffold*	27279	C/T	NA	INTERGENIC	NA
Kabuli_wild_27284	1399945304	*Ca_kabuli_scaffold*	27284	C/T	NA	INTERGENIC	NA
Kabuli_wild_27308	1399945305	*Ca_kabuli_scaffold*	27308	T/G	NA	INTERGENIC	NA
Kabuli_wild_27332	1399945306	*Ca_kabuli_scaffold*	27332	G/A	NA	INTERGENIC	NA
Kabuli_wild_47025	1399945307	*Ca_kabuli_scaffold*	47025	G/A	NA	INTERGENIC	NA
Kabuli_wild_47038	1399945308	*Ca_kabuli_scaffold*	47038	G/A	NA	INTERGENIC	NA
Kabuli_wild_47048	1399945309	*Ca_kabuli_scaffold*	47048	T/G	NA	INTERGENIC	NA
Kabuli_wild_47082	1399945310	*Ca_kabuli_scaffold*	47082	C/A	NA	INTERGENIC	NA
Kabuli_wild_17568	1399945311	*Ca_kabuli_scaffold*	17568	G/A	Gene_Ca_27998	NON_SYNONYMOUS_CODING	NA

SNP IDs	NCBI dbSNP accession IDs (NCBI_SS#)	Chromosomes	Physical positions (bp)	SNPs	Gene accession IDs	Sequence components of genome	Putative functions
Kabuli_wild_265386	1399945312	*Ca_kabuli_scaffold*	265386	C/T	NA	INTERGENIC	NA
Kabuli_wild_265391	1399945313	*Ca_kabuli_scaffold*	265391	G/A	NA	INTERGENIC	NA
Kabuli_wild_265394	1399945314	*Ca_kabuli_scaffold*	265394	C/T	NA	INTERGENIC	NA
Kabuli_wild_265396	1399945315	*Ca_kabuli_scaffold*	265396	C/T	NA	INTERGENIC	NA
Kabuli_wild_265399	1399945316	*Ca_kabuli_scaffold*	265399	C/A	NA	INTERGENIC	NA
Kabuli_wild_265433	1399945317	*Ca_kabuli_scaffold*	265433	G/A	NA	INTERGENIC	NA
Kabuli_wild_265450	1399945318	*Ca_kabuli_scaffold*	265450	A/T	NA	INTERGENIC	NA
Kabuli_wild_265458	1399945319	*Ca_kabuli_scaffold*	265458	C/T	NA	INTERGENIC	NA
Kabuli_wild_265464	1399945320	*Ca_kabuli_scaffold*	265464	A/G	NA	INTERGENIC	NA
Kabuli_wild_49179	1399945321	*Ca_kabuli_scaffold*	49179	T/C	Gene_Ca_26775	DOWNSTREAM	UvrABCcomplex,subunitB
Kabuli_wild_51701	1399945322	*Ca_kabuli_scaffold*	51701	T/C	NA	INTERGENIC	NA
Kabuli_wild_51702	1399945323	*Ca_kabuli_scaffold*	51702	T/A	NA	INTERGENIC	NA
Kabuli_wild_51703	1399945324	*Ca_kabuli_scaffold*	51703	C/G	NA	INTERGENIC	NA
Kabuli_wild_51704	1399945325	*Ca_kabuli_scaffold*	51704	C/A	NA	INTERGENIC	NA
Kabuli_wild_62660	1399945326	*Ca_kabuli_scaffold*	62660	C/T	NA	INTERGENIC	NA
Kabuli_wild_62738	1399945327	*Ca_kabuli_scaffold*	62738	A/G	NA	INTERGENIC	NA
Kabuli_wild_386550	1399945328	*Ca_kabuli_scaffold*	386550	C/G	NA	INTERGENIC	NA
Kabuli_wild_9813	1399945329	*Ca_kabuli_scaffold*	9813	G/A	Gene_Ca_28083	NON_SYNONYMOUS_CODING	Orotidine5'-phosphatedecarboxylasedomain
Kabuli_wild_9851	1399945330	*Ca_kabuli_scaffold*	9851	C/T	Gene_Ca_28083	SYNONYMOUS_CODING	Orotidine5'-phosphatedecarboxylasedomain
Kabuli_wild_52131	1399945331	*Ca_kabuli_scaffold*	52131	G/A	Gene_Ca_26594	NON_SYNONYMOUS_CODING	NA
Kabuli_wild_52136	1399945332	*Ca_kabuli_scaffold*	52136	C/A	Gene_Ca_26594	SYNONYMOUS_CODING	NA
Kabuli_wild_52157	1399945333	*Ca_kabuli_scaffold*	52157	T/G	Gene_Ca_26594	SYNONYMOUS_CODING	NA
Kabuli_wild_52168	1399945334	*Ca_kabuli_scaffold*	52168	C/T	Gene_Ca_26594	NON_SYNONYMOUS_CODING	NA
Kabuli_wild_52209	1399945335	*Ca_kabuli_scaffold*	52209	G/A	Gene_Ca_26594	NON_SYNONYMOUS_CODING	NA
Kabuli_wild_52213	1399945336	*Ca_kabuli_scaffold*	52213	C/T	Gene_Ca_26594	NON_SYNONYMOUS_CODING	NA
Kabuli_wild_117376	1399945337	*Ca_kabuli_scaffold*	117376	G/A	NA	INTERGENIC	NA
Kabuli_wild_118266	1399945338	*Ca_kabuli_scaffold*	118266	G/T	NA	INTERGENIC	NA
Kabuli_wild_232543	1399945339	*Ca_kabuli_scaffold*	232543	G/T	NA	INTERGENIC	NA
Kabuli_wild_232545	1399945340	*Ca_kabuli_scaffold*	232545	C/A	NA	INTERGENIC	NA
Kabuli_wild_232570	1399945341	*Ca_kabuli_scaffold*	232570	T/G	NA	INTERGENIC	NA
Kabuli_wild_232581	1399945342	*Ca_kabuli_scaffold*	232581	C/T	NA	INTERGENIC	NA
Kabuli_wild_232628	1399945343	*Ca_kabuli_scaffold*	232628	G/A	NA	INTERGENIC	NA
Kabuli_wild_232629	1399945344	*Ca_kabuli_scaffold*	232629	C/T	NA	INTERGENIC	NA
Kabuli_wild_358449	1399945345	*Ca_kabuli_scaffold*	358449	T/A	NA	INTERGENIC	NA
Kabuli_wild_396159	1399945346	*Ca_kabuli_scaffold*	396159	T/C	NA	INTERGENIC	NA
Kabuli_wild_396201	1399945347	*Ca_kabuli_scaffold*	396201	A/G	NA	INTERGENIC	NA
Kabuli_wild_396212	1399945348	*Ca_kabuli_scaffold*	396212	G/A	NA	INTERGENIC	NA
Kabuli_wild_396222	1399945349	*Ca_kabuli_scaffold*	396222	A/G	NA	INTERGENIC	NA
Kabuli_wild_396234	1399945350	*Ca_kabuli_scaffold*	396234	A/G	NA	INTERGENIC	NA
Kabuli_wild_609386	1399945351	*Ca_kabuli_scaffold*	609386	C/T	NA	INTERGENIC	NA
Kabuli_wild_24311	1399945352	*Ca_kabuli_scaffold*	24311	A/G	Gene_Ca_27454	INTRON	Sugar/inositol transporter
Kabuli_wild_24316	1399945353	*Ca_kabuli_scaffold*	24316	T/A	Gene_Ca_27454	INTRON	Sugar/inositol transporter
Kabuli_wild_24317	1399945354	*Ca_kabuli_scaffold*	24317	G/T	Gene_Ca_27454	INTRON	Sugar/inositol transporter
Kabuli_wild_24323	1399945355	*Ca_kabuli_scaffold*	24323	A/G	Gene_Ca_27454	INTRON	Sugar/inositol transporter

SNP IDs	NCBI dbSNP accession IDs (NCBI_SS#)	Chromosomes	Physical positions (bp)	SNPs	Gene accession IDs	Sequence components of genome	Putative functions
Kabuli_wild_24336	1399945356	*Ca_kabuli_scaffold*	24336	G/T	Gene_Ca_27454	STOP_GAINED	Sugar/inositol transporter
Kabuli_wild_24349	1399945357	*Ca_kabuli_scaffold*	24349	T/C	Gene_Ca_27454	NON_SYNONYMOUS_CODING	Sugar/inositol transporter
Kabuli_wild_35489	1399945358	*Ca_kabuli_scaffold*	35489	A/G	Gene_Ca_27455	SYNONYMOUS_CODING	Sugar/inositol transporter
Kabuli_wild_35494	1399945359	*Ca_kabuli_scaffold*	35494	T/A	Gene_Ca_27455	NON_SYNONYMOUS_CODING	Sugar/inositol transporter
Kabuli_wild_35495	1399945360	*Ca_kabuli_scaffold*	35495	G/T	Gene_Ca_27455	SYNONYMOUS_CODING	Sugar/inositol transporter
Kabuli_wild_35501	1399945361	*Ca_kabuli_scaffold*	35501	A/G	Gene_Ca_27455	SYNONYMOUS_CODING	Sugar/inositol transporter
Kabuli_wild_35514	1399945362	*Ca_kabuli_scaffold*	35514	G/T	Gene_Ca_27455	STOP_GAINED	Sugar/inositol transporter
Kabuli_wild_35527	1399945363	*Ca_kabuli_scaffold*	35527	T/C	Gene_Ca_27455	NON_SYNONYMOUS_CODING	Sugar/inositol transporter
Kabuli_wild_18453	1399945364	*Ca_kabuli_scaffold*	18453	A/G	NA	INTERGENIC	NA
Kabuli_wild_18621	1399945365	*Ca_kabuli_scaffold*	18621	C/A	NA	INTERGENIC	NA
Kabuli_wild_18693	1399945366	*Ca_kabuli_scaffold*	18693	G/C	NA	INTERGENIC	NA
Kabuli_wild_20978	1399945367	*Ca_kabuli_scaffold*	20978	T/C	Gene_Ca_24246	NON_SYNONYMOUS_CODING	Leucine-rich repeat
Kabuli_wild_40849	1399945368	*Ca_kabuli_scaffold*	40849	A/C	NA	INTERGENIC	NA
Kabuli_wild_113895	1399945369	*Ca_kabuli_scaffold*	113895	A/C	NA	INTERGENIC	NA
Kabuli_wild_113932	1399945370	*Ca_kabuli_scaffold*	113932	T/A	NA	INTERGENIC	NA
Kabuli_wild_158250	1399945371	*Ca_kabuli_scaffold*	158250	A/G	NA	INTERGENIC	NA
Kabuli_wild_63458	1399945372	*Ca_kabuli_scaffold*	63458	G/C	NA	INTERGENIC	NA
Kabuli_wild_63460	1399945373	*Ca_kabuli_scaffold*	63460	C/A	NA	INTERGENIC	NA
Kabuli_wild_91817	1399945374	*Ca_kabuli_scaffold*	91817	C/T	Gene_Ca_26439	INTRON	K Homology
Kabuli_wild_91849	1399945375	*Ca_kabuli_scaffold*	91849	T/C	Gene_Ca_26439	SYNONYMOUS_CODING	K Homology
Kabuli_wild_15785	1399945376	*Ca_kabuli_scaffold*	15785	G/A	NA	INTERGENIC	NA
Kabuli_wild_15786	1399945377	*Ca_kabuli_scaffold*	15786	G/A	NA	INTERGENIC	NA
Kabuli_wild_15799	1399945378	*Ca_kabuli_scaffold*	15799	C/T	NA	INTERGENIC	NA
Kabuli_wild_15810	1399945379	*Ca_kabuli_scaffold*	15810	A/G	NA	INTERGENIC	NA
Kabuli_wild_15811	1399945380	*Ca_kabuli_scaffold*	15811	T/C	NA	INTERGENIC	NA
Kabuli_wild_15813	1399945381	*Ca_kabuli_scaffold*	15813	G/T	NA	INTERGENIC	NA
Kabuli_wild_15816	1399945382	*Ca_kabuli_scaffold*	15816	C/T	NA	INTERGENIC	NA
Kabuli_wild_15818	1399945383	*Ca_kabuli_scaffold*	15818	G/A	NA	INTERGENIC	NA
Kabuli_wild_15819	1399945384	*Ca_kabuli_scaffold*	15819	A/G	NA	INTERGENIC	NA
Kabuli_wild_15822	1399945385	*Ca_kabuli_scaffold*	15822	C/T	NA	INTERGENIC	NA
Kabuli_wild_15834	1399945386	*Ca_kabuli_scaffold*	15834	C/A	NA	INTERGENIC	NA
Kabuli_wild_15836	1399945387	*Ca_kabuli_scaffold*	15836	G/T	NA	INTERGENIC	NA
Kabuli_wild_15841	1399945388	*Ca_kabuli_scaffold*	15841	G/A	NA	INTERGENIC	NA
Kabuli_wild_15842	1399945389	*Ca_kabuli_scaffold*	15842	C/T	NA	INTERGENIC	NA
Kabuli_wild_15845	1399945390	*Ca_kabuli_scaffold*	15845	C/A	NA	INTERGENIC	NA
Kabuli_wild_81301	1399945391	*Ca_kabuli_scaffold*	81301	G/A	NA	INTERGENIC	NA
Kabuli_wild_306129	1399945392	*Ca_kabuli_scaffold*	306129	C/T	NA	INTERGENIC	NA
Kabuli_wild_306135	1399945393	*Ca_kabuli_scaffold*	306135	A/C	NA	INTERGENIC	NA
Kabuli_wild_306164	1399945394	*Ca_kabuli_scaffold*	306164	G/A	NA	INTERGENIC	NA
Kabuli_wild_306169	1399945395	*Ca_kabuli_scaffold*	306169	A/G	NA	INTERGENIC	NA
Kabuli_wild_306180	1399945396	*Ca_kabuli_scaffold*	306180	T/C	NA	INTERGENIC	NA
Kabuli_wild_332494	1399945397	*Ca_kabuli_scaffold*	332494	G/C	Gene_Ca_23215	NON_SYNONYMOUS_CODING	NA
Kabuli_wild_358975	1399945398	*Ca_kabuli_scaffold*	358975	C/T	NA	INTERGENIC	NA
Kabuli_wild_359004	1399945399	*Ca_kabuli_scaffold*	359004	G/T	NA	INTERGENIC	NA

SNP IDs	NCBI dbSNP accession IDs (NCBI_SS#)	Chromosomes	Physical positions (bp)	SNPs	Gene accession IDs	Sequence components of genome	Putative functions
Kabuli_wild_403513	1399945400	*Ca_kabuli_scaffold*	403513	G/A	NA	INTERGENIC	NA
Kabuli_wild_403521	1399945401	*Ca_kabuli_scaffold*	403521	T/G	NA	INTERGENIC	NA
Kabuli_wild_403531	1399945402	*Ca_kabuli_scaffold*	403531	A/C	NA	INTERGENIC	NA
Kabuli_wild_403553	1399945403	*Ca_kabuli_scaffold*	403553	T/C	NA	INTERGENIC	NA
Kabuli_wild_403572	1399945404	*Ca_kabuli_scaffold*	403572	C/G	NA	INTERGENIC	NA
Kabuli_wild_53125	1399945405	*Ca_kabuli_scaffold*	53125	T/A	NA	INTERGENIC	NA
Kabuli_wild_53132	1399945406	*Ca_kabuli_scaffold*	53132	C/T	NA	INTERGENIC	NA
Kabuli_wild_53223	1399945407	*Ca_kabuli_scaffold*	53223	A/C	NA	INTERGENIC	NA
Kabuli_wild_708	1399945408	*Ca_kabuli_scaffold*	708	C/T	Gene_Ca_21034	SYNONYMOUS_CODING	Major facilitator superfamily MFS-1
Kabuli_wild_1036	1399945409	*Ca_kabuli_scaffold*	1036	A/G	Gene_Ca_21034	NON_SYNONYMOUS_CODING	Major facilitator superfamily MFS-1
Kabuli_wild_206029	1399945410	*Ca_kabuli_scaffold*	206029	C/A	Gene_Ca_21044	NON_SYNONYMOUS_CODING	General substrate transporter
Kabuli_wild_206030	1399945411	*Ca_kabuli_scaffold*	206030	A/G	Gene_Ca_21044	SYNONYMOUS_CODING	General substrate transporter
Kabuli_wild_275493	1399945412	*Ca_kabuli_scaffold*	275493	A/T	Gene_Ca_21047	INTRON	C4-dicarboxylatetransporter/malicacidtransportprotein
Kabuli_wild_275497	1399945413	*Ca_kabuli_scaffold*	275497	T/C	Gene_Ca_21047	INTRON	C4-dicarboxylatetransporter/malicacidtransportprotein
Kabuli_wild_275510	1399945414	*Ca_kabuli_scaffold*	275510	A/T	Gene_Ca_21047	INTRON	C4-dicarboxylatetransporter/malicacidtransportprotein
Kabuli_wild_275512	1399945415	*Ca_kabuli_scaffold*	275512	G/T	Gene_Ca_21047	INTRON	C4-dicarboxylatetransporter/malicacidtransportprotein
Kabuli_wild_275519	1399945416	*Ca_kabuli_scaffold*	275519	A/C	Gene_Ca_21047	INTRON	C4-dicarboxylatetransporter/malicacidtransportprotein
Kabuli_wild_295864	1399945417	*Ca_kabuli_scaffold*	295864	G/T	NA	INTERGENIC	NA
Kabuli_wild_402860	1399945418	*Ca_kabuli_scaffold*	402860	A/G	NA	INTERGENIC	NA
Kabuli_wild_119274	1399945419	*Ca_kabuli_scaffold*	119274	T/C	Gene_Ca_22829	INTRON	NA
Kabuli_wild_213783	1399945420	*Ca_kabuli_scaffold*	213783	T/C	NA	INTERGENIC	NA
Kabuli_wild_111921	1399945421	*Ca_kabuli_scaffold*	111921	C/A	Gene_Ca_27326	INTRON	Protein of unknown function DUF3414
Kabuli_wild_121629	1399945422	*Ca_kabuli_scaffold*	121629	T/C	NA	INTERGENIC	NA
Kabuli_wild_183177	1399945423	*Ca_kabuli_scaffold*	183177	T/C	Gene_Ca_27327	UPSTREAM	NA
Kabuli_wild_13212	1399945424	*Ca_kabuli_scaffold*	13212	T/C	NA	INTERGENIC	NA
Kabuli_wild_132087	1399945425	*Ca_kabuli_scaffold*	132087	C/G	NA	INTERGENIC	NA
Kabuli_wild_134580	1399945426	*Ca_kabuli_scaffold*	134580	C/G	Gene_Ca_25988	INTRON	VacuolarATPaseassemblyintegralmembraneproteinVMA21-likedomain
Kabuli_wild_134707	1399945427	*Ca_kabuli_scaffold*	134707	G/A	Gene_Ca_25988	INTRON	VacuolarATPaseassemblyintegralmembraneproteinVMA21-likedomain
Kabuli_wild_29284	1399945428	*Ca_kabuli_scaffold*	29284	A/T	NA	INTERGENIC	NA
Kabuli_wild_12188	1399945429	*Ca_kabuli_scaffold*	12188	C/T	NA	INTERGENIC	NA
Kabuli_wild_21152	1399945430	*Ca_kabuli_scaffold*	21152	C/T	Gene_Ca_27369	NON_SYNONYMOUS_CODING	AThook,DNA-bindingmotif
Kabuli_wild_3061	1399945431	*Ca_kabuli_scaffold*	3061	A/T	Gene_Ca_25481	SYNONYMOUS_CODING	Photosystemantennaprotein-like
Kabuli_wild_42329	1399945432	*Ca_kabuli_scaffold*	42329	T/C	NA	INTERGENIC	NA
Kabuli_wild_57413	1399945433	*Ca_kabuli_scaffold*	57413	A/T	NA	INTERGENIC	NA
Kabuli_wild_21441	1399945434	*Ca_kabuli_scaffold*	21441	C/A	NA	INTERGENIC	NA
Kabuli_wild_21442	1399945435	*Ca_kabuli_scaffold*	21442	G/C	NA	INTERGENIC	NA
Kabuli_wild_21443	1399945436	*Ca_kabuli_scaffold*	21443	A/C	NA	INTERGENIC	NA
Kabuli_wild_23303	1399945437	*Ca_kabuli_scaffold*	23303	T/G	NA	INTERGENIC	NA
Kabuli_wild_27693	1399945438	*Ca_kabuli_scaffold*	27693	T/C	NA	INTERGENIC	NA
Kabuli_wild_27710	1399945439	*Ca_kabuli_scaffold*	27710	T/G	NA	INTERGENIC	NA
Kabuli_wild_28014	1399945440	*Ca_kabuli_scaffold*	28014	T/A	NA	INTERGENIC	NA
Kabuli_wild_28015	1399945441	*Ca_kabuli_scaffold*	28015	A/C	NA	INTERGENIC	NA
Kabuli_wild_28456	1399945442	*Ca_kabuli_scaffold*	28456	A/C	NA	INTERGENIC	NA
Kabuli_wild_29735	1399945443	*Ca_kabuli_scaffold*	29735	G/C	NA	INTERGENIC	NA

SNP IDs	NCBI dbSNP accession IDs (NCBI_SS#)	Chromosomes	Physical positions (bp)	SNPs	Gene accession IDs	Sequence components of genome	Putative functions
Kabuli_wild_29750	1399945444	*Ca_kabuli_scaffold*	29750	G/T	NA	INTERGENIC	NA
Kabuli_wild_29797	1399945445	*Ca_kabuli_scaffold*	29797	C/T	NA	INTERGENIC	NA
Kabuli_wild_29798	1399945446	*Ca_kabuli_scaffold*	29798	C/A	NA	INTERGENIC	NA
Kabuli_wild_56111	1399945447	*Ca_kabuli_scaffold*	56111	G/A	Gene_Ca_27234	NON_SYNONYMOUS_CODING	Peptidase C48, SUMO/Sentrin/Ubl1
Kabuli_wild_7155	1399945448	*Ca_kabuli_scaffold*	7155	G/A	NA	INTERGENIC	NA
Kabuli_wild_7159	1399945449	*Ca_kabuli_scaffold*	7159	T/G	NA	INTERGENIC	NA
Kabuli_wild_7166	1399945450	*Ca_kabuli_scaffold*	7166	C/T	NA	INTERGENIC	NA
Kabuli_wild_7182	1399945451	*Ca_kabuli_scaffold*	7182	C/T	NA	INTERGENIC	NA
Kabuli_wild_7196	1399945452	*Ca_kabuli_scaffold*	7196	G/T	NA	INTERGENIC	NA
Kabuli_wild_40218	1399945453	*Ca_kabuli_scaffold*	40218	G/A	Gene_Ca_26498	INTRON	Peptidase C48, SUMO/Sentrin/Ubl1
Kabuli_wild_137408	1399945454	*Ca_kabuli_scaffold*	137408	A/C	Gene_Ca_26500	DOWNSTREAM	NA
Kabuli_wild_137416	1399945455	*Ca_kabuli_scaffold*	137416	G/A	Gene_Ca_26500	DOWNSTREAM	NA
Kabuli_wild_137433	1399945456	*Ca_kabuli_scaffold*	137433	G/T	Gene_Ca_26500	DOWNSTREAM	NA
Kabuli_wild_137455	1399945457	*Ca_kabuli_scaffold*	137455	G/T	Gene_Ca_26500	DOWNSTREAM	NA
Kabuli_wild_149524	1399945458	*Ca_kabuli_scaffold*	149524	C/T	Gene_Ca_26502	NON_SYNONYMOUS_CODING	NA
Kabuli_wild_149537	1399945459	*Ca_kabuli_scaffold*	149537	G/A	Gene_Ca_26502	SYNONYMOUS_CODING	NA
Kabuli_wild_149545	1399945460	*Ca_kabuli_scaffold*	149545	C/T	Gene_Ca_26502	NON_SYNONYMOUS_CODING	NA
Kabuli_wild_149577	1399945461	*Ca_kabuli_scaffold*	149577	G/T	Gene_Ca_26502	NON_SYNONYMOUS_CODING	NA
Kabuli_wild_149580	1399945462	*Ca_kabuli_scaffold*	149580	G/A	Gene_Ca_26502	NON_SYNONYMOUS_CODING	NA
Kabuli_wild_149583	1399945463	*Ca_kabuli_scaffold*	149583	G/A	Gene_Ca_26502	NON_SYNONYMOUS_CODING	NA
Kabuli_wild_149585	1399945464	*Ca_kabuli_scaffold*	149585	G/A	Gene_Ca_26502	SYNONYMOUS_CODING	NA
Kabuli_wild_149589	1399945465	*Ca_kabuli_scaffold*	149589	G/A	Gene_Ca_26502	NON_SYNONYMOUS_CODING	NA
Kabuli_wild_149613	1399945466	*Ca_kabuli_scaffold*	149613	G/T	Gene_Ca_26502	NON_SYNONYMOUS_CODING	NA
Kabuli_wild_149620	1399945467	*Ca_kabuli_scaffold*	149620	C/G	Gene_Ca_26502	NON_SYNONYMOUS_CODING	NA
Kabuli_wild_149628	1399945468	*Ca_kabuli_scaffold*	149628	G/A	Gene_Ca_26502	NON_SYNONYMOUS_CODING	NA
Kabuli_wild_149631	1399945469	*Ca_kabuli_scaffold*	149631	G/A	Gene_Ca_26502	NON_SYNONYMOUS_CODING	NA
Kabuli_wild_149645	1399945470	*Ca_kabuli_scaffold*	149645	G/T	Gene_Ca_26502	SYNONYMOUS_CODING	NA
Kabuli_wild_149649	1399945471	*Ca_kabuli_scaffold*	149649	C/T	Gene_Ca_26502	NON_SYNONYMOUS_CODING	NA
Kabuli_wild_149661	1399945472	*Ca_kabuli_scaffold*	149661	G/A	Gene_Ca_26502	NON_SYNONYMOUS_CODING	NA
Kabuli_wild_149676	1399945473	*Ca_kabuli_scaffold*	149676	T/C	Gene_Ca_26502	NON_SYNONYMOUS_CODING	NA
Kabuli_wild_149687	1399945474	*Ca_kabuli_scaffold*	149687	A/T	Gene_Ca_26502	SYNONYMOUS_CODING	NA
Kabuli_wild_149689	1399945475	*Ca_kabuli_scaffold*	149689	G/A	Gene_Ca_26502	NON_SYNONYMOUS_CODING	NA
Kabuli_wild_77099	1399945476	*Ca_kabuli_scaffold*	77099	C/T	NA	INTERGENIC	NA
Kabuli_wild_88007	1399945477	*Ca_kabuli_scaffold*	88007	G/T	Gene_Ca_26915	NON_SYNONYMOUS_CODING	NA
Kabuli_wild_3364	1399945478	*Ca_kabuli_scaffold*	3364	G/T	NA	INTERGENIC	NA
Kabuli_wild_13634	1399945479	*Ca_kabuli_scaffold*	13634	C/T	NA	INTERGENIC	NA
Kabuli_wild_13712	1399945480	*Ca_kabuli_scaffold*	13712	A/G	NA	INTERGENIC	NA
Kabuli_wild_13730	1399945481	*Ca_kabuli_scaffold*	13730	A/G	NA	INTERGENIC	NA
Kabuli_wild_13736	1399945482	*Ca_kabuli_scaffold*	13736	G/A	NA	INTERGENIC	NA
Kabuli_wild_13745	1399945483	*Ca_kabuli_scaffold*	13745	G/A	NA	INTERGENIC	NA
Kabuli_wild_82268	1399945484	*Ca_kabuli_scaffold*	82268	T/C	Gene_Ca_27591	INTRON	Endonuclease/exonuclease/phosphatase
Kabuli_wild_83209	1399945485	*Ca_kabuli_scaffold*	83209	T/A	Gene_Ca_27591	INTRON	Endonuclease/exonuclease/phosphatase
Kabuli_wild_83244	1399945486	*Ca_kabuli_scaffold*	83244	T/C	Gene_Ca_27591	INTRON	Endonuclease/exonuclease/phosphatase
Kabuli_wild_84007	1399945487	*Ca_kabuli_scaffold*	84007	C/G	Gene_Ca_27591	INTRON	Endonuclease/exonuclease/phosphatase

SNP IDs	NCBI dbSNP accession IDs (NCBI_SS#)	Chromosomes	Physical positions (bp)	SNPs	Gene accession IDs	Sequence components of genome	Putative functions
Kabuli_wild_84057	1399945488	*Ca_kabuli_scaffold*	84057	A/G	Gene_Ca_27591	NON_SYNONYMOUS_CODING	Endonuclease/exonuclease/phosphatase
Kabuli_wild_84072	1399945489	*Ca_kabuli_scaffold*	84072	G/A	Gene_Ca_27591	NON_SYNONYMOUS_CODING	Endonuclease/exonuclease/phosphatase
Kabuli_wild_84088	1399945490	*Ca_kabuli_scaffold*	84088	C/G	Gene_Ca_27591	NON_SYNONYMOUS_CODING	Endonuclease/exonuclease/phosphatase
Kabuli_wild_3006	1399945491	*Ca_kabuli_scaffold*	3006	G/A	NA	INTERGENIC	NA
Kabuli_wild_77761	1399945492	*Ca_kabuli_scaffold*	77761	A/T	NA	INTERGENIC	NA
Kabuli_wild_77763	1399945493	*Ca_kabuli_scaffold*	77763	C/T	NA	INTERGENIC	NA
Kabuli_wild_77767	1399945494	*Ca_kabuli_scaffold*	77767	A/T	NA	INTERGENIC	NA
Kabuli_wild_78440	1399945495	*Ca_kabuli_scaffold*	78440	A/T	Gene_Ca_28108	NON_SYNONYMOUS_CODING	Photosyntheticreactioncentre,L/M
Kabuli_wild_78525	1399945496	*Ca_kabuli_scaffold*	78525	T/A	Gene_Ca_28108	SYNONYMOUS_CODING	Photosyntheticreactioncentre,L/M
Kabuli_wild_78532	1399945497	*Ca_kabuli_scaffold*	78532	T/G	Gene_Ca_28108	NON_SYNONYMOUS_CODING	Photosyntheticreactioncentre,L/M
Kabuli_wild_5619	1399945498	*Ca_kabuli_scaffold*	5619	G/A	NA	INTERGENIC	NA
Kabuli_wild_10560	1399945499	*Ca_kabuli_scaffold*	10560	G/A	Gene_Ca_18007	SYNONYMOUS_CODING	Peptidase C48, SUMO/Sentrin/Ubl1
Kabuli_wild_10575	1399945500	*Ca_kabuli_scaffold*	10575	C/T	Gene_Ca_18007	NON_SYNONYMOUS_CODING	Peptidase C48, SUMO/Sentrin/Ubl1
Kabuli_wild_10582	1399945501	*Ca_kabuli_scaffold*	10582	A/G	Gene_Ca_18007	SYNONYMOUS_CODING	Peptidase C48, SUMO/Sentrin/Ubl1
Kabuli_wild_40651	1399945502	*Ca_kabuli_scaffold*	40651	T/A	NA	INTERGENIC	NA
Kabuli_wild_40681	1399945503	*Ca_kabuli_scaffold*	40681	A/T	NA	INTERGENIC	NA
Kabuli_wild_382579	1399945504	*Ca_kabuli_scaffold*	382579	C/T	NA	INTERGENIC	NA
Kabuli_wild_436598	1399945505	*Ca_kabuli_scaffold*	436598	T/C	Gene_Ca_18032	UPSTREAM	Metridin-likeShKtoxin
Kabuli_wild_436923	1399945506	*Ca_kabuli_scaffold*	436923	T/C	Gene_Ca_18032	UPSTREAM	Metridin-likeShKtoxin
Kabuli_wild_644893	1399945507	*Ca_kabuli_scaffold*	644893	A/C	Gene_Ca_18050	NON_SYNONYMOUS_CODING	CoA-binding
Kabuli_wild_667013	1399945508	*Ca_kabuli_scaffold*	667013	A/T	Gene_Ca_18053	INTRON	NA
Kabuli_wild_667017	1399945509	*Ca_kabuli_scaffold*	667017	A/G	Gene_Ca_18053	INTRON	NA
Kabuli_wild_667478	1399945510	*Ca_kabuli_scaffold*	667478	T/G	Gene_Ca_18053	INTRON	NA
Kabuli_wild_667494	1399945511	*Ca_kabuli_scaffold*	667494	G/C	Gene_Ca_18053	INTRON	NA
Kabuli_wild_667548	1399945512	*Ca_kabuli_scaffold*	667548	A/G	Gene_Ca_18053	INTRON	NA
Kabuli_wild_667620	1399945513	*Ca_kabuli_scaffold*	667620	A/G	Gene_Ca_18053	INTRON	NA
Kabuli_wild_667713	1399945514	*Ca_kabuli_scaffold*	667713	G/A	Gene_Ca_18053	INTRON	NA
Kabuli_wild_667750	1399945515	*Ca_kabuli_scaffold*	667750	T/C	Gene_Ca_18053	INTRON	NA
Kabuli_wild_674495	1399945516	*Ca_kabuli_scaffold*	674495	A/G	NA	INTERGENIC	NA
Kabuli_wild_674823	1399945517	*Ca_kabuli_scaffold*	674823	G/A	NA	INTERGENIC	NA
Kabuli_wild_674826	1399945518	*Ca_kabuli_scaffold*	674826	C/T	NA	INTERGENIC	NA
Kabuli_wild_674844	1399945519	*Ca_kabuli_scaffold*	674844	T/C	NA	INTERGENIC	NA
Kabuli_wild_674847	1399945520	*Ca_kabuli_scaffold*	674847	T/G	NA	INTERGENIC	NA
Kabuli_wild_674862	1399945521	*Ca_kabuli_scaffold*	674862	G/A	NA	INTERGENIC	NA
Kabuli_wild_674865	1399945522	*Ca_kabuli_scaffold*	674865	T/C	NA	INTERGENIC	NA
Kabuli_wild_674871	1399945523	*Ca_kabuli_scaffold*	674871	A/G	NA	INTERGENIC	NA
Kabuli_wild_674881	1399945524	*Ca_kabuli_scaffold*	674881	G/A	NA	INTERGENIC	NA
Kabuli_wild_90092	1399945525	*Ca_kabuli_scaffold*	90092	G/A	NA	INTERGENIC	NA
Kabuli_wild_48081	1399945526	*Ca_kabuli_scaffold*	48081	T/A	NA	INTERGENIC	NA
Kabuli_wild_48115	1399945527	*Ca_kabuli_scaffold*	48115	T/A	NA	INTERGENIC	NA
Kabuli_wild_48204	1399945528	*Ca_kabuli_scaffold*	48204	T/C	NA	INTERGENIC	NA
Kabuli_wild_56045	1399945529	*Ca_kabuli_scaffold*	56045	C/A	NA	INTERGENIC	NA
Kabuli_wild_56061	1399945530	*Ca_kabuli_scaffold*	56061	A/C	NA	INTERGENIC	NA
Kabuli_wild_56069	1399945531	*Ca_kabuli_scaffold*	56069	C/T	NA	INTERGENIC	NA

SNP IDs	NCBI dbSNP accession IDs (NCBI_SS#)	Chromosomes	Physical positions (bp)	SNPs	Gene accession IDs	Sequence components of genome	Putative functions
Kabuli_wild_56074	1399945532	*Ca_kabuli_scaffold*	56074	C/T	NA	INTERGENIC	NA
Kabuli_wild_56075	1399945533	*Ca_kabuli_scaffold*	56075	T/A	NA	INTERGENIC	NA
Kabuli_wild_56114	1399945534	*Ca_kabuli_scaffold*	56114	C/A	NA	INTERGENIC	NA
Kabuli_wild_56116	1399945535	*Ca_kabuli_scaffold*	56116	C/T	NA	INTERGENIC	NA
Kabuli_wild_56140	1399945536	*Ca_kabuli_scaffold*	56140	G/A	NA	INTERGENIC	NA
Kabuli_wild_78574	1399945537	*Ca_kabuli_scaffold*	78574	T/A	Gene_Ca_27595	INTRON	Uridinekinase
Kabuli_wild_121205	1399945538	*Ca_kabuli_scaffold*	121205	T/C	NA	INTERGENIC	NA
Kabuli_wild_121249	1399945539	*Ca_kabuli_scaffold*	121249	C/A	NA	INTERGENIC	NA
Kabuli_wild_256612	1399945540	*Ca_kabuli_scaffold*	256612	A/C	Gene_Ca_25976	UPSTREAM	NA
Kabuli_wild_73684	1399945541	*Ca_kabuli_scaffold*	73684	G/A	Gene_Ca_27683	STOP_GAINED	NA
Kabuli_wild_73710	1399945542	*Ca_kabuli_scaffold*	73710	G/A	Gene_Ca_27683	NON_SYNONYMOUS_CODING	NA
Kabuli_wild_16310	1399945543	*Ca_kabuli_scaffold*	16310	G/A	NA	INTERGENIC	NA
Kabuli_wild_16328	1399945544	*Ca_kabuli_scaffold*	16328	A/T	NA	INTERGENIC	NA
Kabuli_wild_16333	1399945545	*Ca_kabuli_scaffold*	16333	A/T	NA	INTERGENIC	NA
Kabuli_wild_16336	1399945546	*Ca_kabuli_scaffold*	16336	C/T	NA	INTERGENIC	NA
Kabuli_wild_90003	1399945547	*Ca_kabuli_scaffold*	90003	T/A	NA	INTERGENIC	NA
Kabuli_wild_90013	1399945548	*Ca_kabuli_scaffold*	90013	T/G	NA	INTERGENIC	NA
Kabuli_wild_93921	1399945549	*Ca_kabuli_scaffold*	93921	T/G	Gene_Ca_17178	SYNONYMOUS_CODING	RNA recognition motif domain
Kabuli_wild_93959	1399945550	*Ca_kabuli_scaffold*	93959	C/T	Gene_Ca_17178	NON_SYNONYMOUS_CODING	RNA recognition motif domain
Kabuli_wild_235352	1399945551	*Ca_kabuli_scaffold*	235352	T/C	Gene_Ca_17194	INTRON	Engulfment/cellmotility,ELMO
Kabuli_wild_250462	1399945552	*Ca_kabuli_scaffold*	250462	T/A	Gene_Ca_17196	NON_SYNONYMOUS_CODING	Protein kinase,catalytic domain
Kabuli_wild_303236	1399945553	*Ca_kabuli_scaffold*	303236	C/G	NA	INTERGENIC	NA
Kabuli_wild_332839	1399945554	*Ca_kabuli_scaffold*	332839	T/C	Gene_Ca_17201	NON_SYNONYMOUS_CODING	RibosomalproteinL4/L1e
Kabuli_wild_458152	1399945555	*Ca_kabuli_scaffold*	458152	G/C	Gene_Ca_17211	NON_SYNONYMOUS_CODING	Pathogenesis-related transcriptional factor/ERF, DNA-binding
Kabuli_wild_577874	1399945556	*Ca_kabuli_scaffold*	577874	T/G	NA	INTERGENIC	NA
Kabuli_wild_70280	1399945557	*Ca_kabuli_scaffold*	70280	A/T	NA	INTERGENIC	NA
Kabuli_wild_3379	1399945558	*Ca_kabuli_scaffold*	3379	C/A	Gene_Ca_25913	NON_SYNONYMOUS_CODING	Peptidase C48, SUMO/Sentrin/Ubl1
Kabuli_wild_3396	1399945559	*Ca_kabuli_scaffold*	3396	C/T	Gene_Ca_25913	SYNONYMOUS_CODING	Peptidase C48, SUMO/Sentrin/Ubl1
Kabuli_wild_3415	1399945560	*Ca_kabuli_scaffold*	3415	C/T	Gene_Ca_25913	NON_SYNONYMOUS_CODING	Peptidase C48, SUMO/Sentrin/Ubl1
Kabuli_wild_3438	1399945561	*Ca_kabuli_scaffold*	3438	C/T	Gene_Ca_25913	SYNONYMOUS_CODING	Peptidase C48, SUMO/Sentrin/Ubl1
Kabuli_wild_3463	1399945562	*Ca_kabuli_scaffold*	3463	C/T	Gene_Ca_25913	NON_SYNONYMOUS_CODING	Peptidase C48, SUMO/Sentrin/Ubl1
Kabuli_wild_3464	1399945563	*Ca_kabuli_scaffold*	3464	T/A	Gene_Ca_25913	SYNONYMOUS_CODING	Peptidase C48, SUMO/Sentrin/Ubl1
Kabuli_wild_126986	1399945564	*Ca_kabuli_scaffold*	126986	T/G	Gene_Ca_25916	INTRON	Synaptojanin,N-terminal
Kabuli_wild_127022	1399945565	*Ca_kabuli_scaffold*	127022	G/A	Gene_Ca_25916	INTRON	Synaptojanin,N-terminal
Kabuli_wild_127023	1399945566	*Ca_kabuli_scaffold*	127023	G/T	Gene_Ca_25916	INTRON	Synaptojanin,N-terminal
Kabuli_wild_127054	1399945567	*Ca_kabuli_scaffold*	127054	C/T	Gene_Ca_25916	INTRON	Synaptojanin,N-terminal
Kabuli_wild_8263	1399945568	*Ca_kabuli_scaffold*	8263	A/C	NA	INTERGENIC	NA
Kabuli_wild_8279	1399945569	*Ca_kabuli_scaffold*	8279	G/T	NA	INTERGENIC	NA
Kabuli_wild_8297	1399945570	*Ca_kabuli_scaffold*	8297	A/G	NA	INTERGENIC	NA
Kabuli_wild_271340	1399945571	*Ca_kabuli_scaffold*	271340	C/T	NA	INTERGENIC	NA
Kabuli_wild_344355	1399945572	*Ca_kabuli_scaffold*	344355	A/C	NA	INTERGENIC	NA
Kabuli_wild_344392	1399945573	*Ca_kabuli_scaffold*	344392	G/A	NA	INTERGENIC	NA
Kabuli_wild_344417	1399945574	*Ca_kabuli_scaffold*	344417	G/T	NA	INTERGENIC	NA
Kabuli_wild_344459	1399945575	*Ca_kabuli_scaffold*	344459	G/A	NA	INTERGENIC	NA

SNP IDs	NCBI dbSNP accession IDs (NCBI_SS#)	Chromosomes	Physical positions (bp)	SNPs	Gene accession IDs	Sequence components of genome	Putative functions
Kabuli_wild_3406	1399945576	*Ca_kabuli_scaffold*	3406	T/C	Gene_Ca_21773	INTRON	Pathogenesis-related transcriptional factor/ERF, DNA-binding
Kabuli_wild_114660	1399945577	*Ca_kabuli_scaffold*	114660	C/G	NA	INTERGENIC	NA
Kabuli_wild_114665	1399945578	*Ca_kabuli_scaffold*	114665	A/T	NA	INTERGENIC	NA
Kabuli_wild_394757	1399945579	*Ca_kabuli_scaffold*	394757	G/T	Gene_Ca_21793	INTRON	Transposase,Ptta/En/Spm,plant
Kabuli_wild_394800	1399945580	*Ca_kabuli_scaffold*	394800	T/A	Gene_Ca_21793	INTRON	Transposase,Ptta/En/Spm,plant
Kabuli_wild_394803	1399945581	*Ca_kabuli_scaffold*	394803	C/T	Gene_Ca_21793	INTRON	Transposase,Ptta/En/Spm,plant
Kabuli_wild_394805	1399945582	*Ca_kabuli_scaffold*	394805	C/T	Gene_Ca_21793	INTRON	Transposase,Ptta/En/Spm,plant
Kabuli_wild_394815	1399945583	*Ca_kabuli_scaffold*	394815	T/C	Gene_Ca_21793	INTRON	Transposase,Ptta/En/Spm,plant
Kabuli_wild_394818	1399945584	*Ca_kabuli_scaffold*	394818	G/T	Gene_Ca_21793	INTRON	Transposase,Ptta/En/Spm,plant
Kabuli_wild_394846	1399945585	*Ca_kabuli_scaffold*	394846	T/A	Gene_Ca_21793	INTRON	Transposase,Ptta/En/Spm,plant
Kabuli_wild_394867	1399945586	*Ca_kabuli_scaffold*	394867	C/T	Gene_Ca_21793	INTRON	Transposase,Ptta/En/Spm,plant
Kabuli_wild_394873	1399945587	*Ca_kabuli_scaffold*	394873	A/G	Gene_Ca_21793	INTRON	Transposase,Ptta/En/Spm,plant
Kabuli_wild_394917	1399945588	*Ca_kabuli_scaffold*	394917	C/T	Gene_Ca_21793	INTRON	Transposase,Ptta/En/Spm,plant
Kabuli_wild_394940	1399945589	*Ca_kabuli_scaffold*	394940	G/A	Gene_Ca_21793	INTRON	Transposase,Ptta/En/Spm,plant
Kabuli_wild_395017	1399945590	*Ca_kabuli_scaffold*	395017	A/T	Gene_Ca_21793	INTRON	Transposase,Ptta/En/Spm,plant
Kabuli_wild_395029	1399945591	*Ca_kabuli_scaffold*	395029	C/T	Gene_Ca_21793	INTRON	Transposase,Ptta/En/Spm,plant
Kabuli_wild_63613	1399945592	*Ca_kabuli_scaffold*	63613	A/G	NA	INTERGENIC	NA
Kabuli_wild_63620	1399945593	*Ca_kabuli_scaffold*	63620	C/T	NA	INTERGENIC	NA
Kabuli_wild_63662	1399945594	*Ca_kabuli_scaffold*	63662	T/G	NA	INTERGENIC	NA
Kabuli_wild_63669	1399945595	*Ca_kabuli_scaffold*	63669	C/T	NA	INTERGENIC	NA
Kabuli_wild_63671	1399945596	*Ca_kabuli_scaffold*	63671	T/C	NA	INTERGENIC	NA
Kabuli_wild_63703	1399945597	*Ca_kabuli_scaffold*	63703	T/C	NA	INTERGENIC	NA
Kabuli_wild_45508	1399945598	*Ca_kabuli_scaffold*	45508	C/A	NA	INTERGENIC	NA
Kabuli_wild_142678	1399945599	*Ca_kabuli_scaffold*	142678	A/C	NA	INTERGENIC	NA
Kabuli_wild_251191	1399945600	*Ca_kabuli_scaffold*	251191	C/A	NA	INTERGENIC	NA
Kabuli_wild_97229	1399945601	*Ca_kabuli_scaffold*	97229	C/T	Gene_Ca_26771	NON_SYNONYMOUS_CODING	TAFII28-likeprotein
Kabuli_wild_97358	1399945602	*Ca_kabuli_scaffold*	97358	T/C	Gene_Ca_26771	NON_SYNONYMOUS_CODING	TAFII28-likeprotein
Kabuli_wild_7825	1399945603	*Ca_kabuli_scaffold*	7825	G/A	NA	INTERGENIC	NA
Kabuli_wild_1154	1399945604	*Ca_kabuli_scaffold*	1154	C/A	Gene_Ca_24272	DOWNSTREAM	NA
Kabuli_wild_1164	1399945605	*Ca_kabuli_scaffold*	1164	C/A	Gene_Ca_24272	DOWNSTREAM	NA
Kabuli_wild_1222	1399945606	*Ca_kabuli_scaffold*	1222	G/A	Gene_Ca_24272	STOP_GAINED	NA
Kabuli_wild_1232	1399945607	*Ca_kabuli_scaffold*	1232	C/T	Gene_Ca_24272	SYNONYMOUS_CODING	NA
Kabuli_wild_1234	1399945608	*Ca_kabuli_scaffold*	1234	G/T	Gene_Ca_24272	NON_SYNONYMOUS_CODING	NA
Kabuli_wild_1240	1399945609	*Ca_kabuli_scaffold*	1240	T/G	Gene_Ca_24272	NON_SYNONYMOUS_CODING	NA
Kabuli_wild_1281	1399945610	*Ca_kabuli_scaffold*	1281	G/A	Gene_Ca_24272	NON_SYNONYMOUS_CODING	NA
Kabuli_wild_3150	1399945611	*Ca_kabuli_scaffold*	3150	C/A	NA	INTERGENIC	NA
Kabuli_wild_3159	1399945612	*Ca_kabuli_scaffold*	3159	C/A	NA	INTERGENIC	NA
Kabuli_wild_3160	1399945613	*Ca_kabuli_scaffold*	3160	C/A	NA	INTERGENIC	NA
Kabuli_wild_3166	1399945614	*Ca_kabuli_scaffold*	3166	G/T	NA	INTERGENIC	NA
Kabuli_wild_3191	1399945615	*Ca_kabuli_scaffold*	3191	G/A	NA	INTERGENIC	NA
Kabuli_wild_3219	1399945616	*Ca_kabuli_scaffold*	3219	G/T	NA	INTERGENIC	NA
Kabuli_wild_3228	1399945617	*Ca_kabuli_scaffold*	3228	C/T	NA	INTERGENIC	NA
Kabuli_wild_3236	1399945618	*Ca_kabuli_scaffold*	3236	T/G	NA	INTERGENIC	NA
Kabuli_wild_3240	1399945619	*Ca_kabuli_scaffold*	3240	G/C	NA	INTERGENIC	NA

SNP IDs	NCBI dbSNP accession IDs (NCBI_SS#)	Chromosomes	Physical positions (bp)	SNPs	Gene accession IDs	Sequence components of genome	Putative functions
Kabuli_wild_3244	1399945620	*Ca_kabuli_scaffold*	3244	G/A	NA	INTERGENIC	NA
Kabuli_wild_3248	1399945621	*Ca_kabuli_scaffold*	3248	C/T	NA	INTERGENIC	NA
Kabuli_wild_3249	1399945622	*Ca_kabuli_scaffold*	3249	T/G	NA	INTERGENIC	NA
Kabuli_wild_3297	1399945623	*Ca_kabuli_scaffold*	3297	C/T	NA	INTERGENIC	NA
Kabuli_wild_213927	1399945624	*Ca_kabuli_scaffold*	213927	A/T	Gene_Ca_24284	NON_SYNONYMOUS_CODING	Peptidase C48, SUMO/Sentrin/Ubl1
Kabuli_wild_213932	1399945625	*Ca_kabuli_scaffold*	213932	T/G	Gene_Ca_24284	NON_SYNONYMOUS_CODING	Peptidase C48, SUMO/Sentrin/Ubl1
Kabuli_wild_6852	1399945626	*Ca_kabuli_scaffold*	6852	T/C	NA	INTERGENIC	NA
Kabuli_wild_58074	1399945627	*Ca_kabuli_scaffold*	58074	T/C	NA	INTERGENIC	NA
Kabuli_wild_68233	1399945628	*Ca_kabuli_scaffold*	68233	C/T	NA	INTERGENIC	NA
Kabuli_wild_68255	1399945629	*Ca_kabuli_scaffold*	68255	G/T	NA	INTERGENIC	NA
Kabuli_wild_35084	1399945630	*Ca_kabuli_scaffold*	35084	T/A	NA	INTERGENIC	NA
Kabuli_wild_150599	1399945631	*Ca_kabuli_scaffold*	150599	C/T	Gene_Ca_25534	SYNONYMOUS_CODING	NA
Kabuli_wild_150600	1399945632	*Ca_kabuli_scaffold*	150600	C/T	Gene_Ca_25534	NON_SYNONYMOUS_CODING	NA
Kabuli_wild_150670	1399945633	*Ca_kabuli_scaffold*	150670	C/T	Gene_Ca_25534	NON_SYNONYMOUS_CODING	NA
Kabuli_wild_150672	1399945634	*Ca_kabuli_scaffold*	150672	G/A	Gene_Ca_25534	NON_SYNONYMOUS_CODING	NA
Kabuli_wild_150676	1399945635	*Ca_kabuli_scaffold*	150676	C/T	Gene_Ca_25534	NON_SYNONYMOUS_CODING	NA
Kabuli_wild_150720	1399945636	*Ca_kabuli_scaffold*	150720	G/A	Gene_Ca_25534	NON_SYNONYMOUS_CODING	NA
Kabuli_wild_150752	1399945637	*Ca_kabuli_scaffold*	150752	G/T	Gene_Ca_25534	SYNONYMOUS_CODING	NA
Kabuli_wild_150756	1399945638	*Ca_kabuli_scaffold*	150756	G/A	Gene_Ca_25534	NON_SYNONYMOUS_CODING	NA
Kabuli_wild_150757	1399945639	*Ca_kabuli_scaffold*	150757	C/T	Gene_Ca_25534	NON_SYNONYMOUS_CODING	NA
Kabuli_wild_637	1399945640	*Ca_kabuli_scaffold*	637	T/C	NA	INTERGENIC	NA
Kabuli_wild_669	1399945641	*Ca_kabuli_scaffold*	669	A/G	NA	INTERGENIC	NA
Kabuli_wild_693	1399945642	*Ca_kabuli_scaffold*	693	T/A	NA	INTERGENIC	NA
Kabuli_wild_62204	1399945643	*Ca_kabuli_scaffold*	62204	C/A	NA	INTERGENIC	NA
Kabuli_wild_62210	1399945644	*Ca_kabuli_scaffold*	62210	A/G	NA	INTERGENIC	NA
Kabuli_wild_62252	1399945645	*Ca_kabuli_scaffold*	62252	G/A	NA	INTERGENIC	NA
Kabuli_wild_62263	1399945646	*Ca_kabuli_scaffold*	62263	G/A	NA	INTERGENIC	NA
Kabuli_wild_62264	1399945647	*Ca_kabuli_scaffold*	62264	G/A	NA	INTERGENIC	NA
Kabuli_wild_87275	1399945648	*Ca_kabuli_scaffold*	87275	G/A	NA	INTERGENIC	NA
Kabuli_wild_153319	1399945649	*Ca_kabuli_scaffold*	153319	G/T	NA	INTERGENIC	NA
Kabuli_wild_153349	1399945650	*Ca_kabuli_scaffold*	153349	T/C	NA	INTERGENIC	NA
Kabuli_wild_2438	1399945651	*Ca_kabuli_scaffold*	2438	C/T	NA	INTERGENIC	NA
Kabuli_wild_2454	1399945652	*Ca_kabuli_scaffold*	2454	G/A	NA	INTERGENIC	NA
Kabuli_wild_2457	1399945653	*Ca_kabuli_scaffold*	2457	A/G	NA	INTERGENIC	NA
Kabuli_wild_2215	1399945654	*Ca_kabuli_scaffold*	2215	C/T	Gene_Ca_27403	DOWNSTREAM	NA
Kabuli_wild_2224	1399945655	*Ca_kabuli_scaffold*	2224	A/T	Gene_Ca_27403	DOWNSTREAM	NA
Kabuli_wild_2225	1399945656	*Ca_kabuli_scaffold*	2225	C/T	Gene_Ca_27403	DOWNSTREAM	NA
Kabuli_wild_2226	1399945657	*Ca_kabuli_scaffold*	2226	G/T	Gene_Ca_27403	DOWNSTREAM	NA
Kabuli_wild_2315	1399945658	*Ca_kabuli_scaffold*	2315	T/C	Gene_Ca_27403	DOWNSTREAM	NA
Kabuli_wild_9556	1399945659	*Ca_kabuli_scaffold*	9556	C/T	Gene_Ca_27404	INTRON	Inositolpolyphosphate-relatedphosphatase
Kabuli_wild_9568	1399945660	*Ca_kabuli_scaffold*	9568	A/G	Gene_Ca_27404	INTRON	Inositolpolyphosphate-relatedphosphatase
Kabuli_wild_58302	1399945661	*Ca_kabuli_scaffold*	58302	G/A	NA	INTERGENIC	NA
Kabuli_wild_58316	1399945662	*Ca_kabuli_scaffold*	58316	T/C	NA	INTERGENIC	NA
Kabuli_wild_94196	1399945663	*Ca_kabuli_scaffold*	94196	T/A	Gene_Ca_23728	INTRON	ABC transporter-like

SNP IDs	NCBI dbSNP accession IDs (NCBI_SS#)	Chromosomes	Physical positions (bp)	SNPs	Gene accession IDs	Sequence components of genome	Putative functions
Kabuli_wild_94207	1399945664	*Ca_kabuli_scaffold*	94207	A/C	Gene_Ca_23728	NON_SYNONYMOUS_CODING	ABC transporter-like
Kabuli_wild_112702	1399945665	*Ca_kabuli_scaffold*	112702	G/A	NA	INTERGENIC	NA
Kabuli_wild_341416	1399945666	*Ca_kabuli_scaffold*	341416	T/A	NA	INTERGENIC	NA
Kabuli_wild_360529	1399945667	*Ca_kabuli_scaffold*	360529	A/C	NA	INTERGENIC	NA
Kabuli_wild_360567	1399945668	*Ca_kabuli_scaffold*	360567	T/A	NA	INTERGENIC	NA
Kabuli_wild_360585	1399945669	*Ca_kabuli_scaffold*	360585	C/A	NA	INTERGENIC	NA
Kabuli_wild_423553	1399945670	*Ca_kabuli_scaffold*	423553	A/G	Gene_Ca_23740	DOWNSTREAM	Signal transduction response regulator, receiverdomain
Kabuli_wild_26492	1399945671	*Ca_kabuli_scaffold*	26492	G/A	NA	INTERGENIC	NA
Kabuli_wild_26497	1399945672	*Ca_kabuli_scaffold*	26497	C/T	NA	INTERGENIC	NA
Kabuli_wild_26508	1399945673	*Ca_kabuli_scaffold*	26508	T/G	NA	INTERGENIC	NA
Kabuli_wild_64083	1399945674	*Ca_kabuli_scaffold*	64083	A/G	NA	INTERGENIC	NA
Kabuli_wild_61220	1399945675	*Ca_kabuli_scaffold*	61220	G/A	Gene_Ca_26367	NON_SYNONYMOUS_CODING	Protein of unknown function DUF707
Kabuli_wild_227534	1399945676	*Ca_kabuli_scaffold*	227534	C/A	NA	INTERGENIC	NA
Kabuli_wild_227589	1399945677	*Ca_kabuli_scaffold*	227589	G/A	NA	INTERGENIC	NA
Kabuli_wild_228276	1399945678	*Ca_kabuli_scaffold*	228276	A/G	NA	INTERGENIC	NA
Kabuli_wild_228291	1399945679	*Ca_kabuli_scaffold*	228291	C/T	NA	INTERGENIC	NA
Kabuli_wild_228304	1399945680	*Ca_kabuli_scaffold*	228304	A/T	NA	INTERGENIC	NA
Kabuli_wild_228312	1399945681	*Ca_kabuli_scaffold*	228312	C/G	NA	INTERGENIC	NA
Kabuli_wild_228332	1399945682	*Ca_kabuli_scaffold*	228332	G/A	NA	INTERGENIC	NA
Kabuli_wild_228353	1399945683	*Ca_kabuli_scaffold*	228353	C/T	NA	INTERGENIC	NA
Kabuli_wild_228358	1399945684	*Ca_kabuli_scaffold*	228358	T/A	NA	INTERGENIC	NA
Kabuli_wild_228367	1399945685	*Ca_kabuli_scaffold*	228367	C/T	NA	INTERGENIC	NA
Kabuli_wild_137100	1399945686	*Ca_kabuli_scaffold*	137100	A/G	Gene_Ca_26895	NON_SYNONYMOUS_CODING	NA
Kabuli_wild_112348	1399945687	*Ca_kabuli_scaffold*	112348	G/A	NA	INTERGENIC	NA
Kabuli_wild_114508	1399945688	*Ca_kabuli_scaffold*	114508	C/A	Gene_Ca_25739	SYNONYMOUS_CODING	Staphylococcalnuclease(SNase-like)
Kabuli_wild_192327	1399945689	*Ca_kabuli_scaffold*	192327	G/A	NA	INTERGENIC	NA
Kabuli_wild_209057	1399945690	*Ca_kabuli_scaffold*	209057	G/T	NA	INTERGENIC	NA
Kabuli_wild_902	1399945691	*Ca_kabuli_scaffold*	902	C/T	NA	INTERGENIC	NA
Kabuli_wild_944	1399945692	*Ca_kabuli_scaffold*	944	G/A	NA	INTERGENIC	NA
Kabuli_wild_1098	1399945693	*Ca_kabuli_scaffold*	1098	T/C	NA	INTERGENIC	NA
Kabuli_wild_1110	1399945694	*Ca_kabuli_scaffold*	1110	G/A	NA	INTERGENIC	NA
Kabuli_wild_1134	1399945695	*Ca_kabuli_scaffold*	1134	T/A	NA	INTERGENIC	NA
Kabuli_wild_70490	1399945696	*Ca_kabuli_scaffold*	70490	T/C	NA	INTERGENIC	NA
Kabuli_wild_70494	1399945697	*Ca_kabuli_scaffold*	70494	A/G	NA	INTERGENIC	NA
Kabuli_wild_70496	1399945698	*Ca_kabuli_scaffold*	70496	G/A	NA	INTERGENIC	NA
Kabuli_wild_70506	1399945699	*Ca_kabuli_scaffold*	70506	G/A	NA	INTERGENIC	NA
Kabuli_wild_70526	1399945700	*Ca_kabuli_scaffold*	70526	T/C	NA	INTERGENIC	NA
Kabuli_wild_70535	1399945701	*Ca_kabuli_scaffold*	70535	G/A	NA	INTERGENIC	NA
Kabuli_wild_70544	1399945702	*Ca_kabuli_scaffold*	70544	T/A	NA	INTERGENIC	NA
Kabuli_wild_70547	1399945703	*Ca_kabuli_scaffold*	70547	G/C	NA	INTERGENIC	NA
Kabuli_wild_70549	1399945704	*Ca_kabuli_scaffold*	70549	G/A	NA	INTERGENIC	NA
Kabuli_wild_70552	1399945705	*Ca_kabuli_scaffold*	70552	A/C	NA	INTERGENIC	NA
Kabuli_wild_203004	1399945706	*Ca_kabuli_scaffold*	203004	G/A	Gene_Ca_26186	NON_SYNONYMOUS_CODING	Peptidase C48, SUMO/Sentrin/Ubl1
Kabuli_wild_203005	1399945707	*Ca_kabuli_scaffold*	203005	C/T	Gene_Ca_26186	NON_SYNONYMOUS_CODING	Peptidase C48, SUMO/Sentrin/Ubl1

SNP IDs	NCBI dbSNP accession IDs (NCBI_SS#)	Chromosomes	Physical positions (bp)	SNPs	Gene accession IDs	Sequence components of genome	Putative functions
Kabuli_wild_203081	1399945708	*Ca_kabuli_scaffold*	203081	G/C	Gene_Ca_26186	SYNONYMOUS_CODING	Peptidase C48, SUMO/Sentrin/Ubl1
Kabuli_wild_168957	1399945709	*Ca_kabuli_scaffold*	168957	A/G	NA	INTERGENIC	NA
Kabuli_wild_171308	1399945710	*Ca_kabuli_scaffold*	171308	A/C	NA	INTERGENIC	NA
Kabuli_wild_344239	1399945711	*Ca_kabuli_scaffold*	344239	A/C	NA	INTERGENIC	NA
Kabuli_wild_407641	1399945712	*Ca_kabuli_scaffold*	407641	G/A	Gene_Ca_24073	SYNONYMOUS_CODING	TGS
Kabuli_wild_407658	1399945713	*Ca_kabuli_scaffold*	407658	C/G	Gene_Ca_24073	STOP_GAINED	TGS
Kabuli_wild_407691	1399945714	*Ca_kabuli_scaffold*	407691	T/C	Gene_Ca_24073	NON_SYNONYMOUS_CODING	TGS
Kabuli_wild_407695	1399945715	*Ca_kabuli_scaffold*	407695	T/C	Gene_Ca_24073	SYNONYMOUS_CODING	TGS
Kabuli_wild_447890	1399945716	*Ca_kabuli_scaffold*	447890	C/T	NA	INTERGENIC	NA
Kabuli_wild_505051	1399945717	*Ca_kabuli_scaffold*	505051	C/T	NA	INTERGENIC	NA
Kabuli_wild_520148	1399945718	*Ca_kabuli_scaffold*	520148	A/T	NA	INTERGENIC	NA
Kabuli_wild_520153	1399945719	*Ca_kabuli_scaffold*	520153	C/G	NA	INTERGENIC	NA
Kabuli_wild_520164	1399945720	*Ca_kabuli_scaffold*	520164	T/A	NA	INTERGENIC	NA
Kabuli_wild_5074	1399945721	*Ca_kabuli_scaffold*	5074	C/T	Gene_Ca_24658	INTRON	NA
Kabuli_wild_5089	1399945722	*Ca_kabuli_scaffold*	5089	A/G	Gene_Ca_24658	INTRON	NA
Kabuli_wild_5103	1399945723	*Ca_kabuli_scaffold*	5103	G/C	Gene_Ca_24658	NON_SYNONYMOUS_CODING	NA
Kabuli_wild_5106	1399945724	*Ca_kabuli_scaffold*	5106	G/A	Gene_Ca_24658	NON_SYNONYMOUS_CODING	NA
Kabuli_wild_46904	1399945725	*Ca_kabuli_scaffold*	46904	C/T	Gene_Ca_24659	INTRON	CDP-alcoholphosphatidyltransferase
Kabuli_wild_285926	1399945726	*Ca_kabuli_scaffold*	285926	G/A	Gene_Ca_24669	SYNONYMOUS_CODING	NA
Kabuli_wild_285941	1399945727	*Ca_kabuli_scaffold*	285941	C/T	Gene_Ca_24669	NON_SYNONYMOUS_CODING	NA
Kabuli_wild_285943	1399945728	*Ca_kabuli_scaffold*	285943	G/A	Gene_Ca_24669	NON_SYNONYMOUS_CODING	NA
Kabuli_wild_285956	1399945729	*Ca_kabuli_scaffold*	285956	C/T	Gene_Ca_24669	NON_SYNONYMOUS_CODING	NA
Kabuli_wild_40741	1399945730	*Ca_kabuli_scaffold*	40741	G/T	Gene_Ca_28164	SYNONYMOUS_CODING	PeptidaseS8/S53,subtilisin/kexin/sedolisin
Kabuli_wild_40791	1399945731	*Ca_kabuli_scaffold*	40791	C/T	Gene_Ca_28164	NON_SYNONYMOUS_CODING	PeptidaseS8/S53,subtilisin/kexin/sedolisin
Kabuli_wild_40793	1399945732	*Ca_kabuli_scaffold*	40793	A/G	Gene_Ca_28164	SYNONYMOUS_CODING	PeptidaseS8/S53,subtilisin/kexin/sedolisin
Kabuli_wild_40797	1399945733	*Ca_kabuli_scaffold*	40797	A/T	Gene_Ca_28164	NON_SYNONYMOUS_CODING	PeptidaseS8/S53,subtilisin/kexin/sedolisin
Kabuli_wild_40814	1399945734	*Ca_kabuli_scaffold*	40814	T/A	Gene_Ca_28164	SYNONYMOUS_CODING	PeptidaseS8/S53,subtilisin/kexin/sedolisin
Kabuli_wild_40817	1399945735	*Ca_kabuli_scaffold*	40817	A/G	Gene_Ca_28164	SYNONYMOUS_CODING	PeptidaseS8/S53,subtilisin/kexin/sedolisin
Kabuli_wild_34789	1399945736	*Ca_kabuli_scaffold*	34789	C/T	NA	INTERGENIC	NA
Kabuli_wild_34791	1399945737	*Ca_kabuli_scaffold*	34791	G/A	NA	INTERGENIC	NA
Kabuli_wild_34799	1399945738	*Ca_kabuli_scaffold*	34799	C/T	NA	INTERGENIC	NA
Kabuli_wild_85015	1399945739	*Ca_kabuli_scaffold*	85015	A/C	Gene_Ca_27099	DOWNSTREAM	NA
Kabuli_wild_7812	1399945740	*Ca_kabuli_scaffold*	7812	G/C	NA	INTERGENIC	NA
Kabuli_wild_289	1399945741	*Ca_kabuli_scaffold*	289	G/A	NA	INTERGENIC	NA
Kabuli_wild_330	1399945742	*Ca_kabuli_scaffold*	330	T/C	NA	INTERGENIC	NA
Kabuli_wild_71428	1399945743	*Ca_kabuli_scaffold*	71428	C/T	NA	INTERGENIC	NA
Kabuli_wild_71649	1399945744	*Ca_kabuli_scaffold*	71649	C/T	NA	INTERGENIC	NA
Kabuli_wild_55971	1399945745	*Ca_kabuli_scaffold*	55971	A/G	Gene_Ca_26853	SYNONYMOUS_CODING	Glycosyltransferase,family20
Kabuli_wild_57303	1399945746	*Ca_kabuli_scaffold*	57303	C/A	Gene_Ca_26853	INTRON	Glycosyltransferase,family20
Kabuli_wild_62709	1399945747	*Ca_kabuli_scaffold*	62709	A/G	Gene_Ca_26853	INTRON	Glycosyltransferase,family20
Kabuli_wild_62711	1399945748	*Ca_kabuli_scaffold*	62711	C/T	Gene_Ca_26853	INTRON	Glycosyltransferase,family20
Kabuli_wild_62751	1399945749	*Ca_kabuli_scaffold*	62751	C/T	Gene_Ca_26853	INTRON	Glycosyltransferase,family20
Kabuli_wild_62775	1399945750	*Ca_kabuli_scaffold*	62775	T/C	Gene_Ca_26853	INTRON	Glycosyltransferase,family20
Kabuli_wild_62778	1399945751	*Ca_kabuli_scaffold*	62778	T/A	Gene_Ca_26853	INTRON	Glycosyltransferase,family20

SNP IDs	NCBI dbSNP accession IDs (NCBI_SS#)	Chromosomes	Physical positions (bp)	SNPs	Gene accession IDs	Sequence components of genome	Putative functions
Kabuli_wild_62008	1399945752	*Ca_kabuli_scaffold*	62008	G/A	NA	INTERGENIC	NA
Kabuli_wild_66173	1399945753	*Ca_kabuli_scaffold*	66173	A/T	Gene_Ca_21507	NON_SYNONYMOUS_CODING	Aminotransferase-like,plant mobile domain
Kabuli_wild_66208	1399945754	*Ca_kabuli_scaffold*	66208	G/C	Gene_Ca_21507	NON_SYNONYMOUS_CODING	Aminotransferase-like,plant mobile domain
Kabuli_wild_150505	1399945755	*Ca_kabuli_scaffold*	150505	G/A	NA	INTERGENIC	NA
Kabuli_wild_244153	1399945756	*Ca_kabuli_scaffold*	244153	C/A	Gene_Ca_21517	NON_SYNONYMOUS_CODING	Protein kinase,catalytic domain
Kabuli_wild_316037	1399945757	*Ca_kabuli_scaffold*	316037	A/G	NA	INTERGENIC	NA
Kabuli_wild_398729	1399945758	*Ca_kabuli_scaffold*	398729	T/G	Gene_Ca_21525	NON_SYNONYMOUS_CODING	Armadillo
Kabuli_wild_463286	1399945759	*Ca_kabuli_scaffold*	463286	C/T	NA	INTERGENIC	NA
Kabuli_wild_526442	1399945760	*Ca_kabuli_scaffold*	526442	T/C	NA	INTERGENIC	NA
Kabuli_wild_526446	1399945761	*Ca_kabuli_scaffold*	526446	C/T	NA	INTERGENIC	NA
Kabuli_wild_527008	1399945762	*Ca_kabuli_scaffold*	527008	T/C	NA	INTERGENIC	NA
Kabuli_wild_527012	1399945763	*Ca_kabuli_scaffold*	527012	C/T	NA	INTERGENIC	NA
Kabuli_wild_48753	1399945764	*Ca_kabuli_scaffold*	48753	G/A	NA	INTERGENIC	NA
Kabuli_wild_68241	1399945765	*Ca_kabuli_scaffold*	68241	G/C	Gene_Ca_24805	SYNONYMOUS_CODING	Peptidase C48, SUMO/Sentrin/Ubl1
Kabuli_wild_68258	1399945766	*Ca_kabuli_scaffold*	68258	A/T	Gene_Ca_24805	NON_SYNONYMOUS_CODING	Peptidase C48, SUMO/Sentrin/Ubl1
Kabuli_wild_68264	1399945767	*Ca_kabuli_scaffold*	68264	C/T	Gene_Ca_24805	SYNONYMOUS_CODING	Peptidase C48, SUMO/Sentrin/Ubl1
Kabuli_wild_68275	1399945768	*Ca_kabuli_scaffold*	68275	T/C	Gene_Ca_24805	SYNONYMOUS_CODING	Peptidase C48, SUMO/Sentrin/Ubl1
Kabuli_wild_68284	1399945769	*Ca_kabuli_scaffold*	68284	C/A	Gene_Ca_24805	NON_SYNONYMOUS_CODING	Peptidase C48, SUMO/Sentrin/Ubl1
Kabuli_wild_68290	1399945770	*Ca_kabuli_scaffold*	68290	C/A	Gene_Ca_24805	NON_SYNONYMOUS_CODING	Peptidase C48, SUMO/Sentrin/Ubl1
Kabuli_wild_68311	1399945771	*Ca_kabuli_scaffold*	68311	G/A	Gene_Ca_24805	SYNONYMOUS_CODING	Peptidase C48, SUMO/Sentrin/Ubl1
Kabuli_wild_68324	1399945772	*Ca_kabuli_scaffold*	68324	T/C	Gene_Ca_24805	INTRON	Peptidase C48, SUMO/Sentrin/Ubl1
Kabuli_wild_68327	1399945773	*Ca_kabuli_scaffold*	68327	G/T	Gene_Ca_24805	INTRON	Peptidase C48, SUMO/Sentrin/Ubl1
Kabuli_wild_68330	1399945774	*Ca_kabuli_scaffold*	68330	G/A	Gene_Ca_24805	INTRON	Peptidase C48, SUMO/Sentrin/Ubl1
Kabuli_wild_68354	1399945775	*Ca_kabuli_scaffold*	68354	G/A	Gene_Ca_24805	INTRON	Peptidase C48, SUMO/Sentrin/Ubl1
Kabuli_wild_68359	1399945776	*Ca_kabuli_scaffold*	68359	T/C	Gene_Ca_24805	INTRON	Peptidase C48, SUMO/Sentrin/Ubl1
Kabuli_wild_96310	1399945777	*Ca_kabuli_scaffold*	96310	A/C	NA	INTERGENIC	NA
Kabuli_wild_96358	1399945778	*Ca_kabuli_scaffold*	96358	T/C	NA	INTERGENIC	NA
Kabuli_wild_96399	1399945779	*Ca_kabuli_scaffold*	96399	C/T	NA	INTERGENIC	NA
Kabuli_wild_96409	1399945780	*Ca_kabuli_scaffold*	96409	C/T	NA	INTERGENIC	NA
Kabuli_wild_96411	1399945781	*Ca_kabuli_scaffold*	96411	G/A	NA	INTERGENIC	NA
Kabuli_wild_96414	1399945782	*Ca_kabuli_scaffold*	96414	C/T	NA	INTERGENIC	NA
Kabuli_wild_96428	1399945783	*Ca_kabuli_scaffold*	96428	G/A	NA	INTERGENIC	NA
Kabuli_wild_96429	1399945784	*Ca_kabuli_scaffold*	96429	C/T	NA	INTERGENIC	NA
Kabuli_wild_105504	1399945785	*Ca_kabuli_scaffold*	105504	G/A	NA	INTERGENIC	NA
Kabuli_wild_105507	1399945786	*Ca_kabuli_scaffold*	105507	A/T	NA	INTERGENIC	NA
Kabuli_wild_105553	1399945787	*Ca_kabuli_scaffold*	105553	T/C	NA	INTERGENIC	NA
Kabuli_wild_192068	1399945788	*Ca_kabuli_scaffold*	192068	T/G	NA	INTERGENIC	NA
Kabuli_wild_240723	1399945789	*Ca_kabuli_scaffold*	240723	C/T	Gene_Ca_24811	INTRON	NA
Kabuli_wild_240730	1399945790	*Ca_kabuli_scaffold*	240730	T/C	Gene_Ca_24811	INTRON	NA
Kabuli_wild_240735	1399945791	*Ca_kabuli_scaffold*	240735	G/A	Gene_Ca_24811	INTRON	NA
Kabuli_wild_240749	1399945792	*Ca_kabuli_scaffold*	240749	G/T	Gene_Ca_24811	INTRON	NA
Kabuli_wild_240762	1399945793	*Ca_kabuli_scaffold*	240762	C/T	Gene_Ca_24811	INTRON	NA
Kabuli_wild_44260	1399945794	*Ca_kabuli_scaffold*	44260	A/G	NA	INTERGENIC	NA
Kabuli_wild_24166	1399945795	*Ca_kabuli_scaffold*	24166	G/A	NA	INTERGENIC	NA

SNP IDs	NCBI dbSNP accession IDs (NCBI_SS#)	Chromosomes	Physical positions (bp)	SNPs	Gene accession IDs	Sequence components of genome	Putative functions
Kabuli_wild_49259	1399945796	*Ca_kabuli_scaffold*	49259	G/A	Gene_Ca_26762	SYNONYMOUS_CODING	Transposase,Ptta/En/Spm,plant
Kabuli_wild_51835	1399945797	*Ca_kabuli_scaffold*	51835	T/C	Gene_Ca_26762	DOWNSTREAM	Transposase,Ptta/En/Spm,plant
Kabuli_wild_98343	1399945798	*Ca_kabuli_scaffold*	98343	G/A	NA	INTERGENIC	NA
Kabuli_wild_98399	1399945799	*Ca_kabuli_scaffold*	98399	C/T	NA	INTERGENIC	NA
Kabuli_wild_98400	1399945800	*Ca_kabuli_scaffold*	98400	T/C	NA	INTERGENIC	NA
Kabuli_wild_98402	1399945801	*Ca_kabuli_scaffold*	98402	T/C	NA	INTERGENIC	NA
Kabuli_wild_18173	1399945802	*Ca_kabuli_scaffold*	18173	A/G	Gene_Ca_25873	INTRON	Homeobox
Kabuli_wild_124337	1399945803	*Ca_kabuli_scaffold*	124337	G/C	Gene_Ca_25878	NON_SYNONYMOUS_CODING	Peptidase C48, SUMO/Sentrin/Ubl1
Kabuli_wild_124340	1399945804	*Ca_kabuli_scaffold*	124340	G/A	Gene_Ca_25878	NON_SYNONYMOUS_CODING	Peptidase C48, SUMO/Sentrin/Ubl1
Kabuli_wild_124367	1399945805	*Ca_kabuli_scaffold*	124367	C/T	Gene_Ca_25878	NON_SYNONYMOUS_CODING	Peptidase C48, SUMO/Sentrin/Ubl1
Kabuli_wild_124377	1399945806	*Ca_kabuli_scaffold*	124377	A/T	Gene_Ca_25878	NON_SYNONYMOUS_CODING	Peptidase C48, SUMO/Sentrin/Ubl1
Kabuli_wild_124380	1399945807	*Ca_kabuli_scaffold*	124380	G/A	Gene_Ca_25878	NON_SYNONYMOUS_CODING	Peptidase C48, SUMO/Sentrin/Ubl1
Kabuli_wild_124383	1399945808	*Ca_kabuli_scaffold*	124383	C/A	Gene_Ca_25878	NON_SYNONYMOUS_CODING	Peptidase C48, SUMO/Sentrin/Ubl1
Kabuli_wild_227729	1399945809	*Ca_kabuli_scaffold*	227729	G/A	Gene_Ca_25880	NON_SYNONYMOUS_CODING	Peptidase C48, SUMO/Sentrin/Ubl1
Kabuli_wild_227730	1399945810	*Ca_kabuli_scaffold*	227730	C/T	Gene_Ca_25880	NON_SYNONYMOUS_CODING	Peptidase C48, SUMO/Sentrin/Ubl1
Kabuli_wild_227736	1399945811	*Ca_kabuli_scaffold*	227736	C/T	Gene_Ca_25880	NON_SYNONYMOUS_CODING	Peptidase C48, SUMO/Sentrin/Ubl1
Kabuli_wild_227768	1399945812	*Ca_kabuli_scaffold*	227768	G/A	Gene_Ca_25880	NON_SYNONYMOUS_CODING	Peptidase C48, SUMO/Sentrin/Ubl1
Kabuli_wild_227777	1399945813	*Ca_kabuli_scaffold*	227777	G/A	Gene_Ca_25880	NON_SYNONYMOUS_CODING	Peptidase C48, SUMO/Sentrin/Ubl1
Kabuli_wild_227796	1399945814	*Ca_kabuli_scaffold*	227796	G/A	Gene_Ca_25880	SYNONYMOUS_CODING	Peptidase C48, SUMO/Sentrin/Ubl1
Kabuli_wild_227797	1399945815	*Ca_kabuli_scaffold*	227797	C/G	Gene_Ca_25880	NON_SYNONYMOUS_CODING	Peptidase C48, SUMO/Sentrin/Ubl1
Kabuli_wild_227811	1399945816	*Ca_kabuli_scaffold*	227811	C/T	Gene_Ca_25880	NON_SYNONYMOUS_CODING	Peptidase C48, SUMO/Sentrin/Ubl1
Kabuli_wild_227813	1399945817	*Ca_kabuli_scaffold*	227813	G/T	Gene_Ca_25880	NON_SYNONYMOUS_CODING	Peptidase C48, SUMO/Sentrin/Ubl1
Kabuli_wild_115446	1399945818	*Ca_kabuli_scaffold*	115446	C/A	NA	INTERGENIC	NA
Kabuli_wild_115471	1399945819	*Ca_kabuli_scaffold*	115471	G/A	NA	INTERGENIC	NA
Kabuli_wild_115508	1399945820	*Ca_kabuli_scaffold*	115508	C/T	NA	INTERGENIC	NA
Kabuli_wild_228151	1399945821	*Ca_kabuli_scaffold*	228151	G/A	Gene_Ca_19765	SYNONYMOUS_CODING	NA
Kabuli_wild_228159	1399945822	*Ca_kabuli_scaffold*	228159	A/C	Gene_Ca_19765	NON_SYNONYMOUS_CODING	NA
Kabuli_wild_262208	1399945823	*Ca_kabuli_scaffold*	262208	C/G	Gene_Ca_19766	NON_SYNONYMOUS_CODING	NA
Kabuli_wild_262541	1399945824	*Ca_kabuli_scaffold*	262541	A/T	Gene_Ca_19766	NON_SYNONYMOUS_CODING	NA
Kabuli_wild_541844	1399945825	*Ca_kabuli_scaffold*	541844	G/A	NA	INTERGENIC	NA
Kabuli_wild_24599	1399945826	*Ca_kabuli_scaffold*	24599	C/T	NA	INTERGENIC	NA
Kabuli_wild_42146	1399945827	*Ca_kabuli_scaffold*	42146	C/T	Gene_Ca_21984	DOWNSTREAM	NA
Kabuli_wild_42192	1399945828	*Ca_kabuli_scaffold*	42192	C/T	Gene_Ca_21984	DOWNSTREAM	NA
Kabuli_wild_172084	1399945829	*Ca_kabuli_scaffold*	172084	G/C	NA	INTERGENIC	NA
Kabuli_wild_177145	1399945830	*Ca_kabuli_scaffold*	177145	C/G	NA	INTERGENIC	NA
Kabuli_wild_177159	1399945831	*Ca_kabuli_scaffold*	177159	G/A	NA	INTERGENIC	NA
Kabuli_wild_177171	1399945832	*Ca_kabuli_scaffold*	177171	T/G	NA	INTERGENIC	NA
Kabuli_wild_177172	1399945833	*Ca_kabuli_scaffold*	177172	G/A	NA	INTERGENIC	NA
Kabuli_wild_177178	1399945834	*Ca_kabuli_scaffold*	177178	T/C	NA	INTERGENIC	NA
Kabuli_wild_177182	1399945835	*Ca_kabuli_scaffold*	177182	C/T	NA	INTERGENIC	NA
Kabuli_wild_177188	1399945836	*Ca_kabuli_scaffold*	177188	G/T	NA	INTERGENIC	NA
Kabuli_wild_177191	1399945837	*Ca_kabuli_scaffold*	177191	C/T	NA	INTERGENIC	NA
Kabuli_wild_177192	1399945838	*Ca_kabuli_scaffold*	177192	G/A	NA	INTERGENIC	NA
Kabuli_wild_177193	1399945839	*Ca_kabuli_scaffold*	177193	T/C	NA	INTERGENIC	NA

SNP IDs	NCBI dbSNP accession IDs (NCBI_SS#)	Chromosomes	Physical positions (bp)	SNPs	Gene accession IDs	Sequence components of genome	Putative functions
Kabuli_wild_177200	1399945840	*Ca_kabuli_scaffold*	177200	T/G	NA	INTERGENIC	NA
Kabuli_wild_177210	1399945841	*Ca_kabuli_scaffold*	177210	T/A	NA	INTERGENIC	NA
Kabuli_wild_177212	1399945842	*Ca_kabuli_scaffold*	177212	C/A	NA	INTERGENIC	NA
Kabuli_wild_177219	1399945843	*Ca_kabuli_scaffold*	177219	T/G	NA	INTERGENIC	NA
Kabuli_wild_177234	1399945844	*Ca_kabuli_scaffold*	177234	A/C	NA	INTERGENIC	NA
Kabuli_wild_177235	1399945845	*Ca_kabuli_scaffold*	177235	G/T	NA	INTERGENIC	NA
Kabuli_wild_177240	1399945846	*Ca_kabuli_scaffold*	177240	C/A	NA	INTERGENIC	NA
Kabuli_wild_177241	1399945847	*Ca_kabuli_scaffold*	177241	T/G	NA	INTERGENIC	NA
Kabuli_wild_177242	1399945848	*Ca_kabuli_scaffold*	177242	G/A	NA	INTERGENIC	NA
Kabuli_wild_177247	1399945849	*Ca_kabuli_scaffold*	177247	G/A	NA	INTERGENIC	NA
Kabuli_wild_177248	1399945850	*Ca_kabuli_scaffold*	177248	G/T	NA	INTERGENIC	NA
Kabuli_wild_177256	1399945851	*Ca_kabuli_scaffold*	177256	T/G	NA	INTERGENIC	NA
Kabuli_wild_182067	1399945852	*Ca_kabuli_scaffold*	182067	C/G	NA	INTERGENIC	NA
Kabuli_wild_204656	1399945853	*Ca_kabuli_scaffold*	204656	C/A	Gene_Ca_21998	DOWNSTREAM	RibosomalproteinS9
Kabuli_wild_204659	1399945854	*Ca_kabuli_scaffold*	204659	C/A	Gene_Ca_21998	DOWNSTREAM	RibosomalproteinS9
Kabuli_wild_207153	1399945855	*Ca_kabuli_scaffold*	207153	T/C	Gene_Ca_21998	INTRON	RibosomalproteinS9
Kabuli_wild_335227	1399945856	*Ca_kabuli_scaffold*	335227	T/C	Gene_Ca_22005	SYNONYMOUS_CODING	NA
Kabuli_wild_335232	1399945857	*Ca_kabuli_scaffold*	335232	G/T	Gene_Ca_22005	SYNONYMOUS_CODING	NA
Kabuli_wild_335239	1399945858	*Ca_kabuli_scaffold*	335239	G/T	Gene_Ca_22005	NON_SYNONYMOUS_CODING	NA
Kabuli_wild_366828	1399945859	*Ca_kabuli_scaffold*	366828	C/T	Gene_Ca_22006	INTRON	AldehydedehydrogenaseNAD(P)-dependent
Kabuli_wild_366846	1399945860	*Ca_kabuli_scaffold*	366846	T/C	Gene_Ca_22006	INTRON	AldehydedehydrogenaseNAD(P)-dependent
Kabuli_wild_367576	1399945861	*Ca_kabuli_scaffold*	367576	A/G	Gene_Ca_22006	UPSTREAM	AldehydedehydrogenaseNAD(P)-dependent
Kabuli_wild_367578	1399945862	*Ca_kabuli_scaffold*	367578	G/A	Gene_Ca_22006	UPSTREAM	AldehydedehydrogenaseNAD(P)-dependent
Kabuli_wild_367745	1399945863	*Ca_kabuli_scaffold*	367745	G/A	Gene_Ca_22006	UPSTREAM	AldehydedehydrogenaseNAD(P)-dependent
Kabuli_wild_8115	1399945864	*Ca_kabuli_scaffold*	8115	T/G	NA	INTERGENIC	NA
Kabuli_wild_8154	1399945865	*Ca_kabuli_scaffold*	8154	T/G	NA	INTERGENIC	NA
Kabuli_wild_8221	1399945866	*Ca_kabuli_scaffold*	8221	T/G	NA	INTERGENIC	NA
Kabuli_wild_31000	1399945867	*Ca_kabuli_scaffold*	31000	A/G	Gene_Ca_25520	UPSTREAM	Protein of unknown function DUF617,plant
Kabuli_wild_31086	1399945868	*Ca_kabuli_scaffold*	31086	A/C	Gene_Ca_25520	UPSTREAM	Protein of unknown function DUF617,plant
Kabuli_wild_31087	1399945869	*Ca_kabuli_scaffold*	31087	G/A	Gene_Ca_25520	UPSTREAM	Protein of unknown function DUF617,plant
Kabuli_wild_210315	1399945870	*Ca_kabuli_scaffold*	210315	C/T	NA	INTERGENIC	NA
Kabuli_wild_52743	1399945871	*Ca_kabuli_scaffold*	52743	A/C	Gene_Ca_26838	NON_SYNONYMOUS_CODING	Domain of unknown function DUF641,plant
Kabuli_wild_62434	1399945872	*Ca_kabuli_scaffold*	62434	G/A	NA	INTERGENIC	NA
Kabuli_wild_62506	1399945873	*Ca_kabuli_scaffold*	62506	G/A	NA	INTERGENIC	NA
Kabuli_wild_69648	1399945874	*Ca_kabuli_scaffold*	69648	A/C	NA	INTERGENIC	NA
Kabuli_wild_251505	1399945875	*Ca_kabuli_scaffold*	251505	G/C	NA	INTERGENIC	NA
Kabuli_wild_251520	1399945876	*Ca_kabuli_scaffold*	251520	C/G	NA	INTERGENIC	NA
Kabuli_wild_251544	1399945877	*Ca_kabuli_scaffold*	251544	G/T	NA	INTERGENIC	NA
Kabuli_wild_98354	1399945878	*Ca_kabuli_scaffold*	98354	A/G	NA	INTERGENIC	NA
Kabuli_wild_164	1399945879	*Ca_kabuli_scaffold*	164	G/A	NA	INTERGENIC	NA
Kabuli_wild_172	1399945880	*Ca_kabuli_scaffold*	172	T/A	NA	INTERGENIC	NA
Kabuli_wild_178	1399945881	*Ca_kabuli_scaffold*	178	G/T	NA	INTERGENIC	NA
Kabuli_wild_198	1399945882	*Ca_kabuli_scaffold*	198	G/A	NA	INTERGENIC	NA
Kabuli_wild_145885	1399945883	*Ca_kabuli_scaffold*	145885	G/T	NA	INTERGENIC	NA

SNP IDs	NCBI dbSNP accession IDs (NCBI_SS#)	Chromosomes	Physical positions (bp)	SNPs	Gene accession IDs	Sequence components of genome	Putative functions
Kabuli_wild_149312	1399945884	*Ca_kabuli_scaffold*	149312	T/A	NA	INTERGENIC	NA
Kabuli_wild_149323	1399945885	*Ca_kabuli_scaffold*	149323	T/A	NA	INTERGENIC	NA
Kabuli_wild_149324	1399945886	*Ca_kabuli_scaffold*	149324	C/A	NA	INTERGENIC	NA
Kabuli_wild_149325	1399945887	*Ca_kabuli_scaffold*	149325	T/C	NA	INTERGENIC	NA
Kabuli_wild_149337	1399945888	*Ca_kabuli_scaffold*	149337	T/A	NA	INTERGENIC	NA
Kabuli_wild_4906	1399945889	*Ca_kabuli_scaffold*	4906	G/A	Gene_Ca_25454	STOP_GAINED	NA
Kabuli_wild_4917	1399945890	*Ca_kabuli_scaffold*	4917	G/T	Gene_Ca_25454	NON_SYNONYMOUS_CODING	NA
Kabuli_wild_76627	1399945891	*Ca_kabuli_scaffold*	76627	A/C	NA	INTERGENIC	NA
Kabuli_wild_230210	1399945892	*Ca_kabuli_scaffold*	230210	T/G	NA	INTERGENIC	NA
Kabuli_wild_257838	1399945893	*Ca_kabuli_scaffold*	257838	T/C	Gene_Ca_25461	SYNONYMOUS_CODING	NA
Kabuli_wild_268825	1399945894	*Ca_kabuli_scaffold*	268825	A/T	NA	INTERGENIC	NA
Kabuli_wild_310741	1399945895	*Ca_kabuli_scaffold*	310741	G/A	NA	INTERGENIC	NA
Kabuli_wild_310749	1399945896	*Ca_kabuli_scaffold*	310749	A/T	NA	INTERGENIC	NA
Kabuli_wild_310757	1399945897	*Ca_kabuli_scaffold*	310757	G/T	NA	INTERGENIC	NA
Kabuli_wild_24845	1399945898	*Ca_kabuli_scaffold*	24845	C/T	NA	INTERGENIC	NA
Kabuli_wild_16647	1399945899	*Ca_kabuli_scaffold*	16647	T/C	NA	INTERGENIC	NA
Kabuli_wild_11275	1399945900	*Ca_kabuli_scaffold*	11275	T/C	NA	INTERGENIC	NA
Kabuli_wild_66318	1399945901	*Ca_kabuli_scaffold*	66318	T/C	Gene_Ca_23648	NON_SYNONYMOUS_CODING	NA
Kabuli_wild_142273	1399945902	*Ca_kabuli_scaffold*	142273	A/G	NA	INTERGENIC	NA
Kabuli_wild_242972	1399945903	*Ca_kabuli_scaffold*	242972	A/G	Gene_Ca_23658	SYNONYMOUS_CODING	NA
Kabuli_wild_47306	1399945904	*Ca_kabuli_scaffold*	47306	A/G	Gene_Ca_26687	INTRON	Importin-beta,N-terminal
Kabuli_wild_70354	1399945905	*Ca_kabuli_scaffold*	70354	T/C	Gene_Ca_26687	INTRON	Importin-beta,N-terminal
Kabuli_wild_74434	1399945906	*Ca_kabuli_scaffold*	74434	C/T	Gene_Ca_26687	INTRON	Importin-beta,N-terminal
Kabuli_wild_74436	1399945907	*Ca_kabuli_scaffold*	74436	C/T	Gene_Ca_26687	INTRON	Importin-beta,N-terminal
Kabuli_wild_74529	1399945908	*Ca_kabuli_scaffold*	74529	C/T	Gene_Ca_26687	INTRON	Importin-beta,N-terminal
Kabuli_wild_76809	1399945909	*Ca_kabuli_scaffold*	76809	G/A	Gene_Ca_26687	INTRON	Importin-beta,N-terminal
Kabuli_wild_55466	1399945910	*Ca_kabuli_scaffold*	55466	C/T	Gene_Ca_28129	SYNONYMOUS_CODING	NA
Kabuli_wild_55474	1399945911	*Ca_kabuli_scaffold*	55474	T/G	Gene_Ca_28129	SYNONYMOUS_CODING	NA
Kabuli_wild_55532	1399945912	*Ca_kabuli_scaffold*	55532	G/A	Gene_Ca_28129	NON_SYNONYMOUS_CODING	NA
Kabuli_wild_55533	1399945913	*Ca_kabuli_scaffold*	55533	C/T	Gene_Ca_28129	NON_SYNONYMOUS_CODING	NA
Kabuli_wild_3495	1399945914	*Ca_kabuli_scaffold*	3495	C/A	NA	INTERGENIC	NA
Kabuli_wild_3496	1399945915	*Ca_kabuli_scaffold*	3496	A/T	NA	INTERGENIC	NA
Kabuli_wild_3501	1399945916	*Ca_kabuli_scaffold*	3501	C/A	NA	INTERGENIC	NA
Kabuli_wild_3737	1399945917	*Ca_kabuli_scaffold*	3737	C/A	NA	INTERGENIC	NA
Kabuli_wild_1556	1399945918	*Ca_kabuli_scaffold*	1556	G/T	Gene_Ca_28230	INTRON	Protein kinase,catalytic domain
Kabuli_wild_86080	1399945919	*Ca_kabuli_scaffold*	86080	T/C	Gene_Ca_24032	DOWNSTREAM	NA
Kabuli_wild_86099	1399945920	*Ca_kabuli_scaffold*	86099	G/C	Gene_Ca_24032	DOWNSTREAM	NA
Kabuli_wild_86112	1399945921	*Ca_kabuli_scaffold*	86112	G/T	Gene_Ca_24032	DOWNSTREAM	NA
Kabuli_wild_86117	1399945922	*Ca_kabuli_scaffold*	86117	G/T	Gene_Ca_24032	DOWNSTREAM	NA
Kabuli_wild_86127	1399945923	*Ca_kabuli_scaffold*	86127	C/T	Gene_Ca_24032	DOWNSTREAM	NA
Kabuli_wild_90835	1399945924	*Ca_kabuli_scaffold*	90835	T/A	NA	INTERGENIC	NA
Kabuli_wild_90846	1399945925	*Ca_kabuli_scaffold*	90846	C/A	NA	INTERGENIC	NA
Kabuli_wild_200109	1399945926	*Ca_kabuli_scaffold*	200109	A/C	Gene_Ca_24038	INTRON	Protein kinase,catalytic domain
Kabuli_wild_322130	1399945927	*Ca_kabuli_scaffold*	322130	T/A	NA	INTERGENIC	NA

SNP IDs	NCBI dbSNP accession IDs (NCBI_SS#)	Chromosomes	Physical positions (bp)	SNPs	Gene accession IDs	Sequence components of genome	Putative functions
Kabuli_wild_322287	1399945928	*Ca_kabuli_scaffold*	322287	G/A	NA	INTERGENIC	NA
Kabuli_wild_322289	1399945929	*Ca_kabuli_scaffold*	322289	C/T	NA	INTERGENIC	NA
Kabuli_wild_322315	1399945930	*Ca_kabuli_scaffold*	322315	G/T	NA	INTERGENIC	NA
Kabuli_wild_322383	1399945931	*Ca_kabuli_scaffold*	322383	C/T	NA	INTERGENIC	NA
Kabuli_wild_322390	1399945932	*Ca_kabuli_scaffold*	322390	G/A	NA	INTERGENIC	NA
Kabuli_wild_322400	1399945933	*Ca_kabuli_scaffold*	322400	C/T	NA	INTERGENIC	NA
Kabuli_wild_322414	1399945934	*Ca_kabuli_scaffold*	322414	G/A	NA	INTERGENIC	NA
Kabuli_wild_322415	1399945935	*Ca_kabuli_scaffold*	322415	G/A	NA	INTERGENIC	NA
Kabuli_wild_322446	1399945936	*Ca_kabuli_scaffold*	322446	C/T	NA	INTERGENIC	NA
Kabuli_wild_322448	1399945937	*Ca_kabuli_scaffold*	322448	T/G	NA	INTERGENIC	NA
Kabuli_wild_322450	1399945938	*Ca_kabuli_scaffold*	322450	G/T	NA	INTERGENIC	NA
Kabuli_wild_322456	1399945939	*Ca_kabuli_scaffold*	322456	G/A	NA	INTERGENIC	NA
Kabuli_wild_25045	1399945940	*Ca_kabuli_scaffold*	25045	T/G	Gene_Ca_26808	NON_SYNONYMOUS_CODING	NA
Kabuli_wild_205803	1399945941	*Ca_kabuli_scaffold*	205803	C/T	NA	INTERGENIC	NA
Kabuli_wild_205830	1399945942	*Ca_kabuli_scaffold*	205830	G/A	NA	INTERGENIC	NA
Kabuli_wild_205833	1399945943	*Ca_kabuli_scaffold*	205833	G/A	NA	INTERGENIC	NA
Kabuli_wild_205845	1399945944	*Ca_kabuli_scaffold*	205845	A/G	NA	INTERGENIC	NA
Kabuli_wild_205863	1399945945	*Ca_kabuli_scaffold*	205863	G/A	NA	INTERGENIC	NA
Kabuli_wild_206035	1399945946	*Ca_kabuli_scaffold*	206035	T/C	NA	INTERGENIC	NA
Kabuli_wild_206067	1399945947	*Ca_kabuli_scaffold*	206067	T/G	NA	INTERGENIC	NA
Kabuli_wild_206073	1399945948	*Ca_kabuli_scaffold*	206073	G/A	NA	INTERGENIC	NA
Kabuli_wild_206085	1399945949	*Ca_kabuli_scaffold*	206085	T/C	NA	INTERGENIC	NA
Kabuli_wild_206094	1399945950	*Ca_kabuli_scaffold*	206094	T/C	NA	INTERGENIC	NA
Kabuli_wild_206100	1399945951	*Ca_kabuli_scaffold*	206100	G/A	NA	INTERGENIC	NA
Kabuli_wild_206109	1399945952	*Ca_kabuli_scaffold*	206109	G/C	NA	INTERGENIC	NA
Kabuli_wild_206118	1399945953	*Ca_kabuli_scaffold*	206118	C/T	NA	INTERGENIC	NA
Kabuli_wild_206121	1399945954	*Ca_kabuli_scaffold*	206121	G/A	NA	INTERGENIC	NA
Kabuli_wild_4749	1399945955	*Ca_kabuli_scaffold*	4749	C/T	NA	INTERGENIC	NA
Kabuli_wild_4754	1399945956	*Ca_kabuli_scaffold*	4754	C/T	NA	INTERGENIC	NA
Kabuli_wild_4755	1399945957	*Ca_kabuli_scaffold*	4755	T/A	NA	INTERGENIC	NA
Kabuli_wild_4794	1399945958	*Ca_kabuli_scaffold*	4794	C/A	NA	INTERGENIC	NA
Kabuli_wild_4796	1399945959	*Ca_kabuli_scaffold*	4796	C/T	NA	INTERGENIC	NA
Kabuli_wild_4811	1399945960	*Ca_kabuli_scaffold*	4811	A/C	NA	INTERGENIC	NA
Kabuli_wild_4820	1399945961	*Ca_kabuli_scaffold*	4820	G/A	NA	INTERGENIC	NA
Kabuli_wild_4845	1399945962	*Ca_kabuli_scaffold*	4845	A/T	NA	INTERGENIC	NA
Kabuli_wild_4854	1399945963	*Ca_kabuli_scaffold*	4854	C/T	NA	INTERGENIC	NA
Kabuli_wild_56686	1399945964	*Ca_kabuli_scaffold*	56686	A/T	NA	INTERGENIC	NA
Kabuli_wild_70785	1399945965	*Ca_kabuli_scaffold*	70785	C/T	Gene_Ca_26256	INTRON	Pentatrico peptide repeat
Kabuli_wild_81706	1399945966	*Ca_kabuli_scaffold*	81706	C/T	Gene_Ca_26514	INTRON	Peptidase C48, SUMO/Sentrin/Ubl1
Kabuli_wild_81713	1399945967	*Ca_kabuli_scaffold*	81713	C/T	Gene_Ca_26514	INTRON	Peptidase C48, SUMO/Sentrin/Ubl1
Kabuli_wild_81740	1399945968	*Ca_kabuli_scaffold*	81740	C/T	Gene_Ca_26514	INTRON	Peptidase C48, SUMO/Sentrin/Ubl1
Kabuli_wild_81784	1399945969	*Ca_kabuli_scaffold*	81784	C/A	Gene_Ca_26514	INTRON	Peptidase C48, SUMO/Sentrin/Ubl1
Kabuli_wild_81788	1399945970	*Ca_kabuli_scaffold*	81788	A/C	Gene_Ca_26514	INTRON	Peptidase C48, SUMO/Sentrin/Ubl1
Kabuli_wild_81789	1399945971	*Ca_kabuli_scaffold*	81789	A/G	Gene_Ca_26514	INTRON	Peptidase C48, SUMO/Sentrin/Ubl1

SNP IDs	NCBI dbSNP accession IDs (NCBI_SS#)	Chromosomes	Physical positions (bp)	SNPs	Gene accession IDs	Sequence components of genome	Putative functions
Kabuli_wild_81811	1399945972	*Ca_kabuli_scaffold*	81811	A/G	Gene_Ca_26514	INTRON	Peptidase C48, SUMO/Sentrin/Ubl1
Kabuli_wild_81851	1399945973	*Ca_kabuli_scaffold*	81851	G/C	Gene_Ca_26514	INTRON	Peptidase C48, SUMO/Sentrin/Ubl1
Kabuli_wild_81873	1399945974	*Ca_kabuli_scaffold*	81873	C/A	Gene_Ca_26514	INTRON	Peptidase C48, SUMO/Sentrin/Ubl1
Kabuli_wild_81880	1399945975	*Ca_kabuli_scaffold*	81880	C/T	Gene_Ca_26514	INTRON	Peptidase C48, SUMO/Sentrin/Ubl1
Kabuli_wild_81885	1399945976	*Ca_kabuli_scaffold*	81885	A/G	Gene_Ca_26514	INTRON	Peptidase C48, SUMO/Sentrin/Ubl1
Kabuli_wild_81889	1399945977	*Ca_kabuli_scaffold*	81889	T/G	Gene_Ca_26514	INTRON	Peptidase C48, SUMO/Sentrin/Ubl1
Kabuli_wild_289997	1399945978	*Ca_kabuli_scaffold*	289997	G/T	NA	INTERGENIC	NA
Kabuli_wild_290018	1399945979	*Ca_kabuli_scaffold*	290018	C/A	NA	INTERGENIC	NA
Kabuli_wild_3474	1399945980	*Ca_kabuli_scaffold*	3474	A/C	Gene_Ca_28063	SYNONYMOUS_CODING	Multiantimicrobial extrusion protein
Kabuli_wild_3641	1399945981	*Ca_kabuli_scaffold*	3641	T/C	Gene_Ca_28063	INTRON	Multiantimicrobial extrusion protein
Kabuli_wild_3675	1399945982	*Ca_kabuli_scaffold*	3675	G/C	Gene_Ca_28063	INTRON	Multiantimicrobial extrusion protein
Kabuli_wild_122915	1399945983	*Ca_kabuli_scaffold*	122915	C/T	Gene_Ca_24092	INTRON	NA
Kabuli_wild_160597	1399945984	*Ca_kabuli_scaffold*	160597	C/T	Gene_Ca_24092	NON_SYNONYMOUS_CODING	NA
Kabuli_wild_160599	1399945985	*Ca_kabuli_scaffold*	160599	T/A	Gene_Ca_24092	SYNONYMOUS_CODING	NA
Kabuli_wild_160623	1399945986	*Ca_kabuli_scaffold*	160623	G/T	Gene_Ca_24092	SYNONYMOUS_CODING	NA
Kabuli_wild_160625	1399945987	*Ca_kabuli_scaffold*	160625	C/T	Gene_Ca_24092	NON_SYNONYMOUS_CODING	NA
Kabuli_wild_160709	1399945988	*Ca_kabuli_scaffold*	160709	T/C	Gene_Ca_24092	SYNONYMOUS_CODING	NA
Kabuli_wild_160749	1399945989	*Ca_kabuli_scaffold*	160749	T/C	Gene_Ca_24092	SYNONYMOUS_CODING	NA
Kabuli_wild_189663	1399945990	*Ca_kabuli_scaffold*	189663	G/A	NA	INTERGENIC	NA
Kabuli_wild_189664	1399945991	*Ca_kabuli_scaffold*	189664	G/A	NA	INTERGENIC	NA
Kabuli_wild_189681	1399945992	*Ca_kabuli_scaffold*	189681	C/T	NA	INTERGENIC	NA
Kabuli_wild_189699	1399945993	*Ca_kabuli_scaffold*	189699	C/T	NA	INTERGENIC	NA
Kabuli_wild_189700	1399945994	*Ca_kabuli_scaffold*	189700	A/G	NA	INTERGENIC	NA
Kabuli_wild_197789	1399945995	*Ca_kabuli_scaffold*	197789	G/A	NA	INTERGENIC	NA
Kabuli_wild_197790	1399945996	*Ca_kabuli_scaffold*	197790	G/A	NA	INTERGENIC	NA
Kabuli_wild_197807	1399945997	*Ca_kabuli_scaffold*	197807	T/C	NA	INTERGENIC	NA
Kabuli_wild_197819	1399945998	*Ca_kabuli_scaffold*	197819	T/G	NA	INTERGENIC	NA
Kabuli_wild_197826	1399945999	*Ca_kabuli_scaffold*	197826	G/A	NA	INTERGENIC	NA
Kabuli_wild_197873	1399946000	*Ca_kabuli_scaffold*	197873	C/G	NA	INTERGENIC	NA
Kabuli_wild_240677	1399946001	*Ca_kabuli_scaffold*	240677	T/A	NA	INTERGENIC	NA
Kabuli_wild_240713	1399946002	*Ca_kabuli_scaffold*	240713	T/C	NA	INTERGENIC	NA
Kabuli_wild_20734	1399946003	*Ca_kabuli_scaffold*	20734	G/T	NA	INTERGENIC	NA
Kabuli_wild_20744	1399946004	*Ca_kabuli_scaffold*	20744	G/T	NA	INTERGENIC	NA
Kabuli_wild_33136	1399946005	*Ca_kabuli_scaffold*	33136	A/C	Gene_Ca_28120	NON_SYNONYMOUS_CODING	NA
Kabuli_wild_33297	1399946006	*Ca_kabuli_scaffold*	33297	A/C	Gene_Ca_28120	SYNONYMOUS_CODING	NA
Kabuli_wild_34102	1399946007	*Ca_kabuli_scaffold*	34102	A/C	Gene_Ca_28120	INTRON	NA
Kabuli_wild_98231	1399946008	*Ca_kabuli_scaffold*	98231	A/G	NA	INTERGENIC	NA
Kabuli_wild_20779	1399946009	*Ca_kabuli_scaffold*	20779	T/C	Gene_Ca_26979	DOWNSTREAM	Protein kinase,catalytic domain
Kabuli_wild_10470	1399946010	*Ca_kabuli_scaffold*	10470	G/T	NA	INTERGENIC	NA
Kabuli_wild_10471	1399946011	*Ca_kabuli_scaffold*	10471	T/C	NA	INTERGENIC	NA
Kabuli_wild_10510	1399946012	*Ca_kabuli_scaffold*	10510	T/G	NA	INTERGENIC	NA
Kabuli_wild_26707	1399946013	*Ca_kabuli_scaffold*	26707	G/T	NA	INTERGENIC	NA
Kabuli_wild_86513	1399946014	*Ca_kabuli_scaffold*	86513	G/A	Gene_Ca_26592	DOWNSTREAM	NA
Kabuli_wild_4159	1399946015	*Ca_kabuli_scaffold*	4159	G/T	NA	INTERGENIC	NA

SNP IDs	NCBI dbSNP accession IDs (NCBI_SS#)	Chromosomes	Physical positions (bp)	SNPs	Gene accession IDs	Sequence components of genome	Putative functions
Kabuli_wild_4175	1399946016	*Ca_kabuli_scaffold*	4175	T/A	NA	INTERGENIC	NA
Kabuli_wild_81468	1399946017	*Ca_kabuli_scaffold*	81468	G/C	NA	INTERGENIC	NA
Kabuli_wild_111476	1399946018	*Ca_kabuli_scaffold*	111476	G/A	Gene_Ca_23856	NON_SYNONYMOUS_CODING	Phosphoenolpyruvatecarboxylase,activesite
Kabuli_wild_233787	1399946019	*Ca_kabuli_scaffold*	233787	C/T	Gene_Ca_23863	INTRON	NA
Kabuli_wild_5079	1399946020	*Ca_kabuli_scaffold*	5079	C/A	Gene_Ca_27877	NON_SYNONYMOUS_CODING	Peptidase C48, SUMO/Sentrin/Ubl1
Kabuli_wild_5104	1399946021	*Ca_kabuli_scaffold*	5104	T/G	Gene_Ca_27877	SYNONYMOUS_CODING	Peptidase C48, SUMO/Sentrin/Ubl1
Kabuli_wild_5115	1399946022	*Ca_kabuli_scaffold*	5115	C/T	Gene_Ca_27877	NON_SYNONYMOUS_CODING	Peptidase C48, SUMO/Sentrin/Ubl1
Kabuli_wild_5163	1399946023	*Ca_kabuli_scaffold*	5163	C/T	Gene_Ca_27877	NON_SYNONYMOUS_CODING	Peptidase C48, SUMO/Sentrin/Ubl1
Kabuli_wild_20517	1399946024	*Ca_kabuli_scaffold*	20517	T/C	NA	INTERGENIC	NA
Kabuli_wild_20521	1399946025	*Ca_kabuli_scaffold*	20521	G/T	NA	INTERGENIC	NA
Kabuli_wild_20559	1399946026	*Ca_kabuli_scaffold*	20559	G/A	NA	INTERGENIC	NA
Kabuli_wild_41784	1399946027	*Ca_kabuli_scaffold*	41784	T/C	NA	INTERGENIC	NA
Kabuli_wild_41792	1399946028	*Ca_kabuli_scaffold*	41792	C/T	NA	INTERGENIC	NA
Kabuli_wild_41793	1399946029	*Ca_kabuli_scaffold*	41793	T/G	NA	INTERGENIC	NA
Kabuli_wild_41795	1399946030	*Ca_kabuli_scaffold*	41795	A/C	NA	INTERGENIC	NA
Kabuli_wild_41808	1399946031	*Ca_kabuli_scaffold*	41808	T/A	NA	INTERGENIC	NA
Kabuli_wild_41844	1399946032	*Ca_kabuli_scaffold*	41844	T/C	NA	INTERGENIC	NA
Kabuli_wild_41847	1399946033	*Ca_kabuli_scaffold*	41847	T/A	NA	INTERGENIC	NA
Kabuli_wild_41852	1399946034	*Ca_kabuli_scaffold*	41852	C/T	NA	INTERGENIC	NA
Kabuli_wild_41871	1399946035	*Ca_kabuli_scaffold*	41871	G/A	NA	INTERGENIC	NA
Kabuli_wild_2141	1399946036	*Ca_kabuli_scaffold*	2141	T/C	Gene_Ca_27017	DOWNSTREAM	Peptidase C48, SUMO/Sentrin/Ubl1
Kabuli_wild_2145	1399946037	*Ca_kabuli_scaffold*	2145	G/A	Gene_Ca_27017	DOWNSTREAM	Peptidase C48, SUMO/Sentrin/Ubl1
Kabuli_wild_2148	1399946038	*Ca_kabuli_scaffold*	2148	G/A	Gene_Ca_27017	DOWNSTREAM	Peptidase C48, SUMO/Sentrin/Ubl1
Kabuli_wild_2163	1399946039	*Ca_kabuli_scaffold*	2163	C/T	Gene_Ca_27017	DOWNSTREAM	Peptidase C48, SUMO/Sentrin/Ubl1
Kabuli_wild_2171	1399946040	*Ca_kabuli_scaffold*	2171	T/G	Gene_Ca_27017	DOWNSTREAM	Peptidase C48, SUMO/Sentrin/Ubl1
Kabuli_wild_2182	1399946041	*Ca_kabuli_scaffold*	2182	C/T	Gene_Ca_27017	DOWNSTREAM	Peptidase C48, SUMO/Sentrin/Ubl1
Kabuli_wild_2227	1399946042	*Ca_kabuli_scaffold*	2227	C/T	Gene_Ca_27017	DOWNSTREAM	Peptidase C48, SUMO/Sentrin/Ubl1
Kabuli_wild_2230	1399946043	*Ca_kabuli_scaffold*	2230	C/T	Gene_Ca_27017	DOWNSTREAM	Peptidase C48, SUMO/Sentrin/Ubl1
Kabuli_wild_71861	1399946044	*Ca_kabuli_scaffold*	71861	G/T	NA	INTERGENIC	NA
Kabuli_wild_58985	1399946045	*Ca_kabuli_scaffold*	58985	T/G	NA	INTERGENIC	NA
Kabuli_wild_58988	1399946046	*Ca_kabuli_scaffold*	58988	G/A	NA	INTERGENIC	NA
Kabuli_wild_59020	1399946047	*Ca_kabuli_scaffold*	59020	C/T	NA	INTERGENIC	NA
Kabuli_wild_11828	1399946048	*Ca_kabuli_scaffold*	11828	C/G	NA	INTERGENIC	NA
Kabuli_wild_11873	1399946049	*Ca_kabuli_scaffold*	11873	C/T	NA	INTERGENIC	NA
Kabuli_wild_25514	1399946050	*Ca_kabuli_scaffold*	25514	A/G	Gene_Ca_27084	INTRON	Glycerol-3-phosphatedehydrogenase,NAD-dependent,C-terminal
Kabuli_wild_48917	1399946051	*Ca_kabuli_scaffold*	48917	T/G	Gene_Ca_27472	INTRON	Protein kinase,catalytic domain
Kabuli_wild_48979	1399946052	*Ca_kabuli_scaffold*	48979	T/C	Gene_Ca_27472	INTRON	Protein kinase,catalytic domain
Kabuli_wild_107750	1399946053	*Ca_kabuli_scaffold*	107750	T/A	Gene_Ca_27474	NON_SYNONYMOUS_CODING	Photosyntheticreactioncentre,L/M
Kabuli_wild_193075	1399946054	*Ca_kabuli_scaffold*	193075	G/A	Gene_Ca_27067	NON_SYNONYMOUS_CODING	NA
Kabuli_wild_193086	1399946055	*Ca_kabuli_scaffold*	193086	C/G	Gene_Ca_27067	NON_SYNONYMOUS_CODING	NA
Kabuli_wild_23507	1399946056	*Ca_kabuli_scaffold*	23507	A/G	NA	INTERGENIC	NA
Kabuli_wild_30981	1399946057	*Ca_kabuli_scaffold*	30981	T/C	Gene_Ca_26492	UPSTREAM	Zinc finger,CCHC-type
Kabuli_wild_31028	1399946058	*Ca_kabuli_scaffold*	31028	C/T	Gene_Ca_26492	UPSTREAM	Zinc finger,CCHC-type
Kabuli_wild_61364	1399946059	*Ca_kabuli_scaffold*	61364	T/G	Gene_Ca_26493	INTRON	DAHPsynthetase,classII

SNP IDs	NCBI dbSNP accession IDs (NCBI_SS#)	Chromosomes	Physical positions (bp)	SNPs	Gene accession IDs	Sequence components of genome	Putative functions
Kabuli_wild_143399	1399946060	*Ca_kabuli_scaffold*	143399	T/C	NA	INTERGENIC	NA
Kabuli_wild_143440	1399946061	*Ca_kabuli_scaffold*	143440	C/T	NA	INTERGENIC	NA
Kabuli_wild_177592	1399946062	*Ca_kabuli_scaffold*	177592	T/G	Gene_Ca_26483	SYNONYMOUS_CODING	NA
Kabuli_wild_177623	1399946063	*Ca_kabuli_scaffold*	177623	G/A	Gene_Ca_26483	NON_SYNONYMOUS_CODING	NA
Kabuli_wild_177650	1399946064	*Ca_kabuli_scaffold*	177650	C/T	Gene_Ca_26483	NON_SYNONYMOUS_CODING	NA
Kabuli_wild_177701	1399946065	*Ca_kabuli_scaffold*	177701	C/A	Gene_Ca_26483	NON_SYNONYMOUS_CODING	NA
Kabuli_wild_17107	1399946066	*Ca_kabuli_scaffold*	17107	A/G	Gene_Ca_26202	NON_SYNONYMOUS_CODING	Pleckstrinhomologydomain
Kabuli_wild_146218	1399946067	*Ca_kabuli_scaffold*	146218	G/A	Gene_Ca_26207	NON_SYNONYMOUS_CODING	Peptidase C48, SUMO/Sentrin/Ubl1
Kabuli_wild_83227	1399946068	*Ca_kabuli_scaffold*	83227	G/C	NA	INTERGENIC	NA
Kabuli_wild_83345	1399946069	*Ca_kabuli_scaffold*	83345	A/T	NA	INTERGENIC	NA
Kabuli_wild_83354	1399946070	*Ca_kabuli_scaffold*	83354	T/A	NA	INTERGENIC	NA
Kabuli_wild_15688	1399946071	*Ca_kabuli_scaffold*	15688	T/C	NA	INTERGENIC	NA
Kabuli_wild_26432	1399946072	*Ca_kabuli_scaffold*	26432	G/A	NA	INTERGENIC	NA
Kabuli_wild_303945	1399946073	*Ca_kabuli_scaffold*	303945	T/G	NA	INTERGENIC	NA
Kabuli_wild_303966	1399946074	*Ca_kabuli_scaffold*	303966	C/A	NA	INTERGENIC	NA
Kabuli_wild_332100	1399946075	*Ca_kabuli_scaffold*	332100	T/C	NA	INTERGENIC	NA
Kabuli_wild_70434	1399946076	*Ca_kabuli_scaffold*	70434	A/G	Gene_Ca_27588	UPSTREAM	NA
Kabuli_wild_90363	1399946077	*Ca_kabuli_scaffold*	90363	G/A	NA	INTERGENIC	NA
Kabuli_wild_90371	1399946078	*Ca_kabuli_scaffold*	90371	G/A	NA	INTERGENIC	NA
Kabuli_wild_90419	1399946079	*Ca_kabuli_scaffold*	90419	A/C	NA	INTERGENIC	NA
Kabuli_wild_90430	1399946080	*Ca_kabuli_scaffold*	90430	T/A	NA	INTERGENIC	NA
Kabuli_wild_94984	1399946081	*Ca_kabuli_scaffold*	94984	A/T	Gene_Ca_26648	DOWNSTREAM	Integrase,catalyticcore
Kabuli_wild_82935	1399946082	*Ca_kabuli_scaffold*	82935	A/C	Gene_Ca_26679	SYNONYMOUS_CODING	Plantlipidtransferprotein/Parallergen
Kabuli_wild_14513	1399946083	*Ca_kabuli_scaffold*	14513	T/C	NA	INTERGENIC	NA
Kabuli_wild_27546	1399946084	*Ca_kabuli_scaffold*	27546	T/C	NA	INTERGENIC	NA
Kabuli_wild_27556	1399946085	*Ca_kabuli_scaffold*	27556	A/C	NA	INTERGENIC	NA
Kabuli_wild_27568	1399946086	*Ca_kabuli_scaffold*	27568	A/G	NA	INTERGENIC	NA
Kabuli_wild_27575	1399946087	*Ca_kabuli_scaffold*	27575	T/C	NA	INTERGENIC	NA
Kabuli_wild_27607	1399946088	*Ca_kabuli_scaffold*	27607	T/G	NA	INTERGENIC	NA
Kabuli_wild_27613	1399946089	*Ca_kabuli_scaffold*	27613	G/A	NA	INTERGENIC	NA
Kabuli_wild_62013	1399946090	*Ca_kabuli_scaffold*	62013	C/T	Gene_Ca_26048	NON_SYNONYMOUS_CODING	Annexin
Kabuli_wild_9837	1399946091	*Ca_kabuli_scaffold*	9837	A/C	NA	INTERGENIC	NA
Kabuli_wild_241	1399946092	*Ca_kabuli_scaffold*	241	G/A	NA	INTERGENIC	NA
Kabuli_wild_268	1399946093	*Ca_kabuli_scaffold*	268	C/G	NA	INTERGENIC	NA
Kabuli_wild_295	1399946094	*Ca_kabuli_scaffold*	295	G/A	NA	INTERGENIC	NA
Kabuli_wild_301	1399946095	*Ca_kabuli_scaffold*	301	A/C	NA	INTERGENIC	NA
Kabuli_wild_302	1399946096	*Ca_kabuli_scaffold*	302	G/A	NA	INTERGENIC	NA
Kabuli_wild_3794	1399946097	*Ca_kabuli_scaffold*	3794	A/C	NA	INTERGENIC	NA
Kabuli_wild_3898	1399946098	*Ca_kabuli_scaffold*	3898	G/C	NA	INTERGENIC	NA
Kabuli_wild_14251	1399946099	*Ca_kabuli_scaffold*	14251	C/T	NA	INTERGENIC	NA
Kabuli_wild_14276	1399946100	*Ca_kabuli_scaffold*	14276	C/A	NA	INTERGENIC	NA
Kabuli_wild_14288	1399946101	*Ca_kabuli_scaffold*	14288	A/T	NA	INTERGENIC	NA
Kabuli_wild_19130	1399946102	*Ca_kabuli_scaffold*	19130	T/C	Gene_Ca_27100	INTRON	Protein kinase,catalytic domain
Kabuli_wild_21521	1399946103	*Ca_kabuli_scaffold*	21521	A/G	Gene_Ca_27100	INTRON	Protein kinase,catalytic domain

SNP IDs	NCBI dbSNP accession IDs (NCBI_SS#)	Chromosomes	Physical positions (bp)	SNPs	Gene accession IDs	Sequence components of genome	Putative functions
Kabuli_wild_40669	1399946104	*Ca_kabuli_scaffold*	40669	T/C	NA	INTERGENIC	NA
Kabuli_wild_65999	1399946105	*Ca_kabuli_scaffold*	65999	C/A	Gene_Ca_26630	INTRON	Ribulose-phosphate3-epimerase
Kabuli_wild_85902	1399946106	*Ca_kabuli_scaffold*	85902	G/T	Gene_Ca_26631	DOWNSTREAM	NA
Kabuli_wild_133488	1399946107	*Ca_kabuli_scaffold*	133488	C/T	NA	INTERGENIC	NA
Kabuli_wild_133489	1399946108	*Ca_kabuli_scaffold*	133489	A/G	NA	INTERGENIC	NA
Kabuli_wild_135562	1399946109	*Ca_kabuli_scaffold*	135562	A/G	NA	INTERGENIC	NA
Kabuli_wild_135565	1399946110	*Ca_kabuli_scaffold*	135565	T/A	NA	INTERGENIC	NA
Kabuli_wild_135569	1399946111	*Ca_kabuli_scaffold*	135569	G/C	NA	INTERGENIC	NA
Kabuli_wild_135571	1399946112	*Ca_kabuli_scaffold*	135571	A/G	NA	INTERGENIC	NA
Kabuli_wild_135589	1399946113	*Ca_kabuli_scaffold*	135589	C/T	NA	INTERGENIC	NA
Kabuli_wild_135590	1399946114	*Ca_kabuli_scaffold*	135590	G/A	NA	INTERGENIC	NA
Kabuli_wild_135608	1399946115	*Ca_kabuli_scaffold*	135608	A/G	NA	INTERGENIC	NA
Kabuli_wild_195977	1399946116	*Ca_kabuli_scaffold*	195977	C/T	NA	INTERGENIC	NA
Kabuli_wild_22289	1399946117	*Ca_kabuli_scaffold*	22289	T/G	NA	INTERGENIC	NA
Kabuli_wild_22347	1399946118	*Ca_kabuli_scaffold*	22347	G/A	NA	INTERGENIC	NA
Kabuli_wild_22350	1399946119	*Ca_kabuli_scaffold*	22350	T/G	NA	INTERGENIC	NA
Kabuli_wild_22354	1399946120	*Ca_kabuli_scaffold*	22354	C/T	NA	INTERGENIC	NA
Kabuli_wild_22368	1399946121	*Ca_kabuli_scaffold*	22368	T/C	NA	INTERGENIC	NA
Kabuli_wild_33154	1399946122	*Ca_kabuli_scaffold*	33154	A/G	NA	INTERGENIC	NA
Kabuli_wild_33301	1399946123	*Ca_kabuli_scaffold*	33301	C/A	NA	INTERGENIC	NA
Kabuli_wild_99918	1399946124	*Ca_kabuli_scaffold*	99918	A/G	NA	INTERGENIC	NA
Kabuli_wild_9015	1399946125	*Ca_kabuli_scaffold*	9015	C/T	NA	INTERGENIC	NA
Kabuli_wild_15510	1399946126	*Ca_kabuli_scaffold*	15510	C/T	Gene_Ca_24921	SYNONYMOUS_CODING	UBA/THIF-typeNAD/FADbindingfold
Kabuli_wild_208997	1399946127	*Ca_kabuli_scaffold*	208997	G/A	Gene_Ca_24928	NON_SYNONYMOUS_CODING	WD40 repeat
Kabuli_wild_209030	1399946128	*Ca_kabuli_scaffold*	209030	G/A	Gene_Ca_24928	NON_SYNONYMOUS_CODING	WD40 repeat
Kabuli_wild_192417	1399946129	*Ca_kabuli_scaffold*	192417	A/C	NA	INTERGENIC	NA
Kabuli_wild_192713	1399946130	*Ca_kabuli_scaffold*	192713	G/T	NA	INTERGENIC	NA
Kabuli_wild_193013	1399946131	*Ca_kabuli_scaffold*	193013	T/G	NA	INTERGENIC	NA
Kabuli_wild_266583	1399946132	*Ca_kabuli_scaffold*	266583	G/A	NA	INTERGENIC	NA
Kabuli_wild_456311	1399946133	*Ca_kabuli_scaffold*	456311	G/A	Gene_Ca_23937	INTRON	Peptidase C48, SUMO/Sentrin/Ubl1
Kabuli_wild_456342	1399946134	*Ca_kabuli_scaffold*	456342	G/T	Gene_Ca_23937	INTRON	Peptidase C48, SUMO/Sentrin/Ubl1
Kabuli_wild_456357	1399946135	*Ca_kabuli_scaffold*	456357	G/A	Gene_Ca_23937	INTRON	Peptidase C48, SUMO/Sentrin/Ubl1
Kabuli_wild_496626	1399946136	*Ca_kabuli_scaffold*	496626	G/A	Gene_Ca_23938	STOP_GAINED	NA
Kabuli_wild_528896	1399946137	*Ca_kabuli_scaffold*	528896	A/C	NA	INTERGENIC	NA
Kabuli_wild_528979	1399946138	*Ca_kabuli_scaffold*	528979	T/C	NA	INTERGENIC	NA
Kabuli_wild_625332	1399946139	*Ca_kabuli_scaffold*	625332	A/C	NA	INTERGENIC	NA
Kabuli_wild_625335	1399946140	*Ca_kabuli_scaffold*	625335	G/A	NA	INTERGENIC	NA
Kabuli_wild_625342	1399946141	*Ca_kabuli_scaffold*	625342	C/T	NA	INTERGENIC	NA
Kabuli_wild_625355	1399946142	*Ca_kabuli_scaffold*	625355	C/T	NA	INTERGENIC	NA
Kabuli_wild_625360	1399946143	*Ca_kabuli_scaffold*	625360	T/C	NA	INTERGENIC	NA
Kabuli_wild_625362	1399946144	*Ca_kabuli_scaffold*	625362	C/T	NA	INTERGENIC	NA
Kabuli_wild_625365	1399946145	*Ca_kabuli_scaffold*	625365	T/C	NA	INTERGENIC	NA
Kabuli_wild_625366	1399946146	*Ca_kabuli_scaffold*	625366	T/C	NA	INTERGENIC	NA
Kabuli_wild_625375	1399946147	*Ca_kabuli_scaffold*	625375	G/A	NA	INTERGENIC	NA

SNP IDs	NCBI dbSNP accession IDs (NCBI_SS#)	Chromosomes	Physical positions (bp)	SNPs	Gene accession IDs	Sequence components of genome	Putative functions
Kabuli_wild_625378	1399946148	*Ca_kabuli_scaffold*	625378	G/A	NA	INTERGENIC	NA
Kabuli_wild_625383	1399946149	*Ca_kabuli_scaffold*	625383	C/A	NA	INTERGENIC	NA
Kabuli_wild_625388	1399946150	*Ca_kabuli_scaffold*	625388	C/T	NA	INTERGENIC	NA
Kabuli_wild_625389	1399946151	*Ca_kabuli_scaffold*	625389	A/C	NA	INTERGENIC	NA
Kabuli_wild_625394	1399946152	*Ca_kabuli_scaffold*	625394	C/T	NA	INTERGENIC	NA
Kabuli_wild_625395	1399946153	*Ca_kabuli_scaffold*	625395	A/G	NA	INTERGENIC	NA
Kabuli_wild_625396	1399946154	*Ca_kabuli_scaffold*	625396	G/A	NA	INTERGENIC	NA
Kabuli_wild_128665	1399946155	*Ca_kabuli_scaffold*	128665	A/C	Gene_Ca_27387	UPSTREAM	NA
Kabuli_wild_153879	1399946156	*Ca_kabuli_scaffold*	153879	A/G	Gene_Ca_26478	UPSTREAM	NA
Kabuli_wild_25892	1399946157	*Ca_kabuli_scaffold*	25892	A/G	NA	INTERGENIC	NA
Kabuli_wild_25910	1399946158	*Ca_kabuli_scaffold*	25910	C/T	NA	INTERGENIC	NA
Kabuli_wild_37261	1399946159	*Ca_kabuli_scaffold*	37261	A/G	NA	INTERGENIC	NA
Kabuli_wild_37309	1399946160	*Ca_kabuli_scaffold*	37309	G/T	NA	INTERGENIC	NA
Kabuli_wild_37312	1399946161	*Ca_kabuli_scaffold*	37312	G/T	NA	INTERGENIC	NA
Kabuli_wild_43519	1399946162	*Ca_kabuli_scaffold*	43519	T/A	NA	INTERGENIC	NA
Kabuli_wild_43523	1399946163	*Ca_kabuli_scaffold*	43523	C/T	NA	INTERGENIC	NA
Kabuli_wild_43549	1399946164	*Ca_kabuli_scaffold*	43549	C/T	NA	INTERGENIC	NA
Kabuli_wild_43551	1399946165	*Ca_kabuli_scaffold*	43551	T/A	NA	INTERGENIC	NA
Kabuli_wild_43553	1399946166	*Ca_kabuli_scaffold*	43553	G/C	NA	INTERGENIC	NA
Kabuli_wild_43555	1399946167	*Ca_kabuli_scaffold*	43555	C/A	NA	INTERGENIC	NA
Kabuli_wild_43559	1399946168	*Ca_kabuli_scaffold*	43559	C/A	NA	INTERGENIC	NA
Kabuli_wild_43564	1399946169	*Ca_kabuli_scaffold*	43564	C/T	NA	INTERGENIC	NA
Kabuli_wild_43567	1399946170	*Ca_kabuli_scaffold*	43567	C/G	NA	INTERGENIC	NA
Kabuli_wild_164730	1399946171	*Ca_kabuli_scaffold*	164730	T/G	NA	INTERGENIC	NA
Kabuli_wild_169037	1399946172	*Ca_kabuli_scaffold*	169037	G/T	NA	INTERGENIC	NA
Kabuli_wild_290930	1399946173	*Ca_kabuli_scaffold*	290930	G/T	Gene_Ca_23267	NON_SYNONYMOUS_CODING	Histidine phosphatase superfamily,clade-1
Kabuli_wild_343173	1399946174	*Ca_kabuli_scaffold*	343173	C/T	NA	INTERGENIC	NA
Kabuli_wild_343185	1399946175	*Ca_kabuli_scaffold*	343185	G/A	NA	INTERGENIC	NA
Kabuli_wild_343239	1399946176	*Ca_kabuli_scaffold*	343239	A/T	NA	INTERGENIC	NA
Kabuli_wild_356970	1399946177	*Ca_kabuli_scaffold*	356970	G/A	NA	INTERGENIC	NA
Kabuli_wild_356986	1399946178	*Ca_kabuli_scaffold*	356986	A/C	NA	INTERGENIC	NA
Kabuli_wild_356998	1399946179	*Ca_kabuli_scaffold*	356998	C/T	NA	INTERGENIC	NA
Kabuli_wild_357004	1399946180	*Ca_kabuli_scaffold*	357004	C/T	NA	INTERGENIC	NA
Kabuli_wild_357007	1399946181	*Ca_kabuli_scaffold*	357007	C/G	NA	INTERGENIC	NA
Kabuli_wild_448166	1399946182	*Ca_kabuli_scaffold*	448166	A/G	Gene_Ca_23268	DOWNSTREAM	Integrase,catalytic core
Kabuli_wild_448171	1399946183	*Ca_kabuli_scaffold*	448171	T/C	Gene_Ca_23268	DOWNSTREAM	Integrase,catalytic core
Kabuli_wild_448176	1399946184	*Ca_kabuli_scaffold*	448176	C/T	Gene_Ca_23268	DOWNSTREAM	Integrase,catalytic core
Kabuli_wild_448187	1399946185	*Ca_kabuli_scaffold*	448187	G/T	Gene_Ca_23268	DOWNSTREAM	Integrase,catalytic core
Kabuli_wild_448194	1399946186	*Ca_kabuli_scaffold*	448194	G/A	Gene_Ca_23268	DOWNSTREAM	Integrase,catalytic core
Kabuli_wild_448195	1399946187	*Ca_kabuli_scaffold*	448195	C/T	Gene_Ca_23268	DOWNSTREAM	Integrase,catalytic core
Kabuli_wild_448198	1399946188	*Ca_kabuli_scaffold*	448198	A/C	Gene_Ca_23268	DOWNSTREAM	Integrase,catalytic core
Kabuli_wild_448216	1399946189	*Ca_kabuli_scaffold*	448216	C/T	Gene_Ca_23268	DOWNSTREAM	Integrase,catalytic core
Kabuli_wild_448230	1399946190	*Ca_kabuli_scaffold*	448230	C/A	Gene_Ca_23268	DOWNSTREAM	Integrase,catalytic core
Kabuli_wild_448262	1399946191	*Ca_kabuli_scaffold*	448262	G/T	Gene_Ca_23268	DOWNSTREAM	Integrase,catalytic core

SNP IDs	NCBI dbSNP accession IDs (NCBI_SS#)	Chromosomes	Physical positions (bp)	SNPs	Gene accession IDs	Sequence components of genome	Putative functions
Kabuli_wild_448275	1399946192	*Ca_kabuli_scaffold*	448275	A/C	Gene_Ca_23268	DOWNSTREAM	Integrase,catalytic core
Kabuli_wild_890	1399946193	*Ca_kabuli_scaffold*	890	G/A	NA	INTERGENIC	NA
Kabuli_wild_957	1399946194	*Ca_kabuli_scaffold*	957	G/A	NA	INTERGENIC	NA
Kabuli_wild_972	1399946195	*Ca_kabuli_scaffold*	972	C/T	NA	INTERGENIC	NA
Kabuli_wild_176	1399946196	*Ca_kabuli_scaffold*	176	G/A	NA	INTERGENIC	NA
Kabuli_wild_17033	1399946197	*Ca_kabuli_scaffold*	17033	A/G	NA	INTERGENIC	NA
Kabuli_wild_17068	1399946198	*Ca_kabuli_scaffold*	17068	A/G	NA	INTERGENIC	NA
Kabuli_wild_17111	1399946199	*Ca_kabuli_scaffold*	17111	C/T	NA	INTERGENIC	NA
Kabuli_wild_17117	1399946200	*Ca_kabuli_scaffold*	17117	T/C	NA	INTERGENIC	NA
Kabuli_wild_17120	1399946201	*Ca_kabuli_scaffold*	17120	C/T	NA	INTERGENIC	NA
Kabuli_wild_17123	1399946202	*Ca_kabuli_scaffold*	17123	G/T	NA	INTERGENIC	NA
Kabuli_wild_17132	1399946203	*Ca_kabuli_scaffold*	17132	C/T	NA	INTERGENIC	NA
Kabuli_wild_17150	1399946204	*Ca_kabuli_scaffold*	17150	T/C	NA	INTERGENIC	NA
Kabuli_wild_17151	1399946205	*Ca_kabuli_scaffold*	17151	T/C	NA	INTERGENIC	NA
Kabuli_wild_17177	1399946206	*Ca_kabuli_scaffold*	17177	T/A	NA	INTERGENIC	NA
Kabuli_wild_75173	1399946207	*Ca_kabuli_scaffold*	75173	C/T	Gene_Ca_26000	DOWNSTREAM	Tetra tricopeptide repeat-containing
Kabuli_wild_84683	1399946208	*Ca_kabuli_scaffold*	84683	C/T	Gene_Ca_26001	STOP_GAINED	TetratricopeptideTPR-1
Kabuli_wild_23272	1399946209	*Ca_kabuli_scaffold*	23272	A/C	NA	INTERGENIC	NA
Kabuli_wild_23281	1399946210	*Ca_kabuli_scaffold*	23281	C/T	NA	INTERGENIC	NA
Kabuli_wild_44987	1399946211	*Ca_kabuli_scaffold*	44987	G/C	NA	INTERGENIC	NA
Kabuli_wild_45018	1399946212	*Ca_kabuli_scaffold*	45018	T/C	NA	INTERGENIC	NA
Kabuli_wild_45036	1399946213	*Ca_kabuli_scaffold*	45036	A/C	NA	INTERGENIC	NA
Kabuli_wild_45041	1399946214	*Ca_kabuli_scaffold*	45041	A/T	NA	INTERGENIC	NA
Kabuli_wild_54636	1399946215	*Ca_kabuli_scaffold*	54636	A/T	NA	INTERGENIC	NA
Kabuli_wild_54683	1399946216	*Ca_kabuli_scaffold*	54683	G/A	NA	INTERGENIC	NA
Kabuli_wild_54694	1399946217	*Ca_kabuli_scaffold*	54694	T/G	NA	INTERGENIC	NA
Kabuli_wild_54702	1399946218	*Ca_kabuli_scaffold*	54702	C/T	NA	INTERGENIC	NA
Kabuli_wild_54714	1399946219	*Ca_kabuli_scaffold*	54714	G/A	NA	INTERGENIC	NA
Kabuli_wild_54725	1399946220	*Ca_kabuli_scaffold*	54725	T/A	NA	INTERGENIC	NA
Kabuli_wild_25960	1399946221	*Ca_kabuli_scaffold*	25960	A/G	NA	INTERGENIC	NA
Kabuli_wild_25983	1399946222	*Ca_kabuli_scaffold*	25983	T/G	NA	INTERGENIC	NA
Kabuli_wild_9398	1399946223	*Ca_kabuli_scaffold*	9398	C/A	NA	INTERGENIC	NA
Kabuli_wild_10541	1399946224	*Ca_kabuli_scaffold*	10541	C/G	NA	INTERGENIC	NA
Kabuli_wild_10563	1399946225	*Ca_kabuli_scaffold*	10563	T/C	NA	INTERGENIC	NA
Kabuli_wild_41899	1399946226	*Ca_kabuli_scaffold*	41899	C/A	NA	INTERGENIC	NA
Kabuli_wild_88569	1399946227	*Ca_kabuli_scaffold*	88569	C/T	Gene_Ca_24140	INTRON	Integrase,catalyticcore
Kabuli_wild_8898	1399946228	*Ca_kabuli_scaffold*	8898	T/G	NA	INTERGENIC	NA
Kabuli_wild_16292	1399946229	*Ca_kabuli_scaffold*	16292	C/G	NA	INTERGENIC	NA
Kabuli_wild_16317	1399946230	*Ca_kabuli_scaffold*	16317	C/G	NA	INTERGENIC	NA
Kabuli_wild_16331	1399946231	*Ca_kabuli_scaffold*	16331	A/G	NA	INTERGENIC	NA
Kabuli_wild_29722	1399946232	*Ca_kabuli_scaffold*	29722	G/A	Gene_Ca_26697	DOWNSTREAM	NA
Kabuli_wild_40821	1399946233	*Ca_kabuli_scaffold*	40821	T/G	NA	INTERGENIC	NA
Kabuli_wild_40833	1399946234	*Ca_kabuli_scaffold*	40833	T/A	NA	INTERGENIC	NA
Kabuli_wild_40842	1399946235	*Ca_kabuli_scaffold*	40842	T/G	NA	INTERGENIC	NA

SNP IDs	NCBI dbSNP accession IDs (NCBI_SS#)	Chromosomes	Physical positions (bp)	SNPs	Gene accession IDs	Sequence components of genome	Putative functions
Kabuli_wild_40871	1399946236	*Ca_kabuli_scaffold*	40871	T/A	NA	INTERGENIC	NA
Kabuli_wild_40872	1399946237	*Ca_kabuli_scaffold*	40872	T/G	NA	INTERGENIC	NA
Kabuli_wild_40886	1399946238	*Ca_kabuli_scaffold*	40886	T/A	NA	INTERGENIC	NA
Kabuli_wild_5102	1399946239	*Ca_kabuli_scaffold*	5102	T/C	NA	INTERGENIC	NA
Kabuli_wild_532	1399946240	*Ca_kabuli_scaffold*	532	G/C	Gene_Ca_27847	DOWNSTREAM	NA
Kabuli_wild_562	1399946241	*Ca_kabuli_scaffold*	562	G/C	Gene_Ca_27847	DOWNSTREAM	NA
Kabuli_wild_565	1399946242	*Ca_kabuli_scaffold*	565	T/C	Gene_Ca_27847	DOWNSTREAM	NA
Kabuli_wild_583	1399946243	*Ca_kabuli_scaffold*	583	A/G	Gene_Ca_27847	DOWNSTREAM	NA
Kabuli_wild_106324	1399946244	*Ca_kabuli_scaffold*	106324	C/T	NA	INTERGENIC	NA
Kabuli_wild_309668	1399946245	*Ca_kabuli_scaffold*	309668	G/A	Gene_Ca_25357	INTRON	NA
Kabuli_wild_187	1399946246	*Ca_kabuli_scaffold*	187	A/G	NA	INTERGENIC	NA
Kabuli_wild_191	1399946247	*Ca_kabuli_scaffold*	191	C/A	NA	INTERGENIC	NA
Kabuli_wild_30169	1399946248	*Ca_kabuli_scaffold*	30169	G/A	Gene_Ca_28131	DOWNSTREAM	NA
Kabuli_wild_216180	1399946249	*Ca_kabuli_scaffold*	216180	C/A	NA	INTERGENIC	NA
Kabuli_wild_216231	1399946250	*Ca_kabuli_scaffold*	216231	T/G	NA	INTERGENIC	NA
Kabuli_wild_245929	1399946251	*Ca_kabuli_scaffold*	245929	T/C	Gene_Ca_24715	SYNONYMOUS_CODING	Trehalose-phosphatase
Kabuli_wild_103092	1399946252	*Ca_kabuli_scaffold*	103092	A/G	NA	INTERGENIC	NA
Kabuli_wild_103114	1399946253	*Ca_kabuli_scaffold*	103114	G/A	NA	INTERGENIC	NA
Kabuli_wild_55070	1399946254	*Ca_kabuli_scaffold*	55070	A/T	NA	INTERGENIC	NA
Kabuli_wild_56095	1399946255	*Ca_kabuli_scaffold*	56095	G/C	NA	INTERGENIC	NA
Kabuli_wild_56215	1399946256	*Ca_kabuli_scaffold*	56215	A/G	NA	INTERGENIC	NA
Kabuli_wild_163751	1399946257	*Ca_kabuli_scaffold*	163751	C/T	NA	INTERGENIC	NA
Kabuli_wild_163763	1399946258	*Ca_kabuli_scaffold*	163763	T/G	NA	INTERGENIC	NA
Kabuli_wild_163780	1399946259	*Ca_kabuli_scaffold*	163780	C/T	NA	INTERGENIC	NA
Kabuli_wild_163784	1399946260	*Ca_kabuli_scaffold*	163784	G/A	NA	INTERGENIC	NA
Kabuli_wild_253620	1399946261	*Ca_kabuli_scaffold*	253620	T/C	NA	INTERGENIC	NA
Kabuli_wild_253821	1399946262	*Ca_kabuli_scaffold*	253821	T/G	NA	INTERGENIC	NA
Kabuli_wild_84160	1399946263	*Ca_kabuli_scaffold*	84160	G/A	NA	INTERGENIC	NA
Kabuli_wild_18756	1399946264	*Ca_kabuli_scaffold*	18756	C/T	NA	INTERGENIC	NA
Kabuli_wild_18765	1399946265	*Ca_kabuli_scaffold*	18765	C/T	NA	INTERGENIC	NA
Kabuli_wild_18777	1399946266	*Ca_kabuli_scaffold*	18777	C/T	NA	INTERGENIC	NA
Kabuli_wild_18780	1399946267	*Ca_kabuli_scaffold*	18780	G/A	NA	INTERGENIC	NA
Kabuli_wild_18782	1399946268	*Ca_kabuli_scaffold*	18782	C/A	NA	INTERGENIC	NA
Kabuli_wild_54604	1399946269	*Ca_kabuli_scaffold*	54604	T/C	NA	INTERGENIC	NA
Kabuli_wild_54613	1399946270	*Ca_kabuli_scaffold*	54613	G/A	NA	INTERGENIC	NA
Kabuli_wild_54643	1399946271	*Ca_kabuli_scaffold*	54643	G/A	NA	INTERGENIC	NA
Kabuli_wild_67038	1399946272	*Ca_kabuli_scaffold*	67038	C/T	NA	INTERGENIC	NA
Kabuli_wild_67042	1399946273	*Ca_kabuli_scaffold*	67042	G/A	NA	INTERGENIC	NA
Kabuli_wild_67049	1399946274	*Ca_kabuli_scaffold*	67049	G/C	NA	INTERGENIC	NA
Kabuli_wild_67073	1399946275	*Ca_kabuli_scaffold*	67073	G/A	NA	INTERGENIC	NA
Kabuli_wild_67086	1399946276	*Ca_kabuli_scaffold*	67086	A/C	NA	INTERGENIC	NA
Kabuli_wild_157962	1399946277	*Ca_kabuli_scaffold*	157962	C/A	NA	INTERGENIC	NA
Kabuli_wild_412	1399946278	*Ca_kabuli_scaffold*	412	T/A	NA	INTERGENIC	NA
Kabuli_wild_490	1399946279	*Ca_kabuli_scaffold*	490	G/A	NA	INTERGENIC	NA

SNP IDs	NCBI dbSNP accession IDs (NCBI_SS#)	Chromosomes	Physical positions (bp)	SNPs	Gene accession IDs	Sequence components of genome	Putative functions
Kabuli_wild_517	1399946280	*Ca_kabuli_scaffold*	517	G/A	NA	INTERGENIC	NA
Kabuli_wild_535	1399946281	*Ca_kabuli_scaffold*	535	T/C	NA	INTERGENIC	NA
Kabuli_wild_536	1399946282	*Ca_kabuli_scaffold*	536	C/T	NA	INTERGENIC	NA
Kabuli_wild_537	1399946283	*Ca_kabuli_scaffold*	537	T/G	NA	INTERGENIC	NA
Kabuli_wild_545	1399946284	*Ca_kabuli_scaffold*	545	C/T	NA	INTERGENIC	NA
Kabuli_wild_65136	1399946285	*Ca_kabuli_scaffold*	65136	T/G	Gene_Ca_21252	NON_SYNONYMOUS_CODING	GH3auxin-responsivepromoter
Kabuli_wild_241684	1399946286	*Ca_kabuli_scaffold*	241684	G/T	NA	INTERGENIC	NA
Kabuli_wild_265110	1399946287	*Ca_kabuli_scaffold*	265110	A/C	NA	INTERGENIC	NA
Kabuli_wild_265126	1399946288	*Ca_kabuli_scaffold*	265126	G/T	NA	INTERGENIC	NA
Kabuli_wild_265216	1399946289	*Ca_kabuli_scaffold*	265216	A/G	NA	INTERGENIC	NA
Kabuli_wild_269879	1399946290	*Ca_kabuli_scaffold*	269879	A/G	NA	INTERGENIC	NA
Kabuli_wild_339537	1399946291	*Ca_kabuli_scaffold*	339537	G/A	Gene_Ca_21267	NON_SYNONYMOUS_CODING	Pentatrico peptide repeat
Kabuli_wild_339547	1399946292	*Ca_kabuli_scaffold*	339547	C/T	Gene_Ca_21267	NON_SYNONYMOUS_CODING	Pentatrico peptide repeat
Kabuli_wild_339584	1399946293	*Ca_kabuli_scaffold*	339584	A/G	Gene_Ca_21267	SYNONYMOUS_CODING	Pentatrico peptide repeat
Kabuli_wild_382520	1399946294	*Ca_kabuli_scaffold*	382520	G/A	Gene_Ca_21269	INTRON	Nucleotidyltransferasedomain
Kabuli_wild_382900	1399946295	*Ca_kabuli_scaffold*	382900	C/T	Gene_Ca_21269	INTRON	Nucleotidyltransferasedomain
Kabuli_wild_385683	1399946296	*Ca_kabuli_scaffold*	385683	T/A	Gene_Ca_21269	SYNONYMOUS_CODING	Nucleotidyltransferasedomain
Kabuli_wild_46066	1399946297	*Ca_kabuli_scaffold*	46066	G/T	NA	INTERGENIC	NA
Kabuli_wild_46144	1399946298	*Ca_kabuli_scaffold*	46144	A/T	NA	INTERGENIC	NA
Kabuli_wild_46171	1399946299	*Ca_kabuli_scaffold*	46171	T/A	NA	INTERGENIC	NA
Kabuli_wild_212011	1399946300	*Ca_kabuli_scaffold*	212011	G/A	Gene_Ca_25764	NON_SYNONYMOUS_CODING	Leo1-likeprotein
Kabuli_wild_215883	1399946301	*Ca_kabuli_scaffold*	215883	G/T	Gene_Ca_25764	INTRON	Leo1-likeprotein
Kabuli_wild_215907	1399946302	*Ca_kabuli_scaffold*	215907	A/G	Gene_Ca_25764	INTRON	Leo1-likeprotein
Kabuli_wild_215969	1399946303	*Ca_kabuli_scaffold*	215969	G/C	Gene_Ca_25764	INTRON	Leo1-likeprotein
Kabuli_wild_292507	1399946304	*Ca_kabuli_scaffold*	292507	A/C	NA	INTERGENIC	NA
Kabuli_wild_292511	1399946305	*Ca_kabuli_scaffold*	292511	G/C	NA	INTERGENIC	NA
Kabuli_wild_349325	1399946306	*Ca_kabuli_scaffold*	349325	C/T	Gene_Ca_25768	INTRON	Peptidase C48, SUMO/Sentrin/Ubl1
Kabuli_wild_98575	1399946307	*Ca_kabuli_scaffold*	98575	C/A	NA	INTERGENIC	NA
Kabuli_wild_98592	1399946308	*Ca_kabuli_scaffold*	98592	C/T	NA	INTERGENIC	NA
Kabuli_wild_98594	1399946309	*Ca_kabuli_scaffold*	98594	G/A	NA	INTERGENIC	NA
Kabuli_wild_98611	1399946310	*Ca_kabuli_scaffold*	98611	C/T	NA	INTERGENIC	NA
Kabuli_wild_98634	1399946311	*Ca_kabuli_scaffold*	98634	C/T	NA	INTERGENIC	NA
Kabuli_wild_98656	1399946312	*Ca_kabuli_scaffold*	98656	C/T	NA	INTERGENIC	NA
Kabuli_wild_98662	1399946313	*Ca_kabuli_scaffold*	98662	C/A	NA	INTERGENIC	NA
Kabuli_wild_98663	1399946314	*Ca_kabuli_scaffold*	98663	T/A	NA	INTERGENIC	NA
Kabuli_wild_62009	1399946315	*Ca_kabuli_scaffold*	62009	C/A	NA	INTERGENIC	NA
Kabuli_wild_62014	1399946316	*Ca_kabuli_scaffold*	62014	G/A	NA	INTERGENIC	NA
Kabuli_wild_62085	1399946317	*Ca_kabuli_scaffold*	62085	A/C	NA	INTERGENIC	NA
Kabuli_wild_62121	1399946318	*Ca_kabuli_scaffold*	62121	G/A	NA	INTERGENIC	NA
Kabuli_wild_62131	1399946319	*Ca_kabuli_scaffold*	62131	G/A	NA	INTERGENIC	NA
Kabuli_wild_62145	1399946320	*Ca_kabuli_scaffold*	62145	G/C	NA	INTERGENIC	NA
Kabuli_wild_62147	1399946321	*Ca_kabuli_scaffold*	62147	G/A	NA	INTERGENIC	NA
Kabuli_wild_62154	1399946322	*Ca_kabuli_scaffold*	62154	T/A	NA	INTERGENIC	NA
Kabuli_wild_72277	1399946323	*Ca_kabuli_scaffold*	72277	G/A	NA	INTERGENIC	NA

SNP IDs	NCBI dbSNP accession IDs (NCBI_SS#)	Chromosomes	Physical positions (bp)	SNPs	Gene accession IDs	Sequence components of genome	Putative functions
Kabuli_wild_19707	1399946324	*Ca_kabuli_scaffold*	19707	C/T	Gene_Ca_28045	NON_SYNONYMOUS_CODING	NA
Kabuli_wild_19741	1399946325	*Ca_kabuli_scaffold*	19741	G/A	Gene_Ca_28045	SYNONYMOUS_CODING	NA
Kabuli_wild_111624	1399946326	*Ca_kabuli_scaffold*	111624	C/A	NA	INTERGENIC	NA
Kabuli_wild_111625	1399946327	*Ca_kabuli_scaffold*	111625	G/C	NA	INTERGENIC	NA
Kabuli_wild_141914	1399946328	*Ca_kabuli_scaffold*	141914	C/T	Gene_Ca_26289	NON_SYNONYMOUS_CODING	NA
Kabuli_wild_141927	1399946329	*Ca_kabuli_scaffold*	141927	G/A	Gene_Ca_26289	NON_SYNONYMOUS_CODING	NA
Kabuli_wild_152763	1399946330	*Ca_kabuli_scaffold*	152763	C/A	NA	INTERGENIC	NA
Kabuli_wild_13807	1399946331	*Ca_kabuli_scaffold*	13807	G/A	NA	INTERGENIC	NA
Kabuli_wild_13850	1399946332	*Ca_kabuli_scaffold*	13850	G/C	NA	INTERGENIC	NA
Kabuli_wild_13855	1399946333	*Ca_kabuli_scaffold*	13855	G/A	NA	INTERGENIC	NA
Kabuli_wild_13858	1399946334	*Ca_kabuli_scaffold*	13858	G/T	NA	INTERGENIC	NA
Kabuli_wild_13874	1399946335	*Ca_kabuli_scaffold*	13874	G/A	NA	INTERGENIC	NA
Kabuli_wild_13889	1399946336	*Ca_kabuli_scaffold*	13889	C/T	NA	INTERGENIC	NA
Kabuli_wild_13891	1399946337	*Ca_kabuli_scaffold*	13891	G/T	NA	INTERGENIC	NA
Kabuli_wild_118779	1399946338	*Ca_kabuli_scaffold*	118779	G/T	Gene_Ca_26141	INTRON	NA
Kabuli_wild_118782	1399946339	*Ca_kabuli_scaffold*	118782	C/T	Gene_Ca_26141	INTRON	NA
Kabuli_wild_118967	1399946340	*Ca_kabuli_scaffold*	118967	G/C	Gene_Ca_26141	INTRON	NA
Kabuli_wild_118972	1399946341	*Ca_kabuli_scaffold*	118972	A/G	Gene_Ca_26141	INTRON	NA
Kabuli_wild_118983	1399946342	*Ca_kabuli_scaffold*	118983	C/A	Gene_Ca_26141	INTRON	NA
Kabuli_wild_139538	1399946343	*Ca_kabuli_scaffold*	139538	G/A	NA	INTERGENIC	NA
Kabuli_wild_139539	1399946344	*Ca_kabuli_scaffold*	139539	A/G	NA	INTERGENIC	NA
Kabuli_wild_139541	1399946345	*Ca_kabuli_scaffold*	139541	T/C	NA	INTERGENIC	NA
Kabuli_wild_139544	1399946346	*Ca_kabuli_scaffold*	139544	C/T	NA	INTERGENIC	NA
Kabuli_wild_139545	1399946347	*Ca_kabuli_scaffold*	139545	G/A	NA	INTERGENIC	NA
Kabuli_wild_139548	1399946348	*Ca_kabuli_scaffold*	139548	C/T	NA	INTERGENIC	NA
Kabuli_wild_139549	1399946349	*Ca_kabuli_scaffold*	139549	C/T	NA	INTERGENIC	NA
Kabuli_wild_139560	1399946350	*Ca_kabuli_scaffold*	139560	G/T	NA	INTERGENIC	NA
Kabuli_wild_139562	1399946351	*Ca_kabuli_scaffold*	139562	C/T	NA	INTERGENIC	NA
Kabuli_wild_139574	1399946352	*Ca_kabuli_scaffold*	139574	T/A	NA	INTERGENIC	NA
Kabuli_wild_139575	1399946353	*Ca_kabuli_scaffold*	139575	C/T	NA	INTERGENIC	NA
Kabuli_wild_139581	1399946354	*Ca_kabuli_scaffold*	139581	G/A	NA	INTERGENIC	NA
Kabuli_wild_139584	1399946355	*Ca_kabuli_scaffold*	139584	G/A	NA	INTERGENIC	NA
Kabuli_wild_139596	1399946356	*Ca_kabuli_scaffold*	139596	A/G	NA	INTERGENIC	NA
Kabuli_wild_139597	1399946357	*Ca_kabuli_scaffold*	139597	A/G	NA	INTERGENIC	NA
Kabuli_wild_139603	1399946358	*Ca_kabuli_scaffold*	139603	C/T	NA	INTERGENIC	NA
Kabuli_wild_139604	1399946359	*Ca_kabuli_scaffold*	139604	T/C	NA	INTERGENIC	NA
Kabuli_wild_139609	1399946360	*Ca_kabuli_scaffold*	139609	C/T	NA	INTERGENIC	NA
Kabuli_wild_19478	1399946361	*Ca_kabuli_scaffold*	19478	C/T	NA	INTERGENIC	NA
Kabuli_wild_22105	1399946362	*Ca_kabuli_scaffold*	22105	A/G	Gene_Ca_25400	INTRON	NA
Kabuli_wild_22115	1399946363	*Ca_kabuli_scaffold*	22115	C/T	Gene_Ca_25400	INTRON	NA
Kabuli_wild_22174	1399946364	*Ca_kabuli_scaffold*	22174	G/A	Gene_Ca_25400	INTRON	NA
Kabuli_wild_22188	1399946365	*Ca_kabuli_scaffold*	22188	G/A	Gene_Ca_25400	INTRON	NA
Kabuli_wild_22189	1399946366	*Ca_kabuli_scaffold*	22189	G/A	Gene_Ca_25400	INTRON	NA
Kabuli_wild_437	1399946367	*Ca_kabuli_scaffold*	437	C/A	NA	INTERGENIC	NA

SNP IDs	NCBI dbSNP accession IDs (NCBI_SS#)	Chromosomes	Physical positions (bp)	SNPs	Gene accession IDs	Sequence components of genome	Putative functions
Kabuli_wild_6778	1399946368	*Ca_kabuli_scaffold*	6778	C/A	NA	INTERGENIC	NA
Kabuli_wild_6800	1399946369	*Ca_kabuli_scaffold*	6800	C/T	NA	INTERGENIC	NA
Kabuli_wild_6802	1399946370	*Ca_kabuli_scaffold*	6802	G/T	NA	INTERGENIC	NA
Kabuli_wild_6804	1399946371	*Ca_kabuli_scaffold*	6804	T/C	NA	INTERGENIC	NA
Kabuli_wild_6814	1399946372	*Ca_kabuli_scaffold*	6814	G/T	NA	INTERGENIC	NA
Kabuli_wild_61670	1399946373	*Ca_kabuli_scaffold*	61670	A/G	NA	INTERGENIC	NA
Kabuli_wild_61705	1399946374	*Ca_kabuli_scaffold*	61705	C/T	NA	INTERGENIC	NA
Kabuli_wild_61710	1399946375	*Ca_kabuli_scaffold*	61710	C/T	NA	INTERGENIC	NA
Kabuli_wild_61723	1399946376	*Ca_kabuli_scaffold*	61723	A/G	NA	INTERGENIC	NA
Kabuli_wild_61745	1399946377	*Ca_kabuli_scaffold*	61745	T/G	NA	INTERGENIC	NA
Kabuli_wild_61757	1399946378	*Ca_kabuli_scaffold*	61757	T/C	NA	INTERGENIC	NA
Kabuli_wild_372404	1399946379	*Ca_kabuli_scaffold*	372404	A/T	Gene_Ca_24482	DOWNSTREAM	Signal transduction response regulator, receiverdomain
Kabuli_wild_36698	1399946380	*Ca_kabuli_scaffold*	36698	T/C	NA	INTERGENIC	NA
Kabuli_wild_36759	1399946381	*Ca_kabuli_scaffold*	36759	T/G	NA	INTERGENIC	NA
Kabuli_wild_36782	1399946382	*Ca_kabuli_scaffold*	36782	C/T	NA	INTERGENIC	NA
Kabuli_wild_36810	1399946383	*Ca_kabuli_scaffold*	36810	T/C	NA	INTERGENIC	NA
Kabuli_wild_36837	1399946384	*Ca_kabuli_scaffold*	36837	G/A	NA	INTERGENIC	NA
Kabuli_wild_166615	1399946385	*Ca_kabuli_scaffold*	166615	T/C	NA	INTERGENIC	NA
Kabuli_wild_166660	1399946386	*Ca_kabuli_scaffold*	166660	C/T	NA	INTERGENIC	NA
Kabuli_wild_171183	1399946387	*Ca_kabuli_scaffold*	171183	G/T	NA	INTERGENIC	NA
Kabuli_wild_171197	1399946388	*Ca_kabuli_scaffold*	171197	A/G	NA	INTERGENIC	NA
Kabuli_wild_226179	1399946389	*Ca_kabuli_scaffold*	226179	C/A	NA	INTERGENIC	NA
Kabuli_wild_226210	1399946390	*Ca_kabuli_scaffold*	226210	G/A	NA	INTERGENIC	NA
Kabuli_wild_226219	1399946391	*Ca_kabuli_scaffold*	226219	T/C	NA	INTERGENIC	NA
Kabuli_wild_226233	1399946392	*Ca_kabuli_scaffold*	226233	G/T	NA	INTERGENIC	NA
Kabuli_wild_147568	1399946393	*Ca_kabuli_scaffold*	147568	C/T	Gene_Ca_25015	NON_SYNONYMOUS_CODING	Peptidase C48, SUMO/Sentrin/Ubl1
Kabuli_wild_147583	1399946394	*Ca_kabuli_scaffold*	147583	G/A	Gene_Ca_25015	NON_SYNONYMOUS_CODING	Peptidase C48, SUMO/Sentrin/Ubl1
Kabuli_wild_228608	1399946395	*Ca_kabuli_scaffold*	228608	G/A	NA	INTERGENIC	NA
Kabuli_wild_228653	1399946396	*Ca_kabuli_scaffold*	228653	G/C	NA	INTERGENIC	NA
Kabuli_wild_4821	1399946397	*Ca_kabuli_scaffold*	4821	A/G	Gene_Ca_26928	UPSTREAM	NA
Kabuli_wild_2097	1399946398	*Ca_kabuli_scaffold*	2097	C/A	NA	INTERGENIC	NA
Kabuli_wild_2100	1399946399	*Ca_kabuli_scaffold*	2100	G/C	NA	INTERGENIC	NA
Kabuli_wild_52375	1399946400	*Ca_kabuli_scaffold*	52375	C/A	NA	INTERGENIC	NA
Kabuli_wild_52435	1399946401	*Ca_kabuli_scaffold*	52435	G/A	NA	INTERGENIC	NA
Kabuli_wild_199111	1399946402	*Ca_kabuli_scaffold*	199111	C/T	NA	INTERGENIC	NA
Kabuli_wild_199119	1399946403	*Ca_kabuli_scaffold*	199119	T/G	NA	INTERGENIC	NA
Kabuli_wild_199120	1399946404	*Ca_kabuli_scaffold*	199120	C/A	NA	INTERGENIC	NA
Kabuli_wild_199145	1399946405	*Ca_kabuli_scaffold*	199145	G/A	NA	INTERGENIC	NA
Kabuli_wild_199169	1399946406	*Ca_kabuli_scaffold*	199169	G/T	NA	INTERGENIC	NA
Kabuli_wild_122741	1399946407	*Ca_kabuli_scaffold*	122741	T/C	NA	INTERGENIC	NA
Kabuli_wild_646	1399946408	*Ca_kabuli_scaffold*	646	G/A	NA	INTERGENIC	NA
Kabuli_wild_651	1399946409	*Ca_kabuli_scaffold*	651	G/T	NA	INTERGENIC	NA
Kabuli_wild_682	1399946410	*Ca_kabuli_scaffold*	682	A/C	NA	INTERGENIC	NA
Kabuli_wild_691	1399946411	*Ca_kabuli_scaffold*	691	G/C	NA	INTERGENIC	NA

SNP IDs	NCBI dbSNP accession IDs (NCBI_SS#)	Chromosomes	Physical positions (bp)	SNPs	Gene accession IDs	Sequence components of genome	Putative functions
Kabuli_wild_2002	1399946412	*Ca_kabuli_scaffold*	2002	C/G	NA	INTERGENIC	NA
Kabuli_wild_5520	1399946413	*Ca_kabuli_scaffold*	5520	C/A	NA	INTERGENIC	NA
Kabuli_wild_5538	1399946414	*Ca_kabuli_scaffold*	5538	A/T	NA	INTERGENIC	NA
Kabuli_wild_5562	1399946415	*Ca_kabuli_scaffold*	5562	C/T	NA	INTERGENIC	NA
Kabuli_wild_5570	1399946416	*Ca_kabuli_scaffold*	5570	T/C	NA	INTERGENIC	NA
Kabuli_wild_4436	1399946417	*Ca_kabuli_scaffold*	4436	A/C	NA	INTERGENIC	NA
Kabuli_wild_4446	1399946418	*Ca_kabuli_scaffold*	4446	A/C	NA	INTERGENIC	NA
Kabuli_wild_58959	1399946419	*Ca_kabuli_scaffold*	58959	G/A	Gene_Ca_27480	SYNONYMOUS_CODING	Peptidase C48, SUMO/Sentrin/Ubl1
Kabuli_wild_58974	1399946420	*Ca_kabuli_scaffold*	58974	C/T	Gene_Ca_27480	NON_SYNONYMOUS_CODING	Peptidase C48, SUMO/Sentrin/Ubl1
Kabuli_wild_58091	1399946421	*Ca_kabuli_scaffold*	58091	A/T	NA	INTERGENIC	NA
Kabuli_wild_123799	1399946422	*Ca_kabuli_scaffold*	123799	A/C	Gene_Ca_27079	SYNONYMOUS_CODING	RNA recognition motif domain
Kabuli_wild_126013	1399946423	*Ca_kabuli_scaffold*	126013	A/C	Gene_Ca_27079	SYNONYMOUS_CODING	RNA recognition motif domain
Kabuli_wild_70721	1399946424	*Ca_kabuli_scaffold*	70721	A/G	NA	INTERGENIC	NA
Kabuli_wild_207	1399946425	*Ca_kabuli_scaffold*	207	C/T	NA	INTERGENIC	NA
Kabuli_wild_840	1399946426	*Ca_kabuli_scaffold*	840	G/A	NA	INTERGENIC	NA
Kabuli_wild_845	1399946427	*Ca_kabuli_scaffold*	845	G/T	NA	INTERGENIC	NA
Kabuli_wild_876	1399946428	*Ca_kabuli_scaffold*	876	A/C	NA	INTERGENIC	NA
Kabuli_wild_885	1399946429	*Ca_kabuli_scaffold*	885	G/C	NA	INTERGENIC	NA
Kabuli_wild_895	1399946430	*Ca_kabuli_scaffold*	895	A/T	NA	INTERGENIC	NA
Kabuli_wild_901	1399946431	*Ca_kabuli_scaffold*	901	A/C	NA	INTERGENIC	NA
Kabuli_wild_50806	1399946432	*Ca_kabuli_scaffold*	50806	A/G	NA	INTERGENIC	NA
Kabuli_wild_65482	1399946433	*Ca_kabuli_scaffold*	65482	T/G	Gene_Ca_25421	UPSTREAM	Protein kinase,catalytic domain
Kabuli_wild_65617	1399946434	*Ca_kabuli_scaffold*	65617	G/A	Gene_Ca_25421	UPSTREAM	Protein kinase,catalytic domain
Kabuli_wild_83112	1399946435	*Ca_kabuli_scaffold*	83112	A/G	NA	INTERGENIC	NA
Kabuli_wild_502	1399946436	*Ca_kabuli_scaffold*	502	A/G	NA	INTERGENIC	NA
Kabuli_wild_222695	1399946437	*Ca_kabuli_scaffold*	222695	C/A	Gene_Ca_24349	NON_SYNONYMOUS_CODING	NA
Kabuli_wild_222734	1399946438	*Ca_kabuli_scaffold*	222734	T/C	Gene_Ca_24349	NON_SYNONYMOUS_CODING	NA
Kabuli_wild_222737	1399946439	*Ca_kabuli_scaffold*	222737	G/A	Gene_Ca_24349	NON_SYNONYMOUS_CODING	NA
Kabuli_wild_222749	1399946440	*Ca_kabuli_scaffold*	222749	G/C	Gene_Ca_24349	NON_SYNONYMOUS_CODING	NA
Kabuli_wild_222750	1399946441	*Ca_kabuli_scaffold*	222750	T/G	Gene_Ca_24349	NON_SYNONYMOUS_CODING	NA
Kabuli_wild_139149	1399946442	*Ca_kabuli_scaffold*	139149	A/G	NA	INTERGENIC	NA
Kabuli_wild_1920	1399946443	*Ca_kabuli_scaffold*	1920	T/C	NA	INTERGENIC	NA
Kabuli_wild_1923	1399946444	*Ca_kabuli_scaffold*	1923	C/T	NA	INTERGENIC	NA
Kabuli_wild_1929	1399946445	*Ca_kabuli_scaffold*	1929	C/T	NA	INTERGENIC	NA
Kabuli_wild_3718	1399946446	*Ca_kabuli_scaffold*	3718	C/T	NA	INTERGENIC	NA
Kabuli_wild_3793	1399946447	*Ca_kabuli_scaffold*	3793	C/T	NA	INTERGENIC	NA
Kabuli_wild_3811	1399946448	*Ca_kabuli_scaffold*	3811	C/T	NA	INTERGENIC	NA
Kabuli_wild_3812	1399946449	*Ca_kabuli_scaffold*	3812	C/A	NA	INTERGENIC	NA
Kabuli_wild_3815	1399946450	*Ca_kabuli_scaffold*	3815	T/A	NA	INTERGENIC	NA
Kabuli_wild_3858	1399946451	*Ca_kabuli_scaffold*	3858	G/A	NA	INTERGENIC	NA
Kabuli_wild_3862	1399946452	*Ca_kabuli_scaffold*	3862	T/C	NA	INTERGENIC	NA
Kabuli_wild_3871	1399946453	*Ca_kabuli_scaffold*	3871	A/T	NA	INTERGENIC	NA
Kabuli_wild_3923	1399946454	*Ca_kabuli_scaffold*	3923	T/C	NA	INTERGENIC	NA
Kabuli_wild_12916	1399946455	*Ca_kabuli_scaffold*	12916	C/A	NA	INTERGENIC	NA

SNP IDs	NCBI dbSNP accession IDs (NCBI_SS#)	Chromosomes	Physical positions (bp)	SNPs	Gene accession IDs	Sequence components of genome	Putative functions
Kabuli_wild_12969	1399946456	*Ca_kabuli_scaffold*	12969	G/C	NA	INTERGENIC	NA
Kabuli_wild_12978	1399946457	*Ca_kabuli_scaffold*	12978	A/T	NA	INTERGENIC	NA
Kabuli_wild_12981	1399946458	*Ca_kabuli_scaffold*	12981	C/T	NA	INTERGENIC	NA
Kabuli_wild_62433	1399946459	*Ca_kabuli_scaffold*	62433	A/G	Gene_Ca_27074	UPSTREAM	NA
Kabuli_wild_62473	1399946460	*Ca_kabuli_scaffold*	62473	G/A	Gene_Ca_27074	UPSTREAM	NA
Kabuli_wild_62494	1399946461	*Ca_kabuli_scaffold*	62494	A/G	Gene_Ca_27074	UPSTREAM	NA
Kabuli_wild_62495	1399946462	*Ca_kabuli_scaffold*	62495	C/T	Gene_Ca_27074	UPSTREAM	NA
Kabuli_wild_62496	1399946463	*Ca_kabuli_scaffold*	62496	G/C	Gene_Ca_27074	UPSTREAM	NA
Kabuli_wild_62502	1399946464	*Ca_kabuli_scaffold*	62502	T/C	Gene_Ca_27074	UPSTREAM	NA
Kabuli_wild_62514	1399946465	*Ca_kabuli_scaffold*	62514	C/G	Gene_Ca_27074	UPSTREAM	NA
Kabuli_wild_62527	1399946466	*Ca_kabuli_scaffold*	62527	A/G	Gene_Ca_27074	UPSTREAM	NA
Kabuli_wild_62538	1399946467	*Ca_kabuli_scaffold*	62538	C/T	Gene_Ca_27074	UPSTREAM	NA
Kabuli_wild_62545	1399946468	*Ca_kabuli_scaffold*	62545	G/A	Gene_Ca_27074	UPSTREAM	NA
Kabuli_wild_127851	1399946469	*Ca_kabuli_scaffold*	127851	C/T	NA	INTERGENIC	NA
Kabuli_wild_127855	1399946470	*Ca_kabuli_scaffold*	127855	A/C	NA	INTERGENIC	NA
Kabuli_wild_127870	1399946471	*Ca_kabuli_scaffold*	127870	T/G	NA	INTERGENIC	NA
Kabuli_wild_127884	1399946472	*Ca_kabuli_scaffold*	127884	A/C	NA	INTERGENIC	NA
Kabuli_wild_127905	1399946473	*Ca_kabuli_scaffold*	127905	C/T	NA	INTERGENIC	NA
Kabuli_wild_29923	1399946474	*Ca_kabuli_scaffold*	29923	C/G	NA	INTERGENIC	NA
Kabuli_wild_35402	1399946475	*Ca_kabuli_scaffold*	35402	A/C	Gene_Ca_25129	INTRON	NA
Kabuli_wild_198120	1399946476	*Ca_kabuli_scaffold*	198120	G/T	NA	INTERGENIC	NA
Kabuli_wild_297848	1399946477	*Ca_kabuli_scaffold*	297848	T/C	NA	INTERGENIC	NA
Kabuli_wild_297884	1399946478	*Ca_kabuli_scaffold*	297884	T/C	NA	INTERGENIC	NA
Kabuli_wild_314310	1399946479	*Ca_kabuli_scaffold*	314310	G/A	NA	INTERGENIC	NA
Kabuli_wild_314322	1399946480	*Ca_kabuli_scaffold*	314322	T/C	NA	INTERGENIC	NA
Kabuli_wild_314323	1399946481	*Ca_kabuli_scaffold*	314323	G/A	NA	INTERGENIC	NA
Kabuli_wild_314329	1399946482	*Ca_kabuli_scaffold*	314329	T/C	NA	INTERGENIC	NA
Kabuli_wild_314343	1399946483	*Ca_kabuli_scaffold*	314343	G/A	NA	INTERGENIC	NA
Kabuli_wild_314344	1399946484	*Ca_kabuli_scaffold*	314344	T/C	NA	INTERGENIC	NA
Kabuli_wild_314347	1399946485	*Ca_kabuli_scaffold*	314347	C/T	NA	INTERGENIC	NA
Kabuli_wild_314349	1399946486	*Ca_kabuli_scaffold*	314349	C/G	NA	INTERGENIC	NA
Kabuli_wild_314353	1399946487	*Ca_kabuli_scaffold*	314353	G/A	NA	INTERGENIC	NA
Kabuli_wild_314361	1399946488	*Ca_kabuli_scaffold*	314361	T/G	NA	INTERGENIC	NA
Kabuli_wild_314363	1399946489	*Ca_kabuli_scaffold*	314363	C/A	NA	INTERGENIC	NA
Kabuli_wild_314401	1399946490	*Ca_kabuli_scaffold*	314401	A/C	NA	INTERGENIC	NA
Kabuli_wild_28439	1399946491	*Ca_kabuli_scaffold*	28439	C/T	NA	INTERGENIC	NA
Kabuli_wild_226	1399946492	*Ca_kabuli_scaffold*	226	T/G	NA	INTERGENIC	NA
Kabuli_wild_242	1399946493	*Ca_kabuli_scaffold*	242	C/G	NA	INTERGENIC	NA
Kabuli_wild_251	1399946494	*Ca_kabuli_scaffold*	251	T/G	NA	INTERGENIC	NA
Kabuli_wild_282	1399946495	*Ca_kabuli_scaffold*	282	C/A	NA	INTERGENIC	NA
Kabuli_wild_287	1399946496	*Ca_kabuli_scaffold*	287	C/T	NA	INTERGENIC	NA
Kabuli_wild_3789	1399946497	*Ca_kabuli_scaffold*	3789	C/T	NA	INTERGENIC	NA
Kabuli_wild_58947	1399946498	*Ca_kabuli_scaffold*	58947	T/C	Gene_Ca_26451	SYNONYMOUS_CODING	Domain of unknown function DUF125,transmembrane
Kabuli_wild_57613	1399946499	*Ca_kabuli_scaffold*	57613	C/T	Gene_Ca_27549	NON_SYNONYMOUS_CODING	NA

SNP IDs	NCBI dbSNP accession IDs (NCBI_SS#)	Chromosomes	Physical positions (bp)	SNPs	Gene accession IDs	Sequence components of genome	Putative functions
Kabuli_wild_57626	1399946500	*Ca_kabuli_scaffold*	57626	G/A	Gene_Ca_27549	SYNONYMOUS_CODING	NA
Kabuli_wild_57659	1399946501	*Ca_kabuli_scaffold*	57659	C/T	Gene_Ca_27549	NON_SYNONYMOUS_CODING	NA
Kabuli_wild_57709	1399946502	*Ca_kabuli_scaffold*	57709	G/T	Gene_Ca_27549	NON_SYNONYMOUS_CODING	NA
Kabuli_wild_57725	1399946503	*Ca_kabuli_scaffold*	57725	G/A	Gene_Ca_27549	NON_SYNONYMOUS_CODING	NA
Kabuli_wild_57726	1399946504	*Ca_kabuli_scaffold*	57726	C/A	Gene_Ca_27549	NON_SYNONYMOUS_CODING	NA
Kabuli_wild_57742	1399946505	*Ca_kabuli_scaffold*	57742	T/A	Gene_Ca_27549	NON_SYNONYMOUS_CODING	NA
Kabuli_wild_57762	1399946506	*Ca_kabuli_scaffold*	57762	T/C	Gene_Ca_27549	SYNONYMOUS_CODING	NA
Kabuli_wild_67792	1399946507	*Ca_kabuli_scaffold*	67792	T/G	NA	INTERGENIC	NA
Kabuli_wild_3107	1399946508	*Ca_kabuli_scaffold*	3107	T/C	NA	INTERGENIC	NA
Kabuli_wild_3149	1399946509	*Ca_kabuli_scaffold*	3149	A/C	NA	INTERGENIC	NA
Kabuli_wild_110378	1399946510	*Ca_kabuli_scaffold*	110378	C/T	Gene_Ca_26665	NON_SYNONYMOUS_CODING	Domain of unknown function DUF828
Kabuli_wild_110414	1399946511	*Ca_kabuli_scaffold*	110414	T/G	Gene_Ca_26665	NON_SYNONYMOUS_CODING	Domain of unknown function DUF828
Kabuli_wild_110437	1399946512	*Ca_kabuli_scaffold*	110437	C/T	NA	INTERGENIC	NA
Kabuli_wild_110441	1399946513	*Ca_kabuli_scaffold*	110441	T/A	NA	INTERGENIC	NA
Kabuli_wild_58152	1399946514	*Ca_kabuli_scaffold*	58152	T/G	Gene_Ca_26161	SYNONYMOUS_CODING	WD40 repeat
Kabuli_wild_195270	1399946515	*Ca_kabuli_scaffold*	195270	C/T	NA	INTERGENIC	NA
Kabuli_wild_195276	1399946516	*Ca_kabuli_scaffold*	195276	A/C	NA	INTERGENIC	NA
Kabuli_wild_195292	1399946517	*Ca_kabuli_scaffold*	195292	G/T	NA	INTERGENIC	NA
Kabuli_wild_195305	1399946518	*Ca_kabuli_scaffold*	195305	G/A	NA	INTERGENIC	NA
Kabuli_wild_467848	1399946519	*Ca_kabuli_scaffold*	467848	C/T	NA	INTERGENIC	NA
Kabuli_wild_25377	1399946520	*Ca_kabuli_scaffold*	25377	G/A	NA	INTERGENIC	NA
Kabuli_wild_1375	1399946521	*Ca_kabuli_scaffold*	1375	G/A	NA	INTERGENIC	NA
Kabuli_wild_145570	1399946522	*Ca_kabuli_scaffold*	145570	T/C	Gene_Ca_27567	SYNONYMOUS_CODING	NA
Kabuli_wild_30682	1399946523	*Ca_kabuli_scaffold*	30682	C/T	Gene_Ca_26033	NON_SYNONYMOUS_CODING	Peptidase C48, SUMO/Sentrin/Ubl1
Kabuli_wild_30766	1399946524	*Ca_kabuli_scaffold*	30766	C/T	Gene_Ca_26033	NON_SYNONYMOUS_CODING	Peptidase C48, SUMO/Sentrin/Ubl1
Kabuli_wild_177550	1399946525	*Ca_kabuli_scaffold*	177550	G/T	NA	INTERGENIC	NA
Kabuli_wild_177578	1399946526	*Ca_kabuli_scaffold*	177578	T/C	NA	INTERGENIC	NA
Kabuli_wild_177579	1399946527	*Ca_kabuli_scaffold*	177579	T/C	NA	INTERGENIC	NA
Kabuli_wild_24846	1399946528	*Ca_kabuli_scaffold*	24846	G/A	NA	INTERGENIC	NA
Kabuli_wild_45019	1399946529	*Ca_kabuli_scaffold*	45019	C/T	Gene_Ca_27214	NON_SYNONYMOUS_CODING	NA
Kabuli_wild_52397	1399946530	*Ca_kabuli_scaffold*	52397	G/T	NA	INTERGENIC	NA
Kabuli_wild_52416	1399946531	*Ca_kabuli_scaffold*	52416	G/T	NA	INTERGENIC	NA
Kabuli_wild_75489	1399946532	*Ca_kabuli_scaffold*	75489	T/G	Gene_Ca_27216	NON_SYNONYMOUS_CODING	Glycosidehydrolase,family28
Kabuli_wild_75501	1399946533	*Ca_kabuli_scaffold*	75501	C/A	Gene_Ca_27216	NON_SYNONYMOUS_CODING	Glycosidehydrolase,family28
Kabuli_wild_75546	1399946534	*Ca_kabuli_scaffold*	75546	T/G	Gene_Ca_27216	NON_SYNONYMOUS_CODING	Glycosidehydrolase,family28
Kabuli_wild_75609	1399946535	*Ca_kabuli_scaffold*	75609	A/G	Gene_Ca_27216	NON_SYNONYMOUS_CODING	Glycosidehydrolase,family28
Kabuli_wild_75616	1399946536	*Ca_kabuli_scaffold*	75616	T/G	Gene_Ca_27216	NON_SYNONYMOUS_CODING	Glycosidehydrolase,family28
Kabuli_wild_418	1399946537	*Ca_kabuli_scaffold*	418	T/A	NA	INTERGENIC	NA
Kabuli_wild_980	1399946538	*Ca_kabuli_scaffold*	980	A/G	NA	INTERGENIC	NA
Kabuli_wild_1172	1399946539	*Ca_kabuli_scaffold*	1172	C/T	NA	INTERGENIC	NA
Kabuli_wild_1202	1399946540	*Ca_kabuli_scaffold*	1202	A/G	NA	INTERGENIC	NA
Kabuli_wild_1204	1399946541	*Ca_kabuli_scaffold*	1204	G/A	NA	INTERGENIC	NA
Kabuli_wild_1307	1399946542	*Ca_kabuli_scaffold*	1307	G/A	NA	INTERGENIC	NA
Kabuli_wild_4688	1399946543	*Ca_kabuli_scaffold*	4688	A/C	Gene_Ca_24392	SYNONYMOUS_CODING	Glycosidehydrolase,family9

SNP IDs	NCBI dbSNP accession IDs (NCBI_SS#)	Chromosomes	Physical positions (bp)	SNPs	Gene accession IDs	Sequence components of genome	Putative functions
Kabuli_wild_4706	1399946544	*Ca_kabuli_scaffold*	4706	G/T	Gene_Ca_24392	NON_SYNONYMOUS_CODING	Glycosidehydrolase,family9
Kabuli_wild_4719	1399946545	*Ca_kabuli_scaffold*	4719	G/A	Gene_Ca_24392	INTRON	Glycosidehydrolase,family9
Kabuli_wild_4732	1399946546	*Ca_kabuli_scaffold*	4732	C/G	Gene_Ca_24392	INTRON	Glycosidehydrolase,family9
Kabuli_wild_4738	1399946547	*Ca_kabuli_scaffold*	4738	C/G	Gene_Ca_24392	INTRON	Glycosidehydrolase,family9
Kabuli_wild_4782	1399946548	*Ca_kabuli_scaffold*	4782	C/T	Gene_Ca_24392	INTRON	Glycosidehydrolase,family9
Kabuli_wild_25756	1399946549	*Ca_kabuli_scaffold*	25756	T/G	Gene_Ca_24394	SYNONYMOUS_CODING	NA
Kabuli_wild_25769	1399946550	*Ca_kabuli_scaffold*	25769	A/G	Gene_Ca_24394	NON_SYNONYMOUS_CODING	NA
Kabuli_wild_25771	1399946551	*Ca_kabuli_scaffold*	25771	T/G	Gene_Ca_24394	NON_SYNONYMOUS_CODING	NA
Kabuli_wild_43207	1399946552	*Ca_kabuli_scaffold*	43207	G/A	NA	INTERGENIC	NA
Kabuli_wild_49533	1399946553	*Ca_kabuli_scaffold*	49533	T/A	Gene_Ca_24396	INTRON	Zinc finger, RING-type
Kabuli_wild_49700	1399946554	*Ca_kabuli_scaffold*	49700	A/G	Gene_Ca_24396	INTRON	Zinc finger, RING-type
Kabuli_wild_82193	1399946555	*Ca_kabuli_scaffold*	82193	C/A	NA	INTERGENIC	NA
Kabuli_wild_82232	1399946556	*Ca_kabuli_scaffold*	82232	C/T	NA	INTERGENIC	NA
Kabuli_wild_82249	1399946557	*Ca_kabuli_scaffold*	82249	G/A	NA	INTERGENIC	NA
Kabuli_wild_82250	1399946558	*Ca_kabuli_scaffold*	82250	T/C	NA	INTERGENIC	NA
Kabuli_wild_82253	1399946559	*Ca_kabuli_scaffold*	82253	G/C	NA	INTERGENIC	NA
Kabuli_wild_82261	1399946560	*Ca_kabuli_scaffold*	82261	C/G	NA	INTERGENIC	NA
Kabuli_wild_82262	1399946561	*Ca_kabuli_scaffold*	82262	G/C	NA	INTERGENIC	NA
Kabuli_wild_82302	1399946562	*Ca_kabuli_scaffold*	82302	T/A	Gene_Ca_24399	NON_SYNONYMOUS_CODING	NA
Kabuli_wild_82319	1399946563	*Ca_kabuli_scaffold*	82319	T/C	Gene_Ca_24399	SYNONYMOUS_CODING	NA
Kabuli_wild_82328	1399946564	*Ca_kabuli_scaffold*	82328	A/G	Gene_Ca_24399	SYNONYMOUS_CODING	NA
Kabuli_wild_82342	1399946565	*Ca_kabuli_scaffold*	82342	C/T	Gene_Ca_24399	NON_SYNONYMOUS_CODING	NA
Kabuli_wild_82369	1399946566	*Ca_kabuli_scaffold*	82369	G/A	Gene_Ca_24399	STOP_GAINED	NA
Kabuli_wild_82397	1399946567	*Ca_kabuli_scaffold*	82397	T/C	Gene_Ca_24399	SYNONYMOUS_CODING	NA
Kabuli_wild_82417	1399946568	*Ca_kabuli_scaffold*	82417	C/T	Gene_Ca_24399	NON_SYNONYMOUS_CODING	NA
Kabuli_wild_95823	1399946569	*Ca_kabuli_scaffold*	95823	G/A	NA	INTERGENIC	NA
Kabuli_wild_99364	1399946570	*Ca_kabuli_scaffold*	99364	A/G	NA	INTERGENIC	NA
Kabuli_wild_99368	1399946571	*Ca_kabuli_scaffold*	99368	C/G	NA	INTERGENIC	NA
Kabuli_wild_99477	1399946572	*Ca_kabuli_scaffold*	99477	T/A	Gene_Ca_24401	DOWNSTREAM	NA
Kabuli_wild_99549	1399946573	*Ca_kabuli_scaffold*	99549	C/A	Gene_Ca_24401	DOWNSTREAM	NA
Kabuli_wild_100874	1399946574	*Ca_kabuli_scaffold*	100874	A/C	Gene_Ca_24400	DOWNSTREAM	NA
Kabuli_wild_100928	1399946575	*Ca_kabuli_scaffold*	100928	T/A	Gene_Ca_24400	DOWNSTREAM	NA
Kabuli_wild_101084	1399946576	*Ca_kabuli_scaffold*	101084	C/A	Gene_Ca_24400	DOWNSTREAM	NA
Kabuli_wild_101251	1399946577	*Ca_kabuli_scaffold*	101251	A/G	Gene_Ca_24400	DOWNSTREAM	NA
Kabuli_wild_104549	1399946578	*Ca_kabuli_scaffold*	104549	C/G	NA	INTERGENIC	NA
Kabuli_wild_104583	1399946579	*Ca_kabuli_scaffold*	104583	T/C	Gene_Ca_24401	NON_SYNONYMOUS_CODING	NA
Kabuli_wild_106920	1399946580	*Ca_kabuli_scaffold*	106920	C/T	Gene_Ca_24401	INTRON	NA
Kabuli_wild_106936	1399946581	*Ca_kabuli_scaffold*	106936	A/G	Gene_Ca_24401	INTRON	NA
Kabuli_wild_106974	1399946582	*Ca_kabuli_scaffold*	106974	C/T	Gene_Ca_24401	INTRON	NA
Kabuli_wild_107033	1399946583	*Ca_kabuli_scaffold*	107033	A/T	Gene_Ca_24401	INTRON	NA
Kabuli_wild_107087	1399946584	*Ca_kabuli_scaffold*	107087	G/A	Gene_Ca_24401	INTRON	NA
Kabuli_wild_107180	1399946585	*Ca_kabuli_scaffold*	107180	C/T	Gene_Ca_24401	INTRON	NA
Kabuli_wild_107376	1399946586	*Ca_kabuli_scaffold*	107376	C/T	Gene_Ca_24401	INTRON	NA
Kabuli_wild_107413	1399946587	*Ca_kabuli_scaffold*	107413	A/G	Gene_Ca_24401	INTRON	NA

SNP IDs	NCBI dbSNP accession IDs (NCBI_SS#)	Chromosomes	Physical positions (bp)	SNPs	Gene accession IDs	Sequence components of genome	Putative functions
Kabuli_wild_107437	1399946588	*Ca_kabuli_scaffold*	107437	T/C	Gene_Ca_24401	INTRON	NA
Kabuli_wild_108211	1399946589	*Ca_kabuli_scaffold*	108211	G/A	Gene_Ca_24401	INTRON	NA
Kabuli_wild_108560	1399946590	*Ca_kabuli_scaffold*	108560	T/G	Gene_Ca_24401	INTRON	NA
Kabuli_wild_108599	1399946591	*Ca_kabuli_scaffold*	108599	T/A	Gene_Ca_24401	INTRON	NA
Kabuli_wild_108763	1399946592	*Ca_kabuli_scaffold*	108763	T/G	Gene_Ca_24401	INTRON	NA
Kabuli_wild_112836	1399946593	*Ca_kabuli_scaffold*	112836	T/C	Gene_Ca_24401	INTRON	NA
Kabuli_wild_117430	1399946594	*Ca_kabuli_scaffold*	117430	A/C	NA	INTERGENIC	NA
Kabuli_wild_117440	1399946595	*Ca_kabuli_scaffold*	117440	C/T	NA	INTERGENIC	NA
Kabuli_wild_125716	1399946596	*Ca_kabuli_scaffold*	125716	T/C	Gene_Ca_24402	SYNONYMOUS_CODING	NA
Kabuli_wild_128027	1399946597	*Ca_kabuli_scaffold*	128027	A/G	Gene_Ca_24402	INTRON	NA
Kabuli_wild_133462	1399946598	*Ca_kabuli_scaffold*	133462	G/A	NA	INTERGENIC	NA
Kabuli_wild_56702	1399946599	*Ca_kabuli_scaffold*	56702	G/A	NA	INTERGENIC	NA
Kabuli_wild_89940	1399946600	*Ca_kabuli_scaffold*	89940	C/T	Gene_Ca_26646	SYNONYMOUS_CODING	Lactate/malatedehydrogenase,N-terminal
Kabuli_wild_25626	1399946601	*Ca_kabuli_scaffold*	25626	C/G	NA	INTERGENIC	NA
Kabuli_wild_25655	1399946602	*Ca_kabuli_scaffold*	25655	T/G	NA	INTERGENIC	NA
Kabuli_wild_25668	1399946603	*Ca_kabuli_scaffold*	25668	T/G	NA	INTERGENIC	NA
Kabuli_wild_210	1399946604	*Ca_kabuli_scaffold*	210	A/G	NA	INTERGENIC	NA
Kabuli_wild_213	1399946605	*Ca_kabuli_scaffold*	213	C/T	NA	INTERGENIC	NA
Kabuli_wild_225	1399946606	*Ca_kabuli_scaffold*	225	G/A	NA	INTERGENIC	NA
Kabuli_wild_227	1399946607	*Ca_kabuli_scaffold*	227	T/G	NA	INTERGENIC	NA
Kabuli_wild_234	1399946608	*Ca_kabuli_scaffold*	234	C/T	NA	INTERGENIC	NA
Kabuli_wild_239	1399946609	*Ca_kabuli_scaffold*	239	C/T	NA	INTERGENIC	NA
Kabuli_wild_240	1399946610	*Ca_kabuli_scaffold*	240	T/G	NA	INTERGENIC	NA
Kabuli_wild_243	1399946611	*Ca_kabuli_scaffold*	243	A/G	NA	INTERGENIC	NA
Kabuli_wild_246	1399946612	*Ca_kabuli_scaffold*	246	G/A	NA	INTERGENIC	NA
Kabuli_wild_256	1399946613	*Ca_kabuli_scaffold*	256	T/C	NA	INTERGENIC	NA
Kabuli_wild_258	1399946614	*Ca_kabuli_scaffold*	258	T/C	NA	INTERGENIC	NA
Kabuli_wild_276	1399946615	*Ca_kabuli_scaffold*	276	G/C	NA	INTERGENIC	NA
Kabuli_wild_283	1399946616	*Ca_kabuli_scaffold*	283	A/G	NA	INTERGENIC	NA
Kabuli_wild_285	1399946617	*Ca_kabuli_scaffold*	285	T/G	NA	INTERGENIC	NA
Kabuli_wild_291	1399946618	*Ca_kabuli_scaffold*	291	C/A	NA	INTERGENIC	NA
Kabuli_wild_2356	1399946619	*Ca_kabuli_scaffold*	2356	C/G	Gene_Ca_27862	SYNONYMOUS_CODING	Peptidase C48, SUMO/Sentrin/Ubl1
Kabuli_wild_2369	1399946620	*Ca_kabuli_scaffold*	2369	C/T	Gene_Ca_27862	SYNONYMOUS_CODING	Peptidase C48, SUMO/Sentrin/Ubl1
Kabuli_wild_2372	1399946621	*Ca_kabuli_scaffold*	2372	G/A	Gene_Ca_27862	NON_SYNONYMOUS_CODING	Peptidase C48, SUMO/Sentrin/Ubl1
Kabuli_wild_2388	1399946622	*Ca_kabuli_scaffold*	2388	C/T	Gene_Ca_27862	NON_SYNONYMOUS_CODING	Peptidase C48, SUMO/Sentrin/Ubl1
Kabuli_wild_2403	1399946623	*Ca_kabuli_scaffold*	2403	C/A	Gene_Ca_27862	NON_SYNONYMOUS_CODING	Peptidase C48, SUMO/Sentrin/Ubl1
Kabuli_wild_2409	1399946624	*Ca_kabuli_scaffold*	2409	A/G	Gene_Ca_27862	NON_SYNONYMOUS_CODING	Peptidase C48, SUMO/Sentrin/Ubl1
Kabuli_wild_2421	1399946625	*Ca_kabuli_scaffold*	2421	G/A	Gene_Ca_27862	NON_SYNONYMOUS_CODING	Peptidase C48, SUMO/Sentrin/Ubl1
Kabuli_wild_2444	1399946626	*Ca_kabuli_scaffold*	2444	C/A	Gene_Ca_27862	NON_SYNONYMOUS_CODING	Peptidase C48, SUMO/Sentrin/Ubl1
Kabuli_wild_2464	1399946627	*Ca_kabuli_scaffold*	2464	A/G	Gene_Ca_27862	SYNONYMOUS_CODING	Peptidase C48, SUMO/Sentrin/Ubl1
Kabuli_wild_2469	1399946628	*Ca_kabuli_scaffold*	2469	T/G	Gene_Ca_27862	NON_SYNONYMOUS_CODING	Peptidase C48, SUMO/Sentrin/Ubl1
Kabuli_wild_2489	1399946629	*Ca_kabuli_scaffold*	2489	C/A	Gene_Ca_27862	NON_SYNONYMOUS_CODING	Peptidase C48, SUMO/Sentrin/Ubl1
Kabuli_wild_2517	1399946630	*Ca_kabuli_scaffold*	2517	G/A	Gene_Ca_27862	NON_SYNONYMOUS_CODING	Peptidase C48, SUMO/Sentrin/Ubl1
Kabuli_wild_14852	1399946631	*Ca_kabuli_scaffold*	14852	C/G	Gene_Ca_28160	INTRON	NA

SNP IDs	NCBI dbSNP accession IDs (NCBI_SS#)	Chromosomes	Physical positions (bp)	SNPs	Gene accession IDs	Sequence components of genome	Putative functions
Kabuli_wild_14916	1399946632	*Ca_kabuli_scaffold*	14916	A/G	Gene_Ca_28160	INTRON	NA
Kabuli_wild_14917	1399946633	*Ca_kabuli_scaffold*	14917	G/A	Gene_Ca_28160	INTRON	NA
Kabuli_wild_15086	1399946634	*Ca_kabuli_scaffold*	15086	G/A	Gene_Ca_28160	INTRON	NA
Kabuli_wild_15153	1399946635	*Ca_kabuli_scaffold*	15153	G/A	Gene_Ca_28160	INTRON	NA
Kabuli_wild_15166	1399946636	*Ca_kabuli_scaffold*	15166	G/T	Gene_Ca_28160	INTRON	NA
Kabuli_wild_15170	1399946637	*Ca_kabuli_scaffold*	15170	G/T	Gene_Ca_28160	INTRON	NA
Kabuli_wild_15187	1399946638	*Ca_kabuli_scaffold*	15187	T/A	Gene_Ca_28160	INTRON	NA
Kabuli_wild_56358	1399946639	*Ca_kabuli_scaffold*	56358	T/G	NA	INTERGENIC	NA
Kabuli_wild_56451	1399946640	*Ca_kabuli_scaffold*	56451	A/T	NA	INTERGENIC	NA
Kabuli_wild_50195	1399946641	*Ca_kabuli_scaffold*	50195	G/C	NA	INTERGENIC	NA
Kabuli_wild_31202	1399946642	*Ca_kabuli_scaffold*	31202	G/A	Gene_Ca_27010	NON_SYNONYMOUS_CODING	NA
Kabuli_wild_31205	1399946643	*Ca_kabuli_scaffold*	31205	G/A	Gene_Ca_27010	NON_SYNONYMOUS_CODING	NA
Kabuli_wild_31211	1399946644	*Ca_kabuli_scaffold*	31211	G/A	Gene_Ca_27010	NON_SYNONYMOUS_CODING	NA
Kabuli_wild_31218	1399946645	*Ca_kabuli_scaffold*	31218	C/T	Gene_Ca_27010	NON_SYNONYMOUS_CODING	NA
Kabuli_wild_31269	1399946646	*Ca_kabuli_scaffold*	31269	G/A	Gene_Ca_27010	SYNONYMOUS_CODING	NA
Kabuli_wild_31286	1399946647	*Ca_kabuli_scaffold*	31286	G/T	Gene_Ca_27010	NON_SYNONYMOUS_CODING	NA
Kabuli_wild_48024	1399946648	*Ca_kabuli_scaffold*	48024	A/G	Gene_Ca_27011	SYNONYMOUS_CODING	NA
Kabuli_wild_20485	1399946649	*Ca_kabuli_scaffold*	20485	G/C	NA	INTERGENIC	NA
Kabuli_wild_20488	1399946650	*Ca_kabuli_scaffold*	20488	T/G	NA	INTERGENIC	NA
Kabuli_wild_20496	1399946651	*Ca_kabuli_scaffold*	20496	T/A	NA	INTERGENIC	NA
Kabuli_wild_20533	1399946652	*Ca_kabuli_scaffold*	20533	G/A	NA	INTERGENIC	NA
Kabuli_wild_20552	1399946653	*Ca_kabuli_scaffold*	20552	C/T	NA	INTERGENIC	NA
Kabuli_wild_4880	1399946654	*Ca_kabuli_scaffold*	4880	C/T	Gene_Ca_27574	DOWNSTREAM	NA
Kabuli_wild_263	1399946655	*Ca_kabuli_scaffold*	263	C/T	Gene_Ca_28080	UPSTREAM	Peptidase C48, SUMO/Sentrin/Ubl1
Kabuli_wild_269	1399946656	*Ca_kabuli_scaffold*	269	A/C	Gene_Ca_28080	UPSTREAM	Peptidase C48, SUMO/Sentrin/Ubl1
Kabuli_wild_272	1399946657	*Ca_kabuli_scaffold*	272	C/T	Gene_Ca_28080	UPSTREAM	Peptidase C48, SUMO/Sentrin/Ubl1
Kabuli_wild_32650	1399946658	*Ca_kabuli_scaffold*	32650	C/T	NA	INTERGENIC	NA
Kabuli_wild_32703	1399946659	*Ca_kabuli_scaffold*	32703	A/C	NA	INTERGENIC	NA
Kabuli_wild_32739	1399946660	*Ca_kabuli_scaffold*	32739	C/A	NA	INTERGENIC	NA
Kabuli_wild_32776	1399946661	*Ca_kabuli_scaffold*	32776	T/A	NA	INTERGENIC	NA
Kabuli_wild_33081	1399946662	*Ca_kabuli_scaffold*	33081	A/G	NA	INTERGENIC	NA
Kabuli_wild_33207	1399946663	*Ca_kabuli_scaffold*	33207	C/T	NA	INTERGENIC	NA
Kabuli_wild_33602	1399946664	*Ca_kabuli_scaffold*	33602	C/A	NA	INTERGENIC	NA
Kabuli_wild_33631	1399946665	*Ca_kabuli_scaffold*	33631	G/A	NA	INTERGENIC	NA
Kabuli_wild_33632	1399946666	*Ca_kabuli_scaffold*	33632	T/G	NA	INTERGENIC	NA
Kabuli_wild_33655	1399946667	*Ca_kabuli_scaffold*	33655	G/A	NA	INTERGENIC	NA
Kabuli_wild_319	1399946668	*Ca_kabuli_scaffold*	319	T/C	NA	INTERGENIC	NA
Kabuli_wild_375	1399946669	*Ca_kabuli_scaffold*	375	T/C	NA	INTERGENIC	NA
Kabuli_wild_7348	1399946670	*Ca_kabuli_scaffold*	7348	G/A	NA	INTERGENIC	NA
Kabuli_wild_7388	1399946671	*Ca_kabuli_scaffold*	7388	T/A	NA	INTERGENIC	NA
Kabuli_wild_53536	1399946672	*Ca_kabuli_scaffold*	53536	T/G	Gene_Ca_27261	SYNONYMOUS_CODING	Tetracyclineresistanceprotein,TetA
Kabuli_wild_32790	1399946673	*Ca_kabuli_scaffold*	32790	C/T	Gene_Ca_26723	SYNONYMOUS_CODING	NA
Kabuli_wild_33498	1399946674	*Ca_kabuli_scaffold*	33498	C/T	Gene_Ca_26723	INTRON	NA
Kabuli_wild_33499	1399946675	*Ca_kabuli_scaffold*	33499	T/A	Gene_Ca_26723	INTRON	NA

SNP IDs	NCBI dbSNP accession IDs (NCBI_SS#)	Chromosomes	Physical positions (bp)	SNPs	Gene accession IDs	Sequence components of genome	Putative functions
Kabuli_wild_33501	1399946676	*Ca_kabuli_scaffold*	33501	G/A	Gene_Ca_26723	INTRON	NA
Kabuli_wild_33502	1399946677	*Ca_kabuli_scaffold*	33502	A/C	Gene_Ca_26723	INTRON	NA
Kabuli_wild_33507	1399946678	*Ca_kabuli_scaffold*	33507	A/T	Gene_Ca_26723	INTRON	NA
Kabuli_wild_33513	1399946679	*Ca_kabuli_scaffold*	33513	A/T	Gene_Ca_26723	INTRON	NA
Kabuli_wild_33514	1399946680	*Ca_kabuli_scaffold*	33514	A/G	Gene_Ca_26723	INTRON	NA
Kabuli_wild_33517	1399946681	*Ca_kabuli_scaffold*	33517	A/T	Gene_Ca_26723	INTRON	NA
Kabuli_wild_33522	1399946682	*Ca_kabuli_scaffold*	33522	A/T	Gene_Ca_26723	INTRON	NA
Kabuli_wild_33529	1399946683	*Ca_kabuli_scaffold*	33529	T/C	Gene_Ca_26723	INTRON	NA
Kabuli_wild_33530	1399946684	*Ca_kabuli_scaffold*	33530	G/T	Gene_Ca_26723	INTRON	NA
Kabuli_wild_33531	1399946685	*Ca_kabuli_scaffold*	33531	T/A	Gene_Ca_26723	INTRON	NA
Kabuli_wild_33532	1399946686	*Ca_kabuli_scaffold*	33532	C/A	Gene_Ca_26723	INTRON	NA
Kabuli_wild_4400	1399946687	*Ca_kabuli_scaffold*	4400	G/A	Gene_Ca_28109	SYNONYMOUS_CODING	NA
Kabuli_wild_4405	1399946688	*Ca_kabuli_scaffold*	4405	T/C	Gene_Ca_28109	NON_SYNONYMOUS_CODING	NA
Kabuli_wild_4409	1399946689	*Ca_kabuli_scaffold*	4409	G/A	NA	INTERGENIC	NA
Kabuli_wild_6910	1399946690	*Ca_kabuli_scaffold*	6910	G/A	NA	INTERGENIC	NA
Kabuli_wild_159	1399946691	*Ca_kabuli_scaffold*	159	T/G	NA	INTERGENIC	NA
Kabuli_wild_75639	1399946692	*Ca_kabuli_scaffold*	75639	G/T	NA	INTERGENIC	NA
Kabuli_wild_75945	1399946693	*Ca_kabuli_scaffold*	75945	A/C	NA	INTERGENIC	NA
Kabuli_wild_75977	1399946694	*Ca_kabuli_scaffold*	75977	A/G	NA	INTERGENIC	NA
Kabuli_wild_76005	1399946695	*Ca_kabuli_scaffold*	76005	G/A	NA	INTERGENIC	NA
Kabuli_wild_1355	1399946696	*Ca_kabuli_scaffold*	1355	G/T	NA	INTERGENIC	NA
Kabuli_wild_1396	1399946697	*Ca_kabuli_scaffold*	1396	G/A	NA	INTERGENIC	NA
Kabuli_wild_7438	1399946698	*Ca_kabuli_scaffold*	7438	A/C	NA	INTERGENIC	NA
Kabuli_wild_7442	1399946699	*Ca_kabuli_scaffold*	7442	T/C	NA	INTERGENIC	NA
Kabuli_wild_7443	1399946700	*Ca_kabuli_scaffold*	7443	A/G	NA	INTERGENIC	NA
Kabuli_wild_7444	1399946701	*Ca_kabuli_scaffold*	7444	A/G	NA	INTERGENIC	NA
Kabuli_wild_7462	1399946702	*Ca_kabuli_scaffold*	7462	A/G	NA	INTERGENIC	NA
Kabuli_wild_7464	1399946703	*Ca_kabuli_scaffold*	7464	T/A	NA	INTERGENIC	NA
Kabuli_wild_7472	1399946704	*Ca_kabuli_scaffold*	7472	A/G	NA	INTERGENIC	NA
Kabuli_wild_7477	1399946705	*Ca_kabuli_scaffold*	7477	C/G	NA	INTERGENIC	NA
Kabuli_wild_7480	1399946706	*Ca_kabuli_scaffold*	7480	G/A	NA	INTERGENIC	NA
Kabuli_wild_7485	1399946707	*Ca_kabuli_scaffold*	7485	T/A	NA	INTERGENIC	NA
Kabuli_wild_7490	1399946708	*Ca_kabuli_scaffold*	7490	T/C	NA	INTERGENIC	NA
Kabuli_wild_7493	1399946709	*Ca_kabuli_scaffold*	7493	G/C	NA	INTERGENIC	NA
Kabuli_wild_7499	1399946710	*Ca_kabuli_scaffold*	7499	T/C	NA	INTERGENIC	NA
Kabuli_wild_7581	1399946711	*Ca_kabuli_scaffold*	7581	C/A	NA	INTERGENIC	NA
Kabuli_wild_7582	1399946712	*Ca_kabuli_scaffold*	7582	A/G	NA	INTERGENIC	NA
Kabuli_wild_7583	1399946713	*Ca_kabuli_scaffold*	7583	A/T	NA	INTERGENIC	NA
Kabuli_wild_7593	1399946714	*Ca_kabuli_scaffold*	7593	T/C	NA	INTERGENIC	NA
Kabuli_wild_7594	1399946715	*Ca_kabuli_scaffold*	7594	T/C	NA	INTERGENIC	NA
Kabuli_wild_7598	1399946716	*Ca_kabuli_scaffold*	7598	G/A	NA	INTERGENIC	NA
Kabuli_wild_9404	1399946717	*Ca_kabuli_scaffold*	9404	C/T	Gene_Ca_27112	NON_SYNONYMOUS_CODING	NA
Kabuli_wild_9787	1399946718	*Ca_kabuli_scaffold*	9787	G/A	Gene_Ca_27112	SYNONYMOUS_CODING	NA
Kabuli_wild_29185	1399946719	*Ca_kabuli_scaffold*	29185	A/G	Gene_Ca_24175	SYNONYMOUS_CODING	NA

SNP IDs	NCBI dbSNP accession IDs (NCBI_SS#)	Chromosomes	Physical positions (bp)	SNPs	Gene accession IDs	Sequence components of genome	Putative functions
Kabuli_wild_29191	1399946720	*Ca_kabuli_scaffold*	29191	C/T	Gene_Ca_24175	SYNONYMOUS_CODING	NA
Kabuli_wild_29210	1399946721	*Ca_kabuli_scaffold*	29210	G/C	Gene_Ca_24175	NON_SYNONYMOUS_CODING	NA
Kabuli_wild_29211	1399946722	*Ca_kabuli_scaffold*	29211	C/T	Gene_Ca_24175	NON_SYNONYMOUS_CODING	NA
Kabuli_wild_23885	1399946723	*Ca_kabuli_scaffold*	23885	C/T	Gene_Ca_27209	NON_SYNONYMOUS_CODING	Isocitrate/isopropylmalatedehydrogenase
Kabuli_wild_8939	1399946724	*Ca_kabuli_scaffold*	8939	T/A	NA	INTERGENIC	NA
Kabuli_wild_2917	1399946725	*Ca_kabuli_scaffold*	2917	C/T	NA	INTERGENIC	NA
Kabuli_wild_2926	1399946726	*Ca_kabuli_scaffold*	2926	C/T	NA	INTERGENIC	NA
Kabuli_wild_23138	1399946727	*Ca_kabuli_scaffold*	23138	A/G	NA	INTERGENIC	NA
Kabuli_wild_23190	1399946728	*Ca_kabuli_scaffold*	23190	G/C	NA	INTERGENIC	NA
Kabuli_wild_23201	1399946729	*Ca_kabuli_scaffold*	23201	G/A	NA	INTERGENIC	NA
Kabuli_wild_23270	1399946730	*Ca_kabuli_scaffold*	23270	A/C	NA	INTERGENIC	NA
Kabuli_wild_23315	1399946731	*Ca_kabuli_scaffold*	23315	C/A	NA	INTERGENIC	NA
Kabuli_wild_91103	1399946732	*Ca_kabuli_scaffold*	91103	C/T	NA	INTERGENIC	NA
Kabuli_wild_91108	1399946733	*Ca_kabuli_scaffold*	91108	T/C	NA	INTERGENIC	NA
Kabuli_wild_91112	1399946734	*Ca_kabuli_scaffold*	91112	A/C	NA	INTERGENIC	NA
Kabuli_wild_7136	1399946735	*Ca_kabuli_scaffold*	7136	G/A	NA	INTERGENIC	NA
Kabuli_wild_7143	1399946736	*Ca_kabuli_scaffold*	7143	C/A	NA	INTERGENIC	NA
Kabuli_wild_7198	1399946737	*Ca_kabuli_scaffold*	7198	T/C	NA	INTERGENIC	NA
Kabuli_wild_17935	1399946738	*Ca_kabuli_scaffold*	17935	C/A	NA	INTERGENIC	NA
Kabuli_wild_17948	1399946739	*Ca_kabuli_scaffold*	17948	C/A	Gene_Ca_28178	NON_SYNONYMOUS_CODING	Domain of unknown function DUF828
Kabuli_wild_17956	1399946740	*Ca_kabuli_scaffold*	17956	G/A	Gene_Ca_28178	NON_SYNONYMOUS_CODING	Domain of unknown function DUF828
Kabuli_wild_17959	1399946741	*Ca_kabuli_scaffold*	17959	A/G	Gene_Ca_28178	NON_SYNONYMOUS_CODING	Domain of unknown function DUF828
Kabuli_wild_17963	1399946742	*Ca_kabuli_scaffold*	17963	C/T	Gene_Ca_28178	NON_SYNONYMOUS_CODING	Domain of unknown function DUF828
Kabuli_wild_17999	1399946743	*Ca_kabuli_scaffold*	17999	T/G	Gene_Ca_28178	NON_SYNONYMOUS_CODING	Domain of unknown function DUF828
Kabuli_wild_18022	1399946744	*Ca_kabuli_scaffold*	18022	C/T	Gene_Ca_28178	DOWNSTREAM	Domain of unknown function DUF828
Kabuli_wild_18026	1399946745	*Ca_kabuli_scaffold*	18026	T/A	Gene_Ca_28178	DOWNSTREAM	Domain of unknown function DUF828
Kabuli_wild_411	1399946746	*Ca_kabuli_scaffold*	411	C/T	Gene_Ca_26005	INTRON	NA
Kabuli_wild_444	1399946747	*Ca_kabuli_scaffold*	444	G/A	Gene_Ca_26005	INTRON	NA
Kabuli_wild_69182	1399946748	*Ca_kabuli_scaffold*	69182	A/G	Gene_Ca_26010	INTRON	Protein kinase,catalytic domain
Kabuli_wild_69878	1399946749	*Ca_kabuli_scaffold*	69878	C/A	Gene_Ca_26010	INTRON	Protein kinase,catalytic domain
Kabuli_wild_45363	1399946750	*Ca_kabuli_scaffold*	45363	T/C	Gene_Ca_26273	NON_SYNONYMOUS_CODING	WD40 repeat
Kabuli_wild_13376	1399946751	*Ca_kabuli_scaffold*	13376	T/A	NA	INTERGENIC	NA
Kabuli_wild_84592	1399946752	*Ca_kabuli_scaffold*	84592	A/T	Gene_Ca_25601	INTRON	PeptidaseM76,ATP23
Kabuli_wild_42134	1399946753	*Ca_kabuli_scaffold*	42134	G/A	Gene_Ca_26926	SYNONYMOUS_CODING	Lipase,GDSL
Kabuli_wild_42135	1399946754	*Ca_kabuli_scaffold*	42135	G/T	Gene_Ca_26926	SYNONYMOUS_CODING	Lipase,GDSL
Kabuli_wild_42156	1399946755	*Ca_kabuli_scaffold*	42156	G/A	Gene_Ca_26926	SYNONYMOUS_CODING	Lipase,GDSL
Kabuli_wild_42163	1399946756	*Ca_kabuli_scaffold*	42163	T/C	Gene_Ca_26926	SYNONYMOUS_CODING	Lipase,GDSL
Kabuli_wild_42164	1399946757	*Ca_kabuli_scaffold*	42164	A/G	Gene_Ca_26926	SYNONYMOUS_CODING	Lipase,GDSL
Kabuli_wild_42175	1399946758	*Ca_kabuli_scaffold*	42175	G/A	Gene_Ca_26926	SYNONYMOUS_CODING	Lipase,GDSL
Kabuli_wild_1431	1399946759	*Ca_kabuli_scaffold*	1431	G/A	NA	INTERGENIC	NA
Kabuli_wild_1439	1399946760	*Ca_kabuli_scaffold*	1439	G/A	NA	INTERGENIC	NA
Kabuli_wild_1478	1399946761	*Ca_kabuli_scaffold*	1478	C/A	NA	INTERGENIC	NA
Kabuli_wild_398	1399946762	*Ca_kabuli_scaffold*	398	T/A	NA	INTERGENIC	NA
Kabuli_wild_403	1399946763	*Ca_kabuli_scaffold*	403	A/T	NA	INTERGENIC	NA

SNP IDs	NCBI dbSNP accession IDs (NCBI_SS#)	Chromosomes	Physical positions (bp)	SNPs	Gene accession IDs	Sequence components of genome	Putative functions
Kabuli_wild_425	1399946764	*Ca_kabuli_scaffold*	425	C/A	NA	INTERGENIC	NA
Kabuli_wild_431	1399946765	*Ca_kabuli_scaffold*	431	A/G	NA	INTERGENIC	NA
Kabuli_wild_458	1399946766	*Ca_kabuli_scaffold*	458	G/A	NA	INTERGENIC	NA
Kabuli_wild_107	1399946767	*Ca_kabuli_scaffold*	107	A/G	NA	INTERGENIC	NA
Kabuli_wild_120	1399946768	*Ca_kabuli_scaffold*	120	A/G	NA	INTERGENIC	NA
Kabuli_wild_161	1399946769	*Ca_kabuli_scaffold*	161	C/T	NA	INTERGENIC	NA
Kabuli_wild_231	1399946770	*Ca_kabuli_scaffold*	231	A/C	NA	INTERGENIC	NA
Kabuli_wild_81068	1399946771	*Ca_kabuli_scaffold*	81068	C/T	NA	INTERGENIC	NA
Kabuli_wild_81080	1399946772	*Ca_kabuli_scaffold*	81080	C/A	NA	INTERGENIC	NA
Kabuli_wild_8836	1399946773	*Ca_kabuli_scaffold*	8836	G/A	Gene_Ca_27069	DOWNSTREAM	NA
Kabuli_wild_68378	1399946774	*Ca_kabuli_scaffold*	68378	G/A	Gene_Ca_27523	INTRON	Pseudouridine-5'-phosphateglycosidase
Kabuli_wild_12947	1399946775	*Ca_kabuli_scaffold*	12947	C/A	Gene_Ca_27450	UPSTREAM	NA
Kabuli_wild_26228	1399946776	*Ca_kabuli_scaffold*	26228	C/T	NA	INTERGENIC	NA
Kabuli_wild_64956	1399946777	*Ca_kabuli_scaffold*	64956	C/A	Gene_Ca_27663	DOWNSTREAM	NA
Kabuli_wild_65093	1399946778	*Ca_kabuli_scaffold*	65093	T/C	Gene_Ca_27663	DOWNSTREAM	NA
Kabuli_wild_65127	1399946779	*Ca_kabuli_scaffold*	65127	C/A	Gene_Ca_27663	DOWNSTREAM	NA
Kabuli_wild_65129	1399946780	*Ca_kabuli_scaffold*	65129	T/A	Gene_Ca_27663	DOWNSTREAM	NA
Kabuli_wild_12616	1399946781	*Ca_kabuli_scaffold*	12616	T/A	NA	INTERGENIC	NA
Kabuli_wild_12620	1399946782	*Ca_kabuli_scaffold*	12620	T/A	NA	INTERGENIC	NA
Kabuli_wild_12658	1399946783	*Ca_kabuli_scaffold*	12658	C/A	NA	INTERGENIC	NA
Kabuli_wild_12692	1399946784	*Ca_kabuli_scaffold*	12692	G/A	NA	INTERGENIC	NA
Kabuli_wild_12694	1399946785	*Ca_kabuli_scaffold*	12694	G/A	NA	INTERGENIC	NA
Kabuli_wild_14581	1399946786	*Ca_kabuli_scaffold*	14581	T/A	NA	INTERGENIC	NA
Kabuli_wild_14596	1399946787	*Ca_kabuli_scaffold*	14596	C/T	NA	INTERGENIC	NA
Kabuli_wild_17198	1399946788	*Ca_kabuli_scaffold*	17198	C/T	Gene_Ca_27533	DOWNSTREAM	Poly(A)polymerase,centraldomain
Kabuli_wild_17199	1399946789	*Ca_kabuli_scaffold*	17199	A/G	Gene_Ca_27533	DOWNSTREAM	Poly(A)polymerase,centraldomain
Kabuli_wild_17286	1399946790	*Ca_kabuli_scaffold*	17286	T/A	Gene_Ca_27533	DOWNSTREAM	Poly(A)polymerase,centraldomain
Kabuli_wild_3367	1399946791	*Ca_kabuli_scaffold*	3367	G/A	Gene_Ca_26968	INTRON	NA
Kabuli_wild_3371	1399946792	*Ca_kabuli_scaffold*	3371	G/A	Gene_Ca_26968	INTRON	NA
Kabuli_wild_3399	1399946793	*Ca_kabuli_scaffold*	3399	C/G	Gene_Ca_26968	INTRON	NA
Kabuli_wild_3458	1399946794	*Ca_kabuli_scaffold*	3458	A/G	Gene_Ca_26968	INTRON	NA
Kabuli_wild_3461	1399946795	*Ca_kabuli_scaffold*	3461	T/C	Gene_Ca_26968	INTRON	NA
Kabuli_wild_3516	1399946796	*Ca_kabuli_scaffold*	3516	C/T	Gene_Ca_26968	INTRON	NA
Kabuli_wild_3879	1399946797	*Ca_kabuli_scaffold*	3879	C/T	Gene_Ca_26968	DOWNSTREAM	NA
Kabuli_wild_3880	1399946798	*Ca_kabuli_scaffold*	3880	G/T	Gene_Ca_26968	DOWNSTREAM	NA
Kabuli_wild_7260	1399946799	*Ca_kabuli_scaffold*	7260	T/A	NA	INTERGENIC	NA
Kabuli_wild_13440	1399946800	*Ca_kabuli_scaffold*	13440	C/T	Gene_Ca_26970	INTRON	NA
Kabuli_wild_13468	1399946801	*Ca_kabuli_scaffold*	13468	C/T	Gene_Ca_26970	INTRON	NA
Kabuli_wild_13892	1399946802	*Ca_kabuli_scaffold*	13892	C/G	Gene_Ca_26970	INTRON	NA
Kabuli_wild_16944	1399946803	*Ca_kabuli_scaffold*	16944	T/A	Gene_Ca_26970	INTRON	NA
Kabuli_wild_18889	1399946804	*Ca_kabuli_scaffold*	18889	A/G	Gene_Ca_26970	INTRON	NA
Kabuli_wild_19359	1399946805	*Ca_kabuli_scaffold*	19359	G/A	Gene_Ca_26970	INTRON	NA
Kabuli_wild_19366	1399946806	*Ca_kabuli_scaffold*	19366	G/T	Gene_Ca_26970	INTRON	NA
Kabuli_wild_19367	1399946807	*Ca_kabuli_scaffold*	19367	A/T	Gene_Ca_26970	INTRON	NA

SNP IDs	NCBI dbSNP accession IDs (NCBI_SS#)	Chromosomes	Physical positions (bp)	SNPs	Gene accession IDs	Sequence components of genome	Putative functions
Kabuli_wild_19368	1399946808	*Ca_kabuli_scaffold*	19368	A/C	Gene_Ca_26970	INTRON	NA
Kabuli_wild_6296	1399946809	*Ca_kabuli_scaffold*	6296	G/A	Gene_Ca_27632	SYNONYMOUS_CODING	NA
Kabuli_wild_21038	1399946810	*Ca_kabuli_scaffold*	21038	C/T	Gene_Ca_27633	NON_SYNONYMOUS_CODING	Peptidase C48, SUMO/Sentrin/Ubl1
Kabuli_wild_21071	1399946811	*Ca_kabuli_scaffold*	21071	C/T	Gene_Ca_27633	NON_SYNONYMOUS_CODING	Peptidase C48, SUMO/Sentrin/Ubl1
Kabuli_wild_21104	1399946812	*Ca_kabuli_scaffold*	21104	A/T	Gene_Ca_27633	NON_SYNONYMOUS_CODING	Peptidase C48, SUMO/Sentrin/Ubl1
Kabuli_wild_1575	1399946813	*Ca_kabuli_scaffold*	1575	A/C	NA	INTERGENIC	NA
Kabuli_wild_18144	1399946814	*Ca_kabuli_scaffold*	18144	T/G	NA	INTERGENIC	NA
Kabuli_wild_18147	1399946815	*Ca_kabuli_scaffold*	18147	G/A	NA	INTERGENIC	NA
Kabuli_wild_18155	1399946816	*Ca_kabuli_scaffold*	18155	A/G	NA	INTERGENIC	NA
Kabuli_wild_96223	1399946817	*Ca_kabuli_scaffold*	96223	G/C	Gene_Ca_26888	SYNONYMOUS_CODING	Protein of unknown function DUF1296
Kabuli_wild_130204	1399946818	*Ca_kabuli_scaffold*	130204	G/A	Gene_Ca_26890	NON_SYNONYMOUS_CODING	Peptidase C48, SUMO/Sentrin/Ubl1
Kabuli_wild_130256	1399946819	*Ca_kabuli_scaffold*	130256	C/T	Gene_Ca_26890	NON_SYNONYMOUS_CODING	Peptidase C48, SUMO/Sentrin/Ubl1
Kabuli_wild_130285	1399946820	*Ca_kabuli_scaffold*	130285	G/C	Gene_Ca_26890	NON_SYNONYMOUS_CODING	Peptidase C48, SUMO/Sentrin/Ubl1
Kabuli_wild_1749	1399946821	*Ca_kabuli_scaffold*	1749	G/T	NA	INTERGENIC	NA
Kabuli_wild_1751	1399946822	*Ca_kabuli_scaffold*	1751	T/C	NA	INTERGENIC	NA
Kabuli_wild_1778	1399946823	*Ca_kabuli_scaffold*	1778	G/A	NA	INTERGENIC	NA
Kabuli_wild_1780	1399946824	*Ca_kabuli_scaffold*	1780	T/C	NA	INTERGENIC	NA
Kabuli_wild_1781	1399946825	*Ca_kabuli_scaffold*	1781	C/T	NA	INTERGENIC	NA
Kabuli_wild_1782	1399946826	*Ca_kabuli_scaffold*	1782	A/G	NA	INTERGENIC	NA
Kabuli_wild_1787	1399946827	*Ca_kabuli_scaffold*	1787	G/A	NA	INTERGENIC	NA
Kabuli_wild_1793	1399946828	*Ca_kabuli_scaffold*	1793	A/T	NA	INTERGENIC	NA
Kabuli_wild_1794	1399946829	*Ca_kabuli_scaffold*	1794	A/C	NA	INTERGENIC	NA
Kabuli_wild_1799	1399946830	*Ca_kabuli_scaffold*	1799	T/C	NA	INTERGENIC	NA
Kabuli_wild_1814	1399946831	*Ca_kabuli_scaffold*	1814	G/T	NA	INTERGENIC	NA
Kabuli_wild_16503	1399946832	*Ca_kabuli_scaffold*	16503	C/G	Gene_Ca_27489	NON_SYNONYMOUS_CODING	PeptidaseA1
Kabuli_wild_873	1399946833	*Ca_kabuli_scaffold*	873	G/A	NA	INTERGENIC	NA
Kabuli_wild_1004	1399946834	*Ca_kabuli_scaffold*	1004	T/C	NA	INTERGENIC	NA
Kabuli_wild_1137	1399946835	*Ca_kabuli_scaffold*	1137	T/C	NA	INTERGENIC	NA
Kabuli_wild_1160	1399946836	*Ca_kabuli_scaffold*	1160	T/C	NA	INTERGENIC	NA
Kabuli_wild_1322	1399946837	*Ca_kabuli_scaffold*	1322	T/A	NA	INTERGENIC	NA
Kabuli_wild_1345	1399946838	*Ca_kabuli_scaffold*	1345	C/T	NA	INTERGENIC	NA
Kabuli_wild_93	1399946839	*Ca_kabuli_scaffold*	93	T/A	NA	INTERGENIC	NA
Kabuli_wild_104	1399946840	*Ca_kabuli_scaffold*	104	G/A	NA	INTERGENIC	NA
Kabuli_wild_114	1399946841	*Ca_kabuli_scaffold*	114	G/T	NA	INTERGENIC	NA
Kabuli_wild_9968	1399946842	*Ca_kabuli_scaffold*	9968	T/C	NA	INTERGENIC	NA
Kabuli_wild_10075	1399946843	*Ca_kabuli_scaffold*	10075	A/G	NA	INTERGENIC	NA
Kabuli_wild_13169	1399946844	*Ca_kabuli_scaffold*	13169	A/C	NA	INTERGENIC	NA
Kabuli_wild_13189	1399946845	*Ca_kabuli_scaffold*	13189	T/G	NA	INTERGENIC	NA
Kabuli_wild_13207	1399946846	*Ca_kabuli_scaffold*	13207	A/G	NA	INTERGENIC	NA
Kabuli_wild_53374	1399946847	*Ca_kabuli_scaffold*	53374	T/C	NA	INTERGENIC	NA
Kabuli_wild_9986	1399946848	*Ca_kabuli_scaffold*	9986	G/A	NA	INTERGENIC	NA
Kabuli_wild_1975	1399946849	*Ca_kabuli_scaffold*	1975	A/T	NA	INTERGENIC	NA
Kabuli_wild_1980	1399946850	*Ca_kabuli_scaffold*	1980	T/A	NA	INTERGENIC	NA
Kabuli_wild_1987	1399946851	*Ca_kabuli_scaffold*	1987	G/A	NA	INTERGENIC	NA

SNP IDs	NCBI dbSNP accession IDs (NCBI_SS#)	Chromosomes	Physical positions (bp)	SNPs	Gene accession IDs	Sequence components of genome	Putative functions
Kabuli_wild_1988	1399946852	*Ca_kabuli_scaffold*	1988	C/T	NA	INTERGENIC	NA
Kabuli_wild_1991	1399946853	*Ca_kabuli_scaffold*	1991	T/G	NA	INTERGENIC	NA
Kabuli_wild_2000	1399946854	*Ca_kabuli_scaffold*	2000	A/G	NA	INTERGENIC	NA
Kabuli_wild_2021	1399946855	*Ca_kabuli_scaffold*	2021	T/G	NA	INTERGENIC	NA
Kabuli_wild_4013	1399946856	*Ca_kabuli_scaffold*	4013	G/A	NA	INTERGENIC	NA
Kabuli_wild_183	1399946857	*Ca_kabuli_scaffold*	183	C/A	NA	INTERGENIC	NA
Kabuli_wild_200	1399946858	*Ca_kabuli_scaffold*	200	C/T	NA	INTERGENIC	NA
Kabuli_wild_20003	1399946859	*Ca_kabuli_scaffold*	20003	G/A	Gene_Ca_26054	INTRON	Aminotransferase-like,plant mobile domain
Kabuli_wild_20005	1399946860	*Ca_kabuli_scaffold*	20005	G/A	Gene_Ca_26054	INTRON	Aminotransferase-like,plant mobile domain
Kabuli_wild_20010	1399946861	*Ca_kabuli_scaffold*	20010	G/A	Gene_Ca_26054	INTRON	Aminotransferase-like,plant mobile domain
Kabuli_wild_20038	1399946862	*Ca_kabuli_scaffold*	20038	A/G	Gene_Ca_26054	INTRON	Aminotransferase-like,plant mobile domain
Kabuli_wild_29414	1399946863	*Ca_kabuli_scaffold*	29414	G/T	Gene_Ca_26055	INTRON	Zinc finger,PHD-type
Kabuli_wild_29421	1399946864	*Ca_kabuli_scaffold*	29421	T/A	Gene_Ca_26055	INTRON	Zinc finger,PHD-type
Kabuli_wild_32129	1399946865	*Ca_kabuli_scaffold*	32129	T/A	Gene_Ca_26055	NON_SYNONYMOUS_CODING	Zinc finger,PHD-type
Kabuli_wild_36715	1399946866	*Ca_kabuli_scaffold*	36715	A/T	Gene_Ca_26055	INTRON	Zinc finger,PHD-type
Kabuli_wild_69099	1399946867	*Ca_kabuli_scaffold*	69099	T/C	Gene_Ca_26056	SYNONYMOUS_CODING	NA
Kabuli_wild_70041	1399946868	*Ca_kabuli_scaffold*	70041	T/C	Gene_Ca_26056	SYNONYMOUS_CODING	NA
Kabuli_wild_71089	1399946869	*Ca_kabuli_scaffold*	71089	T/C	Gene_Ca_26056	NON_SYNONYMOUS_CODING	NA
Kabuli_wild_1033	1399946870	*Ca_kabuli_scaffold*	1033	T/C	NA	INTERGENIC	NA
Kabuli_wild_40007	1399946871	*Ca_kabuli_scaffold*	40007	G/A	Gene_Ca_28219	NON_SYNONYMOUS_CODING	Peptidase C48, SUMO/Sentrin/Ubl1
Kabuli_wild_40008	1399946872	*Ca_kabuli_scaffold*	40008	C/T	Gene_Ca_28219	NON_SYNONYMOUS_CODING	Peptidase C48, SUMO/Sentrin/Ubl1
Kabuli_wild_40014	1399946873	*Ca_kabuli_scaffold*	40014	C/T	Gene_Ca_28219	NON_SYNONYMOUS_CODING	Peptidase C48, SUMO/Sentrin/Ubl1
Kabuli_wild_40063	1399946874	*Ca_kabuli_scaffold*	40063	A/G	Gene_Ca_28219	SYNONYMOUS_CODING	Peptidase C48, SUMO/Sentrin/Ubl1
Kabuli_wild_40089	1399946875	*Ca_kabuli_scaffold*	40089	C/A	Gene_Ca_28219	NON_SYNONYMOUS_CODING	Peptidase C48, SUMO/Sentrin/Ubl1
Kabuli_wild_40091	1399946876	*Ca_kabuli_scaffold*	40091	G/T	Gene_Ca_28219	NON_SYNONYMOUS_CODING	Peptidase C48, SUMO/Sentrin/Ubl1
Kabuli_wild_893	1399946877	*Ca_kabuli_scaffold*	893	G/A	NA	INTERGENIC	NA
Kabuli_wild_898	1399946878	*Ca_kabuli_scaffold*	898	G/T	NA	INTERGENIC	NA
Kabuli_wild_929	1399946879	*Ca_kabuli_scaffold*	929	A/C	NA	INTERGENIC	NA
Kabuli_wild_935	1399946880	*Ca_kabuli_scaffold*	935	C/A	NA	INTERGENIC	NA
Kabuli_wild_938	1399946881	*Ca_kabuli_scaffold*	938	G/C	NA	INTERGENIC	NA
Kabuli_wild_954	1399946882	*Ca_kabuli_scaffold*	954	A/C	NA	INTERGENIC	NA
Kabuli_wild_2699	1399946883	*Ca_kabuli_scaffold*	2699	T/C	NA	INTERGENIC	NA
Kabuli_wild_23442	1399946884	*Ca_kabuli_scaffold*	23442	T/C	NA	INTERGENIC	NA
Kabuli_wild_511	1399946885	*Ca_kabuli_scaffold*	511	G/A	NA	INTERGENIC	NA
Kabuli_wild_516	1399946886	*Ca_kabuli_scaffold*	516	G/T	NA	INTERGENIC	NA
Kabuli_wild_547	1399946887	*Ca_kabuli_scaffold*	547	A/C	NA	INTERGENIC	NA
Kabuli_wild_553	1399946888	*Ca_kabuli_scaffold*	553	C/A	NA	INTERGENIC	NA
Kabuli_wild_556	1399946889	*Ca_kabuli_scaffold*	556	G/C	NA	INTERGENIC	NA
Kabuli_wild_572	1399946890	*Ca_kabuli_scaffold*	572	A/C	NA	INTERGENIC	NA
Kabuli_wild_7611	1399946891	*Ca_kabuli_scaffold*	7611	G/C	NA	INTERGENIC	NA
Kabuli_wild_7613	1399946892	*Ca_kabuli_scaffold*	7613	T/A	NA	INTERGENIC	NA
Kabuli_wild_7621	1399946893	*Ca_kabuli_scaffold*	7621	T/C	NA	INTERGENIC	NA
Kabuli_wild_7632	1399946894	*Ca_kabuli_scaffold*	7632	A/G	NA	INTERGENIC	NA
Kabuli_wild_7638	1399946895	*Ca_kabuli_scaffold*	7638	C/T	NA	INTERGENIC	NA

SNP IDs	NCBI dbSNP accession IDs (NCBI_SS#)	Chromosomes	Physical positions (bp)	SNPs	Gene accession IDs	Sequence components of genome	Putative functions
Kabuli_wild_7641	1399946896	*Ca_kabuli_scaffold*	7641	T/C	NA	INTERGENIC	NA
Kabuli_wild_7651	1399946897	*Ca_kabuli_scaffold*	7651	T/C	NA	INTERGENIC	NA
Kabuli_wild_7671	1399946898	*Ca_kabuli_scaffold*	7671	C/T	NA	INTERGENIC	NA
Kabuli_wild_7678	1399946899	*Ca_kabuli_scaffold*	7678	G/A	NA	INTERGENIC	NA
Kabuli_wild_7683	1399946900	*Ca_kabuli_scaffold*	7683	T/C	NA	INTERGENIC	NA
Kabuli_wild_5685	1399946901	*Ca_kabuli_scaffold*	5685	A/G	Gene_Ca_28141	DOWNSTREAM	NA
Kabuli_wild_966	1399946902	*Ca_kabuli_scaffold*	966	G/A	NA	INTERGENIC	NA
Kabuli_wild_969	1399946903	*Ca_kabuli_scaffold*	969	T/C	NA	INTERGENIC	NA
Kabuli_wild_978	1399946904	*Ca_kabuli_scaffold*	978	T/A	NA	INTERGENIC	NA
Kabuli_wild_10546	1399946905	*Ca_kabuli_scaffold*	10546	G/T	Gene_Ca_26707	NON_SYNONYMOUS_CODING	IQ motif,EF-handbinding site
Kabuli_wild_16224	1399946906	*Ca_kabuli_scaffold*	16224	A/T	Gene_Ca_26707	NON_SYNONYMOUS_CODING	IQ motif,EF-handbinding site
Kabuli_wild_16239	1399946907	*Ca_kabuli_scaffold*	16239	G/T	Gene_Ca_26707	NON_SYNONYMOUS_CODING	IQ motif,EF-handbinding site
Kabuli_wild_55347	1399946908	*Ca_kabuli_scaffold*	55347	G/T	Gene_Ca_26708	NON_SYNONYMOUS_CODING	PWWP
Kabuli_wild_72624	1399946909	*Ca_kabuli_scaffold*	72624	G/A	Gene_Ca_26709	INTRON	Peptidoglycan-bindingLysinsubgroup
Kabuli_wild_76922	1399946910	*Ca_kabuli_scaffold*	76922	T/G	NA	INTERGENIC	NA
Kabuli_wild_76950	1399946911	*Ca_kabuli_scaffold*	76950	C/A	NA	INTERGENIC	NA
Kabuli_wild_76961	1399946912	*Ca_kabuli_scaffold*	76961	A/G	NA	INTERGENIC	NA
Kabuli_wild_76964	1399946913	*Ca_kabuli_scaffold*	76964	G/A	NA	INTERGENIC	NA
Kabuli_wild_76965	1399946914	*Ca_kabuli_scaffold*	76965	C/A	NA	INTERGENIC	NA
Kabuli_wild_106820	1399946915	*Ca_kabuli_scaffold*	106820	A/G	Gene_Ca_26711	INTRON	WD40 repeat
Kabuli_wild_112193	1399946916	*Ca_kabuli_scaffold*	112193	A/C	Gene_Ca_26711	INTRON	WD40 repeat
Kabuli_wild_974	1399946917	*Ca_kabuli_scaffold*	974	G/A	NA	INTERGENIC	NA
Kabuli_wild_5839	1399946918	*Ca_kabuli_scaffold*	5839	C/T	Gene_Ca_28200	NON_SYNONYMOUS_CODING	NA
Kabuli_wild_5849	1399946919	*Ca_kabuli_scaffold*	5849	G/C	Gene_Ca_28200	SYNONYMOUS_CODING	NA
Kabuli_wild_5866	1399946920	*Ca_kabuli_scaffold*	5866	T/A	Gene_Ca_28200	NON_SYNONYMOUS_CODING	NA
Kabuli_wild_5891	1399946921	*Ca_kabuli_scaffold*	5891	A/T	Gene_Ca_28200	SYNONYMOUS_CODING	NA
Kabuli_wild_5912	1399946922	*Ca_kabuli_scaffold*	5912	G/A	Gene_Ca_28200	SYNONYMOUS_CODING	NA
Kabuli_wild_14560	1399946923	*Ca_kabuli_scaffold*	14560	T/C	NA	INTERGENIC	NA
Kabuli_wild_14591	1399946924	*Ca_kabuli_scaffold*	14591	G/C	NA	INTERGENIC	NA
Kabuli_wild_6948	1399946925	*Ca_kabuli_scaffold*	6948	C/T	NA	INTERGENIC	NA
Kabuli_wild_6956	1399946926	*Ca_kabuli_scaffold*	6956	G/A	NA	INTERGENIC	NA
Kabuli_wild_4558	1399946927	*Ca_kabuli_scaffold*	4558	C/T	NA	INTERGENIC	NA
Kabuli_wild_2301	1399946928	*Ca_kabuli_scaffold*	2301	A/G	NA	INTERGENIC	NA
Kabuli_wild_20870	1399946929	*Ca_kabuli_scaffold*	20870	A/T	NA	INTERGENIC	NA
Kabuli_wild_20891	1399946930	*Ca_kabuli_scaffold*	20891	T/C	NA	INTERGENIC	NA
Kabuli_wild_20894	1399946931	*Ca_kabuli_scaffold*	20894	C/A	NA	INTERGENIC	NA
Kabuli_wild_20895	1399946932	*Ca_kabuli_scaffold*	20895	A/T	NA	INTERGENIC	NA
Kabuli_wild_1091	1399946933	*Ca_kabuli_scaffold*	1091	G/A	Gene_Ca_28010	NON_SYNONYMOUS_CODING	NA
Kabuli_wild_1323	1399946934	*Ca_kabuli_scaffold*	1323	G/A	Gene_Ca_28010	NON_SYNONYMOUS_CODING	NA
Kabuli_wild_2049	1399946935	*Ca_kabuli_scaffold*	2049	C/T	NA	INTERGENIC	NA
Kabuli_wild_2083	1399946936	*Ca_kabuli_scaffold*	2083	A/G	NA	INTERGENIC	NA
Kabuli_wild_2084	1399946937	*Ca_kabuli_scaffold*	2084	C/T	NA	INTERGENIC	NA
Kabuli_wild_2090	1399946938	*Ca_kabuli_scaffold*	2090	C/T	NA	INTERGENIC	NA
Kabuli_wild_40852	1399946939	*Ca_kabuli_scaffold*	40852	G/T	Gene_Ca_27607	DOWNSTREAM	GCK

SNP IDs	NCBI dbSNP accession IDs (NCBI_SS#)	Chromosomes	Physical positions (bp)	SNPs	Gene accession IDs	Sequence components of genome	Putative functions
Kabuli_wild_85	1399946940	*Ca_kabuli_scaffold*	85	C/T	NA	INTERGENIC	NA
Kabuli_wild_117	1399946941	*Ca_kabuli_scaffold*	117	C/T	NA	INTERGENIC	NA
Kabuli_wild_2561	1399946942	*Ca_kabuli_scaffold*	2561	C/T	NA	INTERGENIC	NA
Kabuli_wild_3329	1399946943	*Ca_kabuli_scaffold*	3329	C/A	NA	INTERGENIC	NA
Kabuli_wild_11300	1399946944	*Ca_kabuli_scaffold*	11300	T/A	NA	INTERGENIC	NA
Kabuli_wild_11312	1399946945	*Ca_kabuli_scaffold*	11312	A/T	NA	INTERGENIC	NA
Kabuli_wild_1353	1399946946	*Ca_kabuli_scaffold*	1353	C/G	NA	INTERGENIC	NA
Kabuli_wild_353	1399946947	*Ca_kabuli_scaffold*	353	G/A	NA	INTERGENIC	NA
Kabuli_wild_365	1399946948	*Ca_kabuli_scaffold*	365	T/G	NA	INTERGENIC	NA
Kabuli_wild_366	1399946949	*Ca_kabuli_scaffold*	366	G/A	NA	INTERGENIC	NA
Kabuli_wild_372	1399946950	*Ca_kabuli_scaffold*	372	T/C	NA	INTERGENIC	NA
Kabuli_wild_386	1399946951	*Ca_kabuli_scaffold*	386	G/A	NA	INTERGENIC	NA
Kabuli_wild_396	1399946952	*Ca_kabuli_scaffold*	396	G/A	NA	INTERGENIC	NA
Kabuli_wild_406	1399946953	*Ca_kabuli_scaffold*	406	C/A	NA	INTERGENIC	NA
Kabuli_wild_202	1399946954	*Ca_kabuli_scaffold*	202	G/A	NA	INTERGENIC	NA
Kabuli_wild_238	1399946955	*Ca_kabuli_scaffold*	238	A/C	NA	INTERGENIC	NA
Kabuli_wild_247	1399946956	*Ca_kabuli_scaffold*	247	G/C	NA	INTERGENIC	NA
Kabuli_wild_2675	1399946957	*Ca_kabuli_scaffold*	2675	T/C	NA	INTERGENIC	NA
Kabuli_wild_24952	1399946958	*Ca_kabuli_scaffold*	24952	C/A	NA	INTERGENIC	NA
Kabuli_wild_1383	1399946959	*Ca_kabuli_scaffold*	1383	T/C	NA	INTERGENIC	NA
Kabuli_wild_1434	1399946960	*Ca_kabuli_scaffold*	1434	C/G	NA	INTERGENIC	NA
Kabuli_wild_1884	1399946961	*Ca_kabuli_scaffold*	1884	G/A	NA	INTERGENIC	NA
Kabuli_wild_1887	1399946962	*Ca_kabuli_scaffold*	1887	T/C	NA	INTERGENIC	NA
Kabuli_wild_1896	1399946963	*Ca_kabuli_scaffold*	1896	A/T	NA	INTERGENIC	NA
Kabuli_wild_2011	1399946964	*Ca_kabuli_scaffold*	2011	C/G	NA	INTERGENIC	NA
Kabuli_wild_2061	1399946965	*Ca_kabuli_scaffold*	2061	A/G	NA	INTERGENIC	NA
Kabuli_wild_2076	1399946966	*Ca_kabuli_scaffold*	2076	G/A	NA	INTERGENIC	NA
Kabuli_wild_2092	1399946967	*Ca_kabuli_scaffold*	2092	C/G	NA	INTERGENIC	NA
Kabuli_wild_24882	1399946968	*Ca_kabuli_scaffold*	24882	A/C	NA	INTERGENIC	NA
Kabuli_wild_36359	1399946969	*Ca_kabuli_scaffold*	36359	G/C	NA	INTERGENIC	NA
Kabuli_wild_192	1399946970	*Ca_kabuli_scaffold*	192	A/G	NA	INTERGENIC	NA
Kabuli_wild_163	1399946971	*Ca_kabuli_scaffold*	163	A/C	NA	INTERGENIC	NA
Kabuli_wild_1297	1399946972	*Ca_kabuli_scaffold*	1297	T/C	Gene_Ca_26783	DOWNSTREAM	NA
Kabuli_wild_65166	1399946973	*Ca_kabuli_scaffold*	65166	A/C	Gene_Ca_26785	NON_SYNONYMOUS_CODING	Pairedamphipathichelix
Kabuli_wild_86716	1399946974	*Ca_kabuli_scaffold*	86716	T/C	NA	INTERGENIC	NA
Kabuli_wild_88603	1399946975	*Ca_kabuli_scaffold*	88603	T/G	NA	INTERGENIC	NA
Kabuli_wild_97407	1399946976	*Ca_kabuli_scaffold*	97407	G/T	Gene_Ca_26786	INTRON	Dehydrogenase,E1component
Kabuli_wild_528	1399946977	*Ca_kabuli_scaffold*	528	C/A	NA	INTERGENIC	NA
Kabuli_wild_599	1399946978	*Ca_kabuli_scaffold*	599	G/A	NA	INTERGENIC	NA
Kabuli_wild_604	1399946979	*Ca_kabuli_scaffold*	604	C/T	NA	INTERGENIC	NA
Kabuli_wild_679	1399946980	*Ca_kabuli_scaffold*	679	C/T	NA	INTERGENIC	NA
Kabuli_wild_718	1399946981	*Ca_kabuli_scaffold*	718	C/G	NA	INTERGENIC	NA
Kabuli_wild_742	1399946982	*Ca_kabuli_scaffold*	742	G/C	NA	INTERGENIC	NA
Kabuli_wild_1458	1399946983	*Ca_kabuli_scaffold*	1458	A/G	NA	INTERGENIC	NA

SNP IDs	NCBI dbSNP accession IDs (NCBI_SS#)	Chromosomes	Physical positions (bp)	SNPs	Gene accession IDs	Sequence components of genome	Putative functions
Kabuli_wild_1489	1399946984	*Ca_kabuli_scaffold*	1489	A/G	NA	INTERGENIC	NA
Kabuli_wild_1500	1399946985	*Ca_kabuli_scaffold*	1500	T/C	NA	INTERGENIC	NA
Kabuli_wild_1669	1399946986	*Ca_kabuli_scaffold*	1669	C/T	NA	INTERGENIC	NA
Kabuli_wild_1675	1399946987	*Ca_kabuli_scaffold*	1675	C/A	NA	INTERGENIC	NA
Kabuli_wild_1678	1399946988	*Ca_kabuli_scaffold*	1678	C/T	NA	INTERGENIC	NA
Kabuli_wild_1718	1399946989	*Ca_kabuli_scaffold*	1718	G/A	NA	INTERGENIC	NA
Kabuli_wild_753	1399946990	*Ca_kabuli_scaffold*	753	T/C	NA	INTERGENIC	NA
Kabuli_wild_777	1399946991	*Ca_kabuli_scaffold*	777	G/A	NA	INTERGENIC	NA
Kabuli_wild_817	1399946992	*Ca_kabuli_scaffold*	817	C/T	NA	INTERGENIC	NA
Kabuli_wild_1030	1399946993	*Ca_kabuli_scaffold*	1030	T/A	NA	INTERGENIC	NA
Kabuli_wild_1032	1399946994	*Ca_kabuli_scaffold*	1032	G/T	NA	INTERGENIC	NA
Kabuli_wild_2837	1399946995	*Ca_kabuli_scaffold*	2837	A/G	NA	INTERGENIC	NA
Kabuli_wild_2854	1399946996	*Ca_kabuli_scaffold*	2854	A/T	NA	INTERGENIC	NA
Kabuli_wild_461	1399946997	*Ca_kabuli_scaffold*	461	G/A	NA	INTERGENIC	NA
Kabuli_wild_141	1399946998	*Ca_kabuli_scaffold*	141	G/A	NA	INTERGENIC	NA
Kabuli_wild_190	1399946999	*Ca_kabuli_scaffold*	190	T/G	NA	INTERGENIC	NA
Kabuli_wild_6825	1399947000	*Ca_kabuli_scaffold*	6825	G/T	Gene_Ca_27012	SYNONYMOUS_CODING	BP28,C-terminal
Kabuli_wild_10842	1399947001	*Ca_kabuli_scaffold*	10842	T/A	Gene_Ca_27012	INTRON	BP28,C-terminal
Kabuli_wild_10856	1399947002	*Ca_kabuli_scaffold*	10856	T/G	Gene_Ca_27012	INTRON	BP28,C-terminal
Kabuli_wild_13651	1399947003	*Ca_kabuli_scaffold*	13651	G/A	Gene_Ca_27012	INTRON	BP28,C-terminal
Kabuli_wild_25674	1399947004	*Ca_kabuli_scaffold*	25674	T/C	Gene_Ca_27013	SYNONYMOUS_CODING	Metallo-dependentphosphatase
Kabuli_wild_1553	1399947005	*Ca_kabuli_scaffold*	1553	T/A	Gene_Ca_27958	SYNONYMOUS_CODING	NA
Kabuli_wild_1791	1399947006	*Ca_kabuli_scaffold*	1791	T/A	Gene_Ca_28082	NON_SYNONYMOUS_CODING	NA
Kabuli_wild_215	1399947007	*Ca_kabuli_scaffold*	215	G/A	NA	INTERGENIC	NA
Kabuli_wild_217	1399947008	*Ca_kabuli_scaffold*	217	A/G	NA	INTERGENIC	NA
Kabuli_wild_23768	1399947009	*Ca_kabuli_scaffold*	23768	T/G	Gene_Ca_27572	INTRON	WD40 repeat
Kabuli_wild_43714	1399947010	*Ca_kabuli_scaffold*	43714	A/G	Gene_Ca_27573	SYNONYMOUS_CODING	WD40 repeat
Kabuli_wild_1870	1399947011	*Ca_kabuli_scaffold*	1870	C/A	NA	INTERGENIC	NA
Kabuli_wild_1891	1399947012	*Ca_kabuli_scaffold*	1891	A/G	NA	INTERGENIC	NA
Kabuli_wild_1931	1399947013	*Ca_kabuli_scaffold*	1931	C/T	NA	INTERGENIC	NA
Kabuli_wild_1934	1399947014	*Ca_kabuli_scaffold*	1934	T/A	NA	INTERGENIC	NA
Kabuli_wild_1285	1399947015	*Ca_kabuli_scaffold*	1285	G/A	NA	INTERGENIC	NA
Kabuli_wild_1288	1399947016	*Ca_kabuli_scaffold*	1288	G/T	NA	INTERGENIC	NA
Kabuli_wild_1290	1399947017	*Ca_kabuli_scaffold*	1290	T/C	NA	INTERGENIC	NA
Kabuli_wild_1317	1399947018	*Ca_kabuli_scaffold*	1317	C/G	NA	INTERGENIC	NA
Kabuli_wild_3893	1399947019	*Ca_kabuli_scaffold*	3893	A/T	NA	INTERGENIC	NA
Kabuli_wild_307	1399947020	*Ca_kabuli_scaffold*	307	A/G	NA	INTERGENIC	NA
Kabuli_wild_1810	1399947021	*Ca_kabuli_scaffold*	1810	C/A	Gene_Ca_28207	SYNONYMOUS_CODING	NA
Kabuli_wild_1826	1399947022	*Ca_kabuli_scaffold*	1826	C/T	Gene_Ca_28207	SYNONYMOUS_CODING	NA
Kabuli_wild_3572	1399947023	*Ca_kabuli_scaffold*	3572	A/G	Gene_Ca_28031	INTRON	CCTdomain
Kabuli_wild_3602	1399947024	*Ca_kabuli_scaffold*	3602	T/C	Gene_Ca_28031	INTRON	CCTdomain
Kabuli_wild_3608	1399947025	*Ca_kabuli_scaffold*	3608	A/G	Gene_Ca_28031	INTRON	CCTdomain
Kabuli_wild_1686	1399947026	*Ca_kabuli_scaffold*	1686	G/C	NA	INTERGENIC	NA
Kabuli_wild_9558	1399947027	*Ca_kabuli_scaffold*	9558	G/A	Gene_Ca_27577	DOWNSTREAM	NA

SNP IDs	NCBI dbSNP accession IDs (NCBI_SS#)	Chromosomes	Physical positions (bp)	SNPs	Gene accession IDs	Sequence components of genome	Putative functions
Kabuli_wild_9563	1399947028	*Ca_kabuli_scaffold*	9563	G/A	Gene_Ca_27577	DOWNSTREAM	NA
Kabuli_wild_2106	1399947029	*Ca_kabuli_scaffold*	2106	G/C	NA	INTERGENIC	NA
Kabuli_wild_2114	1399947030	*Ca_kabuli_scaffold*	2114	A/G	NA	INTERGENIC	NA
Kabuli_wild_2118	1399947031	*Ca_kabuli_scaffold*	2118	T/C	NA	INTERGENIC	NA
Kabuli_wild_2673	1399947032	*Ca_kabuli_scaffold*	2673	C/A	Gene_Ca_27808	SYNONYMOUS_CODING	Peptidase C48, SUMO/Sentrin/Ubl1
Kabuli_wild_2753	1399947033	*Ca_kabuli_scaffold*	2753	C/T	Gene_Ca_27808	NON_SYNONYMOUS_CODING	Peptidase C48, SUMO/Sentrin/Ubl1
Kabuli_wild_3434	1399947034	*Ca_kabuli_scaffold*	3434	A/T	Gene_Ca_27664	SYNONYMOUS_CODING	NA
Kabuli_wild_503	1399947035	*Ca_kabuli_scaffold*	503	C/T	NA	INTERGENIC	NA
Kabuli_wild_525	1399947036	*Ca_kabuli_scaffold*	525	A/C	NA	INTERGENIC	NA
Kabuli_wild_549	1399947037	*Ca_kabuli_scaffold*	549	G/A	NA	INTERGENIC	NA
Kabuli_wild_1000	1399947038	*Ca_kabuli_scaffold*	1000	T/G	NA	INTERGENIC	NA
Kabuli_wild_1002	1399947039	*Ca_kabuli_scaffold*	1002	A/C	NA	INTERGENIC	NA
Kabuli_wild_695	1399947040	*Ca_kabuli_scaffold*	695	C/A	NA	INTERGENIC	NA
Kabuli_wild_727	1399947041	*Ca_kabuli_scaffold*	727	C/T	NA	INTERGENIC	NA
Kabuli_wild_994	1399947042	*Ca_kabuli_scaffold*	994	A/G	NA	INTERGENIC	NA
Kabuli_wild_1024	1399947043	*Ca_kabuli_scaffold*	1024	T/G	NA	INTERGENIC	NA
Kabuli_wild_1042	1399947044	*Ca_kabuli_scaffold*	1042	T/C	NA	INTERGENIC	NA
Kabuli_wild_1049	1399947045	*Ca_kabuli_scaffold*	1049	C/T	NA	INTERGENIC	NA
Kabuli_wild_1075	1399947046	*Ca_kabuli_scaffold*	1075	C/T	NA	INTERGENIC	NA
Kabuli_wild_1078	1399947047	*Ca_kabuli_scaffold*	1078	G/A	NA	INTERGENIC	NA
Kabuli_wild_1147	1399947048	*Ca_kabuli_scaffold*	1147	C/T	NA	INTERGENIC	NA
Kabuli_wild_3326	1399947049	*Ca_kabuli_scaffold*	3326	A/C	NA	INTERGENIC	NA
Kabuli_wild_3351	1399947050	*Ca_kabuli_scaffold*	3351	C/T	NA	INTERGENIC	NA
Kabuli_wild_1090	1399947051	*Ca_kabuli_scaffold*	1090	T/G	NA	INTERGENIC	NA
Kabuli_wild_684	1399947052	*Ca_kabuli_scaffold*	684	T/C	NA	INTERGENIC	NA
Kabuli_wild_726	1399947053	*Ca_kabuli_scaffold*	726	A/C	NA	INTERGENIC	NA
Kabuli_wild_1358	1399947054	*Ca_kabuli_scaffold*	1358	C/T	NA	INTERGENIC	NA
Kabuli_wild_1451	1399947055	*Ca_kabuli_scaffold*	1451	G/A	NA	INTERGENIC	NA
Kabuli_wild_6552	1399947056	*Ca_kabuli_scaffold*	6552	T/C	NA	INTERGENIC	NA
Kabuli_wild_6564	1399947057	*Ca_kabuli_scaffold*	6564	G/A	NA	INTERGENIC	NA
Kabuli_wild_21572	1399947058	*Ca_kabuli_scaffold*	21572	T/A	NA	INTERGENIC	NA
Kabuli_wild_21590	1399947059	*Ca_kabuli_scaffold*	21590	T/C	NA	INTERGENIC	NA
Kabuli_wild_21592	1399947060	*Ca_kabuli_scaffold*	21592	G/T	NA	INTERGENIC	NA
Kabuli_wild_21624	1399947061	*Ca_kabuli_scaffold*	21624	G/A	NA	INTERGENIC	NA
Kabuli_wild_21649	1399947062	*Ca_kabuli_scaffold*	21649	T/C	NA	INTERGENIC	NA
Kabuli_wild_21653	1399947063	*Ca_kabuli_scaffold*	21653	G/A	NA	INTERGENIC	NA
Kabuli_wild_4415	1399947064	*Ca_kabuli_scaffold*	4415	A/G	NA	INTERGENIC	NA
Kabuli_wild_460	1399947065	*Ca_kabuli_scaffold*	460	T/C	NA	INTERGENIC	NA
Kabuli_wild_471	1399947066	*Ca_kabuli_scaffold*	471	C/T	NA	INTERGENIC	NA
Kabuli_wild_472	1399947067	*Ca_kabuli_scaffold*	472	C/T	NA	INTERGENIC	NA
Kabuli_wild_520	1399947068	*Ca_kabuli_scaffold*	520	T/G	NA	INTERGENIC	NA
Kabuli_wild_592	1399947069	*Ca_kabuli_scaffold*	592	A/G	NA	INTERGENIC	NA
Kabuli_wild_2056	1399947070	*Ca_kabuli_scaffold*	2056	A/T	NA	INTERGENIC	NA
Kabuli_wild_2073	1399947071	*Ca_kabuli_scaffold*	2073	G/A	NA	INTERGENIC	NA

SNP IDs	NCBI dbSNP accession IDs (NCBI_SS#)	Chromosomes	Physical positions (bp)	SNPs	Gene accession IDs	Sequence components of genome	Putative functions
Kabuli_wild_3911	1399947072	*Ca_kabuli_scaffold*	3911	T/C	NA	INTERGENIC	NA
Kabuli_wild_3941	1399947073	*Ca_kabuli_scaffold*	3941	G/A	NA	INTERGENIC	NA
Kabuli_wild_3946	1399947074	*Ca_kabuli_scaffold*	3946	T/G	NA	INTERGENIC	NA
Kabuli_wild_3966	1399947075	*Ca_kabuli_scaffold*	3966	A/C	NA	INTERGENIC	NA
Kabuli_wild_3967	1399947076	*Ca_kabuli_scaffold*	3967	G/A	NA	INTERGENIC	NA
Kabuli_wild_3977	1399947077	*Ca_kabuli_scaffold*	3977	A/G	NA	INTERGENIC	NA
Kabuli_wild_4007	1399947078	*Ca_kabuli_scaffold*	4007	T/G	NA	INTERGENIC	NA
Kabuli_wild_4022	1399947079	*Ca_kabuli_scaffold*	4022	C/T	NA	INTERGENIC	NA
Kabuli_wild_4025	1399947080	*Ca_kabuli_scaffold*	4025	T/C	NA	INTERGENIC	NA
Kabuli_wild_4052	1399947081	*Ca_kabuli_scaffold*	4052	T/G	NA	INTERGENIC	NA
Kabuli_wild_4057	1399947082	*Ca_kabuli_scaffold*	4057	G/C	NA	INTERGENIC	NA
Kabuli_wild_747	1399947083	*Ca_kabuli_scaffold*	747	G/T	NA	INTERGENIC	NA
Kabuli_wild_764	1399947084	*Ca_kabuli_scaffold*	764	C/T	NA	INTERGENIC	NA
Kabuli_wild_795	1399947085	*Ca_kabuli_scaffold*	795	A/G	NA	INTERGENIC	NA
Kabuli_wild_796	1399947086	*Ca_kabuli_scaffold*	796	G/C	NA	INTERGENIC	NA
Kabuli_wild_805	1399947087	*Ca_kabuli_scaffold*	805	A/T	NA	INTERGENIC	NA
Kabuli_wild_821	1399947088	*Ca_kabuli_scaffold*	821	T/A	NA	INTERGENIC	NA
Kabuli_wild_20473	1399947089	*Ca_kabuli_scaffold*	20473	T/A	NA	INTERGENIC	NA
Kabuli_wild_1167	1399947090	*Ca_kabuli_scaffold*	1167	C/T	NA	INTERGENIC	NA
Kabuli_wild_1205	1399947091	*Ca_kabuli_scaffold*	1205	A/G	NA	INTERGENIC	NA
Kabuli_wild_5263	1399947092	*Ca_kabuli_scaffold*	5263	A/G	NA	INTERGENIC	NA
Kabuli_wild_1183	1399947093	*Ca_kabuli_scaffold*	1183	G/A	NA	INTERGENIC	NA
Kabuli_wild_402	1399947094	*Ca_kabuli_scaffold*	402	G/A	NA	INTERGENIC	NA
Kabuli_wild_19266	1399947095	*Ca_kabuli_scaffold*	19266	G/A	Gene_Ca_27631	SYNONYMOUS_CODING	NA
Kabuli_wild_112	1399947096	*Ca_kabuli_scaffold*	112	G/A	NA	INTERGENIC	NA
Kabuli_wild_409	1399947097	*Ca_kabuli_scaffold*	409	T/G	NA	INTERGENIC	NA
Kabuli_wild_378	1399947098	*Ca_kabuli_scaffold*	378	A/T	Gene_Ca_28205	UPSTREAM	NA
Kabuli_wild_397	1399947099	*Ca_kabuli_scaffold*	397	G/A	Gene_Ca_28205	UPSTREAM	NA
Kabuli_wild_410	1399947100	*Ca_kabuli_scaffold*	410	A/C	Gene_Ca_28205	UPSTREAM	NA
Kabuli_wild_573	1399947101	*Ca_kabuli_scaffold*	573	T/G	Gene_Ca_28205	UPSTREAM	NA
Kabuli_wild_806	1399947102	*Ca_kabuli_scaffold*	806	A/C	NA	INTERGENIC	NA
Kabuli_wild_807	1399947103	*Ca_kabuli_scaffold*	807	G/A	NA	INTERGENIC	NA
Kabuli_wild_808	1399947104	*Ca_kabuli_scaffold*	808	T/G	NA	INTERGENIC	NA
Kabuli_wild_31673	1399947105	*Ca_kabuli_scaffold*	31673	G/A	NA	INTERGENIC	NA
Kabuli_wild_40193	1399947106	*Ca_kabuli_scaffold*	40193	G/T	NA	INTERGENIC	NA
Kabuli_wild_156485	1399947107	*Ca_kabuli_scaffold*	156485	C/T	Gene_Ca_11572	INTRON	MembraneboundO-acyltransferase,MBOAT
Kabuli_wild_328757	1399947108	*Ca_kabuli_scaffold*	328757	C/T	Gene_Ca_11580	NON_SYNONYMOUS_CODING	NA
Kabuli_wild_385653	1399947109	*Ca_kabuli_scaffold*	385653	C/T	NA	INTERGENIC	NA
Kabuli_wild_562892	1399947110	*Ca_kabuli_scaffold*	562892	G/T	NA	INTERGENIC	NA
Kabuli_wild_562907	1399947111	*Ca_kabuli_scaffold*	562907	A/C	NA	INTERGENIC	NA
Kabuli_wild_696869	1399947112	*Ca_kabuli_scaffold*	696869	A/C	Gene_Ca_11606	INTRON	TetratricopeptideTPR-1
Kabuli_wild_705224	1399947113	*Ca_kabuli_scaffold*	705224	G/T	Gene_Ca_11606	INTRON	TetratricopeptideTPR-1
Kabuli_wild_741607	1399947114	*Ca_kabuli_scaffold*	741607	G/A	Gene_Ca_11609	SYNONYMOUS_CODING	uDENN
Kabuli_wild_52124	1399947115	*Ca_kabuli_scaffold*	52124	C/T	NA	INTERGENIC	NA

SNP IDs	NCBI dbSNP accession IDs (NCBI_SS#)	Chromosomes	Physical positions (bp)	SNPs	Gene accession IDs	Sequence components of genome	Putative functions
Kabuli_wild_52125	1399947116	*Ca_kabuli_scaffold*	52125	C/T	NA	INTERGENIC	NA
Kabuli_wild_52142	1399947117	*Ca_kabuli_scaffold*	52142	G/T	NA	INTERGENIC	NA
Kabuli_wild_52146	1399947118	*Ca_kabuli_scaffold*	52146	T/C	NA	INTERGENIC	NA
Kabuli_wild_330840	1399947119	*Ca_kabuli_scaffold*	330840	C/A	NA	INTERGENIC	NA
Kabuli_wild_45565	1399947120	*Ca_kabuli_scaffold*	45565	A/C	Gene_Ca_11165	SYNONYMOUS_CODING	NA
Kabuli_wild_45566	1399947121	*Ca_kabuli_scaffold*	45566	G/A	Gene_Ca_11165	SYNONYMOUS_CODING	NA
Kabuli_wild_58243	1399947122	*Ca_kabuli_scaffold*	58243	A/G	Gene_Ca_11166	DOWNSTREAM	Methyltransferasetype11
Kabuli_wild_154342	1399947123	*Ca_kabuli_scaffold*	154342	A/C	Gene_Ca_11177	UPSTREAM	Vacuolarsortingprotein9
Kabuli_wild_45461	1399947124	*Ca_kabuli_scaffold*	45461	G/T	Gene_Ca_08692	UPSTREAM	YABBYprotein
Kabuli_wild_118051	1399947125	*Ca_kabuli_scaffold*	118051	T/C	NA	INTERGENIC	NA
Kabuli_wild_122047	1399947126	*Ca_kabuli_scaffold*	122047	T/C	NA	INTERGENIC	NA
Kabuli_wild_122101	1399947127	*Ca_kabuli_scaffold*	122101	T/C	NA	INTERGENIC	NA
Kabuli_wild_134572	1399947128	*Ca_kabuli_scaffold*	134572	G/A	Gene_Ca_08700	DOWNSTREAM	C4-dicarboxylatetransporter/malicacidtransportprotein
Kabuli_wild_150694	1399947129	*Ca_kabuli_scaffold*	150694	C/G	Gene_Ca_08702	SYNONYMOUS_CODING	SNARE associated Golgi protein
Kabuli_wild_151274	1399947130	*Ca_kabuli_scaffold*	151274	A/C	Gene_Ca_08702	NON_SYNONYMOUS_CODING	SNARE associated Golgi protein
Kabuli_wild_184453	1399947131	*Ca_kabuli_scaffold*	184453	G/A	Gene_Ca_08703	SYNONYMOUS_CODING	Aminoacid-bindingACT
Kabuli_wild_251866	1399947132	*Ca_kabuli_scaffold*	251866	A/C	Gene_Ca_08709	NON_SYNONYMOUS_CODING	NA
Kabuli_wild_272718	1399947133	*Ca_kabuli_scaffold*	272718	A/C	NA	INTERGENIC	NA
Kabuli_wild_293051	1399947134	*Ca_kabuli_scaffold*	293051	A/C	NA	INTERGENIC	NA
Kabuli_wild_380225	1399947135	*Ca_kabuli_scaffold*	380225	T/G	Gene_Ca_08725	SYNONYMOUS_CODING	PeptidaseS8/S53,subtilisin/kexin/sedolisin
Kabuli_wild_380232	1399947136	*Ca_kabuli_scaffold*	380232	G/T	Gene_Ca_08725	NON_SYNONYMOUS_CODING	PeptidaseS8/S53,subtilisin/kexin/sedolisin
Kabuli_wild_452011	1399947137	*Ca_kabuli_scaffold*	452011	G/T	Gene_Ca_08730	NON_SYNONYMOUS_CODING	Glucose-methanol-cholineoxidoreductase,N-terminal
Kabuli_wild_454342	1399947138	*Ca_kabuli_scaffold*	454342	G/T	Gene_Ca_08730	UPSTREAM	Glucose-methanol-cholineoxidoreductase,N-terminal
Kabuli_wild_535984	1399947139	*Ca_kabuli_scaffold*	535984	G/T	Gene_Ca_08741	NON_SYNONYMOUS_CODING	IQ motif,EF-handbinding site
Kabuli_wild_552173	1399947140	*Ca_kabuli_scaffold*	552173	G/A	NA	INTERGENIC	NA
Kabuli_wild_552185	1399947141	*Ca_kabuli_scaffold*	552185	T/C	NA	INTERGENIC	NA
Kabuli_wild_553863	1399947142	*Ca_kabuli_scaffold*	553863	G/T	NA	INTERGENIC	NA
Kabuli_wild_554134	1399947143	*Ca_kabuli_scaffold*	554134	A/G	NA	INTERGENIC	NA
Kabuli_wild_599470	1399947144	*Ca_kabuli_scaffold*	599470	G/A	Gene_Ca_08749	SYNONYMOUS_CODING	PeptidaseS8/S53,subtilisin/kexin/sedolisin
Kabuli_wild_652067	1399947145	*Ca_kabuli_scaffold*	652067	A/G	Gene_Ca_08753	NON_SYNONYMOUS_CODING	PeptidaseS8/S53,subtilisin/kexin/sedolisin
Kabuli_wild_667415	1399947146	*Ca_kabuli_scaffold*	667415	T/G	Gene_Ca_08754	SYNONYMOUS_CODING	PeptidaseS8/S53,subtilisin/kexin/sedolisin
Kabuli_wild_667541	1399947147	*Ca_kabuli_scaffold*	667541	A/C	Gene_Ca_08754	SYNONYMOUS_CODING	PeptidaseS8/S53,subtilisin/kexin/sedolisin
Kabuli_wild_730174	1399947148	*Ca_kabuli_scaffold*	730174	A/C	Gene_Ca_08761	SYNONYMOUS_CODING	Fork head-associated (FHA) domain
Kabuli_wild_732450	1399947149	*Ca_kabuli_scaffold*	732450	A/C	Gene_Ca_08761	SYNONYMOUS_CODING	Fork head-associated (FHA) domain
Kabuli_wild_732454	1399947150	*Ca_kabuli_scaffold*	732454	T/G	Gene_Ca_08761	INTRON	Fork head-associated (FHA) domain
Kabuli_wild_732460	1399947151	*Ca_kabuli_scaffold*	732460	T/A	Gene_Ca_08761	INTRON	Fork head-associated (FHA) domain
Kabuli_wild_732463	1399947152	*Ca_kabuli_scaffold*	732463	G/T	Gene_Ca_08761	INTRON	Fork head-associated (FHA) domain
Kabuli_wild_732496	1399947153	*Ca_kabuli_scaffold*	732496	G/T	Gene_Ca_08761	INTRON	Fork head-associated (FHA) domain
Kabuli_wild_750213	1399947154	*Ca_kabuli_scaffold*	750213	C/A	Gene_Ca_08762	INTRON	COGcomplexcomponent,COG2
Kabuli_wild_771102	1399947155	*Ca_kabuli_scaffold*	771102	C/T	Gene_Ca_08765	DOWNSTREAM	FBD
Kabuli_wild_909909	1399947156	*Ca_kabuli_scaffold*	909909	A/G	NA	INTERGENIC	NA
Kabuli_wild_959608	1399947157	*Ca_kabuli_scaffold*	959608	A/C	Gene_Ca_08787	NON_SYNONYMOUS_CODING	Transcription factor jumonji/aspartylbeta-hydroxylase
Kabuli_wild_959772	1399947158	*Ca_kabuli_scaffold*	959772	C/T	Gene_Ca_08787	NON_SYNONYMOUS_CODING	Transcription factor jumonji/aspartylbeta-hydroxylase
Kabuli_wild_959828	1399947159	*Ca_kabuli_scaffold*	959828	G/A	Gene_Ca_08787	SYNONYMOUS_CODING	Transcription factor jumonji/aspartylbeta-hydroxylase

SNP IDs	NCBI dbSNP accession IDs (NCBI_SS#)	Chromosomes	Physical positions (bp)	SNPs	Gene accession IDs	Sequence components of genome	Putative functions
Kabuli_wild_965884	1399947160	*Ca_kabuli_scaffold*	965884	T/C	Gene_Ca_08787	INTRON	Transcription factor jumonji/aspartylbeta-hydroxylase
Kabuli_wild_965961	1399947161	*Ca_kabuli_scaffold*	965961	A/G	Gene_Ca_08787	INTRON	Transcription factor jumonji/aspartylbeta-hydroxylase
Kabuli_wild_1020651	1399947162	*Ca_kabuli_scaffold*	1020651	A/G	NA	INTERGENIC	NA
Kabuli_wild_1022091	1399947163	*Ca_kabuli_scaffold*	1022091	T/C	NA	INTERGENIC	NA
Kabuli_wild_1066818	1399947164	*Ca_kabuli_scaffold*	1066818	A/C	Gene_Ca_08793	INTRON	Peptidase,cysteine peptidase active site
Kabuli_wild_1072750	1399947165	*Ca_kabuli_scaffold*	1072750	C/T	Gene_Ca_08794	SYNONYMOUS_CODING	WD40 repeat
Kabuli_wild_1265627	1399947166	*Ca_kabuli_scaffold*	1265627	T/C	Gene_Ca_08816	SYNONYMOUS_CODING	Protein kinase,catalytic domain
Kabuli_wild_1265635	1399947167	*Ca_kabuli_scaffold*	1265635	T/G	Gene_Ca_08816	NON_SYNONYMOUS_CODING	Protein kinase,catalytic domain
Kabuli_wild_1273341	1399947168	*Ca_kabuli_scaffold*	1273341	G/A	Gene_Ca_08817	DOWNSTREAM	NA
Kabuli_wild_1285241	1399947169	*Ca_kabuli_scaffold*	1285241	G/A	NA	INTERGENIC	NA
Kabuli_wild_1285272	1399947170	*Ca_kabuli_scaffold*	1285272	T/G	NA	INTERGENIC	NA
Kabuli_wild_1285320	1399947171	*Ca_kabuli_scaffold*	1285320	T/G	NA	INTERGENIC	NA
Kabuli_wild_1285324	1399947172	*Ca_kabuli_scaffold*	1285324	T/G	NA	INTERGENIC	NA
Kabuli_wild_1288448	1399947173	*Ca_kabuli_scaffold*	1288448	A/G	Gene_Ca_08820	INTRON	RNAhelicase,ATP-dependent,DEAD-box,conservedsite
Kabuli_wild_1296453	1399947174	*Ca_kabuli_scaffold*	1296453	G/A	NA	INTERGENIC	NA
Kabuli_wild_1320047	1399947175	*Ca_kabuli_scaffold*	1320047	C/G	NA	INTERGENIC	NA
Kabuli_wild_1330288	1399947176	*Ca_kabuli_scaffold*	1330288	A/G	NA	INTERGENIC	NA
Kabuli_wild_1330353	1399947177	*Ca_kabuli_scaffold*	1330353	T/A	NA	INTERGENIC	NA
Kabuli_wild_1330400	1399947178	*Ca_kabuli_scaffold*	1330400	A/G	NA	INTERGENIC	NA
Kabuli_wild_1398870	1399947179	*Ca_kabuli_scaffold*	1398870	C/T	Gene_Ca_08830	DOWNSTREAM	Kinetochore-Ndc80complex,subunitSpc25
Kabuli_wild_1403480	1399947180	*Ca_kabuli_scaffold*	1403480	A/G	NA	INTERGENIC	NA
Kabuli_wild_143813	1399947181	*Ca_kabuli_scaffold*	143813	C/G	Gene_Ca_10872	NON_SYNONYMOUS_CODING	Nucleoporin,Nsp1-like,C-terminal
Kabuli_wild_144354	1399947182	*Ca_kabuli_scaffold*	144354	C/T	Gene_Ca_10872	SYNONYMOUS_CODING	Nucleoporin,Nsp1-like,C-terminal
Kabuli_wild_144486	1399947183	*Ca_kabuli_scaffold*	144486	T/G	Gene_Ca_10872	SYNONYMOUS_CODING	Nucleoporin,Nsp1-like,C-terminal
Kabuli_wild_152512	1399947184	*Ca_kabuli_scaffold*	152512	T/G	Gene_Ca_10872	NON_SYNONYMOUS_CODING	Nucleoporin,Nsp1-like,C-terminal
Kabuli_wild_152576	1399947185	*Ca_kabuli_scaffold*	152576	G/T	Gene_Ca_10872	DOWNSTREAM	Nucleoporin,Nsp1-like,C-terminal
Kabuli_wild_244666	1399947186	*Ca_kabuli_scaffold*	244666	G/A	Gene_Ca_10878	SYNONYMOUS_CODING	Peptidase C48, SUMO/Sentrin/Ubl1
Kabuli_wild_266962	1399947187	*Ca_kabuli_scaffold*	266962	T/C	NA	INTERGENIC	NA
Kabuli_wild_266974	1399947188	*Ca_kabuli_scaffold*	266974	G/T	NA	INTERGENIC	NA
Kabuli_wild_266975	1399947189	*Ca_kabuli_scaffold*	266975	G/A	NA	INTERGENIC	NA
Kabuli_wild_266980	1399947190	*Ca_kabuli_scaffold*	266980	G/A	NA	INTERGENIC	NA
Kabuli_wild_266994	1399947191	*Ca_kabuli_scaffold*	266994	A/G	NA	INTERGENIC	NA
Kabuli_wild_267001	1399947192	*Ca_kabuli_scaffold*	267001	A/T	NA	INTERGENIC	NA
Kabuli_wild_267006	1399947193	*Ca_kabuli_scaffold*	267006	T/C	NA	INTERGENIC	NA
Kabuli_wild_15576	1399947194	*Ca_kabuli_scaffold*	15576	G/A	Gene_Ca_16296	NON_SYNONYMOUS_CODING	PeptidaseA1
Kabuli_wild_15601	1399947195	*Ca_kabuli_scaffold*	15601	T/G	Gene_Ca_16296	NON_SYNONYMOUS_CODING	PeptidaseA1
Kabuli_wild_15616	1399947196	*Ca_kabuli_scaffold*	15616	A/G	Gene_Ca_16296	SYNONYMOUS_CODING	PeptidaseA1
Kabuli_wild_186203	1399947197	*Ca_kabuli_scaffold*	186203	C/G	Gene_Ca_16315	UPSTREAM	Oxysterol-bindingprotein
Kabuli_wild_186218	1399947198	*Ca_kabuli_scaffold*	186218	G/A	Gene_Ca_16315	UPSTREAM	Oxysterol-bindingprotein
Kabuli_wild_186243	1399947199	*Ca_kabuli_scaffold*	186243	A/C	Gene_Ca_16315	UPSTREAM	Oxysterol-bindingprotein
Kabuli_wild_249521	1399947200	*Ca_kabuli_scaffold*	249521	A/T	Gene_Ca_16321	SYNONYMOUS_CODING	Transaldolase
Kabuli_wild_82130	1399947201	*Ca_kabuli_scaffold*	82130	A/C	Gene_Ca_11944	SYNONYMOUS_CODING	Protein of unknown function DUF604
Kabuli_wild_105461	1399947202	*Ca_kabuli_scaffold*	105461	A/G	NA	INTERGENIC	NA
Kabuli_wild_105465	1399947203	*Ca_kabuli_scaffold*	105465	C/T	NA	INTERGENIC	NA

SNP IDs	NCBI dbSNP accession IDs (NCBI_SS#)	Chromosomes	Physical positions (bp)	SNPs	Gene accession IDs	Sequence components of genome	Putative functions
Kabuli_wild_105521	1399947204	*Ca_kabuli_scaffold*	105521	A/C	NA	INTERGENIC	NA
Kabuli_wild_105523	1399947205	*Ca_kabuli_scaffold*	105523	G/A	NA	INTERGENIC	NA
Kabuli_wild_105541	1399947206	*Ca_kabuli_scaffold*	105541	G/A	NA	INTERGENIC	NA
Kabuli_wild_105546	1399947207	*Ca_kabuli_scaffold*	105546	C/T	NA	INTERGENIC	NA
Kabuli_wild_140540	1399947208	*Ca_kabuli_scaffold*	140540	T/C	Gene_Ca_11948	INTRON	Pheromoneshutdown-related,TraB
Kabuli_wild_148483	1399947209	*Ca_kabuli_scaffold*	148483	C/A	Gene_Ca_11948	INTRON	Pheromoneshutdown-related,TraB
Kabuli_wild_148486	1399947210	*Ca_kabuli_scaffold*	148486	C/T	Gene_Ca_11948	INTRON	Pheromoneshutdown-related,TraB
Kabuli_wild_148504	1399947211	*Ca_kabuli_scaffold*	148504	G/T	Gene_Ca_11948	INTRON	Pheromoneshutdown-related,TraB
Kabuli_wild_166935	1399947212	*Ca_kabuli_scaffold*	166935	C/T	Gene_Ca_11949	INTRON	NA
Kabuli_wild_78650	1399947213	*Ca_kabuli_scaffold*	78650	A/G	NA	INTERGENIC	NA
Kabuli_wild_142859	1399947214	*Ca_kabuli_scaffold*	142859	G/C	NA	INTERGENIC	NA
Kabuli_wild_313139	1399947215	*Ca_kabuli_scaffold*	313139	A/T	Gene_Ca_22882	INTRON	NA
Kabuli_wild_313160	1399947216	*Ca_kabuli_scaffold*	313160	A/G	Gene_Ca_22882	INTRON	NA
Kabuli_wild_341136	1399947217	*Ca_kabuli_scaffold*	341136	A/C	NA	INTERGENIC	NA
Kabuli_wild_378975	1399947218	*Ca_kabuli_scaffold*	378975	G/A	NA	INTERGENIC	NA
Kabuli_wild_446882	1399947219	*Ca_kabuli_scaffold*	446882	G/A	Gene_Ca_22884	NON_SYNONYMOUS_CODING	Peptidase C48, SUMO/Sentrin/Ubl1
Kabuli_wild_446912	1399947220	*Ca_kabuli_scaffold*	446912	C/T	Gene_Ca_22884	SYNONYMOUS_CODING	Peptidase C48, SUMO/Sentrin/Ubl1
Kabuli_wild_446931	1399947221	*Ca_kabuli_scaffold*	446931	C/T	Gene_Ca_22884	NON_SYNONYMOUS_CODING	Peptidase C48, SUMO/Sentrin/Ubl1
Kabuli_wild_446954	1399947222	*Ca_kabuli_scaffold*	446954	C/A	Gene_Ca_22884	NON_SYNONYMOUS_CODING	Peptidase C48, SUMO/Sentrin/Ubl1
Kabuli_wild_581413	1399947223	*Ca_kabuli_scaffold*	581413	A/G	NA	INTERGENIC	NA
Kabuli_wild_26108	1399947224	*Ca_kabuli_scaffold*	26108	G/T	Gene_Ca_17229	INTRON	Fork head-associated (FHA) domain
Kabuli_wild_26121	1399947225	*Ca_kabuli_scaffold*	26121	A/C	Gene_Ca_17229	INTRON	Fork head-associated (FHA) domain
Kabuli_wild_26134	1399947226	*Ca_kabuli_scaffold*	26134	C/A	Gene_Ca_17229	INTRON	Fork head-associated (FHA) domain
Kabuli_wild_144885	1399947227	*Ca_kabuli_scaffold*	144885	T/C	Gene_Ca_17233	INTRON	RNA recognition motif domain
Kabuli_wild_161190	1399947228	*Ca_kabuli_scaffold*	161190	C/A	Gene_Ca_17234	INTRON	Cyclin
Kabuli_wild_260488	1399947229	*Ca_kabuli_scaffold*	260488	A/C	NA	INTERGENIC	NA
Kabuli_wild_299	1399947230	*Ca_kabuli_scaffold*	299	A/T	NA	INTERGENIC	NA
Kabuli_wild_306	1399947231	*Ca_kabuli_scaffold*	306	T/C	NA	INTERGENIC	NA
Kabuli_wild_342	1399947232	*Ca_kabuli_scaffold*	342	C/T	NA	INTERGENIC	NA
Kabuli_wild_349	1399947233	*Ca_kabuli_scaffold*	349	C/T	NA	INTERGENIC	NA
Kabuli_wild_550	1399947234	*Ca_kabuli_scaffold*	550	T/G	NA	INTERGENIC	NA
Kabuli_wild_593	1399947235	*Ca_kabuli_scaffold*	593	A/G	NA	INTERGENIC	NA
Kabuli_wild_614	1399947236	*Ca_kabuli_scaffold*	614	C/T	NA	INTERGENIC	NA
Kabuli_wild_616	1399947237	*Ca_kabuli_scaffold*	616	A/G	NA	INTERGENIC	NA
Kabuli_wild_649	1399947238	*Ca_kabuli_scaffold*	649	T/C	NA	INTERGENIC	NA
Kabuli_wild_654	1399947239	*Ca_kabuli_scaffold*	654	C/A	NA	INTERGENIC	NA
Kabuli_wild_689	1399947240	*Ca_kabuli_scaffold*	689	G/A	NA	INTERGENIC	NA
Kabuli_wild_323	1399947241	*Ca_kabuli_scaffold*	323	T/G	NA	INTERGENIC	NA
Kabuli_wild_416	1399947242	*Ca_kabuli_scaffold*	416	G/A	NA	INTERGENIC	NA
Kabuli_wild_925	1399947243	*Ca_kabuli_scaffold*	925	C/T	NA	INTERGENIC	NA
Kabuli_wild_341	1399947244	*Ca_kabuli_scaffold*	341	G/A	NA	INTERGENIC	NA
Kabuli_wild_381	1399947245	*Ca_kabuli_scaffold*	381	T/A	NA	INTERGENIC	NA
Kabuli_wild_1412	1399947246	*Ca_kabuli_scaffold*	1412	A/T	NA	INTERGENIC	NA
Kabuli_wild_1416	1399947247	*Ca_kabuli_scaffold*	1416	T/A	NA	INTERGENIC	NA

SNP IDs	NCBI dbSNP accession IDs (NCBI_SS#)	Chromosomes	Physical positions (bp)	SNPs	Gene accession IDs	Sequence components of genome	Putative functions
Kabuli_wild_1418	1399947248	*Ca_kabuli_scaffold*	1418	A/C	NA	INTERGENIC	NA
Kabuli_wild_1422	1399947249	*Ca_kabuli_scaffold*	1422	G/C	NA	INTERGENIC	NA
Kabuli_wild_1550	1399947250	*Ca_kabuli_scaffold*	1550	T/G	NA	INTERGENIC	NA
Kabuli_wild_1568	1399947251	*Ca_kabuli_scaffold*	1568	A/C	NA	INTERGENIC	NA
Kabuli_wild_656	1399947252	*Ca_kabuli_scaffold*	656	G/A	NA	INTERGENIC	NA
Kabuli_wild_624	1399947253	*Ca_kabuli_scaffold*	624	A/C	NA	INTERGENIC	NA
Kabuli_wild_641	1399947254	*Ca_kabuli_scaffold*	641	A/C	NA	INTERGENIC	NA
Kabuli_wild_2177	1399947255	*Ca_kabuli_scaffold*	2177	G/A	NA	INTERGENIC	NA
Kabuli_wild_3299	1399947256	*Ca_kabuli_scaffold*	3299	A/C	Gene_Ca_27871	INTRON	RibosomalproteinS7
Kabuli_wild_1389	1399947257	*Ca_kabuli_scaffold*	1389	A/T	Gene_Ca_27926	INTRON	DNA-directed RNA polymerase,subunit 2, domain 6
Kabuli_wild_3004	1399947258	*Ca_kabuli_scaffold*	3004	C/A	Gene_Ca_28253	INTRON	Domain of unknown function DUF231,plant
Kabuli_wild_3028	1399947259	*Ca_kabuli_scaffold*	3028	C/T	Gene_Ca_28253	INTRON	Domain of unknown function DUF231,plant
Kabuli_wild_2344	1399947260	*Ca_kabuli_scaffold*	2344	G/A	NA	INTERGENIC	NA
Kabuli_wild_2474	1399947261	*Ca_kabuli_scaffold*	2474	T/A	NA	INTERGENIC	NA
Kabuli_wild_3190	1399947262	*Ca_kabuli_scaffold*	3190	T/G	NA	INTERGENIC	NA
Kabuli_wild_3284	1399947263	*Ca_kabuli_scaffold*	3284	C/G	NA	INTERGENIC	NA
Kabuli_wild_766	1399947264	*Ca_kabuli_scaffold*	766	C/G	Gene_Ca_28268	INTRON	GCN5-related N-acetyltransferase (GNAT) domain
Kabuli_wild_2152	1399947265	*Ca_kabuli_scaffold*	2152	G/C	Gene_Ca_28268	INTRON	GCN5-related N-acetyltransferase (GNAT) domain
Kabuli_wild_2169	1399947266	*Ca_kabuli_scaffold*	2169	T/A	Gene_Ca_28268	INTRON	NA
Kabuli_wild_1464	1399947267	*Ca_kabuli_scaffold*	1464	A/T	Gene_Ca_27806	NON_SYNONYMOUS_CODING	NA
Kabuli_wild_3295	1399947268	*Ca_kabuli_scaffold*	3295	G/A	Gene_Ca_27806	DOWNSTREAM	NA
Kabuli_wild_3533	1399947269	*Ca_kabuli_scaffold*	3533	G/A	Gene_Ca_27806	DOWNSTREAM	NA
Kabuli_wild_4522	1399947270	*Ca_kabuli_scaffold*	4522	A/G	Gene_Ca_28216	NON_SYNONYMOUS_CODING	Cytochrome P450
Kabuli_wild_6174	1399947271	*Ca_kabuli_scaffold*	6174	G/T	NA	INTERGENIC	NA
Kabuli_wild_6178	1399947272	*Ca_kabuli_scaffold*	6178	C/G	NA	INTERGENIC	NA
Kabuli_wild_6210	1399947273	*Ca_kabuli_scaffold*	6210	G/A	NA	INTERGENIC	NA
Kabuli_wild_5784	1399947274	*Ca_kabuli_scaffold*	5784	G/A	Gene_Ca_28056	NON_SYNONYMOUS_CODING	Protein of unknown function DUF2930
Kabuli_wild_148	1399947275	*Ca_kabuli_scaffold*	148	C/T	NA	INTERGENIC	NA
Kabuli_wild_154	1399947276	*Ca_kabuli_scaffold*	154	C/A	NA	INTERGENIC	NA
Kabuli_wild_644	1399947277	*Ca_kabuli_scaffold*	644	A/G	NA	INTERGENIC	NA
Kabuli_wild_1744	1399947278	*Ca_kabuli_scaffold*	1744	T/C	NA	INTERGENIC	NA
Kabuli_wild_3929	1399947279	*Ca_kabuli_scaffold*	3929	T/G	Gene_Ca_27727	NON_SYNONYMOUS_CODING	ABC transporter,trans membrane domain
Kabuli_wild_3936	1399947280	*Ca_kabuli_scaffold*	3936	G/T	Gene_Ca_27727	NON_SYNONYMOUS_CODING	ABC transporter,trans membrane domain
Kabuli_wild_3952	1399947281	*Ca_kabuli_scaffold*	3952	T/C	Gene_Ca_27727	NON_SYNONYMOUS_CODING	ABC transporter,trans membrane domain
Kabuli_wild_2515	1399947282	*Ca_kabuli_scaffold*	2515	T/A	Gene_Ca_28257	INTRON	U3smallnucleolarRNA-associatedprotein10
Kabuli_wild_2529	1399947283	*Ca_kabuli_scaffold*	2529	T/G	Gene_Ca_28257	INTRON	U3smallnucleolarRNA-associatedprotein10
Kabuli_wild_3697	1399947284	*Ca_kabuli_scaffold*	3697	A/T	Gene_Ca_28257	INTRON	U3smallnucleolarRNA-associatedprotein10
Kabuli_wild_3808	1399947285	*Ca_kabuli_scaffold*	3808	G/A	Gene_Ca_28257	SYNONYMOUS_CODING	U3smallnucleolarRNA-associatedprotein10
Kabuli_wild_5324	1399947286	*Ca_kabuli_scaffold*	5324	G/A	Gene_Ca_28257	INTRON	U3smallnucleolarRNA-associatedprotein10
Kabuli_wild_2620	1399947287	*Ca_kabuli_scaffold*	2620	A/C	NA	INTERGENIC	NA
Kabuli_wild_2679	1399947288	*Ca_kabuli_scaffold*	2679	C/T	NA	INTERGENIC	NA
Kabuli_wild_2696	1399947289	*Ca_kabuli_scaffold*	2696	A/C	NA	INTERGENIC	NA
Kabuli_wild_2761	1399947290	*Ca_kabuli_scaffold*	2761	T/C	NA	INTERGENIC	NA
Kabuli_wild_10642	1399947291	*Ca_kabuli_scaffold*	10642	G/C	Gene_Ca_28266	SYNONYMOUS_CODING	Disease resistance protein

SNP IDs	NCBI dbSNP accession IDs (NCBI_SS#)	Chromosomes	Physical positions (bp)	SNPs	Gene accession IDs	Sequence components of genome	Putative functions
Kabuli_wild_12936	1399947292	*Ca_kabuli_scaffold*	12936	T/G	Gene_Ca_28087	SYNONYMOUS_CODING	DNAtopoisomeraseII,eukaryotic-type
Desi_wild_286	NA	*Ca_desi_Chr01*	286	C/A	NA	INTERGENIC	NA
Desi_wild_179189	NA	*Ca_desi_Chr01*	179189	A/C	Gene_Ca_00004.1	INTRON	Putative lipase YOR059C
Desi_wild_382862	NA	*Ca_desi_Chr01*	382862	T/C	Gene_Ca_00011.1	SYNONYMOUS_CODING	Eukaryotic translation initiation factor 3 subunit C
Desi_wild_382872	NA	*Ca_desi_Chr01*	382872	G/A	Gene_Ca_00011.1	NON_SYNONYMOUS_CODING	Eukaryotic translation initiation factor 3 subunit C
Desi_wild_382898	NA	*Ca_desi_Chr01*	382898	T/C	Gene_Ca_00011.1	SYNONYMOUS_CODING	Eukaryotic translation initiation factor 3 subunit C
Desi_wild_436895	NA	*Ca_desi_Chr01*	436895	A/C	Gene_Ca_00021.1	DOWNSTREAM	Ribosome biogenesis protein BOP1 homolog
Desi_wild_461870	NA	*Ca_desi_Chr01*	461870	A/G	Gene_Ca_00023.1	SYNONYMOUS_CODING	Magnesium transporter NIPA2
Desi_wild_462205	NA	*Ca_desi_Chr01*	462205	G/A	Gene_Ca_00023.1	INTRON	Magnesium transporter NIPA2
Desi_wild_477493	NA	*Ca_desi_Chr01*	477493	C/G	NA	INTERGENIC	NA
Desi_wild_498862	NA	*Ca_desi_Chr01*	498862	A/G	NA	INTERGENIC	NA
Desi_wild_513254	NA	*Ca_desi_Chr01*	513254	G/T	NA	INTERGENIC	NA
Desi_wild_513710	NA	*Ca_desi_Chr01*	513710	T/C	NA	INTERGENIC	NA
Desi_wild_517439	NA	*Ca_desi_Chr01*	517439	A/G	Gene_Ca_00030.1	DOWNSTREAM	MLO-like protein 4
Desi_wild_517512	NA	*Ca_desi_Chr01*	517512	T/C	Gene_Ca_00030.1	DOWNSTREAM	MLO-like protein 4
Desi_wild_527099	NA	*Ca_desi_Chr01*	527099	A/G	NA	INTERGENIC	NA
Desi_wild_527113	NA	*Ca_desi_Chr01*	527113	G/T	NA	INTERGENIC	NA
Desi_wild_527118	NA	*Ca_desi_Chr01*	527118	T/C	NA	INTERGENIC	NA
Desi_wild_527135	NA	*Ca_desi_Chr01*	527135	G/A	NA	INTERGENIC	NA
Desi_wild_527142	NA	*Ca_desi_Chr01*	527142	G/T	NA	INTERGENIC	NA
Desi_wild_527148	NA	*Ca_desi_Chr01*	527148	C/T	NA	INTERGENIC	NA
Desi_wild_527176	NA	*Ca_desi_Chr01*	527176	T/C	NA	INTERGENIC	NA
Desi_wild_527181	NA	*Ca_desi_Chr01*	527181	C/A	NA	INTERGENIC	NA
Desi_wild_533125	NA	*Ca_desi_Chr01*	533125	C/T	NA	INTERGENIC	NA
Desi_wild_533127	NA	*Ca_desi_Chr01*	533127	T/A	NA	INTERGENIC	NA
Desi_wild_533182	NA	*Ca_desi_Chr01*	533182	T/A	NA	INTERGENIC	NA
Desi_wild_537785	NA	*Ca_desi_Chr01*	537785	T/G	NA	INTERGENIC	NA
Desi_wild_537937	NA	*Ca_desi_Chr01*	537937	T/C	NA	INTERGENIC	NA
Desi_wild_537971	NA	*Ca_desi_Chr01*	537971	A/G	NA	INTERGENIC	NA
Desi_wild_538012	NA	*Ca_desi_Chr01*	538012	C/T	NA	INTERGENIC	NA
Desi_wild_538086	NA	*Ca_desi_Chr01*	538086	G/T	NA	INTERGENIC	NA
Desi_wild_541727	NA	*Ca_desi_Chr01*	541727	C/T	Gene_Ca_00035.1	INTRON	ATP-dependent DNA helicase Q-like 4A
Desi_wild_547907	NA	*Ca_desi_Chr01*	547907	A/G	Gene_Ca_00035.1	DOWNSTREAM	ATP-dependent DNA helicase Q-like 4A
Desi_wild_547914	NA	*Ca_desi_Chr01*	547914	C/T	Gene_Ca_00035.1	DOWNSTREAM	ATP-dependent DNA helicase Q-like 4A
Desi_wild_547961	NA	*Ca_desi_Chr01*	547961	G/C	NA	INTERGENIC	NA
Desi_wild_548203	NA	*Ca_desi_Chr01*	548203	T/C	NA	INTERGENIC	NA
Desi_wild_551496	NA	*Ca_desi_Chr01*	551496	T/G	NA	INTERGENIC	NA
Desi_wild_648015	NA	*Ca_desi_Chr01*	648015	G/A	Gene_Ca_00046.2	INTRON	UDP-glucose:glycoprotein glucosyltransferase
Desi_wild_664769	NA	*Ca_desi_Chr01*	664769	A/G	Gene_Ca_00046.2	INTRON	UDP-glucose:glycoprotein glucosyltransferase
Desi_wild_664787	NA	*Ca_desi_Chr01*	664787	T/G	Gene_Ca_00046.2	INTRON	UDP-glucose:glycoprotein glucosyltransferase
Desi_wild_664811	NA	*Ca_desi_Chr01*	664811	T/G	Gene_Ca_00046.2	INTRON	UDP-glucose:glycoprotein glucosyltransferase
Desi_wild_685373	NA	*Ca_desi_Chr01*	685373	T/C	Gene_Ca_00048.1	INTRON	Cell division control protein 48 homolog C
Desi_wild_704894	NA	*Ca_desi_Chr01*	704894	T/A	NA	INTERGENIC	NA
Desi_wild_756744	NA	*Ca_desi_Chr01*	756744	T/G	NA	INTERGENIC	NA

SNP IDs	NCBI dbSNP accession IDs (NCBI_SS#)	Chromosomes	Physical positions (bp)	SNPs	Gene accession IDs	Sequence components of genome	Putative functions
Desi_wild_792141	NA	*Ca_desi_Chr01*	792141	C/A	Gene_Ca_00065.2	DOWNSTREAM	NA
Desi_wild_807232	NA	*Ca_desi_Chr01*	807232	T/C	Gene_Ca_00067.1	NON_SYNONYMOUS_CODING	Separin
Desi_wild_836330	NA	*Ca_desi_Chr01*	836330	G/T	NA	INTERGENIC	NA
Desi_wild_836351	NA	*Ca_desi_Chr01*	836351	C/T	NA	INTERGENIC	NA
Desi_wild_836842	NA	*Ca_desi_Chr01*	836842	T/G	NA	INTERGENIC	NA
Desi_wild_836859	NA	*Ca_desi_Chr01*	836859	G/A	NA	INTERGENIC	NA
Desi_wild_845508	NA	*Ca_desi_Chr01*	845508	C/T	NA	INTERGENIC	NA
Desi_wild_872247	NA	*Ca_desi_Chr01*	872247	G/A	Gene_Ca_00076.1	INTRON	Protein FAM135A
Desi_wild_872280	NA	*Ca_desi_Chr01*	872280	C/T	Gene_Ca_00076.1	INTRON	Protein FAM135A
Desi_wild_885643	NA	*Ca_desi_Chr01*	885643	C/T	Gene_Ca_00076.1	INTRON	Protein FAM135A
Desi_wild_885745	NA	*Ca_desi_Chr01*	885745	G/T	Gene_Ca_00076.1	INTRON	Protein FAM135A
Desi_wild_965854	NA	*Ca_desi_Chr01*	965854	T/C	NA	INTERGENIC	NA
Desi_wild_965884	NA	*Ca_desi_Chr01*	965884	G/A	NA	INTERGENIC	NA
Desi_wild_965885	NA	*Ca_desi_Chr01*	965885	T/C	NA	INTERGENIC	NA
Desi_wild_968375	NA	*Ca_desi_Chr01*	968375	T/C	Gene_Ca_00084.1	SYNONYMOUS_CODING	C2 domain-containing protein C31G5.15
Desi_wild_978133	NA	*Ca_desi_Chr01*	978133	T/A	NA	INTERGENIC	NA
Desi_wild_983043	NA	*Ca_desi_Chr01*	983043	C/A	NA	INTERGENIC	NA
Desi_wild_983203	NA	*Ca_desi_Chr01*	983203	G/T	NA	INTERGENIC	NA
Desi_wild_992174	NA	*Ca_desi_Chr01*	992174	C/T	Gene_Ca_00088.1	INTRON	C2 domain-containing protein C31G5.15
Desi_wild_993275	NA	*Ca_desi_Chr01*	993275	G/T	Gene_Ca_00088.1	INTRON	C2 domain-containing protein C31G5.15
Desi_wild_993482	NA	*Ca_desi_Chr01*	993482	T/C	Gene_Ca_00088.1	SYNONYMOUS_CODING	C2 domain-containing protein C31G5.15
Desi_wild_999613	NA	*Ca_desi_Chr01*	999613	T/C	Gene_Ca_00089.1	UPSTREAM	NAC domain-containing protein 68
Desi_wild_1025329	NA	*Ca_desi_Chr01*	1025329	T/C	Gene_Ca_00092.1	NON_SYNONYMOUS_CODING	Uncharacterized 91 kDa protein in cob intron
Desi_wild_1025346	NA	*Ca_desi_Chr01*	1025346	T/G	Gene_Ca_00092.1	SYNONYMOUS_CODING	Uncharacterized 91 kDa protein in cob intron
Desi_wild_1025349	NA	*Ca_desi_Chr01*	1025349	C/A	Gene_Ca_00092.1	NON_SYNONYMOUS_CODING	Uncharacterized 91 kDa protein in cob intron
Desi_wild_1025432	NA	*Ca_desi_Chr01*	1025432	T/C	Gene_Ca_00092.1	INTRON	Uncharacterized 91 kDa protein in cob intron
Desi_wild_1025458	NA	*Ca_desi_Chr01*	1025458	C/T	Gene_Ca_00092.1	INTRON	Uncharacterized 91 kDa protein in cob intron
Desi_wild_1025590	NA	*Ca_desi_Chr01*	1025590	A/G	Gene_Ca_00092.1	INTRON	Uncharacterized 91 kDa protein in cob intron
Desi_wild_1025593	NA	*Ca_desi_Chr01*	1025593	C/A	Gene_Ca_00092.1	INTRON	Uncharacterized 91 kDa protein in cob intron
Desi_wild_1069295	NA	*Ca_desi_Chr01*	1069295	A/C	Gene_Ca_00096.1	UPSTREAM	unknown protein
Desi_wild_1069378	NA	*Ca_desi_Chr01*	1069378	C/T	Gene_Ca_00096.1	UPSTREAM	unknown protein
Desi_wild_1069394	NA	*Ca_desi_Chr01*	1069394	A/C	Gene_Ca_00096.1	UPSTREAM	unknown protein
Desi_wild_1069403	NA	*Ca_desi_Chr01*	1069403	A/G	Gene_Ca_00096.1	UPSTREAM	unknown protein
Desi_wild_1069410	NA	*Ca_desi_Chr01*	1069410	A/T	Gene_Ca_00096.1	UPSTREAM	unknown protein
Desi_wild_1069425	NA	*Ca_desi_Chr01*	1069425	A/C	Gene_Ca_00096.1	UPSTREAM	unknown protein
Desi_wild_1069436	NA	*Ca_desi_Chr01*	1069436	G/A	Gene_Ca_00096.1	UPSTREAM	unknown protein
Desi_wild_1071218	NA	*Ca_desi_Chr01*	1071218	C/T	NA	INTERGENIC	NA
Desi_wild_1071230	NA	*Ca_desi_Chr01*	1071230	C/T	NA	INTERGENIC	NA
Desi_wild_1071275	NA	*Ca_desi_Chr01*	1071275	T/C	NA	INTERGENIC	NA
Desi_wild_1071343	NA	*Ca_desi_Chr01*	1071343	G/A	NA	INTERGENIC	NA
Desi_wild_1072468	NA	*Ca_desi_Chr01*	1072468	C/T	NA	INTERGENIC	NA
Desi_wild_1072524	NA	*Ca_desi_Chr01*	1072524	A/T	NA	INTERGENIC	NA
Desi_wild_1072532	NA	*Ca_desi_Chr01*	1072532	C/T	NA	INTERGENIC	NA
Desi_wild_1072608	NA	*Ca_desi_Chr01*	1072608	G/C	NA	INTERGENIC	NA

SNP IDs	NCBI dbSNP accession IDs (NCBI_SS#)	Chromosomes	Physical positions (bp)	SNPs	Gene accession IDs	Sequence components of genome	Putative functions
Desi_wild_1072623	NA	*Ca_desi_Chr01*	1072623	G/A	NA	INTERGENIC	NA
Desi_wild_1072906	NA	*Ca_desi_Chr01*	1072906	T/C	Gene_Ca_00097.1	NON_SYNONYMOUS_CODING	Putative PAP-specific phosphatase, mitochondrial
Desi_wild_1072930	NA	*Ca_desi_Chr01*	1072930	G/A	Gene_Ca_00097.1	NON_SYNONYMOUS_CODING	Putative PAP-specific phosphatase, mitochondrial
Desi_wild_1072955	NA	*Ca_desi_Chr01*	1072955	A/G	Gene_Ca_00097.1	SYNONYMOUS_CODING	Putative PAP-specific phosphatase, mitochondrial
Desi_wild_1072973	NA	*Ca_desi_Chr01*	1072973	T/C	Gene_Ca_00097.1	INTRON	Putative PAP-specific phosphatase, mitochondrial
Desi_wild_1072976	NA	*Ca_desi_Chr01*	1072976	G/A	Gene_Ca_00097.1	INTRON	Putative PAP-specific phosphatase, mitochondrial
Desi_wild_1073000	NA	*Ca_desi_Chr01*	1073000	A/T	Gene_Ca_00097.1	INTRON	Putative PAP-specific phosphatase, mitochondrial
Desi_wild_1073048	NA	*Ca_desi_Chr01*	1073048	G/A	Gene_Ca_00097.1	INTRON	Putative PAP-specific phosphatase, mitochondrial
Desi_wild_1073054	NA	*Ca_desi_Chr01*	1073054	A/G	Gene_Ca_00097.1	INTRON	Putative PAP-specific phosphatase, mitochondrial
Desi_wild_1073059	NA	*Ca_desi_Chr01*	1073059	G/A	Gene_Ca_00097.1	INTRON	Putative PAP-specific phosphatase, mitochondrial
Desi_wild_1105109	NA	*Ca_desi_Chr01*	1105109	C/T	Gene_Ca_00100.1	SYNONYMOUS_CODING	Probable JmjC domain-containing histone demethylation protein 2C
Desi_wild_1126922	NA	*Ca_desi_Chr01*	1126922	G/C	Gene_Ca_00102.1	INTRON	Equilibrative nucleoside transporter 4
Desi_wild_1140358	NA	*Ca_desi_Chr01*	1140358	A/G	NA	INTERGENIC	NA
Desi_wild_1140404	NA	*Ca_desi_Chr01*	1140404	A/G	NA	INTERGENIC	NA
Desi_wild_1140485	NA	*Ca_desi_Chr01*	1140485	A/T	NA	INTERGENIC	NA
Desi_wild_1145410	NA	*Ca_desi_Chr01*	1145410	A/G	NA	INTERGENIC	NA
Desi_wild_1145493	NA	*Ca_desi_Chr01*	1145493	G/T	NA	INTERGENIC	NA
Desi_wild_1146027	NA	*Ca_desi_Chr01*	1146027	T/C	NA	INTERGENIC	NA
Desi_wild_1146028	NA	*Ca_desi_Chr01*	1146028	G/T	NA	INTERGENIC	NA
Desi_wild_1146076	NA	*Ca_desi_Chr01*	1146076	A/C	NA	INTERGENIC	NA
Desi_wild_1146138	NA	*Ca_desi_Chr01*	1146138	T/G	NA	INTERGENIC	NA
Desi_wild_1146141	NA	*Ca_desi_Chr01*	1146141	C/T	NA	INTERGENIC	NA
Desi_wild_1146211	NA	*Ca_desi_Chr01*	1146211	C/T	NA	INTERGENIC	NA
Desi_wild_1146466	NA	*Ca_desi_Chr01*	1146466	C/T	NA	INTERGENIC	NA
Desi_wild_1146471	NA	*Ca_desi_Chr01*	1146471	G/A	NA	INTERGENIC	NA
Desi_wild_1146489	NA	*Ca_desi_Chr01*	1146489	T/C	NA	INTERGENIC	NA
Desi_wild_1146490	NA	*Ca_desi_Chr01*	1146490	G/A	NA	INTERGENIC	NA
Desi_wild_1146611	NA	*Ca_desi_Chr01*	1146611	C/G	NA	INTERGENIC	NA
Desi_wild_1146626	NA	*Ca_desi_Chr01*	1146626	C/A	NA	INTERGENIC	NA
Desi_wild_1146659	NA	*Ca_desi_Chr01*	1146659	C/T	NA	INTERGENIC	NA
Desi_wild_1156112	NA	*Ca_desi_Chr01*	1156112	A/G	NA	INTERGENIC	NA
Desi_wild_1189512	NA	*Ca_desi_Chr01*	1189512	T/G	Gene_Ca_00110.1	INTRON	G-type lectin S-receptor-like serine/threonine-protein kinase B120
Desi_wild_1189626	NA	*Ca_desi_Chr01*	1189626	C/G	Gene_Ca_00110.1	INTRON	G-type lectin S-receptor-like serine/threonine-protein kinase B120
Desi_wild_1189627	NA	*Ca_desi_Chr01*	1189627	A/T	Gene_Ca_00110.1	INTRON	G-type lectin S-receptor-like serine/threonine-protein kinase B120
Desi_wild_1189628	NA	*Ca_desi_Chr01*	1189628	A/C	Gene_Ca_00110.1	INTRON	G-type lectin S-receptor-like serine/threonine-protein kinase B120
Desi_wild_1189675	NA	*Ca_desi_Chr01*	1189675	A/G	Gene_Ca_00110.1	INTRON	G-type lectin S-receptor-like serine/threonine-protein kinase B120
Desi_wild_1189681	NA	*Ca_desi_Chr01*	1189681	G/C	Gene_Ca_00110.1	INTRON	G-type lectin S-receptor-like serine/threonine-protein kinase B120
Desi_wild_1271491	NA	*Ca_desi_Chr01*	1271491	C/T	Gene_Ca_00124.1	NON_SYNONYMOUS_CODING	NA
Desi_wild_1271498	NA	*Ca_desi_Chr01*	1271498	A/T	Gene_Ca_00124.1	SYNONYMOUS_CODING	NA
Desi_wild_1313526	NA	*Ca_desi_Chr01*	1313526	C/T	NA	INTERGENIC	NA
Desi_wild_1368713	NA	*Ca_desi_Chr01*	1368713	C/T	NA	INTERGENIC	NA
Desi_wild_1375921	NA	*Ca_desi_Chr01*	1375921	C/G	Gene_Ca_00138.1	SYNONYMOUS_CODING	unknown protein
Desi_wild_1389951	NA	*Ca_desi_Chr01*	1389951	C/T	Gene_Ca_00140.2	INTRON	NA
Desi_wild_1445481	NA	*Ca_desi_Chr01*	1445481	T/A	Gene_Ca_00144.1	DOWNSTREAM	unknown protein

SNP IDs	NCBI dbSNP accession IDs (NCBI_SS#)	Chromosomes	Physical positions (bp)	SNPs	Gene accession IDs	Sequence components of genome	Putative functions
Desi_wild_1445548	NA	*Ca_desi_Chr01*	1445548	T/C	Gene_Ca_00144.1	DOWNSTREAM	unknown protein
Desi_wild_1451002	NA	*Ca_desi_Chr01*	1451002	A/G	Gene_Ca_00145.1	DOWNSTREAM	NA
Desi_wild_1451028	NA	*Ca_desi_Chr01*	1451028	A/T	Gene_Ca_00145.1	DOWNSTREAM	NA
Desi_wild_1463203	NA	*Ca_desi_Chr01*	1463203	T/C	NA	INTERGENIC	NA
Desi_wild_1463234	NA	*Ca_desi_Chr01*	1463234	C/T	NA	INTERGENIC	NA
Desi_wild_1463270	NA	*Ca_desi_Chr01*	1463270	A/C	Gene_Ca_00146.1	NON_SYNONYMOUS_CODING	Octicosapeptide/Phox/Bem1p family protein
Desi_wild_1463335	NA	*Ca_desi_Chr01*	1463335	C/T	Gene_Ca_00146.1	SYNONYMOUS_CODING	Octicosapeptide/Phox/Bem1p family protein
Desi_wild_1479425	NA	*Ca_desi_Chr01*	1479425	T/C	Gene_Ca_00149.5	DOWNSTREAM	NA
Desi_wild_1479437	NA	*Ca_desi_Chr01*	1479437	T/C	Gene_Ca_00149.5	DOWNSTREAM	NA
Desi_wild_1480187	NA	*Ca_desi_Chr01*	1480187	T/C	Gene_Ca_00149.5	DOWNSTREAM	NA
Desi_wild_1486057	NA	*Ca_desi_Chr01*	1486057	A/C	Gene_Ca_00148.1	SYNONYMOUS_CODING	DNA polymerase V
Desi_wild_1486086	NA	*Ca_desi_Chr01*	1486086	G/C	Gene_Ca_00148.1	NON_SYNONYMOUS_CODING	DNA polymerase V
Desi_wild_1522221	NA	*Ca_desi_Chr01*	1522221	T/C	NA	INTERGENIC	NA
Desi_wild_1526741	NA	*Ca_desi_Chr01*	1526741	A/C	NA	INTERGENIC	NA
Desi_wild_1526742	NA	*Ca_desi_Chr01*	1526742	G/T	NA	INTERGENIC	NA
Desi_wild_1553958	NA	*Ca_desi_Chr01*	1553958	A/G	Gene_Ca_00154.1	INTRON	Probable peptide/nitrate transporter At1g59740
Desi_wild_1639010	NA	*Ca_desi_Chr01*	1639010	T/A	Gene_Ca_00163.1	INTRON	Probable serine/threonine-protein kinase At1g54610
Desi_wild_1639034	NA	*Ca_desi_Chr01*	1639034	C/T	Gene_Ca_00163.1	INTRON	Probable serine/threonine-protein kinase At1g54610
Desi_wild_1639039	NA	*Ca_desi_Chr01*	1639039	T/G	Gene_Ca_00163.1	INTRON	Probable serine/threonine-protein kinase At1g54610
Desi_wild_1639044	NA	*Ca_desi_Chr01*	1639044	T/C	Gene_Ca_00163.1	INTRON	Probable serine/threonine-protein kinase At1g54610
Desi_wild_1639052	NA	*Ca_desi_Chr01*	1639052	G/T	Gene_Ca_00163.1	INTRON	Probable serine/threonine-protein kinase At1g54610
Desi_wild_1660822	NA	*Ca_desi_Chr01*	1660822	T/C	NA	INTERGENIC	NA
Desi_wild_1727845	NA	*Ca_desi_Chr01*	1727845	T/A	NA	INTERGENIC	NA
Desi_wild_1785109	NA	*Ca_desi_Chr01*	1785109	G/C	NA	INTERGENIC	NA
Desi_wild_1785130	NA	*Ca_desi_Chr01*	1785130	T/A	NA	INTERGENIC	NA
Desi_wild_1785134	NA	*Ca_desi_Chr01*	1785134	C/A	NA	INTERGENIC	NA
Desi_wild_1785171	NA	*Ca_desi_Chr01*	1785171	C/T	NA	INTERGENIC	NA
Desi_wild_1815665	NA	*Ca_desi_Chr01*	1815665	T/C	Gene_Ca_00187.1	DOWNSTREAM	(3R)-hydroxymyristoyl-[acyl-carrier-protein] dehydratase
Desi_wild_1828118	NA	*Ca_desi_Chr01*	1828118	A/G	Gene_Ca_00189.1	DOWNSTREAM	Probable inactive receptor kinase At2g26730
Desi_wild_1828281	NA	*Ca_desi_Chr01*	1828281	A/C	NA	INTERGENIC	NA
Desi_wild_1838149	NA	*Ca_desi_Chr01*	1838149	C/G	Gene_Ca_00191.1	NON_SYNONYMOUS_CODING	Putative pumilio homolog 7, chloroplastic
Desi_wild_1842557	NA	*Ca_desi_Chr01*	1842557	C/T	Gene_Ca_00192.1	SYNONYMOUS_CODING	CD2 antigen cytoplasmic tail-binding protein 2
Desi_wild_1851537	NA	*Ca_desi_Chr01*	1851537	A/C	NA	INTERGENIC	NA
Desi_wild_1851564	NA	*Ca_desi_Chr01*	1851564	G/A	NA	INTERGENIC	NA
Desi_wild_1851644	NA	*Ca_desi_Chr01*	1851644	C/A	NA	INTERGENIC	NA
Desi_wild_1851780	NA	*Ca_desi_Chr01*	1851780	A/T	NA	INTERGENIC	NA
Desi_wild_1851944	NA	*Ca_desi_Chr01*	1851944	T/A	NA	INTERGENIC	NA
Desi_wild_1864433	NA	*Ca_desi_Chr01*	1864433	G/C	NA	INTERGENIC	NA
Desi_wild_1864457	NA	*Ca_desi_Chr01*	1864457	C/G	NA	INTERGENIC	NA
Desi_wild_1881482	NA	*Ca_desi_Chr01*	1881482	A/G	Gene_Ca_00195.1	NON_SYNONYMOUS_CODING	Calmodulin-binding transcription activator 2
Desi_wild_1881584	NA	*Ca_desi_Chr01*	1881584	A/G	Gene_Ca_00195.1	NON_SYNONYMOUS_CODING	Calmodulin-binding transcription activator 2
Desi_wild_1904458	NA	*Ca_desi_Chr01*	1904458	G/A	Gene_Ca_00199.1	INTRON	Metacaspase-1
Desi_wild_1904473	NA	*Ca_desi_Chr01*	1904473	C/T	Gene_Ca_00199.1	INTRON	Metacaspase-1
Desi_wild_1906298	NA	*Ca_desi_Chr01*	1906298	A/C	NA	INTERGENIC	NA

SNP IDs	NCBI dbSNP accession IDs (NCBI_SS#)	Chromosomes	Physical positions (bp)	SNPs	Gene accession IDs	Sequence components of genome	Putative functions
Desi_wild_1906300	NA	*Ca_desi_Chr01*	1906300	T/G	NA	INTERGENIC	NA
Desi_wild_1906308	NA	*Ca_desi_Chr01*	1906308	A/G	NA	INTERGENIC	NA
Desi_wild_1906324	NA	*Ca_desi_Chr01*	1906324	A/C	NA	INTERGENIC	NA
Desi_wild_1906333	NA	*Ca_desi_Chr01*	1906333	A/C	NA	INTERGENIC	NA
Desi_wild_1906363	NA	*Ca_desi_Chr01*	1906363	G/A	NA	INTERGENIC	NA
Desi_wild_1912758	NA	*Ca_desi_Chr01*	1912758	A/C	NA	INTERGENIC	NA
Desi_wild_1912820	NA	*Ca_desi_Chr01*	1912820	C/T	NA	INTERGENIC	NA
Desi_wild_1912850	NA	*Ca_desi_Chr01*	1912850	A/C	NA	INTERGENIC	NA
Desi_wild_1912864	NA	*Ca_desi_Chr01*	1912864	T/A	NA	INTERGENIC	NA
Desi_wild_1920014	NA	*Ca_desi_Chr01*	1920014	C/G	NA	INTERGENIC	NA
Desi_wild_1920100	NA	*Ca_desi_Chr01*	1920100	A/G	NA	INTERGENIC	NA
Desi_wild_1936976	NA	*Ca_desi_Chr01*	1936976	C/T	NA	INTERGENIC	NA
Desi_wild_1937351	NA	*Ca_desi_Chr01*	1937351	C/T	NA	INTERGENIC	NA
Desi_wild_1960896	NA	*Ca_desi_Chr01*	1960896	A/C	NA	INTERGENIC	NA
Desi_wild_1960959	NA	*Ca_desi_Chr01*	1960959	A/G	NA	INTERGENIC	NA
Desi_wild_1960988	NA	*Ca_desi_Chr01*	1960988	A/T	NA	INTERGENIC	NA
Desi_wild_1971575	NA	*Ca_desi_Chr01*	1971575	C/T	NA	INTERGENIC	NA
Desi_wild_1982239	NA	*Ca_desi_Chr01*	1982239	C/A	Gene_Ca_00209.1	NON_SYNONYMOUS_CODING	NA
Desi_wild_2021754	NA	*Ca_desi_Chr01*	2021754	T/A	Gene_Ca_00213.1	INTRON	Chlorophyll a-b binding protein 13, chloroplastic
Desi_wild_2021813	NA	*Ca_desi_Chr01*	2021813	A/G	Gene_Ca_00213.1	INTRON	Chlorophyll a-b binding protein 13, chloroplastic
Desi_wild_2036430	NA	*Ca_desi_Chr01*	2036430	A/G	Gene_Ca_00214.1	NON_SYNONYMOUS_CODING	Protein E6
Desi_wild_2036583	NA	*Ca_desi_Chr01*	2036583	A/G	Gene_Ca_00214.1	NON_SYNONYMOUS_CODING	Protein E6
Desi_wild_2047526	NA	*Ca_desi_Chr01*	2047526	C/T	Gene_Ca_00215.1	DOWNSTREAM	Putative uncharacterized protein
Desi_wild_2067290	NA	*Ca_desi_Chr01*	2067290	C/A	Gene_Ca_00218.1	SYNONYMOUS_CODING	Ethylene-responsive transcription factor 4
Desi_wild_2084396	NA	*Ca_desi_Chr01*	2084396	T/G	Gene_Ca_00219.1	SYNONYMOUS_CODING	Ethylene-responsive transcription factor 12
Desi_wild_2084474	NA	*Ca_desi_Chr01*	2084474	C/A	Gene_Ca_00219.1	NON_SYNONYMOUS_CODING	Ethylene-responsive transcription factor 12
Desi_wild_2084506	NA	*Ca_desi_Chr01*	2084506	G/T	Gene_Ca_00219.1	SYNONYMOUS_CODING	Ethylene-responsive transcription factor 12
Desi_wild_2125865	NA	*Ca_desi_Chr01*	2125865	T/C	Gene_Ca_00223.1	INTRON	Glyoxysomal processing protease, glyoxysomal
Desi_wild_2125885	NA	*Ca_desi_Chr01*	2125885	C/A	Gene_Ca_00223.1	INTRON	Glyoxysomal processing protease, glyoxysomal
Desi_wild_2125886	NA	*Ca_desi_Chr01*	2125886	G/C	Gene_Ca_00223.1	INTRON	Glyoxysomal processing protease, glyoxysomal
Desi_wild_2185003	NA	*Ca_desi_Chr01*	2185003	C/T	NA	INTERGENIC	NA
Desi_wild_2213694	NA	*Ca_desi_Chr01*	2213694	C/T	Gene_Ca_00229.1	SYNONYMOUS_CODING	NA
Desi_wild_2213700	NA	*Ca_desi_Chr01*	2213700	C/T	Gene_Ca_00229.1	SYNONYMOUS_CODING	NA
Desi_wild_2213762	NA	*Ca_desi_Chr01*	2213762	T/C	Gene_Ca_00229.1	NON_SYNONYMOUS_CODING	NA
Desi_wild_2213817	NA	*Ca_desi_Chr01*	2213817	A/G	Gene_Ca_00229.1	SYNONYMOUS_CODING	NA
Desi_wild_2214019	NA	*Ca_desi_Chr01*	2214019	A/G	Gene_Ca_00229.1	NON_SYNONYMOUS_CODING	NA
Desi_wild_2214038	NA	*Ca_desi_Chr01*	2214038	A/T	Gene_Ca_00229.1	NON_SYNONYMOUS_CODING	NA
Desi_wild_2214171	NA	*Ca_desi_Chr01*	2214171	A/G	Gene_Ca_00229.1	SYNONYMOUS_CODING	NA
Desi_wild_2249232	NA	*Ca_desi_Chr01*	2249232	A/G	NA	INTERGENIC	NA
Desi_wild_2263530	NA	*Ca_desi_Chr01*	2263530	G/A	Gene_Ca_00233.2	INTRON	Serine/threonine-protein kinase HT1
Desi_wild_2263604	NA	*Ca_desi_Chr01*	2263604	C/A	Gene_Ca_00233.2	INTRON	Serine/threonine-protein kinase HT1
Desi_wild_2281552	NA	*Ca_desi_Chr01*	2281552	T/C	Gene_Ca_00235.1	UPSTREAM	NA
Desi_wild_2281554	NA	*Ca_desi_Chr01*	2281554	C/T	Gene_Ca_00235.1	UPSTREAM	NA
Desi_wild_2292933	NA	*Ca_desi_Chr01*	2292933	T/C	Gene_Ca_00236.1	UPSTREAM	NA

SNP IDs	NCBI dbSNP accession IDs (NCBI_SS#)	Chromosomes	Physical positions (bp)	SNPs	Gene accession IDs	Sequence components of genome	Putative functions
Desi_wild_2292968	NA	*Ca_desi_Chr01*	2292968	A/T	Gene_Ca_00236.1	UPSTREAM	NA
Desi_wild_2322455	NA	*Ca_desi_Chr01*	2322455	A/C	Gene_Ca_00238.5	NON_SYNONYMOUS_CODING	NA
Desi_wild_2345481	NA	*Ca_desi_Chr01*	2345481	A/T	Gene_Ca_00242.1	DOWNSTREAM	Pentatricopeptide repeat-containing protein At2g29760, chloroplastic
Desi_wild_2349214	NA	*Ca_desi_Chr01*	2349214	C/A	NA	INTERGENIC	NA
Desi_wild_2349258	NA	*Ca_desi_Chr01*	2349258	G/A	NA	INTERGENIC	NA
Desi_wild_2349306	NA	*Ca_desi_Chr01*	2349306	C/T	NA	INTERGENIC	NA
Desi_wild_2349688	NA	*Ca_desi_Chr01*	2349688	C/A	NA	INTERGENIC	NA
Desi_wild_2349888	NA	*Ca_desi_Chr01*	2349888	A/G	NA	INTERGENIC	NA
Desi_wild_2356768	NA	*Ca_desi_Chr01*	2356768	A/G	Gene_Ca_00245.1	DOWNSTREAM	Putative uncharacterized protein
Desi_wild_2356772	NA	*Ca_desi_Chr01*	2356772	A/T	Gene_Ca_00245.1	DOWNSTREAM	Putative uncharacterized protein
Desi_wild_2417658	NA	*Ca_desi_Chr01*	2417658	A/G	Gene_Ca_00256.1	UPSTREAM	Pentatricopeptide repeat-containing protein At2g32230, mitochondrial
Desi_wild_2417675	NA	*Ca_desi_Chr01*	2417675	G/A	Gene_Ca_00256.1	UPSTREAM	Pentatricopeptide repeat-containing protein At2g32230, mitochondrial
Desi_wild_2417711	NA	*Ca_desi_Chr01*	2417711	C/T	Gene_Ca_00256.1	SYNONYMOUS_CODING	Pentatricopeptide repeat-containing protein At2g32230, mitochondrial
Desi_wild_2418095	NA	*Ca_desi_Chr01*	2418095	C/T	Gene_Ca_00256.1	SYNONYMOUS_CODING	Pentatricopeptide repeat-containing protein At2g32230, mitochondrial
Desi_wild_2418149	NA	*Ca_desi_Chr01*	2418149	C/T	Gene_Ca_00256.1	SYNONYMOUS_CODING	Pentatricopeptide repeat-containing protein At2g32230, mitochondrial
Desi_wild_2418212	NA	*Ca_desi_Chr01*	2418212	T/A	Gene_Ca_00256.1	SYNONYMOUS_CODING	Pentatricopeptide repeat-containing protein At2g32230, mitochondrial
Desi_wild_2418647	NA	*Ca_desi_Chr01*	2418647	A/T	Gene_Ca_00256.1	INTRON	Pentatricopeptide repeat-containing protein At2g32230, mitochondrial
Desi_wild_2418752	NA	*Ca_desi_Chr01*	2418752	A/T	Gene_Ca_00256.1	INTRON	Pentatricopeptide repeat-containing protein At2g32230, mitochondrial
Desi_wild_2418782	NA	*Ca_desi_Chr01*	2418782	A/C	Gene_Ca_00256.1	INTRON	Pentatricopeptide repeat-containing protein At2g32230, mitochondrial
Desi_wild_2435977	NA	*Ca_desi_Chr01*	2435977	G/T	Gene_Ca_00257.1	INTRON	sensitive to freezing 6
Desi_wild_2436035	NA	*Ca_desi_Chr01*	2436035	T/C	Gene_Ca_00257.1	INTRON	sensitive to freezing 6
Desi_wild_2438719	NA	*Ca_desi_Chr01*	2438719	T/A	Gene_Ca_00257.1	NON_SYNONYMOUS_CODING	sensitive to freezing 6
Desi_wild_2440248	NA	*Ca_desi_Chr01*	2440248	C/T	Gene_Ca_00257.1	DOWNSTREAM	sensitive to freezing 6
Desi_wild_2448997	NA	*Ca_desi_Chr01*	2448997	C/T	Gene_Ca_00259.1	INTRON	Splicing factor U2af large subunit A
Desi_wild_2456844	NA	*Ca_desi_Chr01*	2456844	T/G	NA	INTERGENIC	NA
Desi_wild_2457014	NA	*Ca_desi_Chr01*	2457014	A/C	NA	INTERGENIC	NA
Desi_wild_2457030	NA	*Ca_desi_Chr01*	2457030	A/G	NA	INTERGENIC	NA
Desi_wild_2471753	NA	*Ca_desi_Chr01*	2471753	G/A	Gene_Ca_00262.1	INTRON	RING finger protein 141
Desi_wild_2471793	NA	*Ca_desi_Chr01*	2471793	C/T	Gene_Ca_00262.1	INTRON	RING finger protein 141
Desi_wild_2471889	NA	*Ca_desi_Chr01*	2471889	T/C	Gene_Ca_00262.1	INTRON	RING finger protein 141
Desi_wild_2471923	NA	*Ca_desi_Chr01*	2471923	C/A	Gene_Ca_00262.1	INTRON	RING finger protein 141
Desi_wild_2471996	NA	*Ca_desi_Chr01*	2471996	G/A	Gene_Ca_00262.1	INTRON	RING finger protein 141
Desi_wild_2491479	NA	*Ca_desi_Chr01*	2491479	T/C	Gene_Ca_00264.1	INTRON	Probable allantoinase 1
Desi_wild_2512165	NA	*Ca_desi_Chr01*	2512165	T/C	Gene_Ca_00267.1	NON_SYNONYMOUS_CODING	NA
Desi_wild_2541722	NA	*Ca_desi_Chr01*	2541722	T/C	Gene_Ca_00268.2	INTRON	NA
Desi_wild_2553120	NA	*Ca_desi_Chr01*	2553120	A/C	Gene_Ca_00270.1	DOWNSTREAM	Putative uncharacterized protein
Desi_wild_2553150	NA	*Ca_desi_Chr01*	2553150	C/T	Gene_Ca_00270.1	DOWNSTREAM	Putative uncharacterized protein
Desi_wild_2553187	NA	*Ca_desi_Chr01*	2553187	A/G	Gene_Ca_00270.1	DOWNSTREAM	Putative uncharacterized protein
Desi_wild_2554326	NA	*Ca_desi_Chr01*	2554326	T/G	Gene_Ca_00270.1	DOWNSTREAM	Putative uncharacterized protein
Desi_wild_2555171	NA	*Ca_desi_Chr01*	2555171	C/G	Gene_Ca_00269.1	DOWNSTREAM	UPF0559 protein
Desi_wild_2573180	NA	*Ca_desi_Chr01*	2573180	A/C	NA	INTERGENIC	NA
Desi_wild_2593870	NA	*Ca_desi_Chr01*	2593870	C/T	Gene_Ca_00273.1	INTRON	Protein STRUBBELIG-RECEPTOR FAMILY 1
Desi_wild_2637577	NA	*Ca_desi_Chr01*	2637577	A/G	Gene_Ca_00275.1	INTRON	Protein of unknown function (DUF707)
Desi_wild_2637591	NA	*Ca_desi_Chr01*	2637591	T/A	Gene_Ca_00275.1	INTRON	Protein of unknown function (DUF707)

SNP IDs	NCBI dbSNP accession IDs (NCBI_SS#)	Chromosomes	Physical positions (bp)	SNPs	Gene accession IDs	Sequence components of genome	Putative functions
Desi_wild_2637647	NA	*Ca_desi_Chr01*	2637647	C/G	Gene_Ca_00275.1	INTRON	Protein of unknown function (DUF707)
Desi_wild_2637849	NA	*Ca_desi_Chr01*	2637849	C/T	Gene_Ca_00275.1	INTRON	Protein of unknown function (DUF707)
Desi_wild_2637890	NA	*Ca_desi_Chr01*	2637890	G/C	Gene_Ca_00275.1	INTRON	Protein of unknown function (DUF707)
Desi_wild_2856666	NA	*Ca_desi_Chr01*	2856666	T/C	NA	INTERGENIC	NA
Desi_wild_2856743	NA	*Ca_desi_Chr01*	2856743	G/A	NA	INTERGENIC	NA
Desi_wild_2887261	NA	*Ca_desi_Chr01*	2887261	C/G	Gene_Ca_00288.2	INTRON	NA
Desi_wild_3083079	NA	*Ca_desi_Chr01*	3083079	C/T	NA	INTERGENIC	NA
Desi_wild_3083080	NA	*Ca_desi_Chr01*	3083080	G/T	NA	INTERGENIC	NA
Desi_wild_3099404	NA	*Ca_desi_Chr01*	3099404	G/C	Gene_Ca_00314.1	DOWNSTREAM	Nucleobase-ascorbate transporter 2
Desi_wild_3101622	NA	*Ca_desi_Chr01*	3101622	G/C	NA	INTERGENIC	NA
Desi_wild_3109883	NA	*Ca_desi_Chr01*	3109883	A/C	Gene_Ca_00315.1	NON_SYNONYMOUS_CODING	Probable LRR receptor-like serine/threonine-protein kinase At2g24230
Desi_wild_3151834	NA	*Ca_desi_Chr01*	3151834	G/C	Gene_Ca_00318.1	UPSTREAM	U4/U6.U5 tri-snRNP-associated protein 2
Desi_wild_3193712	NA	*Ca_desi_Chr01*	3193712	C/T	Gene_Ca_00323.1	SYNONYMOUS_CODING	Oryzain alpha chain
Desi_wild_3328510	NA	*Ca_desi_Chr01*	3328510	C/A	Gene_Ca_00339.1	NON_SYNONYMOUS_CODING	U-box domain-containing protein 17
Desi_wild_3368165	NA	*Ca_desi_Chr01*	3368165	A/C	NA	INTERGENIC	NA
Desi_wild_3440875	NA	*Ca_desi_Chr01*	3440875	C/A	NA	INTERGENIC	NA
Desi_wild_3545305	NA	*Ca_desi_Chr01*	3545305	T/G	NA	INTERGENIC	NA
Desi_wild_3545332	NA	*Ca_desi_Chr01*	3545332	T/G	NA	INTERGENIC	NA
Desi_wild_3585538	NA	*Ca_desi_Chr01*	3585538	T/C	NA	INTERGENIC	NA
Desi_wild_3606890	NA	*Ca_desi_Chr01*	3606890	T/C	NA	INTERGENIC	NA
Desi_wild_3607045	NA	*Ca_desi_Chr01*	3607045	C/G	NA	INTERGENIC	NA
Desi_wild_3628602	NA	*Ca_desi_Chr01*	3628602	G/T	NA	INTERGENIC	NA
Desi_wild_3628719	NA	*Ca_desi_Chr01*	3628719	G/A	NA	INTERGENIC	NA
Desi_wild_3674413	NA	*Ca_desi_Chr01*	3674413	A/C	Gene_Ca_00372.1	INTRON	NA
Desi_wild_3674854	NA	*Ca_desi_Chr01*	3674854	A/C	Gene_Ca_00372.1	INTRON	NA
Desi_wild_3903712	NA	*Ca_desi_Chr01*	3903712	T/A	NA	INTERGENIC	NA
Desi_wild_3903779	NA	*Ca_desi_Chr01*	3903779	T/C	NA	INTERGENIC	NA
Desi_wild_3903801	NA	*Ca_desi_Chr01*	3903801	G/A	NA	INTERGENIC	NA
Desi_wild_3903835	NA	*Ca_desi_Chr01*	3903835	G/A	NA	INTERGENIC	NA
Desi_wild_3911580	NA	*Ca_desi_Chr01*	3911580	A/G	Gene_Ca_00394.1	NON_SYNONYMOUS_CODING	Prolyl 4-hydroxylase subunit alpha-2
Desi_wild_3953451	NA	*Ca_desi_Chr01*	3953451	C/T	NA	INTERGENIC	NA
Desi_wild_3963327	NA	*Ca_desi_Chr01*	3963327	T/C	Gene_Ca_00399.1	INTRON	Squamosa promoter-binding-like protein 7
Desi_wild_3994131	NA	*Ca_desi_Chr01*	3994131	G/A	Gene_Ca_00401.2	SYNONYMOUS_CODING	Probable 1-deoxy-D-xylulose-5-phosphate synthase, chloroplastic
Desi_wild_3995579	NA	*Ca_desi_Chr01*	3995579	C/T	Gene_Ca_00401.2	SYNONYMOUS_CODING	Probable 1-deoxy-D-xylulose-5-phosphate synthase, chloroplastic
Desi_wild_3996356	NA	*Ca_desi_Chr01*	3996356	C/T	Gene_Ca_00401.1	SYNONYMOUS_CODING	Probable 1-deoxy-D-xylulose-5-phosphate synthase, chloroplastic
Desi_wild_3996916	NA	*Ca_desi_Chr01*	3996916	C/T	Gene_Ca_00401.1	SYNONYMOUS_CODING	Probable 1-deoxy-D-xylulose-5-phosphate synthase, chloroplastic
Desi_wild_5004852	NA	*Ca_desi_Chr01*	5004852	G/A	Gene_Ca_00414.2	INTRON	Transcriptional adapter ADA2b
Desi_wild_5004855	NA	*Ca_desi_Chr01*	5004855	A/C	Gene_Ca_00414.2	INTRON	Transcriptional adapter ADA2b
Desi_wild_5004857	NA	*Ca_desi_Chr01*	5004857	G/C	Gene_Ca_00414.2	INTRON	Transcriptional adapter ADA2b
Desi_wild_5004917	NA	*Ca_desi_Chr01*	5004917	C/A	Gene_Ca_00414.2	INTRON	Transcriptional adapter ADA2b
Desi_wild_5022262	NA	*Ca_desi_Chr01*	5022262	G/T	Gene_Ca_00416.1	INTRON	CASP-like protein POPTRDRAFT_818956
Desi_wild_5022390	NA	*Ca_desi_Chr01*	5022390	C/T	Gene_Ca_00416.1	INTRON	CASP-like protein POPTRDRAFT_818956
Desi_wild_5039591	NA	*Ca_desi_Chr01*	5039591	A/G	Gene_Ca_00419.1	UPSTREAM	unknown protein
Desi_wild_5039614	NA	*Ca_desi_Chr01*	5039614	C/A	Gene_Ca_00419.1	UPSTREAM	unknown protein

SNP IDs	NCBI dbSNP accession IDs (NCBI_SS#)	Chromosomes	Physical positions (bp)	SNPs	Gene accession IDs	Sequence components of genome	Putative functions
Desi_wild_5040610	NA	*Ca_desi_Chr01*	5040610	A/G	NA	INTERGENIC	NA
Desi_wild_5040663	NA	*Ca_desi_Chr01*	5040663	A/G	NA	INTERGENIC	NA
Desi_wild_5050027	NA	*Ca_desi_Chr01*	5050027	G/A	NA	INTERGENIC	NA
Desi_wild_5050172	NA	*Ca_desi_Chr01*	5050172	C/T	NA	INTERGENIC	NA
Desi_wild_5056212	NA	*Ca_desi_Chr01*	5056212	T/C	NA	INTERGENIC	NA
Desi_wild_5056260	NA	*Ca_desi_Chr01*	5056260	A/C	NA	INTERGENIC	NA
Desi_wild_5056328	NA	*Ca_desi_Chr01*	5056328	T/C	NA	INTERGENIC	NA
Desi_wild_5061066	NA	*Ca_desi_Chr01*	5061066	T/A	NA	INTERGENIC	NA
Desi_wild_5061125	NA	*Ca_desi_Chr01*	5061125	G/C	NA	INTERGENIC	NA
Desi_wild_5129092	NA	*Ca_desi_Chr01*	5129092	C/G	Gene_Ca_00427.1	INTRON	GLABRA2 expression modulator
Desi_wild_5161857	NA	*Ca_desi_Chr01*	5161857	C/T	Gene_Ca_00430.1	NON_SYNONYMOUS_CODING	Ethylene-responsive transcription factor ESR2
Desi_wild_5197254	NA	*Ca_desi_Chr01*	5197254	A/G	Gene_Ca_00431.1	UPSTREAM	NA
Desi_wild_5197334	NA	*Ca_desi_Chr01*	5197334	C/G	Gene_Ca_00431.1	UPSTREAM	NA
Desi_wild_5197370	NA	*Ca_desi_Chr01*	5197370	G/A	Gene_Ca_00431.1	UPSTREAM	NA
Desi_wild_5197445	NA	*Ca_desi_Chr01*	5197445	C/A	Gene_Ca_00431.1	UPSTREAM	NA
Desi_wild_5202057	NA	*Ca_desi_Chr01*	5202057	A/T	Gene_Ca_00432.1	INTRON	LETM1-like protein
Desi_wild_5202066	NA	*Ca_desi_Chr01*	5202066	T/C	Gene_Ca_00432.1	INTRON	LETM1-like protein
Desi_wild_5218664	NA	*Ca_desi_Chr01*	5218664	T/C	Gene_Ca_00434.1	DOWNSTREAM	Cold-inducible RNA-binding protein
Desi_wild_5240171	NA	*Ca_desi_Chr01*	5240171	G/A	Gene_Ca_00438.1	INTRON	Vacuolar protein sorting-associated protein 55 homolog
Desi_wild_5279501	NA	*Ca_desi_Chr01*	5279501	A/T	Gene_Ca_00442.1	NON_SYNONYMOUS_CODING	1-aminocyclopropane-1-carboxylate oxidase homolog 12
Desi_wild_5292810	NA	*Ca_desi_Chr01*	5292810	A/T	Gene_Ca_00444.1	NON_SYNONYMOUS_CODING	125 kDa kinesin-related protein
Desi_wild_5373612	NA	*Ca_desi_Chr01*	5373612	C/T	Gene_Ca_00455.1	UPSTREAM	Pentatricopeptide repeat-containing protein At2g36240
Desi_wild_5489825	NA	*Ca_desi_Chr01*	5489825	T/C	Gene_Ca_00469.2	SYNONYMOUS_CODING	RNA (guanine-9-)-methyltransferase domain-containing protein 2
Desi_wild_5498868	NA	*Ca_desi_Chr01*	5498868	A/G	Gene_Ca_00470.1	DOWNSTREAM	NA
Desi_wild_5498890	NA	*Ca_desi_Chr01*	5498890	A/G	Gene_Ca_00470.1	DOWNSTREAM	NA
Desi_wild_5506578	NA	*Ca_desi_Chr01*	5506578	G/C	Gene_Ca_00471.1	INTRON	NA
Desi_wild_5511110	NA	*Ca_desi_Chr01*	5511110	A/T	Gene_Ca_00471.1	INTRON	NA
Desi_wild_5511140	NA	*Ca_desi_Chr01*	5511140	A/T	Gene_Ca_00471.1	INTRON	NA
Desi_wild_5511149	NA	*Ca_desi_Chr01*	5511149	T/C	Gene_Ca_00471.1	INTRON	NA
Desi_wild_5511162	NA	*Ca_desi_Chr01*	5511162	T/G	Gene_Ca_00471.1	INTRON	NA
Desi_wild_5537208	NA	*Ca_desi_Chr01*	5537208	C/T	Gene_Ca_00474.1	DOWNSTREAM	SEC14-like protein 4
Desi_wild_5537211	NA	*Ca_desi_Chr01*	5537211	G/A	Gene_Ca_00474.1	DOWNSTREAM	SEC14-like protein 4
Desi_wild_5537394	NA	*Ca_desi_Chr01*	5537394	A/G	Gene_Ca_00474.1	DOWNSTREAM	SEC14-like protein 4
Desi_wild_5537397	NA	*Ca_desi_Chr01*	5537397	T/A	Gene_Ca_00474.1	DOWNSTREAM	SEC14-like protein 4
Desi_wild_5537508	NA	*Ca_desi_Chr01*	5537508	C/T	Gene_Ca_00474.1	DOWNSTREAM	SEC14-like protein 4
Desi_wild_5555967	NA	*Ca_desi_Chr01*	5555967	C/T	Gene_Ca_00475.1	DOWNSTREAM	U-box domain-containing protein 35
Desi_wild_5556081	NA	*Ca_desi_Chr01*	5556081	G/A	Gene_Ca_00475.1	DOWNSTREAM	U-box domain-containing protein 35
Desi_wild_5566993	NA	*Ca_desi_Chr01*	5566993	A/C	Gene_Ca_00477.2	DOWNSTREAM	NA
Desi_wild_5566995	NA	*Ca_desi_Chr01*	5566995	T/C	Gene_Ca_00477.2	DOWNSTREAM	NA
Desi_wild_5567015	NA	*Ca_desi_Chr01*	5567015	T/C	Gene_Ca_00477.2	DOWNSTREAM	NA
Desi_wild_5567027	NA	*Ca_desi_Chr01*	5567027	G/A	Gene_Ca_00477.2	DOWNSTREAM	NA
Desi_wild_5568121	NA	*Ca_desi_Chr01*	5568121	A/C	Gene_Ca_00477.2	INTRON	NA
Desi_wild_5569043	NA	*Ca_desi_Chr01*	5569043	A/C	Gene_Ca_00477.2	INTRON	NA
Desi_wild_5580120	NA	*Ca_desi_Chr01*	5580120	C/T	Gene_Ca_00478.1	SYNONYMOUS_CODING	Farnesyl pyrophosphate synthase 1

SNP IDs	NCBI dbSNP accession IDs (NCBI_SS#)	Chromosomes	Physical positions (bp)	SNPs	Gene accession IDs	Sequence components of genome	Putative functions
Desi_wild_5605911	NA	*Ca_desi_Chr01*	5605911	G/C	NA	INTERGENIC	NA
Desi_wild_5613829	NA	*Ca_desi_Chr01*	5613829	C/A	NA	INTERGENIC	NA
Desi_wild_5644131	NA	*Ca_desi_Chr01*	5644131	G/C	NA	INTERGENIC	NA
Desi_wild_5645015	NA	*Ca_desi_Chr01*	5645015	T/C	NA	INTERGENIC	NA
Desi_wild_5667771	NA	*Ca_desi_Chr01*	5667771	A/G	NA	INTERGENIC	NA
Desi_wild_5676601	NA	*Ca_desi_Chr01*	5676601	T/C	Gene_Ca_00485.1	SYNONYMOUS_CODING	Probable exocyst complex component 4
Desi_wild_5676645	NA	*Ca_desi_Chr01*	5676645	C/A	Gene_Ca_00485.1	NON_SYNONYMOUS_CODING	Probable exocyst complex component 4
Desi_wild_5753424	NA	*Ca_desi_Chr01*	5753424	G/A	Gene_Ca_00495.1	INTRON	Probable leucine-rich repeat receptor-like protein kinase At5g49770
Desi_wild_5753484	NA	*Ca_desi_Chr01*	5753484	T/G	Gene_Ca_00495.1	NON_SYNONYMOUS_CODING	Probable leucine-rich repeat receptor-like protein kinase At5g49770
Desi_wild_5776338	NA	*Ca_desi_Chr01*	5776338	C/A	Gene_Ca_00497.2	NON_SYNONYMOUS_CODING	NA
Desi_wild_5776339	NA	*Ca_desi_Chr01*	5776339	A/G	Gene_Ca_00497.2	SYNONYMOUS_CODING	NA
Desi_wild_5776369	NA	*Ca_desi_Chr01*	5776369	T/C	Gene_Ca_00497.2	SYNONYMOUS_CODING	NA
Desi_wild_5807567	NA	*Ca_desi_Chr01*	5807567	C/G	Gene_Ca_00500.2	INTRON	NA
Desi_wild_5807642	NA	*Ca_desi_Chr01*	5807642	G/A	Gene_Ca_00500.2	INTRON	NA
Desi_wild_5808519	NA	*Ca_desi_Chr01*	5808519	T/G	Gene_Ca_00500.2	INTRON	NA
Desi_wild_5823260	NA	*Ca_desi_Chr01*	5823260	C/T	NA	INTERGENIC	NA
Desi_wild_5853627	NA	*Ca_desi_Chr01*	5853627	T/A	Gene_Ca_00504.1	NON_SYNONYMOUS_CODING	Aspartic proteinase nepenthesin-2
Desi_wild_5854373	NA	*Ca_desi_Chr01*	5854373	C/T	NA	INTERGENIC	NA
Desi_wild_5892893	NA	*Ca_desi_Chr01*	5892893	G/A	NA	INTERGENIC	NA
Desi_wild_6001090	NA	*Ca_desi_Chr01*	6001090	C/G	NA	INTERGENIC	NA
Desi_wild_6037738	NA	*Ca_desi_Chr01*	6037738	T/G	Gene_Ca_00513.1	SYNONYMOUS_CODING	Cleavage and polyadenylation specificity factor CPSF30
Desi_wild_6072205	NA	*Ca_desi_Chr01*	6072205	A/G	NA	INTERGENIC	NA
Desi_wild_6072236	NA	*Ca_desi_Chr01*	6072236	A/G	NA	INTERGENIC	NA
Desi_wild_6080870	NA	*Ca_desi_Chr01*	6080870	T/C	Gene_Ca_00517.1	DOWNSTREAM	Protein CbbY, chromosomal
Desi_wild_6083987	NA	*Ca_desi_Chr01*	6083987	T/G	Gene_Ca_00518.1	SYNONYMOUS_CODING	Protein ROOT HAIR DEFECTIVE 3 homolog 2
Desi_wild_6107673	NA	*Ca_desi_Chr01*	6107673	A/C	Gene_Ca_00519.2	INTRON	Probable exocyst complex component 4
Desi_wild_6107921	NA	*Ca_desi_Chr01*	6107921	G/A	Gene_Ca_00519.2	INTRON	Probable exocyst complex component 4
Desi_wild_6108004	NA	*Ca_desi_Chr01*	6108004	C/A	Gene_Ca_00519.2	INTRON	Probable exocyst complex component 4
Desi_wild_6108008	NA	*Ca_desi_Chr01*	6108008	T/G	Gene_Ca_00519.2	INTRON	Probable exocyst complex component 4
Desi_wild_6108016	NA	*Ca_desi_Chr01*	6108016	C/T	Gene_Ca_00519.2	INTRON	Probable exocyst complex component 4
Desi_wild_6108035	NA	*Ca_desi_Chr01*	6108035	T/A	Gene_Ca_00519.2	INTRON	Probable exocyst complex component 4
Desi_wild_6108094	NA	*Ca_desi_Chr01*	6108094	C/T	Gene_Ca_00519.2	INTRON	Probable exocyst complex component 4
Desi_wild_6108140	NA	*Ca_desi_Chr01*	6108140	A/G	Gene_Ca_00519.2	INTRON	Probable exocyst complex component 4
Desi_wild_6111677	NA	*Ca_desi_Chr01*	6111677	A/G	Gene_Ca_00519.2	SYNONYMOUS_CODING	Probable exocyst complex component 4
Desi_wild_6111732	NA	*Ca_desi_Chr01*	6111732	T/G	Gene_Ca_00519.2	INTRON	Probable exocyst complex component 4
Desi_wild_6112419	NA	*Ca_desi_Chr01*	6112419	T/G	Gene_Ca_00519.2	NON_SYNONYMOUS_CODING	Probable exocyst complex component 4
Desi_wild_6112443	NA	*Ca_desi_Chr01*	6112443	G/A	Gene_Ca_00519.2	SYNONYMOUS_CODING	Probable exocyst complex component 4
Desi_wild_6112451	NA	*Ca_desi_Chr01*	6112451	G/A	Gene_Ca_00519.2	SYNONYMOUS_CODING	Probable exocyst complex component 4
Desi_wild_6112461	NA	*Ca_desi_Chr01*	6112461	G/A	Gene_Ca_00519.2	SYNONYMOUS_CODING	Probable exocyst complex component 4
Desi_wild_6112462	NA	*Ca_desi_Chr01*	6112462	G/A	Gene_Ca_00519.2	SYNONYMOUS_CODING	Probable exocyst complex component 4
Desi_wild_6112469	NA	*Ca_desi_Chr01*	6112469	T/C	Gene_Ca_00519.2	SYNONYMOUS_CODING	Probable exocyst complex component 4
Desi_wild_6113445	NA	*Ca_desi_Chr01*	6113445	T/C	Gene_Ca_00519.2	INTRON	Probable exocyst complex component 4
Desi_wild_6135902	NA	*Ca_desi_Chr01*	6135902	A/G	Gene_Ca_00523.1	INTRON	maternal effect embryo arrest 22
Desi_wild_6135970	NA	*Ca_desi_Chr01*	6135970	A/G	Gene_Ca_00523.1	INTRON	maternal effect embryo arrest 22

SNP IDs	NCBI dbSNP accession IDs (NCBI_SS#)	Chromosomes	Physical positions (bp)	SNPs	Gene accession IDs	Sequence components of genome	Putative functions
Desi_wild_6141126	NA	*Ca_desi_Chr01*	6141126	A/C	Gene_Ca_00523.1	NON_SYNONYMOUS_CODING	maternal effect embryo arrest 22
Desi_wild_6141133	NA	*Ca_desi_Chr01*	6141133	A/C	Gene_Ca_00523.1	NON_SYNONYMOUS_CODING	maternal effect embryo arrest 22
Desi_wild_6149357	NA	*Ca_desi_Chr01*	6149357	C/A	Gene_Ca_00524.1	NON_SYNONYMOUS_CODING	NA
Desi_wild_6262893	NA	*Ca_desi_Chr01*	6262893	T/A	NA	INTERGENIC	NA
Desi_wild_6262953	NA	*Ca_desi_Chr01*	6262953	A/C	NA	INTERGENIC	NA
Desi_wild_6268192	NA	*Ca_desi_Chr01*	6268192	C/A	NA	INTERGENIC	NA
Desi_wild_6268200	NA	*Ca_desi_Chr01*	6268200	C/T	NA	INTERGENIC	NA
Desi_wild_6268215	NA	*Ca_desi_Chr01*	6268215	A/G	NA	INTERGENIC	NA
Desi_wild_6270528	NA	*Ca_desi_Chr01*	6270528	T/C	NA	INTERGENIC	NA
Desi_wild_6270529	NA	*Ca_desi_Chr01*	6270529	C/T	NA	INTERGENIC	NA
Desi_wild_6270568	NA	*Ca_desi_Chr01*	6270568	T/C	NA	INTERGENIC	NA
Desi_wild_6270721	NA	*Ca_desi_Chr01*	6270721	G/C	NA	INTERGENIC	NA
Desi_wild_6303938	NA	*Ca_desi_Chr01*	6303938	A/G	NA	INTERGENIC	NA
Desi_wild_6303965	NA	*Ca_desi_Chr01*	6303965	T/G	NA	INTERGENIC	NA
Desi_wild_6304514	NA	*Ca_desi_Chr01*	6304514	T/G	NA	INTERGENIC	NA
Desi_wild_6304541	NA	*Ca_desi_Chr01*	6304541	A/G	NA	INTERGENIC	NA
Desi_wild_6313759	NA	*Ca_desi_Chr01*	6313759	T/A	Gene_Ca_00540.1	INTRON	Protein KRI1 homolog
Desi_wild_6318808	NA	*Ca_desi_Chr01*	6318808	C/T	NA	INTERGENIC	NA
Desi_wild_6319012	NA	*Ca_desi_Chr01*	6319012	A/G	NA	INTERGENIC	NA
Desi_wild_6319042	NA	*Ca_desi_Chr01*	6319042	A/C	NA	INTERGENIC	NA
Desi_wild_6322174	NA	*Ca_desi_Chr01*	6322174	A/G	Gene_Ca_00541.2	UPSTREAM	Probable LRR receptor-like serine/threonine-protein kinase At4g20940
Desi_wild_6322202	NA	*Ca_desi_Chr01*	6322202	G/T	Gene_Ca_00541.2	UPSTREAM	Probable LRR receptor-like serine/threonine-protein kinase At4g20940
Desi_wild_6333238	NA	*Ca_desi_Chr01*	6333238	C/T	NA	INTERGENIC	NA
Desi_wild_6333247	NA	*Ca_desi_Chr01*	6333247	A/G	NA	INTERGENIC	NA
Desi_wild_6333372	NA	*Ca_desi_Chr01*	6333372	A/C	NA	INTERGENIC	NA
Desi_wild_6333418	NA	*Ca_desi_Chr01*	6333418	G/A	NA	INTERGENIC	NA
Desi_wild_6341138	NA	*Ca_desi_Chr01*	6341138	G/A	Gene_Ca_00544.1	SYNONYMOUS_CODING	WPP domain-associated protein (Fragment)
Desi_wild_6341213	NA	*Ca_desi_Chr01*	6341213	A/C	Gene_Ca_00544.1	NON_SYNONYMOUS_CODING	WPP domain-associated protein (Fragment)
Desi_wild_6341237	NA	*Ca_desi_Chr01*	6341237	C/T	Gene_Ca_00544.1	SYNONYMOUS_CODING	WPP domain-associated protein (Fragment)
Desi_wild_6351975	NA	*Ca_desi_Chr01*	6351975	T/C	Gene_Ca_00545.1	INTRON	NA
Desi_wild_6379945	NA	*Ca_desi_Chr01*	6379945	G/A	Gene_Ca_00549.1	NON_SYNONYMOUS_CODING	Nuclear transcription factor Y subunit A-7
Desi_wild_6399709	NA	*Ca_desi_Chr01*	6399709	T/C	Gene_Ca_00550.2	SYNONYMOUS_CODING	NA
Desi_wild_6399918	NA	*Ca_desi_Chr01*	6399918	C/T	Gene_Ca_00550.1	INTRON	Homeobox-leucine zipper protein ATHB-14
Desi_wild_6747781	NA	*Ca_desi_Chr01*	6747781	C/T	Gene_Ca_00553.1	DOWNSTREAM	unknown protein
Desi_wild_6858906	NA	*Ca_desi_Chr01*	6858906	T/A	Gene_Ca_00561.1	SYNONYMOUS_CODING	Subtilisin-like protease
Desi_wild_6858922	NA	*Ca_desi_Chr01*	6858922	C/T	Gene_Ca_00561.1	SYNONYMOUS_CODING	Subtilisin-like protease
Desi_wild_6858925	NA	*Ca_desi_Chr01*	6858925	C/T	Gene_Ca_00561.1	SYNONYMOUS_CODING	Subtilisin-like protease
Desi_wild_6858952	NA	*Ca_desi_Chr01*	6858952	A/C	Gene_Ca_00561.1	SYNONYMOUS_CODING	Subtilisin-like protease
Desi_wild_6858964	NA	*Ca_desi_Chr01*	6858964	A/C	Gene_Ca_00561.1	SYNONYMOUS_CODING	Subtilisin-like protease
Desi_wild_6859408	NA	*Ca_desi_Chr01*	6859408	C/A	Gene_Ca_00561.1	INTRON	Subtilisin-like protease
Desi_wild_6859622	NA	*Ca_desi_Chr01*	6859622	A/G	Gene_Ca_00561.1	SYNONYMOUS_CODING	Subtilisin-like protease
Desi_wild_6859628	NA	*Ca_desi_Chr01*	6859628	G/A	Gene_Ca_00561.1	SYNONYMOUS_CODING	Subtilisin-like protease
Desi_wild_6859634	NA	*Ca_desi_Chr01*	6859634	T/C	Gene_Ca_00561.1	SYNONYMOUS_CODING	Subtilisin-like protease
Desi_wild_6859661	NA	*Ca_desi_Chr01*	6859661	C/A	Gene_Ca_00561.1	SYNONYMOUS_CODING	Subtilisin-like protease

SNP IDs	NCBI dbSNP accession IDs (NCBI_SS#)	Chromosomes	Physical positions (bp)	SNPs	Gene accession IDs	Sequence components of genome	Putative functions
Desi_wild_6862834	NA	*Ca_desi_Chr01*	6862834	C/A	Gene_Ca_00561.1	NON_SYNONYMOUS_CODING	Subtilisin-like protease
Desi_wild_6862836	NA	*Ca_desi_Chr01*	6862836	T/G	Gene_Ca_00561.1	SYNONYMOUS_CODING	Subtilisin-like protease
Desi_wild_6862866	NA	*Ca_desi_Chr01*	6862866	A/G	Gene_Ca_00561.1	SYNONYMOUS_CODING	Subtilisin-like protease
Desi_wild_6862869	NA	*Ca_desi_Chr01*	6862869	C/T	Gene_Ca_00561.1	SYNONYMOUS_CODING	Subtilisin-like protease
Desi_wild_6862872	NA	*Ca_desi_Chr01*	6862872	A/T	Gene_Ca_00561.1	SYNONYMOUS_CODING	Subtilisin-like protease
Desi_wild_6862873	NA	*Ca_desi_Chr01*	6862873	A/C	Gene_Ca_00561.1	NON_SYNONYMOUS_CODING	Subtilisin-like protease
Desi_wild_6862881	NA	*Ca_desi_Chr01*	6862881	A/C	Gene_Ca_00561.1	SYNONYMOUS_CODING	Subtilisin-like protease
Desi_wild_6862888	NA	*Ca_desi_Chr01*	6862888	T/A	Gene_Ca_00561.1	NON_SYNONYMOUS_CODING	Subtilisin-like protease
Desi_wild_6862889	NA	*Ca_desi_Chr01*	6862889	A/C	Gene_Ca_00561.1	NON_SYNONYMOUS_CODING	Subtilisin-like protease
Desi_wild_6862893	NA	*Ca_desi_Chr01*	6862893	T/C	Gene_Ca_00561.1	SYNONYMOUS_CODING	Subtilisin-like protease
Desi_wild_6918743	NA	*Ca_desi_Chr01*	6918743	T/G	NA	INTERGENIC	NA
Desi_wild_6918766	NA	*Ca_desi_Chr01*	6918766	T/A	NA	INTERGENIC	NA
Desi_wild_6918782	NA	*Ca_desi_Chr01*	6918782	T/G	NA	INTERGENIC	NA
Desi_wild_6918819	NA	*Ca_desi_Chr01*	6918819	T/A	NA	INTERGENIC	NA
Desi_wild_7274246	NA	*Ca_desi_Chr01*	7274246	C/T	Gene_Ca_00579.1	NON_SYNONYMOUS_CODING	unknown protein
Desi_wild_7279097	NA	*Ca_desi_Chr01*	7279097	G/A	Gene_Ca_00579.1	INTRON	unknown protein
Desi_wild_7279106	NA	*Ca_desi_Chr01*	7279106	G/T	Gene_Ca_00579.1	INTRON	unknown protein
Desi_wild_7279115	NA	*Ca_desi_Chr01*	7279115	T/C	Gene_Ca_00579.1	INTRON	unknown protein
Desi_wild_7279116	NA	*Ca_desi_Chr01*	7279116	G/A	Gene_Ca_00579.1	INTRON	unknown protein
Desi_wild_7279147	NA	*Ca_desi_Chr01*	7279147	G/A	Gene_Ca_00579.1	SYNONYMOUS_CODING	unknown protein
Desi_wild_7279148	NA	*Ca_desi_Chr01*	7279148	G/A	Gene_Ca_00579.1	SYNONYMOUS_CODING	unknown protein
Desi_wild_7331987	NA	*Ca_desi_Chr01*	7331987	C/T	NA	INTERGENIC	NA
Desi_wild_7383196	NA	*Ca_desi_Chr01*	7383196	A/C	Gene_Ca_00587.1	NON_SYNONYMOUS_CODING	unknown protein
Desi_wild_7383206	NA	*Ca_desi_Chr01*	7383206	A/G	Gene_Ca_00587.1	NON_SYNONYMOUS_CODING	unknown protein
Desi_wild_7383207	NA	*Ca_desi_Chr01*	7383207	G/A	Gene_Ca_00587.1	NON_SYNONYMOUS_CODING	unknown protein
Desi_wild_7383208	NA	*Ca_desi_Chr01*	7383208	T/A	Gene_Ca_00587.1	NON_SYNONYMOUS_CODING	unknown protein
Desi_wild_7383224	NA	*Ca_desi_Chr01*	7383224	A/G	Gene_Ca_00587.1	NON_SYNONYMOUS_CODING	unknown protein
Desi_wild_7383225	NA	*Ca_desi_Chr01*	7383225	G/A	Gene_Ca_00587.1	NON_SYNONYMOUS_CODING	unknown protein
Desi_wild_7383226	NA	*Ca_desi_Chr01*	7383226	C/A	Gene_Ca_00587.1	NON_SYNONYMOUS_CODING	unknown protein
Desi_wild_7383229	NA	*Ca_desi_Chr01*	7383229	G/A	Gene_Ca_00587.1	SYNONYMOUS_CODING	unknown protein
Desi_wild_7383232	NA	*Ca_desi_Chr01*	7383232	G/A	Gene_Ca_00587.1	SYNONYMOUS_CODING	unknown protein
Desi_wild_7383255	NA	*Ca_desi_Chr01*	7383255	A/G	Gene_Ca_00587.1	NON_SYNONYMOUS_CODING	unknown protein
Desi_wild_7383256	NA	*Ca_desi_Chr01*	7383256	G/A	Gene_Ca_00587.1	SYNONYMOUS_CODING	unknown protein
Desi_wild_7383259	NA	*Ca_desi_Chr01*	7383259	G/A	Gene_Ca_00587.1	SYNONYMOUS_CODING	unknown protein
Desi_wild_7383265	NA	*Ca_desi_Chr01*	7383265	T/G	Gene_Ca_00587.1	SYNONYMOUS_CODING	unknown protein
Desi_wild_7383271	NA	*Ca_desi_Chr01*	7383271	A/G	Gene_Ca_00587.1	SYNONYMOUS_CODING	unknown protein
Desi_wild_7383274	NA	*Ca_desi_Chr01*	7383274	C/T	Gene_Ca_00587.1	SYNONYMOUS_CODING	unknown protein
Desi_wild_7383281	NA	*Ca_desi_Chr01*	7383281	G/A	Gene_Ca_00587.1	NON_SYNONYMOUS_CODING	unknown protein
Desi_wild_7383287	NA	*Ca_desi_Chr01*	7383287	A/G	Gene_Ca_00587.1	NON_SYNONYMOUS_CODING	unknown protein
Desi_wild_7383289	NA	*Ca_desi_Chr01*	7383289	C/T	Gene_Ca_00587.1	SYNONYMOUS_CODING	unknown protein
Desi_wild_7383406	NA	*Ca_desi_Chr01*	7383406	C/T	Gene_Ca_00587.1	SYNONYMOUS_CODING	unknown protein
Desi_wild_7383433	NA	*Ca_desi_Chr01*	7383433	T/A	Gene_Ca_00587.1	SYNONYMOUS_CODING	unknown protein
Desi_wild_7383436	NA	*Ca_desi_Chr01*	7383436	G/A	Gene_Ca_00587.1	SYNONYMOUS_CODING	unknown protein
Desi_wild_7383437	NA	*Ca_desi_Chr01*	7383437	T/C	Gene_Ca_00587.1	SYNONYMOUS_CODING	unknown protein

SNP IDs	NCBI dbSNP accession IDs (NCBI_SS#)	Chromosomes	Physical positions (bp)	SNPs	Gene accession IDs	Sequence components of genome	Putative functions
Desi_wild_7489447	NA	*Ca_desi_Chr01*	7489447	C/A	Gene_Ca_00593.1	SYNONYMOUS_CODING	Eukaryotic initiation factor 4A-15
Desi_wild_7489451	NA	*Ca_desi_Chr01*	7489451	A/T	Gene_Ca_00593.1	SYNONYMOUS_CODING	Eukaryotic initiation factor 4A-15
Desi_wild_7489452	NA	*Ca_desi_Chr01*	7489452	T/G	Gene_Ca_00593.1	SYNONYMOUS_CODING	Eukaryotic initiation factor 4A-15
Desi_wild_7489471	NA	*Ca_desi_Chr01*	7489471	T/A	Gene_Ca_00593.1	SYNONYMOUS_CODING	Eukaryotic initiation factor 4A-15
Desi_wild_7489492	NA	*Ca_desi_Chr01*	7489492	T/A	Gene_Ca_00593.1	SYNONYMOUS_CODING	Eukaryotic initiation factor 4A-15
Desi_wild_7489519	NA	*Ca_desi_Chr01*	7489519	T/A	Gene_Ca_00593.1	SYNONYMOUS_CODING	Eukaryotic initiation factor 4A-15
Desi_wild_7606885	NA	*Ca_desi_Chr01*	7606885	C/G	Gene_Ca_00598.1	SYNONYMOUS_CODING	Protein of unknown function (DUF620)
Desi_wild_7606933	NA	*Ca_desi_Chr01*	7606933	C/T	Gene_Ca_00598.1	SYNONYMOUS_CODING	Protein of unknown function (DUF620)
Desi_wild_7632307	NA	*Ca_desi_Chr01*	7632307	A/G	Gene_Ca_00599.1	INTRON	Myb family transcription factor APL
Desi_wild_7632356	NA	*Ca_desi_Chr01*	7632356	C/T	Gene_Ca_00599.1	INTRON	Myb family transcription factor APL
Desi_wild_7680457	NA	*Ca_desi_Chr01*	7680457	C/T	Gene_Ca_00602.1	UPSTREAM	ALA-interacting subunit 3
Desi_wild_7680543	NA	*Ca_desi_Chr01*	7680543	C/T	Gene_Ca_00602.1	UPSTREAM	ALA-interacting subunit 3
Desi_wild_7712983	NA	*Ca_desi_Chr01*	7712983	T/A	NA	INTERGENIC	NA
Desi_wild_7956977	NA	*Ca_desi_Chr01*	7956977	T/G	Gene_Ca_00610.1	SYNONYMOUS_CODING	AT-rich interactive domain-containing protein 4
Desi_wild_8163297	NA	*Ca_desi_Chr01*	8163297	C/A	Gene_Ca_00616.1	INTRON	Nuclear pore complex protein Nup205
Desi_wild_8173005	NA	*Ca_desi_Chr01*	8173005	T/C	Gene_Ca_00616.1	UPSTREAM	Nuclear pore complex protein Nup205
Desi_wild_8219717	NA	*Ca_desi_Chr01*	8219717	A/G	Gene_Ca_00619.1	NON_SYNONYMOUS_CODING	proton pump interactor 1
Desi_wild_8280837	NA	*Ca_desi_Chr01*	8280837	C/A	Gene_Ca_00622.1	UPSTREAM	Probable serine/threonine-protein kinase At1g54610
Desi_wild_8280838	NA	*Ca_desi_Chr01*	8280838	C/G	Gene_Ca_00622.1	UPSTREAM	Probable serine/threonine-protein kinase At1g54610
Desi_wild_8871945	NA	*Ca_desi_Chr01*	8871945	C/T	NA	INTERGENIC	NA
Desi_wild_8902120	NA	*Ca_desi_Chr01*	8902120	C/A	Gene_Ca_00670.1	INTRON	Uncharacterized basic helix-loop-helix protein At1g06150
Desi_wild_8902125	NA	*Ca_desi_Chr01*	8902125	C/A	Gene_Ca_00670.1	INTRON	Uncharacterized basic helix-loop-helix protein At1g06150
Desi_wild_8902212	NA	*Ca_desi_Chr01*	8902212	A/C	Gene_Ca_00670.1	INTRON	Uncharacterized basic helix-loop-helix protein At1g06150
Desi_wild_8952943	NA	*Ca_desi_Chr01*	8952943	A/G	NA	INTERGENIC	NA
Desi_wild_9014610	NA	*Ca_desi_Chr01*	9014610	C/A	NA	INTERGENIC	NA
Desi_wild_9023130	NA	*Ca_desi_Chr01*	9023130	C/T	NA	INTERGENIC	NA
Desi_wild_9217272	NA	*Ca_desi_Chr01*	9217272	A/C	Gene_Ca_00694.1	INTRON	Nucleolar GTP-binding protein 1
Desi_wild_9217460	NA	*Ca_desi_Chr01*	9217460	C/T	Gene_Ca_00694.1	INTRON	Nucleolar GTP-binding protein 1
Desi_wild_9218827	NA	*Ca_desi_Chr01*	9218827	T/G	Gene_Ca_00694.1	INTRON	Nucleolar GTP-binding protein 1
Desi_wild_9218890	NA	*Ca_desi_Chr01*	9218890	C/A	Gene_Ca_00694.1	UPSTREAM	Nucleolar GTP-binding protein 1
Desi_wild_9298244	NA	*Ca_desi_Chr01*	9298244	C/T	Gene_Ca_00699.1	STOP_GAINED	NA
Desi_wild_9306517	NA	*Ca_desi_Chr01*	9306517	A/C	Gene_Ca_00700.1	INTRON	Ubiquitin-conjugating enzyme E2 35
Desi_wild_9354387	NA	*Ca_desi_Chr01*	9354387	A/G	Gene_Ca_00703.1	INTRON	Dol-P-Man:Man(6)GlcNAc(2)-PP-Dol alpha-1,2-mannosyltransferase
Desi_wild_9354410	NA	*Ca_desi_Chr01*	9354410	A/G	Gene_Ca_00703.1	INTRON	Dol-P-Man:Man(6)GlcNAc(2)-PP-Dol alpha-1,2-mannosyltransferase
Desi_wild_9354518	NA	*Ca_desi_Chr01*	9354518	T/G	Gene_Ca_00703.1	INTRON	Dol-P-Man:Man(6)GlcNAc(2)-PP-Dol alpha-1,2-mannosyltransferase
Desi_wild_9354522	NA	*Ca_desi_Chr01*	9354522	G/A	Gene_Ca_00703.1	INTRON	Dol-P-Man:Man(6)GlcNAc(2)-PP-Dol alpha-1,2-mannosyltransferase
Desi_wild_9361145	NA	*Ca_desi_Chr01*	9361145	G/T	Gene_Ca_00704.1	SYNONYMOUS_CODING	Phosphoenolpyruvate carboxylase
Desi_wild_9361148	NA	*Ca_desi_Chr01*	9361148	A/T	Gene_Ca_00704.1	NON_SYNONYMOUS_CODING	Phosphoenolpyruvate carboxylase
Desi_wild_10002060	NA	*Ca_desi_Chr01*	10002060	A/C	Gene_Ca_00736.1	INTRON	Ras-related protein Rab11D
Desi_wild_10083825	NA	*Ca_desi_Chr01*	10083825	A/G	NA	INTERGENIC	NA
Desi_wild_10106781	NA	*Ca_desi_Chr01*	10106781	G/C	Gene_Ca_00746.1	DOWNSTREAM	NA
Desi_wild_10150102	NA	*Ca_desi_Chr01*	10150102	G/C	Gene_Ca_00751.1	INTRON	Family of unknown function (DUF662)
Desi_wild_10326430	NA	*Ca_desi_Chr01*	10326430	T/G	Gene_Ca_00766.1	INTRON	Nucleosome-remodeling factor subunit BPTF
Desi_wild_10334689	NA	*Ca_desi_Chr01*	10334689	A/C	Gene_Ca_00767.1	INTRON	AP-2 complex subunit alpha-2

SNP IDs	NCBI dbSNP accession IDs (NCBI_SS#)	Chromosomes	Physical positions (bp)	SNPs	Gene accession IDs	Sequence components of genome	Putative functions
Desi_wild_10366604	NA	*Ca_desi_Chr01*	10366604	G/T	Gene_Ca_00769.1	SYNONYMOUS_CODING	Calcium-dependent protein kinase 2
Desi_wild_10366618	NA	*Ca_desi_Chr01*	10366618	T/G	Gene_Ca_00769.1	NON_SYNONYMOUS_CODING	Calcium-dependent protein kinase 2
Desi_wild_10371032	NA	*Ca_desi_Chr01*	10371032	A/G	NA	INTERGENIC	NA
Desi_wild_10441561	NA	*Ca_desi_Chr01*	10441561	T/A	Gene_Ca_00776.3	NON_SYNONYMOUS_CODING	unknown protein
Desi_wild_10441567	NA	*Ca_desi_Chr01*	10441567	C/T	Gene_Ca_00776.3	SYNONYMOUS_CODING	unknown protein
Desi_wild_10441576	NA	*Ca_desi_Chr01*	10441576	C/T	Gene_Ca_00776.3	SYNONYMOUS_CODING	unknown protein
Desi_wild_10441613	NA	*Ca_desi_Chr01*	10441613	G/A	Gene_Ca_00776.3	SYNONYMOUS_CODING	unknown protein
Desi_wild_10441697	NA	*Ca_desi_Chr01*	10441697	A/C	Gene_Ca_00776.3	NON_SYNONYMOUS_CODING	unknown protein
Desi_wild_10441699	NA	*Ca_desi_Chr01*	10441699	G/A	Gene_Ca_00776.3	SYNONYMOUS_CODING	unknown protein
Desi_wild_10441719	NA	*Ca_desi_Chr01*	10441719	A/G	Gene_Ca_00776.3	NON_SYNONYMOUS_CODING	unknown protein
Desi_wild_10441800	NA	*Ca_desi_Chr01*	10441800	C/A	Gene_Ca_00776.3	NON_SYNONYMOUS_CODING	unknown protein
Desi_wild_10441806	NA	*Ca_desi_Chr01*	10441806	C/A	Gene_Ca_00776.3	NON_SYNONYMOUS_CODING	unknown protein
Desi_wild_10441837	NA	*Ca_desi_Chr01*	10441837	G/A	Gene_Ca_00776.3	SYNONYMOUS_CODING	unknown protein
Desi_wild_10530999	NA	*Ca_desi_Chr01*	10530999	T/C	Gene_Ca_00784.1	NON_SYNONYMOUS_CODING	OTU domain-containing protein 5
Desi_wild_10531022	NA	*Ca_desi_Chr01*	10531022	T/A	Gene_Ca_00784.1	NON_SYNONYMOUS_CODING	OTU domain-containing protein 5
Desi_wild_10608421	NA	*Ca_desi_Chr01*	10608421	G/C	Gene_Ca_00795.1	INTRON	Glucan endo-1,3-beta-glucosidase 14
Desi_wild_10608428	NA	*Ca_desi_Chr01*	10608428	T/A	Gene_Ca_00795.1	INTRON	Glucan endo-1,3-beta-glucosidase 14
Desi_wild_10720003	NA	*Ca_desi_Chr01*	10720003	A/T	Gene_Ca_00807.2	INTRON	NA
Desi_wild_10885416	NA	*Ca_desi_Chr01*	10885416	A/G	Gene_Ca_00823.1	SYNONYMOUS_CODING	Serine/threonine-protein phosphatase BSL3
Desi_wild_10972556	NA	*Ca_desi_Chr01*	10972556	G/A	Gene_Ca_00832.1	DOWNSTREAM	Pentatricopeptide repeat-containing protein At1g06140, mitochondrial
Desi_wild_10972557	NA	*Ca_desi_Chr01*	10972557	C/T	Gene_Ca_00832.1	DOWNSTREAM	Pentatricopeptide repeat-containing protein At1g06140, mitochondrial
Desi_wild_10991264	NA	*Ca_desi_Chr01*	10991264	T/A	Gene_Ca_00833.1	INTRON	Serine/threonine-protein kinase Nek5
Desi_wild_10991266	NA	*Ca_desi_Chr01*	10991266	T/A	Gene_Ca_00833.1	INTRON	Serine/threonine-protein kinase Nek5
Desi_wild_10991270	NA	*Ca_desi_Chr01*	10991270	A/C	Gene_Ca_00833.1	INTRON	Serine/threonine-protein kinase Nek5
Desi_wild_11070581	NA	*Ca_desi_Chr01*	11070581	T/G	Gene_Ca_00844.1	UPSTREAM	NA
Desi_wild_11104954	NA	*Ca_desi_Chr01*	11104954	A/G	Gene_Ca_00847.2	INTRON	NA
Desi_wild_11302818	NA	*Ca_desi_Chr01*	11302818	G/A	Gene_Ca_00872.1	INTRON	NA
Desi_wild_11302861	NA	*Ca_desi_Chr01*	11302861	G/A	Gene_Ca_00872.1	INTRON	NA
Desi_wild_11302870	NA	*Ca_desi_Chr01*	11302870	A/G	Gene_Ca_00872.1	INTRON	NA
Desi_wild_11302920	NA	*Ca_desi_Chr01*	11302920	G/A	Gene_Ca_00872.1	INTRON	NA
Desi_wild_11302923	NA	*Ca_desi_Chr01*	11302923	C/T	Gene_Ca_00872.1	INTRON	NA
Desi_wild_11302952	NA	*Ca_desi_Chr01*	11302952	G/C	Gene_Ca_00872.1	INTRON	NA
Desi_wild_11305587	NA	*Ca_desi_Chr01*	11305587	T/G	Gene_Ca_00872.1	INTRON	NA
Desi_wild_11305593	NA	*Ca_desi_Chr01*	11305593	T/G	Gene_Ca_00872.1	INTRON	NA
Desi_wild_11305633	NA	*Ca_desi_Chr01*	11305633	T/G	Gene_Ca_00872.1	INTRON	NA
Desi_wild_11470235	NA	*Ca_desi_Chr01*	11470235	A/G	Gene_Ca_00886.1	INTRON	Serine/threonine-protein kinase CTR1
Desi_wild_11555285	NA	*Ca_desi_Chr01*	11555285	G/T	NA	INTERGENIC	NA
Desi_wild_11569811	NA	*Ca_desi_Chr01*	11569811	A/C	NA	INTERGENIC	NA
Desi_wild_11901538	NA	*Ca_desi_Chr01*	11901538	G/T	Gene_Ca_00906.1	INTRON	Protein SAND
Desi_wild_11909644	NA	*Ca_desi_Chr01*	11909644	A/C	Gene_Ca_00907.1	SYNONYMOUS_CODING	Double-stranded RNA-binding protein 2
Desi_wild_11929981	NA	*Ca_desi_Chr01*	11929981	C/T	Gene_Ca_00908.1	INTRON	Homeobox-leucine zipper protein REVOLUTA
Desi_wild_12082676	NA	*Ca_desi_Chr01*	12082676	A/G	Gene_Ca_00923.1	INTRON	Helicase protein MOM1
Desi_wild_12155983	NA	*Ca_desi_Chr01*	12155983	T/G	NA	INTERGENIC	NA
Desi_wild_12159674	NA	*Ca_desi_Chr01*	12159674	A/C	Gene_Ca_00931.1	DOWNSTREAM	Ankyrin repeat-containing protein At5g02620

SNP IDs	NCBI dbSNP accession IDs (NCBI_SS#)	Chromosomes	Physical positions (bp)	SNPs	Gene accession IDs	Sequence components of genome	Putative functions
Desi_wild_12159861	NA	*Ca_desi_Chr01*	12159861	T/G	NA	INTERGENIC	NA
Desi_wild_12159867	NA	*Ca_desi_Chr01*	12159867	T/A	NA	INTERGENIC	NA
Desi_wild_12163294	NA	*Ca_desi_Chr01*	12163294	C/A	Gene_Ca_00932.1	UPSTREAM	NA
Desi_wild_12171484	NA	*Ca_desi_Chr01*	12171484	A/G	Gene_Ca_00933.1	SYNONYMOUS_CODING	Receptor-like protein kinase HERK 1
Desi_wild_12171505	NA	*Ca_desi_Chr01*	12171505	T/C	Gene_Ca_00933.1	SYNONYMOUS_CODING	Receptor-like protein kinase HERK 1
Desi_wild_12171580	NA	*Ca_desi_Chr01*	12171580	C/T	Gene_Ca_00933.1	INTRON	Receptor-like protein kinase HERK 1
Desi_wild_13652120	NA	*Ca_desi_Chr01*	13652120	C/A	Gene_Ca_00974.1	INTRON	Protein disulfide isomerase-like 1-6
Desi_wild_13652121	NA	*Ca_desi_Chr01*	13652121	G/T	Gene_Ca_00974.1	INTRON	Protein disulfide isomerase-like 1-6
Desi_wild_13661871	NA	*Ca_desi_Chr01*	13661871	T/G	NA	INTERGENIC	NA
Desi_wild_13737053	NA	*Ca_desi_Chr01*	13737053	G/A	Gene_Ca_00986.1	DOWNSTREAM	Pentatricopeptide repeat-containing protein At3g16010
Desi_wild_13766314	NA	*Ca_desi_Chr01*	13766314	G/A	Gene_Ca_00989.1	SYNONYMOUS_CODING	Probable sulfate transporter 3.4
Desi_wild_13798808	NA	*Ca_desi_Chr01*	13798808	A/T	Gene_Ca_00991.1	SYNONYMOUS_CODING	NA
Desi_wild_13856006	NA	*Ca_desi_Chr01*	13856006	T/C	Gene_Ca_00998.1	DOWNSTREAM	NA
Desi_wild_14011049	NA	*Ca_desi_Chr01*	14011049	C/G	NA	INTERGENIC	NA
Desi_wild_14024254	NA	*Ca_desi_Chr01*	14024254	C/T	NA	INTERGENIC	NA
Desi_wild_14093206	NA	*Ca_desi_Chr01*	14093206	T/A	NA	INTERGENIC	NA
Desi_wild_14113485	NA	*Ca_desi_Chr01*	14113485	A/G	NA	INTERGENIC	NA
Desi_wild_14114903	NA	*Ca_desi_Chr01*	14114903	A/C	Gene_Ca_01019.1	UPSTREAM	Probable LRR receptor-like serine/threonine-protein kinase At4g31250
Desi_wild_14128182	NA	*Ca_desi_Chr01*	14128182	T/C	Gene_Ca_01019.1	SYNONYMOUS_CODING	Probable LRR receptor-like serine/threonine-protein kinase At4g31250
Desi_wild_14128326	NA	*Ca_desi_Chr01*	14128326	T/G	Gene_Ca_01019.1	NON_SYNONYMOUS_CODING	Probable LRR receptor-like serine/threonine-protein kinase At4g31250
Desi_wild_14161553	NA	*Ca_desi_Chr01*	14161553	G/T	Gene_Ca_01022.1	INTRON	NA
Desi_wild_14190420	NA	*Ca_desi_Chr01*	14190420	G/A	Gene_Ca_01024.1	INTRON	Mitochondrial transcription termination factor family protein
Desi_wild_16503	NA	*Ca_desi_Chr02*	16503	A/C	NA	INTERGENIC	NA
Desi_wild_16504	NA	*Ca_desi_Chr02*	16504	G/A	NA	INTERGENIC	NA
Desi_wild_161983	NA	*Ca_desi_Chr02*	161983	A/G	Gene_Ca_01037.1	INTRON	NA
Desi_wild_161987	NA	*Ca_desi_Chr02*	161987	G/C	Gene_Ca_01037.1	INTRON	NA
Desi_wild_179173	NA	*Ca_desi_Chr02*	179173	G/T	Gene_Ca_01039.2	SYNONYMOUS_CODING	NA
Desi_wild_179194	NA	*Ca_desi_Chr02*	179194	C/G	Gene_Ca_01039.2	SYNONYMOUS_CODING	NA
Desi_wild_179260	NA	*Ca_desi_Chr02*	179260	T/A	Gene_Ca_01039.2	SYNONYMOUS_CODING	NA
Desi_wild_201869	NA	*Ca_desi_Chr02*	201869	T/C	Gene_Ca_01041.1	SYNONYMOUS_CODING	7-dehydrocholesterol reductase
Desi_wild_205528	NA	*Ca_desi_Chr02*	205528	T/C	NA	INTERGENIC	NA
Desi_wild_205617	NA	*Ca_desi_Chr02*	205617	C/T	NA	INTERGENIC	NA
Desi_wild_278031	NA	*Ca_desi_Chr02*	278031	T/C	Gene_Ca_01047.1	DOWNSTREAM	Cyclic nucleotide-gated ion channel 1
Desi_wild_278283	NA	*Ca_desi_Chr02*	278283	A/T	Gene_Ca_01047.1	DOWNSTREAM	Cyclic nucleotide-gated ion channel 1
Desi_wild_281905	NA	*Ca_desi_Chr02*	281905	T/C	NA	INTERGENIC	NA
Desi_wild_303118	NA	*Ca_desi_Chr02*	303118	C/G	NA	INTERGENIC	NA
Desi_wild_303138	NA	*Ca_desi_Chr02*	303138	A/G	NA	INTERGENIC	NA
Desi_wild_318397	NA	*Ca_desi_Chr02*	318397	A/C	Gene_Ca_01050.1	INTRON	ATP-dependent zinc metalloprotease FTSH 11, chloroplastic/mitochondrial
Desi_wild_318414	NA	*Ca_desi_Chr02*	318414	A/G	Gene_Ca_01050.1	INTRON	ATP-dependent zinc metalloprotease FTSH 11, chloroplastic/mitochondrial
Desi_wild_326926	NA	*Ca_desi_Chr02*	326926	G/T	Gene_Ca_01051.1	INTRON	Polypyrimidine tract-binding protein homolog 2
Desi_wild_327691	NA	*Ca_desi_Chr02*	327691	C/G	Gene_Ca_01051.1	INTRON	Polypyrimidine tract-binding protein homolog 2
Desi_wild_327712	NA	*Ca_desi_Chr02*	327712	T/C	Gene_Ca_01051.1	INTRON	Polypyrimidine tract-binding protein homolog 2
Desi_wild_328513	NA	*Ca_desi_Chr02*	328513	T/A	Gene_Ca_01051.1	INTRON	Polypyrimidine tract-binding protein homolog 2
Desi_wild_328681	NA	*Ca_desi_Chr02*	328681	A/C	Gene_Ca_01051.1	SYNONYMOUS_CODING	Polypyrimidine tract-binding protein homolog 2

SNP IDs	NCBI dbSNP accession IDs (NCBI_SS#)	Chromosomes	Physical positions (bp)	SNPs	Gene accession IDs	Sequence components of genome	Putative functions
Desi_wild_328828	NA	*Ca_desi_Chr02*	328828	C/T	Gene_Ca_01051.1	SYNONYMOUS_CODING	Polypyrimidine tract-binding protein homolog 2
Desi_wild_328850	NA	*Ca_desi_Chr02*	328850	G/A	Gene_Ca_01051.1	NON_SYNONYMOUS_CODING	Polypyrimidine tract-binding protein homolog 2
Desi_wild_398016	NA	*Ca_desi_Chr02*	398016	C/T	NA	INTERGENIC	NA
Desi_wild_429417	NA	*Ca_desi_Chr02*	429417	T/C	Gene_Ca_01057.2	SYNONYMOUS_CODING	NA
Desi_wild_437394	NA	*Ca_desi_Chr02*	437394	T/A	NA	INTERGENIC	NA
Desi_wild_437401	NA	*Ca_desi_Chr02*	437401	C/T	NA	INTERGENIC	NA
Desi_wild_437438	NA	*Ca_desi_Chr02*	437438	T/C	NA	INTERGENIC	NA
Desi_wild_437445	NA	*Ca_desi_Chr02*	437445	T/C	NA	INTERGENIC	NA
Desi_wild_437488	NA	*Ca_desi_Chr02*	437488	T/C	NA	INTERGENIC	NA
Desi_wild_437492	NA	*Ca_desi_Chr02*	437492	T/A	NA	INTERGENIC	NA
Desi_wild_437533	NA	*Ca_desi_Chr02*	437533	A/T	NA	INTERGENIC	NA
Desi_wild_437625	NA	*Ca_desi_Chr02*	437625	T/C	NA	INTERGENIC	NA
Desi_wild_437689	NA	*Ca_desi_Chr02*	437689	A/G	NA	INTERGENIC	NA
Desi_wild_437745	NA	*Ca_desi_Chr02*	437745	T/C	NA	INTERGENIC	NA
Desi_wild_437750	NA	*Ca_desi_Chr02*	437750	C/A	NA	INTERGENIC	NA
Desi_wild_437785	NA	*Ca_desi_Chr02*	437785	G/A	NA	INTERGENIC	NA
Desi_wild_440424	NA	*Ca_desi_Chr02*	440424	T/G	NA	INTERGENIC	NA
Desi_wild_440499	NA	*Ca_desi_Chr02*	440499	C/G	NA	INTERGENIC	NA
Desi_wild_440520	NA	*Ca_desi_Chr02*	440520	G/A	NA	INTERGENIC	NA
Desi_wild_440592	NA	*Ca_desi_Chr02*	440592	A/T	NA	INTERGENIC	NA
Desi_wild_440595	NA	*Ca_desi_Chr02*	440595	T/C	NA	INTERGENIC	NA
Desi_wild_440600	NA	*Ca_desi_Chr02*	440600	T/A	NA	INTERGENIC	NA
Desi_wild_440607	NA	*Ca_desi_Chr02*	440607	A/G	NA	INTERGENIC	NA
Desi_wild_440718	NA	*Ca_desi_Chr02*	440718	G/T	NA	INTERGENIC	NA
Desi_wild_440724	NA	*Ca_desi_Chr02*	440724	C/T	NA	INTERGENIC	NA
Desi_wild_440752	NA	*Ca_desi_Chr02*	440752	A/G	NA	INTERGENIC	NA
Desi_wild_440754	NA	*Ca_desi_Chr02*	440754	T/A	NA	INTERGENIC	NA
Desi_wild_445863	NA	*Ca_desi_Chr02*	445863	C/T	Gene_Ca_01058.1	UPSTREAM	Arabinogalactan peptide 16
Desi_wild_445878	NA	*Ca_desi_Chr02*	445878	C/T	Gene_Ca_01058.1	UPSTREAM	Arabinogalactan peptide 16
Desi_wild_445884	NA	*Ca_desi_Chr02*	445884	C/T	Gene_Ca_01058.1	UPSTREAM	Arabinogalactan peptide 16
Desi_wild_445887	NA	*Ca_desi_Chr02*	445887	A/G	Gene_Ca_01058.1	UPSTREAM	Arabinogalactan peptide 16
Desi_wild_445893	NA	*Ca_desi_Chr02*	445893	A/G	Gene_Ca_01058.1	UPSTREAM	Arabinogalactan peptide 16
Desi_wild_445911	NA	*Ca_desi_Chr02*	445911	A/C	Gene_Ca_01058.1	UPSTREAM	Arabinogalactan peptide 16
Desi_wild_445920	NA	*Ca_desi_Chr02*	445920	A/G	Gene_Ca_01058.1	UPSTREAM	Arabinogalactan peptide 16
Desi_wild_445923	NA	*Ca_desi_Chr02*	445923	A/T	Gene_Ca_01058.1	UPSTREAM	Arabinogalactan peptide 16
Desi_wild_445947	NA	*Ca_desi_Chr02*	445947	A/G	Gene_Ca_01058.1	UPSTREAM	Arabinogalactan peptide 16
Desi_wild_445950	NA	*Ca_desi_Chr02*	445950	A/T	Gene_Ca_01058.1	UPSTREAM	Arabinogalactan peptide 16
Desi_wild_527633	NA	*Ca_desi_Chr02*	527633	T/A	Gene_Ca_01062.1	SYNONYMOUS_CODING	unknown protein
Desi_wild_527634	NA	*Ca_desi_Chr02*	527634	C/A	Gene_Ca_01062.1	NON_SYNONYMOUS_CODING	unknown protein
Desi_wild_527635	NA	*Ca_desi_Chr02*	527635	C/A	Gene_Ca_01062.1	NON_SYNONYMOUS_CODING	unknown protein
Desi_wild_575518	NA	*Ca_desi_Chr02*	575518	A/G	Gene_Ca_01064.1	NON_SYNONYMOUS_CODING	CCR4-NOT transcription complex subunit 4
Desi_wild_593520	NA	*Ca_desi_Chr02*	593520	T/G	Gene_Ca_01065.1	SYNONYMOUS_CODING	Ethylene-responsive transcription factor RAP2-7
Desi_wild_593523	NA	*Ca_desi_Chr02*	593523	T/G	Gene_Ca_01065.1	SYNONYMOUS_CODING	Ethylene-responsive transcription factor RAP2-7
Desi_wild_595819	NA	*Ca_desi_Chr02*	595819	G/A	Gene_Ca_01065.1	INTRON	Ethylene-responsive transcription factor RAP2-7

SNP IDs	NCBI dbSNP accession IDs (NCBI_SS#)	Chromosomes	Physical positions (bp)	SNPs	Gene accession IDs	Sequence components of genome	Putative functions
Desi_wild_595821	NA	*Ca_desi_Chr02*	595821	T/A	Gene_Ca_01065.1	INTRON	Ethylene-responsive transcription factor RAP2-7
Desi_wild_595836	NA	*Ca_desi_Chr02*	595836	T/A	Gene_Ca_01065.1	INTRON	Ethylene-responsive transcription factor RAP2-7
Desi_wild_595837	NA	*Ca_desi_Chr02*	595837	C/A	Gene_Ca_01065.1	INTRON	Ethylene-responsive transcription factor RAP2-7
Desi_wild_595842	NA	*Ca_desi_Chr02*	595842	T/A	Gene_Ca_01065.1	INTRON	Ethylene-responsive transcription factor RAP2-7
Desi_wild_595845	NA	*Ca_desi_Chr02*	595845	G/A	Gene_Ca_01065.1	INTRON	Ethylene-responsive transcription factor RAP2-7
Desi_wild_595850	NA	*Ca_desi_Chr02*	595850	A/T	Gene_Ca_01065.1	INTRON	Ethylene-responsive transcription factor RAP2-7
Desi_wild_595851	NA	*Ca_desi_Chr02*	595851	T/A	Gene_Ca_01065.1	INTRON	Ethylene-responsive transcription factor RAP2-7
Desi_wild_595853	NA	*Ca_desi_Chr02*	595853	C/A	Gene_Ca_01065.1	INTRON	Ethylene-responsive transcription factor RAP2-7
Desi_wild_595862	NA	*Ca_desi_Chr02*	595862	T/A	Gene_Ca_01065.1	INTRON	Ethylene-responsive transcription factor RAP2-7
Desi_wild_595863	NA	*Ca_desi_Chr02*	595863	G/A	Gene_Ca_01065.1	INTRON	Ethylene-responsive transcription factor RAP2-7
Desi_wild_595868	NA	*Ca_desi_Chr02*	595868	T/A	Gene_Ca_01065.1	INTRON	Ethylene-responsive transcription factor RAP2-7
Desi_wild_692584	NA	*Ca_desi_Chr02*	692584	G/A	Gene_Ca_01066.1	INTRON	Uncharacterized protein At5g03900, chloroplastic
Desi_wild_694620	NA	*Ca_desi_Chr02*	694620	A/C	Gene_Ca_01066.1	SYNONYMOUS_CODING	Cell division cycle protein 27 homolog B
Desi_wild_727262	NA	*Ca_desi_Chr02*	727262	G/C	NA	INTERGENIC	NA
Desi_wild_749831	NA	*Ca_desi_Chr02*	749831	C/G	NA	INTERGENIC	NA
Desi_wild_750042	NA	*Ca_desi_Chr02*	750042	A/T	NA	INTERGENIC	NA
Desi_wild_760148	NA	*Ca_desi_Chr02*	760148	C/T	Gene_Ca_01073.1	NON_SYNONYMOUS_CODING	NA
Desi_wild_775943	NA	*Ca_desi_Chr02*	775943	A/G	Gene_Ca_01074.2	DOWNSTREAM	NA
Desi_wild_842908	NA	*Ca_desi_Chr02*	842908	T/C	Gene_Ca_01077.1	INTRON	DUF246 domain-containing protein At1g04910
Desi_wild_842935	NA	*Ca_desi_Chr02*	842935	T/C	Gene_Ca_01077.1	INTRON	DUF246 domain-containing protein At1g04910
Desi_wild_842943	NA	*Ca_desi_Chr02*	842943	T/C	Gene_Ca_01077.1	INTRON	DUF246 domain-containing protein At1g04910
Desi_wild_930040	NA	*Ca_desi_Chr02*	930040	T/C	Gene_Ca_01083.1	NON_SYNONYMOUS_CODING	ATP-dependent zinc metalloprotease FtsH
Desi_wild_941630	NA	*Ca_desi_Chr02*	941630	A/T	Gene_Ca_01084.1	DOWNSTREAM	EVI5-like protein
Desi_wild_972909	NA	*Ca_desi_Chr02*	972909	A/T	Gene_Ca_01087.1	INTRON	Calcium-transporting ATPase 12, plasma membrane-type
Desi_wild_972918	NA	*Ca_desi_Chr02*	972918	C/A	Gene_Ca_01087.1	INTRON	Calcium-transporting ATPase 12, plasma membrane-type
Desi_wild_972921	NA	*Ca_desi_Chr02*	972921	T/C	Gene_Ca_01087.1	INTRON	Calcium-transporting ATPase 12, plasma membrane-type
Desi_wild_972930	NA	*Ca_desi_Chr02*	972930	G/A	Gene_Ca_01087.1	INTRON	Calcium-transporting ATPase 12, plasma membrane-type
Desi_wild_972933	NA	*Ca_desi_Chr02*	972933	A/G	Gene_Ca_01087.1	INTRON	Calcium-transporting ATPase 12, plasma membrane-type
Desi_wild_1019692	NA	*Ca_desi_Chr02*	1019692	G/T	NA	INTERGENIC	NA
Desi_wild_1019708	NA	*Ca_desi_Chr02*	1019708	A/T	NA	INTERGENIC	NA
Desi_wild_1019748	NA	*Ca_desi_Chr02*	1019748	G/A	NA	INTERGENIC	NA
Desi_wild_1019793	NA	*Ca_desi_Chr02*	1019793	C/T	NA	INTERGENIC	NA
Desi_wild_1048657	NA	*Ca_desi_Chr02*	1048657	G/A	Gene_Ca_01094.1	INTRON	TRICHOME BIREFRINGENCE-LIKE 13
Desi_wild_1048681	NA	*Ca_desi_Chr02*	1048681	G/T	Gene_Ca_01094.1	INTRON	Protein furry homolog-like
Desi_wild_1066639	NA	*Ca_desi_Chr02*	1066639	A/C	NA	INTERGENIC	NA
Desi_wild_1066672	NA	*Ca_desi_Chr02*	1066672	T/A	NA	INTERGENIC	NA
Desi_wild_1081581	NA	*Ca_desi_Chr02*	1081581	T/C	Gene_Ca_01095.1	NON_SYNONYMOUS_CODING	Pentatricopeptide repeat-containing protein At3g58590
Desi_wild_1151458	NA	*Ca_desi_Chr02*	1151458	G/T	Gene_Ca_01099.1	DOWNSTREAM	gamete-expressed 3
Desi_wild_1185330	NA	*Ca_desi_Chr02*	1185330	A/G	Gene_Ca_01101.1	INTRON	Putative syntaxin-131
Desi_wild_1205892	NA	*Ca_desi_Chr02*	1205892	A/C	NA	INTERGENIC	NA
Desi_wild_1220559	NA	*Ca_desi_Chr02*	1220559	G/A	NA	INTERGENIC	NA
Desi_wild_1220563	NA	*Ca_desi_Chr02*	1220563	G/C	NA	INTERGENIC	NA
Desi_wild_1367073	NA	*Ca_desi_Chr02*	1367073	C/A	Gene_Ca_01118.1	INTRON	unknown protein
Desi_wild_1521957	NA	*Ca_desi_Chr02*	1521957	A/C	Gene_Ca_01127.1	SYNONYMOUS_CODING	Probable ubiquitin conjugation factor E4

SNP IDs	NCBI dbSNP accession IDs (NCBI_SS#)	Chromosomes	Physical positions (bp)	SNPs	Gene accession IDs	Sequence components of genome	Putative functions
Desi_wild_1621762	NA	*Ca_desi_Chr02*	1621762	C/A	Gene_Ca_01137.1	SYNONYMOUS_CODING	NA
Desi_wild_1823564	NA	*Ca_desi_Chr02*	1823564	A/G	NA	INTERGENIC	NA
Desi_wild_1857277	NA	*Ca_desi_Chr02*	1857277	C/A	NA	INTERGENIC	NA
Desi_wild_1868803	NA	*Ca_desi_Chr02*	1868803	A/C	Gene_Ca_01162.1	SYNONYMOUS_CODING	NA
Desi_wild_1894083	NA	*Ca_desi_Chr02*	1894083	A/T	NA	INTERGENIC	NA
Desi_wild_1954750	NA	*Ca_desi_Chr02*	1954750	A/C	NA	INTERGENIC	NA
Desi_wild_1996699	NA	*Ca_desi_Chr02*	1996699	G/A	Gene_Ca_01174.1	DOWNSTREAM	longifolia1
Desi_wild_2257900	NA	*Ca_desi_Chr02*	2257900	A/C	Gene_Ca_01195.1	SYNONYMOUS_CODING	Protein FLOWERING LOCUS T
Desi_wild_2344280	NA	*Ca_desi_Chr02*	2344280	G/A	Gene_Ca_01201.1	INTRON	Polypyrimidine tract-binding protein homolog 3
Desi_wild_2373768	NA	*Ca_desi_Chr02*	2373768	T/G	Gene_Ca_01203.1	INTRON	Uncharacterized aarF domain-containing protein kinase At1g71810, chloroplastic
Desi_wild_2379602	NA	*Ca_desi_Chr02*	2379602	A/T	NA	INTERGENIC	NA
Desi_wild_2406652	NA	*Ca_desi_Chr02*	2406652	A/G	Gene_Ca_01205.1	INTRON	Uncharacterized aarF domain-containing protein kinase At1g71810, chloroplastic
Desi_wild_2588707	NA	*Ca_desi_Chr02*	2588707	T/A	NA	INTERGENIC	NA
Desi_wild_2600740	NA	*Ca_desi_Chr02*	2600740	G/T	Gene_Ca_01224.1	UPSTREAM	Nuclear receptor corepressor 1
Desi_wild_2600744	NA	*Ca_desi_Chr02*	2600744	G/T	Gene_Ca_01224.1	UPSTREAM	Nuclear receptor corepressor 1
Desi_wild_2600751	NA	*Ca_desi_Chr02*	2600751	C/T	Gene_Ca_01224.1	UPSTREAM	Nuclear receptor corepressor 1
Desi_wild_2607737	NA	*Ca_desi_Chr02*	2607737	T/A	Gene_Ca_01224.1	INTRON	Nuclear receptor corepressor 1
Desi_wild_2607993	NA	*Ca_desi_Chr02*	2607993	A/T	Gene_Ca_01224.1	INTRON	Nuclear receptor corepressor 1
Desi_wild_2611144	NA	*Ca_desi_Chr02*	2611144	A/C	Gene_Ca_01224.1	DOWNSTREAM	Nuclear receptor corepressor 1
Desi_wild_2886290	NA	*Ca_desi_Chr02*	2886290	C/T	Gene_Ca_01243.1	INTRON	AP-1 complex subunit gamma-2
Desi_wild_2938023	NA	*Ca_desi_Chr02*	2938023	T/C	NA	INTERGENIC	NA
Desi_wild_2938087	NA	*Ca_desi_Chr02*	2938087	G/T	NA	INTERGENIC	NA
Desi_wild_2955638	NA	*Ca_desi_Chr02*	2955638	T/C	NA	INTERGENIC	NA
Desi_wild_3069887	NA	*Ca_desi_Chr02*	3069887	A/G	Gene_Ca_01253.1	UPSTREAM	NA
Desi_wild_3069889	NA	*Ca_desi_Chr02*	3069889	T/A	Gene_Ca_01253.1	UPSTREAM	NA
Desi_wild_3266533	NA	*Ca_desi_Chr02*	3266533	T/C	NA	INTERGENIC	NA
Desi_wild_3571882	NA	*Ca_desi_Chr02*	3571882	G/A	NA	INTERGENIC	NA
Desi_wild_3571919	NA	*Ca_desi_Chr02*	3571919	G/A	NA	INTERGENIC	NA
Desi_wild_3604220	NA	*Ca_desi_Chr02*	3604220	C/A	Gene_Ca_01296.1	STOP_GAINED	CASP-like protein 10
Desi_wild_3620996	NA	*Ca_desi_Chr02*	3620996	T/A	Gene_Ca_01297.1	DOWNSTREAM	unknown protein
Desi_wild_3722112	NA	*Ca_desi_Chr02*	3722112	T/C	NA	INTERGENIC	NA
Desi_wild_3722146	NA	*Ca_desi_Chr02*	3722146	A/G	NA	INTERGENIC	NA
Desi_wild_3722164	NA	*Ca_desi_Chr02*	3722164	T/A	NA	INTERGENIC	NA
Desi_wild_3750227	NA	*Ca_desi_Chr02*	3750227	C/A	Gene_Ca_01307.1	DOWNSTREAM	Lachrymatory-factor synthase
Desi_wild_3750295	NA	*Ca_desi_Chr02*	3750295	T/C	Gene_Ca_01307.1	DOWNSTREAM	Lachrymatory-factor synthase
Desi_wild_3750312	NA	*Ca_desi_Chr02*	3750312	A/G	Gene_Ca_01307.1	DOWNSTREAM	Lachrymatory-factor synthase
Desi_wild_3750343	NA	*Ca_desi_Chr02*	3750343	T/A	Gene_Ca_01307.1	DOWNSTREAM	Lachrymatory-factor synthase
Desi_wild_3756414	NA	*Ca_desi_Chr02*	3756414	G/T	NA	INTERGENIC	NA
Desi_wild_3756677	NA	*Ca_desi_Chr02*	3756677	T/C	NA	INTERGENIC	NA
Desi_wild_3773654	NA	*Ca_desi_Chr02*	3773654	A/G	Gene_Ca_01311.1	DOWNSTREAM	Coronatine-insensitive protein 1
Desi_wild_3775127	NA	*Ca_desi_Chr02*	3775127	A/C	Gene_Ca_01311.1	SYNONYMOUS_CODING	Coronatine-insensitive protein 1
Desi_wild_3775385	NA	*Ca_desi_Chr02*	3775385	C/T	Gene_Ca_01311.1	SYNONYMOUS_CODING	Coronatine-insensitive protein 1
Desi_wild_3854173	NA	*Ca_desi_Chr02*	3854173	A/T	Gene_Ca_01319.1	INTRON	Eukaryotic translation initiation factor 3 subunit F
Desi_wild_3930203	NA	*Ca_desi_Chr02*	3930203	C/G	Gene_Ca_01322.1	INTRON	Calcium-dependent protein kinase 13

SNP IDs	NCBI dbSNP accession IDs (NCBI_SS#)	Chromosomes	Physical positions (bp)	SNPs	Gene accession IDs	Sequence components of genome	Putative functions
Desi_wild_4058389	NA	*Ca_desi_Chr02*	4058389	G/A	Gene_Ca_01329.1	INTRON	Cytosolic purine 5'-nucleotidase
Desi_wild_4185142	NA	*Ca_desi_Chr02*	4185142	A/G	Gene_Ca_01337.1	INTRON	60S acidic ribosomal protein P0
Desi_wild_4185394	NA	*Ca_desi_Chr02*	4185394	A/T	Gene_Ca_01337.1	INTRON	60S acidic ribosomal protein P0
Desi_wild_4185408	NA	*Ca_desi_Chr02*	4185408	A/G	Gene_Ca_01337.1	INTRON	60S acidic ribosomal protein P0
Desi_wild_4185422	NA	*Ca_desi_Chr02*	4185422	T/C	Gene_Ca_01337.1	INTRON	60S acidic ribosomal protein P0
Desi_wild_4185428	NA	*Ca_desi_Chr02*	4185428	T/A	Gene_Ca_01337.1	INTRON	60S acidic ribosomal protein P0
Desi_wild_4190767	NA	*Ca_desi_Chr02*	4190767	T/A	Gene_Ca_01337.1	INTRON	60S acidic ribosomal protein P0
Desi_wild_4190802	NA	*Ca_desi_Chr02*	4190802	A/C	Gene_Ca_01337.1	INTRON	60S acidic ribosomal protein P0
[truncated: 598,784 more chars]
